# Paladin, overexpressed in colon cancer, is required for actin polymerisation and liver metastasis dissemination

**DOI:** 10.1038/s41389-022-00416-4

**Published:** 2022-07-26

**Authors:** Gilles Rademaker, Brunella Costanza, Sébastien Pyr dit Ruys, Raphaël Peiffer, Ferman Agirman, Naïma Maloujahmoum, Didier Vertommen, Andrei Turtoi, Akeila Bellahcène, Vincent Castronovo, Olivier Peulen

**Affiliations:** 1 Metastasis Research Laboratory, Giga Cancer University of Liège, Liège, Belgium; 2 Department of Experimental Oncology, European Institute of Oncology (IEO), IRCCS, Milan, 20139 Italy; 3 MassProt platform, de Duve Institute, Université Catholique de Louvain (UCLouvain), Brussels, Belgium; 4 Tumor microenvironment and resistance to treatment Laboratory, Institut de Recherche en Cancérologie de Montpellier (IRCM), Université de Montpellier (UM), Institut Régional du Cancer de Montpellier (ICM), Montpellier, France; 5 Present Address: Department of Anatomy, University of California, San Francisco, San Francisco, CA 94143 USA

**Keywords:** Colorectal cancer, Metastasis, Cell migration, Cytoskeleton

## Abstract

**Introduction:**

Colorectal cancer remains a public health issue and most colon cancer patients succumb to the development of metastases. Using a specific protocol of pressure-assisted interstitial fluid extrusion to recover soluble biomarkers, we identified paladin as a potential colon cancer liver metastases biomarker.

**Methods:**

Using shRNA gene knockdown, we explored the biological function of paladin in colon cancer cells and investigated the phospho-proteome within colon cancer cells. We successively applied in vitro migration assays, in vivo metastasis models and co-immunoprecipitation experiments.

**Results:**

We discovered that paladin is required for colon cancer cell migration and metastasis, and that paladin depletion altered the phospho-proteome within colon cancer cells. Data are available via ProteomeXchange with identifier PXD030803. Thanks to immunoprecipitation experiments, we demonstrated that paladin, was interacting with SSH1, a phosphatase involved in colon cancer metastasis. Finally, we showed that paladin depletion in cancer cells results in a less dynamic actin cytoskeleton.

**Conclusions:**

Paladin is an undervalued protein in oncology. This study highlights for the first time that, paladin is participating in actin cytoskeleton remodelling and is required for efficient cancer cell migration.

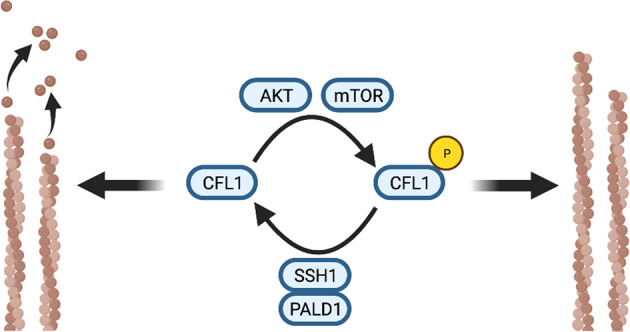

## Introduction

Colorectal cancer is the fifth most diagnosed cancer worldwide and despite important improvement in both diagnosis and therapy, it remains the third cause of death by cancer [1]. Due to lifestyle changes in developing countries, several low-risk regions have endured a rapid increase in case number, highlighting the importance to sustain our efforts to fight this disease [2]. Many colon cancer patients succumb to the development of distant metastasis and not to primary tumour growth. In this context, our laboratory has identified several potential biomarkers involved in the carcinogenesis or dissemination of colon cancer and correlated the abundance of these biomarkers with poor survival [3, 4]. Beyond an interest in diagnosis or for monitoring patients undergoing treatment, the discovery of biomarkers and the subsequent investigation of their biological functions are a source of potential and innovative therapeutic targets. Our research is part of this strategy and combines the screening of potential biomarkers and a functional investigation of the most relevant.

In this work, we explore the function of paladin, a protein encoded by the *PALD1* (*KIAA1274*) gene, and that has never before been investigated in cancer. Paladin is an evolutionarily conserved protein present in all mammal species, with a largely underestimated and initially proposed function as a pseudo-phosphatase, promoting neural crest migration in chick embryos [5]. Paladin contains two phospho-tyrosine phosphatase consensus active site motifs but no other known phosphatase characteristics. A recent report describes paladin as a phosphatidylinositol 4,5-bisphosphate phosphatase that lacks phospho-tyrosine/serine/threonine protein phosphatase activity [6].

The deregulation of kinase/phosphatase balance in cancer is of main interest since these enzymes play an important role in the activation of oncogenic pathways. In colon cancer, several kinases have already been identified as pro-tumorigenic such as MAP kinases [7]. This pro-tumorigenic effect is mostly conferred by mutations leading to constitutive activation of kinases. To date, phosphatases remain poorly studied. In humans, only a few different catalytic subunits of phosphatases exist. However, the variety of regulatory subunits increases the functional complexity of phosphatases, and explains the pleiotropic effects that phosphatases have on cell fate, metabolism or tumour microenvironment. Importantly, each holoenzyme is able to target different proteins.

Here we report, for the first time, the overexpression of paladin in colon tumours, where it is required for cell migration and metastasis. We show that paladin depletion profoundly alters the phospho-proteome within colon cancer cells. Furthermore, using immunoprecipitation experiments, we show that paladin interacts with the protein phosphatase slingshot homologue-1 (SSH1), a key regulator of cell migration [8].

## Results

In a previous report, we described the ExPEL protocol, an innovative methodology for recovering a large variety of biomolecules from fresh tissue samples. This procedure, based on fast pressure-assisted extrusion of interstitial fluid, allows the recovery and subsequent detection of proteins, miRNA, circulating DNA, metabolites and exosomes, without loss of tissue morphology and antigenicity [4]. In the initial report, 7 primary colon tumours and 6 liver metastases were submitted to the ExPEL procedure, whereby paladin was discovered as a potential biomarker in metastases.

### Paladin is overexpressed in colon tumours and correlates with poor survival

**Fig. 1:**
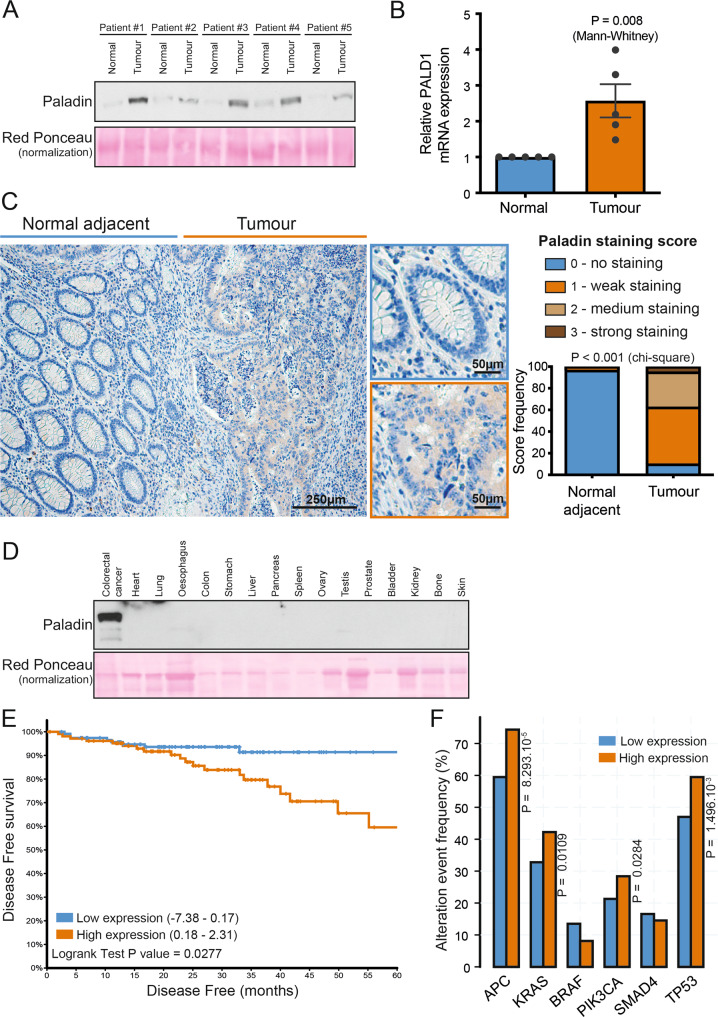
Paladin is overexpressed in colon cancer and is correlated with poor survival. **A** Paladin abundance in primary tumours and normal adjacent colon was evaluated by western blot in 15 µg total protein. Red Ponceau was used as a loading control. **B** Paladin relative gene expression in normal colon and primary tumours. Ribosomal 18 S RNA was used for normalisation. Bars represent mean ± sem (*n* = 5) associated with individual experimental replicates. **C** Immunochemical localisation of paladin in representative normal adjacent colon and primary tumour. The representative image was scored as 0 in normal adjacent and as 2 in tumour. Staining scores were attributed blindly. Score frequencies were compared by Chi-square test (*n* = 28). **D** Paladin abundance was evaluated in healthy human tissues by western blot in 15 µg total protein. Colon cancer was used as a positive control and red Ponceau was used as a loading control. **E** Pan-cancer TCGA colorectal cancer dataset was analysed for disease-free survival (*n* = 222) according to *PALD1* gene expression split according to median. Kaplan–Meier curve was established and log-rank probability calculated. **F** Pan-cancer TCGA colorectal cancer dataset was used to compare the frequency of gene alteration events in low and high *PALD1* expressing groups split according to median. *P* values are derived from the one-sided Fisher exact test.

Knowing that paladin was never been investigated in cancer, we first assessed paladin abundance in several human colon tumour samples and corresponding normal counterparts (Fig. 1A, B). In line with our exploratory ExPEL analysis, we were able to show that protein abundance and gene expression of paladin were significantly higher in tumours. We thus decided to reinforce this observation by immunochemistry, where it appeared that normal colon tissues adjacent to tumours were devoid of staining, while tumours harboured stronger (distribution median 0.5 vs 8.5) staining intensity (Fig. 1C). Encouraged by this discovery, we then evaluated the protein abundance of paladin in several healthy tissues (Fig. 1D). When compared to colon cancer tissue, paladin was consistently expressed at lower levels in all healthy tissues. In order to highlight the clinical significance of our discovery, we took advantage of the pan-cancer TCGA colorectal cancer dataset [9] and segregated 222 patient tumours according to median paladin mRNA expression, expressed as Z-score relative to all samples. We showed that, disease-free survival was significantly lower in patients with high paladin expression (*p* = 0.0277; Fig. 1E). In the same cohort, we evaluated the alteration frequency of 6 key driver genes [10] according to paladin expression. It appeared that *APC*, *KRAS*, *PIK3CA* and *TP53* were significantly more frequently altered in patients with high paladin expression (Fig. 1F).

### Paladin is overexpressed in colon cancer liver metastasis

**Fig. 2:**
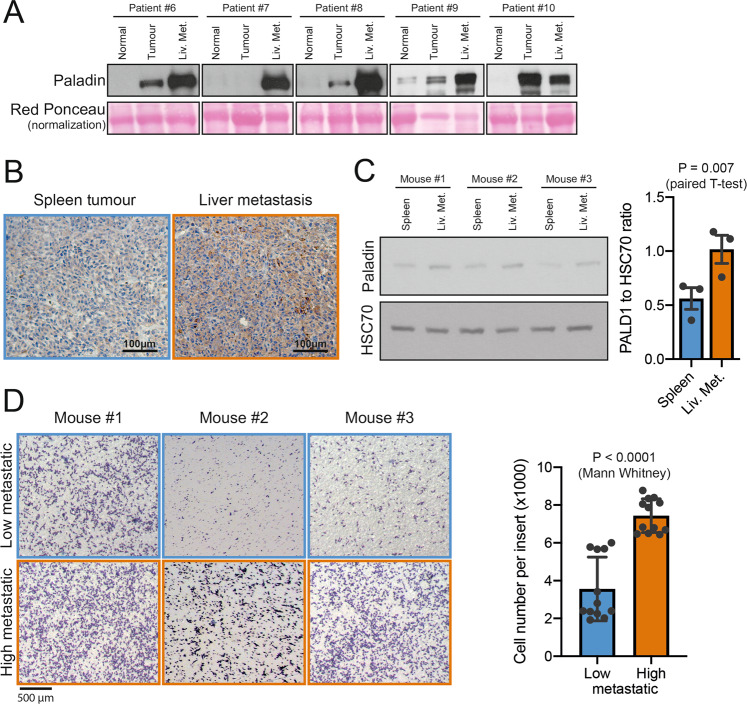
Paladin is overexpressed in human colon cancer liver metastasis and in a metastatic mice model. **A** Paladin abundance was assessed by western blot in normal colon, primary tumour and liver metastasis. Red Ponceau was used as a loading control. **B** Paladin immunohistochemistry in HCT116 spleen tumour and HCT116-derived liver metastasis. **C** Paladin western blot in HCT116 spleen tumour and HCT116-derived liver metastasis. HSC70 was used as loading control. Western blot quantification was performed and groups were compared using a paired *T* test (*n* = 3). **D** Representative images of Boyden chamber migration of spleen tumour-derived HCT116 (low metastatic) and liver metastasis-derived HCT116 (high metastatic) cells. Images were individually analysed and quantified. Bars represent mean ± sem (*n* = 12) associated with individual experimental replicates. Groups were compared by unpaired Mann–Whitney test.

Owing to the reported implication of paladin in neural crest migration of embryos [5], we evaluated the abundance of paladin in colon cancer liver metastasis. Interestingly, we discovered that paladin was more abundant in liver metastasis than in primary colon tumours, suggesting an involvement of this protein in metastatic dissemination (Fig. 2A). To strengthen this hypothesis, we utilised a mice model of experimental metastasis. HCT116 cells were injected into mice's spleen, and tumours were grown over 4 weeks. We collected both the tumour at splenic injection site and the metastatic foci in the liver [11]. Paladin was immunodetected in these tissues. Interestingly, paladin staining was stronger in the liver metastasis than in the primary tumour (Fig. 2B). Using this model, we generated HCT116-derived clones with low metastatic or high metastatic potential, that were collected from spleen or liver metastasis, respectively. Paladin abundance was significantly increased in cells harvested from liver metastasis compared to splenic tumour cells (Fig. 2C). In order to further validate the involvement of paladin in colon cancer metastases, we decided to evaluate the migration potential of low metastatic and high metastatic clones in vitro using a Boyden chamber assay. We successfully showed that low metastatic clones had less migratory potential than high metastatic clones (Fig. 2D).

### Paladin is required for tumour growth, cell migration and metastasis development

**Fig. 3:**
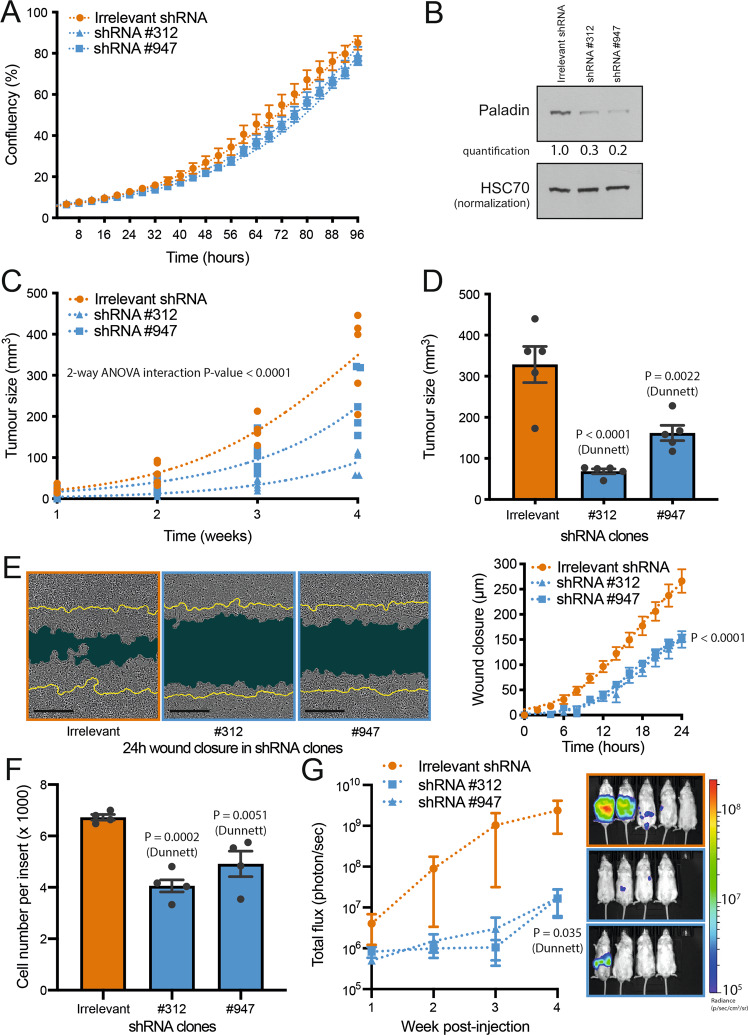
Paladin is required for tumour growth, cell migration, and metastatic dissemination. **A** Cell proliferation was analysed in paladin-deleted HCT116 clones using Incucyte for 96 h. Cell growths were fit by least square regression and compared by the extra sum-of-squares F test (*n* = 4). **B** Paladin western blot in HCT116 paladin shRNA clones. HSC70 was used as a loading control. **C** Kinetic tumour growth of mice (*n* = 5) injected subcutaneously with HCT116 paladin shRNA clones. Individual biological replicates are depicted while curves were fit according to an exponential growth model by least square regression. Results were compared by 2-way ANOVA. **D** Endpoint size of tumour resected from mice injected subcutaneously with HCT116 paladin shRNA clones. Bars represent mean ± sem (*n* = 5) associated with individual experimental replicates. Groups were compared by 1-way ANOVA followed by Dunnett’s pair comparisons. **E** Representative images (24 h post-scratch) of wound-healing experiments with HCT116 paladin shRNA clones. Scale bars represent 400 µm. Images were individually analysed and quantified. Dots represent mean ± sem (*n* = 6) while curves were fit according to a logistic model by least square regression and compared by the extra sum-of-squares F test. **F** Boyden chamber migration of HCT116 paladin shRNA clones was individually analysed and quantified. Bars represent mean ± sem (*n* = 4) associated with individual experimental replicates. Groups were compared by 1-way ANOVA followed by Dunnett’s pair comparisons. **G** In vivo imaging of liver metastatic lesions in mice injected with HCT116 paladin shRNA clones. Results were represented as mean ± sem (*n* = 4–5). Groups were compared by 2-way ANOVA followed by Dunnett’s pair comparisons.

Motivated by these unforeseen results, we decided to evaluate HCT116 behaviour upon shRNA-mediated paladin depletion. Initially, we analysed in vitro cell proliferation which was unchanged in both shRNA clones (Fig. 3A) despite a 70–80% reduction of paladin expression (Fig. 3B). We then subcutaneously injected HCT116 cells in the flank of immunocompromised mice and analysed tumour growth for 4 weeks. We showed a significant reduction in the tumour growth kinetics (Fig. 3C) and the final tumour size, with both shRNA clones (Fig. 3D). Similar reductions were observed with Lovo and LS174T cell lines depleted for paladin by shRNA (Supplementary Fig. 1A–D). Puzzled by the discrepancy between in vitro proliferation results, showing no modification upon paladin depletion, and the decrease of in vivo tumour growth, we evaluated Ki67 staining and cleaved-caspase-3 staining in tumour sections (Supplementary Fig. 1E, F). This analysis showed no significant difference between control and paladin-depleted conditions suggesting a non-autonomous cell effect on in vivo cell growth.

Based on our earlier results describing the overexpression of paladin in liver metastasis and in cells with high migratory potential, we wanted to check whether the shRNA-based depletion of paladin altered cell migration. We first discovered that cell migration, in a wound-healing experiment, was significantly impaired by paladin depletion (Fig. 3E). This result was further validated in a Boyden chamber migration assay using HCT116 (Fig. 3F), Lovo and LS174T cell lines (Supplementary Fig. 2A). Next, we evaluated the implication of paladin in an experimental model of metastatic dissemination by performing intrasplenic injections of paladin-depleted luciferase-expressing HCT116 cells. Four weeks after injection, mice were imaged and showed a significant reduction of the luminescence signal when injected with paladin-depleted luciferase-expressing HCT116 cells (Fig. 3G). Similar results, but less significant, were obtained when LS174T or LoVo cell lines were injected (Supplementary Fig. 2B). Our results demonstrate for the first time that paladin is required for optimal tumour growth and metastatic dissemination.

### Quantitative (phospho)-proteomics reveals paladin implication in kinase/phosphatase activity

**Fig. 4:**
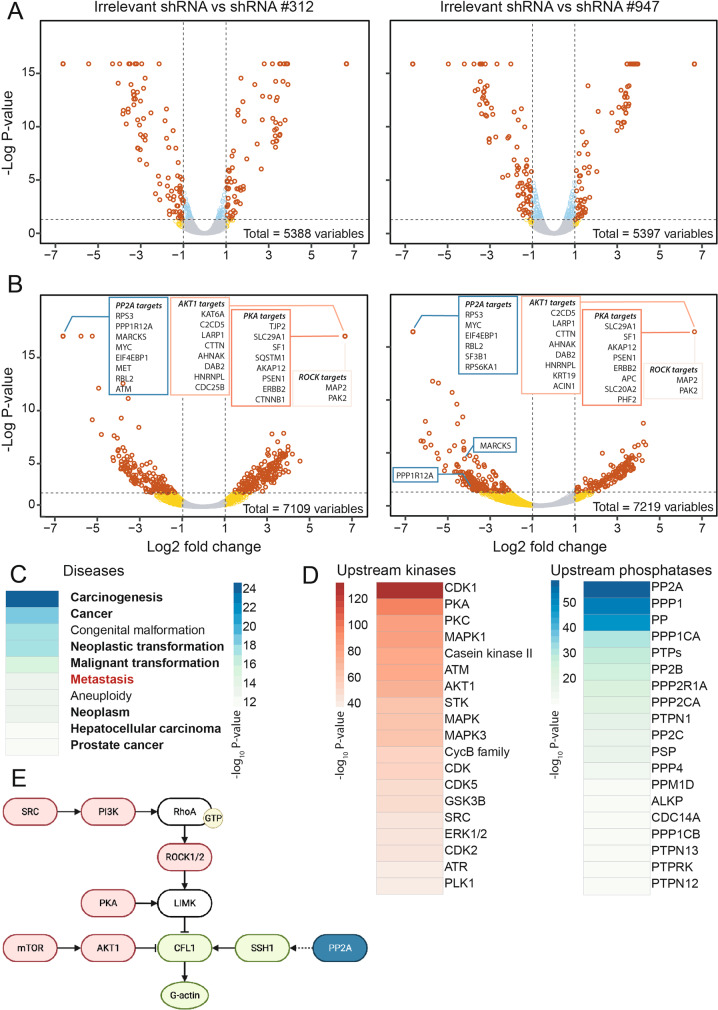
Quantitative (phospho-)proteomics reveals paladin involvement in diseases and kinase/phosphatase activities. **A** Volcano plots displaying relative variation of protein abundance in HCT116 paladin shRNA clones. **B** Volcano plots displaying a relative variation of phospho-protein abundance in HCT116 paladin shRNA clones. **C** Heatmaps representing diseases highlighted by gene enrichment analysis. Results are presented as –log_10_ *P* value. **D** Heatmaps representing top 20 kinases (out of 413) or phosphatases (out of 55) obtained from literature mining and able to catalyse phospho modifications induced by paladin depletion. Results are presented as –log_10_ *P* value. **E** Potential pathway altered by paladin depletion. Kinases and phosphatases able to catalyse phospho modifications induced by paladin depletion are depicted in red and blue, respectively. Inferred proteins, considered the core pathway to further analyse, are depicted in green.

Due to the initially proposed function of paladin as a pseudo-phosphatase, as well as the recent report showing its phosphatase activity, we undertook a (phospho)-proteomic analysis of paladin-depleted HCT116 cells. Overall, we identified more than 5000 proteins (supplementary tables 1 & 2), of which pairwise comparisons detected 556 and 584 differentially (*P* < 0.05) abundant proteins in shRNA #312 and shRNA #947 clones, respectively, when compared with control (Fig. 4A). We then combined proteomic data from both shRNA clones, and restricted further analysis to the overlapping differentially abundant proteins. Functional enrichment revealed, a significant enrichment for phosphoproteins (term KW-0597, FDR = 1.85 × 10^−6^). Proteins were organised as a network and protein selection was restricted to candidates with an average shortest path longer than 3. Cell compartment localisation analysis showed that 16% of the proteins (*n* = 235) had cytoskeletal scores higher than 2.5 out of 5.0 (Supplementary Fig. 3). In comparison with the total proteomic analysis, a much larger number of unique peptides were differentially (*P* < 0.05) phosphorylated upon paladin depletion (728 and 900 in shRNA #312 and shRNA #947 clones, respectively) (Fig. 4B). These unique peptides harboured 1103 and 1480 significantly modulated phosphorylation sites out of 7109 and 7219 detected, respectively (supplementary tables 3 and 4). Differentially phosphorylated peptides were used to highlight gene ontology processes altered by paladin depletion (Fig. 4C). Several disease-related pathways were significantly modified, including cancer and metastasis; reinforcing our abovementioned discoveries. Based on the individual phosphorylation sites known to be specific targets of kinases or phosphatases, and modified by paladin depletion, we established a *P* value ranked list of kinases and phosphatases able to catalyse modifications of the specified phosphorylation sites (Fig. 4D, supplementary tables 5 and 6). By grouping these enzymes based on function, we identified a potentially altered pathway involved in cell migration (Fig. 4E), which included cofilin, the main regulator of actin dynamic instability.

### Paladin interacts with SSH1 and controls cofilin phosphorylation

**Fig. 5:**
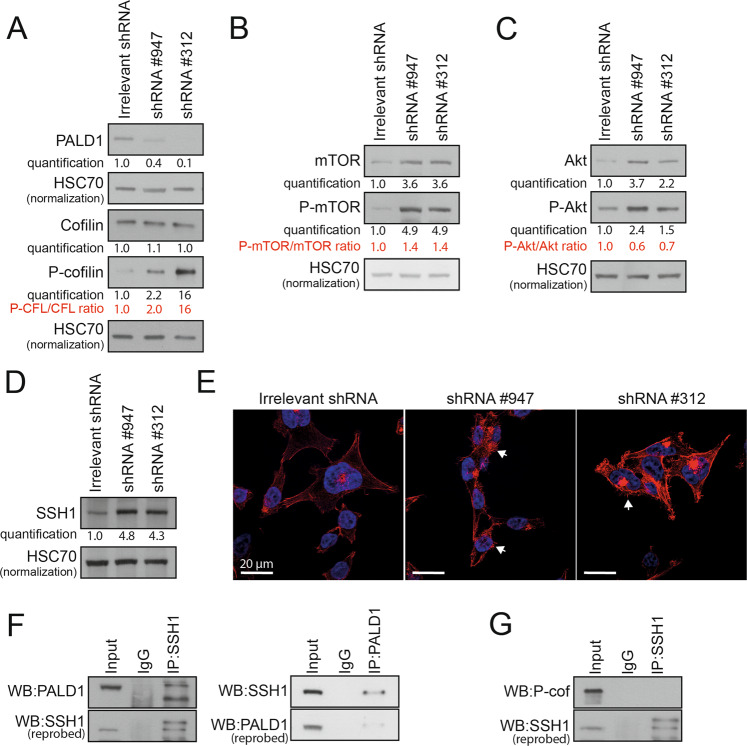
Paladin interacts with slingshot homologue-1 and regulates the phosphorylation level of actin remodelling regulators in HCT116. **A** Paladin and serine 3 phospho-cofilin western blot in HCT116 shRNA clones. **B** mTOR and serine 2481 phospho-mTOR western blot in HCT116 shRNA clones. **C** Akt and serine 473 phospho-AKT western blot in HCT116 shRNA clones. **D** SSH1 western blot in HCT116 shRNA clones. HSC70 was used as a loading control. **E** Actin staining with fluorescent phalloidin in HCT116 shRNA clones. White arrows show filopodia. **F**, **G** HCT116 lysates were immunoprecipitated with SSH1, paladin or irrelevant antibodies (IP) followed by western blot with paladin, SSH1 or phospho-cofilin antibodies (WB).

To better understand the role of paladin in the identified cell migration pathway, we first analysed the abundance of phospho-cofilin in paladin-depleted HCT116. In accordance with the pathway suggested above, serine 3 phospho-cofilin abundance was strongly increased upon paladin depletion (Fig. 5A). Phospho-cofilin is unable to sever actin filaments [12], and is under the control of several kinases including the mTOR/AKT pathway [13]. We thus analysed the phosphorylation level of mTOR and AKT in paladin shRNA HCT116 clones, and indicated that paladin knockdown consistently increased the abundance of total and phosphorylated mTOR and AKT (Fig. 5B, C). We next hypothesised a reduction of the counteracting phosphatase, SSH1. Unexpectedly, our results demonstrated a fourfold increase of SSH1 abundance in paladin shRNA HCT116 clones (Fig. 5D). These results demonstrate that paladin silencing triggers two opposite effects. On one hand, paladin activates the mTOR/AKT pathway and cofilin phosphorylation, and on the other hand the accumulation of SSH1 but without any impact on the dephosphorylation of cofilin. Similar results were obtained in paladin-depleted LoVo cells (Supplementary Fig. 4A–D). In contrast, PALD1 depletion in LS174T cells did not trigger the phosphorylation of cofilin while AKT is activated (Supplementary Fig. 5A–D). Based on these contradictory results, we decided to analyse the actin cytoskeleton in the three PALD1-depleted cell lines. Phalloidin staining revealed only few actin filaments in irrelevant shRNA clones, while an increased density of filopodia was observed in paladin shRNA HCT116 clones (Fig. 5E, Supplementary Figs. 4E and 5E). Considering the increased level of phospho-cofilin, we suggest a stabilisation of actin cytoskeleton inside filopodia resulting in impaired motility. Owing to the documented physical interaction between phospho-cofilin, SSH1 and actin [14–17], we decided to evaluate the participation of paladin in this protein-protein interaction. We performed co-immunoprecipitation experiments and showed that paladin was indeed interacting with SSH1 (Fig. 5F, Supplementary Figs. 4F and 5F). Of note, *SSH*-family gene expression, mainly *SSH1* (Spearman correlation; *P* = 0.0201) and *SSH3* (Spearman correlation; *P* = 0.0200), was positively and significantly correlated to paladin gene expression in pan-cancer TCGA colorectal cancer patients. However, we were unable to show an interaction between paladin and cofilin (Fig. 5G). Therefore, we hypothesise that paladin could act as an adaptor protein in the actin remodelling machinery.

## Discussion

Colon cancer is driven by several mutations in genes such as *APC*, *TP53*, *SMAD4*, *PI3KC3* and *KRAS* [18]. Recurrent alterations have also been observed in MAPK pathways. Interestingly, these mutations not only drive cancer progression but also metastatic dissemination [19], and often lead to erroneous activation of kinases or phosphatases, and their downstream pathways. In the past decade, several compounds targeting pro-tumorigenic kinases have been developed, with promising effects [20]. Yet, phosphatases have received little attention as potential drug targets in cancer, largely due to the unsuccessful attempt to target them selectively [21]. However, phosphatases have recently regained special attention and are now actively investigated in clinicaltrials. As an example, PTPN11 (also known as SHP2) is a protein tyrosine phosphatase working downstream of RTKs and that has been associated with several cancer types [22]. Interrogating *clinicaltrials.gov* databases, several studies involving CRC patients are currently recruiting patients to evaluate allosteric inhibitors of SHP2 (ClinicalTrials.gov Identifiers: NCT04699188, NCT05163028, NCT04252339).

Nevertheless, a large number of kinases (~500) encoded by the human genome, in comparison to the ~250 phosphatases, suggests that there are not enough phosphatases to assign a unique one to each kinase [23, 24]. Consequently, phosphatase inhibitors will inevitably lead to off-target effects; pointed out by the tremendous number of interactions established by phosphatases [24]. For the first time, we identify paladin, encoded by the *PALD1* gene, as overexpressed in colon cancer. We show that elevated paladin expression correlated with disease-free survival in colon cancer, and that *APC*, *KRAS*, *PIK3CA* and *TP53* oncogenes were significantly more frequently altered in colon cancer patients with high paladin expression.

In our hands, paladin depletion did not influence in vitro cell growth, while surprisingly reducing tumour growth in vivo. In accordance with Ki67, cleaved-caspase-3 stainings, and gene ontology analysis of total proteomic data, performed with Pingo application in Cytoscape, were unable to highlight any cell proliferation-associated terms (i.e., #8283, #8284, or #8285), we suggest that the growth impairment observed in vivo was a non-cell-autonomous effect.

Considering that metastatic lesions are the main cause of death in colon cancer patients [25], we analysed the abundance of paladin in colon cancer liver metastases and discovered that its expression was further increased.

Paladin is a conserved protein expressed in neural crest cells in chicks [5] and mice [26]. While paladin promotes neural crest migration in chick [5], *PALD1* ^−/−^ mice harboured unaltered neural crest-derived tissues [27], indicating the absence of direct function for PALD1 in the migration of these cells during embryogenesis in mice. Neural crest migration has significant similarities with cancer metastasis [28], and thus, these two phenomena likely share some mechanisms. Based on this we were prompted to investigate the function of paladin in colon cancer. We discovered that paladin was required for optimal colon cancer cell migration.

Paladin was previously reported as a negative regulator of AKT phosphorylation [29]. Therefore, we decided to investigate the modulation of phospho-proteome upon paladin depletion, and showed that thousands of phosphorylation sites were altered across the proteome. Based on additional findings, it appears that paladin interacts with the phosphatase SSH1. The SSH proteins are a family of phosphatases playing a role in actin dynamics by activating cofilin [17]. Interestingly, *SSH1* expression was recently associated with colon cancer progression through regulation of the epithelial to mesenchymal transition [30]. Furthermore, patients with elevated *SSH1* levels have been shown to exhibit greater lymph node invasion [31]. Based on these results we proposed to analyse the function of cofilin, a main regulator of actin remodelling [12]. Our results showed phosphorylation of cofilin on serine 3 upon paladin depletion. Phosphorylation of cofilin releases it from actin, and inhibits its ability to depolymerise F-actin, reducing the cellular concentration of G-actin and consequently decreasing the turnover rate of actin filaments required for filopodia construction at the leading edge of the migratory cell [32]. A recent report showed a link between cofilin phosphorylation and SRC/AKT/mTOR pathway in melanoma [13]. In our hands, paladin silencing led to AKT and mTOR activation and is likely the origin of cofilin inhibition.

## Conclusions

Paladin identification as a biomarker in tumour or metastases samples was the result of the development of a new method and involved mass spectrometry and western blots. The systematic screening of patients for paladin abundance is still difficult to consider and needs a validated quantitative assay such as ELISA. Our results suggest that paladin participates, mainly in HCT116, in actin remodelling of colon cancer cells. In accordance, paladin silencing impaired colon cancer cell migration. The deciphering of paladin biochemistry would shed light on its potential as a therapeutic target. But, to our knowledge, no specific inhibitor of paladin activity was reported so far. The use of paladin as a therapeutic target still needs validation and development.

## Material and methods

### Cells and reagents

Human colon cancer cells HCT116 (CCL-247) were acquired from ATCC (Manassas, VA, USA). Additional cell lines were bought from ECACC (LS174t, Lovo). Cells were authenticated by Short-Tandem Repeat profiling (DSMZ, Braunschweig, Germany). Cell counting was performed using TTC Casy cell counter (OLS, Bremen, Germany). All reagents were purchased from Sigma-Aldrich (Bornem, Belgium) except when mentioned otherwise. Antibodies were purchased from Santa Cruz Technologies: HSC70 (sc-7298), phospho-cofilin Ser 3 (sc-12912); Sigma-Aldrich: PALD1; Cell Signaling: SSH1 (#13578), phospho-AKT Ser473 (#4060), akt (#9272), mTOR (#2983), phospho-mTOR Ser 2481 (#2974), cleaved-caspase-3 (clone 5A1E, #9664); or Dako: KI67 (M7240).

### Cell culture

HCT116 were cultured in McCoy 5 A supplemented with 10% foetal bovine serum (FBS). LS174T were maintained in Minimum Essential Medium (MEM) supplemented with 2 mM L-glutamine, 10% FBS and non-essential amino acids (Gibco #111040-085). Lovo were maintained in Ham’s F12 supplemented with 10% FBS and 2mM L-glutamine. Cells were cultured in a 37 °C, 5% CO_2_ incubator. Cells were used between passages 1 and 10 and checked monthly for mycoplasma.

### Plasmid transduction

Plasmids were transduced using lentiviral vectors in the GIGA institute viral vector facility. The sequences of shRNA #312 and #947 were TCATGGTCTCGCTGACAGT and ATCACAACAGGGGCCACCT, respectively. The irrelevant shRNA vector (SMARTvector lentiviral control) was purchased from Dharmacon (Lafayette, CO, USA).

### Immunohistochemistry

Primary tumours (*n* = 28) were obtained from our institution Biobank, as formalin-fixed, paraffin-embedded tissue blocks. Tissue sections were stained as described previously [33]. Based on a serial dilution, the selected optimal paladin primary antibody dilution was 1:150. Tissue sections incubated with no primary antibody showed no reactivity. Immunohistochemistry was scored independently and blindly by two investigators (GR, OP), as reported previously [34]. Briefly, Paladin, Ki67 or cleaved-caspase-3 scorings were performed by evaluating extent (%) of each intensity score (ranging from 0 to 3). A global score was the rounded value of the sum of each intensity pondered by its respective extent.

### Cell proliferation assay

Cell proliferation was monitored in phase contrast using the Incucyte S3 (Sartorius, Ann Arbor, MI, USA). Cells were seeded in 96-well plates at 10,000 cells per well and images were acquired every 8 h for 96 h. Quantification was performed using the Incucyte software (2021B gui, Sartorius). Each experiment was performed as *n* = 3 biological replicates.

### Wound-healing assay

Wound-healing assays were performed as reported previously [11] using Incucyte S3 (Sartorius, Ann Arbor, MI, USA). Briefly, cells were seeded in 96-well plates at 20,000 cells per well 24 h before the beginning of the experiment to achieve full confluency. Scratches were performed using the Incucyte WoundMaker tool (Sartorius). Wells were imaged every 2 h for 24 h. Quantification was performed using the Incucyte software (2021B gui, Sartorius). Each experiment was performed as *n* = 3 biological replicates.

### Boyden’s chamber chemotaxis migration assay

Cells were suspended in serum-free Dulbecco’s modified Eagle medium (DMEM) supplemented with 0.1% bovine serum albumin (BSA) and 1% penicillin/streptomycin, and seeded (100,000 cells/well) in Boyden Chamber inserts (Costar, 8 µm pore size). The chemo-attractant medium was supplemented with 10% FBS. After 24 h migration, Boyden chambers were cleaned with coton-swaps and then stained with crystal violet. The number of migrating cells was quantified by ImageJ [35]. Each experiment was performed as *n* = 3 biological replicates.

### Co-immunoprecipitation

Total proteins were extracted using a non-denaturing buffer containing Tris-HCl pH 8 (20 mM), NaCl (137 mM), NP40 (1%), EDTA (2 mM) and supplemented with protease inhibitors. Co-immunoprecipitation was performed as described previously [36]. Each experiment was performed as *n* = 3 biological replicates.

### Western blotting

Cells were lysed using 1% sodium dodecyl sulphate with protease and phosphatase inhibitors. Protein separation was then performed using polyacrylamide gel electrophoresis as described previously [37]. Each experiment was performed as *n* = 3 biological replicates. western blots were quantified using the ImageJ gel analysis tool [35]. Relative quantification was performed according to HSC70 as a loading control.

### Subcutaneous injection

Colon cancer cells (10^6^ cells) were suspended in 100 µl DMEM and injected into the flank of 5-week-old NOD-SCID mice randomly assigned to experimental groups. Tumour size was measured weekly during 3–5 weeks. After sacrifice, tumours were resected and measured. Each experiment was performed as *n* = 3 biological replicates. The sample size was chosen thanks to an a priori *T* test power evaluation (software G-Power 3.1) [38], using a 40% tumour volume reduction and 95% power.

### Intrasplenic injection

Luciferase-positive colon cancer cells (HCT116, LS174T or LoVo—10^6^ cells) were injected into the spleen of NOD-SCID mice, as reported previously [11]. For the experimental metastasis model, splenectomy was performed immediately after cell injection. Mice were imaged each week to assess metastasis spreading using IVIS optical imaging system (Perkin Elmer, Waltham, MA, USA). After 4–6 weeks, mice were sacrificed and imaged.

### Establishment of cell clones with differential liver-tropic migratory potential

Invaded spleen and liver collected from the intrasplenic injection in vivo model were dissociated with a mix of collagenase (1600 mg/mL) and hyaluronidase (300 mg/mL) in Hank’s Balanced Salt Solution at 37 °C for 30 min under agitation. The dissociated tissues were then filtrated (70 µm mesh) and cells were seeded in T25 flasks with DMEM supplemented with 10% FBS, 1% penicillin–streptomycin and 0.4% fungizone. Cells were then kept for 4 passages in culture to eliminate fibroblasts. Subsequent cell culture was used to establish cell clones with different migratory potential.

### Proteomics and phosphoproteomics

Cells were scraped and lysed in ice-cold lysis buffer (100 mM Tris pH 8.5, 4% sodium deoxycholate). The suspension was thoroughly heated to 95 °C for 5 min before being probe-sonicated three times for 15 sec. Clear lysates were obtained by centrifugation at 18,000 × g for 5 min. Protein concentration was determined by bicinchoninic acid according to the manufacturer’s instructions (Thermofisher Scientific). Samples (500 µg) were diluted with lysis buffer to a final volume of 270 µL before being processed as described previously [39]. At the end of the process, enriched phosphopeptides were reconstituted in 8 µL of solvent A (0.1% trifluoracetic acid in 2% acetonitrile), directly loaded onto reversed-phase pre-column (Acclaim PepMap 100, Thermo Scientific) and eluted in backflush mode. An aliquot (800 ng) of the original peptide mixture was also analysed for total proteome determination. Peptide separation was performed using a reversed-phase analytical column (Acclaim PepMap RSLC, 0.075 × 250 mm, Thermo Scientific) with a linear gradient of 4–27.5% solvent B (0.1% formic acid in 80% acetonitrile) for 100 min, 27.5%-40% solvent B for 10 min, 40–95% solvent B for 1 min and holding at 95% for the last 10 min at a constant flow rate of 300 nL/min on an Ultimate 3000 RSLC system. Peptides were analysed by an Orbitrap Fusion Lumos tribrid mass spectrometer (ThermoFisher Scientific). The peptides were subjected to NSI source followed by tandem mass spectrometry (MS/MS) coupled online to the nano-LC. Intact peptides were detected in the Orbitrap at a resolution of 120,000. Peptides were selected for MS/MS using HCD setting at 30, ion fragments were detected in the Orbitrap at a resolution of 60,000. A data-dependent procedure that alternated between one MS scan followed by MS/MS scans was applied for 3 sec for ions above a threshold ion count of 5.0E4 in the MS survey scan with 30.0 s dynamic exclusion. The electrospray voltage applied was 2.1 kV. MS1 spectra were obtained with an AGC target of 4E5 ions and a maximum injection time of 50 ms, and MS2 spectra were acquired with an AGC target of 1E5 ions and a maximum injection set to 110 ms. For MS scans, the m/z scan range was 325 to 1800. The resulting MS/MS data were processed using Sequest HT search engine within Proteome Discoverer 2.4 SP1 against a *Human database* protein database obtained from Uniprot. Trypsin was specified as a cleavage enzyme allowing up to 2 missed cleavages, 4 modifications per peptide and up to 5 charges. A mass error was set to 10 ppm for precursor ions and 0.1 Da for fragment ions. Oxidation on Met (+15.995 Da), phosphorylation on Ser, Thr and Tyr (+79.966 Da), conversion of Gln (−17.027 Da) or Glu (−18.011 Da) to pyro-Glu at the peptide N-term were considered as variable modifications. False discovery rate was assessed using Percolator and thresholds for protein, peptide and modification site were specified at 1%. For abundance comparison, abundance ratios were calculated by Label-Free Quantification (LFQ) of the precursor intensities within Proteome Discoverer 2.4 SP1. The mass spectrometry proteomics data have been deposited to the ProteomeXchange Consortium via the PRIDE [40] partner repository with the dataset identifier PXD030803 and 10.6019/PXD030803. The experiment was performed as *n* = 3 biological replicates.

### Ethics

All animal experimental procedures were performed according to the Federation of European Laboratory Animal Sciences Associations (FELASA) and were accepted after review by the Institutional Animal Care and Ethics Committee of the University of Liège, Belgium (#19-2156). Animals were housed in the animal facility of the GIGA institute.

### Statistics

All results are shown as means with a standard error of the mean (sem). Two-sided statistical tests were used according to experimental design. Specific statistical methods are cited in the figures and were chosen according to group number and homoscedasticity. *P* value < 0.05 was considered statistically significant. Kinase and phosphatase network was constructed by literature mining with Pathway Studio® (May 2020, Elsevier, Amsterdam, The Netherlands). Heatmaps and volcano plots were designed using R v4 installed with pheatmap 1.0.12 and EnhancedVolcano v1.6.0 packages. Gene ontology analysis was performed in Cytoscape v3.9 [41].

## Supplementary information

Supplemental figure legends

Supplemental figure 1

Supplemental figure 2

Supplemental figure 3

Supplemental figure 4

Supplemental figure 5

Supplemental table 1

Supplemental table 2

Supplemental table 3

Supplemental table 4

Supplemental table 5

Supplemental table 6

## Data Availability

The mass spectrometry proteomics data have been deposited to the ProteomeXchange Consortium via the PRIDE (http://www.proteomexchange.org/) partner repository with the dataset identifier PXD030803 and 10.6019/PXD030803.
