# Supplementary material for: Paladin, overexpressed in colon cancer, is required for actin polymerisation and liver metastasis dissemination
Source: Oncogenesis. 2022 Jul 26;11(1):42. doi: 10.1038/s41389-022-00416-4 (PMC9325978; doi:10.1038/s41389-022-00416-4)
Supplement: Supplementary file 1 — Supplemental figure legends [file 41389_2022_416_MOESM1_ESM.docx]

**Supplemental figure legends**

**Supplemental figure S1:** **Paladin is required for tumour growth. A & C.** Kinetic of tumour growth in mice (n=5) injected subcutaneously with Lovo or Ls174T paladin shRNA clones. Individual biological replicates are depicted while curves were fit according to exponential growth model by least square regression. Results were compared by 2-way ANOVA. **B & D.** Endpoint size of tumour resected from mice (n=5) injected subcutaneously with Lovo or Ls174T paladin shRNA clones. Bars represent mean ± sem associated with individual experimental replicates. Groups were compared by 1-way ANOVA followed by Dunnett’s pair comparisons. **E & F.** Ki67 and cleaved-caspase-3 scores evaluated on tumours resected from mice (n=5) injected subcutaneously with HCT116, Lovo or Ls174T paladin shRNA clones. Bars represent mean ± sem associated with individual experimental replicates. Groups were compared by 1-way ANOVA followed by Dunnett’s pair comparisons.

**Supplemental figure S2:** **Paladin is required for cell migration. A.** Boyden chamber migration (n=3) of Lovo or Ls174T paladin shRNA clones. **B.** Quantification of in vivo imaging of liver metastatic lesions in mice injected with LS174T or LoVo paladin shRNA clones. Results were represented as mean ± sem (n=4-5). Groups were compared by 2-way ANOVA followed by Dunnett’s pair comparisons.

**Supplemental figure S3:** **Quantitative proteomics reveals a link between paladin and cytoskeleton-related proteins.** Proteins differentially expressed by paladin depletion in HCT116 cell line were represented as a network and associated with a cytoskeleton score (ranging from 0 to 5).

**Supplemental figure S4: Paladin interacts with slingshot homolog-1 and regulates phosphorylation level of actin remodelling regulators in LoVo. A.** Paladin and serine 3 phospho-cofilin western-blot in Lovo shRNA clones. **B.** mTOR and serine 2481 phospho-mTOR western-blot in Lovo shRNA clones. **C.** Akt and serine 473 phospho-AKT western-blot in Lovo shRNA clones. **D.** SSH1 western-blot in Lovo shRNA clones. HSC70 was used as loading control. **E.** Actin staining with fluorescent phalloidin in Lovo shRNA clones. White arrows show filopodia. **F.** Lovo lysates were immunoprecipitated with SSH1 or irrelevant antibodies (IP) followed by western-blot with paladin antibodies (WB).

**Supplemental figure S5: Paladin interacts with slingshot homolog-1 and regulates phosphorylation level of actin remodelling regulators in LS174T. A.** Paladin and serine 3 phospho-cofilin western-blot in LS174T shRNA clones. **B.** mTOR and serine 2481 phospho-mTOR western-blot in LS174T shRNA clones. **C.** Akt and serine 473 phospho-AKT western-blot in LS174T shRNA clones. **D.** SSH1 western-blot in LS174T shRNA clones. HSC70 was used as loading control. **E.** Actin staining with fluorescent phalloidin in Lovo shRNA clones. White arrows show filopodia. **F.** LS174 lysates were immunoprecipitated with SSH1 or irrelevant antibodies (IP) followed by western-blot with paladin antibodies (WB).
