# Supplementary figures and images for: Paladin, overexpressed in colon cancer, is required for actin polymerisation and liver metastasis dissemination
Source: Oncogenesis. 2022 Jul 26;11(1):42. doi: 10.1038/s41389-022-00416-4 (PMC9325978; doi:10.1038/s41389-022-00416-4)

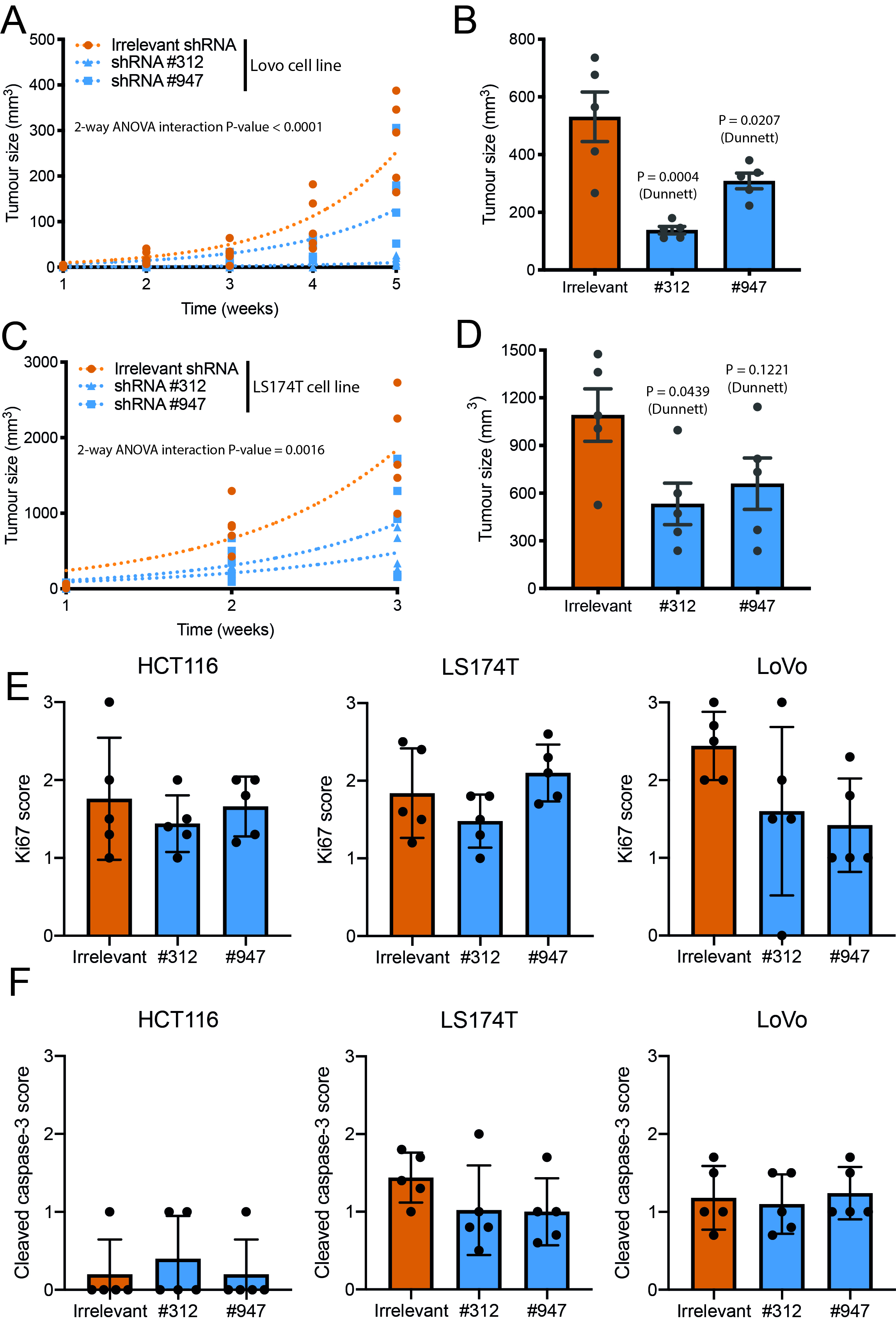

Supplement: Supplementary file 2 — Supplemental figure 1 [file 41389_2022_416_MOESM2_ESM.jpg]

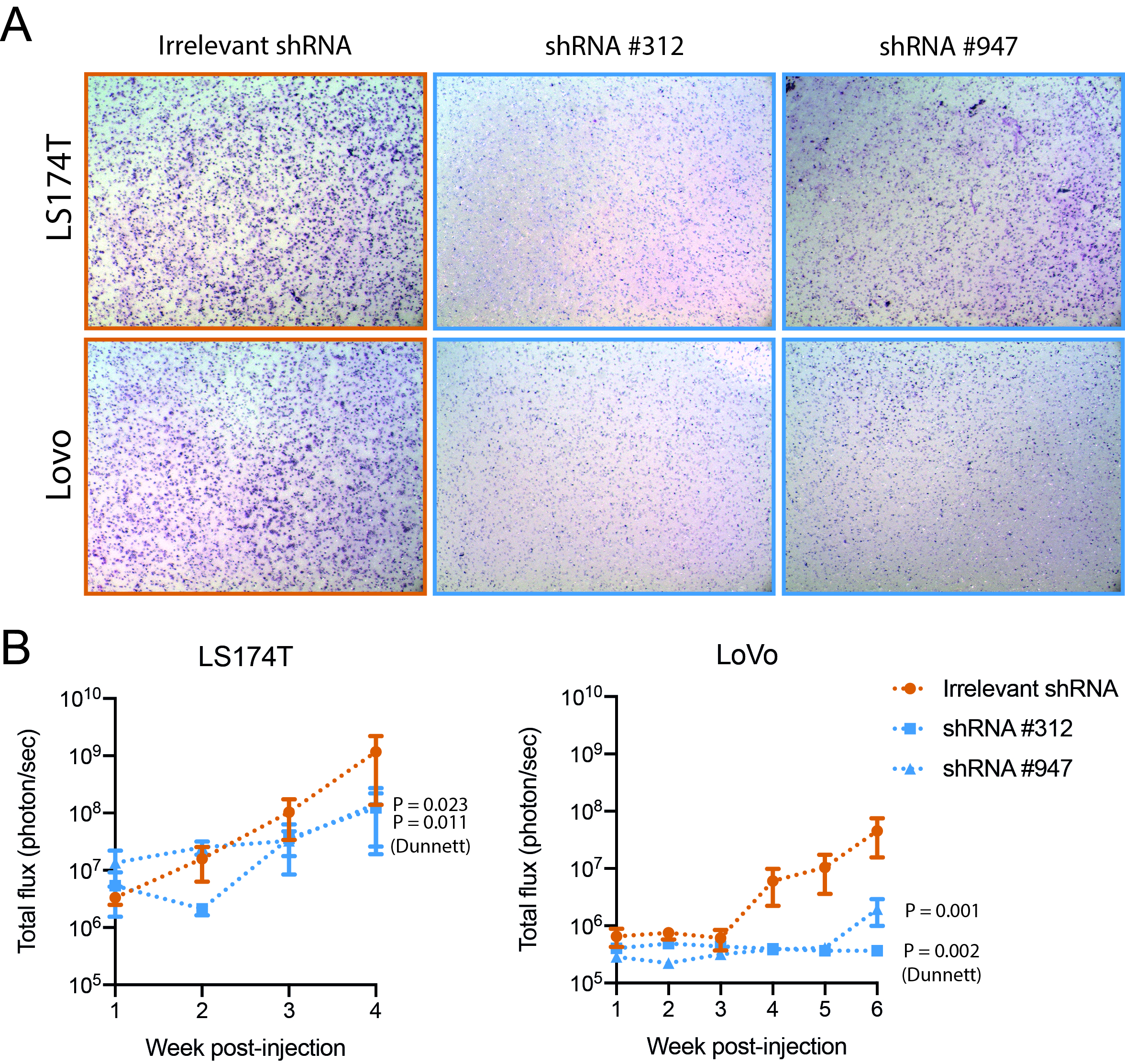

Supplement: Supplementary file 3 — Supplemental figure 2 [file 41389_2022_416_MOESM3_ESM.jpg]

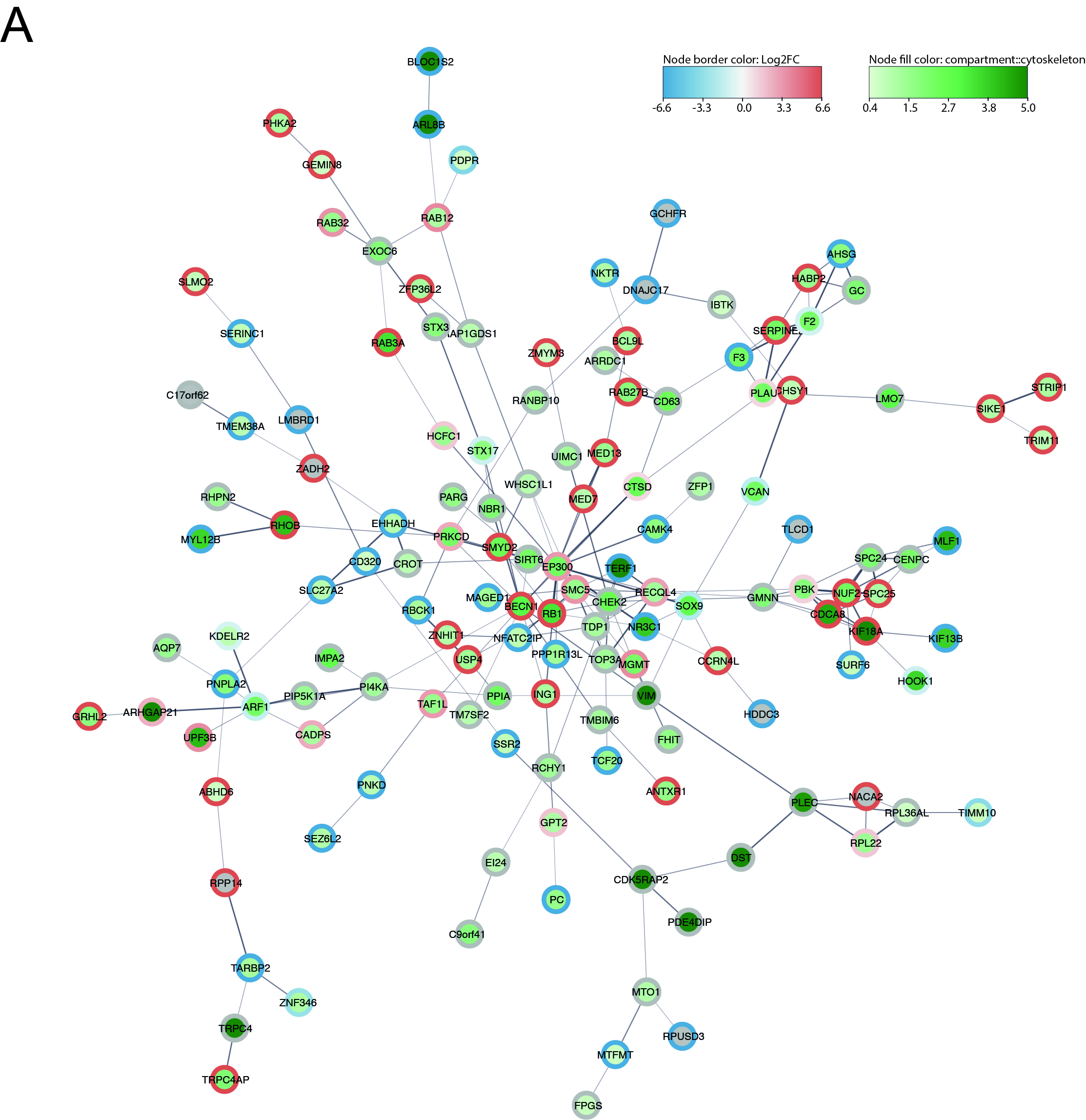

Supplement: Supplementary file 4 — Supplemental figure 3 [file 41389_2022_416_MOESM4_ESM.jpg]

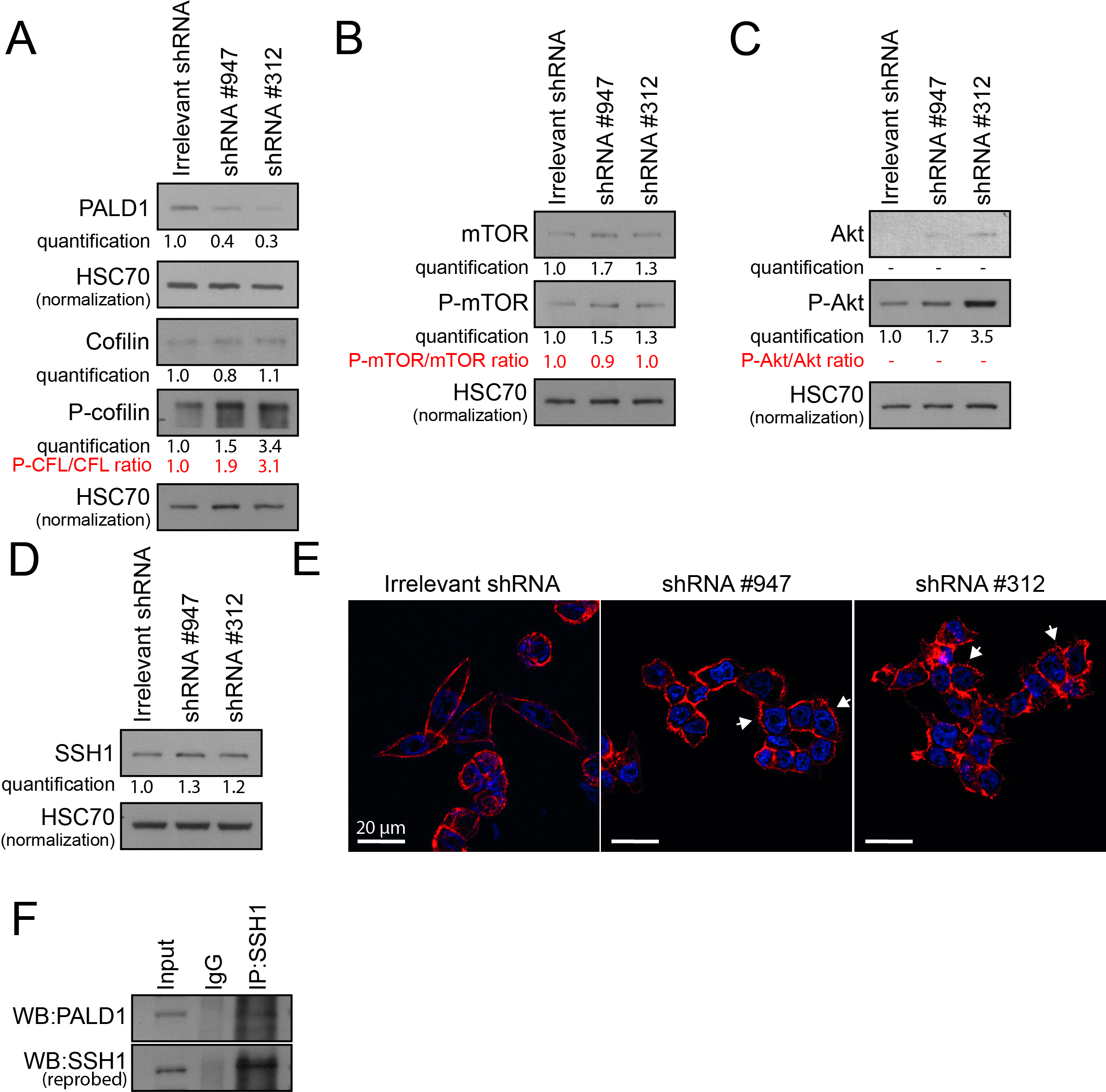

Supplement: Supplementary file 5 — Supplemental figure 4 [file 41389_2022_416_MOESM5_ESM.jpg]

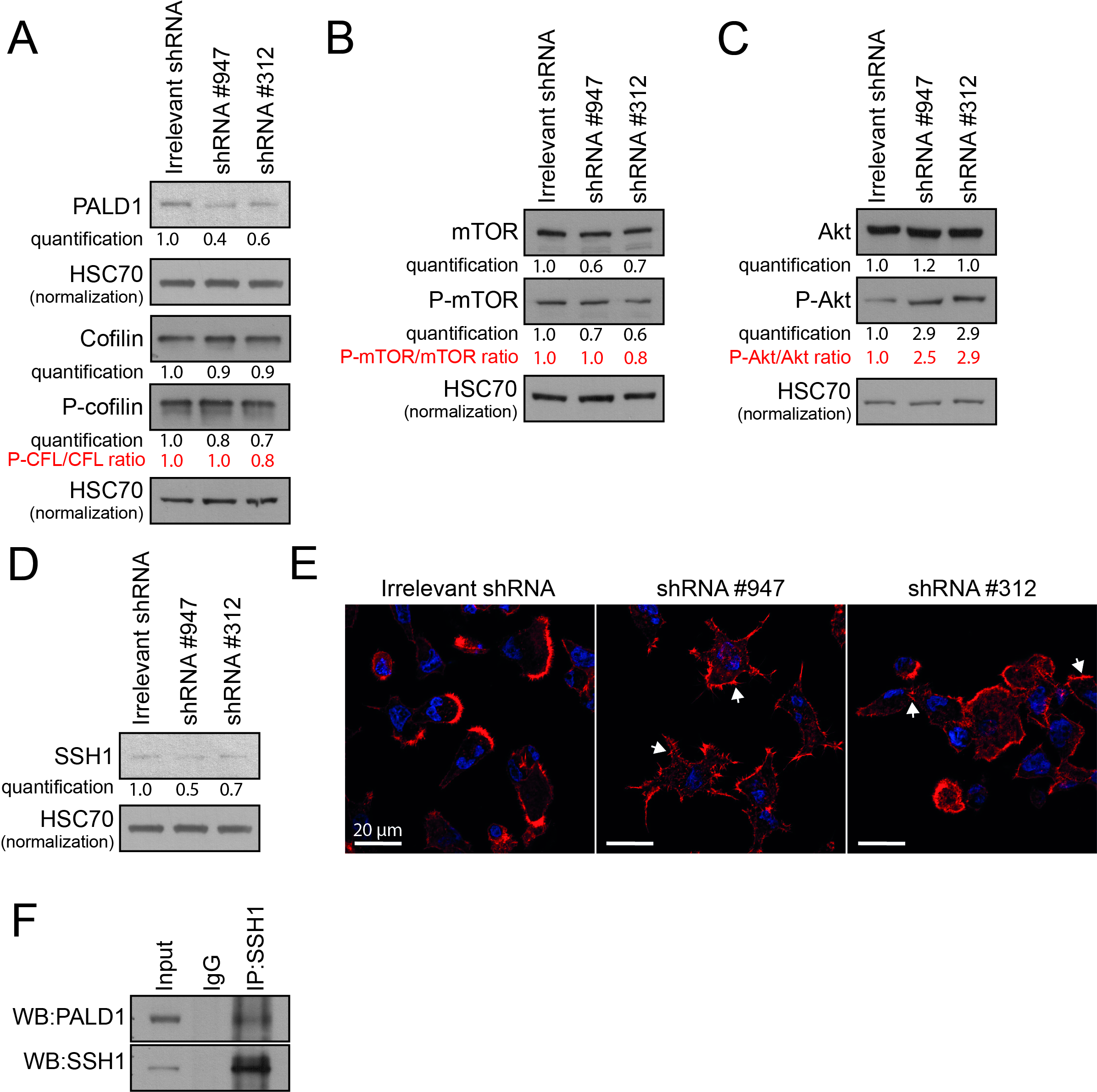

Supplement: Supplementary file 6 — Supplemental figure 5 [file 41389_2022_416_MOESM6_ESM.jpg]
