# Supplementary material for: Paladin, overexpressed in colon cancer, is required for actin polymerisation and liver metastasis dissemination
Source: Oncogenesis. 2022 Jul 26;11(1):42. doi: 10.1038/s41389-022-00416-4 (PMC9325978; doi:10.1038/s41389-022-00416-4)
Supplement: Supplementary file 7 — Supplemental table 1 [file 41389_2022_416_MOESM7_ESM.pdf]

Accession	Abundance Ratio (sh312)/(shNT)	Log FC2	padj
E9PPU0	0.01	-6.64385619	1.27E-16
Q8TDD1-2	0.01	-6.64385619	1.27E-16
O14950	0.01	-6.64385619	1.27E-16
P16989-2	0.01	-6.64385619	1.27E-16
Q567Q0	0.01	-6.64385619	1.27E-16
O14715	0.01	-6.64385619	1.27E-16
Q29963	0.01	-6.64385619	1.27E-16
P08670	0.01	-6.64385619	1.27E-16
Q14141	0.01	-6.64385619	1.27E-16
Q9BZG1	0.01	-6.64385619	1.27E-16
A6NHR9	0.01	-6.64385619	1.27E-16
F8VWZ8	0.01	-6.64385619	1.27E-16
Q8WUD1	0.01	-6.64385619	1.27E-16
Q96AE7	0.01	-6.64385619	1.27E-16
F5H594	0.01	-6.64385619	1.27E-16
Q9Y2T3	0.01	-6.64385619	1.27E-16
Q96HN2	0.01	-6.64385619	1.27E-16
P84157	0.01	-6.64385619	1.27E-16
K7EJV3	0.01	-6.64385619	1.27E-16
P84157-2	0.01	-6.64385619	1.27E-16
P13726	0.01	-6.64385619	1.27E-16
Q5JUW8	0.01	-6.64385619	1.27E-16
Q07812	0.01	-6.64385619	1.27E-16
Q9NVJ2	0.01	-6.64385619	1.27E-16
O14841	0.01	-6.64385619	1.27E-16
Q13043	0.01	-6.64385619	1.27E-16
E9PMR6	0.01	-6.64385619	1.27E-16
Q14UF6	0.01	-6.64385619	1.27E-16
Q9BTV5	0.01	-6.64385619	1.27E-16
F5H2X7	0.01	-6.64385619	1.27E-16
Q5JSZ5	0.01	-6.64385619	1.27E-16
P63218	0.01	-6.64385619	1.27E-16
Q9BQS8	0.01	-6.64385619	1.27E-16
H0YMW2	0.01	-6.64385619	1.27E-16
Q969Q0	0.01	-6.64385619	1.27E-16
F8WBZ2	0.01	-6.64385619	1.27E-16
F8W7V0	0.01	-6.64385619	1.27E-16
Q96L91	0.01	-6.64385619	1.27E-16
Q9Y4I1	0.01	-6.64385619	1.27E-16
O76041-2	0.01	-6.64385619	1.27E-16
B4DQH9	0.01	-6.64385619	1.27E-16
P36959	0.01	-6.64385619	1.27E-16
Q6QNY1	0.01	-6.64385619	1.27E-16
P63146	0.01	-6.64385619	1.27E-16

F8W1Z6	0.01	-6.64385619	1.27E-16
P11498	0.01	-6.64385619	1.27E-16
P21397	0.01	-6.64385619	1.27E-16
Q15633	0.01	-6.64385619	1.27E-16
Q86TV2	0.01	-6.64385619	1.27E-16
Q9NUY8	0.01	-6.64385619	1.27E-16
Q92506	0.01	-6.64385619	1.27E-16
P02795	0.01	-6.64385619	1.27E-16
Q9H0X4	0.01	-6.64385619	1.27E-16
Q96BH1	0.01	-6.64385619	1.27E-16
H7C3Y7	0.01	-6.64385619	1.27E-16
Q8IU85	0.01	-6.64385619	1.27E-16
Q9NZD8	0.01	-6.64385619	1.27E-16
F8W9A1	0.01	-6.64385619	1.27E-16
Q9UII4	0.01	-6.64385619	1.27E-16
Q8NEM2	0.01	-6.64385619	1.27E-16
Q8WXF0	0.01	-6.64385619	1.27E-16
P17252	0.01	-6.64385619	1.27E-16
O96011	0.01	-6.64385619	1.27E-16
E9PJW9	0.01	-6.64385619	1.27E-16
Q5QPD4	0.01	-6.64385619	1.27E-16
Q9UKA4	0.01	-6.64385619	1.27E-16
Q8NCF5	0.01	-6.64385619	1.27E-16
O14975	0.01	-6.64385619	1.27E-16
Q8WUF5	0.01	-6.64385619	1.27E-16
Q9Y5V3	0.01	-6.64385619	1.27E-16
Q9NQT8	0.01	-6.64385619	1.27E-16
B5MEC7	0.01	-6.64385619	1.27E-16
C9JVP0	0.01	-6.64385619	1.27E-16
Q96NB2	0.01	-6.64385619	1.27E-16
Q9NRX5	0.01	-6.64385619	1.27E-16
P58340	0.01	-6.64385619	1.27E-16
E9PLL1	0.01	-6.64385619	1.27E-16
Q9H4H8	0.01	-6.64385619	1.27E-16
O75362	0.01	-6.64385619	1.27E-16
E9PMT2	0.01	-6.64385619	1.27E-16
O14734	0.01	-6.64385619	1.27E-16
Q9NVM6	0.01	-6.64385619	1.27E-16
Q9P1U0	0.01	-6.64385619	1.27E-16
E9PBR5	0.01	-6.64385619	1.27E-16
P98172	0.01	-6.64385619	1.27E-16
C9JSR1	0.01	-6.64385619	1.27E-16
Q8N4P3	0.01	-6.64385619	1.27E-16
P53801	0.01	-6.64385619	1.27E-16
Q04721	0.01	-6.64385619	1.27E-16
B4DUS7	0.01	-6.64385619	1.27E-16

P56937	0.01	-6.64385619	1.27E-16
Q6UXD5	0.01	-6.64385619	1.27E-16
Q5VZZ6	0.01	-6.64385619	1.27E-16
P30047	0.01	-6.64385619	1.27E-16
P30414	0.01	-6.64385619	1.27E-16
H7C4Z8	0.01	-6.64385619	1.27E-16
B7Z588	0.01	-6.64385619	1.27E-16
Q8IZV2	0.01	-6.64385619	1.27E-16
P25445	0.01	-6.64385619	1.27E-16
P29558	0.01	-6.64385619	1.27E-16
Q13433	0.01	-6.64385619	1.27E-16
Q5TFQ8	0.01	-6.64385619	1.27E-16
Q9NV96	0.01	-6.64385619	1.27E-16
Q9NUN5	0.01	-6.64385619	1.27E-16
E2QRF9	0.01	-6.64385619	1.27E-16
Q13432	0.01	-6.64385619	1.27E-16
E9PQV6	0.01	-6.64385619	1.27E-16
Q96RL1	0.01	-6.64385619	1.27E-16
Q96CP7	0.01	-6.64385619	1.27E-16
Q96BJ3	0.01	-6.64385619	1.27E-16
D6R9A1	0.01	-6.64385619	1.27E-16
Q96DP5	0.01	-6.64385619	1.27E-16
P0C7P0	0.01	-6.64385619	1.27E-16
J3KNM5	0.01	-6.64385619	1.27E-16
Q12986	0.01	-6.64385619	1.27E-16
E9PL24	0.01	-6.64385619	1.27E-16
E7EW84	0.01	-6.64385619	1.27E-16
Q7Z309	0.01	-6.64385619	1.27E-16
B1AMB1	0.01	-6.64385619	1.27E-16
P02765	0.01	-6.64385619	1.27E-16
Q9Y4U1	0.01	-6.64385619	1.27E-16
Q86V85	0.01	-6.64385619	1.27E-16
P04150	0.01	-6.64385619	1.27E-16
Q9BSY9	0.01	-6.64385619	1.27E-16
E5RIL0	0.01	-6.64385619	1.27E-16
Q9H6F2	0.01	-6.64385619	1.27E-16
F2Z2Q4	0.01	-6.64385619	1.27E-16
E7EWX0	0.01	-6.64385619	1.27E-16
P43308	0.01	-6.64385619	1.27E-16
Q9UP95	0.01	-6.64385619	1.27E-16
H0YAI4	0.01	-6.64385619	1.27E-16
Q9BYM8	0.01	-6.64385619	1.27E-16
P25686	0.01	-6.64385619	1.27E-16
Q5JXI8	0.01	-6.64385619	1.27E-16
Q9NVA4	0.01	-6.64385619	1.27E-16
Q08426	0.01	-6.64385619	1.27E-16

Q8N490-2	0.01	-6.64385619	1.27E-16
P28799	0.01	-6.64385619	1.27E-16
A6PW57	0.01	-6.64385619	1.27E-16
Q86YH6	0.01	-6.64385619	1.27E-16
H0Y6R1	0.01	-6.64385619	1.27E-16
E7EMP9	0.01	-6.64385619	1.27E-16
F5H2M4	0.01	-6.64385619	1.27E-16
B4DDV3	0.01	-6.64385619	1.27E-16
Q6P087	0.01	-6.64385619	1.27E-16
Q9H9A7	0.01	-6.64385619	1.27E-16
Q63HN8	0.01	-6.64385619	1.27E-16
H0Y432	0.01	-6.64385619	1.27E-16
C9JE56	0.01	-6.64385619	1.27E-16
Q9BRT3	0.01	-6.64385619	1.27E-16
Q5TIH2	0.01	-6.64385619	1.27E-16
E9PM12	0.01	-6.64385619	1.27E-16
Q8TCU4	0.01	-6.64385619	1.27E-16
Q8IWK6	0.01	-6.64385619	1.27E-16
Q9UGU0	0.01	-6.64385619	1.27E-16
P50151	0.01	-6.64385619	1.27E-16
Q9UBT7	0.01	-6.64385619	1.27E-16
P29474	0.01	-6.64385619	1.27E-16
Q9H8P0	0.01	-6.64385619	1.27E-16
Q9BTT4	0.01	-6.64385619	1.27E-16
Q86VX2	0.01	-6.64385619	1.27E-16
H0Y8J2	0.01	-6.64385619	1.27E-16
Q96AD5	0.01	-6.64385619	1.27E-16
F5H7N9	0.01	-6.64385619	1.27E-16
Q9NRG1	0.01	-6.64385619	1.27E-16
Q5CZA5	0.01	-6.64385619	1.27E-16
F8WE49	0.01	-6.64385619	1.27E-16
P23470	0.01	-6.64385619	1.27E-16
Q96AT1	0.01	-6.64385619	1.27E-16
Q9NQG1	0.01	-6.64385619	1.27E-16
P05060	0.01	-6.64385619	1.27E-16
E9PBZ0	0.01	-6.64385619	1.27E-16
H0YC74	0.01	-6.64385619	1.27E-16
Q16566	0.01	-6.64385619	1.27E-16
Q09013-1	0.01	-6.64385619	1.27E-16
Q14207	0.01	-6.64385619	1.27E-16
Q0P670	0.01	-6.64385619	1.27E-16
J3QRG5	0.01	-6.64385619	1.27E-16
Q5T764	0.01	-6.64385619	1.27E-16
Q5JXR6	0.01	-6.64385619	1.27E-16
P38570	0.01	-6.64385619	1.27E-16
Q5T371	0.01	-6.64385619	1.27E-16

Q8NE63	0.01	-6.64385619	1.27E-16
Q9H0F7	0.01	-6.64385619	1.27E-16
Q9NZJ0	0.01	-6.64385619	1.27E-16
B4DWZ5	0.01	-6.64385619	1.27E-16
P48745	0.01	-6.64385619	1.27E-16
Q9NPF0	0.01	-6.64385619	1.27E-16
B7Z3P4	0.01	-6.64385619	1.27E-16
C9J3D7	0.01	-6.64385619	1.27E-16
Q96SN8	0.01	-6.64385619	1.27E-16
F5H5A3	0.01	-6.64385619	1.27E-16
Q5VT25	0.01	-6.64385619	1.27E-16
Q9Y5H6	0.01	-6.64385619	1.27E-16
O75683	0.01	-6.64385619	1.27E-16
P54274	0.01	-6.64385619	1.27E-16
Q7Z5Y7	0.01	-6.64385619	1.27E-16
Q5VYS8	0.023	-5.442222329	1.27E-16
B5MCQ5	0.05	-4.321928095	1.27E-16
Q8NCN5	0.063	-3.988504361	1.27E-16
P62072	0.085	-3.556393349	1.27E-16
Q15149-3	0.088	-3.506352666	1.27E-16
B4E3K2	0.104	-3.265344567	1.27E-16
Q96D05	0.109	-3.19759996	1.27E-16
Q9NX61	0.129	-2.954557029	1.27E-16
A8MUB1	0.227	-2.139235797	1.27E-16
Q15847	6.91	2.788685711	1.27E-16
C9J236	9.03	3.174725988	1.27E-16
Q9Y580	11.441	3.516141251	1.27E-16
Q13637	11.549	3.529696032	1.27E-16
Q86YN1	11.769	3.556919836	1.27E-16
Q9NWS0	13.292	3.732486293	1.27E-16
P16455	14.184	3.826192536	1.27E-16
B0QY16	14.209	3.828733119	1.27E-16
G5E954	14.779	3.88547675	1.27E-16
P52306-3	100	6.64385619	1.27E-16
E7ETY2	100	6.64385619	1.27E-16
J3KPD3	100	6.64385619	1.27E-16
P62745	100	6.64385619	1.27E-16
F8W9J4	100	6.64385619	1.27E-16
Q9NXK8	100	6.64385619	1.27E-16
A8MYB8	100	6.64385619	1.27E-16
Q13107	100	6.64385619	1.27E-16
P20336	100	6.64385619	1.27E-16
Q9NVU0	100	6.64385619	1.27E-16
Q53EL6	100	6.64385619	1.27E-16
J3KNG9	100	6.64385619	1.27E-16
Q5T085	100	6.64385619	1.27E-16

Q9HA77	100	6.64385619	1.27E-16
O14578	100	6.64385619	1.27E-16
A6NF48	100	6.64385619	1.27E-16
Q9NZT1	100	6.64385619	1.27E-16
Q86Y37	100	6.64385619	1.27E-16
Q14202	100	6.64385619	1.27E-16
Q5VSL9	100	6.64385619	1.27E-16
P06400	100	6.64385619	1.27E-16
Q9Y535	100	6.64385619	1.27E-16
Q8NI77	100	6.64385619	1.27E-16
K7ESQ2	100	6.64385619	1.27E-16
O95059	100	6.64385619	1.27E-16
H3BRQ0	100	6.64385619	1.27E-16
Q86UU0	100	6.64385619	1.27E-16
Q9UK39	100	6.64385619	1.27E-16
Q9UK53	100	6.64385619	1.27E-16
H7C0U0	100	6.64385619	1.27E-16
Q8TEL6	100	6.64385619	1.27E-16
Q8WXC6	100	6.64385619	1.27E-16
G5E928	100	6.64385619	1.27E-16
P18827	100	6.64385619	1.27E-16
Q9NRD1	100	6.64385619	1.27E-16
Q8N655	100	6.64385619	1.27E-16
B0QY29	100	6.64385619	1.27E-16
Q9NRG4	100	6.64385619	1.27E-16
O96017	100	6.64385619	1.27E-16
Q9HBM1	100	6.64385619	1.27E-16
Q8IWE4	100	6.64385619	1.27E-16
P31151	100	6.64385619	1.27E-16
O43513	100	6.64385619	1.27E-16
B3KWW1	100	6.64385619	1.27E-16
P17036	100	6.64385619	1.27E-16
Q9Y653	100	6.64385619	1.27E-16
Q8NAF0	100	6.64385619	1.27E-16
J3KTD2	100	6.64385619	1.27E-16
Q9BRV8	100	6.64385619	1.27E-16
Q8IUH3	100	6.64385619	1.27E-16
P47974	100	6.64385619	1.27E-16
A8K040	100	6.64385619	1.27E-16
Q6PIP6	100	6.64385619	1.27E-16
H7C3Q6	100	6.64385619	1.27E-16
O43257	100	6.64385619	1.27E-16
Q14520	100	6.64385619	1.27E-16
Q9UGT4	100	6.64385619	1.27E-16
Q9NXH8	100	6.64385619	1.27E-16
H0YK69	100	6.64385619	1.27E-16

Q15776	100	6.64385619	1.27E-16
Q96RW7	100	6.64385619	1.27E-16
Q5T5L3	100	6.64385619	1.27E-16
Q9NWZ8	100	6.64385619	1.27E-16
P62699	100	6.64385619	1.27E-16
J3KNQ1	100	6.64385619	1.27E-16
Q9Y3B1	100	6.64385619	1.27E-16
O00418	100	6.64385619	1.27E-16
B7ZM03	100	6.64385619	1.27E-16
Q9UHV7	100	6.64385619	1.27E-16
Q6ISB3	100	6.64385619	1.27E-16
Q14457	100	6.64385619	1.27E-16
Q9H009	100	6.64385619	1.27E-16
Q8N4Q0	100	6.64385619	1.27E-16
H7C1J4	100	6.64385619	1.27E-16
Q7L8J4	100	6.64385619	1.27E-16
E9PM05	100	6.64385619	1.27E-16
H0Y5M2	100	6.64385619	1.27E-16
Q8N0Y2	100	6.64385619	1.27E-16
H0YCR6	100	6.64385619	1.27E-16
O00194	100	6.64385619	1.27E-16
Q5JU23	100	6.64385619	1.27E-16
Q9H6X2	100	6.64385619	1.27E-16
Q96K31	100	6.64385619	1.27E-16
Q9BZD4	100	6.64385619	1.27E-16
Q86X52	100	6.64385619	1.27E-16
B4DWA3	100	6.64385619	1.27E-16
E7EQ48	100	6.64385619	1.27E-16
Q96F44-2	100	6.64385619	1.27E-16
E7EPD8	100	6.64385619	1.27E-16
C9JGQ0	100	6.64385619	1.27E-16
P46019	100	6.64385619	1.27E-16
Q9BQE9	100	6.64385619	1.27E-16
Q9UBN4	100	6.64385619	1.27E-16
P07093-3	100	6.64385619	1.27E-16
Q6ZN44	100	6.64385619	1.27E-16
H0Y5P8	100	6.64385619	1.27E-16
E9PAS0	100	6.64385619	1.27E-16
Q8NDT2	100	6.64385619	1.27E-16
J3KN10	100	6.64385619	1.27E-16
E9PLX2	100	6.64385619	1.27E-16
Q69YL0	100	6.64385619	1.27E-16
Q53HL2	100	6.64385619	1.27E-16
Q9BV23	100	6.64385619	1.27E-16
Q53RE8	100	6.64385619	1.27E-16
K7ESE5	100	6.64385619	1.27E-16

E9PFR0	100	6.64385619	1.27E-16
E7EPI0	100	6.64385619	1.27E-16
Q9H799	100	6.64385619	1.27E-16
O00622	0.144	-2.795859283	2.80E-15
P35268	3.244	1.69777382	2.80E-15
Q9NSA3	10.021	3.324954578	5.60E-15
Q8IYT2	12.724	3.669480372	5.60E-15
B4DSD4	0.06	-4.058893689	8.36E-15
J3KMX4	0.091	-3.457989644	1.11E-14
O76070	5.085	2.346247774	1.11E-14
Q6IQ22	14.909	3.898111589	1.11E-14
F5H0B5	0.137	-2.867752202	2.50E-14
F8W9Y0	0.098	-3.351074441	4.98E-14
P56589	0.112	-3.158429363	5.79E-14
Q5JWV1	0.099	-3.336427665	1.02E-13
O60635	0.101	-3.307572802	1.13E-13
F8VSI7	6.555	2.71259578	1.39E-13
O94761	8.278	3.049282249	1.61E-13
P86790	10.547	3.39876079	1.80E-13
Q9NZI8	0.105	-3.251538767	2.53E-13
Q86W56	0.1	-3.321928095	2.55E-13
Q9P0S3	10.214	3.352476059	3.50E-13
Q9HAS0	0.124	-3.011587974	3.93E-13
Q9HBU6	3.454	1.788268083	4.49E-13
Q9UQR0	0.1	-3.321928095	8.79E-13
H3BTK5	0.083	-3.590744853	2.19E-12
P05091	0.288	-1.795859283	2.19E-12
P11166	2.95	1.560714954	2.86E-12
P25054	0.071	-3.816037165	2.97E-12
Q9UHK0	0.083	-3.590744853	4.32E-12
P19971	0.174	-2.522840789	5.06E-12
O60687	0.07	-3.836501268	6.41E-12
P26447	2.938	1.554834396	8.06E-12
Q8N488	9.131	3.190772869	1.65E-11
Q96DE0	0.137	-2.867752202	1.70E-11
Q05655	6.486	2.697329024	2.27E-11
Q6DT37	0.113	-3.145605322	2.29E-11
Q9BZI7	13.61	3.766595162	2.42E-11
Q16822	0.275	-1.862496476	2.80E-11
Q8TD30	3.606	1.850399397	3.10E-11
Q09472	10.021	3.324954578	4.02E-11
Q9BZ95	11.631	3.539903236	5.36E-11
Q5T447	0.078	-3.680382066	5.53E-11
F5H8A0	0.12	-3.058893689	6.99E-11
B4DK80	10.257	3.358536923	1.59E-10
Q8IZX4	9.711	3.279619866	2.09E-10

Q14517	0.358	-1.481968507	2.35E-10
Q68DQ2	11.527	3.526945183	5.31E-10
H0YMQ3	0.124	-3.011587974	5.52E-10
Q8IY18	11.384	3.508935662	6.61E-10
Q9HCJ0	0.145	-2.785875195	6.66E-10
G3XAA0	9.884	3.305095011	7.29E-10
Q9Y3D5	0.135	-2.888968688	9.03E-10
P13611	0.289	-1.790858602	1.22E-09
P78540	0.144	-2.795859283	1.81E-09
Q9BSJ5	10.38	3.375734539	2.09E-09
Q8NG68	10.179	3.347523931	2.29E-09
Q9ULJ3	9.157	3.194875023	2.83E-09
Q5T5U3	5.497	2.458644479	4.19E-09
Q30201	9.97	3.317593505	8.42E-09
P29992	0.311	-1.685013515	9.03E-09
Q9UL40	0.113	-3.145605322	9.95E-09
P29692-3	0.391	-1.354759487	1.04E-08
Q8IY63	0.115	-3.120294234	1.07E-08
G8JLJ4	0.123	-3.023269779	1.42E-08
F5H8J3	2.545	1.347665656	1.89E-08
Q9P0J1	2.425	1.277984747	5.65E-08
Q969L2	3.919	1.970485573	1.06E-07
Q12774	0.121	-3.046921047	1.27E-07
P31949	0.422	-1.244685096	2.32E-07
Q96F24	0.151	-2.727379545	3.38E-07
Q96KB5	2.453	1.294547234	7.08E-07
Q9ULU8	6.278	2.650305029	9.29E-07
P04632	2.153	1.10634832	1.17E-06
Q58FF6	2.215	1.147306699	1.51E-06
O75844	0.414	-1.272297327	4.11E-06
Q6WRX3-2	0.222	-2.171368418	4.32E-06
P10253	0.451	-1.148800661	5.25E-06
E7ER89	0.305	-1.713118852	6.40E-06
P16070-4	2.224	1.153156788	7.02E-06
P31350	0.478	-1.064917477	8.32E-06
P02768	2.07	1.049630768	1.20E-05
P09601	2.644	1.402722177	1.31E-05
P07339	2.076	1.053806444	1.52E-05
Q6ZMU5	2.374	1.247319935	1.59E-05
P48436	0.232	-2.10780329	2.31E-05
Q9BY89	0.447	-1.161653263	3.58E-05
Q01081-2	0.297	-1.751465164	3.66E-05
P31321	3.497	1.806117796	4.00E-05
P67936-2	3.164	1.6617496	5.30E-05
P47895	2.053	1.037733627	5.40E-05
P10515	0.455	-1.13606155	5.70E-05

Q9H1B7	2.17	1.117695043	6.01E-05
Q9BWM7	0.399	-1.325539348	6.32E-05
Q5RHS7	2.842	1.506906555	6.67E-05
C9JRL4	2.144	1.100304906	6.90E-05
Q9Y6X9	2.99	1.580145484	7.10E-05
Q96CX6	3.503	1.808590988	7.10E-05
P15559	1.954	0.966430467	7.55E-05
P00734	0.45	-1.152003093	8.90E-05
D6RF35	3.222	1.687956494	9.09E-05
P84077	0.34	-1.556393349	0.00010928
Q9Y696	0.443	-1.174621396	0.00013116
Q07960	0.473	-1.080087911	0.00014568
C9JYQ9	0.515	-0.957355663	0.00017413
P08582	0.202	-2.307572802	0.00019388
P02786	1.88	0.910732662	0.00023828
P26583	1.876	0.907659828	0.00025548
P54652	0.302	-1.727379545	0.00030333
Q8IV38	2.633	1.396707521	0.00034617
P20290-2	0.368	-1.442222329	0.00035034
O94927	0.457	-1.12973393	0.00038742
Q6YHK3	2.144	1.100304906	0.00052234
Q9GZQ3	0.325	-1.621488377	0.00054274
P38646	0.538	-0.894321922	0.000589
K7ERL7	0.481	-1.055891201	0.00067446
P52292	1.811	0.856786546	0.00068048
G3V5J8	0.232	-2.10780329	0.00071957
P43357	0.36	-1.473931188	0.00073586
Q15642	0.477	-1.067938829	0.00074829
P13674	0.499	-1.002888279	0.00081987
Q86W92-2	0.538	-0.894321922	0.00086918
P63313	1.789	0.839153387	0.00094492
B4DYH1	0.508	-0.977099598	0.00103746
F5H538	2.901	1.536550296	0.00106512
G3V1X9	1.78	0.831877241	0.00110962
Q9UBQ0	2.756	1.462575888	0.00117233
P33947	0.493	-1.020340448	0.0014921
P51610	3.177	1.66766509	0.00167404
J3KS22	0.553	-0.854648614	0.00186903
O43175	0.56	-0.836501268	0.00186903
O00159	0.563	-0.828793173	0.00198744
C9JK39	2.129	1.09017595	0.0020476
Q99988	2.115	1.080657663	0.00244592
P04899	0.571	-0.808437349	0.00249093
Q9UJC3	0.405	-1.304006187	0.00260107
H0YIV9	0.418	-1.258425153	0.00271423
O60841	0.576	-0.795859283	0.00271423

Q9Y2V2	0.517	-0.951763814	0.00295612
Q9H6F5	1.837	0.877351626	0.00318481
F5H4K0	2.061	1.043344505	0.00320484
Q00765	0.54	-0.888968688	0.00323746
Q96S55	0.557	-0.844250767	0.00375366
O00629	1.717	0.779890039	0.00379211
P07099	0.547	-0.870387262	0.0042223
E7ENU4	0.59	-0.76121314	0.00440733
O75822	0.545	-0.875671865	0.00447122
P08243	0.586	-0.77102743	0.00455636
P51570	0.555	-0.849440323	0.00486097
Q14192	0.512	-0.965784285	0.00492134
O95336	0.586	-0.77102743	0.00535812
H7C061	1.801	0.848798181	0.005573
P30455	0.588	-0.76611194	0.00617146
Q9HCY8	1.782	0.833497337	0.00658141
B4DTU4	1.852	0.889084099	0.00662172
P29373	1.605	0.682573297	0.00673761
F5H233	2.548	1.349365278	0.0075081
Q9Y3E2	0.317	-1.657445255	0.00800311
Q9NVS9	0.59	-0.76121314	0.00801138
Q13263	1.637	0.711054322	0.00854817
Q8TEM1	1.638	0.711935357	0.0087279
O00151	0.553	-0.854648614	0.00883309
Q9NZL4	1.736	0.795766948	0.00900569
Q5T2T1	1.935	0.952333566	0.0090207
Q9NZN4	0.561	-0.833927324	0.00912767
C9IYK9	0.447	-1.161653263	0.00946341
O60725	0.363	-1.461958547	0.00954951
Q96HP0	0.389	-1.36215794	0.00967901
Q9P258	0.607	-0.720231578	0.00986044
Q8N392	1.726	0.787432465	0.01011068
Q5SSJ5	0.603	-0.729770093	0.0101942
Q16854	2.693	1.42921423	0.0112147
E9PGM1	1.617	0.693319679	0.01126799
Q9H4G0	2.316	1.211635253	0.01153674
P16949	1.607	0.684369929	0.01271798
Q01813	0.61	-0.713118852	0.01295931
Q15004	0.473	-1.080087911	0.01322437
P48637	0.602	-0.732164608	0.01370761
F8VWK8	0.471	-1.086201035	0.01377466
P51648	1.682	0.750177706	0.01484503
P37802	0.615	-0.701341684	0.01556404
Q14108	0.602	-0.732164608	0.01601654
O95239	1.676	0.745022149	0.01609081
Q9GZP4	1.849	0.886745225	0.01655778

Q15269	0.604	-0.727379545	0.01700296
Q9UPZ3	0.318	-1.652901329	0.01729239
P46013	1.578	0.658097205	0.01873493
Q96JM7	2.07	1.049630768	0.01941652
P02794	1.752	0.809002775	0.01962866
Q04941	0.622	-0.685013515	0.01966033
F5H2F6	0.444	-1.171368418	0.02030205
Q14061	0.562	-0.831357964	0.02051724
O94952-1	0.453	-1.142417045	0.02067803
P08238	1.57	0.650764559	0.02073629
P48200	2.477	1.308593869	0.02106548
Q03135	0.592	-0.756330919	0.02106837
P45954	0.618	-0.694321257	0.02127918
Q9Y5I4	0.584	-0.775959726	0.02158863
E9PHQ0	1.563	0.644317778	0.02210858
P33316	1.664	0.734655433	0.02284
Q7Z5L9	0.468	-1.095419565	0.02532686
Q03519	0.446	-1.164884385	0.02590627
P49588	0.631	-0.66428809	0.02639611
Q8N5K1	1.664	0.734655433	0.02686502
Q13257	1.652	0.724213687	0.02719731
Q562R1	0.587	-0.768567592	0.02791201
P49915	1.547	0.629473197	0.02798317
Q96HC4	0.612	-0.708396442	0.02829386
P23381	0.634	-0.657445255	0.02859564
P29966	0.606	-0.722610301	0.02873141
P49841	1.991	0.993493221	0.02885968
D4QA03	1.607	0.684369929	0.02954542
P50897	1.757	0.813114191	0.02957395
O60488	0.583	-0.778432211	0.03013612
P51572	0.639	-0.646112164	0.03141477
Q9UII2	0.467	-1.098505545	0.03201073
Q13287	2.409	1.268434394	0.03202106
P37235	1.615	0.691534165	0.03253615
O00233	1.644	0.717210299	0.03327466
Q9H0L4	0.474	-1.077041036	0.03355226
P23634	0.424	-1.23786383	0.03496845
P00749	2.235	1.160274831	0.03563127
Q14011	1.556	0.63784206	0.03609363
O00410	1.523	0.606915942	0.03797097
Q02952	0.644	-0.634867407	0.0386169
P48507	1.548	0.630405471	0.03900411
P32322	0.654	-0.612637459	0.03933289
O00116	0.615	-0.701341684	0.04337833
Q9H0H5	1.666	0.736388401	0.04386379
P01023	1.943	0.958285901	0.04430527

P30740	0.644	-0.634867407	0.0454046
Q9P086	2.325	1.217230716	0.04584189
B4DFR2	1.989	0.992043276	0.04629298
Q9BV81	0.474	-1.077041036	0.04726831
O75629	0.521	-0.940644722	0.04830526
P30483	0.626	-0.675765438	0.04962938
O95347	1.558	0.639695233	0.05035553
Q13630	0.626	-0.675765438	0.05084535
P13995	0.623	-0.682695932	0.0509079
P52907	0.656	-0.60823228	0.05099975
Q14126	1.506	0.59072177	0.05112894
Q9C004	1.925	0.944858446	0.05112894
Q86Y79	0.495	-1.01449957	0.05185489
P50336	2.502	1.32308179	0.05305798
B4E241	0.651	-0.619270551	0.05312083
A1XBS5	2.034	1.024319679	0.05312083
P15121	0.642	-0.639354798	0.05354359
Q13217	1.54	0.622930351	0.05517413
Q8NFJ5	0.526	-0.926865295	0.05539714
P09525	0.653	-0.614845103	0.05558969
Q15366-3	1.551	0.633198686	0.05692469
Q6ZN84	1.999	0.999278472	0.05737957
I3L3Y8	1.738	0.797428082	0.05750876
P00918	0.627	-0.673462652	0.05796907
P06703	0.658	-0.603840511	0.05826166
P62328	0.66	-0.59946207	0.05900128
Q13200	1.486	0.571434116	0.05948824
Q5JP53	1.484	0.569491092	0.06064352
P20248	0.473	-1.080087911	0.06076298
Q8NEJ9	1.7	0.765534746	0.06091575
P11047	0.603	-0.729770093	0.06280702
Q7Z7K6	1.707	0.771463058	0.06327711
I3L425	2.265	1.17951105	0.06356098
P07741	0.662	-0.595096878	0.0637867
P17987	1.48	0.565597176	0.06401357
Q9UJU6	0.636	-0.652901329	0.06450036
J3KPV3	0.506	-0.98279071	0.06578382
Q9H813	2.485	1.313245852	0.06590285
O75394	0.617	-0.696657606	0.06610926
O43414	1.835	0.875780063	0.06731312
P31327	0.487	-1.038006323	0.06809101
O14493	1.892	0.919912089	0.06832153
Q15366	1.824	0.86710573	0.06861814
Q658P3	0.515	-0.957355663	0.06893076
Q9NZ08	0.576	-0.795859283	0.06914762
O94855	0.628	-0.671163536	0.06914762

Q96QK1	0.665	-0.588573754	0.06914762
Q969M3	0.459	-1.123433941	0.06936459
Q92597	0.622	-0.685013515	0.06969812
Q15149	0.665	-0.588573754	0.06983226
P27144	0.631	-0.66428809	0.07076028
Q5JRX3	0.638	-0.648371671	0.07505257
Q6ZN57	0.388	-1.365871442	0.0751639
A8MY43	0.488	-1.035046947	0.0751639
P29034	1.867	0.900721928	0.0751639
Q9BX68	0.616	-0.698997744	0.07563628
E9PIE3	1.644	0.717210299	0.07609042
H7BXI1	0.627	-0.673462652	0.07691173
O00429-2	1.94	0.956056652	0.07710186
Q04446	0.632	-0.662003536	0.0771338
P18754	1.434	0.520045024	0.07720553
P05783	1.46	0.545968369	0.07858385
Q96EK9	0.541	-0.886299501	0.07863567
P04818	1.523	0.606915942	0.07937503
Q9Y4Y9	1.651	0.72334012	0.0797552
O75446	2.073	1.051720116	0.0811389
Q8TCT9	0.633	-0.659722595	0.08510486
Q5RI15	1.592	0.670840336	0.0861133
Q9BQ95	1.862	0.896853073	0.0861133
Q5J9I4	1.871	0.903809559	0.08628598
Q14697	0.632	-0.662003536	0.08638745
Q6ICB0	0.524	-0.932361283	0.08665286
Q96AB3	0.618	-0.694321257	0.08730937
Q15654	0.643	-0.637109357	0.08828636
E7EUI8	0.492	-1.023269779	0.08962348
O00764	0.663	-0.592919225	0.09127659
Q13177	1.455	0.541019153	0.09173437
Q9BUL8	1.505	0.589763487	0.09315858
Q9UGI8	0.676	-0.564904848	0.09420657
H0YNG3	1.512	0.59645814	0.09648606
E7EUC7	0.671	-0.575615328	0.09695119
Q5VVR8	2.118	1.082702589	0.09798948
Q969S2	0.45	-1.152003093	0.09813519
Q14344	0.545	-0.875671865	0.09813519
P48165	0.503	-0.991369695	0.09846633
Q96JJ7	0.51	-0.971430848	0.09846633
Q96S97	0.628	-0.671163536	0.09846633
Q15057	1.799	0.847195187	0.10089968
Q9BW04	0.469	-1.092340172	0.1028427
P40121	1.457	0.543000877	0.10291196
P10589	1.912	0.935082523	0.10367378
J3KPV4	1.434	0.520045024	0.10420675

P0DI82	2.112	1.078609835	0.10544429
P98179	1.451	0.537047519	0.10639867
P42166	1.431	0.517023672	0.10790484
E5RJV5	1.912	0.935082523	0.10926017
Q9UDY2	1.68	0.748461233	0.11078029
P56945	1.799	0.847195187	0.11133181
Q9NRA8	1.973	0.980390956	0.11183548
Q96QR8	0.475	-1.074000581	0.11184804
P31937	0.676	-0.564904848	0.11278594
Q15643	0.517	-0.951763814	0.11299916
Q96AE4	1.712	0.775682702	0.11338776
Q92572	0.631	-0.66428809	0.11485266
P49005	1.597	0.675364313	0.11496151
P46199	0.587	-0.768567592	0.11654281
A2ABE6	0.635	-0.655171503	0.1165565
Q96EY8	0.517	-0.951763814	0.12137664
Q68D10	0.431	-1.214240226	0.12142249
Q58FG1	1.783	0.834306703	0.12142249
H0YLF3	0.646	-0.63039393	0.12161484
K7EKI8	0.565	-0.823677227	0.12286288
Q9UI09	1.451	0.537047519	0.1249846
Q9BR76	0.68	-0.556393349	0.12499666
O60524	0.575	-0.798366139	0.12535549
F5H865	1.775	0.827819025	0.12565384
Q99805	1.461	0.546956178	0.12576554
Q15629	0.652	-0.61705613	0.12846077
Q9NUQ2	0.572	-0.805912948	0.13106818
P11413	0.694	-0.526992432	0.1338283
Q15366-6	1.411	0.496717988	0.1338283
Q9UBD5	1.871	0.903809559	0.13393931
Q9UDY8	0.559	-0.839079812	0.13397126
O75436	0.684	-0.54793177	0.1344374
Q96LB3	0.436	-1.19759996	0.13540639
P00491	0.695	-0.524915117	0.13680462
P12429	1.409	0.494671612	0.13686548
P69905	1.392	0.477159211	0.13731249
P21695	0.659	-0.60164963	0.14050108
P35579	0.696	-0.522840789	0.14067207
Q8NI60	1.931	0.949348164	0.14091837
Q02241	1.57	0.650764559	0.1412553
Q96C36	0.666	-0.586405918	0.14127728
P57764	0.654	-0.612637459	0.14243456
Q01105	1.694	0.760433875	0.14416211
P56962	0.426	-1.231074664	0.14428471
Q9HAB8	0.674	-0.569179503	0.14456535
P46939	0.513	-0.962969269	0.14690152

P22102	0.699	-0.516635639	0.14708037
Q8N6M0	1.618	0.694211608	0.14737627
E9PE48	0.563	-0.828793173	0.14782416
P31323	0.563	-0.828793173	0.14857937
P20337	0.657	-0.606034724	0.1495933
Q9Y6E0	1.434	0.520045024	0.1495933
P61978	1.437	0.523060062	0.15004674
P56211	1.722	0.784085143	0.15065454
O95838	0.581	-0.783389931	0.15078401
B4DDV1	1.738	0.797428082	0.15078401
Q92526	1.412	0.497740089	0.15115584
Q8NF64	1.952	0.964953053	0.1515571
P36551	1.398	0.483364361	0.15211464
B4DXZ6	1.431	0.517023672	0.1524132
P16278	0.647	-0.628162383	0.15247788
I3L291	0.535	-0.902389203	0.1539665
P60520	1.666	0.736388401	0.1539665
O15234	0.564	-0.826232932	0.1541655
E7ET52	1.834	0.874993639	0.15508708
Q68D86	0.432	-1.210896782	0.15569632
P26358	1.384	0.468843943	0.15693659
D6RDH4	1.931	0.949348164	0.15693659
P11441	1.631	0.705756782	0.15845067
F5H5P2	0.588	-0.76611194	0.15979046
P30533	0.658	-0.603840511	0.15979046
P43121	0.557	-0.844250767	0.15992415
P49914	0.589	-0.763660461	0.15995019
P56134	0.587	-0.768567592	0.16099621
P09104	0.669	-0.579921884	0.16230521
Q9UPN7	1.563	0.644317778	0.16443463
O75131	1.358	0.44148348	0.16465741
P26440	0.667	-0.584241333	0.16564121
Q8N6T3	0.585	-0.77349147	0.16780054
Q14181	1.72	0.782408565	0.16780054
O95807	0.545	-0.875671865	0.17131312
P06493	1.385	0.469885976	0.1715233
Q6P161	0.585	-0.77349147	0.17192342
O15427	1.441	0.527070336	0.17236475
B2RD65	0.669	-0.579921884	0.17328174
Q14247	0.71	-0.49410907	0.17364875
P61457	1.576	0.656267535	0.17364875
Q75MJ1	1.4	0.485426827	0.17370702
Q14764	1.395	0.480265122	0.17553887
H3BV68	0.487	-1.038006323	0.17710571
P11388	1.434	0.520045024	0.17710571
Q14145	1.677	0.745882689	0.17710571

P36952	1.445	0.531069493	0.1794811
Q53H96	0.676	-0.564904848	0.1796632
Q6GMV3	0.578	-0.790858602	0.17980415
J3QRS3	0.71	-0.49410907	0.18243407
P21583	1.706	0.770617647	0.18243407
P19388	1.558	0.639695233	0.18268461
Q5T5C0	0.442	-1.177881725	0.1846084
P39880	0.63	-0.666576266	0.18494892
P27695	0.711	-0.492078535	0.18597621
Q9HAV7	0.71	-0.49410907	0.1862215
O14908	1.452	0.538041453	0.18653825
G3V5T0	0.674	-0.569179503	0.18681011
Q6PD74	1.691	0.75787666	0.18800319
Q9UHD1	1.376	0.46048047	0.18868821
F8WEE8	1.729	0.789937869	0.18887237
Q9H2H9	0.593	-0.75389599	0.19166547
B4DT70	1.703	0.768078435	0.1923745
P56192	0.723	-0.467932448	0.1928445
Q9HD26	0.669	-0.579921884	0.1939418
P11216	0.712	-0.490050854	0.1939418
Q96GA7	0.681	-0.554273297	0.1949106
Q99956	1.535	0.618238656	0.19599343
Q4G176	0.674	-0.569179503	0.19813589
Q05639	0.714	-0.486004021	0.19889488
P38432	1.683	0.751035177	0.2004386
E9PFT6	1.416	0.501821265	0.20108718
Q13042	0.597	-0.744197163	0.20235676
Q9UHX1-5	1.368	0.45206823	0.20319314
Q9HAT2	0.618	-0.694321257	0.20408056
O75663	1.538	0.621055503	0.20580455
P54840	0.677	-0.562772261	0.20585282
H3BV80	1.403	0.488515009	0.20588375
O95400	0.61	-0.713118852	0.20646612
O15382	0.697	-0.520769439	0.20656974
P55957	1.414	0.49978212	0.20656974
C9J9T0	1.685	0.752748591	0.20656974
Q9UNS1	1.538	0.621055503	0.20673086
Q8IWE2	1.41	0.495695163	0.206817
Q86SQ0	0.677	-0.562772261	0.20722184
Q9NXG6	1.779	0.831066511	0.20809789
G3V5X4	1.679	0.74760223	0.20929527
B5MCN5	1.849	0.886745225	0.20935589
Q3KQV9	0.519	-0.946193556	0.20980144
B4DZH6	0.56	-0.836501268	0.20992875
P12268	0.718	-0.477944251	0.210414
Q8NI36	0.715	-0.483984853	0.21109974

P56537	1.363	0.446785562	0.21300142
P35749	0.687	-0.541617996	0.21385336
Q9H496	1.809	0.855192408	0.21554326
E9PHY5	0.716	-0.481968507	0.21805094
Q9ULT8	1.365	0.448900951	0.2198642
Q9NR45	0.721	-0.471928835	0.21987406
Q9NRW1	0.576	-0.795859283	0.21988604
Q96HQ2	1.486	0.571434116	0.22073036
C9K060	1.526	0.609754962	0.22432993
Q9BUZ4	1.872	0.904580435	0.22477471
Q6ZMG9	0.607	-0.720231578	0.2260582
Q9H2W6	0.694	-0.526992432	0.22768257
O15530	1.897	0.923719679	0.22883941
O95167	0.639	-0.646112164	0.22939176
H3BPE1	0.719	-0.475936324	0.22939176
F5H136	1.666	0.736388401	0.22939176
Q9ULD2	2.075	1.053111336	0.22939176
Q15404	1.377	0.461528559	0.22990093
P31751	2.048	1.034215715	0.23088494
P51452	0.614	-0.703689439	0.23170751
Q9P2J5	0.723	-0.467932448	0.23193395
P00519	0.482	-1.052894948	0.23197918
Q13442	1.395	0.480265122	0.23197918
Q96HV5	1.669	0.738983955	0.23225341
F6U1T9	1.481	0.566571641	0.23226573
I3L1R7	0.609	-0.715485867	0.23304824
G5E9A6	0.727	-0.459972731	0.23309056
Q9P2B2	0.62	-0.689659879	0.23314306
P51809	0.686	-0.543719518	0.23391373
Q15392	1.378	0.462575888	0.23391373
Q96IZ0	0.613	-0.706041021	0.23400555
Q9C0B0	0.551	-0.859875776	0.2346839
Q5T0Y8	1.754	0.810648748	0.23575386
P47985	0.716	-0.481968507	0.23619229
P43490	0.733	-0.448114897	0.23749764
P19823	1.732	0.79243893	0.23906199
Q13541	0.62	-0.689659879	0.23985624
A6NDB9	1.649	0.721591399	0.24021535
Q9Y546	1.879	0.909965067	0.24127109
Q53FT3	1.511	0.595503661	0.24433799
Q15833	0.687	-0.541617996	0.24437289
P18433	0.466	-1.10159814	0.24483176
Q6UWE0	0.573	-0.803392956	0.24483176
Q5T0Y2	0.694	-0.526992432	0.24483176
H3BQP3	0.576	-0.795859283	0.24490077
Q8IXB1	0.608	-0.717856771	0.24509649

H3BSW0	0.614	-0.703689439	0.24614662
Q9Y478	0.608	-0.717856771	0.24693718
Q9P2X0	0.616	-0.698997744	0.24721993
Q8IWW6-2	1.649	0.721591399	0.24736396
H3BUV4	0.617	-0.696657606	0.24843229
H0YH87	0.699	-0.516635639	0.24895857
Q9NZM1-6	1.345	0.427606173	0.24947898
D6RE58	1.774	0.82700601	0.2501449
B4DNJ6	1.324	0.404903122	0.25140458
P49368	1.343	0.425459305	0.25261169
O95628	1.831	0.872631791	0.25286226
P49750	0.72	-0.473931188	0.2531571
P62191	1.342	0.424384672	0.25342249
P50452	0.589	-0.763660461	0.25396604
Q9BX40	1.519	0.60312187	0.25403342
Q5BKX8	1.722	0.784085143	0.25606401
J3KT75	0.69	-0.535331733	0.25676847
Q9UJM3	0.598	-0.74178261	0.25699529
F8VV59	1.619	0.695102986	0.25769114
P42772	1.732	0.79243893	0.26007219
Q9NY99	0.574	-0.800877358	0.26111452
Q9UQ35	1.339	0.421155961	0.26111452
Q96FQ6	1.395	0.480265122	0.26155483
P36639	1.593	0.671746267	0.26155483
Q92844	1.856	0.89219671	0.26155483
B7Z5R6	2.006	1.004321606	0.26361422
P58107	0.724	-0.465938398	0.26391955
Q8TAP9	1.799	0.847195187	0.2642362
P62861	1.339	0.421155961	0.26563491
Q14651	1.379	0.463622457	0.26659164
Q9H1A4	0.698	-0.518701058	0.27012592
Q68D91	0.569	-0.813499442	0.27134072
Q9BXR0	0.702	-0.510457064	0.27149219
P02649	0.686	-0.543719518	0.27219358
F5GZS6	0.734	-0.446148032	0.27225809
Q9GZN8	1.499	0.584000383	0.27352788
Q9HAN9	1.581	0.660837368	0.27384689
Q9BTE3-2	0.744	-0.426625474	0.27436444
P33992	1.333	0.41467678	0.27436444
Q9H2U1	1.498	0.583037624	0.27436444
Q16775	0.689	-0.537424112	0.27618052
Q01650	0.706	-0.502259911	0.27645317
Q96FN5	1.588	0.667210912	0.27660159
P11926	1.685	0.752748591	0.27957361
Q9BRJ6	1.558	0.639695233	0.2801166
B4DY38	0.55	-0.862496476	0.28050775

Q53H82	0.702	-0.510457064	0.28050775
P42695	1.573	0.653518671	0.28054786
O95470	0.715	-0.483984853	0.28225911
P41250	0.737	-0.440263476	0.28325362
A6PVM9	0.51	-0.971430848	0.28346985
B4DY91	0.583	-0.778432211	0.28407929
Q15714-2	1.597	0.675364313	0.28407929
P51665	1.304	0.38294387	0.28530574
A6NN80	0.737	-0.440263476	0.28591236
Q86UY0	0.738	-0.438307279	0.28693539
Q9NXF7	1.602	0.679874148	0.28705536
P30038	0.61	-0.713118852	0.2872011
B0QZ18	0.738	-0.438307279	0.2872011
O75937	0.707	-0.50021788	0.28766824
H0YAH3	1.801	0.848798181	0.28809452
Q9H2G2	1.371	0.455228571	0.28896323
P62873	0.739	-0.436353731	0.28944299
E7EN96	1.686	0.753604536	0.28944299
Q96AE4-2	1.326	0.407080775	0.28972815
Q9ULX6	1.357	0.440420721	0.29100611
Q9NQ34	1.69	0.757023247	0.29179208
Q13185	1.325	0.40599236	0.29255921
P07384	1.323	0.403813062	0.29605816
O15173	0.707	-0.50021788	0.297159
E9PC15	0.694	-0.526992432	0.29877287
Q6NTF9	0.613	-0.706041021	0.29901375
Q6ZSJ8	0.587	-0.768567592	0.29911893
C9JLU1	1.343	0.425459305	0.29927397
Q9Y5J7	0.714	-0.486004021	0.29963448
Q16836	0.711	-0.492078535	0.29977049
Q16740	1.345	0.427606173	0.30034068
P15374	0.718	-0.477944251	0.3008202
P50224	1.583	0.662661255	0.30093991
G3V1V0	0.741	-0.432454552	0.30117513
P16144-2	1.321	0.401630467	0.3016877
Q02447-3	0.571	-0.808437349	0.30175762
Q9H0W8	1.566	0.647084213	0.30175762
Q70Z53	0.594	-0.751465164	0.30309977
Q8NB37	1.725	0.786596362	0.30319168
P25685	0.739	-0.436353731	0.30319683
P04083	0.743	-0.428565884	0.30740376
Q86UE8	1.592	0.670840336	0.3075903
Q15058	1.955	0.967168608	0.30925892
Q6ZWJ1	1.559	0.640620928	0.309643
Q9UNX4	0.745	-0.424687669	0.3098801
P35237	0.745	-0.424687669	0.31074399

P22830	0.71	-0.49410907	0.31153765
O95704	0.585	-0.77349147	0.31265233
Q9H3N1	0.739	-0.436353731	0.31331317
O75487	1.775	0.827819025	0.31627769
Q6PI48	0.733	-0.448114897	0.31659738
P33993	1.315	0.3950628	0.31726151
Q8IV36	0.573	-0.803392956	0.31807073
Q9UHB6-4	0.726	-0.461958547	0.3185787
Q8IZF6	1.549	0.631337144	0.32005916
C9JAW5	0.702	-0.510457064	0.32168292
P19367	0.719	-0.475936324	0.32329178
O15460	0.723	-0.467932448	0.32603948
P52789	0.716	-0.481968507	0.32648802
Q96C19	0.727	-0.459972731	0.32648802
H9KV59	0.602	-0.732164608	0.32812476
Q9H1I8	1.39	0.475084883	0.32843719
E9PMR4	1.507	0.591679417	0.32905953
Q8N138	0.6	-0.736965594	0.3291281
Q9UHW5	1.485	0.570462931	0.3291281
Q96PD2	0.712	-0.490050854	0.32965559
J3QRN6	0.652	-0.61705613	0.32977212
Q04837	0.747	-0.420819852	0.3320122
O15020	1.632	0.706641057	0.33238004
O00330	0.717	-0.479954976	0.33302643
Q5TEC6	1.577	0.65718266	0.33302643
P00390	1.309	0.388465097	0.33313713
Q7Z3C6	1.567	0.64800518	0.33400002
Q14240	0.75	-0.415037499	0.33538315
Q9UNW1	0.721	-0.471928835	0.33636099
F8VW92	1.308	0.387362541	0.33645442
P35613	1.307	0.386259141	0.33918041
Q02252	0.624	-0.680382066	0.33933401
G8JLH6	0.724	-0.465938398	0.34010499
Q96I51	1.56	0.641546029	0.34010499
Q9H7B4	1.629	0.703986604	0.34032062
B7Z6B8	1.318	0.39835037	0.34080207
O00767	1.522	0.605968359	0.34080656
P62195	1.305	0.384049807	0.34236823
Q9UKX7	1.323	0.403813062	0.34410389
Q9Y6M1	1.304	0.38294387	0.344932
O15091	0.655	-0.610433188	0.34525286
Q9C037	0.6	-0.736965594	0.34558594
O43852-4	0.646	-0.63039393	0.34558594
Q9H223	1.342	0.424384672	0.34558594
P27797	0.751	-0.413115187	0.34580939
Q9NRY5	1.55	0.632268215	0.34615181

Q96QZ7	0.576	-0.795859283	0.34657562
E9PL57	1.401	0.486456956	0.34730022
O00154-4	1.303	0.381837084	0.34760209
Q12904	0.752	-0.411195433	0.34984546
E9PBE5	1.542	0.624802765	0.34984546
O14646	1.569	0.649845352	0.34984546
P46821	0.647	-0.628162383	0.35030737
P78356	1.558	0.639695233	0.35183276
P09211	1.301	0.379620962	0.35426327
Q9H1Y0	1.388	0.473007568	0.35426327
P63261	1.3	0.378511623	0.35615813
P49736	1.299	0.377401431	0.35681741
Q92520	1.306	0.385154897	0.35772846
Q7RTV5	0.512	-0.965784285	0.35842593
Q6WCQ1	0.592	-0.756330919	0.35842593
Q6P597	0.639	-0.646112164	0.35842593
P55809	0.748	-0.418889825	0.35943388
Q9NR12	1.378	0.462575888	0.36068049
Q9UHL4	0.626	-0.675765438	0.36234119
F5H569	0.722	-0.469929258	0.36297949
P54105	1.319	0.399444565	0.36297949
P50542	1.541	0.623866862	0.36297949
P00966	0.73	-0.454031631	0.36594336
P04183	0.738	-0.438307279	0.36741064
O00170	1.341	0.423309237	0.3675859
O94964	0.635	-0.655171503	0.36893545
P57772	0.64	-0.64385619	0.36965687
Q9NPA8	1.543	0.625738062	0.36965687
Q9NR28	0.756	-0.40354186	0.3709712
Q9NXV2	1.627	0.702214251	0.3709712
K7EPC1	1.299	0.377401431	0.37168831
C9JA52	1.492	0.577247536	0.37356642
P22033	0.671	-0.575615328	0.37365821
O76003	1.324	0.404903122	0.37368263
Q8TBQ9	0.655	-0.610433188	0.37461843
Q9Y291	0.679	-0.55851652	0.37461843
O94760	0.718	-0.477944251	0.37490332
P62273	0.756	-0.40354186	0.37490332
E9PI68	1.338	0.420078116	0.37763482
Q9BT73	1.539	0.621993232	0.37813544
Q9P1F3	0.733	-0.448114897	0.3790869
Q7L576	0.754	-0.407363571	0.37979325
O15269	1.302	0.380729449	0.37979997
P54577	0.758	-0.399730246	0.38013137
P00374	1.371	0.455228571	0.38091985
F8VZR5	1.742	0.800744624	0.38091985

F6U6P3	0.534	-0.905088353	0.38117974
C9IYK6	0.663	-0.592919225	0.38117974
Q14376	0.746	-0.422752464	0.38117974
P67936	0.763	-0.390245038	0.38117974
J3KRX5	0.759	-0.397828209	0.38275471
D6RDI8	0.536	-0.899695094	0.38296542
P42574	1.665	0.735522177	0.38312932
Q8TCC3	0.635	-0.655171503	0.38347865
E9PGZ1	0.746	-0.422752464	0.38347865
P23528	1.289	0.366252264	0.38352046
P50991	1.289	0.366252264	0.38461927
Q8WW22	0.746	-0.422752464	0.38464349
E7ETA7	0.735	-0.444183845	0.38640409
Q86UW9	1.909	0.932817103	0.38640409
Q9H078-2	1.315	0.3950628	0.38689341
Q8TAF3	1.701	0.766383141	0.38770908
Q92820	1.293	0.370722275	0.38792233
Q7L5Y9	1.514	0.598365205	0.38932259
P57737-3	1.512	0.59645814	0.3910916
P49454	0.628	-0.671163536	0.39355277
O95159	0.66	-0.59946207	0.39355277
P20020	0.738	-0.438307279	0.39355277
Q9NUQ6	1.327	0.408168371	0.39355277
Q9Y4C2	1.393	0.478195258	0.39355277
Q13438	1.495	0.580145484	0.39355277
Q7Z4G1	1.595	0.673556424	0.39355277
P23508	0.652	-0.61705613	0.39663315
Q15437	0.762	-0.392137097	0.39671998
Q6NZI2	0.738	-0.438307279	0.39839087
Q09161	0.767	-0.382701517	0.39839087
Q00341	1.275	0.350497247	0.39839087
Q15819	1.48	0.565597176	0.39839087
D6RJH6	1.698	0.763836459	0.39841122
P24534	0.763	-0.390245038	0.39967843
E9PPW4	0.611	-0.710755715	0.39993677
Q8TCG1	1.503	0.587845009	0.39993677
Q9BXL6	1.659	0.730313886	0.39993677
Q56VL3	0.743	-0.428565884	0.40008308
Q8WUY8	1.647	0.719840555	0.40008308
C9JEX3	1.709	0.773152397	0.40008308
H0YBY3	1.59	0.669026766	0.40116896
P50454	1.281	0.357270476	0.40211201
Q9HCC0	0.74	-0.434402824	0.40553558
Q13243	1.304	0.38294387	0.40553558
P49903	1.3	0.378511623	0.40678915
Q9HBR0	0.592	-0.756330919	0.40704504

H0Y4C3	0.595	-0.749038426	0.40704504
Q9NUQ3	0.73	-0.454031631	0.40758309
P41252	0.765	-0.386468347	0.4088657
Q8WVX9	0.662	-0.595096878	0.40987214
Q13641	1.698	0.763836459	0.40987214
Q01085	0.669	-0.579921884	0.41010163
E5RH51	0.593	-0.75389599	0.41029998
P54819	0.771	-0.375197235	0.41134437
O96013	1.402	0.487486349	0.41134437
Q8TEX9	1.279	0.355016264	0.41163413
P49419-2	0.766	-0.384583703	0.41200056
Q4LE39	1.634	0.708407983	0.41200056
P61962	1.397	0.482332021	0.41211199
Q13526	1.314	0.393965276	0.41218618
Q9UKI8	0.6	-0.736965594	0.41261943
Q8WX93	0.768	-0.380821784	0.4146944
Q9NYU2	0.767	-0.382701517	0.4158302
Q13155	0.769	-0.378944497	0.4158302
Q16587	0.645	-0.632628934	0.41830961
Q9BPX5	0.762	-0.392137097	0.41884151
Q9H832	0.737	-0.440263476	0.41902826
E5RGN3	0.709	-0.496142467	0.41927401
Q9H2J4	1.31	0.389566812	0.41927401
Q8IW92	1.314	0.393965276	0.41934844
C9JIB4	1.67	0.739848103	0.42229155
Q8IYD1	0.602	-0.732164608	0.42256647
Q16881	0.765	-0.386468347	0.42509208
Q9UL63	1.483	0.568518598	0.42509208
P01034	1.501	0.585923977	0.42509208
P08727	0.769	-0.378944497	0.42608875
P00492	0.774	-0.369594529	0.42649826
Q92609	0.676	-0.564904848	0.42679229
O43819	1.482	0.567545448	0.42691617
P53602	1.367	0.451013243	0.42745771
P55011	1.381	0.46571332	0.42745771
Q5U5X0	1.493	0.578214165	0.42761628
Q15386	0.742	-0.430508908	0.42837573
Q567V2	1.567	0.64800518	0.42853809
Q9NXR7-1	0.663	-0.592919225	0.4293645
Q16527	0.743	-0.428565884	0.4293645
O95684	1.515	0.599317794	0.4293645
O43657	0.665	-0.588573754	0.42978057
Q96ME7	0.738	-0.438307279	0.42978057
P26038	0.77	-0.377069649	0.42978057
P16219	0.701	-0.512513651	0.43099323
Q16864	0.746	-0.422752464	0.43149578

Q9H0D6	1.264	0.337996464	0.43217226
Q09666	0.77	-0.377069649	0.43305038
P35998	1.271	0.34596403	0.4330741
Q9Y446	1.315	0.3950628	0.43379477
Q9NXE4	0.69	-0.535331733	0.43405462
P50990	1.27	0.344828497	0.43405462
Q14571	1.314	0.393965276	0.43405462
Q9H6S3	1.493	0.578214165	0.43405462
J3KRV9	1.672	0.741574847	0.43405462
O60244	0.559	-0.839079812	0.43458548
Q53GS7	0.648	-0.625934282	0.43620488
Q15155	0.56	-0.836501268	0.4376856
P61024	1.445	0.531069493	0.43790652
Q9NZE8	0.652	-0.61705613	0.4404237
Q4G0Z9-4	0.617	-0.696657606	0.44220283
P45877	0.682	-0.552156356	0.44260224
Q9HD45	0.772	-0.373327247	0.44334984
Q8NFW8	0.666	-0.586405918	0.44527292
P53007	0.748	-0.418889825	0.44527292
Q08209-2	1.264	0.337996464	0.44561249
Q9BY44	1.285	0.361768359	0.44587261
P12955	0.75	-0.415037499	0.44942458
H0Y6W0	0.607	-0.720231578	0.45464141
Q6P2E9	1.243	0.313826296	0.45641529
Q9BTX3	0.611	-0.710755715	0.45733896
Q6UW68	0.744	-0.426625474	0.45856367
Q9Y6E2	1.279	0.355016264	0.45865121
H7BZJ3	0.78	-0.358453971	0.46128183
Q9UBW8	1.285	0.361768359	0.46128183
O43432	0.612	-0.708396442	0.4613464
Q8NBN7	0.691	-0.533242384	0.4613464
P62318	1.262	0.33571191	0.4613464
P23921	1.272	0.347098671	0.46363165
E9PAY8	0.667	-0.584241333	0.46373515
Q9BRT6	0.68	-0.556393349	0.46392391
P24752	0.776	-0.365871442	0.46436723
O15127	0.744	-0.426625474	0.46478737
P28676	0.644	-0.634867407	0.46544282
Q9Y2B0	0.753	-0.40927823	0.46560777
A6NHL2	1.353	0.436161839	0.46844973
Q8IUI8	1.387	0.471967788	0.46844973
Q96PU8	0.604	-0.727379545	0.46859753
Q9NQY0	0.664	-0.590744853	0.46943958
H0Y861	0.639	-0.646112164	0.46946208
Q9Y2D4	1.626	0.701327257	0.46980385
P30101	0.778	-0.36215794	0.47085458

Q16762	0.695	-0.524915117	0.47096658
Q6IN84	0.748	-0.418889825	0.47096658
Q13011	0.773	-0.371459681	0.47096658
Q8TCF1	1.348	0.430820497	0.47127527
Q99720	0.741	-0.432454552	0.47159017
Q02410	0.686	-0.543719518	0.4726725
Q9UBF2	1.356	0.439357178	0.47477673
P46940	0.779	-0.360304767	0.47528193
Q9UNK0	1.48	0.565597176	0.47542818
Q9NXS2	0.579	-0.788364747	0.47564316
Q9NX14	1.365	0.448900951	0.47593796
P61224	0.78	-0.358453971	0.47736227
O15144	0.784	-0.351074441	0.47736227
Q9UHD2	1.36	0.443606651	0.47736227
Q9BW66	1.39	0.475084883	0.47736227
Q9BRQ8	1.383	0.467801156	0.47814182
G3XAC4	1.472	0.557777671	0.47905682
P09497	0.684	-0.54793177	0.4798917
E7ETA6	1.293	0.370722275	0.4798917
B5ME49	0.569	-0.813499442	0.48021616
P21953	0.697	-0.520769439	0.48021616
Q8N108	1.65	0.722466024	0.48021616
Q96SI9	1.758	0.81393507	0.48021616
Q92817	0.672	-0.573466862	0.48165367
P12277	0.78	-0.358453971	0.48165367
Q9Y6A9	1.344	0.426533138	0.48165367
Q9HCE9	1.538	0.621055503	0.48165367
Q9NYP7	0.717	-0.479954976	0.48233646
Q96SK2-2	0.675	-0.567040593	0.48238214
O00429	1.236	0.305678743	0.48238214
Q9NWK9	0.661	-0.597277823	0.48297496
O43795-2	0.789	-0.341902795	0.48391679
Q9Y3A6	1.343	0.425459305	0.48391679
Q9H993	0.74	-0.434402824	0.48403333
Q9UKB1	0.65	-0.621488377	0.48470329
Q6GMV2	1.446	0.532067552	0.4852786
Q16186	1.282	0.358396262	0.4873543
O60343	0.752	-0.411195433	0.48847713
P42126	0.787	-0.345564459	0.48847713
C9JFR7	0.786	-0.347398782	0.48876979
Q96C01	0.761	-0.394031641	0.48900237
Q8IV48	1.464	0.549915554	0.48900237
Q9UNF0	0.787	-0.345564459	0.49022752
Q9NXA8	0.668	-0.582079992	0.4911574
P15291	0.641	-0.641603738	0.49303573
P14678	1.257	0.32998465	0.49384676

Q96DA6	1.429	0.515005916	0.49384676
Q92522	1.262	0.33571191	0.4939356
E9PI90	1.33	0.411426246	0.49540518
P35908	1.604	0.681674142	0.49557157
Q7Z6J9	1.583	0.662661255	0.49611432
P38117	0.783	-0.352915787	0.49705623
Q9UEU0	0.607	-0.720231578	0.49945534
P07996	0.637	-0.650634722	0.49945534
E7EQ34	0.709	-0.496142467	0.49945534
O75153	1.233	0.3021728	0.49945534
Q8WVC0	1.302	0.380729449	0.49945534
O95229	0.669	-0.579921884	0.49975464
F8VZJ2	1.249	0.320773477	0.49989526
A9UJQ0	1.535	0.618238656	0.50003712
J3KMW7	1.348	0.430820497	0.5003621
O75817	1.497	0.582074221	0.50064486
P58317	0.584	-0.775959726	0.50086612
Q8N4E4	0.653	-0.614845103	0.50109113
Q9H7Z6	1.465	0.550900665	0.50195503
O43324	0.767	-0.382701517	0.50196999
P54886	0.784	-0.351074441	0.50214717
Q9C0B1	1.372	0.456280482	0.50214717
P30046	0.787	-0.345564459	0.50268526
P49441	0.695	-0.524915117	0.50276195
P50213	0.783	-0.352915787	0.50478945
Q53GA4	0.766	-0.384583703	0.50518692
Q9BW19	0.689	-0.537424112	0.50569505
Q12907	0.786	-0.347398782	0.50609908
P17568	0.756	-0.40354186	0.50688877
B0UX83	1.264	0.337996464	0.50697357
O15235	0.69	-0.535331733	0.50699625
O95479	0.691	-0.533242384	0.50699625
B7Z3Z9	0.677	-0.562772261	0.50774068
P62306	0.758	-0.399730246	0.50908825
P43686	1.246	0.317304068	0.50945831
Q9NRF9	1.328	0.409255147	0.50985056
J3KNQ4	1.36	0.443606651	0.51012487
Q9Y2Z4	0.779	-0.360304767	0.51043002
Q86U44	0.684	-0.54793177	0.51050358
Q96LJ7	1.538	0.621055503	0.51074233
O14773	0.768	-0.380821784	0.51179543
Q00653	0.765	-0.386468347	0.51319477
H0YME5	0.699	-0.516635639	0.51374717
P55039	1.35	0.432959407	0.51410666
P30043	0.774	-0.369594529	0.5142944
P10599	1.245	0.316145742	0.5142944

Q14012	1.539	0.621993232	0.51520288
Q9Y530	0.665	-0.588573754	0.51618919
B4DRT2	0.772	-0.373327247	0.51644302
Q6ZRV2	1.437	0.523060062	0.51673547
P21964	1.244	0.314986485	0.51701422
E9PDV0	0.724	-0.465938398	0.51718328
B4DY26	0.6	-0.736965594	0.51788736
F8WC89	1.589	0.668119125	0.51845595
Q8N4N8	0.766	-0.384583703	0.51859492
P67809	1.226	0.293958979	0.5194092
Q96DV4	0.771	-0.375197235	0.51941195
Q9ULC4	1.269	0.343692069	0.51941195
P15311	1.243	0.313826296	0.5209861
Q96FX8	1.515	0.599317794	0.5209861
Q96HE7	0.79	-0.340075442	0.52113004
P26639	0.788	-0.343732465	0.52217415
Q12872	1.588	0.667210912	0.52229168
F5GYQ1	0.767	-0.382701517	0.52405628
Q9UJX4	0.685	-0.545824107	0.52476423
P49790	0.68	-0.556393349	0.52486165
P30044	0.789	-0.341902795	0.52542019
Q5JTJ3	1.417	0.502839758	0.52542019
H0YAN8	0.705	-0.504304837	0.52679184
Q14146	0.763	-0.390245038	0.52788269
Q9NPE3	0.771	-0.375197235	0.52938538
A6NKV8	1.406	0.491596594	0.52938538
I3L1I3	1.466	0.551885103	0.52989665
O75674	1.435	0.521050737	0.52996466
O43681	0.779	-0.360304767	0.53066563
Q9Y4R8	1.295	0.372952098	0.53066563
P26232	1.394	0.479230561	0.53066563
Q8N6L1	1.434	0.520045024	0.53115403
Q9NQW7-3	0.789	-0.341902795	0.53296222
O15182	1.443	0.5290713	0.53296222
Q6JQN1	0.768	-0.380821784	0.53371544
Q5JRA6	0.75	-0.415037499	0.53391
Q9C0C9	0.791	-0.3382504	0.53391
P40227	1.239	0.309176187	0.53412096
O43896	0.668	-0.582079992	0.53430121
D6RER5	0.787	-0.345564459	0.53469209
B4DKY1	0.788	-0.343732465	0.53469209
P54136	0.791	-0.3382504	0.53469209
P61604	1.238	0.308011315	0.53469209
E7EN46	1.553	0.63505783	0.53469209
D3YTH9	1.571	0.651683181	0.53471932
O14949	0.778	-0.36215794	0.53630236

P60059	1.319	0.399444565	0.53702463
Q9H4L7	1.428	0.513995979	0.53702463
Q92995	1.433	0.51903861	0.53702463
Q6IC75	1.503	0.587845009	0.53702463
Q99584	1.268	0.342554745	0.53838751
P14923	1.236	0.305678743	0.53932113
Q8TF01	1.479	0.564622052	0.53956911
Q6UXV4	1.478	0.563646269	0.54145442
Q96CN9	0.684	-0.54793177	0.54211426
Q96RP9	1.274	0.349365278	0.5425544
P42167	1.24	0.310340121	0.5426087
Q86TS9	0.693	-0.529072743	0.54352091
Q8NDD1	0.695	-0.524915117	0.54352091
B7ZKQ9	0.705	-0.504304837	0.54352091
Q8N183	0.761	-0.394031641	0.54352091
Q9ULW0	0.773	-0.371459681	0.54352091
P26006	1.246	0.317304068	0.54352091
Q9H089	1.259	0.332278283	0.54352091
F8W7C6	1.265	0.339137385	0.54352091
Q4G0J3	1.424	0.509949146	0.54352091
P11474	1.514	0.598365205	0.54352091
Q96HY6	1.356	0.439357178	0.54589361
Q9NVS2	0.771	-0.375197235	0.5485735
Q96Q42	1.592	0.670840336	0.5485735
Q5VWU8	0.599	-0.739372092	0.54952154
J3QLH3	1.421	0.506906555	0.54952154
O14647	1.566	0.647084213	0.54952154
H0YMB4	1.632	0.706641057	0.54952154
O00273-2	1.558	0.639695233	0.5495327
P85037	1.392	0.477159211	0.55010204
G8JLK4	0.642	-0.639354798	0.55063747
Q9BZL6	1.34	0.422233001	0.55063747
Q96EY7	0.794	-0.332789088	0.55134841
O95394	0.799	-0.323732592	0.55142198
Q8IVD9	1.424	0.509949146	0.55142198
P15924	0.797	-0.327348371	0.55167223
H3BTY2	0.78	-0.358453971	0.55238209
Q96P48-1	0.781	-0.356605547	0.55238209
P48643	1.232	0.301002256	0.55238209
Q8N8U9	1.549	0.631337144	0.55238209
O75718	0.771	-0.375197235	0.55371487
Q9Y6I9	0.695	-0.524915117	0.55448785
Q96N66	0.757	-0.401634795	0.55531369
P18065	0.715	-0.483984853	0.55552453
P41134	1.571	0.651683181	0.5559346
E9PHN7	1.647	0.719840555	0.5559346

Q5XKP0	1.398	0.483364361	0.55610144
Q9NV56	1.472	0.557777671	0.55830948
P07237	1.23	0.298658316	0.55945525
Q7Z4H8	0.773	-0.371459681	0.56000386
Q9ULR0	1.413	0.498761466	0.56060477
Q68CP9	1.421	0.506906555	0.56060477
P35659	1.226	0.293958979	0.56089953
Q00688	1.243	0.313826296	0.56162587
O95639	1.42	0.50589093	0.5618207
Q8TED0	1.242	0.312665174	0.56205925
P23786	0.783	-0.352915787	0.56232204
Q7LBC6	0.786	-0.347398782	0.56232204
Q6AI08	0.773	-0.371459681	0.56243782
C9JZG2	0.699	-0.516635639	0.56247109
Q9UK22	0.785	-0.349235441	0.56303554
Q96EL2	0.703	-0.508403406	0.56311652
B4DHE8	0.776	-0.365871442	0.56311652
P27449	0.777	-0.364013496	0.56311652
Q9BS26	0.798	-0.325539348	0.56311652
P04792	1.228	0.296310561	0.56311652
P49840	1.345	0.427606173	0.56311652
I3L2A9	1.544	0.626672753	0.56311652
Q9P2D1	1.549	0.631337144	0.56311652
Q96A19	1.422	0.507921465	0.56417366
P16885	0.784	-0.351074441	0.5650502
E9PE51	1.394	0.479230561	0.56568401
P0CG34	1.419	0.504874589	0.56568401
O96007	1.52	0.604071324	0.56568401
Q96GC5	0.782	-0.354759487	0.565785
Q15382	1.308	0.387362541	0.56708903
P11172	1.241	0.311503115	0.56723135
Q5TDH0	1.332	0.413594082	0.56747379
Q8NDC0	1.352	0.435095152	0.56752083
P06744	1.226	0.293958979	0.56791358
Q6P6C2	1.408	0.493647334	0.56791358
Q00535	1.318	0.39835037	0.56848922
P50995	1.221	0.2880632	0.5694816
P27707	1.336	0.417920008	0.5694816
Q96FK6	1.542	0.624802765	0.5694816
Q2M389	0.805	-0.312939312	0.57038444
C9JCC6	1.257	0.32998465	0.57038444
P52732	1.354	0.437227739	0.57133367
P47755	1.237	0.3068455	0.57172998
P27338	1.24	0.310340121	0.57172998
P46459	0.799	-0.323732592	0.57181669
P08758	0.8	-0.321928095	0.57268803

O94874	1.25	0.321928095	0.57389125
Q9NZL9	1.236	0.305678743	0.57408539
P17028	0.693	-0.529072743	0.5745585
Q13201	0.722	-0.469929258	0.57498529
Q9UMX1	1.522	0.605968359	0.57615618
P32119	0.801	-0.320125852	0.57625868
Q01581	0.806	-0.311148256	0.57678336
P0CB43	1.383	0.467801156	0.57763058
Q99848	1.237	0.3068455	0.57871104
O75369	0.801	-0.320125852	0.57898422
P05114	1.401	0.486456956	0.58002209
Q53T59	1.395	0.480265122	0.58023622
Q9Y4P3	1.317	0.397255346	0.58033139
O00743	1.225	0.292781749	0.58034595
P49757	0.659	-0.60164963	0.58065552
P80303	0.778	-0.36215794	0.58065552
P08559	0.796	-0.329159664	0.58065552
Q06210	0.81	-0.304006187	0.58065552
P49366	1.241	0.311503115	0.58065552
Q9BVR6	1.634	0.708407983	0.58065552
P25205	1.221	0.2880632	0.58110719
C9JPV1	1.638	0.711935357	0.58135686
F8W9E7	0.717	-0.479954976	0.58215678
A6NF31	1.528	0.611644543	0.58215678
Q9P0M6	1.237	0.3068455	0.58220615
Q9H981	0.714	-0.486004021	0.58266604
Q96FZ5	0.657	-0.606034724	0.58321975
P30041	0.802	-0.318325858	0.58432437
F8WJN3	1.234	0.303342394	0.58449871
H0Y5T9	1.314	0.393965276	0.58498816
Q9H0S4	1.227	0.295135249	0.58587835
Q9BUE0	1.396	0.481298942	0.58621677
P13797	0.803	-0.316528107	0.58674184
P50416	1.231	0.299830762	0.58762925
O00217	0.793	-0.334607229	0.58774761
H7BXH2	1.232	0.301002256	0.58774761
F8WBW2	1.549	0.631337144	0.58776423
Q9H000	1.406	0.491596594	0.58811227
P08133	0.719	-0.475936324	0.58812846
Q6UWP7	0.717	-0.479954976	0.58865424
A8MPT4	0.71	-0.49410907	0.58897802
Q8IUH4	0.712	-0.490050854	0.59033828
O43447	1.314	0.393965276	0.59033828
Q99832	1.218	0.284514133	0.59053835
P17152	0.702	-0.510457064	0.59173155
O95373	1.218	0.284514133	0.59206748

Q16134	0.622	-0.685013515	0.59253088
Q9HC07	0.789	-0.341902795	0.59253088
F8W8Q9	0.704	-0.506352666	0.59253125
P53350	1.241	0.311503115	0.59386671
Q5BKZ1	1.24	0.310340121	0.5938943
P48735	1.21	0.275007047	0.59403492
P26641	0.805	-0.312939312	0.59446469
F8W9W2	0.706	-0.502259911	0.59501655
Q15125	0.718	-0.477944251	0.59501655
Q92896	0.805	-0.312939312	0.59501655
O75874	0.805	-0.312939312	0.59501655
Q14676	1.248	0.319617934	0.59501655
P14866	1.216	0.282143229	0.59548946
P19525	1.216	0.282143229	0.59639114
Q9UK59	0.794	-0.332789088	0.59728314
Q9Y294	1.303	0.381837084	0.59728314
Q9P032	0.709	-0.496142467	0.59793488
J3KS54	0.659	-0.60164963	0.59899689
B4DS61	1.249	0.320773477	0.59899689
O75525	0.668	-0.582079992	0.59984276
P58557	0.71	-0.49410907	0.60032999
Q9BR61	0.622	-0.685013515	0.60044033
Q9UI12	0.775	-0.367731785	0.60106177
Q9NR31	1.236	0.305678743	0.60168008
P09382	0.807	-0.309359421	0.60259637
P49748	0.811	-0.30222618	0.60259637
P02545-2	1.252	0.324234562	0.60291266
J3QQN7	0.711	-0.492078535	0.60328755
Q8TB52	1.389	0.474046599	0.60329228
J3KNF4	0.721	-0.471928835	0.60461759
Q66K74	1.3	0.378511623	0.60553145
Q9BUL5	1.52	0.604071324	0.60573669
P13051	0.758	-0.399730246	0.60627364
Q92696	1.238	0.308011315	0.6072159
E9PIN5	0.68	-0.556393349	0.60724251
Q15417	0.784	-0.351074441	0.60724251
Q12981-1	1.39	0.475084883	0.60724251
Q9BRX5	1.481	0.566571641	0.60724251
H0Y5B0	0.638	-0.648371671	0.60772397
Q8TBP6	0.638	-0.648371671	0.60772397
E9PQN9	0.779	-0.360304767	0.60772397
Q9BRA2	0.787	-0.345564459	0.60772397
P04264	0.793	-0.334607229	0.60772397
Q9BQP7	0.793	-0.334607229	0.60772397
Q13557	0.799	-0.323732592	0.60772397
G5E9D3	0.807	-0.309359421	0.60772397

Q14566	1.211	0.276198865	0.60772397
P62805	1.212	0.277389699	0.60772397
O95218	1.232	0.301002256	0.60772397
B1AKJ5	1.234	0.303342394	0.60772397
Q9UM07	1.235	0.304511042	0.60772397
Q13619	1.286	0.362890643	0.60772397
Q9H4A5	1.3	0.378511623	0.60772397
Q9BTX1	1.385	0.469885976	0.60772397
Q9H5K3	1.39	0.475084883	0.60772397
Q9NZD2	1.396	0.481298942	0.60772397
H3BPZ8	1.503	0.587845009	0.60772397
B8ZZQ6	1.21	0.275007047	0.60833391
Q9Y673	0.789	-0.341902795	0.60871683
P00505	0.809	-0.305788392	0.60911211
Q9UEE9	1.372	0.456280482	0.60915087
Q8IWB7	0.711	-0.492078535	0.60939167
P19404	1.231	0.299830762	0.60943784
Q9BVC6	0.801	-0.320125852	0.61114338
P60842	1.209	0.273814245	0.61290116
Q9H3G5	0.741	-0.432454552	0.61307427
O94925-3	1.231	0.299830762	0.61348953
Q9BPZ3	1.272	0.347098671	0.61470093
Q9H0U3	0.793	-0.334607229	0.61473867
Q13901	0.623	-0.682695932	0.61502792
E7ES33	0.812	-0.300448367	0.61502792
P78344	1.193	0.254594043	0.61521061
P35637	1.208	0.272620455	0.6152747
Q14847	1.221	0.2880632	0.61573404
B4DZN8	1.597	0.675364313	0.6169098
Q15293	0.811	-0.30222618	0.61701886
Q96KP1	0.733	-0.448114897	0.61715419
P35580	0.808	-0.307572802	0.61719888
Q9H9F9	1.372	0.456280482	0.61732781
Q8NBJ4	0.78	-0.358453971	0.61798559
Q08623	0.794	-0.332789088	0.61798559
Q9H307	1.214	0.279768422	0.61798559
B4DEM7	1.282	0.358396262	0.61798559
E7EVF4	1.436	0.522055749	0.61798559
P55196-1	1.479	0.564622052	0.61798559
P13747	1.516	0.600269754	0.61878001
Q16342	1.435	0.521050737	0.61885295
F5GY99	0.799	-0.323732592	0.61886909
Q9Y639	0.784	-0.351074441	0.62012817
Q9Y3Y2	1.249	0.320773477	0.62060896
Q9P0L0	0.808	-0.307572802	0.62198816
P49321	1.206	0.270229907	0.62370137

Q9NUD5	1.465	0.550900665	0.62474507
A2ABB9	0.738	-0.438307279	0.62516854
P15151	0.768	-0.380821784	0.62516854
P53999	0.813	-0.298672743	0.62516854
O00161	0.815	-0.295128036	0.62516854
Q9Y613	1.483	0.568518598	0.62516854
P42345	0.793	-0.334607229	0.62525077
Q93075	0.777	-0.364013496	0.62540754
E9PQ56	1.293	0.370722275	0.62541637
P08240	1.229	0.297484916	0.62586874
Q9P0J7	1.335	0.416839742	0.62594298
O95361	0.738	-0.438307279	0.62662897
P82650	0.792	-0.336427665	0.62662897
Q96TC7	0.734	-0.446148032	0.62681104
P62633	1.37	0.454175893	0.62681104
P51636	0.732	-0.450084446	0.62775528
P61020	0.716	-0.481968507	0.62783202
B7ZBM3	0.631	-0.66428809	0.62783291
Q9NRV9	0.742	-0.430508908	0.62978382
Q8NFH3	1.243	0.313826296	0.63006095
A8MWW0	0.699	-0.516635639	0.63023118
F5GYC1	0.792	-0.336427665	0.63023118
O43823	1.287	0.364012054	0.63140961
Q5JXX4	1.362	0.445726703	0.63155921
Q96BP2	0.76	-0.395928676	0.63367174
P49585	1.29	0.367371066	0.63367174
Q9NTJ3	1.232	0.301002256	0.63460859
Q8NB90	1.235	0.304511042	0.63526095
Q5T760	1.224	0.291603558	0.63552415
Q7Z3T8	0.676	-0.564904848	0.63586966
Q8IWV8	0.708	-0.498178735	0.63586966
A7YIJ8	1.224	0.291603558	0.63586966
O96000	1.233	0.3021728	0.63586966
Q8NEL9	1.288	0.365132593	0.63586966
Q15843	1.362	0.445726703	0.63586966
Q9UHJ6	1.368	0.45206823	0.63586966
Q8IWT3	1.578	0.658097205	0.63586966
Q9UMX0	1.202	0.265436896	0.63597618
O60774	0.738	-0.438307279	0.63645567
Q8TEQ6	0.815	-0.295128036	0.63645567
Q6N069	1.46	0.545968369	0.63645567
Q3ZCW2	0.725	-0.4639471	0.63688284
Q8N3C0	1.228	0.296310561	0.63872294
O75391	0.739	-0.436353731	0.63878838
K7ELL7	0.815	-0.295128036	0.6388172
P35249	1.216	0.282143229	0.6388172

Q6NW34	0.728	-0.457989644	0.63950024
Q9UGV2	1.251	0.32308179	0.63959166
O00425	0.818	-0.289827252	0.63978225
O95433	1.201	0.264236151	0.63995613
Q9Y508	0.702	-0.510457064	0.64063471
Q96C90	1.223	0.290424404	0.64106633
O95671	0.799	-0.323732592	0.64134032
E9PEI9	0.804	-0.314732593	0.64134032
Q92973-2	1.193	0.254594043	0.6414664
O43520	0.792	-0.336427665	0.64303377
E9PHV5	1.21	0.275007047	0.64316874
Q8IW45	0.729	-0.45600928	0.64342443
Q14166	0.815	-0.295128036	0.64342443
Q5VT52	0.74	-0.434402824	0.6448242
G3V599	0.784	-0.351074441	0.64565096
Q7Z2T5	0.804	-0.314732593	0.64565096
Q96A72	1.336	0.417920008	0.64597012
Q8NC44	0.679	-0.55851652	0.64605037
Q9UL26	0.727	-0.459972731	0.64605037
E7EQC1	0.807	-0.309359421	0.64605037
Q9GZR7	0.817	-0.291592017	0.64605037
H7BY84	1.236	0.305678743	0.64624424
Q9H3E2	1.174	0.231432408	0.64744956
B7ZKS7	1.358	0.44148348	0.64744956
Q9UBQ7	0.813	-0.298672743	0.6476585
B1B0M1	0.733	-0.448114897	0.64782609
C9JVN9	0.799	-0.323732592	0.64782609
Q6YP21	0.802	-0.318325858	0.64782609
Q14157	1.204	0.267835392	0.6485838
P11177	0.812	-0.300448367	0.64859076
O14776	1.219	0.285698126	0.64933685
Q9NP81	1.24	0.310340121	0.649953
Q5VWZ2	0.812	-0.300448367	0.65055757
B5MC67	0.695	-0.524915117	0.6513321
P62304	1.199	0.261831659	0.6513321
Q9NRF8	1.287	0.364012054	0.6513321
Q9Y4X0	1.464	0.549915554	0.6513321
Q9HBL7	1.197	0.259423152	0.65143204
Q9Y450	1.226	0.293958979	0.65227568
Q9NWU2	1.226	0.293958979	0.65227568
Q14241	0.734	-0.446148032	0.6526386
Q9UJ68	0.688	-0.53951953	0.65272984
H0Y368	0.799	-0.323732592	0.65272984
Q16576	1.202	0.265436896	0.65272984
Q9UBR2	1.27	0.344828497	0.65272984
Q96EZ8	1.489	0.574343754	0.65272984

Q9H9B4	0.822	-0.282789701	0.65300923
P31150	0.826	-0.275786313	0.65300923
Q9BVP2	1.201	0.264236151	0.65300923
P30520	1.202	0.265436896	0.65300923
A6NDG6	1.22	0.286881148	0.65300923
Q9NY27	1.26	0.333423734	0.65319638
Q96B49	1.27	0.344828497	0.65323158
Q8WUY1	1.35	0.432959407	0.65323158
E9PFR3	1.359	0.442545456	0.65323158
Q8N8N7	0.744	-0.426625474	0.65431195
O14929	1.207	0.271425676	0.65441978
Q96HY7	0.725	-0.4639471	0.65500577
Q9H0E9	1.446	0.532067552	0.65500577
B4DJW3	1.368	0.45206823	0.65676578
O95602	1.206	0.270229907	0.65704869
P82664	0.764	-0.388355457	0.65742619
J3QL71	0.731	-0.452056689	0.65853471
Q8IYS2	0.776	-0.365871442	0.65853471
Q8IX18	0.805	-0.312939312	0.65853471
Q53GQ0	0.819	-0.288064643	0.65853471
Q9BZX2	1.217	0.283329168	0.65853471
I3L2B0	1.33	0.411426246	0.65853471
O60828	1.336	0.417920008	0.65853471
Q9Y3L5	1.385	0.469885976	0.65853471
Q96P47	1.39	0.475084883	0.65853471
Q9P2S5	1.394	0.479230561	0.65853471
Q9H9L3	1.449	0.535057595	0.65853471
Q14690	0.82	-0.286304185	0.6585902
P0CG08	0.738	-0.438307279	0.65870725
Q12788	0.823	-0.281035664	0.65870725
F5H1X8	0.825	-0.277533976	0.65904471
O43347	0.735	-0.444183845	0.65943904
Q96C86	0.812	-0.300448367	0.66112371
Q9Y276	0.745	-0.424687669	0.66146642
P55010	1.196	0.25821739	0.66200877
P34931	0.743	-0.428565884	0.66205577
B3KTM8	1.207	0.271425676	0.66205577
Q15738	0.803	-0.316528107	0.66224909
P00441	0.821	-0.284545873	0.66224909
P23284	0.821	-0.284545873	0.66224909
Q99615	1.194	0.255802837	0.66224909
C9JXC6	1.454	0.540027269	0.66224909
P82663	0.804	-0.314732593	0.66237541
E7EW20	1.205	0.269033146	0.66251258
Q8IWZ8	1.401	0.486456956	0.66251258
P49773	0.823	-0.281035664	0.66260263

P63151	1.205	0.269033146	0.66267529
D6RAN8	0.806	-0.311148256	0.6627165
G3V1K3	0.812	-0.300448367	0.66277222
Q9Y244	1.269	0.343692069	0.66277222
Q4G0S7	1.306	0.385154897	0.66277222
Q08170	1.39	0.475084883	0.66277222
Q92667	1.409	0.494671612	0.66385213
Q7Z2Z2	0.802	-0.318325858	0.66396395
Q9Y2Z0-2	1.201	0.264236151	0.66550141
O15231	0.693	-0.529072743	0.66558976
F5H365	0.831	-0.267079618	0.66558976
Q9NZZ3	1.323	0.403813062	0.66558976
P42696	1.378	0.462575888	0.66558976
O14657	0.711	-0.492078535	0.66585762
O94992	1.225	0.292781749	0.66585762
Q13610	1.291	0.368489001	0.66585762
O43402	1.332	0.413594082	0.66585762
Q9ULM6	1.417	0.502839758	0.66594068
Q86U38	1.288	0.365132593	0.66596842
P49006	1.216	0.282143229	0.66632579
Q9NR48	0.814	-0.2968993	0.66665887
A8MUM1	1.357	0.440420721	0.66778952
H3BT91	0.721	-0.471928835	0.66813634
H0YGW8	0.791	-0.3382504	0.66813634
P40616	0.801	-0.320125852	0.66813634
Q9H4G4	0.748	-0.418889825	0.66861055
Q15056	1.195	0.257010618	0.67011898
P61009	1.275	0.350497247	0.67130847
Q9UKM7	0.747	-0.420819852	0.6713922
Q8IVP5	0.688	-0.53951953	0.67215558
B5MBX0	0.731	-0.452056689	0.67215558
Q6DD87	1.324	0.404903122	0.67215558
Q9UKL0	1.307	0.386259141	0.67232296
Q8NEW0	0.75	-0.415037499	0.67370716
Q86UA1	1.322	0.402722177	0.67387625
Q9Y3A5	0.804	-0.314732593	0.67453424
P09543	0.802	-0.318325858	0.67468923
O15400	1.191	0.252173413	0.67494914
Q12979-2	0.72	-0.473931188	0.67504223
P13639	0.824	-0.279283757	0.67504223
Q8NFQ8	1.264	0.337996464	0.67504223
G3V2U7	1.267	0.341416524	0.67504223
Q9P2N7	1.453	0.539034703	0.67504223
Q6P4I2	0.689	-0.537424112	0.67577728
Q99575	1.218	0.284514133	0.67589103
P0CG13	1.389	0.474046599	0.67600272

Q8WU90	1.196	0.25821739	0.67620832
Q5T9A4	0.826	-0.275786313	0.67695378
Q5I2W7	0.752	-0.411195433	0.67712552
P11279	0.791	-0.3382504	0.67717717
Q9UNN5	1.268	0.342554745	0.67757622
P61326	1.196	0.25821739	0.6775816
Q96H79	0.754	-0.407363571	0.67925893
P49848	1.37	0.454175893	0.67955406
Q02978	0.825	-0.277533976	0.67995722
Q8WX92	1.212	0.277389699	0.67995722
G5E9B6	1.217	0.283329168	0.67995722
B0V043	0.825	-0.277533976	0.67998086
Q6NZY4	1.46	0.545968369	0.68011084
Q13867	0.824	-0.279283757	0.68057371
Q32M78	0.735	-0.444183845	0.68064447
Q16513	0.816	-0.293358943	0.68158422
Q6GQQ9	1.446	0.532067552	0.68186231
E7EP77	0.744	-0.426625474	0.68191185
P19447	0.751	-0.413115187	0.68222367
O43390	1.186	0.24610401	0.68222367
Q5VV50	1.556	0.63784206	0.68222367
Q9GZT8	1.226	0.293958979	0.68265501
Q13443	0.732	-0.450084446	0.68277621
Q86VN1	0.701	-0.512513651	0.68369252
E9PKF3	0.807	-0.309359421	0.68369252
Q2NKX8	1.21	0.275007047	0.68369252
E7EVC7	1.337	0.418999465	0.68369252
Q7Z7A3	1.358	0.44148348	0.68369252
Q96GN5	0.748	-0.418889825	0.68523897
P12235	0.814	-0.2968993	0.68532758
Q96ER3	1.263	0.336854639	0.68564364
Q9HC35	0.807	-0.309359421	0.68600793
P61964	1.191	0.252173413	0.68600793
Q6P4E1	0.733	-0.448114897	0.68758479
Q2NL82	0.837	-0.256700472	0.68838817
Q8TDN6	1.189	0.249748715	0.68877213
Q9BU89	1.272	0.347098671	0.68895312
O75319	0.745	-0.424687669	0.68904861
Q9NYY8	0.801	-0.320125852	0.68904861
F8WEK6	1.423	0.508935662	0.68904861
Q9BX46	1.425	0.510961919	0.68904861
Q96PC3-4	1.379	0.463622457	0.68998268
E9PRY8	1.183	0.242450074	0.6907164
Q7Z6E9	0.755	-0.40545145	0.69071769
O15498	1.19	0.250961574	0.69188076
P53396	0.828	-0.272297327	0.69225762

Q14657	1.321	0.401630467	0.69272485
H0YA40	1.449	0.535057595	0.69276338
E9PE17	0.804	-0.314732593	0.69312999
Q8IXU6	1.309	0.388465097	0.69312999
P61513	0.807	-0.309359421	0.69329945
P09972	1.182	0.241230036	0.69329945
G3V167	1.381	0.46571332	0.69329945
P49902	0.827	-0.274040765	0.69510803
P49711	1.273	0.348232419	0.69510803
F2Z2Z9	1.324	0.404903122	0.69510803
P09234	1.207	0.271425676	0.69601738
O14545	0.661	-0.597277823	0.6960684
Q9BZE9	0.742	-0.430508908	0.69613889
P10644	1.189	0.249748715	0.69613889
Q9Y6G5	1.422	0.507921465	0.69613889
Q9H467	1.415	0.500802053	0.69727042
Q2TAY7	0.83	-0.268816758	0.6981644
P14174	1.18	0.23878686	0.69901449
Q27J81	1.326	0.407080775	0.69901449
Q96MT7	1.37	0.454175893	0.69901449
Q7LG56	0.738	-0.438307279	0.69916861
G3V1V4	1.299	0.377401431	0.69916861
Q9H0A8	1.338	0.420078116	0.69916861
P01008	0.757	-0.401634795	0.69919995
Q9Y295	1.191	0.252173413	0.70033896
E7EUT4	1.39	0.475084883	0.70041743
Q9NV31	1.209	0.273814245	0.70084267
Q8IZ83	0.818	-0.289827252	0.7013905
P31939	0.83	-0.268816758	0.7013905
Q13283	1.179	0.237563718	0.70161854
Q13618	1.191	0.252173413	0.70174179
P47756-2	0.831	-0.267079618	0.70420808
Q96BM9	1.296	0.374065718	0.70444814
Q6NXG1	1.294	0.371837617	0.70520213
Q9UID3	1.33	0.411426246	0.70553256
E9PK73	0.753	-0.40927823	0.70602091
P51553	0.818	-0.289827252	0.70638372
Q8NFH5	0.82	-0.286304185	0.70638372
Q99757	1.302	0.380729449	0.70638372
Q14444	1.187	0.247319935	0.70647148
P52943	1.33	0.411426246	0.70699369
F8VQZ7	1.178	0.236339539	0.70742334
B4DDF9	0.762	-0.392137097	0.70743549
O95822	0.665	-0.588573754	0.70836157
J3QQX3	0.707	-0.50021788	0.70945323
Q16718	1.25	0.321928095	0.70955254

B1AJY7	0.808	-0.307572802	0.70966219
Q8IWV7	0.825	-0.277533976	0.71019876
Q96HS1	1.192	0.253384236	0.71040087
Q92544	0.802	-0.318325858	0.71053582
P49336	0.66	-0.59946207	0.71125992
Q8N357	0.75	-0.415037499	0.71125992
P55209	1.176	0.23388806	0.71125992
P68104	0.833	-0.263611599	0.71219439
P51151	1.341	0.423309237	0.71219439
Q7L0Y3	0.842	-0.248107862	0.71237459
Q92688	1.166	0.221567789	0.71237459
Q9NW13	1.187	0.247319935	0.71297177
Q8N567	1.358	0.44148348	0.71431905
Q9BV86	1.221	0.2880632	0.71446993
P84243	1.175	0.232660757	0.71473872
Q9NWU1	0.827	-0.274040765	0.71542526
Q6DKK2	1.309	0.388465097	0.71584476
Q9Y6N3	1.314	0.393965276	0.71673626
Q6UW78	1.322	0.402722177	0.71673626
Q969X5	1.203	0.266636643	0.71692108
H0YGS7	1.413	0.498761466	0.71692108
Q9BT22	0.823	-0.281035664	0.71788677
F5H7S9	1.326	0.407080775	0.71969082
P62140	0.813	-0.298672743	0.72025658
P07108	0.833	-0.263611599	0.72025658
O43143	1.174	0.231432408	0.72025658
Q9UDY4	1.219	0.285698126	0.72025658
E9PQ61	1.254	0.326537348	0.72025658
Q86WD7-5	1.368	0.45206823	0.72025658
Q8NDZ4	1.474	0.559736524	0.72025658
Q6FI81	1.192	0.253384236	0.72157252
P00414	0.773	-0.371459681	0.7216185
Q6IA86	0.813	-0.298672743	0.7216185
Q9BY77	1.183	0.242450074	0.72193181
O95816	0.837	-0.256700472	0.72251463
K7EJQ7	1.359	0.442545456	0.72364036
O43760	0.825	-0.277533976	0.72384527
Q9NV70	0.826	-0.275786313	0.72384527
Q9NVI1	1.243	0.313826296	0.72397673
O75323	0.82	-0.286304185	0.72440504
P31153	1.173	0.230203013	0.72440504
Q69YN4	1.247	0.318461465	0.72446751
Q9BXW9	1.297	0.37517848	0.72446751
Q14BN4	0.688	-0.53951953	0.72463657
F6UYM1	1.351	0.434027675	0.72474452
F5H8H2	1.319	0.399444565	0.7247772

Q16658	0.836	-0.258425153	0.72478227
Q9UPN9	1.261	0.334568276	0.72535905
Q9BPW8	0.835	-0.260151897	0.72548923
Q9BZ72	0.81	-0.304006187	0.72590681
O75431	0.822	-0.282789701	0.72590681
O15270	1.198	0.260627908	0.72590681
Q04206	1.24	0.310340121	0.72590681
Q96S82	1.339	0.421155961	0.72590681
F8WDH1	0.763	-0.390245038	0.72594492
Q9BTT6	1.178	0.236339539	0.72594492
O14497	0.816	-0.293358943	0.72657489
Q16778	1.171	0.227741076	0.72707292
Q8N1G2	1.421	0.506906555	0.72707292
Q9P035	0.836	-0.258425153	0.7273551
Q9NYV6	0.773	-0.371459681	0.72744097
E9PCI9	1.18	0.23878686	0.72744097
E9PS80	0.741	-0.432454552	0.72744733
P82675	0.822	-0.282789701	0.72744733
F8WCY5	0.837	-0.256700472	0.72744733
Q9H9T3	1.192	0.253384236	0.72744733
Q8IV50	0.742	-0.430508908	0.72751066
Q9HD20	0.761	-0.394031641	0.72751066
E7ETU9	0.761	-0.394031641	0.72751066
Q4TT38	0.811	-0.30222618	0.72751066
O43615	1.174	0.231432408	0.72842259
Q9HB71	1.17	0.22650853	0.72847183
Q96E14	0.683	-0.550042516	0.7284839
Q5ZPR3	0.815	-0.295128036	0.72888861
O94973	0.818	-0.289827252	0.72888861
Q9Y5X1	0.822	-0.282789701	0.72888861
Q96G25	1.397	0.482332021	0.72916515
Q96QD8	1.172	0.22897257	0.72964048
Q9HC38-2	0.839	-0.253257284	0.73026588
Q9NQS1	1.339	0.421155961	0.73102155
Q9NZ52	1.344	0.426533138	0.73137125
P23434	1.228	0.296310561	0.73176726
C9JIM0	1.353	0.436161839	0.73254818
Q9Y394	1.244	0.314986485	0.73273865
P62253	1.273	0.348232419	0.73447885
Q10471	0.764	-0.388355457	0.73466841
Q9Y3D9	0.831	-0.267079618	0.73466841
Q8N5M9	1.239	0.309176187	0.73466841
Q12933	1.392	0.477159211	0.73466841
P57735	0.753	-0.40927823	0.73480103
Q96EK5	1.188	0.248534836	0.73480103
C9JPG4	1.367	0.451013243	0.73480103

Q92665	0.825	-0.277533976	0.73485274
Q02880	1.174	0.231432408	0.73485274
J3KP15	1.163	0.217851097	0.73496882
P35658	0.81	-0.304006187	0.73509456
Q96I24	0.835	-0.260151897	0.73509456
Q71U36	1.167	0.222804561	0.73509456
Q15291	1.248	0.319617934	0.73509456
Q9NPI6	1.294	0.371837617	0.73509456
Q96RT1	1.428	0.513995979	0.73509456
P08779	1.4	0.485426827	0.73559974
G3V4I5	0.715	-0.483984853	0.73656229
Q86Y82	0.828	-0.272297327	0.73678007
P43487	1.167	0.222804561	0.73737875
Q9ULX3	1.183	0.242450074	0.73776848
Q6ZNB6	0.835	-0.260151897	0.73813685
Q8NB16	0.766	-0.384583703	0.73838467
O00625	1.261	0.334568276	0.73838467
O60443	1.349	0.431890348	0.73988829
Q96B26	1.194	0.255802837	0.74101041
C9JC74	0.735	-0.444183845	0.74120731
Q9BUP3-3	0.759	-0.397828209	0.74120731
Q9NTM9	0.816	-0.293358943	0.74120731
O60493	0.818	-0.289827252	0.74120731
Q53FA7	0.818	-0.289827252	0.74120731
Q8N122	0.826	-0.275786313	0.74120731
Q8WUM4	0.84	-0.251538767	0.74120731
O43396	1.146	0.196607044	0.74120731
Q13885	1.162	0.216610069	0.74120731
Q12972	1.187	0.247319935	0.74120731
Q92925	1.24	0.310340121	0.74120731
P52735	1.246	0.317304068	0.74120731
I3L2L5	1.363	0.446785562	0.74120731
J3KQ48	0.82	-0.286304185	0.74130096
Q8N4T8	0.779	-0.360304767	0.74160487
O15511	0.838	-0.254977851	0.74171869
Q15257	1.189	0.249748715	0.74171869
Q9NRG9	1.201	0.264236151	0.743567
Q8WXX5	1.177	0.23511432	0.74372739
Q15904	0.78	-0.358453971	0.74391152
P30405	0.756	-0.40354186	0.74405935
Q15019	0.842	-0.248107862	0.74408248
P50579	1.213	0.27857955	0.74408248
P36871	0.842	-0.248107862	0.74469578
Q96K12	0.768	-0.380821784	0.74491122
Q9UBI6	0.819	-0.288064643	0.744984
P02545-3	1.383	0.467801156	0.744984

E9PNK6	0.777	-0.364013496	0.74535113
Q86X29	0.812	-0.300448367	0.74535113
P45985	0.827	-0.274040765	0.74535113
Q13492	0.832	-0.265344567	0.74535113
P42766	0.841	-0.249822294	0.74535113
Q9Y5Y0	1.384	0.468843943	0.74553673
Q3B726	0.723	-0.467932448	0.7455605
Q6UX53	0.75	-0.415037499	0.7455605
P42892	0.819	-0.288064643	0.7455605
Q8IZ81	0.82	-0.286304185	0.7455605
Q9BQC6	0.842	-0.248107862	0.7455605
P46109	1.169	0.22527493	0.7455605
E9PCS8	1.375	0.459431619	0.7455605
Q9ULW3	1.291	0.368489001	0.74571135
Q71DI3	0.842	-0.248107862	0.74643723
P10620	1.182	0.241230036	0.74643723
Q6NXE6	0.822	-0.282789701	0.74650181
Q15031	1.334	0.415758667	0.74650181
P31943	1.163	0.217851097	0.74651026
O95551	1.297	0.37517848	0.74651026
O94804	0.836	-0.258425153	0.74654705
Q9GZU8	1.294	0.371837617	0.74665494
Q4AC99	1.186	0.24610401	0.74668687
H0Y990	1.297	0.37517848	0.74668822
Q8WXD5	1.297	0.37517848	0.74673656
Q5T4S7	0.838	-0.254977851	0.74754482
Q9HD34	1.324	0.404903122	0.74754482
B3KY94	1.224	0.291603558	0.74878446
F8WES2	1.281	0.357270476	0.74883414
C9JB30	0.695	-0.524915117	0.74982409
Q14554	0.761	-0.394031641	0.74982409
P11171	0.774	-0.369594529	0.74982409
Q8WUH6	0.78	-0.358453971	0.74982409
Q9H267	0.837	-0.256700472	0.74982409
Q12959	1.177	0.23511432	0.74982409
Q86WB0	1.24	0.310340121	0.74982409
Q9H0U6	0.837	-0.256700472	0.74992326
Q86UD0	0.688	-0.53951953	0.74993136
Q9H1C4	0.761	-0.394031641	0.75002538
P53803	1.269	0.343692069	0.75018008
O95793-3	1.169	0.22527493	0.75044004
Q6SPF0	1.373	0.457331625	0.75091407
O43290	1.165	0.220329955	0.75133704
H7C0N4	1.346	0.42867841	0.75133704
P24385	0.765	-0.386468347	0.75174262
K7EKE6	0.843	-0.246395464	0.75181348

Q9UJ41	0.762	-0.392137097	0.75182439
Q9UI30	1.167	0.222804561	0.75182439
Q02750	0.833	-0.263611599	0.75207064
P06753-2	0.843	-0.246395464	0.75207064
Q86WA6	0.77	-0.377069649	0.75219874
P49643	1.195	0.257010618	0.75326277
H0YLZ8	1.311	0.390667686	0.75372044
H0YK48	0.781	-0.356605547	0.75434926
F8W6X5	0.838	-0.254977851	0.75434926
D6RH22	1.22	0.286881148	0.75434926
G3V1C4	1.241	0.311503115	0.75434926
O00499	1.298	0.376290383	0.75446065
F2Z2U4	1.179	0.237563718	0.75449935
Q9UK76	1.163	0.217851097	0.75477283
P61313	0.844	-0.244685096	0.75488594
P78347	1.16	0.214124805	0.75488594
Q86W42	1.231	0.299830762	0.75488594
C9JK10	1.245	0.316145742	0.75488594
P40939	0.844	-0.244685096	0.75495696
O60216	1.18	0.23878686	0.75502874
Q9UBT2	1.157	0.210388864	0.75565072
O14828	1.178	0.236339539	0.75565072
C9JP00	0.822	-0.282789701	0.75580613
P82933	0.83	-0.268816758	0.75580613
Q9BUB1	0.84	-0.251538767	0.75580613
Q9H1D9	1.291	0.368489001	0.75580613
Q9H6Y2	0.793	-0.334607229	0.75631962
Q8IVT2	1.293	0.370722275	0.75631962
Q08257	1.152	0.204140717	0.75639453
P41214	1.202	0.265436896	0.75639453
P0CG30	0.795	-0.330973234	0.75687751
Q9HD15	0.812	-0.300448367	0.75687751
O15260	0.826	-0.275786313	0.75687751
Q9Y2S0	0.837	-0.256700472	0.75687751
Q7Z406-6	1.165	0.220329955	0.75687751
G5EA30	1.176	0.23388806	0.75687751
Q9UJX3	1.209	0.273814245	0.75869389
Q9UEW8	1.287	0.364012054	0.75869389
Q9P2X3	0.775	-0.367731785	0.75887192
Q96T60	1.274	0.349365278	0.75887887
D6RC56	1.299	0.377401431	0.75887887
O75884	0.777	-0.364013496	0.75918885
A6NCK0	0.842	-0.248107862	0.75918885
Q6F5E8	0.756	-0.40354186	0.75968248
H0YKD8	0.843	-0.246395464	0.75968248
P10619	1.231	0.299830762	0.76014321

P48651	1.26	0.333423734	0.76014321
Q96A35	0.829	-0.270555993	0.76043456
Q15043	1.242	0.312665174	0.76043456
Q14914	1.243	0.313826296	0.76043456
Q71UM5	0.826	-0.275786313	0.76064466
Q93008-1	0.826	-0.275786313	0.76140563
Q9UNT1	1.31	0.389566812	0.76140563
Q6UW56	0.729	-0.45600928	0.76143214
Q15075	0.822	-0.282789701	0.76143214
Q9UKV8	0.834	-0.261880711	0.76143214
Q5JSH3	0.843	-0.246395464	0.76143214
G3V3E8	0.843	-0.246395464	0.76143214
Q96I99	0.845	-0.242976753	0.76143214
Q99460	1.156	0.209141398	0.76143214
Q12905	1.156	0.209141398	0.76143214
Q5QNW6	1.156	0.209141398	0.76143214
Q5RKV6	1.163	0.217851097	0.76143214
Q96B13	1.358	0.44148348	0.76143214
P47813	1.178	0.236339539	0.76154513
Q8IUR0	1.395	0.480265122	0.76228272
Q8N5N7	0.815	-0.295128036	0.76228357
Q9UNP9	1.238	0.308011315	0.76228357
C9JTN7	1.272	0.347098671	0.76228357
Q8ND82	1.288	0.365132593	0.76228357
P23142	1.389	0.474046599	0.76228357
Q13426	1.166	0.221567789	0.76406041
Q9Y605	1.223	0.290424404	0.7646868
P40763	0.829	-0.270555993	0.76692159
Q9H446	0.78	-0.358453971	0.76692615
P00813	0.769	-0.378944497	0.76694807
Q5VW36	1.289	0.366252264	0.76717046
P11717	0.845	-0.242976753	0.76722118
Q9NYJ1	0.781	-0.356605547	0.76728072
Q96DG6	0.846	-0.241270432	0.76728072
A3KN83	1.34	0.422233001	0.76728072
P18077	0.849	-0.236163541	0.76812934
Q92793	1.275	0.350497247	0.76812934
P55060	1.154	0.206643224	0.76946077
Q9UKF6	1.168	0.224040274	0.76946077
Q3LXA3	0.83	-0.268816758	0.76949082
O43583	1.228	0.296310561	0.7699259
O60869	1.188	0.248534836	0.77039294
Q96FW1	1.162	0.216610069	0.77042023
Q13546	1.258	0.331131922	0.77191034
Q96I59	0.83	-0.268816758	0.77226405
Q16401	1.153	0.205392513	0.77226405

B0QYK0	1.16	0.214124805	0.77226405
I3L4X2	1.22	0.286881148	0.77276843
Q13769	0.847	-0.239566125	0.77307438
P21283	1.165	0.220329955	0.77307438
Q6R327	0.699	-0.516635639	0.77358017
Q9NWX6	1.247	0.318461465	0.77388763
Q8NGJ5	1.335	0.416839742	0.77406317
Q99426	0.831	-0.267079618	0.77488058
Q9Y2D8	1.353	0.436161839	0.77488058
P43034	0.846	-0.241270432	0.77501626
C9JZR2	1.138	0.186500558	0.77501626
B4DY08	1.152	0.204140717	0.77501626
Q9H3H3	1.28	0.35614381	0.77501626
P48506	1.16	0.214124805	0.77515606
O00255	0.761	-0.394031641	0.77531818
Q9BSY4	0.704	-0.506352666	0.77594345
P55795	0.832	-0.265344567	0.77594345
P48426	0.847	-0.239566125	0.77594345
F5H5D3	1.151	0.202887833	0.77594345
C9J406	1.235	0.304511042	0.77594345
P08047	1.255	0.327687364	0.77594345
E9PQW4	1.276	0.351628329	0.77594345
J3KQ97	1.31	0.389566812	0.77594345
P34897	0.851	-0.232768963	0.77612547
I3L3N0	0.737	-0.440263476	0.77621883
Q15036	0.785	-0.349235441	0.77621883
Q7Z5K2	1.258	0.331131922	0.77682867
Q8N573	0.692	-0.531156057	0.7768895
Q9BU23	0.725	-0.4639471	0.7768895
O75298	0.736	-0.442222329	0.7768895
Q96QD9	0.758	-0.399730246	0.7768895
Q5TDG9	0.759	-0.397828209	0.7768895
O14964	0.829	-0.270555993	0.7768895
O75691	0.833	-0.263611599	0.7768895
Q9NQT4	0.839	-0.253257284	0.7768895
Q9Y5P6	0.841	-0.249822294	0.7768895
P42765	0.844	-0.244685096	0.7768895
P06737	0.851	-0.232768963	0.7768895
P61247	0.851	-0.232768963	0.7768895
P45974-2	1.151	0.202887833	0.7768895
O43395	1.161	0.215367972	0.7768895
B3KPJ4	1.217	0.283329168	0.7768895
Q14254	1.221	0.2880632	0.7768895
P48723	1.281	0.357270476	0.7768895
O43164	1.3	0.378511623	0.7768895
O75175	1.313	0.392866916	0.7768895

Q9NVQ4	1.326	0.407080775	0.7768895
E5RIK9	1.332	0.413594082	0.7768895
O76080	1.371	0.455228571	0.7768895
Q5T440	1.419	0.504874589	0.7768895
B4DGT8	0.785	-0.349235441	0.77713295
G3XAL7	0.84	-0.251538767	0.77713295
Q8N5C7	1.33	0.411426246	0.77713295
Q9Y4W6	0.847	-0.239566125	0.77718252
P10586	1.278	0.353887836	0.77718252
A0AVT1	0.862	-0.214240226	0.77735415
Q9NX47	0.856	-0.224317298	0.77804952
P68402	0.854	-0.227692025	0.77867481
B4DP11	1.149	0.200378798	0.77892144
Q9BPU6	0.765	-0.386468347	0.77905437
B7WPF4	0.783	-0.352915787	0.77906012
Q9H4L5	1.165	0.220329955	0.77906012
F5H0R1	1.224	0.291603558	0.77906012
P13667	0.852	-0.231074664	0.77944833
B4DIR7	1.256	0.328836464	0.77944833
Q9UJA5	1.275	0.350497247	0.77944833
Q9Y2R9	0.825	-0.277533976	0.77957238
H0YBL1	0.856	-0.224317298	0.77957238
Q9H6T3	1.186	0.24610401	0.77957238
Q9HAU0	1.283	0.35952117	0.77957238
Q9BUF7	0.711	-0.492078535	0.77984012
Q5T653	0.841	-0.249822294	0.77984012
O14980	1.149	0.200378798	0.77984012
P46060	1.148	0.199122642	0.78050523
Q9HAU5	0.767	-0.382701517	0.78062655
P20700	1.148	0.199122642	0.78062655
A6NDA2	1.403	0.488515009	0.78062655
P17174	0.853	-0.229382353	0.7816556
O60563	0.851	-0.232768963	0.78271145
Q92466	0.79	-0.340075442	0.78280584
O00264	0.835	-0.260151897	0.78281155
Q9BYC8	0.796	-0.329159664	0.78285255
Q8IWA5	1.38	0.464668267	0.78285255
Q9P2C4	0.791	-0.3382504	0.78338256
Q96GD4	1.292	0.36960607	0.78338256
Q9BZH6	0.801	-0.320125852	0.78338573
Q9NZM5	0.843	-0.246395464	0.78361237
Q6P1X5	1.242	0.312665174	0.78361237
P38606	0.863	-0.212567535	0.7842041
B4E2W0	0.854	-0.227692025	0.78461208
Q07065	0.852	-0.231074664	0.78527543
P47914	0.859	-0.219269964	0.78527543

P28340	1.219	0.285698126	0.78531664
Q9Y3B3	0.835	-0.260151897	0.78544852
Q9H081	1.377	0.461528559	0.78638865
Q9NRN7	1.162	0.216610069	0.78652051
Q9H7E9	1.205	0.269033146	0.78652051
Q96GD0	1.252	0.324234562	0.78685275
Q96TA1	1.153	0.205392513	0.78734223
Q9Y6M5	0.771	-0.375197235	0.78756671
F8VXU5	0.823	-0.281035664	0.78756671
F6QR24	0.832	-0.265344567	0.78756671
Q9GZL7	0.851	-0.232768963	0.78756671
P62333	1.138	0.186500558	0.78756671
O00571	1.146	0.196607044	0.78756671
P26196	1.146	0.196607044	0.78756671
Q52LJ0-2	1.146	0.196607044	0.78756671
E7EX17	1.153	0.205392513	0.78756671
P36404	1.245	0.316145742	0.78756671
Q5T0N5	1.266	0.340277405	0.78756671
Q15796	1.278	0.353887836	0.78756671
Q7L8L6	1.293	0.370722275	0.78756671
E7EM93	1.335	0.416839742	0.78756671
G3V1P5	0.77	-0.377069649	0.78837865
P61764	0.839	-0.253257284	0.78837865
Q8N684	1.145	0.195347598	0.78837865
Q96B97	1.163	0.217851097	0.78837865
H3BMQ2	1.255	0.327687364	0.78837865
C9JE98	1.398	0.483364361	0.78837865
Q14694	1.15	0.201633861	0.7885144
P47712	1.16	0.214124805	0.7885144
Q86U90	1.224	0.291603558	0.7885144
Q9NQ48	1.181	0.240008965	0.78863708
O95777	1.166	0.221567789	0.78868739
P16333	0.711	-0.492078535	0.78884264
A8KAH5	0.851	-0.232768963	0.78884264
P05556	0.854	-0.227692025	0.78884264
Q9Y237	1.259	0.332278283	0.78884264
P63241	1.144	0.194087052	0.78982415
H3BTN8	0.79	-0.340075442	0.79066143
Q9Y6A5	0.797	-0.327348371	0.7906892
Q9NSE4	0.857	-0.222632891	0.7915488
Q9Y3I1	0.818	-0.289827252	0.79179475
O95298	0.846	-0.241270432	0.79250897
Q9NUQ7	1.261	0.334568276	0.79285804
O75616	1.245	0.316145742	0.79345942
Q7Z333	1.304	0.38294387	0.79387783
Q9Y2R4	0.844	-0.244685096	0.79411633

Q4G0N4	0.846	-0.241270432	0.79411633
B4DGP8	0.857	-0.222632891	0.79411633
Q9Y4E8	1.155	0.207892852	0.79411633
P61960	1.154	0.206643224	0.79415593
Q9Y5Z4	1.172	0.22897257	0.79415593
J3QSZ6	1.337	0.418999465	0.79424765
O43301	1.264	0.337996464	0.79467311
P09429	1.143	0.192825404	0.79539383
E9PBR6	0.802	-0.318325858	0.79542749
P13804	0.858	-0.220950447	0.79560057
P53985	1.156	0.209141398	0.79561261
Q8N5L8	0.789	-0.341902795	0.79603134
P09001	0.846	-0.241270432	0.79603134
Q9NZI7	1.325	0.40599236	0.79603134
O95352	0.795	-0.330973234	0.7963203
Q12768	0.805	-0.312939312	0.7963203
Q13131	0.806	-0.311148256	0.7963305
Q9UDT6	0.711	-0.492078535	0.79688211
P60602	1.251	0.32308179	0.7970961
Q96FV9	1.253	0.325386415	0.7970961
Q5VZL5	1.199	0.261831659	0.79739042
J3QK90	1.147	0.197865391	0.79767497
Q2TAL8	1.209	0.273814245	0.79771357
Q9BVM2	0.839	-0.253257284	0.79792434
Q5T1Z8	1.159	0.212880566	0.79792434
Q13015	1.315	0.3950628	0.79792434
O75449	1.373	0.457331625	0.79792434
E9PG89	0.722	-0.469929258	0.79864092
B4DWJ3	0.803	-0.316528107	0.79864092
B4E351	0.789	-0.341902795	0.79885248
P30153	1.141	0.190298792	0.79912292
P10768	0.858	-0.220950447	0.79913739
Q15645	1.152	0.204140717	0.79913739
O43678	1.263	0.336854639	0.79913739
H3BMT8	0.85	-0.234465254	0.80038144
E7EN22	0.851	-0.232768963	0.80038144
Q13501	1.148	0.199122642	0.80038144
P52758	1.158	0.211635253	0.80038144
Q96FJ0	0.757	-0.401634795	0.8005708
Q7L7V1	0.803	-0.316528107	0.8005708
Q13888	0.852	-0.231074664	0.8005708
Q13620-1	1.122	0.166072676	0.8005708
E7ESY4	1.162	0.216610069	0.8005708
Q13952	1.265	0.339137385	0.8005708
Q9UL15	1.214	0.279768422	0.80064078
P62829	1.14	0.189033824	0.80110613

O60888	1.178	0.236339539	0.80120204
Q9NRW3	1.224	0.291603558	0.801557
P35222	1.136	0.183962835	0.8020274
Q96K37	0.796	-0.329159664	0.80253785
Q9BVC4	0.797	-0.327348371	0.80304524
O75489	0.842	-0.248107862	0.80304524
Q8IWF9	0.848	-0.23786383	0.80310234
P31947	0.86	-0.217591435	0.803444
D6REA0	0.777	-0.364013496	0.80351298
Q8WTS6	0.805	-0.312939312	0.80351298
P13473	0.857	-0.222632891	0.80351298
P23368	0.861	-0.215914857	0.80351298
Q9NYT0	0.775	-0.367731785	0.80392027
O60831	0.784	-0.351074441	0.80392027
Q6UXH1	0.804	-0.314732593	0.80392027
O95372	0.839	-0.253257284	0.80392027
Q9UKK3	0.85	-0.234465254	0.80392027
P15927	1.142	0.191562651	0.80392027
P18583-5	1.146	0.196607044	0.80392027
Q9BW27	1.15	0.201633861	0.80410463
P29317	1.142	0.191562651	0.80432145
F5GWW9	0.795	-0.330973234	0.80478023
Q9H871	0.808	-0.307572802	0.80499986
O15357	0.841	-0.249822294	0.80499986
D3YHP0	1.254	0.326537348	0.80499986
P21589	0.806	-0.311148256	0.80504409
Q9NX40	0.854	-0.227692025	0.80515204
P17980	1.131	0.177598929	0.80515204
Q13601	1.15	0.201633861	0.80515204
Q8WUA2	1.159	0.212880566	0.80515204
B4DJL6	1.257	0.32998465	0.80521561
Q9Y6N5	0.848	-0.23786383	0.80529656
P03886	0.807	-0.309359421	0.80546909
P61289	1.129	0.175045486	0.80546909
Q16512-2	1.145	0.195347598	0.80546909
Q8NFW1	1.207	0.271425676	0.80546909
P11940	1.137	0.185232254	0.80619841
Q8WVM0	1.252	0.324234562	0.80619841
Q4KWH8	0.858	-0.220950447	0.8062452
O96019	1.143	0.192825404	0.8062452
Q9NYB0	0.853	-0.229382353	0.80718797
Q96S94	1.364	0.447843644	0.80746633
Q7L2H7	0.859	-0.219269964	0.80757898
O60930	1.374	0.458382004	0.80757898
Q96CU9	1.231	0.299830762	0.80758316
J3QKK3	1.253	0.325386415	0.80789004

O95674	0.783	-0.352915787	0.80792415
A3KMH1	0.821	-0.284545873	0.80792415
Q8IXH7	0.858	-0.220950447	0.80792415
Q8IUE6	1.136	0.183962835	0.80813682
O14802	1.264	0.337996464	0.80877533
Q96FX7	1.304	0.38294387	0.80893446
E7EWN9	0.762	-0.392137097	0.80940416
Q9BVJ6	0.844	-0.244685096	0.80943058
Q16656	1.271	0.34596403	0.80947661
Q24JP5	0.729	-0.45600928	0.81016343
O43715	0.785	-0.349235441	0.81016343
Q96GC9	0.814	-0.2968993	0.81016343
O15126	0.86	-0.217591435	0.81016343
B1AVU8	0.87	-0.200912694	0.81016343
B8ZZ54	1.139	0.187767747	0.81016343
P08621	1.14	0.189033824	0.81016343
Q9NWH9	1.151	0.202887833	0.81016343
Q13740	1.243	0.313826296	0.81016343
Q14331	1.249	0.320773477	0.81016343
O43813	0.841	-0.249822294	0.81020033
P12814	0.862	-0.214240226	0.81020033
Q9H3K6	1.148	0.199122642	0.81044947
D3YTC9	1.256	0.328836464	0.81044947
Q8IU81	1.258	0.331131922	0.81044947
Q9Y657	1.238	0.308011315	0.81112159
Q9NX20	0.86	-0.217591435	0.8116112
A8CG34	1.26	0.333423734	0.8116112
Q02818	0.842	-0.248107862	0.81170482
P68431	1.135	0.182692298	0.81170482
Q9Y5A9	1.145	0.195347598	0.81170482
Q9NV06	0.843	-0.246395464	0.81193623
F5H667	0.872	-0.19759996	0.81193623
P49756	1.137	0.185232254	0.81193623
Q969U7	1.172	0.22897257	0.81193623
Q9Y3D6	1.233	0.3021728	0.81215182
O95571	0.85	-0.234465254	0.81239921
P22626	1.134	0.18142064	0.81239921
Q9P000	1.236	0.305678743	0.81239921
P62857	0.864	-0.210896782	0.81251006
Q99714	0.863	-0.212567535	0.81254566
P35613-3	1.248	0.319617934	0.81254566
Q7Z7K0	0.783	-0.352915787	0.81371181
Q8NFU3	0.795	-0.330973234	0.81375328
Q6UB35	0.865	-0.209227962	0.81375328
Q8TCD5	0.845	-0.242976753	0.81396289
K7EQ21	0.734	-0.446148032	0.81427881

Q8N6S5	0.764	-0.388355457	0.81427881
J3KQN7	0.794	-0.332789088	0.81427881
P04066	0.803	-0.316528107	0.81427881
Q01518-2	0.864	-0.210896782	0.81427881
Q8IW35	1.315	0.3950628	0.81427881
F8W9T0	1.375	0.459431619	0.81427881
P35251	1.198	0.260627908	0.81443765
P61225	1.193	0.254594043	0.81448401
P11182	1.268	0.342554745	0.81448401
O95365	1.244	0.314986485	0.81470307
D6RG18	0.806	-0.311148256	0.81484344
P11142	0.864	-0.210896782	0.81484344
Q9BXB4	1.238	0.308011315	0.81484344
O43929	1.278	0.353887836	0.81484344
G3V1P0	1.311	0.390667686	0.81484344
Q5T5C7	0.867	-0.205896101	0.81516697
Q14697-2	0.864	-0.210896782	0.81533326
Q9HDC9	0.865	-0.209227962	0.81746985
P46100	0.865	-0.209227962	0.81746985
O43837	0.846	-0.241270432	0.81787858
P17931	1.132	0.178873958	0.81787858
O60784	1.142	0.191562651	0.81787858
P57088	1.146	0.196607044	0.81787858
H0YJV7	1.218	0.284514133	0.81787858
Q5JSL0	1.226	0.293958979	0.81787858
Q9BSH4	0.858	-0.220950447	0.81835149
Q9BWF3	0.87	-0.200912694	0.81846643
O15121	1.221	0.2880632	0.81883933
Q92765	0.854	-0.227692025	0.81902453
Q96AD0	1.195	0.257010618	0.81902453
O43683	0.848	-0.23786383	0.81982114
Q9H4M9	1.138	0.186500558	0.81982114
Q9Y3Z3	1.137	0.185232254	0.81990797
O43809	1.126	0.171206827	0.82055887
Q8NBJ5	1.145	0.195347598	0.82087221
Q8WXA9-2	1.198	0.260627908	0.82087221
P60660-2	0.808	-0.307572802	0.8212887
Q5SZ82	0.824	-0.279283757	0.82136293
O60763	0.87	-0.200912694	0.82137119
P05787	1.131	0.177598929	0.82137119
H7C1N3	0.818	-0.289827252	0.82173512
A6NJ78	0.824	-0.279283757	0.82173512
Q15024	1.144	0.194087052	0.82173512
B4DUA9	1.144	0.194087052	0.82173512
Q9H2P0	1.145	0.195347598	0.82173512
Q6P2C8	1.244	0.314986485	0.82173512

Q9H2D1	1.188	0.248534836	0.82205688
Q9Y2Q9	0.864	-0.210896782	0.82215422
F8WDL1	1.383	0.467801156	0.82216717
Q9H2U2	0.871	-0.199255376	0.82217788
F8W0Q9	0.808	-0.307572802	0.822505
O95232	1.137	0.185232254	0.822505
O94888	1.21	0.275007047	0.822505
Q8N806	1.232	0.301002256	0.822505
Q99661	1.236	0.305678743	0.822505
Q32P41	1.21	0.275007047	0.82276807
Q03154	0.77	-0.377069649	0.82291411
O00220	0.799	-0.323732592	0.82291411
P54709	0.875	-0.192645078	0.82291411
Q15428	1.125	0.169925001	0.82291411
D6RBZ0	1.13	0.176322773	0.82291411
P56182	1.138	0.186500558	0.82291411
Q6DKJ4	1.152	0.204140717	0.82291411
P08579	1.2	0.263034406	0.82291411
Q6PK18	1.247	0.318461465	0.82291411
Q8ND24	1.272	0.347098671	0.82291411
Q9UBK9	1.337	0.418999465	0.82292259
Q9HD33	0.847	-0.239566125	0.82297942
Q15773	1.139	0.187767747	0.82297942
Q8NC51-3	1.129	0.175045486	0.82372836
Q9H9B1	1.272	0.347098671	0.82402186
Q9BYN0	0.869	-0.202571918	0.82444739
P14868	0.867	-0.205896101	0.82448451
Q6UW02	0.792	-0.336427665	0.82454944
Q9NYP9	0.786	-0.347398782	0.8253144
P50552	0.849	-0.236163541	0.8253144
Q08J23	0.866	-0.20756107	0.8253144
C9J7Y4	0.786	-0.347398782	0.8253356
P35244	1.132	0.178873958	0.82539683
P05386	0.868	-0.204233052	0.82599868
Q15742	1.327	0.408168371	0.82599868
Q68CQ4	0.865	-0.209227962	0.82638251
Q712K3	1.238	0.308011315	0.82638251
Q9P2B7	0.737	-0.440263476	0.82752175
G3V3N4	0.802	-0.318325858	0.82752175
P82912	0.81	-0.304006187	0.82752175
Q15067	0.851	-0.232768963	0.82752175
Q8WWH5	0.862	-0.214240226	0.82752175
Q01082	1.128	0.173767068	0.82752175
Q07666	1.128	0.173767068	0.82752175
O00391	1.26	0.333423734	0.82752175
Q8TDB6	1.266	0.340277405	0.82752175

Q15813	1.145	0.195347598	0.82766144
E9PHT6	1.299	0.377401431	0.82791498
O95801	1.137	0.185232254	0.8286128
K7ERE1	0.825	-0.277533976	0.82883976
A6NMQ1	1.232	0.301002256	0.82887969
H0YAP1	1.292	0.36960607	0.82944142
O75438	1.172	0.22897257	0.82959297
Q9P013	0.795	-0.330973234	0.83073909
P46734	0.852	-0.231074664	0.83073909
F8VVT9	0.863	-0.212567535	0.83073909
Q96E39	1.291	0.368489001	0.83073909
P46782	0.869	-0.202571918	0.83080096
Q9Y5M8	0.873	-0.195946441	0.83092352
P23511	1.211	0.276198865	0.83092352
P43155	0.803	-0.316528107	0.83173491
Q96CT7	1.183	0.242450074	0.83173491
A6NIK2	0.796	-0.329159664	0.83231752
Q9Y5S2	1.182	0.241230036	0.83231752
Q16531	1.126	0.171206827	0.83262211
Q92614	0.812	-0.300448367	0.83294966
Q92890-1	1.13	0.176322773	0.83294966
Q9NVH1	1.137	0.185232254	0.83294966
O75116	1.21	0.275007047	0.83294966
Q8N442	1.219	0.285698126	0.83294966
Q9P031	1.297	0.37517848	0.83307565
Q9NVV4	0.863	-0.212567535	0.83352841
O14562	0.836	-0.258425153	0.83408157
Q99643	0.863	-0.212567535	0.83457741
Q6NSJ5	1.282	0.358396262	0.83457741
Q8NC96	1.295	0.372952098	0.83478702
J3KQG6	0.871	-0.199255376	0.83482796
Q9UBS4	1.132	0.178873958	0.83482796
P01024	1.262	0.33571191	0.83509299
Q14165	0.869	-0.202571918	0.83561029
O43707	0.871	-0.199255376	0.83561029
P53384	0.858	-0.220950447	0.8356674
Q15526	0.799	-0.323732592	0.83638467
O75380	1.195	0.257010618	0.83638467
P20929	1.221	0.2880632	0.83695517
P33908	0.795	-0.330973234	0.83802584
P06396	0.861	-0.215914857	0.8381719
O00567	0.871	-0.199255376	0.8381719
Q9NWU5	0.853	-0.229382353	0.83871967
Q9NUQ8	1.117	0.159629186	0.83871967
Q12888	0.852	-0.231074664	0.83880543
P51571	0.87	-0.200912694	0.83880543

P49755	1.122	0.166072676	0.83880543
Q13427	1.185	0.244887059	0.83880543
Q15050	1.133	0.180147861	0.83907225
Q9H773	0.872	-0.19759996	0.83930769
P46531	1.137	0.185232254	0.83952634
Q969G6	1.211	0.276198865	0.83958126
C9JPL0	1.228	0.296310561	0.83958126
F8W8D3	0.819	-0.288064643	0.83968713
J3KNP0	0.82	-0.286304185	0.83989098
K7ERC8	0.828	-0.272297327	0.83989098
Q9BRK5	0.864	-0.210896782	0.83989098
P84098	0.868	-0.204233052	0.83989098
P0CAP1-4	0.775	-0.367731785	0.84032572
P22670	0.802	-0.318325858	0.84032572
K7EMU1	0.875	-0.192645078	0.84032572
Q9Y3B9	0.875	-0.192645078	0.84032572
J3KN69	1.159	0.212880566	0.84032572
H3BSW6	1.206	0.270229907	0.84032572
Q9GZN1	1.281	0.357270476	0.84032572
F5H6A3	0.741	-0.432454552	0.84062208
Q969E2	0.803	-0.316528107	0.84062208
Q9UK45	0.858	-0.220950447	0.84062208
Q9UJZ1	0.872	-0.19759996	0.84062208
C9JUF4	1.184	0.243669081	0.84062208
Q13309	1.205	0.269033146	0.84062208
P62875	1.223	0.290424404	0.84062208
F5H7W8	1.328	0.409255147	0.84062208
Q9BTT0	1.13	0.176322773	0.84065309
Q9NUU7	1.122	0.166072676	0.84086616
Q15365	1.122	0.166072676	0.84146073
O14893	0.818	-0.289827252	0.84202047
Q9Y5J1	0.854	-0.227692025	0.84202047
Q9UJX2	0.858	-0.220950447	0.84202047
Q9Y3A4	0.859	-0.219269964	0.84202047
J3KS15	0.865	-0.209227962	0.84202047
Q7Z4H3	0.868	-0.204233052	0.84202047
Q7KZI7	0.872	-0.19759996	0.84202047
O43765	0.878	-0.187707155	0.84202047
P04350	1.11	0.150559677	0.84202047
Q9BXP5	1.126	0.171206827	0.84202047
P31942	1.131	0.177598929	0.84202047
F8WAK8	1.172	0.22897257	0.84202047
P14618-2	1.188	0.248534836	0.84202047
Q66PJ3	1.216	0.282143229	0.84202047
Q9BWS9	1.261	0.334568276	0.84202047
Q9BVC3	1.273	0.348232419	0.84202047

E9PKN0	1.339	0.421155961	0.84202047
B0UXB6	0.812	-0.300448367	0.84231579
Q8WVY7	1.133	0.180147861	0.84231579
H0YBD2	0.842	-0.248107862	0.84335387
Q96GW9	0.748	-0.418889825	0.84398272
Q6P275	0.749	-0.416962376	0.84398272
F8VVA0	0.779	-0.360304767	0.84398272
Q12996	0.866	-0.20756107	0.84398272
Q99613	0.874	-0.194294815	0.84398272
P26599	1.12	0.163498732	0.84398272
O15347	1.129	0.175045486	0.84398272
P12931	1.222	0.289244285	0.84398272
Q9P246	1.283	0.35952117	0.84398272
Q9BTZ2	0.869	-0.202571918	0.84428551
P27635	0.874	-0.194294815	0.84428551
Q99698	0.749	-0.416962376	0.84440064
Q13636	0.767	-0.382701517	0.84489847
B3KV61	0.769	-0.378944497	0.84489847
Q9NUJ1	0.875	-0.192645078	0.84489847
Q9Y2P8	1.14	0.189033824	0.84489847
P78346	1.182	0.241230036	0.84489847
O14979	1.119	0.162210036	0.84567618
Q96I15	0.866	-0.20756107	0.84572106
Q06587	1.217	0.283329168	0.84572106
Q9NQC3-2	0.872	-0.19759996	0.84621403
Q9NPH2-2	1.202	0.265436896	0.84621403
Q96G23	0.869	-0.202571918	0.84630036
Q7Z6I6	0.804	-0.314732593	0.846428
Q4G0A6	0.757	-0.401634795	0.84648725
O95772	0.769	-0.378944497	0.84648725
Q6NUM9	0.804	-0.314732593	0.84648725
P62158	0.875	-0.192645078	0.84648725
P22061	1.123	0.167357928	0.84648725
Q9NRL2	1.184	0.243669081	0.84648725
O15084-1	1.181	0.240008965	0.84662131
Q9Y320	0.83	-0.268816758	0.84688407
P09669	0.858	-0.220950447	0.84688407
O94919	1.215	0.280956314	0.84688407
H0YIQ7	1.328	0.409255147	0.84689951
O00231	1.118	0.160920188	0.84703249
Q15102	1.125	0.169925001	0.84747936
P48681	0.803	-0.316528107	0.84752301
F5H872	0.808	-0.307572802	0.84821912
P62714	1.125	0.169925001	0.84825851
Q9UIV1	1.206	0.270229907	0.84847932
Q96D46	1.131	0.177598929	0.84862146

P50570	0.875	-0.192645078	0.84911396
Q9NW82	1.219	0.285698126	0.84911396
Q9BQ70	1.123	0.167357928	0.84962435
P68133	1.117	0.159629186	0.84998778
Q12765	0.856	-0.224317298	0.85033463
Q8N766	0.857	-0.222632891	0.85033463
O43237	0.868	-0.204233052	0.85033463
Q9Y6I3	1.275	0.350497247	0.85033463
Q13126	1.117	0.159629186	0.85057448
E5RJ68	0.876	-0.190997225	0.85086644
Q14185	0.812	-0.300448367	0.85094413
E5RGS9	1.216	0.282143229	0.85094413
P61158	0.875	-0.192645078	0.85105562
Q86YS7	0.812	-0.300448367	0.85200197
A6NHS7	0.861	-0.215914857	0.85200197
F8VQ10	1.117	0.159629186	0.85200197
Q86XZ4	1.184	0.243669081	0.85261245
Q96AG4	0.877	-0.189351252	0.85336306
P06132	0.86	-0.217591435	0.85343987
O15143	0.883	-0.179514657	0.85348645
Q8IWR0	1.326	0.407080775	0.85356178
F5GXX5	1.123	0.167357928	0.85453352
O75717	1.165	0.220329955	0.85541669
D6RD44	0.787	-0.345564459	0.85647597
Q8TEA8	0.867	-0.205896101	0.85647597
Q13232	0.869	-0.202571918	0.85647597
Q8N0U8	0.869	-0.202571918	0.85647597
Q8IZP0	0.875	-0.192645078	0.85647597
O60231	1.112	0.153156788	0.85647597
B4DLH4	1.118	0.160920188	0.85647597
Q9BVK6	1.119	0.162210036	0.85647597
F8W038	1.119	0.162210036	0.85647597
Q8NF37	1.185	0.244887059	0.85647597
B7Z9K1	1.322	0.402722177	0.85647597
Q5JTZ9	1.326	0.407080775	0.85670806
Q9Y2C4	1.265	0.339137385	0.856997
P52756	0.881	-0.182786076	0.85712525
Q96A33	1.121	0.164786278	0.85712525
Q9BW60	1.215	0.280956314	0.85712525
B5MDU6	1.218	0.284514133	0.85712525
Q9BVV7	1.212	0.277389699	0.85712676
Q9NP79	0.862	-0.214240226	0.857172
Q14318	1.213	0.27857955	0.85747927
Q9NRR3	1.213	0.27857955	0.85747927
F8WBK6	0.818	-0.289827252	0.85802634
P62166	0.821	-0.284545873	0.85802634

Q8TEB1	0.823	-0.281035664	0.85802634
Q9UMY4	0.825	-0.277533976	0.85802634
Q9H3Z4	0.845	-0.242976753	0.85802634
Q9Y305	0.864	-0.210896782	0.85802634
Q8TC12	0.865	-0.209227962	0.85802634
Q5JPE7	0.875	-0.192645078	0.85802634
Q9UIJ7	0.875	-0.192645078	0.85802634
P68371	1.114	0.155749233	0.85802634
Q9NTI5	1.173	0.230203013	0.85802634
B9A049	0.789	-0.341902795	0.85823086
H3BR29	0.823	-0.281035664	0.85823086
P32455	0.877	-0.189351252	0.85823086
P51970	0.879	-0.18606493	0.85823086
P04004	1.114	0.155749233	0.85823086
Q9Y617	0.879	-0.18606493	0.85830869
Q15637-6	1.114	0.155749233	0.85862928
Q9NYV4	0.825	-0.277533976	0.85865564
P49247	1.155	0.207892852	0.85865564
O43181	1.176	0.23388806	0.85874342
F8WC97	0.809	-0.305788392	0.85886139
Q8N201	0.833	-0.263611599	0.85886139
Q9NZ45	0.86	-0.217591435	0.85886139
B7Z6G2	0.864	-0.210896782	0.85886139
Q9Y3C6	0.88	-0.184424571	0.85886139
D2IYK5	1.291	0.368489001	0.85886139
J3KPG5	1.239	0.309176187	0.85901052
Q8NBJ7	0.863	-0.212567535	0.85910476
E9PBB4	0.747	-0.420819852	0.85912848
E7EWM1	0.767	-0.382701517	0.85912848
Q00796	0.883	-0.179514657	0.85912848
Q9UMS4	1.113	0.154453593	0.85914567
Q9Y399	0.864	-0.210896782	0.85926881
Q9BQ39	1.217	0.283329168	0.85930253
Q2TAA2	0.833	-0.263611599	0.85985441
P07355	0.88	-0.184424571	0.85985441
Q9NQ92	1.268	0.342554745	0.85985441
O43148	0.875	-0.192645078	0.85998436
Q15388	0.879	-0.18606493	0.85998436
Q5W0H4	0.88	-0.184424571	0.85998436
Q13616	1.112	0.153156788	0.85998436
Q13247	1.113	0.154453593	0.85998436
Q9Y4C8	1.203	0.266636643	0.85998436
E9PHH9	1.211	0.276198865	0.85998436
Q96GX9	0.824	-0.279283757	0.86047568
Q12800	1.124	0.168642036	0.86047856
Q96D53	0.816	-0.293358943	0.86067343

Q8WW12	0.871	-0.199255376	0.86091291
Q92945	1.112	0.153156788	0.86091291
F5H012	1.234	0.303342394	0.86091291
Q96RF0	1.268	0.342554745	0.86091291
B4E3W0	1.287	0.364012054	0.86091291
B4DRL5	1.29	0.367371066	0.86091291
B7Z7F3	0.792	-0.336427665	0.86128223
Q9H3P2	0.865	-0.209227962	0.86128223
P28838	0.881	-0.182786076	0.86176759
Q6Y1H2	1.242	0.312665174	0.8625835
Q76FK4	0.815	-0.295128036	0.86329881
Q96AX1	0.818	-0.289827252	0.86329881
P31946	0.881	-0.182786076	0.86332493
F5H2Q7	1.292	0.36960607	0.86332493
P51003	1.121	0.164786278	0.863375
Q9UNI6	1.192	0.253384236	0.86339687
Q5R3B4	1.247	0.318461465	0.86365047
Q9NX02	0.867	-0.205896101	0.86436468
O15297	1.307	0.386259141	0.86436468
Q96B36	1.228	0.296310561	0.86560046
Q9HBI1	0.819	-0.288064643	0.86581379
P05141	0.882	-0.181149439	0.86626174
Q06546	1.189	0.249748715	0.86632816
P18887	1.187	0.247319935	0.86719899
Q9H0E2	1.205	0.269033146	0.86719899
Q15910	1.19	0.250961574	0.86732704
P35914	0.853	-0.229382353	0.86774578
Q69YN2	1.164	0.219091058	0.86790281
O00400	1.211	0.276198865	0.86794259
Q9NWY4	1.169	0.22527493	0.86867064
Q8TDQ7	1.207	0.271425676	0.86869876
P61970	1.105	0.14404637	0.86883536
E7ESP4	1.233	0.3021728	0.86889418
Q96RR1	1.256	0.328836464	0.86889418
A0PJW6	0.776	-0.365871442	0.86954332
Q9Y624	1.169	0.22527493	0.87024174
Q00534	1.236	0.305678743	0.87034235
Q9UBL6	0.837	-0.256700472	0.8704361
F2Z329	0.76	-0.395928676	0.87052714
Q14191	0.794	-0.332789088	0.87052714
P40123	0.84	-0.251538767	0.87052714
F8W8Z9	0.866	-0.20756107	0.87052714
E7EQS0	0.878	-0.187707155	0.87052714
O95299	0.882	-0.181149439	0.87052714
Q9UHB9	0.893	-0.16326792	0.87052714
P04040	1.113	0.154453593	0.87052714

Q15021	1.116	0.158337027	0.87052714
Q13951-2	1.159	0.212880566	0.87052714
Q5SWX3	1.182	0.241230036	0.87052714
Q13003	1.186	0.24610401	0.87052714
O43251-8	1.197	0.259423152	0.87052714
Q8N8R5	1.218	0.284514133	0.87052714
Q9NPF5	1.221	0.2880632	0.87052714
Q6NT55	1.255	0.327687364	0.87052714
Q96MW5	0.798	-0.325539348	0.87057919
Q8IUX4	1.253	0.325386415	0.87134994
Q9UKY7	1.117	0.159629186	0.87153652
Q14103	0.884	-0.177881725	0.87193829
E7EMM4	0.871	-0.199255376	0.87214576
Q8WTT2	0.878	-0.187707155	0.87214576
Q96ED9	1.204	0.267835392	0.87214576
Q8IYE0	1.206	0.270229907	0.87214576
Q6PK04	1.209	0.273814245	0.87214576
O14737	1.111	0.151858817	0.87227187
Q9P265	0.832	-0.265344567	0.87250835
Q8TB36	0.841	-0.249822294	0.87250835
Q9NWV4	0.856	-0.224317298	0.87250835
O75146	0.877	-0.189351252	0.87250835
Q9BRL6	0.892	-0.164884385	0.87250835
P83916	1.114	0.155749233	0.87250835
Q9NTJ5	1.117	0.159629186	0.87250835
P24386	1.179	0.237563718	0.87250835
Q9UBV2	1.194	0.255802837	0.87376338
Q8IYI6	0.795	-0.330973234	0.87452924
P11137	0.82	-0.286304185	0.87452924
P42025	0.827	-0.274040765	0.87452924
K7ERA3	0.835	-0.260151897	0.87452924
Q9UG56	0.837	-0.256700472	0.87452924
Q9P003	0.841	-0.249822294	0.87452924
Q8NE86	0.847	-0.239566125	0.87452924
F5GWH5	0.86	-0.217591435	0.87452924
Q5QJE6	0.869	-0.202571918	0.87452924
Q32MZ4-2	0.871	-0.199255376	0.87452924
Q7Z2W9	0.871	-0.199255376	0.87452924
Q8WVJ2	0.878	-0.187707155	0.87452924
O75439	0.879	-0.18606493	0.87452924
P27348	0.886	-0.174621396	0.87452924
P62942	0.887	-0.17299399	0.87452924
Q86Y39	0.89	-0.168122759	0.87452924
Q9ULV4	0.891	-0.166502663	0.87452924
Q8NI22	0.891	-0.166502663	0.87452924
P17812	1.099	0.136191386	0.87452924

Q9UQE7	1.104	0.142740172	0.87452924
A8MW61	1.111	0.151858817	0.87452924
P46926	1.113	0.154453593	0.87452924
Q9H6Z4	1.113	0.154453593	0.87452924
Q8WWM7	1.116	0.158337027	0.87452924
E9PRJ8	1.138	0.186500558	0.87452924
O75381	1.155	0.207892852	0.87452924
Q9BRX2	1.156	0.209141398	0.87452924
G5E9I6	1.162	0.216610069	0.87452924
Q13112	1.171	0.227741076	0.87452924
H7C1E4	1.184	0.243669081	0.87452924
Q9Y6K9	1.196	0.25821739	0.87452924
Q9UH62	1.197	0.259423152	0.87452924
Q658Y4	1.2	0.263034406	0.87452924
Q8TB61	1.212	0.277389699	0.87452924
F5GYA4	1.238	0.308011315	0.87452924
E9PBM2	1.295	0.372952098	0.87452924
Q9Y4Z0	1.111	0.151858817	0.87499927
Q96T58	0.874	-0.194294815	0.87514343
P04844	1.105	0.14404637	0.87515759
Q71UI9	1.105	0.14404637	0.87532712
Q9P0U1	0.875	-0.192645078	0.87539076
P19623	1.113	0.154453593	0.87539076
Q9Y3E7	0.821	-0.284545873	0.87573485
E9PR17	0.886	-0.174621396	0.87590084
P43307	0.893	-0.16326792	0.87590084
O15355	1.099	0.136191386	0.87590084
Q9UNM6	1.104	0.142740172	0.87590084
P61221	1.104	0.142740172	0.87590084
Q6NS38	1.192	0.253384236	0.87590084
Q13356	1.22	0.286881148	0.87590084
Q5SRE5	0.868	-0.204233052	0.87593134
Q96S59	1.166	0.221567789	0.87652189
P16930	0.878	-0.187707155	0.87685582
Q9NZ01	1.11	0.150559677	0.87685582
Q13188	1.189	0.249748715	0.87689721
O14981	0.875	-0.192645078	0.87705538
E7EXA6	1.268	0.342554745	0.87717379
J3QRY2	0.775	-0.367731785	0.87721646
P34949	0.873	-0.195946441	0.87721646
Q9H2H8	0.889	-0.169744676	0.87721646
Q5T1C6	1.197	0.259423152	0.87721646
Q8IYL3	1.273	0.348232419	0.87721646
P35221	1.103	0.141432791	0.87750654
O15305	0.862	-0.214240226	0.8776503
B4DM74	0.888	-0.171368418	0.8776503

O75368	1.122	0.166072676	0.87779215
P49720	1.099	0.136191386	0.87798724
Q96A65	0.846	-0.241270432	0.87804325
H0YGR4	0.865	-0.209227962	0.87804325
Q00169	0.877	-0.189351252	0.87804325
Q709C8	0.883	-0.179514657	0.87804325
F5GWX5	1.103	0.141432791	0.87804325
P04062	0.846	-0.241270432	0.87849011
Q9BSJ2	1.187	0.247319935	0.87870792
P05423	1.242	0.312665174	0.87906151
P11021	0.888	-0.171368418	0.87910215
Q8IXM3	0.887	-0.17299399	0.87959893
Q9GZP8	1.161	0.215367972	0.87983845
Q9H944	0.852	-0.231074664	0.88008294
G3V3G9	0.829	-0.270555993	0.88051494
Q8NBY1	0.837	-0.256700472	0.88051494
B4DV96	0.839	-0.253257284	0.88051494
Q7Z6V5	0.843	-0.246395464	0.88051494
O76024	0.849	-0.236163541	0.88051494
Q9BXS5	0.887	-0.17299399	0.88051494
Q9NR30	0.888	-0.171368418	0.88051494
P62258	0.888	-0.171368418	0.88051494
P22059	0.888	-0.171368418	0.88051494
O60832	0.893	-0.16326792	0.88051494
Q92900	1.095	0.13093087	0.88051494
P78371	1.102	0.140124224	0.88051494
O75367	1.102	0.140124224	0.88051494
Q96EY5	1.152	0.204140717	0.88051494
Q96HR9	1.19	0.250961574	0.88051494
Q58DX5	1.231	0.299830762	0.88051494
O15066	1.241	0.311503115	0.88063569
O95235	0.886	-0.174621396	0.88082646
Q9GZY8	0.84	-0.251538767	0.88092525
Q9UKM9	0.897	-0.15682011	0.88107322
P51687	0.851	-0.232768963	0.88142462
P49411	0.889	-0.169744676	0.88142462
P49792	0.889	-0.169744676	0.88168148
Q9BT09	0.877	-0.189351252	0.88176203
P55036	1.107	0.146655222	0.8818785
Q9P0P8	0.83	-0.268816758	0.88198828
Q9UK61	0.832	-0.265344567	0.88202423
O43776	0.889	-0.169744676	0.88202423
Q7L5Y1	0.836	-0.258425153	0.88248946
Q9HCG8	1.152	0.204140717	0.88248946
Q8TB70	1.204	0.267835392	0.88256934
Q7L5N7	0.844	-0.244685096	0.88268021

Q96IU4	0.874	-0.194294815	0.88268021
P24666	1.112	0.153156788	0.88268021
Q9H204	1.226	0.293958979	0.88277522
Q9Y343	0.786	-0.347398782	0.88330243
P03923	0.838	-0.254977851	0.88330243
Q8NC56	0.847	-0.239566125	0.88391861
Q02878	0.89	-0.168122759	0.88391861
Q9HBH1	0.831	-0.267079618	0.88395277
O43318	0.833	-0.263611599	0.88395277
P14625	0.89	-0.168122759	0.88395277
P63167	1.1	0.137503524	0.88395277
Q6UXN9	1.157	0.210388864	0.88395277
P40429	0.89	-0.168122759	0.88405188
Q8TD16	1.232	0.301002256	0.88444136
Q92541	0.841	-0.249822294	0.88445059
Q5TFE4	1.102	0.140124224	0.8845262
Q96ST2	0.876	-0.190997225	0.88478745
P17480	1.1	0.137503524	0.88478745
Q9Y3L3	0.85	-0.234465254	0.88559664
Q9UBE0	1.099	0.136191386	0.885723
Q9Y4W2	1.144	0.194087052	0.885723
P35226	1.187	0.247319935	0.88573351
Q4G0P3	0.83	-0.268816758	0.88616304
Q9Y2V7	0.833	-0.263611599	0.88616304
Q13561	1.107	0.146655222	0.88616304
Q8IVS2	1.142	0.191562651	0.88616304
P13929	1.169	0.22527493	0.88616304
Q9Y2Z9	1.174	0.231432408	0.88616304
Q96GM8	1.227	0.295135249	0.88616304
Q9C035	1.27	0.344828497	0.88616304
Q6ZRP7	0.878	-0.187707155	0.8868471
Q9UDW1	1.11	0.150559677	0.88747235
Q16222	0.89	-0.168122759	0.88785472
O15047	0.776	-0.365871442	0.88786591
Q8NEC6	0.828	-0.272297327	0.88786591
Q9UNN8	0.842	-0.248107862	0.88786591
Q96B23-2	0.844	-0.244685096	0.88786591
E9PAZ2	0.847	-0.239566125	0.88786591
Q9BSH5	0.848	-0.23786383	0.88786591
Q8ND76	0.848	-0.23786383	0.88786591
Q9ULE4	0.857	-0.222632891	0.88786591
P18084	0.859	-0.219269964	0.88786591
P53597	0.866	-0.20756107	0.88786591
Q9NRX4	0.869	-0.202571918	0.88786591
Q13907	0.876	-0.190997225	0.88786591
Q08380	0.876	-0.190997225	0.88786591

O95747	0.881	-0.182786076	0.88786591
P62070	0.884	-0.177881725	0.88786591
P13807	0.89	-0.168122759	0.88786591
P52298	0.89	-0.168122759	0.88786591
P62244	0.891	-0.166502663	0.88786591
P62280	0.892	-0.164884385	0.88786591
P12532	0.893	-0.16326792	0.88786591
P18124	0.893	-0.16326792	0.88786591
Q9HCE1	0.894	-0.161653263	0.88786591
C9JZY6	0.896	-0.158429363	0.88786591
O14569	0.896	-0.158429363	0.88786591
P09622	0.898	-0.15521265	0.88786591
P12270	1.088	0.121678557	0.88786591
P00403	1.09	0.124328135	0.88786591
O96008	1.092	0.126972856	0.88786591
P09661	1.094	0.129612738	0.88786591
P52272	1.096	0.132247798	0.88786591
Q01105-2	1.096	0.132247798	0.88786591
Q13813	1.097	0.133563526	0.88786591
P07737	1.097	0.133563526	0.88786591
Q86V81	1.098	0.134878054	0.88786591
P23258	1.103	0.141432791	0.88786591
P18859	1.103	0.141432791	0.88786591
O00411	1.104	0.142740172	0.88786591
G3V325	1.104	0.142740172	0.88786591
Q7L1Q6	1.106	0.145351386	0.88786591
O14880	1.107	0.146655222	0.88786591
Q9H0W5	1.109	0.149259365	0.88786591
Q96GG9	1.119	0.162210036	0.88786591
P49770	1.122	0.166072676	0.88786591
P35813	1.141	0.190298792	0.88786591
F5H315	1.143	0.192825404	0.88786591
Q96JB5	1.149	0.200378798	0.88786591
Q9UNE7	1.149	0.200378798	0.88786591
E9PC74	1.151	0.202887833	0.88786591
Q9NR50	1.153	0.205392513	0.88786591
Q5RGS4	1.165	0.220329955	0.88786591
Q6KC79	1.166	0.221567789	0.88786591
Q99590	1.169	0.22527493	0.88786591
G8JLF3	1.173	0.230203013	0.88786591
O94842	1.179	0.237563718	0.88786591
Q8IYB7	1.181	0.240008965	0.88786591
O75688	1.183	0.242450074	0.88786591
Q9GZP9	1.184	0.243669081	0.88786591
E9PHY0	1.185	0.244887059	0.88786591
Q9Y5Y5	1.215	0.280956314	0.88786591

Q15542	1.245	0.316145742	0.88786591
Q9NXR1	1.261	0.334568276	0.88786591
Q9UJ83	1.262	0.33571191	0.88786591
Q6P2P2	1.271	0.34596403	0.88786591
F5GWD3	0.839	-0.253257284	0.88810301
Q9BQ48	0.84	-0.251538767	0.88810301
P01116	0.849	-0.236163541	0.88810301
Q14674	0.851	-0.232768963	0.88810301
F6W7K9	0.853	-0.229382353	0.88810301
Q13098	0.878	-0.187707155	0.88810301
Q13671	0.878	-0.187707155	0.88810301
Q13405	0.888	-0.171368418	0.88810301
P08754	0.892	-0.164884385	0.88810301
Q8TAE8	0.895	-0.160040413	0.88810301
P30049	0.896	-0.158429363	0.88810301
B4DUT8	1.101	0.138814469	0.88810301
Q71TU5	1.102	0.140124224	0.88810301
Q10570	1.109	0.149259365	0.88810301
O00762	1.136	0.183962835	0.88810301
C9JJ19	1.149	0.200378798	0.88810301
O95139	1.149	0.200378798	0.88810301
Q13033-2	1.151	0.202887833	0.88810301
Q9Y5J9	1.18	0.23878686	0.88810301
O43826	1.217	0.283329168	0.88810301
E7EVL4	1.197	0.259423152	0.88881694
Q9NQE9	1.22	0.286881148	0.88889214
Q9H488	0.903	-0.147202107	0.88918115
O43920	0.893	-0.16326792	0.89002707
Q9UKK6	1.186	0.24610401	0.89019187
H7C089	1.258	0.331131922	0.89019187
E9PB35	0.83	-0.268816758	0.89040299
E9PLY5	0.838	-0.254977851	0.89040299
E9PQ57	0.878	-0.187707155	0.89040299
P17301	0.885	-0.17625064	0.89040299
P61019	0.899	-0.153606979	0.89040299
J3KSL8	0.903	-0.147202107	0.89040299
Q9NZT2	1.107	0.146655222	0.89040299
Q9UMY1	1.147	0.197865391	0.89040299
Q96GM5	1.178	0.236339539	0.89040299
E9PJK4	1.222	0.289244285	0.89040299
Q9BW61	0.855	-0.226003675	0.89050188
B1ANB7	0.856	-0.224317298	0.89052741
H7BXZ6	0.855	-0.226003675	0.89086053
Q9UHY7	0.88	-0.184424571	0.89086053
Q9NSI2	0.892	-0.164884385	0.89086053
O95817	1.101	0.138814469	0.89086053

Q96J02	1.158	0.211635253	0.89086053
Q8N3D4	1.232	0.301002256	0.89086053
Q9Y2I1	1.239	0.309176187	0.89086053
Q9Y4X5	0.894	-0.161653263	0.89157948
Q7Z739	1.106	0.145351386	0.89157948
Q8N7H5	1.118	0.160920188	0.89157948
Q96L92	1.152	0.204140717	0.89157948
Q96CW5	1.184	0.243669081	0.89157948
Q9Y6Q5-2	1.223	0.290424404	0.89157948
P32121	0.89	-0.168122759	0.89157957
E5RFF9	1.161	0.215367972	0.8921887
G3V2F7	1.091	0.125651102	0.89268392
F8W9S7	0.881	-0.182786076	0.89296106
Q14008	1.093	0.128293401	0.89344418
H7C173	1.221	0.2880632	0.89344418
P11217	1.159	0.212880566	0.89358673
J3KQN4	0.882	-0.181149439	0.89386593
Q14573	0.895	-0.160040413	0.89386593
P15529-2	0.821	-0.284545873	0.89401396
Q8IWL3	0.859	-0.219269964	0.89442525
Q13310	0.897	-0.15682011	0.89456373
P07948	1.173	0.230203013	0.89474648
P60510	1.1	0.137503524	0.89534615
Q86UP2	0.905	-0.144010303	0.89577329
Q9Y3C4	0.854	-0.227692025	0.89597413
Q9NS69	1.096	0.132247798	0.89597413
O43913	0.847	-0.239566125	0.89615727
F5GX70	0.802	-0.318325858	0.89623443
Q9Y232	0.857	-0.222632891	0.89623443
Q96G03	0.884	-0.177881725	0.89623443
Q5VTU8	0.887	-0.17299399	0.89623443
Q9NZB2	0.901	-0.150400989	0.89623443
Q99417	0.901	-0.150400989	0.89623443
Q15836	1.149	0.200378798	0.89623443
P02656	1.169	0.22527493	0.89623443
Q53F19	1.17	0.22650853	0.89623443
Q9H0R4	1.197	0.259423152	0.89623443
Q9NVR2	1.202	0.265436896	0.89649282
P62879	0.896	-0.158429363	0.89726147
Q03518	0.857	-0.222632891	0.89751542
B7ZB17	0.896	-0.158429363	0.89786738
G8JL95	1.217	0.283329168	0.89811063
Q8N128	0.854	-0.227692025	0.89842015
Q9NX58	0.9	-0.152003093	0.89842015
Q6NVY1	1.093	0.128293401	0.89852701
Q9Y2H1	0.838	-0.254977851	0.89906368

O75223	0.883	-0.179514657	0.89906368
Q9NTK5	1.09	0.124328135	0.89935155
P60900	1.09	0.124328135	0.89940763
Q8IWA4	1.199	0.261831659	0.89950976
A6NGJ0	0.854	-0.227692025	0.8995614
C9JRZ6	0.882	-0.181149439	0.8995614
Q8TD19	0.887	-0.17299399	0.8995614
P55327	0.899	-0.153606979	0.8995614
O15116	1.141	0.190298792	0.8995614
Q7Z460	1.157	0.210388864	0.8995614
Q9NYB9	1.169	0.22527493	0.8995614
C9JIX4	1.206	0.270229907	0.8995614
H0YCN2	0.787	-0.345564459	0.89976141
Q13509	1.09	0.124328135	0.89976141
O15294	1.091	0.125651102	0.89976141
Q13573	1.094	0.129612738	0.89976141
Q8N8R3	1.204	0.267835392	0.89976141
Q9H116	1.252	0.324234562	0.89976141
P62266	0.899	-0.153606979	0.89977275
C9J185	0.848	-0.23786383	0.8998419
Q13445	0.878	-0.187707155	0.8998419
Q9H8S9	1.081	0.112366523	0.8998419
P27448	0.817	-0.291592017	0.89991252
O14641	0.861	-0.215914857	0.89991252
P13489	1.081	0.112366523	0.90036914
B4DR87	1.098	0.134878054	0.90047471
Q86UE4	1.098	0.134878054	0.90047471
O14907	1.142	0.191562651	0.90047471
P15407	0.863	-0.212567535	0.90073334
O75400-3	1.096	0.132247798	0.90074176
Q9UNQ2	1.103	0.141432791	0.90074176
E9PHA2	1.158	0.211635253	0.90074176
E7EU96	0.899	-0.153606979	0.90101121
O94903	0.888	-0.171368418	0.90102041
Q9H7X3	0.849	-0.236163541	0.90125302
Q5GLZ8	0.911	-0.134477041	0.90125302
P57105	0.912	-0.13289427	0.90125302
Q8IYB3	1.088	0.121678557	0.90125302
P40937	1.092	0.126972856	0.90125302
O43379-2	1.206	0.270229907	0.90125302
Q7Z2K8	1.172	0.22897257	0.90155573
H0Y6I0	0.8	-0.321928095	0.901683
O95182	0.855	-0.226003675	0.901683
Q5JVF3	0.893	-0.16326792	0.901683
Q9HAV4	0.895	-0.160040413	0.901683
O95486	0.903	-0.147202107	0.901683

O75506	1.081	0.112366523	0.901683
Q14980	1.086	0.119024103	0.901683
Q9H3U1	1.095	0.13093087	0.901683
Q9Y312	1.139	0.187767747	0.901683
J3QQY0	1.169	0.22527493	0.901683
Q9NVC6	1.194	0.255802837	0.901683
O43399-3	1.196	0.25821739	0.901683
Q8IWT6	1.205	0.269033146	0.901683
Q99676	1.246	0.317304068	0.901683
Q16563-2	1.094	0.129612738	0.90194532
Q9NYL9	0.887	-0.17299399	0.90258403
P00568	0.9	-0.152003093	0.90264617
K7EN78	0.841	-0.249822294	0.90291156
Q96PK6	0.9	-0.152003093	0.90291156
E9PFH8	0.9	-0.152003093	0.90316097
E9PBJ5	0.901	-0.150400989	0.90316097
O14672	0.867	-0.205896101	0.90320393
P20226	0.853	-0.229382353	0.90341944
P08237	0.898	-0.15521265	0.90341944
F5GXE4	0.857	-0.222632891	0.90412924
P61006	0.9	-0.152003093	0.90412924
P50225	0.904	-0.145605322	0.90412924
P53680	0.911	-0.134477041	0.90412924
Q9BZK7	1.083	0.115033243	0.90412924
Q14692	1.092	0.126972856	0.90412924
Q9H008	1.143	0.192825404	0.90412924
Q5BJF6	1.248	0.319617934	0.90412924
Q6ZN18	0.791	-0.3382504	0.90461902
Q86X83	0.85	-0.234465254	0.90468581
Q16626	0.857	-0.222632891	0.90491185
Q8WUX2	0.861	-0.215914857	0.90491185
P55769	0.897	-0.15682011	0.90491185
Q12931	0.901	-0.150400989	0.90491185
P62424	0.901	-0.150400989	0.90491185
P84085	0.901	-0.150400989	0.90491185
Q5JWF2	1.099	0.136191386	0.90491185
P98175	1.127	0.172487516	0.90491185
Q9UMX5	1.142	0.191562651	0.90491185
B7Z9U0	1.189	0.249748715	0.90491185
Q969H6	1.2	0.263034406	0.90491185
Q15369	1.093	0.128293401	0.90527801
F5GXY5	1.071	0.09895848	0.90537276
Q86TU7	1.157	0.210388864	0.90537276
P11233	0.899	-0.153606979	0.9060234
Q9H074	1.093	0.128293401	0.90615556
F5GZY7	1.197	0.259423152	0.90615556

Q15061	0.892	-0.164884385	0.90654767
Q9UP83	1.196	0.25821739	0.90654767
Q8NFF5	0.875	-0.192645078	0.90696852
E9PHI4	0.895	-0.160040413	0.90696852
Q6NUK1	1.144	0.194087052	0.90696852
Q9C0J8	1.163	0.217851097	0.90696852
K7EQL6	1.242	0.312665174	0.90766543
O95755	1.177	0.23511432	0.90803072
P46976	0.894	-0.161653263	0.90823019
Q9NYH9	0.898	-0.15521265	0.90823019
P61204	0.903	-0.147202107	0.90823019
P55210	0.907	-0.140825544	0.90823019
Q15397	1.086	0.119024103	0.90823019
P52888	1.087	0.12035194	0.90823019
Q9P2W9	1.133	0.180147861	0.90823019
Q9Y2Q5	1.092	0.126972856	0.90837054
Q5VTR2	1.085	0.117695043	0.90843397
E7EQ69	1.086	0.119024103	0.90878548
Q9BZF1	1.089	0.123003954	0.90878548
O95292	0.903	-0.147202107	0.90886998
P78381	0.83	-0.268816758	0.9090125
Q9P2E9	0.9	-0.152003093	0.90905723
Q9Y3B7	0.893	-0.16326792	0.90937676
O95865	0.854	-0.227692025	0.90940654
Q7Z4V5	0.899	-0.153606979	0.90940654
O00299	0.903	-0.147202107	0.90940654
Q9Y6K0	1.154	0.206643224	0.90940654
H7BZK6	1.198	0.260627908	0.90940654
O14979-2	1.134	0.18142064	0.9099386
Q99496	1.134	0.18142064	0.9099386
Q6ZN50	0.86	-0.217591435	0.91017909
H3BUN4	0.871	-0.199255376	0.91017909
Q9Y5U9	0.875	-0.192645078	0.91017909
Q9BVG9	0.877	-0.189351252	0.91017909
P78527	0.904	-0.145605322	0.91017909
Q13045	0.904	-0.145605322	0.91017909
P21281	0.904	-0.145605322	0.91017909
P49189	0.907	-0.140825544	0.91017909
Q9UHR4	0.921	-0.118726939	0.91017909
O60506	1.084	0.116364757	0.91017909
P09012	1.087	0.12035194	0.91017909
P61163	1.088	0.121678557	0.91017909
O60333	1.088	0.121678557	0.91017909
Q9Y316	1.094	0.129612738	0.91017909
E9PII7	1.098	0.134878054	0.91017909
P49023	1.113	0.154453593	0.91017909

Q14997	1.139	0.187767747	0.91017909
P32321	1.139	0.187767747	0.91017909
Q9Y5P4	1.154	0.206643224	0.91017909
Q9NP77	1.166	0.221567789	0.91017909
P38571	1.202	0.265436896	0.91017909
Q9BYV8	1.237	0.3068455	0.91017909
P07900	1.083	0.115033243	0.91024973
Q9NVP2	1.165	0.220329955	0.91049211
F5H757	1.166	0.221567789	0.91049211
P00367	1.082	0.113700499	0.91170888
Q00577	0.873	-0.195946441	0.91205319
Q9Y5Y2	1.135	0.182692298	0.91266173
Q969S3	1.097	0.133563526	0.91267387
P26885	0.894	-0.161653263	0.91284103
P61956	0.906	-0.142417045	0.91284103
P43003	0.857	-0.222632891	0.91290952
Q8NEN9	0.814	-0.2968993	0.9144914
P05496	0.851	-0.232768963	0.9144914
O95396	0.853	-0.229382353	0.9144914
Q9UHN1	0.853	-0.229382353	0.9144914
Q9H173	0.866	-0.20756107	0.9144914
Q9H9A5	0.876	-0.190997225	0.9144914
P08648	0.878	-0.187707155	0.9144914
Q9UNH7	0.892	-0.164884385	0.9144914
O60271	0.897	-0.15682011	0.9144914
O14976	0.901	-0.150400989	0.9144914
Q9H444	0.903	-0.147202107	0.9144914
P61981	0.905	-0.144010303	0.9144914
P62269	0.906	-0.142417045	0.9144914
P62913	0.906	-0.142417045	0.9144914
J3KMY7	0.907	-0.140825544	0.9144914
Q6RW13	0.917	-0.125006361	0.9144914
Q9UKD2	1.076	0.105678078	0.9144914
Q99733	1.081	0.112366523	0.9144914
P28331	1.087	0.12035194	0.9144914
Q9UJS0	1.094	0.129612738	0.9144914
Q9NX62	1.157	0.210388864	0.9144914
Q9NRY4	1.19	0.250961574	0.9144914
O94916	1.214	0.279768422	0.9144914
P60866	0.906	-0.142417045	0.91454399
Q9BUJ2	1.075	0.10433666	0.91468038
Q12906	1.08	0.111031312	0.91475536
O95487	1.099	0.136191386	0.91475536
Q9H9Y2	1.129	0.175045486	0.91475536
I3L2J8	1.152	0.204140717	0.91475536
Q9NY93	0.892	-0.164884385	0.91496381

B4DKT0	1.095	0.13093087	0.91496381
Q8IYB8	1.133	0.180147861	0.91496381
O43156	1.131	0.177598929	0.91508272
P01892	0.892	-0.164884385	0.91591483
Q9BVI4	0.892	-0.164884385	0.91596834
P40925	0.907	-0.140825544	0.91596834
Q07352	0.872	-0.19759996	0.91642248
Q93034	0.896	-0.158429363	0.91642248
O94906	0.91	-0.13606155	0.91670835
Q9P016	1.119	0.162210036	0.91670835
Q15527	1.186	0.24610401	0.91670835
P63220	0.908	-0.139235797	0.91682327
Q8IWX8	1.089	0.123003954	0.91688442
O95155	1.151	0.202887833	0.91695362
P13010	0.907	-0.140825544	0.91720739
P35754	1.113	0.154453593	0.91729818
Q9UHD8	0.908	-0.139235797	0.91774829
Q8WYQ5	1.185	0.244887059	0.91774829
Q9Y5K6	1.094	0.129612738	0.91827269
O95758-1	1.155	0.207892852	0.91836675
Q9UPN3-5	1.189	0.249748715	0.91836675
Q14746	0.922	-0.117161344	0.91884152
E7EN44	0.821	-0.284545873	0.9190596
Q08378	0.902	-0.148800661	0.9190596
P37108	0.913	-0.131313235	0.91909735
Q12849	1.083	0.115033243	0.91909735
Q6NUS6	0.839	-0.253257284	0.91912652
Q6IN85	1.112	0.153156788	0.9191456
Q9NWZ5	0.858	-0.220950447	0.91924769
P55081	0.912	-0.13289427	0.91924769
Q9P0I2	0.831	-0.267079618	0.91933389
Q86U42	1.078	0.108357178	0.91956791
Q9NQW6	0.892	-0.164884385	0.92004409
Q8WVC6	0.871	-0.199255376	0.92042189
Q9BTU6	0.882	-0.181149439	0.92042189
O95453	0.897	-0.15682011	0.92042189
Q6L8Q7	0.911	-0.134477041	0.92042189
O60684	0.918	-0.123433941	0.92042189
Q01518	1.083	0.115033243	0.92042189
O43172	1.086	0.119024103	0.92042189
P04179	1.088	0.121678557	0.92042189
B7ZC38	1.149	0.200378798	0.92042189
J3KNN7	1.152	0.204140717	0.92042189
Q92805	1.193	0.254594043	0.92042189
E9PHX5	1.2	0.263034406	0.92042189
Q9Y3C8	0.884	-0.177881725	0.92044321

Q9BZE1	0.888	-0.171368418	0.92044321
P43897	0.894	-0.161653263	0.92044321
Q7Z7H8	0.896	-0.158429363	0.92044321
F5H0L8	0.919	-0.121863233	0.92044321
O60701	1.067	0.093560176	0.92044321
O76031	1.075	0.10433666	0.92044321
P20290	1.076	0.105678078	0.92044321
Q15717	1.078	0.108357178	0.92044321
G3XAD6	1.107	0.146655222	0.92044321
Q9NX08	1.14	0.189033824	0.92044321
O14656	1.153	0.205392513	0.92044321
P51788	1.219	0.285698126	0.92044321
O94905	0.91	-0.13606155	0.92079063
Q9UBP6	1.179	0.237563718	0.92118795
Q9BRT2	1.14	0.189033824	0.92139201
Q9H3P7	0.904	-0.145605322	0.92173486
A8MTH6	0.906	-0.142417045	0.92221693
Q6GYA4	0.843	-0.246395464	0.92232868
O00471	0.868	-0.204233052	0.92308593
A2A2G4	0.871	-0.199255376	0.92308593
Q03701	0.896	-0.158429363	0.92308593
P21333	0.909	-0.1376478	0.92308593
P28482	1.083	0.115033243	0.92308593
Q14320	1.097	0.133563526	0.92308593
Q6ZRS2	1.125	0.169925001	0.92308593
Q8N4N3	1.126	0.171206827	0.92308593
Q9BQ04	1.144	0.194087052	0.92308593
Q969T4	1.144	0.194087052	0.92308593
P55212	1.155	0.207892852	0.92308593
Q32N00	1.168	0.224040274	0.92308593
Q5PRF9	1.178	0.236339539	0.92308593
H0YIQ8	1.217	0.283329168	0.92308593
Q8WWY3	1.083	0.115033243	0.92310893
O00178	1.127	0.172487516	0.92327885
Q5TA45	0.878	-0.187707155	0.92356335
P22307	0.895	-0.160040413	0.92434367
Q12834	0.918	-0.123433941	0.92434367
Q15363	1.076	0.105678078	0.92434367
Q659C4	1.179	0.237563718	0.92434367
Q86SX6	0.916	-0.126580497	0.92439138
Q9H9Q4	0.866	-0.20756107	0.924403
O43852	0.91	-0.13606155	0.924403
E9PG40	0.918	-0.123433941	0.924403
Q7Z7F7	0.925	-0.112474729	0.924403
Q06830	1.076	0.105678078	0.924403
P53582	1.083	0.115033243	0.924403

Q7KZN9	1.105	0.14404637	0.924403
Q5JR08	1.123	0.167357928	0.924403
E9PS76	0.903	-0.147202107	0.9244118
Q9Y2Y0	1.132	0.178873958	0.9244118
J3KNC0	1.144	0.194087052	0.9244118
Q9P212	1.147	0.197865391	0.9244118
P15056	1.175	0.232660757	0.9244118
P63165	0.91	-0.13606155	0.92470112
Q9BYT8	0.918	-0.123433941	0.92477292
Q9Y2S7	0.891	-0.166502663	0.92479218
Q96KP4	0.911	-0.134477041	0.92480308
O76071	1.137	0.185232254	0.92480308
Q96Q11	1.128	0.173767068	0.92491483
Q8N556	0.828	-0.272297327	0.925135
F8W9X7	0.83	-0.268816758	0.925135
F5GXJ0	0.83	-0.268816758	0.925135
Q86YV9	0.863	-0.212567535	0.925135
Q5C9Z4	0.865	-0.209227962	0.925135
Q9BUA3	0.868	-0.204233052	0.925135
Q9H270	0.872	-0.19759996	0.925135
Q12965	0.881	-0.182786076	0.925135
Q8TCS8	0.897	-0.15682011	0.925135
Q13123	0.902	-0.148800661	0.925135
Q15758	0.902	-0.148800661	0.925135
Q9NQ88	0.905	-0.144010303	0.925135
O76021	0.911	-0.134477041	0.925135
P62081	0.912	-0.13289427	0.925135
Q9UHV9	0.912	-0.13289427	0.925135
P14406	0.913	-0.131313235	0.925135
Q9UPT5	0.919	-0.121863233	0.925135
O75607	0.92	-0.120294234	0.925135
Q9NP92	0.927	-0.109358756	0.925135
E7EN19	1.073	0.101650076	0.925135
P04406	1.074	0.102993993	0.925135
Q8TDD1	1.076	0.105678078	0.925135
Q969V3	1.078	0.108357178	0.925135
Q8TAT6	1.083	0.115033243	0.925135
E5RGS4	1.122	0.166072676	0.925135
Q8TED1	1.123	0.167357928	0.925135
Q8N7B9	1.127	0.172487516	0.925135
Q9Y6K5	1.133	0.180147861	0.925135
D6RED8	1.142	0.191562651	0.925135
Q9Y6X5	1.15	0.201633861	0.925135
P85299-5	1.15	0.201633861	0.925135
Q8ND04	1.169	0.22527493	0.925135
Q96ES7	1.175	0.232660757	0.925135

P17535	1.177	0.23511432	0.925135
G8JLM5	1.204	0.267835392	0.925135
O60264	1.063	0.088141597	0.92528434
Q3ZCQ8	1.081	0.112366523	0.92549872
Q9Y3D0	0.913	-0.131313235	0.9256239
Q9NS86	1.086	0.119024103	0.92567493
Q8NB28	0.874	-0.194294815	0.92599928
O43592	0.912	-0.13289427	0.92599928
P51148	0.915	-0.128156351	0.92599928
Q5VWJ9	1.175	0.232660757	0.92599928
Q9HCE5	1.208	0.272620455	0.92599928
C9JBI3	0.904	-0.145605322	0.92663575
O43504	0.917	-0.125006361	0.92663575
Q7L014	1.073	0.101650076	0.92663575
Q32P44	0.868	-0.204233052	0.9268842
Q8WW59	0.883	-0.179514657	0.9268842
C9JJV1	1.121	0.164786278	0.9268842
Q8TAQ2	1.201	0.264236151	0.9268842
P30048	0.913	-0.131313235	0.9269178
H7BYT1	0.922	-0.117161344	0.92699704
Q15691	1.058	0.081339627	0.92699704
H9KV45	1.07	0.097610797	0.92699704
O95429	1.195	0.257010618	0.92699704
Q9Y262	0.913	-0.131313235	0.92701543
P10176	0.834	-0.261880711	0.92718612
Q9Y490	1.072	0.100304906	0.92724916
E7ERK9	1.167	0.222804561	0.92724916
Q9Y678	0.913	-0.131313235	0.9274164
J3KNF8	1.072	0.100304906	0.9274164
Q5HYZ1	0.876	-0.190997225	0.92764749
Q9UBV8	1.142	0.191562651	0.92777106
Q7L7X3	0.882	-0.181149439	0.92777302
Q9H0P0	1.142	0.191562651	0.92777302
P30536	0.884	-0.177881725	0.92794736
P22695	0.914	-0.12973393	0.92794736
F8VSL3	1.163	0.217851097	0.92797366
Q01085-2	0.914	-0.12973393	0.92814885
P06756	0.852	-0.231074664	0.92853969
Q6PKC0	0.877	-0.189351252	0.92853969
Q9NZJ6	0.877	-0.189351252	0.92853969
Q8WUK0	0.881	-0.182786076	0.92853969
Q6P6B1	0.885	-0.17625064	0.92853969
Q9P2P6	0.888	-0.171368418	0.92853969
P30519	0.891	-0.166502663	0.92853969
Q9NTX5	0.899	-0.153606979	0.92853969
Q96EY1	0.9	-0.152003093	0.92853969

P11908	0.912	-0.13289427	0.92853969
P53621	0.914	-0.12973393	0.92853969
P50914	0.914	-0.12973393	0.92853969
Q9Y6M9	0.923	-0.115597447	0.92853969
Q86WQ0	0.933	-0.100051014	0.92853969
Q9P287	1.062	0.086783766	0.92853969
P16403	1.07	0.097610797	0.92853969
Q14684	1.071	0.09895848	0.92853969
P26368	1.071	0.09895848	0.92853969
B5MDF5	1.071	0.09895848	0.92853969
O95782	1.076	0.105678078	0.92853969
O75940	1.083	0.115033243	0.92853969
P24941	1.114	0.155749233	0.92853969
Q9NT62	1.116	0.158337027	0.92853969
J3QTA6	1.129	0.175045486	0.92853969
O75165	1.138	0.186500558	0.92853969
Q8N2K0	1.153	0.205392513	0.92853969
Q9NYL2-2	1.158	0.211635253	0.92853969
P49959	1.159	0.212880566	0.92853969
E7ERH3	1.159	0.212880566	0.92853969
Q9Y619	1.168	0.224040274	0.92853969
Q92636	1.184	0.243669081	0.92853969
Q8TEY7	1.204	0.267835392	0.92853969
O95251	0.872	-0.19759996	0.92856638
Q5T280	0.89	-0.168122759	0.92856638
E9PHS3	0.918	-0.123433941	0.92856638
P18615	1.119	0.162210036	0.92856638
Q9Y2S2	0.889	-0.169744676	0.92868601
P60983	1.079	0.109694865	0.92868601
Q9Y6X4	0.887	-0.17299399	0.92907359
Q9UKV3	1.076	0.105678078	0.92907359
Q9Y547	1.105	0.14404637	0.92916335
P51580	0.915	-0.128156351	0.92950267
B4DFQ4	0.856	-0.224317298	0.92951146
Q8TBA6	0.892	-0.164884385	0.92951146
Q9H0C8	0.902	-0.148800661	0.92951146
Q9NQX3	0.916	-0.126580497	0.92951146
K7ELG9	1.101	0.138814469	0.92951146
Q9UBB9	1.089	0.123003954	0.93015668
Q3SXM5	0.916	-0.126580497	0.93027762
F5GXQ1	0.838	-0.254977851	0.93045441
Q92664	0.841	-0.249822294	0.93045441
G8JL90	0.878	-0.187707155	0.93045441
Q6XZF7	0.898	-0.15521265	0.93045441
Q9Y3D3	0.902	-0.148800661	0.93045441
O75306	0.918	-0.123433941	0.93045441

Q92922	1.062	0.086783766	0.93045441
Q96QU8	1.137	0.185232254	0.93045441
A8MYT4	1.167	0.222804561	0.93045441
Q9Y285	0.912	-0.13289427	0.93054445
O95297	1.139	0.187767747	0.93054445
O15145	0.897	-0.15682011	0.93063039
P12081	0.916	-0.126580497	0.93063039
Q9Y271	0.856	-0.224317298	0.93090393
Q16670	0.825	-0.277533976	0.93093996
Q8WW01	0.88	-0.184424571	0.93093996
Q96JQ0	0.888	-0.171368418	0.93093996
Q14966	0.904	-0.145605322	0.93093996
P03891	0.907	-0.140825544	0.93093996
P60981	0.914	-0.12973393	0.93093996
Q8WZ42	0.914	-0.12973393	0.93093996
Q15007	0.916	-0.126580497	0.93093996
K7EQF4	1.069	0.096261853	0.93093996
Q9Y5B6	1.073	0.101650076	0.93093996
F5H7R9	1.075	0.10433666	0.93093996
P15104	1.115	0.15704371	0.93093996
O96033	1.16	0.214124805	0.93093996
Q4G0I0	1.169	0.22527493	0.93093996
P49459	0.904	-0.145605322	0.93125823
Q9BRJ2	0.91	-0.13606155	0.93125823
Q92882	1.068	0.094911647	0.93125823
O95376	0.935	-0.09696173	0.93136703
O75150	1.085	0.117695043	0.93161303
Q8NC60	0.88	-0.184424571	0.93210725
Q12929	1.194	0.255802837	0.93210725
Q8WVD3	0.857	-0.222632891	0.93214948
P30825	0.88	-0.184424571	0.93214948
Q8N335	0.881	-0.182786076	0.93214948
Q9UHR6	0.886	-0.174621396	0.93214948
Q9BZL1	0.896	-0.158429363	0.93214948
P61160	0.923	-0.115597447	0.93214948
P55884	1.067	0.093560176	0.93214948
O75351	1.068	0.094911647	0.93214948
P46108	1.077	0.10701825	0.93214948
Q6P3W7	1.105	0.14404637	0.93214948
Q9NPQ8	1.132	0.178873958	0.93214948
Q9Y5N6	1.139	0.187767747	0.93214948
Q6IS24	1.147	0.197865391	0.93214948
Q92979	1.073	0.101650076	0.9322079
P50613	0.898	-0.15521265	0.93241053
Q9NZW5	0.923	-0.115597447	0.93241053
Q16777	0.918	-0.123433941	0.93248322

Q14683	1.066	0.092207438	0.93248322
Q6P9B9	1.165	0.220329955	0.93248322
E9PDK6	0.862	-0.214240226	0.93287669
P01033	0.865	-0.209227962	0.93287669
Q96L34	0.872	-0.19759996	0.93287669
O00221	0.884	-0.177881725	0.93287669
Q86XL3	0.891	-0.166502663	0.93287669
P46736	0.891	-0.166502663	0.93287669
Q5TCM7	0.893	-0.16326792	0.93287669
Q9BVG4	0.906	-0.142417045	0.93287669
H0Y9X1	0.916	-0.126580497	0.93287669
O75251	0.917	-0.125006361	0.93287669
Q06323	0.918	-0.123433941	0.93287669
E9PQY2	0.918	-0.123433941	0.93287669
Q9H0W9	0.921	-0.118726939	0.93287669
Q9BRR6	0.936	-0.095419565	0.93287669
P07954	1.056	0.078609835	0.93287669
Q9Y2T2	1.061	0.085424656	0.93287669
Q14974	1.066	0.092207438	0.93287669
Q13148	1.07	0.097610797	0.93287669
Q9HCN4	1.078	0.108357178	0.93287669
Q9BUN8	1.087	0.12035194	0.93287669
P19838	1.105	0.14404637	0.93287669
E9PEN8	1.107	0.146655222	0.93287669
Q9BXV9	1.118	0.160920188	0.93287669
Q9ULC3	1.134	0.18142064	0.93287669
Q8TDJ6	1.143	0.192825404	0.93287669
Q8NBN3	1.154	0.206643224	0.93287669
Q6DKI1	0.915	-0.128156351	0.93288683
Q5QPM7	0.912	-0.13289427	0.93295852
Q8WWC4	0.927	-0.109358756	0.93295852
Q16763	1.068	0.094911647	0.93295852
P51608	1.133	0.180147861	0.93295852
Q6P1N0	1.162	0.216610069	0.93295852
P63096	0.873	-0.195946441	0.93308747
F5GZ06	0.882	-0.181149439	0.93308747
G3V5Q3	0.923	-0.115597447	0.93344325
Q9BZZ5-2	1.066	0.092207438	0.93345868
O60783	0.892	-0.164884385	0.93358428
Q9BRP1	0.94	-0.089267338	0.93358428
Q15008	1.062	0.086783766	0.93358428
O00139-2	0.922	-0.117161344	0.9337781
Q53S33	1.143	0.192825404	0.93401071
Q96CM3	0.875	-0.192645078	0.93405155
G5E9C2	0.878	-0.187707155	0.93405155
Q8TBB5	0.884	-0.177881725	0.93405155

Q13951	0.885	-0.17625064	0.93405155
O75880	0.889	-0.169744676	0.93405155
Q15345	0.892	-0.164884385	0.93405155
Q02218	0.906	-0.142417045	0.93405155
Q9H299	0.907	-0.140825544	0.93405155
O95757	0.919	-0.121863233	0.93405155
P52597	0.92	-0.120294234	0.93405155
P36578	0.92	-0.120294234	0.93405155
P48444	0.92	-0.120294234	0.93405155
Q04917	0.92	-0.120294234	0.93405155
H7C2W9	0.92	-0.120294234	0.93405155
P42785	0.922	-0.117161344	0.93405155
Q9BRS2	0.928	-0.10780329	0.93405155
P41208	0.932	-0.10159814	0.93405155
P24539	1.059	0.082702589	0.93405155
P54578	1.061	0.085424656	0.93405155
O43399	1.061	0.085424656	0.93405155
Q92538	1.064	0.089498151	0.93405155
P14618	1.065	0.09085343	0.93405155
P62314	1.065	0.09085343	0.93405155
Q9BST9	1.122	0.166072676	0.93405155
Q9Y5A7	1.126	0.171206827	0.93405155
P45983	1.126	0.171206827	0.93405155
Q9NRK6	1.126	0.171206827	0.93405155
Q9H269	1.127	0.172487516	0.93405155
E7EQ95	1.14	0.189033824	0.93405155
E7ENY0	1.142	0.191562651	0.93405155
Q8IWJ2	1.142	0.191562651	0.93405155
E9PGG2	1.143	0.192825404	0.93405155
Q86UX6	1.145	0.195347598	0.93405155
Q66K14	1.163	0.217851097	0.93405155
Q9H6R7	1.184	0.243669081	0.93405155
H3BRM1	1.187	0.247319935	0.93405155
Q15814	0.89	-0.168122759	0.93410643
Q8IZL8	1.065	0.09085343	0.93410643
P24390	0.915	-0.128156351	0.93428365
O14974	0.912	-0.13289427	0.93469059
F6S0T5	1.068	0.094911647	0.93469059
Q9H0G5	1.139	0.187767747	0.93469059
O60476	0.892	-0.164884385	0.93487572
O60573	1.11	0.150559677	0.93487572
P14635	0.921	-0.118726939	0.93512438
Q9H974	0.938	-0.092340172	0.93512438
E9PLN8	0.897	-0.15682011	0.93564712
Q14CX7	0.917	-0.125006361	0.93564712
P06733	0.921	-0.118726939	0.93564712

J3QLM1	0.898	-0.15521265	0.93580733
O95248	0.835	-0.260151897	0.93601362
O00273	1.066	0.092207438	0.93601362
Q8N4V1	1.111	0.151858817	0.93601362
Q9P2N5	1.152	0.204140717	0.9361521
E9PBC1	0.867	-0.205896101	0.93624656
Q96SQ9	0.888	-0.171368418	0.93624656
Q13347	0.917	-0.125006361	0.93624656
P46781	0.921	-0.118726939	0.93624656
A6NMQ7	0.932	-0.10159814	0.93624656
Q96D71	0.925	-0.112474729	0.93628043
P15586	0.934	-0.098505545	0.93628043
Q9NXV6	0.899	-0.153606979	0.93668703
P62263	0.922	-0.117161344	0.93686034
E9PH88	0.888	-0.171368418	0.93689106
A6NIH7	0.905	-0.144010303	0.93689106
Q9P0J0	0.909	-0.1376478	0.93689106
O75521	0.915	-0.128156351	0.93689106
O14745	0.921	-0.118726939	0.93689106
Q9HCD5	0.921	-0.118726939	0.93689106
Q5T6W5	1.062	0.086783766	0.93689106
Q96P11	1.062	0.086783766	0.93689106
E9PAU2	1.063	0.088141597	0.93689106
P46977	1.071	0.09895848	0.93689106
P49642	1.094	0.129612738	0.93689106
Q86X02	0.867	-0.205896101	0.93693621
P57076	1.138	0.186500558	0.93694574
Q15072	0.851	-0.232768963	0.93699216
Q13162	0.922	-0.117161344	0.93706439
P62995	1.064	0.089498151	0.93712872
Q92615	0.926	-0.110915901	0.9373297
Q5VTL8	0.902	-0.148800661	0.93735475
P17858	1.066	0.092207438	0.93735475
Q9Y333	1.06	0.084064265	0.93748957
Q6RFH5	0.91	-0.13606155	0.93756891
B1AK53	0.884	-0.177881725	0.93764672
Q15637-5	0.887	-0.17299399	0.93775698
B7Z7F1	1.147	0.197865391	0.93801051
Q12846	0.901	-0.150400989	0.93850842
B4DNM9	1.126	0.171206827	0.93850842
Q9NVN8	0.88	-0.184424571	0.93854148
Q9UNX3	0.922	-0.117161344	0.93854148
P00167	0.895	-0.160040413	0.9386
O94915	1.124	0.168642036	0.93865935
F5GWY5	0.879	-0.18606493	0.9392421
Q9UPT8	0.849	-0.236163541	0.93938115

H0Y450	0.868	-0.204233052	0.93944559
O75348	0.921	-0.118726939	0.93944559
Q6EEV4	1.141	0.190298792	0.93944559
Q9H8E8	0.838	-0.254977851	0.93944996
Q8WYP5	0.923	-0.115597447	0.93944996
P27708	1.061	0.085424656	0.93944996
O75475	1.064	0.089498151	0.93948337
Q9Y6N1	1.141	0.190298792	0.93948337
Q14767	1.156	0.209141398	0.93948337
Q9BUB7	1.08	0.111031312	0.93965776
B5MCF9	0.924	-0.114035243	0.93966735
O43674	0.925	-0.112474729	0.93966735
O60518	0.868	-0.204233052	0.93996447
Q13451	0.927	-0.109358756	0.94008018
P48047	0.923	-0.115597447	0.9403159
Q8N5G0	1.12	0.163498732	0.94043148
C9J8E1	1.124	0.168642036	0.94060283
Q01658	1.105	0.14404637	0.94065265
Q9Y265	0.923	-0.115597447	0.94068115
A6NDA1	0.891	-0.166502663	0.94087992
P47897	0.924	-0.114035243	0.94087992
Q9UPN9-2	1.171	0.227741076	0.94130475
Q3B7T1	0.906	-0.142417045	0.94140072
Q96EU6	0.903	-0.147202107	0.94151373
Q8NBL1	0.904	-0.145605322	0.94173254
Q9NPF4	0.905	-0.144010303	0.94173254
Q15631	1.068	0.094911647	0.94173254
J3QRV5	1.108	0.147957881	0.94173254
O60678	1.128	0.173767068	0.94173254
Q92878	0.896	-0.158429363	0.94177802
H0Y8C3	0.9	-0.152003093	0.94177802
Q8TC07-2	1.071	0.09895848	0.94177802
O15213	0.901	-0.150400989	0.9421709
P50895	1.103	0.141432791	0.9421709
P52788	1.065	0.09085343	0.94302581
Q5TDF0	1.118	0.160920188	0.94302581
Q9UJ72	1.119	0.162210036	0.94322189
Q92905	1.058	0.081339627	0.9432516
Q9NRX1	0.915	-0.128156351	0.94339203
P62979	0.924	-0.114035243	0.9440001
Q9GZZ9	0.923	-0.115597447	0.94431713
P20339	0.937	-0.093879047	0.94431713
Q9BYC9	1.104	0.142740172	0.94431713
P43378	1.171	0.227741076	0.94431713
D3DS54	1.117	0.159629186	0.94460335
Q08752	1.049	0.069014678	0.94470057

Q8N0T1	0.857	-0.222632891	0.94501344
Q9Y3A3	1.101	0.138814469	0.94501344
Q99633	1.172	0.22897257	0.94501344
O43488	1.06	0.084064265	0.94502462
P09496-2	1.06	0.084064265	0.94513825
P40938	1.069	0.096261853	0.94514284
Q9BW91	1.1	0.137503524	0.94514284
Q14677-3	1.048	0.067638717	0.94515422
Q5W111	0.889	-0.169744676	0.94536358
P07951-2	0.926	-0.110915901	0.94554731
Q9UKN8	1.047	0.066261442	0.9459041
Q9BSJ8	0.929	-0.106249498	0.94602388
Q9NUG6	0.874	-0.194294815	0.94628447
P25098	0.887	-0.17299399	0.94628447
Q16706	0.893	-0.16326792	0.94628447
F8WAJ0	0.894	-0.161653263	0.94628447
Q9BU76	1.141	0.190298792	0.94628447
Q8IXQ4	1.143	0.192825404	0.94628447
B4E0K5	1.105	0.14404637	0.94637721
Q96EV2	1.116	0.158337027	0.94637721
O60504	1.12	0.163498732	0.94637721
Q9Y5T5	0.892	-0.164884385	0.94725605
Q9NRP2	0.903	-0.147202107	0.94725605
Q14807	0.92	-0.120294234	0.94725605
Q13596	0.926	-0.110915901	0.94725605
Q92747	0.927	-0.109358756	0.94725605
P07858	1.062	0.086783766	0.94725605
P52701	1.057	0.079975377	0.94808916
P52565	1.057	0.079975377	0.94810242
Q04760	0.926	-0.110915901	0.94853991
P25398	1.057	0.079975377	0.94857655
Q9Y5X3	1.066	0.092207438	0.94868361
Q9P270	1.112	0.153156788	0.94868361
Q92604	0.86	-0.217591435	0.94884668
Q8WYA6	0.921	-0.118726939	0.94884668
P30154	0.932	-0.10159814	0.94884668
Q96FJ2	0.932	-0.10159814	0.94884668
P18206	1.057	0.079975377	0.94884668
Q8IUF8	1.06	0.084064265	0.94884668
O75477	1.061	0.085424656	0.94884668
Q96AC1	1.067	0.093560176	0.94884668
Q9BV38	1.068	0.094911647	0.94884668
Q9Y6Q5	1.095	0.13093087	0.94884668
Q7Z4S6	1.097	0.133563526	0.94884668
Q8WUA4	1.099	0.136191386	0.94884668
Q99570	1.102	0.140124224	0.94884668

A6ND99	1.109	0.149259365	0.94884668
Q5HYK3	1.123	0.167357928	0.94884668
Q13868	1.066	0.092207438	0.94908438
Q9UH65	0.894	-0.161653263	0.94911933
Q09028	1.056	0.078609835	0.94975588
G3V4A5	0.88	-0.184424571	0.95012323
Q8WZ82	0.901	-0.150400989	0.95012323
O60353	0.907	-0.140825544	0.95012323
Q05193	0.942	-0.086201035	0.95012323
Q96P70	1.054	0.075874867	0.95012323
Q9UIG0	1.059	0.082702589	0.95012323
Q6P1N9	1.088	0.121678557	0.95012323
Q9NUL3	1.099	0.136191386	0.95012323
Q8WUX9	1.103	0.141432791	0.95012323
Q9BVS4	1.106	0.145351386	0.95012323
Q86W50	1.115	0.15704371	0.95012323
P53367-2	1.124	0.168642036	0.95012323
Q9NZQ3	1.136	0.183962835	0.95012323
E7EX15	0.849	-0.236163541	0.95025295
A6NG32	0.877	-0.189351252	0.95025295
Q15853	0.896	-0.158429363	0.95025295
I3L3E4	0.906	-0.142417045	0.95025295
P13984	0.914	-0.12973393	0.95025295
Q9Y2U8	0.949	-0.075520008	0.95025295
Q96MG7	0.897	-0.15682011	0.95041039
Q9GZT9	1.124	0.168642036	0.95052684
P18031	0.932	-0.10159814	0.95080264
Q9UQB8	1.057	0.079975377	0.95080264
Q9Y266	1.055	0.077242999	0.95112147
Q9BW72	1.115	0.15704371	0.95144122
B7Z4R0	1.128	0.173767068	0.95144122
Q9NTG7	1.135	0.182692298	0.95144185
Q9P021	0.86	-0.217591435	0.95217871
Q99747	0.921	-0.118726939	0.95217871
Q9UL46	0.929	-0.106249498	0.95217871
Q9NPD3	1.061	0.085424656	0.95217871
Q0VDF9	1.091	0.125651102	0.95217871
Q8NBU5	1.096	0.132247798	0.95217871
Q9UJY4	1.099	0.136191386	0.95217871
Q96I25	1.106	0.145351386	0.95217871
Q9NRL3	1.107	0.146655222	0.95217871
Q96GK7	1.113	0.154453593	0.95217871
O00193	0.929	-0.106249498	0.95268477
Q16595	1.119	0.162210036	0.95268477
Q9BQ67	1.05	0.070389328	0.95314599
H7C4P4	1.092	0.126972856	0.95314599

Q9HBE1	0.865	-0.209227962	0.95316038
Q9UPQ9	0.869	-0.202571918	0.95316038
P03897	0.896	-0.158429363	0.95316038
P08581	0.9	-0.152003093	0.95316038
E9PCP3	0.902	-0.148800661	0.95316038
O43292	0.903	-0.147202107	0.95316038
O00115	0.903	-0.147202107	0.95316038
P00488	0.903	-0.147202107	0.95316038
P50402	0.925	-0.112474729	0.95316038
O75821	0.927	-0.109358756	0.95316038
Q96RQ3	0.928	-0.10780329	0.95316038
Q7KZF4	0.929	-0.106249498	0.95316038
Q9UHI6	0.93	-0.104697379	0.95316038
O75947	0.931	-0.103146927	0.95316038
Q9NXF1	0.931	-0.103146927	0.95316038
P16435	0.933	-0.100051014	0.95316038
Q96F86	0.938	-0.092340172	0.95316038
O94907	0.939	-0.090802937	0.95316038
Q8IV08	0.94	-0.089267338	0.95316038
Q9H5Q4	0.94	-0.089267338	0.95316038
Q9BYD6	1.051	0.071762669	0.95316038
B7Z7P8	1.054	0.075874867	0.95316038
J3KMX9	1.056	0.078609835	0.95316038
J3KPM8	1.056	0.078609835	0.95316038
Q9H5V8	1.056	0.078609835	0.95316038
Q9HCN8	1.062	0.086783766	0.95316038
O60568	1.086	0.119024103	0.95316038
Q5W0V3	1.091	0.125651102	0.95316038
P51153	1.092	0.126972856	0.95316038
Q8IY17	1.093	0.128293401	0.95316038
B4DMU4	1.127	0.172487516	0.95316038
Q96GE9	1.133	0.180147861	0.95316038
B4DVJ1	1.15	0.201633861	0.95316038
Q5TDC5	1.15	0.201633861	0.95316038
Q9ULJ7	1.148	0.199122642	0.95340489
Q96N67	1.055	0.077242999	0.9537091
Q9BRP4	1.072	0.100304906	0.9537091
Q9H9J2	0.917	-0.125006361	0.95415618
P51858	1.053	0.074505436	0.95415618
Q9NU22	1.057	0.079975377	0.95415618
E9PQJ4	0.884	-0.177881725	0.95417046
P00395	0.935	-0.09696173	0.95417046
Q8IX04	1.132	0.178873958	0.95417046
P19021	1.136	0.183962835	0.95417046
Q05397	0.939	-0.090802937	0.95442573
Q15418	1.114	0.155749233	0.95442573

O15050	0.912	-0.13289427	0.95446082
P14927	0.917	-0.125006361	0.95446082
Q9Y3T9	0.922	-0.117161344	0.95446082
Q9Y487	0.927	-0.109358756	0.95446082
J3KMZ8	0.934	-0.098505545	0.95446082
Q7L2J0	0.942	-0.086201035	0.95446082
P43243	1.052	0.073134705	0.95446082
P51991	1.052	0.073134705	0.95446082
Q8NI27	1.054	0.075874867	0.95446082
P62310	1.055	0.077242999	0.95446082
Q9H7D7	1.098	0.134878054	0.95446082
Q9Y679	1.104	0.142740172	0.95446082
Q0PNE2	1.128	0.173767068	0.95446082
O43865	1.049	0.069014678	0.95475169
Q99828	0.905	-0.144010303	0.95503221
Q8WVV9	0.924	-0.114035243	0.95503221
P84095	0.929	-0.106249498	0.95503221
P16615	1.051	0.071762669	0.95503221
Q86WX3	1.137	0.185232254	0.95503221
P20618	1.051	0.071762669	0.95508501
P60891	0.919	-0.121863233	0.95537684
P08574	0.93	-0.104697379	0.95553165
O75347	0.932	-0.10159814	0.95553165
Q13206	1.053	0.074505436	0.95553165
Q16540	1.092	0.126972856	0.95553165
J3KNH7	1.096	0.132247798	0.95553165
Q9Y3A2	0.953	-0.069451881	0.95578515
Q16774	0.897	-0.15682011	0.9559668
G3V0I6	0.918	-0.123433941	0.9559668
P36954	0.945	-0.081613766	0.9559668
O43242	1.051	0.071762669	0.9559668
Q9H6R0	1.104	0.142740172	0.9559668
Q8WZA0	1.109	0.149259365	0.9559668
Q15797	0.914	-0.12973393	0.95637335
Q9P2I0	0.923	-0.115597447	0.95637335
P25705	1.051	0.071762669	0.95637335
B9ZVT1	1.102	0.140124224	0.95637335
Q9P2B4	0.874	-0.194294815	0.95652409
Q9UIS9	0.88	-0.184424571	0.95652409
A2A2L5	0.913	-0.131313235	0.95652409
D6RCB9	0.931	-0.103146927	0.95652409
P31948	0.932	-0.10159814	0.95652409
Q07021	0.932	-0.10159814	0.95652409
Q96KR1	0.932	-0.10159814	0.95652409
P39019	1.05	0.070389328	0.95652409
P61011	1.054	0.075874867	0.95652409

Q9Y2S6	1.069	0.096261853	0.95652409
Q13425	1.11	0.150559677	0.95652409
K7ENG3	1.112	0.153156788	0.95652409
B1AM43	1.151	0.202887833	0.95652409
Q9HB07	1.051	0.071762669	0.95671449
J3KPU4	0.913	-0.131313235	0.95730116
P54920	1.089	0.123003954	0.95730116
Q9BYD1	0.925	-0.112474729	0.95776211
Q9NUP9	1.119	0.162210036	0.95776211
Q6ZXV5	0.897	-0.15682011	0.95777957
P06280	0.907	-0.140825544	0.95777957
Q9Y221	0.92	-0.120294234	0.95777957
P17096-2	0.921	-0.118726939	0.95777957
Q13895	0.936	-0.095419565	0.95785928
Q9BUR4	1.105	0.14404637	0.95813606
J3QRU1	0.933	-0.100051014	0.95825021
Q13151	1.041	0.057970069	0.95825021
Q14653	0.912	-0.13289427	0.9585652
Q9BWH6	0.915	-0.128156351	0.9585652
Q8TCJ2	0.933	-0.100051014	0.9585652
P48739	0.934	-0.098505545	0.9585652
Q9BTV6	0.87	-0.200912694	0.95965576
Q15648	0.91	-0.13606155	0.95965576
Q9H4A4	0.92	-0.120294234	0.95965576
Q96CS3	0.927	-0.109358756	0.95965576
Q9NRX2	0.933	-0.100051014	0.95965576
P00387	0.941	-0.087733372	0.95965576
Q9H2M9	0.941	-0.087733372	0.95965576
Q9ULK4	0.942	-0.086201035	0.95965576
Q9H2C0	0.956	-0.064917477	0.95965576
Q8NC51	0.964	-0.052894948	0.95965576
P39687	1.049	0.069014678	0.95965576
P63173	1.049	0.069014678	0.95965576
H0Y714	1.095	0.13093087	0.95965576
E9PSI1	1.098	0.134878054	0.95965576
Q8IX01	1.102	0.140124224	0.95965576
O76095	1.123	0.167357928	0.95965576
C9J2Y9	0.934	-0.098505545	0.95969927
Q8IYQ7	0.934	-0.098505545	0.95969927
Q14669-2	1.051	0.071762669	0.95969927
P30085	1.059	0.082702589	0.95969927
Q5F1R6	1.136	0.183962835	0.95969927
O75312	1.065	0.09085343	0.95986508
P42677	0.934	-0.098505545	0.96009878
P60953	0.943	-0.084670324	0.96009878
P35250	1.049	0.069014678	0.96043199

Q9Y676	0.928	-0.10780329	0.96115753
Q8N2F6	1.085	0.117695043	0.96120739
E7EWV1	0.915	-0.128156351	0.96165262
O15226	1.088	0.121678557	0.96176593
Q8TEA1	1.14	0.189033824	0.96207845
P30419	1.055	0.077242999	0.9623796
Q86X55	0.928	-0.10780329	0.96271489
H0YMY5	1.096	0.132247798	0.96288208
O60739	0.909	-0.1376478	0.96292066
J3QSH4	0.913	-0.131313235	0.96292066
O95391	1.108	0.147957881	0.96292066
Q96JP5	0.906	-0.142417045	0.96302421
I3L2C7	0.93	-0.104697379	0.9630409
O95707	1.102	0.140124224	0.9630409
B5MDL5	0.905	-0.144010303	0.96305766
Q96CQ1	0.904	-0.145605322	0.96316306
P45880	0.935	-0.09696173	0.96316306
Q9Y2L1	0.943	-0.084670324	0.96316306
P49590	1.058	0.081339627	0.96316306
Q9UJW0	1.052	0.073134705	0.96342057
P27694	1.034	0.048236186	0.96347129
O43617	0.948	-0.077041036	0.96349546
Q92841	0.935	-0.09696173	0.96362076
P03928	1.081	0.112366523	0.96362853
O75396	0.931	-0.103146927	0.96409108
P05198	0.935	-0.09696173	0.96426533
Q9UN37	1.085	0.117695043	0.96436867
H7BXS8	0.915	-0.128156351	0.96464397
P10636-5	0.932	-0.10159814	0.96464397
Q5T091	0.933	-0.100051014	0.96464397
P49327	1.046	0.064882852	0.96464397
Q14353	1.051	0.071762669	0.96464397
Q86VR2	1.077	0.10701825	0.96464397
Q5QP56	1.097	0.133563526	0.96464397
F8WBK2	0.877	-0.189351252	0.96477873
P61758	0.936	-0.095419565	0.96477873
Q8NBQ5	0.929	-0.106249498	0.96503292
Q9H6R4	0.94	-0.089267338	0.96503292
P56385	1.044	0.062121712	0.96503292
Q9BYG3	1.049	0.069014678	0.96503292
O95295	0.901	-0.150400989	0.96524513
P49427	0.922	-0.117161344	0.96524513
Q9NUQ9	1.04	0.056583528	0.96524513
P10412	1.046	0.064882852	0.96524513
Q9UKV5	1.106	0.145351386	0.96532528
E9PH69	0.913	-0.131313235	0.96571424

E9PQP6	1.079	0.109694865	0.96571424
Q96RQ1	1.089	0.123003954	0.96571424
O15479	1.037	0.052415894	0.96573232
H0YDR3	0.922	-0.117161344	0.96575598
Q13242	0.942	-0.086201035	0.96575598
Q15785	1.054	0.075874867	0.96575598
Q13542	0.914	-0.12973393	0.96658191
B4DFK6	0.919	-0.121863233	0.96658191
Q13111	1.097	0.133563526	0.96658191
B1ANE3	0.88	-0.184424571	0.96672189
C6G496	0.882	-0.181149439	0.96672189
Q5VX15	0.895	-0.160040413	0.96672189
Q9C0D9	0.912	-0.13289427	0.96672189
B7Z9I3	0.913	-0.131313235	0.96672189
Q9UKJ3	0.918	-0.123433941	0.96672189
J3QQW2	0.919	-0.121863233	0.96672189
Q15800	0.923	-0.115597447	0.96672189
P51398	0.924	-0.114035243	0.96672189
B4DT77	0.93	-0.104697379	0.96672189
Q96EL3	0.93	-0.104697379	0.96672189
P61353	0.937	-0.093879047	0.96672189
Q06203	0.941	-0.087733372	0.96672189
E7EMP6	0.946	-0.080087911	0.96672189
P06576	1.045	0.063502942	0.96672189
Q7Z478	1.048	0.067638717	0.96672189
Q9UHY1	1.079	0.109694865	0.96672189
P67775	1.089	0.123003954	0.96672189
Q9Y6A4	1.09	0.124328135	0.96672189
Q86VM9	1.098	0.134878054	0.96672189
H3BUD2	1.099	0.136191386	0.96672189
Q7Z7H5	0.933	-0.100051014	0.96673565
O75794	1.091	0.125651102	0.96673565
Q8NBF6	0.908	-0.139235797	0.96677893
Q9Y666	0.917	-0.125006361	0.96677893
Q16880	0.92	-0.120294234	0.96677893
Q9Y324	0.92	-0.120294234	0.96677893
Q14696	0.927	-0.109358756	0.96677893
P62312	0.935	-0.09696173	0.96677893
P41227	0.947	-0.078563669	0.96677893
Q9BVQ7	0.949	-0.075520008	0.96677893
O43278	0.954	-0.067938829	0.96677893
Q9NYF8	1.04	0.056583528	0.96677893
Q9UQ80	1.044	0.062121712	0.96677893
Q96JH7	1.048	0.067638717	0.96677893
Q92797	1.049	0.069014678	0.96677893
Q2PZI1	1.093	0.128293401	0.96677893

P29084	0.961	-0.057391664	0.96701305
P15153	1.108	0.147957881	0.96701305
Q8TDH9	0.891	-0.166502663	0.9670227
Q15459	1.044	0.062121712	0.96717864
Q9UJK0	1.121	0.164786278	0.96717864
O60220	1.082	0.113700499	0.96723522
P24844	0.899	-0.153606979	0.96754451
P55789	0.911	-0.134477041	0.96758814
P46783	1.044	0.062121712	0.96762693
Q14149	0.915	-0.128156351	0.96765255
B7Z1P2	0.882	-0.181149439	0.96778842
Q16850	0.93	-0.104697379	0.96792502
Q96AY3	0.938	-0.092340172	0.96806216
Q70CQ2	0.888	-0.171368418	0.9681573
P62937	1.043	0.060739158	0.9681573
Q9Y3I0	1.044	0.062121712	0.9681573
O00515	1.073	0.101650076	0.9681573
Q92917	1.081	0.112366523	0.9681573
P41240	1.082	0.113700499	0.9681573
P17029	1.108	0.147957881	0.9681573
O94967	1.133	0.180147861	0.9681573
Q6NUQ4	1.112	0.153156788	0.96887359
O75208	0.924	-0.114035243	0.96899485
Q5VT66	1.099	0.136191386	0.96899485
Q13416	0.89	-0.168122759	0.96933704
P26373	0.939	-0.090802937	0.96942369
Q03405	1.084	0.116364757	0.96942369
Q96EB6	1.093	0.128293401	0.96942369
Q15276	1.099	0.136191386	0.96950639
Q9UMS0	1.072	0.100304906	0.97003052
Q13158	0.923	-0.115597447	0.97033518
B7Z888	1.082	0.113700499	0.97035711
Q9BW71	1.076	0.105678078	0.97054464
Q14118	1.083	0.115033243	0.97077878
P30837	0.945	-0.081613766	0.97089634
Q99595	0.924	-0.114035243	0.9710368
P62249	0.939	-0.090802937	0.9710368
O00483	1.047	0.066261442	0.9710368
P14649	0.913	-0.131313235	0.97117342
O60502	1.029	0.041242982	0.97131403
P34932	1.042	0.059355278	0.97131403
Q99442	1.094	0.129612738	0.97131403
Q14186	1.114	0.155749233	0.97131403
O00488	0.924	-0.114035243	0.97139129
O95104	1.043	0.060739158	0.97139129
Q9Y5K5	1.11	0.150559677	0.97214199

Q6ZSC3	0.928	-0.10780329	0.97214772
P40855	1.106	0.145351386	0.97277335
Q15907	1.042	0.059355278	0.97288487
Q9NW64	1.072	0.100304906	0.97288487
P53992	0.94	-0.089267338	0.97308547
Q96AJ9-1	1.076	0.105678078	0.97308547
Q96EH3	0.903	-0.147202107	0.97325407
Q9H7D0	0.905	-0.144010303	0.97325407
O75909	1.081	0.112366523	0.97325407
Q9Y2G5-1	1.123	0.167357928	0.97325407
P20338	1.078	0.108357178	0.97330911
A6NFI3	1.126	0.171206827	0.97330911
Q9NXX6	1.11	0.150559677	0.97368996
P49458	1.047	0.066261442	0.97373239
P51659	1.041	0.057970069	0.97373373
P50750	1.079	0.109694865	0.97373373
O43719	0.928	-0.10780329	0.97383304
Q15172	0.923	-0.115597447	0.97386162
Q96CW1	1.039	0.055195654	0.97386162
Q9Y584	0.918	-0.123433941	0.97392962
Q05DH4	0.912	-0.13289427	0.97431138
P50583	0.949	-0.075520008	0.97431138
P56556	0.951	-0.072482754	0.97431138
A1L0T0	1.045	0.063502942	0.97431138
Q6PJG6	1.059	0.082702589	0.97431138
E5RIH5	1.088	0.121678557	0.97431138
Q86TI2	0.958	-0.061902439	0.97437575
A1A4S6	0.932	-0.10159814	0.97448405
Q9Y3C1	0.929	-0.106249498	0.97451125
P30084	0.941	-0.087733372	0.97498778
B7Z9G5	0.943	-0.084670324	0.97498778
O43676	0.956	-0.064917477	0.97498778
Q8NFH4	1.039	0.055195654	0.97498778
Q02790	1.04	0.056583528	0.97498778
Q5LJA5	1.044	0.062121712	0.97498778
Q9NPJ6	1.078	0.108357178	0.97498778
P42575	1.084	0.116364757	0.97498778
Q96CP2	1.086	0.119024103	0.97498778
F8W914	1.089	0.123003954	0.97498778
Q15554	1.108	0.147957881	0.97498778
Q4U2R6	1.074	0.102993993	0.97523027
Q9UNY4	0.895	-0.160040413	0.97539236
P37198	1.047	0.066261442	0.97543961
Q08AF3	1.083	0.115033243	0.97543961
Q15149-4	1.105	0.14404637	0.97543961
P62136	0.942	-0.086201035	0.97557145

Q9BUH6	1.089	0.123003954	0.9757118
Q8IWC1	0.923	-0.115597447	0.975718
Q9H0B6	1.046	0.064882852	0.975718
Q96DB5	0.927	-0.109358756	0.97580722
P46087	0.942	-0.086201035	0.97591404
O76075	0.918	-0.123433941	0.97593759
Q8WY36	0.919	-0.121863233	0.97593759
E7ETB3	0.93	-0.104697379	0.97593759
Q13617	0.931	-0.103146927	0.97593759
P61923	0.931	-0.103146927	0.97593759
P60763	0.932	-0.10159814	0.97593759
Q92974	0.933	-0.100051014	0.97593759
P60709	0.942	-0.086201035	0.97593759
Q8IY81	0.943	-0.084670324	0.97593759
Q9NY61	0.943	-0.084670324	0.97593759
P41567	1.042	0.059355278	0.97593759
Q9BPX3	1.042	0.059355278	0.97593759
Q9Y5Q9	1.043	0.060739158	0.97593759
O15446	1.068	0.094911647	0.97593759
Q7Z2W4	1.069	0.096261853	0.97593759
O95983	1.078	0.108357178	0.97593759
P15529	1.088	0.121678557	0.97593759
B4DJ85	1.094	0.129612738	0.97593759
Q8WXI9	1.105	0.14404637	0.97594942
P03905	1.075	0.10433666	0.97603951
C9JWV9	0.929	-0.106249498	0.97627094
B4DLN1	0.939	-0.090802937	0.97627094
Q92620	0.941	-0.087733372	0.97627094
P00846	0.943	-0.084670324	0.97627094
P82909	0.97	-0.043943348	0.97627094
Q15393	1.039	0.055195654	0.97627094
Q0IIM8	1.114	0.155749233	0.97627094
H0YB09	1.12	0.163498732	0.97627094
Q9NWT8	1.122	0.166072676	0.97627094
Q9NX70	0.893	-0.16326792	0.97629953
Q8WVE0	0.908	-0.139235797	0.97629953
P00156	0.922	-0.117161344	0.97629953
Q9Y6G3	0.926	-0.110915901	0.97629953
G8JLB3	0.931	-0.103146927	0.97629953
Q9Y608	0.931	-0.103146927	0.97629953
Q6ZNE5	0.936	-0.095419565	0.97629953
P23919	0.937	-0.093879047	0.97629953
Q99627	0.939	-0.090802937	0.97629953
B2WTI3	0.94	-0.089267338	0.97629953
Q14978-2	0.941	-0.087733372	0.97629953
Q9Y5B9	0.943	-0.084670324	0.97629953

Q15121	1.066	0.092207438	0.97629953
E9PCR4	1.088	0.121678557	0.97629953
Q13136	1.092	0.126972856	0.97629953
Q9HC52	1.092	0.126972856	0.97629953
Q6DD88	1.103	0.141432791	0.97629953
Q9BTY2	1.086	0.119024103	0.97632188
Q9BSR8	1.093	0.128293401	0.97632188
Q92625	1.102	0.140124224	0.97632188
Q9BYD3	0.948	-0.077041036	0.97635855
Q9UKZ1	0.929	-0.106249498	0.97678958
Q7Z2K6	0.908	-0.139235797	0.97688179
Q9UFN0	0.952	-0.070966521	0.97788533
O75330	0.892	-0.164884385	0.97788667
P34913	0.923	-0.115597447	0.97788667
K7ELM1	0.923	-0.115597447	0.97788667
Q5HYI7	0.925	-0.112474729	0.97788667
O14936	0.929	-0.106249498	0.97788667
Q7LBR1	0.937	-0.093879047	0.97788667
P67870	0.942	-0.086201035	0.97788667
P63244	0.944	-0.083141235	0.97788667
Q8NBF2	0.944	-0.083141235	0.97788667
Q7Z6Z7	0.945	-0.081613766	0.97788667
P05023	0.945	-0.081613766	0.97788667
O60508	0.945	-0.081613766	0.97788667
P52306	0.946	-0.080087911	0.97788667
Q4VC31	0.947	-0.078563669	0.97788667
O43818	0.948	-0.077041036	0.97788667
Q02127	0.955	-0.066427362	0.97788667
Q9C0C2	0.97	-0.043943348	0.97788667
Q92783	0.972	-0.040971781	0.97788667
O43684	1.03	0.042644337	0.97788667
E9PLL6	1.035	0.049630768	0.97788667
Q15424	1.036	0.051024003	0.97788667
P12004	1.036	0.051024003	0.97788667
P25788	1.036	0.051024003	0.97788667
Q00325-2	1.037	0.052415894	0.97788667
Q9H0U4	1.037	0.052415894	0.97788667
G5E9Q6	1.037	0.052415894	0.97788667
Q9BY32	1.041	0.057970069	0.97788667
P36873	1.042	0.059355278	0.97788667
Q5SQP8	1.042	0.059355278	0.97788667
G3V3H3	1.043	0.060739158	0.97788667
Q9BV44	1.054	0.075874867	0.97788667
E7EVY0	1.058	0.081339627	0.97788667
O95168	1.064	0.089498151	0.97788667
H7C3C0	1.068	0.094911647	0.97788667

B4E2X3	1.069	0.096261853	0.97788667
P53365	1.07	0.097610797	0.97788667
O75818	1.072	0.100304906	0.97788667
Q8N4Q1	1.072	0.100304906	0.97788667
Q9UI10-2	1.074	0.102993993	0.97788667
J3QR07	1.076	0.105678078	0.97788667
P23497	1.077	0.10701825	0.97788667
A8MXV4	1.077	0.10701825	0.97788667
O43815	1.078	0.108357178	0.97788667
F8W9R9	1.087	0.12035194	0.97788667
O95905	1.094	0.129612738	0.97788667
Q6ZMI0	1.097	0.133563526	0.97788667
P40189	1.105	0.14404637	0.97788667
P49356	1.107	0.146655222	0.97788667
Q8IVM0	1.107	0.146655222	0.97788667
Q5TBB1	1.117	0.159629186	0.97788667
Q8IZD4	1.095	0.13093087	0.97837928
Q8WVM8	0.933	-0.100051014	0.97853368
O14818	1.035	0.049630768	0.97862629
C9JKX0	0.905	-0.144010303	0.97867966
P00558	0.946	-0.080087911	0.97867966
Q15334	1.094	0.129612738	0.97867966
Q7Z2E3	1.096	0.132247798	0.97867966
Q9BYT3	1.096	0.132247798	0.97867966
Q6PCB5	0.903	-0.147202107	0.97888096
F5GXC8	0.972	-0.040971781	0.97888096
Q9UN86-2	1.04	0.056583528	0.97888096
Q99567	1.071	0.09895848	0.97888096
Q8N9T8	1.078	0.108357178	0.97888096
Q8WTW3	1.093	0.128293401	0.97888096
P27816	1.1	0.137503524	0.97888096
P46778	1.035	0.049630768	0.9789667
P37837	1.035	0.049630768	0.97910761
O75533	1.035	0.049630768	0.97916291
Q9H9Q2	0.937	-0.093879047	0.97950561
P48147	0.942	-0.086201035	0.97953792
P12544	1.102	0.140124224	0.9796582
Q9BQC3	0.931	-0.103146927	0.97971785
A6NIW1	0.935	-0.09696173	0.97971785
Q9NQR4	0.943	-0.084670324	0.97971785
Q92499	1.033	0.046840254	0.97971785
P33981	0.915	-0.128156351	0.97993229
O43633	0.927	-0.109358756	0.97993229
Q08379	0.928	-0.10780329	0.97993229
P10301	0.932	-0.10159814	0.97993229
Q9NXC5	0.932	-0.10159814	0.97993229

D6RIE8	0.936	-0.095419565	0.97993229
E9PLK3	0.947	-0.078563669	0.97993229
P30050	0.947	-0.078563669	0.97993229
P01111	0.949	-0.075520008	0.97993229
Q96GA3	0.95	-0.074000581	0.97993229
Q12769	0.953	-0.069451881	0.97993229
Q96BK5	0.955	-0.066427362	0.97993229
Q9NX55	0.961	-0.057391664	0.97993229
O43665-3	0.962	-0.055891201	0.97993229
O15067	1.036	0.051024003	0.97993229
Q99471	1.036	0.051024003	0.97993229
D6RFN0	1.041	0.057970069	0.97993229
Q9BQL6	1.065	0.09085343	0.97993229
O95714	1.066	0.092207438	0.97993229
J3KPP7	1.068	0.094911647	0.97993229
Q8N543	1.069	0.096261853	0.97993229
F5H2M7	1.07	0.097610797	0.97993229
Q5JTY5	1.073	0.101650076	0.97993229
K7EIU8	1.074	0.102993993	0.97993229
G5E9L0	1.074	0.102993993	0.97993229
Q96MX6	1.089	0.123003954	0.97993229
Q96LW4	1.09	0.124328135	0.97993229
G3V1J9	1.091	0.125651102	0.97993229
B4DDP2	1.094	0.129612738	0.97993229
Q9P275	0.928	-0.10780329	0.98023525
Q99497	0.948	-0.077041036	0.98023525
Q9NVI7-2	0.949	-0.075520008	0.98030608
O00186	0.933	-0.100051014	0.98033673
Q96T51	1.077	0.10701825	0.98033673
P83876	0.977	-0.033569533	0.98037683
Q96SZ6	0.939	-0.090802937	0.98079906
P63279	1.038	0.053806444	0.9810115
P05026	0.968	-0.046921047	0.98110609
Q9NUL7	0.94	-0.089267338	0.98129053
Q9NXH9	0.952	-0.070966521	0.98129053
O75964	1.032	0.045442971	0.98129053
P28370	1.075	0.10433666	0.98129053
O43731	1.087	0.12035194	0.98129053
Q00059	0.939	-0.090802937	0.98133961
P36405	1.036	0.051024003	0.98133961
P53367	1.05	0.070389328	0.98139711
Q7Z7E8	0.926	-0.110915901	0.98147812
Q13505-3	0.963	-0.054392297	0.98147812
Q9C0E2	0.978	-0.03209363	0.98147812
Q14558	1.038	0.053806444	0.98147812
Q96KG9	1.068	0.094911647	0.98147812

Q9H329	1.086	0.119024103	0.98147812
Q96BN8	1.088	0.121678557	0.98147812
Q9UGR2	0.939	-0.090802937	0.98158374
B4E040	0.964	-0.052894948	0.98158374
P52435	0.951	-0.072482754	0.98165333
P62487	1.075	0.10433666	0.98174448
B1AT46	1.094	0.129612738	0.98179163
O95163	0.953	-0.069451881	0.98184381
J3KTL2	1.032	0.045442971	0.98184467
P17096	1.02	0.028569152	0.98189605
Q9UPQ8	0.911	-0.134477041	0.98214273
Q8WY21	0.911	-0.134477041	0.98214273
G3V2S6	0.925	-0.112474729	0.98214273
P19174	0.929	-0.106249498	0.98214273
B7Z4M2	0.937	-0.093879047	0.98214273
Q9NQG5	0.939	-0.090802937	0.98214273
P20340-2	0.939	-0.090802937	0.98214273
P15954	0.939	-0.090802937	0.98214273
Q8N5M1	0.942	-0.086201035	0.98214273
Q96GY0	0.943	-0.084670324	0.98214273
J3KPV7	0.944	-0.083141235	0.98214273
Q9BRA0	0.946	-0.080087911	0.98214273
P11766	0.947	-0.078563669	0.98214273
J3QQ67	0.95	-0.074000581	0.98214273
Q9BQ52	0.951	-0.072482754	0.98214273
O75608	0.956	-0.064917477	0.98214273
Q96BP3	0.976	-0.035046947	0.98214273
Q8NHH9	0.977	-0.033569533	0.98214273
F5H6D0	1.021	0.029982866	0.98214273
P09110	1.029	0.041242982	0.98214273
Q5JTV8	1.03	0.042644337	0.98214273
Q13428-3	1.033	0.046840254	0.98214273
O15212	1.035	0.049630768	0.98214273
F8W7Q4	1.035	0.049630768	0.98214273
O60287	1.039	0.055195654	0.98214273
B4E2P2	1.058	0.081339627	0.98214273
O15397	1.067	0.093560176	0.98214273
Q7Z434	1.067	0.093560176	0.98214273
Q9UKS6	1.067	0.093560176	0.98214273
Q96T37	1.073	0.101650076	0.98214273
Q02338	1.074	0.102993993	0.98214273
P50748	1.088	0.121678557	0.98214273
E7EN86	1.1	0.137503524	0.98214273
F8W8A6	1.093	0.128293401	0.98216015
F5H2L4	1.046	0.064882852	0.98229348
Q7Z3B4	1.035	0.049630768	0.98271527

P29083	0.979	-0.030619235	0.98273583
Q15126	1.072	0.100304906	0.9830815
Q15018	0.944	-0.083141235	0.98340441
H3BV60	0.937	-0.093879047	0.98350987
O95140	1.086	0.119024103	0.98350987
Q9Y3E0	0.947	-0.078563669	0.98369227
E7ETK0	1.03	0.042644337	0.98383542
Q13625	0.959	-0.06039728	0.98409505
Q96P16	1.055	0.077242999	0.98409505
Q6IPR3	1.08	0.111031312	0.98409505
Q9NRW7	0.946	-0.080087911	0.98419498
Q96T76	0.948	-0.077041036	0.98419498
P59998	0.951	-0.072482754	0.98419498
G5E975	0.969	-0.045431429	0.98419498
P29350	0.97	-0.043943348	0.98419498
Q08211	1.029	0.041242982	0.98419498
Q29RF7	1.029	0.041242982	0.98419498
O00232	1.029	0.041242982	0.98419498
J3QT22	1.032	0.045442971	0.98419498
Q92643	1.051	0.071762669	0.98419498
Q9UBL3	1.056	0.078609835	0.98419498
Q9BXT8-1	1.07	0.097610797	0.98419498
P29353	1.075	0.10433666	0.98419498
H0Y997	1.081	0.112366523	0.98419498
P08865	0.951	-0.072482754	0.98420743
Q8NE71	1.033	0.046840254	0.98422096
O00148	1.033	0.046840254	0.98427176
Q7L266	0.942	-0.086201035	0.98437047
O75934	1.025	0.03562391	0.98437047
C9JG97	1.043	0.060739158	0.98437047
Q6P2E8	1.068	0.094911647	0.98437047
O60313-2	1.033	0.046840254	0.98448413
Q9UBB6	1.071	0.09895848	0.98448413
P51149	0.952	-0.070966521	0.98458429
Q9Y2R0	0.965	-0.051399153	0.98458429
Q9UL25	1.037	0.052415894	0.98458429
C9JTZ6	1.06	0.084064265	0.98458429
O43598	0.93	-0.104697379	0.98462609
P50453	0.95	-0.074000581	0.98475178
P23246	0.952	-0.070966521	0.98475178
Q01469	0.952	-0.070966521	0.98497674
Q9BV79	0.929	-0.106249498	0.98513798
P13284	0.932	-0.10159814	0.98513798
P06746	0.933	-0.100051014	0.98513798
Q9Y6I4	0.939	-0.090802937	0.98513798
H0Y5K5	0.943	-0.084670324	0.98513798

Q6Y7W6	0.944	-0.083141235	0.98513798
Q8NBM4	0.947	-0.078563669	0.98513798
P53609	0.951	-0.072482754	0.98513798
P22234	0.953	-0.069451881	0.98513798
Q9Y277	0.953	-0.069451881	0.98513798
O75694	0.954	-0.067938829	0.98513798
O95302	0.955	-0.066427362	0.98513798
Q9GZS1-2	0.96	-0.058893689	0.98513798
B1AK40	0.963	-0.054392297	0.98513798
Q9Y315	0.972	-0.040971781	0.98513798
Q8N5C6	0.987	-0.01887801	0.98513798
Q86X76-2	0.988	-0.017417053	0.98513798
P63208	1.022	0.031395196	0.98513798
G5E9E7	1.024	0.034215715	0.98513798
P42224	1.027	0.038436182	0.98513798
P05387	1.027	0.038436182	0.98513798
P50502	1.028	0.039840265	0.98513798
Q99614	1.029	0.041242982	0.98513798
E9PGE1	1.033	0.046840254	0.98513798
Q99543	1.034	0.048236186	0.98513798
A2RTX5	1.05	0.070389328	0.98513798
Q9Y6W5	1.062	0.086783766	0.98513798
Q9GZQ8	1.064	0.089498151	0.98513798
Q9NQH7	1.07	0.097610797	0.98513798
Q9P0K7	1.076	0.105678078	0.98513798
Q2YD98	1.081	0.112366523	0.98513798
H0Y2P2	1.084	0.116364757	0.98513798
G3V5L1	1.094	0.129612738	0.98513798
C9JZB0	1.095	0.13093087	0.98513798
C9JG87	0.943	-0.084670324	0.98531775
Q8N0X4	0.955	-0.066427362	0.98539813
O00268	1.067	0.093560176	0.98550569
O00566	1.021	0.029982866	0.98561355
O43264	1.029	0.041242982	0.98592004
Q8WVI0	1.078	0.108357178	0.98602043
Q92576	0.937	-0.093879047	0.98649842
Q13825	1.051	0.071762669	0.98667316
P62841	0.954	-0.067938829	0.98667496
Q8IWA0	1.03	0.042644337	0.98667496
Q4L180	0.92	-0.120294234	0.98721986
P60903	0.945	-0.081613766	0.98721986
P30566	0.955	-0.066427362	0.98721986
Q7Z417	0.956	-0.064917477	0.98721986
Q15042	0.957	-0.06340917	0.98721986
P30622	0.968	-0.046921047	0.98721986
Q15006	0.968	-0.046921047	0.98721986

P63000	1.025	0.03562391	0.98721986
B8ZZ43	1.064	0.089498151	0.98721986
D6RHI9	1.067	0.093560176	0.98721986
Q12962	0.94	-0.089267338	0.98730221
Q96GQ7	0.958	-0.061902439	0.98730221
Q9HC36	0.971	-0.042456799	0.98730221
Q9H583	1.016	0.022900402	0.98730221
Q9BW85	1.052	0.073134705	0.98730221
P39748	0.951	-0.072482754	0.98741048
E7EPD9	0.953	-0.069451881	0.98741048
Q9Y230	0.955	-0.066427362	0.98741048
P31431	0.923	-0.115597447	0.98746983
O14617	1.03	0.042644337	0.98746983
Q14258	1.026	0.037030731	0.98767341
P57740	1.028	0.039840265	0.98774969
Q9UQR1	0.931	-0.103146927	0.98796187
Q9NZ56	0.95	-0.074000581	0.98799577
P51948	1.062	0.086783766	0.98799577
Q9H6E4	1.059	0.082702589	0.98800068
Q00403	0.957	-0.06340917	0.98818531
H3BQQ2	0.96	-0.058893689	0.98818531
Q5VXN5	0.92	-0.120294234	0.98822326
P21399	1.029	0.041242982	0.98822326
Q6P158	1.063	0.088141597	0.98822326
B7Z2C3	0.918	-0.123433941	0.98829191
Q6P1K2	0.936	-0.095419565	0.98829191
Q9Y4L1	0.955	-0.066427362	0.98829191
Q96ER9	0.958	-0.061902439	0.98829191
Q93009	1.02	0.028569152	0.98829191
Q9NVZ3	1.055	0.077242999	0.98829191
Q86Y07	1.061	0.085424656	0.98829191
Q93074	1.079	0.109694865	0.98829191
P16383	0.949	-0.075520008	0.98853021
O75792	1.067	0.093560176	0.98869775
H7BZH9	0.938	-0.092340172	0.98872833
Q86V48	0.953	-0.069451881	0.98872833
P39023	0.956	-0.064917477	0.98872833
Q969Z0	0.956	-0.064917477	0.98872833
O14787-2	0.971	-0.042456799	0.98872833
P32969	1.024	0.034215715	0.98872833
P18085	1.024	0.034215715	0.98872833
Q8IX12	1.026	0.037030731	0.98872833
Q9BTC0	1.038	0.053806444	0.98872833
Q8WUD4	1.044	0.062121712	0.98872833
Q96J01	1.054	0.075874867	0.98872833
Q9BQ61	1.058	0.081339627	0.98872833

Q9UER7	1.062	0.086783766	0.98872833
O75886	1.062	0.086783766	0.98872833
O96028	1.064	0.089498151	0.98872833
Q9Y496	1.083	0.115033243	0.98872833
E9PEY4	1.087	0.12035194	0.98872833
B4DR17	1.102	0.140124224	0.98872833
H3BNZ1	0.953	-0.069451881	0.98873675
Q5UIP0	1.037	0.052415894	0.98873675
P07919	0.951	-0.072482754	0.98875958
K7ELQ8	0.926	-0.110915901	0.98876923
Q6P4A7	0.931	-0.103146927	0.98876923
Q9UET6	0.935	-0.09696173	0.98876923
F8WDY7	0.935	-0.09696173	0.98876923
Q9H1E5	0.936	-0.095419565	0.98876923
B4E243	0.94	-0.089267338	0.98876923
Q9BSL1	0.942	-0.086201035	0.98876923
B4DFC7	0.942	-0.086201035	0.98876923
Q96EP5	0.944	-0.083141235	0.98876923
P51649	0.945	-0.081613766	0.98876923
H3BP13	0.946	-0.080087911	0.98876923
P43246	0.947	-0.078563669	0.98876923
P61026	0.951	-0.072482754	0.98876923
O75340	0.953	-0.069451881	0.98876923
Q9Y371	0.953	-0.069451881	0.98876923
Q9BXB5	0.953	-0.069451881	0.98876923
F8W8S3	0.955	-0.066427362	0.98876923
Q86VP6	0.957	-0.06340917	0.98876923
P19338	0.957	-0.06340917	0.98876923
Q9Y263	0.957	-0.06340917	0.98876923
P55145	0.957	-0.06340917	0.98876923
Q06265	0.957	-0.06340917	0.98876923
J3KTA4	0.958	-0.061902439	0.98876923
O43252	0.958	-0.061902439	0.98876923
Q8N843	0.958	-0.061902439	0.98876923
B7Z2S9	0.958	-0.061902439	0.98876923
P14550	0.96	-0.058893689	0.98876923
P61201	0.96	-0.058893689	0.98876923
Q5T4U5	0.96	-0.058893689	0.98876923
P13798	0.962	-0.055891201	0.98876923
P82673	0.965	-0.051399153	0.98876923
Q16637	0.967	-0.048412205	0.98876923
Q99622	0.969	-0.045431429	0.98876923
Q9BWU0	0.97	-0.043943348	0.98876923
P63172	0.974	-0.038006323	0.98876923
Q9UHD9	0.976	-0.035046947	0.98876923
Q12789	1.016	0.022900402	0.98876923

Q9Y6G9	1.016	0.022900402	0.98876923
Q99986	1.016	0.022900402	0.98876923
F6XY72	1.019	0.027154052	0.98876923
H3BT71	1.021	0.029982866	0.98876923
Q14204	1.022	0.031395196	0.98876923
P28066	1.022	0.031395196	0.98876923
Q10567-3	1.023	0.032806145	0.98876923
Q7L8W6	1.023	0.032806145	0.98876923
B1AK64	1.024	0.034215715	0.98876923
P24928	1.025	0.03562391	0.98876923
P21291	1.026	0.037030731	0.98876923
Q9Y5L0	1.027	0.038436182	0.98876923
Q99808	1.032	0.045442971	0.98876923
J3QRU4	1.037	0.052415894	0.98876923
Q96HW7	1.043	0.060739158	0.98876923
Q9BYN8	1.049	0.069014678	0.98876923
O43427	1.055	0.077242999	0.98876923
J3KTE9	1.056	0.078609835	0.98876923
Q96BW5	1.057	0.079975377	0.98876923
G3V150	1.058	0.081339627	0.98876923
Q9BTA9	1.059	0.082702589	0.98876923
Q96MW1	1.059	0.082702589	0.98876923
F5H157	1.06	0.084064265	0.98876923
P56282	1.061	0.085424656	0.98876923
J3QSX6	1.062	0.086783766	0.98876923
Q9NQZ5	1.062	0.086783766	0.98876923
J3KNX7	1.064	0.089498151	0.98876923
Q8WU76	1.068	0.094911647	0.98876923
Q8N729	1.069	0.096261853	0.98876923
Q96KM6	1.069	0.096261853	0.98876923
P35080	1.07	0.097610797	0.98876923
B4DWI3	1.07	0.097610797	0.98876923
A2RRP1	1.073	0.101650076	0.98876923
Q8N5A5-2	1.074	0.102993993	0.98876923
O60232	1.075	0.10433666	0.98876923
Q92989	1.079	0.109694865	0.98876923
Q5T2R2	1.082	0.113700499	0.98876923
O95630	1.083	0.115033243	0.98876923
P29590	1.099	0.136191386	0.98876923
Q9Y383	1.073	0.101650076	0.98880175
Q9H4I9	1.089	0.123003954	0.98880175
P20674	0.964	-0.052894948	0.98908705
Q96B54	0.945	-0.081613766	0.98918699
Q5TD07	0.946	-0.080087911	0.98918699
F8VV52	0.955	-0.066427362	0.98918699
O75915	0.955	-0.066427362	0.98918699

H0YF29	0.956	-0.064917477	0.98918699
P05388	1.021	0.029982866	0.98918699
Q969S9	1.057	0.079975377	0.98947666
Q5T1J5	1.057	0.079975377	0.98962951
P78537	1.059	0.082702589	0.98978187
Q6IAN0	0.967	-0.048412205	0.98982301
P36957	0.962	-0.055891201	0.98991874
O00487	1.024	0.034215715	0.98991874
Q9NQ29	0.956	-0.064917477	0.99009017
P06865	0.957	-0.06340917	0.99009017
J9JID7	0.96	-0.058893689	0.99009017
P43304	0.961	-0.057391664	0.99009017
Q96IX5	0.966	-0.049904906	0.99009017
Q86WR0	0.972	-0.040971781	0.99009017
P14735	1.02	0.028569152	0.99009017
Q9H9M0	1.038	0.053806444	0.99009017
Q9UJX6	1.048	0.067638717	0.99009017
O14757	1.053	0.074505436	0.99009017
Q9H5N1	1.054	0.075874867	0.99009017
D6RD48	1.061	0.085424656	0.99009017
O00189	1.064	0.089498151	0.99009017
Q96KQ7	1.065	0.09085343	0.99009017
Q49AR2	1.065	0.09085343	0.99009017
Q8NBM8	1.057	0.079975377	0.99026313
Q9UIU6	1.067	0.093560176	0.99026313
Q9BSV6	0.969	-0.045431429	0.99026384
Q92804	0.949	-0.075520008	0.99031084
Q9Y314	0.955	-0.066427362	0.99031084
Q12792	0.962	-0.055891201	0.99031084
Q8TCA0	1.063	0.088141597	0.99031084
O00291	1.062	0.086783766	0.99041203
Q4KMP7	0.948	-0.077041036	0.99061941
Q9H7Z7	0.951	-0.072482754	0.99061941
B4DQJ8	0.961	-0.057391664	0.99061941
Q8NDX5	0.968	-0.046921047	0.99061941
Q5T1B0	0.97	-0.043943348	0.99061941
Q9UK41	0.973	-0.03948829	0.99061941
P36507	1.009	0.012926174	0.99061941
H0YDU8	1.012	0.01720929	0.99061941
O75179	1.049	0.069014678	0.99061941
D6RAK3	1.056	0.078609835	0.99061941
Q9BRD0	1.085	0.117695043	0.99061941
P21796	0.961	-0.057391664	0.99077474
Q9Y2P5	1.07	0.097610797	0.99077474
Q7RTS9	0.934	-0.098505545	0.99086442
Q9HBH5	0.956	-0.064917477	0.99086442

Q9H0U9	0.958	-0.061902439	0.99086442
O60361	0.961	-0.057391664	0.99086442
Q7Z4Q2	0.962	-0.055891201	0.99086442
Q96BR5	0.976	-0.035046947	0.99086442
P39656	1.019	0.027154052	0.99086442
P61086	1.021	0.029982866	0.99086442
Q9Y5B8	1.031	0.044044333	0.99086442
P35610	1.054	0.075874867	0.99086442
Q08AD1	1.09	0.124328135	0.99086442
Q5JUR7	0.945	-0.081613766	0.99087093
F8W938	0.951	-0.072482754	0.99087093
H0YNE9	0.956	-0.064917477	0.99087093
Q9BSF4	0.96	-0.058893689	0.99087093
Q16643	0.961	-0.057391664	0.99087093
Q9GZR2-2	1.034	0.048236186	0.99087093
Q9UFW8	1.049	0.069014678	0.99087093
Q9UIL1	1.05	0.070389328	0.99087093
P82914	0.949	-0.075520008	0.99093373
Q969N2	0.951	-0.072482754	0.99093373
O43670	0.952	-0.070966521	0.99093373
Q9NVM9	0.952	-0.070966521	0.99093373
Q8N8S7	0.953	-0.069451881	0.99093373
Q13409-2	0.955	-0.066427362	0.99093373
Q8TAA9	0.956	-0.064917477	0.99093373
J3KNL6	0.958	-0.061902439	0.99093373
P62330	0.958	-0.061902439	0.99093373
Q9Y520	0.96	-0.058893689	0.99093373
E9PRV2	0.96	-0.058893689	0.99093373
G3V4K3	0.96	-0.058893689	0.99093373
O75600	0.961	-0.057391664	0.99093373
Q86XP3	0.962	-0.055891201	0.99093373
P60174-1	0.962	-0.055891201	0.99093373
P31930	0.962	-0.055891201	0.99093373
A2A274	0.962	-0.055891201	0.99093373
P08708	0.962	-0.055891201	0.99093373
Q8WZA9	0.963	-0.054392297	0.99093373
P22681	0.963	-0.054392297	0.99093373
Q53GS9	0.964	-0.052894948	0.99093373
Q16795	0.964	-0.052894948	0.99093373
P36542	0.964	-0.052894948	0.99093373
Q96T88	0.965	-0.051399153	0.99093373
E9PF32	0.965	-0.051399153	0.99093373
Q9P0V9	0.966	-0.049904906	0.99093373
P48960	0.97	-0.043943348	0.99093373
O95219	0.973	-0.03948829	0.99093373
P17066	0.974	-0.038006323	0.99093373

Q9Y697	0.976	-0.035046947	0.99093373
Q13464	0.978	-0.03209363	0.99093373
E9PNM1	0.98	-0.029146346	0.99093373
Q9P2R3	0.982	-0.02620507	0.99093373
O15371	1.008	0.011495639	0.99093373
Q9UBB4	1.009	0.012926174	0.99093373
P28070	1.013	0.018634174	0.99093373
P29401	1.016	0.022900402	0.99093373
Q9Y2X3	1.016	0.022900402	0.99093373
P04843	1.016	0.022900402	0.99093373
Q9BTV4	1.016	0.022900402	0.99093373
P23396	1.017	0.024319679	0.99093373
P25789	1.017	0.024319679	0.99093373
Q16629	1.017	0.024319679	0.99093373
P63104	1.018	0.025737561	0.99093373
O95881	1.02	0.028569152	0.99093373
Q99536	1.021	0.029982866	0.99093373
P10606	1.021	0.029982866	0.99093373
Q7RTV0	1.035	0.049630768	0.99093373
Q9P253	1.037	0.052415894	0.99093373
Q8IZ69	1.039	0.055195654	0.99093373
Q8NAV1	1.044	0.062121712	0.99093373
Q9NZJ9	1.048	0.067638717	0.99093373
Q9Y6Y0	1.053	0.074505436	0.99093373
D6RIZ4	1.053	0.074505436	0.99093373
Q8N0Z6	1.055	0.077242999	0.99093373
Q86UL3	1.058	0.081339627	0.99093373
P52179	1.058	0.081339627	0.99093373
F5H604	1.063	0.088141597	0.99093373
Q96R06	1.064	0.089498151	0.99093373
C9J7N1	1.064	0.089498151	0.99093373
P23025	1.065	0.09085343	0.99093373
O95989	1.066	0.092207438	0.99093373
O75113	1.068	0.094911647	0.99093373
F2Z3C5	1.068	0.094911647	0.99093373
Q9Y3U8	1.008	0.011495639	0.99100439
Q9Y6B6	1.046	0.064882852	0.99100439
E5RGS7	0.943	-0.084670324	0.9911888
Q86YD1	0.945	-0.081613766	0.9911888
P62491	0.952	-0.070966521	0.9911888
P36915	0.953	-0.069451881	0.9911888
Q6YN16	0.959	-0.06039728	0.9911888
Q8WY22	0.961	-0.057391664	0.9911888
Q9BUT1	0.962	-0.055891201	0.9911888
Q49AL5	0.963	-0.054392297	0.9911888
Q13435	0.964	-0.052894948	0.9911888

P41091	0.964	-0.052894948	0.9911888
Q96C23	0.964	-0.052894948	0.9911888
P18669	0.965	-0.051399153	0.9911888
P55263	0.966	-0.049904906	0.9911888
P84090	0.966	-0.049904906	0.9911888
O94776	0.967	-0.048412205	0.9911888
Q7Z6M1	0.968	-0.046921047	0.9911888
Q8TF05-2	0.971	-0.042456799	0.9911888
Q9BXY0	0.979	-0.030619235	0.9911888
Q8NEY1	0.979	-0.030619235	0.9911888
Q9NWT1	1.004	0.005759269	0.9911888
P53634	1.013	0.018634174	0.9911888
Q16891	1.015	0.021479727	0.9911888
Q15046	1.015	0.021479727	0.9911888
P07305	1.019	0.027154052	0.9911888
Q9NQZ2	1.026	0.037030731	0.9911888
P36543	1.027	0.038436182	0.9911888
K4DID8	1.041	0.057970069	0.9911888
Q5BJF2	1.042	0.059355278	0.9911888
P11802	1.044	0.062121712	0.9911888
Q99470	1.046	0.064882852	0.9911888
O75170	1.047	0.066261442	0.9911888
Q15022	1.048	0.067638717	0.9911888
Q06330	1.05	0.070389328	0.9911888
A8MTG8	1.055	0.077242999	0.9911888
Q96FN4	1.056	0.078609835	0.9911888
Q69YU5	1.061	0.085424656	0.9911888
Q86UY8	1.072	0.100304906	0.9911888
Q96JQ2	0.937	-0.093879047	0.99140859
Q96EE3	0.946	-0.080087911	0.99140859
P62993	0.955	-0.066427362	0.99140859
P17612	0.956	-0.064917477	0.99140859
Q96S44	0.957	-0.06340917	0.99140859
Q9BTE6	0.958	-0.061902439	0.99140859
P58546	0.958	-0.061902439	0.99140859
Q96CN7	0.961	-0.057391664	0.99140859
Q5VY60	0.962	-0.055891201	0.99140859
P07814	0.966	-0.049904906	0.99140859
P62277	0.966	-0.049904906	0.99140859
P13073	0.966	-0.049904906	0.99140859
P62753	0.966	-0.049904906	0.99140859
Q96ST3	0.968	-0.046921047	0.99140859
Q9NR09	0.984	-0.023269779	0.99140859
Q9BRU9	1.006	0.008630305	0.99140859
O95202	1.007	0.010063683	0.99140859
P49721	1.007	0.010063683	0.99140859

P18074	1.007	0.010063683	0.99140859
Q9UNS2	1.009	0.012926174	0.99140859
O94826	1.013	0.018634174	0.99140859
O95456	1.016	0.022900402	0.99140859
F5H442	1.036	0.051024003	0.99140859
Q9BVL2	1.038	0.053806444	0.99140859
Q9NRZ9	1.044	0.062121712	0.99140859
Q9Y4B6	1.045	0.063502942	0.99140859
Q9BV20	1.047	0.066261442	0.99140859
Q70UQ0	1.048	0.067638717	0.99140859
Q8NFV4	1.051	0.071762669	0.99140859
Q96TA2	1.052	0.073134705	0.99140859
Q6P1A2	1.053	0.074505436	0.99140859
K7EK56	1.055	0.077242999	0.99140859
P19387	1.024	0.034215715	0.9915628
Q92575	0.989	-0.015957574	0.99161637
F8W0J4	1.072	0.100304906	0.99161637
Q14019	0.966	-0.049904906	0.99168767
Q9Y376	0.98	-0.029146346	0.99168767
P12956	1.013	0.018634174	0.99168767
O95825	0.969	-0.045431429	0.99191196
E7ENA9	0.97	-0.043943348	0.99191196
Q99961	1.019	0.027154052	0.99191196
A2A2V1	1.055	0.077242999	0.99191196
Q9Y5L4	0.968	-0.046921047	0.99212654
Q15370	0.97	-0.043943348	0.99238715
P11387	0.967	-0.048412205	0.99239241
Q15599	0.956	-0.064917477	0.99281235
O95478	0.965	-0.051399153	0.99281235
Q9BUQ8	0.968	-0.046921047	0.99281235
P61081	0.973	-0.03948829	0.99281235
G5E9Z2	1.006	0.008630305	0.99281235
Q16555	1.021	0.029982866	0.99281235
O15111	1.027	0.038436182	0.99281235
Q7L3B6	1.041	0.057970069	0.99281235
Q92734	0.97	-0.043943348	0.99289342
O76094	1.016	0.022900402	0.99289342
Q2TA77	1.036	0.051024003	0.99289342
Q96E11	1.042	0.059355278	0.99289342
Q13153	1.037	0.052415894	0.99310515
Q9UPY3	0.949	-0.075520008	0.99313884
Q8NCW5	0.959	-0.06039728	0.99313884
P62854	0.967	-0.048412205	0.99313884
P60468	0.973	-0.03948829	0.99313884
Q9BRP8	0.984	-0.023269779	0.99313884
Q8NCA5	1.022	0.031395196	0.99313884

P05161	0.964	-0.052894948	0.99315214
Q9NPH2	0.963	-0.054392297	0.99334412
Q9H3K2	1.069	0.096261853	0.99334412
Q53EU6	0.965	-0.051399153	0.99340198
Q7KZ85	1.013	0.018634174	0.99340198
Q15750	1.027	0.038436182	0.99343401
Q9UEG4	0.975	-0.036525876	0.99347018
Q1KMD3	1.02	0.028569152	0.99372798
Q9C005	1.011	0.015782997	0.99407158
Q9H8J5	1.065	0.09085343	0.99407158
A6NEM2	1.018	0.025737561	0.99442464
Q92610	0.958	-0.061902439	0.99443223
Q86Y56	0.965	-0.051399153	0.99443223
P15531	0.969	-0.045431429	0.99443223
Q8N1G4	0.969	-0.045431429	0.99443223
O95169	0.982	-0.02620507	0.99443223
Q13057	0.984	-0.023269779	0.99443223
Q92616	1.011	0.015782997	0.99443223
Q15029	1.011	0.015782997	0.99443223
P04080	1.011	0.015782997	0.99443223
Q13823	1.011	0.015782997	0.99443223
A6NFX8	1.016	0.022900402	0.99443223
P82979	1.017	0.024319679	0.99443223
P51116	1.021	0.029982866	0.99443223
D6RB85	1.024	0.034215715	0.99443223
G5E9L8	1.036	0.051024003	0.99443223
A8MWR6	1.042	0.059355278	0.99443223
Q9HB90	1.044	0.062121712	0.99443223
E5RJI9	1.044	0.062121712	0.99443223
P48634	1.048	0.067638717	0.99443223
P32780	1.049	0.069014678	0.99443223
Q6XQN6	1.059	0.082702589	0.99443223
P82921	0.974	-0.038006323	0.99496176
O75083	0.969	-0.045431429	0.9953101
P54727	1.011	0.015782997	0.9953101
Q00013	1.05	0.070389328	0.99541269
P33176	1.011	0.015782997	0.99560256
Q8TBC4	1.025	0.03562391	0.99582275
Q8N8A6	0.968	-0.046921047	0.99589302
Q86YP4	1.032	0.045442971	0.99593832
F5H721	0.984	-0.023269779	0.99603583
P53701	1.044	0.062121712	0.99603583
O00399	1.048	0.067638717	0.99603583
Q6IBS0	0.969	-0.045431429	0.99607268
Q9H814	1.034	0.048236186	0.99618593
Q5T200	1.045	0.063502942	0.99626552

Q14527	0.96	-0.058893689	0.99648175
H7BYQ6	0.961	-0.057391664	0.99648175
Q16543	0.963	-0.054392297	0.99648175
Q14152	0.969	-0.045431429	0.99648175
P48163	0.971	-0.042456799	0.99648175
O60826	0.976	-0.035046947	0.99648175
Q96K17	0.977	-0.033569533	0.99648175
P20042	1.009	0.012926174	0.99648175
H7BXW3	1.032	0.045442971	0.99648175
Q96ME1-4	1.05	0.070389328	0.99648175
F8WBK5	1.039	0.055195654	0.99649756
Q99519	1.055	0.077242999	0.99649756
Q9NY12	0.972	-0.040971781	0.99683288
Q7Z7N9	0.973	-0.03948829	0.99691842
Q9UGP8	1.041	0.057970069	0.99700444
Q5SW79	1.051	0.071762669	0.99720421
Q9NVP1	0.965	-0.051399153	0.99738939
Q5T3I0	0.987	-0.01887801	0.99738939
B4DR61	1.008	0.011495639	0.99738939
O75531	1.008	0.011495639	0.99738939
O95573	1.018	0.025737561	0.99738939
Q96JM3	1.041	0.057970069	0.99738939
P14373	1.047	0.066261442	0.99738939
Q58FF7	1.066	0.092207438	0.99738939
P12830	0.97	-0.043943348	0.99743301
O00443	0.97	-0.043943348	0.99763752
P49406	0.966	-0.049904906	0.99779308
P17655	0.97	-0.043943348	0.99779308
Q63ZY3	1.038	0.053806444	0.99779308
Q9H900	1.055	0.077242999	0.99779327
Q9UHG3	1.006	0.008630305	0.9980328
Q96DI7	1.016	0.022900402	0.9980328
Q14139	0.987	-0.01887801	0.99804859
B4DR12	0.962	-0.055891201	0.99806311
E7EM64	0.964	-0.052894948	0.99806311
P00338	0.97	-0.043943348	0.99806311
O75643	0.971	-0.042456799	0.99806311
Q13523	0.971	-0.042456799	0.99806311
Q9UL18	0.976	-0.035046947	0.99806311
Q9H3S7	0.986	-0.020340448	0.99806311
Q5T6F2	0.987	-0.01887801	0.99806311
Q8N983-4	1.009	0.012926174	0.99806311
F5GZX4	1.039	0.055195654	0.99806311
Q9UBQ5	0.971	-0.042456799	0.9982978
P53611	1.036	0.051024003	0.99838832
Q8NEC7	1.043	0.060739158	0.99843379

P51532-4	0.963	-0.054392297	0.99845563
P30040	0.971	-0.042456799	0.99845563
G5E9X3	0.963	-0.054392297	0.99863192
Q86XN8	0.964	-0.052894948	0.99863192
P09960	0.971	-0.042456799	0.99863192
O75390	0.971	-0.042456799	0.99863192
J3KPW7	0.971	-0.042456799	0.99863192
P04181	0.971	-0.042456799	0.99863192
Q86XI2	0.972	-0.040971781	0.99863192
Q9NVH2	0.974	-0.038006323	0.99863192
P12236	0.975	-0.036525876	0.99863192
Q8NEZ2	0.981	-0.027674958	0.99863192
Q9BV40	0.985	-0.02180437	0.99863192
P22314	1.008	0.011495639	0.99863192
E5RI99	1.008	0.011495639	0.99863192
Q8IUD2	1.031	0.044044333	0.99863192
Q9Y6V7	1.032	0.045442971	0.99863192
Q9NQX4	1.038	0.053806444	0.99863192
P78362	1.033	0.046840254	0.99863346
Q9H501	1.016	0.022900402	0.99871515
J3KNI1	1.035	0.049630768	0.99871515
P61106	0.972	-0.040971781	0.99874494
Q9P0S9	0.987	-0.01887801	0.9988384
P40222	1.008	0.011495639	0.99896752
Q9BUR5	1.04	0.056583528	0.99896752
Q7L523	0.967	-0.048412205	0.99914505
P21912	0.96	-0.058893689	0.99920944
P09874	0.972	-0.040971781	0.99925211
Q9Y5B0	0.976	-0.035046947	0.99925211
Q9H553	0.979	-0.030619235	0.99925211
Q9NQ55	0.982	-0.02620507	0.99925211
Q9NQT5	1.012	0.01720929	0.99925211
O00401	1.056	0.078609835	0.99925211
P55735	1.001	0.001441974	0.99961317
O14561	1.002	0.002882509	0.99969526
Q9Y4F1	1.036	0.051024003	0.99976014
Q9H0A0	0.972	-0.040971781	0.99992055
Q92600	0.988	-0.017417053	0.99999255
E5RFN5	0.96	-0.058893689	1
Q6ZN55	0.962	-0.055891201	1
O60884	0.964	-0.052894948	1
Q969X6	0.966	-0.049904906	1
O00505	0.967	-0.048412205	1
Q9H7B2	0.968	-0.046921047	1
Q96II8	0.968	-0.046921047	1
Q8WXW3	0.968	-0.046921047	1

P14621	0.968	-0.046921047	1
Q5T8P6-2	0.969	-0.045431429	1
Q9NWT6	0.969	-0.045431429	1
A0MZ66	0.97	-0.043943348	1
Q14562	0.97	-0.043943348	1
Q9H4A6	0.971	-0.042456799	1
P23526	0.972	-0.040971781	1
Q15435	0.972	-0.040971781	1
Q9GZR2	0.972	-0.040971781	1
Q12874	0.973	-0.03948829	1
P33240	0.973	-0.03948829	1
Q9BQA1	0.973	-0.03948829	1
E7ESD2	0.973	-0.03948829	1
Q92558	0.973	-0.03948829	1
Q9Y6M7	0.973	-0.03948829	1
H0YCP6	0.973	-0.03948829	1
Q5VYK3	0.974	-0.038006323	1
P42285	0.974	-0.038006323	1
P60228	0.974	-0.038006323	1
P22087	0.974	-0.038006323	1
P35232	0.974	-0.038006323	1
P62701	0.974	-0.038006323	1
Q9Y6C9	0.974	-0.038006323	1
Q96RE7	0.974	-0.038006323	1
P06748	0.975	-0.036525876	1
Q969H8	0.975	-0.036525876	1
Q9H5V9	0.975	-0.036525876	1
Q71RC2	0.976	-0.035046947	1
K7EJV9	0.976	-0.035046947	1
Q9H6S0	0.976	-0.035046947	1
P38919	0.977	-0.033569533	1
Q99873-3	0.977	-0.033569533	1
P30086	0.977	-0.033569533	1
Q9P015	0.977	-0.033569533	1
Q00610	0.978	-0.03209363	1
P11586	0.978	-0.03209363	1
P19784	0.978	-0.03209363	1
J3KQU9	0.978	-0.03209363	1
Q9NTZ6	0.978	-0.03209363	1
J3KMZ7	0.978	-0.03209363	1
Q49AN9	0.978	-0.03209363	1
H0YLB5	0.978	-0.03209363	1
P49207	0.978	-0.03209363	1
P50395	0.979	-0.030619235	1
E7EVA0	0.979	-0.030619235	1
Q7L2E3-2	0.979	-0.030619235	1

Q9BWD1	0.979	-0.030619235	1
Q9UI26	0.979	-0.030619235	1
P61088	0.979	-0.030619235	1
P62316	0.979	-0.030619235	1
O95716	0.979	-0.030619235	1
G3XAG2	0.979	-0.030619235	1
Q9BXK1	0.979	-0.030619235	1
Q15181	0.98	-0.029146346	1
Q13308	0.98	-0.029146346	1
P16989	0.98	-0.029146346	1
O75976	0.98	-0.029146346	1
Q01081	0.98	-0.029146346	1
G3V0I5	0.98	-0.029146346	1
Q9NPD8	0.98	-0.029146346	1
Q96QC0	0.98	-0.029146346	1
B1AKN6	0.98	-0.029146346	1
Q9Y512	0.981	-0.027674958	1
P78318	0.981	-0.027674958	1
J3QQQ9	0.981	-0.027674958	1
Q8WWQ0	0.981	-0.027674958	1
Q9Y248	0.981	-0.027674958	1
Q14137	0.982	-0.02620507	1
Q6PD62	0.982	-0.02620507	1
Q5T1M5	0.982	-0.02620507	1
P52948	0.983	-0.024736678	1
Q9NSD9	0.983	-0.024736678	1
B3KSH1	0.983	-0.024736678	1
P49257	0.983	-0.024736678	1
O95861	0.983	-0.024736678	1
P07686	0.983	-0.024736678	1
Q99598	0.983	-0.024736678	1
E9PNS7	0.983	-0.024736678	1
Q6I9Y2	0.983	-0.024736678	1
P63272	0.983	-0.024736678	1
E7ENJ7	0.983	-0.024736678	1
P15880	0.984	-0.023269779	1
Q8IWZ3-6	0.984	-0.023269779	1
B9A041	0.984	-0.023269779	1
Q9NXG2	0.984	-0.023269779	1
Q8WXF1	0.984	-0.023269779	1
Q01433	0.984	-0.023269779	1
F8W840	0.984	-0.023269779	1
Q6ZN16	0.984	-0.023269779	1
Q15233	0.985	-0.02180437	1
Q8N1F7	0.985	-0.02180437	1
B3KS98	0.985	-0.02180437	1

P09455	0.985	-0.02180437	1
Q9BRG1	0.985	-0.02180437	1
P62891	0.985	-0.02180437	1
Q8N163	0.986	-0.020340448	1
Q01780	0.986	-0.020340448	1
Q10713	0.986	-0.020340448	1
P10155	0.986	-0.020340448	1
Q6P3X3	0.986	-0.020340448	1
Q4G0F5	0.986	-0.020340448	1
O00461	0.986	-0.020340448	1
Q9P2J8	0.986	-0.020340448	1
O14579	0.987	-0.01887801	1
Q9UBC2	0.987	-0.01887801	1
P30042	0.987	-0.01887801	1
P35269	0.987	-0.01887801	1
Q9BZJ0	0.987	-0.01887801	1
Q9Y3B4	0.987	-0.01887801	1
Q9UBM7	0.987	-0.01887801	1
P20645	0.987	-0.01887801	1
E7EWI9	0.988	-0.017417053	1
Q08945	0.988	-0.017417053	1
Q6PJT7	0.988	-0.017417053	1
Q9GZS3	0.988	-0.017417053	1
H3BN98	0.988	-0.017417053	1
D3YTB1	0.988	-0.017417053	1
Q9NZ63	0.988	-0.017417053	1
Q5UCC4	0.988	-0.017417053	1
Q8N323	0.988	-0.017417053	1
Q5JTH9	0.989	-0.015957574	1
O00267	0.989	-0.015957574	1
Q13085	0.989	-0.015957574	1
P62906	0.989	-0.015957574	1
Q6PKG0	0.989	-0.015957574	1
Q9H8H0	0.989	-0.015957574	1
Q9BXW7	0.989	-0.015957574	1
Q05048	0.989	-0.015957574	1
Q12830	0.989	-0.015957574	1
Q5BJD5	0.989	-0.015957574	1
G8JLE5	0.989	-0.015957574	1
H7C4S8	0.989	-0.015957574	1
Q9UGL1	0.989	-0.015957574	1
Q6P2Q9	0.99	-0.01449957	1
P02545	0.99	-0.01449957	1
Q9Y2A7	0.99	-0.01449957	1
Q05086	0.99	-0.01449957	1
Q9H845	0.99	-0.01449957	1

Q8TBX8	0.99	-0.01449957	1
Q9NP72	0.99	-0.01449957	1
B4DV38	0.99	-0.01449957	1
O43464	0.99	-0.01449957	1
H0YAA3	0.99	-0.01449957	1
Q8TEU8	0.99	-0.01449957	1
Q9HCK1	0.99	-0.01449957	1
P40926	0.991	-0.013043037	1
P25787	0.991	-0.013043037	1
Q9UBX3	0.991	-0.013043037	1
Q9H936	0.991	-0.013043037	1
O75530	0.991	-0.013043037	1
Q9UIQ6	0.991	-0.013043037	1
P09132	0.991	-0.013043037	1
O43924	0.991	-0.013043037	1
Q9Y3B2	0.991	-0.013043037	1
P13196	0.991	-0.013043037	1
Q9Y5N5	0.991	-0.013043037	1
P53618	0.992	-0.011587974	1
P23193	0.992	-0.011587974	1
E7EWS7	0.992	-0.011587974	1
P54725	0.992	-0.011587974	1
Q13418	0.992	-0.011587974	1
O15514	0.992	-0.011587974	1
O60942	0.992	-0.011587974	1
D6R9Q5	0.992	-0.011587974	1
Q15262	0.992	-0.011587974	1
P08107	0.993	-0.010134377	1
P07195	0.993	-0.010134377	1
P78316	0.993	-0.010134377	1
Q16204	0.993	-0.010134377	1
Q14739	0.993	-0.010134377	1
Q9BV57	0.993	-0.010134377	1
Q8NEZ5	0.993	-0.010134377	1
B4DTG6	0.993	-0.010134377	1
Q6P1L8	0.993	-0.010134377	1
Q92759	0.993	-0.010134377	1
Q9HBL8	0.993	-0.010134377	1
F6WCX7	0.993	-0.010134377	1
Q76N25	0.993	-0.010134377	1
P62917	0.994	-0.008682243	1
P52294	0.994	-0.008682243	1
A8MYA6	0.994	-0.008682243	1
O60749	0.994	-0.008682243	1
Q9NVX2	0.994	-0.008682243	1
Q9UBU9	0.994	-0.008682243	1

Q13363	0.994	-0.008682243	1
Q9Y2Q3	0.994	-0.008682243	1
Q969T9	0.994	-0.008682243	1
Q6NUQ1	0.994	-0.008682243	1
B7Z355	0.994	-0.008682243	1
P55072	0.995	-0.007231569	1
Q14789	0.995	-0.007231569	1
Q13547	0.995	-0.007231569	1
P25786	0.995	-0.007231569	1
Q96PZ0	0.995	-0.007231569	1
P43362	0.995	-0.007231569	1
P78417	0.995	-0.007231569	1
P62820	0.995	-0.007231569	1
O75746	0.995	-0.007231569	1
O00442	0.995	-0.007231569	1
O75569	0.995	-0.007231569	1
E9PCW1	0.995	-0.007231569	1
E9PGM9	0.995	-0.007231569	1
Q96JB2	0.995	-0.007231569	1
O60256	0.996	-0.005782353	1
P09417	0.996	-0.005782353	1
Q68E01	0.996	-0.005782353	1
Q9BXK5	0.996	-0.005782353	1
Q9ULP9	0.996	-0.005782353	1
Q99653	0.996	-0.005782353	1
P62851	0.996	-0.005782353	1
Q6AI12	0.996	-0.005782353	1
Q9Y6J9	0.996	-0.005782353	1
Q99459	0.997	-0.00433459	1
P46777	0.997	-0.00433459	1
Q06124	0.997	-0.00433459	1
F5H345	0.997	-0.00433459	1
Q93052	0.997	-0.00433459	1
P53004	0.997	-0.00433459	1
J3QL65	0.997	-0.00433459	1
Q16537	0.997	-0.00433459	1
P35573	0.997	-0.00433459	1
Q9NPL8	0.997	-0.00433459	1
P63010	0.998	-0.002888279	1
F8VRQ1	0.998	-0.002888279	1
Q9NY33	0.998	-0.002888279	1
Q15020	0.998	-0.002888279	1
P29144	0.998	-0.002888279	1
Q99623	0.998	-0.002888279	1
Q96SB3	0.998	-0.002888279	1
P41236	0.998	-0.002888279	1

P28072	0.998	-0.002888279	1
B1APJ0	0.998	-0.002888279	1
Q9NWB6	0.998	-0.002888279	1
B7ZBY5	0.999	-0.001443417	1
Q9UIC8	0.999	-0.001443417	1
Q9NVT9	0.999	-0.001443417	1
Q96CB9	0.999	-0.001443417	1
Q92621	1	0	1
Q9BXJ9	1	0	1
A5YKK6	1	0	1
O75534	1	0	1
E9PC51	1	0	1
Q8N0Y7	1	0	1
P35527	1	0	1
Q15942	1	0	1
Q07864	1	0	1
A6NDU8	1	0	1
E9PMQ6	1	0	1
O43159	1	0	1
Q9NR56-5	1	0	1
Q5MIZ7	1	0	1
Q08345	1	0	1
Q9NS87	1	0	1
G5EA09	1	0	1
Q96HA7	1	0	1
Q8IYU8	1	0	1
Q7Z794	1	0	1
Q01167	1	0	1
P98171	1	0	1
F5H5E2	1	0	1
Q96PV6	1	0	1
P22413	1	0	1
E7ES96	1	0	1
C9JQS9	1	0	1
F5H420	1	0	1
Q5JPI3	1	0	1
F8VQE3	1	0	1
P46934	1	0	1
C9JPT4	1	0	1
Q9BV68	1	0	1
Q9UKG1	1	0	1
P83111	1	0	1
O15264	1	0	1
Q86YM7	1	0	1
O75420	1	0	1
Q9UNH6	1	0	1

Q9Y6J0	1	0	1
G3V128	1	0	1
C9JZN9	1	0	1
F6RY50	1	0	1
Q9NPG1	1	0	1
O60427	1	0	1
Q9BQG0	1.001	0.001441974	1
Q9BUF5	1.001	0.001441974	1
O95831	1.001	0.001441974	1
P06730	1.001	0.001441974	1
Q96IJ6	1.001	0.001441974	1
Q96EK6	1.001	0.001441974	1
O95197-3	1.001	0.001441974	1
P83731	1.001	0.001441974	1
P13645	1.001	0.001441974	1
Q9UBI1	1.001	0.001441974	1
O95721	1.001	0.001441974	1
P42704	1.002	0.002882509	1
O14744	1.002	0.002882509	1
P49916	1.002	0.002882509	1
Q6P1J9	1.002	0.002882509	1
Q9BZE4	1.003	0.004321606	1
P62241	1.003	0.004321606	1
P48556	1.003	0.004321606	1
P98194	1.003	0.004321606	1
O75190	1.003	0.004321606	1
F5H4E4	1.003	0.004321606	1
Q99959	1.003	0.004321606	1
P13521	1.003	0.004321606	1
P35606	1.004	0.005759269	1
Q9BTW9	1.004	0.005759269	1
P07910	1.004	0.005759269	1
B7Z4Q3	1.004	0.005759269	1
Q8IY37	1.004	0.005759269	1
Q9BTE7	1.004	0.005759269	1
Q9NVU7	1.004	0.005759269	1
Q9BQ69	1.004	0.005759269	1
Q96F63	1.004	0.005759269	1
O60870	1.004	0.005759269	1
E7EUN9	1.004	0.005759269	1
P04075	1.005	0.007195501	1
Q00839	1.005	0.007195501	1
P31689	1.005	0.007195501	1
P16422	1.005	0.007195501	1
Q9BWJ5	1.005	0.007195501	1
P83436	1.005	0.007195501	1

Q9UBS8	1.005	0.007195501	1
Q14498-2	1.006	0.008630305	1
O15160	1.006	0.008630305	1
P61586	1.006	0.008630305	1
Q9Y5S9	1.006	0.008630305	1
J3QRU8	1.006	0.008630305	1
P48059-2	1.006	0.008630305	1
Q9UH17	1.006	0.008630305	1
Q8N999	1.006	0.008630305	1
P10809	1.007	0.010063683	1
Q9Y2W1	1.007	0.010063683	1
P28074	1.007	0.010063683	1
Q9Y224	1.007	0.010063683	1
Q99797	1.007	0.010063683	1
Q9H2P9	1.007	0.010063683	1
Q4JDL3	1.007	0.010063683	1
Q6P3R8	1.007	0.010063683	1
Q92888	1.008	0.011495639	1
Q15427	1.008	0.011495639	1
Q9NX46	1.008	0.011495639	1
Q5VV41	1.008	0.011495639	1
Q96RD7	1.008	0.011495639	1
Q96S52	1.008	0.011495639	1
P82932	1.008	0.011495639	1
Q9BUK6	1.008	0.011495639	1
Q9Y5R8	1.008	0.011495639	1
Q96C24	1.008	0.011495639	1
Q9NPA0	1.009	0.012926174	1
Q5VZK9	1.009	0.012926174	1
P10636-4	1.01	0.014355293	1
Q32P28	1.01	0.014355293	1
Q6IAA8	1.01	0.014355293	1
Q8IYS1	1.01	0.014355293	1
Q4J6C6	1.01	0.014355293	1
Q12899	1.01	0.014355293	1
Q8WUM0	1.011	0.015782997	1
Q96RS6	1.011	0.015782997	1
Q01970	1.011	0.015782997	1
O60306	1.011	0.015782997	1
P21741	1.011	0.015782997	1
Q9H9A6	1.011	0.015782997	1
Q10469	1.011	0.015782997	1
O15042	1.012	0.01720929	1
P35270	1.012	0.01720929	1
E9PC97	1.012	0.01720929	1
Q9HCU5	1.012	0.01720929	1

O00422	1.012	0.01720929	1
Q9UFC0	1.012	0.01720929	1
P62877	1.012	0.01720929	1
Q9NVV0	1.012	0.01720929	1
O15031	1.013	0.018634174	1
O94762	1.013	0.018634174	1
I3L213	1.013	0.018634174	1
B4DFR4	1.014	0.020057652	1
Q99436	1.014	0.020057652	1
P52594	1.014	0.020057652	1
E7ENA2	1.014	0.020057652	1
O60499	1.014	0.020057652	1
F8VUA2	1.014	0.020057652	1
P07711	1.014	0.020057652	1
Q9NVA1	1.014	0.020057652	1
P33552	1.014	0.020057652	1
Q04323	1.015	0.021479727	1
Q9H788	1.015	0.021479727	1
P62834	1.015	0.021479727	1
Q5JRI3	1.015	0.021479727	1
P41223	1.015	0.021479727	1
Q9HB40	1.015	0.021479727	1
Q8NA72	1.015	0.021479727	1
Q86WJ1	1.016	0.022900402	1
Q5JPB2	1.016	0.022900402	1
O60812	1.017	0.024319679	1
P78345	1.017	0.024319679	1
P00533	1.017	0.024319679	1
Q9Y289	1.017	0.024319679	1
Q9BTD8	1.018	0.025737561	1
Q96G46	1.018	0.025737561	1
J3KPN1	1.018	0.025737561	1
P03915	1.019	0.027154052	1
Q9BYD2	1.02	0.028569152	1
Q13049	1.02	0.028569152	1
Q96A49	1.021	0.029982866	1
Q9BSC4	1.021	0.029982866	1
Q9Y6D9	1.021	0.029982866	1
D6RAA5	1.022	0.031395196	1
Q5MNZ6	1.022	0.031395196	1
Q8TAD8	1.023	0.032806145	1
Q9NNW5	1.024	0.034215715	1
Q15311	1.024	0.034215715	1
Q9BPY3	1.024	0.034215715	1
Q8NHQ9	1.025	0.03562391	1
O60927	1.025	0.03562391	1

Q96K76	1.025	0.03562391	1
E7EVG2	1.025	0.03562391	1
Q92692	1.025	0.03562391	1
B0V109	1.026	0.037030731	1
Q8WVK2	1.026	0.037030731	1
O43314	1.026	0.037030731	1
Q9UQ13	1.026	0.037030731	1
Q9BTM9	1.026	0.037030731	1
Q9Y281	1.027	0.038436182	1
B8ZZC8	1.027	0.038436182	1
A1X283	1.028	0.039840265	1
Q9Y6V0	1.028	0.039840265	1
Q6UX04	1.029	0.041242982	1
Q96L35	1.029	0.041242982	1
A4D1E9	1.029	0.041242982	1
F8W6V6	1.029	0.041242982	1
O43390-2	1.03	0.042644337	1
Q7L5D6	1.03	0.042644337	1
Q99549	1.03	0.042644337	1
Q8WUQ7	1.03	0.042644337	1
Q86VS8	1.03	0.042644337	1
Q9P260	1.03	0.042644337	1
B7Z5R1	1.031	0.044044333	1
F8W9D1	1.031	0.044044333	1
B4DDD1	1.032	0.045442971	1
Q9NRR5	1.033	0.046840254	1
O60566	1.033	0.046840254	1
Q13459	1.033	0.046840254	1
Q9UJ70	1.033	0.046840254	1
Q68EM7	1.033	0.046840254	1
J9JIE6	1.033	0.046840254	1
O95926	1.033	0.046840254	1
C9JRJ5	1.034	0.048236186	1
Q15561	1.034	0.048236186	1
Q13572	1.034	0.048236186	1
Q9Y6W3	1.035	0.049630768	1
J3KNN5	1.036	0.051024003	1
J3KPS0	1.036	0.051024003	1
Q7L3T8	1.036	0.051024003	1
O75151	1.038	0.053806444	1
Q147X3	1.039	0.055195654	1
Q9NZ32	1.04	0.056583528	1
Q5SY16	1.04	0.056583528	1
K7EM91	1.041	0.057970069	1
H7C4I6	1.041	0.057970069	1
Q9H1E3	1.042	0.059355278	1

B4E2B6	1.045	0.063502942	1
A6NFV8	1.045	0.063502942	1
Q8NFZ8	1.046	0.064882852	1
Q14790	1.05	0.070389328	1
Q08AM6	1.051	0.071762669	1
F5H577	1.052	0.073134705	1
O60344-4	1.052	0.073134705	1
F8WEM2	1.059	0.082702589	1
