# Supplementary material for: Paladin, overexpressed in colon cancer, is required for actin polymerisation and liver metastasis dissemination
Source: Oncogenesis. 2022 Jul 26;11(1):42. doi: 10.1038/s41389-022-00416-4 (PMC9325978; doi:10.1038/s41389-022-00416-4)
Supplement: Supplementary file 8 — Supplemental table 2 [file 41389_2022_416_MOESM8_ESM.pdf]

Accession	Abundance Ratio (sh947)/(shNT)	Log FC2	padj
E9PPU0	0.01	-6.64385619	1.34E-16
Q9NPH2-2	0.01	-6.64385619	1.34E-16
Q567Q0	0.01	-6.64385619	1.34E-16
O14715	0.01	-6.64385619	1.34E-16
Q01081-2	0.01	-6.64385619	1.34E-16
E7ERK9	0.01	-6.64385619	1.34E-16
P53367-2	0.01	-6.64385619	1.34E-16
P08670	0.01	-6.64385619	1.34E-16
Q6NXG1	0.01	-6.64385619	1.34E-16
Q9Y6Q5-2	0.01	-6.64385619	1.34E-16
Q96AE7	0.01	-6.64385619	1.34E-16
Q9Y2T3	0.01	-6.64385619	1.34E-16
P84157-2	0.01	-6.64385619	1.34E-16
P27449	0.01	-6.64385619	1.34E-16
P13726	0.01	-6.64385619	1.34E-16
O95361	0.01	-6.64385619	1.34E-16
Q6N069	0.01	-6.64385619	1.34E-16
F5H0B5	0.01	-6.64385619	1.34E-16
Q8NDX5	0.01	-6.64385619	1.34E-16
Q96P47	0.01	-6.64385619	1.34E-16
Q5I2W7	0.01	-6.64385619	1.34E-16
Q969Q0	0.01	-6.64385619	1.34E-16
P12830	0.01	-6.64385619	1.34E-16
F8WBZ2	0.01	-6.64385619	1.34E-16
Q6ZMI0	0.01	-6.64385619	1.34E-16
Q6QNY1	0.01	-6.64385619	1.34E-16
Q15633	0.01	-6.64385619	1.34E-16
Q9HA77	0.01	-6.64385619	1.34E-16
P16333	0.01	-6.64385619	1.34E-16
Q6P4I2	0.01	-6.64385619	1.34E-16
Q7Z794	0.01	-6.64385619	1.34E-16
Q9NUY8	0.01	-6.64385619	1.34E-16
P02795	0.01	-6.64385619	1.34E-16
D6RF35	0.01	-6.64385619	1.34E-16
Q86Y37	0.01	-6.64385619	1.34E-16
Q05655	0.01	-6.64385619	1.34E-16
Q6Y1H2	0.01	-6.64385619	1.34E-16
Q14202	0.01	-6.64385619	1.34E-16
E9PJW9	0.01	-6.64385619	1.34E-16
Q24JP5	0.01	-6.64385619	1.34E-16
Q9Y5V3	0.01	-6.64385619	1.34E-16
Q9NQT8	0.01	-6.64385619	1.34E-16
B5MEC7	0.01	-6.64385619	1.34E-16
Q96NB2	0.01	-6.64385619	1.34E-16

Q9NRX5	0.01	-6.64385619	1.34E-16
P58340	0.01	-6.64385619	1.34E-16
E9PLL1	0.01	-6.64385619	1.34E-16
O95059	0.01	-6.64385619	1.34E-16
Q9BR61	0.01	-6.64385619	1.34E-16
C9JB30	0.01	-6.64385619	1.34E-16
E9PMT2	0.01	-6.64385619	1.34E-16
Q9NVM6	0.01	-6.64385619	1.34E-16
Q9UK53	0.01	-6.64385619	1.34E-16
E9PBR5	0.01	-6.64385619	1.34E-16
Q8N4P3	0.01	-6.64385619	1.34E-16
H0Y997	0.01	-6.64385619	1.34E-16
F8W9Y0	0.01	-6.64385619	1.34E-16
Q5VX15	0.01	-6.64385619	1.34E-16
P53801	0.01	-6.64385619	1.34E-16
B4DUS7	0.01	-6.64385619	1.34E-16
P00749	0.01	-6.64385619	1.34E-16
Q6UXD5	0.01	-6.64385619	1.34E-16
Q4G0A6	0.01	-6.64385619	1.34E-16
Q13636	0.01	-6.64385619	1.34E-16
P30414	0.01	-6.64385619	1.34E-16
Q8IZV2	0.01	-6.64385619	1.34E-16
Q9P0I2	0.01	-6.64385619	1.34E-16
O96017	0.01	-6.64385619	1.34E-16
Q13433	0.01	-6.64385619	1.34E-16
Q9NUN5	0.01	-6.64385619	1.34E-16
E2QRF9	0.01	-6.64385619	1.34E-16
E9PDK6	0.01	-6.64385619	1.34E-16
Q96RL1	0.01	-6.64385619	1.34E-16
Q96CP7	0.01	-6.64385619	1.34E-16
Q96BJ3	0.01	-6.64385619	1.34E-16
Q96DP5	0.01	-6.64385619	1.34E-16
P0C7P0	0.01	-6.64385619	1.34E-16
Q16854	0.01	-6.64385619	1.34E-16
J3KNM5	0.01	-6.64385619	1.34E-16
E9PL24	0.01	-6.64385619	1.34E-16
F8VWK8	0.01	-6.64385619	1.34E-16
E7EW84	0.01	-6.64385619	1.34E-16
J3KRV9	0.01	-6.64385619	1.34E-16
Q8N5C7	0.01	-6.64385619	1.34E-16
Q9NSA3	0.01	-6.64385619	1.34E-16
P02765	0.01	-6.64385619	1.34E-16
O60635	0.01	-6.64385619	1.34E-16
P04150	0.01	-6.64385619	1.34E-16
B1AKN6	0.01	-6.64385619	1.34E-16
F8VQE3	0.01	-6.64385619	1.34E-16

B4E3W0	0.01	-6.64385619	1.34E-16
B4DSD4	0.01	-6.64385619	1.34E-16
Q9H6F2	0.01	-6.64385619	1.34E-16
F2Z2Q4	0.01	-6.64385619	1.34E-16
E7EWX0	0.01	-6.64385619	1.34E-16
P43308	0.01	-6.64385619	1.34E-16
P62072	0.01	-6.64385619	1.34E-16
P41134	0.01	-6.64385619	1.34E-16
H0YAI4	0.01	-6.64385619	1.34E-16
Q9BYM8	0.01	-6.64385619	1.34E-16
P25686	0.01	-6.64385619	1.34E-16
Q9UGT4	0.01	-6.64385619	1.34E-16
A6NDA2	0.01	-6.64385619	1.34E-16
Q9BV68	0.01	-6.64385619	1.34E-16
Q8N490-2	0.01	-6.64385619	1.34E-16
Q86YH6	0.01	-6.64385619	1.34E-16
P03923	0.01	-6.64385619	1.34E-16
B4DDV3	0.01	-6.64385619	1.34E-16
P83111	0.01	-6.64385619	1.34E-16
Q96GE9	0.01	-6.64385619	1.34E-16
Q6P087	0.01	-6.64385619	1.34E-16
H0Y432	0.01	-6.64385619	1.34E-16
Q96E14	0.01	-6.64385619	1.34E-16
Q86YM7	0.01	-6.64385619	1.34E-16
Q14457	0.01	-6.64385619	1.34E-16
Q9BRT3	0.01	-6.64385619	1.34E-16
Q5TIH2	0.01	-6.64385619	1.34E-16
B7Z4M2	0.01	-6.64385619	1.34E-16
Q9UGU0	0.01	-6.64385619	1.34E-16
Q9BUF7	0.01	-6.64385619	1.34E-16
Q9UBT7	0.01	-6.64385619	1.34E-16
Q6ZSJ8	0.01	-6.64385619	1.34E-16
H0Y8J2	0.01	-6.64385619	1.34E-16
Q8N488	0.01	-6.64385619	1.34E-16
Q96AD5	0.01	-6.64385619	1.34E-16
Q5T5U3	0.01	-6.64385619	1.34E-16
Q8TEU8	0.01	-6.64385619	1.34E-16
Q5CZA5	0.01	-6.64385619	1.34E-16
Q13637	0.01	-6.64385619	1.34E-16
Q8IZX4	0.01	-6.64385619	1.34E-16
Q969L2	0.01	-6.64385619	1.34E-16
Q96AT1	0.01	-6.64385619	1.34E-16
C9JGQ0	0.01	-6.64385619	1.34E-16
Q9NQG1	0.01	-6.64385619	1.34E-16
E9PBZ0	0.01	-6.64385619	1.34E-16
Q16566	0.01	-6.64385619	1.34E-16

Q0P670	0.01	-6.64385619	1.34E-16
Q9HAS0	0.01	-6.64385619	1.34E-16
Q8IY63	0.01	-6.64385619	1.34E-16
Q5T764	0.01	-6.64385619	1.34E-16
Q5JXR6	0.01	-6.64385619	1.34E-16
P48436	0.01	-6.64385619	1.34E-16
Q96CB9	0.01	-6.64385619	1.34E-16
Q5T371	0.01	-6.64385619	1.34E-16
Q5JWV1	0.01	-6.64385619	1.34E-16
Q9H0F7	0.01	-6.64385619	1.34E-16
A0PJW6	0.01	-6.64385619	1.34E-16
Q8IWT6	0.01	-6.64385619	1.34E-16
Q9NPF0	0.01	-6.64385619	1.34E-16
C9J3D7	0.01	-6.64385619	1.34E-16
Q8IWT3	0.01	-6.64385619	1.34E-16
Q96SN8	0.01	-6.64385619	1.34E-16
Q9BW61	0.01	-6.64385619	1.34E-16
O75683	0.01	-6.64385619	1.34E-16
P54274	0.01	-6.64385619	1.34E-16
E7ET52	0.01	-6.64385619	1.34E-16
Q9NPG1	0.01	-6.64385619	1.34E-16
Q9ULU8	0.01	-6.64385619	1.34E-16
O94761	0.01	-6.64385619	1.34E-16
Q5VYS8	0.032	-4.965784285	1.34E-16
P07996	0.054	-4.210896782	1.34E-16
F8WC97	0.074	-3.756330919	1.34E-16
Q15149-3	0.09	-3.473931188	1.34E-16
Q86VX2	0.092	-3.442222329	1.34E-16
J3QRG5	0.096	-3.380821784	1.34E-16
P11498	0.097	-3.365871442	1.34E-16
H0Y6R1	0.107	-3.224317298	1.34E-16
P06703	0.157	-2.671163536	1.34E-16
Q16777	0.247	-2.017417053	1.34E-16
Q5MIZ7	11.005	3.46008724	1.34E-16
F8WBW2	11.142	3.477936316	1.34E-16
P98171	12.136	3.601221086	1.34E-16
E7ES96	12.818	3.68009927	1.34E-16
P50336	13.147	3.716661725	1.34E-16
P16455	13.559	3.761178876	1.34E-16
Q9BZ95	14.027	3.810134583	1.34E-16
Q5JPI3	14.071	3.814652957	1.34E-16
Q8NCF5	14.781	3.885671972	1.34E-16
Q6ISB3	15.409	3.945701333	1.34E-16
Q9UBN4	15.732	3.975630186	1.34E-16
P52306-3	100	6.64385619	1.34E-16
E9PC51	100	6.64385619	1.34E-16

E7ETY2	100	6.64385619	1.34E-16
F8W950	100	6.64385619	1.34E-16
P62745	100	6.64385619	1.34E-16
Q9NVJ2	100	6.64385619	1.34E-16
F8W9J4	100	6.64385619	1.34E-16
Q9HBI1	100	6.64385619	1.34E-16
Q8N1B4	100	6.64385619	1.34E-16
Q9NXK8	100	6.64385619	1.34E-16
A8MYB8	100	6.64385619	1.34E-16
Q9NR56-5	100	6.64385619	1.34E-16
Q13107	100	6.64385619	1.34E-16
Q9NVU0	100	6.64385619	1.34E-16
Q96JJ7	100	6.64385619	1.34E-16
Q08345	100	6.64385619	1.34E-16
Q9NS87	100	6.64385619	1.34E-16
Q96HA7	100	6.64385619	1.34E-16
Q9NZI8	100	6.64385619	1.34E-16
J3KNG9	100	6.64385619	1.34E-16
Q5T085	100	6.64385619	1.34E-16
O75781	100	6.64385619	1.34E-16
A6NF48	100	6.64385619	1.34E-16
Q5VSL9	100	6.64385619	1.34E-16
P06400	100	6.64385619	1.34E-16
K7ESQ2	100	6.64385619	1.34E-16
H3BRQ0	100	6.64385619	1.34E-16
Q86UU0	100	6.64385619	1.34E-16
Q9H6D7	100	6.64385619	1.34E-16
F8VSI7	100	6.64385619	1.34E-16
P61626	100	6.64385619	1.34E-16
Q9UK39	100	6.64385619	1.34E-16
B7Z5R6	100	6.64385619	1.34E-16
Q8TEL6	100	6.64385619	1.34E-16
Q6IQ22	100	6.64385619	1.34E-16
Q9H410	100	6.64385619	1.34E-16
P30047	100	6.64385619	1.34E-16
Q9NRD1	100	6.64385619	1.34E-16
Q8N655	100	6.64385619	1.34E-16
B0QY29	100	6.64385619	1.34E-16
Q9NRG4	100	6.64385619	1.34E-16
P29558	100	6.64385619	1.34E-16
Q9HBM1	100	6.64385619	1.34E-16
Q8IWE4	100	6.64385619	1.34E-16
O43513	100	6.64385619	1.34E-16
B3KWW1	100	6.64385619	1.34E-16
Q8IWY9	100	6.64385619	1.34E-16
J3KQE0	100	6.64385619	1.34E-16

A8DPD7	100	6.64385619	1.34E-16
Q12894	100	6.64385619	1.34E-16
Q8NAF0	100	6.64385619	1.34E-16
Q9BRV8	100	6.64385619	1.34E-16
Q8IYT2	100	6.64385619	1.34E-16
Q9Y448	100	6.64385619	1.34E-16
P47974	100	6.64385619	1.34E-16
A8K040	100	6.64385619	1.34E-16
Q6PIP6	100	6.64385619	1.34E-16
O43257	100	6.64385619	1.34E-16
P46934	100	6.64385619	1.34E-16
Q14520	100	6.64385619	1.34E-16
Q9NXH8	100	6.64385619	1.34E-16
H0YK69	100	6.64385619	1.34E-16
A6PW57	100	6.64385619	1.34E-16
Q15776	100	6.64385619	1.34E-16
G8JL95	100	6.64385619	1.34E-16
Q96RW7	100	6.64385619	1.34E-16
Q5T5L3	100	6.64385619	1.34E-16
Q9NWZ8	100	6.64385619	1.34E-16
Q63HN8	100	6.64385619	1.34E-16
J3KNQ1	100	6.64385619	1.34E-16
Q96CB8	100	6.64385619	1.34E-16
Q9Y3B1	100	6.64385619	1.34E-16
B7ZM03	100	6.64385619	1.34E-16
Q9UHV7	100	6.64385619	1.34E-16
B4DK80	100	6.64385619	1.34E-16
P18846	100	6.64385619	1.34E-16
Q9H009	100	6.64385619	1.34E-16
Q8N4Q0	100	6.64385619	1.34E-16
H7C1J4	100	6.64385619	1.34E-16
Q7L8J4	100	6.64385619	1.34E-16
E9PM05	100	6.64385619	1.34E-16
B0QY16	100	6.64385619	1.34E-16
Q9UNH6	100	6.64385619	1.34E-16
H0Y5M2	100	6.64385619	1.34E-16
Q8N0Y2	100	6.64385619	1.34E-16
O95057	100	6.64385619	1.34E-16
O00194	100	6.64385619	1.34E-16
Q3KRA9	100	6.64385619	1.34E-16
Q5JU23	100	6.64385619	1.34E-16
G3V128	100	6.64385619	1.34E-16
Q9H6X2	100	6.64385619	1.34E-16
Q96K31	100	6.64385619	1.34E-16
Q9BZD4	100	6.64385619	1.34E-16
Q86X52	100	6.64385619	1.34E-16

B4DWA3	100	6.64385619	1.34E-16
H0YDE2	100	6.64385619	1.34E-16
H3BNT3	100	6.64385619	1.34E-16
E7EQ48	100	6.64385619	1.34E-16
Q96F44-2	100	6.64385619	1.34E-16
E7EPD8	100	6.64385619	1.34E-16
P46019	100	6.64385619	1.34E-16
P28749	100	6.64385619	1.34E-16
O95704	100	6.64385619	1.34E-16
P07093-3	100	6.64385619	1.34E-16
H0Y5P8	100	6.64385619	1.34E-16
E9PAS0	100	6.64385619	1.34E-16
Q8NDT2	100	6.64385619	1.34E-16
J3KN10	100	6.64385619	1.34E-16
Q8N3J2	100	6.64385619	1.34E-16
J3QSW1	100	6.64385619	1.34E-16
Q7Z5L3	100	6.64385619	1.34E-16
B7Z7K7	100	6.64385619	1.34E-16
Q53HL2	100	6.64385619	1.34E-16
Q8WWV3	100	6.64385619	1.34E-16
Q9BV23	100	6.64385619	1.34E-16
Q53RE8	100	6.64385619	1.34E-16
K7ESE5	100	6.64385619	1.34E-16
E9PFR0	100	6.64385619	1.34E-16
E7EPI0	100	6.64385619	1.34E-16
O60427	100	6.64385619	1.34E-16
Q9BSF4	0.091	-3.457989644	5.94E-15
Q8N5A5-2	0.114	-3.13289427	1.48E-14
P35268	3.079	1.622461867	1.48E-14
Q8NI77	11.778	3.558022674	1.48E-14
Q9P0S3	11.238	3.4903134	1.77E-14
O14975	0.106	-3.23786383	2.07E-14
O14545	0.084	-3.573466862	3.24E-14
Q9Y508	9.066	3.180466161	3.82E-14
Q9ULD2	0.077	-3.698997744	4.39E-14
Q8WUF5	0.101	-3.307572802	5.26E-14
Q86W56	0.103	-3.279283757	5.54E-14
Q9NRA8	10.579	3.403131355	6.99E-14
Q8N0Y7	10.522	3.39533705	9.28E-14
Q659C4	0.101	-3.307572802	1.47E-13
P98172	10.174	3.346815094	1.61E-13
J3KPD3	10.266	3.359802261	1.76E-13
F8WBK2	11.089	3.471057365	1.78E-13
Q8IWA5	0.098	-3.351074441	2.15E-13
A6NG32	0.108	-3.210896782	3.80E-13
Q9BUZ4	0.114	-3.13289427	6.16E-13

Q9NV96	0.112	-3.158429363	6.73E-13
Q9ULJ3	10.601	3.406128457	7.41E-13
F6RY50	11.714	3.550161894	7.57E-13
Q5T447	0.089	-3.490050854	8.56E-13
Q9H8E8	11.509	3.52469058	1.14E-12
F5H5A3	8.468	3.082021269	1.52E-12
Q14BN4	9.796	3.292192774	1.65E-12
Q6P9B9	0.131	-2.932361283	1.79E-12
P16422	0.349	-1.518701058	2.09E-12
Q09472	10.175	3.346956889	2.24E-12
Q5RHS7	4.293	2.101986173	3.72E-12
Q9Y6Q5	0.275	-1.862496476	4.18E-12
Q9BZI7	10.652	3.413052429	4.92E-12
P20336	6.723	2.74910515	5.02E-12
Q8IY18	10.635	3.410748128	5.45E-12
Q08426	0.095	-3.395928676	6.08E-12
Q96E39	0.137	-2.867752202	7.00E-12
F5H7W8	0.108	-3.210896782	8.16E-12
O60930	0.112	-3.158429363	1.01E-11
B4DYH1	0.315	-1.666576266	2.35E-11
Q9Y6J0	9.769	3.288210889	3.76E-11
Q8NG68	10.11	3.337711092	3.98E-11
F8VVA0	8.234	3.041593449	4.08E-11
O95822	9.099	3.185707999	5.08E-11
P04083	0.389	-1.36215794	1.06E-10
O75420	9.268	3.212258044	1.10E-10
F5H420	7.929	2.987138926	2.32E-10
P07339	2.553	1.352193538	2.99E-10
P57735	0.232	-2.10780329	8.37E-10
Q8N8U9	0.151	-2.727379545	9.21E-10
O95838	0.224	-2.158429363	1.15E-09
B5ME49	0.122	-3.035046947	1.33E-09
E9PBM2	0.121	-3.046921047	1.51E-09
Q9H8P0	0.122	-3.035046947	2.91E-09
C9JZY6	2.918	1.544979883	2.91E-09
P09455	2.723	1.445196982	5.10E-09
P47895	0.302	-1.727379545	8.06E-09
Q9BWD1	2.305	1.204766751	3.31E-08
Q8TD30	3.073	1.619647767	3.70E-08
Q02410	0.275	-1.862496476	1.27E-07
P27338	0.414	-1.272297327	1.27E-07
P01008	0.304	-1.717856771	4.52E-07
Q562R1	0.416	-1.265344567	4.56E-07
O75368	0.409	-1.289827252	5.77E-07
B4DFR2	3.233	1.692873507	5.81E-07
E9PNS7	0.319	-1.648371671	6.98E-07

Q6DD88	2.731	1.449429314	7.04E-07
Q01085	0.308	-1.698997744	1.09E-06
P29317	2.11	1.077242999	1.29E-06
P33947	0.384	-1.380821784	1.54E-06
P15559	0.484	-1.046921047	1.58E-06
C9JZN9	4.106	2.037733627	1.59E-06
Q9UH17	3.127	1.644779219	2.14E-06
O00515	0.41	-1.286304185	2.66E-06
P00414	0.299	-1.74178261	3.07E-06
G3V1J9	0.225	-2.152003093	3.62E-06
P13611	2.406	1.266636643	3.85E-06
Q96KB5	2.327	1.218471211	4.04E-06
G3V5J8	0.189	-2.40354186	5.48E-06
P36952	0.451	-1.148800661	1.08E-05
Q5T2T1	2.313	1.209765266	1.43E-05
Q5JP53	1.972	0.979659552	1.61E-05
P14625	0.516	-0.954557029	1.76E-05
O75629	0.354	-1.498178735	1.90E-05
O14950	0.334	-1.582079992	1.96E-05
P35527	0.425	-1.234465254	2.85E-05
E9PCI9	1.986	0.989865623	3.34E-05
Q12907	0.526	-0.926865295	3.70E-05
P00734	0.454	-1.139235797	3.83E-05
Q9NZ08	0.403	-1.311148256	4.08E-05
O94964	0.31	-1.689659879	4.13E-05
D3DS54	2.506	1.325386415	6.31E-05
Q9UJC3	0.367	-1.446148032	6.59E-05
E7ETA7	0.46	-1.120294234	7.34E-05
Q4KWH8	0.473	-1.080087911	7.38E-05
P23381	0.541	-0.886299501	8.53E-05
C9JYQ9	0.541	-0.886299501	8.75E-05
O95782	0.525	-0.929610672	8.82E-05
Q9NRR5	0.47	-1.089267338	9.36E-05
F5H7R9	0.526	-0.926865295	9.96E-05
H7C061	2.121	1.084744621	0.00010274
P50453	1.884	0.913798965	0.00010417
P48735	1.858	0.893750502	0.00011992
Q8NCN5	0.366	-1.450084446	0.00018993
A8MUB1	0.555	-0.849440323	0.00020127
Q86Y79	0.378	-1.40354186	0.0002013
P16949	1.82	0.86393845	0.0002319
P11137	0.312	-1.680382066	0.00024367
E9PCP3	0.315	-1.666576266	0.00027614
P11021	0.562	-0.831357964	0.00029338
P05161	1.966	0.975263322	0.00031192
P14618-2	0.458	-1.126580497	0.00032917

P17931	0.564	-0.826232932	0.00033283
P46013	1.797	0.845590409	0.00034326
P55072	1.796	0.84478735	0.00035063
Q14517	0.567	-0.81857936	0.00037877
P02649	0.448	-1.158429363	0.00038758
P51610	3.258	1.703986604	0.00041426
P26583	1.785	0.835924074	0.00042135
Q9BXS5	1.971	0.978927776	0.00045394
P31947	0.57	-0.810966176	0.00046282
Q8NFJ5	0.413	-1.275786313	0.00050497
Q14012	2.798	1.484395963	0.00060505
Q9Y5I4	0.516	-0.954557029	0.00066918
Q9HDC9	0.579	-0.788364747	0.0007211
O60828	2.439	1.286289758	0.00073298
P63096	2.184	1.126972856	0.00074891
Q9BQ69	0.512	-0.965784285	0.00081718
Q01433	0.523	-0.935117148	0.00082704
Q14696	0.524	-0.932361283	0.00088266
Q53T59	2.425	1.277984747	0.00089663
Q10471	0.418	-1.258425153	0.00093175
P84077	0.434	-1.204233052	0.00094933
P09972	1.736	0.795766948	0.00096373
E5RJV5	2.721	1.444136957	0.00100951
Q8IVT2	0.425	-1.234465254	0.00103135
P13674	0.538	-0.894321922	0.00110744
J3KTE9	0.501	-0.997117491	0.00122044
Q96HC4	1.716	0.779049553	0.00133569
P11388	1.835	0.875780063	0.00133875
P31949	0.574	-0.800877358	0.00157787
Q8TCC3	0.372	-1.426625474	0.00165155
Q13885	1.703	0.768078435	0.00165733
P60981	0.598	-0.74178261	0.00181341
Q9Y624	0.531	-0.913216234	0.0019463
O00264	1.755	0.811471031	0.00199457
E9PEY4	0.479	-1.061902439	0.00208617
Q9H0W8	2.334	1.222804561	0.00238264
Q66K74	1.957	0.968643756	0.00251681
Q96QZ7	0.344	-1.53951953	0.00256355
Q9BRL6	0.607	-0.720231578	0.00306257
P52943	2.326	1.217851097	0.00306327
P36404	1.977	0.983312871	0.0032156
Q96QD8	0.591	-0.758769964	0.00327606
Q9NQ34	2.638	1.399444565	0.00346277
P04818	1.815	0.859969548	0.00353553
Q6XZF7	1.943	0.958285901	0.0036924
P43304	1.647	0.719840555	0.00405721

P29034	2.212	1.145351386	0.00419633
B4DQH9	2.604	1.380729449	0.00423696
P53602	1.847	0.885183866	0.00467748
Q16822	0.569	-0.813499442	0.0046918
P60903	1.757	0.813114191	0.00492308
Q9Y5A9	0.572	-0.805912948	0.00502756
O94907	1.829	0.871055075	0.00517734
P33316	1.792	0.841570637	0.00534939
P56962	0.325	-1.621488377	0.00545297
H0YGS7	2.845	1.508428653	0.00545359
P27797	0.626	-0.675765438	0.00595123
A0MZ66	0.577	-0.793356776	0.00600224
P13667	0.626	-0.675765438	0.00604223
P35221	0.627	-0.673462652	0.00628709
P04350	1.619	0.695102986	0.00628709
P11926	0.402	-1.314732593	0.00635665
J3KNQ4	1.819	0.863145543	0.00636972
Q96GA7	0.562	-0.831357964	0.00664247
Q15742	0.378	-1.40354186	0.00674313
Q5T0Y2	0.565	-0.823677227	0.00674313
Q8N7B9	0.525	-0.929610672	0.00684615
Q6UXH1	0.501	-0.997117491	0.00724484
Q8TEM1	1.646	0.718964336	0.00761361
Q14764	1.647	0.719840555	0.00789774
Q2M389	0.634	-0.657445255	0.00815402
G8JLJ4	2.7	1.432959407	0.00950941
Q58FF6	1.622	0.69777382	0.00963001
E9PK73	0.434	-1.204233052	0.00971012
P55809	0.639	-0.646112164	0.0099598
E9PHN7	2.559	1.355580147	0.01006634
Q7L0Y3	0.625	-0.678071905	0.01032628
P04632	1.585	0.66448284	0.01044812
Q9H4M9	1.584	0.663572335	0.01058487
Q15058	2.804	1.487486349	0.0107524
B4DGT8	0.483	-1.049904906	0.01080423
P08107	0.642	-0.639354798	0.01088691
Q16880	0.436	-1.19759996	0.01089013
Q9H1Y0	1.775	0.827819025	0.01094679
Q9H7D0	0.398	-1.329159664	0.01155969
K4DID8	1.944	0.959028219	0.01164339
P08243	0.644	-0.634867407	0.01204879
F5H6D0	0.598	-0.74178261	0.01255462
Q9BZK7	0.646	-0.63039393	0.01259864
Q92844	2.511	1.328262029	0.01261623
Q86W92-2	1.571	0.651683181	0.01295605
O00762	1.716	0.779049553	0.01413955

P50225	0.574	-0.800877358	0.01418531
Q9NZZ3	1.868	0.901494455	0.01418531
P04792	0.65	-0.621488377	0.01471955
Q86XZ4	1.806	0.852797893	0.01473566
Q2PZI1	1.947	0.961252884	0.01481363
Q9P246	2.307	1.206018004	0.01557145
Q15847	0.463	-1.110915901	0.01578452
P69905	1.555	0.63691458	0.01604475
Q15021	1.653	0.725086725	0.01612358
I3L4X2	1.807	0.853596506	0.01644132
Q8IWF9	0.572	-0.805912948	0.01693258
Q96C24	0.513	-0.962969269	0.01776596
P52306	1.551	0.633198686	0.01777839
Q9UBS4	0.651	-0.619270551	0.01787993
O94855	0.598	-0.74178261	0.01836709
P48163	0.645	-0.632628934	0.01863214
P98179	1.545	0.627606838	0.01877085
B7ZBM3	0.368	-1.442222329	0.01894858
Q9NQS1	2.2	1.137503524	0.01894858
Q9UGI8	0.658	-0.603840511	0.01903355
A6NDG6	1.666	0.736388401	0.01993666
K7EM91	0.539	-0.891642822	0.02092974
Q8IXB1	0.52	-0.943416472	0.02113123
Q9UHB6-4	0.612	-0.708396442	0.02113123
C9JK10	1.7	0.765534746	0.02113123
Q9NP81	1.7	0.765534746	0.0214444
Q8NBJ4	0.619	-0.691988685	0.02169969
E9PL57	1.782	0.833497337	0.0223069
F8W9D1	1.883	0.913033	0.02262909
Q9Y6J9	2.513	1.329410671	0.0227283
P39748	1.531	0.614474283	0.02274158
Q15262	1.674	0.743299528	0.02318891
Q02241	1.771	0.824564212	0.0233728
O43278	0.596	-0.746615764	0.0237195
P21912	0.619	-0.691988685	0.0238075
Q9NV70	1.663	0.733788169	0.02392782
F5H233	2.378	1.249748715	0.02416292
E9PQN9	0.594	-0.751465164	0.02485898
K7EKI8	0.569	-0.813499442	0.02545326
K7ERL7	0.611	-0.710755715	0.02565412
P54105	1.64	0.713695815	0.02732271
O15067	1.517	0.601221086	0.02752839
P21583	0.509	-0.974262439	0.02779884
F8WDH1	0.512	-0.965784285	0.02840413
Q02952	0.671	-0.575615328	0.02942559
Q9BTW9	1.605	0.682573297	0.02947195

Q8N8N7	0.503	-0.991369695	0.03004231
Q9HD20	0.55	-0.862496476	0.03132796
Q14011	1.584	0.663572335	0.03183879
Q99720	0.605	-0.724992953	0.03200974
Q86U90	0.613	-0.706041021	0.0326219
Q99661	1.827	0.869476634	0.03357262
Q02252	0.534	-0.905088353	0.03435348
P08727	0.677	-0.562772261	0.03443146
Q9Y4L1	0.677	-0.562772261	0.0344617
P80303	0.635	-0.655171503	0.03508468
O14964	0.632	-0.662003536	0.03509819
P31350	0.677	-0.562772261	0.03519393
B4E3K2	2.204	1.140124224	0.03566394
Q32P28	1.718	0.780730036	0.0357966
P16144-2	1.53	0.613531653	0.03601464
P16070-4	1.616	0.692427198	0.03601464
O95239	1.587	0.666302128	0.0366709
B4DTU4	1.66	0.731183242	0.03685901
Q96JM7	1.947	0.961252884	0.03737305
E9PBJ5	0.68	-0.556393349	0.03835895
Q5ZPR3	1.714	0.777367109	0.03889164
P23921	1.491	0.576280258	0.03920193
Q16555	1.489	0.574343754	0.04040111
O75323	0.633	-0.659722595	0.04209932
P05787	0.684	-0.54793177	0.04320069
Q14247	0.685	-0.545824107	0.04442771
B3KPJ4	1.649	0.721591399	0.04502423
F8W9E7	0.542	-0.883635243	0.04534638
P58107	0.686	-0.543719518	0.04561834
P05496	0.469	-1.092340172	0.04682124
Q16643	1.477	0.562669826	0.0473247
P55210	0.623	-0.682695932	0.04783293
F5H538	2.044	1.031395196	0.04832755
P41214	0.634	-0.657445255	0.0492258
P26232	0.589	-0.763660461	0.05207812
Q8N4E4	0.478	-1.064917477	0.05377831
Q68D86	0.414	-1.272297327	0.05425839
O00273-2	2.301	1.202260984	0.05485101
E9PNK6	0.587	-0.768567592	0.05546011
Q8TCG1	1.922	0.942608336	0.05586139
Q9NPQ8	1.89	0.918386234	0.05731881
Q15404	1.521	0.605020153	0.05833725
Q9BYN0	0.613	-0.706041021	0.05884968
Q04206	1.604	0.681674142	0.05907913
Q5XKP0	1.805	0.851998837	0.05913446
P56945	1.953	0.965691949	0.05936025

B5MCQ5	0.696	-0.522840789	0.0598196
P15924	0.696	-0.522840789	0.05997337
Q9HCY8	0.648	-0.625934282	0.06047259
Q96EZ8	2.153	1.10634832	0.06166191
F8VW92	1.454	0.540027269	0.06335731
Q16881	0.665	-0.588573754	0.06410925
Q92820	1.46	0.545968369	0.06525562
E7EQ34	0.611	-0.710755715	0.06639098
P56134	0.57	-0.810966176	0.06669995
F5H1X8	0.7	-0.514573173	0.06714148
P17096-2	0.652	-0.61705613	0.06773258
E7ESP4	1.901	0.926758532	0.06793916
Q13126	0.702	-0.510457064	0.07067227
I3L291	0.508	-0.977099598	0.07167064
P30043	0.672	-0.573466862	0.07179768
P08574	0.703	-0.508403406	0.07182335
Q9UHL4	0.526	-0.926865295	0.0726802
Q6UW68	0.653	-0.614845103	0.0726802
Q9Y446	0.657	-0.606034724	0.07297495
Q92625	0.502	-0.994240731	0.07432058
Q02127	0.645	-0.632628934	0.07448643
Q9Y5Y5	0.497	-1.008682243	0.07477614
F5H569	0.641	-0.641603738	0.07530257
Q9Y5X1	0.661	-0.597277823	0.0761608
E5RH51	0.484	-1.046921047	0.07627291
P17096	0.706	-0.502259911	0.07627291
Q16658	1.438	0.524063676	0.07739475
H3BTY2	0.659	-0.60164963	0.07824034
G3V1X9	1.47	0.555816155	0.0783317
P46109	1.507	0.591679417	0.07913464
B8ZZQ6	0.708	-0.498178735	0.08004645
P05783	0.708	-0.498178735	0.08024529
P84157	1.716	0.779049553	0.08069987
Q96IU4	0.665	-0.588573754	0.08073062
P42126	0.696	-0.522840789	0.08073062
P78318	1.56	0.641546029	0.08098498
Q9NWS0	1.869	0.902266569	0.0813667
Q9P1F3	0.662	-0.595096878	0.08237897
Q5T280	1.806	0.852797893	0.0825951
Q13263	1.431	0.517023672	0.08331298
Q8WX93	0.69	-0.535331733	0.08343981
Q5VWJ9	0.49	-1.029146346	0.08388385
H7C089	2.171	1.118359726	0.08480988
P52292	1.43	0.516015147	0.0848336
O60271	0.663	-0.592919225	0.08546961
P42167	1.452	0.538041453	0.08546961

P42696	1.866	0.899948986	0.08546961
F5GX70	0.463	-1.110915901	0.08559081
Q9BT73	1.823	0.866314561	0.08559081
E7EMP6	0.66	-0.59946207	0.08608516
P00966	0.612	-0.708396442	0.08663256
Q9Y281	1.56	0.641546029	0.08878917
P30101	0.712	-0.490050854	0.08881704
Q9UPN3-5	2.108	1.075874867	0.08884504
P17858	0.7	-0.514573173	0.08979559
Q7L5Y1	0.557	-0.844250767	0.09387745
J3QRN6	0.617	-0.696657606	0.09387745
Q9Y4P3	1.596	0.674460652	0.09451834
Q16850	1.497	0.582074221	0.09491955
P06493	1.42	0.50589093	0.09522944
Q8TB36	0.584	-0.775959726	0.0957977
P17066	0.666	-0.586405918	0.09608992
Q01650	0.683	-0.550042516	0.09608992
F8WEE8	1.85	0.887525271	0.09608992
P35580	0.716	-0.481968507	0.09735585
Q8NF64	2.004	1.002882509	0.09801635
Q9Y2D4	2.154	1.10701825	0.09821588
Q13740	0.635	-0.655171503	0.09872267
P17987	1.416	0.501821265	0.09942745
Q9Y6V0	0.482	-1.052894948	0.09958831
Q8NBY1	0.591	-0.758769964	0.10019228
Q9BQL6	0.671	-0.575615328	0.10277743
H7BZJ3	0.719	-0.475936324	0.10376432
P68371	1.412	0.497740089	0.10387472
F8WES2	0.622	-0.685013515	0.10444202
F5GYQ1	0.666	-0.586405918	0.10492587
Q9BUF5	1.409	0.494671612	0.10879469
Q00341	1.409	0.494671612	0.10910094
P10412	1.408	0.493647334	0.10912425
Q96HY6	0.67	-0.577766999	0.11069542
Q14676	1.515	0.599317794	0.1109792
H3BUV4	0.598	-0.74178261	0.11116684
Q8WW22	0.685	-0.545824107	0.11116684
Q6YHK3	1.59	0.669026766	0.11181484
Q01167	1.889	0.917622702	0.11264552
Q15738	1.486	0.571434116	0.11309614
Q9Y4F1	1.982	0.986956963	0.11346101
Q96HV5	0.557	-0.844250767	0.11410206
Q92896	1.403	0.488515009	0.11528624
Q9Y4Y9	1.62	0.695993813	0.11569028
Q14320	1.512	0.59645814	0.11596882
O95299	0.704	-0.506352666	0.11602073

Q8WWH5	0.694	-0.526992432	0.11631736
P43357	0.599	-0.739372092	0.11750339
Q56VL3	0.685	-0.545824107	0.11776079
P54840	0.676	-0.564904848	0.11830144
H0YK48	0.631	-0.66428809	0.12102534
F5GZS6	0.726	-0.461958547	0.12164938
P63261	1.398	0.483364361	0.12180532
Q8TBB5	1.873	0.9053509	0.12180532
O60518	2.014	1.010063683	0.1226781
P29590	1.971	0.978927776	0.12517169
H0YGW8	0.679	-0.55851652	0.12570094
P29353	1.956	0.96790637	0.12647537
Q02790	0.728	-0.457989644	0.12718462
Q16763	1.464	0.549915554	0.12788608
Q9BX40	1.565	0.646162657	0.12838468
Q9C0B0	0.531	-0.913216234	0.12958229
Q86SQ0	1.532	0.615416297	0.12966489
Q9Y547	1.515	0.599317794	0.13004034
O14841	1.947	0.961252884	0.13127096
P11441	1.661	0.732052073	0.1314013
Q15437	0.705	-0.504304837	0.13192999
Q9NVI1	1.515	0.599317794	0.13196379
Q9H0H5	1.572	0.652601218	0.13286717
O15234	0.601	-0.734563104	0.13363138
P35222	0.73	-0.454031631	0.13363581
Q86UW9	2.122	1.085424656	0.13363581
F8WDL1	2.074	1.052415894	0.13431006
B4DR61	0.705	-0.504304837	0.13621213
Q9UI12	0.685	-0.545824107	0.13672829
B1AKJ5	1.444	0.530070742	0.13672829
E9PIE3	1.591	0.669933836	0.13686409
Q9H0U3	0.684	-0.54793177	0.13774693
P52789	0.705	-0.504304837	0.13792946
Q2NKX8	1.474	0.559736524	0.13872355
P49005	1.621	0.696884091	0.13938928
B4DUT8	1.429	0.515005916	0.14032288
Q9BQ70	0.716	-0.481968507	0.1403482
Q13257	1.503	0.587845009	0.14181517
O75817	1.799	0.847195187	0.14220707
P18206	1.384	0.468843943	0.14350313
P53985	1.455	0.541019153	0.14375626
P61960	0.697	-0.520769439	0.14421986
O00767	1.698	0.763836459	0.14548494
B4DGP8	0.734	-0.446148032	0.14580027
Q15155	0.502	-0.994240731	0.14690028
O00425	0.718	-0.477944251	0.14740109

Q93052	0.697	-0.520769439	0.14872251
I3L213	0.479	-1.061902439	0.14938042
P0CG30	0.651	-0.619270551	0.14938042
C9IYK6	0.638	-0.648371671	0.1496818
Q53GA4	0.687	-0.541617996	0.1497914
C9JIM0	1.782	0.833497337	0.14985178
P67936-2	1.919	0.940354712	0.15124736
Q9H813	2.017	1.012211084	0.15211401
F5H5D3	1.378	0.462575888	0.15298198
Q96Q42	1.947	0.961252884	0.15301341
F5GY99	1.576	0.656267535	0.15337364
Q9P0J1	1.425	0.510961919	0.15360919
Q8IWX8	0.712	-0.490050854	0.15398496
Q6NZI2	1.419	0.504874589	0.15573675
P12931	1.706	0.770617647	0.15573675
F8W6V6	0.572	-0.805912948	0.1568638
Q00765	0.696	-0.522840789	0.15692826
Q9HCE5	2.024	1.01720929	0.15861065
O60313-2	0.72	-0.473931188	0.16121593
Q9UL40	2.023	1.01649632	0.16131218
Q13546	1.623	0.698663	0.1615589
P50897	1.556	0.63784206	0.16220837
B4E040	0.708	-0.498178735	0.16284314
Q53GS7	1.859	0.89452677	0.16294049
Q9Y696	1.524	0.607862903	0.16394717
Q13451	0.725	-0.4639471	0.16505059
Q6IN84	0.694	-0.526992432	0.16591518
Q9ULW0	1.505	0.589763487	0.16650213
Q9UIL1	0.587	-0.768567592	0.16749516
P04264	0.667	-0.584241333	0.16749516
O43520	0.694	-0.526992432	0.16854353
Q9Y2C4	1.971	0.978927776	0.16862614
Q96PD2	1.459	0.544979883	0.16919821
Q8TCD5	1.532	0.615416297	0.16919821
F5H865	1.651	0.72334012	0.16919821
Q7Z4H3	0.702	-0.510457064	0.16927932
Q8N5K1	1.483	0.568518598	0.17403585
P08758	1.366	0.449957484	0.17446874
P26440	0.705	-0.504304837	0.17691675
P07954	0.744	-0.426625474	0.1772185
O43175	0.744	-0.426625474	0.17783166
P54577	0.744	-0.426625474	0.1783038
Q96H79	1.596	0.674460652	0.17848686
Q15643	0.54	-0.888968688	0.17941125
Q9NYU2	0.745	-0.424687669	0.18033307
H0YAH3	1.873	0.9053509	0.18140481

O14908	1.467	0.552868871	0.18172503
B7Z2S9	1.673	0.742437445	0.1832725
G3V167	1.902	0.927517246	0.18416248
Q8WVI0	0.576	-0.795859283	0.1844117
Q7L7V1	0.633	-0.659722595	0.1844117
O00154-4	1.36	0.443606651	0.18485432
B4DJW3	1.736	0.795766948	0.18485432
Q9NVQ4	1.861	0.896078055	0.18485432
H3BUN4	0.654	-0.612637459	0.18516955
O95347	1.465	0.550900665	0.18641522
Q99698	0.504	-0.988504361	0.18680043
Q9NVS9	0.731	-0.452056689	0.18726559
Q9NR12	1.463	0.548929769	0.18904483
Q9UKX7	1.422	0.507921465	0.19036406
Q99959	0.545	-0.875671865	0.19375702
Q7Z406-6	0.742	-0.430508908	0.19377633
Q9P2S5	0.576	-0.795859283	0.19387402
F5GXY5	1.356	0.439357178	0.19437478
P62875	1.585	0.66448284	0.19787887
F8W9X7	0.523	-0.935117148	0.19836047
Q93075	0.679	-0.55851652	0.19836047
P43490	0.75	-0.415037499	0.19887526
Q9GZP4	1.518	0.602171791	0.19900647
Q9UBD5	0.57	-0.810966176	0.19912849
Q14254	1.469	0.554834396	0.19936437
E9PGM1	1.353	0.436161839	0.19944867
H0Y861	0.562	-0.831357964	0.20014989
P49841	1.665	0.735522177	0.20014989
P60763	1.714	0.777367109	0.20015561
F8WEK6	1.796	0.84478735	0.20116287
H7BXH2	1.406	0.491596594	0.20164954
P40123	1.608	0.685267407	0.20164954
Q71DI3	0.751	-0.413115187	0.20213572
Q9NPH2	0.751	-0.413115187	0.20311477
P84095	1.445	0.531069493	0.20329483
Q13426	1.452	0.538041453	0.20329483
Q9HCJ0	1.877	0.90842865	0.20329483
P62937	1.35	0.432959407	0.20399385
Q12933	1.895	0.922197848	0.20399703
H3BTK5	1.932	0.950095094	0.20564392
P48960	0.597	-0.744197163	0.20573066
Q96KP4	0.753	-0.40927823	0.20675066
H0Y8C3	0.661	-0.597277823	0.20734943
P04080	1.348	0.430820497	0.20765411
Q9UBF2	1.503	0.587845009	0.20909365
Q9UQR0	1.843	0.882056071	0.20996299

Q96GM5	1.626	0.701327257	0.2104834
Q15366	1.657	0.728573603	0.21054122
Q96CW1	0.72	-0.473931188	0.21057924
Q9Y3C4	1.663	0.733788169	0.21091113
Q9Y490	1.346	0.42867841	0.2118163
Q9UKB1	0.589	-0.763660461	0.212989
O60812	1.872	0.904580435	0.21424656
O14907	1.501	0.585923977	0.21691907
Q9BS26	0.742	-0.430508908	0.21739848
Q9BPX3	1.413	0.498761466	0.22328232
O60443	1.694	0.760433875	0.22328232
P36959	1.86	0.895302621	0.22328232
O43657	0.655	-0.610433188	0.22354761
E7EN46	1.907	0.931304844	0.22400743
Q13011	1.343	0.425459305	0.22436338
P61764	1.491	0.576280258	0.22477174
C9JK39	1.575	0.655351829	0.22595843
Q9GZQ3	0.607	-0.720231578	0.22627354
O60927	0.626	-0.675765438	0.22772863
Q99808	1.428	0.513995979	0.23001163
P19174	1.555	0.63691458	0.23046171
P49588	0.759	-0.397828209	0.23094466
Q0VDF9	1.489	0.574343754	0.23104748
P22830	0.724	-0.465938398	0.23380767
Q96N66	0.717	-0.479954976	0.23454137
P54819	0.76	-0.395928676	0.23454137
O76070	0.613	-0.706041021	0.23561729
Q9UKK3	0.721	-0.471928835	0.23722281
P07099	1.355	0.438292852	0.23792182
Q13907	1.391	0.47612242	0.23819433
P40763	0.724	-0.465938398	0.2384619
Q14008	1.334	0.415758667	0.23881919
P84243	1.334	0.415758667	0.23927159
Q9GZZ9	0.734	-0.446148032	0.23977302
Q9UII2	0.647	-0.628162383	0.24000494
P43121	0.659	-0.60164963	0.24007949
D6RDI8	0.523	-0.935117148	0.24170683
E9PPW4	0.57	-0.810966176	0.24170683
P42166	1.333	0.41467678	0.24170683
Q9Y6N5	1.434	0.520045024	0.24170683
Q9NNW5	1.646	0.718964336	0.24170683
Q14558	1.397	0.482332021	0.24173977
Q8N335	0.663	-0.592919225	0.24222176
P60468	0.682	-0.552156356	0.24403476
P12235	0.736	-0.442222329	0.24587327
P14927	0.739	-0.436353731	0.24866581

Q16774	0.576	-0.795859283	0.25024781
F8W7Q4	0.73	-0.454031631	0.25025407
Q12789	0.729	-0.45600928	0.25103225
Q9H008	1.503	0.587845009	0.25270855
Q8IW45	0.611	-0.710755715	0.25353254
P08582	0.524	-0.932361283	0.25359596
O95757	0.737	-0.440263476	0.25463527
P19971	0.702	-0.510457064	0.25485617
Q15181	0.765	-0.386468347	0.25485617
E7EN19	1.327	0.408168371	0.25485617
Q9NQY0	1.65	0.722466024	0.25485617
B4DRL5	1.783	0.834306703	0.25485617
Q8IV36	0.578	-0.790858602	0.25513637
Q96HP0	0.594	-0.751465164	0.25543196
Q6ZMG9	0.678	-0.560642822	0.2555966
Q7Z6J9	1.861	0.896078055	0.25607677
O95671	1.422	0.507921465	0.25620799
Q99805	1.385	0.469885976	0.25642256
Q8TDD1-2	0.526	-0.926865295	0.25662472
Q71U36	1.326	0.407080775	0.25669583
P78540	0.603	-0.729770093	0.25902945
E5RIL0	0.591	-0.758769964	0.26009385
Q70UQ0	1.82	0.86393845	0.26009385
Q13042	0.681	-0.554273297	0.2606183
O75937	1.389	0.474046599	0.26133981
Q9BRS2	0.723	-0.467932448	0.2614642
Q9NTX5	0.735	-0.444183845	0.26374918
O00170	1.388	0.473007568	0.26409738
Q15800	1.585	0.66448284	0.26475823
O76041-2	1.705	0.769771739	0.2658377
O95684	1.653	0.725086725	0.26640698
Q96CX6	1.845	0.883620816	0.26734803
P12270	1.321	0.401630467	0.26752441
P36639	1.591	0.669933836	0.26752441
P26358	1.321	0.401630467	0.2677987
J3KMZ7	0.68	-0.556393349	0.26861502
Q92890-1	1.358	0.44148348	0.26901332
A1L0T0	1.392	0.477159211	0.26985067
P63241	1.32	0.40053793	0.27018528
P21964	0.769	-0.378944497	0.27019687
Q4G0P3	0.589	-0.763660461	0.27174075
P20020	0.73	-0.454031631	0.27289216
Q9Y6K5	0.732	-0.450084446	0.27332654
Q9Y5M8	0.757	-0.401634795	0.27332654
Q9UHG3	1.383	0.467801156	0.27332654
Q96GW9	1.852	0.889084099	0.27332654

Q9H3K2	0.706	-0.502259911	0.27438941
P11172	1.357	0.440420721	0.27562271
P21589	0.709	-0.496142467	0.27585758
P38571	1.781	0.832687516	0.27586443
Q13541	0.668	-0.582079992	0.27634603
P13645	0.671	-0.575615328	0.27727621
Q9Y2Y0	1.459	0.544979883	0.27910125
Q86UY8	1.527	0.610700062	0.27931486
Q08752	0.771	-0.375197235	0.27939752
Q96AB3	0.723	-0.467932448	0.28013318
P21695	0.746	-0.422752464	0.28124627
Q8WZ82	1.611	0.687956494	0.28279838
Q6ZNB6	0.726	-0.461958547	0.28286988
P53004	1.369	0.453122447	0.28286988
Q8NHH9	0.711	-0.492078535	0.28312276
P51153	1.412	0.497740089	0.28312276
Q01813	0.772	-0.373327247	0.28416091
P58317	0.547	-0.870387262	0.28439637
K7EJV3	0.6	-0.736965594	0.28439637
O43414	1.562	0.643394453	0.28523523
P17301	1.405	0.49057013	0.28577043
P46199	0.717	-0.479954976	0.28647024
Q6I9Y2	0.66	-0.59946207	0.28719561
P21291	1.371	0.455228571	0.28736993
P52788	0.747	-0.420819852	0.28738117
B4DT70	1.629	0.703986604	0.287661
Q8NFW1	1.491	0.576280258	0.28783405
O60343	0.744	-0.426625474	0.28934347
Q96BK5	0.731	-0.452056689	0.29082542
P05023	0.774	-0.369594529	0.29082542
Q8N5M1	0.737	-0.440263476	0.29161872
P09622	0.774	-0.369594529	0.29175783
Q8N183	0.736	-0.442222329	0.29282049
P11216	0.774	-0.369594529	0.29282049
Q14353	1.394	0.479230561	0.29397899
C9JRL4	1.396	0.481298942	0.29498521
Q99584	1.362	0.445726703	0.29502109
Q9H2H9	0.678	-0.560642822	0.29530251
H7C1N3	0.705	-0.504304837	0.29530251
Q96S97	0.73	-0.454031631	0.29530251
Q15392	1.36	0.443606651	0.29530251
Q92783	0.692	-0.531156057	0.29800199
Q9HCN8	0.744	-0.426625474	0.29800199
K7EK56	0.639	-0.646112164	0.29905987
P82909	0.711	-0.492078535	0.29905987
P35908	0.578	-0.790858602	0.29971183

Q9BWH6	1.582	0.6617496	0.29971183
Q5TFQ8	0.607	-0.720231578	0.30093393
Q13813	1.307	0.386259141	0.30135193
Q9BUR4	1.593	0.671746267	0.30186354
O75390	0.777	-0.364013496	0.30251497
Q9ULC3	1.62	0.695993813	0.30294903
Q5W0V3	1.593	0.671746267	0.30449063
P02768	1.306	0.385154897	0.30450818
F8WAJ0	0.689	-0.537424112	0.30524261
Q8TB52	1.614	0.690640579	0.30524261
O15297	1.797	0.845590409	0.30524261
E9PI90	1.402	0.487486349	0.30584954
P62873	1.305	0.384049807	0.30629823
B4DXZ6	1.36	0.443606651	0.30689952
Q6ZN57	0.547	-0.870387262	0.30741656
Q99470	0.683	-0.550042516	0.30741656
P30038	0.687	-0.541617996	0.30741656
Q9UJU6	0.746	-0.422752464	0.30741656
O75223	0.751	-0.413115187	0.30741656
Q8TCT9	0.755	-0.40545145	0.30741656
Q9UHX1-5	1.304	0.38294387	0.30741656
Q9H0R4	1.624	0.699551633	0.30741656
Q9UHN1	1.665	0.735522177	0.30802937
P85037	0.679	-0.55851652	0.30835309
Q14192	0.74	-0.434402824	0.30920536
Q7Z434	0.743	-0.428565884	0.30988882
O94925-3	1.377	0.461528559	0.31104965
P53350	1.364	0.447843644	0.31215092
Q9BPZ3	1.391	0.47612242	0.31346923
Q96AC1	1.359	0.442545456	0.31373119
P63313	1.302	0.380729449	0.31475536
Q15691	1.301	0.379620962	0.31585795
Q9Y2V7	0.609	-0.715485867	0.31611116
P55209	1.301	0.379620962	0.31692519
E7ER89	1.479	0.564622052	0.31692519
Q9H204	1.681	0.749319725	0.31692519
P62195	1.301	0.379620962	0.31700788
B4E351	0.675	-0.567040593	0.31706438
Q15043	1.43	0.516015147	0.31801615
Q12981-1	0.643	-0.637109357	0.31841586
Q8N9T8	1.435	0.521050737	0.31860992
C9JPG4	0.604	-0.727379545	0.31884874
P15291	0.624	-0.680382066	0.31984099
Q14181	1.549	0.631337144	0.31984099
Q5SW79	1.613	0.689746438	0.32009518
Q9Y6Y0	0.676	-0.564904848	0.32275778

O00178	1.434	0.520045024	0.32291778
Q9NR45	0.782	-0.354759487	0.32357136
Q9ULK4	0.751	-0.413115187	0.3245867
P62942	0.782	-0.354759487	0.3245867
Q9Y2V2	1.419	0.504874589	0.32468459
D3YHP0	0.68	-0.556393349	0.32484531
Q9BUL8	1.389	0.474046599	0.3256146
B4DVJ1	1.771	0.824564212	0.32600235
Q5TDC5	1.771	0.824564212	0.32600235
E7ENY0	0.722	-0.469929258	0.32822896
Q09666	0.783	-0.352915787	0.32822896
Q8NFQ8	1.406	0.491596594	0.32870678
F8WCY5	0.771	-0.375197235	0.32920767
Q9GZL7	0.783	-0.352915787	0.32920853
Q01518	1.351	0.434027675	0.33084028
Q00796	0.784	-0.351074441	0.33086501
Q53H82	0.751	-0.413115187	0.33117046
P31946	0.784	-0.351074441	0.3314795
H7BYT1	1.422	0.507921465	0.33215309
Q15334	1.691	0.75787666	0.33321957
O75431	0.753	-0.40927823	0.33369311
P06132	1.388	0.473007568	0.33422795
Q8N2F6	0.701	-0.512513651	0.33492202
Q13951-2	1.429	0.515005916	0.33580899
P18754	1.293	0.370722275	0.33684179
P36551	0.785	-0.349235441	0.33697292
D6RAK3	0.621	-0.687334826	0.3384356
P09497	0.705	-0.504304837	0.3384356
P53680	0.74	-0.434402824	0.3384356
Q13283	1.292	0.36960607	0.3384356
P51151	1.627	0.702214251	0.3384356
Q92696	1.375	0.459431619	0.34045652
Q6PI48	0.754	-0.407363571	0.34151809
P61313	0.786	-0.347398782	0.34193586
Q5BJF6	0.558	-0.841662973	0.34281393
Q12979-2	0.627	-0.673462652	0.34281393
Q9H173	0.645	-0.632628934	0.34281393
Q92526	1.311	0.390667686	0.34470356
Q9NXH9	0.747	-0.420819852	0.34593117
Q4G176	0.766	-0.384583703	0.34621096
Q9P086	1.744	0.80240004	0.34741198
Q7KZF4	0.787	-0.345564459	0.3486506
E9PQP6	1.392	0.477159211	0.34914144
O75880	0.632	-0.662003536	0.34915175
P16403	1.289	0.366252264	0.34915175
I3L1I3	1.599	0.677169939	0.34915175

P62306	0.758	-0.399730246	0.3501068
P63167	1.338	0.420078116	0.35013299
Q96I15	0.755	-0.40545145	0.35027972
Q99536	1.288	0.365132593	0.35027972
Q15366-6	1.288	0.365132593	0.35083618
O75083	1.287	0.364012054	0.35211549
Q9NTI5	1.385	0.469885976	0.35211549
O15357	1.474	0.559736524	0.35275862
P03915	0.762	-0.392137097	0.35279352
Q8TAP9	1.702	0.767231037	0.35279352
Q6IBS0	1.305	0.384049807	0.35375867
Q5VZZ6	1.728	0.789103218	0.35399772
P45877	0.733	-0.448114897	0.35569973
D6RJH6	0.611	-0.710755715	0.35647458
J3KQ48	1.329	0.410341105	0.35647458
Q9BY32	1.337	0.418999465	0.35657486
Q9BUA3	0.644	-0.634867407	0.35761226
Q9NZL4	1.356	0.439357178	0.35761226
Q01469	0.79	-0.340075442	0.35781214
O75131	1.285	0.361768359	0.3583967
Q9Y2B0	0.769	-0.378944497	0.36044701
Q8IYE0	0.692	-0.531156057	0.3615215
Q9BX46	1.674	0.743299528	0.3615215
E9PHA2	1.486	0.571434116	0.36159488
Q9H6F5	1.366	0.449957484	0.36161531
P46060	1.284	0.360645202	0.36247667
Q8IWE2	1.348	0.430820497	0.36294213
Q9NZT2	1.356	0.439357178	0.36294213
O75600	1.477	0.562669826	0.36294213
P12004	1.283	0.35952117	0.36355694
K7EPC1	0.776	-0.365871442	0.36531025
Q92995	0.688	-0.53951953	0.36577536
H3BSW0	0.714	-0.486004021	0.36593654
Q9Y244	1.373	0.457331625	0.36593654
Q8IUR0	1.667	0.737254104	0.36636643
Q09013-1	0.663	-0.592919225	0.36637669
Q9BTM9	0.712	-0.490050854	0.36637669
E7EWI9	1.348	0.430820497	0.36637669
Q3SXM5	1.419	0.504874589	0.36637669
H7BXS8	1.667	0.737254104	0.36637669
O94973	1.321	0.401630467	0.36643165
K7ERC8	1.528	0.611644543	0.36682212
Q9H446	0.662	-0.595096878	0.36743457
P48165	0.668	-0.582079992	0.3675386
Q8WY36	0.661	-0.597277823	0.36785121
Q96JH7	1.357	0.440420721	0.36839892

Q8IWB7	0.695	-0.524915117	0.36922725
Q96ME7	0.761	-0.394031641	0.37014122
H7BXI1	1.357	0.440420721	0.37070663
G3V3N4	0.644	-0.634867407	0.37179579
I3L3Y8	1.412	0.497740089	0.37310674
Q96JB5	0.753	-0.40927823	0.37331239
P53384	0.765	-0.386468347	0.37336644
Q9ULE4	0.706	-0.502259911	0.37394785
P15954	0.772	-0.373327247	0.37394785
Q9NUP9	1.468	0.553851968	0.37427651
Q658P3	1.544	0.626672753	0.37587027
Q6P3R8	0.69	-0.535331733	0.37626172
P39880	0.744	-0.426625474	0.37632134
P49459	1.453	0.539034703	0.37668178
Q7Z2K6	0.62	-0.689659879	0.37818096
P49419-2	0.795	-0.330973234	0.37818096
Q9UHR4	0.74	-0.434402824	0.37830399
P0CG13	1.559	0.640620928	0.38017477
O94915	0.735	-0.444183845	0.38048633
P56937	1.676	0.745022149	0.38106072
Q9Y4R8	1.367	0.451013243	0.38112629
Q96AE4	1.486	0.571434116	0.38161495
Q5T1B0	0.629	-0.668868078	0.38295376
O94874	0.771	-0.375197235	0.38295376
P54920	1.357	0.440420721	0.38305605
Q01082	1.276	0.351628329	0.38308772
J3KQN4	0.768	-0.380821784	0.3834031
Q96L92	1.388	0.473007568	0.3834031
P51636	0.711	-0.492078535	0.38451999
O15127	0.767	-0.382701517	0.38514232
A9UJQ0	1.607	0.684369929	0.38682294
Q9BXV9	0.742	-0.430508908	0.38746554
G3V599	0.767	-0.382701517	0.38755311
Q6RW13	0.762	-0.392137097	0.38931584
Q6ZMU5	0.755	-0.40545145	0.39021916
P62070	0.766	-0.384583703	0.39021916
P31942	1.302	0.380729449	0.39142362
Q13501	0.776	-0.365871442	0.39315525
Q9H9B4	0.798	-0.325539348	0.39315525
P10636-4	1.547	0.629473197	0.3941748
A8MWR6	0.717	-0.479954976	0.3948608
O15460	0.766	-0.384583703	0.39503013
P11279	0.77	-0.377069649	0.39506278
P67870	0.798	-0.325539348	0.39598259
Q8TDQ7	0.725	-0.4639471	0.39627775
P36871	0.798	-0.325539348	0.3968326

E9PMR6	0.642	-0.639354798	0.39698827
O14929	1.308	0.387362541	0.39711937
P56211	1.488	0.573374526	0.39736006
Q9HD45	0.775	-0.367731785	0.39785223
Q92900	1.271	0.34596403	0.39817675
Q7Z5K2	1.505	0.589763487	0.39825023
O14773	1.339	0.421155961	0.39858685
P24752	1.271	0.34596403	0.39913543
O60701	0.799	-0.323732592	0.39923264
Q9NUD5	0.673	-0.57132159	0.39962232
Q9BTE6	0.769	-0.378944497	0.40147934
J3KMZ8	1.375	0.459431619	0.40147934
Q13641	1.682	0.750177706	0.40159365
B4DV38	0.715	-0.483984853	0.40180066
E9PE48	0.724	-0.465938398	0.40225365
P18077	0.8	-0.321928095	0.40225365
F5GYA4	1.62	0.695993813	0.40225365
Q13625	0.764	-0.388355457	0.40365727
Q6ZN84	1.602	0.679874148	0.40365727
O75607	0.796	-0.329159664	0.40375053
D4QA03	1.309	0.388465097	0.40375053
E9PE51	1.496	0.581110175	0.40375053
H0YC74	1.683	0.751035177	0.40428166
Q96HQ2	1.358	0.44148348	0.40472315
P61956	0.8	-0.321928095	0.40489951
B7Z4R0	1.548	0.630405471	0.40761939
Q9UFW8	1.53	0.613531653	0.4089692
Q9Y6N3	0.718	-0.477944251	0.41120698
B4DEM7	1.363	0.446785562	0.41199316
Q5BKX8	1.615	0.691534165	0.41359689
P42224	1.266	0.340277405	0.41365847
O60232	0.769	-0.378944497	0.41389922
Q9NTJ3	1.323	0.403813062	0.41389922
Q15366-3	1.349	0.431890348	0.41389922
Q13561	1.267	0.341416524	0.41536131
O43488	1.304	0.38294387	0.41536131
Q86TS9	0.708	-0.498178735	0.41569502
F5GXE4	0.728	-0.457989644	0.41569502
Q1KMD3	1.265	0.339137385	0.41569502
O75391	0.716	-0.481968507	0.41577445
Q9BW71	1.494	0.579180148	0.41609517
P23634	0.645	-0.632628934	0.41641745
P08238	1.265	0.339137385	0.41641745
O43251-8	1.48	0.565597176	0.41641745
P51788	0.587	-0.768567592	0.41665509
Q9BVR6	1.684	0.751892138	0.41688745

Q8TB70	1.522	0.605968359	0.4174791
Q7L5N7	0.673	-0.57132159	0.41768152
Q9NVM9	0.728	-0.457989644	0.41768152
O43447	1.388	0.473007568	0.41832398
H0YNG3	1.338	0.420078116	0.41901563
Q9BYV8	1.7	0.765534746	0.41901563
Q04446	0.778	-0.36215794	0.41915469
O15269	1.274	0.349365278	0.41915469
I3L425	1.642	0.715454127	0.41915469
J3KPM8	0.773	-0.371459681	0.42057531
Q08170	1.565	0.646162657	0.42057531
C9JZG2	0.696	-0.522840789	0.42133756
Q9UDY2	0.729	-0.45600928	0.42133756
Q6UW56	0.637	-0.650634722	0.42188202
Q9H832	0.777	-0.364013496	0.42467065
P48506	0.801	-0.320125852	0.42467065
Q9P2R3	1.384	0.468843943	0.42467065
Q9UHK0	0.634	-0.657445255	0.42559602
Q9H0D6	1.261	0.334568276	0.42585184
Q9Y5K5	0.656	-0.60823228	0.42654888
Q8WVM0	1.456	0.542010356	0.42700615
Q9UBM7	1.306	0.385154897	0.42730296
D6RED8	0.723	-0.467932448	0.42735965
Q9Y673	0.781	-0.356605547	0.42735965
F5H7S9	1.524	0.607862903	0.42883708
Q9P0P8	1.612	0.688851744	0.42948752
Q86UE8	1.512	0.59645814	0.42961958
F8W9W2	0.713	-0.488026018	0.42967455
O00330	0.769	-0.378944497	0.43091879
Q9P2X0	0.72	-0.473931188	0.43095549
Q9C0D9	0.732	-0.450084446	0.43115917
P41236	0.785	-0.349235441	0.43115917
A6NKV8	1.486	0.571434116	0.43183644
P15529-2	0.638	-0.648371671	0.4321913
D6REA0	0.664	-0.590744853	0.4321913
Q9HBH5	0.69	-0.535331733	0.4321913
P23284	0.806	-0.311148256	0.4321913
O15182	1.514	0.598365205	0.4340526
Q96I59	0.765	-0.386468347	0.43531343
C9JZR2	0.807	-0.309359421	0.43552387
P15121	1.266	0.340277405	0.43552387
O60493	0.775	-0.367731785	0.43656603
P11766	1.257	0.32998465	0.43656603
B1AVU8	1.257	0.32998465	0.43656603
Q9HAT2	0.756	-0.40354186	0.43743439
Q15629	0.778	-0.36215794	0.43810496

Q9Y5Q9	0.785	-0.349235441	0.43810496
I3L2C7	1.274	0.349365278	0.43819217
K7EIU8	1.371	0.455228571	0.43819217
Q8ND04	1.536	0.619178216	0.43819217
P23368	0.808	-0.307572802	0.43854697
Q9NP72	1.256	0.328836464	0.43896232
Q13201	0.726	-0.461958547	0.43964147
P40227	1.256	0.328836464	0.43964147
Q9BRT2	1.467	0.552868871	0.43964147
H3BTN8	0.718	-0.477944251	0.43973709
P24390	0.783	-0.352915787	0.43976593
Q92945	1.256	0.328836464	0.44038277
P00491	0.808	-0.307572802	0.44064539
Q9Y6E0	1.301	0.379620962	0.44064539
O00622	0.776	-0.365871442	0.44159549
P07686	1.256	0.328836464	0.4425962
Q7Z7N9	0.67	-0.577766999	0.44264335
Q96R06	1.643	0.71633248	0.44304217
Q9Y2W1	0.809	-0.305788392	0.44340554
Q16513	1.335	0.416839742	0.44421655
Q13188	1.463	0.548929769	0.4456282
Q8NFU3	0.738	-0.438307279	0.44607284
A4D1E9	0.763	-0.390245038	0.44607284
E9PS76	0.776	-0.365871442	0.44607284
O95336	0.81	-0.304006187	0.44607284
P52565	1.253	0.325386415	0.44607284
P17812	1.254	0.326537348	0.44607284
P47755	1.274	0.349365278	0.44607284
Q9HBU6	1.298	0.376290383	0.44607284
P14635	1.333	0.41467678	0.44607284
Q96GA3	1.335	0.416839742	0.44607284
Q8WY22	1.463	0.548929769	0.44607284
Q9BSY9	1.523	0.606915942	0.44607284
Q9BRX5	1.57	0.650764559	0.44607284
B7Z9K1	1.673	0.742437445	0.44607284
Q8IVD9	1.496	0.581110175	0.44773634
Q9NU22	0.779	-0.360304767	0.44781013
O43598	0.783	-0.352915787	0.44781013
Q5JRX3	0.788	-0.343732465	0.44865344
Q9H9F9	1.513	0.597411988	0.44915935
E7EQC1	1.265	0.339137385	0.4501991
Q7Z3B4	1.314	0.393965276	0.4501991
P34913	0.73	-0.454031631	0.45059882
P62861	1.265	0.339137385	0.45059882
Q8IVP5	0.653	-0.614845103	0.45090202
Q969E2	0.733	-0.448114897	0.45182862

Q07065	1.251	0.32308179	0.45182862
P09543	1.288	0.365132593	0.45182862
J3KT75	1.383	0.467801156	0.45182862
Q6UW78	0.721	-0.471928835	0.45306085
Q969S9	0.734	-0.446148032	0.45306085
Q99643	0.789	-0.341902795	0.45306085
P56182	1.31	0.389566812	0.45306085
Q92989	1.53	0.613531653	0.45306085
P11182	0.719	-0.475936324	0.45325078
Q96PV6	1.586	0.665392771	0.45325078
E9PMR4	1.433	0.51903861	0.45380392
Q9BV57	1.301	0.379620962	0.45432915
Q5TD07	1.442	0.528071165	0.45475646
G5E9Z2	0.766	-0.384583703	0.45577865
F8WJN3	1.269	0.343692069	0.45607161
O95229	0.723	-0.467932448	0.45610871
P49368	1.249	0.320773477	0.45629771
P19823	1.516	0.600269754	0.45778911
Q13459	1.534	0.617298483	0.45791305
Q9UEU0	0.65	-0.621488377	0.45862207
P61513	0.796	-0.329159664	0.45958008
F5H5E2	1.438	0.524063676	0.459587
Q9ULM6	1.593	0.671746267	0.459587
C9JCC6	1.304	0.38294387	0.4598194
O00499	1.465	0.550900665	0.4598194
Q8N573	0.606	-0.722610301	0.4605143
Q15293	0.814	-0.2968993	0.4611769
Q8TAT6	1.247	0.318461465	0.4611769
Q13003	1.463	0.548929769	0.46137044
P35237	1.247	0.318461465	0.46222982
Q9P2W9	0.783	-0.352915787	0.46239636
P37198	1.309	0.388465097	0.46295394
P00374	1.325	0.40599236	0.46514178
F5H5P2	0.736	-0.442222329	0.4654693
Q8WW12	0.781	-0.356605547	0.46572115
P29350	0.789	-0.341902795	0.46572115
Q6ZRS2	0.794	-0.332789088	0.46572115
Q12905	1.245	0.316145742	0.46572115
P07195	1.246	0.317304068	0.46572115
Q9NXR1	1.574	0.654435541	0.46572115
K7EN78	0.661	-0.597277823	0.46614184
O75348	0.795	-0.330973234	0.46614184
Q6ZRV2	1.429	0.515005916	0.46672672
Q9Y546	1.613	0.689746438	0.46720887
P41240	1.36	0.443606651	0.46769043
Q5VT66	0.735	-0.444183845	0.46820427

C9JAW5	0.789	-0.341902795	0.46907988
P13798	1.244	0.314986485	0.46907988
Q5T4U5	0.8	-0.321928095	0.46966076
A2A274	0.805	-0.312939312	0.46969503
Q15149	0.816	-0.293358943	0.46969503
P46926	0.816	-0.293358943	0.46969503
Q07666	1.244	0.314986485	0.46969503
C9JVP0	1.618	0.694211608	0.46969503
P78381	0.657	-0.606034724	0.47039586
H0YGR4	0.791	-0.3382504	0.47142598
Q9H3P2	1.286	0.362890643	0.47156287
P16930	0.792	-0.336427665	0.47302075
P28676	0.694	-0.526992432	0.4734547
P17174	0.817	-0.291592017	0.47431726
E9PRV2	0.711	-0.492078535	0.47445647
P08779	1.597	0.675364313	0.47449941
Q14118	1.373	0.457331625	0.47463159
P48200	1.61	0.687060688	0.474711
Q9UM07	1.29	0.367371066	0.47514381
O15031	1.377	0.461528559	0.47526827
P28370	1.459	0.544979883	0.47645522
Q99595	0.759	-0.397828209	0.47721766
Q13557	0.789	-0.341902795	0.47721766
P52735	1.386	0.470927257	0.47721766
E9PBE5	1.441	0.527070336	0.47762492
Q96K17	1.316	0.396159489	0.4776857
F5H8H2	1.489	0.574343754	0.47836956
Q9NXS2	0.653	-0.614845103	0.47844275
P04183	1.307	0.386259141	0.47915825
P10636-5	1.318	0.39835037	0.47915825
Q8NEW0	0.744	-0.426625474	0.47933529
G3V3H3	1.241	0.311503115	0.47933529
J3KSL8	0.796	-0.329159664	0.47959072
Q5GLZ8	0.77	-0.377069649	0.48086412
Q16864	0.791	-0.3382504	0.4820605
C9JRZ6	0.793	-0.334607229	0.48206966
P23528	1.24	0.310340121	0.48211727
E9PNM1	1.338	0.420078116	0.4825967
Q5JSL0	1.4	0.485426827	0.4825967
P22670	1.577	0.65718266	0.4829086
Q5VWU8	0.627	-0.673462652	0.48316925
Q6NUS6	0.659	-0.60164963	0.48316925
K7EMU1	0.787	-0.345564459	0.48316925
Q8NEL9	1.354	0.437227739	0.48316925
P53611	1.434	0.520045024	0.48316925
O75179	1.441	0.527070336	0.48316925

P18074	1.28	0.35614381	0.4838108
O15260	0.799	-0.323732592	0.48431439
Q96PU8	1.549	0.631337144	0.48431439
Q7Z3C6	1.477	0.562669826	0.4843896
Q15642	1.345	0.427606173	0.48479596
Q9UH65	0.707	-0.50021788	0.48486426
P23508	0.752	-0.411195433	0.48486426
P55769	0.82	-0.286304185	0.48486426
Q9Y2L1	0.816	-0.293358943	0.48530091
P33908	0.69	-0.535331733	0.48827449
P61163	1.237	0.3068455	0.488755
P09496-2	0.805	-0.312939312	0.4889503
P31321	1.577	0.65718266	0.48950131
A1A4S6	1.411	0.496717988	0.4899455
P19525	1.237	0.3068455	0.49190111
E9PQ56	1.319	0.399444565	0.49374694
O43815	1.457	0.543000877	0.49395716
O95219	1.391	0.47612242	0.49503862
P22059	0.803	-0.316528107	0.49548515
Q9UBE0	0.822	-0.282789701	0.49548515
P19021	1.494	0.579180148	0.49548515
P52758	1.279	0.355016264	0.49789961
Q29963	0.67	-0.577766999	0.49879757
P26196	1.235	0.304511042	0.49879757
Q6R327	0.635	-0.655171503	0.49914352
Q13416	0.656	-0.60823228	0.49914352
Q9Y316	1.289	0.366252264	0.49914352
Q9NX61	1.498	0.583037624	0.49914352
F8W938	0.671	-0.575615328	0.50044749
Q9Y530	0.704	-0.506352666	0.50044749
O00217	0.797	-0.327348371	0.50044749
O14744	0.823	-0.281035664	0.50044749
O60361	0.823	-0.281035664	0.50044749
A8MXV4	1.442	0.528071165	0.50044749
Q5JXI8	1.552	0.634128558	0.50044749
Q92973-2	1.233	0.3021728	0.50088026
Q96DV4	0.801	-0.320125852	0.5013766
P35244	1.238	0.308011315	0.50149701
Q13308	1.264	0.337996464	0.50198598
O15400	0.801	-0.320125852	0.50295013
P02786	0.824	-0.279283757	0.50295013
Q9H467	1.559	0.640620928	0.50368226
Q96ER3	1.327	0.408168371	0.50651612
Q8TDN6	1.232	0.301002256	0.50779687
E9PHY5	1.239	0.309176187	0.50920507
B9A041	1.253	0.325386415	0.50927032

Q9P2B7	0.639	-0.646112164	0.50955202
Q9Y2Z9	0.76	-0.395928676	0.50955202
P27144	0.801	-0.320125852	0.50955202
Q14019	0.825	-0.277533976	0.50955202
Q99614	1.285	0.361768359	0.50955202
Q9BRP4	1.304	0.38294387	0.50955202
Q9UP95	1.572	0.652601218	0.50955202
F5GXJ0	1.58	0.659924558	0.50955202
Q6XQN6	1.344	0.426533138	0.50968901
P61009	1.376	0.46048047	0.51035887
J3KPV3	0.745	-0.424687669	0.5106061
Q9C005	1.268	0.342554745	0.51239131
P06396	1.277	0.352758525	0.51255694
Q14694	1.23	0.298658316	0.51306447
Q69YU5	0.639	-0.646112164	0.51331478
D6R9A1	1.558	0.639695233	0.51367482
Q6P3W7	0.802	-0.318325858	0.51373572
Q9GZY8	0.762	-0.392137097	0.51436807
Q9NXX6	0.688	-0.53951953	0.51458854
Q14527	0.761	-0.394031641	0.51458854
Q9NX40	0.804	-0.314732593	0.51458854
J3QRV5	0.806	-0.311148256	0.51458854
Q9H000	1.463	0.548929769	0.51458854
Q13825	0.753	-0.40927823	0.51499087
J3QL71	0.69	-0.535331733	0.51503521
Q99961	1.286	0.362890643	0.51503521
P04062	1.401	0.486456956	0.51550379
B4E2W0	0.826	-0.275786313	0.51566407
O43676	0.805	-0.312939312	0.51571905
Q9BU89	1.333	0.41467678	0.51571905
Q02218	0.801	-0.320125852	0.51626126
Q9H5V8	0.806	-0.311148256	0.51701045
Q9NRV9	0.778	-0.36215794	0.51727904
Q5JTH9	0.826	-0.275786313	0.51735355
P61225	0.804	-0.314732593	0.51774281
P47756-2	1.228	0.296310561	0.51841541
Q9NVS2	0.79	-0.340075442	0.51875519
Q66K14	0.692	-0.531156057	0.51988838
Q8NBJ7	0.798	-0.325539348	0.51991102
Q96EH3	0.675	-0.567040593	0.52037843
P17568	0.805	-0.312939312	0.5205337
Q6GMV2	1.398	0.483364361	0.52082933
P49366	0.811	-0.30222618	0.52111319
Q8NBL1	1.427	0.512985335	0.52183962
Q9Y6X4	0.769	-0.378944497	0.52444256
P52732	1.377	0.461528559	0.52444256

Q9BRP8	1.335	0.416839742	0.52549067
Q9NWU5	0.807	-0.309359421	0.52573383
Q00610	0.828	-0.272297327	0.52573383
P07108	1.226	0.293958979	0.52573383
Q9Y478	1.437	0.523060062	0.52573383
Q96EY8	1.502	0.586884813	0.52573383
Q6ZN50	0.702	-0.510457064	0.52574444
Q96KP1	1.427	0.512985335	0.52574444
Q9NPA8	1.413	0.498761466	0.52628227
Q86UA1	0.753	-0.40927823	0.526849
Q96DG6	0.817	-0.291592017	0.52704283
Q9NVR2	1.499	0.584000383	0.52739182
Q9H116	0.627	-0.673462652	0.52776793
K7ERA3	0.719	-0.475936324	0.5278408
P22061	1.259	0.332278283	0.52785909
A6NHS7	0.8	-0.321928095	0.52823241
Q9H5N1	1.385	0.469885976	0.52843614
P49959	1.475	0.560714954	0.52851716
Q969G6	0.785	-0.349235441	0.52971662
P67809	1.225	0.292781749	0.52978539
Q9UK22	0.805	-0.312939312	0.53001671
Q86TI2	0.811	-0.30222618	0.53001671
O95298	0.799	-0.323732592	0.53008109
Q96HY7	0.741	-0.432454552	0.53059159
E9PJK4	0.682	-0.552156356	0.53062688
Q6NTF9	0.748	-0.418889825	0.53062688
Q96GD0	0.772	-0.373327247	0.53062688
A2ABB9	0.784	-0.351074441	0.53062688
P53999	0.823	-0.281035664	0.53062688
E9PI68	1.285	0.361768359	0.53062688
Q15796	1.45	0.5360529	0.53062688
Q53EL6	1.512	0.59645814	0.53062688
B0UX83	1.254	0.326537348	0.53141904
O60504	1.431	0.517023672	0.53376249
H7C3Y7	0.666	-0.586405918	0.53480847
Q8TBQ9	0.77	-0.377069649	0.53480847
F8W6X5	0.793	-0.334607229	0.53480847
O76075	0.768	-0.380821784	0.5349666
Q6UWP7	1.43	0.516015147	0.53560089
F6U6P3	0.639	-0.646112164	0.53580006
P49711	1.385	0.469885976	0.53580006
O43920	0.804	-0.314732593	0.53585024
P28838	0.831	-0.267079618	0.53620664
P63010	0.831	-0.267079618	0.53640769
Q9NUL3	1.409	0.494671612	0.53640769
P23919	1.263	0.336854639	0.53654752

H0Y990	1.447	0.533064922	0.53703846
P54709	0.831	-0.267079618	0.53712696
Q9GZN1	0.687	-0.541617996	0.53987507
O43818	0.82	-0.286304185	0.53987507
Q13617	1.259	0.332278283	0.54018564
Q8NDD1	0.738	-0.438307279	0.54047355
Q15382	1.312	0.39176772	0.54047355
Q8NEZ2	1.469	0.554834396	0.54047355
O60831	0.773	-0.371459681	0.54139876
Q14241	0.739	-0.436353731	0.54141899
P49773	0.832	-0.265344567	0.54165318
O00399	1.467	0.552868871	0.542136
O00193	1.279	0.355016264	0.54382337
J3KMW7	1.339	0.421155961	0.54397831
Q6NSJ5	1.51	0.59454855	0.54470885
P23786	0.807	-0.309359421	0.54479974
Q96FN4	1.468	0.553851968	0.54481953
P55263	0.82	-0.286304185	0.54508289
P29083	1.335	0.416839742	0.54611548
P46100	0.805	-0.312939312	0.54643922
Q9NRK6	1.425	0.510961919	0.54690899
Q9HBR0	0.695	-0.524915117	0.54729141
Q8IYU8	1.441	0.527070336	0.5484562
Q86UX6	1.507	0.591679417	0.5484562
Q9Y4C8	1.371	0.455228571	0.5490285
Q9P032	0.771	-0.375197235	0.54908256
Q68D91	0.695	-0.524915117	0.54937426
Q8WUK0	0.756	-0.40354186	0.54937426
O43181	0.812	-0.300448367	0.54937591
B8ZZ54	0.811	-0.30222618	0.55267892
Q9Y5P6	0.813	-0.298672743	0.55279095
Q53H96	0.806	-0.311148256	0.55325302
J3QT22	1.237	0.3068455	0.55325302
Q8NBJ5	1.267	0.341416524	0.55420383
O00442	0.806	-0.311148256	0.55540934
P26885	0.819	-0.288064643	0.55706137
P37802	0.835	-0.260151897	0.5581942
P02545-2	1.281	0.357270476	0.55852576
Q9UBQ7	0.816	-0.293358943	0.55853344
Q9H1E5	1.526	0.609754962	0.55853344
Q9NYP7	0.8	-0.321928095	0.55860552
P02794	0.807	-0.309359421	0.5594412
P03897	0.767	-0.382701517	0.56191741
A8MPT4	0.776	-0.365871442	0.56218412
Q9BUR5	0.813	-0.298672743	0.56218412
Q14165	0.823	-0.281035664	0.56218412

O94826	0.835	-0.260151897	0.56218412
Q8WWM7	1.237	0.3068455	0.56218412
Q2NL82	0.831	-0.267079618	0.56243059
B7Z9G5	1.286	0.362890643	0.56244023
P16278	1.321	0.401630467	0.56313071
D6RD44	0.693	-0.529072743	0.56451568
B1B0M1	0.768	-0.380821784	0.56451568
Q7L523	1.395	0.480265122	0.56451568
Q5QPD4	1.5	0.584962501	0.56451568
Q9GZR2-2	1.391	0.47612242	0.56469312
Q96FK6	1.496	0.581110175	0.56473149
Q9UEW8	0.78	-0.358453971	0.56489236
Q4L180	0.663	-0.592919225	0.564927
O00400	0.757	-0.401634795	0.56505947
Q14061	0.804	-0.314732593	0.56541474
E9PFT6	1.242	0.312665174	0.56573854
Q7Z4H8	1.346	0.42867841	0.56632571
Q5T0N5	1.42	0.50589093	0.56632571
O14497	1.287	0.364012054	0.56807302
Q6NUQ4	1.364	0.447843644	0.5696449
F8W8Z9	0.818	-0.289827252	0.56985458
Q16656	1.451	0.537047519	0.57028524
P14174	1.213	0.27857955	0.57062289
O95297	1.434	0.520045024	0.57081963
B4DKY1	0.825	-0.277533976	0.57148538
Q13620-1	1.212	0.277389699	0.57148538
P36915	0.821	-0.284545873	0.57172405
P00156	0.782	-0.354759487	0.57186125
P35613	1.212	0.277389699	0.57186125
P28340	1.36	0.443606651	0.57190628
P26447	0.837	-0.256700472	0.5719337
P27708	1.212	0.277389699	0.5720661
Q13057	0.805	-0.312939312	0.57250611
Q9NQ92	1.491	0.576280258	0.57295156
E9PEN8	1.295	0.372952098	0.57310733
Q96RD7	1.422	0.507921465	0.57310733
Q969U7	1.28	0.35614381	0.57320452
Q9H2C0	1.36	0.443606651	0.57321438
P23470	1.523	0.606915942	0.57437305
P17028	0.747	-0.420819852	0.57482862
D6RIZ4	1.419	0.504874589	0.57482862
Q9Y4E8	0.812	-0.300448367	0.57515888
Q9Y289	1.376	0.46048047	0.57552231
Q92667	1.447	0.533064922	0.57589748
P15311	0.838	-0.254977851	0.5771162
O75616	1.369	0.453122447	0.5771162

Q6PKC0	1.384	0.468843943	0.57768854
B4DDV1	0.775	-0.367731785	0.579513
P10809	0.839	-0.253257284	0.579513
P15927	1.22	0.286881148	0.57957374
B4DY26	0.686	-0.543719518	0.5807825
O60832	0.834	-0.261880711	0.58164177
K7ERE1	1.364	0.447843644	0.5820399
B4DDD1	0.784	-0.351074441	0.58204799
O60488	0.822	-0.282789701	0.58231
Q13616	1.22	0.286881148	0.58231
Q99988	1.371	0.455228571	0.58231
Q6NZY4	1.493	0.578214165	0.58231
Q07960	0.817	-0.291592017	0.58255552
Q9NTM9	1.277	0.352758525	0.58310475
Q9BV40	0.8	-0.321928095	0.58352199
B2RD65	1.324	0.404903122	0.58353713
Q9NRN7	1.268	0.342554745	0.58418279
P03905	0.826	-0.275786313	0.58459646
P56537	1.208	0.272620455	0.58475122
Q99797	0.824	-0.279283757	0.58513992
Q8NDC0	1.356	0.439357178	0.58513992
P17535	1.491	0.576280258	0.58513992
G3V1P0	1.5	0.584962501	0.58513992
Q6YN16	1.257	0.32998465	0.58525812
P55011	1.29	0.367371066	0.58525812
Q6NT55	1.495	0.580145484	0.58525812
P40189	0.715	-0.483984853	0.58551066
Q9Y5P4	0.785	-0.349235441	0.58611779
P27694	1.208	0.272620455	0.58656379
O15347	1.212	0.277389699	0.58753517
Q9HCE9	1.439	0.525066592	0.58804664
Q96BP2	0.804	-0.314732593	0.58948349
Q9Y6I3	1.481	0.566571641	0.58979776
H0YCN2	0.656	-0.60823228	0.58990159
C9JFR7	0.841	-0.249822294	0.59007186
Q5JSH3	0.821	-0.284545873	0.59032875
P14406	0.833	-0.263611599	0.59032875
P00568	1.207	0.271425676	0.59032875
E5RFN5	0.656	-0.60823228	0.59099874
B7Z3P4	1.473	0.55875743	0.59112087
Q7Z7K6	1.365	0.448900951	0.59203768
Q9BVC3	0.72	-0.473931188	0.59255847
Q96S55	0.816	-0.293358943	0.59255847
Q9BQA1	0.818	-0.289827252	0.59255847
Q9P275	0.779	-0.360304767	0.59256911
Q9H1A4	0.817	-0.291592017	0.59256911

P61758	0.823	-0.281035664	0.59256911
Q9Y617	1.206	0.270229907	0.59256911
O14757	1.379	0.463622457	0.59256911
P60059	0.824	-0.279283757	0.59265028
Q08380	0.826	-0.275786313	0.59283211
Q9H8S9	1.24	0.310340121	0.59389784
Q9BZF1	0.828	-0.272297327	0.59460855
E7EU96	0.842	-0.248107862	0.59484231
Q96AG4	0.842	-0.248107862	0.59484231
P62913	0.842	-0.248107862	0.59484231
Q9Y3Y2	1.267	0.341416524	0.59491742
Q8IU81	1.404	0.489542936	0.59532305
P45985	1.299	0.377401431	0.59554421
P18084	1.364	0.447843644	0.5957956
P39687	0.843	-0.246395464	0.59620618
P43378	1.507	0.591679417	0.59620618
Q9Y3L3	0.78	-0.358453971	0.59625448
Q6P161	0.791	-0.3382504	0.59663623
Q15311	0.726	-0.461958547	0.5969595
P24941	1.291	0.368489001	0.59718425
Q9UEG4	0.705	-0.504304837	0.59758598
Q9P013	0.732	-0.450084446	0.59758598
Q14697	0.829	-0.270555993	0.59865569
P49441	0.776	-0.365871442	0.59865801
E9PSI1	0.795	-0.330973234	0.5986918
P62273	0.825	-0.277533976	0.5986968
F5GWD3	0.739	-0.436353731	0.59922755
E9PFH8	0.843	-0.246395464	0.59954354
Q9H1D9	1.411	0.496717988	0.6001421
P31943	1.203	0.266636643	0.60022813
O14787-2	1.268	0.342554745	0.60103279
Q9ULC4	1.243	0.313826296	0.60150422
Q99832	1.203	0.266636643	0.60201023
O60344-4	0.718	-0.477944251	0.60307992
Q9H944	0.805	-0.312939312	0.60307992
Q86Y39	0.814	-0.2968993	0.60307992
Q9Y487	0.823	-0.281035664	0.60307992
Q15833	0.827	-0.274040765	0.60307992
Q8TAE8	0.829	-0.270555993	0.60307992
Q15428	1.24	0.310340121	0.60307992
P07910	1.354	0.437227739	0.60307992
Q9Y232	1.402	0.487486349	0.60307992
Q9Y6M5	1.454	0.540027269	0.60307992
O75487	1.506	0.59072177	0.60307992
H0YMQ3	1.513	0.597411988	0.60307992
P07355	1.202	0.265436896	0.60357662

Q13015	1.481	0.566571641	0.60370362
Q49AR2	1.467	0.552868871	0.60435924
Q13185	1.202	0.265436896	0.60541844
B7Z6B8	1.225	0.292781749	0.6064401
P62330	1.25	0.321928095	0.60646254
P30046	0.835	-0.260151897	0.60705758
O60870	0.782	-0.354759487	0.60794727
Q9BY89	1.25	0.321928095	0.60855763
P62857	0.845	-0.242976753	0.60922214
A3KMH1	0.811	-0.30222618	0.60947108
E7EWS7	1.201	0.264236151	0.60947108
Q15126	1.356	0.439357178	0.60947108
Q99653	0.829	-0.270555993	0.6099813
Q96K37	0.78	-0.358453971	0.61001429
O75298	1.474	0.559736524	0.61001429
Q969T4	0.774	-0.369594529	0.61078166
P43686	1.2	0.263034406	0.6115693
P36543	0.83	-0.268816758	0.61256304
P11171	0.791	-0.3382504	0.61323266
P24844	1.442	0.528071165	0.61401297
Q9UIS9	0.712	-0.490050854	0.61485943
O75489	0.828	-0.272297327	0.61485943
B4DM74	0.847	-0.239566125	0.61485943
Q12834	1.203	0.266636643	0.61485943
D6RDH4	1.462	0.547943311	0.61485943
Q8NFF5	0.813	-0.298672743	0.61499621
E7EWM1	0.694	-0.526992432	0.61654838
E9PRY8	1.198	0.260627908	0.61654838
P60891	1.233	0.3021728	0.61654838
Q00577	1.354	0.437227739	0.61654838
Q15904	0.811	-0.30222618	0.61656316
P52907	1.198	0.260627908	0.61656316
Q9NSD9	0.847	-0.239566125	0.61712965
Q10570	1.243	0.313826296	0.6184895
H0YLF3	1.297	0.37517848	0.6184895
Q8TD16	0.734	-0.446148032	0.61866616
Q9BRA0	0.752	-0.411195433	0.61866616
P50579	1.263	0.336854639	0.61866616
A6NMQ1	1.368	0.45206823	0.61866616
Q14145	1.369	0.453122447	0.61866616
O14656	1.393	0.478195258	0.61866616
Q8TF05-2	0.809	-0.305788392	0.61874268
P09104	1.233	0.3021728	0.61874268
C9JPV1	1.491	0.576280258	0.61874268
P0CB43	1.366	0.449957484	0.61982271
Q8IZ69	0.794	-0.332789088	0.61986764

Q9Y6X9	0.754	-0.407363571	0.62064488
Q9BTT6	1.197	0.259423152	0.62076928
Q9Y3E7	0.764	-0.388355457	0.62083222
Q8IV38	1.377	0.461528559	0.62083222
Q8TDD1	0.826	-0.275786313	0.6214967
P29373	1.197	0.259423152	0.6214967
O95486	0.834	-0.261880711	0.62170526
Q9BTD8	0.835	-0.260151897	0.62172087
O75718	1.307	0.386259141	0.62301431
F6XY72	0.849	-0.236163541	0.62461715
A8MY43	0.772	-0.373327247	0.62487715
Q13526	1.248	0.319617934	0.62487715
P30519	0.828	-0.272297327	0.62502239
P33176	1.196	0.25821739	0.62502239
P51148	0.849	-0.236163541	0.62510568
P51809	0.831	-0.267079618	0.62514628
P38606	0.846	-0.241270432	0.62514628
Q9H3P7	0.825	-0.277533976	0.62552063
P13284	0.792	-0.336427665	0.62560268
P16435	1.196	0.25821739	0.62616853
P30740	0.841	-0.249822294	0.62634002
P49914	0.793	-0.334607229	0.62702431
H0YIV9	1.372	0.456280482	0.62721427
Q69YN4	1.286	0.362890643	0.62822191
P57088	1.224	0.291603558	0.62823149
Q92614	0.801	-0.320125852	0.62895052
Q92506	0.792	-0.336427665	0.63028794
B4DV96	0.799	-0.323732592	0.63028794
Q86VP6	1.194	0.255802837	0.63028794
Q9H299	1.228	0.296310561	0.63070402
Q08257	1.194	0.255802837	0.63074972
Q13112	1.317	0.397255346	0.63074972
Q9BRJ6	1.368	0.45206823	0.63074972
P50990	1.194	0.255802837	0.63084923
P51572	0.845	-0.242976753	0.63144055
H0Y9X1	0.832	-0.265344567	0.63164383
B1AK40	1.303	0.381837084	0.6319511
Q13217	0.827	-0.274040765	0.63266747
Q96RF0	0.73	-0.454031631	0.63281189
J3QTA6	0.816	-0.293358943	0.63281189
Q9P0S9	0.834	-0.261880711	0.63281189
P63279	1.193	0.254594043	0.63281189
O75940	1.249	0.320773477	0.63281189
B0V109	1.329	0.410341105	0.63281627
Q969S2	0.721	-0.471928835	0.63288975
Q5JTY5	0.828	-0.272297327	0.63288975

E9PCS8	0.722	-0.469929258	0.63333279
Q9Y666	0.781	-0.356605547	0.63338182
Q9BV81	0.74	-0.434402824	0.63374793
Q9H6S0	0.803	-0.316528107	0.63374793
F5H577	0.82	-0.286304185	0.63412379
D3YTH9	1.457	0.543000877	0.63412379
G3V150	0.793	-0.334607229	0.63437515
P46734	0.833	-0.263611599	0.63453246
D3YTC9	1.383	0.467801156	0.63453246
Q9NUQ2	0.815	-0.295128036	0.63466999
F6UYM1	1.42	0.50589093	0.63618564
Q9BTT4	0.721	-0.471928835	0.63671249
E9PB35	1.429	0.515005916	0.63671249
Q9NXG2	0.852	-0.231074664	0.63732614
O95352	1.387	0.471967788	0.63732614
Q92544	0.83	-0.268816758	0.63738332
O15397	1.317	0.397255346	0.63761135
Q8WXD5	1.388	0.473007568	0.6377752
Q9H0U6	0.839	-0.253257284	0.63789175
Q9NR31	1.225	0.292781749	0.63899658
Q9Y6M9	0.837	-0.256700472	0.63907326
Q15257	1.243	0.313826296	0.6396722
K7EJQ7	1.427	0.512985335	0.6396722
O75446	1.437	0.523060062	0.6396722
P54886	0.853	-0.229382353	0.64053633
A6NIK2	0.741	-0.432454552	0.64064986
F5H2X7	0.715	-0.483984853	0.64133855
Q9BVS4	1.281	0.357270476	0.64133855
E9PQ61	1.308	0.387362541	0.64133855
Q9GZS1-2	0.841	-0.249822294	0.64204258
P41227	0.848	-0.23786383	0.64204258
E7EN44	1.465	0.550900665	0.64227073
P50416	0.834	-0.261880711	0.64299568
P51003	1.224	0.291603558	0.64309166
Q6P1K2	1.444	0.530070742	0.64309166
Q9Y613	0.716	-0.481968507	0.6437381
E9PAY8	0.797	-0.327348371	0.64425495
Q969M3	0.735	-0.444183845	0.64464522
Q8TAD8	1.38	0.464668267	0.64505318
Q9H3U1	1.189	0.249748715	0.64610229
Q9H2J4	1.22	0.286881148	0.64610229
Q9UHD2	1.265	0.339137385	0.64675355
E9PGE1	1.189	0.249748715	0.64680209
Q9NUQ9	1.189	0.249748715	0.64680209
O94992	1.24	0.310340121	0.64680209
Q96I99	0.843	-0.246395464	0.64703225

P14923	0.854	-0.227692025	0.64703225
Q96EV2	1.357	0.440420721	0.64703225
Q6UW02	1.433	0.51903861	0.64703225
O95139	0.841	-0.249822294	0.64705202
P61160	1.188	0.248534836	0.64715373
D6RH22	1.259	0.332278283	0.64785509
Q9UEE9	1.338	0.420078116	0.64785509
Q9BQC6	0.822	-0.282789701	0.64795771
O75915	0.806	-0.311148256	0.64813515
Q9UJ68	1.428	0.513995979	0.64816051
Q9Y3D5	1.429	0.515005916	0.64816051
Q9H0E9	1.431	0.517023672	0.64816051
P04181	1.188	0.248534836	0.64924599
O43823	1.306	0.385154897	0.64932716
P13051	0.829	-0.270555993	0.64942173
H3BNZ1	0.841	-0.249822294	0.65056614
P09601	1.35	0.432959407	0.65056614
F5H2Q7	1.45	0.5360529	0.65056614
Q9H936	0.838	-0.254977851	0.65111211
Q6UB35	1.187	0.247319935	0.65111211
Q9BZH6	0.826	-0.275786313	0.65156193
P10176	0.712	-0.490050854	0.6522208
H0YBD2	0.823	-0.281035664	0.6522208
A6NGJ0	1.349	0.431890348	0.65292431
Q13158	1.4	0.485426827	0.65292431
P0CG34	1.371	0.455228571	0.65337938
P28072	0.856	-0.224317298	0.65376107
Q9NX14	1.3	0.378511623	0.65405502
P63151	1.215	0.280956314	0.65457413
Q96ED9	0.784	-0.351074441	0.65475171
O75475	1.194	0.255802837	0.65475171
O43427	1.342	0.424384672	0.65475171
Q6P2C8	1.363	0.446785562	0.65475171
Q96DE0	1.435	0.521050737	0.65475171
Q9Y5Y0	1.454	0.540027269	0.65475171
Q5JTZ9	1.464	0.549915554	0.65475171
O95628	1.471	0.556797247	0.65475171
P50502	0.856	-0.224317298	0.65565496
Q9Y3A5	0.838	-0.254977851	0.65619791
O95881	1.235	0.304511042	0.65674382
Q9UHI6	1.234	0.303342394	0.65695277
O75717	1.246	0.317304068	0.65709512
Q9BTV6	1.453	0.539034703	0.65753182
Q9NR28	0.852	-0.231074664	0.6579282
F5H872	0.764	-0.388355457	0.65798441
E9PS80	0.749	-0.416962376	0.65838869

P12429	0.857	-0.222632891	0.65960633
Q13888	0.845	-0.242976753	0.66002009
Q96P70	1.184	0.243669081	0.66002009
Q9HB40	1.335	0.416839742	0.66002009
Q9NRW1	1.37	0.454175893	0.66009431
Q7L576	0.858	-0.220950447	0.66058414
E7EVC7	1.338	0.420078116	0.66109246
P13473	0.835	-0.260151897	0.66187088
Q01581	1.183	0.242450074	0.66187088
Q9H3S7	0.845	-0.242976753	0.661916
Q9UNY4	0.719	-0.475936324	0.6632806
Q7L2E3-2	1.183	0.242450074	0.6632806
Q92979	1.183	0.242450074	0.6632806
Q93074	1.365	0.448900951	0.6632806
H7C4P4	0.8	-0.321928095	0.6633086
P11233	0.844	-0.244685096	0.6633086
Q92621	1.183	0.242450074	0.6633086
Q8WZ42	0.845	-0.242976753	0.66336164
Q96G23	1.233	0.3021728	0.66336164
Q9H0E2	0.805	-0.312939312	0.66351278
P11217	1.348	0.430820497	0.66351278
Q9NQ29	0.799	-0.323732592	0.6636712
Q5VWZ2	0.837	-0.256700472	0.6636712
G3XAL7	0.838	-0.254977851	0.6636712
Q12872	1.429	0.515005916	0.6636712
P12955	1.213	0.27857955	0.66409944
O14672	0.819	-0.288064643	0.66438816
E5RFF9	1.346	0.42867841	0.66499091
Q14746	0.825	-0.277533976	0.66499478
P10644	1.182	0.241230036	0.66681287
Q9UNS1	1.274	0.349365278	0.66840312
Q9Y2R4	0.845	-0.242976753	0.66859924
G3V1C4	1.27	0.344828497	0.66873724
A0AVT1	1.181	0.240008965	0.66892358
O95716	1.366	0.449957484	0.66906826
Q9BWM7	0.847	-0.239566125	0.66928134
H3BN98	0.86	-0.217591435	0.67066858
Q5VT52	1.343	0.425459305	0.67070805
E7ESY4	1.227	0.295135249	0.67181092
Q75MJ1	1.181	0.240008965	0.67185932
J3KNX7	1.257	0.32998465	0.67185932
G3V5X4	1.332	0.413594082	0.67185932
Q13405	0.842	-0.248107862	0.6733292
Q9UJM3	0.796	-0.329159664	0.67353048
H0YF29	0.829	-0.270555993	0.67353048
Q9UBV2	1.339	0.421155961	0.67353048

F5GXC8	0.828	-0.272297327	0.67375128
Q14186	1.383	0.467801156	0.67382001
Q8NFZ8	1.304	0.38294387	0.6738402
Q9NV56	1.403	0.488515009	0.67435461
P20226	0.794	-0.332789088	0.67518224
Q96P11	0.844	-0.244685096	0.67539337
Q15031	1.383	0.467801156	0.67542575
Q9NVP2	0.83	-0.268816758	0.67621471
Q8IYQ7	0.849	-0.236163541	0.67678475
B4DR12	0.738	-0.438307279	0.67683873
Q14554	0.803	-0.316528107	0.67683873
Q96A35	0.836	-0.258425153	0.67683873
Q969X5	0.837	-0.256700472	0.67683873
O00422	0.837	-0.256700472	0.67683873
O60826	1.366	0.449957484	0.67683873
F8W914	1.367	0.451013243	0.67683873
Q9GZT8	1.236	0.305678743	0.67707139
Q9UJX4	0.813	-0.298672743	0.67717373
P07951-2	1.179	0.237563718	0.67734739
Q08378	1.253	0.325386415	0.67789346
Q9NTG7	0.748	-0.418889825	0.67849021
P46777	0.861	-0.215914857	0.67849021
J3KPV4	1.178	0.236339539	0.67849021
O76094	1.187	0.247319935	0.678976
J3QSH4	0.749	-0.416962376	0.67915475
Q06203	0.862	-0.214240226	0.67915475
Q96S59	0.853	-0.229382353	0.67996555
Q9BYT8	0.858	-0.220950447	0.68013827
O95163	1.185	0.244887059	0.68013827
Q5BKZ1	0.843	-0.246395464	0.68038782
F2Z2Z9	0.816	-0.293358943	0.68039249
Q9Y291	0.844	-0.244685096	0.68039249
Q96HE7	0.862	-0.214240226	0.68039249
Q9NSE4	0.862	-0.214240226	0.68039249
D6R9Q5	1.419	0.504874589	0.68039249
Q9Y6A9	1.28	0.35614381	0.68104145
Q14653	0.789	-0.341902795	0.68160985
P08133	1.332	0.413594082	0.68204527
Q9BW19	1.364	0.447843644	0.68204527
Q9UBK9	1.414	0.49978212	0.68204527
Q9UDW1	0.846	-0.241270432	0.68205088
Q9Y3Z3	1.208	0.272620455	0.68215267
J3QQ67	0.863	-0.212567535	0.68402666
P40939	0.863	-0.212567535	0.68434425
Q13425	1.336	0.417920008	0.68517671
O95159	0.818	-0.289827252	0.6855479

P61353	0.863	-0.212567535	0.68631361
Q10469	1.403	0.488515009	0.68633756
J3QSZ6	1.44	0.526068812	0.68641454
Q8IUX4	0.761	-0.394031641	0.68765383
Q7Z417	1.232	0.301002256	0.68855083
P15374	0.844	-0.244685096	0.68889586
Q9H089	0.849	-0.236163541	0.68890666
Q5SWX3	1.305	0.384049807	0.68925507
Q9BW04	0.742	-0.430508908	0.68978416
Q6GYA4	0.743	-0.428565884	0.68978416
Q96BR5	0.856	-0.224317298	0.68978416
E9PHT6	1.398	0.483364361	0.68983883
Q5TBB1	1.444	0.530070742	0.68988533
Q9P287	0.864	-0.210896782	0.68999628
Q02338	0.821	-0.284545873	0.69093258
Q9UBT2	0.864	-0.210896782	0.69195781
C6G496	0.708	-0.498178735	0.69343101
Q8IU85	0.746	-0.422752464	0.69351257
E9PLN8	0.815	-0.295128036	0.69351257
Q9BXW7	0.865	-0.209227962	0.69356294
P49336	0.701	-0.512513651	0.69418248
E7EUC7	0.842	-0.248107862	0.69435256
Q9Y4C2	1.287	0.364012054	0.69451082
Q15843	0.812	-0.300448367	0.69455507
Q8IX18	0.853	-0.229382353	0.69532654
Q9BRT6	0.82	-0.286304185	0.69539394
Q9H9A6	0.851	-0.232768963	0.69539394
P36507	1.201	0.264236151	0.69539394
Q86TV2	0.763	-0.390245038	0.6956494
Q9GZN8	1.301	0.379620962	0.69646565
Q5F1R6	1.396	0.481298942	0.69646565
Q6DKJ4	1.222	0.289244285	0.69693398
Q5T653	0.854	-0.227692025	0.69718253
B7Z2C3	1.416	0.501821265	0.69718253
G5E9D3	0.849	-0.236163541	0.69744777
Q96C86	1.224	0.291603558	0.69762242
P24385	0.786	-0.347398782	0.69799784
Q9BST9	1.32	0.40053793	0.69799784
Q3B7T1	0.828	-0.272297327	0.69867545
P63146	0.748	-0.418889825	0.69906996
Q71UI9	1.172	0.22897257	0.69941273
Q9NYY8	0.851	-0.232768963	0.69981753
P09001	0.851	-0.232768963	0.69981753
P49903	1.215	0.280956314	0.69981753
Q5BJF2	1.343	0.425459305	0.69984635
B4DWJ3	0.833	-0.263611599	0.70027328

Q6IAN0	1.349	0.431890348	0.70027328
Q9BSL1	0.757	-0.401634795	0.70091003
Q58FF7	0.753	-0.40927823	0.70099786
D6RAN8	0.852	-0.231074664	0.70230817
H0YAN8	0.815	-0.295128036	0.70282001
Q6P1A2	0.785	-0.349235441	0.70355434
Q6ZNE5	0.79	-0.340075442	0.70355434
Q9Y6I4	0.813	-0.298672743	0.70355434
Q8TCS8	0.85	-0.234465254	0.70355434
O43324	0.85	-0.234465254	0.70355434
G5E9Q6	0.855	-0.226003675	0.70355434
Q92804	0.856	-0.224317298	0.70355434
P47914	0.867	-0.205896101	0.70355434
P29992	1.231	0.299830762	0.70355434
Q9Y6K9	1.295	0.372952098	0.70355434
O95365	1.342	0.424384672	0.70355434
G8JL90	1.366	0.449957484	0.70355434
F5H604	1.434	0.520045024	0.70355434
P61006	1.217	0.283329168	0.70356833
Q7Z309	1.393	0.478195258	0.70356833
Q96T37	1.253	0.325386415	0.70407068
Q6ZSC3	0.842	-0.248107862	0.70420794
Q9NWT6	1.313	0.392866916	0.70471886
Q9H9T3	1.198	0.260627908	0.7051717
E7EM93	1.389	0.474046599	0.7051717
Q9BUL5	1.404	0.489542936	0.7051717
Q5T760	1.2	0.263034406	0.70565434
E9PFR3	1.314	0.393965276	0.7057682
P16219	0.853	-0.229382353	0.7058112
Q9HCK1	0.868	-0.204233052	0.70616256
Q9GZR7	0.868	-0.204233052	0.70673801
O60869	1.222	0.289244285	0.70863868
P59998	1.177	0.23511432	0.70932256
Q9GZU8	0.826	-0.275786313	0.71031249
Q9BU23	0.747	-0.420819852	0.71046954
P33992	1.169	0.22527493	0.71046954
P46108	1.213	0.27857955	0.7107257
P30533	0.86	-0.217591435	0.71091565
H7C2W9	0.869	-0.202571918	0.71091565
Q13509	1.168	0.224040274	0.71091565
O75381	1.256	0.328836464	0.71091565
Q9H4L7	1.32	0.40053793	0.71091565
Q12972	1.211	0.276198865	0.71123589
O95167	0.846	-0.241270432	0.71204959
P60842	1.168	0.224040274	0.71253246
P15104	1.248	0.319617934	0.71308001

P61962	1.262	0.33571191	0.7132493
Q7Z4G1	1.357	0.440420721	0.71506222
Q9Y271	0.756	-0.40354186	0.71513962
E9PQW4	1.32	0.40053793	0.71535152
Q9UBL6	0.829	-0.270555993	0.71560453
P25445	0.754	-0.407363571	0.71577105
P05060	0.765	-0.386468347	0.71577105
Q13952	0.822	-0.282789701	0.71577105
P22695	0.87	-0.200912694	0.71577105
P62314	1.167	0.222804561	0.71577105
P53597	0.854	-0.227692025	0.71584143
P43246	1.194	0.255802837	0.71634108
Q5JRA6	0.853	-0.229382353	0.71658079
P52948	1.175	0.232660757	0.71658079
Q9NX46	1.223	0.290424404	0.71658079
Q9UPQ9	1.409	0.494671612	0.71658079
Q6P1X5	0.821	-0.284545873	0.71716367
Q8WXF1	0.854	-0.227692025	0.71716367
H7BXW3	1.221	0.2880632	0.71716367
O75663	1.253	0.325386415	0.71716819
O00411	1.21	0.275007047	0.7185021
Q9NW64	0.861	-0.215914857	0.71903846
Q9BYN8	1.247	0.318461465	0.72001305
Q8N4Q1	0.863	-0.212567535	0.72020776
C9JTN7	0.837	-0.256700472	0.72067415
Q6NUQ1	0.82	-0.286304185	0.72095024
Q9H496	0.761	-0.394031641	0.72118055
Q9P0J0	0.861	-0.215914857	0.7221411
Q9Y3C6	0.862	-0.214240226	0.7221411
P41252	0.871	-0.199255376	0.72220554
Q9UJ72	1.323	0.403813062	0.72224818
Q96P16	1.231	0.299830762	0.72240742
Q9P2C4	1.336	0.417920008	0.72240742
Q5TCM7	0.844	-0.244685096	0.72450045
Q8NBN7	0.846	-0.241270432	0.72450045
P53634	1.192	0.253384236	0.72450045
O43395	1.196	0.25821739	0.72499073
Q96S44	1.307	0.386259141	0.72513081
Q13438	0.825	-0.277533976	0.72589035
Q96D71	0.865	-0.209227962	0.72589035
P30040	0.872	-0.19759996	0.72589035
O43143	1.164	0.219091058	0.72591497
D6RBZ0	0.872	-0.19759996	0.72609057
O43670	1.191	0.252173413	0.72609057
Q9UIV1	1.291	0.368489001	0.72768552
Q9NQX3	0.862	-0.214240226	0.72817142

O75175	1.37	0.454175893	0.72817142
Q14674	0.794	-0.332789088	0.72879667
Q13151	1.163	0.217851097	0.73004624
Q9Y2Q5	0.851	-0.232768963	0.73088102
Q9Y5B6	0.855	-0.226003675	0.73088102
Q6NVY1	0.857	-0.222632891	0.73102001
P12532	0.873	-0.195946441	0.73200816
J3KPN1	1.374	0.458382004	0.73200816
E9PLL6	0.873	-0.195946441	0.73222281
H3BMT8	0.856	-0.224317298	0.73234277
Q8NGJ5	1.37	0.454175893	0.73234277
O95478	0.851	-0.232768963	0.73299811
Q8IYS1	0.869	-0.202571918	0.73315713
O43865	1.162	0.216610069	0.73356884
Q13618	1.181	0.240008965	0.73356884
Q8NC44	0.763	-0.390245038	0.73407443
Q96CW5	1.335	0.416839742	0.73462886
Q9BUH6	1.317	0.397255346	0.7347051
Q8N8A6	1.287	0.364012054	0.73558242
Q9NQE9	0.757	-0.401634795	0.73612511
Q14157	1.161	0.215367972	0.73612511
H0Y6W0	0.759	-0.397828209	0.73673126
Q03519	0.782	-0.354759487	0.73673126
B5MDL5	0.788	-0.343732465	0.73673126
H0Y368	0.854	-0.227692025	0.73673126
Q9UHY7	0.859	-0.219269964	0.73673126
P18031	0.875	-0.192645078	0.73673126
Q5RKV6	1.181	0.240008965	0.73673126
Q9ULX3	1.19	0.250961574	0.73673126
Q9Y237	1.283	0.35952117	0.73673126
J3KQ97	1.347	0.429749851	0.73673126
P48745	1.359	0.442545456	0.73673126
Q9BQ39	1.327	0.408168371	0.73721565
P04899	1.16	0.214124805	0.7373764
Q9UIG0	1.167	0.222804561	0.73749374
Q16576	1.16	0.214124805	0.73906387
Q96EK6	0.866	-0.20756107	0.73925512
C9JE56	0.726	-0.461958547	0.73958359
Q07352	0.821	-0.284545873	0.73975023
Q8N1G2	1.362	0.445726703	0.73975023
C9JKX0	1.374	0.458382004	0.73975023
Q96T76	0.864	-0.210896782	0.73986116
Q9Y5T5	0.77	-0.377069649	0.74013272
Q9BW27	1.186	0.24610401	0.74013272
P27707	1.26	0.333423734	0.74013272
Q8ND76	1.343	0.425459305	0.74013272

Q6PD74	0.828	-0.272297327	0.74020347
Q8N4V1	1.311	0.390667686	0.7402278
F6S0T5	0.856	-0.224317298	0.74082103
Q9HCC0	0.863	-0.212567535	0.74082103
A6NMQ7	0.864	-0.210896782	0.74082103
Q9Y3U8	0.876	-0.190997225	0.74082103
Q15022	1.285	0.361768359	0.74082103
P10589	1.364	0.447843644	0.74082103
Q9Y3B3	0.86	-0.217591435	0.74087458
Q8N5G0	1.32	0.40053793	0.74087458
Q7Z7A3	1.339	0.421155961	0.74087458
O15050	0.829	-0.270555993	0.74096639
P14373	0.75	-0.415037499	0.74135642
Q9NVA4	0.777	-0.364013496	0.74135642
Q9BTZ2	0.872	-0.19759996	0.74135642
P41567	0.876	-0.190997225	0.74135642
Q9NP79	1.182	0.241230036	0.74178274
H0YBY3	1.337	0.418999465	0.74178274
F8W0J4	1.361	0.444667067	0.74178274
Q9UMX1	1.397	0.482332021	0.74178274
O14737	1.169	0.22527493	0.74220445
Q9BWU0	1.249	0.320773477	0.74235047
B1AM43	0.772	-0.373327247	0.74240304
B7Z9I3	0.829	-0.270555993	0.74240304
Q9UI30	1.185	0.244887059	0.74240304
E7EUT4	1.372	0.456280482	0.74240304
Q9UER7	1.318	0.39835037	0.74340225
Q5U5X0	1.318	0.39835037	0.74367458
O15511	1.163	0.217851097	0.74371614
P35251	1.221	0.2880632	0.74386337
Q9NZI7	0.768	-0.380821784	0.74390107
Q12906	1.158	0.211635253	0.74390107
O15143	1.158	0.211635253	0.74390107
Q7Z2E3	1.379	0.463622457	0.74390107
Q12768	0.829	-0.270555993	0.74429012
Q9P0V9	0.854	-0.227692025	0.74429012
F8VXU5	0.864	-0.210896782	0.74429012
P55060	1.157	0.210388864	0.74429012
Q92747	1.207	0.271425676	0.74429012
Q9BTU6	1.286	0.362890643	0.74429012
Q9HB90	1.323	0.403813062	0.74429012
C9JQS9	1.365	0.448900951	0.74429012
Q9Y6A4	1.234	0.303342394	0.74452929
Q9NRX4	1.185	0.244887059	0.74477881
O00567	0.878	-0.187707155	0.74486815
Q9Y4B6	1.239	0.309176187	0.74519403

C9J9T0	1.308	0.387362541	0.74519403
Q8TBP6	1.388	0.473007568	0.74519716
Q9Y5J7	0.872	-0.19759996	0.74542588
Q00059	0.858	-0.220950447	0.74577193
P00367	0.878	-0.187707155	0.74577193
P21399	1.168	0.224040274	0.74632053
E9PHI4	0.859	-0.219269964	0.74651421
Q13111	1.282	0.358396262	0.74711404
P42766	0.878	-0.187707155	0.74750303
F5GZY7	1.352	0.435095152	0.74832562
O15084-1	0.871	-0.199255376	0.74884987
Q8TED0	1.162	0.216610069	0.74884987
Q7L8L6	1.312	0.39176772	0.74884987
Q9H9B1	1.355	0.438292852	0.74884987
Q15599	1.374	0.458382004	0.74884987
P49750	1.193	0.254594043	0.74885179
Q9NR48	1.231	0.299830762	0.74885179
P27816	1.266	0.340277405	0.74885179
Q9UDY4	0.868	-0.204233052	0.74892023
Q5JWF2	1.19	0.250961574	0.74892023
P21741	0.869	-0.202571918	0.74896468
Q99714	0.879	-0.18606493	0.7490254
P35270	0.879	-0.18606493	0.74947726
Q9P2J5	0.879	-0.18606493	0.74960185
Q9NYF8	1.155	0.207892852	0.74960185
Q2TAA2	1.28	0.35614381	0.74960185
P46821	1.296	0.374065718	0.75075197
P32121	1.278	0.353887836	0.75177675
Q9BWF3	0.88	-0.184424571	0.75236169
Q9P0L0	0.88	-0.184424571	0.75240998
Q9H3E2	1.154	0.206643224	0.75251376
Q96EK5	1.183	0.242450074	0.75332377
Q01105	1.315	0.3950628	0.75332377
Q96SI9	1.374	0.458382004	0.75332377
Q9Y3I1	0.859	-0.219269964	0.75374107
Q99543	1.199	0.261831659	0.75432083
O60524	0.858	-0.220950447	0.75453741
E5RGS4	1.232	0.301002256	0.75453741
Q9BZL6	1.257	0.32998465	0.75453741
P40222	1.153	0.205392513	0.75469938
P18124	0.88	-0.184424571	0.75497919
P30085	0.865	-0.209227962	0.75561842
P24534	0.88	-0.184424571	0.75561842
Q53EU6	0.841	-0.249822294	0.75594618
Q15554	1.277	0.352758525	0.75594618
Q01780	0.866	-0.20756107	0.75616894

B4DFR4	1.199	0.261831659	0.75616894
A6NHR9	1.287	0.364012054	0.75616894
O95755	0.822	-0.282789701	0.75635001
Q9Y3E0	0.857	-0.222632891	0.75635001
P30042	0.861	-0.215914857	0.75635001
Q9H3N1	0.872	-0.19759996	0.75635001
E9PHQ0	1.153	0.205392513	0.75635001
P30455	1.153	0.205392513	0.75635001
Q96C36	1.167	0.222804561	0.75635001
Q9BXB4	1.271	0.34596403	0.75635001
O75438	0.864	-0.210896782	0.75647134
G3XAC4	1.291	0.368489001	0.75670944
E9PQV6	0.775	-0.367731785	0.75701059
P11908	1.152	0.204140717	0.75701059
Q01105-2	0.881	-0.182786076	0.75735203
E7ENU4	1.152	0.204140717	0.75735203
Q9UJ41	1.301	0.379620962	0.75735203
A6NEM2	1.152	0.204140717	0.75735493
P62805	1.152	0.204140717	0.75735493
Q9H6T3	1.204	0.267835392	0.75735493
B7Z888	1.227	0.295135249	0.75735493
Q8NA72	1.356	0.439357178	0.75735493
F5H442	1.212	0.277389699	0.75750961
P22033	0.867	-0.205896101	0.75781204
Q6ICB0	0.83	-0.268816758	0.75808068
Q06265	0.865	-0.209227962	0.75832569
Q14444	1.152	0.204140717	0.75855705
P36578	0.882	-0.181149439	0.75886355
Q8WUY8	1.343	0.425459305	0.75943733
P49916	0.874	-0.194294815	0.75950035
O00743	1.158	0.211635253	0.75950035
P61011	1.17	0.22650853	0.75950035
Q6P6B1	1.316	0.396159489	0.75950035
Q0PNE2	1.342	0.424384672	0.75950035
P68133	1.151	0.202887833	0.75950113
Q9NRG9	1.2	0.263034406	0.75950113
P10599	0.882	-0.181149439	0.75996882
F5GWH5	0.868	-0.204233052	0.76021193
Q8NE71	1.153	0.205392513	0.76021193
Q96CT7	1.204	0.267835392	0.76021193
J3QQY0	1.308	0.387362541	0.76021193
Q8WVD3	1.379	0.463622457	0.76021193
P62714	1.151	0.202887833	0.76021689
D6RFN0	1.153	0.205392513	0.76021689
Q07812	1.243	0.313826296	0.76021689
Q9BPU6	1.341	0.423309237	0.76021689

P10586	0.836	-0.258425153	0.76030837
Q9UPT5	1.22	0.286881148	0.76030837
Q9H7B2	0.868	-0.204233052	0.76354645
Q8IZP0	1.189	0.249748715	0.76354645
P45983	1.271	0.34596403	0.76354645
O00221	1.287	0.364012054	0.76354645
H0YAP1	1.335	0.416839742	0.76354645
Q13177	1.155	0.207892852	0.76367007
P61026	0.873	-0.195946441	0.76370543
Q13428-3	1.155	0.207892852	0.76370543
O96028	1.311	0.390667686	0.76370543
Q5VW36	0.844	-0.244685096	0.76383445
P84085	0.884	-0.177881725	0.76404375
G3V2F7	1.149	0.200378798	0.76404375
Q6ZN18	0.735	-0.444183845	0.76425977
Q08AF3	0.852	-0.231074664	0.76425977
Q8WXX5	1.174	0.231432408	0.76425977
Q63ZY3	1.275	0.350497247	0.76425977
P61020	0.848	-0.23786383	0.76503263
Q96FW1	1.148	0.199122642	0.76503263
Q8IWR0	1.355	0.438292852	0.76503263
Q9BWS9	0.803	-0.316528107	0.76506456
Q9NR30	0.884	-0.177881725	0.76506456
O75165	1.27	0.344828497	0.76515496
Q9NZN4	0.868	-0.204233052	0.76516868
O00483	0.876	-0.190997225	0.76516868
Q15910	1.284	0.360645202	0.76516868
Q9NZQ3	1.344	0.426533138	0.76516868
Q08AD1	0.781	-0.356605547	0.76543638
E7ETU9	0.832	-0.265344567	0.76579413
Q96GC9	0.863	-0.212567535	0.76579413
Q96B97	0.865	-0.209227962	0.76579413
O00410	1.148	0.199122642	0.76579413
P18669	1.148	0.199122642	0.76579413
P00395	0.878	-0.187707155	0.76629533
Q13542	0.799	-0.323732592	0.76646965
O60476	0.825	-0.277533976	0.76669373
Q92759	0.841	-0.249822294	0.76669373
K7EQF4	1.148	0.199122642	0.76669373
C9J2Y9	1.152	0.204140717	0.76669373
P83916	1.167	0.222804561	0.76669373
H7BY84	0.876	-0.190997225	0.76731157
Q9Y679	1.284	0.360645202	0.76767035
P12277	0.885	-0.17625064	0.76806006
P49411	0.885	-0.17625064	0.76920147
Q7RTS9	0.755	-0.40545145	0.77164994

P28799	0.82	-0.286304185	0.77173209
P36405	1.176	0.23388806	0.77204454
P30044	1.146	0.196607044	0.77234286
Q99633	0.751	-0.413115187	0.77250286
H7C4Z8	0.794	-0.332789088	0.77250286
Q8N323	0.872	-0.19759996	0.77250286
P98175	1.209	0.273814245	0.77250286
Q6SPF0	1.364	0.447843644	0.77250286
P41250	0.886	-0.174621396	0.77315208
Q9NYJ1	0.855	-0.226003675	0.77346172
P11047	0.878	-0.187707155	0.77377403
O43776	0.886	-0.174621396	0.77377403
P47897	1.145	0.195347598	0.77479922
O00231	1.145	0.195347598	0.77479922
Q13043	1.351	0.434027675	0.77479922
Q86UY0	0.886	-0.174621396	0.77483146
P00488	0.84	-0.251538767	0.77504093
Q6GQQ9	0.782	-0.354759487	0.77624566
Q9UKJ3	0.83	-0.268816758	0.77624566
P63000	1.145	0.195347598	0.77624566
Q9NZ01	1.149	0.200378798	0.77624566
O94760	1.201	0.264236151	0.77624566
B7Z5R1	1.234	0.303342394	0.77624566
F5H2M7	1.245	0.316145742	0.77624566
Q8IYB7	1.265	0.339137385	0.77624566
H3BV60	0.85	-0.234465254	0.7769317
O43390	1.144	0.194087052	0.77754108
Q96FN5	1.297	0.37517848	0.77755173
B7Z588	1.33	0.411426246	0.77805346
P09669	0.875	-0.192645078	0.77915931
Q92520	0.88	-0.184424571	0.77993716
P35659	1.144	0.194087052	0.7800935
E9PAU2	1.17	0.22650853	0.78048409
Q4VC31	0.875	-0.192645078	0.78117855
Q9HAU0	1.292	0.36960607	0.7825213
P29144	0.88	-0.184424571	0.78268815
P26641	0.888	-0.171368418	0.78268815
Q15648	1.328	0.409255147	0.78268815
Q9NXA8	0.825	-0.277533976	0.78273556
Q6EEV4	0.838	-0.254977851	0.78273556
P49427	0.839	-0.253257284	0.78273556
P49770	0.882	-0.181149439	0.78273556
Q9C0B1	0.887	-0.17299399	0.78273556
Q9Y5L0	1.151	0.202887833	0.78273556
P33240	1.166	0.221567789	0.78273556
Q96AD0	1.231	0.299830762	0.78273556

B4DY38	1.337	0.418999465	0.78273556
Q8TAQ2	1.342	0.424384672	0.78273556
Q8NDZ4	0.763	-0.390245038	0.78314099
E9PHX5	1.34	0.422233001	0.78314099
Q9UBQ0	0.803	-0.316528107	0.78315133
Q99986	1.172	0.22897257	0.78315133
Q9H2U2	0.888	-0.171368418	0.78319063
Q9ULJ7	0.773	-0.371459681	0.78327758
Q9UFC0	0.844	-0.244685096	0.7838918
Q86WQ0	0.888	-0.171368418	0.78410302
Q9HC52	1.251	0.32308179	0.78439821
Q6PJG6	1.192	0.253384236	0.78452453
Q12931	0.889	-0.169744676	0.78532007
Q14331	1.285	0.361768359	0.78532007
Q5VXN5	1.338	0.420078116	0.78532007
Q9Y5K6	0.872	-0.19759996	0.78609539
P12236	0.889	-0.169744676	0.78609539
O14802	1.309	0.388465097	0.78609539
Q15942	1.19	0.250961574	0.78644895
O00273	1.156	0.209141398	0.78664945
H0YKD8	0.889	-0.169744676	0.78667827
Q8WXI9	1.256	0.328836464	0.78754057
Q8IX04	1.345	0.427606173	0.78754057
Q14684	1.165	0.220329955	0.78765225
Q3KQV9	0.792	-0.336427665	0.78784732
O43760	0.882	-0.181149439	0.78796986
Q15631	0.887	-0.17299399	0.78796986
H3BRM1	1.334	0.415758667	0.78796986
Q6UX04	0.888	-0.171368418	0.7880037
P46736	0.871	-0.199255376	0.78812847
Q96RQ3	0.875	-0.192645078	0.788172
G3V0I6	0.846	-0.241270432	0.78961804
Q9BQ67	1.166	0.221567789	0.78975957
P82675	0.887	-0.17299399	0.78977661
H0YB09	0.777	-0.364013496	0.78988457
O43731	0.827	-0.274040765	0.78988457
Q15365	1.14	0.189033824	0.7902693
Q9BQS8	0.792	-0.336427665	0.79091853
P31939	0.89	-0.168122759	0.79091853
Q13596	1.147	0.197865391	0.79091853
P46531	1.167	0.222804561	0.79091853
Q9NYH9	0.875	-0.192645078	0.79093833
Q13287	0.795	-0.330973234	0.79142244
O43264	0.878	-0.187707155	0.79142244
P31930	0.891	-0.166502663	0.79142244
Q8N6M0	0.893	-0.16326792	0.79142244

Q6P1N0	1.341	0.423309237	0.79142244
F5H136	0.847	-0.239566125	0.79147278
Q9NQR4	1.155	0.207892852	0.79170213
H9KV45	1.166	0.221567789	0.79170213
Q05048	1.169	0.22527493	0.79170213
P30825	1.288	0.365132593	0.79170213
Q9UJK0	0.792	-0.336427665	0.79179992
Q9NX70	0.762	-0.392137097	0.7920139
Q7Z333	0.791	-0.3382504	0.7920139
P62280	0.891	-0.166502663	0.79209955
Q8TF01	0.821	-0.284545873	0.79257951
Q6NS38	0.863	-0.212567535	0.79257951
Q66PJ3	0.867	-0.205896101	0.79257951
O43674	0.888	-0.171368418	0.79257951
K7EKE6	0.891	-0.166502663	0.79257951
P25205	1.139	0.187767747	0.79257951
Q15717	1.139	0.187767747	0.79257951
Q92600	1.194	0.255802837	0.79257951
P62633	1.265	0.339137385	0.79257951
O96007	1.315	0.3950628	0.79257951
C9JIB4	1.347	0.429749851	0.79257951
Q8N7H5	0.88	-0.184424571	0.79285088
Q8N392	0.887	-0.17299399	0.79285088
K7ELL7	0.892	-0.164884385	0.79285088
P23258	1.154	0.206643224	0.79285088
P55957	1.163	0.217851097	0.79285088
Q9UHW5	1.247	0.318461465	0.79285088
Q9BUK6	1.264	0.337996464	0.79285088
Q9NZD8	1.311	0.390667686	0.79285088
Q96FJ0	1.321	0.401630467	0.79285088
Q6GMV3	0.861	-0.215914857	0.79304361
O14646	1.283	0.35952117	0.79304361
Q9H0X4	1.329	0.410341105	0.79358804
Q96BM9	1.266	0.340277405	0.79368728
Q6AI08	1.211	0.276198865	0.79377884
E9PGG2	1.244	0.314986485	0.79392173
O95793-3	1.142	0.191562651	0.79424649
F5H667	0.89	-0.168122759	0.79432111
P11142	0.892	-0.164884385	0.79432111
Q9UNX3	0.892	-0.164884385	0.79432111
Q6P2E9	1.138	0.186500558	0.79432111
J3KNF8	1.138	0.186500558	0.79432111
Q7Z739	1.177	0.23511432	0.79432111
O96013	1.248	0.319617934	0.79432111
Q96A72	1.254	0.326537348	0.79432111
P51687	0.862	-0.214240226	0.79433226

Q13409-2	1.158	0.211635253	0.79475557
Q147X3	0.763	-0.390245038	0.79487982
Q8N6T3	1.246	0.317304068	0.7950894
O95758-1	0.892	-0.164884385	0.795217
P02656	0.873	-0.195946441	0.79524381
P57772	1.279	0.355016264	0.79531271
P07919	0.884	-0.177881725	0.7963944
Q9BRJ2	0.876	-0.190997225	0.79672946
Q15036	1.271	0.34596403	0.79680939
Q12849	1.15	0.201633861	0.79735018
Q6F5E8	0.814	-0.2968993	0.79768546
Q7Z7H5	0.874	-0.194294815	0.79800811
P62879	1.142	0.191562651	0.79819011
P63172	1.211	0.276198865	0.79819011
Q8IX01	0.862	-0.214240226	0.79834086
P61086	1.136	0.183962835	0.79834086
Q9NSI2	0.88	-0.184424571	0.79865014
F8W0Q9	0.862	-0.214240226	0.79915024
O14776	1.163	0.217851097	0.79915024
J3QRU1	1.171	0.227741076	0.79915024
Q9Y2G5-1	1.329	0.410341105	0.79915024
Q99460	1.136	0.183962835	0.79944936
C9JP00	1.204	0.267835392	0.79981025
Q15836	1.233	0.3021728	0.80015789
Q5R3B4	0.799	-0.323732592	0.80029549
Q8TDB6	1.304	0.38294387	0.80036078
Q9NXR7-1	0.845	-0.242976753	0.80066846
P11717	1.135	0.182692298	0.80082448
Q99733	1.135	0.182692298	0.80087663
E5RGS9	1.248	0.319617934	0.80087663
Q32N00	1.302	0.380729449	0.80087663
O95429	1.328	0.409255147	0.80087663
P06865	0.862	-0.214240226	0.80093052
Q6NXE6	0.883	-0.179514657	0.80105667
P00387	0.892	-0.164884385	0.80173611
Q7Z2W9	0.882	-0.181149439	0.80239227
O43809	1.135	0.182692298	0.80295731
P51665	1.135	0.182692298	0.80348467
Q13671	1.232	0.301002256	0.80348467
Q8IYD1	1.312	0.39176772	0.80411625
P20700	1.134	0.18142064	0.80470781
Q9Y6M7	0.803	-0.316528107	0.80478311
Q9BSR8	0.853	-0.229382353	0.80478311
Q9BT09	0.889	-0.169744676	0.80478311
P30153	1.134	0.18142064	0.80478311
O43684	1.134	0.18142064	0.80478311

P20338	1.221	0.2880632	0.80478311
Q9BV20	1.234	0.303342394	0.80478311
A1X283	1.277	0.352758525	0.80478311
O95202	0.896	-0.158429363	0.80486825
O95168	0.891	-0.166502663	0.80524637
Q9Y6X5	0.852	-0.231074664	0.80554753
Q9NR50	0.896	-0.158429363	0.80554753
Q9UGL1	1.25	0.321928095	0.80557777
P22681	1.256	0.328836464	0.8060429
O60244	0.777	-0.364013496	0.80619309
E7EQ69	0.887	-0.17299399	0.80636457
E9PDV0	0.883	-0.179514657	0.80668524
Q7Z7F7	0.895	-0.160040413	0.80678664
P62917	0.896	-0.158429363	0.80678664
Q9Y676	1.172	0.22897257	0.80678664
Q8IZF6	0.866	-0.20756107	0.80682983
B3KSH1	1.133	0.180147861	0.80682983
Q9UBI6	1.214	0.279768422	0.80682983
Q9NX47	0.895	-0.160040413	0.80684574
P62266	0.896	-0.158429363	0.80684574
O60784	1.156	0.209141398	0.80684574
A8MYT4	1.301	0.379620962	0.80694753
H0YIQ7	1.317	0.397255346	0.80716547
Q9H9J2	0.882	-0.181149439	0.80737123
P34949	0.884	-0.177881725	0.80744315
Q9Y6I9	0.849	-0.236163541	0.80789762
P23193	0.889	-0.169744676	0.80792634
P46782	0.897	-0.15682011	0.80819005
Q9H1C4	0.863	-0.212567535	0.80863084
Q02878	0.897	-0.15682011	0.80872789
Q9H4H8	1.311	0.390667686	0.80891068
Q9NYV6	0.867	-0.205896101	0.80916181
Q86VR2	0.886	-0.174621396	0.80916181
Q8TB61	1.27	0.344828497	0.80916181
P60228	0.897	-0.15682011	0.80930411
Q14344	1.242	0.312665174	0.80957172
P50991	1.132	0.178873958	0.8099258
Q9Y2P8	1.163	0.217851097	0.8099258
Q96J02	1.271	0.34596403	0.8099258
B7ZKS7	1.272	0.347098671	0.8099258
P11802	0.9	-0.152003093	0.81000035
O43148	1.167	0.222804561	0.81000035
Q9UQ13	1.291	0.368489001	0.81033189
O43924	1.243	0.313826296	0.81043699
P30050	0.897	-0.15682011	0.81047075
P49721	0.897	-0.15682011	0.81083118

Q9NQH7	1.241	0.311503115	0.81127239
Q9UL18	0.851	-0.232768963	0.81150363
Q9H1B7	1.198	0.260627908	0.81150363
Q9Y4U1	1.299	0.377401431	0.81150363
Q8N108	1.304	0.38294387	0.81150363
O60220	1.204	0.267835392	0.81195583
Q6P3X3	0.896	-0.158429363	0.81306793
O43159	0.863	-0.212567535	0.81350654
Q2TAL8	1.204	0.267835392	0.81362525
Q9UJ83	0.782	-0.354759487	0.81377234
Q9BTC0	1.177	0.23511432	0.81427814
Q6ZXV5	1.3	0.378511623	0.81427814
Q9UL25	0.89	-0.168122759	0.8143177
Q15075	0.896	-0.158429363	0.81535851
J3KNF4	1.251	0.32308179	0.81571968
P61158	1.13	0.176322773	0.81582484
A6NJ78	0.888	-0.171368418	0.8166199
Q9H2G2	1.152	0.204140717	0.81743404
Q8TEY7	0.765	-0.386468347	0.81798149
Q7LBC6	1.158	0.211635253	0.81798149
Q9UJ70	1.225	0.292781749	0.81798149
O94762	0.765	-0.386468347	0.81813006
Q4J6C6	0.865	-0.209227962	0.81845595
B0UXB6	1.294	0.371837617	0.81845595
Q9Y2I1	0.803	-0.316528107	0.81847033
B4DQJ8	1.129	0.175045486	0.81865785
P49189	0.899	-0.153606979	0.81924326
P49321	1.129	0.175045486	0.81977655
Q9UN86-2	1.129	0.175045486	0.81977655
P01034	0.862	-0.214240226	0.81988313
P28482	1.129	0.175045486	0.81988313
P30154	1.184	0.243669081	0.81988313
B7ZC38	1.225	0.292781749	0.81988313
Q99956	1.234	0.303342394	0.81988313
Q9Y294	1.184	0.243669081	0.82002793
O60502	0.886	-0.174621396	0.82028583
Q32M78	0.845	-0.242976753	0.82072964
Q12965	0.846	-0.241270432	0.82072964
B1ANB7	0.868	-0.204233052	0.82072964
J3KN69	0.894	-0.161653263	0.82072964
Q8NEJ9	1.2	0.263034406	0.82072964
O14936	1.266	0.340277405	0.82072964
B4DLN1	1.128	0.173767068	0.82093504
P35610	1.264	0.337996464	0.82100533
Q6DKK2	0.879	-0.18606493	0.82176833
O14561	0.889	-0.169744676	0.82176833

Q13895	1.129	0.175045486	0.82178273
F8W9A1	1.29	0.367371066	0.8218193
Q13200	1.128	0.173767068	0.82183319
P40121	1.152	0.204140717	0.82183319
O75477	1.134	0.18142064	0.8233937
Q96J01	1.185	0.244887059	0.8233937
J3KNP0	1.236	0.305678743	0.8233937
C9JA52	0.868	-0.204233052	0.82343829
Q15813	1.154	0.206643224	0.82343829
Q9Y6C9	0.901	-0.150400989	0.82344595
P49327	1.127	0.172487516	0.82344595
F5H7N9	0.787	-0.345564459	0.82374428
J3KS22	0.892	-0.164884385	0.82374428
O43617	1.199	0.261831659	0.82374428
G5E954	1.305	0.384049807	0.82374428
P56192	0.901	-0.150400989	0.82387484
E9PHV5	1.132	0.178873958	0.82387484
B3KV61	0.814	-0.2968993	0.82405178
Q9H0A0	0.901	-0.150400989	0.82436266
E7EUI8	0.829	-0.270555993	0.82541439
Q96B26	1.173	0.230203013	0.82556586
Q86YV9	0.829	-0.270555993	0.82576199
Q03135	0.885	-0.17625064	0.8258179
Q13243	1.128	0.173767068	0.8258179
P27348	0.902	-0.148800661	0.82589434
O95169	0.898	-0.15521265	0.82742967
O95630	1.227	0.295135249	0.82742967
Q9H981	0.871	-0.199255376	0.82745317
Q9NYB0	0.895	-0.160040413	0.82755484
Q9UMS4	1.126	0.171206827	0.82755484
B4DR87	1.143	0.192825404	0.82755484
Q9P0J7	1.244	0.314986485	0.82755484
Q96GK7	1.244	0.314986485	0.82755484
Q9HAU5	1.284	0.360645202	0.82755484
D6RB85	0.897	-0.15682011	0.82771102
P61289	1.125	0.169925001	0.82771102
Q9Y6D9	1.245	0.316145742	0.82771102
B5MDF5	1.125	0.169925001	0.82774688
Q68EM7	1.247	0.318461465	0.82787902
Q8IWW6-2	1.271	0.34596403	0.82842983
Q7Z6I6	1.286	0.362890643	0.82842983
B4DY91	0.86	-0.217591435	0.82870225
P46976	0.887	-0.17299399	0.82870225
Q9H2W6	0.893	-0.16326792	0.82870225
O43837	0.896	-0.158429363	0.82870225
Q8NBQ5	1.158	0.211635253	0.82870225

H0Y5T9	1.193	0.254594043	0.82870225
Q9UKA4	1.299	0.377401431	0.82870225
E9PHS3	0.894	-0.161653263	0.82886934
O00233	1.171	0.227741076	0.82886934
Q9NRF9	1.205	0.269033146	0.82886934
G3V5L1	0.774	-0.369594529	0.82889959
Q5JUW8	0.815	-0.295128036	0.82889959
Q92817	0.878	-0.187707155	0.82889959
Q9UQB8	0.891	-0.166502663	0.82889959
Q9BTE7	0.901	-0.150400989	0.82889959
P46459	0.903	-0.147202107	0.82889959
O43665-3	0.905	-0.144010303	0.82889959
Q9NWU1	0.907	-0.140825544	0.82889959
O75367	1.125	0.169925001	0.82889959
H0YBL1	1.125	0.169925001	0.82889959
O14617	1.126	0.171206827	0.82889959
Q9Y3D3	1.149	0.200378798	0.82889959
Q9NQ88	1.166	0.221567789	0.82889959
K7ELG9	1.17	0.22650853	0.82889959
Q13033-2	1.205	0.269033146	0.82889959
Q8NB37	1.282	0.358396262	0.82889959
Q96B13	1.285	0.361768359	0.82889959
Q9Y3C1	0.888	-0.171368418	0.82924015
Q71UM5	0.89	-0.168122759	0.82924015
Q9UK76	0.903	-0.147202107	0.8294578
P03886	0.872	-0.19759996	0.82947537
F8WBK5	0.907	-0.140825544	0.82977196
O95573	0.903	-0.147202107	0.83009051
Q7Z2W4	1.176	0.23388806	0.83049522
Q12904	0.904	-0.145605322	0.83066934
Q8IUH3	0.827	-0.274040765	0.830723
Q2YD98	0.828	-0.272297327	0.830723
Q9H0G5	0.852	-0.231074664	0.830723
P42575	0.873	-0.195946441	0.830723
H7C0N4	1.28	0.35614381	0.830723
B4DJL6	0.868	-0.204233052	0.83072689
Q5VT25	1.299	0.377401431	0.83072689
P50552	1.141	0.190298792	0.83084241
Q13162	0.904	-0.145605322	0.83103553
Q9NQZ2	0.9	-0.152003093	0.83123175
P78347	1.122	0.166072676	0.83123175
F8VZJ2	1.123	0.167357928	0.83123175
P55036	1.125	0.169925001	0.83123175
Q13442	1.144	0.194087052	0.83123175
Q08623	1.193	0.254594043	0.83123175
H7C1E4	1.248	0.319617934	0.83123175

Q9NZ52	1.275	0.350497247	0.83123175
Q99519	1.281	0.357270476	0.83123175
Q9P2N7	1.294	0.371837617	0.83123175
Q96CN9	0.85	-0.234465254	0.83252617
Q8IWC1	0.829	-0.270555993	0.83267095
Q5HYK3	0.863	-0.212567535	0.83267095
P47712	1.159	0.212880566	0.8326958
J3QLH3	1.239	0.309176187	0.83271661
P78316	0.89	-0.168122759	0.83285878
C9JRJ5	1.253	0.325386415	0.8329619
P48643	1.122	0.166072676	0.8330276
Q9UBL3	1.186	0.24610401	0.8330276
P08581	1.273	0.348232419	0.8330276
P14649	0.83	-0.268816758	0.83304044
P17029	0.852	-0.231074664	0.83343583
Q8TC12	1.144	0.194087052	0.83343583
P42025	1.256	0.328836464	0.83343583
O14949	0.902	-0.148800661	0.83381453
E9PEI9	1.183	0.242450074	0.83381453
A8MTH6	0.893	-0.16326792	0.83389634
Q8NFW8	0.858	-0.220950447	0.83401027
P57764	1.166	0.221567789	0.83401027
O75362	1.284	0.360645202	0.83421937
O14828	1.148	0.199122642	0.83451084
P01892	1.143	0.192825404	0.83456947
P82932	1.19	0.250961574	0.83469195
Q9H0W5	1.144	0.194087052	0.83522773
Q9Y263	0.892	-0.164884385	0.83569128
Q8N5C6	0.897	-0.15682011	0.83569128
P10768	0.906	-0.142417045	0.83569128
Q5T5C7	0.906	-0.142417045	0.83569128
P35658	1.126	0.171206827	0.83569128
Q96TC7	0.892	-0.164884385	0.83632798
E9PH88	0.839	-0.253257284	0.83636994
P51571	0.898	-0.15521265	0.83636994
Q14690	0.906	-0.142417045	0.83636994
P60510	1.144	0.194087052	0.83636994
Q6P6C2	1.26	0.333423734	0.83636994
B4DMU4	1.274	0.349365278	0.83636994
Q4G0F5	0.888	-0.171368418	0.83645396
J3KRX5	0.906	-0.142417045	0.83645396
P43307	0.904	-0.145605322	0.83681717
P00390	0.906	-0.142417045	0.83681717
Q15072	1.281	0.357270476	0.83681717
Q9H773	0.899	-0.153606979	0.83750715
Q5T1M5	1.226	0.293958979	0.83782719

E9PKN0	0.791	-0.3382504	0.83804966
O00116	1.134	0.18142064	0.83813524
E9PLK3	0.907	-0.140825544	0.83837183
F8VSL3	0.844	-0.244685096	0.83840329
P19338	0.907	-0.140825544	0.83840329
P62877	0.888	-0.171368418	0.83841385
Q6P158	0.887	-0.17299399	0.83844041
Q9BSC4	0.899	-0.153606979	0.83844041
E9PKF3	0.899	-0.153606979	0.83844041
P13747	1.272	0.347098671	0.83844041
P50583	0.895	-0.160040413	0.83877335
P20290-2	0.877	-0.189351252	0.83929949
Q16795	0.895	-0.160040413	0.83929949
Q9Y285	0.907	-0.140825544	0.83929949
Q9UHD8	1.119	0.162210036	0.83929949
Q9UDY8	1.241	0.311503115	0.83929949
Q9H900	1.27	0.344828497	0.83929949
P61247	0.907	-0.140825544	0.83947659
O15042	0.894	-0.161653263	0.83992626
Q9BW66	1.187	0.247319935	0.83992626
P36873	0.894	-0.161653263	0.84038127
Q8N442	1.217	0.283329168	0.84038127
Q96S82	1.247	0.318461465	0.84038127
P53396	1.119	0.162210036	0.84040132
B8ZZC8	0.856	-0.224317298	0.84104695
J3QLM1	0.889	-0.169744676	0.84104695
Q9BRU9	0.901	-0.150400989	0.84104695
A6NHL2	1.172	0.22897257	0.84104695
P57740	1.123	0.167357928	0.84113388
P08754	0.892	-0.164884385	0.84180055
Q9NUQ3	0.904	-0.145605322	0.84180055
Q96T58	0.912	-0.13289427	0.84180055
Q14966	1.135	0.182692298	0.842008
Q9ULT8	1.126	0.171206827	0.8420594
P46939	0.835	-0.260151897	0.84248036
P21281	0.9	-0.152003093	0.84248036
O43913	0.876	-0.190997225	0.8433664
Q5VTU8	0.9	-0.152003093	0.8433664
Q68D10	0.804	-0.314732593	0.84368769
H3BR29	0.885	-0.17625064	0.84368769
Q9UHY1	0.908	-0.139235797	0.84368769
Q9BRQ8	0.911	-0.134477041	0.84368769
P11940	1.118	0.160920188	0.84368769
Q12765	1.137	0.185232254	0.84368769
Q8WX92	1.148	0.199122642	0.84368769
E7ENA9	1.218	0.284514133	0.84368769

O15294	1.137	0.185232254	0.84371537
Q7Z7E8	1.271	0.34596403	0.84371537
J3KPS0	1.213	0.27857955	0.84374311
Q9NWK9	0.826	-0.275786313	0.84384878
Q969V3	1.131	0.177598929	0.84384878
E5RJI9	0.82	-0.286304185	0.84403856
P15153	0.858	-0.220950447	0.84403856
F5H2F6	0.875	-0.192645078	0.84403856
Q9NY93	0.903	-0.147202107	0.84403856
P42704	0.909	-0.1376478	0.84403856
O75306	0.909	-0.1376478	0.84403856
O95983	0.913	-0.131313235	0.84403856
Q9BRP1	0.914	-0.12973393	0.84403856
B4DY08	1.117	0.159629186	0.84403856
Q86Y07	1.23	0.298658316	0.84403856
O00115	1.245	0.316145742	0.84403856
Q86XN8	1.272	0.347098671	0.84403856
A3KN83	1.29	0.367371066	0.84403856
C9JVN9	0.903	-0.147202107	0.84405736
P28070	0.909	-0.1376478	0.8440741
P20290	1.117	0.159629186	0.84439677
Q8WVV9	1.146	0.196607044	0.84496354
Q9UMY4	0.891	-0.166502663	0.84559352
Q9UMX0	1.116	0.158337027	0.84559352
O95865	1.223	0.290424404	0.84559352
Q96QC0	0.899	-0.153606979	0.84607441
Q9BZL1	0.901	-0.150400989	0.84607441
P61604	0.91	-0.13606155	0.84650506
P20337	1.181	0.240008965	0.84650506
O15514	1.243	0.313826296	0.84650506
Q3ZCW2	0.865	-0.209227962	0.84662736
E9PCR4	1.243	0.313826296	0.84662736
Q6RFH5	0.896	-0.158429363	0.84687577
G8JLB3	0.896	-0.158429363	0.84687577
Q14166	1.116	0.158337027	0.84687577
Q13148	1.116	0.158337027	0.84687577
O14641	1.226	0.293958979	0.84687577
Q15424	1.116	0.158337027	0.84707513
O95232	0.9	-0.152003093	0.84712084
Q9UHV9	0.902	-0.148800661	0.84717531
Q01658	1.212	0.277389699	0.84717531
Q6WRX3-2	0.839	-0.253257284	0.84735795
P62995	0.901	-0.150400989	0.84735795
Q9Y265	0.91	-0.13606155	0.84735795
Q13951	1.242	0.312665174	0.84735795
P40429	0.91	-0.13606155	0.84742767

O43292	1.242	0.312665174	0.8477172
P62310	1.142	0.191562651	0.8477788
Q12874	0.91	-0.13606155	0.84802606
Q9H0A8	1.242	0.312665174	0.84802606
Q96LB3	1.267	0.341416524	0.84802606
P11474	1.26	0.333423734	0.84810797
Q5JPB2	0.839	-0.253257284	0.84842487
Q9Y314	1.149	0.200378798	0.84842487
Q8N0T1	0.8	-0.321928095	0.84862412
Q8IWV8	0.847	-0.239566125	0.84862412
Q96PC3-4	0.847	-0.239566125	0.84862412
O15530	1.29	0.367371066	0.84862412
Q9UDT6	0.794	-0.332789088	0.84935127
Q96CM3	0.84	-0.251538767	0.84935127
B8ZZ43	0.874	-0.194294815	0.84935127
Q96RQ1	0.89	-0.168122759	0.84935127
B4DHE8	0.898	-0.15521265	0.84935127
P23434	0.899	-0.153606979	0.84935127
P53621	0.911	-0.134477041	0.84935127
P51858	0.911	-0.134477041	0.84935127
Q96A33	0.911	-0.134477041	0.84935127
P62841	0.911	-0.134477041	0.84935127
P06737	0.912	-0.13289427	0.84935127
Q7Z2Z2	0.912	-0.13289427	0.84935127
B4DNJ6	1.114	0.155749233	0.84935127
P11166	1.119	0.162210036	0.84935127
O95602	1.129	0.175045486	0.84935127
P62491	1.133	0.180147861	0.84935127
Q92888	1.14	0.189033824	0.84935127
Q6YP21	1.148	0.199122642	0.84935127
F8VV59	1.235	0.304511042	0.84935127
Q8NBM4	1.243	0.313826296	0.84935127
Q9Y5H6	1.274	0.349365278	0.84935127
B5MBX0	0.888	-0.171368418	0.84935482
Q9BZG1	1.146	0.196607044	0.8496003
Q53FA7	1.178	0.236339539	0.84981135
O75691	0.905	-0.144010303	0.85052907
Q9P000	0.903	-0.147202107	0.85122786
P49207	0.912	-0.13289427	0.85123845
Q16595	0.909	-0.1376478	0.85138064
Q9Y224	0.91	-0.13606155	0.85138064
E9PE17	1.163	0.217851097	0.85138064
F8VQZ7	1.113	0.154453593	0.85138604
P49840	1.199	0.261831659	0.85138604
Q99459	1.117	0.159629186	0.85148338
P34931	0.895	-0.160040413	0.85156783

Q4JDL3	1.227	0.295135249	0.85156783
H0YDU8	1.136	0.183962835	0.8517271
B4E2P2	0.912	-0.13289427	0.85182977
P42574	1.256	0.328836464	0.85182977
P49642	1.156	0.209141398	0.85202557
O15226	1.179	0.237563718	0.85202557
Q96JQ2	1.26	0.333423734	0.85237569
O75150	1.15	0.201633861	0.85240905
P49736	1.112	0.153156788	0.85271404
Q76FK4	1.263	0.336854639	0.85271404
Q8TBC4	1.155	0.207892852	0.85325089
Q8TCA0	0.844	-0.244685096	0.85335445
F8W1Z6	0.851	-0.232768963	0.85393573
F8W8S3	0.855	-0.226003675	0.85393573
Q9H6R0	0.894	-0.161653263	0.85393573
Q9UKV8	0.915	-0.128156351	0.85393573
Q15750	1.217	0.283329168	0.85393573
Q9NZJ9	1.221	0.2880632	0.85393573
A6NIW1	1.251	0.32308179	0.85393573
P06756	1.257	0.32998465	0.85468732
Q7Z4V5	1.142	0.191562651	0.85474274
F8W8A6	0.907	-0.140825544	0.85476717
Q7L8W6	1.117	0.159629186	0.85496373
Q7LG56	1.247	0.318461465	0.8549901
O00391	0.878	-0.187707155	0.85516575
Q9UJX6	1.164	0.219091058	0.85531892
P38117	0.909	-0.1376478	0.85544359
Q14697-2	0.914	-0.12973393	0.85557715
Q9UKY7	1.121	0.164786278	0.85580785
Q8IZD4	1.252	0.324234562	0.85580785
Q9H2P0	0.902	-0.148800661	0.85641123
Q9UJA5	1.237	0.3068455	0.85648358
F2Z329	0.798	-0.325539348	0.85677164
Q8NEC7	0.833	-0.263611599	0.85677164
P27448	0.841	-0.249822294	0.85677164
Q9NZD2	0.884	-0.177881725	0.85677164
D6RG18	0.887	-0.17299399	0.85677164
Q9UG56	0.89	-0.168122759	0.85677164
Q9H8H0	0.903	-0.147202107	0.85677164
Q9Y2A7	0.907	-0.140825544	0.85677164
Q9BVQ7	0.908	-0.139235797	0.85677164
P20645	0.908	-0.139235797	0.85677164
Q86Y82	0.913	-0.131313235	0.85677164
O43852	0.914	-0.12973393	0.85677164
O75874	0.914	-0.12973393	0.85677164
P15531	0.914	-0.12973393	0.85677164

P12268	0.915	-0.128156351	0.85677164
P31689	0.915	-0.128156351	0.85677164
P55327	0.915	-0.128156351	0.85677164
Q9NRW3	0.921	-0.118726939	0.85677164
Q96I25	0.922	-0.117161344	0.85677164
Q96AE4-2	1.109	0.149259365	0.85677164
O43707	1.11	0.150559677	0.85677164
Q9HBL7	1.11	0.150559677	0.85677164
F5H365	1.111	0.151858817	0.85677164
P62158	1.111	0.151858817	0.85677164
P08559	1.112	0.153156788	0.85677164
Q9BY44	1.122	0.166072676	0.85677164
P08240	1.13	0.176322773	0.85677164
Q9Y450	1.133	0.180147861	0.85677164
Q5QPM7	1.135	0.182692298	0.85677164
F8WAK8	1.161	0.215367972	0.85677164
Q69YN2	1.167	0.222804561	0.85677164
Q9Y3A3	1.175	0.232660757	0.85677164
Q14318	1.205	0.269033146	0.85677164
Q9NXV2	1.249	0.320773477	0.85677164
Q9H9Q4	1.251	0.32308179	0.85677164
Q49AL5	1.252	0.324234562	0.85677164
J3KS54	1.253	0.325386415	0.85677164
Q86V85	1.263	0.336854639	0.85677164
I3L2A9	1.265	0.339137385	0.85677164
G5E9L8	1.268	0.342554745	0.85677164
Q8WTW3	1.247	0.318461465	0.85764184
Q9NPI6	0.894	-0.161653263	0.85809376
Q96JP5	0.9	-0.152003093	0.85809376
P61923	0.908	-0.139235797	0.85809376
P05198	0.915	-0.128156351	0.85809376
P50613	1.206	0.270229907	0.85809376
Q12774	0.799	-0.323732592	0.85824605
P00918	0.904	-0.145605322	0.85824605
O75818	0.925	-0.112474729	0.85824605
O75530	1.123	0.167357928	0.85824605
Q92882	1.109	0.149259365	0.85836036
Q9UKS6	1.193	0.254594043	0.85836036
Q96MT7	1.246	0.317304068	0.85836036
Q9Y580	1.249	0.320773477	0.85836036
Q00013	1.252	0.324234562	0.85836036
Q9UKI8	1.255	0.327687364	0.85836036
O95453	0.902	-0.148800661	0.85841059
P51648	0.904	-0.145605322	0.85841059
Q9NPA0	0.91	-0.13606155	0.85841059
Q8IUE6	1.108	0.147957881	0.85948598

Q8NBF2	1.147	0.197865391	0.85956439
P62269	0.916	-0.126580497	0.85978518
O95747	1.14	0.189033824	0.85978518
Q16527	1.183	0.242450074	0.85978518
B4DR17	0.84	-0.251538767	0.85979237
Q8N729	1.246	0.317304068	0.86009488
Q969X6	0.905	-0.144010303	0.86012216
Q02880	1.111	0.151858817	0.86018108
Q92734	1.113	0.154453593	0.86142707
Q8N138	0.856	-0.224317298	0.86158175
P00519	0.818	-0.289827252	0.86162044
Q9NZJ6	0.876	-0.190997225	0.86162044
Q5HYI7	0.893	-0.16326792	0.86162044
Q9NVT9	0.9	-0.152003093	0.86162044
P26006	0.903	-0.147202107	0.86162044
Q8TCJ2	0.906	-0.142417045	0.86162044
Q7LBR1	0.911	-0.134477041	0.86162044
Q16537	1.172	0.22897257	0.86162044
O95251	1.213	0.27857955	0.86162044
Q8N6S5	1.247	0.318461465	0.86162044
O43681	0.905	-0.144010303	0.86164742
P22234	0.917	-0.125006361	0.86164742
P07948	0.918	-0.123433941	0.86164742
O60256	1.11	0.150559677	0.86164742
Q16670	1.247	0.318461465	0.86164742
O95807	0.872	-0.19759996	0.86246146
Q96RE7	0.901	-0.150400989	0.86246146
H0YDR3	0.916	-0.126580497	0.86246146
Q8N163	1.107	0.146655222	0.86246146
Q13418	1.142	0.191562651	0.86246146
P15407	1.213	0.27857955	0.86246146
Q92610	0.846	-0.241270432	0.86248369
Q9P016	0.91	-0.13606155	0.86248369
Q9Y5Z4	0.911	-0.134477041	0.86248369
E9PF32	0.912	-0.13289427	0.86248369
O75396	0.917	-0.125006361	0.86248369
Q9Y6B6	0.927	-0.109358756	0.86248369
O75351	1.124	0.168642036	0.86248369
O60888	1.146	0.196607044	0.86248369
P55212	1.201	0.264236151	0.86248369
Q9UID3	1.223	0.290424404	0.86248369
Q96GM8	1.246	0.317304068	0.86248369
O14647	1.265	0.339137385	0.86248369
Q9BZE4	0.917	-0.125006361	0.86250338
P49458	1.109	0.149259365	0.8627687
Q86WB0	1.19	0.250961574	0.8627687

P50914	0.918	-0.123433941	0.86284252
Q9P0U1	1.137	0.185232254	0.86284252
Q96EL3	0.913	-0.131313235	0.86288124
Q99426	1.126	0.171206827	0.86310075
Q13045	1.109	0.149259365	0.8634286
Q53F19	0.917	-0.125006361	0.86361391
O95394	0.918	-0.123433941	0.8637509
O75909	1.216	0.282143229	0.8637509
Q7RTV5	0.804	-0.314732593	0.86378766
C9JXC6	0.846	-0.241270432	0.86378766
B3KY94	1.173	0.230203013	0.86378766
P04066	1.221	0.2880632	0.86378766
O75525	0.85	-0.234465254	0.86402504
E7EVL4	1.235	0.304511042	0.86402504
Q13573	1.12	0.163498732	0.86453376
H0Y6I0	0.814	-0.2968993	0.86474334
C9JWV9	0.856	-0.224317298	0.86474334
A8MWW0	0.86	-0.217591435	0.86474334
B4DZH6	0.886	-0.174621396	0.86474334
P51649	0.903	-0.147202107	0.86474334
Q5JTV8	0.905	-0.144010303	0.86474334
P43897	0.906	-0.142417045	0.86474334
G3V0I5	0.908	-0.139235797	0.86474334
Q9UJZ1	0.919	-0.121863233	0.86474334
Q8N0U8	0.926	-0.110915901	0.86474334
Q9UQ35	1.105	0.14404637	0.86474334
P51398	1.119	0.162210036	0.86474334
J3KPV7	1.128	0.173767068	0.86474334
Q99567	1.145	0.195347598	0.86474334
Q6P597	1.201	0.264236151	0.86474334
Q9Y6G3	1.223	0.290424404	0.86474334
B9A049	1.24	0.310340121	0.86474334
A1XBS5	1.242	0.312665174	0.86474334
F5GWX5	0.919	-0.121863233	0.86476296
P33993	1.105	0.14404637	0.86476296
Q9Y248	1.202	0.265436896	0.86493565
Q9H7B4	0.885	-0.17625064	0.8652016
P26639	0.919	-0.121863233	0.8652016
Q9NVP1	0.919	-0.121863233	0.86535979
C9JLU1	1.117	0.159629186	0.86535979
Q96QD9	0.858	-0.220950447	0.8655534
O60216	1.131	0.177598929	0.8655534
P54727	0.919	-0.121863233	0.86556059
Q9NRW7	0.902	-0.148800661	0.86562538
O94967	0.808	-0.307572802	0.86656231
Q9Y512	0.919	-0.121863233	0.86656231

P36957	0.919	-0.121863233	0.86656231
P62244	0.92	-0.120294234	0.86656231
Q15773	1.136	0.183962835	0.86656231
Q9BVC6	1.162	0.216610069	0.86656231
Q9Y2R0	1.167	0.222804561	0.86656231
Q96HR9	1.21	0.275007047	0.86656231
J3KQU9	0.908	-0.139235797	0.86662451
Q6P2E8	1.214	0.279768422	0.86672844
E9PBB4	0.808	-0.307572802	0.86692036
Q86X02	0.849	-0.236163541	0.86692036
P55735	0.91	-0.13606155	0.86734765
Q9C0C2	0.928	-0.10780329	0.86734765
E9PHY0	1.222	0.289244285	0.86734765
P53609	1.234	0.303342394	0.86734765
C9JZB0	1.239	0.309176187	0.86734765
Q96MG7	0.896	-0.158429363	0.86765133
P09960	0.92	-0.120294234	0.86765133
Q9BV86	1.141	0.190298792	0.86765133
P35914	1.199	0.261831659	0.8677719
Q96CP2	1.206	0.270229907	0.8677719
Q96C23	1.211	0.276198865	0.86781421
P04040	1.107	0.146655222	0.86807086
Q8NAV1	1.185	0.244887059	0.86867123
P51948	1.212	0.277389699	0.86880104
Q8WVY7	1.128	0.173767068	0.86884179
P56589	0.856	-0.224317298	0.868886
O43813	0.922	-0.117161344	0.8695433
O15020	0.883	-0.179514657	0.86963824
Q9H6R4	0.914	-0.12973393	0.86963824
Q9NV06	0.908	-0.139235797	0.86965267
Q9P212	0.903	-0.147202107	0.86969481
P05423	1.256	0.328836464	0.86969481
P39023	0.921	-0.118726939	0.86972087
Q9UKF6	1.123	0.167357928	0.86972087
Q9H814	1.207	0.271425676	0.86987072
E7EP77	0.905	-0.144010303	0.87007735
Q9BYD3	0.91	-0.13606155	0.87007735
P62891	0.915	-0.128156351	0.87007735
D2IYK5	1.241	0.311503115	0.87007735
Q96KM6	1.26	0.333423734	0.87009364
Q9H078-2	1.127	0.172487516	0.87028232
P54725	1.151	0.202887833	0.87083493
E9PR17	1.151	0.202887833	0.87083493
Q5VY60	1.205	0.269033146	0.87083493
P49585	0.915	-0.128156351	0.87102513
Q9H0U9	1.231	0.299830762	0.87102513

Q00653	1.118	0.160920188	0.87119937
P35998	1.102	0.140124224	0.87128483
Q9H6Z4	1.122	0.166072676	0.87128483
C9J7Y4	0.857	-0.222632891	0.87164051
Q9NX62	0.927	-0.109358756	0.87173288
P15586	0.931	-0.103146927	0.87173288
Q5VV41	0.918	-0.123433941	0.87174857
Q15050	1.119	0.162210036	0.87187759
B5MCF9	0.922	-0.117161344	0.87330675
Q9NPL8	0.911	-0.134477041	0.87389499
Q13427	1.175	0.232660757	0.87427892
Q6PCB5	0.829	-0.270555993	0.87435705
Q9UBV8	0.91	-0.13606155	0.87435705
P68402	1.101	0.138814469	0.87487081
P52272	1.101	0.138814469	0.87487144
Q8TEA8	0.922	-0.117161344	0.87489805
B2WTI3	0.909	-0.1376478	0.87503565
O60353	1.233	0.3021728	0.87503565
P54578	1.101	0.138814469	0.87506595
E9PLY5	1.231	0.299830762	0.87506595
G5E9L0	0.91	-0.13606155	0.87540536
B7Z7F3	0.853	-0.229382353	0.87580756
P56385	0.912	-0.13289427	0.87580756
H3BMQ2	0.921	-0.118726939	0.87580756
Q96PK6	0.923	-0.115597447	0.87580756
O95639	1.197	0.259423152	0.87580756
Q9BPY3	1.23	0.298658316	0.87580756
Q9Y376	0.914	-0.12973393	0.87737999
Q8TEX9	1.1	0.137503524	0.87737999
F5H757	1.212	0.277389699	0.87737999
Q6P1J9	0.934	-0.098505545	0.87751902
Q9BV44	1.137	0.185232254	0.87751902
Q8WY21	0.83	-0.268816758	0.87766752
C9JEX3	0.856	-0.224317298	0.87766752
J3QQX3	0.858	-0.220950447	0.87766752
Q8WUD1	0.884	-0.177881725	0.87766752
O00189	0.889	-0.169744676	0.87766752
Q32P44	0.9	-0.152003093	0.87766752
Q5BJD5	0.908	-0.139235797	0.87766752
Q02818	0.91	-0.13606155	0.87766752
Q8WW01	0.91	-0.13606155	0.87766752
P10155	0.913	-0.131313235	0.87766752
O60942	0.913	-0.131313235	0.87766752
O60763	0.923	-0.115597447	0.87766752
Q9UHB9	0.923	-0.115597447	0.87766752
P40926	0.923	-0.115597447	0.87766752

O95831	0.923	-0.115597447	0.87766752
O75251	0.927	-0.109358756	0.87766752
Q8N4N3	0.93	-0.104697379	0.87766752
Q8WUA4	0.933	-0.100051014	0.87766752
P09382	1.1	0.137503524	0.87766752
O15160	1.103	0.141432791	0.87766752
Q9UMS0	1.144	0.194087052	0.87766752
Q9NX08	1.192	0.253384236	0.87766752
Q96C19	0.912	-0.13289427	0.87770067
Q8WZA9	1.193	0.254594043	0.87792883
P13521	1.189	0.249748715	0.87836638
P09417	1.118	0.160920188	0.87844943
Q9Y2H1	0.868	-0.204233052	0.87857704
O75380	0.936	-0.095419565	0.87861169
B7WPF4	1.196	0.25821739	0.87890927
H0YME5	0.909	-0.1376478	0.87906396
P53992	1.104	0.142740172	0.87906396
O15498	1.125	0.169925001	0.87906396
P06744	1.099	0.136191386	0.87920537
Q9Y394	0.933	-0.100051014	0.87943684
Q96RP9	1.127	0.172487516	0.87943684
Q9NVU7	1.17	0.22650853	0.87943684
Q16342	1.224	0.291603558	0.87943684
Q7Z6M1	0.906	-0.142417045	0.87974029
Q9BRK5	0.92	-0.120294234	0.87974029
Q07864	0.925	-0.112474729	0.87974029
G3V1V4	1.189	0.249748715	0.87974029
Q92878	0.903	-0.147202107	0.88001164
P49755	0.924	-0.114035243	0.88001164
F5H2L4	1.136	0.183962835	0.88001164
C9J185	0.869	-0.202571918	0.8802223
Q15561	0.904	-0.145605322	0.8802223
P13073	0.924	-0.114035243	0.8802223
Q8TEB1	0.867	-0.205896101	0.8807537
Q5SRE5	0.919	-0.121863233	0.88088092
Q71TU5	1.116	0.158337027	0.88088901
P49643	1.134	0.18142064	0.8810359
Q92922	1.098	0.134878054	0.88111647
P45954	0.918	-0.123433941	0.88111946
P16383	0.859	-0.219269964	0.8815858
Q8N806	0.924	-0.114035243	0.8815858
P34932	0.925	-0.112474729	0.8815858
Q9Y6G9	1.116	0.158337027	0.8815858
J9JIE6	1.154	0.206643224	0.8815858
Q9H269	1.201	0.264236151	0.8815858
Q9UKV5	1.223	0.290424404	0.8815858

Q01970	1.099	0.136191386	0.88160495
Q8TDJ6	0.927	-0.109358756	0.88168949
Q15149-4	1.222	0.289244285	0.88168949
Q9NY33	0.925	-0.112474729	0.88172464
Q8WUD4	1.198	0.260627908	0.88172464
P38570	1.222	0.289244285	0.88172464
P36542	1.097	0.133563526	0.88218724
Q15024	1.108	0.147957881	0.88218724
P14621	1.194	0.255802837	0.88295772
Q96F63	1.208	0.272620455	0.88304038
P51553	1.13	0.176322773	0.88324348
B9ZVT1	0.93	-0.104697379	0.8832665
Q8N6L1	1.193	0.254594043	0.88440329
Q9C0C9	1.097	0.133563526	0.88473351
P19838	1.14	0.189033824	0.88473351
Q8TDH9	1.238	0.308011315	0.88473351
Q6KC79	1.194	0.255802837	0.88519258
Q9H4L5	1.114	0.155749233	0.88548918
O95302	1.186	0.24610401	0.88548918
P17980	1.096	0.132247798	0.88605293
F5GXX5	1.115	0.15704371	0.88606779
P61088	0.926	-0.110915901	0.88614444
P58546	1.107	0.146655222	0.88614444
P49023	1.132	0.178873958	0.88614444
F8VV52	1.207	0.271425676	0.88614444
Q96S94	1.229	0.297484916	0.88614444
P02545-3	1.238	0.308011315	0.88614444
O43156	1.156	0.209141398	0.8867381
O96033	1.22	0.286881148	0.8867381
P14868	0.926	-0.110915901	0.88674298
Q16186	1.119	0.162210036	0.88691843
Q9UBU9	1.126	0.171206827	0.88691868
Q7L5Y9	1.206	0.270229907	0.88717032
Q6DT37	0.848	-0.23786383	0.88731859
P48739	0.916	-0.126580497	0.88731859
Q9Y315	1.143	0.192825404	0.88731859
O94842	1.193	0.254594043	0.88731859
Q5SY16	1.156	0.209141398	0.88742188
P08579	1.151	0.202887833	0.88759233
Q6PD62	0.916	-0.126580497	0.88774915
P23526	0.927	-0.109358756	0.88774915
P26373	0.927	-0.109358756	0.88774915
P51532-4	1.111	0.151858817	0.88774915
Q12959	1.118	0.160920188	0.88774915
O60287	1.119	0.162210036	0.88774915
Q8WVC0	1.133	0.180147861	0.88774915

Q13619	1.138	0.186500558	0.88790527
O43715	0.875	-0.192645078	0.88810083
O15212	1.119	0.162210036	0.88857357
P30837	1.095	0.13093087	0.88973206
Q9HCD5	1.121	0.164786278	0.88973206
Q9NRY5	1.191	0.252173413	0.88973206
O15121	1.19	0.250961574	0.89014722
Q96SZ6	0.903	-0.147202107	0.89021734
Q8WTS6	0.912	-0.13289427	0.89042513
Q9H0S4	1.095	0.13093087	0.89075849
Q02750	0.923	-0.115597447	0.89084912
Q9NXF1	0.924	-0.114035243	0.89084912
Q03405	1.203	0.266636643	0.89118491
Q9BTV4	1.113	0.154453593	0.89149401
Q8IZ83	1.125	0.169925001	0.89150031
P04179	0.924	-0.114035243	0.89153536
Q9HC38-2	0.928	-0.10780329	0.89161343
P05386	0.928	-0.10780329	0.89161343
B4DP11	0.928	-0.10780329	0.89161343
Q8NEC6	0.872	-0.19759996	0.89181653
P62081	0.928	-0.10780329	0.89181653
F5H6A3	1.207	0.271425676	0.89181653
Q8N357	1.21	0.275007047	0.89181653
Q9HD34	1.215	0.280956314	0.89181653
B4DFK6	0.871	-0.199255376	0.89194521
O43719	0.924	-0.114035243	0.89194521
Q86X29	0.924	-0.114035243	0.89194521
Q9BRD0	1.22	0.286881148	0.89194521
Q15020	0.929	-0.106249498	0.89201818
P25685	1.093	0.128293401	0.89211132
O75821	1.101	0.138814469	0.89211132
Q9UN37	1.168	0.224040274	0.89211132
Q8NEN9	0.852	-0.231074664	0.89284092
C9JJ19	1.14	0.189033824	0.89290121
J3QK90	0.929	-0.106249498	0.89298176
Q01518-2	1.093	0.128293401	0.89298176
Q9UI26	0.918	-0.123433941	0.89316384
P20340-2	0.919	-0.121863233	0.89316384
Q96C01	0.93	-0.104697379	0.89316384
O60749	1.094	0.129612738	0.89316384
Q9H2U1	1.159	0.212880566	0.89316384
Q04917	1.093	0.128293401	0.89342473
Q14258	1.093	0.128293401	0.89346982
P50748	1.211	0.276198865	0.89363204
Q92692	1.216	0.282143229	0.89363204
Q9Y2X3	0.929	-0.106249498	0.893659

Q9UNE7	1.17	0.22650853	0.893659
A8MUM1	1.187	0.247319935	0.893659
J3QKK3	0.908	-0.139235797	0.89397937
Q9Y5Y2	0.941	-0.087733372	0.89397937
Q9UFN0	0.944	-0.083141235	0.89397937
Q9H845	1.121	0.164786278	0.89397937
Q7Z4S6	0.941	-0.087733372	0.89518507
C9JJV1	0.918	-0.123433941	0.89532969
Q9ULX6	1.122	0.166072676	0.89627622
Q9GZR2	1.185	0.244887059	0.89677655
F8WC89	1.212	0.277389699	0.89683473
Q6P275	0.841	-0.249822294	0.897271
G5E9C2	0.868	-0.204233052	0.897271
Q9P0K7	0.871	-0.199255376	0.897271
Q5VZK9	0.871	-0.199255376	0.897271
Q03154	0.871	-0.199255376	0.897271
Q8N766	0.919	-0.121863233	0.897271
Q9NQT5	0.923	-0.115597447	0.897271
Q93034	0.925	-0.112474729	0.897271
Q00839	0.93	-0.104697379	0.897271
E9PCW1	0.932	-0.10159814	0.897271
Q14139	0.943	-0.084670324	0.897271
O43399	1.091	0.125651102	0.897271
P63173	1.091	0.125651102	0.897271
Q8N1F7	1.092	0.126972856	0.897271
P78417	1.092	0.126972856	0.897271
D6RER5	1.093	0.128293401	0.897271
A8MW61	1.101	0.138814469	0.897271
B4E2X3	1.139	0.187767747	0.897271
Q92925	1.147	0.197865391	0.897271
Q9NVV0	1.195	0.257010618	0.897271
E7EWV1	1.206	0.270229907	0.897271
Q5PRF9	1.212	0.277389699	0.897271
Q4LE39	1.214	0.279768422	0.897271
O00291	1.206	0.270229907	0.89740705
Q7Z2T5	0.936	-0.095419565	0.89745964
Q00535	1.161	0.215367972	0.89745964
Q96G46	1.209	0.273814245	0.89757742
Q8N5M9	1.13	0.176322773	0.89767056
Q92538	1.091	0.125651102	0.8977721
Q8IXM3	0.945	-0.081613766	0.89789835
P48637	0.931	-0.103146927	0.89815362
O43347	1.194	0.255802837	0.89832023
O75688	1.185	0.244887059	0.89840998
P10619	1.136	0.183962835	0.89866294
Q6DKI1	0.921	-0.118726939	0.89873522

Q15125	1.185	0.244887059	0.89873522
G3V2S6	0.921	-0.118726939	0.89875171
P23246	0.931	-0.103146927	0.89875171
P21796	1.09	0.124328135	0.89875171
P35637	1.09	0.124328135	0.89875171
Q92917	1.148	0.199122642	0.89875171
Q05193	1.176	0.23388806	0.89875171
Q14657	1.183	0.242450074	0.89875171
P52701	1.106	0.145351386	0.89879821
P61221	0.931	-0.103146927	0.89892977
Q9Y6V7	0.935	-0.09696173	0.89892977
Q9Y5S2	0.942	-0.086201035	0.89892977
Q9BTE3-2	1.09	0.124328135	0.89892977
Q9Y3A6	1.127	0.172487516	0.89892977
O75436	0.925	-0.112474729	0.89899323
P52594	0.937	-0.093879047	0.89899323
Q12800	1.119	0.162210036	0.89991913
Q8NEY1	0.88	-0.184424571	0.90004195
P06576	1.09	0.124328135	0.90004195
P30520	0.926	-0.110915901	0.9001294
O43826	1.203	0.266636643	0.9001294
Q9Y6G5	1.223	0.290424404	0.9001294
O60841	1.09	0.124328135	0.90022323
O75340	0.922	-0.117161344	0.90033325
P26038	1.089	0.123003954	0.90116324
Q8IWA4	1.195	0.257010618	0.90120994
Q13443	0.903	-0.147202107	0.90137644
Q9UII4	0.85	-0.234465254	0.90145333
Q9Y2R9	0.923	-0.115597447	0.90162342
P55010	0.923	-0.115597447	0.90172436
Q969Z0	1.089	0.123003954	0.90253675
P78537	0.92	-0.120294234	0.90269259
P13807	0.926	-0.110915901	0.90286795
P62277	0.932	-0.10159814	0.90286795
O43172	0.931	-0.103146927	0.90300841
Q9H7Z7	0.925	-0.112474729	0.90310155
P14866	1.089	0.123003954	0.90310155
F5H345	1.109	0.149259365	0.90310155
F8VWZ8	1.181	0.240008965	0.90310155
H3BPZ8	1.212	0.277389699	0.90310155
P31751	0.856	-0.224317298	0.9041005
P22102	0.933	-0.100051014	0.9041005
Q9NVI7-2	1.091	0.125651102	0.9041005
Q9NZ63	0.923	-0.115597447	0.90413121
B4E2B6	1.172	0.22897257	0.90413121
Q53S33	0.876	-0.190997225	0.90433812

H0Y5K5	0.894	-0.161653263	0.90433812
P37108	0.933	-0.100051014	0.90433812
Q9NRX2	0.935	-0.09696173	0.90433812
E7EMM4	0.938	-0.092340172	0.90433812
Q9UKV3	1.09	0.124328135	0.90433812
Q9Y2Z0-2	1.096	0.132247798	0.90433812
P51608	1.192	0.253384236	0.90433812
E7EMP9	1.197	0.259423152	0.90443843
Q86WD7-5	1.204	0.267835392	0.90443843
H0YCR6	0.871	-0.199255376	0.90457464
Q8N5L8	0.925	-0.112474729	0.90457464
O95182	0.929	-0.106249498	0.90457464
C9JG97	0.932	-0.10159814	0.90457464
O76071	0.942	-0.086201035	0.90457464
O75531	1.087	0.12035194	0.90457464
P14678	1.088	0.121678557	0.90457464
O95487	1.118	0.160920188	0.90457464
Q9HCU5	1.143	0.192825404	0.90457464
F5H4K0	1.158	0.211635253	0.90457464
O60783	1.18	0.23878686	0.90457464
Q9NVA1	1.183	0.242450074	0.90457464
E9PM12	1.196	0.25821739	0.90457464
H0Y4C3	1.199	0.261831659	0.90457464
Q96AX1	1.205	0.269033146	0.90457464
Q8IYI6	1.207	0.271425676	0.90457464
Q9Y4X0	1.223	0.290424404	0.90457464
Q9Y262	0.934	-0.098505545	0.90481475
P55145	0.934	-0.098505545	0.90481475
P78362	1.175	0.232660757	0.90481475
Q15853	1.199	0.261831659	0.90481475
E7EUN9	0.877	-0.189351252	0.90549585
Q7L014	1.087	0.12035194	0.90556274
P62851	1.087	0.12035194	0.90556274
Q96DB5	0.926	-0.110915901	0.90578882
Q9HD15	0.939	-0.090802937	0.90586243
Q96RS6	1.087	0.12035194	0.90586243
P49454	1.203	0.266636643	0.90586243
O94906	0.929	-0.106249498	0.905967
F8WBK6	0.929	-0.106249498	0.905967
P50224	1.177	0.23511432	0.905967
O95989	0.945	-0.081613766	0.90606535
Q93009	0.935	-0.09696173	0.90664701
F5H012	1.187	0.247319935	0.90664701
Q9NQZ5	1.188	0.248534836	0.90664701
O95470	0.942	-0.086201035	0.90762261
B7Z3Z9	1.189	0.249748715	0.90841743

B4DZN8	0.84	-0.251538767	0.90857932
P12544	0.88	-0.184424571	0.90857932
O60725	0.886	-0.174621396	0.90857932
Q712K3	0.921	-0.118726939	0.90857932
P58557	0.924	-0.114035243	0.90857932
Q13492	0.925	-0.112474729	0.90857932
O15173	0.927	-0.109358756	0.90857932
Q8N128	0.927	-0.109358756	0.90857932
Q7RTV0	0.927	-0.109358756	0.90857932
Q9UIC8	0.928	-0.10780329	0.90857932
O60306	0.931	-0.103146927	0.90857932
Q8IY81	0.935	-0.09696173	0.90857932
P61204	0.935	-0.09696173	0.90857932
P03928	0.941	-0.087733372	0.90857932
E7EW20	1.097	0.133563526	0.90857932
Q04323	1.119	0.162210036	0.90857932
O95235	1.138	0.186500558	0.90857932
O14569	1.141	0.190298792	0.90857932
O75794	1.152	0.204140717	0.90857932
Q9Y6K0	1.189	0.249748715	0.90857932
Q9UK61	1.195	0.257010618	0.90857932
H7BZK6	1.212	0.277389699	0.90857932
Q9Y343	0.878	-0.187707155	0.90902761
G3V1K3	0.926	-0.110915901	0.90902761
Q9H223	0.927	-0.109358756	0.90902761
P00533	0.929	-0.106249498	0.90902761
Q9Y371	0.929	-0.106249498	0.90902761
Q14847	0.93	-0.104697379	0.90902761
Q9Y305	0.934	-0.098505545	0.90902761
P62424	0.936	-0.095419565	0.90902761
P05556	0.936	-0.095419565	0.90902761
P54136	0.937	-0.093879047	0.90902761
P32969	0.937	-0.093879047	0.90902761
P05388	0.937	-0.093879047	0.90902761
B7ZKQ9	0.942	-0.086201035	0.90902761
Q96Q11	0.947	-0.078563669	0.90902761
Q9H3G5	0.947	-0.078563669	0.90902761
Q9BSV6	0.951	-0.072482754	0.90902761
Q86TU7	0.952	-0.070966521	0.90902761
O43583	0.954	-0.067938829	0.90902761
O75534	1.083	0.115033243	0.90902761
P50395	1.084	0.116364757	0.90902761
F5H0L8	1.084	0.116364757	0.90902761
P05387	1.084	0.116364757	0.90902761
Q14204	1.085	0.117695043	0.90902761
Q9NS69	1.085	0.117695043	0.90902761

P48047	1.085	0.117695043	0.90902761
Q9BPW8	1.086	0.119024103	0.90902761
Q8IZL8	1.095	0.13093087	0.90902761
O14974	1.105	0.14404637	0.90902761
Q9UBC2	1.113	0.154453593	0.90902761
Q5JVF3	1.119	0.162210036	0.90902761
Q9Y605	1.151	0.202887833	0.90902761
O95140	1.163	0.217851097	0.90902761
Q96QU8	1.17	0.22650853	0.90902761
Q9NVZ3	1.171	0.227741076	0.90902761
Q9BSJ2	1.172	0.22897257	0.90902761
Q15714-2	1.174	0.231432408	0.90902761
P23497	1.178	0.236339539	0.90902761
Q8WUX9	1.18	0.23878686	0.90902761
Q8IV48	1.183	0.242450074	0.90902761
E7EN96	1.19	0.250961574	0.90902761
Q6UWE0	1.195	0.257010618	0.90902761
Q14191	1.195	0.257010618	0.90902761
B4E243	1.195	0.257010618	0.90902761
Q8N567	1.2	0.263034406	0.90902761
Q9P031	1.201	0.264236151	0.90902761
O96019	1.083	0.115033243	0.90913413
Q9H0C8	0.935	-0.09696173	0.90931583
Q5RGS4	1.171	0.227741076	0.90931583
G8JLF3	0.949	-0.075520008	0.90935422
O00429	1.083	0.115033243	0.90935422
O75643	0.938	-0.092340172	0.91043709
F8WEM2	0.892	-0.164884385	0.91056865
Q9UJS0	0.929	-0.106249498	0.91056865
Q96CN7	0.929	-0.106249498	0.91056865
F5GWY5	0.932	-0.10159814	0.91056865
K7ELM1	0.932	-0.10159814	0.91056865
Q8IVS2	0.936	-0.095419565	0.91056865
Q08379	0.943	-0.084670324	0.91056865
J3KS15	0.953	-0.069451881	0.91056865
Q96E11	0.956	-0.064917477	0.91056865
O14976	1.126	0.171206827	0.91056865
O95571	1.136	0.183962835	0.91056865
Q9UL15	1.159	0.212880566	0.91056865
Q969T9	1.164	0.219091058	0.91056865
A6NDB9	1.169	0.22527493	0.91056865
Q86YD1	1.187	0.247319935	0.91056865
Q9BYT3	1.189	0.249748715	0.91056865
B4DDP2	1.195	0.257010618	0.91056865
P12814	1.082	0.113700499	0.91081527
O43765	0.938	-0.092340172	0.91122262

J9JID7	0.938	-0.092340172	0.91143119
Q15654	1.099	0.136191386	0.91143119
Q7Z5L9	0.928	-0.10780329	0.9114489
O95396	1.168	0.224040274	0.9114489
G3V3G9	1.186	0.24610401	0.9114489
Q9H501	0.942	-0.086201035	0.91149176
Q9BVI4	0.929	-0.106249498	0.91153779
Q9P2E9	0.938	-0.092340172	0.91153779
P06753-2	1.082	0.113700499	0.91153779
Q92643	1.169	0.22527493	0.91153779
Q9P021	1.18	0.23878686	0.91153779
Q9BV38	0.93	-0.104697379	0.91183771
Q13232	0.936	-0.095419565	0.91194323
P31937	0.93	-0.104697379	0.91207337
D6RC56	0.931	-0.103146927	0.91207337
Q15542	1.204	0.267835392	0.91207337
Q96JB2	0.923	-0.115597447	0.91237299
Q96T51	0.93	-0.104697379	0.91237299
P35226	0.934	-0.098505545	0.91237299
Q6P1N9	1.124	0.168642036	0.91237299
G5E975	1.137	0.185232254	0.91237299
Q13309	1.159	0.212880566	0.91237299
C9J8E1	1.18	0.23878686	0.91237299
Q6UX53	1.188	0.248534836	0.91237299
Q9H9L3	1.199	0.261831659	0.91237299
Q96EK9	1.168	0.224040274	0.91268051
P30405	0.935	-0.09696173	0.91270612
Q9Y2D8	0.885	-0.17625064	0.91309533
B1ANE3	0.852	-0.231074664	0.91323867
P50750	1.15	0.201633861	0.91323867
A6PVM9	1.192	0.253384236	0.91323867
P60900	1.081	0.112366523	0.91414984
Q9UMY1	1.136	0.183962835	0.91414984
B4DRT2	0.93	-0.104697379	0.91438904
Q5C9Z4	0.899	-0.153606979	0.91468285
H3BP13	0.9	-0.152003093	0.91468285
Q8NI27	0.934	-0.098505545	0.91468285
P48444	0.939	-0.090802937	0.91468285
Q8NBM8	1.168	0.224040274	0.91499111
P50995	0.94	-0.089267338	0.91500578
Q8NBN3	0.913	-0.131313235	0.91517651
Q9NZE8	0.925	-0.112474729	0.91517651
O95104	0.929	-0.106249498	0.91517651
Q9NPF4	1.171	0.227741076	0.91517651
P62191	1.08	0.111031312	0.91546591
P10515	0.93	-0.104697379	0.91546876

Q5VTR2	1.08	0.111031312	0.91590375
Q9BYG3	0.934	-0.098505545	0.91604039
P49006	1.1	0.137503524	0.91605403
Q9UHD9	1.135	0.182692298	0.91605403
O00161	0.934	-0.098505545	0.91612171
E9PG89	0.855	-0.226003675	0.91620722
Q9NZ45	0.934	-0.098505545	0.91620722
Q8N8R5	0.936	-0.095419565	0.91620722
O75521	0.939	-0.090802937	0.91620722
Q9H0U4	1.09	0.124328135	0.91620722
P78344	0.94	-0.089267338	0.91628795
P62140	1.088	0.121678557	0.91628795
P55795	1.089	0.123003954	0.91628795
G5E9I6	1.142	0.191562651	0.91628795
Q96A19	1.175	0.232660757	0.91628795
I3L3N0	0.89	-0.168122759	0.91665068
P11177	1.08	0.111031312	0.91665068
P48634	1.13	0.176322773	0.91665068
Q9Y3C8	1.161	0.215367972	0.91665068
Q9NRP2	1.171	0.227741076	0.91665068
E7EWN9	0.894	-0.161653263	0.9171223
O60678	0.947	-0.078563669	0.91720323
P29084	1.142	0.191562651	0.91733415
P61024	1.16	0.214124805	0.91733415
O14734	1.201	0.264236151	0.91733415
D6RIE8	0.905	-0.144010303	0.91734012
P20929	0.938	-0.092340172	0.91734012
O60231	0.941	-0.087733372	0.91734012
O95777	1.097	0.133563526	0.91734012
Q99757	1.151	0.202887833	0.91734012
Q14914	1.131	0.177598929	0.91744558
B4DWI3	0.892	-0.164884385	0.91792754
G5E9E7	0.933	-0.100051014	0.91792754
Q8NBU5	0.94	-0.089267338	0.91792754
P15529	1.175	0.232660757	0.91792754
Q9Y5J1	0.936	-0.095419565	0.9179739
Q8WW59	0.94	-0.089267338	0.91804988
Q9BSH4	0.947	-0.078563669	0.91804988
B4DUA9	0.931	-0.103146927	0.91831083
Q5T1J5	0.94	-0.089267338	0.91831083
O00571	1.079	0.109694865	0.91831083
Q08209-2	0.935	-0.09696173	0.91913513
Q9H9Y2	0.941	-0.087733372	0.91913513
Q8NC51-3	1.078	0.108357178	0.91913513
Q9GZP8	1.149	0.200378798	0.91913513
Q96ME1-4	1.187	0.247319935	0.91913513

P13995	1.087	0.12035194	0.91925946
Q96K12	0.933	-0.100051014	0.91927015
Q6P4A7	0.941	-0.087733372	0.91927015
P68104	0.942	-0.086201035	0.91927015
Q16629	0.942	-0.086201035	0.91927015
Q8TAA9	1.173	0.230203013	0.91927015
Q9NUL7	0.939	-0.090802937	0.91928814
Q9UK45	1.11	0.150559677	0.91928814
Q9H270	0.94	-0.089267338	0.91930544
Q96EY1	0.933	-0.100051014	0.91943911
Q96SQ9	0.903	-0.147202107	0.91965038
P00505	0.942	-0.086201035	0.91972498
Q8WVJ2	0.959	-0.06039728	0.91972498
Q15819	1.162	0.216610069	0.91972498
O43929	1.178	0.236339539	0.91972498
P49790	0.932	-0.10159814	0.92033215
Q9BUB7	1.111	0.151858817	0.92033215
Q15172	0.899	-0.153606979	0.92042338
Q15276	0.938	-0.092340172	0.92042338
O00566	0.933	-0.100051014	0.92043703
P03891	0.954	-0.067938829	0.92043703
Q8N8S7	1.09	0.124328135	0.92043703
O15264	1.171	0.227741076	0.92043703
P30084	0.937	-0.093879047	0.92116777
O14493	0.939	-0.090802937	0.92116777
P25054	1.17	0.22650853	0.92116777
Q9H9M0	0.94	-0.089267338	0.92139536
O75113	0.86	-0.217591435	0.92140298
P40616	0.94	-0.089267338	0.92140298
P30566	0.942	-0.086201035	0.92140298
Q9UBI1	0.952	-0.070966521	0.92140298
Q96N67	1.087	0.12035194	0.92140298
P35269	1.091	0.125651102	0.92140298
Q9BVL2	1.113	0.154453593	0.92140298
Q6NUM9	1.18	0.23878686	0.92140298
Q8IW35	1.183	0.242450074	0.92140298
Q96CQ1	0.898	-0.15521265	0.92176783
Q8IVM0	0.907	-0.140825544	0.92176783
H7C3C0	0.941	-0.087733372	0.92176783
O43314	0.942	-0.086201035	0.92176783
J3QRS3	0.943	-0.084670324	0.92176783
P12956	1.077	0.10701825	0.92176783
Q9H4A4	1.091	0.125651102	0.92176783
F8W840	1.184	0.243669081	0.92176783
H0YMW2	1.191	0.252173413	0.92176783
B4DNM9	1.151	0.202887833	0.92181354

Q9P0M6	1.089	0.123003954	0.92261931
P19447	1.161	0.215367972	0.92261931
Q9UNX4	0.94	-0.089267338	0.92286221
P25789	0.943	-0.084670324	0.92286221
O15145	1.092	0.126972856	0.92286221
Q96A49	1.12	0.163498732	0.92286221
Q92558	1.175	0.232660757	0.92286221
Q9HCN4	1.1	0.137503524	0.92317451
Q8NE63	0.857	-0.222632891	0.92322596
Q9GZS3	0.941	-0.087733372	0.92363068
Q5LJA5	0.938	-0.092340172	0.92380125
P62166	0.942	-0.086201035	0.92380125
Q9NXC5	0.908	-0.139235797	0.92476432
Q8N201	1.17	0.22650853	0.92476432
P32119	1.076	0.105678078	0.92509763
Q9UH62	0.937	-0.093879047	0.92535509
A6NIH7	0.952	-0.070966521	0.92535509
Q9NWX6	1.156	0.209141398	0.92540117
Q9UPT8	0.868	-0.204233052	0.9254504
Q99627	0.938	-0.092340172	0.9254504
Q9NZM1-6	0.944	-0.083141235	0.9254504
O95197-3	0.948	-0.077041036	0.9254504
Q9H9A5	0.953	-0.069451881	0.9254504
Q96TA2	0.954	-0.067938829	0.9254504
P21283	1.097	0.133563526	0.9254504
O75146	1.098	0.134878054	0.9254504
Q9Y320	1.153	0.205392513	0.9254504
Q5JXX4	1.16	0.214124805	0.9254504
Q14185	1.169	0.22527493	0.9254504
P84090	1.075	0.10433666	0.92552524
Q15388	0.954	-0.067938829	0.92554303
Q15637-6	1.075	0.10433666	0.92554303
Q15386	0.961	-0.057391664	0.92577103
Q9NX55	1.112	0.153156788	0.92614298
Q99622	1.116	0.158337027	0.92614298
Q9BXY0	0.954	-0.067938829	0.92642187
Q9UK59	1.127	0.172487516	0.92666076
Q7L266	1.174	0.231432408	0.92667768
P61106	0.944	-0.083141235	0.92672286
P09661	1.075	0.10433666	0.92672286
Q15042	1.101	0.138814469	0.92693253
D6RCB9	0.95	-0.074000581	0.92706591
Q96EU6	0.953	-0.069451881	0.92723847
O94927	0.962	-0.055891201	0.92723847
Q9NT62	0.957	-0.06340917	0.92731759
K7EJV9	0.945	-0.081613766	0.92749185

Q16718	0.946	-0.080087911	0.92749185
Q7KZN9	0.952	-0.070966521	0.92749185
P05091	0.94	-0.089267338	0.92769597
O15446	0.945	-0.081613766	0.92769597
Q9BT22	0.955	-0.066427362	0.92769597
Q9H6E4	1.168	0.224040274	0.92769597
Q13432	1.172	0.22897257	0.92769597
Q5T2R2	0.877	-0.189351252	0.92850047
Q14739	0.941	-0.087733372	0.92850047
Q9Y5B9	0.945	-0.081613766	0.92850047
P30622	0.949	-0.075520008	0.92850047
Q5TDG9	0.912	-0.13289427	0.92853416
Q13867	0.94	-0.089267338	0.92853416
Q96ST3	0.942	-0.086201035	0.92853416
P16989	0.945	-0.081613766	0.92853416
Q8IY37	0.951	-0.072482754	0.92853416
P52756	0.953	-0.069451881	0.92853416
E9PC15	0.963	-0.054392297	0.92853416
Q49AN9	1.08	0.111031312	0.92853416
Q8NI22	1.151	0.202887833	0.92853416
P32321	1.16	0.214124805	0.92853416
Q96G25	1.172	0.22897257	0.92853416
Q9UNT1	1.177	0.23511432	0.92853416
P30483	0.937	-0.093879047	0.928561
E5RJ68	0.94	-0.089267338	0.92865607
O00220	0.943	-0.084670324	0.92865607
Q9BQ61	0.963	-0.054392297	0.92865607
P43155	0.946	-0.080087911	0.92868736
Q9ULP9	0.95	-0.074000581	0.92868736
Q8N5N7	0.954	-0.067938829	0.92868736
Q9BVG4	1.091	0.125651102	0.92868736
Q9NWU2	1.093	0.128293401	0.92868736
Q96GG9	1.106	0.145351386	0.92868736
D6RAA5	1.148	0.199122642	0.92868736
Q12899	1.166	0.221567789	0.92868736
P48681	1.174	0.231432408	0.92897636
Q12788	0.946	-0.080087911	0.92908222
Q8N556	0.895	-0.160040413	0.92939065
F8VZR5	0.901	-0.150400989	0.92939065
Q99442	0.945	-0.081613766	0.92939065
G3V5Q3	0.955	-0.066427362	0.92939065
Q68CQ4	0.956	-0.064917477	0.92939065
P62333	1.073	0.101650076	0.92939065
P84098	1.073	0.101650076	0.92939065
Q9Y2Q9	1.141	0.190298792	0.92939065
Q9UPN9-2	1.172	0.22897257	0.92939065

Q9Y4W2	1.112	0.153156788	0.92960262
Q00688	1.087	0.12035194	0.92985747
Q9Y6W5	0.966	-0.049904906	0.92996194
Q9H5K3	0.944	-0.083141235	0.93022873
Q07021	0.947	-0.078563669	0.93026667
Q9C004	1.153	0.205392513	0.93026667
Q9HBH1	1.153	0.205392513	0.93031159
P28331	0.947	-0.078563669	0.93037796
Q8N1G4	1.072	0.100304906	0.93062908
P63208	0.947	-0.078563669	0.93063199
Q9Y5N6	1.151	0.202887833	0.9308454
O43390-2	1.169	0.22527493	0.9308454
F2Z2U4	1.09	0.124328135	0.93120814
O75151	0.943	-0.084670324	0.93134556
Q29RF7	1.081	0.112366523	0.93147524
E7EX17	1.072	0.100304906	0.9316279
P16989-2	0.936	-0.095419565	0.93175531
Q16134	1.159	0.212880566	0.93233998
Q9UL26	0.949	-0.075520008	0.93266236
P18065	0.946	-0.080087911	0.9328503
Q9UPZ3	0.867	-0.205896101	0.93288543
Q76N25	0.877	-0.189351252	0.93288543
Q8WU76	0.906	-0.142417045	0.93288543
Q96QR8	0.911	-0.134477041	0.93288543
A8MTG8	0.914	-0.12973393	0.93288543
Q8IWJ2	0.932	-0.10159814	0.93288543
Q8IUH4	0.94	-0.089267338	0.93288543
Q71RC2	0.941	-0.087733372	0.93288543
Q6AI12	0.942	-0.086201035	0.93288543
Q8IX12	0.945	-0.081613766	0.93288543
Q96GX9	0.945	-0.081613766	0.93288543
Q9BQ95	0.946	-0.080087911	0.93288543
Q92841	0.949	-0.075520008	0.93288543
J3QRU8	0.953	-0.069451881	0.93288543
Q9H5Q4	0.959	-0.06039728	0.93288543
O14562	0.96	-0.058893689	0.93288543
Q9NVV4	0.964	-0.052894948	0.93288543
P63165	1.103	0.141432791	0.93288543
Q9BUE0	1.15	0.201633861	0.93288543
Q8WUA2	0.943	-0.084670324	0.93302832
Q8N122	0.959	-0.06039728	0.93302832
P61978	1.083	0.115033243	0.93302832
Q96GC5	0.954	-0.067938829	0.93348708
Q7Z7H8	1.146	0.196607044	0.93348708
O95295	1.15	0.201633861	0.93348708
O00268	1.159	0.212880566	0.93348708

Q14CX7	0.947	-0.078563669	0.93350703
O95714	0.953	-0.069451881	0.93350703
O95218	1.097	0.133563526	0.93350703
J3KNL6	1.16	0.214124805	0.93350703
A2A2V1	0.931	-0.103146927	0.9335379
H0YCP6	0.94	-0.089267338	0.9335379
P13797	0.949	-0.075520008	0.9335379
E5RI99	1.07	0.097610797	0.9335379
Q9BTV5	1.167	0.222804561	0.9335379
Q9BZE1	0.942	-0.086201035	0.93418734
P35613-3	1.148	0.199122642	0.93488534
Q00403	0.942	-0.086201035	0.93514256
Q86SX6	1.102	0.140124224	0.93528173
P35749	1.094	0.129612738	0.93528889
Q99873-3	1.069	0.096261853	0.93535754
A7YIJ8	1.084	0.116364757	0.93535754
P30048	0.95	-0.074000581	0.93569322
P61457	1.12	0.163498732	0.93626216
J3QL65	0.943	-0.084670324	0.9363417
D6RHI9	0.947	-0.078563669	0.9363417
P61081	0.949	-0.075520008	0.9363417
P49792	0.95	-0.074000581	0.9363417
Q01081	0.95	-0.074000581	0.9363417
P13489	1.069	0.096261853	0.9363417
Q9H329	1.17	0.22650853	0.9363417
P82664	0.96	-0.058893689	0.93645111
Q9H307	1.073	0.101650076	0.93656189
Q8IWK6	0.884	-0.177881725	0.93673349
Q15291	0.973	-0.03948829	0.93673349
Q9UKZ1	0.952	-0.070966521	0.93758142
Q8TEQ6	0.95	-0.074000581	0.93775384
Q9Y5S9	0.95	-0.074000581	0.93795108
Q9NXE4	1.14	0.189033824	0.93810537
Q9Y657	0.96	-0.058893689	0.9384098
Q8WUY1	0.968	-0.046921047	0.93858709
Q9UJX2	0.964	-0.052894948	0.9386823
P46977	1.068	0.094911647	0.93886874
Q15758	0.945	-0.081613766	0.93890661
Q8WVM8	0.947	-0.078563669	0.93911515
Q8WUQ7	1.154	0.206643224	0.93911515
Q96DI7	1.068	0.094911647	0.93929875
Q6WCQ1	1.167	0.222804561	0.93929875
Q9NWT1	0.946	-0.080087911	0.93931406
P20042	1.068	0.094911647	0.93931406
Q9H6S3	1.146	0.196607044	0.93931406
Q9NY99	1.16	0.214124805	0.93931406

G5E9B6	1.086	0.119024103	0.93963514
P62312	0.951	-0.072482754	0.94003724
Q9Y5U9	0.961	-0.057391664	0.94003724
Q9UIJ7	0.957	-0.06340917	0.94028575
Q06124	0.943	-0.084670324	0.94088089
Q86XP3	0.951	-0.072482754	0.94095342
Q12769	1.067	0.093560176	0.94095342
Q09161	1.067	0.093560176	0.94095342
Q9P1U0	1.154	0.206643224	0.94137235
C9JG87	0.948	-0.077041036	0.94138785
F8WE49	1.17	0.22650853	0.94138785
Q92805	0.909	-0.1376478	0.94155746
O60739	0.913	-0.131313235	0.94155746
Q05DH4	0.918	-0.123433941	0.94155746
P32322	0.952	-0.070966521	0.94155746
Q8NB90	1.095	0.13093087	0.94155746
Q9Y2P5	1.157	0.210388864	0.94168614
Q9P270	1.147	0.197865391	0.9418224
P40925	1.067	0.093560176	0.94204527
P53618	0.952	-0.070966521	0.94230959
P52179	0.922	-0.117161344	0.9423495
O60684	0.952	-0.070966521	0.9423495
Q9NVH2	1.152	0.204140717	0.94251192
Q15269	0.947	-0.078563669	0.94288629
Q9UGR2	0.953	-0.069451881	0.94288629
Q08J23	1.066	0.092207438	0.94288629
Q86XI2	1.153	0.205392513	0.94288629
Q92793	0.96	-0.058893689	0.94299193
Q9Y3D6	0.969	-0.045431429	0.94299193
F8W038	1.067	0.093560176	0.94299193
P82673	1.098	0.134878054	0.94299193
Q9BXW9	1.152	0.204140717	0.94299193
Q96G03	1.073	0.101650076	0.94313314
K7EQ21	0.913	-0.131313235	0.94316798
H0YLB5	0.926	-0.110915901	0.94316798
O95292	0.948	-0.077041036	0.94316798
O43318	0.948	-0.077041036	0.94316798
B7Z7P8	0.953	-0.069451881	0.94316798
Q9NZM5	0.954	-0.067938829	0.94316798
P30536	0.955	-0.066427362	0.94316798
P53367	0.959	-0.06039728	0.94316798
Q9NP92	0.976	-0.035046947	0.94316798
J3KP15	1.066	0.092207438	0.94316798
P62993	1.074	0.102993993	0.94316798
Q12888	1.075	0.10433666	0.94316798
Q6FI81	1.075	0.10433666	0.94316798

Q99747	1.139	0.187767747	0.94316798
Q13769	1.144	0.194087052	0.94316798
E9PQJ4	1.154	0.206643224	0.94316798
Q14767	1.156	0.209141398	0.94316798
Q06210	0.953	-0.069451881	0.94323933
B1AMB1	0.896	-0.158429363	0.94336393
Q9Y3B2	0.966	-0.049904906	0.94336393
G5E9X3	0.911	-0.134477041	0.94353868
Q96BH1	0.918	-0.123433941	0.94353868
P15056	0.939	-0.090802937	0.94353868
P52294	0.947	-0.078563669	0.94353868
Q9BXK5	0.951	-0.072482754	0.94353868
O60884	0.952	-0.070966521	0.94353868
Q9NY12	0.956	-0.064917477	0.94353868
Q9Y3A4	0.958	-0.061902439	0.94353868
O15091	0.971	-0.042456799	0.94353868
O75964	1.072	0.100304906	0.94353868
B4DS61	1.08	0.111031312	0.94353868
Q6Y7W6	1.081	0.112366523	0.94353868
Q9NPD8	1.084	0.116364757	0.94353868
Q9NY27	1.098	0.134878054	0.94353868
Q15006	1.122	0.166072676	0.94353868
H7BXZ6	1.144	0.194087052	0.94353868
P0DI82	1.157	0.210388864	0.94353868
O14981	0.95	-0.074000581	0.9436913
Q9HD26	0.968	-0.046921047	0.9436913
O15144	1.064	0.089498151	0.9436913
H3BT71	1.064	0.089498151	0.9436913
E7EN22	1.128	0.173767068	0.9436913
Q9C0J8	1.135	0.182692298	0.9436913
Q8N4T8	0.964	-0.052894948	0.94397816
J3KQG6	0.954	-0.067938829	0.94401903
Q14376	0.948	-0.077041036	0.94443779
P48147	0.954	-0.067938829	0.94443779
Q9Y697	0.968	-0.046921047	0.94443779
P15880	0.954	-0.067938829	0.94470718
F8VVT9	0.948	-0.077041036	0.94476609
P21333	1.064	0.089498151	0.94476609
P32780	1.149	0.200378798	0.94476609
P13196	0.918	-0.123433941	0.94486579
P13804	1.063	0.088141597	0.94486579
Q9NWY4	1.133	0.180147861	0.94486579
Q8IWL3	0.958	-0.061902439	0.9448904
H0Y714	0.959	-0.06039728	0.9448904
Q9BTT0	1.064	0.089498151	0.9448904
Q02978	1.064	0.089498151	0.9448904

Q9UBS8	1.083	0.115033243	0.9448904
Q9Y276	1.137	0.185232254	0.9448904
O15382	0.95	-0.074000581	0.94489078
O75439	0.948	-0.077041036	0.94532968
Q9NPE3	0.979	-0.030619235	0.94536119
Q15046	0.955	-0.066427362	0.94551551
P14550	0.955	-0.066427362	0.94551551
P61224	0.955	-0.066427362	0.94562751
Q9NV31	0.962	-0.055891201	0.94595655
P50402	0.948	-0.077041036	0.94613432
B7Z7F1	1.153	0.205392513	0.94613432
Q92609	1.136	0.183962835	0.94623044
O14893	0.946	-0.080087911	0.94636399
Q15397	0.952	-0.070966521	0.94636399
Q58FG1	0.961	-0.057391664	0.94636399
J3QRU4	0.963	-0.054392297	0.94636399
P61201	1.064	0.089498151	0.94636399
Q5T1Z8	1.074	0.102993993	0.94636399
Q99497	1.062	0.086783766	0.94636686
P17152	1.144	0.194087052	0.94664116
Q9BSH5	1.151	0.202887833	0.94664116
Q969H8	0.956	-0.064917477	0.94665019
Q16762	1.126	0.171206827	0.94665019
P55039	1.127	0.172487516	0.94665019
Q6PK04	1.143	0.192825404	0.94665019
Q9BTA9	1.147	0.197865391	0.94665019
J3KNH7	0.976	-0.035046947	0.94665176
K7EQL6	0.894	-0.161653263	0.94668734
P78345	0.958	-0.061902439	0.94668734
Q8N543	1.119	0.162210036	0.94668734
P06748	0.956	-0.064917477	0.94688431
P05026	0.976	-0.035046947	0.94688431
Q8IWZ3-6	0.951	-0.072482754	0.94699052
P43034	0.956	-0.064917477	0.94699052
Q9UI09	0.963	-0.054392297	0.9476289
E7EX15	0.885	-0.17625064	0.94766741
P49757	0.92	-0.120294234	0.94766741
C9JBI3	0.953	-0.069451881	0.94766741
P08621	1.062	0.086783766	0.94766741
P42285	0.956	-0.064917477	0.94770871
Q9UIU6	0.932	-0.10159814	0.94795916
Q9Y3E2	0.925	-0.112474729	0.94822885
Q04837	0.952	-0.070966521	0.94822885
Q6P4E1	0.949	-0.075520008	0.94829993
Q96B54	1.144	0.194087052	0.94829993
Q9UP83	1.15	0.201633861	0.94856897

H0YIQ8	0.894	-0.161653263	0.94869501
Q9NQW7-3	0.952	-0.070966521	0.94869501
P46087	0.957	-0.06340917	0.94869501
P63272	0.962	-0.055891201	0.94869501
O00488	0.964	-0.052894948	0.94869501
Q14974	1.061	0.085424656	0.94869501
O00232	1.061	0.085424656	0.94869501
Q96HS1	1.073	0.101650076	0.94869501
Q99848	1.075	0.10433666	0.94869501
Q9Y619	1.154	0.206643224	0.94869501
G3V1P5	0.925	-0.112474729	0.94871657
G3V4I5	0.931	-0.103146927	0.94871657
Q7L3T8	0.947	-0.078563669	0.94871657
Q14137	0.958	-0.061902439	0.94871657
Q9NZL9	0.958	-0.061902439	0.94871657
O60499	0.963	-0.054392297	0.94871657
Q5TDF0	0.972	-0.040971781	0.94871657
Q92765	0.972	-0.040971781	0.94871657
Q9H1I8	0.978	-0.03209363	0.94871657
Q8N684	1.06	0.084064265	0.94871657
Q96I24	1.062	0.086783766	0.94871657
O43683	1.066	0.092207438	0.94871657
O75822	1.073	0.101650076	0.94871657
Q9HC36	1.115	0.15704371	0.94871657
O14979-2	1.116	0.158337027	0.94871657
Q9UGP8	1.125	0.169925001	0.94871657
F5H4E4	1.128	0.173767068	0.94871657
Q9BTX1	1.133	0.180147861	0.94871657
A2RRP1	1.143	0.192825404	0.94871657
Q8TAF3	1.152	0.204140717	0.94871657
E7EN86	0.889	-0.169744676	0.94888045
P01024	0.935	-0.09696173	0.94893509
Q9NX02	0.951	-0.072482754	0.94895053
Q96A65	1.136	0.183962835	0.94895053
O60687	0.89	-0.168122759	0.9490268
Q8NFH3	0.961	-0.057391664	0.9490268
O75884	0.965	-0.051399153	0.9490268
P25705	1.059	0.082702589	0.9490268
Q6PJT7	1.059	0.082702589	0.9490268
P62328	1.059	0.082702589	0.9490268
Q9HAN9	1.138	0.186500558	0.9490268
P78356	1.142	0.191562651	0.9490268
E7EXA6	1.144	0.194087052	0.9490268
E5RGS7	1.147	0.197865391	0.9490268
Q14789	1.093	0.128293401	0.94923316
Q9BYD1	0.955	-0.066427362	0.94936776

Q9H553	0.961	-0.057391664	0.94936776
P27695	0.959	-0.06039728	0.94994995
Q13901	0.893	-0.16326792	0.95000964
Q8WXW3	0.901	-0.150400989	0.95000964
Q8IWA0	0.952	-0.070966521	0.95000964
G8JLH6	0.955	-0.066427362	0.95000964
Q5T8P6-2	0.956	-0.064917477	0.95000964
Q2TAY7	0.959	-0.06039728	0.95000964
Q15459	0.96	-0.058893689	0.95000964
Q9BZJ0	0.964	-0.052894948	0.95000964
Q9UBX3	0.967	-0.048412205	0.95000964
Q86VM9	0.968	-0.046921047	0.95000964
P78346	0.972	-0.040971781	0.95000964
Q9BXR0	0.984	-0.023269779	0.95000964
Q15233	1.058	0.081339627	0.95000964
Q9NQC3-2	1.058	0.081339627	0.95000964
P30419	1.058	0.081339627	0.95000964
Q05397	1.058	0.081339627	0.95000964
Q6IN85	1.091	0.125651102	0.95000964
P56556	1.092	0.126972856	0.95000964
Q6UXN9	1.093	0.128293401	0.95000964
P42892	1.117	0.159629186	0.95000964
P38432	1.127	0.172487516	0.95000964
B4DIR7	1.128	0.173767068	0.95000964
Q9BYC8	1.135	0.182692298	0.95000964
Q96L35	1.137	0.185232254	0.95000964
I3L3E4	1.137	0.185232254	0.95000964
E9PH69	1.138	0.186500558	0.95000964
J3QSX6	1.14	0.189033824	0.95000964
O00401	1.142	0.191562651	0.95000964
Q9P260	1.144	0.194087052	0.95000964
O76024	0.974	-0.038006323	0.95022472
Q9UHJ6	1.136	0.183962835	0.95022472
Q14146	0.975	-0.036525876	0.95050677
Q96GQ7	0.953	-0.069451881	0.95056265
P33981	0.933	-0.100051014	0.95082627
O00139-2	0.964	-0.052894948	0.95084562
Q9BXB5	1.142	0.191562651	0.9508642
Q86X83	0.969	-0.045431429	0.95124624
P62487	1.103	0.141432791	0.95124624
P00441	1.057	0.079975377	0.95136352
Q9BR76	0.955	-0.066427362	0.95137334
Q5QP56	0.972	-0.040971781	0.95137334
Q86U38	1.122	0.166072676	0.95137334
P30049	0.956	-0.064917477	0.95168949
Q9UNW1	0.969	-0.045431429	0.95168949

P04075	1.057	0.079975377	0.95168949
Q9NRG1	0.958	-0.061902439	0.95175281
J3QQN7	0.963	-0.054392297	0.95175281
O95456	1.073	0.101650076	0.95175281
Q99598	0.959	-0.06039728	0.95177172
O75674	0.962	-0.055891201	0.95177172
P49915	1.057	0.079975377	0.95177172
Q9H974	1.129	0.175045486	0.95177172
H0YA40	1.143	0.192825404	0.95177172
O76080	1.136	0.183962835	0.95192669
O00629	0.959	-0.06039728	0.95214169
Q9Y639	0.974	-0.038006323	0.95214169
Q9UI10-2	0.977	-0.033569533	0.95214169
Q14669-2	1.058	0.081339627	0.95214169
Q9UJX3	1.085	0.117695043	0.95214169
Q9BW60	1.124	0.168642036	0.95214169
H7C4I6	1.132	0.178873958	0.95214169
Q9UIQ6	0.969	-0.045431429	0.95263747
Q9UNM6	1.056	0.078609835	0.95285529
O43399-3	0.955	-0.066427362	0.95314544
Q9BXJ9	0.961	-0.057391664	0.95314544
Q9BUN8	1.084	0.116364757	0.95314544
Q96FX7	1.132	0.178873958	0.95314544
Q14683	1.056	0.078609835	0.95344262
O15231	0.933	-0.100051014	0.95366854
Q9Y3D0	0.957	-0.06340917	0.95366854
Q99471	0.962	-0.055891201	0.95366854
P49848	0.967	-0.048412205	0.95366854
Q9Y266	1.056	0.078609835	0.95366854
P50452	1.135	0.182692298	0.95366854
Q15121	1.088	0.121678557	0.95376011
Q8IUF8	1.067	0.093560176	0.9538856
Q96ER9	0.97	-0.043943348	0.95396555
Q96II8	0.968	-0.046921047	0.95424123
O43678	0.973	-0.03948829	0.95456559
Q92604	0.914	-0.12973393	0.95458442
P07711	0.933	-0.100051014	0.95458442
Q6DD87	1.124	0.168642036	0.95458442
P23025	1.128	0.173767068	0.95458442
Q9H7D7	0.985	-0.02180437	0.95471523
Q5MNZ6	1.127	0.172487516	0.95485182
Q96GY0	0.969	-0.045431429	0.95492395
O60333	0.957	-0.06340917	0.95538695
P35080	0.983	-0.024736678	0.95538695
Q5T1C6	1.133	0.180147861	0.95538695
F8W7V0	0.912	-0.13289427	0.95554836

Q5VYK3	0.962	-0.055891201	0.95554836
Q7KZI7	0.982	-0.02620507	0.95554836
E9PGM9	1.128	0.173767068	0.95554836
P07305	0.956	-0.064917477	0.95557342
Q99549	0.957	-0.06340917	0.95557342
O00461	0.97	-0.043943348	0.95557342
Q9BY77	1.065	0.09085343	0.95557342
Q5JR08	1.11	0.150559677	0.95557342
Q9BUT1	1.121	0.164786278	0.95557342
Q9UET6	1.127	0.172487516	0.95557342
B4DFC7	1.128	0.173767068	0.95557342
Q6ZWJ1	0.959	-0.06039728	0.95575035
B1APJ0	0.976	-0.035046947	0.95575035
Q96RR1	1.13	0.176322773	0.95575035
P50151	1.13	0.176322773	0.95575035
Q9Y3L5	1.136	0.183962835	0.95575035
Q16587	1.137	0.185232254	0.95575035
J3KMX4	1.138	0.186500558	0.95585885
O43615	0.959	-0.06039728	0.95609238
P49590	0.969	-0.045431429	0.95609238
O15235	0.973	-0.03948829	0.95609238
Q13523	1.066	0.092207438	0.95609238
F2Z3C5	1.125	0.169925001	0.95609238
Q8ND24	1.126	0.171206827	0.95609238
O75394	1.083	0.115033243	0.95609576
Q4G0N4	0.982	-0.02620507	0.95633723
Q9P2B2	1.119	0.162210036	0.95633723
Q15061	0.957	-0.06340917	0.95646607
P43243	1.054	0.075874867	0.95646607
Q7L2J0	1.105	0.14404637	0.95646607
Q15907	0.963	-0.054392297	0.95695773
Q9UBB9	1.076	0.105678078	0.95696963
O43896	0.944	-0.083141235	0.95701346
Q15369	0.964	-0.052894948	0.95701346
Q6PK18	0.971	-0.042456799	0.95701346
Q9H7E9	0.975	-0.036525876	0.95701346
O15305	1.065	0.09085343	0.95701346
Q8TBX8	1.082	0.113700499	0.95701346
P53803	1.121	0.164786278	0.95701346
Q9HAB8	0.958	-0.061902439	0.9572987
P57105	0.989	-0.015957574	0.95752127
Q16531	1.053	0.074505436	0.95764101
Q5JUR7	1.136	0.183962835	0.95764101
O43633	1.12	0.163498732	0.95772448
Q9H2P9	1.121	0.164786278	0.95772448
Q15393	0.964	-0.052894948	0.95790638

P50213	0.964	-0.052894948	0.95815904
P07900	0.964	-0.052894948	0.95830033
Q15526	0.952	-0.070966521	0.95865794
P39656	1.053	0.074505436	0.95865794
Q9UQE7	1.053	0.074505436	0.95877717
Q9NRX1	1.065	0.09085343	0.95877717
Q5TDH0	1.098	0.134878054	0.95877717
Q8N8R3	1.124	0.168642036	0.95877717
Q86W42	1.104	0.142740172	0.95916229
O15479	0.964	-0.052894948	0.95922403
G3V4K3	0.976	-0.035046947	0.9593974
P05141	1.053	0.074505436	0.9593974
F5H8J3	0.98	-0.029146346	0.95953634
Q96K76	0.987	-0.01887801	0.95961154
A2ABE6	0.976	-0.035046947	0.95965284
P57737-3	0.979	-0.030619235	0.95965284
Q9Y678	0.965	-0.051399153	0.96014882
Q96HW7	0.976	-0.035046947	0.96014882
Q9UHD1	1.052	0.073134705	0.96014882
Q9UBW8	0.97	-0.043943348	0.9602416
Q86W50	0.985	-0.02180437	0.9602416
P43487	1.052	0.073134705	0.9602416
Q8NC51	1.118	0.160920188	0.9602416
E7EVG2	1.123	0.167357928	0.9602416
P55789	0.943	-0.084670324	0.96029167
P07814	0.965	-0.051399153	0.96029167
Q08945	0.965	-0.051399153	0.96029167
Q52LJ0-2	0.965	-0.051399153	0.96029167
P51659	0.966	-0.049904906	0.96029167
P08237	0.966	-0.049904906	0.96029167
Q5HYZ1	0.978	-0.03209363	0.96029167
O75369	1.052	0.073134705	0.96029167
Q96C90	1.066	0.092207438	0.96029167
P50895	1.119	0.162210036	0.96029167
Q4G0S7	1.119	0.162210036	0.96029167
Q7Z7K0	1.123	0.167357928	0.96029167
P29474	1.124	0.168642036	0.96029167
J3KPU4	1.128	0.173767068	0.96029167
Q9UQR1	1.133	0.180147861	0.96029167
Q5W0H4	0.966	-0.049904906	0.96036278
Q8IYS2	0.984	-0.023269779	0.96043696
O95707	1.125	0.169925001	0.96046114
Q9Y3T9	0.961	-0.057391664	0.9604627
H3BUD2	0.967	-0.048412205	0.9604627
Q8N0X4	0.973	-0.03948829	0.9604627
Q9Y3I0	1.051	0.071762669	0.9604627

Q16204	1.071	0.09895848	0.9604627
P01116	0.976	-0.035046947	0.96096071
Q15370	0.962	-0.055891201	0.96112205
P18859	1.065	0.09085343	0.96112205
Q969H6	0.946	-0.080087911	0.96146762
Q86XL3	0.979	-0.030619235	0.96146762
Q9H6R7	0.921	-0.118726939	0.96162216
Q6NUK1	0.983	-0.024736678	0.96162216
Q9P2P6	0.987	-0.01887801	0.96162216
Q9UNN8	1.124	0.168642036	0.96162216
Q9NXG6	1.127	0.172487516	0.96162216
Q8IWV7	0.996	-0.005782353	0.96166341
Q8NI60	0.951	-0.072482754	0.96166969
E7EVF4	0.952	-0.070966521	0.96166969
Q14108	0.962	-0.055891201	0.96166969
Q9UNQ2	0.964	-0.052894948	0.96166969
Q5JPE7	1.05	0.070389328	0.96166969
Q86UP2	1.05	0.070389328	0.96166969
Q4TT38	1.078	0.108357178	0.96166969
O43852-4	1.12	0.163498732	0.96166969
P62263	0.967	-0.048412205	0.96176501
P42785	0.981	-0.027674958	0.96176501
F8VRQ1	1.05	0.070389328	0.96176501
Q96P48-1	1.067	0.093560176	0.96176501
E5RIH5	1.122	0.166072676	0.96176501
P19404	0.972	-0.040971781	0.96213288
Q9NY61	0.964	-0.052894948	0.96252331
Q92576	0.979	-0.030619235	0.96252331
O15116	1.092	0.126972856	0.96252331
Q9NUQ7	1.113	0.154453593	0.96256982
Q8WVC6	0.967	-0.048412205	0.9625917
Q9Y4Z0	1.054	0.075874867	0.9625917
Q9UHR6	1.116	0.158337027	0.9625917
Q96D05	0.954	-0.067938829	0.96260257
O15213	1.06	0.084064265	0.96260257
Q9BZX2	0.963	-0.054392297	0.96263537
Q5UCC4	0.965	-0.051399153	0.96263537
Q9BUQ8	0.964	-0.052894948	0.96264868
P32455	0.968	-0.046921047	0.96264868
O60506	1.049	0.069014678	0.96264868
Q9NUU7	1.049	0.069014678	0.96264868
Q53GS9	1.052	0.073134705	0.96264868
Q9Y312	0.994	-0.008682243	0.96307663
E9PHH9	0.989	-0.015957574	0.96327704
Q12929	1.116	0.158337027	0.96327704
A2A2G4	0.961	-0.057391664	0.96355544

E7EQS0	0.963	-0.054392297	0.96355544
P49406	0.965	-0.051399153	0.96355544
P22626	0.968	-0.046921047	0.96355544
P63244	0.968	-0.046921047	0.96355544
Q9UNK0	0.974	-0.038006323	0.96355544
A2RTX5	0.98	-0.029146346	0.96355544
H7BYQ6	0.981	-0.027674958	0.96355544
P52597	1.049	0.069014678	0.96355544
Q15019	1.049	0.069014678	0.96355544
Q13601	1.056	0.078609835	0.96355544
Q86UE4	1.069	0.096261853	0.96355544
Q9UPN9	1.108	0.147957881	0.96355544
Q9UKL0	1.111	0.151858817	0.96355544
Q8NC60	1.115	0.15704371	0.96355544
Q96FV9	1.115	0.15704371	0.96355544
Q9BQG0	1.048	0.067638717	0.96375378
Q6PKG0	0.963	-0.054392297	0.96399006
J3KMY7	0.968	-0.046921047	0.96399006
H7BZH9	0.981	-0.027674958	0.96399006
O95376	0.982	-0.02620507	0.96399006
Q8NB16	0.972	-0.040971781	0.96401967
B4DLH4	1.06	0.084064265	0.9641185
Q32P41	1.09	0.124328135	0.9641185
Q9H583	0.969	-0.045431429	0.96424661
Q14126	1.052	0.073134705	0.96424661
B7ZB17	0.991	-0.013043037	0.96455804
Q86Y56	0.965	-0.051399153	0.96465232
P00403	1.048	0.067638717	0.96465232
Q6JQN1	1.108	0.147957881	0.96465232
Q9BSY4	0.927	-0.109358756	0.96481151
O00764	0.965	-0.051399153	0.96481151
O75947	0.969	-0.045431429	0.96481151
P61964	1.048	0.067638717	0.96481151
P19387	1.071	0.09895848	0.96481151
Q68E01	1.079	0.109694865	0.96481151
Q9H488	1.107	0.146655222	0.96481151
Q96MX6	1.12	0.163498732	0.96481151
F5GZ06	1.122	0.166072676	0.96481151
Q9UK41	1.078	0.108357178	0.9649065
Q9UJW0	1.066	0.092207438	0.96505348
Q68CP9	1.118	0.160920188	0.96505348
O75449	0.938	-0.092340172	0.96530428
P82650	0.966	-0.049904906	0.96530428
J3KTA4	0.969	-0.045431429	0.96530428
Q92636	1.115	0.15704371	0.96530428
Q9NWT8	0.952	-0.070966521	0.96556219

G8JLE5	0.952	-0.070966521	0.96559274
Q9NYB9	0.969	-0.045431429	0.96560617
P08648	0.982	-0.02620507	0.96560617
O60568	1.079	0.109694865	0.96560617
Q9Y4X5	1.099	0.136191386	0.96560617
P09429	0.97	-0.043943348	0.96609254
Q8IYB8	1.087	0.12035194	0.9663639
Q96IZ0	1.108	0.147957881	0.96648119
Q96CS3	0.964	-0.052894948	0.96663076
Q5RI15	0.977	-0.033569533	0.9666564
P14735	0.97	-0.043943348	0.96670488
P00167	0.985	-0.02180437	0.96670488
Q6ZRP7	0.995	-0.007231569	0.96678632
Q9NPF5	1.116	0.158337027	0.96681313
Q8IWZ8	0.958	-0.061902439	0.96689458
J3KPG5	0.973	-0.03948829	0.96689458
B3KTM8	1.072	0.100304906	0.96689458
Q8WXA9-2	1.088	0.121678557	0.96689458
A6NF31	1.107	0.146655222	0.96689458
Q8NCW5	0.966	-0.049904906	0.96727959
O94905	0.983	-0.024736678	0.96727959
Q9P258	1.046	0.064882852	0.96727959
Q13464	1.098	0.134878054	0.96727959
A6NDA1	1.115	0.15704371	0.96727959
P62820	1.046	0.064882852	0.96745812
B0QZ18	1.046	0.064882852	0.9675133
Q13547	1.046	0.064882852	0.96762107
Q10713	0.965	-0.051399153	0.96769302
F5H0R1	0.998	-0.002888279	0.9677868
Q8WYA6	0.967	-0.048412205	0.96784584
E7EM64	1.052	0.073134705	0.96784584
C9K060	1.109	0.149259365	0.96811869
Q96ES7	1.117	0.159629186	0.96811869
O96011	0.953	-0.069451881	0.96835444
O00429-2	0.96	-0.058893689	0.96835444
Q9HB07	0.971	-0.042456799	0.96835444
A8CG34	0.986	-0.020340448	0.96835444
Q92797	1.045	0.063502942	0.96835444
P61981	1.045	0.063502942	0.96835444
O95801	1.051	0.071762669	0.96835444
Q9BZ72	1.075	0.10433666	0.96835444
Q9UBP6	1.11	0.150559677	0.96835444
Q96FX8	0.952	-0.070966521	0.96835556
Q5QJE6	0.967	-0.048412205	0.96835556
P54652	0.983	-0.024736678	0.96835556
Q12830	1.07	0.097610797	0.96835556

Q96ST2	1.074	0.102993993	0.96855626
O75533	0.971	-0.042456799	0.96872898
F8W7C6	0.97	-0.043943348	0.96886296
O00267	0.967	-0.048412205	0.96902587
Q13435	1.045	0.063502942	0.96902587
Q14566	1.045	0.063502942	0.96902587
O00299	1.045	0.063502942	0.96902587
Q9NWH9	1.054	0.075874867	0.96902587
Q92905	1.056	0.078609835	0.96902587
F6U1T9	1.074	0.102993993	0.96902587
Q9H8J5	1.106	0.145351386	0.96902587
Q7Z460	1.107	0.146655222	0.96902587
O75170	1.108	0.147957881	0.96902587
Q7L7X3	1.109	0.149259365	0.96902587
Q96EL2	1.109	0.149259365	0.96902587
P49247	0.986	-0.020340448	0.96914576
Q96HN2	0.958	-0.061902439	0.96918307
F5GYC1	0.967	-0.048412205	0.96918307
P19623	0.969	-0.045431429	0.96918307
Q8TD19	1.073	0.101650076	0.96918307
E7ENJ7	1.104	0.142740172	0.96918307
Q7Z2K8	1.107	0.146655222	0.96928294
Q70CQ2	1.107	0.146655222	0.96932663
P42772	0.954	-0.067938829	0.96937269
Q14240	0.972	-0.040971781	0.96937269
Q04760	0.973	-0.03948829	0.96937269
Q02447-3	0.974	-0.038006323	0.96937269
Q9BUP3-3	0.99	-0.01449957	0.96937269
Q9NR09	0.991	-0.013043037	0.96937269
Q06587	0.993	-0.010134377	0.96937269
O75886	0.994	-0.008682243	0.96937269
P56282	1.005	0.007195501	0.96937269
O14745	1.043	0.060739158	0.96937269
P62318	1.043	0.060739158	0.96937269
P46940	1.044	0.062121712	0.96937269
Q96F86	1.083	0.115033243	0.96937269
P52298	1.084	0.116364757	0.96937269
Q9BV79	1.103	0.141432791	0.96937269
Q9NUG6	1.105	0.14404637	0.96937269
Q8ND82	1.111	0.151858817	0.96937269
B5MDU6	1.113	0.154453593	0.96937269
O43396	0.973	-0.03948829	0.96969408
P42677	1.043	0.060739158	0.96969408
Q9BWJ5	1.054	0.075874867	0.96969408
Q9GZQ8	1.11	0.150559677	0.96969408
Q9Y6N1	1.115	0.15704371	0.96969408

Q3LXA3	0.975	-0.036525876	0.96972139
G3V2U7	1.078	0.108357178	0.96972139
P34897	0.973	-0.03948829	0.97010301
P31153	0.973	-0.03948829	0.97010301
B4E241	0.973	-0.03948829	0.97010301
O76003	1.051	0.071762669	0.97010301
E9PC97	1.082	0.113700499	0.97010301
P20339	1.084	0.116364757	0.97010301
O15047	1.084	0.116364757	0.97010301
Q8NEM2	1.105	0.14404637	0.97010301
P46781	0.973	-0.03948829	0.97021556
Q9NTK5	0.974	-0.038006323	0.97021556
Q7Z6E9	0.998	-0.002888279	0.97021556
Q9P2J8	0.931	-0.103146927	0.97055766
Q9H4G0	0.962	-0.055891201	0.97055766
P09525	0.974	-0.038006323	0.97055766
Q9H5V9	0.985	-0.02180437	0.97055766
Q06546	1.092	0.126972856	0.97055766
F6W7K9	1.101	0.138814469	0.97055766
Q8WZA0	1.105	0.14404637	0.97055766
Q9Y2S0	0.995	-0.007231569	0.97068643
Q5T0Y8	0.96	-0.058893689	0.97070583
Q12962	0.966	-0.049904906	0.97070583
Q9H0B6	0.972	-0.040971781	0.97070583
Q9UL63	0.988	-0.017417053	0.97070583
H7C4S8	0.992	-0.011587974	0.97070583
P22314	1.042	0.059355278	0.97070583
P17655	1.042	0.059355278	0.97070583
Q8WWQ0	1.107	0.146655222	0.97070583
Q96L91	1.107	0.146655222	0.97070583
Q9BQ52	0.971	-0.042456799	0.97075727
P17252	0.957	-0.06340917	0.97082378
Q4U2R6	0.991	-0.013043037	0.97082378
O75153	1.042	0.059355278	0.97082378
F8WDY7	1.107	0.146655222	0.97082378
P27635	0.974	-0.038006323	0.97084712
O75506	0.97	-0.043943348	0.97094212
Q9Y5N5	0.971	-0.042456799	0.97094212
P13010	0.974	-0.038006323	0.97094212
Q8N2K0	0.978	-0.03209363	0.97094212
Q9HD33	0.983	-0.024736678	0.97094212
P78527	1.042	0.059355278	0.97094212
Q9UKM9	1.042	0.059355278	0.97094212
F5H157	1.08	0.111031312	0.97094212
Q53FT3	1.103	0.141432791	0.97094212
Q9NRL2	1.105	0.14404637	0.97094212

Q9Y333	1.052	0.073134705	0.9712479
Q5VV50	0.925	-0.112474729	0.97127645
Q9Y496	0.938	-0.092340172	0.97127645
Q9NYL2-2	0.978	-0.03209363	0.97127645
E9PRJ8	0.981	-0.027674958	0.97127645
Q9BRA2	1.051	0.071762669	0.97127645
Q9P2B4	1.104	0.142740172	0.97127645
Q86VN1	0.963	-0.054392297	0.97137642
P25098	0.967	-0.048412205	0.97137642
B0V043	0.975	-0.036525876	0.97137642
E7ES33	0.975	-0.036525876	0.97137642
Q9P253	0.991	-0.013043037	0.97137642
O00151	1.045	0.063502942	0.97137642
Q9H074	1.047	0.066261442	0.97137642
P60983	1.051	0.071762669	0.97137642
Q7Z6V5	1.111	0.151858817	0.97137642
O95772	0.958	-0.061902439	0.9715888
Q92522	1.062	0.086783766	0.9715888
O75569	1.066	0.092207438	0.9715888
P35573	0.983	-0.024736678	0.9716042
H0YJV7	1.102	0.140124224	0.97164027
Q8N983-4	0.971	-0.042456799	0.97180209
Q8NC56	1.001	0.001441974	0.97180209
O15371	1.041	0.057970069	0.97180209
B0QYK0	1.041	0.057970069	0.97180209
Q9ULV4	1.041	0.057970069	0.97180209
H3BPE1	1.043	0.060739158	0.97180209
P05114	1.101	0.138814469	0.97180209
C9IYK9	1.103	0.141432791	0.97180209
Q96D53	0.97	-0.043943348	0.97222621
B7Z4Q3	0.972	-0.040971781	0.97222621
Q9BYD6	0.972	-0.040971781	0.97222621
Q15427	0.972	-0.040971781	0.97222621
Q969N2	0.992	-0.011587974	0.97222621
O43464	0.992	-0.011587974	0.97222621
Q9H0P0	0.997	-0.00433459	0.97222621
P35579	1.04	0.056583528	0.97222621
P49720	1.04	0.056583528	0.97222621
B4DT77	1.042	0.059355278	0.97222621
Q32MZ4-2	1.043	0.060739158	0.97222621
F8W9T0	1.08	0.111031312	0.97222621
Q9BRR6	1.096	0.132247798	0.97222621
P01023	1.098	0.134878054	0.97222621
B1AK53	1.106	0.145351386	0.97222621
Q9H3K6	0.976	-0.035046947	0.97238177
B4DDF9	1.098	0.134878054	0.97292746

Q8IYL3	0.951	-0.072482754	0.97314423
Q9NZJ0	0.961	-0.057391664	0.97314423
H0Y450	0.962	-0.055891201	0.97314423
Q96MW5	0.966	-0.049904906	0.97314423
Q8NC96	0.971	-0.042456799	0.97314423
Q9BVK6	0.973	-0.03948829	0.97314423
Q01085-2	0.974	-0.038006323	0.97314423
Q9NUQ8	0.974	-0.038006323	0.97314423
Q00169	0.976	-0.035046947	0.97314423
Q9NQ55	0.985	-0.02180437	0.97314423
O15111	0.986	-0.020340448	0.97314423
Q13356	0.992	-0.011587974	0.97314423
Q8WUX2	0.993	-0.010134377	0.97314423
Q16775	0.998	-0.002888279	0.97314423
E5RGN3	1.011	0.015782997	0.97314423
P12081	1.039	0.055195654	0.97314423
O14818	1.039	0.055195654	0.97314423
G3V1V0	1.039	0.055195654	0.97314423
P20674	1.039	0.055195654	0.97314423
P78371	1.04	0.056583528	0.97314423
O00487	1.04	0.056583528	0.97314423
P43362	1.04	0.056583528	0.97314423
Q99436	1.04	0.056583528	0.97314423
Q16836	1.047	0.066261442	0.97314423
Q9UBB4	1.049	0.069014678	0.97314423
P47813	1.049	0.069014678	0.97314423
Q9Y5L4	1.053	0.074505436	0.97314423
P51116	1.056	0.078609835	0.97314423
Q9C0E2	1.056	0.078609835	0.97314423
P82921	1.067	0.093560176	0.97314423
Q5VZL5	1.068	0.094911647	0.97314423
Q9BTX3	1.097	0.133563526	0.97314423
Q96I51	1.098	0.134878054	0.97314423
P21953	1.099	0.136191386	0.97314423
H3BQQ2	1.104	0.142740172	0.97314423
Q9P2N5	1.105	0.14404637	0.97314423
Q14141	1.108	0.147957881	0.97314423
Q86WJ1	1.109	0.149259365	0.97314423
Q03518	1.105	0.14404637	0.97330032
O14657	0.966	-0.049904906	0.97347777
J3QQW2	1.104	0.142740172	0.97349921
Q96SK2-2	0.998	-0.002888279	0.97367689
G3XAG2	0.966	-0.049904906	0.97374019
Q9Y324	0.97	-0.043943348	0.97374019
P09012	0.972	-0.040971781	0.97374019
Q9BX68	0.989	-0.015957574	0.97374019

H9KV59	0.996	-0.005782353	0.97374019
I3L2J8	0.998	-0.002888279	0.97374019
P36954	1.003	0.004321606	0.97374019
P82979	1.038	0.053806444	0.97374019
P50570	1.039	0.055195654	0.97374019
Q14651	1.047	0.066261442	0.97374019
Q96EB6	1.101	0.138814469	0.97374019
Q7Z5Y7	0.954	-0.067938829	0.97400535
Q8N3D4	0.965	-0.051399153	0.97400535
Q9Y584	0.973	-0.03948829	0.97400535
P35250	0.974	-0.038006323	0.97400535
Q9UBQ5	0.975	-0.036525876	0.97400535
Q9BVM2	0.975	-0.036525876	0.97400535
J3KMX9	0.976	-0.035046947	0.97400535
E7ETB3	0.976	-0.035046947	0.97400535
Q13242	0.976	-0.035046947	0.97400535
O76021	0.978	-0.03209363	0.97400535
P62854	0.978	-0.03209363	0.97400535
Q13247	0.978	-0.03209363	0.97400535
Q8WYQ5	0.981	-0.027674958	0.97400535
F8W9R9	0.993	-0.010134377	0.97400535
P51452	1.001	0.001441974	0.97400535
Q8IXU6	1.002	0.002882509	0.97400535
Q13505-3	1.004	0.005759269	0.97400535
Q9H2D1	1.006	0.008630305	0.97400535
Q15029	1.038	0.053806444	0.97400535
P51991	1.038	0.053806444	0.97400535
Q92575	1.075	0.10433666	0.97400535
Q13610	1.075	0.10433666	0.97400535
C9JE98	1.085	0.117695043	0.97400535
Q5TA45	1.092	0.126972856	0.97400535
Q8NBF6	1.093	0.128293401	0.97400535
F5H721	1.095	0.13093087	0.97400535
B4DWZ5	1.096	0.132247798	0.97400535
P0CAP1-4	1.101	0.138814469	0.97400535
Q8N843	1.101	0.138814469	0.97400535
Q92466	1.102	0.140124224	0.97400535
P16885	1.008	0.011495639	0.97411116
Q15645	1.045	0.063502942	0.97411116
Q8N0Z6	1.101	0.138814469	0.97423519
Q9NQX4	0.973	-0.03948829	0.97425176
Q9P003	1.094	0.129612738	0.97425176
P48426	1.047	0.066261442	0.97432007
P51570	0.975	-0.036525876	0.97432803
O75608	1.062	0.086783766	0.97432803
Q9H3Z4	1.093	0.128293401	0.97432803

P62701	1.037	0.052415894	0.97445858
P08047	1.093	0.128293401	0.97451179
Q14562	1.045	0.063502942	0.97493633
O00255	0.969	-0.045431429	0.97496874
Q96BP3	1.011	0.015782997	0.97496874
P24928	1.037	0.052415894	0.97496874
J3KQN7	1.002	0.002882509	0.97535534
Q05639	1.037	0.052415894	0.97535534
Q9Y6E2	1.037	0.052415894	0.97535534
Q9H2H8	1.037	0.052415894	0.97535534
Q96EP5	1.046	0.064882852	0.97535534
Q96FJ2	0.979	-0.030619235	0.97587622
Q9NXV6	1.002	0.002882509	0.97626448
P20618	1.036	0.051024003	0.97626448
Q6IS24	1.102	0.140124224	0.97626448
Q16891	0.98	-0.029146346	0.97631855
Q8IUD2	0.998	-0.002888279	0.97631855
Q9NZW5	1.01	0.014355293	0.97631855
Q9UNH7	1.047	0.066261442	0.97631855
Q969S3	1.058	0.081339627	0.97631855
P23142	1.1	0.137503524	0.97631855
P67936	0.98	-0.029146346	0.97636679
P49756	0.98	-0.029146346	0.97646327
Q99496	0.991	-0.013043037	0.97646327
Q9NTJ5	0.978	-0.03209363	0.97685427
Q8TED1	0.999	-0.001443417	0.97691327
P07384	0.98	-0.029146346	0.97716484
Q6P1L8	1.012	0.01720929	0.97752118
Q8NEZ5	1.069	0.096261853	0.97766374
O43164	1.102	0.140124224	0.97766374
Q99615	0.98	-0.029146346	0.97805617
O60508	0.992	-0.011587974	0.97840461
P49902	0.977	-0.033569533	0.97852085
Q9UKN8	0.978	-0.03209363	0.97852085
Q5VVR8	0.978	-0.03209363	0.97852085
P10620	0.979	-0.030619235	0.97852085
B4DTG6	0.993	-0.010134377	0.97852085
Q96DA6	1.003	0.004321606	0.97852085
Q8WTT2	1.037	0.052415894	0.97852085
O75934	1.041	0.057970069	0.97852085
Q9UGV2	1.062	0.086783766	0.97852085
H0YMB4	1.072	0.100304906	0.97852085
A6NFI3	1.075	0.10433666	0.97852085
C9JSR1	1.087	0.12035194	0.97852085
G8JLK4	1.091	0.125651102	0.97852085
Q9Y5R8	1.091	0.125651102	0.97852085

Q3B726	1.094	0.129612738	0.97852085
O95674	1.096	0.132247798	0.97852085
Q9P2D1	1.097	0.133563526	0.97852085
Q9H4G4	1.098	0.134878054	0.97852085
Q6NW34	1.1	0.137503524	0.97852085
Q8NI36	0.981	-0.027674958	0.97859264
Q6ZN16	1.044	0.062121712	0.97859264
F8VUA2	1.084	0.116364757	0.97883219
Q12846	1	0	0.97885706
P37235	1.059	0.082702589	0.97885706
Q92499	0.981	-0.027674958	0.97932533
Q92541	1.003	0.004321606	0.97932533
Q99570	1.091	0.125651102	0.97932533
Q8WUM4	0.982	-0.02620507	0.97947679
Q9BU76	1.095	0.13093087	0.97949327
H0YAA3	0.954	-0.067938829	0.97983954
Q7Z3T8	0.977	-0.033569533	0.97983954
Q8N3C0	0.982	-0.02620507	0.97983954
Q9HBL8	1.002	0.002882509	0.97983954
P00492	1.034	0.048236186	0.97983954
H3BV80	1.042	0.059355278	0.97983954
Q15057	1.093	0.128293401	0.97983954
Q15797	1.093	0.128293401	0.97983954
Q9P2X3	1.096	0.132247798	0.97983954
Q9NQ48	1.056	0.078609835	0.98001014
Q96F24	1.087	0.12035194	0.98003192
Q9NWV4	1.088	0.121678557	0.98005058
P41208	1.072	0.100304906	0.98015843
O43252	1.034	0.048236186	0.98032328
O00505	1.039	0.055195654	0.98052119
Q9ULR0	1.003	0.004321606	0.98071465
P21397	1.089	0.123003954	0.98077766
B3KS98	0.982	-0.02620507	0.98175188
Q15418	1.093	0.128293401	0.98175253
P31150	0.982	-0.02620507	0.98177784
Q9BQP7	1.089	0.123003954	0.98179397
Q92620	1.055	0.077242999	0.98195682
O75208	1.009	0.012926174	0.98199334
Q9NX58	1.034	0.048236186	0.98199334
Q13123	1.04	0.056583528	0.98199334
Q9BXT8-1	1.093	0.128293401	0.98199334
Q9Y5B0	1.007	0.010063683	0.98211247
P42345	0.98	-0.029146346	0.98234891
Q6P2P2	1.073	0.101650076	0.98234891
Q9NXF7	1.086	0.119024103	0.98234891
F5H594	0.979	-0.030619235	0.98244215

Q9Y3B7	0.98	-0.029146346	0.98244215
A8MYA6	0.982	-0.02620507	0.98244215
Q5T4S7	0.983	-0.024736678	0.98244215
P83731	0.983	-0.024736678	0.98244215
O94804	1.005	0.007195501	0.98244215
A6NDU8	1.005	0.007195501	0.98244215
Q9NX20	1.006	0.008630305	0.98244215
P02545	1.033	0.046840254	0.98244215
Q9BZZ5-2	1.033	0.046840254	0.98244215
Q9NWZ5	1.081	0.112366523	0.98244215
Q9BYC9	1.005	0.007195501	0.98255846
Q9Y4W6	0.98	-0.029146346	0.98263661
Q9Y2Q3	0.983	-0.024736678	0.98263661
Q9UNS2	0.984	-0.023269779	0.98263661
Q86WR0	1.017	0.024319679	0.98263661
Q9Y3B4	0.986	-0.020340448	0.982963
P04406	1.032	0.045442971	0.982963
O43237	1.052	0.073134705	0.982963
P82914	1.006	0.008630305	0.98296358
P07237	1.032	0.045442971	0.98296358
Q9Y277	1.032	0.045442971	0.98296358
Q9UNF0	1.032	0.045442971	0.98296358
Q10567-3	1.035	0.049630768	0.98296358
Q6IA86	1.041	0.057970069	0.98296358
Q6IPR3	1.081	0.112366523	0.98296358
J3KNC0	1.086	0.119024103	0.98296358
Q13131	1.088	0.121678557	0.98296358
P51970	1.058	0.081339627	0.98305445
Q5W111	1.08	0.111031312	0.98305445
Q14152	0.984	-0.023269779	0.98308834
P30086	0.984	-0.023269779	0.98308834
Q86V48	1.085	0.117695043	0.98311075
O75190	1.007	0.010063683	0.98326366
Q16512-2	1.041	0.057970069	0.98332664
Q9NQT4	0.996	-0.005782353	0.98384153
J3KPW7	0.98	-0.029146346	0.98398571
Q96KQ7	0.999	-0.001443417	0.98398571
P55196-1	0.982	-0.02620507	0.98444341
Q5T6W5	0.984	-0.023269779	0.98444341
Q7L2H7	0.985	-0.02180437	0.98444341
Q92665	0.994	-0.008682243	0.98444341
Q14571	0.997	-0.00433459	0.98444341
Q86UL3	1.009	0.012926174	0.98444341
P16615	1.031	0.044044333	0.98444341
Q9HAV7	1.031	0.044044333	0.98444341
Q13363	1.037	0.052415894	0.98444341

Q5UIP0	1.052	0.073134705	0.98444341
Q9UBB6	1.068	0.094911647	0.98444341
Q96KG9	1.077	0.10701825	0.98444341
Q658Y4	1.084	0.116364757	0.98444341
Q8N4N8	1.087	0.12035194	0.98444341
Q15637-5	0.999	-0.001443417	0.9845471
Q8WXF0	1	0	0.9845471
Q9NYT0	1	0	0.9845471
P06746	1.007	0.010063683	0.9845471
O94952-1	1.009	0.012926174	0.9845471
Q5SQP8	1.038	0.053806444	0.9845471
Q86YS7	1.077	0.10701825	0.9845471
H7C173	1.078	0.108357178	0.9845471
Q9UJY4	1.085	0.117695043	0.9845471
Q9UMX5	1.006	0.008630305	0.98512438
Q9UQ80	0.985	-0.02180437	0.98513975
Q9P035	0.982	-0.02620507	0.98532186
Q96TA1	0.985	-0.02180437	0.98532186
O60566	1.089	0.123003954	0.98532186
Q9Y4I1	0.982	-0.02620507	0.9854617
Q86X55	0.987	-0.01887801	0.9854617
Q9NW82	1.085	0.117695043	0.98563589
O43819	1.012	0.01720929	0.98570317
P26368	1.03	0.042644337	0.98570317
Q8TCU4	1.076	0.105678078	0.98570317
Q6UXV4	0.988	-0.017417053	0.98575409
J3QR07	1.011	0.015782997	0.98581699
Q96LJ7	0.978	-0.03209363	0.98605183
P52888	0.981	-0.027674958	0.98605183
Q16563-2	0.985	-0.02180437	0.98605183
P10606	0.986	-0.020340448	0.98605183
G5EA09	1	0	0.98605183
P26599	1.03	0.042644337	0.98605183
Q9H9Q2	1.036	0.051024003	0.98605183
O96000	1.038	0.053806444	0.98605183
Q96L34	1.08	0.111031312	0.98605183
Q92688	0.986	-0.020340448	0.98615568
B1AK64	0.986	-0.020340448	0.98615568
P31327	1.011	0.015782997	0.98615568
J3KTL2	1.029	0.041242982	0.98615568
Q8NFH4	1.036	0.051024003	0.98615568
P01033	1.078	0.108357178	0.98615568
Q9NVC6	1.086	0.119024103	0.98615568
P25786	0.986	-0.020340448	0.98626523
Q8NFV4	1.007	0.010063683	0.98626523
P19388	1.08	0.111031312	0.98629094

P61019	1.029	0.041242982	0.98637197
Q9C035	0.975	-0.036525876	0.98672956
Q9NYL9	0.984	-0.023269779	0.98672956
O76031	0.986	-0.020340448	0.98672956
P09874	0.987	-0.01887801	0.98672956
Q13310	0.987	-0.01887801	0.98672956
P62136	0.987	-0.01887801	0.98672956
P29401	0.988	-0.017417053	0.98672956
P51149	0.988	-0.017417053	0.98672956
Q04721	0.988	-0.017417053	0.98672956
Q03701	0.99	-0.01449957	0.98672956
Q13823	0.99	-0.01449957	0.98672956
Q16740	0.997	-0.00433459	0.98672956
Q8IW92	0.997	-0.00433459	0.98672956
Q709C8	1.001	0.001441974	0.98672956
Q96EY5	1.001	0.001441974	0.98672956
Q86U44	1.003	0.004321606	0.98672956
P20248	1.011	0.015782997	0.98672956
F5GWW9	1.012	0.01720929	0.98672956
Q9P265	1.015	0.021479727	0.98672956
Q8NHQ9	1.018	0.025737561	0.98672956
Q9BRG1	1.019	0.027154052	0.98672956
Q9NPJ6	1.02	0.028569152	0.98672956
P42765	1.022	0.031395196	0.98672956
G3V5T0	1.023	0.032806145	0.98672956
Q8WUM0	1.027	0.038436182	0.98672956
P11387	1.028	0.039840265	0.98672956
P35606	1.029	0.041242982	0.98672956
Q14103	1.029	0.041242982	0.98672956
P39019	1.029	0.041242982	0.98672956
P53007	1.031	0.044044333	0.98672956
Q12996	1.032	0.045442971	0.98672956
A8KAH5	1.036	0.051024003	0.98672956
P48059-2	1.064	0.089498151	0.98672956
O94888	1.07	0.097610797	0.98672956
Q8TCF1	1.073	0.101650076	0.98672956
H0Y5B0	1.079	0.109694865	0.98672956
J3KNN5	1.079	0.109694865	0.98672956
Q16540	1.079	0.109694865	0.98672956
G3V3E8	1.081	0.112366523	0.98672956
E7ERH3	1.081	0.112366523	0.98672956
Q8WVX9	1.083	0.115033243	0.98672956
O00186	1.085	0.117695043	0.98672956
I3L1R7	1.086	0.119024103	0.98672956
Q9H7Z6	1.087	0.12035194	0.98672956
F8W8D3	1.013	0.018634174	0.98717381

F6WCX7	0.974	-0.038006323	0.98718017
A6NN80	1.028	0.039840265	0.98722064
Q9H3H3	1.082	0.113700499	0.98724925
P08865	0.988	-0.017417053	0.98730076
P85299-5	1.016	0.022900402	0.98730076
Q9BXP5	1.027	0.038436182	0.9873271
O43290	0.988	-0.017417053	0.98742783
Q9BVJ6	0.991	-0.013043037	0.98742783
Q15004	1.021	0.029982866	0.98742783
B4DJ85	1.024	0.034215715	0.98742783
O15427	1.036	0.051024003	0.98742783
Q96SB3	1.053	0.074505436	0.98742783
D6RE58	1.069	0.096261853	0.98742783
P15151	1.079	0.109694865	0.98742783
H3BV68	1.079	0.109694865	0.98742783
P53582	0.985	-0.02180437	0.98746729
G3V325	0.985	-0.02180437	0.98774965
O75844	1.033	0.046840254	0.98774965
Q6IC75	0.987	-0.01887801	0.98779856
Q86U42	0.988	-0.017417053	0.98792604
E7ETK0	0.989	-0.015957574	0.98792604
Q8IXQ4	0.993	-0.010134377	0.98792604
Q9HCG8	1.001	0.001441974	0.98792604
B7Z6G2	1.002	0.002882509	0.98792604
J3KPP7	1.013	0.018634174	0.98792604
P53701	1.014	0.020057652	0.98792604
Q13153	1.017	0.024319679	0.98792604
P09211	1.026	0.037030731	0.98792604
P29692-3	1.026	0.037030731	0.98792604
Q6L8Q7	1.026	0.037030731	0.98792604
P37837	1.027	0.038436182	0.98792604
Q9HC07	1.029	0.041242982	0.98792604
Q9Y5X3	1.032	0.045442971	0.98792604
P40938	1.034	0.048236186	0.98792604
Q96FQ6	1.035	0.049630768	0.98792604
K7ELQ8	1.05	0.070389328	0.98792604
Q8WWC4	1.062	0.086783766	0.98792604
B5MCN5	1.068	0.094911647	0.98792604
B4E0K5	1.07	0.097610797	0.98792604
P49356	1.073	0.101650076	0.98792604
Q9BQ04	1.075	0.10433666	0.98792604
E9PMQ6	1.075	0.10433666	0.98792604
O43402	1.077	0.10701825	0.98792604
C9J7N1	1.078	0.108357178	0.98792604
Q96B23-2	1.082	0.113700499	0.98792604
Q9NP77	1.021	0.029982866	0.9880196

C9JIX4	0.993	-0.010134377	0.98804031
E9PQY2	0.998	-0.002888279	0.98804031
P00846	1.034	0.048236186	0.98804031
Q9H4A5	1.075	0.10433666	0.98804031
P18615	1.082	0.113700499	0.98804031
B1AJY7	0.987	-0.01887801	0.98887252
P06280	1.082	0.113700499	0.98887252
Q9C037	1.017	0.024319679	0.98888695
Q5T3I0	1.026	0.037030731	0.98888695
Q5T5C0	0.959	-0.06039728	0.98923863
P22087	0.99	-0.01449957	0.98923863
Q96T60	1.014	0.020057652	0.98923863
Q9NRR3	1.017	0.024319679	0.98923863
P62304	1.026	0.037030731	0.98923863
Q4G0I0	1.072	0.100304906	0.98948591
P51580	1.025	0.03562391	0.98960866
P29966	1.035	0.049630768	0.98960866
O75116	1.03	0.042644337	0.98964963
P17612	1.033	0.046840254	0.98964963
H0YMY5	1.08	0.111031312	0.98964963
Q8TEA1	0.963	-0.054392297	0.98991137
E7EVA0	0.99	-0.01449957	0.98991137
Q09028	0.99	-0.01449957	0.98991137
Q9UPQ8	0.995	-0.007231569	0.98991137
Q04941	1.025	0.03562391	0.98991137
Q13136	1.075	0.10433666	0.98991137
Q96LW4	1.078	0.108357178	0.98991137
Q9BYD2	1.045	0.063502942	0.990143
Q96EY7	0.991	-0.013043037	0.99016067
Q13098	0.993	-0.010134377	0.99016067
Q9NVH1	0.995	-0.007231569	0.99016067
O95905	0.995	-0.007231569	0.99016067
P35813	0.999	-0.001443417	0.99016067
Q5J9I4	1.02	0.028569152	0.99016067
Q8WUH6	1.021	0.029982866	0.99016067
Q5TFE4	1.024	0.034215715	0.99016067
P63104	1.025	0.03562391	0.99016067
O95817	1.026	0.037030731	0.99016067
Q8WYP5	1.046	0.064882852	0.99016067
Q9H993	1.047	0.066261442	0.99016067
F5H2M4	1.069	0.096261853	0.99016067
J3QQQ9	1.074	0.102993993	0.99016067
Q13445	1.074	0.102993993	0.99016067
Q96CU9	1.074	0.102993993	0.99016067
O94903	1.037	0.052415894	0.99047111
Q7KZ85	1.026	0.037030731	0.99057321

O00148	0.989	-0.015957574	0.99071708
P31948	0.991	-0.013043037	0.99071708
Q99613	0.991	-0.013043037	0.99071708
P46778	0.991	-0.013043037	0.99071708
P62249	0.991	-0.013043037	0.99071708
Q96EE3	0.994	-0.008682243	0.99071708
Q9NS86	0.996	-0.005782353	0.99071708
Q16222	0.997	-0.00433459	0.99071708
Q5JSZ5	1	0	0.99071708
P22413	1	0	0.99071708
G5E928	1	0	0.99071708
Q9NYP9	1	0	0.99071708
Q86YN1	1	0	0.99071708
C9J236	1	0	0.99071708
P48507	1.002	0.002882509	0.99071708
E5RIK9	1.002	0.002882509	0.99071708
Q99828	1.003	0.004321606	0.99071708
P35754	1.006	0.008630305	0.99071708
Q5T6F2	1.009	0.012926174	0.99071708
J3KNN7	1.019	0.027154052	0.99071708
Q96GN5	1.02	0.028569152	0.99071708
D6RD48	1.021	0.029982866	0.99071708
Q7Z6Z7	1.023	0.032806145	0.99071708
Q9BUJ2	1.023	0.032806145	0.99071708
O95816	1.023	0.032806145	0.99071708
Q5QNW6	1.023	0.032806145	0.99071708
Q9H267	1.028	0.039840265	0.99071708
Q8WWY3	1.029	0.041242982	0.99071708
Q9Y3B9	1.029	0.041242982	0.99071708
E7ETA6	1.045	0.063502942	0.99071708
Q9NRF8	1.05	0.070389328	0.99071708
Q9UPY3	1.054	0.075874867	0.99071708
F5GZX4	1.056	0.078609835	0.99071708
Q14UF6	1.066	0.092207438	0.99071708
F5H8A0	1.07	0.097610797	0.99071708
Q9Y5J9	1.071	0.09895848	0.99071708
Q92615	1.072	0.100304906	0.99071708
F8W8Q9	1.072	0.100304906	0.99071708
H3BQP3	1.072	0.100304906	0.99071708
P13929	1.073	0.101650076	0.99071708
Q4G0J3	1.073	0.101650076	0.99071708
O94919	1.075	0.10433666	0.99071708
O95551	1.078	0.108357178	0.99071708
P18433	0.966	-0.049904906	0.99075349
Q3ZCQ8	0.993	-0.010134377	0.99075349
P86790	1	0	0.99075349

Q9BVV7	1.024	0.034215715	0.99075349
Q7Z478	1.026	0.037030731	0.99075349
P09132	1.066	0.092207438	0.99075349
Q6ZN55	1.074	0.102993993	0.99075349
B4DFQ4	0.998	-0.002888279	0.99085801
Q86UD0	0.984	-0.023269779	0.99097747
P40937	0.99	-0.01449957	0.99097747
Q9NQW6	0.991	-0.013043037	0.99097747
O95372	0.992	-0.011587974	0.99097747
P09110	0.993	-0.010134377	0.99097747
Q9NQG5	0.993	-0.010134377	0.99097747
P48556	0.993	-0.010134377	0.99097747
O95861	0.993	-0.010134377	0.99097747
D3YTB1	0.993	-0.010134377	0.99097747
Q06323	0.994	-0.008682243	0.99097747
Q13155	0.994	-0.008682243	0.99097747
O43795-2	0.995	-0.007231569	0.99097747
O14979	0.995	-0.007231569	0.99097747
P68431	0.995	-0.007231569	0.99097747
Q9Y295	0.997	-0.00433459	0.99097747
A6NCK0	0.998	-0.002888279	0.99097747
Q9NPD3	0.998	-0.002888279	0.99097747
P49257	0.999	-0.001443417	0.99097747
Q9UKG1	1	0	0.99097747
Q9BQE9	1	0	0.99097747
Q6ZN44	1	0	0.99097747
Q4G0Z9-4	1.003	0.004321606	0.99097747
G3V4A5	1.005	0.007195501	0.99097747
E9PBC1	1.005	0.007195501	0.99097747
Q9NUQ6	1.006	0.008630305	0.99097747
Q8WU90	1.006	0.008630305	0.99097747
Q7L5D6	1.009	0.012926174	0.99097747
Q92572	1.009	0.012926174	0.99097747
Q9H444	1.009	0.012926174	0.99097747
Q9BW85	1.013	0.018634174	0.99097747
Q8NF37	1.018	0.025737561	0.99097747
O75792	1.018	0.025737561	0.99097747
Q08211	1.02	0.028569152	0.99097747
P62258	1.02	0.028569152	0.99097747
P07737	1.02	0.028569152	0.99097747
P47985	1.02	0.028569152	0.99097747
Q6IAA8	1.02	0.028569152	0.99097747
Q9BSJ8	1.021	0.029982866	0.99097747
P25787	1.021	0.029982866	0.99097747
Q15102	1.021	0.029982866	0.99097747
P06730	1.021	0.029982866	0.99097747

O60563	1.021	0.029982866	0.99097747
O00625	1.021	0.029982866	0.99097747
P00338	1.022	0.031395196	0.99097747
O00159	1.022	0.031395196	0.99097747
P28074	1.022	0.031395196	0.99097747
P25788	1.022	0.031395196	0.99097747
P60953	1.022	0.031395196	0.99097747
Q9BQC3	1.022	0.031395196	0.99097747
O95825	1.023	0.032806145	0.99097747
P31431	1.023	0.032806145	0.99097747
O95479	1.024	0.034215715	0.99097747
P19784	1.025	0.03562391	0.99097747
Q9UKM7	1.025	0.03562391	0.99097747
Q99590	1.025	0.03562391	0.99097747
Q9Y5B8	1.025	0.03562391	0.99097747
P82912	1.026	0.037030731	0.99097747
Q9H871	1.026	0.037030731	0.99097747
P50542	1.027	0.038436182	0.99097747
Q15067	1.029	0.041242982	0.99097747
Q92597	1.029	0.041242982	0.99097747
Q08AM6	1.029	0.041242982	0.99097747
P53365	1.031	0.044044333	0.99097747
P52435	1.031	0.044044333	0.99097747
P83876	1.035	0.049630768	0.99097747
F6QR24	1.038	0.053806444	0.99097747
Q5T440	1.038	0.053806444	0.99097747
Q96BW5	1.045	0.063502942	0.99097747
B7ZBY5	1.048	0.067638717	0.99097747
Q9UPN7	1.055	0.077242999	0.99097747
Q70Z53	1.057	0.079975377	0.99097747
I3L2L5	1.058	0.081339627	0.99097747
Q58DX5	1.059	0.082702589	0.99097747
Q96S52	1.062	0.086783766	0.99097747
Q4KMP7	1.065	0.09085343	0.99097747
O76095	1.065	0.09085343	0.99097747
O60774	1.065	0.09085343	0.99097747
Q2TA77	1.067	0.093560176	0.99097747
E7EPD9	1.067	0.093560176	0.99097747
P23511	1.068	0.094911647	0.99097747
E9PAZ2	1.068	0.094911647	0.99097747
Q7Z4Q2	1.069	0.096261853	0.99097747
Q9GZP9	1.07	0.097610797	0.99097747
Q8N999	1.07	0.097610797	0.99097747
P33552	1.07	0.097610797	0.99097747
O95926	1.072	0.100304906	0.99097747
Q14149	1.074	0.102993993	0.99097747

Q9H788	1.075	0.10433666	0.99097747
P43003	1.075	0.10433666	0.99097747
Q96JQ0	1.075	0.10433666	0.99097747
E7EQ95	1.076	0.105678078	0.99097747
Q15363	1	0	0.9910309
O96008	1.02	0.028569152	0.9910309
P24539	1.02	0.028569152	0.99125102
O15066	1.018	0.025737561	0.99131059
Q16626	1.072	0.100304906	0.99131059
P61970	1.02	0.028569152	0.9914537
F8VQ10	0.995	-0.007231569	0.99147003
Q8TBA6	1.028	0.039840265	0.99147003
Q12986	1	0	0.99148293
O60573	1.059	0.082702589	0.99148293
O95248	0.973	-0.03948829	0.9915204
E9PGZ1	1.026	0.037030731	0.9915204
H0YLZ8	1.024	0.034215715	0.99158839
O95433	0.996	-0.005782353	0.99192327
P62979	0.996	-0.005782353	0.99197934
Q9NZ56	1.028	0.039840265	0.99197934
P28066	0.996	-0.005782353	0.99202047
Q8IV08	1.01	0.014355293	0.99202047
P25398	1.019	0.027154052	0.99208183
P00558	0.996	-0.005782353	0.99241156
P63220	0.996	-0.005782353	0.99241156
O94916	0.996	-0.005782353	0.99241156
Q9Y6M1	0.997	-0.00433459	0.99241156
Q00325-2	0.997	-0.00433459	0.99241156
Q16778	0.997	-0.00433459	0.99241156
Q9Y520	1.001	0.001441974	0.99241156
O94776	1.018	0.025737561	0.99241156
Q86V81	1.019	0.027154052	0.99241156
Q9NTZ6	1.02	0.028569152	0.99241156
Q9Y3A2	1.031	0.044044333	0.99241156
O43301	1.032	0.045442971	0.99241156
Q9Y2U8	1.037	0.052415894	0.99241156
Q86YP4	1.042	0.059355278	0.99241156
Q9Y399	1.049	0.069014678	0.99241156
Q96B36	1.061	0.085424656	0.99241156
Q9BVC4	1.062	0.086783766	0.99241156
Q16706	1.063	0.088141597	0.99241156
Q86WA6	1.064	0.089498151	0.99241156
A2A2L5	1.064	0.089498151	0.99241156
Q5JTJ3	1.065	0.09085343	0.99241156
Q9Y6A5	1.068	0.094911647	0.99241156
P62253	1.068	0.094911647	0.99241156

Q14790	1.071	0.09895848	0.99241156
Q9BXK1	1.071	0.09895848	0.99241156
P24666	1.018	0.025737561	0.99246143
O95721	1.055	0.077242999	0.99246143
Q8IUI8	1.056	0.078609835	0.99246143
Q9H9A7	1.058	0.081339627	0.99246143
Q7L3B6	1.068	0.094911647	0.99246143
Q9H7X3	1.02	0.028569152	0.99258709
P24386	1.027	0.038436182	0.99260745
Q9BTY2	1.067	0.093560176	0.99269013
H0YNE9	1.069	0.096261853	0.99269013
B4DKT0	1.001	0.001441974	0.99284611
Q9UL46	0.997	-0.00433459	0.99290237
Q13630	0.998	-0.002888279	0.99290237
P62699	1	0	0.99290237
F5H315	1.013	0.018634174	0.99290237
Q9Y6W3	1.032	0.045442971	0.99290237
Q9H1E3	1.06	0.084064265	0.99290237
O75746	1.031	0.044044333	0.99304319
E9PC74	1.012	0.01720929	0.99338044
Q96KR1	1.018	0.025737561	0.99338044
P08708	0.998	-0.002888279	0.99338386
Q9NRY4	1.056	0.078609835	0.99338386
Q16543	0.998	-0.002888279	0.99349107
O14980	1.018	0.025737561	0.99349107
Q12792	0.998	-0.002888279	0.99389778
Q96QK1	1.017	0.024319679	0.99389778
Q96JM3	1.065	0.09085343	0.99389778
P35232	0.998	-0.002888279	0.9939915
Q8NCA5	1.006	0.008630305	0.9939915
P18583-5	1.016	0.022900402	0.9939915
Q9Y2T2	1.022	0.031395196	0.9939915
P10301	1.033	0.046840254	0.9939915
P50454	0.998	-0.002888279	0.99411611
Q5SSJ5	0.998	-0.002888279	0.99418586
Q93008-1	0.998	-0.002888279	0.99418586
Q14207	1	0	0.99418586
F8W9S7	1.017	0.024319679	0.99418586
O95155	1.067	0.093560176	0.99443041
O00471	1.03	0.042644337	0.994498
Q9UKD2	1.017	0.024319679	0.99472178
P62829	0.999	-0.001443417	0.99476327
Q8IYB3	1	0	0.99476327
Q5T091	1	0	0.99476327
O43379-2	1.015	0.021479727	0.99476327
P61586	1.017	0.024319679	0.99476327

P62316	1.017	0.024319679	0.99476327
P57076	1.034	0.048236186	0.99476327
J3QRY2	1.036	0.051024003	0.99476327
B5MC67	1.05	0.070389328	0.99476327
P67775	1.057	0.079975377	0.99476327
B7Z355	1.058	0.081339627	0.99476327
O75319	1.059	0.082702589	0.99476327
Q15814	1.063	0.088141597	0.99476327
Q5SZ82	1.065	0.09085343	0.99476327
Q5T200	1.066	0.092207438	0.99476327
P04843	0.999	-0.001443417	0.99524361
Q92616	1.016	0.022900402	0.99524361
P55884	1.016	0.022900402	0.99524361
Q9H2M9	1.046	0.064882852	0.99524361
Q8IY17	1.06	0.084064265	0.99524361
Q9Y230	0.999	-0.001443417	0.99550298
O75976	1	0	0.99550298
Q96T88	1.015	0.021479727	0.99550298
P22307	1.018	0.025737561	0.99550298
H0Y2P2	1.018	0.025737561	0.99550298
P42695	1.031	0.044044333	0.99550298
Q8NB28	1.033	0.046840254	0.99550298
Q9ULW3	1.038	0.053806444	0.99550298
C9JC74	1.045	0.063502942	0.99550298
Q13572	1.05	0.070389328	0.99550298
H3BT91	1.058	0.081339627	0.99550298
Q9Y5A7	1.059	0.082702589	0.99550298
Q9NRL3	1.062	0.086783766	0.99550298
Q15008	1	0	0.99553296
Q5JRI3	1	0	0.99553296
Q8WVE0	1.01	0.014355293	0.99553296
P35249	1.015	0.021479727	0.99553296
Q9Y3D9	1.016	0.022900402	0.99553296
Q9H0W9	1.028	0.039840265	0.99553296
Q9BZE9	1.033	0.046840254	0.99553296
Q8TC07-2	1.004	0.005759269	0.99562455
P23396	1	0	0.99574122
P49748	1.015	0.021479727	0.99574122
P30041	1.015	0.021479727	0.99574122
C9JPL0	1.032	0.045442971	0.99574122
Q96AY3	1.035	0.049630768	0.99574122
Q14997	1.061	0.085424656	0.99591389
O15270	1.015	0.021479727	0.9960454
Q14573	0.997	-0.00433459	0.99606705
Q9BUB1	0.998	-0.002888279	0.99606705
Q96D46	0.999	-0.001443417	0.99606705

C9JPT4	1	0	0.99606705
Q8IXH7	1.001	0.001441974	0.99606705
O43504	1.013	0.018634174	0.99606705
Q96IX5	1.014	0.020057652	0.99606705
P11586	1.015	0.021479727	0.99606705
P62906	1.015	0.021479727	0.99606705
Q9BXL6	1.018	0.025737561	0.99606705
P60520	1.033	0.046840254	0.99606705
P41223	1.037	0.052415894	0.99606705
H0YH87	1.039	0.055195654	0.99606705
E9PG40	1.044	0.062121712	0.99606705
Q9NYV4	1.044	0.062121712	0.99606705
C9JTZ6	1.047	0.066261442	0.99606705
P48723	1.061	0.085424656	0.99606705
Q9BVP2	0.999	-0.001443417	0.99635566
P46783	1.014	0.020057652	0.99635566
Q06330	1.058	0.081339627	0.99635566
P04844	1.001	0.001441974	0.99649112
P45974-2	1.014	0.020057652	0.99649112
A6ND99	1.055	0.077242999	0.99649112
E7ENA2	1.057	0.079975377	0.99649112
Q96B49	1.034	0.048236186	0.99650661
Q16401	1.001	0.001441974	0.99678813
A5YKK6	1.014	0.020057652	0.99678813
Q9HAV4	1.014	0.020057652	0.99686005
P31323	1.055	0.077242999	0.99692717
Q13347	1.014	0.020057652	0.99700154
Q8IV50	1.03	0.042644337	0.99700154
Q9H0L4	1.033	0.046840254	0.99700154
B1AT46	1.039	0.055195654	0.99700154
Q8IZ81	1.042	0.059355278	0.99700154
Q9Y608	1.042	0.059355278	0.99700154
Q13049	1.06	0.084064265	0.99700154
Q9NRZ9	1.061	0.085424656	0.99700154
Q9NW13	1.007	0.010063683	0.99741317
Q9HC35	1.007	0.010063683	0.99741317
Q9Y2S7	1.018	0.025737561	0.99741317
E9PQ57	1.021	0.029982866	0.99741317
O75312	1.032	0.045442971	0.99741317
P98194	1.039	0.055195654	0.99741317
Q86WX3	1.044	0.062121712	0.99741317
Q96FZ5	1.046	0.064882852	0.99741317
Q96MW1	1.054	0.075874867	0.99741317
Q9H081	1.038	0.053806444	0.99744125
O15355	1.013	0.018634174	0.99745574
Q9GZT9	1.06	0.084064265	0.99745574

P10253	1	0	0.99746768
G3XAA0	1	0	0.99746768
O75400-3	1.001	0.001441974	0.99746768
P13984	1.002	0.002882509	0.99746768
Q14980	1.003	0.004321606	0.99746768
Q06830	1.003	0.004321606	0.99746768
G5EA30	1.004	0.005759269	0.99746768
O15126	1.006	0.008630305	0.99746768
Q96PZ0	1.007	0.010063683	0.99746768
F5GXQ1	1.008	0.011495639	0.99746768
Q9P015	1.01	0.014355293	0.99746768
Q99676	1.011	0.015782997	0.99746768
Q6P2Q9	1.012	0.01720929	0.99746768
Q9HB71	1.012	0.01720929	0.99746768
P14618	1.013	0.018634174	0.99746768
P60709	1.013	0.018634174	0.99746768
Q92974	1.013	0.018634174	0.99746768
Q15435	1.016	0.022900402	0.99746768
Q9H4I9	1.016	0.022900402	0.99746768
E9PII7	1.021	0.029982866	0.99746768
Q9HBE1	1.022	0.031395196	0.99746768
Q9BW91	1.033	0.046840254	0.99746768
Q9UKK6	1.035	0.049630768	0.99746768
P60660-2	1.041	0.057970069	0.99746768
O43432	1.041	0.057970069	0.99746768
Q9BW72	1.041	0.057970069	0.99746768
Q9UNP9	1.043	0.060739158	0.99746768
Q9NZ32	1.044	0.062121712	0.99746768
B7Z9U0	1.044	0.062121712	0.99746768
K7ENG3	1.046	0.064882852	0.99746768
Q9H4A6	1.052	0.073134705	0.99746768
C9J406	1.058	0.081339627	0.99746768
P13639	1.003	0.004321606	0.9977693
P38919	1.003	0.004321606	0.9977693
G3XAD6	1.017	0.024319679	0.99788237
Q15527	1.049	0.069014678	0.99788237
Q8NE86	1.053	0.074505436	0.9980336
Q99575	1.011	0.015782997	0.9980437
Q9UBR2	1.051	0.071762669	0.99816078
Q15007	1.056	0.078609835	0.99833089
B7Z1P2	0.995	-0.007231569	0.99847655
Q8WVK2	1.042	0.059355278	0.99876615
Q4AC99	1.016	0.022900402	0.9988329
Q9H6Y2	1.046	0.064882852	0.9988329
I3L2B0	1.057	0.079975377	0.9988329
P62834	1.045	0.063502942	0.99892158

Q96GD4	1.056	0.078609835	0.99892158
O60264	1.004	0.005759269	0.99901897
Q14692	1.004	0.005759269	0.99901897
Q15056	1.004	0.005759269	0.99901897
J3KNI1	1.043	0.060739158	0.99901897
Q14807	1.054	0.075874867	0.99901897
Q13085	1.004	0.005759269	0.9992212
A6NFX8	1.004	0.005759269	0.9992212
Q9BPX5	1.011	0.015782997	0.9992212
Q9Y2S6	1.03	0.042644337	0.9992212
P0CG08	1.039	0.055195654	0.9992212
P55081	1.042	0.059355278	0.9992212
Q86VS8	1.052	0.073134705	0.9992212
Q9UNN5	1.056	0.078609835	0.9992212
O95391	1.056	0.078609835	0.9992212
P00813	1.052	0.073134705	0.99955193
Q14978-2	1.005	0.007195501	0.99974023
Q05086	1.008	0.011495639	0.99998111
P17036	1	0	1
Q68DQ2	1	0	1
Q30201	1	0	1
Q9BSJ5	1	0	1
Q14498-2	1.003	0.004321606	1
Q53GQ0	1.004	0.005759269	1
Q9NZB2	1.005	0.007195501	1
P19367	1.005	0.007195501	1
Q7L1Q6	1.005	0.007195501	1
P07741	1.005	0.007195501	1
O75694	1.006	0.008630305	1
O14579	1.006	0.008630305	1
Q9HCE1	1.006	0.008630305	1
Q9NUJ1	1.006	0.008630305	1
P17480	1.006	0.008630305	1
P62241	1.006	0.008630305	1
P82933	1.006	0.008630305	1
O75330	1.006	0.008630305	1
Q9NWB6	1.006	0.008630305	1
P41091	1.007	0.010063683	1
Q99623	1.007	0.010063683	1
Q5T9A4	1.007	0.010063683	1
Q15785	1.007	0.010063683	1
O43592	1.007	0.010063683	1
P60866	1.007	0.010063683	1
O00443	1.007	0.010063683	1
P06733	1.008	0.011495639	1
O95373	1.008	0.011495639	1

P11413	1.008	0.011495639	1
O43242	1.008	0.011495639	1
Q13206	1.008	0.011495639	1
Q9P2I0	1.008	0.011495639	1
O75347	1.008	0.011495639	1
P18085	1.008	0.011495639	1
P07858	1.008	0.011495639	1
P62753	1.008	0.011495639	1
P38646	1.009	0.012926174	1
G5E9A6	1.009	0.012926174	1
P45880	1.009	0.012926174	1
Q13868	1.009	0.012926174	1
Q15417	1.009	0.012926174	1
P61326	1.009	0.012926174	1
P04004	1.009	0.012926174	1
P60174-1	1.01	0.014355293	1
Q14677-3	1.011	0.015782997	1
G8JLM5	1.011	0.015782997	1
Q9Y221	1.012	0.01720929	1
O14880	1.012	0.01720929	1
Q9Y2Z4	1.013	0.018634174	1
P09234	1.013	0.018634174	1
Q9NVX2	1.017	0.024319679	1
Q92664	1.018	0.025737561	1
E7EVY0	1.02	0.028569152	1
Q9BRX2	1.021	0.029982866	1
P01111	1.024	0.034215715	1
Q96IJ6	1.025	0.03562391	1
P63218	1.025	0.03562391	1
Q99417	1.026	0.037030731	1
E9PIN5	1.026	0.037030731	1
Q16637	1.028	0.039840265	1
Q0IIM8	1.029	0.041242982	1
Q96RT1	1.033	0.046840254	1
P82663	1.034	0.048236186	1
Q567V2	1.035	0.049630768	1
Q00534	1.036	0.051024003	1
P40855	1.038	0.053806444	1
Q8NFH5	1.039	0.055195654	1
Q5TEC6	1.042	0.059355278	1
A6NFV8	1.043	0.060739158	1
Q86X76-2	1.044	0.062121712	1
Q15345	1.044	0.062121712	1
P48651	1.044	0.062121712	1
P60602	1.044	0.062121712	1
Q9BVG9	1.044	0.062121712	1

Q9BQ48	1.046	0.064882852	1
Q9UNI6	1.047	0.066261442	1
H3BSW6	1.048	0.067638717	1
Q9NVN8	1.048	0.067638717	1
Q27J81	1.049	0.069014678	1
Q15018	1.049	0.069014678	1
E9PBR6	1.049	0.069014678	1
E7ESD2	1.05	0.070389328	1
Q96BN8	1.05	0.070389328	1
Q5VTL8	1.05	0.070389328	1
Q9Y2S2	1.05	0.070389328	1
O95400	1.051	0.071762669	1
C9JUF4	1.051	0.071762669	1
Q96AJ9-1	1.051	0.071762669	1
P18887	1.054	0.075874867	1
P83436	1.058	0.081339627	1
Q9Y383	1.059	0.082702589	1
