# Supplementary material for: Paladin, overexpressed in colon cancer, is required for actin polymerisation and liver metastasis dissemination
Source: Oncogenesis. 2022 Jul 26;11(1):42. doi: 10.1038/s41389-022-00416-4 (PMC9325978; doi:10.1038/s41389-022-00416-4)
Supplement: Supplementary file 9 — Supplemental table 3 [file 41389_2022_416_MOESM9_ESM.pdf]

Accession	Phosphorylation sites	Abundance Ratio (sh312)/(shNT)	Log FC2	padj
A0JLT2	1xPhospho [S226(100); S234(0); S235(0); S238(0); S239(0); S240(0); S241(0); S242(0)]	100	6.64385619	8.35E-17
A0JLT2	2xPhospho [S226(4.7); S234(47.7); S235(47.7); S238(0.9); S239(89.4); S240(8.7); S241(0.9); S242(0.1)]	0.101	-3.307572802	0.005371039
A0JLT2	1xPhospho [S226(100); S234(0); S235(0); S238(0); S239(0); S240(0); S241(0); S242(0)]	3.598	1.847195187	0.031727694
A0JLT2	2xPhospho [S226(91.2); S234(4.4); S235(4.4); S238(0.2); S239(4.2); S240(87.2); S241(4.2); S242(4.2)]	3.958	1.984771612	0.134600378
A0JLT2	2xPhospho [S226(100); S234(0); S235(97.5); S238(2.4); S239(0.1); S240(0); S241(0); S242(0)]	1.432	0.518031493	0.792401604
A0JLT2	2xPhospho [S226(100); S234(2.7); S235(97.3); S238(0); S239(0); S240(0); S241(0); S242(0)]	1.048	0.067638717	0.9699948
A0JNW5	3xPhospho [S414(100); S418(100); T420(0); S423(100); T427(0); T429(0)]	100	6.64385619	8.35E-17
A0JNW5	1xPhospho [S414(0); S418(100); T420(0); S423(0); T427(0); T429(0)]	7.918	2.985136067	0.002859469
A0JNW5	2xPhospho [S414(100); S418(100); T420(0); S423(0); T427(0); T429(0)]	3.652	1.868686765	0.006358587
A0MZ66	2xPhospho [S506(100); S512(0); S514(49.5); S515(49.5); T517(1)]	0.535	-0.902389203	0.839866652
A0MZ66	1xPhospho [S506(100); S512(0); S514(0); S515(0); T517(0)]	1.218	0.284514133	0.886912356
A0MZ66	1xPhospho [S249(100); S255(0); S256(0)]	0.945	-0.081613766	0.980895643
A1L390	2xPhospho [S1154(100); T1156(0); S1162(0); S1163(0.4); S1168(6.3); S1169(93.3); S1183(0)]	2.175	1.121015401	0.314784622
A1L390	2xPhospho [S1037(100); S1040(100); T1042(0); T1044(0); S1046(0)]	0.862	-0.214240226	0.973599548
A1L390	1xPhospho [S76(100); S80(0)]	0.816	-0.293358943	0.999342468
A1X283	1xPhospho [T768(2.4); S769(2.4); S770(90.5); S771(2.4); S772(2.4)]	2.423	1.276794405	0.303515893
A1X283	1xPhospho [S491(100)]	1.181	0.240008965	0.919163774
A2A2M0	1xPhospho [T31(0); Y43(0); S46(0); Y47(0); S48(100); S53(0); T59(0)]	100	6.64385619	8.35E-17
A2A2M0	1xPhospho [T31(0); Y43(0); S46(0); Y47(0); S48(100); S53(0); T59(0)]	0.907	-0.140825544	0.980895643
A2ABF8	1xPhospho [S190(0); T192(0); S197(99.9); S200(0.1); S203(0)]	100	6.64385619	8.35E-17
A2ABF8	1xPhospho [S197(100); S200(0); S203(0)]	1.222	0.289244285	0.925912375
A2AJT9	2xPhospho [Y70(0); Y71(0); S73(6.8); Y74(0); S78(96.6); S80(96.6)]	0.938	-0.092340172	0.998448514
A2AJT9	2xPhospho [S15(100); S17(100)]	0.961	-0.057391664	1
A2RU67	2xPhospho [S16(0); Y22(0); T26(0); S30(100); S33(100)]	0.955	-0.066427362	1
A3KN83	2xPhospho [Y678(0); S679(0); T686(0); S689(0); S692(0.1); S693(99.9); S697(100)]	0.01	-6.64385619	8.35E-17
A3KN83	2xPhospho [Y678(0); S679(0); T686(0); S689(0.1); S692(51.1); S693(51.1); S697(97.7)]	1.44	0.526068812	0.792141506
A3KN83	3xPhospho [Y678(0); S679(0); T686(0); S689(66.7); S692(66.7); S693(66.7); S697(100)]	1.421	0.506906555	0.83347241
A3KN83	1xPhospho [S794(100); S799(0); S802(0); S807(0); S811(0)]	0.812	-0.300448367	0.980895643
A6ND36	1xMet-loss+Acetyl [N-Term]; 1xPhospho [S4(100)]	1.214	0.279768422	0.906218259
A6ND36	1xPhospho [S634(100); T642(0)]	0.693	-0.529072743	0.94813191
A6ND36	2xPhospho [S610(100); S613(0); S614(100); S616(0); Y619(0)]	1.09	0.124328135	0.969680228
A6NDB9	1xPhospho [S401(0); S406(0); T412(0); S420(100)]	100	6.64385619	8.35E-17
A6NDB9	2xPhospho [T531(0.7); T534(99.2); S544(0.4); S548(49.8); S550(49.8); T566(0); T568(0)]	2.424	1.277389699	0.254630871
A6NDB9	2xPhospho [S143(100); T151(100); S155(0); S157(0)]	1.816	0.860764203	0.557696597
A6NDB9	3xPhospho [S143(100); T151(100); S155(100); S157(0)]	1.075	0.10433666	0.975403456
A6NDB9	3xPhospho [S143(100); T151(100); S155(97.8); S157(2.2)]	0.877	-0.189351252	0.979498769
A6NFV8	2xPhospho [S312(50); S317(50); S322(100); T331(0); T335(0); S336(0); T338(0)]	0.733	-0.448114897	0.861817554
A6NG51	1xPhospho [S1029(4.4); S1031(95.6); S1041(0)]	100	6.64385619	8.35E-17
A6NG51	2xPhospho [S1029(0); S1031(100); S1041(100)]	2.516	1.331131922	0.225839425
A6NG51	1xPhospho [S1217(100)]	1.722	0.784085143	0.656374509
A6NKD9	1xPhospho [S246(100); S251(0)]	1.062	0.086783766	0.9699948
A6NL94	1xPhospho [S204(0); S205(0); S220(100); S224(0); Y227(0)]	1.214	0.279768422	0.92458333
A6NN40	1xPhospho [S186(2.1); S188(97.9); S190(0)]	100	6.64385619	8.35E-17
A6NNK5	1xPhospho [S227(100); S237(0); S238(0)]	0.01	-6.64385619	8.35E-17
A6NNK5	1xPhospho [T122(2.8); S123(2.8); S124(94.4); S129(0); S132(0)]	100	6.64385619	8.35E-17
A6NNK5	3xPhospho [T1087(50.5); S1091(50.5); S1099(99); S1106(87.9); S1109(12.1)]	100	6.64385619	8.35E-17
A6NNK5	1xPhospho [S487(0.1); S488(97.1); S489(2.8); T491(0); S495(0)]	100	6.64385619	8.35E-17
A6NNK5	2xPhospho [T1627(50); S1628(50); S1633(100)]	100	6.64385619	8.35E-17
A6NNK5	1xPhospho [S264(0); S270(100)]	13.628	3.768501947	1.57E-05
A6NNK5	1xPhospho [S571(0); S585(100)]	0.369	-1.438307279	0.223362863
A6NNK5	2xPhospho [S1091(0); S1099(100); S1106(50); S1109(50)]	0.596	-0.746615764	0.716501219
A6NNK5	1xPhospho [S1072(98.4); S1073(1.6)]	0.593	-0.75389599	0.765892485
A6NNK5	2xPhospho [S523(0); T524(0); S525(0); Y527(0); S528(100); S530(100)]	0.607	-0.720231578	0.810156821
A6NNK5	2xPhospho [S998(0); T1001(0); S1004(100); S1007(100); T1017(0)]	1.352	0.435095152	0.831039794
A6NNK5	1xPhospho [S823(0); T825(0); T828(0); S835(0.1); S836(99.9); S839(0)]	0.617	-0.696657606	0.851160627
A6NNK5	1xPhospho [S1118(2.2); S1119(97.8); T1127(0)]	0.758	-0.399730246	0.855534442
A6NNK5	2xPhospho [S1704(2.7); T1711(7.5); S1713(94.9); S1714(94.9)]	1.287	0.364012054	0.86543833
A6NNK5	2xPhospho [S1207(0); S1213(0); T1219(7); S1221(93.1); S1224(100)]	1.357	0.440420721	0.872730156
A6NNK5	2xPhospho [T1627(0); S1628(100); S1633(100)]	1.267	0.341416524	0.878856551
A6NNK5	2xPhospho [T1369(0); S1381(100); S1385(100)]	1.305	0.384049807	0.8905523
A6NNK5	2xPhospho [S1091(0); S1099(100); S1106(99.9); S1109(0.1)]	0.797	-0.327348371	0.890736743
A6NNK5	1xPhospho [S1091(0); S1099(100); S1106(0); S1109(0)]	1.228	0.296310561	0.899435673
A6NNK5	2xPhospho [S718(0); T725(0); S726(100); S729(0); S732(100)]	1.241	0.311503115	0.900590519
A6NNK5	1xPhospho [T1627(0); S1628(0); S1633(100)]	0.627	-0.673462652	0.90092486
A6NNK5	1xPhospho [S285(4.1); S292(0.2); S299(95.7); S306(0); T307(0); S315(0)]	0.616	-0.698997744	0.908086456
A6NNK5	2xPhospho [S1207(6.9); S1213(18); T1219(81.4); S1221(81.4); S1224(12.2)]	1.151	0.202887833	0.908086456
A6NNK5	3xPhospho [S1091(0); S1099(100); S1106(100); S1109(100)]	0.762	-0.392137097	0.937021119
A6NNK5	2xPhospho [S1415(50); S1417(50); S1429(0); S1435(0); S1436(100); S1440(0)]	1.169	0.22527493	0.941100356
A6NNK5	1xPhospho [S377(0); T378(0); S384(0); S385(98.3); T387(1.7)]	0.82	-0.286304185	0.956041591

A6NNK5	1xPhospho [T1627(0); S1628(0); S1633(100)]	1.094	0.129612738	0.962617576
A6NNK5	1xPhospho [T548(0); T553(0); S557(100); S562(0)]	0.863	-0.212567535	0.968707708
A6NNK5	1xPhospho [T1627(0); S1628(1.6); S1633(98.4)]	0.797	-0.327348371	0.979697817
A6NNK5	2xPhospho [T1627(0); S1628(100); S1633(100)]	1.011	0.015782997	0.980895643
A6NNK5	1xPhospho [S100(0); S101(0); T105(0); S108(100); S110(0)]	0.903	-0.147202107	0.988250665
A6NNK5	1xPhospho [S299(100); S306(0); T307(0); S315(0)]	0.99	-0.01449957	0.99184412
A6NNK5	1xPhospho [S505(100); S512(0); T514(0); S517(0)]	1.014	0.020057652	0.992376868
A6NNK5	2xPhospho [T399(0); S400(100); S403(100)]	0.934	-0.098505545	0.998586735
A7MBM2	2xPhospho [S1252(100); S1263(100); S1270(0)]	1.136	0.183962835	0.946145993
A8CG34	3xPhospho [S322(66.7); S323(66.7); S325(66.7); S328(99.9); S336(0); S341(0); S348(0)]	0.663	-0.592919225	0.761264768
A8CG34	2xPhospho [S368(0); S369(0); S370(97.4); S372(2.6); S373(99.9); T375(0.1); Y378(0); T379(0); S380(0); S384(0); S385(0); S386(0)]	0.66	-0.59946207	0.822854327
A8CG34	3xPhospho [S322(99.7); S323(95.3); S325(95.3); S328(9.8); S336(0); S341(0); S348(0)]	1.159	0.212880566	0.952675081
A8CG34	2xPhospho [S322(4.9); S323(4.9); S325(90.2); S328(100); S336(0); S341(0); S348(0)]	0.865	-0.209227962	0.981208663
A8CG34	1xPhospho [S410(0); S411(0.1); S412(99.9); S415(0.1); S416(0); S419(0); S420(0)]; Q96HA1 1xPhospho [S433(0); S434(0.1); S435(99.9); S438(0.1); S439(0); S442(0); S443(0)]	0.876	-0.190997225	0.991765633
A8CZ64	2xPhospho [T181(0); T185(100); Y187(97.5); T190(2.5)]	0.966	-0.049904906	0.999263007
A8K855	2xPhospho [S200(100); S206(0); S212(100)]	0.316	-1.662003536	0.178300075
A8MVR0	1xPhospho [S118(0.1); S120(99.9); S134(0); Y138(0)]	1.614	0.690640579	0.658368583
A8MVW0	1xPhospho [S446(100)]	0.965	-0.051399153	0.981858798
A8MWU4	2xPhospho [S729(100); S731(99.8); S734(0.2); S739(0); S743(0); S744(0)]	0.948	-0.077041036	0.985318278
A8MWU4	2xPhospho [S774(100); S776(4); S777(96.1); T782(0); S784(0)]	0.918	-0.123433941	1
A8MXP9	1xPhospho [S4(100); S8(0); S9(0); S11(0)]	14.351	3.843079365	1.07E-05
A8MXP9	2xPhospho [S54(0); S61(0); S62(0); S63(0); S71(0.1); S74(92.7); S75(7.8); S77(92.1); T78(7.3); S79(0.1); S80(0); S85(0); S91(0)]	2.514	1.32998465	0.137510904
A8MXP9	2xPhospho [S4(0); S8(0); S9(0); S11(100); S14(100)]	1.849	0.886745225	0.474269009
A8MXP9	1xPhospho [S188(97.7); S195(2.3); Y202(0); S206(0)]	1.627	0.702214251	0.643284326
A8MXP9	1xPhospho [S195(0); Y202(0); S206(100)]	0.52	-0.943416472	0.828459192
A8MXP9	1xPhospho [T789(0); S795(0); S796(0); T806(0.4); S807(0); S814(99.6); Y821(0); T822(0); Y827(0)]	1.17	0.22650853	0.950730609
A8MXP9	1xPhospho [T806(0); S807(0); S814(100); Y821(0); T822(0); Y827(0)]	0.892	-0.164884385	0.989554868
B0AZS9	2xPhospho [S22(4.1); S23(4.1); S24(92.4); S25(99.5)]	1.63	0.704871964	0.663136789
B0AZV0	2xPhospho [T231(0); T232(0); S241(100); S244(100); S250(0)]	1.295	0.372952098	0.879063032
B0QY16	1xPhospho [S147(0); T148(0); T151(0.1); S152(89.7); T156(3.4); S160(3.4); S161(3.4); S164(0)]	12.784	3.676267408	1.45E-05
B0QY16	1xPhospho [S207(0); S209(100); S213(0)]	1.335	0.416839742	0.850354242
B0QY16	2xPhospho [T171(0); S172(0); S174(100); T175(0); T178(100)]	1.327	0.408168371	0.878071918
B0QY16	1xPhospho [S189(100); S191(0)]	1.118	0.160920188	0.962617576
B0QY16	2xPhospho [S147(0); T148(0); T151(3.8); S152(96.2); T156(0); S160(0); S161(0); S164(100)]	1.056	0.078609835	0.980895643
B0QY53	1xPhospho [S18(0); S20(100); S22(0); S26(0)]	1.248	0.319617934	0.899197246
B0QYK0	1xPhospho [S273(0); S274(0); Y278(0); S282(0); S286(100)]	0.988	-0.017417053	0.996295697
B0UX83	2xPhospho [S958(100); S967(100); T972(0); T973(0); S979(0)]	3.702	1.888304895	0.014622692
B0UX83	1xPhospho [T92(0); S96(0); S99(0); S100(0); T102(0); S104(0); S106(0); T108(0); S113(100); T117(0)]	1.835	0.875780063	0.385507135
B0UX83	1xPhospho [Y1110(0); S1111(100)]	1.722	0.784085143	0.689563924
B0UX83	2xPhospho [T92(0); S96(0); S99(0); S100(0); T102(0); S104(100); S106(0); T108(0); S113(100); T117(0)]	1.401	0.486456956	0.848293321
B0UX83	1xPhospho [S958(100)]	1.279	0.355016264	0.872730156
B0UX83	2xPhospho [S958(100); S967(100); T972(0); T973(0); S979(0)]	1.139	0.187767747	0.965365436
B0V0C1	2xPhospho [S337(47.6); S338(47.6); T344(4.8); S350(95.9); S352(1); S356(1); S357(1); S359(1); S363(0); S366(0)]	0.132	-2.921390165	0.000121305
B0V0F8	3xPhospho [S103(100); S106(100); S107(100); T110(0); S112(0)]	0.01	-6.64385619	8.35E-17
B0V0F8	2xPhospho [S103(100); S106(100); S107(0); T110(0); S112(0)]	1.371	0.455228571	0.851597639
B0V0F8	1xPhospho [T155(0); S160(100)]	1.347	0.429749851	0.884991416
B1AK63	3xPhospho [S96(99.9); T98(99.9); S100(85.2); T102(7.5); S103(7.5); S114(0); S124(0)]	0.243	-2.040971781	0.006746107
B1AK63	2xPhospho [S96(97.3); T98(84.7); S100(1.7); T102(8.2); S103(8.2); S114(0); S124(0)]	0.73	-0.454031631	0.879063032
B1AK63	2xPhospho [S96(0.5); T98(0.5); S100(95.9); T102(51.6); S103(51.6); S114(0); S124(0)]	0.944	-0.083141235	0.980895643
B1AKC7	1xPhospho [S219(100); S222(0.1); S225(0); S228(0); T229(0)]	0.627	-0.673462652	0.921247725
B1AKC7	2xPhospho [T864(0); S866(100); S869(100); T871(0); S875(0)]	0.934	-0.098505545	0.97998947
B1AKC7	2xPhospho [S1017(100); S1021(100); S1023(0)]	0.887	-0.17299399	0.980895643
B1AKC7	2xPhospho [T760(0); S764(0); S765(0); S769(0); S771(99.7); S773(5.4); S774(94.9)]	0.828	-0.272297327	0.982492189
B1AKD8	2xPhospho [S369(100); T373(0); S375(100)]	100	6.64385619	8.35E-17
B1AKJ5	2xPhospho [S86(100); S94(100); S96(0)]	0.653	-0.614845103	0.894853453
B1AKJ5	1xPhospho [S94(100); S96(0)]	0.94	-0.089267338	1
B1AKL3	1xPhospho [S412(100); T417(0)]	1.149	0.200378798	0.921247725
B1AKL3	2xPhospho [S69(0); Y71(0); S74(100); S77(2.6); S78(97.4); S82(0)]	1.073	0.101650076	0.981858798
B1AKL3	1xPhospho [S5(100); T9(0); S11(0)]	0.887	-0.17299399	0.997462902
B1AKL3	1xPhospho [S389(100); S393(0); T398(0)]	0.902	-0.148800661	1
B1AKN5	2xPhospho [S278(100); S281(0); S283(90.1); S285(4.9); S286(4.9); S297(0); T299(0); T300(0)]	0.01	-6.64385619	8.35E-17
B1AKN5	1xPhospho [S278(100); S281(0); S283(0); S285(0); S286(0); S297(0); T299(0); T300(0)]	0.91	-0.13606155	0.99184412
B1AKZ3	1xPhospho [S137(100)]	1.863	0.897627674	0.505687266
B1AN62	1xPhospho [S109(0); S117(100); T119(0)]	1.79	0.839959587	0.491829311
B1APS8	2xPhospho [S411(100); S415(100)]	0.639	-0.646112164	0.75891781
B1B0M1	2xPhospho [T614(0); T616(0); S619(0.1); S620(3.5); S621(96.4); S623(3.5); S624(96.4); S626(0.1); Y627(0)]	0.397	-1.332789088	0.574122105
B1B0M1	1xPhospho [S643(0); S645(0.2); S646(4.2); S647(95.6); S659(0)]	0.691	-0.533242384	0.908602715

B1B0M1	2xPhospho [S643(3.6); S645(96.4); S646(96.4); S647(3.6); S659(0)]	0.984	-0.023269779	1
B1Q2X3	3xPhospho [S1686(96.1); S1692(52); S1695(52); S1701(92.5); T1703(7.5)]	0.25	-2	0.156336573
B1Q2X3	2xPhospho [S1159(0); S1162(0); T1171(0.2); S1173(95.8); T1175(8.1); S1176(96); S1179(0); S1180(0); T1182(0)]	0.898	-0.15521265	0.988312267
B2R8E1	2xPhospho [S241(0); S244(0); Y245(0); S248(100); S253(0); S254(0); S256(95); S258(4.7); S260(0.2); S270(0)]	0.45	-1.152003093	0.346002457
B3KNX7	1xPhospho [T219(0); S220(0); S223(0); T225(0); T229(2); T230(98); T236(0)]	3.815	1.931683057	0.03665222
B3KNX7	2xPhospho [T219(0); S220(0); S223(0.3); T225(0.3); T229(99.5); T230(100); T236(0)]	1.836	0.876566059	0.597773138
B3KNX7	1xPhospho [S165(0); T167(0); S174(100); T185(0); T197(0)]	0.766	-0.384583703	0.930376591
B3KNX7	2xPhospho [S165(99.7); T167(0.3); S174(100); T185(0); T197(0)]	0.927	-0.109358756	0.973942845
B3KNX7	1xPhospho [S204(100); T212(0); T214(0)]	0.879	-0.18606493	0.979498769
B3KNX7	2xPhospho [S204(100); T212(1.7); T214(98.3)]	0.907	-0.140825544	0.990299313
B3KP49	3xPhospho [S129(5.3); S132(94.7); Y133(0); S136(97.3); S138(51.4); S140(51.4)]	0.01	-6.64385619	8.35E-17
B3KP49	2xPhospho [S129(0); S132(0); Y133(0); S136(100); S138(0.1); S140(100)]	0.476	-1.070966521	0.6359169
B3KPY8	3xPhospho [T61(100); S65(0); T82(100); S86(93.4); S88(6.7); T91(0); S93(0)]	0.405	-1.304006187	0.381726616
B3KR00	1xPhospho [S67(0); T70(0); Y72(0); S74(0); S76(100)]	0.664	-0.590744853	0.939053555
B3KRH1	1xPhospho [S96(100); S104(0); S105(0); S108(0); S111(0); T112(0); S114(0); S121(0)]	1.924	0.944108799	0.638311892
B3KRH1	1xPhospho [S96(100); S104(0); S105(0); S108(0); S111(0); T112(0); S114(0); S121(0)]	1.267	0.341416524	0.886912356
B3KS98	1xPhospho [S197(100)]	2.274	1.185232254	0.490907173
B4DDC6	2xPhospho [S88(0); S112(100); S115(100)]	0.01	-6.64385619	8.35E-17
B4DDC6	1xPhospho [S88(0); S112(0); S115(100)]	0.01	-6.64385619	8.35E-17
B4DDC6	1xPhospho [S88(0); S112(0); S115(100)]	100	6.64385619	8.35E-17
B4DDC6	1xPhospho [S64(0); S77(100); S82(0)]	3.461	1.791188942	0.027084583
B4DDC6	1xPhospho [S77(100); S82(0)]	1.764	0.818850561	0.450314104
B4DDC6	2xPhospho [S88(0); S112(100); S115(100)]	0.779	-0.360304767	0.872730156
B4DDC6	1xPhospho [S77(100); S82(0)]	0.953	-0.069451881	0.980895643
B4DDC6	2xPhospho [S88(0); S112(100); S115(100)]	0.906	-0.142417045	0.989838391
B4DDD1	1xPhospho [S68(100)]	0.565	-0.823677227	0.85992414
B4DE04	1xPhospho [S230(2.1); S234(2.1); S235(95.8)]	0.578	-0.790858602	0.613487532
B4DE04	1xPhospho [S203(100)]	1.068	0.094911647	0.965139891
B4DEJ6	2xPhospho [S36(100); T41(0); S47(48.4); S49(48.4); T58(3.2); S72(0); S73(0)]	3.062	1.614474283	0.105218811
B4DEJ6	1xPhospho [S78(100); T96(0); S100(0); S101(0)]	0.985	-0.02180437	0.971838536
B4DEU8	2xPhospho [S432(100); S436(100)]	0.356	-1.490050854	0.486592754
B4DF41	1xPhospho [S700(100)]	1.483	0.568518598	0.790142755
B4DG44	3xPhospho [S76(0); S80(0.2); T82(1.2); T84(9.5); S87(79.5); S90(9.5); S106(63.1); T107(63.1); S109(63.1); S111(10.8)]	100	6.64385619	8.35E-17
B4DG44	2xPhospho [S201(100); S202(100)]	2.243	1.16542962	0.227012222
B4DG44	2xPhospho [S201(100); S202(100)]	1.559	0.640620928	0.651510528
B4DG44	2xPhospho [S76(0); S80(0); T82(0); T84(0); S87(0); S90(0); S106(94.4); T107(5.6); S109(99.6); S111(0.4)]	0.574	-0.800877358	0.755151641
B4DGP8	1xPhospho [S589(100); T597(0); S599(0)]	3.17	1.66448284	0.021361795
B4DGP8	1xPhospho [S589(0); T597(0); S599(100)]	1.496	0.581110175	0.669208958
B4DGP8	2xPhospho [S589(100); T597(2.4); S599(97.6)]	1.121	0.164786278	0.965013037
B4DGP8	1xPhospho [S618(100)]	0.923	-0.115597447	0.973763863
B4DGP8	2xPhospho [S589(100); T597(0); S599(100)]	1.072	0.100304906	0.980895643
B4DGP8	1xPhospho [S589(100); T597(0); S599(0)]	1.024	0.034215715	0.995570754
B4DGQ0	1xPhospho [S138(100)]	2.318	1.212880566	0.366420152
B4DGQ0	1xPhospho [S78(0); S90(100); S94(0); S95(0); Y96(0)]	1.535	0.618238656	0.643513638
B4DGQ0	2xPhospho [S78(100); S90(99.4); S94(0.3); S95(0.3); Y96(0)]	0.623	-0.682695932	0.730212284
B4DGQ0	2xPhospho [S78(100); S90(100); S94(0); S95(0); Y96(0)]	0.57	-0.810966176	0.797966154
B4DGQ0	1xPhospho [S43(100); T48(0)]	1.255	0.327687364	0.879063032
B4DGQ0	2xPhospho [S78(100); S90(100); S94(0); S95(0); Y96(0)]	0.844	-0.244685096	0.933107775
B4DGQ0	1xPhospho [S78(0); S90(100); S94(0); S95(0); Y96(0)]	0.979	-0.030619235	1
B4DH53	2xPhospho [S566(93.9); S569(6.1); S574(0); S577(0); T582(0); S584(100)]	100	6.64385619	8.35E-17
B4DH53	1xPhospho [S520(97.8); S521(2.2)]	3.43	1.778208576	0.147880089
B4DH53	2xPhospho [S620(0); S625(0); S629(100); S631(100)]	2.333	1.222186307	0.192162957
B4DH53	3xPhospho [S703(100); S705(100); S715(100)]	2.459	1.298071735	0.402830868
B4DH53	1xPhospho [S733(100); S736(0); S737(0); S740(0); S741(0)]	1.556	0.63784206	0.730212284
B4DH53	2xPhospho [S629(100); S631(100)]	1.526	0.609754962	0.751633178
B4DH53	2xPhospho [T434(0); S446(100); S449(96.1); S452(3.9)]	1.332	0.413594082	0.879063032
B4DH53	2xPhospho [S703(100); S705(100); S715(0)]	1.227	0.295135249	0.921350951
B4DH53	3xPhospho [S605(50.1); S606(50.1); T612(99.9); S614(99.7); S617(0.3)]	1.219	0.285698126	0.938029982
B4DH53	1xPhospho [S629(0); S631(100)]	1.121	0.164786278	0.963156927
B4DIL8	2xPhospho [S1332(33.3); S1333(33.3); S1334(33.3); S1337(99.9); S1339(0.1)]	0.619	-0.691988685	0.763457172
B4DIT1	1xPhospho [S1093(100); S1100(0)]	1.007	0.010063683	0.965248196
B4DJ58	2xPhospho [T244(0); T245(100); S247(0); S249(100)]	2.233	1.158983251	0.245916135
B4DJ58	1xPhospho [T138(0); S141(100)]	0.667	-0.584241333	0.690335172
B4DJ84	2xPhospho [S405(100); S407(100); S414(0)]	100	6.64385619	8.35E-17
B4DJ84	3xPhospho [S255(100); S257(0); S259(0); S268(100); S270(100)]	10.154	3.34397626	0.000369348
B4DJ84	2xPhospho [S336(100); S340(99.7); T349(0.4); S355(0); S358(0); S360(0)]	0.318	-1.652901329	0.041234718
B4DJ84	2xPhospho [T74(0); T76(0); S87(100); S89(95.3); S91(4.7)]	1.634	0.708407983	0.570285159
B4DJ84	2xPhospho [S294(100); S296(100)]	1.3	0.378511623	0.848670625
B4DJ84	3xPhospho [S336(98.1); S340(98.1); T349(5.2); S355(86.7); S358(11.6); S360(0.3)]	0.698	-0.518701058	0.917263707
B4DJ84	2xPhospho [S336(100); S340(100); T349(0); S355(0); S358(0); S360(0)]	1.188	0.248534836	0.938029982

B4DJ84	3xPhospho [T74(0); S76(100); S87(99.9); S89(99.9); S91(0.2)]	0.784	-0.351074441	0.943512809
B4DJ84	2xPhospho [T442(0); S445(100); S447(100); S456(0)]	1.122	0.166072676	0.943796521
B4DJ84	2xPhospho [S405(100); S407(100); S414(0)]	0.913	-0.131313235	0.965795497
B4DJ84	2xPhospho [S242(100); S244(100)]; 2xPhospho [S268(100); S270(100)]	0.911	-0.134477041	0.971838536
B4DJ84	2xPhospho [S336(100); S340(0); T349(100); S355(0); S358(0); S360(0)]	0.83	-0.268816758	0.973763863
B4DJ84	2xPhospho [T442(0); S445(100); S447(100); S456(0)]	0.882	-0.181149439	0.980895643
B4DJM0	2xPhospho [Y197(0); S199(100); S201(0.2); S202(96); S203(3.8); S204(0.2); S209(0); S210(0); S213(0); S214(0); S215(0)]	1	0	0.97998947
B4DKT0	1xPhospho [S444(0); T445(0); S448(0); S453(0); S461(0); S463(100)]	1.664	0.734655433	0.701261726
B4DKT0	2xPhospho [S444(0.1); T445(99.9); S448(0); S453(0); S461(0); S463(100)]	1.071	0.09895848	0.965553053
B4DLH4	1xPhospho [S52(0); S53(0); S58(0); T63(0); S65(100); S66(0)]	2.21	1.14404637	0.266091451
B4DLH4	2xPhospho [S213(50); S214(50); S216(100); Y218(0)]	0.661	-0.597277823	0.939695119
B4DLH4	1xPhospho [S73(100); T79(0)]	1	0	0.971220368
B4DLR8	1xPhospho [S191(100); T194(0)]	3.652	1.868686765	0.045870107
B4DLZ9	1xPhospho [S177(100); T188(0); Y191(0); Y196(0); T197(0); T205(0)]	100	6.64385619	8.35E-17
B4DLZ9	2xPhospho [S43(99.9); T45(50); S47(50); S50(0); S51(0); T53(0); S59(0)]	1.247	0.318461465	0.945506512
B4DNK1	1xPhospho [S522(99.9); S525(0.1); T540(0)]	0.992	-0.011587974	0.980895643
B4DNU6	1xPhospho [S302(100); T311(0)]	0.465	-1.104697379	0.412309751
B4DP31	1xPhospho [T131(0); S141(100)]	1.47	0.555816155	0.75891781
B4DP61	1xPhospho [T25(0); S28(100); S31(0); T37(0)]	0.849	-0.236163541	0.973263372
B4DP61	2xPhospho [T25(0); S28(100); S31(100); T37(0)]	1.044	0.062121712	0.988250665
B4DP61	2xPhospho [T25(0); S28(100); S31(100); T37(0)]	0.954	-0.067938829	1
B4DP61	3xPhospho [T25(100); S28(100); S31(100); T37(0)]	0.994	-0.008682243	1
B4DQ30	2xPhospho [S113(0); S118(99.9); S119(95.9); S120(2.1); S121(2.1)]	100	6.64385619	8.35E-17
B4DR64	2xPhospho [T335(0.1); Y336(0); S337(3.6); S340(96.1); S342(33.4); S343(33.4); S344(33.4); S347(0); T350(0); S356(0)]	100	6.64385619	8.35E-17
B4DR64	3xPhospho [T335(0.1); Y336(0); S337(99.9); S340(0); S342(99.9); S343(50); S344(50); S347(0); T350(0)]	1.262	0.33571191	0.886312012
B4DR64	2xPhospho [S340(0); S342(0); S343(100); S344(100); S347(0); T350(0)]	1.187	0.247319935	0.943912599
B4DR64	2xPhospho [S340(0); S342(0); S343(100); S344(100); S347(0); T350(0); S356(0)]	0.809	-0.305788392	0.958702638
B4DR64	1xPhospho [S340(0); S342(0); S343(0); S344(100); S347(0); T350(0)]	1.023	0.032806145	0.973323945
B4DR64	1xPhospho [S340(87.7); S342(4); S343(4); S344(4); S347(0.2); T350(0); S356(0)]	0.82	-0.286304185	0.985318278
B4DR64	3xPhospho [T335(8.7); Y336(0); S337(91.2); S340(95.3); S342(6.9); S343(48.9); S344(48.9); S347(0); T350(0); S356(0)]	0.951	-0.072482754	0.988839902
B4DR64	2xPhospho [T335(0); Y336(0); S337(0); S340(100); S342(98); S343(2); S344(0); S347(0); T350(0)]	1.008	0.011495639	0.989898705
B4DRH2	2xPhospho [S456(4.5); T457(95.5); Y458(0); S472(100)]	0.859	-0.219269964	0.975403456
B4DRS7	1xPhospho [S23(0); S25(100)]	1.492	0.577247536	0.759239289
B4DRS7	1xPhospho [T71(0); S75(0); S79(100)]	1.431	0.517023672	0.772467589
B4DRS7	1xPhospho [S36(100); S46(0); T47(0); S51(0)]	1.406	0.491596594	0.79866369
B4DS55	2xPhospho [S272(0); Y274(0); Y277(0); S279(100); S281(100)]	0.352	-1.506352666	0.251774426
B4DS55	2xPhospho [S212(98); S214(2); S216(0); S218(100); S220(0); S225(0); T226(0); S227(0); S228(0)]	1.652	0.724213687	0.659227788
B4DS61	2xPhospho [S17(99.9); S21(0.1); S23(0); S35(100); Y46(0)]	1.639	0.712815854	0.630082428
B4DS61	2xPhospho [S293(100); S295(100)]	1.538	0.621055503	0.644556338
B4DS61	3xPhospho [S17(99.7); S21(100); S23(0.3); S35(100); Y46(0)]	0.616	-0.698997744	0.75802349
B4DS61	1xPhospho [S293(0); S295(100)]	1.396	0.481298942	0.857906468
B4DS61	1xPhospho [S17(0); S21(0); S23(0); S35(100); Y46(0)]	1.119	0.162210036	0.964978421
B4DSB0	1xPhospho [S444(100); S448(0); S450(0)]	1.876	0.907659828	0.564643378
B4DSB0	1xPhospho [S382(100); T384(0); S387(0); T388(0)]	1.563	0.644317778	0.703538855
B4DSB0	2xPhospho [S395(99.7); S397(0.3); S408(0.3); S412(94.2); T414(5.2); T419(0.3); S421(0); S427(0)]	0.764	-0.388355457	0.915988867
B4DST5	2xPhospho [S995(0); S996(0.3); S997(99.7); S1000(100); T1005(0); S1007(0); T1018(0)]	0.673	-0.57132159	0.841465156
B4DT67	1xPhospho [S523(0); T528(100); S535(0)]	8.137	3.024496991	0.000840977
B4DT67	1xPhospho [S439(0); S440(0); S443(98); T444(2); S448(0); T451(0)]	1.5	0.584962501	0.790280431
B4DT67	1xPhospho [S145(0); S146(0); S147(0); T148(0); S150(100)]	1.076	0.105678078	0.963131675
B4DT67	1xPhospho [S189(100); T200(0); T201(0)]	0.939	-0.090802937	1
B4DTU4	1xPhospho [S36(100); S46(0)]	100	6.64385619	8.35E-17
B4DTU4	1xPhospho [T102(0); S111(100)]	100	6.64385619	8.35E-17
B4DTU4	2xPhospho [S58(7.6); S61(92.4); T67(7); S68(7); S74(85.3); S76(0.6); T78(0); S79(0); S83(0); S84(0); S86(0)]	2.762	1.46571332	0.097541626
B4DTU4	1xPhospho [T151(0); T152(100)]	2.148	1.102993993	0.356416942
B4DTU4	2xPhospho [S58(0); S61(100); T67(50); S68(50); S74(0); S76(0); T78(0); S79(0); S83(0); S84(0); S86(0)]	1.804	0.851199339	0.479116982
B4DTU4	2xPhospho [S36(100); S46(100)]	1.613	0.689746438	0.646029575
B4DTU4	3xPhospho [S58(0); S61(100); T67(92); S68(8); S74(0); S76(0); T78(0); S79(0); S83(7.4); S84(85.3); S86(7.4)]	1.609	0.686164326	0.679191074
B4DTU4	1xPhospho [T102(0); S111(100)]	1.406	0.491596594	0.79866369
B4DTU4	2xPhospho [S17(100); S19(0); S21(100)]	1.13	0.176322773	0.952591019
B4DUI3	1xMet-loss+Acetyl [N-Term]; 1xPhospho [S11(100); S13(0); S20(0)]	0.35	-1.514573173	0.059175794
B4DUI3	1xMet-loss+Acetyl [N-Term]; 1xPhospho [S11(99.9); S13(0.1); S20(0)]	0.403	-1.311148256	0.371564406
B4DV38	2xPhospho [S112(5.5); S113(88.5); S115(5.5); S116(0.4); S119(0); S121(0); S122(0); S123(0); S127(0); S138(99.5); S140(0.4); S142(0); S143(0)]	0.567	-0.81857936	0.713216184
B4DV38	1xPhospho [S112(4); S113(87.9); S115(4); S116(4); S119(0); S121(0); S122(0); S123(0); S127(0); S138(0); S140(0); S142(0); S143(0)]	1.496	0.581110175	0.750433149
B4DV38	1xPhospho [S649(1.9); S650(1.9); S651(96.3); T657(0); T658(0); T660(0); S661(0); S664(0); T667(0)]	1.261	0.334568276	0.879063032

B4DV38	2xPhospho [S112(95.2); S113(4.8); S115(0); S116(0.1); S119(25); S121(25); S122(25); S123(25); S127(0); S138(0); S140(0); S142(0); S143(0)]	1.068	0.094911647	0.963156927
B4DVB8	1xPhospho [S224(0); Y227(0); S229(100)]	1.824	0.86710573	0.647620981
B4DWL9	1xPhospho [S284(100); S287(0); Y292(0); S296(0)]	1.689	0.756169328	0.666930668
B4DY08	2xPhospho [S240(100); S247(100)]	0.471	-1.086201035	0.256668325
B4DY08	1xPhospho [S100(0); Y105(0); S107(100); S108(0); Y113(0)]	1.182	0.241230036	0.938730361
B4DY08	1xPhospho [S240(0); S247(100)]	0.746	-0.422752464	0.954539739
B4DY08	1xPhospho [S240(0); S247(100)]; P07910 1xPhospho [S253(0); S260(100)]	1.02	0.028569152	0.997077639
B4DYR1	1xPhospho [S1048(0); S1051(100)]	1.943	0.958285901	0.525769281
B4DZC3	1xPhospho [S445(96); S447(4)]	100	6.64385619	8.35E-17
B4DZC3	2xPhospho [S445(100); S447(100)]	4.45	2.153805336	0.004551272
B4DZC3	1xPhospho [S416(0); S417(0); S419(0); S421(0); T424(0); S425(97.1); T427(2.8); S428(0.1); S431(0); S433(0)]	1.484	0.569491092	0.699388119
B4DZC3	2xPhospho [S416(96); S417(4.2); S419(95.8); S421(4.1); T424(0); S425(0); T427(0); S428(0); S431(0); S433(0)]	1.192	0.253384236	0.933847529
B4DZI8	2xPhospho [S818(50); T819(50); S830(100); T832(0); S838(0); T840(0)]	1.323	0.403813062	0.878812277
B4DZI8	2xPhospho [S818(3.6); T819(96.4); S830(99.9); T832(0.2); S838(0); T840(0)]	1.321	0.401630467	0.879063032
B4DZI8	1xPhospho [S818(0); T819(0); S830(100); T832(0); S838(0); T840(0)]	0.866	-0.20756107	0.952758634
B4DZI8	1xPhospho [S818(0); T819(0); S830(97.4); T832(2.6); S838(0); T840(0)]	1.105	0.14404637	0.972363997
B4E1E4	1xPhospho [S13(100); S23(0); T24(0)]	1.116	0.158337027	0.963156927
B4E1M0	2xPhospho [S48(100); S54(100); S58(0); S63(0)]	0.964	-0.052894948	0.977837438
B4E1N6	2xPhospho [S68(0); S70(0); S72(0); S74(0); S76(0); S79(0); S80(3.8); S81(96.2); S86(96.2); T88(3.8); S91(0)]	100	6.64385619	8.35E-17
B4E1N6	1xPhospho [S42(0); T43(0); S44(100); T45(0); S52(0); T53(0); T54(0); S59(0)]	1.375	0.459431619	0.847936919
B4E1N6	3xPhospho [S68(0); S70(0.3); S72(5.1); S74(89.4); S76(5.1); S79(0.2); S80(49.9); S81(49.9); S86(99.7); T88(0.3); S91(0)]	0.919	-0.121863233	0.984462328
B4E322	2xPhospho [S300(2.2); T303(48.9); S304(48.9); S308(99.8); S313(0.2)]	0.104	-3.265344567	0.001763198
B4E322	2xPhospho [T303(50); S304(50); S308(100); S313(0)]	2.737	1.452595435	0.072925614
B5MCH7	2xPhospho [S336(100); S338(100)]	1.682	0.750177706	0.607772032
B5MCH7	2xPhospho [S336(100); S338(100)]	0.882	-0.181149439	0.962617576
B5MDQ0	2xPhospho [Y1003(0); S1011(99.8); S1012(99.8); S1016(0.5); Y1018(0); T1019(0); S1024(0); T1027(0); S1029(0); S1030(0)]	100	6.64385619	8.35E-17
B5MDQ0	1xPhospho [S697(100); T703(0); S705(0); S706(0)]	1.186	0.24610401	0.936043234
B5MDQ0	2xPhospho [S845(0); S846(0); S848(0); S857(100); S861(95.1); S865(5)]	0.966	-0.049904906	0.973910166
B5MEB7	2xPhospho [S92(100); S94(0); S97(100)]	1.207	0.271425676	0.93019184
B5MEB7	1xPhospho [S92(0); S94(0); S97(100)]	0.891	-0.166502663	1
B7WP74	1xPhospho [S3(100); S4(0.1); S11(0); S12(0)]	5.74	2.521050737	0.018532237
B7WP74	1xPhospho [S27(0); S28(0); Y33(0); S39(100)]	1.493	0.578214165	0.764901318
B7WP74	2xPhospho [S27(1.4); S28(98.6); Y33(0); S39(100)]	1.362	0.445726703	0.849882438
B7WP74	3xPhospho [S27(100); S28(100); Y33(0); S39(100)]	1.14	0.189033824	0.965139891
B7WPF4	1xPhospho [T969(0); S972(0); S974(0); S975(0); S976(0); S978(0); S979(0); S981(0.2); S983(91.2); S985(4.3); S986(4.3)]	0.13	-2.943416472	0.009680638
B7WPF4	1xPhospho [S1883(0); S1887(100)]	0.838	-0.254977851	1
B7Z1L3	1xPhospho [S54(0); S57(100)]	0.601	-0.734563104	0.532963545
B7Z1L3	1xPhospho [T126(0); Y128(0); S129(100); S138(0)]	1.281	0.357270476	0.879063032
B7Z1L3	1xPhospho [T126(0); Y128(0); S129(100); S138(0)]	1.025	0.03562391	0.994865845
B7Z341	1xPhospho [S242(0); T244(0); S245(0); S248(0); T251(0); S254(0); S257(100)]	3.062	1.614474283	0.1227296
B7Z341	3xPhospho [T225(3.2); S227(93.8); T228(9.2); S229(96.8); S230(96.8); S231(0.2); S234(0); S237(0); S240(0)]	0.519	-0.946193556	0.682039777
B7Z341	2xPhospho [T225(0); S227(100); T228(0); S229(0.1); S230(99.9); S231(0.1); S234(0); S237(0); S240(0)]	1.148	0.199122642	0.962617576
B7Z341	3xPhospho [S223(0.1); T225(99.9); S227(100); T228(0); S229(0); S230(99.9); S231(0.1); S234(0); S237(0); S240(0)]	0.849	-0.236163541	0.970242377
B7Z341	2xPhospho [S223(0); T225(100); S227(100); T228(0); S229(0); S230(0); S231(0); S234(0); S237(0); S240(0)]	1.012	0.01720929	0.998448514
B7Z382	1xPhospho [S498(5); S500(95); T516(0)]	0.329	-1.603840511	0.184642375
B7Z3Q7	1xPhospho [S563(0); T571(97); T573(3)]	1.002	0.002882509	0.978877443
B7Z4K4	1xPhospho [T165(0); T177(0); S189(0); S191(100)]	0.799	-0.323732592	0.973763863
B7Z5N7	1xPhospho [S112(0); S113(0); S118(100)]	0.63	-0.666576266	0.868254505
B7Z5R1	3xPhospho [S60(7.3); S62(92.7); S64(100); S67(100); S75(0)]	1.072	0.100304906	0.980895643
B7Z5R1	2xPhospho [S60(2.3); S62(97.7); S64(0); S67(100); S75(0)]	0.983	-0.024736678	0.991390224
B7Z6G2	2xPhospho [S149(50); T151(50); S154(100); T161(0); T162(0)]	1.309	0.388465097	0.854283637
B7Z6G2	1xPhospho [S149(0); T151(0); S154(100); T161(0); T162(0)]	1.382	0.466757616	0.855557911
B7Z6G2	1xPhospho [S415(100); Y419(0)]	1.301	0.379620962	0.904079048
B7Z7F3	2xPhospho [S151(0); S153(0); S167(99.8); S168(99.8); S171(0.4)]	1.658	0.729444007	0.658251577
B7Z7F3	3xPhospho [S28(0); S32(100); S33(100); S40(100); Y47(0)]; Q9H6Z4-3 3xPhospho [S28(0); S32(100); S33(100); S40(100); Y47(0)]	0.626	-0.675765438	0.669115412
B7Z7F3	3xPhospho [S28(0); S32(100); S33(100); S40(100); Y47(0)]; Q9H6Z4-3 3xPhospho [S28(0); S32(100); S33(100); S40(100); Y47(0)]	0.626	-0.675765438	0.669115412
B7Z7F3	2xPhospho [S260(100); S268(2.4); S269(97.6)]; Q9H6Z4-3 2xPhospho [S265(100); S273(2.4); S274(97.6)]	0.468	-1.095419565	0.75891781
B7Z7F3	1xPhospho [S28(0); S32(0); S33(0); S40(100); Y47(0)]; Q9H6Z4-3 1xPhospho [S28(0); S32(0); S33(0); S40(100); Y47(0)]	1.458	0.54399072	0.775393256

B7Z7F3	1xPhospho [S28(0); S32(0); S33(0); S40(100); Y47(0)]; Q9H6Z4-3 1xPhospho [S28(0); S32(0); S33(0); S40(100); Y47(0)]	1.458	0.54399072	0.775393256
B7Z7F3	3xPhospho [S28(99.8); S32(96); S33(4.2); S40(100); Y47(0)]; Q9H6Z4-3 3xPhospho [S28(99.8); S32(96); S33(4.2); S40(100); Y47(0)]	0.697	-0.520769439	0.848293321
B7Z7F3	2xPhospho [S28(0.1); S32(99.7); S33(0.1); S40(100); Y47(0)]; Q9H6Z4-3 2xPhospho [S28(0.1); S32(99.7); S33(0.1); S40(100); Y47(0)]	1.325	0.40599236	0.86531088
B7Z7F3	1xPhospho [S28(0); S32(0); S33(0); S40(100); Y47(0)]; Q9H6Z4-3 1xPhospho [S28(0); S32(0); S33(0); S40(100); Y47(0)]	1.365	0.448900951	0.869295771
B7Z7F3	1xPhospho [S151(98.1); S153(2); S167(0); S168(0); S171(0)]	1.122	0.166072676	0.94432975
B7Z7F3	2xPhospho [S28(0); S32(95.3); S33(4.9); S40(99.8); Y47(0)]; Q9H6Z4-3 2xPhospho [S28(0); S32(95.3); S33(4.9); S40(99.8); Y47(0)]	1.099	0.136191386	0.973263372
B7Z7F3	1xPhospho [S260(100); S268(0); S269(0)]; Q9H6Z4-3 1xPhospho [S265(100); S273(0); S274(0)]	0.828	-0.272297327	0.973763863
B7Z7F3	2xPhospho [S28(0); S32(3.9); S33(96.1); S40(100); Y47(0)]; Q9H6Z4-3 2xPhospho [S28(0); S32(3.9); S33(96.1); S40(100); Y47(0)]	1.051	0.071762669	0.985461924
B7Z7Y1	1xPhospho [S644(100); T655(0); S656(0)]	1.408	0.493647334	0.807541994
B7Z848	1xPhospho [S562(0); S563(0.1); S565(100)]	1.515	0.599317794	0.769999353
B7Z888	1xPhospho [S1092(97.1); S1094(2.9)]	0.631	-0.66428809	0.907438338
B7Z888	1xPhospho [S670(0); S671(0); T677(0); T680(2); T681(97.9); S683(0); T685(0)]	1.137	0.185232254	0.938029982
B7Z8L8	2xPhospho [S72(0); S77(100); S88(100); S102(0)]	2.207	1.14208663	0.407981646
B7Z8L8	2xPhospho [S72(100); S77(100)]	1.298	0.376290383	0.879063032
B7Z8L8	1xPhospho [S88(100); S102(0)]	1.191	0.252173413	0.921247725
B7Z8L8	3xPhospho [S72(100); S77(100); S88(100); S102(0)]	0.85	-0.234465254	0.939268934
B7Z8Q0	1xPhospho [T401(99.9); T405(0.1); Y408(0); Y416(0); Y419(0)]	0.33	-1.59946207	0.438384169
B7ZB17	1xPhospho [S341(100); S347(0)]	100	6.64385619	8.35E-17
B7ZB17	2xPhospho [T337(100); S341(100); S347(0)]	1.135	0.182692298	0.943939997
B7ZB17	1xPhospho [S687(100)]	0.693	-0.529072743	0.963131675
B7ZB17	3xPhospho [T337(100); S341(100); S347(100)]	0.81	-0.304006187	0.973599548
B7ZBM3	1xPhospho [S291(97.2); S292(2.8); S293(0.1); T297(0); S300(0); Y304(0)]	0.684	-0.54793177	0.938029982
B7ZBY5	1xPhospho [S401(0); S404(0); S407(0); S410(0); S416(100)]	0.963	-0.054392297	1
B7ZKL3	2xPhospho [S479(99.9); T481(0.1); S482(0); T485(0); S495(50); S496(50); T499(0)]	1.794	0.84317989	0.467067441
B7ZKL3	2xPhospho [S475(0); S479(0); T481(0); S482(0); T485(100); S495(50); S496(50); T499(0)]	1.753	0.809825996	0.599010435
B7ZKS7	2xPhospho [S891(0); S898(99.9); S899(50); S900(50); T902(0.2); S922(0); T926(0)]	1.274	0.349365278	0.901860353
B7ZKZ1	1xPhospho [S219(0); S227(100)]	0.796	-0.329159664	0.949541112
B8ZZQ6	1xMet-loss+Acetyl [N-Term]; 1xPhospho [S2(0); T8(100); S9(0); S10(0); T13(0); T14(0)]	1.792	0.841570637	0.383584545
B8ZZQ6	1xMet-loss [N-Term]; 1xPhospho [S2(100); T8(0); S9(0); S10(0); T13(0); T14(0)]	1.406	0.491596594	0.797966154
B8ZZS2	1xPhospho [S401(93.1); S402(6.9); S406(0); T411(0); S412(0); S415(0)]	1.978	0.984042426	0.406234707
B9DI81	3xPhospho [S873(88.7); T879(33.9); S889(88.7); S894(88.7)]	0.85	-0.234465254	1
B9EK38	2xPhospho [S873(100); S874(97.9); S877(2.1)]	0.888	-0.171368418	0.97008929
B9EK38	1xPhospho [S795(0); S796(0); S797(99.9); T798(0); S799(0); T806(0)]	0.924	-0.114035243	1
B9ZVN9	2xPhospho [T357(0); T359(100); T360(100); S366(0)]	0.524	-0.932361283	0.810764434
C9J1X3	2xPhospho [T782(8.4); S789(91.5); T798(0.1); S799(0.1); S806(100)]	1.24	0.310340121	0.879063032
C9J2I0	1xPhospho [S84(100); S89(0); T92(0)]	100	6.64385619	8.35E-17
C9J2I0	2xPhospho [S84(0); S89(0); T92(0); T99(100); S101(50); S103(50)]	0.781	-0.356605547	0.93019184
C9J3D2	1xPhospho [T81(0); T82(0); T83(0); T84(0); S86(100); Y92(0); S96(0)]	1.711	0.77483976	0.670368081
C9J3D2	2xPhospho [T81(3.4); T82(89.9); T83(3.4); T84(3.4); S86(100); Y92(0); S96(0)]	1.126	0.171206827	0.945036082
C9J3D2	2xPhospho [S166(100); S169(100); T174(0)]	1.184	0.243669081	0.955790722
C9J3D2	2xPhospho [T81(0.1); T82(0.1); T83(0.1); T84(99.6); S86(100); Y92(0); S96(0)]	0.948	-0.077041036	0.989716573
C9J3I2	1xPhospho [S118(0); T123(0); S124(0); S125(0); S126(0); T127(0); S131(0); S132(0); S133(0); S135(0); S136(0); Y137(0); S138(100); S140(0)]	0.01	-6.64385619	8.35E-17
C9J5J4	2xPhospho [Y284(0); T285(0); S290(100); S302(100)]	100	6.64385619	8.35E-17
C9J8L0	3xPhospho [T61(0); S70(100); S73(99.9); S79(91.4); S82(8.6); T94(0)]	0.01	-6.64385619	8.35E-17
C9J8L0	2xPhospho [T42(100); S44(100); T46(0); T53(0); S55(0)]	0.682	-0.552156356	0.860037477
C9J8V3	1xPhospho [S21(100); S29(0)]	1.817	0.86155842	0.601737067
C9J9D2	3xPhospho [S23(100); S25(100); S27(100); S29(0)]	0.242	-2.046921047	0.124185961
C9JAQ9	1xPhospho [S225(100)]	1.294	0.371837617	0.879063032
C9JAQ9	1xPhospho [T37(0); S42(100)]	1.164	0.219091058	0.929390092
C9JAQ9	1xPhospho [S8(100); Y11(0); Y13(0)]	0.869	-0.202571918	0.996295697
C9JBZ4	1xPhospho [S128(0); T129(0); S130(98.3); S132(1.7)]	1.869	0.902266569	0.599534831
C9JC60	1xPhospho [S559(0); S565(100)]	0.69	-0.535331733	0.96293109
C9JFV4	1xPhospho [S878(0); S887(3.6); T889(96.5); T893(0); T899(0)]	0.01	-6.64385619	8.35E-17
C9JFV4	1xPhospho [S625(100); S629(0); T632(0); S636(0)]	2.635	1.397802962	0.104021017
C9JFV4	2xPhospho [S621(100); S625(100); S629(0); T632(0); S636(0)]	1.199	0.261831659	0.931904324
C9JFZ1	2xPhospho [T1087(4.5); S1088(95.5); S1092(99.8); T1094(0.2); S1096(0); S1102(0); S1108(0)]	0.882	-0.181149439	0.989554868
C9JIG9	1xPhospho [T331(0); S339(100); S347(0)]	100	6.64385619	8.35E-17
C9JIL1	1xPhospho [S461(100)]	100	6.64385619	8.35E-17
C9JLZ0	2xPhospho [S1099(0); S1107(0); T1115(0.1); S1116(99.8); S1117(0.1); S1124(3.3); S1126(96.7)]	0.422	-1.244685096	0.262095693
C9JLZ0	2xPhospho [T1077(0); S1083(100); T1090(100); S1092(0.1)]	0.373	-1.422752464	0.479116982
C9JLZ0	1xPhospho [T123(0); S124(0); T134(0); S139(100)]	1.282	0.358396262	0.855557911
C9JLZ0	1xPhospho [S1099(0); S1107(0); T1115(0); S1116(0); S1117(0); S1124(96.6); S1126(3.4)]	1.289	0.366252264	0.9072193
C9JME2	3xPhospho [S909(0); T914(0); S920(100); S925(49.2); S927(49.2); T929(1.8); S930(99.9)]	1.381	0.46571332	0.830121021
C9JME2	2xPhospho [S909(0); T914(0); S920(0); S925(97.2); S927(2.8); T929(0.2); S930(99.9)]	1.242	0.312665174	0.921519407
C9JNL1	3xPhospho [S203(33.3); T204(33.3); T205(33.3); S207(100); T209(0); S210(0.1); S211(99.9)]	0.795	-0.330973234	0.955430698
C9JNL1	2xPhospho [S203(0); T204(0); T205(0); S207(96.6); T209(0.3); S210(6.6); S211(96.6)]	0.854	-0.227692025	0.963131675
C9JNL1	3xPhospho [S203(3.9); T204(3.9); T205(92.2); S207(100); T209(0); S210(0); S211(100)]	0.869	-0.202571918	0.965139891

C9JNL1	2xPhospho [S203(0); T204(0); T205(0); S207(96.6); T209(3.4); S210(99.9); S211(0.1)]	0.948	-0.077041036	0.982345843
C9JPL0	1xPhospho [S352(100); S355(0)]	1.4	0.485426827	0.83187574
C9JPL0	3xPhospho [T325(0); S329(0); T330(0); S335(100); S338(100); S341(0.1); S342(99.9)]	1.341	0.423309237	0.848012696
C9JPL0	2xPhospho [S352(100); S355(100)]	1.409	0.494671612	0.872768733
C9JPL0	2xPhospho [T325(0); S329(0); T330(0); S335(100); S338(0); S341(2.8); S342(97.2)]	0.892	-0.164884385	0.965013037
C9JPT4	3xPhospho [S411(100); S417(100); S420(100)]	0.223	-2.164884385	0.132779461
C9JPT4	2xPhospho [S411(0); S417(100); S420(100)]	1.067	0.093560176	0.980895643
C9JRJ5	1xPhospho [S314(3.7); S316(96.3); S323(0); S327(0)]	1.658	0.729444007	0.669782374
C9JRJ5	2xPhospho [S272(100); S277(100)]	0.64	-0.64385619	0.879063032
C9JRJ5	1xPhospho [S384(100); T386(0); S387(0); S394(0); T395(0); S400(0)]	1.32	0.40053793	0.879426164
C9JSK5	2xPhospho [S71(0); S73(0); S75(0); S77(100); S83(100); T85(0)]	2.445	1.289834465	0.121595606
C9JSK5	3xPhospho [S71(0.2); S73(99.8); S75(50); S77(50); S83(100); T85(0)]	2.241	1.164142649	0.332208392
C9JSN6	1xPhospho [Y29(0); T30(0); T39(0); S46(100); T50(0); T52(0); S56(0); S61(0); T63(0); S64(0)]	0.01	-6.64385619	8.35E-17
C9JSN6	2xPhospho [T416(0); S419(0); S420(0); S427(0); S428(100); S437(50); S438(50)]	0.388	-1.365871442	0.24072149
C9JSN6	1xPhospho [S673(100); T678(0)]	0.663	-0.592919225	0.783976635
C9JSN6	1xPhospho [S641(100)]	1.313	0.392866916	0.87877743
C9JSN6	2xPhospho [S726(0); S729(97); S730(2.9); T731(0.1); S733(100); S739(0); T744(0); S750(0); S752(0)]	0.768	-0.380821784	0.90092486
C9JSN6	2xPhospho [S527(0); T529(100); S531(94.9); T534(2.6); T537(2.6)]	0.83	-0.268816758	0.942954921
C9JSN6	2xPhospho [T548(100); S552(100); S554(0); S555(0)]	0.845	-0.242976753	0.94797789
C9JSN6	2xPhospho [T492(100); T498(100); S501(0); S502(0)]	0.758	-0.399730246	0.965013037
C9JSN6	2xPhospho [T703(0); Y711(0); S713(100); S717(0); T720(1.8); S721(98.2)]	1.125	0.169925001	0.965351295
C9JSN6	3xPhospho [T703(0); Y711(0); S713(100); S717(100); T720(2.9); S721(97.1)]	1.116	0.158337027	0.969680228
C9JSN6	1xPhospho [S673(100); T678(0)]	0.782	-0.354759487	0.981858798
C9JSN6	1xPhospho [T529(0); S531(98.4); T534(1.6); T537(0)]	0.896	-0.158429363	1
C9JSN6	1xPhospho [S726(0); S729(0); S730(0); T731(0); S733(100); S739(0); T744(0); S750(0); S752(0)]	0.973	-0.03948829	1
C9JSR2	2xPhospho [S134(100); S137(100); S141(0); S142(0); S143(0); S144(0); T146(0); S152(0)]	0.552	-0.857259828	0.763990921
C9JSZ1	1xPhospho [S105(100); T108(0)]	0.01	-6.64385619	8.35E-17
C9JTT7	1xPhospho [T8(2.1); S9(97.9); S11(0); T12(0); T17(0)]	1.485	0.570462931	0.79866369
C9JUF4	1xMet-loss+Acetyl [N-Term]; 1xPhospho [S2(100); T14(0)]	13.081	3.709400929	1.30E-05
C9JUF4	1xMet-loss+Acetyl [N-Term]; 1xPhospho [S2(100); T14(0); T21(0)]	1.09	0.124328135	0.964978421
C9JUF4	1xPhospho [Y87(0); S90(100); Y93(0)]; P56211 1xPhospho [Y59(0); S62(100); Y65(0)]	1.017	0.024319679	0.973763863
C9JUF4	2xPhospho [T116(0); T122(0); S131(100); S132(100); T135(0); S136(0)]	0.92	-0.120294234	0.980895643
C9JUF4	1xPhospho [Y41(0); S43(100); S51(0)]	0.846	-0.241270432	0.983706057
C9JUG3	1xPhospho [S9(100); S13(0); S16(0); T17(0)]	1.493	0.578214165	0.797966154
C9JUG3	2xPhospho [S9(49.9); S13(49.9); S16(6.9); T17(93.4)]	0.674	-0.569179503	0.891862526
C9JUG3	1xPhospho [S70(0); T79(0); S85(0); S87(100)]	1.243	0.313826296	0.922868578
C9JUG3	1xPhospho [S37(100)]	1.068	0.094911647	0.972363997
C9JUG3	1xPhospho [S70(0); T79(0); S85(0); S87(100)]	1.015	0.021479727	0.980895643
C9JVU9	2xPhospho [S267(100); S278(100)]	1.347	0.429749851	0.836554173
C9JW18	1xPhospho [T268(0); S270(0); S271(0); S284(100); T292(0); S293(0); Y298(0); T304(0)]	100	6.64385619	8.35E-17
C9JW18	1xPhospho [S879(100); T881(0); T890(0)]	0.58	-0.785875195	0.816750637
C9JW18	2xPhospho [S251(100); S260(95.1); S264(4.9)]	0.859	-0.219269964	0.980895643
C9JW18	2xPhospho [S745(100); S763(96.2); S765(3.8)]	0.899	-0.153606979	0.980895643
C9JW18	1xPhospho [Y200(0); S210(100); S225(0); S227(0)]	1	0	0.980895643
C9JW18	1xPhospho [S745(0); S763(96.6); S765(3.4)]	1.064	0.089498151	0.980895643
C9JW18	1xPhospho [S251(0); S260(100); S264(0)]	0.993	-0.010134377	0.99184412
C9JX83	3xPhospho [S23(100); S27(100); S31(100); S38(0)]	0.01	-6.64385619	8.35E-17
C9JX83	3xPhospho [S23(100); S27(100); S31(100); S38(0); S44(0)]	0.01	-6.64385619	8.35E-17
C9JX83	1xPhospho [S102(100); S112(0)]	100	6.64385619	8.35E-17
C9JX83	3xPhospho [S18(0.1); S23(99.9); S27(100); S31(100); S38(0)]	0.508	-0.977099598	0.266630118
C9JX83	3xPhospho [S23(100); S27(100); S31(100); S38(0)]	0.636	-0.652901329	0.624540466
C9JX83	2xPhospho [S18(0); S23(100); S27(100); S31(0); S38(0)]	0.597	-0.744197163	0.915837201
C9JX83	2xPhospho [S23(0); S27(100); S31(100); S38(0)]	1.044	0.062121712	0.973263372
C9JX83	2xPhospho [S23(0); S27(100); S31(100); S38(0)]	0.892	-0.164884385	0.999714477
C9JX92	2xPhospho [S1693(0); S1698(100); S1701(100); S1702(0); T1709(0)]	100	6.64385619	8.35E-17
C9JX92	2xPhospho [T1181(0); S1182(0); S1184(95.5); T1185(4.5); T1190(0); T1194(100); Y1202(0); T1206(0); T1208(0); Y1209(0); T1210(0)]	0.364	-1.457989644	0.33990982
C9JXI1	1xPhospho [T23(0); Y25(0); T27(0); S32(0); S34(100)]	2.076	1.053806444	0.563031663
C9JYQ9	2xPhospho [Y107(0); S111(0); S117(100); S119(100)]	0.231	-2.114035243	0.00346143
C9JYQ9	1xPhospho [Y107(0); S111(0); S117(100); S119(0)]	0.673	-0.57132159	0.703278942
C9JZR2	1xPhospho [T916(0); S920(100)]	100	6.64385619	8.35E-17
C9JZR2	1xPhospho [S920(100)]	4.997	2.321062218	0.00924067
C9JZR2	1xPhospho [S320(100); Y321(0); S331(0); Y334(0); Y335(0)]	2.791	1.480782124	0.12942406
C9JZR2	1xPhospho [Y600(0); T611(0); S617(100)]	2.239	1.162854528	0.269635367
C9JZR2	2xPhospho [T916(100); S920(100)]	2.031	1.02219024	0.374360028
C9JZR2	2xPhospho [S346(0); S349(100); S352(100)]	0.582	-0.780908942	0.477649934
C9JZR2	1xPhospho [S268(100); S269(0)]	1.819	0.863145543	0.491917574
C9JZR2	1xPhospho [S879(100); S891(0); S895(0); T897(0)]	1.714	0.777367109	0.823333961
C9JZR2	1xPhospho [Y217(0); Y221(0); S225(0); Y228(0); S230(100); S232(0)]	1.193	0.254594043	0.912209811
C9JZR2	2xPhospho [S346(100); S349(100); S352(0)]	1.266	0.340277405	0.91490394
C9JZR2	1xPhospho [S288(100); Y291(0); Y296(0); S300(0); Y302(0); T304(0)]	1.257	0.32998465	0.921247725
C9JZR2	3xPhospho [S346(100); S349(100); S352(100)]	0.634	-0.657445255	0.938029982
C9JZR2	1xPhospho [S346(100); S349(0); S352(0)]	1.146	0.196607044	0.943912599

C9JZR2	3xPhospho [S346(100); S349(100); S352(100)]	0.936	-0.095419565	0.979498769
C9JZR2	1xPhospho [S252(100); Y257(0)]	0.966	-0.049904906	0.981858798
C9JZR2	1xPhospho [T152(0); S168(0); S169(0); S171(100); Y174(0); T177(0)]	0.902	-0.148800661	1
C9JZW3	1xPhospho [Y79(0); T87(0); T88(0); S90(0); T93(0); S95(0); S106(100); S112(0)]	0.529	-0.918660373	0.325586593
C9JZW3	1xPhospho [S106(100); S112(0)]	0.521	-0.940644722	0.48101191
C9JZW3	2xPhospho [Y79(0); T87(0); T88(0); S90(0); T93(0.2); S95(99.8); S106(100); S112(0)]	0.653	-0.614845103	0.681621987
C9JZW3	1xPhospho [S106(100); S112(0)]	0.714	-0.486004021	0.824414104
C9JZW3	2xPhospho [Y79(0); T87(0); T88(0); S90(2.5); T93(48.8); S95(48.8); S106(100); S112(0)]	1.137	0.185232254	0.964978421
C9JZW3	1xPhospho [Y79(0); T87(0); T88(0); S90(0); T93(0); S95(0); S106(100); S112(0)]	0.912	-0.13289427	0.971838536
C9K0U5	1xPhospho [S32(0); S34(1.5); S36(98.5)]	0.652	-0.61705613	0.885613358
D3DQV9	1xPhospho [S403(0); T418(96.9); S420(3.1)]	1.607	0.684369929	0.690107651
D3DQV9	1xPhospho [S432(0); S436(0); Y439(0); S443(0); S448(0); S454(100)]	1.162	0.216610069	0.965013037
D3DQV9	1xPhospho [T506(0); T508(100); T514(0)]	0.914	-0.12973393	0.986075329
D3K174	1xPhospho [S264(0); S267(100); S275(0)]	1.365	0.448900951	0.810764434
D3K174	1xPhospho [S85(0); S93(100); S95(0); Y96(0); T98(0); S100(0)]	1.343	0.425459305	0.883306273
D3K174	1xPhospho [S251(100)]	1.117	0.159629186	0.943939997
D3YHP0	2xPhospho [S252(100); Y253(0); S256(0); S259(6.3); S260(6.3); S263(87.3); S265(0); S267(0); Y272(0); S273(0)]	2.279	1.188400925	0.28501004
D4QA03	2xPhospho [S165(0); T171(0); S176(91.3); T178(4.3); S179(4.3); S189(100)]	100	6.64385619	8.35E-17
D4QA03	2xPhospho [T8(100); S16(100); S20(0)]	1.976	0.982582947	0.493928854
D4QA03	2xPhospho [S165(0); T171(4.1); S176(95.5); T178(0.2); S179(0.2); S189(100)]	1.575	0.655351829	0.714380386
D4QA03	3xPhospho [S165(0); T171(100); S176(100); T178(0); S179(0); S189(100)]	0.673	-0.57132159	0.848772937
D4QA03	3xPhospho [S165(0); T171(100); S176(100); T178(0); S179(0); S189(100)]	0.823	-0.281035664	0.958702638
D6R9L5	2xPhospho [S37(100); S56(100)]	100	6.64385619	8.35E-17
D6R9L5	1xPhospho [S37(100)]	2.652	1.407080775	0.131022882
D6R9L5	1xPhospho [T72(0); S76(0); S77(100)]	1.837	0.877351626	0.504163686
D6R9S4	2xPhospho [S65(49.3); S67(49.3); S68(1.6); T69(99.9)]	1.648	0.720716243	0.737789345
D6R9T3	1xPhospho [T255(0); S261(0); S267(0.1); S268(99.9)]	0.801	-0.320125852	0.94932168
D6RA26	1xPhospho [S152(94.9); T158(4.8); S162(0.3); T167(0); T170(0)]	100	6.64385619	8.35E-17
D6RA26	1xPhospho [T72(0); S74(100); Y75(0)]	2.413	1.270827916	0.469342919
D6RA44	1xPhospho [S43(100); S45(0)]	1.141	0.190298792	0.943912599
D6RAA5	1xPhospho [S657(3.1); S659(93.8); T661(3.1); S664(0); S666(0); S667(0); T671(0); T677(0)]	1.266	0.340277405	0.862820654
D6RAA5	2xPhospho [S437(100); S444(0); Y445(0); T451(5.5); S455(94.5)]	0.655	-0.610433188	0.93019184
D6RAA5	1xPhospho [S437(100); S444(0); Y445(0); T451(0); S455(0)]	0.777	-0.364013496	0.94432975
D6RAA5	2xPhospho [S657(0.2); S659(99.8); T661(99.7); S664(0.2); S666(0.2); S667(0); T671(0); T677(0)]	0.909	-0.1376478	0.98356941
D6RAA5	2xPhospho [T392(0); S395(100); S399(100)]	1.006	0.008630305	1
D6RAT0	1xPhospho [Y219(0); S226(100)]	1.268	0.342554745	0.880477214
D6RAU6	1xPhospho [S8(0); T10(0.1); S12(99.9); S18(0)]	0.757	-0.401634795	0.974924677
D6RB01	3xPhospho [S311(0.2); T312(3.7); S316(96.2); S318(100); S319(100)]	0.01	-6.64385619	8.35E-17
D6RB01	2xPhospho [S575(100); S576(100); S577(0)]	0.353	-1.502259911	0.138424584
D6RB01	2xPhospho [S575(100); S576(50); S577(50)]	0.548	-0.867752202	0.657415306
D6RB01	1xPhospho [S466(100); S467(0); T472(0); S474(0)]	1.201	0.264236151	0.929187903
D6RB01	1xPhospho [S218(100); Y221(0); S225(0); Y229(0)]	0.94	-0.089267338	1
D6RCP9	1xPhospho [S63(0); T64(0); T72(0); S74(100)]	0.928	-0.10780329	0.99184412
D6RD44	1xPhospho [S102(100); S108(0); T110(0)]	0.853	-0.229382353	0.99184412
D6RD83	2xPhospho [S25(0); S34(0); S36(3.3); S37(96.7); S41(99.9); T45(0.1)]	1.117	0.159629186	0.965013037
D6RER5	1xPhospho [S17(100); S19(0); S26(0)]	0.736	-0.442222329	0.939053555
D6RER5	1xPhospho [S407(0); S412(100); T417(0)]	0.859	-0.219269964	0.973763863
D6RF77	1xPhospho [S83(0); T84(0); T90(0); S92(100)]	0.576	-0.795859283	0.774421636
D6RHI7	2xPhospho [T241(100); S248(100)]	100	6.64385619	8.35E-17
D6RIC3	1xPhospho [Y15(0); S16(100)]	0.838	-0.254977851	0.971937853
D6RIE8	2xPhospho [S149(0); S152(99.7); T154(86.8); S155(4.3); S158(4.6); T159(4.6); T161(0); T164(0); T166(0); T169(0); S171(0); S173(0); T174(0)]	0.852	-0.231074664	0.973763863
D6RIS1	3xPhospho [T110(100); T114(0.1); T115(99.9); S121(100)]	0.01	-6.64385619	8.35E-17
D6RIS1	2xPhospho [T110(100); T114(2.1); T115(97.9)]	2.013	1.009347172	0.505417445
D6RIY6	2xPhospho [T359(0); S364(0); S368(0); S376(100); S378(100)]	100	6.64385619	8.35E-17
D6RIY6	1xPhospho [T289(0); S290(100)]	1.259	0.332278283	0.884551994
E1P5H9	2xPhospho [S34(0); T37(0); S40(100); S43(100)]	2.341	1.227124934	0.403398043
E5RHA3	1xPhospho [S160(100); T165(0)]	0.886	-0.174621396	0.995968957
E5RHI2	1xMet-loss+Acetyl [N-Term]; 1xPhospho [S6(100)]	100	6.64385619	8.35E-17
E5RHI2	1xPhospho [T187(0); S188(100)]	1.232	0.301002256	0.907632224
E5RIF2	1xPhospho [S1023(100); S1029(0)]	0.975	-0.036525876	1
E5RIS7	1xPhospho [T96(0); S97(0); S100(100)]	4.748	2.247319935	0.000561468
E5RIS7	1xPhospho [T96(0); S97(0); S100(100)]	2.418	1.273814245	0.570904793
E5RIT6	1xPhospho [S23(100)]	100	6.64385619	8.35E-17
E5RJ68	1xPhospho [Y225(0); S227(100)]	2.928	1.549915554	0.045907182
E5RJI2	1xPhospho [S7(100); T32(0); T38(0); T39(0); S40(0); S41(0); S42(0); S44(0)]	1.915	0.937344392	0.539112137
E5RJM2	1xPhospho [S119(100)]	0.532	-0.910501849	0.848293321
E7EN20	1xPhospho [S751(100)]	1.93	0.948600847	0.434658428
E7EN20	2xPhospho [T717(0); T734(100); S737(100)]	1.195	0.257010618	0.938190912
E7EN73	2xPhospho [T974(100); S978(100); T984(0); S985(0)]	1.012	0.01720929	0.974924677
E7ENQ1	3xPhospho [T319(0); Y321(0); Y323(0); S324(100); S326(100); S341(0); S342(0); S350(50); T351(50)]	2.325	1.217230716	0.326165136

E7ENQ1	1xPhospho [S592(0); S594(0); S595(0); S596(0); S598(0); S599(0); S601(0); S603(0); S607(0); S611(100); S613(0); S615(0)]	1.267	0.341416524	0.908086456
E7ENQ1	2xPhospho [S767(98.1); S768(50.9); S769(50.9); S770(0.1); T772(0)]	1.123	0.167357928	0.942574721
E7ENQ1	1xPhospho [S516(0); S519(100)]	1.075	0.10433666	0.969482098
E7ENQ1	1xPhospho [S815(100); T822(0); T824(0); S828(0); T830(0)]	1.032	0.045442971	0.978952227
E7ENQ1	2xPhospho [T319(0); Y321(0); Y323(0); S324(100); S326(100); S341(0); S342(0); S350(0); T351(0)]	0.926	-0.110915901	0.986075329
E7ENQ1	2xPhospho [S592(94.5); S594(5.4); S595(0); S596(0); S598(0); S599(0); S601(0); S603(0); S607(0.3); S611(99.7); S613(0); S615(0)]	0.939	-0.090802937	1
E7EPC6	1xPhospho [S678(100)]	8.24	3.042644337	1.44E-05
E7EPC6	1xPhospho [S678(100)]	4.09	2.032100843	0.000776833
E7EPC6	1xPhospho [S669(0); S676(0); S678(100)]	5.593	2.48362233	0.005405822
E7EPC6	1xPhospho [S669(0); S676(0); S678(100)]	2.757	1.463099267	0.026789262
E7EPC6	2xPhospho [S669(50); S676(50); S678(100)]	2.826	1.498761466	0.096505146
E7EPU2	2xPhospho [T92(9.2); S98(90.8); T104(0); T105(0); Y106(0); T107(0); S108(0); S111(0); S120(0.1); T121(0.1); S123(99.7)]	0.01	-6.64385619	8.35E-17
E7EQL5	1xPhospho [S86(0); S88(0); T89(0); S91(0); S95(100); S98(0); S105(0)]	100	6.64385619	8.35E-17
E7EQL5	2xPhospho [S66(0); T70(0); S73(100); S81(0.2); S83(3.9); S84(95.9)]	1.488	0.573374526	0.789364451
E7EQV9	1xPhospho [T80(99.9); Y81(0.1)]	2.253	1.171847314	0.485808589
E7ER89	1xPhospho [S19(0); T20(0); Y24(100); S30(0)]	0.01	-6.64385619	8.35E-17
E7ERL6	1xPhospho [S144(0); S148(98.5); S151(1.5); T153(0)]	0.121	-3.046921047	0.000332545
E7ERY9	1xPhospho [S898(100); T908(0); T916(0)]	0.517	-0.951763814	0.698613591
E7ERY9	3xPhospho [S920(100); S921(100); S925(100); S935(0); T940(0)]	0.651	-0.619270551	0.907438338
E7ERY9	2xPhospho [S920(0.1); S921(99.9); S925(100); S935(0); T940(0)]	0.848	-0.23786383	0.94797789
E7ERY9	2xPhospho [S898(100); T908(100); T916(0)]	0.852	-0.231074664	0.96229367
E7ERY9	3xPhospho [S920(100); S921(100); S925(100); S935(0); T940(0)]	1.109	0.149259365	0.965013037
E7ERY9	2xPhospho [S898(100); T908(100); T916(0)]	1.045	0.063502942	0.982345843
E7ESD2	2xPhospho [S158(100); S160(100)]	0.308	-1.698997744	0.159012073
E7ESD2	3xPhospho [S688(0); S697(100); S700(100); S704(100); T707(0); S708(0); T713(0)]	0.276	-1.857259828	0.221126321
E7ESD2	2xPhospho [S614(0); S619(100); S620(100); S631(0); T633(0); S637(0); S639(0)]	0.515	-0.957355663	0.331363251
E7ESD2	2xPhospho [T331(100); S333(100)]	0.55	-0.862496476	0.727550885
E7ESD2	2xPhospho [S284(100); T287(0); S288(100)]	0.611	-0.710755715	0.75891781
E7ESD2	1xPhospho [T331(0); S333(100)]	1.377	0.461528559	0.85992414
E7ESD2	2xPhospho [T331(100); S333(100)]	0.6	-0.736965594	0.879063032
E7ESD2	2xPhospho [T246(0); T247(0); S250(100); S265(100); T279(0)]	0.686	-0.543719518	0.897258645
E7ESU4	1xPhospho [S912(100); S915(0)]	1.487	0.572404647	0.767551297
E7ESU4	2xPhospho [S912(100); S915(100)]	0.87	-0.200912694	1
E7ET96	3xPhospho [S428(0); T431(0); S434(100); S435(100); T438(0); S442(100)]	0.674	-0.569179503	0.880267055
E7ET96	1xPhospho [S547(0); S548(0); T555(0); T559(0.1); S563(100)]	1.035	0.049630768	0.981671686
E7ET96	2xPhospho [S428(0); T431(0); S434(100); S435(100); T438(0); S442(0)]	0.967	-0.048412205	0.99184412
E7ETG4	1xPhospho [S7(100)]	0.269	-1.894321922	0.223400676
E7ETI4	2xPhospho [S206(0); S210(0); S213(100); S219(100); S223(0); T227(0)]	1.285	0.361768359	0.910211107
E7ETI4	3xPhospho [S206(100); S210(0); S213(100); S219(100); S223(0); T227(0)]	0.833	-0.263611599	0.965248196
E7ETY2	1xPhospho [T1097(0); S1101(0); T1105(0); T1109(0); S1110(3.2); S1113(93.6); T1114(3.2); S1115(0.1)]	4.282	2.098284796	0.002100879
E7ETY2	2xPhospho [T1097(0); S1101(100); T1105(0); T1109(0); S1110(100); S1113(0); T1114(0); S1115(0)]	1.181	0.240008965	0.944828278
E7EU19	2xPhospho [S863(100); S866(100); S871(0); Y876(0)]	1.088	0.121678557	0.969680228
E7EUA9	2xPhospho [S1186(100); S1189(50); S1190(50); T1193(0); T1197(0)]	100	6.64385619	8.35E-17
E7EUA9	2xPhospho [S1269(0); S1271(0); T1272(0); T1278(0); S1282(100); S1286(100); S1288(0); S1291(0); S1292(0); S1293(0)]	1.544	0.626672753	0.767575271
E7EUG6	1xPhospho [S300(0); T304(96.8); S306(3.2); S311(0); T312(0); S313(0); S314(0); T315(0)]	0.01	-6.64385619	8.35E-17
E7EUG6	3xPhospho [S292(100); S295(100); S296(100); S300(0); T304(0); S306(0); S311(0); T312(0); S313(0); S314(0); T315(0)]	100	6.64385619	8.35E-17
E7EUG6	3xPhospho [S300(100); T304(100); S306(4.5); S311(4.5); T312(4.5); S313(4.5); S314(4.5); T315(77.7)]	100	6.64385619	8.35E-17
E7EUG6	1xPhospho [T342(0); S346(100); S348(0)]	1.337	0.418999465	0.880267055
E7EUG6	2xPhospho [T342(0); S346(0); S348(100); S351(50); T352(50); S357(0)]	1.247	0.318461465	0.893580559
E7EUG6	2xPhospho [T342(0); S346(0); S348(100); S351(2.4); T352(97.6); S357(0)]	1.149	0.200378798	0.94797789
E7EUG6	1xPhospho [T360(0.1); T361(99.9); S364(0.1); T370(0)]	1.163	0.217851097	0.960178862
E7EUG6	2xPhospho [S300(100); T304(50); S306(50); S311(0); T312(0); S313(0); S314(0); T315(0)]	1.13	0.176322773	0.965139891
E7EUG6	2xPhospho [S351(0); T352(0); S357(99.9); T360(0.3); T361(99.9); S364(0); T370(0)]	0.852	-0.231074664	0.973263372
E7EUG6	3xPhospho [T342(0); S346(0.4); S348(99.6); S351(94.4); T352(5.7); S357(100)]	1	0	0.97998947
E7EUG6	3xPhospho [S351(50); T352(50); S357(95.9); T360(4.3); T361(99.8); S364(0); T370(0)]	0.956	-0.064917477	0.980895643
E7EUN2	1xPhospho [S580(100); T584(0); S585(0)]	9.814	3.294841271	0.005437703
E7EUT8	3xPhospho [T299(0); S312(100); S314(100); S316(100); Y325(0)]	0.609	-0.715485867	0.744877142
E7EUY0	1xPhospho [S2598(0); T2599(0); T2602(0); T2608(0); S2611(100); T2614(0); T2617(0)]	0.569	-0.813499442	0.659358171
E7EUY0	2xPhospho [S2598(0); T2599(0); T2602(0); T2608(100); S2611(100); T2614(0); T2617(0)]	0.732	-0.450084446	0.879063032
E7EUY0	1xPhospho [T2637(100); T2644(0); T2646(0); T2648(0)]	1.191	0.252173413	0.93019184
E7EUY0	1xPhospho [S2598(0); T2599(0); T2602(0); T2608(0); S2611(100); T2614(0); T2617(0)]	0.848	-0.23786383	0.938483927
E7EUY0	2xPhospho [T2637(100); T2644(0); T2646(100); T2648(0)]	0.855	-0.226003675	0.943912599
E7EUY0	2xPhospho [S2598(0); T2599(0); T2602(0); T2608(100); S2611(100); T2614(0); T2617(0)]	0.759	-0.397828209	0.96293109
E7EUY0	2xPhospho [S2653(0); S2654(0); T2659(0); S2661(0); S2662(0); T2663(0); T2670(48.5); S2671(48.5); S2673(9.5); S2674(93); S2676(0.5)]	0.955	-0.066427362	1
E7EV56	2xPhospho [T77(0.4); T79(99.6); T82(94.5); S86(5.5)]	100	6.64385619	8.35E-17
E7EV56	1xPhospho [T61(0); S65(100); S68(0); S69(0)]	1.889	0.917622702	0.543749652

E7EV56	3xPhospho [S1258(100); S1261(100); S1263(96); S1264(4); T1272(0); T1273(0); T1275(0)]	0.556	-0.846843212	0.728968862
E7EV56	1xPhospho [Y88(0); S90(0); S93(100)]	1.597	0.675364313	0.74970909
E7EV56	2xPhospho [T530(96.9); S533(3.1); Y535(0); S537(100); S542(0); T546(0)]	1.483	0.568518598	0.772467589
E7EV56	2xPhospho [T61(0); S65(100); S68(100); S69(0)]	1.354	0.437227739	0.854283637
E7EV56	3xPhospho [T530(100); S533(100); Y535(0); S537(100); S542(0); T546(0)]	1.295	0.372952098	0.875854788
E7EV56	2xPhospho [S1711(100); S1714(100); S1722(0); S1728(0); S1732(0)]	0.79	-0.340075442	0.881459128
E7EV56	1xPhospho [S1676(100); T1691(0); T1693(0)]	1.253	0.325386415	0.896967324
E7EV56	2xPhospho [T61(0); S65(100); S68(100); S69(0)]	1.252	0.324234562	0.922868578
E7EV56	1xPhospho [S961(100); Y966(0)]	0.794	-0.332789088	0.94813191
E7EV56	3xPhospho [S1258(50); S1261(50); S1263(100); S1264(100); T1272(0); T1273(0); T1275(0)]	1.137	0.185232254	0.960178862
E7EV56	2xPhospho [S1711(50); S1714(50); S1722(100); S1728(0); S1732(0)]	1.059	0.082702589	0.965248196
E7EV56	3xPhospho [S1711(100); S1714(100); S1722(100); S1728(0); S1732(0)]	0.866	-0.20756107	0.971453936
E7EV56	1xPhospho [S110(100)]	1.008	0.011495639	0.980895643
E7EV56	2xPhospho [T1175(0); Y1177(0); S1186(0.2); S1187(0.2); S1188(99.7); S1189(100)]	1.019	0.027154052	0.986075329
E7EVA0	1xPhospho [S1391(2.5); S1392(97.5)]	1.617	0.693319679	0.820661452
E7EVC7	1xPhospho [S304(100); S306(0); S307(0); T317(0); S321(0)]	1.136	0.183962835	0.958702638
E7EVD1	1xPhospho [T559(0); S560(0); T562(0); S570(100)]	1.293	0.370722275	0.905689075
E7EVG2	2xPhospho [S343(0); T346(0); Y351(0); S353(100); S355(100)]	100	6.64385619	8.35E-17
E7EVG2	2xPhospho [S343(0); T346(0); Y351(0); S353(100); S355(100)]	0.408	-1.293358943	0.174949405
E7EVG2	3xPhospho [T9(0.6); S10(0.6); S12(7.2); S13(91.6); S14(99.3); S16(0.7); S25(6.8); S27(6.8); T28(86.4); S32(0.1)]	1.46	0.545968369	0.763576928
E7EVG2	2xPhospho [T9(47.9); S10(47.9); S12(2.1); S13(2.1); S14(100); S16(0); S25(0); S27(0); T28(0); S32(0)]	1.114	0.155749233	0.964978421
E7EVX2	1xPhospho [S271(100); T281(0); S282(0)]	1.42	0.50589093	0.820702501
E7EVX2	1xPhospho [T39(0); S40(0); S41(100); T43(0); T50(0)]	1.236	0.305678743	0.921519407
E7EVX2	1xPhospho [S93(100); S96(0)]	0.81	-0.304006187	0.97998947
E7EVX8	1xPhospho [S439(0.2); S440(0.2); T442(0.2); S444(0.2); S445(0.2); T449(99)]	100	6.64385619	8.35E-17
E7EW05	1xPhospho [Y543(0); S548(100)]	1.516	0.600269754	0.774332996
E7EW05	1xPhospho [Y543(0); S548(100); S558(0)]	0.908	-0.139235797	0.980895643
E7EWI9	1xPhospho [S94(100); T102(0)]	1.441	0.527070336	0.755586549
E7EWI9	2xPhospho [S299(0); S300(0); S302(100); Y304(0); Y308(0); S310(100); S314(0); Y317(0); S319(0)]	1.229	0.297484916	0.920957334
E7EWI9	1xPhospho [S299(0); S300(0); S302(0); Y304(0); Y308(0); S310(100); S314(0); Y317(0); S319(0)]	1.165	0.220329955	0.953387553
E7EWM1	1xPhospho [S565(100); S575(0)]	0.01	-6.64385619	8.35E-17
E7EX17	1xPhospho [S192(100); T195(0); T197(0)]	0.149	-2.746615764	0.021768171
E7EX17	1xPhospho [S359(100); T369(0)]	2.717	1.442014566	0.047617772
E7EX17	1xPhospho [T205(0); S207(100); Y211(0)]	0.649	-0.623709617	0.729469105
E7EX17	2xPhospho [S406(100); S409(1.5); T412(98.5)]	0.654	-0.612637459	0.766475576
E7EX17	2xPhospho [S447(50); S450(50); T455(0); S464(99.9); T466(0); S467(0)]	0.723	-0.467932448	0.79316576
E7EX17	2xPhospho [S450(100); T455(0); S464(100); T466(0); S467(0)]	0.685	-0.545824107	0.818010303
E7EX17	2xPhospho [S406(100); S409(100); T412(0)]	1.334	0.415758667	0.851986218
E7EX17	2xPhospho [S447(100); S450(100); T455(0)]	0.759	-0.397828209	0.855302203
E7EX17	1xPhospho [S93(100); Y96(0); T97(0); Y105(0); T108(0); S111(0)]	0.693	-0.529072743	0.867705101
E7EX17	1xPhospho [Y598(0); S602(100); Y614(0)]	0.688	-0.53951953	0.920957334
E7EX17	1xPhospho [S450(0); T455(0); S464(100); T466(0); S467(0)]	1.226	0.293958979	0.921519407
E7EX73	2xPhospho [S1022(100); S1024(100)]	100	6.64385619	8.35E-17
E7EX73	2xPhospho [S934(0); S935(0); S938(0); S943(0.1); S947(99.9); T954(96.7); S955(3.3); T956(0)]	6.486	2.697329024	9.77E-05
E7EX73	1xPhospho [T910(0); S914(100); S917(0)]	3.137	1.649385529	0.008262176
E7EX73	1xPhospho [S934(0); S935(0); S938(0); S943(0); S947(100); T954(0); S955(0); T956(0)]	3.417	1.772730248	0.024034163
E7EX73	1xPhospho [Y430(0); Y432(0); S434(100)]	1.606	0.683471893	0.737789345
E7EX73	1xPhospho [S1031(100); S1036(0)]	1.444	0.530070742	0.79670828
E7EX73	1xPhospho [S961(0); T969(0); S971(100); T972(0)]	1.467	0.552868871	0.815248216
E7EX73	1xPhospho [S1068(100)]	0.704	-0.506352666	0.870911172
E7EX73	1xPhospho [S1022(0); S1024(100)]	1.327	0.408168371	0.872730156
E7EX73	1xPhospho [S1022(0); S1024(100)]	1.208	0.272620455	0.889306217
E7EX73	1xPhospho [S1068(100)]	1.284	0.360645202	0.890292207
E7EX73	2xPhospho [S1022(0); S1024(100); S1031(100)]	0.679	-0.55851652	0.920957334
E7EX73	2xPhospho [S1022(100); S1024(100)]	1.142	0.191562651	0.965013037
E7EX73	2xPhospho [T38(0.2); S40(4.5); T41(95.2); T43(100); T47(0); T59(0)]	0.898	-0.15521265	0.98527838
E7EXA6	1xPhospho [S1074(0); S1080(100)]	1.64	0.713695815	0.663136789
E9PAT7	1xPhospho [S134(100)]	100	6.64385619	8.35E-17
E9PAV9	2xPhospho [T119(0); T121(0); S122(0); S133(100); S135(100); S142(0)]	0.01	-6.64385619	8.35E-17
E9PAV9	3xPhospho [T119(0); T121(0); S122(0); S133(100); S135(100); S142(0.4); S144(99.6)]	0.078	-3.680382066	0.000182361
E9PAV9	1xPhospho [T119(0); T121(0); S122(0); S133(0); S135(100); S142(0)]	0.107	-3.224317298	0.004911621
E9PAV9	2xPhospho [T119(0); T121(0); S122(0); S133(100); S135(100); S142(0)]	0.636	-0.652901329	0.79670828
E9PAZ2	1xPhospho [S315(100)]	0.978	-0.03209363	0.989838391
E9PB19	2xPhospho [S494(100); S502(2.7); S503(97.3)]	100	6.64385619	8.35E-17
E9PB38	2xPhospho [Y322(0); S327(0); S329(48.8); S330(48.8); S331(2.5); T333(0); S337(100); S340(0)]	1.093	0.128293401	0.960178862
E9PB51	1xPhospho [S86(100); T88(0); T90(0)]	1.157	0.210388864	0.935688648
E9PB61	1xPhospho [S241(100); S246(0); Y257(0)]	1.065	0.09085343	0.977823205
E9PBB4	1xPhospho [S380(0); S381(100); S383(0); S386(0); T389(0)]	0.976	-0.035046947	0.980895643
E9PBC1	1xPhospho [S172(1.7); S173(98.4); S178(0); T179(0); S180(0); S182(0); Y186(0)]	1.255	0.327687364	0.904774194
E9PBP4	1xPhospho [S689(100); S692(0); S693(0)]	1.729	0.789937869	0.643284326
E9PBP4	3xPhospho [S684(100); S686(100); S689(100); S692(0); S693(0)]	0.666	-0.586405918	0.949541112
E9PBP4	2xPhospho [S684(0); S686(100); S689(100); S692(0); S693(0)]	1.144	0.194087052	0.962617576

E9PBR5	2xPhospho [T539(2); T540(49); S542(49); S551(96); S554(4)]	10.184	3.348232419	0.000240476
E9PBR5	3xPhospho [T474(0); S477(0); T479(0); S484(100); S487(100); S489(100); S494(0); T499(0)]	0.212	-2.23786383	0.02160852
E9PBR5	2xPhospho [T474(0); S477(0); T479(0); S484(100); S487(97.7); S489(2.3)]	0.724	-0.465938398	0.85992414
E9PBR5	3xPhospho [T446(0); S448(0.2); T450(99.8); S452(100); T454(0); T455(0); T459(0); T468(50); S469(50)]	1.108	0.147957881	0.963156927
E9PBR5	1xPhospho [S316(100)]	0.759	-0.397828209	0.964978421
E9PBR5	1xPhospho [S330(100); T335(0)]	1.009	0.012926174	0.980895643
E9PBR5	2xPhospho [T446(0); S448(0); T450(0.2); S452(99.8); T454(0); T455(0); T459(0); T468(50); S469(50)]	0.911	-0.134477041	0.985318278
E9PBR5	2xPhospho [T474(0); S477(0); T479(0); S484(100); S487(100); S489(0); S494(0); T499(0)]	0.967	-0.048412205	1
E9PBS1	1xPhospho [Y22(0); S27(100)]	1.55	0.632268215	0.769621572
E9PC66	2xPhospho [S321(3.4); S323(96.6); S325(3.4); S326(96.6); T329(0); T330(0); T336(0); T338(0)]	100	6.64385619	8.35E-17
E9PC66	1xPhospho [S172(100)]	0.812	-0.300448367	0.980895643
E9PC69	1xPhospho [S409(100); T418(0); S419(0); S421(0); Y422(0); S423(0)]	0.01	-6.64385619	8.35E-17
E9PC69	1xPhospho [T465(0); T466(0); T468(0); S470(100); T471(0); S473(0); S476(0); T477(0); S478(0); T479(0)]	0.422	-1.244685096	0.651672032
E9PC69	1xPhospho [S482(0); S485(100)]	1.52	0.604071324	0.797921038
E9PC69	2xPhospho [T465(0); T466(0); T468(0); S470(100); T471(0); S473(100); S476(0); T477(0); S478(0); T479(0)]	1.279	0.355016264	0.867884785
E9PC69	2xPhospho [Y614(0); T617(0); S620(100); S622(97.6); S625(2.4)]	0.736	-0.442222329	0.936043234
E9PC69	1xPhospho [S570(0); S572(100); S577(0); S578(0); S579(0)]	1.225	0.292781749	0.939268934
E9PC69	2xPhospho [S570(0); S572(100); S577(49.5); S578(49.5); S579(1)]	1.121	0.164786278	0.954539739
E9PC69	2xPhospho [T465(0); T466(0); T468(0); S470(2.5); T471(97.5); S473(100); S476(0); T477(0); S478(0); T479(0)]	0.964	-0.052894948	0.979214627
E9PC69	1xPhospho [Y614(0); T617(0); S620(100); S622(0); S625(0)]	0.984	-0.023269779	0.982743754
E9PC97	1xPhospho [S49(0); S50(0); S54(4.6); S55(4.6); S57(90.5); S61(0.3); S66(0)]; Q8IWS0 1xPhospho [S49(0); S50(0); S54(4.6); S55(4.6); S57(90.5); S61(0.3); S66(0)]	100	6.64385619	8.35E-17
E9PC97	2xPhospho [T134(0); S138(0); S146(100); S155(0.3); S156(99.7)]	1	0	0.983654603
E9PCH6	2xPhospho [S914(100); S924(0); S930(96.4); S931(3.7)]	0.01	-6.64385619	8.35E-17
E9PCM6	2xPhospho [S752(100); S754(99.9); S756(0.1); T763(0); T764(0)]	0.333	-1.586405918	0.238410909
E9PCP3	1xPhospho [S387(100)]	0.555	-0.849440323	0.65190629
E9PCP3	2xPhospho [S222(0); S223(0); S224(100); S225(0); T227(0); S231(100)]	0.692	-0.531156057	0.907312294
E9PCP3	1xPhospho [S222(0); S223(0.1); S224(99.9); S225(0.1); T227(0); S231(0)]	0.772	-0.373327247	0.958702638
E9PCP3	2xPhospho [S222(100); S223(0); S224(0); S225(0); T227(0); S231(100)]	1.046	0.064882852	0.973763863
E9PCP3	2xPhospho [S58(99.6); S59(0.9); S63(99.5); S66(0); S73(0)]	0.846	-0.241270432	0.981671686
E9PCP3	1xPhospho [S58(0.1); S59(3.2); S63(96.7); S66(0); S73(0)]	0.931	-0.103146927	0.99277609
E9PCR4	2xPhospho [S778(100); S780(100)]	0.577	-0.793356776	0.607772032
E9PCT1	2xPhospho [T220(100); S227(100)]	100	6.64385619	8.35E-17
E9PCT1	1xPhospho [Y608(0); S609(100)]	4.523	2.177279996	0.012426465
E9PCT1	2xPhospho [S402(100); T406(100)]	2.522	1.334568276	0.063021527
E9PCT1	1xPhospho [S461(100); S463(0)]	3.152	1.656267535	0.108551616
E9PCT1	2xPhospho [S461(100); S463(100)]	2.407	1.267236142	0.132610738
E9PCT1	2xPhospho [S389(100); S391(50); S393(50)]	2.244	1.166072676	0.136183278
E9PCT1	2xPhospho [S429(0); S431(100); T435(0); S436(100); T438(0)]	1.99	0.992768431	0.419323444
E9PCT1	1xPhospho [Y608(0); S609(100)]	1.529	0.612588407	0.679191074
E9PCT1	2xPhospho [S725(100); S727(99.9); S729(0.1); T730(0)]	1.8	0.847996907	0.691787621
E9PCT1	2xPhospho [S389(100); S391(0); S393(100)]	1.516	0.600269754	0.755151641
E9PCT1	1xPhospho [T884(0); S886(100)]	1.37	0.454175893	0.816296058
E9PCT1	2xPhospho [S402(100); T406(100)]	1.478	0.563646269	0.832048448
E9PCT1	1xPhospho [T220(100); S227(0)]	1.22	0.286881148	0.899633425
E9PCT1	3xPhospho [S781(100); S785(0); S787(100); S789(0); T790(0); S793(100)]	0.812	-0.300448367	0.908086456
E9PCT1	3xPhospho [S426(0); S429(100); S431(100); T435(0.1); S436(99.9); T438(0)]	0.821	-0.284545873	0.941415413
E9PCT1	2xPhospho [S781(100); S785(0); S787(100); S789(0); T790(0); S793(0)]	1.115	0.15704371	0.94797789
E9PCT1	2xPhospho [T884(100); S886(100)]	0.893	-0.16326792	0.965013037
E9PCT1	1xPhospho [S234(0); T239(1.1); S240(97.9); T241(1.1); S242(0)]	1.129	0.175045486	0.965013037
E9PCT1	2xPhospho [S781(100); S785(0); S787(100); S789(0); T790(0); S793(0)]	0.92	-0.120294234	0.973599548
E9PCT1	1xPhospho [T220(100); S227(0)]	1.118	0.160920188	0.973763863
E9PCT1	1xPhospho [S402(100); T406(0)]	1.008	0.011495639	0.976789257
E9PCT1	3xPhospho [S781(100); S785(0); S787(100); S789(0); T790(0); S793(100)]	1.04	0.056583528	0.989838391
E9PCT1	2xPhospho [S572(100); S574(100)]	1.047	0.066261442	0.99184412
E9PCT1	1xPhospho [T220(100); S227(0)]	0.994	-0.008682243	0.999263007
E9PCT3	3xPhospho [S18(100); Y19(0); S20(100); S23(100); Y27(0)]	0.368	-1.442222329	0.033513483
E9PCT3	3xPhospho [S18(100); Y19(0); S20(100); S23(100); Y27(0)]	0.454	-1.139235797	0.244428417
E9PCT3	2xPhospho [S18(0); Y19(0); S20(100); S23(100); Y27(0)]	0.493	-1.020340448	0.414966031
E9PCT3	2xPhospho [S18(0); Y19(0); S20(100); S23(100); Y27(0)]	0.615	-0.701341684	0.568553006
E9PCV4	2xPhospho [S361(99.9); T364(0.1); T366(0); S376(100)]	0.01	-6.64385619	8.35E-17
E9PCY7	1xPhospho [T100(0); S104(100); T107(0)]	1.616	0.692427198	0.594518516
E9PCY7	1xPhospho [Y266(0); S269(0); S272(100)]	1.273	0.348232419	0.879063032
E9PD50	1xPhospho [S764(100); S769(0); Y774(0); S780(0)]	1.387	0.471967788	0.823333961
E9PD55	2xPhospho [S153(100); S157(100)]	0.886	-0.174621396	0.985461924
E9PDE8	2xPhospho [S553(100); S560(97.6); S561(2.4)]	0.01	-6.64385619	8.35E-17
E9PDE8	1xPhospho [S553(100); S560(0); S561(0)]	0.01	-6.64385619	8.35E-17
E9PDE8	1xPhospho [S48(100); T56(0)]	0.699	-0.516635639	0.951285684
E9PDL6	1xPhospho [T258(0); S260(0); S262(0); S263(99.9); T265(0.1); S266(0); T270(0)]	100	6.64385619	8.35E-17

E9PDL6	1xPhospho [S292(0); S294(0.2); T296(4.1); S297(4.1); T305(91.3); S308(0.2); S314(0)]	0.11	-3.184424571	0.003511194
E9PDL6	1xPhospho [S294(2.4); T296(97.5); S297(0.1); T305(0); S308(0); S314(0)]	0.662	-0.595096878	0.827205803
E9PDL6	2xPhospho [S256(0.1); T258(99.9); S260(0); S262(2.5); S263(97.5); T265(0); S266(0); T270(0)]	0.576	-0.795859283	0.896581478
E9PDL6	2xPhospho [S292(2.8); S294(97.2); T296(99.9); S297(0.1); T305(0); S308(0); S314(0)]	1.193	0.254594043	0.921238122
E9PDL6	2xPhospho [T258(0); S260(100); S262(0); S263(100); T265(0); S266(0); T270(0)]	0.889	-0.169744676	0.991003044
E9PEI0	1xPhospho [S962(100); S966(0); T967(0); T971(0)]	0.811	-0.30222618	0.980895643
E9PEL7	1xPhospho [S427(99.8); S428(0.2); T431(0)]	14.425	3.850499414	1.47E-05
E9PEL7	2xPhospho [T337(0); S353(100); S355(100)]	0.572	-0.805912948	0.44984964
E9PEL7	1xPhospho [S486(100)]	0.561	-0.833927324	0.848293321
E9PEL7	2xPhospho [Y26(0); S29(100); S31(100)]	0.766	-0.384583703	0.863867749
E9PEL7	1xPhospho [S399(0); S401(100); S406(0)]	0.604	-0.727379545	0.898105871
E9PEL7	1xPhospho [S382(0); S385(0); S393(100); T396(0)]	0.62	-0.689659879	0.906218259
E9PEL7	2xPhospho [Y26(0); S29(100); S31(100)]	1.032	0.045442971	1
E9PEW0	1xPhospho [T403(0); S413(100); S415(0); S416(0); T419(0)]	1.827	0.869476634	0.639406282
E9PEW0	2xPhospho [S367(100); S371(0); T373(0); T374(0); T375(0); S379(0.1); T380(0); S382(99.9)]	0.71	-0.49410907	0.879063032
E9PEW0	1xPhospho [T181(0); T182(0); S190(100)]	1.066	0.092207438	0.965351295
E9PEW9	2xPhospho [S188(100); S191(100); T195(0)]	100	6.64385619	8.35E-17
E9PEW9	2xPhospho [S540(100); S542(100); S551(0)]	1.186	0.24610401	0.922868578
E9PEW9	1xPhospho [S655(98.4); T656(1.6); S658(0)]	0.68	-0.556393349	0.939858086
E9PEW9	1xPhospho [S92(100); S95(0); S104(0)]	1.054	0.075874867	0.963305044
E9PFC1	2xPhospho [T461(50); T465(50); S466(0.1); T472(100); T478(0); T483(0)]	0.01	-6.64385619	8.35E-17
E9PFC1	2xPhospho [S916(100); S918(100); T928(0); S932(0)]	100	6.64385619	8.35E-17
E9PFC1	1xPhospho [T288(0); S290(100); S292(0)]	1.595	0.673556424	0.719352351
E9PFC1	2xPhospho [S267(2.7); T268(97.3); S270(100); T276(0)]	1.54	0.622930351	0.808799965
E9PFC1	2xPhospho [S860(0); S862(0); S871(0); T872(0); S881(100); S884(99.7); T888(0.3); S890(0); T891(0); S893(0)]	0.597	-0.744197163	0.862820654
E9PFC7	2xPhospho [S1211(0); S1212(0); S1217(100); S1218(100)]	0.01	-6.64385619	8.35E-17
E9PFC7	2xPhospho [S1211(0); S1212(0); S1217(100); S1218(100)]	0.01	-6.64385619	8.35E-17
E9PFC7	1xPhospho [Y1543(0); S1545(100)]	0.422	-1.244685096	0.660888692
E9PFC7	2xPhospho [S1328(0); S1330(0); S1336(100); S1342(100)]	0.506	-0.98279071	0.697933569
E9PFC7	3xPhospho [S1187(100); S1189(100); S1191(100); T1194(0); T1196(0); Y1202(0)]	0.65	-0.621488377	0.764462664
E9PFF5	3xPhospho [S414(0); S415(0); S417(0); S418(0); S425(2.5); Y428(2.5); S429(18.7); T434(87.1); S436(87.1); T439(89); T442(12.9); S445(0.3); S447(0); S450(0); T451(0)]	100	6.64385619	8.35E-17
E9PFF5	2xPhospho [S425(0); Y428(0); S429(0); T434(4.5); S436(95.5); T439(0); T442(0); S445(100); S447(0); S450(0); T451(0)]	100	6.64385619	8.35E-17
E9PFF5	3xPhospho [Y346(0); T347(0); S348(0); Y350(0); T352(100); S354(0); S357(100); S360(95.5); T362(4.3); S364(0.2)]	2.243	1.16542962	0.277764895
E9PFF5	2xPhospho [S414(0); S415(0); S417(0); S418(0); S425(0); Y428(0); S429(0); T434(99.7); S436(50.2); T439(50.2); T442(0); S445(0); S447(0); S450(0); T451(0)]	1.746	0.804053559	0.597773138
E9PFF5	2xPhospho [S371(100); S374(100); S383(0)]	1.625	0.700439718	0.614296568
E9PFF5	2xPhospho [Y346(0); T347(0); S348(0); Y350(0); T352(0); S354(0); S357(100); S360(97.3); T362(2.6); S364(0.1)]	1.558	0.639695233	0.714106081
E9PFF5	1xPhospho [S425(0); Y428(0); S429(0); T434(0); S436(100); T439(0); T442(0); S445(0); S447(0); S450(0); T451(0)]	1.474	0.559736524	0.757994886
E9PFF5	1xPhospho [Y346(0); T347(0); S348(0); Y350(0); T352(0); S354(0); S357(0); S360(100); T362(0); S364(0)]	1.485	0.570462931	0.775899177
E9PFF5	2xPhospho [Y346(0); T347(0); S348(0); Y350(0); T352(100); S354(0); S357(0); S360(100); T362(0.1); S364(0)]	1.321	0.401630467	0.870654365
E9PFI8	1xPhospho [Y600(5.7); S604(82.8); S608(5.7); S610(5.7); S617(0); S618(0); T622(0); T625(0)]	0.623	-0.682695932	0.797966154
E9PFQ8	2xPhospho [S21(98.3); S22(3.4); S24(98.3)]	1.698	0.763836459	0.62233267
E9PFQ8	1xPhospho [S21(0); S22(100); S24(0)]	1.431	0.517023672	0.794478982
E9PG73	2xPhospho [S341(100); T343(100)]	1.805	0.851998837	0.356884055
E9PG73	2xPhospho [S341(100); T343(100)]	1.82	0.86393845	0.401517235
E9PG73	3xPhospho [S239(0); S241(100); S242(100); S244(100); S255(0); T256(0)]	0.454	-1.139235797	0.690107651
E9PG73	1xPhospho [S371(0); S382(100)]	1.27	0.344828497	0.872730156
E9PG73	2xPhospho [S341(100); T343(100)]	0.928	-0.10780329	0.980895643
E9PG89	2xPhospho [S132(100); S138(100); Y143(0)]	0.786	-0.347398782	0.93864152
E9PG89	2xPhospho [T1654(0); S1660(100); S1664(100)]	0.949	-0.075520008	0.991765633
E9PG89	1xPhospho [S771(0); S772(100); T774(0); T778(0)]	0.877	-0.189351252	0.998271017
E9PGC8	3xPhospho [T1118(0); T1121(0); Y1126(0); S1129(0); T1132(0); S1134(99.9); T1136(66.7); S1137(66.7); S1138(66.7)]	100	6.64385619	8.35E-17
E9PGC8	2xPhospho [S764(100); S765(100); T770(0)]	100	6.64385619	8.35E-17
E9PGC8	2xPhospho [T790(0); T801(0); S808(0); T809(0); T814(100); S817(100)]	100	6.64385619	8.35E-17
E9PGC8	2xPhospho [T1118(0); T1121(0); Y1126(0); S1129(4.1); T1132(0.2); S1134(95.7); T1136(0.2); S1137(4.1); S1138(95.7)]	100	6.64385619	8.35E-17
E9PGC8	2xPhospho [S850(100); T854(100); T860(0)]	1.966	0.975263322	0.439163092
E9PGC8	2xPhospho [S764(100); S765(100); T770(0)]	1.944	0.959028219	0.560990292
E9PGC8	1xPhospho [S1410(100); T1415(0); Y1418(0)]	0.523	-0.935117148	0.797966154
E9PGC8	2xPhospho [S2029(0); S2035(100); S2039(100)]	1.187	0.247319935	0.922868578
E9PGM9	2xPhospho [T212(0); S221(0); S228(100); S230(100)]	0.644	-0.634867407	0.767755861
E9PGM9	1xPhospho [T212(0); S221(0); S228(0); S230(100)]	1.039	0.055195654	0.989838391
E9PGU7	2xPhospho [S226(100); S228(100); T234(0)]	2.116	1.081339627	0.406373833
E9PGU7	2xPhospho [S236(100); S239(99.9); S241(0.1); T245(0)]	0.679	-0.55851652	0.938730361
E9PGU7	3xPhospho [S236(100); S239(97.7); S241(2.3); T245(100)]	1.093	0.128293401	0.960298686

E9PGU7	2xPhospho [S226(100); S228(100); T234(0)]	0.988	-0.017417053	1
E9PGY0	2xPhospho [S554(96.3); S555(3.8); S560(3.6); S564(92.8); S565(3.6)]	10.406	3.379343707	0.001720523
E9PGY0	1xPhospho [S554(0.4); S555(0.4); S560(98.8); S564(0.4); S565(0)]	2.55	1.350497247	0.221449696
E9PGY0	1xPhospho [S554(0); S555(0); S560(100); S564(0); S565(0)]	1.49	0.575312331	0.831039794
E9PH18	1xPhospho [S162(100)]	1.926	0.945607703	0.450501922
E9PH69	2xPhospho [S832(0); T839(0); S846(100); S847(100); T853(0); S854(0); S855(0)]	0.787	-0.345564459	0.879063032
E9PH69	1xPhospho [S94(100)]	0.647	-0.628162383	0.886312012
E9PHH5	1xMet-loss+Acetyl [N-Term]	1.033	0.046840254	0.973263372
E9PHI6	2xPhospho [T392(0.1); S394(99.8); T396(0.1); T397(0); T399(0.1); S400(99.9); T402(0); S407(0)]	1.658	0.729444007	0.644556338
E9PHI6	1xPhospho [T392(0); S394(0); T396(0); T397(0); T399(0.1); S400(99.9); T402(0); S407(0)]	1.472	0.557777671	0.744877142
E9PHI6	1xPhospho [Y81(0); S91(100)]	0.906	-0.142417045	0.973763863
E9PHI6	1xPhospho [Y81(0); S91(100)]	1.065	0.09085343	0.975403456
E9PIE3	2xPhospho [S183(0); S197(100); S198(100); S205(0)]	0.813	-0.298672743	0.936043234
E9PIF8	2xPhospho [Y52(0); S56(100); S57(100)]	0.78	-0.358453971	0.87877743
E9PIM0	2xPhospho [S12(35.4); S14(35.4); S16(35.4); T18(79.7); S20(7.1); S22(7.1); T28(0); S30(0); S33(0); T36(0)]	100	6.64385619	8.35E-17
E9PIQ6	2xPhospho [S101(100); S107(0); S109(0); S110(0); S112(100); S114(0); T116(0); S118(0)]	100	6.64385619	8.35E-17
E9PIQ6	1xPhospho [S101(100); S107(0); S109(0); S110(0); S112(0); S114(0); T116(0); S118(0)]	1.189	0.249748715	0.936043234
E9PJ24	1xPhospho [T955(0); T957(0); T960(0); S969(100); T977(0)]	12.948	3.694657366	9.14E-06
E9PJ24	1xPhospho [S932(100); S944(0); S946(0); T947(0); S951(0)]	8.32	3.056583528	0.000119619
E9PJ24	2xPhospho [S426(0); S428(0); Y434(0); S441(99.8); S442(0.2); S446(0); S451(96); S454(4)]	9.378	3.229280279	0.000265975
E9PJ24	2xPhospho [S1355(0.1); S1356(99.9); S1367(100); S1369(0); S1371(0)]	1.669	0.738983955	0.569357532
E9PJ24	2xPhospho [S911(100); T913(100); T919(0); S921(0)]	1.502	0.586884813	0.764901318
E9PJ24	1xPhospho [S911(99.8); T913(0.2); T919(0); S921(0)]	1.386	0.470927257	0.797966154
E9PJ24	1xPhospho [S1198(100); T1214(0)]	1.41	0.495695163	0.813575855
E9PJ24	2xPhospho [S987(100); S989(100)]	1.352	0.435095152	0.855557911
E9PJ24	2xPhospho [S932(100); S944(100); S946(0); T947(0); S951(0)]	0.966	-0.049904906	0.99184412
E9PK47	1xPhospho [S430(100); S437(0)]	0.648	-0.625934282	0.908462992
E9PK52	2xPhospho [S38(0); S39(0); S47(0); S55(0.2); S57(99.8); S58(100)]	0.331	-1.595096878	0.048279389
E9PK52	1xPhospho [S87(98.1); Y88(0); T89(2)]	0.672	-0.573466862	0.904079048
E9PK52	1xPhospho [S38(0); S39(0); S47(0); S55(100); S57(0); S58(0)]	0.934	-0.098505545	0.975403456
E9PK91	2xPhospho [S300(100); T304(100)]; Q9NYF8-4 2xPhospho [S300(100); T304(100)]	100	6.64385619	8.35E-17
E9PK91	1xPhospho [S177(100)]; Q9NYF8-4 1xPhospho [S177(100)]	6.106	2.61022759	0.000261875
E9PK91	1xPhospho [S397(100); T402(0); T405(0); Y408(0)]	1.862	0.896853073	0.363377543
E9PK91	2xPhospho [S422(100); T425(0); S427(0.1); T431(99.9)]	1.882	0.912266628	0.574788308
E9PK91	3xPhospho [S272(0); S274(0); S276(0); S281(50); S282(50); Y284(0); S285(100); S287(0); S290(100); S297(0)]; Q9NYF8-4 3xPhospho [S272(0); S274(0); S276(0); S281(50); S282(50); Y284(0); S285(100); S287(0); S290(100); S297(0)]	1.601	0.678973308	0.598714672
E9PK91	1xPhospho [S319(100); S320(0); Y322(0); T330(0)]; Q9NYF8-4 1xPhospho [S319(100); S320(0); Y322(0); T330(0)]	1.731	0.791605725	0.624540466
E9PK91	2xPhospho [Y383(0); S385(100); S389(100)]	1.62	0.695993813	0.694198512
E9PK91	2xPhospho [S397(100); T402(100); T405(0); Y408(0)]	1.585	0.66448284	0.697129868
E9PK91	1xPhospho [S414(0); S422(100); T425(0); S427(0)]	1.563	0.644317778	0.718778149
E9PK91	3xPhospho [Y284(0); S285(100); S287(100); S290(100); S297(0)]; Q9NYF8-4 3xPhospho [Y284(0); S285(100); S287(100); S290(100); S297(0)]	0.594	-0.751465164	0.730212284
E9PK91	1xPhospho [Y383(0); S385(100); S389(0)]	1.507	0.591679417	0.755213977
E9PK91	2xPhospho [S272(0); S274(0); S276(0); S281(50.1); S282(50.1); Y284(0); S285(99.8); S287(0); S290(0); S297(0)]; Q9NYF8-4 2xPhospho [S272(0); S274(0); S276(0); S281(50.1); S282(50.1); Y284(0); S285(99.8); S287(0); S290(0); S297(0)]	1.533	0.616357697	0.774421636
E9PK91	1xPhospho [S397(100); T402(0); T405(0); Y408(0)]	1.377	0.461528559	0.782682971
E9PK91	2xPhospho [S183(100); T190(0); S196(50); S198(50)]; Q9NYF8-4 2xPhospho [S183(100); T190(0); S196(50); S198(50)]	0.644	-0.634867407	0.805601686
E9PK91	1xPhospho [S525(0); T526(0); S531(100)]; Q9NYF8-4 1xPhospho [S352(0); T353(0); S358(100)]	1.317	0.397255346	0.848293321
E9PK91	1xPhospho [T190(0); S196(1.1); S198(98.9)]; Q9NYF8-4 1xPhospho [T190(0); S196(1.1); S198(98.9)]	1.351	0.434027675	0.867705101
E9PK91	2xPhospho [Y383(0); S385(100); S389(100)]	1.244	0.314986485	0.872730156
E9PK91	2xPhospho [S397(100); T402(100); T405(0); Y408(0)]	1.319	0.399444565	0.879063032
E9PK91	1xPhospho [Y284(0); S285(0); S287(0); S290(100); S297(0)]; Q9NYF8-4 1xPhospho [Y284(0); S285(0); S287(0); S290(100); S297(0)]	1.28	0.35614381	0.880386458
E9PK91	2xPhospho [S181(0.2); S183(99.8); T190(0); S196(4.3); S198(95.7)]; Q9NYF8-4 2xPhospho [S181(0.2); S183(99.8); T190(0); S196(4.3); S198(95.7)]	0.67	-0.577766999	0.883306273
E9PK91	1xPhospho [S183(100); T190(0); S196(0); S198(0)]; Q9NYF8-4 1xPhospho [S183(100); T190(0); S196(0); S198(0)]	0.757	-0.401634795	0.892457589
E9PK91	1xPhospho [S205(0); S206(0); T208(0); S209(0); S217(0); Y219(0); S222(100)]; Q9NYF8-4 1xPhospho [S205(0); S206(0); T208(0); S209(0); S217(0); Y219(0); S222(100)]	0.633	-0.659722595	0.93019184
E9PK91	1xPhospho [Y383(0); S385(98); S389(2)]	1.148	0.199122642	0.933760225
E9PK91	1xPhospho [S177(100); S181(0)]; Q9NYF8-4 1xPhospho [S177(100); S181(0)]	1.227	0.295135249	0.936345162
E9PK91	2xPhospho [Y284(0.1); S285(99.9); S287(0); S290(100); S297(0)]; Q9NYF8-4 2xPhospho [Y284(0.1); S285(99.9); S287(0); S290(100); S297(0)]	1.154	0.206643224	0.939695119
E9PK91	2xPhospho [S414(100); S422(100); T425(0); S427(0)]	1.191	0.252173413	0.942907978
E9PK91	1xPhospho [Y511(0); S512(100)]	1.141	0.190298792	0.962617576
E9PK91	1xPhospho [Y511(0); S512(100)]	1.143	0.192825404	0.965139891

E9PK91	2xPhospho [S225(100); S228(100); S230(0); T234(0); S237(0); S239(0); S240(0); S241(0); S243(0); S249(0); T250(0); S253(0)]; Q9NYF8-4 2xPhospho [S225(100); S228(100); S230(0); T234(0); S237(0); S239(0); S240(0); S241(0); S243(0); S249(0); T250(0); S253(0)]	0.817	-0.291592017	0.969482098
E9PK91	2xPhospho [S525(100); T526(0); S531(100)]; Q9NYF8-4 2xPhospho [S352(100); T353(0); S358(100)]	0.883	-0.179514657	0.969680228
E9PK91	1xPhospho [S658(100); S660(0); T661(0)]; Q9NYF8-4 1xPhospho [S485(100); S487(0); T488(0)]	1.117	0.159629186	0.972363997
E9PK91	2xPhospho [Y284(0); S285(100); S287(0); S290(100); S297(0)]; Q9NYF8-4 2xPhospho [Y284(0); S285(100); S287(0); S290(100); S297(0)]	1.086	0.119024103	0.976868831
E9PK91	2xPhospho [T257(0); S259(0); S262(0); S264(0.1); S268(99.4); S272(82.3); S274(8.2); S276(8.2); S281(0.9); S282(0.9)]; Q9NYF8-4 2xPhospho [T257(0); S259(0); S262(0); S264(0.1); S268(99.4); S272(82.3); S274(8.2); S276(8.2); S281(0.9); S282(0.9)]	1.093	0.128293401	0.979498769
E9PK91	1xPhospho [S181(2.8); S183(97.3); T190(0); S196(0); S198(0)]; Q9NYF8-4 1xPhospho [S181(2.8); S183(97.3); T190(0); S196(0); S198(0)]	0.835	-0.260151897	0.980895643
E9PK91	2xPhospho [Y383(0); S385(100); S389(100)]	0.921	-0.118726939	0.989102507
E9PK91	1xPhospho [S658(100); S660(0); T661(0)]; Q9NYF8-4 1xPhospho [S485(100); S487(0); T488(0)]	1.036	0.051024003	0.990299313
E9PK91	2xPhospho [S414(100); S422(100); T425(0); S427(0)]	1.028	0.039840265	0.991765633
E9PKN0	1xPhospho [S705(100)]	1.331	0.412510571	0.872730156
E9PL71	1xPhospho [S40(96.2); S41(3.8); S46(0); S47(0); T49(0); S50(0)]	0.486	-1.040971781	0.701261726
E9PLD2	1xPhospho [S6(100)]	1.057	0.079975377	0.973763863
E9PLD2	1xPhospho [S140(0); S141(100)]	0.782	-0.354759487	0.977860291
E9PLE4	1xPhospho [S16(100)]	1.517	0.601221086	0.748211972
E9PLE4	1xPhospho [S16(100)]	1.282	0.358396262	0.909037613
E9PLL6	1xPhospho [S68(100); T72(0)]	0.456	-1.13289427	0.352372839
E9PLM7	2xPhospho [S322(100); S327(0); T329(0); S333(100)]	0.35	-1.514573173	0.306672643
E9PLM7	2xPhospho [T441(0); S446(0); T449(100); S452(50); S453(50)]	0.34	-1.556393349	0.44984964
E9PLM7	1xPhospho [S425(0); S429(100)]	0.899	-0.153606979	0.999714477
E9PLV2	2xPhospho [S94(0); S100(0.1); S101(99.9); S106(96.7); T108(3.3)]	0.603	-0.729770093	0.920518605
E9PLZ3	1xPhospho [T176(0); S178(100)]	1.32	0.40053793	0.878856551
E9PMQ6	1xPhospho [S238(100); S242(0)]	1.922	0.942608336	0.622135501
E9PMQ6	2xPhospho [T290(0); T292(0); S298(100); T302(33.3); S303(33.3); T304(33.3)]	0.661	-0.597277823	0.679191074
E9PMQ6	2xPhospho [S249(100); S254(50); S255(50); T258(0); S261(0); T263(0); S268(0)]	1.008	0.011495639	0.965013037
E9PMQ6	2xPhospho [S238(100); S242(100)]	1.019	0.027154052	0.998448514
E9PMR3	2xPhospho [T3(0); T6(0); S16(0); S26(100); S30(100)]	100	6.64385619	8.35E-17
E9PMR3	1xPhospho [S54(100); S61(0); S64(0)]	2.171	1.118359726	0.523663955
E9PMR3	2xPhospho [S54(100); S61(100); S64(0)]	1.335	0.416839742	0.854283637
E9PMR3	2xPhospho [S74(0); S76(0); T77(0); T81(0); S85(98.1); S87(1.9); S90(100)]	1.211	0.276198865	0.943512809
E9PMR3	2xPhospho [T3(0); T6(0); S16(0); S26(100); S30(100)]	1.174	0.231432408	0.94797789
E9PN10	1xPhospho [S36(100); T41(0); T44(0); T45(0); Y48(0); S49(0); S50(0)]	0.689	-0.537424112	0.862322824
E9PN10	1xPhospho [S36(100); T41(0); T44(0); T45(0); Y48(0); S49(0); S50(0)]	0.774	-0.369594529	0.904079048
E9PNH5	2xPhospho [Y100(0); S103(100); S107(100)]	1.414	0.49978212	0.848293321
E9PNJ4	1xPhospho [T331(0); S339(100)]	1.538	0.621055503	0.755716034
E9PNJ4	1xPhospho [S443(0); S445(100); S447(0); S448(0); T453(0); S455(0); S460(0)]	0.983	-0.024736678	0.97998947
E9PNJ7	1xMet-loss+Acetyl [N-Term]; 1xPhospho [S5(0); S7(0); S12(100); S14(0)]	1.56	0.641546029	0.694333278
E9PNJ7	1xMet-loss [N-Term]; 2xPhospho [S5(0); S7(100); S12(100); S14(0)]	1.236	0.305678743	0.879063032
E9PNJ7	1xPhospho [T111(0); T117(0.3); S121(5.1); S125(94.6)]	0.724	-0.465938398	0.938029982
E9PNJ7	1xPhospho [T111(0); T117(0); S121(0); S125(100)]	1.069	0.096261853	0.980895643
E9PNM5	2xPhospho [T44(0); S45(0.5); S47(0.5); S50(98.3); S52(0.5); Y57(92.9); Y59(0.5); Y61(6.6)]	100	6.64385619	8.35E-17
E9PNR6	2xPhospho [S44(0); S47(0); S49(1.8); S50(98.3); S51(100); T56(0)]	1.291	0.368489001	0.872730156
E9PNS0	1xPhospho [T37(0); S41(100)]	0.433	-1.20756107	0.66845931
E9PP21	1xPhospho [S159(100)]	0.749	-0.416962376	0.854283637
E9PP50	1xMet-loss+Acetyl [N-Term]; 1xPhospho [S3(100); S8(0)]	1.58	0.659924558	0.682758347
E9PPJ0	3xPhospho [S414(100); S418(100); S419(100)]	0.336	-1.573466862	0.015659436
E9PPJ0	3xPhospho [S285(50); S286(50); S290(100); S292(95.7); T294(4.4); T298(0); S300(0); S302(0)]	0.508	-0.977099598	0.790142755
E9PPJ0	2xPhospho [S414(0); S418(100); S419(100)]	0.613	-0.706041021	0.810156821
E9PPJ0	2xPhospho [S285(0); S286(0); S290(100); S292(100); T294(0); T298(0); S300(0); S302(0)]	0.798	-0.325539348	0.942907978
E9PPY3	2xPhospho [S51(0); S53(0); S58(0); S62(100); S64(100)]	0.434	-1.204233052	0.199385932
E9PPY3	2xPhospho [S162(0); T163(0); T164(0); S171(99.6); S174(33.5); T175(33.5); S176(33.5); T182(0); S184(0)]	0.565	-0.823677227	0.872730156
E9PQG5	2xPhospho [S28(97.6); T29(4.8); S30(97.6)]	2.085	1.060047384	0.210385549
E9PQP7	2xPhospho [T486(0); S490(99.9); S492(0.1); T495(95); S496(2.5); S497(2.5); S499(0.1); T500(0)]	1.743	0.801572569	0.563415239
E9PQP7	3xPhospho [T280(0); T285(0); T286(0); S291(100); S292(100); S293(100)]	0.548	-0.867752202	0.624566719
E9PQP7	1xPhospho [S619(97.6); S620(1.2); S621(1.2)]	1.71	0.773996325	0.670368081
E9PQP7	3xPhospho [T280(0); T285(0); T286(0); S291(100); S292(100); S293(100)]	0.63	-0.666576266	0.792141506
E9PQP7	2xPhospho [T280(0); T285(0); T286(0); S291(99.9); S292(97.1); S293(3)]	1.418	0.503857533	0.792141506
E9PQP7	1xPhospho [S347(100)]	1.179	0.237563718	0.925853562
E9PQP7	2xPhospho [T280(0); T285(0); T286(0); S291(95.7); S292(4.5); S293(99.8)]	1.042	0.059355278	0.971196575
E9PQP7	1xPhospho [S385(100)]	1.003	0.004321606	0.999714477
E9PQW4	2xPhospho [T198(0); T202(100); Y204(50); T207(50)]	1.417	0.502839758	0.860444802
E9PR34	1xPhospho [S98(0); S99(0); S100(100); T104(0); S105(0); S107(0)]	2.444	1.289244285	0.369703851
E9PRV2	1xPhospho [S61(100); T63(0)]	6.223	2.637610246	0.001165874
E9PRV2	2xPhospho [S127(49.9); S128(49.9); S131(0.2); S138(100); T141(0); T143(0); T145(0); S149(0); T154(0); T156(0)]	1.386	0.470927257	0.797966154
E9PRV2	1xPhospho [S251(100); S260(0); S262(0)]	0.693	-0.529072743	0.918281661
E9PRV2	1xPhospho [S296(0); T300(0); T307(0); S312(0); S313(100); S317(0)]	0.724	-0.465938398	0.941072235

E9PS50	1xPhospho [S12(100); S14(0); Y18(0)]	0.614	-0.703689439	0.658368583
E9PSH4	2xPhospho [S60(100); T64(0.1); S65(33.3); S68(33.3); S70(33.3)]	1.352	0.435095152	0.894595581
F2Z2K0	1xPhospho [S114(100)]	1.655	0.726831217	0.660888692
F2Z2K0	1xPhospho [S114(100)]	1.453	0.539034703	0.822469768
F2Z2K0	2xPhospho [Y95(0); S99(100); S102(100)]	1.206	0.270229907	0.93019184
F2Z2T1	1xPhospho [T21(0); S22(100); T27(0); S33(0)]	0.605	-0.724992953	0.660888692
F2Z2U8	3xPhospho [S2002(100); S2013(0); S2016(100); S2022(100)]	0.73	-0.454031631	0.943191333
F2Z357	2xPhospho [S515(100); T522(100); S525(0); S529(0)]	100	6.64385619	8.35E-17
F2Z357	1xPhospho [S498(97.1); S499(2.9)]	0.81	-0.304006187	0.980895643
F5GWS0	2xPhospho [S229(0); S235(0); S246(100); S253(46.8); S254(46.8); S256(3.1); S257(3.1); T258(0.2); S259(0); S260(0)]	0.01	-6.64385619	8.35E-17
F5GWS0	1xPhospho [S229(0); S235(0); S246(0); S253(2.6); S254(97.2); S256(0.1); S257(0.1); T258(0.1); S259(0); S260(0)]	0.01	-6.64385619	8.35E-17
F5GWS0	1xPhospho [S1136(100)]	1.493	0.578214165	0.772098477
F5GWS0	1xPhospho [S598(0); S600(100); S609(0)]	1.454	0.540027269	0.797966154
F5GWS0	1xPhospho [S246(0); S253(0); S254(100); S256(0); S257(0); T258(0); S259(0); S260(0)]	1.247	0.318461465	0.879063032
F5GWS0	1xPhospho [S1063(0); S1064(0); T1066(0); S1067(0); S1070(100); S1072(0); T1074(0)]	1.174	0.231432408	0.94797789
F5GWX5	3xPhospho [S1524(99.5); S1528(82.5); S1530(5.8); T1533(6.1); T1535(6.1); S1537(0.5); T1538(0.5); T1542(99); T1546(0)]; Q14839 3xPhospho [S1531(99.5); S1535(82.5); S1537(5.8); T1540(6.1); T1542(6.1); S1544(0.5); T1545(0.5); T1549(99); T1553(0)]	100	6.64385619	8.35E-17
F5GWX5	2xPhospho [S78(0); S79(0); S96(100); S98(99.1); S101(0.8); Y103(0); T104(0.1)]	100	6.64385619	8.35E-17
F5GWX5	1xPhospho [S1832(100)]; Q14839 1xPhospho [S1839(100)]	100	6.64385619	8.35E-17
F5GWX5	3xPhospho [S508(100); T510(0); T522(100); S524(100)]; Q14839 3xPhospho [S515(100); T517(0); T529(100); S531(100)]	2.465	1.301587647	0.129953013
F5GWX5	1xPhospho [S296(100)]; Q14839 1xPhospho [S303(100)]	3.3	1.722466024	0.160901297
F5GWX5	3xPhospho [S78(0); S79(0); S96(100); S98(100); S101(100); Y103(0); T104(0.1)]	1.596	0.674460652	0.781247135
F5GWX5	1xPhospho [S421(100)]; Q14839 1xPhospho [S428(100)]	1.348	0.430820497	0.854283637
F5GWX5	2xPhospho [S508(100); T510(0); T522(0.2); S524(99.8)]; Q14839 2xPhospho [S515(100); T517(0); T529(0.2); S531(99.8)]	1.301	0.379620962	0.872730156
F5GWX5	3xPhospho [S301(100); S302(100); S303(100); S312(0); S318(0); S321(0); Y322(0); S323(0); S325(0); S328(0); T329(0); S330(0)]; Q14839 3xPhospho [S308(100); S309(100); S310(100); S319(0); S325(0); S328(0); Y329(0); S330(0); S332(0); S335(0); T336(0); S337(0)]	0.888	-0.171368418	0.962617576
F5GWX5	3xPhospho [S78(0); S79(0); S96(99.9); S98(99.9); S101(98.8); Y103(0); T104(1.4)]	1.086	0.119024103	0.965795497
F5GWX5	2xPhospho [S1524(0); S1528(100); S1530(100); T1533(0); T1535(0); S1537(0); T1538(0); T1542(0); T1546(0)]; Q14839 2xPhospho [S1531(0); S1535(100); S1537(100); T1540(0); T1542(0); S1544(0); T1545(0); T1549(0); T1553(0)]	1.05	0.070389328	0.97726974
F5GWX5	3xPhospho [S78(0); S79(0); S96(100); S98(100); S101(100); Y103(0); T104(0)]	0.892	-0.164884385	0.97998947
F5GWX5	2xPhospho [S301(92.9); S302(3.5); S303(3.5); S312(100); S318(0); S321(0); Y322(0); S323(0); S325(0); S328(0); T329(0); S330(0)]; Q14839 2xPhospho [S308(92.9); S309(3.5); S310(3.5); S319(100); S325(0); S328(0); Y329(0); S330(0); S332(0); S335(0); T336(0); S337(0)]	0.871	-0.199255376	0.991003044
F5GX65	1xPhospho [S45(100); T50(0); S53(0); Y54(0)]	0.872	-0.19759996	0.97998947
F5GX74	1xPhospho [S681(100)]	3.63	1.859969548	0.048563317
F5GX74	2xPhospho [S121(100); T126(100); S131(0)]	2.214	1.146655222	0.425044655
F5GX74	1xPhospho [S121(100); T126(0); S131(0)]	1.457	0.543000877	0.830379242
F5GX74	1xPhospho [S681(100)]	1.16	0.214124805	0.930376591
F5GXF5	1xPhospho [S1188(100); S1204(0); S1206(0); S1208(0); S1209(0)]	0.543	-0.880975897	0.62233267
F5GXM1	2xPhospho [S200(100); S213(84.2); S216(7.9); S217(7.9)]	0.095	-3.395928676	1.38E-05
F5GXM1	1xPhospho [S200(100)]	1.333	0.41467678	0.862213304
F5GXM1	1xPhospho [S200(100)]	1.317	0.397255346	0.870180034
F5GXM1	2xPhospho [S228(100); S230(100)]	0.789	-0.341902795	0.880267055
F5GXM1	2xPhospho [S228(100); S230(100)]	0.832	-0.265344567	0.95754047
F5GXM1	1xPhospho [S200(100)]	1.142	0.191562651	0.96293109
F5GXM1	2xPhospho [S228(100); S230(100)]	1	0	0.967569514
F5GXM1	2xPhospho [S228(100); S230(100)]	0.983	-0.024736678	0.988508396
F5GXQ6	1xPhospho [S202(0); S208(0.1); S209(99.9); S212(0); T215(0); S216(0)]	1.454	0.540027269	0.822894134
F5GXQ6	2xPhospho [S202(0); S208(100); S209(100); S212(0); T215(0); S216(0)]	0.865	-0.209227962	0.999714477
F5GY18	1xPhospho [S161(0); S168(0); S172(5.7); S176(94.3)]	1.062	0.086783766	0.965139891
F5GY92	1xPhospho [S329(100); S335(0); S341(0); S343(0); T344(0)]	2.089	1.062812492	0.578933372
F5GYB0	2xPhospho [S128(100); S129(100); S134(0)]	1.243	0.313826296	0.933760225
F5GYB1	2xPhospho [S130(0); S132(100); S137(0); S140(100)]	0.661	-0.597277823	0.868254505
F5GZ78	1xPhospho [T316(0); S318(0); S319(0); S320(0); S330(0); S334(0); S338(100); S341(0)]	1.611	0.687956494	0.58296191
F5GZ78	2xPhospho [S124(100); S128(100); T130(0); S133(0); T134(0); S135(0); S138(0); S141(0)]	0.632	-0.662003536	0.77127773
F5GZ78	3xPhospho [S124(99.9); S128(50.1); T130(50.1); S133(0); T134(0); S135(99.7); S138(0.3); S141(0)]	0.54	-0.888968688	0.860444802
F5GZ78	1xPhospho [T316(0.1); S318(2.8); S319(94.4); S320(2.8); S330(0); S334(0); S338(0); S341(0)]	1.417	0.502839758	0.862999305
F5GZ78	2xPhospho [S124(100); S128(100); T130(0); S133(0); T134(0); S135(0); S138(0); S141(0)]	0.689	-0.537424112	0.962617576
F5GZ78	1xPhospho [S81(100); S82(0); S83(0); Y86(0); S88(0); S89(0)]	1.05	0.070389328	0.973599548
F5GZ78	2xPhospho [S124(49.9); S128(49.9); T130(81.6); S133(8.7); T134(8.7); S135(1); S138(0.1); S141(0.1)]	1	0	0.97726974
F5GZ78	2xPhospho [T316(0); S318(3.4); S319(93.3); S320(3.4); S330(0); S334(0); S338(100); S341(0)]	0.89	-0.168122759	0.981858798
F5GZ78	2xPhospho [T316(0.1); S318(3.4); S319(93); S320(3.4); S330(0); S334(0); S338(100); S341(0)]	1.036	0.051024003	0.990299313
F5GZ78	1xPhospho [T92(0); S93(0); S94(0); S96(0); S101(0); S104(99.9); S107(0.1)]	0.915	-0.128156351	0.99728556
F5GZB1	1xMet-loss+Acetyl [N-Term]; 1xPhospho [S2(1.9); T3(98.1)]	1.115	0.15704371	0.938029982
F5H0Q6	1xPhospho [S285(100); S288(0); Y289(0)]	1.348	0.430820497	0.85961296
F5H0Q6	1xPhospho [S285(100); S288(0); Y289(0)]	0.846	-0.241270432	0.989554868

F5H0X3	3xPhospho [T482(0); S483(0); S484(0); S487(100); S489(100); S492(100)]	0.01	-6.64385619	8.35E-17
F5H0X3	1xPhospho [S695(100); S703(0)]	2.846	1.508935662	0.107357371
F5H0X3	2xPhospho [S459(100); S462(0); T463(0); S465(0); S466(0); T469(0.1); S471(100)]	1.667	0.737254104	0.673203213
F5H0X3	2xPhospho [T723(0); S725(0); T727(0); S734(100); S736(100); S738(0); S749(0)]	1.334	0.415758667	0.853287961
F5H0X3	2xPhospho [S51(100); S55(0); S56(0); S60(0); T64(0.3); S65(4.9); T66(4.9); S67(90)]	1.31	0.389566812	0.897746962
F5H0X3	2xPhospho [T533(1); T536(26); S537(26); T538(26); T539(26); S541(94.9)]	1.184	0.243669081	0.908086456
F5H0X3	2xPhospho [T773(100); S776(0); T777(0); T778(0); S781(0); S783(99.8); T787(0.2); T791(0)]	1.158	0.211635253	0.96293109
F5H0X3	2xPhospho [T508(0); S513(100); S515(0.2); S517(99.8); S522(0); S526(0); T530(0); T531(0)]	0.835	-0.260151897	0.966604693
F5H0X3	1xPhospho [T508(0); S513(0); S515(2.1); S517(97.9); S522(0); S526(0); T530(0); T531(0)]	0.989	-0.015957574	0.97998947
F5H0X3	1xPhospho [T536(0); S537(0); T538(0); T539(0); S541(100)]	0.796	-0.329159664	0.981858798
F5H0X3	3xPhospho [T508(0); S513(100); S515(0); S517(100); S522(100); S526(0); T530(0); T531(0)]	0.869	-0.202571918	0.983133428
F5H0X3	2xPhospho [S459(100); S462(0); T463(0); S465(0); S466(0); T469(0); S471(100)]	0.905	-0.144010303	0.986075329
F5H0X3	1xPhospho [S459(100); S462(0); T463(0); S465(0); S466(0); T469(0); S471(0)]	0.837	-0.256700472	0.986276919
F5H0X3	2xPhospho [T773(100); S776(0); T777(0); T778(0); S781(0); S783(100); T787(0); T791(0)]	1.008	0.011495639	0.986736984
F5H0X3	3xPhospho [S51(100); S55(3.6); S56(3.6); S60(92.9); T64(3.4); S65(3.4); T66(3.4); S67(89.7)]	0.975	-0.036525876	0.99184412
F5H0Y0	1xPhospho [S304(0); S308(100); T313(0); S315(0)]	0.438	-1.190997225	0.513166947
F5H0Y0	1xPhospho [S457(100); T470(0)]	0.777	-0.364013496	0.980895643
F5H1K3	1xPhospho [S86(0); T93(0); S97(0.1); S98(99.9)]	0.856	-0.224317298	0.989898705
F5H1M1	1xPhospho [S262(100); S275(0); S276(0); T277(0); S279(0)]	1.265	0.339137385	0.921247725
F5H1N7	2xPhospho [T961(0.2); S962(4.1); T964(95.7); S968(100); S971(0)]	0.323	-1.63039393	0.216045078
F5H1N7	2xPhospho [S1062(100); S1065(99.9); S1069(0.1)]	0.698	-0.518701058	0.965013037
F5H1N7	1xPhospho [S921(0); S933(3); S934(96.9); T936(0.1)]	1.015	0.021479727	0.973263372
F5H1N7	1xPhospho [S1035(100); T1041(0); S1047(0); T1049(0)]	0.861	-0.215914857	0.990375321
F5H1U2	2xPhospho [S90(0); S94(100); S95(100); S101(0)]	0.517	-0.951763814	0.440397264
F5H1U2	2xPhospho [S90(0); S94(100); S95(100); S101(0)]	0.467	-1.098505545	0.45570878
F5H1X8	1xPhospho [S1231(100); S1233(0); S1236(0); S1237(0)]	2.012	1.008630305	0.560990292
F5H1X8	1xMet-loss+Acetyl [N-Term]; 1xPhospho [S3(0); S10(100); T14(0)]	0.876	-0.190997225	0.99184412
F5H1X8	1xMet-loss+Acetyl [N-Term]; 1xPhospho [S3(6.4); S10(93.6); T14(0); T26(0); T28(0); S34(0)]	0.904	-0.145605322	1
F5H2M7	1xPhospho [S167(0); S170(100); S174(0)]	100	6.64385619	8.35E-17
F5H2M7	1xPhospho [S2525(0); S2527(0); S2530(100); S2535(0); S2536(0)]	2.657	1.409798228	0.11825442
F5H2M7	2xPhospho [S2500(0); S2503(0); S2509(100); S2510(100); S2518(0)]	0.42	-1.251538767	0.229013916
F5H2M7	2xPhospho [S2500(0); S2503(0); S2509(100); S2510(100); S2518(0)]	0.587	-0.768567592	0.667231556
F5H2M7	1xPhospho [S2500(0); S2503(0); S2509(2.6); S2510(97.4); S2518(0)]	1.439	0.525066592	0.768341527
F5H2M7	2xPhospho [S167(100); S170(0); S174(100)]	0.647	-0.628162383	0.94797789
F5H2M7	1xPhospho [S2476(100)]	0.762	-0.392137097	0.97998947
F5H2R7	2xPhospho [S418(100); S422(100); T424(0)]	0.815	-0.295128036	0.990182098
F5H2T0	3xPhospho [S814(0.2); S818(33.4); T820(33.3); S821(33.5); S822(99.6); S825(100); S827(0); S830(0)]	0.287	-1.800877358	0.301237915
F5H2T0	2xPhospho [S814(0); S818(0); T820(0); S821(0.3); S822(99.7); S825(99.7); S827(0.3); S830(0)]	1.323	0.403813062	0.851597639
F5H2X7	1xPhospho [S385(100)]	1.106	0.145351386	0.938029982
F5H385	2xPhospho [S114(0); T120(0); S125(100); S129(100); S131(0); Y133(0); S135(0)]	1.556	0.63784206	0.733427607
F5H385	2xPhospho [S114(0); T120(0); S125(100); S129(96.3); S131(3.7); Y133(0); S135(0)]	0.953	-0.069451881	0.984718011
F5H3F1	2xPhospho [S52(100); Y55(100)]	0.792	-0.336427665	0.938673385
F5H3F1	1xPhospho [S52(100); Y55(0)]	1.184	0.243669081	0.942574721
F5H3J2	2xPhospho [T413(100); S416(100); T426(0); S433(0)]	0.435	-1.200912694	0.67671989
F5H3J2	1xPhospho [S681(0); S687(100); S692(0); S705(0)]; Q5T624 1xPhospho [S379(0); S385(100); S390(0); S403(0)]	1.413	0.498761466	0.817964976
F5H3J2	1xPhospho [T609(0); S613(0); S614(0); T615(0); S616(0); T619(0); S625(0); S626(100); S628(0); S632(0)]; Q5T624 1xPhospho [T307(0); S311(0); S312(0); T313(0); S314(0); T317(0); S323(0); S324(100); S326(0); S330(0)]	1.132	0.178873958	0.965013037
F5H3J2	1xPhospho [T609(0); S613(0); S614(0); T615(0); S616(0); T619(0); S625(96.8); S626(3.2); S628(0); S632(0)]; Q5T624 1xPhospho [T307(0); S311(0); S312(0); T313(0); S314(0); T317(0); S323(96.8); S324(3.2); S326(0); S330(0)]	0.982	-0.02620507	0.973263372
F5H3J2	2xPhospho [T609(0); S613(0); S614(0); T615(0); S616(0); T619(0); S625(99.9); S626(0.2); S628(99.9); S632(0)]; Q5T624 2xPhospho [T307(0); S311(0); S312(0); T313(0); S314(0); T317(0); S323(99.9); S324(0.2); S326(99.9); S330(0)]	1.043	0.060739158	0.980895643
F5H3J2	2xPhospho [T609(0); S613(0); S614(0); T615(0); S616(0); T619(0); S625(97.5); S626(2.5); S628(100); S632(0)]; Q5T624 2xPhospho [T307(0); S311(0); S312(0); T313(0); S314(0); T317(0); S323(97.5); S324(2.5); S326(100); S330(0)]	1.061	0.085424656	0.980895643
F5H420	1xPhospho [S154(0); T155(0); T157(0); S169(100)]	100	6.64385619	8.35E-17
F5H4X1	2xPhospho [S927(100); S937(100)]	0.408	-1.293358943	0.128799151
F5H4X1	2xPhospho [S927(100); S937(100)]	0.548	-0.867752202	0.563415239
F5H4X1	1xPhospho [S927(100); S937(0)]	0.65	-0.621488377	0.743965448
F5H4X1	1xPhospho [S927(100); S937(0)]	0.606	-0.722610301	0.797966154
F5H4X1	1xPhospho [S927(100); S937(0)]	1.123	0.167357928	0.93019184
F5H4X1	1xPhospho [S927(100); S937(0)]	0.889	-0.169744676	0.973599548
F5H4X1	1xPhospho [S702(99.9); Y703(0); S704(0.1)]	0.822	-0.282789701	0.973763863
F5H538	3xPhospho [S63(0.2); S66(99.8); S72(100); T75(0); T77(0); Y81(0); T83(0); S84(100); S87(0); T88(0)]	0.372	-1.426625474	0.458550853
F5H553	1xPhospho [S123(100)]	0.758	-0.399730246	0.978952227
F5H578	1xPhospho [S15(0); S19(0); S23(100)]	0.958	-0.061902439	0.980895643
F5H5D3	1xPhospho [Y502(0); S509(100); Y519(0)]	0.785	-0.349235441	0.963156927
F5H5D3	1xPhospho [T111(0); S118(100); T121(0); S124(0); T126(0)]; P68363 1xPhospho [T41(0); S48(100); T51(0); S54(0); T56(0)]	0.979	-0.030619235	0.998271017

F5H5W4	1xPhospho [T439(3.8); S441(96.2)]	1.045	0.063502942	0.965013037
F5H5Y2	1xPhospho [T218(0); S219(0); S220(0); S227(0); T233(0); S241(100)]	0.407	-1.2968993	0.156977549
F5H604	2xPhospho [S556(0.3); S557(0.3); S558(4.6); S559(94.9); S560(100); S563(0); S569(0); S570(0)]	0.01	-6.64385619	8.35E-17
F5H604	1xPhospho [S601(0); S603(100)]	1.297	0.37517848	0.879063032
F5H604	2xPhospho [S756(0); S758(100); S762(100)]	1.258	0.331131922	0.900153261
F5H6Q1	1xPhospho [S16(2.9); S18(97.1); Y26(0)]	1	0	0.980895643
F5H721	3xPhospho [S353(100); S361(100); S364(100); S370(0)]	0.01	-6.64385619	8.35E-17
F5H721	2xPhospho [S353(100); S361(100); S364(0)]	100	6.64385619	8.35E-17
F5H721	3xPhospho [S353(100); S361(100); S364(100)]	0.436	-1.19759996	0.424797925
F5H721	1xPhospho [Y236(0); S237(100)]	1.5	0.584962501	0.744877142
F5H721	3xPhospho [S353(100); S361(100); S364(100); S370(0)]	0.468	-1.095419565	0.750945933
F5H721	1xPhospho [Y236(0); S237(100)]	1.144	0.194087052	0.94797789
F5H777	2xPhospho [S219(50); S220(50); T227(100)]	0.105	-3.251538767	0.006721728
F5H7J5	1xPhospho [S719(100); S723(0); S724(0); T725(0); S726(0); S727(0); S728(0); S729(0); T731(0)]	0.528	-0.921390165	0.494577496
F5H7J5	2xPhospho [S719(100); S723(0); S724(0); T725(0.1); S726(0.1); S727(2.6); S728(2.6); S729(94.6); T731(0.1)]	0.694	-0.526992432	0.810156821
F5H7J5	2xPhospho [T695(0); S696(0); S697(0); T699(0); S701(100); S705(100); T707(0)]	0.843	-0.246395464	0.933760225
F5H7J5	1xPhospho [T695(0); S696(0); S697(0); T699(0); S701(0); S705(98.6); T707(1.4)]	0.811	-0.30222618	0.964908419
F5H7T8	2xPhospho [S333(100); S337(100); S340(0); T343(0); T344(0)]	0.11	-3.184424571	0.002670098
F5H865	1xPhospho [S571(100); T577(0); S579(0); S581(0); T587(0)]	3.477	1.797843067	0.044699055
F5H865	2xPhospho [T319(0); Y321(0); Y323(0); S324(100); S326(100); S335(0); S339(0); S340(0); S348(0); T349(0)]	2.84	1.50589093	0.053683207
F5H865	2xPhospho [S672(100); S678(100)]	2.494	1.318461465	0.199739264
F5H865	1xPhospho [S611(100); T614(0); S615(0); T622(0)]	1.903	0.928275562	0.499482643
F5H865	1xPhospho [S737(0); S740(100)]	1.697	0.762986565	0.645378063
F5H865	1xPhospho [S539(0); S541(100); S543(0); S545(0)]	1.47	0.555816155	0.774421636
F5H865	2xPhospho [S735(2.1); S737(98); S740(100)]	1.553	0.63505783	0.788815962
F5H865	1xPhospho [S672(0); S678(100)]	1.346	0.42867841	0.855534442
F5H865	1xPhospho [T552(100)]	1.366	0.449957484	0.855557911
F5H865	2xPhospho [T844(0); S849(0); Y853(0); T860(0); T865(88.4); S866(5.8); S870(5.8); S872(0); S874(0.4); S876(99.6)]	0.857	-0.222632891	0.964978421
F5H865	2xPhospho [S539(4.5); S541(95.5); S543(0); S545(0); T552(100)]	1.11	0.150559677	0.965013037
F5H865	2xPhospho [S735(100); S737(98.2); S740(1.9)]	1.109	0.149259365	0.971937853
F5H865	2xPhospho [T647(0); T648(0); S649(100); S651(100)]	1.048	0.067638717	0.980895643
F6QR24	1xPhospho [T202(0); S203(100); S209(0)]	100	6.64385619	8.35E-17
F6QR24	2xPhospho [S330(0); S333(0); S334(100); S338(100)]	0.422	-1.244685096	0.651510528
F6QR24	2xPhospho [S330(0); S333(2.3); S334(97.7); S338(100)]	0.5	-1	0.787236772
F6QR24	1xPhospho [S1487(0); S1488(0); S1489(0); T1491(0); S1492(100); S1494(0)]	1.021	0.029982866	0.973599548
F6RY50	1xPhospho [S53(0); S55(100); T64(0); S67(0)]	100	6.64385619	8.35E-17
F6RY50	2xPhospho [S53(3.1); S55(96.9); T64(0); S67(100)]	0.08	-3.64385619	6.44E-05
F6TFQ5	1xPhospho [S126(0); S128(0); S134(0); S138(0.1); T141(99.9)]	0.851	-0.232768963	0.988508396
F6UYM1	2xPhospho [T421(0); S424(100); S429(100)]	0.794	-0.332789088	0.962617576
F6UYM1	3xPhospho [S418(100); T421(100); S424(100)]	0.821	-0.284545873	0.965139891
F6UYM1	1xPhospho [S404(100)]	0.859	-0.219269964	0.99184412
F6XS94	1xPhospho [S2094(100)]	1.034	0.048236186	0.9950833
F8VQ10	2xPhospho [S38(100); Y39(0); S41(100); S44(0); S45(0)]	1.155	0.207892852	0.938730361
F8VQ10	1xPhospho [S38(100); Y39(0); S41(0); S44(0); S45(0)]	1.17	0.22650853	0.949541112
F8VQP2	1xPhospho [T616(0); S617(0); T622(0); S623(0); S624(100)]	100	6.64385619	8.35E-17
F8VQP2	2xPhospho [S578(0); S583(0); S584(0); S585(0); T588(0); S589(0); S591(0); S592(0); S596(100); S598(0); S600(99.8); S602(0.2); S605(0); T607(0)]	0.756	-0.40354186	0.915837201
F8VQP2	1xPhospho [S544(0); S545(0); T546(0); S547(0); S549(100)]	1.255	0.327687364	0.916167518
F8VQP2	1xPhospho [S166(100)]	1.2	0.263034406	0.928912804
F8VQP2	3xPhospho [S578(0); S583(0); S584(0); S585(0); T588(0); S589(0); S591(5.1); S592(94.9); S596(100); S598(0); S600(99.7); S602(0); S605(0.3); T607(0)]	0.836	-0.258425153	0.930376591
F8VQR7	1xPhospho [S94(100); T101(0); T102(0); T106(0); S107(0)]	2.231	1.157690514	0.405723512
F8VW32	1xPhospho [S51(0); S61(100); S65(0)]	0.89	-0.168122759	0.989838391
F8VW92	1xPhospho [S25(0); T33(0); T35(0); Y36(0); S40(100)]	100	6.64385619	8.35E-17
F8VW92	1xPhospho [S78(0); S95(100)]; Q5JP53 1xPhospho [S60(0); S77(100)]	0.879	-0.18606493	0.97998947
F8VWC0	1xPhospho [Y94(0); T95(0); S103(100); S105(0); T110(0); T115(0); S116(0)]	0.841	-0.249822294	0.999263007
F8VXY0	1xPhospho [S4(0); S6(100)]; P09651 1xPhospho [S4(0); S6(100)]	1.383	0.467801156	0.803642804
F8VXY0	1xPhospho [S4(0); S6(100)]; P09651 1xPhospho [S4(0); S6(100)]	1.247	0.318461465	0.879063032
F8VXY0	1xPhospho [S95(100); T103(0)]; P09651 1xPhospho [S95(100); T103(0)]	1.215	0.280956314	0.942574721
F8VXY0	1xMet-loss+Acetyl [N-Term]; 1xPhospho [S2(0); S4(100); S6(0)]; P09651 1xMet-loss+Acetyl [N-Term]; 1xPhospho [S2(0); S4(100); S6(0)]	1.136	0.183962835	0.964978421
F8VXY0	1xMet-loss [N-Term]; 1xPhospho [S2(2.1); S4(97.9); S6(0)]; P09651 1xMet-loss [N-Term]; 1xPhospho [S2(2.1); S4(97.9); S6(0)]	1.147	0.197865391	0.976737179
F8VXY0	1xPhospho [S269(98.7); S270(1.3); Y273(0); Y279(0)]; P09651 1xPhospho [S337(98.7); S338(1.3); Y341(0); Y347(0)]	1.057	0.079975377	0.981858798
F8VYL4	2xPhospho [S11(10.5); S13(94.8); T15(94.8); S20(0); S28(0); S29(0); S30(0); S33(0)]	100	6.64385619	8.35E-17
F8VYL4	3xPhospho [S11(100); S13(100); T15(100); S20(0); S28(0); S29(0); S30(0); S33(0)]	0.633	-0.659722595	0.824557245
F8VYL4	3xPhospho [S11(100); S13(100); T15(100); S20(0); S28(0); S29(0); S30(0); S33(0)]	1.27	0.344828497	0.868254505
F8VZJ2	1xPhospho [S72(0); T78(0); T80(0); T82(0); S87(100); T95(0)]	1.244	0.314986485	0.908086456
F8VZJ2	2xPhospho [S72(100); T78(0); T80(0); T82(0.3); S87(99.8); T95(0)]	0.969	-0.045431429	0.99184412
F8W036	1xPhospho [S14(4.6); S22(95.4); T28(0); S31(0)]	1.174	0.231432408	0.922170166

F8W6L6	2xPhospho [S1968(100); S1972(100); T1976(0); S1978(0)]	0.428	-1.224317298	0.310811232
F8W6L6	2xPhospho [S1968(100); S1972(99.9); T1976(0.1); S1978(0)]	0.62	-0.689659879	0.920957334
F8W6L6	1xPhospho [S1968(0); S1972(100); T1976(0); S1978(0)]	1.051	0.071762669	0.973763863
F8W6N3	1xPhospho [Y376(0); S377(100); Y383(0); T394(0); S396(0)]	0.01	-6.64385619	8.35E-17
F8W6N3	2xPhospho [S564(4.6); S565(90.8); S567(4.6); S574(0); S577(0); S578(4.9); S579(95.1)]	0.01	-6.64385619	8.35E-17
F8W6N3	1xPhospho [S287(0); T292(0); S301(0); S307(2.9); S309(97.1)]	0.632	-0.662003536	0.85992414
F8W714	3xPhospho [S84(100); S85(100); S87(100)]	0.62	-0.689659879	0.910211107
F8W7D6	2xPhospho [S157(100); S163(100); T167(0); T168(0); S169(0)]	0.302	-1.727379545	0.04860251
F8W7D6	2xPhospho [S157(100); S163(100); T167(0); T168(0); S169(0)]	0.478	-1.064917477	0.236169458
F8W7D6	3xPhospho [S157(100); S163(100); T167(0.2); T168(0.2); S169(99.7)]	1.659	0.730313886	0.551311165
F8W7D6	3xPhospho [S157(100); S163(100); T167(87.2); T168(6.4); S169(6.4); T174(0)]	1.061	0.085424656	0.971220368
F8W7D6	3xPhospho [S157(100); S163(100); T167(1.7); T168(49.2); S169(49.2)]	1.022	0.031395196	0.99319806
F8W840	1xPhospho [S291(0); S296(3.6); S298(3.6); S300(92.9); S308(0); S311(0); S314(0)]	0.947	-0.078563669	0.981858798
F8W860	2xPhospho [S179(100); T185(50); T187(50)]	0.697	-0.520769439	0.96293109
F8W860	1xPhospho [S96(100); T106(0)]	1.019	0.027154052	0.97998947
F8W878	1xPhospho [S374(2.9); S375(2.9); S376(91.4); T377(2.9); Y393(0); S397(0)]	0.807	-0.309359421	0.918006975
F8W878	1xPhospho [S256(0); S258(0); Y259(0); S260(0); T262(100)]	0.811	-0.30222618	0.974924677
F8W878	1xPhospho [S274(0); Y275(0); T277(0); T278(0); T282(100)]	0.765	-0.386468347	0.977860291
F8W8D3	1xPhospho [S59(100); T61(0); T62(0)]	0.747	-0.420819852	0.908462992
F8W8P5	2xPhospho [S56(100); S58(100); S60(0); S62(0); S65(0)]	0.693	-0.529072743	0.922251444
F8W8S3	2xPhospho [Y238(0); S240(100); S245(100); S253(0); T259(0)]	0.325	-1.621488377	0.124031046
F8W9A9	1xPhospho [S336(100)]	1.2	0.263034406	0.916033193
F8W9J4	2xPhospho [S7401(100); S7404(100); S7409(0); S7413(0); S7416(0); T7420(0); T7424(0); T7428(0)]	0.633	-0.659722595	0.707838706
F8W9T0	1xPhospho [S332(100); T333(0); S335(0); S338(0); T344(0)]	0.63	-0.666576266	0.851160627
F8W9T0	1xPhospho [S332(98.7); T333(1.3); S335(0); S338(0); T344(0)]	0.733	-0.448114897	0.904774194
F8WA86	1xPhospho [Y274(0); Y276(0); S277(100); Y283(0)]	0.611	-0.710755715	0.925853562
F8WA86	1xPhospho [T149(0); T156(0); T157(6.4); S159(93.1); T164(0.5)]	0.794	-0.332789088	0.983191868
F8WAK8	2xPhospho [S978(0); T987(0); S989(0); S992(100); S995(50); S996(50)]	0.603	-0.729770093	0.815248216
F8WAK8	1xPhospho [S978(0); T987(0); S989(100); S992(0); S995(0); S996(0)]	0.789	-0.341902795	0.980895643
F8WAN1	1xPhospho [S220(100)]	100	6.64385619	8.35E-17
F8WAN1	1xPhospho [S384(100); S385(0); S389(0); S392(0); T397(0)]	0.815	-0.295128036	0.969482098
F8WBF5	2xPhospho [S328(0); T330(0); Y331(0); S337(9.7); T338(9.7); S341(90.3); T342(90.3); Y345(0)]; Q9HAZ1 2xPhospho [S326(0); T328(0); Y329(0); S335(9.7); T336(9.7); S339(90.3); T340(90.3); Y343(0)]	5.73	2.518535139	0.001972751
F8WBF5	1xPhospho [S140(100); S153(0); S158(0)]	1.608	0.685267407	0.677251709
F8WBF5	2xPhospho [T138(100); S140(100); S153(0); S158(0)]	1.478	0.563646269	0.763548117
F8WBF5	2xPhospho [S328(0); T330(0); Y331(0); S337(0); T338(0); S341(100); T342(100)]; Q9HAZ1 2xPhospho [S326(0); T328(0); Y329(0); S335(0); T336(0); S339(100); T340(100)]	1.421	0.506906555	0.828459192
F8WBK2	1xPhospho [T296(2.1); S298(97.9); T302(0); S306(0)]	1.019	0.027154052	0.979498769
F8WC70	1xMet-loss+Acetyl [N-Term]; 1xPhospho [S2(100); S7(0)]	0.355	-1.49410907	0.436105186
F8WCL7	2xPhospho [S84(99.1); T87(99.1); T89(1.8); S97(0); S99(0); Y119(0)]	100	6.64385619	8.35E-17
F8WCL7	1xPhospho [T60(0); S63(100)]	1.184	0.243669081	0.93019184
F8WCX2	2xPhospho [S225(95); S227(5); S239(5); S241(0.3); S242(94.7)]	0.746	-0.422752464	0.921238122
F8WCX2	1xPhospho [T246(0); S251(0); S253(100)]	1.074	0.102993993	0.96293109
F8WEE8	1xPhospho [S19(100); S31(0)]	1.294	0.371837617	0.87431027
F8WEE8	1xPhospho [S17(1.9); S19(98.2); S31(0)]	0.833	-0.263611599	0.96293109
F8WEE8	2xPhospho [S17(100); S19(100); S31(0)]	0.944	-0.083141235	0.97998947
F8WF16	1xPhospho [S111(0); S113(100); Y114(0); S115(0); S119(0)]	3.161	1.660381035	0.332208392
F8WF16	1xPhospho [T80(1.9); S85(98.1)]	1.64	0.713695815	0.630710862
F8WF16	1xPhospho [S113(0); Y114(0); S115(0); S119(100)]	1.239	0.309176187	0.884652412
F8WF16	1xPhospho [T80(0); S85(100)]	1.199	0.261831659	0.920957334
G3V0I6	1xPhospho [S531(0); T532(0); T537(0); Y541(0); T543(0); S545(3.2); S546(93.7); S548(3.2)]	0.01	-6.64385619	8.35E-17
G3V0I6	2xPhospho [S1023(100); S1024(100); S1031(0)]	0.52	-0.943416472	0.498895167
G3V0I6	1xPhospho [T414(0); S416(100)]	1.644	0.717210299	0.701118528
G3V0I6	2xPhospho [S1023(100); S1024(100); S1031(0)]	0.781	-0.356605547	0.908086456
G3V159	2xPhospho [S347(100); S349(100); S353(0); S355(0); S358(0); T360(0); S362(0)]	0.589	-0.763660461	0.742967319
G3V1K1	1xPhospho [Y49(0); S50(100); Y53(0); S56(0); S58(0)]	1.14	0.189033824	0.947600551
G3V1K6	2xPhospho [S5(0); S6(0); T9(0.1); S10(1); S14(99.5); S15(99.5); T25(0); S30(0); S33(0); S35(0)]	7.117	2.831269236	0.000332545
G3V1R4	2xPhospho [S507(100); S515(100); T519(0)]	0.764	-0.388355457	0.857141178
G3V1R4	1xPhospho [S237(0); S239(100); T241(0); T243(0); T246(0); T252(0); S260(0)]	0.628	-0.671163536	0.879063032
G3V1R4	2xPhospho [S210(0.1); T213(96.6); S216(3.2); T217(0.1); S220(100); T225(0); T230(0); S233(0)]	0.775	-0.367731785	0.965013037
G3V1R4	1xPhospho [S507(100); S515(0); T519(0)]	1.042	0.059355278	0.99184412
G3V1S3	2xPhospho [S1111(100); T1113(2.5); S1114(97.5); T1116(0)]	0.853	-0.229382353	0.984063817
G3V1V1	2xPhospho [S165(0); T166(0); Y167(0); S169(100); S175(100)]	0.675	-0.567040593	0.945786032
G3V1V1	2xPhospho [S165(0); T166(0); Y167(0); S169(100); S175(100)]	0.985	-0.02180437	0.998086195
G3V1X9	1xPhospho [S92(100); S95(0); S97(0)]	2.65	1.40599236	0.163999738
G3V2B5	2xPhospho [S327(97.7); T328(2.3); S337(100); S339(0); T343(0)]	0.53	-0.915935735	0.697933569
G3V2W9	2xPhospho [Y80(0); Y81(0); Y85(0); S88(100); S90(100); T100(0)]	0.669	-0.579921884	0.799129383
G3V2W9	2xPhospho [Y80(0); Y81(0); Y85(0); S88(100); S90(100)]	0.737	-0.440263476	0.908602715
G3V3A7	2xPhospho [S83(5.2); S85(94.8); T94(0); S97(100); S101(0); Y105(0)]	0.123	-3.023269779	0.000514461
G3V3F1	1xPhospho [T512(0); S513(0); S517(0); T522(0); S523(100); S525(0); S526(0); T529(0)]	1.259	0.332278283	0.915988867
G3V3N4	2xPhospho [T311(0); S313(100); T315(0); S316(0); S319(100)]	4.222	2.077926579	0.012483044
G3V3N4	2xPhospho [S327(95.6); T329(8.7); S331(95.4); S333(0.2); S334(0); S336(0); T338(0); S340(0); S341(0); T347(0); S351(0)]	1.487	0.572404647	0.749797187

G3V3N4	3xPhospho [S309(0); T311(100); S313(100); T315(0); S316(0); S319(100)]	1.091	0.125651102	0.962617576
G3V3N4	3xPhospho [S327(0.3); T329(99.7); S331(100); S333(99.7); S334(0.3); S336(0); T338(0); S340(0); S341(0); T347(0); S351(0)]	0.879	-0.18606493	0.99184412
G3V4T7	2xPhospho [S308(100); T309(0.2); S311(87.6); S313(4.1); S314(4.1); S315(4.1); T317(0); S318(0); T324(0)]	1.91	0.933572638	0.630710862
G3V4T7	2xPhospho [S445(100); T446(0); S448(100); T452(0)]	1.235	0.304511042	0.902447274
G3V4T7	3xPhospho [S308(93.5); T309(6.5); S311(99.4); S313(32.8); S314(32.8); S315(32.8); T317(2.3); S318(0); T324(0)]	1.272	0.347098671	0.920253354
G3V4T7	1xPhospho [S465(0); S467(100)]	0.979	-0.030619235	0.980895643
G3V4X8	1xPhospho [S284(99.9); Y286(0); S289(0.1)]	100	6.64385619	8.35E-17
G3V4X8	1xPhospho [S62(100); S70(0); S72(0)]	1.577	0.65718266	0.688416082
G3V4X8	2xPhospho [S62(100); S70(100); S72(0)]	0.704	-0.506352666	0.886312012
G3V4X8	2xPhospho [S62(100); S70(100); S72(0)]	0.765	-0.386468347	0.921519407
G3V4X8	2xPhospho [S62(100); S70(100); S72(0)]	1.1	0.137503524	0.973263372
G3V4X8	2xPhospho [S62(100); S70(100); S72(0.1)]	1.054	0.075874867	0.983199623
G3V515	1xPhospho [S123(100); T125(0); S128(0); S129(0)]	1.144	0.194087052	0.938029982
G3V583	2xPhospho [S30(0); T33(0); Y37(0); S38(100); T39(100)]	0.01	-6.64385619	8.35E-17
G3V583	2xPhospho [S30(0); T33(0); Y37(0); S38(100); T39(100)]	0.733	-0.448114897	0.86233669
G3V583	2xPhospho [S30(0); T33(0); Y37(0); S38(100); T39(100)]	0.731	-0.452056689	0.929390092
G3V583	2xPhospho [S30(0); T33(0); Y37(0); S38(100); T39(100)]	0.878	-0.187707155	0.99184412
G3V599	3xPhospho [S1125(100); T1127(0.2); S1129(99.9); S1131(100)]	0.01	-6.64385619	8.35E-17
G3V599	2xPhospho [S1125(0); T1127(0); S1129(100); S1131(100)]	0.509	-0.974262439	0.794478982
G3V599	3xPhospho [T1118(0); S1125(100); T1127(50); S1129(50); S1131(100)]	0.563	-0.828793173	0.797921038
G3V599	1xPhospho [S1177(0); S1182(100)]	1.352	0.435095152	0.849852772
G3V599	2xPhospho [S1125(0); T1127(0); S1129(100); S1131(100)]	1.13	0.176322773	0.950730609
G3V5D0	2xPhospho [T83(0); S86(100); S88(97.9); T91(2.1)]	0.01	-6.64385619	8.35E-17
G3XA95	2xPhospho [S265(98); T267(51); T268(51); T272(0); T274(0); T275(0); Y279(0); S281(0)]	1.08	0.111031312	0.971774764
G3XAA0	2xPhospho [S1213(50); S1214(50); T1215(2.7); S1217(97.3); S1223(0); S1226(0); S1228(0); S1229(0)]	0.395	-1.340075442	0.552024958
G3XAC6	1xPhospho [S136(100); T146(0)]	1.125	0.169925001	0.965553053
G3XAD6	3xPhospho [T64(0); T66(0); S70(100); S72(100); S74(100); T84(0)]	0.01	-6.64385619	8.35E-17
G3XAD6	1xPhospho [S486(100); T488(0); S490(0); S494(0)]	1.661	0.732052073	0.552934674
G3XAD6	3xPhospho [S486(100); T488(0); S490(100); S494(100)]	1.646	0.718964336	0.655156419
G3XAD6	3xPhospho [S486(100); T488(2.9); S490(97.1); S494(100); Y499(0)]	1.533	0.616357697	0.742967319
G3XAD6	2xPhospho [S486(94.5); T488(5.5); S490(5.5); S494(94.5); Y499(0)]	1.398	0.483364361	0.806224461
G3XAD6	1xPhospho [S486(100); T488(0); S490(0); S494(0)]	1.367	0.451013243	0.839392617
G3XAD6	1xPhospho [T243(0); S244(0); S247(100)]	1.242	0.312665174	0.899170792
G3XAD6	3xPhospho [S486(100); T488(0); S490(100); S494(100)]	1.247	0.318461465	0.925808485
G3XAD6	2xPhospho [S486(100); T488(0); S490(0); S494(100); Y499(0)]	0.812	-0.300448367	0.925912375
G3XAD6	2xPhospho [S486(100); T488(0); S490(0); S494(100)]	1.189	0.249748715	0.939053555
G3XAD6	2xPhospho [S486(49.6); T488(49.6); S490(0.8); S494(100)]	0.888	-0.171368418	0.964495484
G3XAL9	2xPhospho [T212(100); T217(100)]	1.924	0.944108799	0.452638679
G5E9D3	2xPhospho [T258(0.5); S264(92.8); S265(6.7); S278(0); S279(0); T282(0); S285(93.2); T287(6.8)]	0.051	-4.293358943	2.27E-05
G5E9D3	1xPhospho [T258(0); S264(0); S265(0); S278(0); S279(0); T282(0); S285(100); T287(0)]	0.631	-0.66428809	0.701439311
G5E9D5	2xPhospho [S189(100); S193(0.1); S194(99.9); S196(0); S198(0); S210(0)]	1.721	0.783247097	0.481943049
G5E9D5	1xPhospho [S180(100); S186(0)]	1.331	0.412510571	0.872730156
G5E9D5	3xPhospho [S189(0.3); S193(99.7); S194(99.8); S196(5.1); S198(95.2); S210(0)]	1.089	0.123003954	0.971838536
G5E9D7	1xPhospho [S64(100)]	0.01	-6.64385619	8.35E-17
G5E9D7	2xPhospho [S165(100); Y175(0); S177(100); Y181(0)]	0.622	-0.685013515	0.690335172
G5E9E7	2xPhospho [T844(5.2); S845(94.5); Y847(0.3); T850(100); T852(0); Y857(0); T858(0); T865(0); T872(0); S876(0); T879(0)]	0.01	-6.64385619	8.35E-17
G5E9E7	1xPhospho [S1290(6); S1302(0.4); S1305(93.6); S1313(0); S1317(0); S1320(0); S1321(0); Y1322(0); S1323(0); S1324(0)]	0.132	-2.921390165	0.003111144
G5E9E7	2xPhospho [S1290(6.6); S1302(93.4); S1305(0); S1313(98.9); S1317(0.5); S1320(0.5); S1321(0); Y1322(0); S1323(0); S1324(0)]	3.425	1.776103988	0.05982589
G5E9E7	2xPhospho [S1501(3.1); S1503(96.9); S1512(0); T1516(0); S1518(100)]	0.467	-1.098505545	0.458787027
G5E9E7	1xPhospho [S621(100); S626(0); T632(0)]	1.651	0.72334012	0.543749652
G5E9E7	1xPhospho [S1104(0); S1112(100)]	1.987	0.990591873	0.607772032
G5E9E7	2xPhospho [T271(0); S279(0); S281(0); S284(0); S288(0.1); S294(0.8); S298(99.1); S301(0.8); S304(99.3)]	0.66	-0.59946207	0.836491646
G5E9E7	1xPhospho [S621(100); S626(0); T632(0)]	1.391	0.47612242	0.855302203
G5E9E7	2xPhospho [T271(0); S279(0); S281(100); S284(100); S288(0)]	0.72	-0.473931188	0.920518605
G5E9E7	2xPhospho [S121(0); S129(100); S135(100); Y136(0)]	0.826	-0.275786313	0.922868578
G5E9E7	1xPhospho [S121(0); S129(93.5); S135(6.5); Y136(0)]	1.201	0.264236151	0.926103516
G5E9E7	1xPhospho [S889(0); S890(0); T898(0); Y899(0); Y902(0); S903(100)]	0.776	-0.365871442	0.936043234
G5E9E7	2xPhospho [S121(0); S129(100); S135(100); Y136(0)]	0.88	-0.184424571	0.962617576
G5E9E7	1xPhospho [S916(100); S923(0); Y927(0)]	0.82	-0.286304185	0.965139891
G5E9E7	2xPhospho [S294(0); S298(0); S301(100); S304(100)]	0.711	-0.492078535	0.970754282
G5E9E7	1xPhospho [Y1024(0); S1026(0); S1035(100); S1040(0)]	1.021	0.029982866	0.981858798
G5E9E7	1xPhospho [S1501(2); S1503(98); S1512(0); T1516(0); S1518(0)]	0.792	-0.336427665	0.982345843
G5E9E7	2xPhospho [S1479(0); T1481(0); S1488(4.5); S1489(4.5); T1491(90.8); T1493(0.5); S1494(99.7)]	0.857	-0.222632891	1
G5E9E7	3xPhospho [S294(0); S298(1.3); S301(99.6); S304(99.6); S307(99.6)]	0.929	-0.106249498	1
G5E9I1	1xPhospho [S366(0); S373(100); T375(0)]	1.629	0.703986604	0.781247135
G5E9I1	1xPhospho [S366(0); S373(100); T375(0)]	0.937	-0.093879047	0.981858798

G5E9L8	1xPhospho [S685(100); T690(0); S695(0)]	4.019	2.006836577	0.082095072
G5E9M5	1xPhospho [S56(0); S57(0); S62(100); S73(0)]	3.909	1.966799585	0.006044482
G5E9M5	1xPhospho [Y781(0); S787(0); Y788(0); S789(0); Y790(0); S791(0); S793(0); Y794(0); S796(100); S803(0); Y805(0); Y807(0); S809(0)]	2.129	1.09017595	0.37844647
G5E9M5	1xPhospho [S482(100); T486(0); S503(0); T504(0); S506(0); S511(0); T514(0)]	1.313	0.392866916	0.900153261
G5E9M5	1xPhospho [S748(93.4); Y749(0); S751(6.2); Y753(0); S755(0.4); S762(0); Y763(0); S766(0); Y768(0); S769(0); Y771(0)]	0.993	-0.010134377	0.972294713
G5E9M5	2xPhospho [S482(99.6); T486(0.4); S503(94.3); T504(5.7); S506(0); S511(0); T514(0)]	0.864	-0.210896782	0.987554799
G5E9M5	1xPhospho [S820(0); S824(0); Y825(0); S827(0); S831(0); Y832(0); S836(0); Y840(0); S844(0); S848(97.5); S849(2.5); Y850(0)]	0.961	-0.057391664	1
G5E9X3	3xPhospho [S202(3.6); S203(96.4); S206(50); S207(50); S213(100)]	0.747	-0.420819852	0.94913059
G5EA30	2xPhospho [S18(51.7); S21(51.7); S22(96.2); S25(0.5)]	0.677	-0.562772261	0.950730609
G5EA48	3xPhospho [S936(0); T938(100); T941(0.4); S942(93.8); T943(6.2); S945(99.6); S947(0)]	0.01	-6.64385619	8.35E-17
G5EA48	1xPhospho [S772(100); T774(0); Y781(0)]	0.01	-6.64385619	8.35E-17
G5EA48	2xPhospho [S936(0); T938(0); T941(0); S942(99.9); T943(0.2); S945(99.9); S947(0)]	0.373	-1.422752464	0.168561678
G5EA48	1xPhospho [S854(100); T856(0); S859(0)]	0.553	-0.854648614	0.769999353
G8JLB5	1xPhospho [S477(0); Y479(0); S481(100); T485(0)]	1.6	0.678071905	0.714293599
G8JLC6	1xPhospho [S1892(0); S1894(0); T1895(0); S1896(0); S1900(0); S1906(100)]	0.81	-0.304006187	0.960831064
G8JLC6	1xPhospho [S1717(0); S1721(0); S1725(0); S1727(0); S1731(0); T1733(0); T1735(0); S1739(96.2); S1740(1.9); S1741(1.9)]	0.852	-0.231074664	0.965139891
G8JLD5	1xPhospho [S583(0); S592(100)]	1.124	0.168642036	0.962617576
G8JLF3	2xPhospho [S310(100); S312(100)]	0.455	-1.13606155	0.636224002
G8JLF3	2xPhospho [S339(0); S349(0); T353(0); S355(100); S356(100); T366(0)]	1.499	0.584000383	0.744877142
G8JLF3	3xPhospho [S339(100); S349(0); T353(0); S355(100); S356(100); T366(0)]	0.663	-0.592919225	0.851597639
G8JLF3	2xPhospho [S310(100); S312(100)]	0.723	-0.467932448	0.892604794
G8JLF3	2xPhospho [S481(100); T484(100)]	0.893	-0.16326792	0.965013037
G8JLF3	2xPhospho [S510(0); S525(100); S526(100)]	0.742	-0.430508908	0.965139891
G8JLF3	2xPhospho [S481(100); T484(100)]	0.929	-0.106249498	0.975403456
G8JLF3	2xPhospho [S510(0); S525(100); S526(100)]	0.828	-0.272297327	0.980895643
G8JLF3	2xPhospho [S510(0); S525(100); S526(100)]	0.916	-0.126580497	0.984145373
G8JLM5	1xPhospho [S44(100); T51(0); T53(0); T55(0)]	100	6.64385619	8.35E-17
G8JLM5	2xPhospho [T287(4.6); S289(95); S292(0.2); S296(0.2); T302(0); S303(0); T304(0); T305(0); T307(0); S310(100); T314(0); T315(0); T318(0); S320(0); T321(0); S322(0)]	0.78	-0.358453971	0.96955242
H0Y2Y8	1xPhospho [S271(0); T274(0.2); S276(99.8); S281(0); T283(0); Y284(0)]	1.903	0.928275562	0.47518367
H0Y2Y8	2xPhospho [S271(100); T274(0); S276(0); S281(99.9); T283(0.2); Y284(0)]	1.304	0.38294387	0.872730156
H0Y2Y8	1xPhospho [S312(100); T320(0)]	1.227	0.295135249	0.900153261
H0Y337	2xPhospho [Y195(0); T200(0); T209(100); S211(0); S213(100); T221(0); S223(0)]	100	6.64385619	8.35E-17
H0Y390	2xPhospho [S4276(96.9); T4278(49.9); S4280(49.7); S4281(3); S4282(0); S4283(0); S4285(0.2); S4287(0.2); S4290(0); T4292(0); S4293(0); S4296(0); S4297(0); T4300(0); S4303(0); T4305(0)]	0.01	-6.64385619	8.35E-17
H0Y390	1xPhospho [S1567(100)]	100	6.64385619	8.35E-17
H0Y390	2xPhospho [S4376(100); S4379(100); T4386(0); S4388(0); S4391(0); T4393(0); S4394(0); S4396(0); S4397(0); S4405(0)]	0.417	-1.261880711	0.44984964
H0Y390	2xPhospho [T4319(0); T4321(0); S4324(50); S4325(50); T4327(0.1); S4328(100); T4333(0); S4334(0); S4336(0); S4337(0); S4338(0); S4341(0); T4342(0)]	0.683	-0.550042516	0.723988927
H0Y390	3xPhospho [T4319(0); T4321(0); S4324(4.3); S4325(95.5); T4327(0.2); S4328(100); T4333(32.8); S4334(32.8); S4336(32.8); S4337(1.5); S4338(0.1); S4341(0.1); T4342(0)]	0.868	-0.204233052	0.962617576
H0Y390	1xPhospho [T4319(0); T4321(0); S4324(1.7); S4325(98.3)]	0.935	-0.09696173	0.980895643
H0Y390	1xPhospho [S4013(99.9); S4015(0.1); T4018(0); S4026(0); S4028(0)]	0.832	-0.265344567	0.984063817
H0Y449	1xPhospho [Y258(0); S259(100)]	0.243	-2.040971781	0.238574596
H0Y449	1xPhospho [Y208(0); Y212(0); S215(0); S217(0); S224(0); S226(100)]	1.204	0.267835392	0.933107775
H0Y449	2xPhospho [Y208(0); Y212(0); S215(50); S217(50); S224(0); S226(100)]	1.137	0.185232254	0.964978421
H0Y449	3xPhospho [Y208(0); Y212(0); S215(100); S217(0); S224(100); S226(100)]	0.733	-0.448114897	0.971220368
H0Y4C3	2xPhospho [T842(0); S843(0); T849(0.3); S850(99.7); S853(50); S854(50); T858(0)]	0.01	-6.64385619	8.35E-17
H0Y4C3	2xPhospho [T842(0); S843(0); T849(0); S850(3.1); S853(99.9); S854(97.1); T858(0)]	0.419	-1.254977851	0.463267339
H0Y4E8	1xPhospho [S908(100); S911(0); S916(0)]	0.289	-1.790858602	0.225839425
H0Y4E8	1xPhospho [S772(2.8); S773(97.2)]	0.75	-0.415037499	0.964978421
H0Y4Q6	1xPhospho [S410(100); T412(0); S413(0)]	1.112	0.153156788	0.943939997
H0Y4Q6	1xPhospho [S401(0); S402(0); T404(100)]	1.002	0.002882509	0.999714477
H0Y4V9	1xPhospho [T76(0); S77(0); S78(0); T80(0); S82(0); T84(0); S90(99.8); S92(0.2); S98(0); S99(0)]	0.01	-6.64385619	8.35E-17
H0Y4V9	2xPhospho [T76(0); S77(0); S78(0); T80(0.2); S82(49.9); T84(49.9); S90(5.8); S92(94.2); S98(0); S99(0)]	0.563	-0.828793173	0.526777654
H0Y4V9	2xPhospho [S151(0); S155(0); S159(0); T161(0); Y162(0); S165(100); S167(100)]	1.202	0.265436896	0.928642034
H0Y4V9	1xPhospho [S165(0); S167(100)]	1.14	0.189033824	0.935764035
H0Y4V9	3xPhospho [T76(6.6); S77(6.6); S78(6.6); T80(1.4); S82(81.9); T84(96.8); S90(99.6); S92(0.4); S98(0); S99(0)]	0.886	-0.174621396	0.989898705
H0Y579	2xPhospho [T25(0); T29(0); S30(100); T32(0); T34(0); S36(92.9); T37(3.6); S38(3.6); S41(0); S42(0)]	1.854	0.890641244	0.374346263
H0Y579	1xPhospho [T25(0); T29(0); S30(100); T32(0); T34(0); S36(0); T37(0); S38(0); S41(0); S42(0)]	1.305	0.384049807	0.870080472
H0Y589	2xPhospho [Y432(0); T433(100); T434(100)]	0.272	-1.878321443	0.044193091
H0Y589	2xPhospho [Y578(0); Y580(0); S582(100); S585(100)]	0.401	-1.318325858	0.392224068
H0Y5B0	2xPhospho [S258(100); S261(100)]; E9PK52 2xPhospho [S645(100); S648(100)]	100	6.64385619	8.35E-17
H0Y5B0	1xPhospho [S225(98.9); S226(1.1); T229(0)]; E9PK52 1xPhospho [S612(98.9); S613(1.1); T616(0)]	0.572	-0.805912948	0.534130879
H0Y5B0	1xPhospho [S258(0); S261(100)]; E9PK52 1xPhospho [S645(0); S648(100)]	0.527	-0.924125133	0.646029575
H0Y5B0	1xPhospho [S41(100); S53(0)]; E9PK52 1xPhospho [S550(100); S562(0)]	0.533	-0.907792562	0.724397901
H0Y5B0	1xPhospho [S138(100); S140(0)]	0.815	-0.295128036	0.980895643

H0Y5S9	2xPhospho [T37(100); T40(100)]	11.564	3.531568609	6.42E-05
H0Y5S9	1xPhospho [S108(4.5); T110(91); S111(4.5)]	2.006	1.004321606	0.579084172
H0Y5S9	2xPhospho [S46(100); S53(100); T54(0); S57(0)]	1.316	0.396159489	0.86239429
H0Y5S9	2xPhospho [S108(100); T110(0); S111(100)]	1.138	0.186500558	0.964495484
H0Y6J6	1xPhospho [S62(100); Y68(0)]	1.876	0.907659828	0.435983252
H0Y6J6	1xPhospho [S76(100); T86(0); S87(0)]	1.336	0.417920008	0.862820654
H0Y6J6	1xPhospho [S129(100); T131(0); S134(0); S136(0); T144(0)]	0.888	-0.171368418	0.964978421
H0Y6J6	2xPhospho [S76(100); T86(2.7); S87(97.3)]	0.702	-0.510457064	0.965139891
H0Y6J6	1xPhospho [S129(100); T131(0); S134(0); S136(0); T144(0)]	1.122	0.166072676	0.967238018
H0Y6J6	1xPhospho [Y121(0.4); S129(99.2); T131(0.4); S134(0); S136(0); T144(0)]	1.015	0.021479727	0.981671686
H0Y6J6	2xPhospho [S129(96); T131(6); S134(49); S136(49); T144(0)]	0.885	-0.17625064	0.994865845
H0Y6J6	1xPhospho [S76(100); T86(0); S87(0)]	0.96	-0.058893689	1
H0Y6S1	2xPhospho [S15(100); T19(0); Y22(0); S27(100)]	0.994	-0.008682243	0.987425362
H0Y720	2xPhospho [S1118(100); Y1120(0); T1130(0); T1135(0); S1141(0); S1147(94.5); T1150(5.5)]	100	6.64385619	8.35E-17
H0Y720	1xPhospho [T70(0); S73(0); S75(0); S76(0); S80(95.8); T81(4.2); T85(0); S86(0)]	0.802	-0.318325858	0.984970017
H0Y7L2	3xPhospho [S66(50); S68(50); S70(99.6); S72(0.4); S77(0); T79(0); T81(0); S82(0); S89(100); S93(0)]	100	6.64385619	8.35E-17
H0Y7L2	2xPhospho [T30(0); Y31(0); S34(3.7); S35(3.7); S36(92.7); S45(99.8); Y48(0); T50(0.2)]	2.366	1.242450074	0.264761573
H0Y7L2	2xPhospho [S68(0); S70(0); S72(100); S77(0); T79(0); T81(0); S82(0); S89(99.9); S93(0.1)]	1.926	0.945607703	0.47167409
H0Y7L2	2xPhospho [S593(0); S595(100); S597(0); S601(100)]	0.508	-0.977099598	0.667874538
H0Y7L2	1xPhospho [S593(0); S595(0); S597(0); S601(100)]	1.219	0.285698126	0.933452347
H0Y7L2	3xPhospho [S68(52.4); S70(52.4); S72(95.1); S77(0); T79(0); T81(0); S82(0); S89(90.8); S93(9.2)]	1.139	0.187767747	0.94158127
H0Y7L2	2xPhospho [S68(0); S70(0); S72(100); S77(0); T79(0); T81(0); S82(100)]	1.193	0.254594043	0.94797789
H0Y7L2	1xPhospho [S54(0); S60(100)]	0.815	-0.295128036	0.967144405
H0Y7L2	3xPhospho [S66(0.1); S68(98.1); S70(50.9); S72(50.9); S77(0); T79(0); T81(0); S82(100)]	0.87	-0.200912694	0.974924677
H0Y7L2	2xPhospho [S66(2.5); S68(97.5); S70(0); S72(100); S77(0); T79(0); T81(0); S82(0)]	1	0	0.994773029
H0Y7W6	1xPhospho [S299(0); S301(0); S303(0); S311(100)]	0.637	-0.650634722	0.927625482
H0Y8C4	1xPhospho [S475(100); Y482(0); T483(0)]	1.37	0.454175893	0.793785899
H0Y8C6	1xPhospho [T635(0); S637(0); T647(0); S654(100); S669(0)]	1.576	0.656267535	0.682660189
H0Y8K6	1xPhospho [S149(100); T161(0)]	1.674	0.743299528	0.720161581
H0Y8P3	2xPhospho [S47(0); T50(100); S52(100)]	0.552	-0.857259828	0.742559804
H0Y8P3	1xPhospho [S553(100); T559(0); T561(0)]	0.729	-0.45600928	0.964978421
H0Y917	1xPhospho [S113(100); S115(0)]	0.01	-6.64385619	8.35E-17
H0Y994	2xPhospho [S23(0); Y25(0); S28(0.1); S30(50.7); S34(50.7); S37(98.5)]	0.774	-0.369594529	0.969680228
H0Y9I4	3xPhospho [S219(33.3); T220(33.3); S223(33.3); S232(100); S234(100); Y238(0); S239(0); S248(0); S249(0); S250(0)]	1.097	0.133563526	0.941951779
H0Y9I4	1xPhospho [S195(0); S206(100)]	0.742	-0.430508908	0.973263372
H0Y9K3	2xPhospho [S104(50); S105(50); S108(100)]	0.081	-3.625934282	0.000516392
H0Y9K3	1xPhospho [S29(100); S35(0); S36(0)]	1.184	0.243669081	0.933498766
H0YAJ5	1xPhospho [S377(0); S379(100); S382(0)]	7.199	2.847796518	0.001624831
H0YAJ5	1xPhospho [S113(100); T115(0); T121(0); S122(0)]	0.657	-0.606034724	0.935251324
H0YAU4	2xPhospho [T217(3); T219(0); T221(97); S225(100); T227(0)]	0.412	-1.279283757	0.327266203
H0YB16	2xPhospho [S512(100); S515(100); S522(0); Y533(0)]	0.01	-6.64385619	8.35E-17
H0YB16	2xPhospho [S559(100); S562(100); S564(0); S565(0); S569(0); Y570(0)]	100	6.64385619	8.35E-17
H0YB67	1xPhospho [S140(95.7); S144(4.1); S145(0.2)]	0.844	-0.244685096	0.984145373
H0YBA8	2xPhospho [S383(3.8); T385(96.2); S387(0); S389(100); T391(0); S395(0); T397(0)]	9.54	3.253989266	7.80E-05
H0YBA8	1xPhospho [S23(100); S28(0); T30(0); T31(0); S33(0)]	1.513	0.597411988	0.742967319
H0YBW3	1xPhospho [S10(0); T11(0); T23(0); S24(0); S28(98); S30(2)]	1.219	0.285698126	0.93019184
H0YC88	2xPhospho [Y21(0); S22(100); T27(100); T29(0); Y32(0)]	0.01	-6.64385619	8.35E-17
H0YC88	2xPhospho [Y21(0); S22(100); T27(97.5); T29(2.5); Y32(0)]	0.01	-6.64385619	8.35E-17
H0YC88	2xPhospho [Y21(0); S22(97.4); T27(51.3); T29(51.3); Y32(0)]	0.01	-6.64385619	8.35E-17
H0YCG0	2xPhospho [S291(0); S294(0); S296(0); S302(100); S305(100)]	100	6.64385619	8.35E-17
H0YCK3	1xPhospho [T386(0); T391(0); S392(0); S395(100); T400(0); T401(0)]	0.549	-0.865121946	0.684328394
H0YDE2	2xPhospho [T474(0); S485(0); S487(0); T488(0); S489(0); S491(0); S498(100); T501(50); S502(50)]	0.547	-0.870387262	0.625045843
H0YEH2	1xPhospho [T129(100)]	1.86	0.895302621	0.614521403
H0YEH2	1xPhospho [S92(100); Y100(0)]	0.572	-0.805912948	0.872730156
H0YEH2	2xPhospho [S214(0); S219(0); S223(100); S226(100)]	0.733	-0.448114897	0.93651142
H0YEH2	2xPhospho [S229(96.9); S231(3.1); S237(0); Y241(0); S244(0); S245(0.1); S246(99.9); S250(0)]	0.665	-0.588573754	0.944356286
H0YEH2	1xPhospho [S229(0); S231(0); S237(0); Y241(0); S244(0); S245(0); S246(100); S250(0)]	0.883	-0.179514657	0.996295697
H0YEH2	1xPhospho [S141(100)]	0.863	-0.212567535	0.99811985
H0YEX2	2xPhospho [T358(0); S360(0.2); S361(99.8); S364(100)]	1.153	0.205392513	0.938029982
H0YEX2	2xPhospho [S184(50); T186(50); S190(100)]	0.796	-0.329159664	0.984063817
H0YFK6	2xPhospho [Y7(0); S8(0); S13(100); S15(100); S21(0); T23(0)]	1.318	0.39835037	0.872329982
H0YFK6	2xPhospho [Y7(0); S8(0); S13(100); S15(100); S21(0); T23(0)]	0.965	-0.051399153	0.993670231
H0YFN7	2xPhospho [S879(0); S882(0.2); T884(99.8); S887(3.9); T888(96.1); T890(0); S895(0); S896(0); S897(0)]	0.244	-2.035046947	0.054153864
H0YFS2	1xPhospho [S100(0); S105(100)]	1.515	0.599317794	0.698520686
H0YFS2	2xPhospho [S100(100); S105(100)]	1.028	0.039840265	0.983654603
H0YFS2	1xPhospho [S100(0); S105(100)]	0.951	-0.072482754	1
H0YFV6	1xPhospho [T298(0); S300(0); T306(1.6); S308(98.4); S313(0); S314(0)]	1.203	0.266636643	0.907438338
H0YG16	1xPhospho [S260(0); S262(100); T264(0)]	1.639	0.712815854	0.71389078
H0YG16	1xPhospho [S230(0); S231(100)]	1.14	0.189033824	0.965139891
H0YG16	1xPhospho [S152(100); S155(0)]	1.062	0.086783766	0.980895643
H0YG16	2xPhospho [S152(100); S155(100)]	0.913	-0.131313235	0.990288616

H0YH66	1xPhospho [S174(100)]	1.94	0.956056652	0.469342919
H0YHA7	2xPhospho [T137(100); S140(97.9); T142(2.1); Y145(0)]	1.862	0.896853073	0.560423398
H0YHA7	1xPhospho [T137(100); S140(0); T142(0); Y145(0)]	1.609	0.686164326	0.724583498
H0YHG0	1xPhospho [S475(0); S476(98.9); S478(1)]	0.84	-0.251538767	0.980895643
H0YHG0	1xPhospho [T498(0.1); S499(96.7); S500(3.2); T503(0); T505(0); T506(0); T509(0)]	0.948	-0.077041036	0.998672612
H0YI46	1xPhospho [S176(0); S179(97.3); T180(2.7)]	0.858	-0.220950447	0.999263007
H0YIQ2	1xPhospho [S1352(100); S1354(0)]	1.574	0.654435541	0.747592617
H0YIQ2	1xPhospho [S407(100); S413(0); T422(0); S423(0); S424(0)]	0.982	-0.02620507	0.980895643
H0YIS7	1xPhospho [Y126(0); S129(0); S137(100)]	1.101	0.138814469	0.962617576
H0YIS7	2xPhospho [T180(95.1); S182(4.9); S187(0.3); S191(99.7); T202(0)]	0.884	-0.177881725	0.999714477
H0YIX9	1xPhospho [S218(0); S219(100); S221(0.1); S224(0); T227(0)]	9.845	3.299391206	0.000871966
H0YIX9	1xPhospho [S30(2.4); S31(97.6); Y34(0); T35(0); S37(0); T38(0); T49(0)]; E9PBB4 1xPhospho [S141(2.4); S142(97.6); Y145(0); T146(0); S148(0); T149(0); T160(0)]	0.979	-0.030619235	0.969680228
H0YJA2	1xPhospho [Y531(0); S536(100)]	2.551	1.351062899	0.268448849
H0YJA2	1xPhospho [Y237(0); S239(100)]	2.02	1.014355293	0.496491776
H0YJA2	1xPhospho [S431(100)]	1.646	0.718964336	0.656131188
H0YJA2	1xPhospho [T241(99.9); S243(0.1); S245(0); S246(0); S247(0); S249(0)]	1.321	0.401630467	0.872730156
H0YJA2	1xPhospho [S325(100); T333(0)]	1.269	0.343692069	0.900153261
H0YJA2	2xPhospho [T241(97.7); S243(2.3); S245(0); S246(0); S247(0); S249(100)]	1.18	0.23878686	0.930376591
H0YJA2	1xPhospho [S156(100)]	0.907	-0.140825544	1
H0YJA2	1xPhospho [S325(100); T333(0); S337(0)]	0.908	-0.139235797	1
H0YJL9	1xPhospho [S37(100); T39(0); Y41(0)]	1.222	0.289244285	0.930376591
H0YJV7	1xPhospho [S13(0); Y14(0); S16(100)]	1.593	0.671746267	0.675336505
H0YJV7	1xPhospho [T65(0); S76(100); Y80(0); S81(0); Y83(0); T85(0)]	1.604	0.681674142	0.703538855
H0YJV7	1xPhospho [T65(0); S76(100); Y80(0); S81(0); Y83(0); T85(0)]	1.353	0.436161839	0.839392617
H0YJY9	3xPhospho [Y56(100); T70(100); Y73(100)]	0.939	-0.090802937	0.981858798
H0YK58	3xPhospho [S21(0); S28(100); S35(100); T37(100); T44(0); S50(0)]	0.391	-1.354759487	0.150189095
H0YK58	2xPhospho [S21(0); S28(100); S35(94.8); T37(5.2); T44(0); S50(0)]	0.575	-0.798366139	0.607744787
H0YKC9	3xPhospho [S291(100); S296(100); S298(100); S299(0)]	0.154	-2.698997744	9.29E-05
H0YKM5	1xPhospho [S105(100); T108(0); Y110(0)]	100	6.64385619	8.35E-17
H0YLT6	2xPhospho [S210(0); S218(100); S224(100); Y225(0)]	0.625	-0.678071905	0.66184163
H0YLT6	2xPhospho [S210(0); S218(100); S224(100); Y225(0)]	0.748	-0.418889825	0.899435673
H0YMD0	2xPhospho [S12(0); S18(0); T19(0); S22(100); Y24(0); S26(100)]	1.7	0.765534746	0.724102603
H0YMD0	1xPhospho [S12(0); S18(0); T19(0); S22(100); Y24(0); S26(0)]	0.755	-0.40545145	0.879063032
H0YME5	1xPhospho [T473(100); S478(0)]	0.85	-0.234465254	1
H0YMH7	1xPhospho [S104(100); S112(0); Y113(0); T116(0)]	1.834	0.874993639	0.538037433
H0YMH7	1xPhospho [S104(100); S112(0); Y113(0); T116(0)]	2.212	1.145351386	0.539112137
H0YMP9	1xPhospho [S66(100); S76(0); T77(0); Y81(0); S87(0)]	100	6.64385619	8.35E-17
H3BNZ1	1xPhospho [S709(96.3); S710(3.7); T716(0); S720(0); S721(0); T722(0); S730(0); T734(0); T737(0)]	0.01	-6.64385619	8.35E-17
H3BNZ1	3xPhospho [S687(0); S691(0.1); T693(99.9); S694(99.9); S695(0.2); S697(99.9)]	0.269	-1.894321922	0.078139125
H3BNZ1	2xPhospho [S687(0); S691(97.3); T693(0.2); S694(5.3); S695(97.3); S697(0)]	0.425	-1.234465254	0.29564479
H3BNZ1	2xPhospho [S683(0.4); S687(100); S691(99.7); T693(0); S694(0); S695(0); S697(0)]	1.134	0.18142064	0.942574721
H3BNZ1	3xPhospho [S683(100); S687(100); S691(0); T693(2.2); S694(47.8); S695(47.8); S697(2.2)]	1.108	0.147957881	0.959719138
H3BNZ1	1xPhospho [S687(0); S691(0); T693(0); S694(0); S695(97.5); S697(2.5)]	1.009	0.012926174	0.971838536
H3BNZ1	2xPhospho [S683(49.9); S687(49.9); S691(0.1); T693(0.2); S694(4); S695(95.9); S697(0)]	0.81	-0.304006187	0.973763863
H3BNZ1	1xPhospho [S752(0); S759(100)]	1.063	0.088141597	0.975403456
H3BP95	2xPhospho [T24(0); S28(100); T36(99.2); S39(0.8); S42(0); Y43(0); S45(0)]	0.01	-6.64385619	8.35E-17
H3BQZ7	3xPhospho [S161(100); T165(100); S168(100)]	0.01	-6.64385619	8.35E-17
H3BQZ7	1xPhospho [S226(4.1); S228(95.9); T244(0); T250(0); Y251(0); T252(0); S253(0); S260(0)]	0.204	-2.293358943	0.018256781
H3BQZ7	1xPhospho [S161(100); T165(0); S168(0)]	1.163	0.217851097	0.954539739
H3BQZ7	1xPhospho [S161(100); T165(0); S168(0)]	0.975	-0.036525876	0.980895643
H3BQZ7	2xPhospho [S161(100); T165(100); S168(0)]	0.931	-0.103146927	0.989838391
H3BR05	2xPhospho [S227(100); S234(100); S236(0)]	100	6.64385619	8.35E-17
H3BR05	1xPhospho [S227(0); S234(100); S236(0)]	1.859	0.89452677	0.506667356
H3BR84	2xPhospho [S12(100); S14(100); T19(0)]	1.114	0.155749233	0.960298686
H3BRC0	1xPhospho [S57(0); S59(0); S63(0); T65(0); S75(5.8); S76(5.8); S78(88.4)]	0.01	-6.64385619	8.35E-17
H3BRM1	1xPhospho [S262(100); T270(0)]	1.701	0.766383141	0.701261726
H3BRM1	1xPhospho [S115(100); Y120(0)]	1.197	0.259423152	0.925732896
H3BRV0	3xPhospho [S168(100); S171(100); S172(100); S182(0); T185(0)]	0.301	-1.732164608	0.324183609
H3BRV0	2xPhospho [T6(0); T7(0.2); S9(99.7); S11(96.1); S13(4.1); S15(0); S16(0); S18(0); T24(0); Y31(0)]	0.687	-0.541617996	0.867320208
H3BRV0	1xPhospho [S39(100); T45(0)]	0.863	-0.212567535	0.963170174
H3BRV0	1xPhospho [S39(100); T45(0)]	0.843	-0.246395464	0.981858798
H3BS19	2xPhospho [S2567(100); S2570(0); S2577(100); T2579(0)]	1.575	0.655351829	0.688448945
H3BS86	2xPhospho [S19(100); S23(99.8); S25(0.2)]	2.196	1.134878054	0.289774883
H3BS86	1xPhospho [S19(100); S23(0); S25(0)]	1.569	0.649845352	0.701261726
H3BSV5	2xPhospho [S342(0); T343(0); Y344(0); S345(100); S347(100); T351(0); S353(0)]	1.129	0.175045486	0.9616322
H3BSW6	1xPhospho [S579(100); S586(0)]	100	6.64385619	8.35E-17
H3BT57	3xPhospho [S470(100); S479(100); S482(100); S487(0); S494(0); S499(0)]	0.837	-0.256700472	0.968663655
H3BU49	1xPhospho [S111(0); S112(0); S113(0); S117(100)]	100	6.64385619	8.35E-17
H3BU49	1xPhospho [S111(0); S112(0); S113(0); S117(100)]	11.977	3.582194682	1.67E-05
H3BV80	1xPhospho [S157(100)]	1.775	0.827819025	0.586785198
H3BV90	1xPhospho [S239(100); S241(0)]	0.741	-0.432454552	0.942574721
H3BV90	1xPhospho [S182(0); S183(0); S185(100)]	0.947	-0.078563669	0.980895643
H7BXD4	2xPhospho [T468(0); S470(100); S479(100)]	0.407	-1.2968993	0.301640205

H7BXF3	2xPhospho [T60(100); T62(0.3); Y66(0); T71(90); Y72(0); S74(4.9); S75(4.9)]	7.663	2.937909306	0.001320024
H7BXF3	2xPhospho [S103(100); S105(100); Y107(0); Y108(0); S109(0)]; Q13595 2xPhospho [S260(100); S262(100); Y264(0); Y265(0); S266(0)]	1.66	0.731183242	0.749797187
H7BXF3	1xPhospho [T60(100); T62(0); Y66(0); T71(0); Y72(0); S74(0); S75(0)]	1.35	0.432959407	0.855557911
H7BXF3	2xPhospho [T60(100); T62(0); Y66(0); T71(3.5); Y72(0); S74(93); S75(3.5)]	1.114	0.155749233	0.950730609
H7BXI1	2xPhospho [S699(99.1); S700(91.4); S701(9.4); S702(0.1); S706(0); S711(0)]	0.01	-6.64385619	8.35E-17
H7BXI1	3xPhospho [T647(50); S648(50); S651(14.2); S654(92.9); S656(92.9); S662(0); T664(0); S667(0); T668(0); S674(0)]	0.141	-2.826232932	0.001712423
H7BXI1	1xPhospho [S651(0.1); S654(99.7); S656(0.1); S662(0); T664(0); S667(0); T668(0); S674(0)]	0.23	-2.120294234	0.009695638
H7BXI1	3xPhospho [T716(0); S718(100); S721(100); S724(100); T729(0)]	0.29	-1.785875195	0.016180951
H7BXI1	2xPhospho [T647(91.2); S648(8.8); S651(83.7); S654(8.1); S656(8.1); S662(0.1); T664(0.1); S667(0); T668(0); S674(0)]	0.231	-2.114035243	0.047330495
H7BXI1	2xPhospho [S651(0); S654(0); S656(0); S662(0.2); T664(99.8); S667(0.2); T668(99.8); S674(0)]	0.306	-1.708396442	0.056855115
H7BXI1	2xPhospho [S699(0); S700(0); S701(100); S702(100); S706(0); S711(0)]	0.396	-1.336427665	0.057433542
H7BXI1	3xPhospho [S699(0.1); S700(0.1); S701(99.9); S702(100); S706(100); S711(0)]	0.349	-1.518701058	0.465165189
H7BXI1	3xPhospho [T647(50); S648(50); S651(51.9); S654(51.9); S656(96.1); S662(0); T664(0); S667(0); T668(0); S674(0)]	0.435	-1.200912694	0.489974409
H7BXI1	2xPhospho [T716(0); S718(0); S721(100); S724(100); T729(0)]	0.574	-0.800877358	0.546703084
H7BXP5	2xPhospho [S193(0); S197(0); S203(100); T206(0); S208(0); T210(0); S213(100); S217(0); T219(0); S221(0)]	0.01	-6.64385619	8.35E-17
H7BXV2	2xPhospho [Y218(0); S220(50); S221(50); T223(0); Y225(0); S227(100); T229(0); Y234(0); T236(0)]	100	6.64385619	8.35E-17
H7BXW3	1xPhospho [T123(0); S128(0); S139(100); S144(0); T150(0)]	0.553	-0.854648614	0.642489534
H7BXW3	1xPhospho [T63(100); S66(0); S67(0); T68(0); T69(0); T72(0); T74(0)]	1.015	0.021479727	0.973763863
H7BZL0	2xPhospho [S27(99.8); T29(0.1); T32(0.1); T36(0); S39(100); T43(0); T47(0); T49(0)]	0.337	-1.569179503	0.211617792
H7BZL0	2xPhospho [T36(0); S39(0); T43(100); T47(100); T49(0)]	1.227	0.295135249	0.902527096
H7BZL0	1xPhospho [T36(0); S39(100); T43(0); T47(0); T49(0)]	0.758	-0.399730246	0.97038417
H7BZL0	1xPhospho [S56(100)]	1	0	0.97726974
H7BZL0	1xPhospho [S56(100)]	0.982	-0.02620507	1
H7C0J3	2xPhospho [S371(100); S375(100); S381(0)]	0.592	-0.756330919	0.793785899
H7C0R6	2xPhospho [S55(0); T56(0); S64(5.6); T69(94.4); S72(94.4); S73(5.6); T77(0)]	0.102	-3.293358943	8.27E-05
H7C127	2xPhospho [T605(6.2); S608(93.8); S611(0.1); S614(99.1); S616(0.5); S619(0.5); T630(0)]	100	6.64385619	8.35E-17
H7C127	1xPhospho [T259(100); S263(0); S267(0)]	1.408	0.493647334	0.804811993
H7C127	2xPhospho [T605(0); S608(100); S611(0); S614(96.1); S616(3.9); S619(0); T630(0)]	1.1	0.137503524	0.969680228
H7C127	2xPhospho [T259(100); S263(100); S267(0)]	0.828	-0.272297327	0.988977631
H7C183	2xPhospho [S1035(100); S1036(100); T1041(0); S1044(0); S1047(0)]	0.794	-0.332789088	0.943797629
H7C189	2xPhospho [S394(0); S414(9); T416(91.8); S419(99.2); S429(0); T431(0); T432(0)]	3.042	1.605020153	0.108961873
H7C189	2xPhospho [S394(0); S414(7.6); T416(92.4); S419(99.9); S429(0); T431(0); T432(0)]	1.13	0.176322773	0.962617576
H7C1J4	2xPhospho [T1101(0); S1103(100); S1106(100); S1111(0); S1114(0); S1115(0)]	0.75	-0.415037499	0.954539739
H7C1Q8	2xPhospho [T143(0); S156(100); S163(49.9); S164(49.9); T165(0.1); S166(0.1)]	1.586	0.665392771	0.704919614
H7C1Q8	1xPhospho [T143(0); S156(100); S163(0); S164(0); T165(0); S166(0)]	1.156	0.209141398	0.958702638
H7C1V4	2xPhospho [T514(0); S515(0); S518(0.5); S525(99.6); S528(0.4); S529(5.7); T531(88.3); S535(5.7)]	0.01	-6.64385619	8.35E-17
H7C1V4	2xPhospho [T514(0); S515(0); S518(0); S525(93.5); S528(6.5); S529(6.5); T531(93.5)]	0.01	-6.64385619	8.35E-17
H7C2H2	2xPhospho [S31(50); S34(50); S46(100); S49(0)]	1.196	0.25821739	0.891862526
H7C2H2	2xPhospho [S31(1.6); S34(98.4); S46(100); S49(0)]	0.76	-0.395928676	0.932883109
H7C2H2	2xPhospho [S53(0); T64(100); S67(100)]	0.698	-0.518701058	0.965013037
H7C2K6	2xPhospho [S8(0); S15(100); S19(100); S21(0)]; Q9H4G0 2xPhospho [S430(0); S437(100); S441(100); S443(0)]	100	6.64385619	8.35E-17
H7C2K6	2xPhospho [S39(0); S44(100); T53(99.9); T55(0.1)]; Q9H4G0 2xPhospho [S461(0); S466(100); T475(99.9); T477(0.1)]	100	6.64385619	8.35E-17
H7C2K6	3xPhospho [S39(100); S44(100); T53(100); T55(0)]; Q9H4G0 3xPhospho [S461(100); S466(100); T475(100); T477(0)]	100	6.64385619	8.35E-17
H7C2K6	2xPhospho [S106(0.4); S107(0.4); S110(93.8); S112(93.8); T116(11.5)]	100	6.64385619	8.35E-17
H7C2K6	1xPhospho [S76(100)]; Q9H4G0 1xPhospho [S510(100)]	2.647	1.404358195	0.211617792
H7C2K6	3xPhospho [S8(100); S15(4.9); S19(99.8); S21(95.3)]; Q9H4G0 3xPhospho [S430(100); S437(4.9); S441(99.8); S443(95.3)]	1.998	0.998556583	0.508351221
H7C2K6	2xPhospho [S8(0); S15(100); S19(100); S21(0)]; Q9H4G0 2xPhospho [S430(0); S437(100); S441(100); S443(0)]	1.799	0.847195187	0.534130879
H7C2K6	2xPhospho [S8(0.6); S15(99.4); S19(100); S21(0); S39(0)]; Q9H4G0 2xPhospho [S430(0.6); S437(99.4); S441(100); S443(0); S461(0)]	1.616	0.692427198	0.751204811
H7C2N3	2xPhospho [S16(0.3); S17(99.7); S19(99.7); T21(0.3); T26(0)]	1.176	0.23388806	0.943796521
H7C2W9	1xPhospho [S86(100); Y91(0); T92(0); T95(0); Y96(0); T100(0); T101(0)]	0.665	-0.588573754	0.956041591
H7C3E5	2xPhospho [S27(100); S32(0); S35(0); S41(50); S43(50)]	0.447	-1.161653263	0.689563924
H7C3Y7	2xPhospho [S1078(100); S1081(100); S1082(0); T1085(0); Y1087(0); T1090(0)]	0.415	-1.268816758	0.555298965
H7C4L8	1xPhospho [S50(2.7); S51(94.6); S53(2.7); S55(0.1); T58(0); S60(0); Y70(0)]	0.948	-0.077041036	0.977573898
H7C4P4	2xPhospho [S58(100); S62(100); S66(0); S72(0)]	0.563	-0.828793173	0.660888692
H7C4P4	1xPhospho [S58(0); S62(100); S66(0); S72(0)]	1.041	0.057970069	0.983654603
H7C4P4	1xPhospho [S62(0); S66(100); S72(0)]	0.911	-0.134477041	0.984145373
H9KVB4	3xPhospho [S991(100); S995(100); S999(100)]	0.783	-0.352915787	0.980895643
H9KVB4	2xPhospho [S1127(100); S1136(97.3); S1138(2.7)]	0.957	-0.06340917	0.980895643
H9KVB4	2xPhospho [S1016(0); Y1021(0); Y1023(0); T1026(0); S1028(0); S1029(0); T1032(95.6); S1033(4.4); S1038(100); T1042(0); T1046(0)]	0.93	-0.104697379	0.998086195
I3L080	3xPhospho [S230(2.4); S232(67.6); S234(67.6); S236(67.1); S240(95.3)]	0.01	-6.64385619	8.35E-17
I3L080	2xPhospho [S366(7.6); S369(96.2); S370(96.2)]	0.432	-1.210896782	0.472389016
I3L080	3xPhospho [S366(100); S369(100); S370(100)]	0.579	-0.788364747	0.559441041

I3L080	1xPhospho [S657(100)]	0.775	-0.367731785	0.872730156
I3L080	3xPhospho [S366(100); S369(100); S370(100)]	0.76	-0.395928676	0.879063032
I3L080	3xPhospho [S230(0); S232(100); S234(50); S236(50); S240(100)]	0.69	-0.535331733	0.918006975
I3L080	2xPhospho [S230(0); S232(0); S234(97.1); S236(5.9); S240(97.1)]	0.63	-0.666576266	0.918281661
I3L080	1xPhospho [S618(0); S630(100)]	1.423	0.508935662	0.931841768
I3L080	1xPhospho [S657(100)]	0.862	-0.214240226	0.96293109
I3L1L3	1xPhospho [S1079(0); T1081(0); S1083(100); S1086(0)]	100	6.64385619	8.35E-17
I3L1L3	1xPhospho [S1179(0); S1187(100); T1189(0)]	1.731	0.791605725	0.479116982
I3L1L3	1xPhospho [S1179(0); S1187(100); T1189(0)]	1.573	0.653518671	0.708169902
I3L1L3	2xPhospho [S1106(100); T1110(0); T1116(0); T1120(3.5); S1123(96.5); S1127(0)]	0.606	-0.722610301	0.848293321
I3L1L3	1xPhospho [S1228(0); S1230(100); S1234(0)]	1.305	0.384049807	0.887701668
I3L1L3	1xPhospho [S1106(0); T1110(0); T1116(0); T1120(0); S1123(100); S1127(0)]	1.078	0.108357178	0.962617576
I3L1L3	1xPhospho [S1106(0); T1110(0); T1116(0); T1120(0); S1123(100); S1127(0)]	1.121	0.164786278	0.965139891
I3L1L3	1xPhospho [S1106(100); T1110(0); T1116(0); T1120(0); S1123(0); S1127(0)]	0.96	-0.058893689	0.99184412
I3L254	1xPhospho [S61(0); S63(1.9); S64(98.1)]	1.033	0.046840254	0.973263372
I3L291	2xPhospho [T393(0); Y398(0); T400(0); Y402(0); T403(0.1); T404(99.9); S408(100)]	100	6.64385619	8.35E-17
I3L2J0	2xPhospho [S1051(11.7); T1053(94.1); S1055(94.1); S1063(0); S1066(0)]	100	6.64385619	8.35E-17
I3L2J0	1xPhospho [S1186(100); S1187(0.1); S1188(0); T1193(0); S1194(0)]	1.446	0.532067552	0.869438434
I3L2J0	2xPhospho [S1186(99.9); S1187(99.9); S1188(0.1); T1193(0); S1194(0)]	0.771	-0.375197235	0.986075329
I3L2J0	2xPhospho [S2279(100); T2281(0); S2284(0.1); T2287(2.6); S2288(97.3)]	1.032	0.045442971	0.986113884
I3L2L5	1xPhospho [S16(100); S19(0); S20(0); S21(0); T25(0)]	1.306	0.385154897	0.869435859
I3L2L5	2xPhospho [T11(0.2); S12(99.8); S13(0.2); S16(99.9); S19(0); S20(0); S21(0); T25(0)]	0.838	-0.254977851	0.969482098
I3L2R9	1xPhospho [Y133(0); S136(100)]	11.304	3.498761466	3.62E-05
I3L2R9	2xPhospho [S160(99.9); S161(93.2); T162(3.5); S163(3.5)]	1.001	0.001441974	0.972501767
I3L419	3xPhospho [S185(0); Y187(0); S188(0); S189(0); T190(0); S195(0); S197(100); S199(100); S201(100)]	0.01	-6.64385619	8.35E-17
I3L419	1xPhospho [S16(100); S26(0)]	1.209	0.273814245	0.91490394
J3KMW7	1xPhospho [S327(100)]	100	6.64385619	8.35E-17
J3KMW7	2xPhospho [S2482(50.9); S2483(50.9); S2485(98.2); T2494(0); Y2505(0)]	1.1	0.137503524	0.965013037
J3KMW7	1xPhospho [S2485(100); T2494(0); Y2505(0)]	0.963	-0.054392297	0.97726974
J3KMY0	1xPhospho [S68(0.1); T70(99.9)]	100	6.64385619	8.35E-17
J3KMY0	1xPhospho [S29(98.4); S31(1.6)]	1.652	0.724213687	0.767755861
J3KMY0	1xPhospho [S64(0); S68(97.8); T70(2.2)]	1.4	0.485426827	0.819921743
J3KMY7	1xPhospho [T99(0); S100(100)]	0.717	-0.479954976	0.93019184
J3KN59	1xPhospho [S235(100); T237(0); Y239(0); T240(0)]	1.182	0.241230036	0.956843841
J3KN68	2xPhospho [S274(100); S276(0); S281(93.6); S283(3.2); S285(3.2); S287(0.1); S290(0)]	0.729	-0.45600928	0.932557671
J3KNB0	1xPhospho [S539(99); T540(1); S548(0); S552(0)]	1.503	0.587845009	0.848670625
J3KNG6	1xPhospho [S465(0); S467(99.9); S471(0.1)]	0.62	-0.689659879	0.921303906
J3KNG9	2xPhospho [S31(100); T33(0); T36(0); T38(0); S50(100)]	1.737	0.796597754	0.597444241
J3KNG9	2xPhospho [T87(50); S88(50); S90(93.3); S91(6.7); T101(0)]	1.211	0.276198865	0.925853562
J3KNH7	1xPhospho [S212(100); S217(0)]	1.056	0.078609835	0.945786032
J3KNH7	1xPhospho [S169(100); T176(0); S181(0)]	0.946	-0.080087911	0.981858798
J3KNH7	2xPhospho [S169(100); T176(0); S181(100)]	0.947	-0.078563669	0.989838391
J3KNL6	3xPhospho [Y1215(0); S1222(50); S1223(50); S1226(100); S1228(4.3); S1229(95.7)]	0.436	-1.19759996	0.248365564
J3KNL6	3xPhospho [S587(0); S589(100); S592(0); T593(100); S595(100); T600(0); T605(0); S606(0); S609(0); S610(0)]	0.737	-0.440263476	0.851389813
J3KNL6	2xPhospho [S587(0); S589(0); S592(4.5); T593(95.5); S595(4.5); T600(95.5); T605(0); S606(0); S609(0); S610(0)]	0.551	-0.859875776	0.85992414
J3KNL6	2xPhospho [S1410(0); S1413(0); S1414(0); Y1421(0); Y1423(0); T1427(0); S1436(94.3); S1437(5.7); T1440(0.4); S1441(99.6)]	0.787	-0.345564459	0.946290051
J3KNL6	1xPhospho [S1356(100); S1359(0); S1362(0); S1365(0)]	1.1	0.137503524	0.964978421
J3KNL6	1xPhospho [S1069(100)]	1.104	0.142740172	0.965139891
J3KNL6	2xPhospho [S1410(0); S1413(0); S1414(0); Y1421(0); Y1423(0); T1427(0); S1436(49.9); S1437(49.9); T1440(4.9); S1441(95.2)]	0.785	-0.349235441	0.971937853
J3KNL6	1xPhospho [T1001(0); Y1009(0); S1012(0); S1014(0); S1016(99.8); S1019(0.2); S1022(0); S1025(0)]	0.994	-0.008682243	0.97998947
J3KNN5	2xPhospho [T31(0); S39(100); S41(100); Y51(0); Y54(0)]	0.688	-0.53951953	0.733427607
J3KNN5	2xPhospho [S84(100); S86(100); S101(0); S104(0)]	0.747	-0.420819852	0.908462992
J3KNN5	1xPhospho [S84(4.8); S86(95.2); S101(0); S104(0)]	1.18	0.23878686	0.938673385
J3KNN5	1xPhospho [S41(100); Y51(0); Y54(0)]	1.133	0.180147861	0.958702638
J3KNP0	2xPhospho [S184(3.3); S186(0.1); T188(93.2); S190(3.3); S195(0); T196(0); T197(0); S199(0); S202(3.5); S206(96.5)]	0.444	-1.171368418	0.385507135
J3KNP0	2xPhospho [S184(50); S186(50); T188(0); S190(0); S195(0); T196(0); T197(0); S199(7.1); S202(85.7); S206(7.1)]	0.493	-1.020340448	0.62233267
J3KNP0	2xPhospho [S184(4.1); S186(0.2); T188(4.1); S190(91.6); S195(0); T196(0); T197(0); S199(0); S202(4.3); S206(95.7)]	0.603	-0.729770093	0.822854327
J3KNP0	1xPhospho [S184(0); S186(0); T188(0); S190(0); S195(0); T196(0); T197(0); S199(0); S202(0.1); S206(99.9)]	0.705	-0.504304837	0.87877743
J3KNP0	2xPhospho [S434(0); S435(100); S438(100); T441(0)]	1.166	0.221567789	0.943939997
J3KNP0	2xPhospho [S356(0.2); S357(99.7); T358(0.2); S365(100); S376(0); S377(0)]	0.746	-0.422752464	0.956734015
J3KNP0	2xPhospho [S434(0); S435(100); S438(100); T441(0)]	1.016	0.022900402	0.963156927
J3KNP0	1xPhospho [S434(0); S435(100); S438(0); T441(0)]	0.788	-0.343732465	0.967050102
J3KNP0	1xPhospho [T154(0); S156(100); S161(0)]; Q3I768 1xPhospho [T148(0); S150(100); S155(0)]	0.874	-0.194294815	0.980895643
J3KNP0	1xPhospho [T229(0); S232(2.8); S234(97.2); S236(0); T240(0); S241(0)]	1.006	0.008630305	0.982743754

J3KNP0	1xPhospho [S184(0); S186(0); T188(0); S190(0); S195(0); T196(0); T197(0); S199(0); S202(100); S206(0)]	0.913	-0.131313235	0.99184412
J3KNP0	1xPhospho [S315(100); Y325(0)]	0.962	-0.055891201	1
J3KNQ4	2xPhospho [S48(0); S50(0); S54(100); T56(0.1); S59(100); S62(0)]	0.858	-0.220950447	0.979498769
J3KNV1	1xPhospho [S634(0); Y636(0); S637(0); T638(0); S649(100)]	1.211	0.276198865	0.908086456
J3KNW5	1xPhospho [S422(0); S428(100)]	1.422	0.507921465	0.8446011
J3KNX6	2xPhospho [T777(2.2); S778(97.8); S781(100)]	1.256	0.328836464	0.906786159
J3KNX7	2xPhospho [T149(0); S150(0); S152(0.2); S153(0); S154(0); S155(0); S156(0); S157(0); S158(0); S159(3.8); S160(95.8); S162(0.2); S164(100); S171(0); T174(0)]	0.9	-0.152003093	1
J3KNX7	2xPhospho [T149(0); S150(0); S152(0); S153(0); S154(0); S155(0); S156(0); S157(0.2); S158(4.4); S159(4.4); S160(91); S162(0); S164(100); S171(0); T174(0)]	0.912	-0.13289427	1
J3KNZ9	2xPhospho [S179(0.2); S180(0); T183(0.2); S189(99.8); S190(99.8)]	2.169	1.117030053	0.254834652
J3KNZ9	2xPhospho [S219(0); Y224(0); S230(100); S237(100); S244(0); T246(0); S248(0); T249(0); S250(0)]	0.695	-0.524915117	0.953387553
J3KNZ9	3xPhospho [S179(99.8); S180(0.3); T183(0); S189(100); S190(100)]	0.906	-0.142417045	0.986075329
J3KP14	2xPhospho [Y1758(0); T1761(100); S1765(99.9); T1767(0.1)]	0.845	-0.242976753	0.982743754
J3KP19	2xPhospho [S1543(0); S1544(0); S1547(100); S1551(0.1); T1552(95.7); T1554(2.2); S1555(2.2)]	0.274	-1.867752202	0.100651062
J3KP19	3xPhospho [S1618(0); S1624(100); S1626(100); S1628(2.7); S1629(97.4); T1631(0); T1636(0)]	0.297	-1.751465164	0.342410353
J3KP19	2xPhospho [T1367(97.4); S1369(2.6); S1370(0.1); S1372(100); S1377(0)]	0.602	-0.732164608	0.879063032
J3KP30	1xPhospho [S324(0); T329(2.8); S330(97.2); S336(0)]	100	6.64385619	8.35E-17
J3KP30	3xPhospho [S134(0); Y135(0); T136(0); S141(100); S145(100); S148(100); S151(0)]	0.582	-0.780908942	0.612909889
J3KP30	2xPhospho [S134(0); Y135(0); T136(0); S141(100); S145(100); S148(0); S151(0)]	0.589	-0.763660461	0.660888692
J3KP30	2xPhospho [S170(0); T172(0); S175(0); S178(0); T180(0); S184(100); T188(0); S189(48.9); S190(48.9); S191(2.1); S194(0); S196(0); T200(0)]	0.649	-0.623709617	0.688026385
J3KP30	3xPhospho [T129(100); T131(0); S134(0); Y135(0); T136(0); S141(100); S145(100); S148(0); S151(0)]	0.572	-0.805912948	0.797966154
J3KP30	1xPhospho [S117(100); S120(0); S121(0)]	0.54	-0.888968688	0.862999305
J3KP30	2xPhospho [S170(99.7); T172(0.3); S175(0); S178(0); T180(0); S184(100); T188(0); S189(0); S190(0); S191(0); S194(0); S196(0); T200(0)]	0.654	-0.612637459	0.880267055
J3KP30	3xPhospho [T79(0); S82(0); T83(0); T87(50); S88(50); S92(100); Y94(0); S95(100); S97(0); T102(0)]	0.776	-0.365871442	0.934211842
J3KP30	3xPhospho [S170(0); T172(0); S175(0.4); S178(0.4); T180(0); S184(99.1); T188(5.8); S189(88.5); S190(5.8); S191(0); S194(99.6); S196(0.4); T200(0)]	0.688	-0.53951953	0.953220244
J3KP30	1xPhospho [S251(100); S253(0); Y261(0); S270(0)]	1.18	0.23878686	0.953252794
J3KP30	1xPhospho [S349(0); S352(100); S356(0)]	0.986	-0.020340448	0.973263372
J3KP30	2xPhospho [T79(0); S82(5.3); T83(94.7); T87(5.3); S88(94.7); S92(0); Y94(0); S95(0); S97(0); T102(0)]	0.924	-0.114035243	0.973763863
J3KP30	2xPhospho [T129(100); T131(0); S134(0); Y135(0); T136(0); S141(100); S145(0); S148(0); S151(0)]	0.937	-0.093879047	0.994026759
J3KP75	2xPhospho [T398(0); T404(0); S411(96.2); S412(3.8); S414(96.2); S416(3.8); S418(0)]	0.496	-1.011587974	0.76282888
J3KP75	1xPhospho [S70(0); S72(100)]	1.248	0.319617934	0.893580559
J3KP75	1xPhospho [S510(100); T514(0); T515(0)]	1.282	0.358396262	0.91490394
J3KPG5	1xPhospho [S1367(100); T1369(0)]	2.096	1.067638717	0.395415966
J3KPK6	1xPhospho [S308(0); S310(100); S320(0)]	2.083	1.058662839	0.38139799
J3KPL2	1xPhospho [T243(0); S247(100); S254(0)]	1.171	0.227741076	0.934211842
J3KPL2	2xPhospho [S13(100); S17(100); S20(0)]	0.801	-0.320125852	0.969254392
J3KPP7	1xPhospho [T410(0); S412(100)]	1.582	0.6617496	0.704080794
J3KPP7	1xPhospho [T410(0); S412(100)]	0.977	-0.033569533	0.973942845
J3KPV4	2xPhospho [S118(100); S129(93.5); S132(6.5)]	0.646	-0.63039393	0.913059807
J3KPW7	1xPhospho [S488(100)]	1.818	0.8623522	0.524415697
J3KPW7	2xPhospho [S488(100); S501(100)]	1.855	0.891419187	0.52992463
J3KPW7	1xPhospho [S488(100)]	1.327	0.408168371	0.855534442
J3KPW7	2xPhospho [S516(100); S518(100)]	0.789	-0.341902795	0.880267055
J3KPW7	2xPhospho [S516(100); S518(100)]	0.853	-0.229382353	0.965013037
J3KPW7	1xPhospho [S488(100)]	1.135	0.182692298	0.965139891
J3KPW7	2xPhospho [S516(100); S518(100)]	0.988	-0.017417053	1
J3KPW7	2xPhospho [S488(100); S501(100)]	1.009	0.012926174	1
J3KQ69	2xPhospho [S622(4); S626(99.8); T628(96.2); S635(0)]	9.019	3.172967481	0.005010034
J3KQ69	1xPhospho [S622(0); S626(100); T628(0); S635(0)]	2.994	1.582074221	0.181365669
J3KQ69	1xPhospho [S658(0); Y659(0); Y662(0); S665(100); T667(0); T676(0)]	1.356	0.439357178	0.830379242
J3KQ69	3xPhospho [S658(0); Y659(0); Y662(0); S665(100); T667(100); T676(100)]	0.692	-0.531156057	0.836491646
J3KQ69	3xPhospho [S658(0); Y659(0); Y662(0); S665(100); T667(100); T676(100)]	0.709	-0.496142467	0.861558374
J3KQ69	2xPhospho [S658(0); Y659(0); Y662(0); S665(100); T667(100); T676(0)]	1.172	0.22897257	0.920957334
J3KQ69	1xPhospho [S658(0); Y659(0); Y662(0); S665(100); T667(0.1); T676(0)]	1.159	0.212880566	0.95142727
J3KQ69	2xPhospho [S658(0); Y659(0); Y662(0); S665(100); T667(100); T676(0)]	1.013	0.018634174	1
J3KQK0	2xPhospho [S1010(0); S1012(97.8); S1013(2.3); S1015(0); T1020(0); S1021(100); T1024(0)]	1.364	0.447843644	0.855534442
J3KQS2	2xPhospho [S268(100); S269(100); S271(0); S274(0)]	0.589	-0.763660461	0.701261726
J3KQS6	2xPhospho [S44(100); S47(50); S49(50); S57(0); S62(0); T65(0); S66(0)]	0.412	-1.279283757	0.634227881
J3KR59	1xPhospho [Y52(0); S60(100); T62(0); T64(0); S65(0)]	1.325	0.40599236	0.851160627
J3KS33	2xPhospho [S16(96.9); S17(49.3); S18(50.7); S20(3.1)]	0.839	-0.253257284	0.93864152
J3KSG2	2xPhospho [S417(0); S419(100); S422(95.5); S424(4.5); S428(0); T434(0)]	0.627	-0.673462652	0.862506613
J3KSI9	2xPhospho [S10(35.5); S11(35.5); T14(32.3); T17(1.1); Y20(0); S21(94.3); S26(1.1); T27(0.1)]	1.075	0.10433666	0.968707708
J3KSS7	1xPhospho [T83(0); S87(100)]	0.977	-0.033569533	0.981526996
J3KTD2	2xPhospho [T298(0); S301(0); S305(100); S308(0); S309(0.1); S310(99.9); S314(0)]	1.222	0.289244285	0.93019184
J3KTL2	1xPhospho [S199(100); S201(0.1); Y202(0)]	0.102	-3.293358943	0.004839169
J3KTL2	2xPhospho [S199(100); S201(100); Y202(0)]	0.737	-0.440263476	0.815248216
J3QL76	3xPhospho [T62(50); S63(50); S66(0); S76(100); S79(0.4); S81(99.6); S88(0); S89(0)]	0.682	-0.552156356	0.91490394

J3QLH3	3xPhospho [S9(0); S10(0); Y14(0); S18(100); S22(100); S33(100)]	0.01	-6.64385619	8.35E-17
J3QLH3	1xPhospho [S113(100)]	1.996	0.997111721	0.539112137
J3QLH3	3xPhospho [Y61(0); S69(100); S72(100); S77(92.5); T79(7.5); T91(0)]	1.33	0.411426246	0.801897757
J3QLH3	2xPhospho [S9(0); S10(0); Y14(0); S18(100); S22(100); S33(0)]	1.355	0.438292852	0.858930256
J3QLH3	3xPhospho [Y61(0); S69(100); S72(100); S77(93.5); T79(6.5)]	1.357	0.440420721	0.867705101
J3QLH3	2xPhospho [S9(0); S10(0); Y14(0); S18(100); S22(100); S33(0)]	1.265	0.339137385	0.87998572
J3QLH3	2xPhospho [Y61(0); S69(5.4); S72(94.6); S77(94.6); T79(5.4)]	1.217	0.283329168	0.884599031
J3QLH3	2xPhospho [Y61(0); S69(6.1); S72(93.9); S77(6.1); T79(93.9); T91(0)]	1.275	0.350497247	0.884991416
J3QLH3	3xPhospho [S9(0); S10(0); Y14(0); S18(100); S22(100); S33(100)]	1.078	0.108357178	0.971838536
J3QLL0	1xPhospho [S54(0); S55(0); T61(0); S62(100)]	1.427	0.512985335	0.714293599
J3QQM5	1xPhospho [S27(0); S29(100); S31(0)]	1.106	0.145351386	0.93546001
J3QQQ9	1xPhospho [S23(100)]	2.566	1.35952117	0.187336464
J3QQY0	1xPhospho [S362(100); S371(0); S373(0); T375(0)]	1.05	0.070389328	0.980895643
J3QQY7	2xPhospho [S57(100); S60(3.5); S61(96.6); T65(0); S68(0); S70(0)]	0.662	-0.595096878	0.749797187
J3QR07	1xPhospho [S416(3.9); S417(88.3); S419(3.9); S424(3.9); S435(0)]	100	6.64385619	8.35E-17
J3QR07	1xPhospho [S308(100)]	1.578	0.658097205	0.763548117
J3QR07	1xPhospho [S71(0); S75(0); S76(2); S77(98); S79(0)]	1.284	0.360645202	0.907438338
J3QR65	1xPhospho [T570(0); S571(0); S572(100)]	3.612	1.852797893	0.054913274
J3QR65	1xPhospho [S904(100); S908(0); S909(0); S911(0); T916(0)]	2.003	1.002162421	0.749797187
J3QRU1	3xPhospho [Y21(0); T26(0); S31(4.5); T32(4.5); S33(90.8); S35(0.2); Y37(0); T42(0.2); T43(4.7); S45(95.1); S49(95.1); S50(4.7); S51(0.2)]	0.377	-1.407363571	0.599468013
J3QRU1	2xPhospho [Y21(0); T26(0); S31(0); T32(0); S33(0); S35(0); Y37(0); T42(4.2); T43(4.2); S45(91.6); S49(95.4); S50(4.4); S51(0.2)]	0.704	-0.506352666	0.878370497
J3QRU8	3xPhospho [S373(0); S375(100); Y383(0); S385(100); S388(100); T392(0)]	0.262	-1.932361283	0.072158634
J3QRU8	2xPhospho [S592(100); S596(100); Y598(0); T601(0); S603(0)]	0.429	-1.220950447	0.100427249
J3QRU8	2xPhospho [S373(0); S375(0); Y383(0); S385(100); S388(100); T392(0)]	0.432	-1.210896782	0.213792548
J3QRU8	1xPhospho [S359(0); S361(0); S362(1.3); T364(98.7); S370(0)]	0.672	-0.573466862	0.847652956
J3QRU8	2xPhospho [S410(100); S413(100); S414(0); S417(0); T422(0); Y426(0)]	0.749	-0.416962376	0.94813191
J3QRU8	1xPhospho [S592(100); S596(0); Y598(0); T601(0); S603(0)]	0.83	-0.268816758	0.965139891
J3QRV5	1xPhospho [S961(0); T963(0); S965(100)]	1.741	0.799916203	0.602499568
J3QRV5	2xPhospho [S961(100); T963(97.8); S965(2.2)]	1.083	0.115033243	0.96293109
J3QS11	2xPhospho [Y171(0); S172(0); T173(0); T175(0); S176(50); S178(50); S179(100); S182(0)]	0.01	-6.64385619	8.35E-17
J3QS11	2xPhospho [S87(0); S89(100); S91(99.9); S95(0.1); S97(0); T100(0)]	0.01	-6.64385619	8.35E-17
J3QS11	3xPhospho [Y153(0); S155(100); T157(50); S159(50); S162(100); S164(0); T165(0); T166(0)]	0.01	-6.64385619	8.35E-17
J3QS11	2xPhospho [S115(100); T116(1.8); S118(98.3)]	0.01	-6.64385619	8.35E-17
J3QS11	2xPhospho [S115(50.3); T116(50.3); S118(99.3)]	0.191	-2.388355457	0.000766148
J3QS11	3xPhospho [Y171(0); S172(0); T173(0); T175(0.4); S176(99.2); S178(6.1); S179(94.3); S182(100); T185(0); T191(0); T193(0)]	0.224	-2.158429363	0.126580166
J3QS11	1xPhospho [S87(97.9); S89(2.1); S91(0); S95(0); S97(0); T100(0)]	0.589	-0.763660461	0.797966154
J3QSD7	3xPhospho [S341(100); S343(100); S345(100)]	0.543	-0.880975897	0.807956501
J3QSD7	3xPhospho [S341(100); S343(100); S345(100)]	0.572	-0.805912948	0.867320208
J3QSW1	1xPhospho [S664(99.8); T669(0.1); S670(0.1); T687(0)]	100	6.64385619	8.35E-17
J3QSX6	2xPhospho [T141(0); S143(100); T145(0); S147(100); Y151(0)]	0.687	-0.541617996	0.860444802
J3QSX6	2xPhospho [T63(50); S64(50); S65(0); S67(0); Y69(0); S70(0); S74(0); S75(0); S79(100); T83(0); Y85(0)]	0.709	-0.496142467	0.862506613
J3QSX6	1xPhospho [T141(0); S143(100); T145(0); S147(0); Y151(0)]	0.631	-0.66428809	0.862820654
J3QSX6	1xPhospho [S162(0); T163(0); S164(100); S167(0); S170(0); Y173(0); S174(0)]	0.925	-0.112474729	0.989898705
J3QT22	1xPhospho [S15(0); S22(0); S25(100); S27(0)]	1.464	0.549915554	0.738872836
J9JID9	1xPhospho [T314(0); S322(0); S323(0); S331(100)]	100	6.64385619	8.35E-17
K7EIG1	1xPhospho [S586(0); S596(100); S601(0); S602(0); S604(0)]	100	6.64385619	8.35E-17
K7EIU8	1xPhospho [S138(0); S144(0); T147(0); S150(0); S154(2.9); S155(97.1)]	100	6.64385619	8.35E-17
K7EJF0	2xPhospho [S173(100); S180(0); S188(100)]	0.08	-3.64385619	0.000451211
K7EJF0	2xPhospho [T35(0); S40(100); S44(100)]	2.103	1.07244885	0.432707567
K7EJL1	2xPhospho [T72(0); T80(100); T82(100); S86(0)]	0.412	-1.279283757	0.318513278
K7EJV9	1xPhospho [T35(2.9); S36(94.1); T38(2.9)]	0.937	-0.093879047	0.984970017
K7EK22	1xPhospho [S112(0); S114(0); S115(0); S121(100)]	100	6.64385619	8.35E-17
K7EK22	1xPhospho [S173(100); S178(0); S187(0)]	1.541	0.623866862	0.742093927
K7EK22	2xPhospho [S914(100); S915(100); Y920(0); T926(0)]	1.38	0.464668267	0.822894134
K7EK22	2xPhospho [S112(49); S114(2); S115(49); S121(100)]	1.287	0.364012054	0.883306273
K7EK53	1xPhospho [S122(0); S124(0); S125(0); S129(0.1); S130(99.9)]	0.667	-0.584241333	0.893481203
K7EL20	2xPhospho [T38(98.4); T41(50.8); S42(50.8)]	0.627	-0.673462652	0.701261726
K7EL20	1xPhospho [T38(0.1); T41(2.7); S42(97.2)]	0.783	-0.352915787	0.933107775
K7ELG9	1xPhospho [T73(0); T75(100); S81(0); S85(0)]	1.297	0.37517848	0.886912356
K7ELR1	1xPhospho [S349(0); T350(0); S351(0); T352(0); S354(0); T355(0); T364(0); S368(0); S370(0); S371(0); S373(96.4); T375(3.6)]	1.186	0.24610401	0.93019184
K7EM16	1xPhospho [S91(0.1); S92(2.4); S93(97.4); S94(0.1); T96(0.1); T97(0); S98(0); T100(0); T104(0); S106(0); S107(0); S108(0); Y110(0); S111(0)]	1.327	0.408168371	0.87998572
K7EM16	2xPhospho [S91(99.8); S92(4.1); S93(92.2); S94(3.9); T96(0); T97(0); S98(0); T100(0); T104(0); S106(0); S107(0); S108(0); Y110(0); S111(0)]	0.807	-0.309359421	0.980895643
K7EM16	2xPhospho [S91(50.9); S92(50.9); S93(94.2); S94(3.9); T96(0); T97(0); S98(0); T100(0); T104(0); S106(0); S107(0); S108(0); Y110(0); S111(0)]	0.914	-0.12973393	0.986075329
K7EM32	2xPhospho [S30(100); S36(0.7); S37(0.7); S38(0.1); S41(0.7); S45(0.7); S46(0.7); Y53(0.7); T58(96); Y63(0); T64(0); S71(0)]	0.01	-6.64385619	8.35E-17
K7EMU1	1xPhospho [T328(0); S333(100); S337(0); S339(0); S343(0)]	100	6.64385619	8.35E-17

K7EMU1	2xPhospho [T328(0); S333(100); S337(0); S339(100); S343(0)]	0.458	-1.126580497	0.548192147
K7EMU1	1xPhospho [S333(0); S337(0); S339(100); S343(0)]	1.211	0.276198865	0.933760225
K7EMU1	2xPhospho [T328(0); S333(100); S337(0); S339(99.9); S343(0.1)]	0.686	-0.543719518	0.938673385
K7EMU1	2xPhospho [S284(96.3); S286(3.7); T293(0); S294(0); Y299(0); Y300(0); T301(0); S302(0); S304(0); S305(96.2); T307(3.7); S308(0.1); S309(0); S310(0)]	1.175	0.232660757	0.94797789
K7EMU1	2xPhospho [T328(0); S333(100); S337(0); S339(100); S343(0)]	0.815	-0.295128036	0.961452497
K7EMU1	2xPhospho [T328(0); S333(100); S337(0); S339(100); S343(0)]	0.846	-0.241270432	0.965248196
K7EMU1	1xPhospho [T314(0); S322(0); S323(100)]	1.018	0.025737561	0.984145373
K7ENG3	1xPhospho [S321(0); S327(100)]	1.383	0.467801156	0.776177942
K7ENG3	1xPhospho [S316(0); S321(0); S327(100)]	1.061	0.085424656	0.980895643
K7ENG3	2xPhospho [S316(0); S321(100); S327(100)]	1.021	0.029982866	0.983092362
K7EPF0	2xPhospho [S35(100); S38(100); T52(0)]	0.1	-3.321928095	0.002616656
K7EPZ6	3xPhospho [S233(0); S236(100); S238(100); S244(100); Y248(0)]	0.01	-6.64385619	8.35E-17
K7EPZ6	2xPhospho [S233(0); S236(100); S238(97.6); S244(2.4); Y248(0)]	0.427	-1.227692025	0.49866048
K7EPZ6	1xPhospho [S196(0); S198(100); T202(0)]	1.789	0.839153387	0.573146021
K7EQ21	1xPhospho [S142(0); S143(0); T150(0); S152(100)]	1.291	0.368489001	0.861278237
K7EQ21	2xPhospho [S136(96); S138(6.1); S142(49); S143(49); T150(0); S152(0)]	1.216	0.282143229	0.925724009
K7EQI9	3xPhospho [T210(0); T213(100); S216(100); S223(50); S228(50)]	0.531	-0.913216234	0.584517452
K7EQI9	3xPhospho [S161(100); S163(100); S177(7.3); T178(92.7)]	1.539	0.621993232	0.797966154
K7EQI9	2xPhospho [S161(100); S163(100); S177(0); T178(0)]	0.763	-0.390245038	0.856079731
K7EQI9	2xPhospho [T210(0); T213(0); S216(100); S223(100); S228(0)]	0.794	-0.332789088	0.907368665
K7EQI9	2xPhospho [S161(100); S163(100); S177(0); T178(0)]	0.768	-0.380821784	0.933760225
K7EQI9	3xPhospho [T186(100); S188(100); S191(100); S196(0); T198(0); T202(0); S206(0)]	0.758	-0.399730246	0.973763863
K7EQL6	2xPhospho [S132(1.1); S136(9.7); T137(88.2); S139(9.8); S142(81.3); T144(8.9); S147(1); Y151(0); S152(0); T154(0)]	0.348	-1.522840789	0.431663411
K7EQL6	2xPhospho [S1031(0); S1033(0); S1034(0); S1036(0); S1041(50); S1042(50); S1046(100); S1048(0); S1049(0); S1050(0)]	1.249	0.320773477	0.907438338
K7EQL6	3xPhospho [S1031(93.1); S1033(6.8); S1034(0); S1036(0); S1041(93.6); S1042(12.8); S1046(93.6); S1048(0); S1049(0); S1050(0)]	0.901	-0.150400989	0.99811985
K7EQN8	1xPhospho [S31(100); T32(0); Y36(0)]	0.954	-0.067938829	0.981858798
K7EQP1	1xPhospho [T61(0); S73(0); S80(95.9); S88(4.1)]	100	6.64385619	8.35E-17
K7ER53	2xPhospho [Y277(0); S278(100); S282(99.9); T284(0.1); S286(0); S290(0)]	0.279	-1.841662973	0.160901297
K7ERG7	2xPhospho [S73(100); S75(100); S91(0)]	0.866	-0.20756107	0.99184412
K7ERG7	2xPhospho [S73(100); S75(100); S91(0)]	0.905	-0.144010303	0.994865845
K7ERS5	2xPhospho [S164(6.5); S168(93.5); T177(0); S183(0); S188(0); S190(6.5); T191(93.5); Y194(0)]	100	6.64385619	8.35E-17
K7ERS5	2xPhospho [S121(98.1); S123(51); S124(51); S136(0); T139(0); S141(0)]	1.356	0.439357178	0.857906468
K7ERT9	1xPhospho [S19(93.9); S23(3); S24(3); S31(0.1); S32(0); S36(0); S39(0)]	1.536	0.619178216	0.74242706
K7ESA4	2xPhospho [T42(0); S44(0); T51(99.9); T53(50.1); T55(50.1)]	0.878	-0.187707155	0.980895643
K7ESE3	2xPhospho [S123(99.8); S125(0); S128(0.2); T131(0); T132(0); S133(0); S136(99.8); S138(0.2); S140(0); S143(0); S144(0); S146(0); S147(0)]	1.889	0.917622702	0.395524514
K7ESE3	1xPhospho [S123(0); S125(0); S128(0); T131(0); T132(0); S133(0); S136(100); S138(0); S140(0); S143(0); S144(0); S146(0); S147(0)]	1.107	0.146655222	0.97726974
K7ESE9	2xPhospho [S251(100); S257(0); S258(0.1); S259(99.9); S261(0); S263(0)]	0.729	-0.45600928	0.90089653
O00139	1xPhospho [S121(0); S127(0); S128(0); S129(0); S135(97.2); S137(2.8); S140(0)]	0.759	-0.397828209	0.96293109
O00161	1xPhospho [T101(0); T102(0); S110(100); S116(0)]	0.982	-0.02620507	0.980895643
O00178	1xPhospho [T576(0); T577(0); S580(97.6); S584(2.4)]	1.764	0.818850561	0.566110553
O00178	2xPhospho [S8(0.1); S12(99.9); S17(0); S24(0.1); S25(99.9)]	1.439	0.525066592	0.786602611
O00178	2xPhospho [S44(100); S47(100); T62(0); S63(0)]	0.743	-0.428565884	0.825221762
O00193	1xPhospho [S82(0); Y84(0); S87(100); S90(0)]	1.319	0.399444565	0.878394132
O00193	2xPhospho [S15(100); S17(100); S24(0); S25(0)]	1.266	0.340277405	0.908086456
O00193	1xPhospho [S82(0); Y84(0); S87(100); S90(0)]	1.206	0.270229907	0.938029982
O00193	1xPhospho [S15(0.1); S17(100); S24(0); S25(0)]	1.156	0.209141398	0.94797789
O00193	1xPhospho [S15(0); S17(100); S24(0); S25(0)]	1.107	0.146655222	0.972294713
O00267	2xPhospho [S32(100); S36(100); Y54(0)]	2.901	1.536550296	0.174097537
O00267	1xPhospho [T768(0); Y771(0); S773(100); T775(0); Y778(0); S780(0); S782(0)]	1.595	0.673556424	0.679191074
O00267	2xPhospho [S32(100); S36(100); Y54(0)]	0.714	-0.486004021	0.854283637
O00267	1xPhospho [T784(0); Y787(0); S789(100); T791(0); S797(0)]	1.309	0.388465097	0.886912356
O00267	1xPhospho [T655(0); T658(0); S666(100)]	1.067	0.093560176	0.965013037
O00267	1xPhospho [T799(0); Y802(0); S804(100); T806(0); S812(0)]	1.035	0.049630768	0.973263372
O00267	2xPhospho [T799(100); Y802(0); S804(99.9); T806(0.1); S812(0)]	0.974	-0.038006323	0.979498769
O00400	1xPhospho [S20(0); S22(100)]	1.512	0.59645814	0.771836185
O00401	2xPhospho [S484(100); S485(100)]	0.529	-0.918660373	0.724583498
O00401	1xPhospho [Y256(100)]	0.732	-0.450084446	0.971196575
O00418	3xPhospho [Y69(0); S70(0); S71(50); S72(50); S74(100); S78(100)]	0.01	-6.64385619	8.35E-17
O00418	1xPhospho [S435(96.5); S438(3.5); S441(0); Y443(0); S445(0)]	100	6.64385619	8.35E-17
O00418	3xPhospho [S345(100); T348(100); T353(100)]	0.262	-1.932361283	0.02174739
O00418	2xPhospho [Y69(0); S70(0); S71(0.1); S72(100); S74(100); S78(0)]	0.289	-1.790858602	0.129503493
O00418	2xPhospho [S27(100); S31(100); Y37(0); T43(0); S47(0); S48(0); S54(0)]	0.36	-1.473931188	0.141559953
O00418	2xPhospho [S435(100); S438(0); S441(0); Y443(0); S445(100)]	0.519	-0.946193556	0.699388119
O00458	2xPhospho [T9(87.9); S14(87.9); S15(12.1); S21(12.1); T27(0); T30(0)]	0.101	-3.307572802	0.002767569
O00458	2xPhospho [T9(100); S14(93.7); S15(3.1); S21(3.1); T27(0); T30(0)]	2.274	1.185232254	0.346209255
O00499	1xPhospho [S296(0); S298(100); S303(0); T307(0)]	1.804	0.851199339	0.597444241
O00499	1xPhospho [S296(97.6); S298(2.4); S303(0); T307(0)]	1.662	0.732920382	0.679191074
O00499	2xPhospho [S296(100); S298(0); S303(100); T307(0)]	1.494	0.579180148	0.774421636

O00499	3xPhospho [S296(100); S298(100); S303(100); T307(0)]	1.359	0.442545456	0.831968889
O00505	1xPhospho [S56(0); S60(100)]	0.726	-0.461958547	0.962617576
O00505	2xPhospho [S56(100); S60(100)]	0.694	-0.526992432	0.96293109
O00505	1xPhospho [S56(0); S60(100)]	0.979	-0.030619235	0.998448514
O00512	1xPhospho [S133(0); S136(100); T139(0); S142(0); T144(0); S146(0); T149(0)]	100	6.64385619	8.35E-17
O00512	1xPhospho [T115(100); S120(0); S127(0)]	0.812	-0.300448367	0.980895643
O00512	1xPhospho [S687(98.6); S689(1.4)]	0.86	-0.217591435	0.999263007
O00515	1xPhospho [S38(0); S39(0); T40(0); T41(0); S49(100)]	0.01	-6.64385619	8.35E-17
O00515	2xPhospho [S121(100); S123(100); T128(0); S134(0)]	1.901	0.926758532	0.457433758
O00515	1xPhospho [S269(0); S272(100); T274(0)]	1.391	0.47612242	0.851160627
O00515	1xPhospho [S177(100)]	1.256	0.328836464	0.879063032
O00515	1xPhospho [S121(0); S123(100); T128(0); S134(0)]	0.878	-0.187707155	0.980895643
O00515	1xPhospho [S121(100); S123(0); T128(0); S134(0)]	1.037	0.052415894	0.981858798
O00534	1xPhospho [S626(0); S634(87.4); S639(6.3); S641(6.3)]	0.79	-0.340075442	0.967144405
O00567	2xPhospho [S519(100); S520(100); T525(0); S528(0); T529(0); S530(0)]	0.384	-1.380821784	0.046704498
O00567	2xPhospho [S519(100); S520(100); T525(0); S528(0); T529(0); S530(0)]	0.496	-1.011587974	0.467067441
O00567	3xPhospho [S511(100); S513(0); S519(100); S520(100); T525(0); S528(0); T529(0); S530(0)]	0.486	-1.040971781	0.505417445
O00567	2xPhospho [S563(0); S569(100); S570(100)]	0.508	-0.977099598	0.531171581
O00567	3xPhospho [S511(100); S513(0); S519(100); S520(100); T525(0); S528(0); T529(0); S530(0)]	0.572	-0.805912948	0.604774389
O00567	2xPhospho [S511(0); S513(0); S519(100); S520(100); T525(0); S528(0); T529(0); S530(0)]	0.554	-0.852042119	0.655090632
O00567	2xPhospho [S461(0); S462(3.9); S465(3.9); S466(92.2); S467(99.8); T468(0.2); S476(0)]	0.628	-0.671163536	0.701641709
O00567	2xPhospho [S511(99.9); S513(0.1); S519(0); S520(100); T525(0); S528(0); T529(0); S530(0)]	0.669	-0.579921884	0.771363293
O00567	1xPhospho [S500(97); S502(3); S504(0)]	1.276	0.351628329	0.879063032
O00567	2xPhospho [S511(100); S513(0); S519(0.1); S520(99.9); T525(0); S528(0); T529(0); S530(0)]	0.758	-0.399730246	0.93019184
O00567	2xPhospho [S569(100); S570(100)]	0.839	-0.253257284	0.950730609
O00567	1xPhospho [S314(100); T316(0)]	1.006	0.008630305	0.980895643
O00567	1xPhospho [S519(0); S520(100); T525(0); S528(0); T529(0); S530(0)]	1.021	0.029982866	0.980895643
O00567	1xPhospho [S537(100); T538(0); T543(0)]	0.926	-0.110915901	0.989554868
O00567	3xPhospho [S563(100); S569(100); S570(100)]	0.835	-0.260151897	0.993670231
O00571	1xPhospho [Y69(0); S70(0); S71(100); S74(0)]	1.984	0.988412026	0.378265448
O00571	1xPhospho [S82(100); S83(0); S86(0)]	2.092	1.064882852	0.469342919
O00571	1xMet-loss+Acetyl [N-Term]; 1xPhospho [S2(0); S23(0); S24(0); S28(0); S31(97.7); T32(2.3); S34(0)]	1.536	0.619178216	0.638149561
O00571	2xPhospho [S82(100); S83(100); S86(0)]	1.521	0.605020153	0.742559804
O00571	2xPhospho [S57(0.8); S58(24.8); S61(24.8); S62(24.8); S63(24.8); Y69(0); S70(0.1); S71(99.8); S74(0.1)]	1.085	0.117695043	0.96293109
O00571	1xPhospho [S594(100)]	1.164	0.219091058	0.963131675
O00623	3xPhospho [S275(99.9); T277(99); T281(1.1); Y290(0); S292(50); S294(50)]	2.505	1.324810603	0.070022301
O00629	1xPhospho [S60(100); Y66(0)]	1.571	0.651683181	0.703341962
O00762	1xMet-loss+Acetyl [N-Term]; 1xPhospho [S3(100); T11(0); S12(0)]	1.353	0.436161839	0.855445086
O14497	2xPhospho [S696(100); S698(0); S702(99.9); S705(0.1); S709(0)]	0.01	-6.64385619	8.35E-17
O14497	1xPhospho [S79(100); S90(0)]	0.01	-6.64385619	8.35E-17
O14497	1xPhospho [S696(100); S698(0); S702(0); S705(0); S709(0)]	0.01	-6.64385619	8.35E-17
O14497	2xPhospho [T1599(50); S1600(50); S1602(0.1); S1604(100); S1609(0)]	0.331	-1.595096878	0.112574394
O14497	1xPhospho [S1182(0); S1184(100); S1195(0); S1197(0); T1198(0)]	0.422	-1.244685096	0.273463684
O14497	2xPhospho [S357(0); S363(100); S366(100); S367(0)]	0.397	-1.332789088	0.465165189
O14497	1xPhospho [T1743(0); S1751(100)]	0.998	-0.002888279	0.974924677
O14497	1xPhospho [S357(0); S363(0); S366(100); S367(0)]	0.889	-0.169744676	0.97998947
O14523	1xPhospho [S660(2.8); S662(97.2); S667(0); T670(0); T672(0); S674(0)]	0.671	-0.575615328	0.853943017
O14523	2xPhospho [S464(100); S466(0); S468(100); S470(0); T475(0)]	1.198	0.260627908	0.94813191
O14545	1xPhospho [S468(0); T469(0); S470(100); S479(0); S480(0)]	1.744	0.80240004	0.590720204
O14545	1xPhospho [S409(0); T414(0); S415(100)]	1.094	0.129612738	0.969482098
O14617	3xPhospho [S632(100); S634(100); S636(100)]	0.668	-0.582079992	0.694333278
O14617	1xPhospho [T824(0); T826(0); S827(0); S829(100)]	1.07	0.097610797	0.967238018
O14617	1xPhospho [S658(100)]	0.91	-0.13606155	1
O14640	2xPhospho [S600(100); S602(0); S604(100); T607(0); S610(0); S614(0); S615(0)]	0.65	-0.621488377	0.900153261
O14641	2xPhospho [Y192(0); S194(3.2); S195(93.7); S196(3.2); T197(0); T200(0); S201(0); S205(0); T206(0); S207(0); S211(100); T216(0); S218(0)]	2.404	1.265436896	0.244965218
O14646	2 1xPhospho [S1688(100); S1693(0)]	2.268	1.18142064	0.384697169
O14646	2 2xPhospho [S1683(0); Y1684(0); S1686(100); S1688(100); S1693(0)]	1.141	0.190298792	0.929390092
O14647	2xPhospho [S231(0); Y232(0); T240(100); S242(99.7); T249(0.3); S260(0); T262(0)]	0.01	-6.64385619	8.35E-17
O14647	3xPhospho [S1795(100); S1799(97); S1801(3); S1807(100)]	100	6.64385619	8.35E-17
O14647	2xPhospho [S1789(0); S1792(0); S1795(100); S1799(0.1); S1801(100)]	3.331	1.735955354	0.251099621
O14647	2xPhospho [S1795(0); S1799(0); S1801(100); S1807(100)]	2.123	1.086104371	0.457047036
O14681	2xPhospho [S326(100); S330(100)]	1.305	0.384049807	0.879063032
O14681	1xPhospho [S326(100); S330(0)]	1.116	0.158337027	0.933452347
O14681	2xPhospho [T308(0); Y310(0); S313(0); S316(0); S317(0); S318(0); T319(0); S320(0); S326(100); S330(100)]	1.091	0.125651102	0.973263372
O14745	1xPhospho [S280(100)]	0.632	-0.662003536	0.851160627
O14745	1xPhospho [S288(0); S290(100); S291(0); T293(0); S294(0); S299(0); S302(0)]	1.303	0.381837084	0.879063032
O14745	2xPhospho [S269(100); S280(100)]	0.806	-0.311148256	0.939608401
O14757	2xPhospho [S296(100); S301(0); S305(0); S307(0); S308(100)]	1.013	0.018634174	1
O14924	2xPhospho [S661(100); S667(0); T669(4.8); S671(90.5); T676(4.8)]	0.01	-6.64385619	8.35E-17
O14929	1xPhospho [S361(100); Y363(0)]	100	6.64385619	8.35E-17

O14974	1xPhospho [S299(100); S304(0); T305(0); S313(0)]	0.01	-6.64385619	8.35E-17
O14974	2xPhospho [S852(0); T853(0); S856(0); T859(0); S862(100); S871(99.7); T873(0.3); S877(0)]	0.494	-1.017417053	0.511981566
O14974	2xPhospho [S852(0); T853(0); S856(0.4); T859(0.4); S862(99.3); S871(99.6); T873(0.4); S877(0)]	0.58	-0.785875195	0.593977425
O14974	2xPhospho [S695(100); T696(100); T700(0); T702(0)]	1.456	0.542010356	0.804811993
O14974	1xPhospho [T443(0); S445(100); Y446(0); T453(0); S455(0)]	0.634	-0.657445255	0.869295771
O14974	1xPhospho [S299(100); S304(0); T305(0); S313(0)]	0.858	-0.220950447	0.965013037
O14974	1xPhospho [S695(0); T696(100); T700(0); T702(0)]	1.008	0.011495639	0.966759019
O14974	1xPhospho [S299(100); S304(0); T305(0); S313(0)]	0.734	-0.446148032	0.967050102
O14976	3xPhospho [S815(0); S817(0); S826(100); S829(100); S834(100); S837(0); S838(0)]	0.01	-6.64385619	8.35E-17
O14976	2xPhospho [S815(0); S817(0); S826(100); S829(100); S834(0); S837(0); S838(0)]	0.646	-0.63039393	0.879426164
O14976	2xPhospho [S815(0); S817(0); S826(100); S829(100); S834(0); S837(0); S838(0)]	0.811	-0.30222618	0.920518605
O15013	3xPhospho [S1279(0); S1282(0.7); S1283(93.7); S1284(93.7); S1285(15.4); S1287(94.1); S1289(0.1); S1291(0.7); S1294(0.4); S1295(0.4); S1296(0.8)]	0.152	-2.717856771	0.038159644
O15013	2xPhospho [S1279(0); S1282(0); S1283(0.1); S1284(96.5); S1285(3.4); S1287(100); S1289(0); S1291(0); S1294(0); S1295(0); S1296(0)]	0.485	-1.043943348	0.229123164
O15013	1xPhospho [Y42(0); T44(0); S59(100)]	0.532	-0.910501849	0.432935188
O15014	1xPhospho [S413(0); S416(0); S417(0); S424(0); S425(0); T426(0); S427(0); S433(99.9); S435(0.1); S436(0)]	15.037	3.910444862	8.50E-05
O15014	1xPhospho [S413(99.8); S416(0.1); S417(0.1); S424(0); S425(0); T426(0); S427(0)]	14.266	3.834508974	0.000369348
O15014	1xPhospho [S446(0); S452(98); S453(2); S454(0); S457(0)]	2.318	1.212880566	0.393266291
O15014	3xPhospho [S413(33.3); S416(33.3); S417(33.3); S424(2); S425(32.7); T426(32.7); S427(32.7); S433(93.9); S435(5.7); S436(0.4)]	0.53	-0.915935735	0.813170869
O15014	1xPhospho [S491(100)]	0.775	-0.367731785	0.969482098
O15014	2xPhospho [S413(99.6); S416(0.2); S417(0.2); S424(0); S425(0); T426(0); S427(0); S433(99.8); S435(0.2); S436(0)]	1.07	0.097610797	0.979697817
O15014	2xPhospho [S358(100); T360(2.2); S361(97.8)]	0.903	-0.147202107	0.999714477
O15020	1xPhospho [S2161(0); S2162(0); S2171(100)]	0.274	-1.867752202	0.094720436
O15040	2xPhospho [S448(100); S449(100)]	0.49	-1.029146346	0.617226186
O15042	1xPhospho [Y478(0); S479(0); S485(100); T487(0)]	100	6.64385619	8.35E-17
O15042	1xPhospho [S18(0); S19(0); S23(2.5); S24(92.6); S26(2.5); S27(2.5); S34(0); S37(0); S39(0); S43(0)]	10.082	3.333709954	0.002055084
O15042	1xPhospho [S18(0); S19(0); S23(94.4); S24(2.8); S26(0.1); S27(2.8); S34(0); S37(0); S39(0); S43(0)]	0.534	-0.905088353	0.825221762
O15042	1xPhospho [S174(100); S175(0); S181(0); T187(0)]	0.785	-0.349235441	0.894595581
O15042	2xPhospho [S174(95.2); S175(95.2); S181(9.7); T187(0)]	0.706	-0.502259911	0.925146414
O15042	2xPhospho [S788(100); S800(100); T806(0); S808(0); S809(0)]	0.955	-0.066427362	1
O15047	2xPhospho [S504(0.2); S508(100); T510(99.8); S517(0); S518(0)]	100	6.64385619	8.35E-17
O15047	2xPhospho [S468(100); S470(100); T475(0); T476(0); S479(0); S486(0); S487(0); S490(0)]	0.57	-0.810966176	0.79670828
O15047	2xPhospho [T526(0); S528(0); S532(5.3); S534(89.3); T540(5.3); T554(0); S556(0); T562(0.4); S565(99.6)]	0.846	-0.241270432	0.965248196
O15047	1xPhospho [S468(97.8); S470(2.2); T475(0); T476(0); S479(0); S486(0); S487(0); S490(0)]	0.792	-0.336427665	0.969482098
O15055	2xPhospho [T625(0); S627(0); S639(100); S643(100)]	1.537	0.620117165	0.788815962
O15063	2xPhospho [S945(49.9); S946(49.9); T951(0.2); S952(0); T953(0); S956(100); T961(0); T962(0)]	0.01	-6.64385619	8.35E-17
O15067	1xPhospho [S569(100); T576(0); S579(0)]	1.261	0.334568276	0.883522037
O15075	2xPhospho [S330(0); S332(0); S334(0); T336(97.6); S337(97.6); S340(4.8)]	0.01	-6.64385619	8.35E-17
O15085	2xPhospho [S663(100); T668(99.9); T672(0.1)]	0.16	-2.64385619	0.023762557
O15085	3xPhospho [S242(0); S245(100); S251(100); T254(50); S255(50)]	0.45	-1.152003093	0.556645393
O15085	1xPhospho [S633(0); S635(100); T646(0)]	1.373	0.457331625	0.873058511
O15085	2xPhospho [S242(0); S245(0); S251(100); T254(100); S255(0)]	0.742	-0.430508908	0.891862526
O15085	2xPhospho [S545(2.8); S546(2.8); S547(94.4); S549(0.1); T550(0); S556(100)]	0.69	-0.535331733	0.908083211
O15085	2xPhospho [S242(0); S245(0); S251(99.9); T254(50); S255(50)]	0.598	-0.74178261	0.91281798
O15085	2xPhospho [T1475(4.9); S1478(99.8); S1480(95.4); S1489(0); S1491(0)]	0.702	-0.510457064	0.925808485
O15085	2xPhospho [S1452(0); S1457(0); S1458(100); T1461(2.8); T1462(97.2); S1466(0); T1469(0)]	0.799	-0.323732592	0.954539739
O15085	2xPhospho [Y200(0); T203(0.7); S204(98.6); S208(0.7); S213(0.1); S216(99.9); S221(0.1); S225(0); T227(0)]	0.988	-0.017417053	0.971220368
O15119	2xPhospho [S371(100); S375(100); S381(0)]	1.47	0.555816155	0.786760931
O15119	2xPhospho [S432(100); S435(0.2); S438(99.9); T441(0); S443(0); S444(0); S445(0); T446(0)]	1.492	0.577247536	0.831039794
O15143	1xPhospho [S310(2.5); S311(97.5); T315(0); S323(0)]	1.072	0.100304906	0.972363997
O15169	2xPhospho [Y578(0); S579(50); S581(50); S589(0); S595(100)]	100	6.64385619	8.35E-17
O15169	2xPhospho [T481(100); S486(100)]	1.464	0.549915554	0.790665834
O15173	2xPhospho [S208(100); Y210(0); T211(100); T216(0)]	0.547	-0.870387262	0.376214997
O15173	2xPhospho [S208(100); Y210(0); T211(100); T216(0)]	0.537	-0.897006007	0.597123729
O15173	1xPhospho [S208(0); Y210(0); T211(100); T216(0)]	0.571	-0.808437349	0.670368081
O15173	1xPhospho [S208(0); Y210(0); T211(100); T216(0)]	0.897	-0.15682011	0.965248196
O15173	1xPhospho [S208(0); Y210(0); T211(100); T216(0)]	0.751	-0.413115187	0.973599548
O15198	2xPhospho [T454(0); S458(0); S464(0.4); S465(99.8); S467(99.8)]	1.015	0.021479727	0.979498769
O15231	2xPhospho [S152(2.3); S153(95.4); T154(2.3); S155(0.1); T158(100); S170(0); S171(0)]	0.485	-1.043943348	0.636316981
O15231	2xPhospho [S152(2.4); S153(97.5); T154(0.1); S155(0); T158(100); S170(0); S171(0)]	0.629	-0.668868078	0.753025546
O15231	2xPhospho [T63(5.2); S64(94.8); S66(50); S67(50); S78(0); T79(0)]	0.63	-0.666576266	0.921519407
O15231	1xPhospho [T63(0.1); S64(99.9); S66(0); S67(0); S78(0); T79(0)]	0.662	-0.595096878	0.93019184
O15231	2xPhospho [T505(0); S512(0); T513(100); S519(100); S521(0); S523(0)]	0.623	-0.682695932	0.931904324
O15231	1xPhospho [S465(99.9); S468(0.1); S469(0); T472(0)]	0.844	-0.244685096	0.992376868
O15234	1xPhospho [S140(0); T141(0); T143(0); S148(100); S154(0); T155(0)]	1.709	0.773152397	0.581555081
O15234	2xPhospho [S363(100); S373(99.5); S381(0.5)]	1.844	0.882838656	0.632632269
O15371	2xPhospho [Y520(0); S521(0.4); T526(5.7); S528(94); S529(100); T548(0)]	0.275	-1.862496476	0.083790749
O15379	1xPhospho [Y404(0); S405(0); Y414(0); S424(100)]	1.309	0.388465097	0.879063032

O15440	2xPhospho [T496(0.1); S501(2.4); S502(2.4); S504(47.6); S505(47.6); S509(100)]	100	6.64385619	8.35E-17
O15446	1xPhospho [S124(0); S126(0); S128(0); S136(100)]	0.21	-2.251538767	0.082921465
O15446	2xPhospho [S124(0); S126(0); S128(100); S136(100)]	0.472	-1.083141235	0.195864976
O15446	1xPhospho [T475(0); S488(0); S490(97.8); T496(2.2)]	0.855	-0.226003675	0.980895643
O15479	1xPhospho [T31(0); S42(0); S43(0); S44(0.3); S46(0); S51(5); S52(89.8); S53(5)]	0.018	-5.795859283	8.35E-17
O15479	2xPhospho [T67(0); T68(0); S77(1.6); S78(1.6); T79(96.8); S81(100)]	0.694	-0.526992432	0.815077185
O15530	1xPhospho [S241(100); T245(0); Y248(0); S250(0); T255(0)]	1.28	0.35614381	0.879063032
O15534	2xPhospho [S683(100); S687(50); T692(50)]	100	6.64385619	8.35E-17
O15541	1xPhospho [Y241(0); Y244(0); Y249(0); S253(100)]	1.328	0.409255147	0.879063032
O15541	2xPhospho [Y80(0); S84(100); S85(100); S95(0); Y100(0)]	0.905	-0.144010303	0.965859951
O43150	1xPhospho [S701(100)]	0.711	-0.492078535	0.934211842
O43237	1xPhospho [Y184(0); S194(100)]	0.083	-3.590744853	0.000225732
O43237	1xPhospho [Y184(0); S194(100)]	2.222	1.151858817	0.253657486
O43237	1xPhospho [Y184(0); S194(100)]	2.21	1.14404637	0.264646281
O43237	2xPhospho [S402(0); S405(92.4); S406(3.8); S407(3.8); T410(50); S411(50)]	0.529	-0.918660373	0.815248216
O43251	8 1xPhospho [S26(100); S45(0)]	0.01	-6.64385619	8.35E-17
O43290	1xPhospho [S448(100); S463(0); T466(0)]	1.119	0.162210036	0.963617643
O43290	1xPhospho [Y337(0); S348(100)]	1.092	0.126972856	0.969680228
O43290	2xPhospho [S474(100); S486(100)]	0.894	-0.161653263	0.973763863
O43294	1xPhospho [S68(100); S80(0); S81(0); S82(0); S83(0); T88(0)]	0.01	-6.64385619	8.35E-17
O43294	3xPhospho [S137(100); S140(50); S141(50); S143(100)]	0.01	-6.64385619	8.35E-17
O43294	2xPhospho [S137(100); S140(50); S141(50); S143(0)]	0.189	-2.40354186	0.002507964
O43310	2xPhospho [T285(0); T289(0); S292(4.4); S293(95.6); S299(99.8); T302(0.2); S308(0)]	1.391	0.47612242	0.810156821
O43314	2xPhospho [T491(36); S492(36); S493(36); S498(91.3); S504(0.8)]	0.701	-0.512513651	0.9234483
O43314	2xPhospho [T482(0); Y483(0); T491(7.1); S492(92.9); S493(92.9); S498(7.1)]	0.974	-0.038006323	0.980895643
O43318	2xPhospho [S439(100); T444(0); T446(0); T448(0); S454(50); S455(50)]	0.362	-1.465938398	0.325321907
O43318	2xPhospho [S439(100); T444(0); T446(0); T448(0); S454(2.3); S455(97.7)]	0.623	-0.682695932	0.902718801
O43318	1xPhospho [S439(100); T444(0); T446(0); T448(0); S454(0); S455(0)]	1.134	0.18142064	0.96560904
O43353	1xPhospho [S529(0); S531(100)]	1.128	0.173767068	0.964246233
O43379	2xPhospho [T1021(0); S1022(0); T1033(0); S1038(0); S1044(0); S1047(0.3); S1048(5.5); S1049(94.2); T1053(100)]	100	6.64385619	8.35E-17
O43379	1xPhospho [S32(0.1); S33(99.9)]	1.103	0.141432791	0.953670877
O43399	1xPhospho [S186(100); S191(0); S192(0); S195(0); S201(0)]; O43399-3 1xPhospho [S189(100); S194(0); S195(0); S198(0); S204(0)]	1.522	0.605968359	0.73347811
O43399	1xPhospho [T140(0); S141(0); S145(98.3); T146(1.7); S149(0); S152(0)]; O43399-3 1xPhospho [T120(0); S121(0); S125(98.3); T126(1.7); S129(0); S132(0)]	1.531	0.614474283	0.738084678
O43399	1xPhospho [S19(100); S21(0); T23(0); T29(0)]; O43399-3 1xPhospho [S19(100); S21(0); T23(0); T29(0)]	0.627	-0.673462652	0.765377786
O43399	1xPhospho [S96(100); S103(0); S104(0); Y106(0)]	0.789	-0.341902795	0.939053555
O43399	2xPhospho [S161(100); T163(0); S166(100)]; O43399-3 2xPhospho [S164(100); T166(0); S169(100)]	1.176	0.23388806	0.949541112
O43399	3 1xPhospho [S96(100); S103(0); S104(0); Y106(0)]	1.15	0.201633861	0.969482098
O43432	2xPhospho [S230(100); S232(100)]	0.667	-0.584241333	0.950730609
O43432	1xPhospho [S495(100); T504(0)]	0.701	-0.512513651	0.957473289
O43432	1xPhospho [S1156(99.9); S1157(0.1); S1165(0)]	0.982	-0.02620507	0.980895643
O43432	1xPhospho [S1156(100); S1157(0); S1165(0)]	0.993	-0.010134377	0.980895643
O43432	1xPhospho [S495(100); T504(0)]	1.024	0.034215715	0.984145373
O43583	1xPhospho [T69(2.4); S73(97.6); S81(0); T86(0)]	100	6.64385619	8.35E-17
O43665	3 1xPhospho [S16(0); S21(1.9); S24(94.2); S25(1.9); S26(0); S27(0); S28(1.9); S31(0)]	1.672	0.741574847	0.66187646
O43665	3 2xPhospho [S16(100); S21(0); S24(32.7); S25(32.7); S26(32.7); S27(0.9); S28(0.9); S31(0)]	1.284	0.360645202	0.874743016
O43665	3 1xPhospho [S16(100); S21(0); S24(0); S25(0); S26(0); S27(0); S28(0); S31(0)]	0.801	-0.320125852	0.973263372
O43665	3 2xPhospho [S16(2.9); S21(97.2); S24(0); S25(0.1); S26(2.8); S27(2.8); S28(94.4); S31(0)]	0.803	-0.316528107	0.973763863
O43683	2xPhospho [S655(100); S661(100)]	1.357	0.440420721	0.855534442
O43683	1xPhospho [S596(100); T601(0); S602(0); S608(0); T609(0)]	0.569	-0.813499442	0.879063032
O43683	2xPhospho [T589(0); S593(100); S596(100); T601(0); S602(0); S608(0); T609(0)]	0.745	-0.424687669	0.961452497
O43683	1xPhospho [S655(100)]	0.995	-0.007231569	0.973263372
O43719	1xPhospho [S624(100); T633(0); Y634(0)]	100	6.64385619	8.35E-17
O43719	1xPhospho [S676(100)]	0.136	-2.878321443	0.002930238
O43719	1xPhospho [S479(0.1); S481(99.9); S485(0)]	0.558	-0.841662973	0.765070727
O43719	3xPhospho [S597(100); S600(100); S607(100)]	0.583	-0.778432211	0.803655393
O43719	2xPhospho [S597(100); S600(100); S607(0)]	1.312	0.39176772	0.872730156
O43719	3xPhospho [S702(100); S713(100); S714(100); S721(0)]	0.779	-0.360304767	0.904774194
O43719	1xPhospho [S452(97.4); S453(2.6); S467(0)]	1.249	0.320773477	0.922251444
O43719	1xPhospho [T30(100); T32(0); S40(0); T46(0); T48(0); Y50(0)]	0.74	-0.434402824	0.954539739
O43719	1xPhospho [S676(100)]	0.836	-0.258425153	0.965351295
O43719	1xPhospho [S642(100); Y650(0)]	1.071	0.09895848	0.975403456
O43719	2xPhospho [S713(100); S714(100); S721(0)]	1.035	0.049630768	0.980895643
O43719	2xPhospho [S616(100); S624(100); T633(0); Y634(0)]	0.961	-0.057391664	0.984563609
O43719	2xPhospho [T434(0); S445(0); S452(100); S453(100)]	0.971	-0.042456799	0.988250665
O43719	1xPhospho [S579(100)]	1.01	0.014355293	0.989898705
O43719	2xPhospho [S702(100); S713(99.9); S714(0.1); S721(0)]	0.96	-0.058893689	0.99184412
O43719	1xMet-loss+Acetyl [N-Term]; 1xPhospho [S2(98); T4(2)]	0.829	-0.270555993	0.994773029
O43741	1xPhospho [S108(100); Y125(0)]	100	6.64385619	8.35E-17
O43741	1xPhospho [S182(0); S183(0.1); S184(99.9); Y189(0); Y194(0)]	1.058	0.081339627	0.965351295

O43765	2xPhospho [S77(100); T81(99.9); S84(0.1); S88(0)]	3.151	1.655809754	0.075936768
O43765	2xPhospho [S77(100); T81(100); S84(0); S88(0)]	1.266	0.340277405	0.922170166
O43765	2xPhospho [S77(100); T81(100); S84(0); S88(0)]	1.054	0.075874867	0.983265186
O43815	1xPhospho [S239(0); S245(100)]	1.098	0.134878054	0.955790722
O43815	1xPhospho [Y121(0); T123(0); S134(5); Y135(0); S137(94.8); T143(0.3); S151(0)]	0.983	-0.024736678	0.971196575
O60264	2xPhospho [S47(96.9); S50(3.1); S66(100)]	0.01	-6.64385619	8.35E-17
O60264	2xPhospho [T99(0); T113(50); T115(50); S116(99.9)]	0.654	-0.612637459	0.820702501
O60264	2xPhospho [S47(4.2); S50(95.8); S66(100)]	1.205	0.269033146	0.921247725
O60264	1xMet-loss+Acetyl [N-Term]	0.8	-0.321928095	0.962617576
O60264	1xPhospho [S47(0); S50(0); S66(100)]	0.881	-0.182786076	0.965795497
O60271	1xPhospho [S183(100); S185(0); S190(0); T191(0); S194(0)]	0.01	-6.64385619	8.35E-17
O60271	2xPhospho [S203(100); T217(100)]	0.558	-0.841662973	0.783976635
O60271	2xPhospho [S728(0.1); S730(99.9); S732(0.1); S733(99.9)]	0.619	-0.691988685	0.852940464
O60271	2xPhospho [S728(0); S730(100); S732(0); S733(100)]	0.758	-0.399730246	0.852940464
O60293	2xPhospho [S1298(0); S1301(0); S1303(100); S1304(100); S1309(0); T1310(0)]	0.52	-0.943416472	0.557471588
O60293	1xPhospho [T647(0); S648(0); S649(0); S651(0); S655(100); S662(0)]	0.778	-0.36215794	0.980895643
O60307	2xPhospho [S680(100); T685(100); S690(0); S691(0); T692(0); S698(0); S699(0); S701(0)]	0.825	-0.277533976	0.979498769
O60318	2xPhospho [S527(100); S529(0); T530(0); S538(100)]	0.692	-0.531156057	0.891862526
O60341	1xPhospho [S166(100); S172(0); S181(0)]	1.506	0.59072177	0.749797187
O60341	2xPhospho [S126(0); S131(100); Y135(0); Y136(0); S137(100)]	1.285	0.361768359	0.893580559
O60341	2xPhospho [S126(0); S131(100); Y135(0); Y136(0); S137(100)]	1.05	0.070389328	0.985318278
O60343	3xPhospho [T749(0.1); S750(0.1); S751(99.7); T752(0.1); S754(100); S757(100); S759(0); T763(0); S764(0); T766(0)]	0.01	-6.64385619	8.35E-17
O60343	2xPhospho [S588(100); S591(100)]	0.415	-1.268816758	0.245916135
O60343	1xPhospho [S566(0); T568(0); S569(0); S570(100); S576(0)]	0.605	-0.724992953	0.916571051
O60343	2xPhospho [S566(100); T568(1.8); S569(1.8); S570(96.4); S576(0)]	0.686	-0.543719518	0.934211842
O60343	1xPhospho [S588(100); S591(0)]	0.946	-0.080087911	0.991765633
O60353	2xPhospho [S653(100); S656(99.7); T659(0.4); T661(0); S666(0); S673(0); S674(0); S675(0); S678(0); S679(0)]	0.073	-3.775959726	3.16E-05
O60353	2xPhospho [S620(100); S624(97.1); S626(3)]	2.351	1.233274539	0.101887983
O60353	3xPhospho [S620(100); S624(100); S626(0.1); S629(99.9)]	1.091	0.125651102	0.969680228
O60353	3xPhospho [S620(100); S624(100); S626(50); S629(50)]	1.061	0.085424656	0.979697817
O60502	1xPhospho [T501(3.6); S505(96.4); S509(0)]	12.04	3.589763487	0.000152412
O60502	1xPhospho [S364(100); T370(0); Y374(0); S375(0)]	0.63	-0.666576266	0.933760225
O60506	1xPhospho [T580(99.7); S587(0.3); S601(0); Y604(0); Y606(0)]	0.824	-0.279283757	0.966549726
O60524	2xPhospho [S747(100); S748(100); Y754(0)]	0.365	-1.454031631	0.075136356
O60524	2xPhospho [S747(100); S748(100); Y754(0)]	0.33	-1.59946207	0.383579446
O60678	2xPhospho [S25(100); S27(100)]	0.659	-0.60164963	0.675409227
O60749	1xPhospho [S117(100); S119(0.1)]	0.836	-0.258425153	0.978952227
O60784	2xPhospho [T154(0); S160(100); T164(100)]	1.303	0.381837084	0.862820654
O60825	2xPhospho [Y482(0); S483(100); S486(0); S493(100)]	0.612	-0.708396442	0.855534442
O60825	1xPhospho [S466(100); T468(0); S471(0); S472(0); S473(0); T475(0)]	0.755	-0.40545145	0.962617576
O60825	1xPhospho [S466(100); T468(0); S471(0); S472(0); S473(0); T475(0)]	0.881	-0.182786076	0.988250665
O60832	3xPhospho [S451(100); S453(100); S455(100); T458(0)]	0.396	-1.336427665	0.058320199
O60832	1xPhospho [S485(0); S494(100); T496(0); T497(0)]	3.02	1.59454855	0.093339751
O60832	1xPhospho [S485(0); S494(99.9); T496(0.1); T497(0.1)]	2.518	1.332278283	0.182397452
O60832	1xPhospho [S21(100); S42(0)]	1.647	0.719840555	0.679191074
O60832	3xPhospho [S451(100); S453(100); S455(100); T458(0)]	0.591	-0.758769964	0.728446408
O60832	2xPhospho [S451(100); S453(100); S455(0); T458(0)]	0.86	-0.217591435	0.94797789
O60832	2xPhospho [S451(100); S453(100); S455(0); T458(0)]	0.997	-0.00433459	1
O60841	2xPhospho [Y134(0); S135(100); S137(100)]	0.01	-6.64385619	8.35E-17
O60841	2xPhospho [S9(99.9); S12(50.1); T13(50.1)]	0.01	-6.64385619	8.35E-17
O60841	2xPhospho [Y134(0); S135(100); S137(100)]	0.374	-1.418889825	0.037993617
O60841	2xPhospho [Y134(0); S135(100); S137(100)]	0.384	-1.380821784	0.046211353
O60841	2xPhospho [S107(100); S113(100); S121(0)]	0.4	-1.321928095	0.062491056
O60841	2xPhospho [S214(100); S222(100)]	0.435	-1.200912694	0.150189095
O60841	1xPhospho [Y134(0); S135(0); S137(100)]	0.513	-0.962969269	0.688026385
O60841	2xPhospho [Y134(0); S135(100); S137(100)]	1.539	0.621993232	0.713954373
O60841	1xPhospho [S214(100); S222(0)]	0.718	-0.477944251	0.786292245
O60841	1xPhospho [S107(0); S113(100); S121(0)]	0.753	-0.40927823	0.847652956
O60841	3xPhospho [S182(100); S183(100); S186(100); S190(0); S196(0)]	1	0	0.973763863
O60841	1xPhospho [S107(0); S113(100); S121(0)]	0.921	-0.118726939	0.99184412
O60841	1xPhospho [S107(0); S113(100)]	0.916	-0.126580497	0.999359791
O60884	2xPhospho [Y391(0); S394(100); S395(100); S399(0); S400(0); S401(0)]	0.545	-0.875671865	0.848293321
O60884	1xPhospho [S78(0); S89(0); S104(100)]	1.025	0.03562391	0.973263372
O60885	1xPhospho [S1064(0); S1070(0); S1074(0); S1078(0); T1080(0); S1083(100)]	5.641	2.495950937	0.002002717
O60885	2xPhospho [S1064(6.2); S1070(87.6); S1074(6.2); S1078(0); T1080(0.5); S1083(99.5)]	1.843	0.882056071	0.592242927
O60885	3xPhospho [S1064(100); S1070(100); S1074(0); S1078(0); T1080(0.1); S1083(99.9)]	0.9	-0.152003093	0.985318278
O60927	3xPhospho [S73(66.7); S74(66.7); T75(66.7); S77(99.8); T88(0)]	0.705	-0.504304837	0.965013037
O60934	1xPhospho [Y429(0); S432(99.9); T434(0.1); S438(0)]	0.71	-0.49410907	0.964495484
O75052	2xPhospho [S172(0); T176(0); S188(3.7); S192(96.3); S194(0.2); S195(99.9)]	0.718	-0.477944251	0.968598771
O75116	2xPhospho [S1121(0); S1132(0.1); S1133(3.5); S1134(96.4); S1137(100); S1151(0)]	0.206	-2.279283757	0.001416855
O75151	1xPhospho [S537(2.9); S539(2.9); T541(94.3); T552(0)]	100	6.64385619	8.35E-17
O75151	2xPhospho [S534(91); S537(9); S539(4.8); T541(95.2); T552(0)]	0.871	-0.199255376	0.998086195

O75152	2xPhospho [S143(0); S144(0); S149(100); T151(0); S171(100); T177(0)]	13.487	3.753497571	1.20E-07
O75152	1xPhospho [T316(0); T321(100)]	1.418	0.503857533	0.83018047
O75152	1xPhospho [S758(89.5); S759(3.5); S761(3.5); T762(3.5); S768(0)]	1.276	0.351628329	0.880267055
O75152	2xPhospho [S758(100); S759(0); S761(99.9); T762(0.1); S768(0)]	0.802	-0.318325858	0.899197246
O75179	3xPhospho [Y2038(0); S2041(0.3); S2042(7.5); S2044(96.1); S2045(96.1); S2047(100); S2056(0)]	0.532	-0.910501849	0.572952566
O75179	2xPhospho [Y2038(0); S2041(0); S2042(0); S2044(2.8); S2045(97.3); S2047(99.9); S2056(0)]	1.065	0.09085343	0.965553053
O75179	1xPhospho [S2401(0.2); S2405(0.2); S2406(0.2); S2411(90.8); S2415(4.3); S2418(4.3)]	0.786	-0.347398782	0.966027585
O75179	1xPhospho [S1635(100); S1639(0); T1644(0); T1645(0); T1646(0); S1648(0); S1649(0)]	0.851	-0.232768963	0.990299313
O75351	1xPhospho [S93(0); S102(100); S108(0)]	2.629	1.394514142	0.119438986
O75351	1xPhospho [S93(0); S102(100); S108(0)]	2.14	1.097610797	0.251774426
O75362	2xPhospho [S441(100); S445(100)]	0.653	-0.614845103	0.94813191
O75367	1xPhospho [S170(2.5); S173(97.4); T174(0.1); T175(0); T178(0); T184(0); S187(0); T188(0)]	0.661	-0.597277823	0.945786032
O75379	1xPhospho [S30(100)]	0.01	-6.64385619	8.35E-17
O75381	2xPhospho [S247(0); S249(0); S251(0); S252(0); S260(0); S261(6.2); S262(93.7); S265(0.7); S268(83.3); S271(5.2); T272(5.2); S273(5.2); S274(0.3); S275(0)]	100	6.64385619	8.35E-17
O75381	1xPhospho [S247(0); S249(0); S251(0); S252(0); S260(0); S261(0); S262(0); S265(0); S268(0); S271(0); T272(0); S273(0); S274(2.4); S275(97.7)]	1.371	0.455228571	0.854283637
O75385	2xPhospho [S477(100); S479(100)]	0.454	-1.139235797	0.577062363
O75446	2xPhospho [S131(100); S138(100); T145(0); Y151(0); T157(0)]	1.932	0.950095094	0.573362551
O75446	2xPhospho [S131(100); S138(100); T145(0); Y151(0); T157(0)]	1.161	0.215367972	0.926103516
O75475	2xPhospho [T115(0); S116(0); S118(0); T122(100); S129(99.9); T134(0.1)]	4.31	2.107687869	0.010205786
O75475	2xPhospho [T159(0); T166(0); T167(0); T169(0); S171(100); S177(100)]	1.547	0.629473197	0.724603564
O75475	1xPhospho [T115(0); S116(0); S118(0); T122(0); S129(100); T134(0)]	1.37	0.454175893	0.873410737
O75475	2xPhospho [S102(4.1); S105(91.9); S106(4.1); T115(0); S116(99.8); S118(0.2); T122(0)]	1.226	0.293958979	0.925912375
O75475	1xPhospho [S102(0); S105(4.7); S106(95.3); T115(0); S116(0); S118(0); T122(0)]	0.785	-0.349235441	0.973942845
O75530	2xPhospho [S24(0); S25(0); S29(0); S34(100); S43(0); S46(0); T48(0); T50(0.3); T55(99.4); T57(0.3); T59(0)]	1.135	0.182692298	0.963156927
O75533	2xPhospho [T299(0); T303(100); S308(100); T313(0)]	0.052	-4.265344567	4.47E-05
O75533	2xPhospho [T257(0.2); S259(99.7); T261(50.1); T267(50.1)]	9.743	3.284366066	0.000142059
O75533	2xPhospho [T341(0); S344(99.9); S349(50.1); T350(50.1); T354(0)]	0.121	-3.046921047	0.003867255
O75533	1xPhospho [T341(0); S344(100); S349(0); T350(0); T354(0)]	1.774	0.82700601	0.535933059
O75533	2xPhospho [T296(97.3); T299(51.3); T303(51.3); S308(0); T313(0)]	1.752	0.809002775	0.598133479
O75533	3xPhospho [T296(100); T299(50); T303(50); S308(0.2); T313(99.8)]	1.774	0.82700601	0.629286646
O75533	2xPhospho [T257(0); S259(0.5); T261(0.5); T267(99); T273(100); T278(0); T285(0); S286(0); S287(0)]	1.384	0.468843943	0.848293321
O75533	2xPhospho [S336(0); T341(0); S344(99.9); S349(6.5); T350(93.5); T354(0)]	1.322	0.402722177	0.879063032
O75533	2xPhospho [S216(0); S217(0); T223(100); T227(100); S229(0)]	1.341	0.423309237	0.879063032
O75533	2xPhospho [Y54(0); T56(96); S57(4); T61(0); Y70(0); S71(0); S72(0); S73(0); T74(0); S75(100)]	1.208	0.272620455	0.908882713
O75533	2xPhospho [T125(0); S129(100); T142(100)]	1.287	0.364012054	0.918281661
O75533	1xPhospho [Y54(0); T56(0); S57(0); T61(0); Y70(0); S71(0); S72(0); S73(0); T74(2.2); S75(97.8)]	1.199	0.261831659	0.925912375
O75533	3xPhospho [T257(0.2); S259(0.9); T261(0.8); T267(98.2); T273(99.9); T278(100); T285(0); S286(0); S287(0)]	1.23	0.298658316	0.929708014
O75533	1xPhospho [T142(100)]	1.122	0.166072676	0.932557671
O75533	2xPhospho [T432(0); T434(100); T436(100); T442(0); T448(0)]	1.034	0.048236186	0.977860291
O75569	2xPhospho [S18(100); T20(100); S22(0)]	0.01	-6.64385619	8.35E-17
O75569	1xPhospho [S18(100); T20(0); S22(0)]	1.964	0.97379493	0.437240494
O75569	2xPhospho [S18(97.5); T20(97.5); S22(5)]	0.758	-0.399730246	0.908602715
O75592	2xPhospho [S2643(0); T2645(100); S2649(100); S2652(0)]	0.634	-0.657445255	0.883306273
O75592	3xPhospho [S137(1.3); S140(90.8); S143(91.7); S145(91.8); S151(24.4)]	0.996	-0.005782353	0.979498769
O75607	3xPhospho [T141(0); S143(0); S147(100); S151(100); S158(100)]	0.597	-0.744197163	0.520082466
O75607	1xMet-loss+Acetyl [N-Term]	1.552	0.634128558	0.755213977
O75607	3xPhospho [T141(0); S143(0); S147(100); S151(100); S158(100)]	0.749	-0.416962376	0.836554173
O75607	2xPhospho [T141(0); S143(0); S147(0); S151(100); S158(100)]	0.807	-0.309359421	0.958702638
O75676	2xPhospho [S681(50); S682(50); T687(99.9); S693(0); S694(0)]	1.221	0.2880632	0.921247725
O75676	2xPhospho [Y342(0); S343(100); S347(100)]	0.877	-0.189351252	1
O75683	1xPhospho [S138(100)]	100	6.64385619	8.35E-17
O75781	2xPhospho [S116(100); S124(100)]	0.573	-0.803392956	0.878394132
O75794	2xPhospho [T54(0); S56(0); S60(100); S70(5.7); T75(88.5); T77(5.7); T79(0); T88(0)]	1.208	0.272620455	0.919287981
O75940	1xPhospho [S197(0); S201(0); S204(98.6); T206(1.4)]	1.12	0.163498732	0.973263372
O75940	1xPhospho [S51(0); T52(0); S55(0); T57(0); S60(0); S61(0); S63(100); S66(0); T67(0); T70(0); S72(0)]	0.92	-0.120294234	0.980895643
O75976	2xPhospho [S1358(0); S1361(0); T1368(100); T1370(100); T1374(0); Y1376(0); S1377(0); S1378(0)]	0.662	-0.595096878	0.681177379
O75976	2xPhospho [S1358(0); S1361(0); T1368(100); T1370(100); T1374(0); Y1376(0); S1377(0); S1378(0)]	1.168	0.224040274	0.962617576
O75976	3xPhospho [S1358(100); S1361(0); T1368(100); T1370(100); T1374(0); Y1376(0); S1377(0); S1378(0)]	0.764	-0.388355457	0.964978421
O76080	1xPhospho [S48(100); T52(0); S54(0); S56(0); S58(0); T60(0); S61(0); S63(0); S65(0)]	2.305	1.204766751	0.428891186
O76094	3xPhospho [T618(0); S620(3.8); S621(96.2); T624(96.2); S625(3.8); S630(100); T633(0); S635(0); S637(0); T638(0); S639(0)]	0.429	-1.220950447	0.177756659
O76094	2xPhospho [T618(0); S620(50); S621(50); T624(96.4); S625(3.6); S630(0); T633(0); S635(0); S637(0); T638(0); S639(0)]	0.764	-0.388355457	0.874925894
O94762	1xPhospho [Y804(0); T805(0); S815(100); T821(0)]	0.811	-0.30222618	0.965013037
O94762	1xPhospho [S720(0); Y723(0); S727(100)]	0.814	-0.2968993	0.973263372
O94804	2xPhospho [S448(1.9); S450(98.1); S454(100); S455(0); T459(0)]	0.595	-0.749038426	0.550634186
O94804	1xPhospho [S448(0); S450(0); S454(2.4); S455(97.7); T459(0)]	0.707	-0.50021788	0.965139891

O94806	2xPhospho [S13(0); T17(0); S27(98.7); S30(50.7); S31(50.7)]	0.01	-6.64385619	8.35E-17
O94826	1xPhospho [S91(100); S96(0); S110(0)]	0.833	-0.263611599	0.93019184
O94826	1xPhospho [S91(100); S96(0); S110(0)]	0.961	-0.057391664	0.99277609
O94854	2xPhospho [S35(99.8); S36(0.1); S37(0.1); T40(0); S42(100); S43(0)]	2.268	1.18142064	0.302119512
O94854	1xPhospho [S35(0); S36(0); S37(0); T40(0); S42(100); S43(0)]	1.224	0.291603558	0.938190912
O94916	1xPhospho [S627(0); S630(0); T631(0); T634(0); S639(0); S646(100); S648(0); S649(0); S651(0); S652(0); S653(0); S657(0)]	6.514	2.70354372	0.002356897
O94921	1xPhospho [S95(100)]	0.01	-6.64385619	8.35E-17
O94929	2xPhospho [S503(100); S504(100)]	0.459	-1.123433941	0.72445075
O94964	2 2xPhospho [T156(0); S165(0); S175(95.3); S180(4.7); S184(95.3); S185(4.7); S188(0); S189(0)]	100	6.64385619	8.35E-17
O94992	2xPhospho [S97(100); S98(100); T112(0); S125(0); S128(0)]	0.01	-6.64385619	8.35E-17
O94992	3xPhospho [S233(94.5); T236(94.5); S237(11); S252(100); S260(0)]	0.289	-1.790858602	0.285917758
O95155	2xPhospho [S45(0); S50(0.1); S52(0.1); T61(99.7); T64(9); S65(91.1); S70(0)]	100	6.64385619	8.35E-17
O95155	1xPhospho [S103(0); S105(100); S112(0)]	11.564	3.531568609	3.83E-05
O95155	1xPhospho [S76(0.1); S78(2.8); S79(97.1); S83(0); S84(0); S86(0); S87(0); S88(0.1); S90(0); S92(0); T95(0); S97(0); S99(0); S101(0)]	1.159	0.212880566	0.938190912
O95155	2xPhospho [S76(4.6); S78(90.8); S79(4.6); S83(4.4); S84(4.4); S86(86.6); S87(4.4); S88(0.2); S90(0); S92(0); T95(0); S97(0); S99(0); S101(0)]	0.753	-0.40927823	0.941415413
O95218	2 1xPhospho [S153(100); S165(0)]	1.945	0.959770155	0.295667598
O95218	2 1xPhospho [S120(100); Y124(0)]	2.046	1.032806145	0.348863514
O95218	2 1xPhospho [Y114(0); S120(100); Y124(0)]	2.256	1.173767068	0.374514204
O95218	2 1xPhospho [Y183(0); S188(100); S193(0)]	1.956	0.96790637	0.39631056
O95218	2 1xPhospho [Y183(0); S188(100); S193(0)]	1.716	0.779049553	0.588287551
O95218	2 3xPhospho [T303(100); S305(100); S307(100); S310(0); T317(0)]	1.662	0.732920382	0.630082428
O95218	2 1xPhospho [Y114(0); S120(100); Y124(0)]	1.482	0.567545448	0.688350005
O95218	2 2xPhospho [S305(100); S307(100); S310(0); T317(0)]	1.502	0.586884813	0.703278942
O95218	2 2xPhospho [T303(0); S305(100); S307(100); S310(0); T317(0)]	1.501	0.585923977	0.769999353
O95218	2 2xPhospho [S181(100); Y183(0); S188(100); S193(0)]	1.425	0.510961919	0.786957957
O95218	2 1xPhospho [Y167(0); S181(0); Y183(0); S188(100); S193(0)]	1.358	0.44148348	0.865762755
O95218	2 2xPhospho [Y167(0); S181(100); Y183(0); S188(100); S193(0)]	0.993	-0.010134377	1
O95232	1xPhospho [S410(0); T412(0); T419(0); S420(0); S425(0); T429(0); S431(100)]	0.01	-6.64385619	8.35E-17
O95232	1xPhospho [T419(0); S420(0); S425(0); T429(0); S431(100)]	1.853	0.889862881	0.644921119
O95232	1xPhospho [S110(0); S115(0); S116(100); T122(0)]	0.9	-0.152003093	1
O95235	2xPhospho [T253(0); S254(0); T255(4.9); S256(95.1); S259(0); S265(0); S266(0); S268(0); T271(4.8); S272(94.9); S273(0.3); S274(0); T279(0); S280(0)]	10.461	3.386948865	5.34E-05
O95239	2xPhospho [Y1046(0); S1048(100); S1051(100); T1073(0)]	100	6.64385619	8.35E-17
O95239	2xPhospho [T799(100); S801(100); T803(0)]	2.915	1.543495883	0.217201928
O95239	1xPhospho [T799(0); S801(100); T803(0)]	1.297	0.37517848	0.872730156
O95251	3xPhospho [T97(7.7); S99(96.2); S100(96.2); S102(100); T104(0); S111(0)]	0.01	-6.64385619	8.35E-17
O95251	2xPhospho [S99(2.5); S100(2.5); S102(95.1); T104(0); S111(100); T115(0)]	100	6.64385619	8.35E-17
O95251	2xPhospho [T118(0); S124(100); T128(100); T130(0); S135(0); S136(0); S138(0); S143(0); S144(0); S148(0); S152(0)]	9.199	3.201477038	1.38E-07
O95251	3xPhospho [T118(0); S124(100); T128(100); T130(0); S135(48.6); S136(48.6); S138(2.7); S143(0); S144(0); S148(0); S152(0)]	1.493	0.578214165	0.738682212
O95251	1xPhospho [S158(0); S162(100); S164(0); S167(0)]	1.18	0.23878686	0.921519407
O95251	2xPhospho [S99(50); S100(50); S102(100); T104(0); S111(0)]	0.944	-0.083141235	0.999714477
O95295	1xPhospho [S126(0); Y129(0); S133(100)]	0.062	-4.011587974	1.63E-05
O95297	2xPhospho [S229(3.8); S232(48.1); S234(48.1); Y241(100); S247(0); S252(0)]	0.479	-1.061902439	0.29954996
O95297	2xPhospho [S258(0); S260(100); Y263(100)]	1.179	0.237563718	0.904079048
O95297	1xPhospho [S258(0); S260(0); Y263(100)]	1.027	0.038436182	0.969680228
O95365	1xPhospho [S511(0); T515(0); T517(0); S525(97.9); S526(2.1)]	0.01	-6.64385619	8.35E-17
O95365	1xPhospho [S549(100)]	1.017	0.024319679	0.976737179
O95365	2xPhospho [S337(100); S341(100); Y351(0); Y352(0)]	0.88	-0.184424571	0.994678177
O95391	1xPhospho [S235(100); Y243(0); S256(0)]	1.38	0.464668267	0.824489359
O95400	2xPhospho [S139(100); S141(100); S146(0); T150(0); S151(0); S153(0)]	0.01	-6.64385619	8.35E-17
O95425	2 1xPhospho [S313(100)]; O95425 1xPhospho [S707(100)]	1.921	0.941857519	0.508853196
O95425	2 2xPhospho [S261(100); S263(0); S270(100)]	0.59	-0.76121314	0.907368665
O95487	1xPhospho [T329(100); T332(0); T341(0); S342(0); T344(0); S347(0)]	1.09	0.124328135	0.964978421
O95628	1xPhospho [S424(0); S430(0); S432(100); T434(0); S435(0); S439(0); S440(0); S441(0); T443(0); T444(0); T445(0)]; O95628-9 1xPhospho [S421(0); S427(0); S429(100); T431(0); S432(0); S436(0); S437(0); S438(0); T440(0); T441(0); T442(0)]	0.905	-0.144010303	0.995968957
O95639	1xPhospho [T218(0); S227(0); S230(97.8); S231(2.3)]	1.247	0.318461465	0.899590329
O95639	1xPhospho [S200(0); S201(0); S202(99.9); T207(0); S208(0); S211(0); S212(0)]	1.22	0.286881148	0.920957334
O95639	2xPhospho [S200(3.2); S201(3.2); S202(93.6); T207(0); S208(0); S211(0); S212(100)]	0.827	-0.274040765	0.983654603
O95674	2xPhospho [S21(100); S23(0); T31(0); S33(96.6); S35(3.3); S37(0.1)]	0.575	-0.798366139	0.492376138
O95684	2xPhospho [T143(0); T144(0); S152(0); S156(100); S160(100)]	1.615	0.691534165	0.595798185
O95684	3xPhospho [T143(0); T144(0); S152(100); S156(100); S160(100)]	1.789	0.839153387	0.655232554
O95684	2xPhospho [T143(0); T144(0); S152(0); S156(100); S160(100)]	1.406	0.491596594	0.786602611
O95714	1xPhospho [S2928(100); S2940(0); S2942(0)]	0.105	-3.251538767	0.00013116
O95714	2xPhospho [S1938(0); S1942(100); T1944(100); S1948(0); T1953(0)]	0.675	-0.567040593	0.868176795
O95772	3xPhospho [S204(0); Y209(0); S210(100); S214(100); S218(100)]	0.309	-1.694321257	0.313771001
O95785	3xPhospho [S1006(100); S1010(96.2); S1012(3.8); S1017(100)]	100	6.64385619	8.35E-17
O95785	2xPhospho [S1006(0); S1010(0); S1012(100); S1017(100)]	1.423	0.508935662	0.808799965
O95785	2xPhospho [S1146(100); S1151(0); T1153(50); S1155(50); T1159(0)]	1.001	0.001441974	0.97998947

O95793	1xMet-loss+Acetyl [N-Term]; 1xPhospho [S2(100); S12(0); S16(0); S18(0)]	0.577	-0.793356776	0.879063032
O95793	1xPhospho [T269(0); T277(2.5); S278(97.5); Y281(0); S289(0)]	1.102	0.140124224	0.9616322
O95793	1xPhospho [T384(0); S390(100); T396(0); S397(0)]	1.028	0.039840265	0.980895643
O95817	3xPhospho [S283(0); S284(0); T285(100); S289(100); S291(100)]	2.005	1.003602237	0.467067441
O95817	3xPhospho [S377(99.9); S381(0.1); S385(0.1); S386(99.9); S389(100); T392(0)]	0.452	-1.145605322	0.527472712
O95817	2xPhospho [S283(0); S284(0); T285(100); S289(98.5); S291(1.5)]	0.731	-0.452056689	0.804029785
O95817	2xPhospho [S377(100); S381(0); S385(50); S386(50)]	0.746	-0.422752464	0.83187574
O95817	3xPhospho [S279(0.3); S283(7.5); S284(96.1); T285(96.1); S289(96.1); S291(3.9)]	0.71	-0.49410907	0.880267055
O95817	1xPhospho [S348(3.2); S352(96.8); S359(0)]	1.324	0.404903122	0.924586883
O95817	1xPhospho [S283(0); S284(0); T285(100); S289(0); S291(0)]	1.199	0.261831659	0.938029982
O95817	1xPhospho [S377(100); S381(0); S385(0); S386(0)]	0.802	-0.318325858	0.965013037
O95817	1xPhospho [S171(2.2); S173(97.8); S177(0); S180(0); S181(0); S182(0); S183(0); S184(0); S185(0); S187(0); S190(0); S191(0)]	1.045	0.063502942	0.974924677
O95817	3xPhospho [S377(100); S381(100); S385(96); S386(4)]	0.752	-0.411195433	0.977858961
O95817	2xPhospho [S377(0); S381(0); S385(0.1); S386(99.9); S389(99.9); T392(0.1)]	0.824	-0.279283757	0.97998947
O95817	3xPhospho [S268(0); S269(0); S274(50.1); S275(50.1); S279(99.9); S283(95.3); S284(4.5); T285(0.2); S289(0); S291(0)]	0.829	-0.270555993	0.980895643
O95817	2xPhospho [S279(100); S283(100); S284(0); T285(0); S289(0); S291(0)]	0.918	-0.123433941	0.989102507
O95817	1xPhospho [T231(0); Y233(0); Y240(0); T242(0); Y247(100)]	0.898	-0.15521265	1
O95835	1xPhospho [S462(0); S464(100)]	0.725	-0.4639471	0.945786032
O95983	1xPhospho [Y52(0); S56(100); S60(0); T61(0)]	0.914	-0.12973393	0.99811985
O95997	1xPhospho [S165(100)]	0.491	-1.02620507	0.786602611
O96013	2xPhospho [S97(0.2); S99(99.8); S104(100)]	3.093	1.629006833	0.188267944
O96013	1xPhospho [S474(100); T478(0); Y480(0); S488(0)]	1.549	0.631337144	0.763457172
O96013	1xPhospho [S181(100); T187(0); S195(0)]	1.244	0.314986485	0.910434651
O96013	1xPhospho [S181(100); T187(0); S195(0)]	0.974	-0.038006323	0.980895643
O96017	1xPhospho [S500(0); S505(0); T506(0); S516(0.1); T517(0.1); S518(99.9)]	100	6.64385619	8.35E-17
O96020	1xPhospho [S17(0); T19(0); S21(100)]	0.803	-0.316528107	0.96955242
P00338	1xPhospho [Y10(100)]	1.21	0.275007047	0.925853562
P00338	1xPhospho [Y10(100); T18(0)]	0.889	-0.169744676	1
P01036	2xPhospho [T7(100); T14(100); S21(0); S22(0); S23(0)]	1.988	0.991317757	0.342984125
P01106	3xPhospho [S277(11.4); S279(94.2); S281(94.2); S283(0.1); S288(0.1); S293(100)]	0.01	-6.64385619	8.35E-17
P01106	2xPhospho [T58(100); S62(50); S64(50)]	100	6.64385619	8.35E-17
P01106	2xPhospho [S277(0.2); S279(49.9); S281(49.9); S283(0); S288(0); S293(100)]	1.626	0.701327257	0.664608712
P01106	1xMet-loss [N-Term]; 1xPhospho [S6(100); T8(0)]	1.259	0.332278283	0.886312012
P01106	2xPhospho [T58(100); S62(100); S64(0)]	1.001	0.001441974	0.980895643
P01106	2xPhospho [T58(100); S62(100); S64(0)]	0.817	-0.291592017	0.984145373
P01106	2xPhospho [S277(0); S279(0); S281(100); S283(0); S288(0); S293(100)]	0.942	-0.086201035	0.987542027
P01213	1xPhospho [S99(0); S101(100)]	0.646	-0.63039393	0.703341962
P02533	1xPhospho [S427(0); S428(0); S429(0); S432(0); S433(2); S435(98); S437(0); S438(0)]	0.358	-1.481968507	0.44984964
P02545	2xPhospho [S546(11.1); T548(83.3); S568(31.2); S571(34.7); S572(34.7); S573(5.1); Y579(0)]	5.326	2.413052429	0.004427419
P02545	2xPhospho [S599(0); S601(0); S603(0); S612(0); S613(0); S615(0); S616(100); S618(0.1); S619(99.8); T621(0.1); T623(0)]	0.502	-0.994240731	0.432713576
P02545	2xPhospho [S628(100); S632(0); S636(100); T643(0)]	0.463	-1.110915901	0.568328245
P02545	1xPhospho [S12(0); S17(100); S18(0); T19(0); S22(0); T24(0)]	1.786	0.83673208	0.673141093
P02545	3xPhospho [S546(0); T548(0.2); S568(91.4); S571(91.6); S572(91.6); S573(25.2); Y579(0)]	1.539	0.621993232	0.725384349
P02545	2xPhospho [S390(100); S392(100); T394(0); S395(0)]	1.446	0.532067552	0.751204811
P02545	1xPhospho [S458(100); S463(0)]	1.304	0.38294387	0.879568708
P02545	1xPhospho [S628(0); S632(0); S636(100); T643(0)]	0.791	-0.3382504	0.883154252
P02545	2xPhospho [S423(0.1); T424(99.9); S426(0); S428(0.1); S429(97.7); S431(2.3)]	0.661	-0.597277823	0.886312012
P02545	1xPhospho [S599(0); S601(0); S603(0); S612(0); S613(0); S615(0); S616(0); S618(0.1); S619(99.9); T621(0.1); T623(0)]	0.765	-0.386468347	0.918281661
P02545	1xPhospho [S390(100); S392(0); T394(0); S395(0)]	1.049	0.069014678	0.985461924
P02794	1xPhospho [S164(0); Y169(0); T175(0); S179(3.4); S183(96.6)]	1.508	0.592636429	0.742967319
P02794	1xPhospho [S164(0); Y169(0); T175(0); S179(99.9); S183(0.1)]	1.359	0.442545456	0.862820654
P04083	2xPhospho [S34(100); S37(100); Y39(0); T41(0); S45(0); S46(0)]	0.713	-0.488026018	0.965139891
P04083	1xPhospho [S34(100); S37(0); Y39(0); T41(0); S45(0); S46(0)]	1.041	0.057970069	0.989304921
P04183	1xPhospho [S231(100)]	0.67	-0.577766999	0.897258645
P04183	1xMet-loss+Acetyl [N-Term]	0.704	-0.506352666	0.93019184
P04264	1xPhospho [S13(0); S18(0); S19(0); S21(100); Y27(0)]	100	6.64385619	8.35E-17
P04626	2xPhospho [S1073(0); S1078(100); S1083(100)]	100	6.64385619	8.35E-17
P04792	1xPhospho [S82(100); S83(0); S86(0)]	1.498	0.583037624	0.706163724
P04792	1xPhospho [S199(100); T202(0)]	1.339	0.421155961	0.860874709
P04792	1xPhospho [S15(100)]	1.19	0.250961574	0.93019184
P04792	1xPhospho [T174(0); S176(0); T180(0); T184(0); S187(100)]	0.937	-0.093879047	0.99811985
P05023	1xPhospho [Y10(0); S16(100)]	1.184	0.243669081	0.908086456
P05114	2xPhospho [T81(0); T83(50); S86(50); S89(100)]	0.885	-0.17625064	0.986075329
P05204	2xPhospho [S25(100); S29(100)]	0.01	-6.64385619	8.35E-17
P05386	2xPhospho [S101(100); S104(100)]; P05387 2xPhospho [S102(100); S105(100)]	100	6.64385619	8.35E-17
P05386	1xPhospho [S101(100); S104(0)]; P05387 1xPhospho [S102(100); S105(0)]	0.758	-0.399730246	0.855534442
P05386	1xPhospho [S101(0); S104(100)]; P05387 1xPhospho [S102(0); S105(100)]	0.701	-0.512513651	0.93019184
P05386	1xPhospho [S58(0); S83(96.8); T84(3.2)]	1.105	0.14404637	0.973263372
P05386	2xPhospho [S101(100); S104(100)]; P05387 2xPhospho [S102(100); S105(100)]	0.977	-0.033569533	0.991765633
P05386	2xPhospho [S101(100); S104(100)]; P05387 2xPhospho [S102(100); S105(100)]	0.985	-0.02180437	0.993670231

P05387	1xPhospho [Y3(0); S6(0); Y7(0); S16(0); S17(98.2); S19(1.8)]	1.817	0.86155842	0.409638695
P05387	1xPhospho [S64(0); S74(0); S79(100); S86(0)]	0.706	-0.502259911	0.776223339
P05387	2xPhospho [S102(100); S105(100)]	1.144	0.194087052	0.962617576
P05387	2xPhospho [S102(100); S105(100)]	1.037	0.052415894	1
P05388	2xPhospho [S304(100); S307(100)]	100	6.64385619	8.35E-17
P05388	1xPhospho [S304(98.3); S307(1.7)]	0.45	-1.152003093	0.755213977
P05388	1xPhospho [S304(99.8); S307(0.2)]	1	0	0.973263372
P05388	2xPhospho [S304(100); S307(100)]	0.978	-0.03209363	0.99184412
P05388	2xPhospho [S304(100); S307(100)]	0.985	-0.02180437	1
P05455	1xPhospho [S92(98.7); S94(1.3); T101(0); Y104(0)]	1.229	0.297484916	0.946054239
P05455	1xPhospho [S366(100); T379(0)]	1.141	0.190298792	0.963156927
P05455	1xPhospho [S366(100); T379(0)]	1.063	0.088141597	0.986075329
P05455	1xPhospho [T362(0); S366(100); T379(0)]	1.04	0.056583528	0.986098104
P05534	2xPhospho [S343(0); Y344(0); S345(0); S349(0); S350(0); S352(0); S356(100); S359(100); T361(0)]	0.641	-0.641603738	0.930641583
P05556	1xPhospho [T777(0); Y783(100)]	1.866	0.899948986	0.581579159
P05783	2xPhospho [S15(0.3); S18(99.7); S23(100); Y24(0); S30(0); S31(0); S34(0); Y36(0); S42(0); S44(0)]	3.512	1.812292845	0.011836825
P05783	3xPhospho [S15(0.4); S18(99.6); S23(100); Y24(0); S30(93.7); S31(5.9); S34(0.4); Y36(0); S42(0); S44(0)]	1.245	0.316145742	0.915593348
P05783	1xPhospho [S398(0); S399(0); S401(100); T404(0)]	1.212	0.277389699	0.930376591
P05783	1xPhospho [S60(99.8); T65(0.3)]	1.063	0.088141597	0.969680228
P05787	1xPhospho [S34(100); S35(0); S36(0); S37(0); S39(0)]	100	6.64385619	8.35E-17
P05787	1xPhospho [S274(100); S280(0); Y282(0)]	2.186	1.128293401	0.439534821
P05787	3xPhospho [T415; T416; S441; S442; S445; S449; S451; S453]	0.608	-0.717856771	0.647620981
P05787	2xPhospho [T415(48.6); T416(48.6); S417(2.8); Y419(0); S424(0); S425(0); Y427(0); T431(0); S432(0); S436(0); Y437(0); S438(0); S441(0); S442(0); S445(0); S449(4.8); S450(4.8); S451(85.5); S453(4.8)]	0.673	-0.57132159	0.746950683
P05787	1xPhospho [S404(0); S410(100); T413(0)]	0.528	-0.921390165	0.832861216
P05787	2xPhospho [S24(0); Y25(0); T26(0); S27(0); S31(100); S34(100); S35(0); S36(0); S37(0); S39(0)]	1.242	0.312665174	0.911330706
P05787	1xPhospho [T415(0); T416(0); S417(0); Y419(0); S424(0); S425(0); Y427(0); T431(0); S432(0); S436(0); Y437(0); S438(0); S441(0); S442(0); S445(0.2); S449(95.2); S450(4.4); S451(0.2); S453(0)]	0.843	-0.246395464	0.965013037
P05787	2xPhospho [S475(100); S477(1.4); S478(98.6)]	1.043	0.060739158	0.971220368
P05787	1xPhospho [S475(100); S477(0); S478(0)]	0.823	-0.281035664	0.97998947
P05787	1xPhospho [S253(100); S258(0)]	0.979	-0.030619235	0.980895643
P06400	1xPhospho [S360(0); T373(100)]	100	6.64385619	8.35E-17
P06400	3xPhospho [T766(0); Y771(0); S773(0.5); T774(0.1); T778(49.8); S780(49.8); S788(99); Y790(1); S794(8.8); S795(91.2)]	0.226	-2.145605322	0.0936828
P06400	1xPhospho [T342(0); T345(0); S347(0); S350(99.9); T353(0.1); T356(0)]	2.487	1.314406508	0.344536514
P06400	2xPhospho [T583(0); S588(0); T601(0); Y606(0); S608(100); S612(100)]	0.468	-1.095419565	0.562873618
P06400	2xPhospho [Y805(0); S807(100); S811(100); Y813(0)]	0.737	-0.440263476	0.851160627
P06400	2xPhospho [T583(0); S588(0); T601(0); Y606(0); S608(100); S612(100)]	0.706	-0.502259911	0.851597639
P06400	2xPhospho [T583(0); S588(0); T601(0); Y606(0); S608(100); S612(100)]	1.178	0.236339539	0.904079048
P06400	2xPhospho [S788(100); Y790(0); S794(1.4); S795(98.6)]	0.889	-0.169744676	0.964978421
P06400	2xPhospho [S816(0); T821(97.6); T823(2.4); T826(100)]	1.053	0.074505436	0.973263372
P06400	1xPhospho [T241(0); S249(100)]	0.799	-0.323732592	0.985461924
P06400	1xPhospho [T9(0); T12(0); S37(100)]	0.91	-0.13606155	1
P06733	1xPhospho [S37(0); S40(0); T41(0); Y44(100)]	0.471	-1.086201035	0.370464672
P06748	1xPhospho [T46(0); S48(0); Y67(0); S70(100); T75(0); T78(0)]	0.01	-6.64385619	8.35E-17
P06748	1xPhospho [Y67(0); S70(100)]	1.894	0.921436331	0.337028176
P06748	1xAcetyl [N-Term]; 2xPhospho [S4(100); S10(100); Y17(0)]	1.788	0.838346737	0.416337943
P06748	1xPhospho [S106(0); S112(0); S125(100)]	0.657	-0.606034724	0.669115412
P06748	1xPhospho [Y67(0); S70(100)]	1.637	0.711054322	0.724983097
P06748	1xPhospho [Y67(0); S70(100); T75(0); T78(0)]	1.41	0.495695163	0.811661161
P06748	1xPhospho [S137(100); S139(0)]	1.169	0.22527493	0.950730609
P06748	1xPhospho [S137(0); S139(100)]	1	0	0.980895643
P06748	1xPhospho [S106(0); S112(0); S125(100)]	1.045	0.063502942	0.987542027
P07814	2xPhospho [T737(4); S739(96); T745(3.8); T746(3.8); S747(92.3); S750(0); Y754(0)]	0.01	-6.64385619	8.35E-17
P07814	2xPhospho [Y872(0); S880(0); S882(2.5); S883(97.5); S885(0.1); S886(97.4); T888(2.5)]	2.412	1.270229907	0.256244824
P07814	1xPhospho [T737(0); S739(100); T745(0); T746(0); S747(0); S750(0); Y754(0)]	0.859	-0.219269964	0.97497938
P07900	1xPhospho [T594(0); S595(0); T601(0.1); S602(2.8); T603(97); Y604(0.1); T607(0)]	0.01	-6.64385619	8.35E-17
P07900	1xPhospho [S252(0); S263(100)]	1.697	0.762986565	0.516504611
P07900	1xPhospho [S252(0); S263(100)]	1.621	0.696884091	0.590805667
P07900	1xPhospho [S252(0); S263(100)]	1.461	0.546956178	0.71190995
P07900	1xPhospho [S252(0); S263(100)]	1.4	0.485426827	0.790665834
P07900	1xPhospho [S252(0); S263(100)]	1.356	0.439357178	0.830379242
P07910	1xPhospho [S253(95.8); S260(4.2)]	0.01	-6.64385619	8.35E-17
P07910	2xPhospho [S253(100); S260(100)]	0.026	-5.265344567	8.35E-17
P07910	1xPhospho [S100(0); Y105(0); S107(99.9); T109(0.2); S113(0); S115(0); S119(0); S120(0); S121(0); Y126(0)]	100	6.64385619	8.35E-17
P07910	2xPhospho [S253(100); S260(100)]	0.071	-3.816037165	2.38E-12
P07910	1xPhospho [S253(0); S260(100)]	0.119	-3.070966521	3.29E-08
P07910	1xPhospho [S253(0); S260(100)]	2.116	1.081339627	0.318585357
P07910	1xPhospho [S253(0); S260(100)]	1.79	0.839959587	0.425789298
P07910	2xPhospho [S100(0); Y105(0); S107(0); T109(0.2); S113(99.8); S115(100); S119(0); S120(0); S121(0); Y126(0)]	1.167	0.222804561	0.933036209

P07910	2xPhospho [S253(100); S260(100)]	1.091	0.125651102	0.962617576
P07910	2xPhospho [S253(100); S260(100)]	1	0	1
P07948	1xPhospho [S11(0); S13(100)]	1.378	0.462575888	0.841807363
P07948	2xPhospho [S11(100); S13(100)]	0.799	-0.323732592	0.973263372
P08047	2xPhospho [S40(51.2); S41(51.2); S42(91.9); T43(5.5); S45(0); S46(0); S47(0); S48(0); T49(0); S56(0); S59(0); T67(0); S69(0)]	0.789	-0.341902795	0.9616322
P08047	3xPhospho [S40(50); S41(50); S42(90.2); T43(10.3); S45(92.4); S46(7); S47(0.1); S48(0.1); T49(0.1); S56(0.1); S59(0); T67(0); S69(0)]	1.101	0.138814469	0.964978421
P08238	1xPhospho [S255(100); S261(0)]	7.964	2.993493221	1.03E-05
P08238	1xPhospho [S226(100)]	5.091	2.347949066	0.000200686
P08238	1xPhospho [S255(100); S261(0)]	3.481	1.799501814	0.042533967
P08238	1xPhospho [S255(100); S261(0)]	2.54	1.344828497	0.374360028
P08238	1xPhospho [S586(0); S587(0); T593(0.1); S594(2.3); T595(97.6); Y596(0); T599(0)]	0.892	-0.164884385	0.986298098
P08240	3xPhospho [T284(0); S286(0); S296(100); S297(100); S298(100); S307(0); T308(0); S311(0); T313(0)]	0.01	-6.64385619	8.35E-17
P08240	2xPhospho [T284(6.8); S286(93.2); S296(92.7); S297(6.8); S298(0.5); S307(0); T308(0); S311(0); T313(0)]	1.135	0.182692298	0.939053555
P08559	2xPhospho [Y289(0); S293(100); S295(0); S300(100); Y301(0)]	0.01	-6.64385619	8.35E-17
P08559	2xPhospho [Y289(0); S293(99.9); S295(0.1); S300(100); Y301(0)]	0.01	-6.64385619	8.35E-17
P08559	1xPhospho [Y289(0); S293(100); S295(0); S300(0); Y301(0)]	0.481	-1.055891201	0.769621572
P08559	1xPhospho [Y289(0); S293(100); S295(0); S300(0); Y301(0)]	0.805	-0.312939312	0.954133217
P08581	3xPhospho [S988(0.1); S990(100); T992(0); T993(0); S997(100); S1000(100); Y1003(0)]	0.01	-6.64385619	8.35E-17
P08621	1xPhospho [S410(100)]	0.01	-6.64385619	8.35E-17
P08621	1xPhospho [Y219(0); S226(100)]	1.112	0.153156788	0.956843841
P08621	1xPhospho [S320(100)]	1.051	0.071762669	0.974924677
P08621	2xPhospho [T216(50); S217(50); Y219(0); S226(100)]	0.905	-0.144010303	1
P08708	1xPhospho [S113(100); S115(0); T120(0); T123(0)]	0.958	-0.061902439	0.987464758
P08727	1xPhospho [S9(0); S10(0); T12(0.2); S13(96.2); S14(3.7); S22(0)]	0.01	-6.64385619	8.35E-17
P08727	1xPhospho [S382(0); Y391(0); S395(0); S397(100)]	0.778	-0.36215794	0.908655869
P09543	1xPhospho [S170(100)]	0.636	-0.652901329	0.898100811
P09661	1xPhospho [S178(1.7); T180(98.3); T189(0)]	1.022	0.031395196	0.963131675
P09661	1xPhospho [S178(0); T180(100); T189(0)]	0.842	-0.248107862	0.967977286
P0C1Z6	2xPhospho [Y244(0); T246(0); S249(100); S252(100)]	0.605	-0.724992953	0.827205803
P0CI00	1xPhospho [S91(100)]	1.028	0.039840265	0.998086195
P0DJ93	3xPhospho [T48(0); S50(0); S58(100); S60(100); T62(100); T66(0); S67(0); S68(0); S69(0)]	0.38	-1.395928676	0.306672643
P0DJ93	2xPhospho [T48(0); S50(0); S58(100); S60(0.1); T62(99.9); T66(0); S67(0); S68(0); S69(0)]	1.534	0.617298483	0.67428256
P10412	1xMet-loss [N-Term]; 1xPhospho [S2(0); T4(0); T18(100)]	1.864	0.89840186	0.504163686
P10412	1xMet-loss+Acetyl [N-Term]; 1xPhospho [S2(0); T4(0); T18(100)]	1.753	0.809825996	0.579751615
P10412	1xMet-loss+Acetyl [N-Term]; 1xPhospho [S2(0); T4(0); T18(100)]	1.481	0.566571641	0.721876373
P10412	1xPhospho [S36(100); S41(0); T45(0)]; P16403 1xPhospho [S36(100); S41(0); T45(0)]	1.532	0.615416297	0.769621572
P10412	1xPhospho [S36(100); S41(0); T45(0)]; P16403 1xPhospho [S36(100); S41(0); T45(0)]	1.425	0.510961919	0.797921038
P10644	1xPhospho [T75(0); S77(0); S83(100)]	1.373	0.457331625	0.792141506
P10644	1xPhospho [S83(100)]	1.185	0.244887059	0.94813191
P10644	2xPhospho [T75(2.3); S77(97.7); S83(100)]	1.136	0.183962835	0.962617576
P11137	2xPhospho [T1613(0); T1616(100); T1619(100); S1621(0); Y1622(0)]	100	6.64385619	8.35E-17
P11137	3xPhospho [T1780(0); S1782(100); S1786(50); S1787(50); S1790(100)]	1.182	0.241230036	0.91281798
P11137	2xPhospho [S1347(100); S1353(100); S1362(0); Y1364(0)]	0.689	-0.537424112	0.920253354
P11137	2xPhospho [S1588(1.8); T1590(1.8); S1591(46.5); T1592(46.5); T1594(1.8); T1595(1.8); S1598(0); T1599(0); T1602(0); T1605(99.9); S1608(0.2); Y1609(0); S1610(0); S1611(0)]	0.749	-0.416962376	0.920518605
P11137	2xPhospho [T1649(100); S1653(100); T1656(0)]	0.917	-0.125006361	0.972363997
P11137	3xPhospho [T1649(100); S1653(100); T1656(100)]	0.955	-0.066427362	0.974924677
P11137	1xPhospho [S1742(100)]	0.92	-0.120294234	0.991334844
P11142	1xPhospho [Y611(0); S613(0); S633(99.2); S637(0.4); S638(0.4); T641(0)]	0.475	-1.074000581	0.24072149
P11166	1xPhospho [T234(0); T238(0); S248(100)]	100	6.64385619	8.35E-17
P11166	1xPhospho [S473(0); S475(0); T478(0); S490(100)]	100	6.64385619	8.35E-17
P11166	1xPhospho [T478(0); S490(100)]	2.128	1.089498151	0.369620915
P11274	1xPhospho [S93(0); S95(0); S122(100)]	0.01	-6.64385619	8.35E-17
P11274	2xPhospho [S215(100); S221(33.3); S222(33.3); S227(33.3); Y231(0)]	0.01	-6.64385619	8.35E-17
P11274	2xPhospho [Y455(0); S459(100); S461(99.8); S462(0.1); S463(0.1); S467(0); S468(0)]	0.01	-6.64385619	8.35E-17
P11387	1xMet-loss+Acetyl [N-Term]; 1xPhospho [S2(0); S10(100)]	0.905	-0.144010303	0.969680228
P11388	1xPhospho [Y1521(0); S1525(100)]	100	6.64385619	8.35E-17
P11388	1xPhospho [T1244(0); S1247(100)]	7.271	2.862153795	5.43E-05
P11388	1xPhospho [S1374(100); S1377(0)]	2.285	1.192194165	0.289774883
P11388	1xPhospho [S1387(0); S1391(0.1); S1392(2.9); S1393(97.1); T1397(0); T1403(0); T1406(0)]	1.953	0.965691949	0.43602229
P11388	3xPhospho [T1327(0); S1332(100); S1337(100); T1343(100); S1351(0); S1354(0)]	0.79	-0.340075442	0.945786032
P11388	1xPhospho [T1244(0); S1247(100)]	1.097	0.133563526	0.956843841
P11388	2xPhospho [S1374(100); S1377(100)]	1.014	0.020057652	0.979586453
P11388	2xPhospho [S1387(0); S1391(0.1); S1392(99.9); S1393(100); T1397(0); T1403(0); T1406(0)]	0.939	-0.090802937	0.99184412
P11474	3xPhospho [S19(100); S22(0.1); S26(96.2); S27(51.7); T29(51.7); T31(0.3); T44(0)]	0.63	-0.666576266	0.855534442
P11717	1xPhospho [S2400(0); S2401(0); S2409(100); T2415(0)]	0.604	-0.727379545	0.622135501
P11717	2xPhospho [S2479(100); S2484(100)]	0.52	-0.943416472	0.627840228
P11717	1xPhospho [S2479(0); S2484(100)]	0.574	-0.800877358	0.641603458
P11717	2xPhospho [S2400(97.6); S2401(2.5); S2409(100); T2415(0)]	0.691	-0.533242384	0.823929745
P11912	2 2xPhospho [Y74(50); T75(50); T102(100)]	0.516	-0.954557029	0.690725835

P12270	1xPhospho [T2146(0); S2155(100)]	0.01	-6.64385619	8.35E-17
P12270	1xPhospho [S2034(0); S2037(0); T2042(0); S2047(98.5); S2048(1.5); S2050(0); S2054(0)]	1.716	0.779049553	0.585755693
P12270	1xPhospho [S909(0); S917(1.4); S919(1.4); S920(97.2)]	0.694	-0.526992432	0.921519407
P12270	1xPhospho [T2175(0); S2176(0); S2177(0); S2178(0); S2180(0); S2187(100); Y2194(0); T2196(0); S2206(0)]	1	0	0.97137454
P12931	2xPhospho [S17(100); S35(0); T37(0); S39(0); S43(100)]	0.404	-1.307572802	0.134803591
P12931	2xPhospho [S17(100); S35(0); T37(0); S39(0); S43(100)]	2.009	1.006477564	0.406144957
P12931	1xPhospho [S17(100); S35(0); T37(0); S39(0); S43(0)]	1.047	0.066261442	0.972294713
P13051	2xPhospho [T60(100); S63(0); S64(100); S67(0)]	0.599	-0.739372092	0.525769281
P13051	1xPhospho [T60(100); S63(0); S64(0); S67(0)]	0.739	-0.436353731	0.939053555
P13051	1xPhospho [S23(100); T31(0); S40(0)]	1.018	0.025737561	0.980895643
P13521	1xPhospho [S259(0); T261(0); S268(100)]	1.018	0.025737561	0.973763863
P13639	1xPhospho [Y434(0); T435(100)]	100	6.64385619	8.35E-17
P13667	1xPhospho [S468(2.1); S470(97.9); S482(0)]	0.657	-0.606034724	0.91490394
P13746	2xPhospho [S343(0); Y344(0); T345(0); S349(0); S350(0); S352(0); S356(100); S359(99.9); T361(0.1)]	0.01	-6.64385619	8.35E-17
P13746	2xPhospho [S343(0); Y344(0); T345(0); S349(0); S350(0); S352(0); S356(100); S359(100); T361(0)]	0.922	-0.117161344	0.99319806
P13807	2xPhospho [S710(0); T713(0); T715(0); S716(0); S717(0); S718(0); S720(0); T721(0); S723(0); S727(100); T729(0.1); S730(3.5); S731(96.3)]	1.18	0.23878686	0.941100356
P13807	3xPhospho [Y636(0); S641(100); S645(100); S647(100); S649(0.1)]	0.929	-0.106249498	0.999263007
P13984	1xPhospho [S135(0); S136(0); S142(100)]	0.711	-0.492078535	0.933452347
P14618	1xPhospho [S37(100); T41(0)]	0.863	-0.212567535	0.980895643
P14866	1xPhospho [T63(100); Y92(0)]	100	6.64385619	8.35E-17
P14866	1xPhospho [T63(100); Y92(0)]	1.505	0.589763487	0.679191074
P14868	1xPhospho [Y245(0); S249(100); Y253(0)]	1.16	0.214124805	0.945786032
P15056	2xPhospho [S394(0); T395(0); T396(0); S399(0.2); T401(99.9); S405(100); S409(0); T411(0)]	0.632	-0.662003536	0.886113781
P15056	1xPhospho [S363(0.1); S364(0.1); S365(99.9); T373(0)]	0.781	-0.356605547	0.965139891
P15311	1xPhospho [T533(0); S535(0); S536(0); S539(100)]	0.538	-0.894321922	0.755716034
P15407	2xPhospho [S251(3.5); S252(3.5); S253(3.5); S254(3.5); S255(86.1); S259(0); S260(0); S265(100); T267(0)]	5.474	2.452595435	0.006746107
P15407	1xPhospho [S101(100)]	3.942	1.978927776	0.017241331
P15408	1xPhospho [S200(100)]	0.567	-0.81857936	0.827205803
P15923	1xPhospho [S379(97.9); S381(2.1); Y382(0); S391(0)]	1.374	0.458382004	0.810156821
P15924	2xPhospho [S2815(0); Y2817(0); S2820(0); S2821(100); S2825(100)]	0.573	-0.803392956	0.719382659
P15924	2xPhospho [S2815(0); Y2817(0); S2820(1.9); S2821(98.1); S2825(100)]	1.072	0.100304906	0.965139891
P15924	1xPhospho [S2207(0); S2209(100)]	1.05	0.070389328	0.977860291
P15924	1xPhospho [Y172(0); T173(0); S176(99.9); S178(0.1); T184(0)]	0.825	-0.277533976	0.980895643
P15924	2xPhospho [S165(100); S166(100); Y172(0); T173(0); S176(0); S178(0); T184(0)]	0.884	-0.177881725	0.980895643
P16383	2xPhospho [T192(100); T197(100)]	2.028	1.020057652	0.562720839
P16383	2xPhospho [S419(0); T428(0); S429(100); S430(100); S436(0)]	0.674	-0.569179503	0.862322824
P16383	2xPhospho [T92(0.1); S96(89.4); T97(89.4); S106(15.7); S107(2.7); S109(2.7); S116(0); S117(0); S119(0); S120(0); S121(0); S122(0)]	1.198	0.260627908	0.945786032
P16383	2xPhospho [T92(0); S96(100); T97(100); S106(0); S107(0); S109(0)]	1.004	0.005759269	1
P16403	1xMet-loss+Acetyl [N-Term]; 1xPhospho [S2(100); T4(0)]	1.543	0.625738062	0.755586549
P16949	1xPhospho [S31(0); S38(100)]	0.608	-0.717856771	0.719352351
P16949	1xPhospho [S28(0); S31(0); S38(100)]	1.473	0.55875743	0.73347811
P16949	2xPhospho [S28(0); S31(100); S38(100)]	0.629	-0.668868078	0.783976635
P16949	1xPhospho [S16(100); S25(0)]	1.347	0.429749851	0.839392617
P16949	1xPhospho [S31(0); S38(100)]	1.048	0.067638717	0.960178862
P16949	2xPhospho [S16(100); S25(100)]	1.093	0.128293401	0.970944072
P16949	1xPhospho [S46(100)]	1.037	0.052415894	0.971220368
P16949	2xPhospho [S16(100); S25(100)]	0.919	-0.121863233	0.973263372
P16949	1xPhospho [S16(0); S25(100)]	0.957	-0.06340917	0.984563609
P16989	1xPhospho [S250(100)]	2.038	1.027154052	0.727800493
P16989	1xPhospho [Y192(0); S201(0); S203(100); S204(0); T212(0)]	0.919	-0.121863233	0.991765633
P16989	2xPhospho [Y192(0); S201(50); S203(50); S204(100); T212(0)]	0.972	-0.040971781	1
P17096	2 3xPhospho [S88(100); S91(100); S92(100)]; P17096 3xPhospho [S99(100); S102(100); S103(100)]	0.067	-3.899695094	4.46E-09
P17096	2 3xPhospho [S88(100); S91(100); S92(100)]; P17096 3xPhospho [S99(100); S102(100); S103(100)]	0.278	-1.846843212	0.002671256
P17096	2 1xPhospho [S88(0); S91(100); S92(0)]; P17096 1xPhospho [S99(0); S102(100); S103(0)]	2.339	1.225891862	0.103610713
P17096	1xPhospho [S36(0); T39(0); S44(100); S49(0); T53(0)]	0.584	-0.775959726	0.66184163
P17096	2 2xPhospho [S38(100); T42(100)]	1.686	0.753604536	0.706843266
P17096	1xPhospho [S36(0); T39(0); S44(100); S49(0); T53(0)]	0.7	-0.514573173	0.753411074
P17096	2xPhospho [S36(100); T39(0); S44(100); S49(0); T53(0)]	0.708	-0.498178735	0.767320275
P17096	2 1xPhospho [S38(0); T42(100)]	1.428	0.513995979	0.795092507
P17096	2xPhospho [S36(99.8); T39(0.2); S44(100); S49(0); T53(0)]	0.667	-0.584241333	0.806224461
P17096	3xPhospho [S36(100); T39(0); S44(3.9); S49(96.2); T53(100)]	0.68	-0.556393349	0.872730156
P17096	1xPhospho [S36(100); T39(0); S44(0)]	1.333	0.41467678	0.879063032
P17096	2 2xPhospho [S88(0); S91(100); S92(100)]; P17096 2xPhospho [S99(0); S102(100); S103(100)]	0.816	-0.293358943	0.91281798
P17096	1xPhospho [S36(0); T39(0); S44(100)]	1.084	0.116364757	0.977823205
P17096	2 1xPhospho [S88(0); S91(98.6); S92(1.4)]; P17096 1xPhospho [S99(0); S102(98.6); S103(1.4)]	0.915	-0.128156351	0.99184412
P17096	2 2xPhospho [S88(0); S91(100); S92(100)]; P17096 2xPhospho [S99(0); S102(100); S103(100)]	1.014	0.020057652	1
P17275	2xPhospho [S251(0); T255(100); S259(100)]	100	6.64385619	8.35E-17
P17275	2xPhospho [S251(0); T255(100); S259(100)]	0.741	-0.432454552	0.890244073
P17275	3xPhospho [S251(100); T255(100); S259(100)]	1.308	0.387362541	0.894195982

P17275	2xPhospho [T255(100); S259(100)]	1.142	0.191562651	0.958702638
P17302	2xPhospho [S325(95.6); T326(4.5); S328(50); S330(50); S344(0)]	0.01	-6.64385619	8.35E-17
P17535	2xPhospho [T245(0); S251(0); S255(100); S259(100); T265(0)]	0.761	-0.394031641	0.907632224
P17535	2xPhospho [T245(0); S251(0); S255(100); S259(100); T265(0)]	0.961	-0.057391664	1
P17987	1xPhospho [S544(100); Y545(0); S551(0)]	1.915	0.937344392	0.516216698
P17987	1xPhospho [S544(0); Y545(0); S551(100)]	1.729	0.789937869	0.573612996
P18583	3xPhospho [S1766(0); S1769(0); S1773(0.3); S1774(0.3); S1780(53); S1782(53); S1783(96.7); S1784(96.7); Y1790(0)]	0.01	-6.64385619	8.35E-17
P18583	2xPhospho [S2029(100); S2031(100)]	2.248	1.168642036	0.134520999
P18583	1xPhospho [S2129(100); Y2139(0)]	1.982	0.986956963	0.41370633
P18583	3xPhospho [S1948(100); S1950(100); S1952(50); S1954(50)]	1.417	0.502839758	0.775732596
P18583	3xPhospho [S1766(0); S1769(0); S1773(0); S1774(0); S1780(100); S1782(100); S1783(100); S1784(0); Y1790(0)]	0.659	-0.60164963	0.855816069
P18583	2xPhospho [S1688(0); T1691(0); S1697(51.2); S1701(51.2); S1702(97.6)]	0.639	-0.646112164	0.907925578
P18583	3xPhospho [S1948(100); S1950(100); S1952(100); S1954(0.1)]	0.774	-0.369594529	0.960831064
P18583	1xPhospho [S1688(0); T1691(0); S1697(100)]	0.752	-0.411195433	0.961452497
P18583	2xPhospho [S1780(0); S1782(100); S1783(100); S1784(0); Y1790(0)]	0.845	-0.242976753	0.963131675
P18583	2xPhospho [S2009(0); S2011(100); S2013(100)]	1.139	0.187767747	0.965139891
P18583	3xPhospho [S2009(100); S2011(100); S2013(100)]	0.946	-0.080087911	0.980895643
P18583	1xPhospho [S1670(100); S1676(0)]	0.821	-0.284545873	0.990182098
P18583	1xPhospho [S2097(2.4); T2101(97.6); T2106(0)]	0.866	-0.20756107	1
P18615	1xPhospho [S131(100); Y133(0); S135(0); S138(0); S139(0); S140(0)]	1.057	0.079975377	0.952591019
P18615	2xPhospho [S131(100); Y133(0); S135(100); S138(0); S139(0); S140(0)]	0.839	-0.253257284	0.991765633
P18615	1xPhospho [S343(0); S347(0); S353(100)]	0.891	-0.166502663	0.999263007
P18669	1xPhospho [S14(100)]	1.324	0.404903122	0.854283637
P18669	1xPhospho [S118(100); Y119(0); Y133(0); S134(0); S137(0)]	0.853	-0.229382353	0.97023559
P18669	1xPhospho [S118(100); Y119(0); Y133(0); S134(0); S137(0)]	1.01	0.014355293	0.982029512
P18754	1xPhospho [S11(100)]	1.646	0.718964336	0.656697695
P18887	2xPhospho [T257(100); S259(0); S266(100); S268(0)]	1.425	0.510961919	0.793785899
P18887	1xPhospho [T437(0); T440(0); S446(0.1); S447(100); T453(0); T457(0)]	1.313	0.392866916	0.832048448
P18887	3xPhospho [S461(100); S475(0); S485(100); T488(100)]	1.416	0.501821265	0.848293321
P18887	2xPhospho [T198(96.2); S199(3.8); T202(0); S204(0); S210(0); Y211(0); T215(0); S219(0); S220(0); S223(0); S224(0); S226(96.2); S229(3.8)]	0.621	-0.687334826	0.853656829
P18887	2xPhospho [T437(0); T440(94.1); S446(3); S447(3); T453(3.1); T457(97)]	1.091	0.125651102	0.974924677
P18887	1xPhospho [T257(0); S259(0); S266(100); S268(0)]	0.948	-0.077041036	1
P19174	1xPhospho [S1233(100); S1236(0)]	0.955	-0.066427362	0.980895643
P19174	1xPhospho [S1263(100); S1273(0)]	0.932	-0.10159814	0.991765633
P19338	1xPhospho [S145(0); S153(100)]	0.01	-6.64385619	8.35E-17
P19338	2xPhospho [S28(100); S34(100); S41(0); S42(0)]	0.01	-6.64385619	8.35E-17
P19338	3xPhospho [S28(100); S34(100); S41(99.6); S42(0.4)]	0.287	-1.800877358	0.028487733
P19338	3xPhospho [S28(100); S34(100); S41(50); S42(50)]	0.396	-1.336427665	0.102761382
P19338	3xPhospho [S28(100); S34(100); S41(95.5); S42(4.5)]	0.546	-0.873027144	0.394158532
P19338	3xPhospho [S28(100); S34(100); S41(99.8); S42(0.2)]	0.581	-0.783389931	0.524223839
P19338	2xPhospho [S145(100); S153(100)]	0.611	-0.710755715	0.560268747
P19338	2xPhospho [S145(100); S153(100)]	0.712	-0.490050854	0.774223796
P19338	2xPhospho [S184(100); S206(100); T213(0); T214(0)]	0.741	-0.432454552	0.904079048
P19338	1xPhospho [S184(0); S206(100); T213(0); T214(0)]	1.074	0.102993993	0.971220368
P19338	2xPhospho [S184(100); S206(100); T213(0); T214(0)]	1.015	0.021479727	1
P19838	2xPhospho [T897(0); T898(0); S899(0); S903(100); S907(100); S910(0); T911(0)]	0.911	-0.134477041	0.983706057
P20042	1xPhospho [S105(100); T111(0)]	0.636	-0.652901329	0.855185501
P20042	1xPhospho [S105(100); T111(0)]	0.77	-0.377069649	0.964495484
P20290	1xPhospho [T28(0.1); S30(100)]	17.944	4.16542962	1.09E-05
P20700	2xPhospho [S391(100); S393(100); S395(0); S396(0)]; J9JID7 2xPhospho [S405(100); S407(100); S409(0); S410(0)]	1.949	0.962734091	0.296268685
P20700	1xPhospho [S391(100); S393(0); S395(0); S396(0)]; J9JID7 1xPhospho [S405(100); S407(0); S409(0); S410(0)]	1.509	0.593592806	0.726057431
P21333	1xPhospho [S2152(100); S2158(0); S2163(0)]	0.706	-0.502259911	0.862322824
P21333	1xPhospho [S2152(0); S2158(100); S2163(0)]	0.761	-0.394031641	0.894035878
P21333	2xPhospho [S2152(100); S2158(100); S2163(0)]	0.781	-0.356605547	0.933452347
P21709	2xPhospho [S906(100); S908(50); S910(50); Y915(0)]	0.538	-0.894321922	0.81248148
P21796	1xPhospho [T98(0); S101(0); S102(0); S104(100); T107(0)]	1.48	0.565597176	0.766139312
P22059	2xPhospho [S190(100); S193(100); S198(0); S200(0); T202(0); T205(0); T210(0)]	0.607	-0.720231578	0.548439372
P22059	2xPhospho [S190(100); S193(100); S198(0); S200(0); T202(0); T205(0); T210(0)]	0.562	-0.831357964	0.559108689
P22059	2xPhospho [S190(100); S193(100); S198(0); S200(0); T202(0)]	0.476	-1.070966521	0.701261726
P22059	1xPhospho [S351(100); T367(0)]	0.784	-0.351074441	0.879063032
P22059	1xPhospho [S351(100); T367(0)]	0.761	-0.394031641	0.93019184
P22059	2xPhospho [S190(100); S193(100); S198(0); S200(0); T202(0)]	1.231	0.299830762	0.93019184
P22059	3xPhospho [T377(0.1); S379(99.9); S382(100); S385(99.9); S386(0.1); S389(0); Y394(0)]	0.737	-0.440263476	0.938421269
P22059	2xPhospho [T377(99.9); S379(0.1); S382(100); S385(0); S386(0); S389(0); Y394(0)]	1.009	0.012926174	0.973763863
P22314	2xPhospho [S810(0); S816(100); S820(100); S824(0)]	0.265	-1.915935735	0.012740945
P22314	1xPhospho [S13(100); S21(0); S24(0); S28(0); S31(0); S35(0); T38(0)]	3.278	1.712815854	0.038581838
P22314	1xPhospho [S46(100); Y55(0); S56(0)]	1.065	0.09085343	0.958702638
P22314	2xPhospho [S13(100); S21(50); S24(50); S28(0); S31(0); S35(0); T38(0)]	0.838	-0.254977851	0.964978421
P22612	1xPhospho [T196(1.8); T198(98.2); T202(0); Y205(0); S213(0)]	2.108	1.075874867	0.408944629

P22626	1xPhospho [Y244(0); Y247(0); S259(100); Y262(0)]	1.915	0.937344392	0.287183793
P22626	1xPhospho [S225(100)]	1.942	0.957543201	0.301780562
P22626	2xPhospho [Y331(0); Y336(0); S341(100); S344(100); Y347(0)]	2.343	1.228356954	0.312106436
P22626	1xPhospho [Y331(0); Y336(0); S341(0); S344(100); Y347(0)]	1.819	0.863145543	0.399827263
P22626	1xPhospho [S212(100)]	1.531	0.614474283	0.651285809
P22626	1xPhospho [Y331(0); Y336(0); S341(0); S344(100); Y347(0)]	0.826	-0.275786313	0.965139891
P22681	1xPhospho [S483(100); S486(0); S492(0)]	100	6.64385619	8.35E-17
P23246	1xPhospho [Y642(0); T652(0); S654(0.2); S656(99.8); S660(0)]	3.97	1.989139007	0.027410315
P23246	2xPhospho [Y642(0); T652(50); S654(50); S656(11.3); S660(88.7)]	1.085	0.117695043	0.972363997
P23396	2xPhospho [S83(47.2); Y87(5.7); T93(47.2); S104(100)]	0.01	-6.64385619	8.35E-17
P23396	2xPhospho [S209(100); T220(4.1); T221(95.7); S224(0.2)]	100	6.64385619	8.35E-17
P23396	1xPhospho [S209(100)]	0.651	-0.619270551	0.809185244
P23396	1xPhospho [T242(100)]	1.064	0.089498151	0.971838536
P23396	2xPhospho [T220(0); T221(100); S224(100)]	0.991	-0.013043037	0.980895643
P23497	3xPhospho [S407(100); S409(100); S410(100); S419(0); S420(0)]	0.01	-6.64385619	8.35E-17
P23497	1xPhospho [S350(2.3); S362(96.9); T372(0.4); S374(0.4)]	0.01	-6.64385619	8.35E-17
P23497	1xPhospho [S350(0); S362(100); T372(0); S374(0)]	0.386	-1.373327247	0.252662533
P23497	3xPhospho [S407(100); S409(100); S410(100); S419(0); S420(0)]	0.337	-1.569179503	0.458787027
P23497	1xPhospho [S18(100); S31(0)]	0.833	-0.263611599	0.980895643
P24844	1xPhospho [T19(2.5); S20(97.6); S29(0)]	1.147	0.197865391	0.962617576
P24844	1xPhospho [T19(2.3); S20(97.7); S29(0)]	0.865	-0.209227962	0.973763863
P24928	2xPhospho [Y1874(0); S1875(0); T1877(0); S1878(100); T1880(0); Y1881(0); S1882(49.5); T1884(49.5); T1885(1)]	1.41	0.495695163	0.797966154
P24928	2xPhospho [Y1909(0); S1910(0); T1912(0); S1913(97.4); T1915(2.5); Y1916(0); S1917(0.1); T1919(2.5); S1920(97.5)]; 2xPhospho [Y1923(0); S1924(0); T1926(0); S1927(97.4); T1929(2.5); Y1930(0); S1931(0.1); T1933(2.5); S1934(97.5)]	1.125	0.169925001	0.964221121
P24928	2xPhospho [Y1888(0); S1889(0); T1891(3.9); S1892(96.1); T1894(0); Y1895(0); S1896(0); T1898(0); S1899(0); Y1902(0); T1903(0); T1905(3.9); S1906(96.1)]	0.796	-0.329159664	0.972363997
P24928	1xPhospho [Y1874(0); S1875(0); T1877(0); S1878(100); T1880(0); Y1881(0); S1882(0); T1884(0); T1885(0)]	0.964	-0.052894948	0.980895643
P24928	2xPhospho [Y1839(0); T1840(0); T1842(0); S1843(99.9); S1845(0.1); Y1846(0); S1847(0); S1849(0); S1850(100); Y1853(0); T1854(0); T1856(0); S1857(0)]	0.929	-0.106249498	1
P24941	2xPhospho [T14(100); Y15(100); Y19(0)]; P06493 2xPhospho [T14(100); Y15(100); Y19(0)]	0.748	-0.418889825	0.835933921
P24941	1xPhospho [T14(100); Y15(0); Y19(0)]; P06493 1xPhospho [T14(100); Y15(0); Y19(0)]	1.207	0.271425676	0.935170018
P25054	1xPhospho [S770(0); T772(0); S780(100)]	100	6.64385619	8.35E-17
P25054	3xPhospho [S2088(100); S2093(100); S2096(100)]	0.646	-0.63039393	0.861057798
P25054	2xPhospho [S2531(0); S2533(100); S2535(100); S2537(0.1)]	0.632	-0.662003536	0.879063032
P25054	1xPhospho [S2110(0); S2113(0); S2114(0); S2125(100)]	1.134	0.18142064	0.953387553
P25054	3xPhospho [S2459(6.7); S2461(0); S2464(93.3); S2466(0); S2468(6.7); S2469(93.3); S2473(99.5); T2475(0.5)]	1.058	0.081339627	0.960298686
P25054	2xPhospho [S2459(0); S2461(0); S2464(0); S2466(0); S2468(3); S2469(99.9); S2473(94.1); T2475(2.9)]	1.09	0.124328135	0.96308594
P25054	2xPhospho [S2088(100); S2093(100); S2096(0)]	1.145	0.195347598	0.965013037
P25054	1xPhospho [S744(100); S747(0); S748(0); S751(0)]	1.016	0.022900402	0.979498769
P25098	1xPhospho [S670(100); S676(0)]	1.621	0.696884091	0.682660189
P25705	1xPhospho [S39(2.9); T41(97.1)]	1.913	0.935836874	0.712836242
P25788	1xPhospho [S243(2.5); S250(97.5)]	0.781	-0.356605547	0.94797789
P26196	1xPhospho [T28(0); T36(100); T38(0)]	2.764	1.466757616	0.179323131
P26196	1xPhospho [T48(0); T50(0); T55(0); S61(100); T63(0); T64(0); T65(0)]	1.901	0.926758532	0.31700683
P26196	1xPhospho [T48(0); T50(0); T55(0); S61(99.9); T63(0.1); T64(0); T65(0)]	1.63	0.704871964	0.699388119
P26196	1xPhospho [T6(0); S15(0); S16(100)]	1.331	0.412510571	0.863653123
P26358	1xPhospho [S714(100)]	2.728	1.447843644	0.111823236
P26358	2xPhospho [S127(100); S133(50); T137(50)]	2.279	1.188400925	0.425201113
P26358	1xPhospho [S127(100); S133(0)]	1.613	0.689746438	0.636840295
P26358	1xPhospho [S714(100)]	1.606	0.683471893	0.649239517
P26368	1xPhospho [S79(100)]	1.355	0.438292852	0.938190912
P26368	1xPhospho [S79(100)]	1.095	0.13093087	0.973763863
P26368	1xMet-loss+Acetyl [N-Term]; 1xPhospho [S2(100)]	0.953	-0.069451881	1
P26373	1xPhospho [S106(100); T107(0); S109(0)]	1.612	0.688851744	0.749797187
P26599	1xPhospho [S16(100); S21(0); T22(0); T25(0); S32(0); S33(0); S35(0); S37(0); S44(0)]	2.004	1.002882509	0.549598365
P26641	1xPhospho [T46(100)]	1.025	0.03562391	0.973263372
P27348	1xPhospho [T226(0); T229(2.8); S230(94.3); S232(2.8)]	1.499	0.584000383	0.763329142
P27448	2xPhospho [S42(100); S45(100); Y56(0)]; P27448-7 2xPhospho [S42(100); S45(100); Y56(0)]	1.376	0.46048047	0.792141506
P27448	2xPhospho [T574(0); T576(0); Y577(0); S583(100); S585(0); S587(100); T591(0); S594(0); T596(0)]; P27448-7 2xPhospho [T581(0); T583(0); Y584(0); S590(100); S592(0); S594(100); T598(0); S601(0); T603(0)]	0.701	-0.512513651	0.819921743
P27448	7 1xPhospho [S391(0); S392(0); S397(0); S398(0); S403(0); T404(0); S407(100)]	1.46	0.545968369	0.855113813
P27448	1xPhospho [S390(1.9); S391(98.1); S396(0); T397(0); S400(0)]	1.291	0.368489001	0.879063032
P27448	2xPhospho [T536(0); S540(100); T541(0); S543(100); S545(0); S546(0); T549(0)]; P27448-7 2xPhospho [T543(0); S547(100); T548(0); S550(100); S552(0); S553(0); T556(0)]	0.942	-0.086201035	0.976345951
P27708	1xPhospho [S1900(100); T1906(0); T1914(0); S1915(0); S1920(0); S1928(0); T1933(0)]	1.929	0.947853143	0.47518367
P27708	1xPhospho [S1859(100)]	1.169	0.22527493	0.950730609
P27708	1xPhospho [S1859(100)]	0.838	-0.254977851	0.965013037

P27816	2xPhospho [T909(0); S910(0); S914(50); S915(50); T916(0.1); T917(0); S921(100)]; E7EVA0 2xPhospho [T2054(0); S2055(0); S2059(50); S2060(50); T2061(0.1); T2062(0); S2066(100)]	3.738	1.902266569	0.061162219
P27816	1xPhospho [S68(0); T70(0); S71(0); T76(0); S78(0); S79(0); T82(0); S94(0); T96(0); T97(0); S99(99.6); T101(0.4)]	3.326	1.733788169	0.066164317
P27816	1xPhospho [S358(100); S367(0)]; 1xPhospho [S384(100); S393(0)]; E7EVA0 1xPhospho [S375(100); S384(0)]; 1xPhospho [S401(100); S410(0)]	2.494	1.318461465	0.255980734
P27816	2xPhospho [S507(100); S510(0); T512(0); T521(100); T526(0)]; E7EVA0 2xPhospho [S524(100); S527(0); T529(0); T538(100); T543(0)]	1.894	0.921436331	0.551021356
P27816	2xPhospho [S507(100); S510(100); T512(0)]; E7EVA0 2xPhospho [S524(100); S527(100); T529(0)]	0.512	-0.965784285	0.571547296
P27816	2xPhospho [S760(100); S764(100); S768(0)]; E7EVA0 2xPhospho [S1905(100); S1909(100); S1913(0)]	2.063	1.044743821	0.574788308
P27816	1xPhospho [S742(100); S745(0)]; E7EVA0 1xPhospho [S1887(100); S1890(0)]	1.572	0.652601218	0.725383305
P27816	1xPhospho [S825(100); S827(0); T828(0)]; E7EVA0 1xPhospho [S1970(100); S1972(0); T1973(0)]	1.484	0.569491092	0.75736259
P27816	1xPhospho [T892(0); S896(0); S899(1.6); T900(98.4); T901(0)]; E7EVA0 1xPhospho [T2037(0); S2041(0); S2044(1.6); T2045(98.4); T2046(0)]	1.417	0.502839758	0.81171187
P27816	1xPhospho [S1073(100)]; E7EVA0 1xPhospho [S2218(100)]	0.777	-0.364013496	0.925724009
P27816	1xPhospho [T925(0); T927(0); S928(100)]; E7EVA0 1xPhospho [T2070(0); T2072(0); S2073(100)]	0.763	-0.390245038	0.960831064
P27816	2xPhospho [S68(0); T70(0); S71(0); T76(0); S78(0); S79(0); T82(0); S94(99.2); T96(0.7); T97(5.8); S99(88.7); T101(5.5)]	0.885	-0.17625064	0.9616322
P27816	1xPhospho [S853(100); S859(0)]; E7EVA0 1xPhospho [S1998(100); S2004(0)]	1.114	0.155749233	0.971220368
P27816	2xPhospho [S742(100); S745(100)]; E7EVA0 2xPhospho [S1887(100); S1890(100)]	1.04	0.056583528	0.979498769
P27816	2xPhospho [S495(100); T496(100)]; E7EVA0 2xPhospho [S512(100); T513(100)]	0.835	-0.260151897	0.980895643
P27816	1xPhospho [T521(100); T526(0)]; E7EVA0 1xPhospho [T538(100); T543(0)]	0.829	-0.270555993	0.981526996
P27816	1xPhospho [S280(100); T282(0); T287(0)]; E7EVA0 1xPhospho [S297(100); T299(0); T304(0)]	0.97	-0.043943348	0.991003044
P28290	1xPhospho [S767(100); S770(0); T772(0); Y773(0)]	100	6.64385619	8.35E-17
P28290	3xPhospho [S1055(100); S1060(100); S1063(100); T1072(0); S1079(0); Y1080(0)]	0.143	-2.805912948	0.001156053
P28290	2xPhospho [S1152(0); T1156(100); T1158(0); S1161(100)]; H7BZ26 2xPhospho [S97(0); T101(100); T103(0); S106(100)]	3.521	1.815985227	0.049649837
P28290	1xPhospho [S111(100); S117(0); S128(0)]	0.321	-1.639354798	0.07457184
P28290	2xPhospho [T461(8.3); S466(91.7); S474(50); T475(50); T485(0); Y486(0); T491(0)]	1.851	0.888304895	0.543749652
P28290	3xPhospho [T1163(50); S1165(50); T1168(100); S1174(50); S1175(50)]; H7BZ26 3xPhospho [T108(50); S110(50); T113(100); S119(50); S120(50)]	0.502	-0.994240731	0.548348393
P28290	2xPhospho [T87(100); S92(100); S97(0); S98(0); S99(0); T100(0)]	1.741	0.799916203	0.563415239
P28290	1xPhospho [S1152(0); T1156(0); T1158(0); S1161(100)]; H7BZ26 1xPhospho [S97(0); T101(0); T103(0); S106(100)]	1.79	0.839959587	0.590872099
P28290	3xPhospho [S1152(86.2); T1156(86.2); T1158(17.6); S1161(4.5); T1163(4.5); S1165(3.7); T1168(97.4); S1174(0); S1175(0)]; H7BZ26 3xPhospho [S97(86.2); T101(86.2); T103(17.6); S106(4.5); T108(4.5); S110(3.7); T113(97.4); S119(0); S120(0)]	0.557	-0.844250767	0.769999353
P28290	3xPhospho [S270(96.1); S272(7.8); S273(96.1); T275(50); S276(50); T280(0); T287(0)]	0.621	-0.687334826	0.774332996
P28290	2xPhospho [S270(100); S272(0.2); S273(99.7); T275(0.2); S276(0); T280(0); T287(0)]	1.396	0.481298942	0.870911172
P28290	1xPhospho [T87(0); S92(100); S97(0); S98(0); S99(0); T100(0)]	1.333	0.41467678	0.885499461
P28290	2xPhospho [S419(0); S424(100); S425(0); S427(100); S432(0); S433(0)]	0.681	-0.554273297	0.900511889
P28290	3xPhospho [S270(0); S272(100); S273(100); T275(0.2); S276(99.6); T280(0.2); T287(0)]	0.719	-0.475936324	0.919907076
P28290	2xPhospho [T864(3); S866(93.9); T867(3.1); S869(99.9); S874(0); T877(0); S879(0); S883(0); Y889(0); S890(0)]	0.749	-0.416962376	0.921519407
P28290	1xPhospho [T399(0); S401(2.7); S404(97.4); S410(0); S415(0)]	1.11	0.150559677	0.93019184
P28290	1xPhospho [S380(0); S385(100); T391(0); S396(0)]	1.119	0.162210036	0.930376591
P28290	2xPhospho [T87(0); S92(100); S97(100); S98(0); S99(0); T100(0)]	0.784	-0.351074441	0.938730361
P28290	2xPhospho [T1163(50); S1165(50); T1168(100); S1174(0); S1175(0)]; H7BZ26 2xPhospho [T108(50); S110(50); T113(100); S119(0); S120(0)]	0.784	-0.351074441	0.94797789
P28290	1xPhospho [S737(0); S739(100); T742(0); T743(0); S746(0)]	1.116	0.158337027	0.94797789
P28290	1xPhospho [T461(0.2); S466(99.9); S474(0); T475(0); T485(0); Y486(0); T491(0)]	0.804	-0.314732593	0.956843841
P28290	2xPhospho [S111(99.6); S117(50.2); S128(50.2)]	0.77	-0.377069649	0.971838536
P28290	1xPhospho [S466(100); S474(0); T475(0); T485(0); Y486(0); T491(0)]	1.051	0.071762669	0.973942845
P28290	1xPhospho [T298(0); S300(100)]	0.777	-0.364013496	0.980895643
P28290	1xPhospho [S665(0); S668(100)]	0.783	-0.352915787	0.980895643
P28290	1xPhospho [S591(97.9); S593(2.1); T601(0); T605(0); T607(0)]	0.972	-0.040971781	0.985461924
P28290	2xPhospho [S737(0); S739(100); T742(0); T743(0); S746(100)]	0.923	-0.115597447	0.994656428
P28370	2xPhospho [T102(0); S112(0.4); S116(93.7); T118(5.9); S119(100)]	0.067	-3.899695094	9.82E-05
P28715	2xPhospho [S558(0); S559(0); S562(100); S563(100); T567(0)]	1.252	0.324234562	0.887088694
P28749	2xPhospho [S1037(100); S1041(100)]	100	6.64385619	8.35E-17
P28749	1xPhospho [S980(0); S984(0); Y986(0); S988(100)]	1.276	0.351628329	0.921446515
P28749	2xPhospho [S1037(100); S1041(100)]	0.677	-0.562772261	0.962617576
P28749	2xPhospho [S749(100); S752(0); T754(0); S757(0); S762(100)]	0.849	-0.236163541	0.980895643
P28749	2xPhospho [S980(0); S984(100); Y986(0); S988(100)]	0.949	-0.075520008	1
P29083	1xPhospho [T223(0); T224(0); S229(100)]	3.264	1.706641057	0.086050391
P29084	1xPhospho [S55(0); S58(2.4); S61(97.6)]	0.928	-0.10780329	0.989102507
P29317	3xPhospho [S897(100); T898(0); S899(100); S901(100)]	0.45	-1.152003093	0.498736736
P29317	2xPhospho [S897(0); T898(0); S899(100); S901(100)]	0.898	-0.15521265	0.966027585
P29317	1xPhospho [S897(100); T898(0); S899(0); S901(0)]	0.853	-0.229382353	0.973763863
P29350	1xPhospho [Y564(100); T569(0)]	1.157	0.210388864	0.965013037
P29590	3xPhospho [S518(100); S527(100); S530(100); S535(0); S542(0); S547(0)]	1.376	0.46048047	0.771511437
P29590	1xPhospho [S8(100); T22(0); T28(0); S30(0)]; H3BT57 1xPhospho [S8(100); T22(0); T28(0); S30(0)]	1.434	0.520045024	0.831039794
P29590	2xPhospho [S518(0); S527(100); S530(99.7); S535(0.3); S542(0); S547(0)]	0.687	-0.541617996	0.908086456

P29590	2xPhospho [S518(100); S527(100)]; H3BT57 2xPhospho [S470(100); S479(100)]	0.836	-0.258425153	0.94432975
P29692	2xPhospho [T147(100); S162(100)]; E9PL71 2xPhospho [T123(100); S138(100)]	1.474	0.559736524	0.739679432
P29692	1xPhospho [T147(0); S162(100)]; E9PL71 1xPhospho [T123(0); S138(100)]	1.343	0.425459305	0.8446011
P29692	1xPhospho [T147(0); S162(100)]; E9PL71 1xPhospho [T123(0); S138(100)]	1.341	0.423309237	0.847914016
P29692	1xPhospho [S60(100); S64(0); S65(0); S70(0); S71(0); T73(0); S74(0)]	1.304	0.38294387	0.862506613
P29692	2xPhospho [T147(100); S162(100)]; E9PL71 2xPhospho [T123(100); S138(100)]	1.307	0.386259141	0.868254505
P29692	1xPhospho [T125(0); T129(1.8); S133(98.3)]; E9PL71 1xPhospho [T101(0); T105(1.8); S109(98.3)]	1.241	0.311503115	0.886312012
P29692	1xPhospho [T147(0); S162(100)]; E9PL71 1xPhospho [T123(0); S138(100)]	1.211	0.276198865	0.942907978
P29692	2xPhospho [T147(100); S162(100)]; E9PL71 2xPhospho [T123(100); S138(100)]	1.084	0.116364757	0.962617576
P29966	1xPhospho [S26(0); S27(0.3); S29(99.7)]	0.01	-6.64385619	8.35E-17
P29966	2xPhospho [S163(0); S167(100); S170(100)]	0.01	-6.64385619	8.35E-17
P29966	3xPhospho [S163(100); S167(100); S170(100)]	0.081	-3.625934282	1.80E-05
P29966	2xPhospho [S163(0); S167(100); S170(100)]	0.23	-2.120294234	0.010096085
P29966	1xPhospho [S167(0); S170(100)]	0.194	-2.365871442	0.012511512
P29966	2xPhospho [S167(100); S170(100)]	0.225	-2.152003093	0.020397378
P29966	1xPhospho [S167(0); S170(100)]	0.294	-1.76611194	0.045941642
P29966	1xPhospho [S26(0); S27(100); S29(0)]	1.609	0.686164326	0.681112257
P29966	2xPhospho [S118(50); T120(50); S128(0); S131(0); S132(0); T133(0.4); S134(5.9); S135(93.7)]	0.817	-0.291592017	0.980895643
P30041	1xPhospho [S186(100); T192(0)]	0.913	-0.131313235	0.99811985
P30050	1xPhospho [S38(100)]	0.717	-0.479954976	0.929187903
P30086	1xPhospho [T51(1.2); S52(98.7); S54(0); S60(0)]	1.11	0.150559677	0.965139891
P30305	2xPhospho [S229(50); S230(50); S238(100)]	100	6.64385619	8.35E-17
P30305	1xPhospho [S373(2.3); S375(97.7); S387(0)]	100	6.64385619	8.35E-17
P30305	1xPhospho [S321(0.1); S323(99.9); S327(0)]	2.584	1.36960607	0.225233658
P30307	1xPhospho [S214(0.1); S216(99.9)]	11.088	3.470927257	7.48E-07
P30414	3xPhospho [S463(100); S467(2.3); S468(2.3); S470(97.7); S471(95.5); T472(2.3)]	0.583	-0.778432211	0.703538855
P30414	2xPhospho [S463(0); S467(3); S468(3); S470(94.1); S471(50); T472(50)]	0.534	-0.905088353	0.75891781
P30484	2xPhospho [S343(0); Y344(0); S345(0); S349(0); S350(0); S352(0); S356(100); S359(100); T361(0)]	0.01	-6.64385619	8.35E-17
P30511	1xPhospho [S216(6.2); T221(93.9)]	0.01	-6.64385619	8.35E-17
P30622	2xPhospho [T146(50); S147(50); T151(4.6); S152(0.3); T153(4.6); S155(85.9); S158(4.6); S159(0); S160(0); S162(0); T163(0); S165(0); S172(0)]	0.769	-0.378944497	0.933107775
P30622	1xPhospho [S1348(0); Y1362(0); S1364(100)]	0.804	-0.314732593	0.960178862
P30622	3xPhospho [T191(0); S193(0); S195(100); S197(0.1); S200(99.9); S204(100)]	1.08	0.111031312	0.965013037
P30622	3xPhospho [T191(0); S193(0); S195(100); S197(0); S200(100); S204(100)]	0.888	-0.171368418	0.982345843
P30622	2xPhospho [T191(0); S193(0); S195(0); S197(0); S200(100); S204(100)]	0.897	-0.15682011	0.99184412
P30622	2xPhospho [T191(0); S193(0); S195(0); S197(0); S200(100); S204(100)]	0.964	-0.052894948	0.99184412
P30989	2xPhospho [S401(99.9); S403(2.6); S404(97.4); T407(0); S409(0); S410(0); T413(0)]	0.796	-0.329159664	0.976789257
P31323	2xPhospho [S83(100); S85(100)]	0.01	-6.64385619	8.35E-17
P31323	2xPhospho [S83(100); S85(100)]	0.346	-1.531156057	0.05315148
P31350	1xPhospho [T12(0); S20(100)]	0.25	-2	0.001874325
P31350	1xPhospho [S376(3.8); S377(92.3); T379(3.8); S382(0); T384(0)]	0.688	-0.53951953	0.942907978
P31942	1xPhospho [Y262(0); S269(0); T270(0); S275(0); S280(100); Y285(0)]	0.725	-0.4639471	0.973763863
P32519	2xPhospho [T180(0); S187(100); T190(49); T191(49); S195(1.9)]	0.892	-0.164884385	0.992782906
P33176	1xPhospho [S950(100)]	1.585	0.66448284	0.617281983
P33240	2xPhospho [T507(0); S513(100); S518(0); S524(100); T531(0)]	100	6.64385619	8.35E-17
P33240	1xPhospho [T507(0); S513(0); S518(0); S524(99.6); T531(0.4)]	1.14	0.189033824	0.965139891
P33240	1xPhospho [T507(0); S513(0); S518(0); S524(96); T531(4)]	1.04	0.056583528	0.989838391
P34932	1xPhospho [T538(0); S546(99.6); T551(0.2); S552(0.2); S556(0)]	100	6.64385619	8.35E-17
P34932	1xPhospho [T822(0); T824(0); S828(100); S830(0)]	0.873	-0.195946441	0.967050102
P34932	1xPhospho [T822(0); T824(0); S828(0.1); S830(99.9)]	0.952	-0.070966521	0.989838391
P34972	3xPhospho [S335(44.9); S336(44.9); T338(7.2); T340(6.5); T347(98); S352(98.6)]	0.54	-0.888968688	0.519030203
P35222	2xPhospho [T551(97.4); S552(97.4); T556(5.3)]	100	6.64385619	8.35E-17
P35222	1xPhospho [T551(98); S552(2); T556(0)]	1.465	0.550900665	0.763548117
P35240	1xPhospho [S518(100)]	1.148	0.199122642	0.955790722
P35251	1xPhospho [T161(0); T163(0); S164(0); Y168(0); T171(0); S173(100)]	0.108	-3.210896782	0.001056314
P35251	1xPhospho [S156(100)]	2.163	1.113033665	0.392224068
P35251	2xPhospho [Y67(0); S69(100); S71(100); S73(0); T76(0)]	0.562	-0.831357964	0.53763856
P35251	2xPhospho [T105(0); Y106(0); S108(100); T110(100)]	0.481	-1.055891201	0.586560987
P35251	2xPhospho [S156(100); T161(100); T163(0); S164(0); Y168(0); T171(0); S173(0)]	1.556	0.63784206	0.724913856
P35251	3xPhospho [S156(100); T161(100); T163(0); S164(0); Y168(0); T171(0.1); S173(99.9)]	1.313	0.392866916	0.870504088
P35579	1xPhospho [S1943(100)]	0.952	-0.070966521	0.980895643
P35579	1xPhospho [S1943(100)]	0.782	-0.354759487	0.981208663
P35611	1xPhospho [T11(0); S12(100); T16(0); T17(0)]	0.589	-0.763660461	0.79866369
P35611	1xPhospho [T724(0); S726(100)]; Q9UEY8 1xPhospho [T691(0); S693(100)]	0.858	-0.220950447	0.966604693
P35611	1xPhospho [T724(0); S726(100)]; Q9UEY8 1xPhospho [T691(0); S693(100)]	0.95	-0.074000581	0.980895643
P35613	1xPhospho [S362(100)]	1.089	0.123003954	0.962617576
P35613	1xPhospho [S362(100)]	1.048	0.067638717	0.973263372
P35637	1xPhospho [S273(95.4); S277(4.4); S282(0.2); T286(0); T297(0); S300(0); Y304(0)]	0.01	-6.64385619	8.35E-17
P35637	1xPhospho [S462(100); Y468(0)]	100	6.64385619	8.35E-17
P35637	1xPhospho [S462(100); Y468(0)]	3.751	1.907275263	0.001944442
P35637	1xPhospho [S462(100); Y468(0)]	3.182	1.669933836	0.030319021
P35637	1xPhospho [S462(100); Y468(0)]	2.15	1.10433666	0.147636357
P35637	1xPhospho [S277(100); S282(0); T286(0); T297(0); S300(0); Y304(0)]	0.895	-0.160040413	1
P35658	1xPhospho [T1021(0.1); S1023(99.9); S1027(0)]	0.01	-6.64385619	8.35E-17

P35658	1xPhospho [T670(0); S678(100)]	2.964	1.567545448	0.098359932
P35658	2xPhospho [T670(100); S678(100)]	1.382	0.466757616	0.855557911
P36578	1xPhospho [S295(100)]	1.6	0.678071905	0.767755861
P36871	1xPhospho [T115(0); S117(100)]	1.028	0.039840265	0.979125671
P36915	3xPhospho [T48(100); T50(100); S51(100); S55(0); T57(0)]	9.002	3.170245565	0.001019773
P36915	2xPhospho [T48(100); T50(50); S51(50); S55(0); T57(0)]	4.747	2.24701605	0.032620614
P36915	3xPhospho [T48(100); T50(100); S51(100); S55(0); T57(0)]	0.403	-1.311148256	0.209926353
P36915	1xPhospho [T48(0); T50(0); S51(100); S55(0); T57(0)]	1.528	0.611644543	0.738084678
P36915	3xPhospho [T48(100); T50(100); S51(100); S55(0); T57(0)]	0.703	-0.508403406	0.75891781
P36915	1xPhospho [T48(0); T50(1.6); S51(98.4); S55(0); T57(0)]	1.364	0.447843644	0.855534442
P36915	1xPhospho [S68(100)]	1.294	0.371837617	0.863653123
P36915	2xPhospho [T48(100); T50(0.1); S51(100); S55(0); T57(0)]	0.82	-0.286304185	0.918281661
P36915	2xPhospho [T48(50); T50(50); S51(100); S55(0); T57(0)]	1.048	0.067638717	0.985870803
P37802	1xPhospho [S163(100)]	0.509	-0.974262439	0.469512534
P38159	1xPhospho [Y134(0); S135(0); S141(100); S142(0); S143(0)]	1.993	0.99494171	0.264761573
P38159	1xPhospho [T85(0); S88(100); S91(0)]	0.583	-0.778432211	0.806224461
P38159	2xPhospho [Y310(1); Y313(0.1); S314(6.4); S315(6.4); S316(86.1); Y320(0); S323(7.4); S326(92.6); Y327(0); S328(0); S329(0); S330(0)]	1.294	0.371837617	0.876025607
P38159	1xPhospho [Y134(0); S135(0); S141(99.9); S142(0.1); S143(0)]	1.243	0.313826296	0.879063032
P38159	1xPhospho [Y206(0); S208(100)]	1.13	0.176322773	0.965013037
P38159	3xPhospho [Y320(0); S323(0.2); S326(99.8); Y327(0); S328(33.3); S329(33.3); S330(33.3); S332(100); Y335(0); S336(0); S337(0)]	0.87	-0.200912694	0.965139891
P38159	2xPhospho [Y320(0); S323(0.2); S326(99.8); Y327(0); S328(3.8); S329(92.5); S330(3.8); S332(0); Y335(0); S336(0); S337(0)]	1.147	0.197865391	0.965139891
P38159	2xPhospho [S326(0); Y327(0); S328(98); S329(2); S330(0); S332(100); Y335(0); S336(0); S337(0)]	1.046	0.064882852	0.971220368
P38432	1xPhospho [S301(0); T303(98.5); S305(1.5)]	100	6.64385619	8.35E-17
P38432	1xPhospho [T122(100)]	2.137	1.095586908	0.370694157
P39023	1xPhospho [S13(100)]	0.805	-0.312939312	0.956843841
P39880	2xPhospho [S1215(0.2); S1216(99.8); S1218(0); S1220(0); S1227(0); T1230(0); Y1232(0); S1233(0); S1237(100)]	0.01	-6.64385619	8.35E-17
P40818	1xPhospho [S716(0); Y717(0); S718(100); S719(0); T723(0)]	0.776	-0.365871442	0.96293109
P41208	1xPhospho [S20(100); T26(0)]	2.634	1.397255346	0.411842732
P41208	1xPhospho [S20(100); T26(0)]	1.585	0.66448284	0.749797187
P41208	1xPhospho [S20(100); T26(0)]	1.34	0.422233001	0.862820654
P41212	2xPhospho [S182(4.6); S184(95); T187(0.5); T188(0.5); S193(99.5)]	1.418	0.503857533	0.806224461
P41212	2xPhospho [S16(0.1); Y17(0); T18(99.9); S22(100); S26(0); Y27(0); S29(0); S30(0); T31(0)]	1.151	0.202887833	0.971196575
P41236	1xPhospho [T177(0); S182(96.7); T185(3.3); S188(0); S192(0); T193(0); S195(0)]	5.916	2.564622052	0.001101633
P41236	1xPhospho [S121(100); S122(0); S127(0); S130(0)]	1.513	0.597411988	0.810533611
P41236	1xPhospho [S72(0); T73(0); Y75(0); S77(0); S87(99.8); T89(0.2); T92(0)]	1.198	0.260627908	0.935688648
P41236	2xPhospho [S121(100); S122(100); S127(0); S130(0)]	1.072	0.100304906	0.972943798
P41567	1xMet-loss+Acetyl [N-Term]; 1xPhospho [S2(100); S9(0); S17(0); T27(0); Y30(0)]	100	6.64385619	8.35E-17
P41567	1xMet-loss+Acetyl [N-Term]; 1xPhospho [S2(100); S9(0); S17(0)]	1.097	0.133563526	0.971220368
P42167	1xPhospho [S222(2.7); Y223(0); S224(97.3); T229(0); T231(0); T234(0); S235(0); S237(0); S238(0)]	100	6.64385619	8.35E-17
P42167	1xPhospho [T57(0); S59(100)]	3.083	1.624334889	0.130907828
P42167	3xPhospho [T154(0.3); S156(99.4); S158(0.7); S159(99.7); T160(100); T164(0); S166(0); S167(0); S168(0); T172(0)]	2.756	1.462575888	0.143379972
P42167	1xPhospho [S177(0); S180(0); Y183(0); S184(100); S190(0)]	1.743	0.801572569	0.469512534
P42167	3xPhospho [S66(100); S67(100); T74(0); S79(100)]	0.597	-0.744197163	0.670368081
P42167	1xPhospho [T57(7.3); S59(92.7); S66(0); S67(0); T74(0); S79(0)]	1.735	0.794935663	0.682660189
P42167	1xPhospho [S306(100); T312(0); S315(0)]	1.539	0.621993232	0.755716034
P42167	3xPhospho [T154(99.1); S156(92.1); S158(36.3); S159(36.3); T160(36.3); T164(0); S166(0); S167(0); S168(0); T172(0)]	0.639	-0.646112164	0.93019184
P42167	2xPhospho [S66(100); S67(100); T74(0); S79(0)]	0.85	-0.234465254	0.939053555
P42167	2xPhospho [S158(0); S159(0); T160(100); T164(0); S166(100); S167(0); S168(0); T172(0)]	1.111	0.151858817	0.965013037
P42167	1xPhospho [S66(0); S67(100); T74(0); S79(0)]	1.068	0.094911647	0.980895643
P42167	3xPhospho [T57(0); S59(100); S66(100); S67(100); T74(0); S79(0)]	1.054	0.075874867	0.989102507
P42167	2xPhospho [S177(0); S180(100); Y183(0); S184(100); S190(0)]	1.031	0.044044333	0.99184412
P42345	3xPhospho [T2471(48.8); T2473(48.8); T2474(2.4); S2478(100); S2481(100)]	1.575	0.655351829	0.679191074
P42345	1xPhospho [T1162(100); S1166(0)]	1.51	0.59454855	0.697129868
P42345	2xPhospho [T2471(0); T2473(0); T2474(0); S2478(100); S2481(100)]	1.435	0.521050737	0.763890654
P42345	2xPhospho [T2446(0); S2448(100); Y2449(0); S2450(0); S2454(100)]	1.358	0.44148348	0.870654365
P42345	2xPhospho [T2444(98.6); T2446(98.6); S2448(1.4); Y2449(0); S2450(1.4); S2454(0)]	0.59	-0.76121314	0.904774194
P42694	1xPhospho [S1614(100); S1620(0); S1623(0); S1624(0); T1625(0); S1628(0); S1629(0); S1632(0); S1638(0)]	1.325	0.40599236	0.862999305
P42694	2xPhospho [S1614(100); S1620(100); S1623(0); S1624(0); T1625(0); S1628(0); S1629(0); S1632(0); S1638(0)]	0.901	-0.150400989	0.965139891
P42694	2xPhospho [T1736(0); S1738(97.5); S1739(2.5); S1740(2.5); S1741(97.6); S1744(0); Y1748(0)]	0.776	-0.365871442	0.973184413
P42695	1xPhospho [S1372(0); T1379(0); S1382(0); S1384(100)]	0.619	-0.691988685	0.930376591
P42858	2xPhospho [S417(50); S419(50); S429(0); S430(0); S432(100); S436(0)]	0.69	-0.535331733	0.855557911
P43487	2xPhospho [T7(0); T13(97.3); S14(51.3); T15(51.3); T18(0); S21(0); S32(0)]	1.588	0.667210912	0.624540466
P45974	1xPhospho [S779(0); S783(100); S785(0); S787(0)]	1.237	0.3068455	0.907438338
P46013	1xPhospho [S1496(100); T1503(0); T1505(0); S1506(0); S1507(0); S1511(0)]; P46013-2 1xPhospho [S1136(100); T1143(0); T1145(0); S1146(0); S1147(0); S1151(0)]	100	6.64385619	8.35E-17

P46013	2xPhospho [S2105(100); S2110(0); T2113(33); T2115(1.1); S2116(33); T2117(33)]; P46013-2 2xPhospho [S1745(100); S1750(0); T1753(33); T1755(1.1); S1756(33); T1757(33)]	100	6.64385619	8.35E-17
P46013	2xPhospho [S1131(100); S1136(0); T1139(100); T1141(0); S1142(0); T1143(0)]; P46013-2 2xPhospho [S771(100); S776(0); T779(100); T781(0); S782(0); T783(0)]	100	6.64385619	8.35E-17
P46013	2xPhospho [S1861(100); S1864(0); T1869(2.8); T1871(97.1); T1873(0.1)]; P46013-2 2xPhospho [S1501(100); S1504(0); T1509(2.8); T1511(97.1); T1513(0.1)]	100	6.64385619	8.35E-17
P46013	1xPhospho [S1131(100); S1136(0); T1139(0); T1141(0); S1142(0); T1143(0)]; P46013-2 1xPhospho [S771(100); S776(0); T779(0); T781(0); S782(0); T783(0)]	0.102	-3.293358943	0.00171606
P46013	1xPhospho [T1963(0); T1967(0); S1970(0); T1972(0); T1977(0); S1980(0.9); S1983(99.1)]; P46013-2 1xPhospho [T1603(0); T1607(0); S1610(0); T1612(0); T1617(0); S1620(0.9); S1623(99.1)]	0.275	-1.862496476	0.024674332
P46013	1xPhospho [T1977(0); S1980(0); S1983(100)]; P46013-2 1xPhospho [T1617(0); S1620(0); S1623(100)]	3.452	1.787432465	0.071841451
P46013	2 1xPhospho [S127(2.2); S128(95.7); S130(2.2); S131(0); S136(0)]	3.029	1.598841578	0.090744318
P46013	2xPhospho [S2223(100); T2231(100); T2233(0); S2239(0)]; P46013-2 2xPhospho [S1863(100); T1871(100); T1873(0); S1879(0)]	2.759	1.464145457	0.095779335
P46013	1xPhospho [S1815(100)]; P46013-2 1xPhospho [S1455(100)]	2.601	1.379066399	0.306672643
P46013	2xPhospho [T1977(0); S1980(3.4); S1983(96.6); T1991(99.9); T1993(0.1); S1994(0); S1995(0)]; P46013-2 2xPhospho [T1617(0); S1620(3.4); S1623(96.6); T1631(99.9); T1633(0.1); S1634(0); S1635(0)]	2.099	1.069702167	0.307954477
P46013	2xPhospho [S125(97.8); S127(2.2); S128(100); S130(0.1); S131(0)]	2.153	1.10634832	0.325537511
P46013	2xPhospho [S2708(100); T2716(0); T2717(0.1); S2719(97.6); T2720(2.4)]; P46013-2 2xPhospho [S2348(100); T2356(0); T2357(0.1); S2359(97.6); T2360(2.4)]	2.064	1.045442971	0.420545406
P46013	2xPhospho [S538(100); T543(100)]; P46013-2 2xPhospho [S178(100); T183(100)]	1.952	0.964953053	0.431663411
P46013	2xPhospho [S538(100); T543(100)]; P46013-2 2xPhospho [S178(100); T183(100)]	1.894	0.921436331	0.5066667
P46013	1xPhospho [S296(0); S299(0); S308(100)]	1.679	0.74760223	0.519030203
P46013	2xPhospho [S2223(100); T2231(100); T2233(0); S2239(0)]; P46013-2 2xPhospho [S1863(100); T1871(100); T1873(0); S1879(0)]	1.845	0.883620816	0.550634186
P46013	2xPhospho [S296(100); S299(0); S308(100)]	1.778	0.830255324	0.679191074
P46013	1xPhospho [T1323(0); T1327(0); S1329(100)]; 1xPhospho [T1565(0); T1569(0); S1571(100)]; P46013-2 1xPhospho [T963(0); T967(0); S969(100)]; 1xPhospho [T1205(0); T1209(0); S1211(100)]	1.557	0.638768944	0.716990671
P46013	2 2xPhospho [S125(50.9); S127(50.9); S128(98.1); S130(0); S131(0); S136(0)]	1.436	0.522055749	0.774421636
P46013	1xPhospho [S1861(99.9); S1864(0.1); T1869(0); T1871(0); T1873(0)]; P46013-2 1xPhospho [S1501(99.9); S1504(0.1); T1509(0); T1511(0); T1513(0)]	1.403	0.488515009	0.790295254
P46013	1xPhospho [T2623(0); T2625(0); S2626(0); S2629(0); T2630(0); T2632(0); S2638(100)]; P46013-2 1xPhospho [T2263(0); T2265(0); S2266(0); S2269(0); T2270(0); T2272(0); S2278(100)]	1.371	0.455228571	0.849882438
P46013	1xPhospho [S2223(100); T2231(0); T2233(0); S2239(0)]; P46013-2 1xPhospho [S1863(100); T1871(0); T1873(0); S1879(0)]	1.329	0.410341105	0.855031791
P46013	1xPhospho [S152(100)]	1.362	0.445726703	0.878612438
P46013	2xPhospho [S575(0); S579(100); S584(100)]; P46013-2 2xPhospho [S215(0); S219(100); S224(100)]	1.258	0.331131922	0.879063032
P46013	2xPhospho [T2623(0); T2625(0); S2626(1.9); S2629(49.1); T2630(49.1); T2632(0); S2638(100)]; P46013-2 2xPhospho [T2263(0); T2265(0); S2266(1.9); S2269(49.1); T2270(49.1); T2272(0); S2278(100)]	1.281	0.357270476	0.879063032
P46013	1xPhospho [S3041(100); S3042(0)]; P46013-2 1xPhospho [S2681(100); S2682(0)]	1.364	0.447843644	0.879063032
P46013	2 2xPhospho [S127(0.3); S128(0.3); S130(99.7); S131(99.7); S136(0)]	1.164	0.219091058	0.933760225
P46013	1xPhospho [S2223(100); T2231(0); T2233(0); S2239(0)]; P46013-2 1xPhospho [S1863(100); T1871(0); T1873(0); S1879(0)]	1.109	0.149259365	0.971985173
P46013	1xPhospho [Y1552(100); T1557(0)]; P46013-2 1xPhospho [Y1192(100); T1197(0)]	0.878	-0.187707155	0.992376868
P46013	1xPhospho [T2460(0); S2463(0); S2466(100); S2471(0)]; P46013-2 1xPhospho [T2100(0); S2103(0); S2106(100); S2111(0)]	0.959	-0.06039728	0.992680338
P46013	1xPhospho [S575(0); S579(0); S584(100)]; P46013-2 1xPhospho [S215(0); S219(0); S224(100)]	0.917	-0.125006361	1
P46019	2xPhospho [S1039(92.8); S1040(3.6); T1041(3.6); S1043(96.3); S1044(3.7); T1046(0); T1048(0); S1049(0); S1050(0); S1051(0); S1053(0)]	1.16	0.214124805	0.931841768
P46060	1xPhospho [T419(0); S427(0); S428(0); S435(3.7); T436(0.2); S442(96.2)]	100	6.64385619	8.35E-17
P46060	2xPhospho [T419(0); S427(3.9); S428(96.1); S435(0); T436(0); S442(100)]	11.916	3.574828124	5.53E-06
P46087	1xPhospho [S96(100)]	2.105	1.073820233	0.284608018
P46087	1xPhospho [S96(100)]	2.518	1.332278283	0.299783371
P46087	1xPhospho [S659(0); S662(0); T663(0); S666(98.7); T668(1.3)]	1.201	0.264236151	0.920957334
P46087	2xPhospho [T776(100); T784(3.3); S786(96.7)]	1.07	0.097610797	0.962617576
P46087	1xPhospho [T784(0); S786(100)]	0.943	-0.084670324	0.986075329
P46087	3xPhospho [S181(100); T185(100); T195(95.9); S198(4.1)]	0.958	-0.061902439	0.990182098
P46087	1xPhospho [T723(0); T725(0); T727(0); S732(100); S734(0)]	0.953	-0.069451881	0.998586735
P46087	2xPhospho [S181(100); T185(100); T195(0); S198(0)]	1.009	0.012926174	1
P46100	3xPhospho [Y89(0); S92(100); T102(0); S108(100); S112(100); T117(0); S120(0)]	0.01	-6.64385619	8.35E-17
P46100	3xPhospho [T668(0); T670(0); T674(100); S675(100); S677(100); T684(0)]	0.01	-6.64385619	8.35E-17
P46100	2xPhospho [S722(0); T724(0); S729(100); S731(100)]	0.01	-6.64385619	8.35E-17
P46100	3xPhospho [Y1009(0); S1011(51.6); S1012(51.6); S1013(97); T1016(99.8)]	0.01	-6.64385619	8.35E-17
P46100	2xPhospho [S722(0); T724(0); S729(100); S731(100)]	0.338	-1.564904848	0.244965218
P46100	1xPhospho [S703(0); S704(0); S706(100)]	0.383	-1.384583703	0.385507135
P46100	1xPhospho [T591(0); S594(0); S596(0); S598(100)]	0.533	-0.907792562	0.650065098
P46100	1xPhospho [Y89(0); S92(100); T102(0); S108(0); S112(0); T117(0); S120(0)]	1.435	0.521050737	0.792141506
P46100	2xPhospho [T1230(0); S1236(0); S1237(0); T1239(0); S1244(100); S1245(100); S1251(0)]	0.708	-0.498178735	0.836554173
P46100	1xPhospho [T668(0); T670(0); T674(0); S675(0); S677(100); T684(0)]	1.243	0.313826296	0.918281661
P46100	1xPhospho [S25(0); S26(0); S29(0); T32(0); S33(1.5); S34(98.4)]	1.124	0.168642036	0.930376591
P46100	3xPhospho [Y1009(0); S1011(100); S1012(94.7); S1013(94.7); T1016(10.7)]	0.712	-0.490050854	0.938483927

P46100	1xPhospho [S1061(100); Y1063(0)]	1.098	0.134878054	0.950730609
P46100	2xPhospho [T591(0); S594(100); S596(0); S598(100)]	1.086	0.119024103	0.972363997
P46100	1xPhospho [Y309(0); T312(2.3); S313(95.4); S316(2.3)]	0.854	-0.227692025	0.973599548
P46100	2xPhospho [S25(100); S26(0.1); S29(0); T32(0.1); S33(0.1); S34(99.9)]	0.825	-0.277533976	0.97998947
P46100	2xPhospho [T591(0); S594(100); S596(0); S598(100); Y615(0)]	0.945	-0.081613766	0.980895643
P46100	1xPhospho [S471(100); T480(0)]	0.891	-0.166502663	0.992863986
P46100	2xPhospho [T591(0); S594(100); S596(0.2); S598(99.8)]	0.969	-0.045431429	1
P46783	1xPhospho [S146(100); T148(0)]	0.89	-0.168122759	0.965013037
P46783	1xPhospho [S146(100); T148(0)]	0.94	-0.089267338	0.985318278
P46821	2xPhospho [S1501(100); T1503(0); S1508(0); S1512(100); T1517(0)]	0.01	-6.64385619	8.35E-17
P46821	2xPhospho [S992(100); S995(100)]	0.01	-6.64385619	8.35E-17
P46821	2xPhospho [S2209(100); S2211(100)]	0.01	-6.64385619	8.35E-17
P46821	2xPhospho [S828(0); S831(100); S832(99.9); T837(0.1); T851(0)]	0.01	-6.64385619	8.35E-17
P46821	2xPhospho [S541(100); S544(100); S554(0)]	0.01	-6.64385619	8.35E-17
P46821	2xPhospho [S1792(0.1); S1793(99.9); Y1796(0); S1797(2.8); T1799(94.5); S1801(2.8); S1803(0); T1804(0); S1805(0)]	0.01	-6.64385619	8.35E-17
P46821	2xPhospho [S1396(100); S1400(100); S1406(0); S1408(0); Y1410(0); S1412(0); S1415(0)]	0.01	-6.64385619	8.35E-17
P46821	1xPhospho [S1501(100); T1503(0); S1508(0); S1512(0)]	0.01	-6.64385619	8.35E-17
P46821	2xPhospho [S828(0); S831(100); S832(100); T837(0)]	0.01	-6.64385619	8.35E-17
P46821	1xPhospho [S2265(0); S2271(100)]	0.01	-6.64385619	8.35E-17
P46821	2xPhospho [S992(100); S995(100)]	0.199	-2.329159664	0.006480788
P46821	3xPhospho [S1772(0); S1779(100); S1782(100); S1785(0); T1788(100)]	0.246	-2.023269779	0.01330465
P46821	3xPhospho [S1252(0); S1254(0); S1256(100); S1258(97); S1260(3); S1262(0.1); S1265(99.9)]	0.256	-1.965784285	0.019499401
P46821	2xPhospho [S2209(100); S2211(100)]	0.299	-1.74178261	0.034571361
P46821	2xPhospho [S1772(0); S1779(100); S1782(99.9); S1785(0.1); T1788(0)]	0.243	-2.040971781	0.043690337
P46821	2xPhospho [S828(0); S831(100); S832(100); T837(0)]	0.315	-1.666576266	0.146188764
P46821	2xPhospho [S1256(0); S1258(0); S1260(100); S1262(0); S1265(100)]	0.266	-1.910501849	0.244965218
P46821	2xPhospho [T1317(0); S1322(98.9); S1324(0.6); S1326(0.6); T1328(0.5); S1330(92.9); T1334(0); Y1336(0); Y1337(0); S1339(6.5); T1341(0)]	1.527	0.610700062	0.680084646
P46821	2xPhospho [S1501(93.2); T1503(6.8); S1508(6.8); S1512(93.1); T1517(0)]	0.541	-0.886299501	0.74242706
P46821	2xPhospho [S1289(0); S1298(100); T1302(0); S1312(100)]	0.794	-0.332789088	0.973763863
P47914	1xPhospho [S142(100)]	1.528	0.611644543	0.751397104
P47914	1xPhospho [Y29(0); S31(100)]	1.04	0.056583528	0.973763863
P47974	2xPhospho [S57(99.9); S59(0.1); S70(49.2); S73(1.5); S75(49.2)]	0.556	-0.846843212	0.751447693
P48436	2xPhospho [S211(0); S214(0); S215(0); S216(0); S219(0); S223(0); S228(0); S231(0); T236(6.4); T239(94); T240(99.6)]	4.568	2.191562651	0.001016344
P48444	1xPhospho [S188(0); S189(0); S192(0); S195(99.9); T196(0.1); T201(0); T203(0); T207(0)]	0.344	-1.53951953	0.252659863
P48444	2xPhospho [S188(3.9); S189(96); S192(0.2); S195(3.9); T196(96.1); T201(0); T203(0); T207(0)]	0.765	-0.386468347	0.934211842
P48634	2xPhospho [T1083(0.1); S1085(99.9); T1087(0.1); S1089(100); S1092(0); Y1094(0)]	0.01	-6.64385619	8.35E-17
P48634	2xPhospho [S1106(0); T1108(0); S1110(0.3); T1112(0.3); S1115(99.4); S1120(99.7); T1126(0.3)]	0.01	-6.64385619	8.35E-17
P48634	2xPhospho [T1083(2.1); S1085(97.8); T1087(0.1); S1089(100); S1092(0); Y1094(0)]	1.515	0.599317794	0.749797187
P48634	2xPhospho [S1106(50.8); T1108(50.8); S1110(98.1); T1112(0.2); S1115(0); S1120(0)]	1.452	0.538041453	0.769621572
P48634	1xPhospho [S1219(100)]	0.63	-0.666576266	0.780967157
P48634	3xPhospho [S1106(100); T1108(94.7); S1110(94.8); T1112(10.2); S1115(0.3); S1120(0); T1126(0)]	1	0	0.970242377
P48634	3xPhospho [T1083(0); S1085(100); T1087(0); S1089(100); S1092(100); Y1094(0)]	0.883	-0.179514657	0.971838536
P48634	2xPhospho [S342(100); S350(100)]	0.918	-0.123433941	0.973263372
P48634	1xPhospho [S30(100)]	0.994	-0.008682243	0.975343735
P48634	3xPhospho [T1083(0); S1085(100); T1087(0); S1089(100); S1092(100); Y1094(0)]	0.968	-0.046921047	1
P48681	2xPhospho [S1409(100); S1418(100)]	1.184	0.243669081	0.95142727
P48681	1xPhospho [S768(100); T776(0)]	0.86	-0.217591435	0.967238018
P48730	2 2xPhospho [T337(0); T344(0); T347(0); T349(96.2); S350(3.8); T352(3.7); T355(92.6); S356(3.7); S361(0)]; P48730 2xPhospho [T337(0); T344(0); T347(0); T349(96.2); S350(3.8); T352(3.7); T355(92.6); S356(3.7); S361(0)]	0.384	-1.380821784	0.192853733
P48730	2xPhospho [S406(0); S407(100); S411(100)]	1.966	0.975263322	0.345445046
P48730	2 2xPhospho [S396(100); T397(0); S398(3); S401(97)]	1.686	0.753604536	0.527474942
P48730	2 2xPhospho [S382(99.1); S383(50.5); S384(50.5); T387(0)]; P48730 2xPhospho [S382(99.1); S383(50.5); S384(50.5); T387(0)]	0.67	-0.577766999	0.775393256
P48730	2 3xPhospho [S396(100); T397(5.7); S398(94.6); S401(99.7)]	0.501	-0.997117491	0.776177942
P48730	2 3xPhospho [T337(0); T344(0); T347(0); T349(49.9); S350(49.9); T352(0.1); T355(95.5); S356(4.5); S361(100)]; P48730 3xPhospho [T337(0); T344(0); T347(0); T349(49.9); S350(49.9); T352(0.1); T355(95.5); S356(4.5); S361(100)]	0.726	-0.461958547	0.79792008
P48730	2 3xPhospho [T337(0); T344(0); T347(48.6); T349(48.6); S350(2.7); T352(0.2); T355(5.2); S356(94.8); S361(100)]; P48730 3xPhospho [T337(0); T344(0); T347(48.6); T349(48.6); S350(2.7); T352(0.2); T355(5.2); S356(94.8); S361(100)]	0.703	-0.508403406	0.863867749
P48730	2 3xPhospho [S396(100); T397(4.3); S398(95.7); S401(100)]	0.672	-0.573466862	0.887284207
P48730	3xPhospho [S406(100); S407(100); S411(100)]	1.245	0.316145742	0.905539755
P48730	2 3xPhospho [T337(100); T344(0); T347(0); T349(3.9); S350(50); T352(50); T355(96.1); S356(0); S361(0)]; P48730 3xPhospho [T337(100); T344(0); T347(0); T349(3.9); S350(50); T352(50); T355(96.1); S356(0); S361(0)]	0.718	-0.477944251	0.942907978
P48730	2 1xPhospho [S396(100); T397(0); S398(0); S401(0)]	1.028	0.039840265	0.989838391
P48730	2 2xPhospho [S396(0); T397(0.1); S398(99.9); S401(100)]	0.992	-0.011587974	0.998449689
P48730	2 1xPhospho [S382(0); S383(1.4); S384(98.7); T387(0)]; P48730 1xPhospho [S382(0); S383(1.4); S384(98.7); T387(0)]	0.948	-0.077041036	1

P48730	2 2xPhospho [T337(0); T344(0); T347(0); T349(4.3); S350(95.7); T352(0); T355(50); S356(50); S361(0)]; P48730 2xPhospho [T337(0); T344(0); T347(0); T349(4.3); S350(95.7); T352(0); T355(50); S356(50); S361(0)]	1.011	0.015782997	1
P48740	4 3xPhospho [S2(99.7); T4(98.1); S7(3.1); S10(49.5); T16(49.5); Y22(0)]	0.688	-0.53951953	0.879063032
P49006	2xPhospho [S116(99.9); S117(50); S119(50); S120(0.1); T122(0); S135(0); T139(0)]	0.01	-6.64385619	8.35E-17
P49006	2xPhospho [S101(100); S104(100)]	0.01	-6.64385619	8.35E-17
P49006	1xPhospho [S116(97.7); S117(2.3); S119(0); S120(0); T122(0); S135(0); T139(0)]	100	6.64385619	8.35E-17
P49006	2xPhospho [S101(100); S104(100)]	0.269	-1.894321922	0.010712325
P49006	2xPhospho [S101(100); S104(100)]	0.264	-1.921390165	0.071929198
P49006	2xPhospho [S101(100); S104(100)]	0.361	-1.469929258	0.189256987
P49006	1xPhospho [S101(0); S104(100)]	0.646	-0.63039393	0.703341962
P49006	2xPhospho [S116(96.5); S117(3.5); S119(99.9); S120(0.1); T122(0); S135(0); T139(0)]	0.563	-0.828793173	0.766139179
P49006	1xPhospho [S116(96.9); S117(3.1); S119(0); S120(0); T122(0); S135(0); T139(0)]	1.206	0.270229907	0.933452347
P49006	1xPhospho [S162(5); S165(95); T174(0); S177(0); T178(0); S180(0); S184(0); T187(0); S190(0)]	1.168	0.224040274	0.939053555
P49006	1xPhospho [S48(0); T54(0); T61(0); S71(100)]	0.909	-0.1376478	0.989340879
P49006	1xPhospho [S101(0); S104(100)]	0.912	-0.13289427	0.989838391
P49069	1xPhospho [S55(100); S61(0); T63(0)]	0.799	-0.323732592	0.980895643
P49247	1xPhospho [S104(0); S106(98.2); T107(1.8)]	1.285	0.361768359	0.862999305
P49327	1xPhospho [S2198(0); T2204(100)]	1.477	0.562669826	0.769621572
P49327	1xPhospho [S2236(100); S2239(0); S2240(0); S2253(0); T2254(0); T2255(0); S2259(0); S2262(0)]	1.262	0.33571191	0.906218259
P49327	1xPhospho [S974(98); T976(2); T980(0); Y991(0)]	1.059	0.082702589	0.972943798
P49643	1xPhospho [S484(0); S485(0); S489(0); S492(0); S493(0); Y504(0); S506(96); S509(4)]	0.236	-2.083141235	0.113778047
P49736	1xPhospho [T25(0); S26(0); S27(100)]	4.244	2.085424656	0.018780405
P49736	1xPhospho [Y90(0); T106(0); S108(100)]	0.277	-1.852042119	0.09762892
P49736	1xPhospho [Y137(0); S139(100)]	1.094	0.129612738	0.973763863
P49756	1xPhospho [S583(100)]	100	6.64385619	8.35E-17
P49756	1xPhospho [S583(100)]	2.389	1.256406854	0.345649696
P49756	1xPhospho [Y218(0); S219(0); S220(0); S226(0); S229(100); S231(0)]	0.801	-0.320125852	0.934930293
P49756	1xPhospho [S693(0); S703(100)]	0.872	-0.19759996	0.99184412
P49761	1 1xPhospho [S135(100)]	1.494	0.579180148	0.747299091
P49761	1 1xPhospho [S51(100); Y57(0)]	1.334	0.415758667	0.862322824
P49761	1 2xPhospho [S76(100); S78(100); Y83(0); Y84(0); S87(0)]	1.243	0.313826296	0.879063032
P49761	1 1xPhospho [Y7(0); S9(100); Y15(0); S17(0); Y18(0)]	1.173	0.230203013	0.904227116
P49761	1 2xPhospho [S76(100); S78(100); Y83(0); Y84(0); S87(0)]	1	0	0.973763863
P49761	1 2xPhospho [S49(100); S51(100); Y57(0)]	0.948	-0.077041036	0.980895643
P49761	1 2xPhospho [S132(50); S133(50); S135(100)]	0.962	-0.055891201	0.998448514
P49768	2xPhospho [Y315(0); S319(0.2); T320(0.2); S324(99.7); T327(100); S337(0)]	100	6.64385619	8.35E-17
P49768	2xPhospho [S365(0); S366(100); S367(100)]	0.678	-0.560642822	0.923236933
P49768	1xPhospho [S43(100); S51(0); S59(0)]	1.121	0.164786278	0.943796521
P49792	1xPhospho [S948(0); T951(0); S955(100)]	0.01	-6.64385619	8.35E-17
P49792	1xPhospho [S2237(0); S2239(0); S2240(0); S2241(0); S2242(0); S2246(0.1); S2250(2.6); S2251(97.4)]	0.01	-6.64385619	8.35E-17
P49792	1xPhospho [T1156(3.9); S1160(96.1)]	0.372	-1.426625474	0.088153676
P49792	3xPhospho [S2237(0); S2239(0); S2240(0); S2241(0.3); S2242(99.7); S2246(100); S2250(4.8); S2251(95.2)]	0.423	-1.241270432	0.178323432
P49792	2xPhospho [T2734(0); S2741(100); T2743(100); S2755(0)]	0.564	-0.826232932	0.600153916
P49792	3xPhospho [S2237(0.3); S2239(5.1); S2240(0.3); S2241(0.3); S2242(94.1); S2246(100); S2250(0.3); S2251(99.7)]	0.502	-0.994240731	0.640854399
P49792	2xPhospho [S2237(0); S2239(0); S2240(0); S2241(0); S2242(0); S2246(100); S2250(50); S2251(50)]	0.53	-0.915935735	0.701261726
P49792	1xPhospho [S2900(100); S2917(0)]	1.625	0.700439718	0.716990671
P49792	2xPhospho [S792(0); Y793(0); Y795(0); S796(100); T799(100)]	1.4	0.485426827	0.813248891
P49792	1xPhospho [Y2666(0); S2668(100); T2679(0)]	0.602	-0.732164608	0.851160627
P49792	2xPhospho [S2447(0); T2450(100); S2454(100)]	1.299	0.377401431	0.851160627
P49792	2xPhospho [T1992(0); S1993(0); Y2003(0); T2005(100); S2008(100)]	0.729	-0.45600928	0.863668848
P49792	1xPhospho [S1447(0); S1449(0); S1450(0); S1456(100)]	0.64	-0.64385619	0.877952584
P49792	1xPhospho [S1107(0); S1110(100); T1112(0); S1117(0); S1118(0)]	1.208	0.272620455	0.926103516
P49792	1xPhospho [T1156(0); S1160(100)]	0.832	-0.265344567	0.965139891
P49792	1xPhospho [T2734(0); S2741(99.9); T2743(0.1); S2755(0)]	0.838	-0.254977851	0.969680228
P49792	1xPhospho [S2447(0); T2450(0); S2454(100)]	0.798	-0.325539348	0.972326459
P49792	2xPhospho [T1393(0); T1396(100); T1399(2.3); S1400(97.7)]	1.132	0.178873958	0.973263372
P49792	2xPhospho [S2237(0); S2239(0); S2240(0); S2241(0); S2242(0); S2246(100); S2250(2.8); S2251(97.2)]	1.076	0.105678078	0.97998947
P49792	2xPhospho [S2802(0.7); S2804(90.6); S2805(7.4); S2807(0.1); S2809(0.1); S2810(0.1); T2812(1.3); S2820(8.1); T2821(91.7)]	0.827	-0.274040765	0.99277609
P49792	2xPhospho [S778(0); T779(0.2); S781(99.7); T783(96.4); Y785(0); S786(3.7); S788(0); S790(0)]	0.9	-0.152003093	0.998448514
P49840	1xPhospho [S278(100); Y279(0); S282(0)]; P49841 1xPhospho [S215(100); Y216(0); S219(0)]	1.63	0.704871964	0.665926698
P49840	2xPhospho [S52(100); S63(7.1); S64(7.1); S65(71.6); S72(7.1); S77(7.1); T84(0); S85(0)]	1.498	0.583037624	0.7535184
P49841	2xPhospho [T7(0); T8(0); S9(99.9); S13(0); S21(5.8); S25(94.2)]	1.619	0.695102986	0.751447693
P49848	2xPhospho [T622(49); S623(49); S626(2.1); S633(0.2); S634(99.8); S636(0); S639(0); S641(0)]	1.022	0.031395196	0.989838391
P49916	2xPhospho [T203(0); T204(0); T205(0); T209(0); S210(100); S216(100); T219(0); S220(0); T221(0)]	0.711	-0.492078535	0.883697728
P49916	1xPhospho [S227(0); S230(0); S236(0); S241(3.2); S242(93.6); T244(3.2)]	1.115	0.15704371	0.930376591
P49959	3xPhospho [S688(100); S689(100); T700(1.9); S701(49.1); S702(49.1)]	0.406	-1.300448367	0.38667379
P49959	3xPhospho [S688(100); S689(100); T700(48.8); S701(48.8); S702(2.4)]	0.384	-1.380821784	0.579084172
P49959	1xPhospho [S619(100)]	1.015	0.021479727	0.977837438

P49959	2xPhospho [S688(100); S689(100); T700(0); S701(0); S702(0)]	1.017	0.024319679	0.982743754
P50402	3xPhospho [S120(0); T122(0); S123(0); S141(100); S142(100); S143(100)]	0.491	-1.02620507	0.2246667
P50402	2xPhospho [S49(96.4); S52(3.6); S53(0); S54(0); S57(0); S58(0); Y59(0); S60(0); S62(0); S66(96.4); T67(3.6); Y74(0)]	2.538	1.343692069	0.227855233
P50402	2xPhospho [S120(0); T122(0); S123(0); S141(51.7); S142(51.7); S143(96.6)]	1.23	0.298658316	0.920957334
P50402	1xPhospho [S159(0); Y161(0); S163(0); T165(0); Y167(0); S171(1.7); S173(98.4)]	1.054	0.075874867	0.971325257
P50402	2xPhospho [S159(0); Y161(0); S163(100); T165(0); Y167(0); S171(3.5); S173(96.5)]	0.999	-0.001443417	0.981858798
P50402	2xPhospho [S49(3.2); S52(3.2); S53(3.2); S54(90.3); S57(0); S58(0); Y59(0); S60(100); S62(0); S66(0); T67(0)]	0.926	-0.110915901	0.98356941
P50443	2xPhospho [S12(100); S16(100); S22(0); Y23(0); S25(0)]	1.009	0.012926174	0.987464758
P50502	3xPhospho [S75(100); S76(100); S79(100)]	0.26	-1.943416472	0.001347188
P50502	3xPhospho [S75(100); S76(100); S79(100)]	0.563	-0.828793173	0.657415306
P50502	2xPhospho [S75(0); S76(100); S79(100)]	1.052	0.073134705	0.971973958
P50502	2xPhospho [S75(1.6); S76(98.4); S79(100)]	1.048	0.067638717	0.980895643
P50548	3xPhospho [S431(100); S435(100); T441(0); S444(100)]	0.149	-2.746615764	0.017378326
P50548	3xPhospho [S185(100); S187(100); S190(100); T193(0); S194(0)]	0.17	-2.556393349	0.0399865
P50548	2xPhospho [T3(100); T7(0); Y16(0); S20(33.3); S21(33.3); S24(33.3)]	0.53	-0.915935735	0.625045843
P50548	2xPhospho [T3(100); T7(0); Y16(0); S20(0.1); S21(0.1); S24(99.8)]	0.518	-0.948975997	0.688108927
P50548	2xPhospho [S147(0); T148(0); S150(0.4); S154(99.2); T156(0.4); S161(100); S166(0); S167(0); S168(0); S169(0); S170(0); S171(0); S174(0)]	0.875	-0.192645078	0.980895643
P50548	3xPhospho [S147(5.5); T148(94.5); S150(0); S154(100); T156(0); S161(100); S166(0); S167(0); S168(0); S169(0); S170(0); S171(0); S174(0)]	0.907	-0.140825544	0.994865845
P50914	1xPhospho [S139(100)]	0.933	-0.100051014	0.99184412
P51003	1xMet-loss [N-Term]; 1xPhospho [T6(0); T7(0); S10(100); T13(0)]	0.7	-0.514573173	0.855113813
P51116	2xPhospho [Y409(0); S410(0); T411(100); S414(3); S415(3); S416(3); S417(87.8); S418(3); T422(0)]	100	6.64385619	8.35E-17
P51116	2xPhospho [T598(0); S601(100); S603(100); T610(0); Y614(0); S616(0)]	0.653	-0.614845103	0.660888692
P51116	1xPhospho [T558(0); S566(100); T572(0); S580(0)]	1.04	0.056583528	0.985870803
P51397	1xPhospho [S49(0); S51(100); T56(0); S60(0)]	0.654	-0.612637459	0.820380622
P51531	1xPhospho [S1377(100)]	0.01	-6.64385619	8.35E-17
P51532	2xPhospho [T596(0); T609(5.4); S610(94.6); S613(100)]	0.01	-6.64385619	8.35E-17
P51532	2xPhospho [T596(0); T609(0.2); S610(99.8); S613(100)]	0.2	-2.321928095	0.013304534
P51532	1xPhospho [S1452(100); T1459(0)]	2.317	1.212258044	0.330660896
P51532	3xPhospho [S1570(100); S1575(100); S1586(99.9); S1588(0.1); S1590(0)]	0.58	-0.785875195	0.692423571
P51532	1xPhospho [T596(0); T609(3.6); S610(92.8); S613(3.6)]	0.663	-0.592919225	0.947600551
P51532	1xPhospho [S1452(100); T1459(0)]	1.03	0.042644337	0.985461924
P51610	1xPhospho [T1386(0); S1389(0); S1398(95.8); T1400(4.2); T1405(0); T1413(0)]	0.01	-6.64385619	8.35E-17
P51610	1xPhospho [S666(100); S669(0); S674(0); S678(0)]	0.45	-1.152003093	0.496087995
P51610	1xPhospho [T1485(0); T1486(0); T1488(0); S1490(0); T1491(0); S1497(0); S1507(100)]	0.819	-0.288064643	0.977837438
P51784	2xPhospho [S938(0); S942(0.1); S943(0.1); S948(99.9); S952(50); S953(50); S956(0); S957(0)]	0.387	-1.369594529	0.397877541
P51784	2xPhospho [S938(0); S942(0); S943(0); S948(100); S952(5.6); S953(94.4); S956(0); S957(0)]	0.464	-1.10780329	0.714293599
P51784	1xPhospho [Y642(0); T644(0); S648(100); S671(0); T672(0); S675(0)]	0.815	-0.295128036	0.938730361
P52272	1xPhospho [S618(100)]	2.31	1.207892852	0.114239834
P52272	1xPhospho [S397(100)]	2.528	1.337996464	0.183417508
P52272	1xPhospho [S618(100)]	1.825	0.867896464	0.491829311
P52272	1xPhospho [S633(100); S637(0)]	1.469	0.554834396	0.733427607
P52272	1xPhospho [S22(0); S29(100)]	1.601	0.678973308	0.748349543
P52272	2xPhospho [S633(100); S637(100)]	1.431	0.517023672	0.763548117
P52735	3 2xPhospho [S768(1.9); S769(98.2); S771(100); T775(0)]	1.153	0.205392513	0.960314241
P52756	2xPhospho [Y620(0); S621(100); S624(100)]	0.614	-0.703689439	0.75802349
P52756	3xPhospho [S69(100); S72(100); Y76(0); S78(100); Y82(0); Y87(0)]	0.725	-0.4639471	0.799931389
P52756	1xPhospho [Y620(0); S621(0); S624(100)]	0.729	-0.45600928	0.879063032
P52756	3xPhospho [S69(100); S72(100); Y76(0.1); S78(100); Y82(0); Y87(0)]	0.737	-0.440263476	0.908083211
P52756	2xPhospho [S69(3.4); S72(96.7); Y76(0); S78(100); Y82(0); Y87(0)]	0.815	-0.295128036	0.980895643
P52888	1xPhospho [S16(100); S19(0)]	0.762	-0.392137097	0.963170174
P52943	1xPhospho [S114(0); S115(100); T117(0); T118(0); T120(0); T125(0)]	2.21	1.14404637	0.227819448
P52948	1xPhospho [Y883(0); S888(100); S897(0)]	1.499	0.584000383	0.675336505
P52948	3xPhospho [S608(0); S612(100); S618(100); S623(100); S625(0); Y627(0)]	0.503	-0.991369695	0.769999353
P52948	2xPhospho [S608(100); S612(100)]	1.318	0.39835037	0.855816069
P52948	1xPhospho [S608(0); S612(100)]	1.116	0.158337027	0.965351295
P52948	1xPhospho [S618(0); S623(100); S625(0); Y627(0)]	1	0	0.979498769
P52948	1xPhospho [S1043(100); S1051(0)]	1	0	0.979498769
P53350	1xPhospho [S326(0); S330(0); S331(100); S335(0)]	0.789	-0.341902795	0.97998947
P53365	1xPhospho [S72(2.2); S74(97.8); T75(0.1); T76(0); S78(0)]	2.019	1.013640911	0.483067456
P53367	1xPhospho [S36(100); S39(0); S44(0); T46(0); T49(0); S50(0); T56(0)]	1.633	0.707524791	0.638149561
P53367	2xPhospho [S36(0.4); S39(93.7); S44(99.6); T46(6.3); T49(0); S50(0); T56(0)]	0.626	-0.675765438	0.904030095
P53367	1xPhospho [S9(0); T16(0); S17(0); S24(0); S28(100)]	0.983	-0.024736678	1
P53396	2xPhospho [T453(0); S455(100); S457(0); S459(100)]	0.983	-0.024736678	0.985318278
P53396	1xPhospho [S478(0); S481(100)]	0.978	-0.03209363	1
P53621	1xPhospho [S173(100); S179(0)]	1.565	0.646162657	0.711284181
P53985	1xPhospho [T466(0); S467(100); T478(0)]	1.942	0.957543201	0.35260057
P53985	2xPhospho [S461(0); T466(100); S467(100); T478(0)]	1.845	0.883620816	0.376994864
P53985	1xPhospho [S461(100); T466(0); S467(0); T478(0)]	1.895	0.922197848	0.385507135
P53985	3xPhospho [S461(100); T466(100); S467(100); T478(0)]	1.297	0.37517848	0.879063032
P54105	1xPhospho [S90(0); S102(100); T110(0)]	1.128	0.173767068	0.965139891

P54259	2xPhospho [Y734(0); T736(0); S739(0); S746(100); S748(100)]	1.726	0.787432465	0.618224241
P54259	3xPhospho [Y734(0); T736(0); S739(100); S746(100); S748(100)]	0.602	-0.732164608	0.742588401
P54259	2xPhospho [T651(0); T653(100); Y657(0); S661(100); S664(0)]	1.235	0.304511042	0.900153261
P54259	2xPhospho [T92(0); S101(100); S103(0); S107(100)]	1.175	0.232660757	0.961452497
P54259	2xPhospho [S73(0); S77(100); S79(100); S81(0); T84(0)]	0.925	-0.112474729	0.989898705
P54578	1xPhospho [S143(100); S148(0); Y151(0); T153(0)]	0.949	-0.075520008	1
P55010	2xPhospho [S389(100); S390(100); Y405(0); S406(0)]	0.704	-0.506352666	0.830379242
P55072	1xPhospho [S775(0); S784(0); S787(0); T791(0); S794(100); Y796(0); T797(0); Y805(0)]	0.48	-1.058893689	0.321795737
P55072	1xMet-loss+Acetyl [N-Term]; 1xPhospho [S3(100); S7(0); S13(0); T14(0)]	0.667	-0.584241333	0.872730156
P55081	2xPhospho [S116(100); S118(100)]	0.567	-0.81857936	0.431663411
P55081	2xPhospho [Y391(0); T392(0); T399(33.3); T400(33.3); S401(33.3); S404(100); S410(0); T414(0)]	0.433	-1.20756107	0.491829311
P55081	2xPhospho [S132(100); S133(100)]	1.704	0.768925336	0.712836242
P55081	2xPhospho [Y47(0); S52(100); S53(100)]	0.685	-0.545824107	0.726359449
P55081	2xPhospho [S132(100); S133(100)]	0.704	-0.506352666	0.759239289
P55081	2xPhospho [S116(100); S118(100)]	0.626	-0.675765438	0.794223792
P55081	2xPhospho [S132(100); S133(100)]	0.664	-0.590744853	0.81171187
P55081	1xPhospho [S258(0); T267(100); Y276(0)]	0.682	-0.552156356	0.886312012
P55081	2xPhospho [Y47(0); S52(100); S53(100)]	0.793	-0.334607229	0.942907978
P55081	2xPhospho [S132(100); S133(100)]	0.967	-0.048412205	0.99184412
P55198	2xPhospho [Y369(0); S370(0); T373(0); S378(100); S382(99.8); S384(0.2)]	2.288	1.194087052	0.424606504
P55199	1xPhospho [T431(0); S437(0); S442(3); S444(97.1)]	0.844	-0.244685096	0.973263372
P55199	1xPhospho [S401(0); S407(100)]	0.827	-0.274040765	0.988977631
P55201	1xPhospho [S844(0); T846(0); T858(0); S860(100); S865(0); S866(0); S867(0); T870(0); S871(0)]	100	6.64385619	8.35E-17
P55201	1xPhospho [S917(87.2); T920(0.5); S922(6.2); S926(6.2); S937(0); S938(0)]	0.075	-3.736965594	5.50E-06
P55201	2xPhospho [S844(0); T846(0); T858(100); S860(100); S865(0); S866(0); S867(0); T870(0); S871(0)]	0.366	-1.450084446	0.306672643
P55201	2xPhospho [S460(100); S462(100); S479(0); S480(0)]	0.85	-0.234465254	0.971937853
P55201	2xPhospho [S460(100); S462(100)]	0.977	-0.033569533	1
P55327	1xPhospho [T191(0); S204(0); S209(96.9); T211(2.9); T212(0.1); T213(0.1)]	0.01	-6.64385619	8.35E-17
P55327	1xPhospho [S171(0); T173(0); S176(100)]	1.247	0.318461465	0.907438338
P55327	2xPhospho [S151(0); S155(100); S156(0); S159(100); T162(0)]	1.204	0.267835392	0.945786032
P55327	1xPhospho [S176(100)]	0.836	-0.258425153	0.973763863
P55884	3xPhospho [S152(100); S154(100); S164(100)]	100	6.64385619	8.35E-17
P55884	2xPhospho [S152(100); S154(100); S164(0)]	0.818	-0.289827252	0.962617576
P56211	1xPhospho [T22(0.1); S23(99.9)]	1.63	0.704871964	0.764901318
P56537	1xPhospho [S230(0); T231(0); T234(0.3); S235(5); S239(94.4); S243(0.3); T245(0)]	1.247	0.318461465	0.91490394
P57740	1xPhospho [S4(100); S10(0); S11(0)]	0.832	-0.265344567	0.984390586
P58107	1xPhospho [S2716(100); S2718(0); T2722(0); S2723(0); T2730(0)]; 1xPhospho [S3250(100); S3252(0); T3256(0); S3257(0); T3264(0)]; 1xPhospho [S3784(100); S3786(0); T3790(0); S3791(0); T3798(0)]; 1xPhospho [S4318(100); S4320(0); T4324(0); S4325(0); T4332(0)]; 1xPhospho [S4852(100); S4854(0); T4858(0); S4859(0); T4866(0)]	0.435	-1.200912694	0.267894422
P60174	1 1xPhospho [S21(100); T28(0)]	0.389	-1.36215794	0.050687442
P60174	1 1xPhospho [S21(100); T28(0)]	0.458	-1.126580497	0.716711094
P60468	1xMet-loss [N-Term]; 1xPhospho [T5(0); S7(0); T9(0); S13(0); S14(0); S17(100); S19(0)]	5.782	2.531568609	0.000232868
P60981	1xMet-loss+Acetyl [N-Term]; 1xPhospho [S3(100)]	1.303	0.381837084	0.878612438
P61244	2 1xMet-loss+Acetyl [N-Term]; 1xPhospho [S2(100); S11(0)]	100	6.64385619	8.35E-17
P61978	1xPhospho [S214(0); S216(100)]	100	6.64385619	8.35E-17
P61978	1xPhospho [T107(0); S116(95.9); T118(3.9); T120(0.2); S121(0); S127(0); Y135(0); Y138(0)]	0.684	-0.54793177	0.724913856
P61978	2xPhospho [T107(0); S116(99.8); T118(4.8); T120(90.9); S121(4.6); S127(0); Y135(0); Y138(0)]	0.718	-0.477944251	0.864539087
P61978	1xPhospho [S379(100); Y380(0); T389(0); T390(0); T393(0)]	0.677	-0.562772261	0.908086456
P62070	1xPhospho [S186(100); T190(0)]	1.4	0.485426827	0.821573905
P62070	1xPhospho [S186(100); T190(0)]	0.845	-0.242976753	0.966549726
P62070	1xPhospho [S186(100); T190(0)]	1.012	0.01720929	0.977823205
P62269	1xPhospho [Y95(98.1); S96(1.9)]	0.589	-0.763660461	0.796472939
P62328	1xPhospho [T21(2); T23(98); S31(0)]	0.067	-3.899695094	0.000208215
P62328	1xPhospho [S31(0); T34(100)]	0.784	-0.351074441	0.879063032
P62491	1xPhospho [S190(99.9); S192(0.2); T203(0); T204(0)]	2.611	1.384602458	0.243156974
P62491	1xPhospho [S190(100); S192(0); T203(0); T204(0)]	1.552	0.634128558	0.749797187
P62753	2xPhospho [S235(0); S236(100); S240(100); T241(0); S242(0)]	3.474	1.796597754	0.004169439
P62753	1xPhospho [S6(100); T10(0)]	0.754	-0.407363571	0.976345951
P62753	2xPhospho [S235(100); S236(100)]	0.981	-0.027674958	0.990299313
P62753	3xPhospho [S235(100); S236(100); S240(100); T241(0); S242(0)]	1.013	0.018634174	1
P63165	1xMet-loss+Acetyl [N-Term]; 1xPhospho [S2(100); S9(0); T10(0)]	1.126	0.171206827	0.965248196
P63165	1xMet-loss+Acetyl [N-Term]; 1xPhospho [S2(100); S9(0); T10(0)]	0.854	-0.227692025	0.971196575
P63313	1xMet-loss [N-Term]; 1xPhospho [S12(100)]	3.225	1.689299161	0.054217096
P63313	1xPhospho [T34(0); S41(100); S44(0)]	1.83	0.871843649	0.47054459
P63313	1xPhospho [T28(0); T31(0); T34(100)]	1.655	0.726831217	0.557471588
P63313	1xMet-loss+Acetyl [N-Term]; 1xPhospho [S12(100)]	1.183	0.242450074	0.936043234
P67870	1xPhospho [Y197(0); S205(0); S209(100)]	0.793	-0.334607229	0.94813191
P68133	1xPhospho [S54(100); Y55(0); S62(0)]; P63261 1xPhospho [S52(100); Y53(0); S60(0)]	1.381	0.46571332	0.810764434
P68363	1xPhospho [Y432(0); S439(100); Y451(0)]	0.096	-3.380821784	0.001871363
P68402	1xMet-loss+Acetyl [N-Term]; 1xPhospho [S2(100); S6(0)]	0.586	-0.77102743	0.618224241
P78316	1xPhospho [Y91(0); S93(0); S96(100)]	2.153	1.10634832	0.260501789
P78316	1xPhospho [Y91(0); S93(0); S96(100)]	1.865	0.89917563	0.552537375
P78316	2xPhospho [S146(100); S148(100); T155(0); S157(0); T161(0)]	0.795	-0.330973234	0.950730609

P78345	1xPhospho [S253(100); T255(0)]	1.219	0.285698126	0.908083211
P78346	1xPhospho [S251(100); S261(0)]	1.213	0.27857955	0.907609406
P78347	2 1xPhospho [S789(3.8); S790(96.2); T795(0); T796(0); S798(0); T809(0)]	100	6.64385619	8.35E-17
P78347	2 2xPhospho [S243(100); Y248(0); Y249(0); Y251(0); S256(0); S259(0); S260(0); T266(0); Y277(0); S278(99.7); S281(0.3)]	2.063	1.044743821	0.403398043
P78347	2 2xPhospho [S207(0); S210(100); S214(100)]	0.634	-0.657445255	0.936043234
P78347	2 2xPhospho [S633(4); S636(96.1); S638(99.8); T646(0); T657(0); S658(0)]	0.799	-0.323732592	0.971973958
P78347	2 1xPhospho [S207(0); S210(100); S214(0)]	1.022	0.031395196	0.973763863
P78356	2xPhospho [S319(4.7); Y320(0); T322(95.3); S326(100); S332(0)]	1	0	0.980895643
P78536	1xPhospho [S791(100); T796(0); T801(0)]	1.025	0.03562391	0.985318278
P79522	2 2xPhospho [T14(0); T26(100); S33(100)]	100	6.64385619	8.35E-17
P82094	2xPhospho [S328(0); S330(100); S333(97.6); S336(2.4)]	0.01	-6.64385619	8.35E-17
P83731	1xPhospho [S149(100)]	0.833	-0.263611599	0.981858798
P85037	2xPhospho [S236(0); S239(0.3); S243(0.3); T245(4.9); T247(94.4); S249(0.3); S253(0.3); S257(99.7)]	0.187	-2.418889825	0.002474229
P85037	2xPhospho [S431(0); T436(100); S441(100)]	2.519	1.332851122	0.117213667
P85037	1xPhospho [S416(100); S420(0); T422(0); S428(0)]	2.227	1.155101558	0.305105183
P85037	3xPhospho [S416(100); S420(100); T422(0); S428(100)]	2.072	1.051024003	0.358064901
P85037	3xPhospho [S431(0); T436(100); S441(100); S445(100); S455(0)]	1.737	0.796597754	0.431663411
P85037	2xPhospho [S416(100); S420(100); T422(0); S428(0)]	1.696	0.76213617	0.517670867
P85037	2xPhospho [S416(100); S420(100); T422(0); S428(0)]	1.505	0.589763487	0.719382659
P85037	3xPhospho [S236(0.3); S239(99.7); S243(0.3); T245(94.9); T247(4.9); S249(0); S253(0); S257(100)]	0.566	-0.821126042	0.729469105
P85037	2xPhospho [S288(0); T294(0.5); S295(99.5); S299(100); S304(0); S309(0); Y310(0); S319(0); S320(0)]	0.696	-0.522840789	0.85992414
P85037	1xPhospho [S213(100); Y219(0); S223(0)]	1.205	0.269033146	0.899435673
P85037	1xPhospho [S431(0); T436(0); S441(100)]	1.284	0.360645202	0.901860353
P85037	1xPhospho [S445(100); S455(0)]	1.228	0.296310561	0.923820271
P85037	2xPhospho [T202(0); S203(0); Y205(0); S213(100); Y219(0); S223(100)]	0.776	-0.365871442	0.962617576
P85037	2xPhospho [S213(100); Y219(0); S223(100)]	0.886	-0.174621396	0.963156927
P85037	2xPhospho [S431(0); T436(0); S441(100); S445(100); S455(0)]	0.938	-0.092340172	0.989304921
P98082	1xPhospho [S393(0); S394(0); S401(100)]	100	6.64385619	8.35E-17
P98175	3xPhospho [Y732(0); S733(100); S736(100); S738(100)]	0.09	-3.473931188	0.000191368
P98175	2xPhospho [Y838(0); S842(100); T843(0.1); S845(100); T852(0)]	9.268	3.212258044	0.000497271
P98175	1xPhospho [S718(0); S723(100)]	2.161	1.111699072	0.374360028
P98175	2xPhospho [Y57(0); S60(97.6); S61(2.4); S65(100); S69(0); Y70(0); S73(0); S76(0); T78(0)]	1.695	0.761285273	0.617610466
P98175	1xPhospho [S89(100); T91(0)]	1.586	0.665392771	0.701118528
P98175	3xPhospho [Y57(0); S60(100); S61(100); S65(100); S69(0); Y70(0); S73(0); S76(0); T78(0)]	0.733	-0.448114897	0.974924677
P98175	2xPhospho [Y732(0); S733(0); S736(100); S738(100)]	0.947	-0.078563669	1
P98179	1xPhospho [Y143(0); Y146(0); S147(100); Y151(0)]	1.049	0.069014678	0.980895643
Q00341	1xPhospho [T28(0); S31(100); S35(0); T39(0); Y40(0)]	1.492	0.577247536	0.811661161
Q00341	1xMet-loss+Acetyl [N-Term]; 1xPhospho [S2(0); S3(0); T8(0); S11(100)]	1.136	0.183962835	0.9616322
Q00587	1xPhospho [S121(100); Y130(0); S132(0)]	0.153	-2.708396442	0.002888572
Q00587	2xPhospho [S190(0); S192(100); S195(100)]	0.375	-1.415037499	0.532144188
Q00587	2xPhospho [S350(100); S353(100)]	1.849	0.886745225	0.603050301
Q00587	2xPhospho [S190(0); S192(100); S195(100)]	0.584	-0.775959726	0.752453774
Q00653	1xPhospho [S22(0); S23(100)]	100	6.64385619	8.35E-17
Q00839	1xPhospho [S271(100); T286(0); T292(0); Y293(0)]	0.483	-1.049904906	0.351184483
Q00839	1xPhospho [S59(0); S66(100)]	1.692	0.758729568	0.522040717
Q00839	1xPhospho [Y669(0); S673(100)]	1.679	0.74760223	0.701313837
Q00839	2xPhospho [S59(100); S66(100)]	0.681	-0.554273297	0.719352351
Q00839	1xPhospho [S59(0); S66(100)]	1.305	0.384049807	0.864391305
Q00839	2xPhospho [S59(100); S66(100)]	0.771	-0.375197235	0.864937954
Q00839	1xPhospho [S187(0); S188(0); T191(1.6); S192(98.4); T197(0)]	1.239	0.309176187	0.875977821
Q00839	2xPhospho [S187(0.1); S188(0.1); T191(99.8); S192(100); T197(0)]	1.303	0.381837084	0.886912356
Q00839	1xPhospho [S271(100); T286(0); T292(0); Y293(0)]	0.895	-0.160040413	1
Q01082	2xPhospho [T2328(100); T2331(0); S2332(0); T2335(0); T2337(0); S2338(0); S2340(50); S2341(50)]	0.06	-4.058893689	5.37E-06
Q01082	1xPhospho [S2128(0); S2138(100)]; Q01082-3 1xPhospho [S2115(0); S2125(100)]	2.874	1.523060062	0.147626049
Q01082	1xPhospho [S2358(100)]	1.345	0.427606173	0.879690128
Q01082	3 2xPhospho [T6(33.3); S7(33.3); S8(33.3); S10(0.1); S14(99.9); Y17(0); T18(0); Y23(0); Y25(0)]	0.747	-0.420819852	0.921446515
Q01105	2 1xPhospho [S15(0); T23(0); S24(100)]	2.678	1.421155961	0.119484707
Q01105	2 2xPhospho [S15(0); T23(100); S24(100)]	0.766	-0.384583703	0.934569753
Q01167	3xPhospho [S369(97.8); S373(1.1); S381(1.1); S384(0); S385(0); T389(0); S392(50); S394(50); S398(99.9)]	0.01	-6.64385619	8.35E-17
Q01167	2xPhospho [S369(0); S373(0); S381(0); S384(0.2); S385(1); T389(1); S392(49.4); S394(49.4); S398(98.9)]	0.01	-6.64385619	8.35E-17
Q01167	1xPhospho [S369(0); S373(100); S381(0); S384(0); S385(0); T389(0); S392(0); S394(0)]	0.01	-6.64385619	8.35E-17
Q01167	2xPhospho [S424(100); S428(100); S431(0); S432(0); T438(0)]	100	6.64385619	8.35E-17
Q01167	2xPhospho [S180(0); T183(0); T189(0); S195(0); S199(91.1); T201(8.1); T203(0.7); S205(0.1); S209(0.1); S212(8.1); S213(91.8)]	2.261	1.176960992	0.330526379
Q01167	3xPhospho [S369(100); S373(100); S381(0); S384(0); S385(0.2); T389(4); S392(91.7); S394(4)]	0.56	-0.836501268	0.548439372
Q01167	2xPhospho [S369(100); S373(100); S381(0); S384(0); S385(0); T389(0); S392(0); S394(0)]	0.639	-0.646112164	0.763548117
Q01167	1xPhospho [S424(0); S428(100); S431(0); S432(0); T438(0)]	0.626	-0.675765438	0.920957334
Q01433	1xPhospho [S100(100)]	0.921	-0.118726939	0.988809106
Q01518	2xPhospho [S295(0); S301(0); T307(98); S308(2); S310(100)]	1.841	0.880489627	0.380617646

Q01518	2xPhospho [S295(0); S301(0); T307(97.1); S308(3); S310(99.9)]	1.56	0.641546029	0.776790129
Q01518	1xPhospho [S295(0); S301(0); T307(1.7); S308(98.3); S310(0)]	0.786	-0.347398782	0.885613358
Q01581	1xPhospho [T471(0); T476(0); S486(0); T490(0); S495(100)]	0.583	-0.778432211	0.689396319
Q01581	1xPhospho [T506(0); S516(100)]	0.896	-0.158429363	0.989838391
Q01813	1xPhospho [S386(100); T393(0); Y394(0)]	0.01	-6.64385619	8.35E-17
Q01813	1xPhospho [S386(100); T393(0); Y394(0)]	0.389	-1.36215794	0.199028486
Q01970	1xPhospho [S537(100)]	2.454	1.295135249	0.289383166
Q01970	1xPhospho [T924(0); T925(0); S926(0); S929(0); T930(0); S931(3); S933(94.1); S934(3)]	1.127	0.172487516	0.965013037
Q02078	2xPhospho [S479(100); T489(0.2); S494(89.4); S496(10.4)]	1.169	0.22527493	0.9234483
Q02241	2 1xPhospho [S155(1.6); S160(98.4)]	1.631	0.705756782	0.689012209
Q02241	2 1xPhospho [S839(100); Y845(0)]	1.084	0.116364757	0.962617576
Q02750	1xPhospho [S212(0); S218(0); S222(100); T226(0)]	0.935	-0.09696173	0.992863986
Q02880	3xPhospho [S1336(100); S1340(100); S1342(100); S1344(0); T1349(0)]	0.01	-6.64385619	8.35E-17
Q02880	1xPhospho [S1400(0); T1403(0); S1413(100); Y1421(0); T1422(0); S1424(0)]	100	6.64385619	8.35E-17
Q02880	2xPhospho [S1522(100); S1524(50); S1526(50)]	0.514	-0.960159735	0.47518367
Q02880	2xPhospho [S1400(100); T1403(0); S1413(0); Y1421(0); T1422(0); S1424(100)]	1.621	0.696884091	0.579751615
Q02880	3xPhospho [S1400(100); T1403(0); S1413(100); Y1421(0); T1422(0); S1424(100)]	0.697	-0.520769439	0.749797187
Q02880	2xPhospho [S1522(100); S1524(100); S1526(0)]	0.671	-0.575615328	0.816750637
Q02880	3xPhospho [S1400(100); T1403(0); S1413(100); Y1421(0); T1422(0); S1424(100)]	1.372	0.456280482	0.880267055
Q02880	3xPhospho [S1522(100); S1524(100); S1526(100)]	0.602	-0.732164608	0.907438338
Q02880	1xPhospho [S1457(0); S1461(0); S1466(100); S1471(0); S1473(0); S1476(0); S1478(0)]	1.165	0.220329955	0.934211842
Q02880	1xPhospho [T1575(0); S1576(0); S1581(100); S1588(0); T1592(0); S1596(0)]	1.066	0.092207438	0.971196575
Q02880	2xPhospho [T1575(50); S1576(50); S1581(100); S1588(0); T1592(0); S1596(0)]	1.059	0.082702589	0.973263372
Q02880	1xPhospho [S1466(100); S1471(0); S1473(0); S1476(0); S1478(0)]	1	0	0.973763863
Q02880	2xPhospho [S1457(0); S1461(0); S1466(100); S1471(0); S1473(100); S1476(0); S1478(0)]	1.081	0.112366523	0.976345951
Q02880	1xPhospho [T1575(96.1); S1576(2); S1581(2); S1588(0); T1592(0); S1596(0)]	1.096	0.132247798	0.980895643
Q02880	2xPhospho [S1400(0); T1403(0); S1413(100); Y1421(0); T1422(0); S1424(100)]	0.893	-0.16326792	0.983092362
Q02952	2xPhospho [S490(0); S498(100); S504(0); S505(100)]	0.01	-6.64385619	8.35E-17
Q02952	1xPhospho [T608(0); S612(100)]	0.01	-6.64385619	8.35E-17
Q02952	1xPhospho [S1753(0.1); S1755(99.9); T1760(0)]	0.01	-6.64385619	8.35E-17
Q02952	1xPhospho [S248(97); S253(3); S258(0)]	0.01	-6.64385619	8.35E-17
Q02952	1xPhospho [S627(98.8); S629(1.2)]	100	6.64385619	8.35E-17
Q02952	3xPhospho [T723(0); T725(0); S732(0.1); S741(91.6); S742(9.1); S743(99.2); S749(99.2); T751(0.9); S757(0); T758(0); S761(0)]	0.244	-2.035046947	0.011673285
Q02952	3xPhospho [T723(0); T725(0); S732(18.7); S741(18.7); S742(81.4); S743(81.4); S749(100); T751(0); S757(0); T758(0); S761(0)]	0.304	-1.717856771	0.031316279
Q02952	1xPhospho [T608(0); S612(100)]	0.247	-2.017417053	0.036316854
Q02952	2xPhospho [T723(94.5); T725(5.5); S732(0); S741(0); S742(0); S743(0); S749(100); T751(0); S757(0); T758(0); S761(0)]	0.332	-1.590744853	0.069898924
Q02952	2xPhospho [S627(100); S629(100)]	0.432	-1.210896782	0.105051116
Q02952	2xPhospho [T351(0); S353(0); S364(100); S371(100); Y374(0)]	0.45	-1.152003093	0.135635756
Q02952	2xPhospho [S280(0); S283(100); T285(0.1); S286(99.9); T289(0); S290(0); T292(0); S294(0); T295(0)]	0.422	-1.244685096	0.219009307
Q02952	1xPhospho [T608(0); S612(100)]	0.381	-1.392137097	0.317432231
Q02952	1xPhospho [S248(100); S253(0); S258(0)]	0.53	-0.915935735	0.326910511
Q02952	1xPhospho [S248(100); S253(0); S258(0)]	0.539	-0.891642822	0.457443685
Q02952	2xPhospho [S627(100); S629(100)]	0.548	-0.867752202	0.469401188
Q02952	1xPhospho [S371(100); Y374(0); S381(0); S386(0); S388(0); S392(0); T401(0)]	0.506	-0.98279071	0.5230624
Q02952	1xPhospho [S490(0); S498(0); S504(0); S505(100)]	0.564	-0.826232932	0.550634186
Q02952	2xPhospho [S1287(100); T1290(0); T1293(1.6); S1295(98.4)]	0.453	-1.142417045	0.590872099
Q02952	1xPhospho [T351(0); S353(0); S364(0); S371(100); Y374(0)]	0.651	-0.619270551	0.71990449
Q02952	2xPhospho [S371(100); Y374(0); S381(100); S386(0); S388(0); S392(0); T401(0)]	0.69	-0.535331733	0.737789345
Q02952	1xPhospho [T597(0); S598(100)]	0.742	-0.430508908	0.823333961
Q02952	1xPhospho [S381(100); S386(0); S388(0); S392(0); T401(0)]	0.733	-0.448114897	0.836554173
Q02952	1xPhospho [S544(100); T548(0); S554(0); S557(0)]	1.362	0.445726703	0.848293321
Q02952	2xPhospho [S780(100); S783(100); S787(0); S792(0); T793(0); T796(0); S803(0); S806(0)]	1.36	0.443606651	0.852940464
Q02952	1xPhospho [S885(2.4); S887(97.6); T900(0)]	0.61	-0.713118852	0.856190795
Q02952	2xPhospho [S1324(0); S1328(100); S1331(100)]	0.693	-0.529072743	0.872730156
Q02952	1xPhospho [S780(0); S783(100); S787(0); S792(0); T793(0); T796(0); S803(0); S806(0)]	0.712	-0.490050854	0.889021562
Q02952	1xPhospho [T351(0); S353(0); S364(100)]	0.662	-0.595096878	0.900153261
Q02952	1xPhospho [S248(100); S253(0); S258(0)]	0.824	-0.279283757	0.921519407
Q02952	3xPhospho [S371(100); Y374(0); S381(100); S386(0.8); S388(91); S392(8.2); T401(0)]	0.725	-0.4639471	0.93864152
Q02952	1xPhospho [S1225(0); S1226(100); T1233(0)]	0.805	-0.312939312	0.950730609
Q02952	2xPhospho [S248(100); S253(0); S258(100)]	0.728	-0.457989644	0.954539739
Q02952	1xPhospho [S280(0); S283(0); T285(0); S286(100); T289(0); S290(0); T292(0); S294(0); T295(0)]	0.781	-0.356605547	0.958562891
Q02952	1xPhospho [T469(0); S472(0); T477(0); S483(100)]	0.767	-0.382701517	0.959719138
Q02952	1xPhospho [S627(100); S629(0)]	0.843	-0.246395464	0.9616322
Q02952	1xPhospho [T723(0); T725(0); S732(0); S741(0); S742(0); S743(0); S749(0); T751(0); S757(0); T758(0); S761(100)]	0.758	-0.399730246	0.965351295
Q02952	1xPhospho [T1115(0); T1116(99.9); S1119(0)]	1.081	0.112366523	0.97023559
Q02952	2xPhospho [S1390(100); S1391(100); S1395(0); T1408(0)]	1.07	0.097610797	0.973763863
Q02952	1xPhospho [S1618(0); S1620(0); T1621(0); S1628(100); S1631(0)]	1.033	0.046840254	0.977858961
Q02952	2xPhospho [S381(100); S386(0.1); S388(99.9); S392(0); T401(0)]	1.034	0.048236186	0.980895643
Q02952	1xPhospho [T597(0); S598(100)]	1.027	0.038436182	0.981858798

Q02952	2xPhospho [S780(0); S783(100); S787(0); S792(0); T793(0); T796(0); S803(0); S806(100)]	0.892	-0.164884385	0.983084471
Q02952	2xPhospho [T723(0); T725(0); S732(0); S741(48.4); S742(48.4); S743(3.3); S749(93.7); T751(6.3); S757(0); T758(0); S761(0)]	1.045	0.063502942	0.986075329
Q03164	1xPhospho [S1854(2.2); S1858(97.8)]	1.767	0.82130204	0.664881585
Q03164	2xPhospho [S1106(4.4); S1107(45.7); S1114(45.7); S1115(4.5); S1119(99.9); T1136(0)]	1.21	0.275007047	0.921238122
Q03164	2xPhospho [S3502(0); S3503(0); S3508(0); T3510(0); S3511(100); S3515(0); S3517(0.1); S3518(99.9); S3520(0); S3521(0)]	1.181	0.240008965	0.930641583
Q03164	2xPhospho [S1837(100); T1839(0); T1845(100); S1850(0); T1851(0)]	1.11	0.150559677	0.949541112
Q03164	1xPhospho [S1837(96.4); T1839(3.6); T1845(0); S1850(0); T1851(0)]	1.002	0.002882509	0.972294713
Q03164	2xPhospho [S3026(2.8); S3027(48.6); T3028(48.6); S3036(100); T3038(0)]	0.923	-0.115597447	0.989838391
Q03188	1xPhospho [T69(0); S73(100); S75(0)]	1.182	0.241230036	0.934519081
Q03468	2xPhospho [S429(100); S430(100); S438(0)]	0.608	-0.717856771	0.703278942
Q03468	1xPhospho [S1337(0); S1340(0); S1345(3); S1346(3); T1347(91); S1348(3); T1350(0.1)]	1.419	0.504874589	0.819087074
Q03468	2xPhospho [S486(100); S489(100)]	0.747	-0.420819852	0.94797789
Q03468	2xPhospho [S1337(94.9); S1340(5.1); S1345(24.7); S1346(24.7); T1347(24.7); S1348(24.7); T1350(1.3)]	0.702	-0.510457064	0.965139891
Q03468	2xPhospho [S486(100); S489(100)]	0.958	-0.061902439	0.980895643
Q03701	1xPhospho [T951(0); S959(100)]	1.088	0.121678557	0.961452497
Q03701	1xPhospho [S621(0); S629(100)]	0.848	-0.23786383	0.967050102
Q04323	2xPhospho [Y179(0); S182(3.6); S185(96.4); S199(3.6); S200(96.3); S202(0.1); T207(0)]	0.197	-2.343732465	0.016896209
Q04323	2xPhospho [T65(0.2); S66(0.2); S67(99.6); S75(0); S77(100); S87(0)]	1.641	0.714575239	0.652347789
Q04323	1xPhospho [T65(0); S66(0); S67(0); S75(0); S77(100); S87(0)]	1.515	0.599317794	0.706674105
Q04323	1xPhospho [Y179(0); S182(0); S185(0); S199(0.1); S200(99.8); S202(0.1); T207(0)]	0.822	-0.282789701	0.966027585
Q04656	3xPhospho [S1430(0.7); S1432(99.3); S1435(100); T1443(6.9); S1444(86.2); S1447(6.9)]	0.277	-1.852042119	0.202761754
Q04656	3xPhospho [S1463(100); S1466(100); S1469(100)]	1.06	0.084064265	0.973263372
Q04759	1xPhospho [S695(100)]	1.156	0.209141398	0.94797789
Q04912	2xPhospho [S1019(0); S1031(0); T1032(0); T1033(0); S1039(0.2); S1041(99.8); S1043(100); S1047(0)]	1.239	0.309176187	0.920957334
Q05655	2xPhospho [S302(0.1); S304(99.8); S306(0.1); S307(0.1); Y313(100)]	0.589	-0.763660461	0.772467589
Q05655	1xPhospho [S654(0); S658(0); S664(100)]	1.284	0.360645202	0.899435673
Q05655	1xPhospho [S117(0); Y120(0); S130(100)]	1.171	0.227741076	0.930376591
Q05655	2xPhospho [S299(100); S302(100); S304(0); S306(0); S307(0); Y313(0)]	1.186	0.24610401	0.942907978
Q05655	1xPhospho [S302(0); S304(100); S306(0); S307(0); Y313(0)]	0.824	-0.279283757	0.96293109
Q05655	1xPhospho [S117(0); Y120(0); S130(100)]	0.983	-0.024736678	0.989898705
Q05682	5 2xPhospho [T492(100); S498(95.9); S504(4.2); S512(0); S513(0)]	1.476	0.561692721	0.779248382
Q05682	5 1xPhospho [S12(100); S17(0); Y21(0)]	0.627	-0.673462652	0.780165563
Q05682	5 1xPhospho [S196(100)]	0.676	-0.564904848	0.802946045
Q05682	5 2xPhospho [S12(100); S17(100); Y21(0)]	0.619	-0.691988685	0.830932391
Q05682	5 1xPhospho [T492(0); S498(100); S504(0); S512(0); S513(0)]	0.75	-0.415037499	0.973599548
Q05682	5 1xPhospho [S430(99.9); T434(0.1); S439(0)]	0.969	-0.045431429	0.981858798
Q05682	5 2xPhospho [S462(95.5); S463(4.5); T465(0); T469(95.5); T474(4.5)]	0.923	-0.115597447	1
Q05GC8	2xPhospho [T632(0); S634(100); S638(100)]	1.507	0.591679417	0.737789345
Q06210	1xPhospho [S261(100); T262(0); T263(0)]	0.805	-0.312939312	0.962617576
Q06587	1xPhospho [S163(0); T166(0); T167(0.2); T168(4); S170(95.9); S187(0); S188(0); S190(0); S194(0)]	2.288	1.194087052	0.358007677
Q06587	2xPhospho [S163(0); T166(3.9); T167(92.2); T168(3.9); S170(0); S187(0.2); S188(95.7); S190(4.1); S194(0)]	0.553	-0.854648614	0.73347811
Q06587	2xPhospho [T238(98.9); T243(1.1); S248(0); S254(100)]	0.792	-0.336427665	0.908086456
Q07666	2xPhospho [S14(0); S15(0); S18(100); S20(100); S24(0); S29(0)]	0.01	-6.64385619	8.35E-17
Q07666	1xPhospho [T33(2.4); S35(97.7)]	2.564	1.358396262	0.183343379
Q07666	2xPhospho [S58(99.6); T61(0.5); S70(0); T72(0); T77(0); T84(0); S90(3.2); T92(48.4); S94(48.4)]	2.039	1.027861775	0.308158065
Q07666	1xPhospho [S18(1.4); S20(98.6); S24(0); S29(0)]	2.081	1.057276965	0.414966031
Q07666	1xPhospho [S18(0); S20(100); S24(0); S29(0)]	1.064	0.089498151	0.973263372
Q07666	1xPhospho [S58(99.7); T61(0.3); S70(0); T72(0); T77(0); T84(0); S90(0); T92(0); S94(0)]	0.886	-0.174621396	0.97998947
Q07889	2xPhospho [S1242(0); S1252(0); S1254(0); T1257(0); T1263(100); S1265(95.4); T1269(4.6)]	0.01	-6.64385619	8.35E-17
Q07889	2xPhospho [S1275(100); T1279(0); S1286(100)]	1.354	0.437227739	0.831039794
Q08211	1xPhospho [S77(0); S87(100); T92(0); T94(0); T97(0); T98(0); T107(0); T108(0)]	0.559	-0.839079812	0.71026613
Q08357	2xPhospho [Y430(0); S432(99.9); Y433(0); S434(50); S435(50); Y436(0)]	0.01	-6.64385619	8.35E-17
Q08357	2xPhospho [Y430(0); S432(100); Y433(0); S434(4.1); S435(95.7); Y436(0.2)]	5.147	2.363731783	0.002629058
Q08357	1xPhospho [S385(100); Y386(0); T387(0); Y389(0); T390(0); T401(0)]	0.391	-1.354759487	0.229641558
Q08357	2xPhospho [S256(100); S259(95.2); S261(4.8); S268(0)]	0.363	-1.461958547	0.487476349
Q08357	2xPhospho [S253(0.2); S256(99.8); S259(100); S261(0); S268(0)]	0.511	-0.968604804	0.66845931
Q08357	2xPhospho [S253(0); S256(100); S259(100); S261(0)]	0.817	-0.291592017	0.96229367
Q08378	1xPhospho [T251(0); S254(0); S259(0); S268(100); T269(0); S272(0)]	1.824	0.86710573	0.508957366
Q08752	2xPhospho [S198(100); S201(100)]	0.67	-0.577766999	0.875084133
Q08752	2xPhospho [S198(100); S201(100)]	0.73	-0.454031631	0.969680228
Q08752	1xPhospho [S198(100); S201(0)]	0.87	-0.200912694	0.989898705
Q08945	1xPhospho [S657(0); S659(100)]	1.757	0.813114191	0.574614598
Q08945	2xPhospho [S437(100); Y438(0); Y441(0); S444(100); Y452(0)]	0.751	-0.413115187	0.880267055
Q08945	1xPhospho [S437(0); Y438(0); Y441(0); S444(100); Y452(0)]	0.825	-0.277533976	0.93019184
Q08945	1xPhospho [S437(0); Y438(0); Y441(0); S444(100); Y452(0)]	1.043	0.060739158	0.988508396
Q08999	2xPhospho [S1051(0); Y1057(0); S1059(100); T1065(0.2); S1068(99.8)]	0.01	-6.64385619	8.35E-17
Q08AD1	2xPhospho [S1311(0); S1313(100); S1319(100); S1321(0)]	8.212	3.037733627	0.002144274
Q08AD1	1xPhospho [S462(0); S464(100); T469(0); S473(0); S476(0)]	0.938	-0.092340172	0.985870803
Q09666	1xPhospho [Y5836(0); T5839(0); S5841(100); T5845(0)]	100	6.64385619	8.35E-17

Q09666	2xPhospho [S2387(100); S2397(100)]	0.128	-2.965784285	0.000738672
Q09666	2xPhospho [S5749(100); S5752(100); S5762(0); S5763(0)]	0.368	-1.442222329	0.071839558
Q09666	1xPhospho [S2387(0); S2397(100)]	0.305	-1.713118852	0.254834652
Q09666	1xPhospho [S2708(100)]; 1xPhospho [S3092(100)]; 1xPhospho [S3220(100)]; 1xPhospho [S3544(100)]; 1xPhospho [S3746(100)]; 1xPhospho [S3874(100)]; 1xPhospho [S4722(100)]	0.47	-1.089267338	0.452638679
Q09666	2xPhospho [S210(0); S212(0); S216(100); T218(1.6); S220(98.4)]	0.562	-0.831357964	0.570904793
Q09666	1xPhospho [S570(100); S572(0)]	0.399	-1.325539348	0.584435247
Q09666	1xPhospho [T5824(0); S5830(100)]	0.414	-1.272297327	0.607772032
Q09666	1xPhospho [S5110(100)]	0.507	-0.979942348	0.714293599
Q09666	1xPhospho [S5749(0); S5752(0); S5762(1.9); S5763(98.1)]	0.446	-1.164884385	0.748856955
Q09666	1xPhospho [S5448(100)]	0.478	-1.064917477	0.751995949
Q09666	1xPhospho [S539(100); T545(0.1); T551(0); T553(0)]	0.568	-0.816037165	0.863653123
Q09666	1xPhospho [S210(0); S212(0); S216(100); T218(0); S220(0)]	0.768	-0.380821784	0.933452347
Q09666	1xPhospho [S5641(100); S5643(0); S5651(0); S5653(0)]	0.739	-0.436353731	0.939053555
Q09666	1xPhospho [S93(100); T99(0); T101(0)]	0.798	-0.325539348	0.942954921
Q09666	3xPhospho [S5745(94.8); S5746(10.4); S5749(94.8); S5752(100); S5762(0); S5763(0)]	0.753	-0.40927823	0.958702638
Q09666	1xPhospho [S176(0); S177(100)]	0.806	-0.311148256	0.963219686
Q09666	3xPhospho [S5745(0); S5746(0); S5749(100); S5752(100); S5762(0); S5763(100)]	1.041	0.057970069	0.967050102
Q09666	1xPhospho [S501(0); S508(0); S511(100)]	1	0	0.971937853
Q09666	1xPhospho [S5530(100); S5542(0)]	0.855	-0.226003675	0.971973958
Q09666	2xPhospho [S5530(100); S5542(100)]	0.841	-0.249822294	0.97998947
Q09666	2xPhospho [T5729(0); S5731(0); S5735(100); S5737(0); S5739(100)]	0.881	-0.182786076	0.980148962
Q09666	1xPhospho [S4953(100); S4960(0)]	0.826	-0.275786313	0.980895643
Q09666	1xPhospho [S135(100); T146(0); S148(0)]	0.928	-0.10780329	0.982029512
Q09666	3xPhospho [S5749(100); S5752(100); S5762(0); S5763(100)]	0.892	-0.164884385	0.999717367
Q09666	1xPhospho [S281(0); S282(0); S283(100)]	0.84	-0.251538767	1
Q0JRZ9	2xPhospho [S478(0); S488(100); T492(0); S493(4.1); T495(0.2); S496(95.8)]	6.053	2.597650351	0.000344791
Q10570	1xPhospho [S765(0.1); S766(99.9)]	1.102	0.140124224	0.962617576
Q12774	2xPhospho [S445(100); S450(100); S452(0)]	0.41	-1.286304185	0.585755693
Q12774	1xPhospho [S1011(100); S1018(0); S1019(0); S1026(0)]	0.774	-0.369594529	0.973763863
Q12789	1xPhospho [T725(0); S726(0); S739(100); S746(0); S748(0); S751(0); S754(0); S756(0); S757(0)]	0.473	-1.080087911	0.565309099
Q12789	2xPhospho [S1062(100); S1063(0); T1064(0); S1068(100); S1073(0); S1080(0)]	1.142	0.191562651	0.933107775
Q12789	2xPhospho [S1865(100); S1868(100); T1873(0); T1878(0); T1881(0)]	1.205	0.269033146	0.939053555
Q12789	2xPhospho [S1840(91.6); S1841(4.2); S1842(4.2); S1845(0); S1854(95.6); S1856(4.4)]	1.08	0.111031312	0.943796521
Q12802	2xPhospho [S1642(0); S1645(100); S1647(100); S1653(0)]	1.377	0.461528559	0.786602611
Q12802	1xPhospho [S2390(0); T2391(0); S2398(94.4); T2400(2.8); S2402(2.8)]	1.212	0.277389699	0.9234483
Q12802	2xPhospho [S1927(0); S1929(100); T1930(0); S1932(100)]	0.999	-0.001443417	1
Q12872	1xPhospho [S709(0); S712(0); S716(0); S717(0); T718(0); T719(0); T725(0); T732(0); S742(5.8); S743(94.2)]	8.822	3.14110576	0.000307537
Q12872	1xPhospho [S279(0); S282(2.6); S283(97.4); Y296(0); S300(0); S304(0)]	1.275	0.350497247	0.879426164
Q12872	1xPhospho [S604(100); S610(0); S616(0); S617(0); S618(0); S619(0); T623(0)]	1.2	0.263034406	0.93019184
Q12872	2xPhospho [S824(0); T827(0); Y829(0); S832(99.9); S834(100); S838(0.1)]	1.004	0.005759269	1
Q12904	1xPhospho [S140(100); S144(0); S147(0); S153(0)]	0.01	-6.64385619	8.35E-17
Q12904	1xPhospho [S140(100); S144(0); S147(0); S153(0)]	0.705	-0.504304837	0.87621477
Q12923	2xPhospho [S1082(100); S1085(100); S1086(0)]	0.442	-1.177881725	0.679613588
Q12923	2xPhospho [S908(100); T909(0); S911(100); S914(0); S915(0); T916(0)]	0.797	-0.327348371	0.988250665
Q12923	1xPhospho [S240(100); S245(0)]	0.919	-0.121863233	0.989898705
Q12948	2xPhospho [T228(8.7); T232(90.4); S235(0.9); S241(100); S248(0); S250(0)]	0.504	-0.988504361	0.730212284
Q12959	2 2xPhospho [S693(0); T697(0); S698(100); T705(0); S706(0.3); S709(94.2); S711(5.2); S713(0.3); S714(0); Y715(0)]	2.519	1.332851122	0.352920541
Q12959	2 1xPhospho [S693(0); T697(0); S698(0); T705(0); S706(0); S709(100); S711(0); S713(0); S714(0); Y715(0)]	1.511	0.595503661	0.660888692
Q12959	2 1xPhospho [S693(0); T697(0); S698(0); T705(0); S706(0); S709(98.2); S711(1.8); S713(0); S714(0); Y715(0)]	1.597	0.675364313	0.688026385
Q12986	1xPhospho [Y48(0); S49(0); S50(100); S57(0)]	0.789	-0.341902795	0.96229367
Q12986	2xPhospho [S150(97.1); S152(51.4); S154(51.4)]	1.076	0.105678078	0.96293109
Q12996	1xPhospho [S691(100); Y708(0)]	1.452	0.538041453	0.786760931
Q13033	1xPhospho [S240(0); S241(0); T247(0); S257(100)]	100	6.64385619	8.35E-17
Q13033	1xPhospho [S240(0); S241(0); T247(0); S257(100)]	0.712	-0.490050854	0.879063032
Q13033	1xPhospho [S229(100)]	0.988	-0.017417053	1
Q13043	1xPhospho [T175(0.1); T177(99.9)]; Q13188 1xPhospho [T172(0.1); T174(99.9)]	0.751	-0.413115187	0.973263372
Q13045	1xPhospho [S856(0); S860(100)]	2.729	1.448372395	0.274090177
Q13049	2xPhospho [S328(0); S335(100); S339(100)]	0.528	-0.921390165	0.797921038
Q13085	1xPhospho [S23(0); S25(0); S29(100); S34(0)]	0.54	-0.888968688	0.6093249
Q13085	2xPhospho [S1257(0.2); S1259(99.8); S1263(100); T1265(0); T1272(0); S1273(0); Y1275(0)]	0.732	-0.450084446	0.806224461
Q13085	2xPhospho [S23(2.6); S25(97.4); S29(100); S34(0)]	0.695	-0.524915117	0.872729954
Q13085	2xPhospho [S1257(5); S1259(95); S1263(99.7); T1265(0.3); T1272(0); S1273(0); Y1275(0)]	0.634	-0.657445255	0.92054942
Q13085	2xPhospho [S1257(4.4); S1259(95.6); S1263(99.8); T1265(0.2); T1272(0); S1273(0); Y1275(0)]	0.885	-0.17625064	0.980895643
Q13085	1xPhospho [S77(0); S78(0); S80(100)]	0.979	-0.030619235	0.99811985
Q13111	3xPhospho [S767(1.3); T770(10.4); S772(88.3); S775(99.2); S777(1.5); T778(0.4); T779(0.4); Y780(0.1); T783(88); T785(10.2); S787(0.2); S794(0)]	100	6.64385619	8.35E-17
Q13111	1xPhospho [T108(0.7); S109(0.1); S113(0.1); T114(0.1); T120(7.2); S123(84.8); S129(7.2)]	0.119	-3.070966521	0.006763337
Q13111	1xPhospho [S65(100); S71(0); T74(0); S83(0)]	1.681	0.749319725	0.64773208
Q13111	1xPhospho [S775(100); S777(0); T778(0); T779(0); Y780(0); T783(0); T785(0); S787(0); S794(0)]	1.046	0.064882852	0.99184412

Q13111	2xPhospho [S767(0); T770(4.9); S772(95.1); S775(100); S777(0); T778(0); T779(0); Y780(0); T783(0); T785(0); S787(0); S794(0)]	0.975	-0.036525876	0.999714477
Q13112	1xPhospho [S409(0); S410(100); T419(0); S422(0)]	1.615	0.691534165	0.676726843
Q13112	2xPhospho [S403(0.8); T405(11.3); S409(94); S410(94); T419(0); S422(0)]	0.825	-0.277533976	0.980895643
Q13123	1xPhospho [S442(0); S444(0); Y445(0); Y449(0); T452(0); S460(100); Y466(0); S467(0)]	6.112	2.611644543	0.000341277
Q13127	2xPhospho [S854(2.2); S856(97.8); T857(0); S861(0); S864(100)]	0.985	-0.02180437	0.980895643
Q13129	1xPhospho [T1623(0); S1626(0); S1628(99.8); S1632(0.1); S1633(0.1); T1639(0); S1644(0); S1645(0)]	100	6.64385619	8.35E-17
Q13129	1xPhospho [S632(100); S634(0); S642(0)]	100	6.64385619	8.35E-17
Q13131	1xPhospho [T183(98.4); S184(1.6); S187(0); Y190(0); S197(0)]	1.441	0.527070336	0.776177942
Q13136	3xPhospho [S677(0); S679(0); S680(0); Y684(0); S687(32.2); S688(32.2); S691(32.2); S692(1.7); S693(1.7); S697(0); S700(100); T701(100)]	0.01	-6.64385619	8.35E-17
Q13136	2xPhospho [S773(0); T774(0); S776(0); S782(0); S785(0); S786(0); S787(0.2); S789(4); S790(96); S793(99.8)]	0.076	-3.717856771	7.45E-05
Q13136	2xPhospho [S773(0); T774(0); S776(0); S782(0); S785(0); S786(0); S787(0); S789(50.2); S790(50.2); S793(99.7)]	0.096	-3.380821784	0.002248926
Q13136	2xPhospho [S238(0); S239(0); S242(100); S244(100)]	0.732	-0.450084446	0.806224461
Q13136	3xPhospho [S238(0); S239(100); S242(100); S244(100)]	1.153	0.205392513	0.962617576
Q13136	1xPhospho [S773(0); T774(0); S776(0); S782(100); S785(0); S786(0); S787(0); S789(0); S790(0); S793(0)]	1.121	0.164786278	0.965139891
Q13136	1xPhospho [T761(0); S763(100)]	0.896	-0.158429363	0.980895643
Q13151	1xPhospho [Y180(0); S181(0); S188(100)]	1.26	0.333423734	0.883306273
Q13177	1xPhospho [S32(0.1); S36(99.9); S42(0)]	100	6.64385619	8.35E-17
Q13177	1xPhospho [S19(100); S20(0); T21(0); S24(0); T25(0)]	3.331	1.735955354	0.028916674
Q13177	1xPhospho [S197(100); S209(0)]	1.455	0.541019153	0.746167942
Q13177	3xPhospho [S19(66.7); S20(66.7); T21(66.7); S24(0); T25(0); S32(98.1); S36(0.9); S42(0.9)]	1.599	0.677169939	0.749797187
Q13177	2xPhospho [S19(100); S20(99.8); T21(0.2); S24(0); T25(0)]	1.424	0.509949146	0.775418491
Q13177	1xPhospho [Y139(0); S141(100); T143(0); S152(0); T154(0)]	1.433	0.51903861	0.797966154
Q13177	1xPhospho [S55(100); S58(0); T60(0)]	1.478	0.563646269	0.797966154
Q13177	2xPhospho [S19(99.8); S20(99.8); T21(0.3); S24(0); T25(0); S32(0); S36(0); S42(0)]	1.317	0.397255346	0.827205803
Q13177	2xPhospho [S197(100); S209(100)]	1.244	0.314986485	0.908086456
Q13177	1xPhospho [S152(98.5); T154(1.5)]	1.248	0.319617934	0.930376591
Q13177	1xMet-loss+Acetyl [N-Term]; 1xPhospho [S2(100)]	1.204	0.267835392	0.933107775
Q13177	1xPhospho [Y139(0); S141(100); T143(0)]	0.863	-0.212567535	0.962617576
Q13177	1xPhospho [S19(99.4); S20(0.3); T21(0.3); S24(0); T25(0); S32(0); S36(0); S42(0)]	1.051	0.071762669	0.967238018
Q13177	2xPhospho [S19(100); S20(97.6); T21(2.5); S24(0); T25(0)]	0.75	-0.415037499	0.971937853
Q13177	2xPhospho [S55(100); S58(100); T60(0)]	0.929	-0.106249498	0.99184412
Q13177	2xPhospho [Y139(0); S141(100); T143(0); S152(99.9); T154(0.1)]	0.95	-0.074000581	1
Q13185	1xPhospho [T173(1.2); S176(98.8)]	1.342	0.424384672	0.814722979
Q13188	1xPhospho [S406(100); S421(0)]	4.004	2.001441974	0.034076329
Q13188	1xPhospho [T384(0.2); S385(99.8); S392(0); Y396(0)]	1.776	0.828631582	0.563451327
Q13200	1xPhospho [S16(100); T24(0); S29(0)]	2.387	1.255198566	0.099054707
Q13200	1xPhospho [S16(100); T24(0); S29(0)]	2.357	1.236951759	0.100107117
Q13206	1xPhospho [S602(100); S606(0); T610(0); S611(0)]	1.182	0.241230036	0.930376591
Q13206	1xPhospho [S829(0); S831(100); T839(0); S840(0)]	1.156	0.209141398	0.930641583
Q13206	1xPhospho [S829(1.4); S831(98.6); T839(0); S840(0)]	1.025	0.03562391	0.971973958
Q13242	1xPhospho [S211(100); Y214(0); S216(0); Y221(0)]	0.902	-0.148800661	0.968531169
Q13242	2xPhospho [S211(100); Y214(0); S216(100); Y221(0)]	0.94	-0.089267338	0.97998947
Q13247	1xPhospho [S301(0); S303(100); S310(0)]	1.455	0.541019153	0.79861982
Q13247	3xPhospho [S295(0.4); S297(4.5); S299(99.6); S301(95.5); S303(100); S310(0)]	1.079	0.109694865	0.965139891
Q13263	1xPhospho [S473(100); S479(0)]	2.848	1.509949146	0.098223126
Q13263	2xPhospho [S33(0); T34(0); S37(0); S41(4.8); S43(0.3); S45(94.8); S49(5); S50(95.2)]	2.6	1.378511623	0.111787157
Q13263	1xPhospho [S471(0); S473(100); S479(0)]	2.516	1.331131922	0.12555006
Q13263	1xPhospho [S437(3.5); S439(93.1); S440(3.5); Y449(0); S453(0); Y458(0); S459(0); S460(0); S466(0)]	1.649	0.721591399	0.550634186
Q13263	2xPhospho [S806(93.1); S816(6.8); S823(87.3); S824(6.4); S828(6.4)]	0.632	-0.662003536	0.64957454
Q13263	1xPhospho [S33(0); T34(0); S37(0); S41(0); S43(0); S45(0); S49(4); S50(96)]	1.619	0.695102986	0.653831121
Q13263	1xPhospho [T498(0); S501(100)]	0.421	-1.248107862	0.686714309
Q13263	1xPhospho [T415(2.2); S417(2.2); T418(95.7)]	1.658	0.729444007	0.694333278
Q13263	3xPhospho [S594(96.5); S596(44.5); S598(42.9); T599(30.3); S600(42.9); S601(42.9); T611(0); S612(0); T620(0); S624(0); T626(0)]	1.471	0.556797247	0.774421636
Q13263	1xPhospho [S806(0); S816(0); S823(4.8); S824(90.4); S828(4.8)]	0.838	-0.254977851	0.93651142
Q13263	1xMet-loss+Acetyl [N-Term]; 1xPhospho [S4(0); S9(0); S14(0); S17(0); S19(100); S26(0)]	1.169	0.22527493	0.950730609
Q13263	2xPhospho [S594(0.3); S596(0.3); S598(95.7); T599(95.4); S600(7.7); S601(0.5); T611(0); S612(0); T620(0); S624(0); T626(0)]	0.929	-0.106249498	0.980895643
Q13283	2xPhospho [S280(100); S286(0); S290(100)]	1.77	0.82374936	0.519253406
Q13283	1xPhospho [S280(0); S286(0); S290(100)]	1.304	0.38294387	0.848293321
Q13283	1xPhospho [Y133(0); T143(0); S149(100)]	0.88	-0.184424571	0.962617576
Q13283	1xPhospho [T248(0); S250(0); S253(98.9); T255(1.1); S256(0)]	0.947	-0.078563669	0.989102507
Q13283	1xPhospho [S230(0); S231(0); S232(100); T241(0)]	1.028	0.039840265	0.99277609
Q13315	1xPhospho [S2996(100); S3001(0)]	0.01	-6.64385619	8.35E-17
Q13322	3xPhospho [T132(33.3); S133(33.3); S134(33.3); S150(100); T155(0); S158(100); S162(0)]	0.01	-6.64385619	8.35E-17
Q13356	1xPhospho [S463(0); S464(0); S470(97.5); T475(2.5)]	0.931	-0.103146927	0.996295697
Q13371	2xPhospho [S288(0); T290(2); S293(98); S296(100)]	1.115	0.15704371	0.958702638

Q13425	1xPhospho [S110(100)]	0.01	-6.64385619	8.35E-17
Q13425	2xPhospho [S208(0); S211(0); S222(100); S224(0); S226(0); S228(0); S231(0); S233(100)]	0.592	-0.756330919	0.731924668
Q13425	1xPhospho [S110(100)]	1.326	0.407080775	0.883529589
Q13425	1xPhospho [S208(0); S211(0); S222(0); S224(0); S226(0); S228(0.1); S231(2.7); S233(97.3)]	0.58	-0.785875195	0.891020458
Q13425	1xPhospho [S393(98.4); S395(1.6); S398(0); T401(0); T404(0)]	0.705	-0.504304837	0.93019184
Q13425	1xPhospho [S95(100)]	0.732	-0.450084446	0.93019184
Q13426	1xPhospho [S256(100); S259(0); S260(0); T264(0); S269(0)]	100	6.64385619	8.35E-17
Q13426	2xPhospho [T215(0); S219(6.5); T222(93.4); Y229(0.5); S232(93); T233(6.5); S237(0); T241(0); S244(0); S248(0); S252(0)]	100	6.64385619	8.35E-17
Q13426	1xPhospho [S299(0); S303(98.3); S304(1.7); T308(0); S309(0)]	8.563	3.098116325	0.000106884
Q13426	2xPhospho [S315(0); S320(0); T323(0); S327(100); S328(100)]	0.56	-0.836501268	0.660888692
Q13426	2xPhospho [S315(0); S320(0); T323(0); S327(100); S328(100)]	1.106	0.145351386	0.971937853
Q13426	2xPhospho [T215(0); S219(0); T222(0); Y229(0); S232(100); T233(100); S237(0); T241(0); S244(0); S248(0); S252(0)]	0.91	-0.13606155	1
Q13428	3 2xPhospho [T1180(0.4); T1182(5.8); T1187(89); S1191(94.1); S1192(10.7); S1200(0); S1202(0); S1205(0); Y1207(0); T1209(0); T1213(0); S1217(0); S1220(0)]; E7ETY2 2xPhospho [T1179(0.4); T1181(5.8); T1186(89); S1190(94.1); S1191(10.7); S1199(0); S1201(0); S1204(0); Y1206(0); T1208(0); T1212(0); S1216(0); S1219(0)]	0.363	-1.461958547	0.213816602
Q13428	3 2xPhospho [S571(0); T581(98.5); S582(50.7); S583(50.7)]; E7ETY2 2xPhospho [S571(0); T581(98.5); S582(50.7); S583(50.7)]	0.482	-1.052894948	0.489974409
Q13428	3 1xPhospho [S1377(0); S1379(100)]; E7ETY2 1xPhospho [S1376(0); S1378(100)]	1.517	0.601221086	0.744427507
Q13428	3 1xPhospho [S503(100); S510(0); T511(0)]; E7ETY2 1xPhospho [S503(100); S510(0); T511(0)]	1.36	0.443606651	0.80163956
Q13428	3 2xPhospho [T1094(0); S1097(0.3); S1098(0.3); S1102(99.5); T1106(0); T1110(0); S1111(99.7); S1114(0.3); T1115(0); S1116(0)]	0.643	-0.637109357	0.860444802
Q13428	3 1xPhospho [S1258(100)]; E7ETY2 1xPhospho [S1257(100)]	1.321	0.401630467	0.872729954
Q13428	3 2xPhospho [S1377(100); S1379(100)]; E7ETY2 2xPhospho [S1376(100); S1378(100)]	1.276	0.351628329	0.884786699
Q13428	3 1xPhospho [S1258(100)]; E7ETY2 1xPhospho [S1257(100)]	1.252	0.324234562	0.908086456
Q13428	3 1xPhospho [S1228(0); S1229(0); S1231(100); S1233(0); S1234(0); T1235(0)]; E7ETY2 1xPhospho [S1227(0); S1228(0); S1230(100); S1232(0); S1233(0); T1234(0)]	1.141	0.190298792	0.957850451
Q13428	3 1xPhospho [S107(0.1); T108(3.4); S110(96.3); S111(0.1); S119(0); S120(0)]; E7ETY2 1xPhospho [S107(0.1); T108(3.4); S110(96.3); S111(0.1); S119(0); S120(0)]	1.141	0.190298792	0.962617576
Q13428	3 1xPhospho [S1168(100); T1171(0); T1176(0); S1178(0)]; E7ETY2 1xPhospho [S1167(100); T1170(0); T1175(0); S1177(0)]	1.074	0.102993993	0.971220368
Q13428	3 1xPhospho [S1377(0); S1379(100)]; E7ETY2 1xPhospho [S1376(0); S1378(100)]	0.973	-0.03948829	0.972363997
Q13428	3 1xPhospho [T147(0); S153(0); S156(100)]; E7ETY2 1xPhospho [T147(0); S153(0); S156(100)]	0.901	-0.150400989	0.974924677
Q13428	3 3xPhospho [T1180(0); T1182(0); T1187(5.8); S1191(94.5); S1192(99.7); S1200(0); S1202(0); S1205(0); Y1207(0); T1209(100); T1213(0); S1217(0); S1220(0)]; E7ETY2 3xPhospho [T1179(0); T1181(0); T1186(5.8); S1190(94.5); S1191(99.7); S1199(0); S1201(0); S1204(0); Y1206(0); T1208(100); T1212(0); S1216(0); S1219(0)]	0.832	-0.265344567	0.980895643
Q13428	3 2xPhospho [S571(0); T581(100); S582(0); S583(100)]; E7ETY2 2xPhospho [S571(0); T581(100); S582(0); S583(100)]	0.928	-0.10780329	0.990299313
Q13428	3 1xPhospho [S571(0); T581(0); S582(2.1); S583(97.9)]; E7ETY2 1xPhospho [S571(0); T581(0); S582(2.1); S583(97.9)]	0.865	-0.209227962	0.99184412
Q13428	3 1xPhospho [S571(0); T581(1.5); S582(1.5); S583(97)]; E7ETY2 1xPhospho [S571(0); T581(1.5); S582(1.5); S583(97)]	0.988	-0.017417053	1
Q13442	1xPhospho [S176(100); S178(0)]	100	6.64385619	8.35E-17
Q13442	1xPhospho [S176(100); S178(0)]	4.809	2.265736926	0.01379888
Q13442	2xPhospho [S57(0); S60(100); S63(100); Y70(0)]	1.465	0.550900665	0.788815962
Q13442	1xPhospho [S176(100); S178(0)]	0.597	-0.744197163	0.815248216
Q13442	2xPhospho [S57(0); S60(100); S63(100); Y70(0)]	1.169	0.22527493	0.951285684
Q13443	3xPhospho [S758(100); T761(100); Y769(100)]	1.585	0.66448284	0.680828739
Q13443	2xPhospho [S758(0); T761(100); Y769(100)]	1.525	0.608809243	0.783976635
Q13451	1xPhospho [T443(0); S445(100)]	0.678	-0.560642822	0.923894189
Q13451	1xPhospho [S13(100); T15(0); T17(0); T26(0); S27(0)]	0.866	-0.20756107	0.986075329
Q13459	1xPhospho [S1290(100); S1294(0); T1295(0)]	1.504	0.588804567	0.723988927
Q13459	2xPhospho [S1109(0); S1114(2.2); S1115(97.8); S1122(100)]	1.526	0.609754962	0.738084678
Q13459	1xPhospho [S716(0); S717(100); S722(0)]	1.363	0.446785562	0.863653123
Q13459	1xPhospho [S1397(0); S1398(0); S1405(2.4); S1408(97.6); S1412(0.1)]	1.139	0.187767747	0.948093638
Q13459	2xPhospho [S1261(0); S1267(100); T1271(100)]	0.944	-0.083141235	1
Q13464	3xPhospho [S1098(0); S1100(0); T1101(3.7); S1102(96.3); S1105(100); S1108(100); T1112(0); S1119(0)]	0.026	-5.265344567	6.37E-09
Q13464	2xPhospho [S1098(0); S1100(0.2); T1101(4.3); S1102(95.5); S1105(100); S1108(0); T1112(0); S1119(0)]	0.357	-1.486004021	0.144851642
Q13469	2xPhospho [S755(51.1); S757(51.1); S759(92.8); S761(4.9); Y765(0); S776(0)]	0.333	-1.586405918	0.363369345
Q13470	2xPhospho [S502(100); S505(0); S508(0); T514(1.3); S518(49.4); S519(49.4)]	0.674	-0.569179503	0.714293599
Q13501	3xPhospho [S282(0); S283(0.4); S284(99.6); S287(0.4); S288(99.6); S291(100); S294(0.1); T304(0); S306(0)]	100	6.64385619	8.35E-17
Q13501	3xPhospho [S318(0); S328(100); S332(100); T339(0); S342(4.7); S343(95.3)]	100	6.64385619	8.35E-17
Q13501	2xPhospho [S349(0); T350(0); S355(100); S361(0); S365(0); S366(100); S370(0); T375(0)]	3.687	1.882447416	0.007545124
Q13501	1xPhospho [S318(0); S328(0); S332(99.7); T339(0.3); S342(0); S343(0)]	1.769	0.822934048	0.573362551
Q13501	2xPhospho [S282(0.3); S283(0.3); S284(99.2); S287(50.1); S288(50.1); S291(0); S294(0); T304(0); S306(0)]	1.421	0.506906555	0.776177942
Q13501	2xPhospho [S282(0.3); S283(0.3); S284(99.3); S287(5); S288(90); S291(5); S294(0); T304(0); S306(0)]	1.421	0.506906555	0.806224461

Q13501	1xPhospho [S282(4.1); S283(4.1); S284(91.6); S287(0.2); S288(0); S291(0); S294(0); T304(0); S306(0)]	0.722	-0.469929258	0.855557911
Q13501	2xPhospho [S318(0); S328(0); S332(100); T339(0); S342(99.8); S343(0.2)]	1.312	0.39176772	0.863668848
Q13501	1xPhospho [S318(0); S328(0); S332(100); T339(0); S342(0); S343(0)]	1.282	0.358396262	0.879690128
Q13501	2xPhospho [S318(0); S328(100); S332(100); T339(0); S342(0); S343(0)]	1.232	0.301002256	0.884551994
Q13501	2xPhospho [S266(0); T269(100); S272(100); S275(0); S276(0); S277(0); T278(0)]	0.857	-0.222632891	0.944443377
Q13501	1xPhospho [S266(1.7); T269(98.3); S272(0); S275(0); S276(0); S277(0); T278(0)]	1.143	0.192825404	0.956841977
Q13501	2xPhospho [S266(0); T269(100); S272(100); S275(0); S276(0); S277(0); T278(0)]	0.881	-0.182786076	0.969482098
Q13501	1xPhospho [S349(0); T350(0); S355(0); S361(0); S365(5.3); S366(94.7); S370(0); T375(0)]	0.892	-0.164884385	0.990182098
Q13523	3xPhospho [S20(100); S23(100); S32(100); S36(0)]	6.67	2.737686761	0.000623682
Q13523	2xPhospho [S366(100); S368(100)]	3.117	1.640158155	0.217894234
Q13523	3xPhospho [S518(100); S519(100); S520(100)]	0.405	-1.304006187	0.413053723
Q13523	2xPhospho [S20(100); S23(100); S32(0); S36(0)]	0.325	-1.621488377	0.466291605
Q13523	2xPhospho [S20(100); S23(100); S32(0); S36(0)]	1.406	0.491596594	0.780165563
Q13523	2xPhospho [S366(100); S368(100)]	1.444	0.530070742	0.810156821
Q13523	1xPhospho [S87(0); S93(100)]	1.526	0.609754962	0.822854327
Q13523	2xPhospho [S87(100); S93(100)]	1.213	0.27857955	0.901860353
Q13523	2xPhospho [S87(100); S93(100)]	0.672	-0.573466862	0.921964784
Q13523	2xPhospho [S427(0); S431(100); S437(99.9); T439(0.1)]	0.725	-0.4639471	0.930641583
Q13523	2xPhospho [S431(100); S437(100); T439(0)]	1.182	0.241230036	0.934208701
Q13523	2xPhospho [S87(100); S93(100)]	1.171	0.227741076	0.956281413
Q13523	2xPhospho [S131(0); Y140(0); S142(100); S144(100)]	0.89	-0.168122759	0.965013037
Q13523	2xPhospho [T576(0); S578(100); S580(100)]	1.073	0.101650076	0.971196575
Q13523	3xPhospho [S427(100); S431(100); S437(2); T439(98)]	1.086	0.119024103	0.973763863
Q13523	2xPhospho [S131(0); Y140(0); S142(100); S144(100)]	0.904	-0.145605322	0.980895643
Q13523	1xPhospho [S837(0); S839(0); T847(2); Y849(95.9); S852(2)]	1.006	0.008630305	0.99277609
Q13541	3xPhospho [S65(99.5); T68(6.9); T70(93.1); T77(0.5); T82(0); S83(0); S85(93.5); S86(6.5); S94(0); S96(0)]	0.01	-6.64385619	8.35E-17
Q13541	2xPhospho [S65(99.9); T68(50.1); T70(50.1); T77(0); T82(0); S83(0); S85(0); S86(0); S94(0); S96(0)]	0.47	-1.089267338	0.177745404
Q13541	2xPhospho [T70(0.2); T77(99.8); T82(0); S83(0); S85(99.8); S86(0.2); S94(0); S96(0)]	0.587	-0.768567592	0.713954373
Q13541	2xPhospho [T77(0); T82(4.8); S83(4.8); S85(4.8); S86(85.7); S94(5.3); S96(94.7)]	0.542	-0.883635243	0.750433149
Q13541	2xPhospho [Y34(0); S35(0); T36(0); T37(100); T41(0); S44(0); T45(0.2); T46(99.8); T50(0)]	0.699	-0.516635639	0.751204811
Q13541	2xPhospho [T70(100); T77(0); T82(0); S83(0); S85(0); S86(0); S94(5.4); S96(5.4); S101(89.2)]	0.701	-0.512513651	0.808799965
Q13541	2xPhospho [Y34(0); S35(0); T36(0.3); T37(99.7); T41(0); S44(0.3); T45(5.3); T46(94.4); T50(0)]	0.76	-0.395928676	0.854346269
Q13541	1xPhospho [T70(100); T77(0); T82(0); S83(0); S85(0); S86(0); S94(0); S96(0)]	0.682	-0.552156356	0.879063032
Q13541	2xPhospho [T77(0); T82(7.5); S83(7.5); S85(77.6); S86(7.5); S94(4.6); S96(47.7); S101(47.7)]	0.615	-0.701341684	0.891862526
Q13541	3xPhospho [S65(99.8); T68(50.1); T70(50.1); T77(0); T82(0); S83(0); S85(0); S86(100); S94(0); S96(0)]	0.753	-0.40927823	0.925724009
Q13541	2xPhospho [T77(0); T82(0); S83(0); S85(0); S86(0); S94(50.1); S96(50.1); S101(99.8)]	0.761	-0.394031641	0.933760225
Q13541	1xPhospho [T77(0); T82(0); S83(0); S85(0); S86(0); S94(0); S96(0); S101(100)]	0.988	-0.017417053	1
Q13542	2xPhospho [S65(100); T70(100); T82(0); S83(0); T86(0); S91(0)]	0.293	-1.77102743	0.045481574
Q13542	2xPhospho [S65(100); T70(100); T82(0); S83(0); T86(0); S91(0)]	0.499	-1.002888279	0.244442028
Q13542	2xPhospho [S65(0); T70(100); T82(0.3); S83(5.5); T86(94.2); S91(0)]	0.439	-1.187707155	0.334898212
Q13542	3xPhospho [S65(100); T70(100); T82(0.5); S83(99.6); T86(0); S91(0)]	0.633	-0.659722595	0.879063032
Q13542	2xPhospho [T21(0); S25(0); Y34(0); T36(0.2); T37(99.8); T41(0); S44(0.2); T45(4.6); T46(95.2); T50(0)]	0.848	-0.23786383	0.938673385
Q13542	1xPhospho [S65(0); T70(100); T82(0); S83(0); T86(0); S91(0)]	1.102	0.140124224	0.972363997
Q13542	1xPhospho [S65(96.6); T70(3.4); T82(0); S83(0); T86(0); S91(0)]	0.97	-0.043943348	0.984634599
Q13555	2xPhospho [S349(100); S352(0); T362(0); T368(3.8); T369(96.2); T375(0)]	2.325	1.217230716	0.206709629
Q13555	9 2xPhospho [S338(0.4); T343(99.6); T344(99.6); T350(0.4)]	1.009	0.012926174	0.971838536
Q13555	9 2xPhospho [S315(100); S319(100)]; Q13555 2xPhospho [S315(100); S319(100)]; Q13555-7 2xPhospho [S315(100); S319(100)]	0.849	-0.236163541	1
Q13557	2xPhospho [S315(100); S319(100)]	0.439	-1.187707155	0.651510528
Q13557	2xPhospho [S330(100); T331(0); S333(0); S334(0); T336(0); T337(100)]	0.637	-0.650634722	0.737526822
Q13557	1xPhospho [S330(100); T331(0); S333(0); S334(0); T336(0); T337(0)]	1.186	0.24610401	0.933452347
Q13561	1xPhospho [T79(0); Y81(0); S83(100); Y86(0)]	0.409	-1.289827252	0.564692999
Q13595	2xPhospho [T202(100); T204(0.1); Y208(0); T213(6.9); S215(93)]	3.048	1.607862903	0.209541255
Q13595	1xPhospho [T202(100); T204(0); Y208(0); T213(0); S215(0)]	2.177	1.122341408	0.263493654
Q13595	1xMet-loss+Acetyl [N-Term]; 1xPhospho [S2(100)]	0.901	-0.150400989	0.981858798
Q13595	2xPhospho [T202(100); T204(0.1); Y208(89.4); T213(5.3); S215(5.3)]	0.966	-0.049904906	0.99811985
Q13601	1xMet-loss+Acetyl [N-Term]; 1xPhospho [S3(100); S5(0)]	1.204	0.267835392	0.94813191
Q13610	1xPhospho [S484(0); S485(100); S487(0); S492(0)]	100	6.64385619	8.35E-17
Q13610	2xPhospho [S50(100); T55(0); S57(50); S59(50); S65(0)]	3.491	1.803640357	0.035113343
Q13610	1xPhospho [S50(100); T55(0); S57(0); S59(0); S65(0)]	2.778	1.474046599	0.16823951
Q13610	1xPhospho [S50(100); T55(0); S57(0); S59(0); S65(0)]	1.22	0.286881148	0.886312012
Q13614	2xPhospho [S4(99.5); S5(93.4); S6(6.6); S9(0.5); S12(0); S21(0); S24(0); S26(0); S27(0); S29(0); T30(0); S31(0); S33(0); S36(0); T39(0)]	1.457	0.543000877	0.819921743
Q13620	1 2xPhospho [S43(0); S44(0); S45(0); S46(0); S47(0); S48(0.2); S49(2.7); S51(48.6); S52(48.6); S60(0); T61(0); S62(0); S63(0); S64(0); S65(0.3); S66(5.2); T67(94.4)]	0.805	-0.312939312	0.953252794
Q13620	1 1xPhospho [S43(0); S44(0); S45(0); S46(0); S47(0); S48(0); S49(0); S51(0); S52(0); S60(0); T61(0); S62(3.8); S63(92.3); S64(3.8); S65(0.2); S66(0); T67(0)]	0.883	-0.179514657	0.99184412
Q13625	1xPhospho [S698(100); T700(0)]	1.342	0.424384672	0.862297675
Q13625	1xPhospho [S480(100); S481(0)]	1.253	0.325386415	0.886312012
Q13625	1xPhospho [S736(98.4); S737(1.6); T739(0)]	0.993	-0.010134377	0.980895643

Q13671	1xPhospho [S349(0); S351(100); S356(0)]	0.491	-1.02620507	0.630417345
Q13769	2xPhospho [S307(0); S312(100); S314(100); T321(0); T322(0)]	0.531	-0.913216234	0.401700359
Q13769	2xPhospho [S307(0); S312(100); S314(100); T321(0); T322(0)]	0.693	-0.529072743	0.93105732
Q13796	1xPhospho [S193(99.9); S195(0.1); S196(0); S202(0); S204(0)]	3.71	1.891419187	0.108935291
Q13796	2xPhospho [T1163(0); T1165(0); T1168(0.1); S1169(3.3); S1171(96.6); S1173(100)]	1.214	0.279768422	0.907438338
Q13796	2xPhospho [S1425(100); S1427(100); S1434(0)]	0.675	-0.567040593	0.943796521
Q13895	2xPhospho [T160(0); T164(0); S167(100); S170(100)]	0.637	-0.650634722	0.878612438
Q13895	2xPhospho [T160(0); T164(95.9); S167(4.1); S170(100)]	0.588	-0.76611194	0.907438338
Q13895	1xPhospho [S98(100); T107(0)]	1.209	0.273814245	0.910211107
Q13895	1xPhospho [S98(100); T107(0)]	1.09	0.124328135	0.963131675
Q14004	1xPhospho [Y862(0); S863(0); S864(0); S867(0); Y870(0); T871(100)]	1.999	0.999278472	0.412640022
Q14004	2xPhospho [S1048(0); S1054(50); T1056(50); T1058(100); S1065(0); S1066(0)]	1.684	0.751892138	0.705680833
Q14004	3xPhospho [S395(100); S397(100); Y399(0); S400(100)]	1.371	0.455228571	0.830379242
Q14004	2xPhospho [S395(50.6); S397(50.6); Y399(0); S400(98.7)]	1.072	0.100304906	0.962617576
Q14004	3xPhospho [S315(100); S317(100); S325(100); S328(0)]	1.073	0.101650076	0.966549726
Q14004	2xPhospho [S315(100); S317(100)]	1.082	0.113700499	0.967238018
Q14004	2xPhospho [S436(0); S437(100); S439(100); S441(0); T442(0); T444(0)]	1.035	0.049630768	0.980895643
Q14008	2xPhospho [S828(2.5); T829(50); S830(50); T832(97.5); S845(0)]	0.44	-1.184424571	0.436078107
Q14008	2xPhospho [S1995(0); S1999(0.1); T2002(25); S2004(25); S2005(25); T2007(25); T2009(0); S2010(0); S2011(0); S2012(5.5); S2013(94.5); T2014(0)]	1.737	0.796597754	0.647794814
Q14008	1xPhospho [S1995(0); S1999(0); T2002(0); S2004(0); S2005(0); T2007(0); T2009(0); S2010(0); S2011(0); S2012(4.3); S2013(95.5); T2014(0.2)]	0.807	-0.309359421	0.941024499
Q14008	1xPhospho [S828(2.4); T829(2.4); S830(2.4); T832(92.7); S845(0)]	0.881	-0.182786076	0.994773029
Q14011	2xPhospho [S155(0.2); S156(0.2); S159(99.7); Y160(0); S163(100); Y164(0); S166(0); Y167(0); T169(0)]	1.706	0.770617647	0.642636279
Q14011	1xPhospho [S155(0); S156(0); S159(0); Y160(0); S163(100); Y164(0); S166(0); Y167(0); T169(0)]	1.206	0.270229907	0.904079048
Q14106	2xPhospho [S232(1.8); S234(1.8); S236(48.2); S239(48.2); T244(0); S251(0.1); S254(99.9)]	1.21	0.275007047	0.930939466
Q14126	1xPhospho [S671(0); S680(100)]	0.816	-0.293358943	0.94797789
Q14141	1xPhospho [T401(0); S408(0); S411(100); S416(0); T418(0)]	0.945	-0.081613766	0.991003044
Q14147	2xPhospho [S749(100); S750(100); S763(0)]	8.116	3.020768865	4.14E-06
Q14152	1xPhospho [S1028(100)]	4.67	2.22342255	0.012483044
Q14152	1xPhospho [S1256(0); S1257(0); S1258(0.1); S1262(99.9); S1263(0.1)]	1.632	0.706641057	0.75736259
Q14152	1xPhospho [S949(100)]	0.746	-0.422752464	0.962617576
Q14152	1xPhospho [S584(100)]	0.732	-0.450084446	0.965013037
Q14153	1xPhospho [S165(0); S166(0); S167(98.9); S169(1.1); S172(0)]	1.126	0.171206827	0.94797789
Q14157	1 2xPhospho [Y602(0); S604(2.5); S605(97.5); S607(95.1); S608(2.4); S609(2.4)]	4.151	2.053458932	0.004056653
Q14157	1 1xPhospho [Y602(0); S604(0); S605(0); S607(0); S608(0); S609(100)]	1.794	0.84317989	0.41370633
Q14157	1 1xPhospho [S410(0); S412(0); S416(100); T419(0)]	1.943	0.958285901	0.549414358
Q14157	1 1xPhospho [S356(100); T357(0); T358(0); S360(0)]	1.417	0.502839758	0.837628743
Q14157	1 2xPhospho [Y602(0); S604(0.3); S605(99.4); S607(0.6); S608(5); S609(94.7); T615(0)]	0.561	-0.833927324	0.855816069
Q14157	1 1xPhospho [S622(0); S623(0); T627(0); T631(0); T632(3.2); S634(96.8); T639(0); S641(0)]	1.28	0.35614381	0.886312012
Q14157	1 1xPhospho [S439(0); T444(0); S445(0); T446(0); S453(1.6); S454(96.8); S458(1.6)]	1.228	0.296310561	0.925724009
Q14157	1 1xPhospho [S460(3.3); T461(3.3); S462(3.3); S467(83.7); S470(3.3); S471(3.3); S475(0); S476(0); S477(0)]	0.653	-0.614845103	0.93019184
Q14157	1 2xPhospho [S439(0); T444(33.3); S445(33.3); T446(33.3); S453(2.6); S454(2.6); S458(94.8)]	1.082	0.113700499	0.969482098
Q14161	2xPhospho [S415(0.1); S418(99.9); S421(100); T426(0)]	0.01	-6.64385619	8.35E-17
Q14161	3xPhospho [S415(100); S418(100); S421(100); T426(0)]	0.01	-6.64385619	8.35E-17
Q14161	2xPhospho [T375(0); S381(0); S384(0); Y392(0); S394(100); S397(100); T401(0); T405(0); T406(0); S408(0)]	0.341	-1.552156356	0.082074952
Q14166	1xPhospho [S15(0.1); S16(99.9); T20(0)]	0.86	-0.217591435	0.94797789
Q14185	1xPhospho [S1681(100)]	1.145	0.195347598	0.969482098
Q14191	2xPhospho [S426(100); T434(0); S435(0); Y436(0); S440(100)]; 2xPhospho [S453(100); T461(0); S462(0); Y463(0); S467(100)]	0.673	-0.57132159	0.86443084
Q14191	2xPhospho [S421(0.1); T422(0.1); S426(99.7); T434(0); S435(0); Y436(0); S440(100)]	0.765	-0.386468347	0.952591019
Q14202	2xPhospho [S256(0); T257(0); S259(0); S263(100); S267(100)]	1.71	0.773996325	0.50378641
Q14202	2xPhospho [S256(0); T257(0); S259(0); S263(100); S267(100)]	1.621	0.696884091	0.676719385
Q14202	2xPhospho [S256(0); T257(0); S259(0); S263(100); S267(100)]	0.816	-0.293358943	0.965013037
Q14202	2xPhospho [S256(7.1); T257(7.1); S259(0.6); S263(85.3); S267(99.9)]	1.109	0.149259365	0.965139891
Q14204	1xPhospho [S1230(100)]	0.721	-0.471928835	0.963156927
Q14204	1xPhospho [T4366(0.1); S4368(97.1); T4369(2.8); S4370(0)]	0.782	-0.354759487	0.965351295
Q14247	2 3xPhospho [T362(5.9); T364(94.1); S368(100); T374(0.4); S380(93.8); S381(5.8); Y384(0); S389(0)]; Q14247 3xPhospho [T399(5.9); T401(94.1); S405(100); T411(0.4); S417(93.8); S418(5.8); Y421(0); S426(0)]	0.01	-6.64385619	8.35E-17
Q14247	2 2xPhospho [T43(100); S47(100)]; Q14247 2xPhospho [T43(100); S47(100)]	100	6.64385619	8.35E-17
Q14247	2 1xPhospho [T43(100); S47(0)]; Q14247 1xPhospho [T43(100); S47(0)]	6.978	2.802813598	0.005177865
Q14247	2 2xPhospho [S380(0); S381(100); Y384(0); S389(100)]; Q14247 2xPhospho [S417(0); S418(100); Y421(0); S426(100)]	0.481	-1.055891201	0.200646924
Q14247	2 1xPhospho [S380(0); S381(0); Y384(0); S389(100)]; Q14247 1xPhospho [S417(0); S418(0); Y421(0); S426(100)]	0.531	-0.913216234	0.38667379
Q14247	2 2xPhospho [T362(0); T364(100); S368(100); T374(0)]; Q14247 2xPhospho [T399(0); T401(100); S405(100); T411(0)]	0.686	-0.543719518	0.727550885
Q14247	2 2xPhospho [T362(0); T364(100); S368(100); T374(0)]; Q14247 2xPhospho [T399(0); T401(100); S405(100); T411(0)]	0.731	-0.452056689	0.804512505

Q14247	2 1xPhospho [S285(0); T286(0); T291(0); S294(0); S295(100); Y297(0)]; Q14247 1xPhospho [S322(0); T323(0); T328(0); S331(0); S332(100); Y334(0)]	0.687	-0.541617996	0.86936135
Q14247	2 1xPhospho [S5(0.1); S11(100); T24(0); S33(0)]; Q14247 1xPhospho [S5(0.1); S11(100); T24(0); S33(0)]	1.29	0.367371066	0.920518605
Q14247	2 1xPhospho [S261(100); Y265(0); S266(0.1)]	0.704	-0.506352666	0.962926915
Q14247	2 1xPhospho [T362(0); T364(100); S368(0); T374(0)]; Q14247 1xPhospho [T399(0); T401(100); S405(0); T411(0)]	1.073	0.101650076	0.976345951
Q14247	1xPhospho [S261(100); Y265(0)]	1	0	0.981858798
Q14258	1xPhospho [S97(0); S100(100)]	1.25	0.321928095	0.925808485
Q14331	1xPhospho [S215(100)]	1.704	0.768925336	0.749797187
Q14444	2xPhospho [S14(94.7); S15(5.3); S21(49.8); S23(49.8); S24(0.2); S26(0.2); S39(0); T44(0); T46(0); T51(0)]	0.488	-1.035046947	0.456984613
Q14444	1xPhospho [S14(0); S15(0); S21(0); S23(0); S24(0); S26(0); S39(99.7); T44(0.2); T46(0.2); T51(0)]	0.699	-0.516635639	0.767755861
Q14517	1xPhospho [S4353(100); Y4356(0); S4357(0)]	0.01	-6.64385619	8.35E-17
Q14517	2xPhospho [S4353(100); Y4356(0); S4357(100)]	0.178	-2.490050854	0.027410754
Q14517	1xPhospho [S4272(100); S4276(0); S4285(0); T4286(0); S4291(0)]	0.184	-2.442222329	0.033513483
Q14568	1xPhospho [S263(100)]	1.947	0.961252884	0.509800099
Q14568	1xPhospho [S263(100)]	1.611	0.687956494	0.719382659
Q14573	1xPhospho [S1832(100); S1834(0); S1838(0); S1839(0); S1840(0)]	0.878	-0.187707155	1
Q14669	3 1xPhospho [S76(4.2); S78(4.2); S79(4.2); S80(83.1); S81(4.2)]	0.01	-6.64385619	8.35E-17
Q14669	3 2xPhospho [S1415(0); S1424(100); T1425(100); S1429(0)]	0.42	-1.251538767	0.303183907
Q14669	3 1xPhospho [Y1063(0); S1064(100)]	1.459	0.544979883	0.776177942
Q14669	3 1xPhospho [S1035(100); S1039(0)]	1.316	0.396159489	0.87431027
Q14669	3 2xPhospho [S352(0); S354(100); S361(100)]	0.617	-0.696657606	0.908086456
Q14669	3 2xPhospho [S1365(100); S1370(100); S1377(0); S1385(0)]	0.871	-0.199255376	0.956841977
Q14669	3 1xPhospho [S352(0); S354(100); S361(0)]	1.037	0.052415894	0.978877443
Q14669	3 2xPhospho [S352(100); S354(100); S361(0)]	1.051	0.071762669	0.979586453
Q14676	2xPhospho [S495(100); S498(100); S504(0); S505(0)]	0.01	-6.64385619	8.35E-17
Q14676	3xPhospho [S485(0.1); S495(100); S498(100); S504(93.3); S505(6.7)]	100	6.64385619	8.35E-17
Q14676	1xPhospho [S963(0); S964(0); T966(0); T980(0); S981(0); S986(0.1); S988(96.9); S990(3.1)]	100	6.64385619	8.35E-17
Q14676	3xPhospho [S445(0.2); T447(95.9); T448(4); T449(7.6); S453(92.4); T455(100)]	0.393	-1.347398782	0.363369345
Q14676	2xPhospho [S372(0); S376(100); T378(100)]	0.589	-0.763660461	0.703538855
Q14676	1xPhospho [S793(100); T797(0)]	1.482	0.567545448	0.746362255
Q14676	2xPhospho [S292(0); S299(100); T301(3.9); S307(96.1)]	1.367	0.451013243	0.819921743
Q14676	2xPhospho [S329(100); T331(99.7); T341(0.3)]	1.441	0.527070336	0.831039794
Q14676	2xPhospho [S485(100); S495(0); S498(0); S504(50); S505(50)]	1.448	0.534061602	0.836380628
Q14676	1xPhospho [S168(100); S176(0)]	1.316	0.396159489	0.853500932
Q14676	2xPhospho [T1280(0); T1287(91); S1294(4.5); T1295(4.5); S1296(0); T1297(0); T1302(99.9); T1306(0); S1307(0)]	1.339	0.421155961	0.869365103
Q14676	2xPhospho [S372(0); S376(100); T378(100)]	0.775	-0.367731785	0.870654365
Q14676	2xPhospho [S329(100); T331(100)]	0.716	-0.481968507	0.879063032
Q14676	1xPhospho [S372(0); S376(97.9); T378(2.1)]	1.309	0.388465097	0.884551994
Q14676	2xPhospho [S963(0); S964(0); T966(0); T980(0); S981(0); S986(100); S988(95.9); S990(4.1); S995(0); S998(0)]	0.686	-0.543719518	0.898100811
Q14676	2xPhospho [S1563(92.4); S1564(3.7); T1567(0.2); S1570(3.7); S1581(33.3); T1582(33.3); S1583(33.3)]	1.145	0.195347598	0.945628616
Q14676	1xPhospho [S485(100); S495(0); S498(0)]	1.148	0.199122642	0.948093638
Q14676	2xPhospho [S329(100); T331(100); T341(0)]	1.144	0.194087052	0.962617576
Q14676	3xPhospho [S495(100); S498(100); S504(4.2); S505(95.9)]	1.165	0.220329955	0.962617576
Q14676	2xPhospho [S495(100); S498(100)]	0.737	-0.440263476	0.969482098
Q14676	2xPhospho [S445(0); T447(0); T448(0); T449(0); S453(100); T455(100)]	0.922	-0.117161344	0.973763863
Q14676	2xPhospho [S1766(100); T1768(0); S1775(100); T1781(0); S1786(0)]	1.043	0.060739158	0.980895643
Q14676	2xPhospho [S329(100); T331(100); T341(0)]	0.899	-0.153606979	0.983706057
Q14676	2xPhospho [S394(0); S397(0); S402(100); T404(100); S411(0); T415(0)]	0.902	-0.148800661	0.984063817
Q14676	3xPhospho [S394(0.2); S397(99.8); S402(100); T404(100); S411(0); T415(0)]	0.855	-0.226003675	0.985461924
Q14676	2xPhospho [S394(0); S397(0); S402(100); T404(100); S411(0); T415(0)]	1.024	0.034215715	0.994865845
Q14676	2xPhospho [S485(0); S495(100); S498(100)]	0.917	-0.125006361	1
Q14677	2xPhospho [T284(0); Y293(0); T294(95.9); S299(4.1); S305(0.2); T306(0.2); T308(99.6); S311(0); S312(0)]	0.56	-0.836501268	0.624540466
Q14677	1xPhospho [T284(0); Y293(0); T294(0); S299(100); S305(0); T306(0); T308(0); S311(0); S312(0)]	0.685	-0.545824107	0.763576928
Q14684	3xPhospho [T451(99.2); S452(89.8); S453(5.5); T454(5.5); S458(99.7); S460(0.3)]	0.246	-2.023269779	0.013454834
Q14684	3xPhospho [T451(86.7); S452(86.7); S453(13.3); T454(13.3); S458(99.7); S460(0.3)]	0.401	-1.318325858	0.41723937
Q14684	2xPhospho [T728(100); T730(50); S731(50); S732(0.1); S735(0); S736(0)]	1.6	0.678071905	0.656356957
Q14684	2xPhospho [S335(1.3); S338(1.3); S339(1.3); S341(1.3); S344(93.5); S350(1.5); S359(99.8)]	1.492	0.577247536	0.767551297
Q14684	3xPhospho [T728(99.6); T730(0.6); S731(49.8); S732(49.8); S735(5.8); S736(94.3)]	1.409	0.494671612	0.805428458
Q14684	1xPhospho [S513(100); T515(0)]	1.417	0.502839758	0.853656829
Q14684	2xPhospho [S661(0); S662(0); T663(0); T665(0); T677(97.1); S679(5.7); S680(94.4); S681(2.9)]	1.319	0.399444565	0.880267055
Q14684	2xPhospho [T451(0); S452(3.8); S453(96.1); T454(0.2); S458(0); S460(100)]	0.799	-0.323732592	0.893481203
Q14684	2xPhospho [T451(0.1); S452(33.3); S453(33.3); T454(33.3); S458(99.8); S460(0.2)]	0.771	-0.375197235	0.954977633
Q14684	1xPhospho [S489(0); S498(99.9); S500(0.1); S503(0.1); S505(0)]	0.806	-0.311148256	0.965013037
Q14684	1xPhospho [T451(0); S452(0); S453(0); T454(0); S458(100); S460(0)]	1.11	0.150559677	0.965139891
Q14684	1xPhospho [S245(100); S255(0)]	0.836	-0.258425153	0.980895643
Q14684	1xPhospho [S422(100)]	1.026	0.037030731	0.99184412
Q14690	2xPhospho [T1002(1.4); T1006(92); S1010(92); T1012(14.6); T1024(0); T1027(0)]	2.505	1.324810603	0.179675012

Q14690	3xPhospho [S1534(0); S1535(0); S1545(0); T1547(0); S1556(100); S1557(100); S1559(100); T1569(0)]	0.949	-0.075520008	1
Q14692	1xPhospho [T638(4.3); S639(95.7); Y651(0); S658(0)]	0.835	-0.260151897	0.962617576
Q14694	1xPhospho [S563(0); S576(100)]	1.515	0.599317794	0.690850307
Q14694	1xPhospho [Y364(0); S365(100); S370(0); S374(0)]	1.699	0.764685853	0.701261726
Q14694	2xPhospho [Y364(0); S365(100); S370(100); S374(0)]	1.283	0.35952117	0.86936135
Q14694	2xPhospho [S563(100); S576(100)]	1.166	0.221567789	0.947600551
Q14694	3xPhospho [T208(4); S211(96); S215(96); T216(4.2); S218(99.8); S220(0); S226(0); S234(0); T236(0)]	0.99	-0.01449957	0.980895643
Q14694	1xPhospho [T208(0.1); S211(99.9); S215(0); T216(0); S218(0); S220(0); S226(0); S234(0); T236(0)]	1.005	0.007195501	0.989340879
Q14694	2xPhospho [T208(3.7); S211(96.3); S215(0); T216(3.7); S218(96.3); S220(0); S226(0); S234(0); T236(0)]	0.951	-0.072482754	1
Q14789	1xPhospho [S6(0); S17(99.9); T22(0.1)]	0.703	-0.508403406	0.963156927
Q147X3	2xPhospho [S39(100); S53(93); S55(7); S61(0); T63(0)]	1.095	0.13093087	0.972501767
Q147X3	1xPhospho [S39(100); S53(0)]	0.917	-0.125006361	1
Q14839	3xPhospho [S85(0); S86(0); S103(98.9); S105(99.9); S108(90.2); Y110(0.2); T111(10.9)]	0.517	-0.951763814	0.533086874
Q14839	3xPhospho [S85(0); S86(0); S103(100); S105(100); S108(100); Y110(0); T111(0)]	0.567	-0.81857936	0.754584839
Q14966	2xPhospho [S508(100); S510(100); Y514(0); Y516(0)]	0.37	-1.434402824	0.498736736
Q14966	2xPhospho [S508(100); S510(100); Y514(0); Y516(0)]	1.455	0.541019153	0.791280679
Q14966	2xPhospho [S508(100); S510(100); Y514(0); Y516(0)]	0.702	-0.510457064	0.920957334
Q14966	1xPhospho [T1487(0); S1490(99.9); T1493(0.1); S1498(0)]	0.987	-0.01887801	0.973263372
Q14978	2xPhospho [S387(50); S388(50); T394(0); T395(0); S397(100)]	100	6.64385619	8.35E-17
Q14978	1xPhospho [S686(0); S688(0); S693(0); S698(100)]	0.441	-1.181149439	0.254834652
Q14978	2xPhospho [T607(100); T610(100)]	2.054	1.038436182	0.284397229
Q14978	2xPhospho [T607(100); T610(100)]	1.741	0.799916203	0.581555081
Q14978	1xPhospho [S387(0); S388(0); T394(0); T395(0.1); S397(99.9)]	1.808	0.854394678	0.690107651
Q14978	1xPhospho [S637(100)]	1.638	0.711935357	0.705680833
Q14978	1xPhospho [S686(100); S688(0); S693(0)]	1.231	0.299830762	0.912090563
Q14978	2xPhospho [T607(100); T610(100)]	1.236	0.305678743	0.920957334
Q14978	1xPhospho [Y289(92.9); S290(3.5); S291(3.5); S296(0)]	1.08	0.111031312	0.939608401
Q14978	2xPhospho [S686(0); S688(0); S693(100); S698(100)]	0.893	-0.16326792	0.965013037
Q14978	1xPhospho [Y289(0); S290(100); S291(0); S296(0)]	1.111	0.151858817	0.971220368
Q14978	3xPhospho [S686(100); S688(0); S693(100); S698(100)]	1.043	0.060739158	0.980895643
Q14C86	6 2xPhospho [T562(0); S566(100); S569(100); S576(0); S580(0)]	0.48	-1.058893689	0.462645628
Q14C86	6 2xPhospho [S757(51); S758(51); S761(94); T762(4.1); S766(0); S769(0); S772(0); T774(0); S775(0)]	0.668	-0.582079992	0.693687388
Q14C86	6 2xPhospho [S566(100); S569(100); S576(0); S580(0)]	0.478	-1.064917477	0.711284181
Q14C86	6 2xPhospho [S757(99.6); S758(95); S761(5); T762(0.5); S766(0); S769(0); S772(0); T774(0); S775(0)]	0.68	-0.556393349	0.716501219
Q14C86	6 3xPhospho [S757(50); S758(50); S761(100); T762(100); S766(0); S769(0); S772(0); T774(0); S775(0)]	0.822	-0.282789701	0.948093638
Q14C86	6 1xPhospho [S1105(100); S1112(0); S1114(0); S1122(0); S1125(0); Y1126(0)]	1.18	0.23878686	0.948782091
Q14C86	6 3xPhospho [T965(90.5); S966(9.5); S973(83.3); S974(12.1); S975(12.1); S976(44); S977(44); S979(4.7)]	1.033	0.046840254	0.9616322
Q14C86	6 3xPhospho [S757(53.1); S758(53.1); S761(96.9); T762(96.9); S766(0); S769(0); S772(0); T774(0); S775(0)]	0.821	-0.284545873	0.976345951
Q14C86	6 1xPhospho [S929(98.7); S930(1.3); S934(0); S935(0)]	0.824	-0.279283757	0.99184412
Q15003	1xMet-loss [N-Term]; 1xPhospho [T11(0); S15(99.9); S16(0); S17(0); T19(0)]	1.661	0.732052073	0.618224241
Q15007	2xPhospho [S326(0); Y328(0); S333(0); Y336(0); S338(100); S341(99.9); T343(0.1); S345(0); S348(0); T350(0); S353(0); T356(0); S358(0); S359(0)]	2.455	1.295723025	0.248110757
Q15018	1xPhospho [S397(0); T410(92.2); T412(3.9); S413(3.9)]	4.385	2.132576843	0.03936892
Q15018	3xPhospho [S368(100); S372(100); S375(100); Y377(0); T384(0); S387(0); S389(0); Y391(0); S392(0); S394(0)]	0.247	-2.017417053	0.05315148
Q15019	1xPhospho [Y211(0); S218(100); T228(0)]	0.787	-0.345564459	0.879063032
Q15019	1xPhospho [Y211(0); S218(100)]	0.596	-0.746615764	0.886312012
Q15022	1xPhospho [S539(0); S541(0); S546(100)]	1.471	0.556797247	0.749797187
Q15022	1xPhospho [S14(2.4); S17(95.3); S20(2.4); S28(0); T35(0); S37(0)]	0.682	-0.552156356	0.848293321
Q15047	1xPhospho [Y1061(0); Y1063(0); S1066(100)]	0.964	-0.052894948	0.973763863
Q15056	1xPhospho [S193(100)]	1.27	0.344828497	0.904774194
Q15056	1xPhospho [S193(100); T201(0)]	1.164	0.219091058	0.949541112
Q15057	1xPhospho [Y516(0); S517(0); S519(0); S521(100)]	100	6.64385619	8.35E-17
Q15061	1xPhospho [S431(100); S437(0)]	2.452	1.293958979	0.403375271
Q15149	4 2xPhospho [T19(97.4); S20(97.4); S21(5.1); Y26(0)]	0.088	-3.506352666	0.000482669
Q15149	4 1xPhospho [S4505(100); T4509(0); S4511(0); S4514(0); T4516(0); S4518(0); S4519(0); S4520(0); S4521(0); Y4522(0); S4523(0); S4524(0); S4525(0); Y4527(0)]; Q15149 1xPhospho [S4642(100); T4646(0); S4648(0); S4651(0); T4653(0); S4655(0); S4656(0); S4657(0); S4658(0); Y4659(0); S4660(0); S4661(0); S4662(0); Y4664(0)]; Q15149-2 1xPhospho [S4532(100); T4536(0); S4538(0); S4541(0); T4543(0); S4545(0); S4546(0); S4547(0); S4548(0); Y4549(0); S4550(0); S4551(0); S4552(0); Y4554(0)]	0.403	-1.311148256	0.290974584
Q15149	4 1xPhospho [T19(0); S20(0); S21(100); Y26(0)]	0.477	-1.067938829	0.309161518
Q15149	4 2xPhospho [Y4474(0); Y4475(0); S4476(0); Y4478(0); S4479(0); S4481(0); S4483(0); S4485(99.9); T4486(0); S4489(100)]; Q15149 2xPhospho [Y4611(0); Y4612(0); S4613(0); Y4615(0); S4616(0); S4618(0); S4620(0); S4622(99.9); T4623(0); S4626(100)]; Q15149-2 2xPhospho [Y4501(0); Y4502(0); S4503(0); Y4505(0); S4506(0); S4508(0); S4510(0); S4512(99.9); T4513(0); S4516(100)]	0.623	-0.682695932	0.701261726

Q15149	4 1xPhospho [S4247(0); S4248(0); S4249(0); S4252(0); S4253(0); S4254(0); S4255(0); Y4256(0); S4259(0); S4263(100)]; Q15149 1xPhospho [S4384(0); S4385(0); S4386(0); S4389(0); S4390(0); S4391(0); S4392(0); Y4393(0); S4396(0); S4400(100)]; Q15149-2 1xPhospho [S4274(0); S4275(0); S4276(0); S4279(0); S4280(0); S4281(0); S4282(0); Y4283(0); S4286(0); S4290(100)]	0.714	-0.486004021	0.777165105
Q15149	1xPhospho [T113(0); S125(100)]	0.527	-0.924125133	0.783976635
Q15149	4 2xPhospho [S4505(100); T4509(0); S4511(0); S4514(0); T4516(0); S4518(0); S4519(0); S4520(0.1); S4521(99.8); Y4522(0); S4523(0.1); S4524(0); S4525(0); Y4527(0)]; Q15149 2xPhospho [S4642(100); T4646(0); S4648(0); S4651(0); T4653(0); S4655(0); S4656(0); S4657(0.1); S4658(99.8); Y4659(0); S4660(0.1); S4661(0); S4662(0); Y4664(0)]; Q15149-2 2xPhospho [S4532(100); T4536(0); S4538(0); S4541(0); T4543(0); S4545(0); S4546(0); S4547(0.1); S4548(99.8); Y4549(0); S4550(0.1); S4551(0); S4552(0); Y4554(0)]	0.7	-0.514573173	0.808648811
Q15149	4 2xPhospho [S4247(0.1); S4248(0.1); S4249(99.9); S4252(97.3); S4253(2.6); S4254(0.1); S4255(0.1); Y4256(0); S4259(0); S4263(0)]; Q15149 2xPhospho [S4384(0.1); S4385(0.1); S4386(99.9); S4389(97.3); S4390(2.6); S4391(0.1); S4392(0.1); Y4393(0); S4396(0); S4400(0)]; Q15149-2 2xPhospho [S4274(0.1); S4275(0.1); S4276(99.9); S4279(97.3); S4280(2.6); S4281(0.1); S4282(0.1); Y4283(0); S4286(0); S4290(0)]	0.779	-0.360304767	0.872730156
Q15149	4 1xPhospho [S1595(100)]; Q15149 1xPhospho [S1732(100)]; Q15149-2 1xPhospho [S1622(100)]	0.955	-0.066427362	0.981858798
Q15223	1xPhospho [Y418(0); S422(100)]	0.691	-0.533242384	0.965139891
Q15223	1xPhospho [Y418(0); S422(100)]	0.869	-0.202571918	0.972363997
Q15233	1xPhospho [T440(0); T450(100)]	1.131	0.177598929	0.965013037
Q15262	2xPhospho [Y849(0); T854(0.2); S856(99.8); Y858(0); T860(100)]	100	6.64385619	8.35E-17
Q15269	3xPhospho [S885(0); S891(100); S898(94.6); S902(52.7); S913(52.7)]	0.286	-1.805912948	0.03919504
Q15276	2xPhospho [S407(99.8); T408(0.2); S410(95.7); T413(0.2); S414(0); S416(4.1); S419(0)]	10.081	3.333566851	0.000113912
Q15276	2xPhospho [S374(100); S377(100); S386(0); S391(0)]	1.462	0.547943311	0.767575271
Q15311	2xPhospho [T44(0); S48(100); S62(100)]	1.264	0.337996464	0.860444802
Q15311	3xPhospho [S22(33.1); T25(33.1); T27(33.1); S29(0.5); S30(0.5); S34(99.9); T36(99.7)]	1.049	0.069014678	0.960178862
Q15311	2xPhospho [T27(0); S29(100); S30(0); S34(100); T36(0); Y42(0)]	0.847	-0.239566125	0.965013037
Q15311	2xPhospho [T82(0); Y85(0); S92(100); S93(100); S99(0); S101(0)]	0.854	-0.227692025	0.973599548
Q15311	2xPhospho [T44(0.3); S48(99.7); S62(100)]	1.022	0.031395196	0.985461924
Q15311	2xPhospho [S22(0); T25(3.9); T27(96.1); S29(100); S30(0); S34(0); T36(0)]	0.988	-0.017417053	0.98830288
Q15361	2xPhospho [T12(100); S15(100)]	6.094	2.6073895	0.012165185
Q15361	1xPhospho [S64(0); S65(100)]	0.931	-0.103146927	0.982743754
Q15365	1xPhospho [S246(0); T255(0); S262(0.2); S263(3.7); S264(96.2)]	0.465	-1.104697379	0.344598215
Q15365	1xPhospho [T169(0); S171(0); S173(100)]	0.657	-0.606034724	0.797966154
Q15365	1xPhospho [T169(0); S171(0); S173(100)]	0.895	-0.160040413	0.977860291
Q15365	1xPhospho [T180(0); Y183(0); S189(0.1); S190(99.9)]	1.014	0.020057652	0.987464758
Q15366	1xPhospho [S254(0); T259(0); T263(0); S266(0); S270(0); S271(0.2); S272(99.8)]	0.453	-1.142417045	0.282829812
Q15366	1xPhospho [T179(0); Y182(0); S187(2.5); S188(0.1); S189(97.5)]	0.722	-0.469929258	0.886312012
Q15366	1xPhospho [T169(0); S171(0); S173(100)]	0.941	-0.087733372	1
Q15398	1 1xPhospho [T579(0); T580(4.4); S581(95.6); T590(0)]	100	6.64385619	8.35E-17
Q15418	1xPhospho [S363(100); S369(0)]	100	6.64385619	8.35E-17
Q15418	2xPhospho [T359(100); S363(100); S369(0)]	0.504	-0.988504361	0.385604967
Q15424	1xPhospho [T307(0); S321(0); S322(0); S325(0); S331(0); S332(0); T340(0); S344(100)]	0.01	-6.64385619	8.35E-17
Q15424	3xPhospho [T307(0); S321(46.4); S322(46.4); S325(7.7); S331(49.7); S332(49.7); T340(0); S344(100)]	0.053	-4.23786383	2.04E-07
Q15424	1xPhospho [T188(100); T194(0); S195(0); S196(0); S197(0); T200(0); S209(0)]	7.658	2.93696766	0.000275388
Q15424	1xPhospho [S227(0); S234(0); S235(0); T245(0.1); S246(2.5); S247(97.4)]	1.435	0.521050737	0.724913856
Q15424	1xPhospho [S227(0); S234(0); S235(0); T245(0); S246(0.1); S247(100)]	1.524	0.607862903	0.73347811
Q15424	2xPhospho [S601(100); S604(100)]	0.677	-0.562772261	0.872730156
Q15424	2xPhospho [S601(100); S604(100)]	0.707	-0.50021788	0.872730156
Q15424	1xPhospho [S601(100); S604(0)]	1.315	0.3950628	0.87941354
Q15424	2xPhospho [T307(0); S321(0); S322(0); S325(0.2); S331(49.9); S332(49.9); T340(0); S344(100)]	0.847	-0.239566125	0.962617576
Q15424	2xPhospho [S601(100); S604(100)]	0.822	-0.282789701	0.964495484
Q15424	1xPhospho [S601(0); S604(100)]	1.11	0.150559677	0.965139891
Q15424	1xMet-loss+Acetyl [N-Term]; 1xPhospho [T4(2.3); S6(97.7); S11(0); S20(0); S21(0); S23(0); S24(0); T26(0); T28(0)]	0.774	-0.369594529	0.971973958
Q15424	2xPhospho [S227(0); S234(96.4); S235(3.6); T245(1.9); S246(49.1); S247(49.1)]	1.109	0.149259365	0.979214627
Q15424	1xPhospho [S601(0); S604(100)]	0.879	-0.18606493	0.989340879
Q15428	1xPhospho [S222(100)]	1.791	0.840765337	0.552024958
Q15459	1xPhospho [S320(0); S329(100)]	1.555	0.63691458	0.655156419
Q15459	1xPhospho [S344(0); T350(0); S359(100)]	1.407	0.492622329	0.81171187
Q15459	1xPhospho [S451(100); Y456(0); S464(0); S465(0)]	0.555	-0.849440323	0.84718129
Q15527	2xPhospho [S183(0); T184(0); T190(100); T195(100)]	0.707	-0.50021788	0.769621572
Q15527	2xPhospho [S183(0); T184(0); T190(100); T195(100)]	0.829	-0.270555993	0.93864152
Q15527	1xPhospho [S183(0); T184(0); T190(100); T195(0)]	0.772	-0.373327247	0.973263372
Q15545	1xPhospho [S264(100); T274(0)]	1.147	0.197865391	0.969680228
Q15554	1xPhospho [T317(0); S323(100)]	0.523	-0.935117148	0.724184987
Q15569	2xPhospho [S437(100); S440(0); S441(100)]	1.251	0.32308179	0.872730156
Q15637	5 2xPhospho [T192(0); S205(100); S207(100); Y212(0); S214(0)]	100	6.64385619	8.35E-17
Q15637	5 1xPhospho [S112(100)]	1.327	0.408168371	0.855302203
Q15637	5 2xPhospho [S205(100); S207(100); Y212(0); S214(0)]	1.464	0.549915554	0.855534442
Q15637	5 1xPhospho [S427(100)]	1.247	0.318461465	0.921446515
Q15637	5 2xPhospho [T192(0); S205(100); S207(100); Y212(0); S214(0)]	1.005	0.007195501	1
Q15642	2xPhospho [S296(100); S298(0); S299(100); T302(0); S304(0)]	0.351	-1.510457064	0.024034163

Q15642	1xPhospho [S296(100); S298(0); S299(0); T302(0); S304(0)]	0.546	-0.873027144	0.66184163
Q15643	1xPhospho [S1842(100); T1846(0); S1854(0); S1858(0); S1859(0); S1861(0)]	0.936	-0.095419565	0.99184412
Q15643	1xPhospho [S1882(0); S1891(100)]	0.893	-0.16326792	1
Q15643	2xPhospho [S1842(100); T1846(100); S1854(0); S1858(0); S1859(0); S1861(0)]	0.995	-0.007231569	1
Q15648	2xPhospho [T1017(93.5); S1021(6); S1023(0.4); S1025(0); S1026(0); S1027(0); T1032(0); T1035(0); S1036(0); T1037(0); S1040(0); S1042(99.6); S1045(0.4)]	1.897	0.923719679	0.505994612
Q15648	1xPhospho [T1071(0); S1078(0); S1079(0); S1081(0); Y1083(0); T1084(0); S1085(0.1); S1086(0.1); S1088(97.4); S1090(2.4); S1091(0.1); S1092(0); S1094(0)]	1.283	0.35952117	0.889306217
Q15648	2xPhospho [S770(1.8); S771(50); S772(50); S774(98.2); T780(0); S784(0); S791(0)]	0.678	-0.560642822	0.907438338
Q15648	2xPhospho [S1475(0); Y1476(0); S1479(100); S1481(0.1); S1482(99.9); Y1492(0); S1493(0); T1494(0)]	0.813	-0.298672743	0.908086456
Q15648	2xPhospho [T1017(0); S1021(100); S1023(0); S1025(83.6); S1026(5.5); S1027(5.5); T1032(5.5); T1035(0); S1036(0); T1037(0); S1040(0)]	0.687	-0.541617996	0.956734015
Q15648	1xPhospho [Y1431(0); S1433(100); S1437(0); S1439(0); T1440(0)]	1.04	0.056583528	0.971183767
Q15648	1xPhospho [S1021(99.9); S1023(0.1); S1025(0); S1026(0); S1027(0); T1032(0); T1035(0); S1036(0); T1037(0); S1040(0)]	1.022	0.031395196	0.977858961
Q15648	2xPhospho [T1017(0); S1021(100); S1023(0); S1025(0.3); S1026(0.3); S1027(4.7); T1032(94.8); T1035(0); S1036(0); T1037(0); S1040(0)]	0.998	-0.002888279	0.987554799
Q15648	3xPhospho [S1447(50); S1449(50); S1451(0.3); S1453(99.8); Y1456(0); T1457(0); S1463(99.8); S1465(0.2); S1467(0); S1469(0); S1470(0)]	0.858	-0.220950447	0.989102507
Q15648	2xPhospho [S1207(100); T1215(33.3); S1218(33.3); S1219(33.3)]	1.007	0.010063683	0.998271017
Q15652	2xPhospho [S2045(7.1); S2047(92.9); T2052(92.9); S2053(7.1); S2057(0); S2064(0); T2065(0)]	0.01	-6.64385619	8.35E-17
Q15652	2xPhospho [S638(1.7); S639(98.4); S641(100)]	2.213	1.146003451	0.226297325
Q15654	1xPhospho [T27(100)]	0.412	-1.279283757	0.535347951
Q15678	3xPhospho [T697(86.7); S699(86.7); T702(95.4); S707(14); S708(14); S710(3.2); S720(0)]	100	6.64385619	8.35E-17
Q15678	1xPhospho [T459(0); S461(0); S463(2.4); S465(97.6)]	0.64	-0.64385619	0.922170166
Q15678	1xPhospho [S642(100); S648(0)]	0.753	-0.40927823	0.969296885
Q15678	2xPhospho [Y589(0); S591(0); S593(100); S594(100); T599(0)]	0.879	-0.18606493	0.989838391
Q15691	1xPhospho [T135(0); S140(100)]	3.625	1.857980995	0.037786912
Q15691	1xPhospho [T154(0); S155(0); S156(0); S157(0); S165(100); T166(0)]	1.487	0.572404647	0.701261726
Q15691	2xPhospho [T154(0); S155(100); S156(0); S157(0); S165(100); T166(0)]	0.937	-0.093879047	0.979498769
Q15742	2xPhospho [S157(0); S159(100); S162(100)]	1.336	0.417920008	0.870080472
Q15742	3xPhospho [S157(4.4); S159(95.6); S162(100); S171(100)]	1.162	0.216610069	0.963131675
Q15742	2xPhospho [S162(100); S171(100)]	1.109	0.149259365	0.964978421
Q15750	1xPhospho [S7(0); S11(0); S16(100); T18(0); S27(0); S31(0); S33(0)]	1.381	0.46571332	0.851597639
Q15751	2xPhospho [T2701(99.6); S2705(0.2); S2706(0.2); T2709(0.2); S2710(95.7); S2712(4.2)]	0.121	-3.046921047	0.003770035
Q15751	3xPhospho [T2701(100); S2705(50); S2706(50); T2709(0); S2710(100); S2712(0)]	0.884	-0.177881725	0.980895643
Q15773	1xPhospho [S238(98.8); S240(1.3)]	1.212	0.277389699	0.93455291
Q15785	1xPhospho [S186(100)]	1.205	0.269033146	0.925724009
Q15847	1xPhospho [T16(0); S22(100); T36(0)]	100	6.64385619	8.35E-17
Q15847	1xPhospho [T57(0); S62(0); T64(0); S66(100)]	100	6.64385619	8.35E-17
Q15910	2xPhospho [S362(3.7); S363(96.3); S366(0.2); T367(99.9); T369(0); S375(0)]	1.865	0.89917563	0.411842732
Q15910	2xPhospho [S362(0.1); S363(100); S366(50); T367(50); T369(0); S375(0)]	1.549	0.631337144	0.642636279
Q16204	3xPhospho [T349; S351; S352; Y356; T357; S359; S361; S362; S363; S367; Y372; S374; T376; T380; S384; T386]	0.672	-0.573466862	0.792141506
Q16204	1xPhospho [S240(0); S244(100)]	1.05	0.070389328	0.969482098
Q16204	2xPhospho [S240(100); S244(100)]	1.086	0.119024103	0.976789257
Q16204	2xPhospho [T349(0); S351(0); S352(0); Y356(0); T357(0); S359(0.3); S361(51.5); S362(51.5); S363(96.1); S367(0.5); S371(0); Y372(0); S374(0); T376(0); T380(0); T383(0); S384(0); T386(0)]	0.975	-0.036525876	0.99277609
Q16513	1xPhospho [S603(0); T605(0); S615(100)]	0.346	-1.531156057	0.432550331
Q16513	1xPhospho [S559(0); S561(98.3); T562(1.7); T564(0)]	0.4	-1.321928095	0.554164534
Q16513	1xPhospho [T13(0); S19(100)]	0.707	-0.50021788	0.963156927
Q16513	2xPhospho [S110(99.7); T116(50); T121(50); T124(0.3)]	0.857	-0.222632891	0.980895643
Q16514	1xPhospho [S51(100); T59(0)]	1.33	0.411426246	0.878612438
Q16514	2xPhospho [T43(100); S51(100); T59(0)]	1.262	0.33571191	0.904227116
Q16543	1xPhospho [T370(3.6); S377(96.4)]	1.573	0.653518671	0.691787621
Q16543	1xPhospho [S127(100); T131(0); T136(0); S140(0)]	1.49	0.575312331	0.75802349
Q16543	1xMet-loss+Acetyl [N-Term]; 1xPhospho [Y4(0); S5(0); S13(100); T19(0); T25(0); S27(0)]	1.19	0.250961574	0.922170166
Q16555	1xPhospho [S537(0); S540(100); S542(0)]	0.616	-0.698997744	0.751204811
Q16555	2xPhospho [S537(100); S540(100); S542(0)]	0.689	-0.537424112	0.963250338
Q16566	2xPhospho [S341(0); S343(0); S344(0.1); S345(0.1); S348(0); S352(8); S356(91.8); S360(100)]	1.03	0.042644337	0.973263372
Q16584	2xPhospho [S780(0); S783(0); S789(100); S793(100)]	1.3	0.378511623	0.878612438
Q16643	1xPhospho [S302(96.1); S304(3.7); S307(0.2); S312(0); S320(0); S324(0)]	0.393	-1.347398782	0.189479773
Q16643	1xPhospho [S141(98.8); S142(1.3)]	1.52	0.604071324	0.698520686
Q16643	2xPhospho [T331(0); T335(50); S337(50); S339(0); S341(0); S342(0.1); T343(2.6); S345(97.3); T346(0.1)]	1.397	0.482332021	0.766475576
Q16643	2xPhospho [S302(99.8); S304(0.2); S307(100); S312(0); S320(0); S324(0)]	0.65	-0.621488377	0.788815962
Q16643	1xPhospho [S337(0); S339(100); S341(0); S342(0); T343(0); S345(0); T346(0)]	1.229	0.297484916	0.920957334
Q16643	1xPhospho [T591(99.9); S594(0); Y597(0); S599(0); S601(0.1); S609(0)]	0.793	-0.334607229	0.965351295
Q16643	2xPhospho [S337(0); S339(100); S341(0); S342(99.9); T343(0); S345(0); T346(0)]	0.942	-0.086201035	0.998448514
Q16763	1xPhospho [S173(99.9); T175(0.1); S178(0); S179(0); T180(0)]	1.065	0.09085343	0.972294713
Q16828	1xPhospho [T346(0); S350(0.1); S351(100)]	2.515	1.3305584	0.332983505
Q16828	1xPhospho [S328(0); S331(100)]; Q99956 1xPhospho [S325(0); S328(100)]	1.085	0.117695043	0.974924677
Q16890	2xPhospho [T168(0); S174(100); S180(0); S181(0); T182(0); S186(0); S189(0); S194(100)]	1.492	0.577247536	0.748856955

Q16890	1xPhospho [S144(0); T146(0); S149(100)]	0.848	-0.23786383	0.983092362
Q1ED39	2xPhospho [S297(0); T308(100); T310(100)]	0.01	-6.64385619	8.35E-17
Q1ED39	2xPhospho [T47(50); S48(50); S50(0.1); S52(99.9)]	0.172	-2.53951953	6.55E-05
Q1MSJ5	1 2xPhospho [T1151(100); S1155(50); S1156(50)]	0.579	-0.788364747	0.694198512
Q27J81	2xPhospho [T1135(0); S1136(0); S1147(50); T1148(50); S1149(100); S1158(0)]	1.136	0.183962835	0.956843841
Q29RF7	1xPhospho [S1223(0); T1227(0); S1232(98.5); S1233(1.6)]	1.445	0.531069493	0.786602611
Q29RF7	1xPhospho [S1206(0); T1208(100)]	1.223	0.290424404	0.933760225
Q2KHR2	2xPhospho [S349(96.7); T351(3.2); T352(0.1); T356(93.9); S357(3.1); S358(3.1)]	0.486	-1.040971781	0.743965448
Q2KHR3	1xPhospho [S981(0); T983(0); S987(100); S991(0); S997(0); T998(0)]	1.404	0.489542936	0.797966154
Q2KHR3	2xPhospho [T1334(0); T1341(100); T1346(0); S1348(100); Y1354(0)]	1.195	0.257010618	0.938730361
Q2KHR3	2xPhospho [S1227(0); S1228(0); S1230(100); S1231(100); Y1238(0); S1239(0); Y1243(0)]	0.984	-0.023269779	0.987937869
Q2M2I8	1xPhospho [S14(0); S18(3.2); S20(93.7); S21(3.2); S26(0); T27(0); S28(0); S32(0); Y34(0)]	0.01	-6.64385619	8.35E-17
Q2M2I8	2xPhospho [S618(0); T620(100); S623(1.6); S624(98.4)]	1.483	0.568518598	0.766139179
Q2M2Z5	2xPhospho [S317(100); S321(100); S326(0); Y328(0); S331(0)]	0.949	-0.075520008	0.992376868
Q2NKQ1	1xPhospho [S229(0.1); S230(96.9); S232(3); S238(0); S240(0)]	1.126	0.171206827	0.97998947
Q2PPJ7	2xPhospho [S373(50.7); S375(50.7); S376(98.6); S379(0)]	0.927	-0.109358756	0.992375794
Q2YD98	2xPhospho [S273(0); T275(0); T277(0); S281(100); S287(100)]	0.585	-0.77349147	0.783976635
Q32MZ4	1xPhospho [T665(0); T676(0); T683(0); S686(97.8); S687(2.2)]	0.469	-1.092340172	0.554526265
Q32MZ4	1xPhospho [T727(0); S733(100); S735(0); S739(0); S746(0); T747(0)]	1.194	0.255802837	0.936043234
Q32MZ4	1xPhospho [S104(0); T107(0); S110(0); T114(1.7); S115(96.7); S116(1.7)]	0.739	-0.436353731	0.94797789
Q32N00	1xPhospho [S201(100); T205(0); T208(0)]	2.172	1.119024103	0.41769478
Q32P44	1xPhospho [S176(100); S177(0)]	0.715	-0.483984853	0.935658245
Q3B726	2xPhospho [S297(94.2); S299(35.3); S300(35.3); Y302(0); S304(35.3)]	0.251	-1.994240731	0.176976273
Q3B726	1xPhospho [S297(0); S299(0); S300(0); Y302(0); S304(100)]	1.575	0.655351829	0.607509232
Q3B726	1xPhospho [S297(0); S299(0.1); S300(97.7); Y302(0); S304(2.2)]	1.548	0.630405471	0.660888692
Q3B726	2xPhospho [S316(100); T322(0); S328(100)]	0.625	-0.678071905	0.675715704
Q3B726	1xPhospho [S316(100); T322(0)]	0.657	-0.606034724	0.86443084
Q3B726	2xPhospho [S316(100); T322(0); S328(100)]	0.73	-0.454031631	0.870654365
Q3B726	3xPhospho [S316(100); T322(100); S328(100)]	1.292	0.36960607	0.882100453
Q3B726	2xPhospho [S297(0); S299(50); S300(50); Y302(0); S304(100)]	1.022	0.031395196	0.981208663
Q3B726	2xPhospho [S297(33.3); S299(33.3); S300(33.3); Y302(0); S304(100)]	0.916	-0.126580497	0.99184412
Q3B820	2xPhospho [S650(100); S655(100); S659(0)]	100	6.64385619	8.35E-17
Q3KQU3	3xPhospho [S539(0); S544(100); S548(100); S552(96.3); T554(3.7)]	0.01	-6.64385619	8.35E-17
Q3KQU3	2xPhospho [S112(2.7); S113(97.3); S116(97.3); T118(2.7); S123(0); S125(0); T128(0)]	0.509	-0.974262439	0.467067441
Q3KQU3	3xPhospho [S112(4.1); S113(95.9); S116(4.1); T118(95.9); S123(0); S125(100); T128(0)]	1.575	0.655351829	0.688448945
Q3KQU3	2xPhospho [S539(0); S544(100); S548(1.8); S552(49.1); T554(49.1)]	0.969	-0.045431429	0.97008929
Q3L8U1	2xPhospho [S1468(100); S1472(100)]	1.468	0.553851968	0.783976635
Q3L8U1	3xPhospho [S611(100); S612(100); S616(100); T625(0)]	0.834	-0.261880711	0.980895643
Q3MII6	1xPhospho [S506(100)]	1.144	0.194087052	0.930376591
Q3MIP1	1xPhospho [S112(100); S119(0)]	0.659	-0.60164963	0.906218259
Q3T8J9	1xPhospho [S1886(0); S1895(1.5); S1896(98.6)]	1.865	0.89917563	0.716990671
Q4G0J3	3xPhospho [S337(100); T338(100); T344(0); S350(50); S351(50)]	10.716	3.421694581	7.02E-05
Q4G0J3	1xPhospho [S254(0); T257(0); S258(0); S261(100); S265(0); T266(0)]	2.813	1.492109553	0.055125602
Q4G0J3	2xPhospho [S337(100); T338(100); T344(0); S350(0); S351(0)]	1.742	0.800744624	0.58509702
Q4G0J3	1xPhospho [S298(0); S299(0); S300(100); S305(0)]	1.872	0.904580435	0.645640221
Q4G0J3	2xPhospho [S337(100); T338(100); T344(0)]	1.465	0.550900665	0.72445075
Q4G0J3	2xPhospho [S254(0); T257(0); S258(100); S261(100); S265(0); T266(0)]	1.146	0.196607044	0.950730609
Q4G0J3	2xPhospho [S298(98.1); S299(98.1); S300(3.8); S305(0)]	1.088	0.121678557	0.962617576
Q4G0J3	1xPhospho [S298(97.6); S299(2.4); S300(0.1); S305(0)]	1.106	0.145351386	0.965013037
Q4G0J3	2xPhospho [S254(0); T257(50); S258(50); S261(100); S265(0); T266(0)]	0.842	-0.248107862	0.983706057
Q4KMP7	2xPhospho [T135(0); S141(0); T148(100); T150(0.9); T152(49.5); T154(49.5)]	1.596	0.674460652	0.707492269
Q4KMP7	1xPhospho [S22(100)]	1.782	0.833497337	0.719382659
Q4KMP7	1xPhospho [S678(100)]	1.337	0.418999465	0.862506613
Q4KMP7	2xPhospho [S687(100); T697(0); S704(0); S707(100); T709(0); S712(0); T713(0); S717(0); S718(0)]	0.731	-0.452056689	0.904774194
Q4KMP7	2xPhospho [S656(0); S657(0); S658(100); S661(100)]	1.022	0.031395196	0.962617576
Q4KMP7	1xPhospho [S22(100)]	1.111	0.151858817	0.96293109
Q4KMP7	2xPhospho [S656(0); S657(2.4); S658(97.6); S661(100)]	1.048	0.067638717	0.97726974
Q4LE39	1xPhospho [S468(0); S471(100)]	2.95	1.560714954	0.374360028
Q4LE39	2xPhospho [S666(100); T672(0); S675(100); S680(0)]	1.58	0.659924558	0.783976635
Q4LE39	2xPhospho [S790(100); T793(100); Y795(0); T801(0)]	0.659	-0.60164963	0.818315873
Q4LE39	1xPhospho [S468(0); S471(100)]	1.562	0.643394453	0.838893449
Q4LE39	2xPhospho [S790(100); T793(0); Y795(0); T801(100)]	0.895	-0.160040413	0.981858798
Q4TT58	2xPhospho [S49(0.2); S51(95.8); T56(0.4); S60(7.8); S61(95.9)]	0.823	-0.281035664	0.989898705
Q504X1	2xPhospho [S114(1.3); S115(0.1); S120(92.4); S121(92.4); S122(13.8); T126(0)]	100	6.64385619	8.35E-17
Q504X1	1xPhospho [S566(0); S567(0); T568(0); T576(0); S587(100)]	100	6.64385619	8.35E-17
Q504X1	1xPhospho [T191(0); S192(0); S194(0); S198(0); S201(100); S206(0)]	100	6.64385619	8.35E-17
Q504X1	1xPhospho [S213(0); S215(100); S220(0); S221(0); S222(0); S223(0); T224(0)]	2.122	1.085424656	0.353682182
Q504X1	3xPhospho [T230(0.3); T231(99.7); S240(100); Y243(100)]	2.003	1.002162421	0.372082627
Q504X1	2xPhospho [S213(100); S215(100); S220(0); S221(0); S222(0); S223(0); T224(0)]	1.462	0.547943311	0.711063179
Q504X1	2xPhospho [T230(4.2); T231(95.8); S240(100); Y243(0)]	1.339	0.421155961	0.848293321
Q504X1	1xPhospho [S176(100)]	1.12	0.163498732	0.965248196
Q52LR7	1xPhospho [S538(100); S540(0); T544(0); S545(0)]	100	6.64385619	8.35E-17
Q52LW3	1xPhospho [S1185(96.2); S1187(3.8); T1194(0)]	0.01	-6.64385619	8.35E-17
Q52LW3	1xPhospho [S488(0); S489(0); S492(0); S499(100)]	0.01	-6.64385619	8.35E-17

Q52LW3	2xPhospho [S925(0); S929(3.1); S930(96.9); T936(0); S937(0.1); S939(96.8); S941(3.1)]	0.01	-6.64385619	8.35E-17
Q52LW3	2xPhospho [S488(49.5); S489(49.5); S492(1.1); S499(100)]	0.01	-6.64385619	8.35E-17
Q52LW3	2xPhospho [S171(0); S176(100); S179(100); S182(0); S183(0); S184(0)]	0.01	-6.64385619	8.35E-17
Q52LW3	2xPhospho [S552(100); S555(49.2); S557(49.2); S559(1.7)]	0.01	-6.64385619	8.35E-17
Q52LW3	1xPhospho [S1029(100)]	0.235	-2.089267338	0.148316631
Q53ET0	3xPhospho [S64(0); S65(0); Y67(0); S70(100); S79(0); S86(100); S90(100); S94(0); S95(0)]	0.378	-1.40354186	0.321435351
Q53ET0	1xPhospho [S64(0); S65(0); Y67(0); S70(100); S79(0); S86(0); S90(0); S94(0); S95(0)]	1.619	0.695102986	0.716711094
Q53ET0	1xPhospho [T169(0); S170(0); S171(0); S173(0); T177(3.5); S178(96.5); S183(0); T187(0); Y188(0); T192(0); S195(0); S199(0)]	1.146	0.196607044	0.942907978
Q53EZ4	1xPhospho [S428(98.7); T430(1.3); S436(0)]	100	6.64385619	8.35E-17
Q53EZ4	2xPhospho [S425(99.9); S428(97.7); T430(2.3); S436(0)]	0.77	-0.377069649	0.96293109
Q53F19	2xPhospho [S25(100); S30(100)]	100	6.64385619	8.35E-17
Q53F19	3xPhospho [S209(100); S210(100); S225(50); S226(50); T232(0); S234(0); S240(0)]	0.838	-0.254977851	0.954977633
Q53GS9	1xPhospho [S65(100)]	1.513	0.597411988	0.737789345
Q53H80	2xPhospho [T10(0); S18(100); S21(100)]	100	6.64385619	8.35E-17
Q53H80	2xPhospho [T10(0); S18(100); S21(100)]	9.898	3.307137042	0.000313129
Q53LP3	1xPhospho [S212(1.8); S213(98.2)]	0.1	-3.321928095	0.000194273
Q53SF7	1xPhospho [T709(0); S710(0); S715(0); T716(0); S719(0); S722(0); S723(0); S726(100); T729(0); S730(0)]	0.448	-1.158429363	0.724913856
Q53T59	1xPhospho [S249(100); S254(0); S258(0); S259(0)]	1.339	0.421155961	0.860444802
Q58WW2	2xPhospho [S649(0); T654(100); S657(100)]	0.697	-0.520769439	0.86239429
Q59FP8	1xPhospho [S863(100); T867(0); S872(0); S875(0)]	0.997	-0.00433459	0.990375578
Q5BJD5	1xPhospho [S10(100); T17(0); T18(0); T27(0)]	0.929	-0.106249498	1
Q5BKZ1	2xPhospho [Y154(0); S155(0); S156(0); Y157(0); S158(99.7); S159(5.6); S161(94.5); S162(0.3); S172(0)]	100	6.64385619	8.35E-17
Q5BKZ1	1xPhospho [S48(0); S51(0); Y52(0); S56(0); Y57(0); S63(99.9); S69(0.1)]	1.558	0.639695233	0.7222669
Q5BKZ1	1xPhospho [S266(0.1); S268(3); S270(93.9); T272(0.1); T274(3)]	1.052	0.073134705	0.973263372
Q5C9Z4	1xPhospho [S280(100); T287(0)]	100	6.64385619	8.35E-17
Q5C9Z4	2xPhospho [S280(100); T287(100)]	15.57	3.960697039	2.08E-06
Q5C9Z4	3xPhospho [S317(100); S320(100); S321(100); T327(0)]	0.068	-3.878321443	0.000109122
Q5C9Z4	1xPhospho [S135(0); S139(100)]	2.044	1.031395196	0.652403988
Q5F1R6	1xPhospho [S357(0); S370(100)]	100	6.64385619	8.35E-17
Q5J9I4	1xPhospho [S28(99.9); S29(0.1); S30(0); S42(0); S43(0); S44(0)]	100	6.64385619	8.35E-17
Q5JP53	1xMet-loss [N-Term]; 1xPhospho [S7(0); T15(0); T17(0); Y18(0); S22(100)]	100	6.64385619	8.35E-17
Q5JR04	1xPhospho [S913(100); S915(0); T916(0); S917(0); S921(0); Y924(0)]	1.255	0.327687364	0.904079048
Q5JRI3	1xPhospho [S157(100); S159(0); S167(0); T170(0); Y172(0); S173(0); S174(0); Y176(0); Y177(0); T178(0); S179(0)]	1.917	0.938850337	0.467067441
Q5JRI3	1xPhospho [S133(100); Y136(0); Y138(0)]	1.433	0.51903861	0.797966154
Q5JRI3	2xPhospho [S155(95); S157(9.5); S159(0.5); S167(95); T170(0); Y172(0); S173(0); S174(0); Y176(0); Y177(0); T178(0); S179(0)]	1.297	0.37517848	0.872730156
Q5JRI3	2xPhospho [S131(100); S133(100); Y136(0); Y138(0)]	1.206	0.270229907	0.931904324
Q5JRI3	3xPhospho [S129(100); S131(100); S133(100); Y136(0); Y138(0)]	1.026	0.037030731	0.994353955
Q5JSB5	1xPhospho [S23(100)]	1.34	0.422233001	0.848293321
Q5JSH3	2xPhospho [T464(0); S470(5.9); S471(94.5); S472(99.7); Y479(0); T480(0)]	1.22	0.286881148	0.906218259
Q5JSH3	3xPhospho [S456(0); T464(0); S470(100); S471(100); S472(100); Y479(0); T480(0)]	1	0	0.980895643
Q5JSH3	1xPhospho [Y395(0); S397(0); S403(100); S411(0); T414(0); T416(0)]	1.001	0.001441974	0.984563609
Q5JSH3	1xPhospho [T158(95); T160(2.4); S161(2.4); S162(0.1); T163(0.1); T171(0); T173(0)]	0.871	-0.199255376	0.999834587
Q5JSL0	2xPhospho [S20(100); S24(100); T31(0)]	0.757	-0.401634795	0.922988402
Q5JSL0	1xPhospho [S24(100); T31(0)]	1.081	0.112366523	0.960831064
Q5JSL0	2xPhospho [S20(100); S24(100); T31(0)]	0.784	-0.351074441	0.980895643
Q5JSZ5	3xPhospho [S1132(100); T1134(0); S1136(100); S1139(100); Y1141(0)]	0.01	-6.64385619	8.35E-17
Q5JSZ5	5 1xPhospho [S480(100)]; Q5JSZ5 1xPhospho [S480(100)]	2.068	1.048236186	0.461938204
Q5JSZ5	5 2xPhospho [S776(100); S786(0); S789(0); S790(0); S792(0); S795(99.9); Y797(0)]	0.875	-0.192645078	0.99184412
Q5JSZ5	5 1xPhospho [S388(100)]; Q5JSZ5 1xPhospho [S388(100)]	0.918	-0.123433941	0.99184412
Q5JTD0	1xPhospho [S545(100); T547(0)]	0.777	-0.364013496	0.969482098
Q5JTD0	1xPhospho [S300(100)]	0.787	-0.345564459	0.97726974
Q5JTH9	1xPhospho [S64(1.8); S66(98.2)]	0.01	-6.64385619	8.35E-17
Q5JTH9	1xPhospho [S1072(0); S1080(100)]	0.843	-0.246395464	0.966549726
Q5JTH9	1xPhospho [S72(100); T77(0); T88(0)]	0.972	-0.040971781	0.980895643
Q5JTV8	2xPhospho [S215(0); T220(0); S227(50); S228(50); S230(96.2); S231(3.5); T233(0.1); T234(0.1)]	0.645	-0.632628934	0.644556338
Q5JTV8	3xPhospho [S154(100); S156(100); S157(100); S163(0); S164(0); T166(0); S169(0); T171(0); S173(0)]	0.698	-0.518701058	0.750338864
Q5JTV8	3xPhospho [S154(100); S156(100); S157(100); S163(0); S164(0); T166(0); S169(0); T171(0); S173(0)]	0.79	-0.340075442	0.947600551
Q5JTV8	1xPhospho [S215(0); T220(100); S227(0); S228(0); S230(0); S231(0); T233(0); T234(0)]	0.866	-0.20756107	0.973763863
Q5JTV8	2xPhospho [S154(98); S156(51); S157(51); S163(0); S164(0); T166(0); S169(0); T171(0); S173(0)]	0.871	-0.199255376	0.984063817
Q5JTV8	2xPhospho [S154(0); S156(100); S157(100); S163(0); S164(0); T166(0); S169(0); T171(0); S173(0)]	0.983	-0.024736678	0.99277609
Q5JU85	1xPhospho [S212(3.2); S213(3.2); S214(93.7); S222(0); T223(0); S224(0); T225(0); S226(0); T227(0); S228(0); T231(0); T232(0)]	1.277	0.352758525	0.872730156
Q5JUE6	3xPhospho [S108(92.5); T110(15); S111(92.5); S116(7.5); S117(85); S120(7.5); T131(0)]	2.003	1.002162421	0.429315665
Q5PRF9	1xPhospho [T263(0); T264(0); S271(100); S274(0); S275(0); S278(0); S279(0); S281(0); T284(0); S289(0); S290(0)]	1.807	0.853596506	0.59656225
Q5PRF9	2xPhospho [T263(0); T264(0); S271(100); S274(99.9); S275(0.1); S278(0); S279(0); S281(0); T284(0); S289(0); S290(0)]	0.996	-0.005782353	0.979498769

Q5QP82	2xPhospho [S68(0); S79(0.2); S80(0.2); T81(0.1); S83(0.2); S89(99.6); S92(99.6)]	0.604	-0.727379545	0.576849086
Q5SQI0	1xPhospho [S315(100)]	0.01	-6.64385619	8.35E-17
Q5SRE5	1xPhospho [S1708(0); S1709(99.9); T1712(0.1); S1717(0)]	100	6.64385619	8.35E-17
Q5SRE5	2xPhospho [T1115(100); T1116(100); T1124(0); S1126(0)]	2.16	1.111031312	0.360736161
Q5SRE5	2xPhospho [S1708(0); S1709(100); T1712(0); S1717(100)]	0.774	-0.369594529	0.939053555
Q5SSJ5	3xPhospho [S431(0); S441(100); S442(100); S446(100)]	0.01	-6.64385619	8.35E-17
Q5SVL1	1xPhospho [S29(1.4); S30(98.6); S34(0); S37(0)]	0.637	-0.650634722	0.796314138
Q5SVL1	1xMet-loss+Acetyl [N-Term]; 1xPhospho [S16(100); S21(0)]	1.085	0.117695043	0.965139891
Q5SW79	1xPhospho [T564(0); T565(0); S566(0); S571(0); T575(0.2); S576(4); S577(91.6); S578(4); S580(0.2)]	2.164	1.113700499	0.534130879
Q5SW79	2xPhospho [T564(0); T565(0); S566(0); S571(98.1); T575(49.2); S576(49.2); S577(1.8); S578(1.8); S580(0.1)]	1.872	0.904580435	0.575076751
Q5SW79	2xPhospho [S252(100); T255(0.2); S257(4.1); T258(91.6); T264(4.1)]	1.639	0.712815854	0.671094422
Q5SW79	2xPhospho [S1521(50); S1522(50); S1529(100); T1533(0); T1535(0)]	1.422	0.507921465	0.840636242
Q5SW79	1xPhospho [S838(100); T840(0); S845(0)]	0.641	-0.641603738	0.879063032
Q5SW79	2xPhospho [S356(100); S359(100); Y364(0)]	0.783	-0.352915787	0.94813191
Q5SW79	1xPhospho [S1521(0); S1522(0); S1529(100); T1533(0); T1535(0)]	1.157	0.210388864	0.957850451
Q5SW79	1xPhospho [T1055(0); T1058(0); S1059(100); S1066(0)]	1.106	0.145351386	0.958702638
Q5SW79	2xPhospho [T350(0); S354(0); S356(100); S359(100); Y364(0)]	0.862	-0.214240226	0.962617576
Q5SW79	2xPhospho [T484(0); S485(0); T487(0); S488(0); S497(100); T501(100); Y502(0); T503(0); S511(0)]	1.084	0.116364757	0.974924677
Q5SW79	2xPhospho [S1160(100); S1162(0); S1165(100); S1167(0); T1170(0); S1172(0)]	0.876	-0.190997225	1
Q5SY20	2xPhospho [S169(0.1); S171(99.9); S179(100); S183(0); S187(0); S188(0)]	0.613	-0.706041021	0.786760931
Q5SY20	1xPhospho [S169(2.8); S171(97.2); S179(0); S183(0); S187(0); S188(0)]	1.322	0.402722177	0.872730156
Q5SYE7	1xPhospho [S1233(100); T1235(0); T1236(0); S1241(0); S1244(0)]	0.01	-6.64385619	8.35E-17
Q5SYE7	2xPhospho [S1386(100); S1388(100); T1392(0); T1394(0); S1399(0); S1402(0)]	0.709	-0.496142467	0.885630563
Q5SYE7	1xPhospho [S1250(0); S1251(0); S1257(0); T1260(0); T1264(0); S1265(0); S1274(3.7); S1276(3.7); S1278(92.7)]	1.207	0.271425676	0.908086456
Q5SZ07	1xPhospho [S123(100)]	100	6.64385619	8.35E-17
Q5SZ07	2xPhospho [S123(0); S148(100); S149(100)]	0.464	-1.10780329	0.379738971
Q5SZ07	1xPhospho [S181(100)]	1.696	0.76213617	0.604301121
Q5SZ07	1xPhospho [S181(100)]	1.571	0.651683181	0.694108746
Q5SZ07	2xPhospho [S215(5.1); T216(94.7); S218(0.3); S222(100); T241(0)]	0.564	-0.826232932	0.716990671
Q5SZ07	1xPhospho [S148(2.5); S149(97.5)]	1.437	0.523060062	0.821573905
Q5SZ07	2xPhospho [S148(100); S149(100)]	0.766	-0.384583703	0.870911172
Q5SZ07	1xPhospho [S181(100)]	1.245	0.316145742	0.908083211
Q5SZ07	2xPhospho [S148(100); S149(100)]	0.83	-0.268816758	0.926103516
Q5SZ07	1xPhospho [S181(100)]	1.191	0.252173413	0.956841977
Q5SZ07	1xPhospho [S215(0); T216(0); S218(0); S222(100); T241(0)]	0.831	-0.267079618	0.965139891
Q5SZ07	2xPhospho [S148(100); S149(100)]	1.026	0.037030731	0.993670231
Q5SZ59	2xPhospho [S100(100); S103(2.6); S104(97.3); S105(0.1); S108(0)]	0.885	-0.17625064	0.992959743
Q5T035	1xPhospho [T62(0); S66(100); Y67(0); S68(0)]	0.848	-0.23786383	0.96061834
Q5T0N5	2xPhospho [S488(96.3); S489(3.7); T496(0); S501(100); S505(0); Y506(0); T507(0)]	1.415	0.500802053	0.749797187
Q5T0N5	2xPhospho [S488(0); S489(0); T496(3.3); S501(96.7); S505(100); Y506(0); T507(0)]	0.811	-0.30222618	0.973763863
Q5T1M5	3xPhospho [T925(0); S939(100); S940(100); S941(100)]	0.01	-6.64385619	8.35E-17
Q5T1M5	3xPhospho [T925(0); S939(100); S940(100); S941(100)]	0.01	-6.64385619	8.35E-17
Q5T1M5	3xPhospho [S1092(9); S1093(1.3); T1094(1.3); S1097(90.3); T1099(90.8); S1100(7.4); S1114(100)]	0.01	-6.64385619	8.35E-17
Q5T1M5	2xPhospho [S1092(0.1); S1093(1.3); T1094(1.3); S1097(98.6); T1099(92.7); S1100(6.1); S1114(0)]	0.01	-6.64385619	8.35E-17
Q5T1M5	3xPhospho [S1097(100); T1099(95.3); S1100(4.8); S1114(100)]	0.32	-1.64385619	0.301623982
Q5T1M5	2xPhospho [S1161(0); S1162(100); S1164(100)]	0.616	-0.698997744	0.675336505
Q5T1M5	2xPhospho [S307(4); S311(96.1); S320(0); S326(99.8); T329(0.2); S330(0)]	1.057	0.079975377	0.965127389
Q5T1M5	2xPhospho [S295(0.2); S297(0.2); S301(2.2); S303(32.5); S304(32.5); S307(32.5); S311(100); S320(0); S326(0); T329(0); S330(0)]	0.985	-0.02180437	0.971196575
Q5T1M5	2xPhospho [S956(99.9); S960(0.1); S962(100); S964(0); S965(0)]	0.968	-0.046921047	0.988668305
Q5T1M5	1xPhospho [S956(100); S960(0); S962(0); S964(0); S965(0)]	0.997	-0.00433459	0.994109131
Q5T200	2xPhospho [S1452(4.8); S1455(95.3); Y1461(0); S1465(100)]	0.01	-6.64385619	8.35E-17
Q5T200	2xPhospho [S204(0); S207(100); S209(100); S211(0)]	1.949	0.962734091	0.296268685
Q5T200	1xPhospho [S198(100)]	2.531	1.339707507	0.369609069
Q5T200	1xPhospho [S77(100); T82(0)]	2.032	1.022900402	0.393207667
Q5T200	1xPhospho [T237(0); S238(0); S241(0); S242(100)]	2.153	1.10634832	0.481943049
Q5T200	1xPhospho [S943(100); T948(0); S949(0)]	1.645	0.718087584	0.724913856
Q5T200	3xPhospho [S370(100); S372(100); Y374(0); S376(0); S378(0); S380(2); S381(98)]	1.495	0.580145484	0.740489326
Q5T200	1xPhospho [S1404(0); T1405(2.4); S1406(2.4); S1409(95.3)]	1.448	0.534061602	0.823333961
Q5T200	2xPhospho [S204(0); S207(100); S209(0); S211(100)]	1.333	0.41467678	0.845666559
Q5T200	2xPhospho [T263(100); S265(100)]	0.701	-0.512513651	0.848293321
Q5T200	2xPhospho [T1062(0); S1065(100); S1068(100)]	1.364	0.447843644	0.854283637
Q5T200	2xPhospho [S370(100); S372(100); Y374(0); S376(0); S378(0); S380(0); S381(0)]	1.345	0.427606173	0.855534442
Q5T200	2xPhospho [S875(100); S877(100); S879(0); T882(0)]	1.298	0.376290383	0.879063032
Q5T200	2xPhospho [T263(100); S265(100)]	1.253	0.325386415	0.908602715
Q5T200	1xPhospho [S581(100); S585(0); S587(0); S588(0); S590(0); Y592(0); S595(0); T598(0)]	1.149	0.200378798	0.909920976
Q5T200	1xPhospho [S875(0); S877(100); S879(0); T882(0)]	1.262	0.33571191	0.920518605
Q5T200	2xPhospho [T263(100); S265(100)]	0.852	-0.231074664	0.971937853
Q5T200	1xPhospho [S943(100)]	1.135	0.182692298	0.978952227
Q5T200	2xPhospho [T984(0); T985(0); S986(100); S993(100)]	1.004	0.005759269	0.97998947
Q5T200	3xPhospho [T134(100); S137(99.9); T146(7.8); S151(92.3); Y158(0); Y160(0); S165(0)]	1.036	0.051024003	0.980895643

Q5T321	2xPhospho [S1711(100); S1714(100); T1716(0); S1720(0); T1722(0); T1727(0); S1728(0); S1730(0); S1731(0); S1733(0); T1735(0)]	0.01	-6.64385619	8.35E-17
Q5T321	2xPhospho [S1259(0.8); S1262(9.8); T1264(94.7); S1267(94.7); S1273(0)]	0.01	-6.64385619	8.35E-17
Q5T4P9	2xPhospho [S55(96); S56(8.1); S58(96); T64(0)]	0.591	-0.758769964	0.879989557
Q5T4S7	3xPhospho [T2715(0); S2718(0); S2719(100); S2722(100); T2724(100)]	0.059	-4.083141235	3.42E-07
Q5T4S7	2xPhospho [T360(0.1); S362(97.2); T363(4.1); S364(49.3); S365(49.3); Y370(0); S372(0); T376(0)]	0.358	-1.481968507	0.47171767
Q5T4S7	3xPhospho [T2715(0); S2718(0.2); S2719(99.8); S2722(100); T2724(100)]	0.53	-0.915935735	0.591305389
Q5T4S7	2xPhospho [T2884(0); S2885(0); S2892(100); S2895(99.8); S2897(0.2); S2900(0); S2904(0); S2910(0); S2912(0)]	0.799	-0.323732592	0.962617576
Q5T4S7	2xPhospho [T2715(0); S2718(0.1); S2719(99.9); S2722(50); T2724(50)]	0.851	-0.232768963	0.999834587
Q5T4S7	2xPhospho [T2715(100); S2718(50); S2719(50)]	0.899	-0.153606979	1
Q5T4U8	1xMet-loss+Acetyl [N-Term]; 1xPhospho [T3(100)]	1.167	0.222804561	0.922868578
Q5T593	2xPhospho [Y95(0); S96(0); S97(0); S101(0); T112(100); S114(100); S121(0)]	0.378	-1.40354186	0.303539347
Q5T5U3	2xPhospho [S1623(0); S1627(0); T1634(100); S1636(100); S1638(0); T1645(0); T1648(0); S1649(0)]	1.347	0.429749851	0.855113813
Q5T5Y3	1xPhospho [S862(100); S864(0); S872(0); S876(0)]	0.01	-6.64385619	8.35E-17
Q5T5Y3	2xPhospho [S738(100); S740(100); S762(0)]	10.535	3.397118409	2.30E-05
Q5T5Y3	1xPhospho [S431(100)]	1.529	0.612588407	0.703341962
Q5T5Y3	1xPhospho [T1396(0); S1398(0); S1400(99.9); S1401(0.1); S1403(0); S1406(0); T1409(0); T1410(0); S1414(0); S1417(0); T1420(0); S1422(0)]	1.58	0.659924558	0.76582364
Q5T5Y3	1xPhospho [S1080(100); T1082(0)]	0.658	-0.603840511	0.898138725
Q5T5Y3	1xPhospho [S575(100); T582(0); S583(0); S585(0); S589(0)]	1.014	0.020057652	0.980895643
Q5T5Y3	2xPhospho [T1396(0); S1398(100); S1400(100); S1401(0); S1403(0); S1406(0); T1409(0); T1410(0); S1414(0); S1417(0); T1420(0); S1422(0)]	1.053	0.074505436	0.980895643
Q5T624	2xPhospho [T96(0); S100(0); T111(100); S114(100); T124(0); S131(0)]	1.311	0.390667686	0.855557911
Q5T670	2xPhospho [S150(100); S154(100)]	0.383	-1.384583703	0.06771457
Q5T6C5	3xPhospho [S347(10.2); S350(95); S351(95); S353(99.7)]	1.552	0.634128558	0.716990671
Q5T757	1xPhospho [Y365(0); Y372(0); S374(100)]	2.554	1.352758525	0.092930052
Q5T757	1xPhospho [Y365(0); Y372(0); S374(100)]	1.937	0.953823954	0.434327749
Q5T757	1xPhospho [S147(100); S149(0)]	1.214	0.279768422	0.91281798
Q5T757	2xPhospho [S147(100); S149(3.3); S152(96.5); T154(0.1); S155(0.1); S156(0)]	0.922	-0.117161344	1
Q5T8E0	2xPhospho [S202(0); S203(0); S205(100); S209(100)]	1.198	0.260627908	0.922868578
Q5TAQ8	2xPhospho [S129(100); S130(100); S141(0); S142(0); T144(0); S145(0)]	0.01	-6.64385619	8.35E-17
Q5TAQ8	1xPhospho [S99(100)]	1.196	0.25821739	0.934211842
Q5TBQ1	1xPhospho [S154(0); T163(0); S165(100)]	1.152	0.204140717	0.9616322
Q5TC82	2xPhospho [S529(33.3); S530(33.3); S531(33.3); S535(100); S542(0)]	0.606	-0.722610301	0.879063032
Q5TCT4	1xPhospho [S14(0); S28(100); Y33(0)]	1.558	0.639695233	0.774332996
Q5TCT4	2xPhospho [S14(100); S28(100); Y33(0)]	1.42	0.50589093	0.816750637
Q5TCT4	1xPhospho [S90(0); T91(0); S92(0); S99(100)]	0.608	-0.717856771	0.855302203
Q5TCZ1	2xPhospho [S593(100); S596(0.1); S597(99.9)]	0.404	-1.307572802	0.290126505
Q5TDH0	2xPhospho [T115(0); S118(3.7); S120(46.2); S121(46.2); T126(0.4); S127(3.7); S128(99.9)]	8.979	3.16655478	9.37E-05
Q5TEC6	1xPhospho [Y42(0); T46(100)]; P68431 1xPhospho [Y42(0); T46(100)]; Q71DI3 1xPhospho [Y42(0); T46(100)]	1.895	0.922197848	0.499845416
Q5TEJ0	1xPhospho [S36(0); S41(0); S50(100); T58(0)]	100	6.64385619	8.35E-17
Q5TH69	2xPhospho [T1047(0); S1049(0); S1061(100); S1066(100)]	0.01	-6.64385619	8.35E-17
Q5THK1	1xPhospho [S1018(0); S1019(0); S1021(0); S1024(0); S1029(100)]	1.09	0.124328135	0.962617576
Q5U5Q3	3xPhospho [S537(100); S540(95.3); T541(95.3); S545(9.5); T547(0); S551(0)]	1.065	0.09085343	0.972326459
Q5UIP0	3xPhospho [S1542(100); S1547(0); S1548(0); S1552(100); S1554(100); S1556(0); S1557(0); S1564(0)]	0.01	-6.64385619	8.35E-17
Q5UIP0	1xPhospho [S1542(100); S1547(0); S1548(0); S1552(0); S1554(0); S1556(0); S1557(0)]	100	6.64385619	8.35E-17
Q5UIP0	1xPhospho [S1148(0); S1149(0); S1151(0); S1157(0); T1161(0); S1162(100); S1166(0); S1168(0)]	5.135	2.360364277	0.003679842
Q5UIP0	1xPhospho [S1008(100); T1013(0)]	1.355	0.438292852	0.79316576
Q5UIP0	1xPhospho [S2124(0); S2129(0); T2131(0); S2134(0); S2144(100)]	0.696	-0.522840789	0.798672039
Q5UIP0	1xPhospho [S1688(100); S1693(0); S1697(0); S1698(0)]	0.721	-0.471928835	0.870654365
Q5UIP0	1xPhospho [S2154(0); S2157(0); S2161(100); S2163(0); T2167(0)]	0.62	-0.689659879	0.897900043
Q5UIP0	1xPhospho [S782(100); S787(0); S790(0)]	1.095	0.13093087	0.957395303
Q5UIP0	2xPhospho [S1542(100); S1547(0); S1548(0); S1552(0); S1554(100); S1556(0); S1557(0); S1564(0)]	0.853	-0.229382353	0.973763863
Q5VSL9	1xPhospho [S335(100); S339(0); S341(0)]	1.067	0.093560176	0.972501767
Q5VT25	2xPhospho [S1719(0); S1721(100); S1724(99.9); T1725(0.1); S1729(0)]	0.01	-6.64385619	8.35E-17
Q5VT25	1xPhospho [S1664(0); S1666(0); S1669(0); S1671(100); S1672(0.1); S1679(0)]	0.01	-6.64385619	8.35E-17
Q5VT52	1xPhospho [S589(0); Y592(0); S593(100); S596(0); S597(0); T598(0); S599(0); S602(0); S603(0); T604(0); S605(0); S607(0)]	0.01	-6.64385619	8.35E-17
Q5VT52	1xPhospho [S1099(100); T1105(0); S1108(0)]	100	6.64385619	8.35E-17
Q5VT52	2xPhospho [S925(0.5); S928(99.2); S930(50.1); S932(50.1); S936(0); T939(0); S942(0); S946(0); S948(0); S950(0); T951(0); T952(0); S956(0)]	2.053	1.037733627	0.228459706
Q5VT52	2xPhospho [S758(0); S761(33.3); S762(33.3); T763(33.3); S765(95.7); S766(4); T767(0.2); S769(0.2)]	2.004	1.002882509	0.430840855
Q5VT52	1xPhospho [T470(0); S473(0); S476(0.1); S479(0.1); T482(0.1); T484(3.6); S485(92.5); S487(3.6); T490(0); S491(0)]	1.993	0.99494171	0.574566069
Q5VT52	1xPhospho [S374(100); S381(0)]	1.618	0.694211608	0.658258935
Q5VT52	3xPhospho [S925(91.4); S928(9); S930(49.8); S932(49.8); S936(7.9); T939(84.1); S942(7.9); S946(0.1); S948(0); S950(0); T951(0); T952(0); S956(0)]	1.079	0.109694865	0.965351295
Q5VT52	2xPhospho [T470(0); S473(0); S476(0.2); S479(99.6); T482(0.2); T484(0); S485(99.8); S487(0.2); T490(0); S491(0)]	0.947	-0.078563669	0.999608205
Q5VTL8	1xMet-loss+Acetyl [N-Term]; 1xPhospho [S5(0); T9(0.4); S12(99.7); T35(0); S40(0)]	0.523	-0.935117148	0.563451327

Q5VTR2	2xPhospho [S136(100); S138(100)]	0.47	-1.089267338	0.767186846
Q5VTR2	2xPhospho [S515(0); S517(0); S522(0.2); S524(96.2); S525(7.4); T526(96.3); S540(0); S544(0); S545(0); S547(0); S548(0); S550(0)]	0.756	-0.40354186	0.872730156
Q5VTR2	1xPhospho [S136(0); S138(100)]	0.785	-0.349235441	0.906786159
Q5VTR2	1xPhospho [S136(3); S138(97.1)]	0.642	-0.639354798	0.93019184
Q5VUA4	1xPhospho [S136(100); S141(0); S143(0)]	100	6.64385619	8.35E-17
Q5VUA4	2xPhospho [S2030(100); S2035(100); T2037(0)]	0.431	-1.214240226	0.690335172
Q5VUA4	1xPhospho [S709(100)]	1.561	0.642470537	0.744877142
Q5VUB5	1xPhospho [S723(100); Y724(0)]	0.01	-6.64385619	8.35E-17
Q5VUB5	1xPhospho [S356(4); T357(4); S358(87.9); S360(0); S367(4)]	0.01	-6.64385619	8.35E-17
Q5VUB5	2xPhospho [S356(0); T357(0); S358(100); S360(100); S367(0)]	0.376	-1.411195433	0.310317459
Q5VUB5	2xPhospho [S356(0); T357(0); S358(100); S360(100); S367(0)]	0.412	-1.279283757	0.567775792
Q5VUB5	2xPhospho [S356(33.3); T357(33.3); S358(33.3); S360(100); S367(0)]	0.439	-1.187707155	0.661025253
Q5VV41	1xPhospho [S208(100); Y216(0)]	100	6.64385619	8.35E-17
Q5VV41	1xPhospho [T226(0); S227(0); S230(100); S238(0); S239(0); S240(0); T244(0)]	2.549	1.349931373	0.248861391
Q5VV41	2xPhospho [T226(96.1); S227(96.1); S230(7.9); S238(0); S239(0); S240(0); T244(0)]	0.421	-1.248107862	0.455589875
Q5VV41	1xPhospho [S107(100); S114(0)]	1.046	0.064882852	0.975403456
Q5VVJ2	1xPhospho [S218(100); T226(0); S233(0); S234(0); T236(0)]	100	6.64385619	8.35E-17
Q5VVR8	1xPhospho [S46(0); S47(0); S52(0); T54(0); S58(0); S59(0); S60(100); S62(0)]	100	6.64385619	8.35E-17
Q5VVR8	2xPhospho [S177(100); S179(100); S185(0)]	1.039	0.055195654	0.980895643
Q5VWG9	2xPhospho [S229(99.3); S236(0.3); T237(0.3); S243(100)]	0.955	-0.066427362	0.995622978
Q5VWN6	1xPhospho [T1014(0); T1016(0); S1025(100); S1034(0)]	0.72	-0.473931188	0.938730361
Q5VWQ0	1xPhospho [S104(100)]	1.015	0.021479727	0.977837438
Q5VWV2	2xPhospho [S658(50); S660(50); Y662(0); S663(0); S671(96.9); S673(3.1)]	0.01	-6.64385619	8.35E-17
Q5VWV2	2xPhospho [S143(97.4); S144(2.6); S152(0); T153(0); S154(0); S156(99.9); S158(0.1); S161(0.1); S162(0); S166(0)]	0.511	-0.968604804	0.650553284
Q5VWV2	1xPhospho [S143(97.7); S144(2.3); S152(0); T153(0); S154(0); S156(0); S158(0); S161(0); S162(0); S166(0)]	1.567	0.64800518	0.678654866
Q5VWV2	2xPhospho [S898(100); S902(0); S904(0); T905(0); S907(0); S911(50); S913(50)]	0.59	-0.76121314	0.816388446
Q5VWV2	1xPhospho [S792(100); T801(0)]	1.398	0.483364361	0.855031791
Q5VWV2	1xPhospho [S898(0.1); S902(99.8); S904(0.1); T905(0.1); S907(0); S911(0); S913(0)]	1.351	0.434027675	0.870080472
Q5VX15	1xPhospho [S441(100); T443(0); S447(0); T451(0)]	1.268	0.342554745	0.932557671
Q5VZ89	2 2xPhospho [S569(50); S570(50); S574(99.6); S577(0.5); S581(0); S583(0)]; Q5VZ89 2xPhospho [S1096(50); S1097(50); S1101(99.6); S1104(0.5); S1108(0); S1110(0)]	0.01	-6.64385619	8.35E-17
Q5VZ89	2 1xPhospho [S144(100); S149(0); S150(0); T151(0); S155(0); Y159(0); S161(0)]	100	6.64385619	8.35E-17
Q5VZ89	3xPhospho [S763(100); S764(96.1); T765(3.9); S767(100); S769(0); S774(0); T775(0); T788(0)]	0.253	-1.98279071	0.053738706
Q5VZ89	2 2xPhospho [S514(2.6); S515(97.4); S522(0); S530(0.1); S533(97.3); S534(2.6); S538(0)]; Q5VZ89 2xPhospho [S1041(2.6); S1042(97.4); S1049(0); S1057(0.1); S1060(97.3); S1061(2.6); S1065(0)]	0.526	-0.926865295	0.54831873
Q5VZ89	2 2xPhospho [S875(7.3); S877(92.7); T888(0); S896(0.1); T897(0.1); S899(99.9); S903(0)]; Q5VZ89 2xPhospho [S1402(7.3); S1404(92.7); T1415(0); S1423(0.1); T1424(0.1); S1426(99.9); S1430(0)]	0.734	-0.446148032	0.908602715
Q5VZ89	2 2xPhospho [S205(0); S208(100); S213(100); T215(0); S222(0); S223(0)]; Q5VZ89 2xPhospho [S729(0); S732(100); S737(100); T739(0); S746(0); S747(0)]	0.791	-0.3382504	0.938029982
Q5VZ89	2 1xPhospho [S514(2.1); S515(98); S522(0); S530(0)]; Q5VZ89 1xPhospho [S1041(2.1); S1042(98); S1049(0); S1057(0)]	0.641	-0.641603738	0.93864152
Q5VZK9	1xPhospho [S1331(100); Y1339(0)]	0.01	-6.64385619	8.35E-17
Q5VZK9	2xPhospho [T1278(0); S1280(0); S1288(100); S1290(97.2); S1291(2.8)]	0.962	-0.055891201	0.989898705
Q5VZK9	1xPhospho [S1357(0); S1360(100)]	0.962	-0.055891201	0.99277609
Q5VZL5	2xPhospho [S103(0); S104(0); T107(100); S110(100)]	0.533	-0.907792562	0.636224002
Q5VZL5	1xPhospho [T118(0); S122(100)]	1.543	0.625738062	0.746950683
Q5VZL5	1xPhospho [T118(0); S122(100)]	1.376	0.46048047	0.783976635
Q5VZL5	1xPhospho [S1181(100)]	1.196	0.25821739	0.939695119
Q5VZL5	1xPhospho [S1181(100)]	1.127	0.172487516	0.953387553
Q5VZS7	1xPhospho [S57(0); S62(100); S64(0.1); S66(0); S68(0)]	100	6.64385619	8.35E-17
Q5W0B1	2xPhospho [S434(47.9); S435(47.9); S438(8.2); S442(4.7); S443(91.3); S448(0.1)]	0.01	-6.64385619	8.35E-17
Q5W0B1	1xPhospho [S539(0); S543(0); S546(0); S548(0.1); S551(0); S553(99.9)]	0.755	-0.40545145	0.963305044
Q5W0W1	1xPhospho [S323(0); Y324(0); S325(100); S330(0); S336(0)]	1.45	0.5360529	0.764000554
Q63ZY3	1xPhospho [S375(100)]	0.82	-0.286304185	0.978877443
Q658Y7	1xPhospho [S200(100)]	2.634	1.397255346	0.551311165
Q658Y7	1xPhospho [T502(0); T509(0); Y511(0); S513(100); S519(0); S523(0); Y524(0)]	0.602	-0.732164608	0.810156821
Q658Y7	2xPhospho [T165(0); T166(0); T167(0); T169(0); T171(0); S178(100); S181(99.9); S185(0.1)]	0.738	-0.438307279	0.906261639
Q658Y7	2xPhospho [T165(0); T166(0); T167(0); T169(0); T171(0); S178(100); S181(100); S185(0)]	0.866	-0.20756107	0.952591019
Q66GS9	2xPhospho [T1121(0); S1125(0); S1126(0.1); T1127(3); S1130(96.8); S1132(0.1); S1134(100)]	3.311	1.727267011	0.072706211
Q66GS9	3xPhospho [T1121(100); S1125(50); S1126(50); T1127(0.2); S1130(0.2); S1132(3.8); S1134(95.9)]	1.241	0.311503115	0.870911172
Q66K14	2xPhospho [S411(100); S416(0); T417(0); S419(0); S420(0); S427(0); S432(0); S435(100); S438(0); S439(0)]	0.712	-0.490050854	0.886912356
Q66PJ3	1xPhospho [S271(100); T282(0)]	1.244	0.314986485	0.920957334
Q68CQ4	1xPhospho [S8(0); S10(100); T15(0); T17(0)]	0.689	-0.537424112	0.865762755
Q68CQ4	2xPhospho [S6(100); S8(100); S10(0); T15(0); T17(0)]	0.673	-0.57132159	0.945786032
Q68CZ2	1xPhospho [S649(0); T656(0); S660(97.2); S662(2.8)]	2.237	1.161565256	0.35260057
Q68CZ2	2xPhospho [S361(33.3); S362(33.3); S363(33.3); T377(7.6); S379(91.1); S383(0.1); T386(0.7); S388(0.7); S390(0); S391(0); S393(0); S396(0); T397(0); S399(0)]	1.542	0.624802765	0.754711916
Q68D10	2xPhospho [S317(0); S318(0); T319(0); S320(95.7); S321(95.7); S323(8.5)]	0.495	-1.01449957	0.770935417
Q68D10	1xPhospho [S470(0); S471(100)]	0.513	-0.962969269	0.78580526
Q68DB1	1xPhospho [S254(0); S260(0); S262(100); Y264(0); T265(0); T266(0); S267(0)]	1.014	0.020057652	0.974924677

Q68DK2	2xPhospho [S609(0); S611(50); S615(50); S617(0); S619(100)]	3.082	1.623866862	0.108982093
Q68E01	1xPhospho [S514(0); S522(0); S527(0); Y529(0); S537(100); S545(0)]	1.109	0.149259365	0.969680228
Q68EM7	2xPhospho [S595(100); S599(0); S610(0); S614(0); S625(99.7); T628(0.3)]	0.01	-6.64385619	8.35E-17
Q68EM7	1xPhospho [S808(0); S820(100); S824(0); S825(0); T827(0); T829(0); T832(0); S834(0)]	100	6.64385619	8.35E-17
Q68EM7	1xPhospho [S595(0); S599(0); S610(0); S614(0); S625(95.2); T628(4.8)]	0.363	-1.461958547	0.051932716
Q68EM7	1xPhospho [S551(99.5); S552(0.5); S553(0); T557(0); S560(0); S561(0); S570(0); S575(0)]	0.358	-1.481968507	0.341780279
Q68EM7	1xPhospho [S850(0); S857(0); S859(2.7); S861(97.3)]	1.367	0.451013243	0.860867908
Q68EM7	1xPhospho [S484(100)]	1.29	0.367371066	0.891708578
Q68EM7	2xPhospho [S551(99.9); S552(0.1); S553(0); T557(0); S560(0); S561(0); S570(0); S575(100)]	1.149	0.200378798	0.949541112
Q68EM7	1xPhospho [S484(100)]	1.139	0.187767747	0.954054893
Q68EM7	2xPhospho [S674(48.7); S676(48.7); T679(1.3); T682(1.3); S690(0); S693(100); S696(0)]	0.965	-0.051399153	0.999263007
Q69YN4	1xPhospho [S133(98); S138(2.1); S154(0)]	0.01	-6.64385619	8.35E-17
Q69YN4	2xPhospho [T184(100); S204(0); Y217(0); S222(100)]	3.87	1.952333566	0.018743399
Q69YN4	1xPhospho [S173(100)]	2.135	1.09423607	0.276198761
Q69YN4	1xPhospho [S1572(0); S1575(0); S1578(0); S1579(100)]	1.642	0.715454127	0.730212284
Q69YN4	2xPhospho [S133(50); S138(50); S154(100)]	0.947	-0.078563669	0.981858798
Q6AI08	2xPhospho [S336(100); S337(100); T346(0); T348(0)]	0.071	-3.816037165	2.64E-06
Q6AI08	1xPhospho [S635(0); T639(2.4); S640(2.4); S642(2.4); S643(92.7)]	0.584	-0.775959726	0.872730156
Q6AI08	1xPhospho [T297(0); S304(100); S306(0); S307(0); S309(0); T312(0)]	1.21	0.275007047	0.921780519
Q6AI39	1xPhospho [S674(3.9); S675(96.1); S685(0); S687(0)]	100	6.64385619	8.35E-17
Q6DCA0	2xPhospho [S27(0); S29(0); T31(0); S33(0); S38(0); T39(0); T40(0); S44(51.1); S45(51.1); S46(97.7); S57(0.2); S58(0); S59(0)]	0.01	-6.64385619	8.35E-17
Q6DN90	1xPhospho [S512(100); S515(0); S519(0)]	0.715	-0.483984853	0.94813191
Q6DT37	1xPhospho [S1475(0); S1482(100); S1484(0)]	1.325	0.40599236	0.85992414
Q6F5E8	1xPhospho [S1315(100); S1319(0); S1321(0)]	0.556	-0.846843212	0.873058511
Q6F5E8	1xPhospho [S1246(100); S1253(0); S1254(0); T1255(0); S1261(0)]	1.092	0.126972856	0.938029982
Q6F5E8	1xPhospho [S991(97.6); S993(2.5)]	1.057	0.079975377	0.971220368
Q6IN85	3xPhospho [T754(0); T757(0); S758(0); S760(0); S761(0); T762(0); T763(0); S768(0); S771(100); S774(100); S777(99.7); S780(0.3); S783(0)]	100	6.64385619	8.35E-17
Q6IN85	2xPhospho [T754(0); T757(0); S758(0); S760(0); S761(0); T762(0); T763(0); S768(0); S771(0.2); S774(99.8); S777(0.2); S780(99.7); S783(0.2)]	0.739	-0.436353731	0.907609406
Q6IQ26	2xPhospho [T1067(0); S1068(0); T1079(100); S1085(50); S1087(50)]	0.885	-0.17625064	0.992863986
Q6KC79	1xPhospho [T2663(0); T2667(0); S2672(100); T2681(0); S2682(0); S2684(0)]	100	6.64385619	8.35E-17
Q6KC79	2 1xPhospho [Y345(0); S349(0); S350(98.1); S352(1.8)]; Q6KC79 1xPhospho [Y345(0); S349(0); S350(98.1); S352(1.8)]	100	6.64385619	8.35E-17
Q6KC79	2 1xPhospho [T2663(0); T2667(0); S2672(99.9); T2681(0); S2682(0.1)]	2.613	1.385707125	0.238410909
Q6KC79	2 2xPhospho [S269(0); S274(100); S280(100); S284(0); T287(0)]; Q6KC79 2xPhospho [S269(0); S274(100); S280(100); S284(0); T287(0)]	0.44	-1.184424571	0.5253891
Q6KC79	2 2xPhospho [S553(0); S556(1.3); T558(8.3); S561(91.7); S563(98.7)]; Q6KC79 2xPhospho [S553(0); S556(1.3); T558(8.3); S561(91.7); S563(98.7)]	0.744	-0.426625474	0.969482098
Q6KC79	2 1xPhospho [T2651(0); S2652(0); S2658(100)]; Q6KC79 1xPhospho [T2651(0); S2652(0); S2658(100)]	1.033	0.046840254	0.980895643
Q6MZP7	2xPhospho [S310(100); S314(100)]	1.121	0.164786278	0.976789257
Q6NUK4	2xPhospho [S180(0); S185(0); S187(0); Y190(0); T201(100); Y209(0); S210(99.3); T216(0.7)]	0.01	-6.64385619	8.35E-17
Q6NUK4	1xPhospho [S150(94.8); S152(5.2); T157(0); T158(0); Y170(0)]	100	6.64385619	8.35E-17
Q6NUK4	2xPhospho [S180(0); S185(0); S187(0); Y190(0); T201(100); Y209(0); S210(99.9); T216(0.1)]	0.244	-2.035046947	0.00233357
Q6NUK4	2xPhospho [T201(100); Y209(0); S210(100); T216(0)]	0.312	-1.680382066	0.153691246
Q6NWY9	1xPhospho [S764(100); S766(0); S767(0)]	0.565	-0.823677227	0.781247135
Q6NZI2	2xPhospho [S202(100); S203(100); S216(0)]	0.694	-0.526992432	0.748662687
Q6NZI2	2xPhospho [S175(0); S202(100); S203(100); S216(0)]	0.704	-0.506352666	0.759239289
Q6NZI2	2xPhospho [S365(100); S366(100); T376(0); S379(0)]	0.639	-0.646112164	0.886312012
Q6NZI2	3xPhospho [S365(50); S366(50); T376(0); S379(0); S387(100); S389(100)]	0.685	-0.545824107	0.908083211
Q6P0N0	2xPhospho [T810(0); S811(0); T821(100); S824(100); Y829(0)]	0.784	-0.351074441	0.96293109
Q6P158	2xPhospho [S127(100); S132(100)]	9.944	3.313826296	9.22E-06
Q6P158	1xPhospho [S132(100)]	1.73	0.790772038	0.663136789
Q6P1L5	3xPhospho [S136(100); T149(0); S151(4.7); S152(95.3); S154(91.1); S155(4.5); S156(4.5); T158(0); T162(0); S166(0)]	0.42	-1.251538767	0.292990089
Q6P275	2xPhospho [S1126(0); S1138(100); S1142(100)]	0.066	-3.921390165	0.000276689
Q6P2E9	1xPhospho [T702(0); T704(0); S705(0); S708(0); S723(0.1); S725(99.9)]	0.01	-6.64385619	8.35E-17
Q6P2E9	2xPhospho [T702(0); T704(0); S705(0); S708(100); S723(50); S725(50)]	100	6.64385619	8.35E-17
Q6P2E9	3xPhospho [T702(0); T704(51.1); S705(51.1); S708(97.9); S723(4.2); S725(95.8)]	0.287	-1.800877358	0.25780477
Q6P2E9	1xPhospho [T727(2.4); S729(97.6); S734(0); S735(0); S737(0); T738(0); S741(0); S749(0); S753(0)]	0.797	-0.327348371	0.952591019
Q6P2E9	1xPhospho [S871(0); S875(0); S879(100); S887(0); S890(0); S892(0); T897(0)]	1.032	0.045442971	0.983706057
Q6P2E9	2xPhospho [S871(0); S875(0); S879(100); S887(3.1); S890(3.1); S892(93.8); T897(0)]	0.984	-0.023269779	0.987542027
Q6P2E9	2xPhospho [T727(0.1); S729(99.9); S734(0); S735(0); S737(0); T738(0); S741(0); S749(0); S753(100)]	0.924	-0.114035243	1
Q6P3S6	2xPhospho [T534(48.6); T539(48.6); S545(2.9); S552(100)]	0.846	-0.241270432	1
Q6P4E1	1xPhospho [S232(0.1); S233(99.9)]	0.516	-0.954557029	0.783976635
Q6P597	2xPhospho [Y156(0); S162(3.8); S165(50); S167(50); S173(95.8); S176(0.5); S180(0)]	0.538	-0.894321922	0.743519332
Q6P597	1xPhospho [S466(100); T469(0)]	0.827	-0.274040765	0.966027585
Q6P6C2	1xPhospho [Y60(0); S64(100); S69(0); Y71(0)]	1.914	0.93659083	0.575551715
Q6P6C2	1xPhospho [S350(100); T355(0)]	1.81	0.855989697	0.614078599
Q6P6C2	2xPhospho [Y60(0); S64(100); S69(100); Y71(0)]	1.277	0.352758525	0.884551994
Q6P6C2	1xPhospho [S312(100)]	1.215	0.280956314	0.891020458

Q6P6C2	2xPhospho [Y60(0); S64(100); S69(100); Y71(0)]	1.016	0.022900402	0.980895643
Q6P6C2	2xPhospho [Y60(0); S64(100); S69(100); Y71(0)]	1.074	0.102993993	0.980895643
Q6PCB5	2xPhospho [S102(0); S103(1.1); S105(33); S107(33); T110(33); S114(0); S115(0); S117(0); S119(0); S121(100)]	0.748	-0.418889825	0.971196575
Q6PCE3	1xPhospho [T173(0.1); S175(99.9)]	1	0	1
Q6PD62	1xPhospho [T925(100); S932(0)]	0.608	-0.717856771	0.81289358
Q6PD62	1xPhospho [T925(100); S932(0)]	1.002	0.002882509	0.980895643
Q6PJF5	2xPhospho [S323(0); S325(100); S328(100)]	0.426	-1.231074664	0.350862969
Q6PJF5	3xPhospho [S323(100); S325(100); S328(100)]	0.369	-1.438307279	0.495170209
Q6PJG2	3xPhospho [T698(0.1); S700(99.9); T704(100); S709(0); T715(100); S718(0)]	0.082	-3.60823228	0.000118516
Q6PJG2	3xPhospho [T698(50); S700(50); T704(100); S709(0); T715(96.9); S718(3.1)]	0.733	-0.448114897	0.972363997
Q6PJG6	1xPhospho [S742(100); T745(0); S747(0); T751(0)]	1.526	0.609754962	0.759239289
Q6PKG0	2xPhospho [T622(0); T624(0); S627(100); S631(100); Y633(0)]	100	6.64385619	8.35E-17
Q6PKG0	2xPhospho [S766(100); T769(0); T770(0); S774(100); Y777(0)]	0.557	-0.844250767	0.402718746
Q6PKG0	2xPhospho [T622(0); T624(0); S627(100); S631(100); Y633(0)]	0.722	-0.469929258	0.836554173
Q6PKG0	2xPhospho [T622(0); T624(0); S627(100); S631(100); Y633(0)]	0.736	-0.442222329	0.855113813
Q6PKG0	2xPhospho [S75(100); S90(100)]	0.735	-0.444183845	0.855557911
Q6PKG0	2xPhospho [T782(50); T785(50); T788(100)]	0.704	-0.506352666	0.872730156
Q6PKG0	2xPhospho [T622(0); T624(0); S627(100); S631(100); Y633(0)]	1.292	0.36960607	0.884551994
Q6PKG0	2xPhospho [T845(4.1); S847(95.9); S849(48.9); S850(48.9); S851(2.1); S853(0); T856(0); T858(0); S861(0); Y862(0); T865(0); S868(0)]	0.748	-0.418889825	0.905689075
Q6PKG0	1xPhospho [T137(0); T138(0); S143(100); S148(0)]	0.846	-0.241270432	0.950270408
Q6PKG0	1xPhospho [S546(0.1); S548(100); S554(0)]	1.062	0.086783766	0.969680228
Q6PKG0	1xPhospho [S580(0); T583(0); S584(0); S591(99.9); S596(0.1)]	0.754	-0.407363571	0.972363997
Q6PKG0	1xPhospho [S75(0); S90(100)]	0.907	-0.140825544	0.974924677
Q6PKG0	1xPhospho [T647(0); T649(100); Y652(0)]	0.974	-0.038006323	0.974924677
Q6PKG0	1xPhospho [S766(0); T769(0); T770(0); S774(100); Y777(0)]	1.003	0.004321606	1
Q6PL18	1xPhospho [S1243(100); T1245(0); S1255(0)]	0.417	-1.261880711	0.659307027
Q6QNY0	2xPhospho [T63(100); S65(100); T74(0)]	0.01	-6.64385619	8.35E-17
Q6R327	2xPhospho [S1385(100); Y1386(0); S1388(100); S1396(0); T1402(0)]	0.692	-0.531156057	0.855545836
Q6R327	1xPhospho [S21(100); T31(0)]	0.676	-0.564904848	0.85992414
Q6R327	2xPhospho [T1172(0); S1174(100); T1175(0); S1177(100)]	0.733	-0.448114897	0.907632224
Q6R327	1xPhospho [S21(100); T31(0)]	0.776	-0.365871442	0.954539739
Q6UB99	2xPhospho [S1983(100); S1990(100)]	1.58	0.659924558	0.768341527
Q6ULP2	2xPhospho [S372(0); T375(0.1); S376(99.9); S382(100); S387(0)]	0.822	-0.282789701	0.986075329
Q6UUV7	2xPhospho [S391(100); T394(0); S396(100); S406(0)]	0.447	-1.161653263	0.724983097
Q6UUV7	2xPhospho [S391(100); T394(0.2); S396(99.8); S406(0)]	1.183	0.242450074	0.94797789
Q6UUV7	1xPhospho [S329(100); S332(0); T336(0)]	1.046	0.064882852	0.975403456
Q6UUV7	1xPhospho [S368(0); S370(100); S373(0); S375(0); T376(0); T377(0); S380(0); S383(0)]	0.95	-0.074000581	0.980895643
Q6UUV7	1xPhospho [T56(0); Y58(0); S62(100); S67(0)]	1.009	0.012926174	0.980895643
Q6UUV9	2xPhospho [T149(96.3); S151(3.7); S153(0); S158(0); T159(0); T161(0); T163(0); S167(0); S169(0); S170(0); S172(100)]	0.8	-0.321928095	0.972363997
Q6UUV9	1xPhospho [T149(4); S151(96); S153(0); S158(0); T159(0); T161(0); T163(0); S167(0); S169(0); S170(0); S172(0)]	0.977	-0.033569533	0.975607937
Q6VY07	2xPhospho [T526(0); S528(0.1); S529(97.6); S531(4.8); S534(97.6); S540(0); T541(0)]	0.75	-0.415037499	0.906261639
Q6VY07	1xPhospho [S407(0); S409(0); S410(0); S411(100); T413(0); S417(0); S420(0)]	0.76	-0.395928676	0.952675081
Q6WCQ1	2xPhospho [T210(0); S217(0); S218(2.5); S220(97.5); S224(100); S226(0); S228(0); S234(0); S235(0)]	0.429	-1.220950447	0.324232304
Q6WCQ1	1xPhospho [S991(0); S993(100)]	0.539	-0.891642822	0.759239289
Q6WCQ1	2xPhospho [S289(100); S292(2.4); S294(95.1); T295(2.4)]	0.704	-0.506352666	0.837895353
Q6WCQ1	1xPhospho [S991(0); S993(100)]	0.765	-0.386468347	0.980895643
Q6XZF7	2xPhospho [T341(3.5); S342(96.5); S354(0); T358(0.1); S359(99.9); T365(0); S366(0); Y368(0); T370(0)]	0.571	-0.808437349	0.548348393
Q6Y7W6	3xPhospho [S335(100); S337(100); S340(100)]	0.223	-2.164884385	0.024654093
Q6Y7W6	2xPhospho [S19(0); S20(0); S23(0); T25(0.1); S26(100); S30(100)]	0.33	-1.59946207	0.031703264
Q6Y7W6	3xPhospho [S335(100); S337(100); S340(100)]	0.287	-1.800877358	0.117268654
Q6Y7W6	3xPhospho [S335(100); S337(100); S340(100)]	0.446	-1.164884385	0.502522351
Q6Y7W6	1xPhospho [T188(1.7); S189(98.3)]	1.873	0.9053509	0.569260694
Q6Y7W6	1xPhospho [S236(100)]	1.693	0.759581973	0.636224002
Q6Y7W6	3xPhospho [S335(100); S337(100); S340(98.6); T351(1.4)]	0.676	-0.564904848	0.863668848
Q6Y7W6	1xPhospho [S367(0); T370(0); T373(0); S374(0); S375(0); S376(0); S377(0.1); T382(99.9); S384(0); S388(0); S392(0)]	1.223	0.290424404	0.903986081
Q6Y7W6	1xPhospho [S335(99.6); S337(0.4); S340(0)]	1.213	0.27857955	0.927625482
Q6Y7W6	1xPhospho [S19(0); S20(0); S23(0); T25(0.1); S26(100); S30(0)]	0.898	-0.15521265	0.990299313
Q6ZN18	1xPhospho [S206(100); S210(0); S211(0); S217(0)]	2.006	1.004321606	0.459641806
Q6ZN18	2xPhospho [S206(100); S210(50); S211(50); S217(0)]	1.179	0.237563718	0.956843841
Q6ZN18	1xPhospho [S390(100); T402(0)]	0.74	-0.434402824	0.971196575
Q6ZN55	2xPhospho [T152(3); T154(97); S164(100)]	1.382	0.466757616	0.840824379
Q6ZN55	1xPhospho [S298(100); T306(0); S312(0); S320(0)]	0.78	-0.358453971	0.930376591
Q6ZNB6	2xPhospho [S41(100); S47(0); T49(0); S50(0); T56(0.4); T57(6); S62(93.6)]	0.661	-0.597277823	0.90624343
Q6ZNB6	1xPhospho [S41(0); S47(0); T49(0); S50(0); T56(0); T57(0); S62(100)]	1.081	0.112366523	0.96229367
Q6ZNB6	1xPhospho [S65(97.9); S69(2.1); T74(0); T75(0); S78(0); S82(0)]	1.053	0.074505436	0.967238018
Q6ZNB6	1xPhospho [S41(100); S47(0); T49(0); S50(0); T56(0); T57(0); S62(0)]	0.776	-0.365871442	0.980895643
Q6ZRP7	1xPhospho [T570(0); Y571(0); S572(0); S578(98.5); S579(1.5); T583(0)]	0.657	-0.606034724	0.797921038

Q6ZRS2	1xPhospho [S1855(0); S1859(99.8); S1862(0); T1863(0); T1865(0); S1866(0.1)]	1.148	0.199122642	0.929414017
Q6ZRV2	1xPhospho [S998(100)]	0.568	-0.816037165	0.842978097
Q6ZRV2	3xPhospho [S5(100); S7(100); S8(50); S9(50); Y19(0); Y24(0)]	1.143	0.192825404	0.957850451
Q6ZRV2	2xPhospho [Y510(0); S513(100); S514(50); S516(50)]	0.798	-0.325539348	0.971196575
Q6ZRV2	1xPhospho [S892(100); T894(0); T895(0)]	0.807	-0.309359421	0.973599548
Q6ZRV2	3xPhospho [S5(4.7); S7(95.7); S8(99.8); S9(99.8); Y19(0); Y24(0)]	0.88	-0.184424571	0.97998947
Q6ZSJ8	2xPhospho [S82(100); S94(100)]	5.405	2.434294618	0.001955195
Q6ZSJ8	1xPhospho [S82(100); S94(0)]	0.851	-0.232768963	0.99184412
Q6ZSZ5	1xPhospho [S1101(0); S1103(100)]	0.84	-0.251538767	0.980895643
Q6ZTN6	2xPhospho [S465(100); T469(100); S477(0)]	0.759	-0.397828209	0.922868578
Q6ZW31	3xPhospho [Y229(0); S231(100); S235(100); S244(100); T246(0); S247(0); Y251(0)]	0.579	-0.788364747	0.87431027
Q6ZWJ1	2xPhospho [S460(97.4); T462(51.1); S463(51.2); T465(0.3)]	0.429	-1.220950447	0.652403988
Q70Z53	3xPhospho [S270(0); S273(0); S278(100); S283(98.6); S285(50.7); S287(50.7)]	0.672	-0.573466862	0.862506613
Q70Z53	2xPhospho [S270(0); S273(0); S278(100); S283(100); S285(0); S287(0)]	0.808	-0.307572802	0.956843841
Q70Z53	2xPhospho [S270(0); S273(0); S278(100); S283(100); S285(0); S287(0)]	0.8	-0.321928095	0.973599548
Q71RC2	2xPhospho [S643(51.7); S647(51.7); T649(96.6); S660(0)]	2.077	1.054501216	0.396443896
Q71RC2	1xPhospho [Y717(0); S722(100)]	2.117	1.082021269	0.534130879
Q71RC2	1xPhospho [S381(0); S382(0); S385(2.7); S388(0); T389(0); S392(97.3); S394(0)]	1.617	0.693319679	0.71389078
Q71RC2	1xPhospho [S594(1.5); T595(98.5); S597(0); T607(0)]	0.783	-0.352915787	0.879063032
Q71RC2	1xPhospho [T578(0); T579(0); S583(100); S586(0); T587(0); T588(0); S591(0)]	0.779	-0.360304767	0.912232379
Q71RC2	2xPhospho [S643(100); S647(99.8); T649(0.2); S660(0)]	1.067	0.093560176	0.956843841
Q71RC2	2xPhospho [T578(0); T579(0); S583(100); S586(33.1); T587(33.1); T588(33.1); S591(0.8)]	0.983	-0.024736678	0.980895643
Q71RC2	1xPhospho [S643(0); S647(97); T649(3); S660(0)]	1.023	0.032806145	0.989486735
Q71UM5	1xPhospho [T74(0); S78(100)]	1.22	0.286881148	0.911330706
Q76FK4	1xPhospho [S1098(0); S1099(100); S1104(0)]	1.916	0.938097561	0.469512534
Q76FK4	2xPhospho [Y376(0); S378(100); T381(100)]	1.083	0.115033243	0.962617576
Q76FK4	2xPhospho [T888(100); S890(100)]	0.805	-0.312939312	0.973763863
Q76FK4	2xPhospho [Y376(0); S378(100); T381(100)]	1.082	0.113700499	0.976345951
Q76L83	1xPhospho [S530(100); T541(0); T549(0)]	0.866	-0.20756107	0.987464758
Q7KZ85	3xPhospho [S73(100); S75(100); S78(100)]	0.01	-6.64385619	8.35E-17
Q7KZ85	1xPhospho [S125(100); Y135(0)]	0.01	-6.64385619	8.35E-17
Q7KZ85	1xPhospho [S125(100); Y135(0)]	1.549	0.631337144	0.716519243
Q7KZ85	1xPhospho [S125(100); Y135(0)]	1.534	0.617298483	0.787236772
Q7KZ85	2xPhospho [Y1515(0); T1523(100); S1525(0.9); S1526(33); S1527(33); S1528(33)]	1.158	0.211635253	0.922395265
Q7KZ85	2xPhospho [T1530(1.9); T1532(98.1); S1535(100); T1539(0); T1549(0)]	1.152	0.204140717	0.963156927
Q7KZ85	3xPhospho [T1530(51.2); T1532(51.2); S1535(97.5); T1539(100); T1549(0)]	0.976	-0.035046947	0.977858961
Q7L014	2xPhospho [Y294(0); S295(100); S296(100); T305(0); T308(0); Y310(0); T312(0)]	1.289	0.366252264	0.885449652
Q7L014	1xPhospho [S804(100); S819(0); S823(0)]	1.198	0.260627908	0.939053555
Q7L014	2xPhospho [Y294(0); S295(100); S296(100); T305(0); T308(0); Y310(0); T312(0)]	1.137	0.185232254	0.965139891
Q7L014	2xPhospho [Y294(0); S295(100); S296(100); T305(0); T308(0); Y310(0); T312(0)]	1.114	0.155749233	0.970451963
Q7L2J0	3xPhospho [S57(0); S60(100); S69(100); T75(32.7); S76(32.7); S77(32.7); S78(1.9)]	0.01	-6.64385619	8.35E-17
Q7L2J0	1xPhospho [S94(0.1); S101(99.9)]	0.01	-6.64385619	8.35E-17
Q7L2J0	2xPhospho [S94(100); S101(100)]	0.364	-1.457989644	0.391213424
Q7L2J0	2xPhospho [S57(0); S60(100); S69(100); T75(0); S76(0); S77(0); S78(0)]	0.735	-0.444183845	0.811560495
Q7L2J0	2xPhospho [T208(0); T213(100); S216(100); S217(0)]	0.778	-0.36215794	0.872730156
Q7L2J0	1xPhospho [T236(0); S240(0); T243(0); T245(0); S254(100)]	0.59	-0.76121314	0.902447274
Q7L3B6	1xPhospho [S139(100)]	1.096	0.132247798	0.971838536
Q7L4I2	1xPhospho [T425(1.2); S426(98.8); S427(0)]	1.66	0.731183242	0.679191074
Q7L590	2xPhospho [S150(100); S155(100)]	0.421	-1.248107862	0.609882489
Q7L9B9	2xPhospho [S10(0); S16(100); S19(99.8); S21(0.3)]	1.331	0.412510571	0.851597639
Q7LBC6	2xPhospho [S725(33.1); S726(33.1); S727(33.1); T729(0.3); S730(0.3); S731(0.3); T733(0); T740(0); S742(0.7); S743(7.3); S744(84.7); T746(7.3); T751(0.1)]	0.01	-6.64385619	8.35E-17
Q7LBC6	2xPhospho [S725(24.9); S726(24.9); S727(24.9); T729(24.9); S730(0.2); S731(0.2); T733(0); T740(0); S742(0); S743(0.6); S744(92.4); T746(7.1); T751(0)]	0.744	-0.426625474	0.888644895
Q7RTP6	1xPhospho [S1649(99.9); T1651(0.1); T1654(0)]	1.232	0.301002256	0.922868578
Q7Z2K8	2xPhospho [S381(99.7); S382(0.3); S389(0); T392(33.3); T394(33.3); T395(33.3); S397(0.1)]	0.369	-1.438307279	0.513538425
Q7Z2K8	1xPhospho [S381(0); S382(0); S389(97.9); T392(2.1); T394(0.1); T395(0); S397(0)]	1.16	0.214124805	0.938029982
Q7Z2K8	2xPhospho [S381(99.9); S382(0.1); S389(99.9); T392(0.1); T394(0); T395(0); S397(0)]	0.687	-0.541617996	0.954539739
Q7Z2T5	1xPhospho [Y691(0); S692(0); T693(0); T695(0); Y696(0); T697(0); S701(0); S703(0); S707(100); S709(0); T712(0); T714(0)]	0.618	-0.694321257	0.759239289
Q7Z2T5	1xPhospho [S612(100); T617(0)]	1.236	0.305678743	0.946252189
Q7Z2W4	3xPhospho [S310(99.9); S312(50); S313(50); T316(0); T321(3.7); S322(96.3); T326(0); S327(0)]	100	6.64385619	8.35E-17
Q7Z2W4	1xPhospho [S335(100); S346(0); T347(0); Y348(0); S351(0); S353(0); T354(0); S355(0)]	100	6.64385619	8.35E-17
Q7Z2W4	2xPhospho [S492(2.9); S494(97); T497(0.2); S498(94.3); T499(2.8); T500(2.8); S501(0); S502(0)]	6.298	2.654893757	0.000682247
Q7Z2W4	3xPhospho [S335(100); S346(50); T347(50); Y348(0); S351(95.2); S353(4.8); T354(0); S355(0)]	1.775	0.827819025	0.59656225
Q7Z2W4	2xPhospho [S386(50); S387(50); S390(100); T393(0); T398(0); T399(0)]	1.793	0.842375488	0.612909889
Q7Z2W4	1xPhospho [S271(0); T273(0); S275(100); S280(0)]	1.703	0.768078435	0.657139363
Q7Z2W4	2xPhospho [S335(100); S346(96.4); T347(3.6); Y348(0); S351(0); S353(0); T354(0); S355(0)]	1.725	0.786596362	0.660888692
Q7Z2W4	1xPhospho [T375(0); S378(100); T380(0)]	0.514	-0.960159735	0.783976635
Q7Z2W4	2xPhospho [S310(100); S312(99.9); S313(0.1); T316(0); T321(0); S322(0); T326(0); S327(0)]	1.202	0.265436896	0.929708014
Q7Z2W4	2xPhospho [S271(0); T273(0); S275(7.3); S280(92.7); S284(99.9); T294(0.1)]	1.188	0.248534836	0.933107775
Q7Z2W4	1xPhospho [T298(0); Y299(0); S302(100)]	1.082	0.113700499	0.963156927
Q7Z2W4	2xPhospho [S271(0); T273(100); S275(100); S280(0)]	1.067	0.093560176	0.973263372
Q7Z2W4	1xPhospho [S284(100); T294(0)]	0.838	-0.254977851	0.980895643

Q7Z2Z1	2xPhospho [S834(99.9); S836(0.1); S838(100); S841(0)]	0.01	-6.64385619	8.35E-17
Q7Z2Z1	1xPhospho [S599(100)]	0.01	-6.64385619	8.35E-17
Q7Z2Z1	2xPhospho [S1428(0); S1430(100); S1433(100)]	0.079	-3.662003536	5.44E-05
Q7Z2Z1	2xPhospho [S1298(0); T1299(0); T1301(33.3); S1302(33.3); S1303(33.3); T1307(100)]	1.597	0.675364313	0.701261726
Q7Z309	1xPhospho [S58(100)]	0.01	-6.64385619	8.35E-17
Q7Z309	3 2xPhospho [T131(0); S134(100); S138(100); T140(0)]; Q7Z309 2xPhospho [T112(0); S115(100); S119(100); T121(0)]	0.41	-1.286304185	0.073479723
Q7Z309	3 2xPhospho [S149(0); S150(0); S151(0); S156(100); S160(100)]; Q7Z309 2xPhospho [S130(0); S131(0); S132(0); S137(100); S141(100)]	0.399	-1.325539348	0.082125891
Q7Z309	3 2xPhospho [T131(0); S134(100); S138(100); T140(0)]; Q7Z309 2xPhospho [T112(0); S115(100); S119(100); T121(0)]	0.375	-1.415037499	0.101796665
Q7Z309	3 1xPhospho [S58(0); S62(0); S63(1.2); S64(98.8)]	0.478	-1.064917477	0.374360028
Q7Z309	3 2xPhospho [S149(0); S150(0); S151(0); S156(100); S160(100)]; Q7Z309 2xPhospho [S130(0); S131(0); S132(0); S137(100); S141(100)]	0.791	-0.3382504	0.982345843
Q7Z333	1xPhospho [S1655(0); S1663(100); S1667(0)]	0.01	-6.64385619	8.35E-17
Q7Z333	2xPhospho [S1017(100); S1019(100)]	0.661	-0.597277823	0.958562891
Q7Z372	1xPhospho [S91(100); S100(0)]	1.092	0.126972856	0.965013037
Q7Z3B3	1xPhospho [S991(100); S994(0); S999(0); T1003(0)]	1.637	0.711054322	0.704203351
Q7Z3B3	2xPhospho [T955(93.5); T956(6.5); S961(0); S965(0); T966(0); S971(99.9); S975(0); S976(0); S977(0); S979(0); S981(0); Y983(0); S984(0); S988(0)]	0.572	-0.805912948	0.888360817
Q7Z3B3	2xPhospho [S991(100); S994(100); S999(0); T1003(0)]	0.909	-0.1376478	0.99811985
Q7Z3C6	2xPhospho [S14(100); Y15(0); S16(0); S18(100); S33(0)]	0.619	-0.691988685	0.91281798
Q7Z3C6	1xPhospho [S828(100)]	0.836	-0.258425153	0.981320581
Q7Z3K6	3xPhospho [S146(0); T148(0); S156(100); T163(100); S165(0.1); S168(99.9)]	0.01	-6.64385619	8.35E-17
Q7Z3K6	2xPhospho [S146(0); T148(0); S156(100); T163(100); S165(0); S168(0)]	0.715	-0.483984853	0.886312012
Q7Z3K6	1xPhospho [S146(0); T148(0); S156(100); T163(0); S165(0); S168(0)]	0.964	-0.052894948	0.986075329
Q7Z417	1xPhospho [Y619(0); S623(0); S629(100); T631(0); T633(0); S637(0)]	2.051	1.036327492	0.389428643
Q7Z417	2xPhospho [Y619(0); S623(0); S629(100); T631(0.1); T633(99.9); S637(0)]	1.743	0.801572569	0.527472712
Q7Z417	1xPhospho [T571(0); S572(100); S578(0)]	1.633	0.707524791	0.652713846
Q7Z417	1xPhospho [S652(100); S655(0)]	0.827	-0.274040765	0.943796521
Q7Z417	1xPhospho [T571(2); S572(98); S578(0)]	0.673	-0.57132159	0.94797789
Q7Z417	1xPhospho [S112(100); S113(0); T117(0); S121(0)]	1.161	0.215367972	0.958702638
Q7Z434	1xPhospho [T244(99.5); S246(0.4); S249(0); T250(0); T252(0); S253(0); S255(0); S256(0); S257(0); S258(0); S263(0)]	0.528	-0.921390165	0.590872099
Q7Z434	2xPhospho [T244(95.6); S246(4.4); S249(0.2); T250(99.6); T252(0.2); S253(0); S255(0); S256(0); S257(0); S258(0); S263(0)]	0.767	-0.382701517	0.934211842
Q7Z478	1xPhospho [S192(0); T198(0); S200(100)]	1.628	0.7031007	0.728446408
Q7Z4H3	1xMet-loss+Acetyl [N-Term]; 1xPhospho [S3(100); S5(0); S6(0); T8(0); S10(0)]	1.1	0.137503524	0.956041591
Q7Z4S6	2xPhospho [T1291(0); S1293(0); T1297(0); S1298(0); S1304(100); S1307(97.2); S1309(2.7); S1310(0.1); S1312(0)]	0.718	-0.477944251	0.931558535
Q7Z4S6	2xPhospho [S853(99.3); S854(0.3); S855(0.3); T862(0); S864(50); S865(50); T871(0); S874(0)]	0.705	-0.504304837	0.942574721
Q7Z4S6	1xPhospho [S1212(100); S1219(0)]	0.726	-0.461958547	0.942907978
Q7Z4S6	2xPhospho [S1261(0); S1263(0); T1265(0); S1266(0); S1269(0); S1271(100); S1274(0.1); S1275(99.9); S1278(0)]	1.152	0.204140717	0.945786032
Q7Z4S6	1xPhospho [S1239(100); T1242(0)]	1.061	0.085424656	0.980329546
Q7Z4S6	1xPhospho [S1662(2.9); T1664(97.1); S1673(0)]	0.898	-0.15521265	1
Q7Z4S6	1xPhospho [S853(95.4); S854(2.3); S855(2.3); T862(0); S864(0); S865(0); T871(0); S874(0)]	0.944	-0.083141235	1
Q7Z589	2xPhospho [T207(99.9); S209(91.7); S210(2.1); S211(2.1); S212(2.1); S213(2.1)]	1.539	0.621993232	0.71389078
Q7Z5K2	2xPhospho [S221(100); S223(0); S226(100)]	1.164	0.219091058	0.954054893
Q7Z5K2	1xPhospho [S68(0); T69(0); S77(100); S81(0); S85(0); S86(0)]	1.142	0.191562651	0.965013037
Q7Z5K2	2xPhospho [Y452(0); S459(100); S461(100)]	1.014	0.020057652	0.982029512
Q7Z5K2	2xPhospho [S221(100); S223(2.1); S226(97.9); S231(0)]	0.932	-0.10159814	0.99811985
Q7Z5L9	1xPhospho [S175(100)]	0.464	-1.10780329	0.302075459
Q7Z5L9	2xPhospho [T392(90); T394(3.3); S395(3.3); S396(3.3); S399(0); T404(50); S406(50); S409(0.1)]	0.449	-1.15521265	0.543115231
Q7Z5L9	1xPhospho [T392(0); T394(0); S395(0); S396(0); S399(0); T404(96.8); S406(3.2); S409(0)]	0.492	-1.023269779	0.617226186
Q7Z5L9	1xPhospho [S360(100)]	0.797	-0.327348371	0.954539739
Q7Z5L9	2xPhospho [S240(50.4); S242(50.4); S243(99.2); S244(0)]	0.913	-0.131313235	0.990299313
Q7Z5L9	1xPhospho [S360(100)]	0.967	-0.048412205	0.99184412
Q7Z6E9	3xPhospho [T1632(0); Y1641(0); S1644(100); S1646(100); S1648(0); S1651(100); S1657(0); S1658(0); S1662(0)]	0.01	-6.64385619	8.35E-17
Q7Z6E9	1xPhospho [T1256(0); T1258(0); S1261(0); S1262(0); S1263(0); T1264(0); Y1268(0); T1269(0); S1270(0); T1271(0); S1272(0); S1273(0); T1274(0); S1277(100)]	0.01	-6.64385619	8.35E-17
Q7Z6E9	3xPhospho [Y766(0); S768(100); S770(100); S772(100)]	3.964	1.986956963	0.016243773
Q7Z6E9	2xPhospho [T971(0); S973(0); S979(0.3); T984(99.7); S995(99.7); T997(0.3)]	1.547	0.629473197	0.720161581
Q7Z6E9	1xPhospho [T1173(0); S1179(100)]	0.751	-0.413115187	0.936043234
Q7Z6E9	1xPhospho [S1328(100)]	1.127	0.172487516	0.952591019
Q7Z6E9	2xPhospho [T1256(0); T1258(0); S1261(0); S1262(0); S1263(0); T1264(0); Y1268(0); T1269(0); S1270(0); T1271(0); S1272(5.7); S1273(94.3); T1274(94.3); S1277(5.7)]	1.036	0.051024003	0.99277609
Q7Z6E9	2xPhospho [T984(100); S995(100); T997(0)]	0.951	-0.072482754	1
Q7Z6J0	1xPhospho [S304(99.9); T306(0.1); S307(0); T309(0)]	1.242	0.312665174	0.91490394
Q7Z6J0	2xPhospho [S735(100); S739(100); T741(0); S748(0)]	1.035	0.049630768	0.980895643
Q7Z6J9	2xPhospho [S225(94.5); S229(5.2); S230(0.3); S235(0); S238(0); S248(50); S249(50)]	1.578	0.658097205	0.746167942
Q7Z6Z7	2xPhospho [S3808(100); S3816(100); S3818(0); T3822(0); S3824(0); S3827(0); T3830(0)]	0.381	-1.392137097	0.21587309
Q7Z6Z7	1xPhospho [S3808(0); S3816(100); S3818(0); T3822(0); S3824(0); S3827(0); T3830(0)]	0.529	-0.918660373	0.576849086

Q7Z6Z7	3xPhospho [S3906(0); S3919(100); T3924(100); T3927(91.6); S3929(4.2); S3930(4.2); S3936(0)]	0.65	-0.621488377	0.75891781
Q7Z6Z7	1xPhospho [S2887(99.9); S2888(0); T2889(0)]	0.962	-0.055891201	0.980895643
Q7Z6Z7	1xPhospho [S1903(0); T1905(0); S1907(100)]	1.058	0.081339627	0.983265186
Q7Z739	1xPhospho [S372(0); S383(5.2); S385(94.8); S387(0); S388(0)]	100	6.64385619	8.35E-17
Q7Z7F0	1xPhospho [S474(0); Y479(0); T487(0); T491(0); S494(0); S495(0); S504(0); S508(91.3); S509(4.4); S510(4.4)]	100	6.64385619	8.35E-17
Q7Z7F0	2xPhospho [Y567(0); S571(100); S572(100)]	1.118	0.160920188	0.971196575
Q7Z7G8	2xPhospho [S999(100); S1002(100)]	0.908	-0.139235797	0.985461924
Q86SQ0	2xPhospho [S71(100); S73(100); T77(0); S78(0)]	0.01	-6.64385619	8.35E-17
Q86SQ0	2xPhospho [S384(100); S387(100); S397(0)]	0.01	-6.64385619	8.35E-17
Q86SQ0	2xPhospho [S384(100); S387(100); S397(0)]	0.01	-6.64385619	8.35E-17
Q86SQ0	1xPhospho [T550(100); S555(0); S556(0); T557(0)]	0.01	-6.64385619	8.35E-17
Q86SQ0	2xPhospho [S489(100); S493(100); S501(0); T504(0)]	0.01	-6.64385619	8.35E-17
Q86SQ0	1xPhospho [S513(100); S521(0); S524(0); S526(0); S528(0); S529(0); S531(0); T534(0)]	0.01	-6.64385619	8.35E-17
Q86SQ0	1xPhospho [S932(2.9); S934(97.1); S937(0); S942(0)]	0.01	-6.64385619	8.35E-17
Q86SQ0	2xPhospho [S513(100); S521(0); S524(0); S526(0); S528(49.3); S529(1.3); S531(49.3); T534(0)]	0.21	-2.251538767	0.007136141
Q86SQ0	1xPhospho [S932(0); S934(100); S937(0); S942(0)]	0.222	-2.171368418	0.008188385
Q86SQ0	1xPhospho [S489(99.9); S493(0.1); S501(0); T504(0)]	0.88	-0.184424571	1
Q86T82	2xPhospho [S642(0); S643(0); S650(100); S652(100)]	0.01	-6.64385619	8.35E-17
Q86TB9	4 2xPhospho [S177(0); T178(0); S179(100); S184(100)]	0.661	-0.597277823	0.825610199
Q86TB9	4 1xPhospho [S177(0); T178(0); S179(100); S184(0)]	1.436	0.522055749	0.831443663
Q86TB9	4 2xPhospho [S177(0); T178(0); S179(100); S184(100)]	0.848	-0.23786383	0.960831064
Q86U06	1xPhospho [S149(100); S159(0)]	1.127	0.172487516	0.965013037
Q86U42	1xMet-loss+Acetyl [N-Term]; 1xPhospho [S19(100)]	0.763	-0.390245038	0.855816069
Q86U42	1xPhospho [S150(100); S162(0)]	0.719	-0.475936324	0.85992414
Q86U90	1xPhospho [S37(100)]	1.041	0.057970069	0.986075329
Q86UD0	2xPhospho [T362(100); S369(100)]	0.859	-0.219269964	0.972501767
Q86UE4	1xPhospho [S415(0); S426(100)]	1.718	0.780730036	0.495170209
Q86UE4	1xPhospho [S251(100); S258(0)]	1.217	0.283329168	0.91490394
Q86UE4	1xPhospho [S251(100); S258(0)]	1.183	0.242450074	0.934211842
Q86UP2	1xPhospho [S75(100); S77(0); S79(0)]	1.485	0.570462931	0.751633178
Q86UP2	1xPhospho [S75(98.8); S77(1.2); S79(0)]	1.375	0.459431619	0.848293321
Q86UP2	2xPhospho [S75(100); S77(50); S79(50)]	0.697	-0.520769439	0.876066502
Q86US8	2xPhospho [T469(0); T471(0); T479(100); S484(100); T486(0); S487(0); S491(0)]	1.058	0.081339627	0.976868831
Q86UU0	1xPhospho [S915(100); T918(0); S920(0); S926(0)]	2.378	1.249748715	0.309407871
Q86UU0	2xPhospho [S21(100); S25(100)]	1.512	0.59645814	0.741297748
Q86UU0	2xPhospho [S934(96.3); T936(3.7); S938(0); S942(0); T946(3.6); S947(92.9); S949(3.6)]	0.698	-0.518701058	0.939053555
Q86UU0	1xPhospho [S975(100); S981(0); S982(0); S984(0)]	0.685	-0.545824107	0.962617576
Q86UU1	2xPhospho [S518(100); S520(100); T522(0); S526(0)]	100	6.64385619	8.35E-17
Q86UU1	2xPhospho [S578(100); T580(0); S583(100); Y592(0)]	0.78	-0.358453971	0.879063032
Q86UU1	2xPhospho [S578(100); T580(0); S583(100); Y592(0)]	0.785	-0.349235441	0.963219686
Q86UU1	2xPhospho [T516(0); S518(100); S520(100); T522(0); S526(0)]	1.065	0.09085343	0.969482098
Q86UU1	3xPhospho [T516(100); S518(100); S520(99.9); T522(0.1); S526(0)]	1.046	0.064882852	0.977858961
Q86V48	1xPhospho [S659(100); S667(0); T670(0)]	0.495	-1.01449957	0.646029575
Q86V48	1xPhospho [S611(100); S620(0); T621(0); S622(0)]	0.806	-0.311148256	0.956734015
Q86VM9	1xPhospho [S532(1.9); S534(98.1); S536(0)]	1.613	0.689746438	0.699388119
Q86VM9	3xPhospho [S59(0); S67(100); S74(100); S78(99.6); S83(0.4); S89(0)]	1.321	0.401630467	0.862820654
Q86VM9	2xPhospho [T93(0); S94(0); S95(0); T109(0.2); S110(99.8); S117(49.9); S118(49.9); T120(0.1)]	1.296	0.374065718	0.892457589
Q86VM9	2xPhospho [S532(100); S534(98.4); S536(1.6)]	1.193	0.254594043	0.933107775
Q86VM9	3xPhospho [T93(0); S94(0.3); S95(99.7); T109(0); S110(0); S117(100); S118(99.7); T120(0.3)]	1.193	0.254594043	0.945893265
Q86VM9	1xPhospho [S790(0); S791(0); S795(3.4); T796(96.6); T809(0)]	1.135	0.182692298	0.965013037
Q86VM9	2xPhospho [S59(0); S67(100); S74(100); S78(0); S83(0); S89(0)]	1.064	0.089498151	0.983788249
Q86VM9	2xPhospho [S532(100); S534(100); S536(0)]	1.036	0.051024003	0.998448514
Q86VM9	2xPhospho [S32(0.3); S34(99.7); S46(100); S53(0)]	0.904	-0.145605322	1
Q86VX9	1xPhospho [S56(100); Y57(0); T61(0); S63(0); S69(0); S72(0)]	1.565	0.646162657	0.701261726
Q86VX9	1xPhospho [S31(100); T33(0)]	1.311	0.390667686	0.862820654
Q86VY9	1xPhospho [S350(100); S354(0); S356(0); S357(0); Y359(0)]	0.01	-6.64385619	8.35E-17
Q86VY9	2xPhospho [S384(97.6); T386(51.2); S387(51.2); S392(0); S393(0); S395(0)]	0.01	-6.64385619	8.35E-17
Q86VY9	2xPhospho [S19(100); S22(100); S29(0); S31(0); T34(0)]	0.302	-1.727379545	0.257416476
Q86W56	3xPhospho [S190(0); S197(100); T199(100); S201(100); T212(0); T213(0)]	0.01	-6.64385619	8.35E-17
Q86W56	1xPhospho [S312(0); S316(100); T322(0); S323(0); S333(0); S334(0); S335(0); T337(0); S342(0)]	2.486	1.313826296	0.193210854
Q86W56	2xPhospho [S190(0); S197(100); T199(100); S201(0); T212(0); T213(0)]	0.665	-0.588573754	0.829211044
Q86W56	2xPhospho [S312(0); S316(99.8); T322(0.4); S323(99.8); S333(0); S334(0); S335(0); T337(0); S342(0)]	1.275	0.350497247	0.879063032
Q86W56	1xPhospho [S130(0); S133(0); S137(1.7); T139(98.3)]	0.643	-0.637109357	0.93461208
Q86W56	1xPhospho [S190(0); S197(99.9); T199(0.1); S201(0); T212(0); T213(0)]	1.053	0.074505436	0.97198185
Q86W56	2xPhospho [S130(0); S133(100); S137(100); T139(0)]	0.935	-0.09696173	0.973452084
Q86W92	2 2xPhospho [Y28(0); S29(0); S37(99.9); T39(3.1); S40(97); S45(0)]	0.329	-1.603840511	0.123307226
Q86W92	2 1xPhospho [S593(0); S595(100); T596(0); T597(0); S604(0)]	0.429	-1.220950447	0.126193902
Q86W92	2 2xPhospho [Y336(0); S342(0); S343(0.1); S344(99.9); S346(0.1); S347(99.9); S355(0)]	0.505	-0.985644707	0.300697818
Q86W92	2 1xPhospho [S630(100); S632(0)]	0.432	-1.210896782	0.31868954
Q86W92	2 1xPhospho [S565(0); S573(100); S576(0)]	0.535	-0.902389203	0.33990982
Q86W92	2 1xPhospho [Y28(0); S29(0); S37(100); T39(0); S40(0); S45(0)]	0.459	-1.123433941	0.598714672
Q86W92	2 1xPhospho [S788(100); T792(0); S796(0)]	0.592	-0.756330919	0.855557911

Q86WB0	1xPhospho [T52(0); S53(0); T55(0); S56(0); S58(0); S62(100); S69(0); S72(0); T73(0); S74(0)]	1.521	0.605020153	0.75802349
Q86WB0	1xPhospho [S381(0); T384(0); T387(0); S394(1.9); S395(98.2)]	1.495	0.580145484	0.801883298
Q86WB0	2xPhospho [S354(0); S357(1.3); S358(49.4); S359(49.4); S370(100); T372(0); T373(0)]	1.203	0.266636643	0.930376591
Q86WB0	2xPhospho [T352(0.3); S354(99.7); S357(48.7); S358(48.7); S359(2.6); S370(0); T372(0); T373(0)]	1.137	0.185232254	0.938029982
Q86WB0	1xPhospho [S329(0); T333(0); S335(0); S338(0); S344(100); S350(0)]	0.825	-0.277533976	0.965013037
Q86WB0	2xPhospho [S24(100); T28(100)]	1.031	0.044044333	0.965795497
Q86WB0	1xPhospho [S321(100)]	1.026	0.037030731	0.971220368
Q86WB0	2xPhospho [S381(100); T384(0); T387(0); S394(0.1); S395(99.9)]	0.783	-0.352915787	0.976345951
Q86WB0	2xPhospho [S329(0); T333(0); S335(100); S338(0); S344(100); S350(0)]	1.032	0.045442971	0.981858798
Q86WB0	3xPhospho [S329(0); T333(0); S335(100); S338(100); S344(100); S350(0)]	0.898	-0.15521265	0.985318278
Q86WB0	1xPhospho [S321(100)]	0.931	-0.103146927	0.99184412
Q86WR7	1xPhospho [S179(100)]	1.869	0.902266569	0.62084251
Q86X02	2xPhospho [S303(50); S304(50); S308(100)]	1.099	0.136191386	0.959183327
Q86X29	3xPhospho [S493(90.2); T501(9.8); S504(0); T505(0); S508(0); S510(1.5); S512(1.5); T514(97.4); S515(99.6)]	0.01	-6.64385619	8.35E-17
Q86X29	3xPhospho [T453(100); S464(100); S467(100)]	0.01	-6.64385619	8.35E-17
Q86X29	2xPhospho [T453(0); S464(100); S467(100)]	0.393	-1.347398782	0.076396464
Q86X29	2xPhospho [S643(100); S646(100)]	0.52	-0.943416472	0.299786659
Q86X29	1xPhospho [S530(100); Y535(0); S540(0)]	2.02	1.014355293	0.353682182
Q86X29	2xPhospho [S530(100); Y535(0); S540(100)]	0.559	-0.839079812	0.771290474
Q86X29	1xPhospho [S493(100); T501(0); S504(0); T505(0); S508(0); S510(0)]	0.754	-0.407363571	0.924586883
Q86X29	2xPhospho [S643(100); S646(100)]	0.763	-0.390245038	0.925853562
Q86X29	1xPhospho [S493(100); T501(0); S504(0); T505(0); S508(0); S510(0)]	0.763	-0.390245038	0.939268934
Q86X29	1xPhospho [S530(100); Y535(0); S540(0)]	0.844	-0.244685096	0.965013037
Q86X29	2xPhospho [S493(100); T501(100); S504(0); T505(0); S508(0); S510(0)]	1.007	0.010063683	0.979125671
Q86X29	1xPhospho [S528(0); S530(100); Y535(0); S540(0)]	0.887	-0.17299399	0.97998947
Q86X29	2xPhospho [S493(100); T501(100); S504(0); T505(0); S508(0); S510(0)]	0.984	-0.023269779	0.992376868
Q86XK2	1xPhospho [S143(100); S147(0); Y154(0)]	0.18	-2.473931188	0.004461349
Q86XN8	1xPhospho [S514(100); T516(0)]	1.626	0.701327257	0.747042609
Q86XN8	2xPhospho [S505(0); S506(0); T510(100); S514(100); T516(0)]	1.314	0.393965276	0.855545836
Q86XN8	3xPhospho [S505(95.8); S506(4.4); T510(99.8); S514(100); T516(0)]	0.882	-0.181149439	0.986075329
Q86XP3	1xPhospho [S705(100); Y707(0)]	13.616	3.767231037	2.07E-05
Q86XP3	1xPhospho [S709(0); S715(100); S717(0)]	1.912	0.935082523	0.428495392
Q86XP3	2xPhospho [S723(0); S724(0); S729(0); T732(0.5); S733(86.7); S736(6.9); S739(6.9); T742(99); S744(0); S751(0); S754(0); T757(0); S758(0)]	0.583	-0.778432211	0.629286646
Q86XP3	1xPhospho [T769(0.2); S773(99.9); T778(0); Y779(0); S781(0); T790(0); S792(0); S796(0)]	0.654	-0.612637459	0.777156028
Q86XP3	2xPhospho [S35(0); S37(100); T42(2.7); S43(2.7); S44(89.3); S45(2.7); S46(2.7)]	1.263	0.336854639	0.862820654
Q86XP3	1xPhospho [Y75(0); S83(0); S84(0); Y90(0); S96(96.6); T98(3.4)]	1.154	0.206643224	0.920957334
Q86XP3	1xPhospho [S723(0); S724(0); S729(0); T732(0); S733(0); S736(100); S739(0); T742(0); S744(0); S751(0); S754(0); T757(0); S758(0)]	1.178	0.236339539	0.933760225
Q86XP3	1xPhospho [Y163(0); T169(0); Y183(0); S185(100); T193(0)]	0.897	-0.15682011	0.971838536
Q86XP3	2xPhospho [S104(0); S109(100); S111(100)]	0.922	-0.117161344	0.973763863
Q86XP3	1xPhospho [Y75(0); S83(0); S84(0); Y90(0); S96(96.8); T98(3.2)]	1.049	0.069014678	0.974924677
Q86XP3	1xPhospho [S51(0); S58(100); Y60(0)]	0.778	-0.36215794	0.980895643
Q86XP3	1xPhospho [Y163(0); T169(0); Y183(0); S185(100); T193(0)]	0.911	-0.134477041	0.987554799
Q86XP3	2xPhospho [S104(0); S109(100); S111(100)]	1.021	0.029982866	0.998086195
Q86Y07	1xPhospho [S331(100); T336(0); S339(0)]	1.518	0.602171791	0.810625976
Q86Y91	3xPhospho [S416(0); S425(8); S426(92.1); S434(100); T438(100)]	100	6.64385619	8.35E-17
Q86YP4	2xPhospho [S100(100); S107(100); S113(0); S114(0)]	1.342	0.424384672	0.835464048
Q86YP4	3xPhospho [S100(100); S107(100); S113(2.2); S114(97.8)]	0.854	-0.227692025	0.942907978
Q86YS7	1xPhospho [T291(0); Y292(0); S293(0); S295(98.5); S297(1.5)]	100	6.64385619	8.35E-17
Q86YS7	2xPhospho [S249(0.1); S250(99.9); S260(96.7); S262(3.3)]	1.758	0.81393507	0.630710862
Q86YS7	2xPhospho [T278(0.1); S280(49); S281(49); S284(5.8); T285(96)]	0.697	-0.520769439	0.797966154
Q86YS7	2xPhospho [S657(0); S659(100); S661(50); S662(50); T666(0); S671(0)]	0.759	-0.397828209	0.963156927
Q86YS7	1xPhospho [S249(0); S250(0); S260(98.4); S262(1.6)]	1.027	0.038436182	0.978067937
Q86YV5	2xPhospho [T767(0); S769(0); S774(0); S775(0); S780(95.9); T782(4.3); S784(99.8)]	2.337	1.224657734	0.444626397
Q86YV5	1xPhospho [S692(0); S694(100)]	0.772	-0.373327247	0.980895643
Q8IU60	3xPhospho [S246(100); S247(100); S249(100); S254(0); S255(0); T256(0); S258(0); T259(0); T264(0)]	0.01	-6.64385619	8.35E-17
Q8IU81	1xPhospho [S436(100)]	1.132	0.178873958	0.933452347
Q8IUW5	2xPhospho [S244(50.2); S247(50.2); S249(90.4); T253(9.1); T261(0)]	0.01	-6.64385619	8.35E-17
Q8IV36	1xPhospho [T591(0.2); S593(99.8); T597(0); S598(0); S602(0); T612(0)]	0.386	-1.373327247	0.361960227
Q8IV50	2xPhospho [S29(2.5); S31(2.5); S33(95.1); S35(99.8); S41(0); S43(0)]	0.01	-6.64385619	8.35E-17
Q8IV50	2xPhospho [S21(0.1); S24(99.9); S29(100)]	1.224	0.291603558	0.921519407
Q8IV50	1xPhospho [S21(0); S24(100)]	0.832	-0.265344567	0.981526996
Q8IV63	2xPhospho [S54(0); S55(100); T58(97.8); S59(2.2)]	10.21	3.351910961	7.41E-05
Q8IV63	2xPhospho [S54(5.4); S55(94.6); T58(50); S59(50)]	1.101	0.138814469	0.96229367
Q8IVF2	1xPhospho [S842(100); S846(0)]; 1xPhospho [S1007(100); S1011(0)]; 1xPhospho [S1337(100); S1341(0)]; 1xPhospho [S1502(100); S1506(0)]; 1xPhospho [S1667(100); S1671(0)]; 1xPhospho [S1832(100); S1836(0)]; 1xPhospho [S2162(100); S2166(0)]; 1xPhospho [S2492(100); S2496(0)]; 1xPhospho [S2822(100); S2826(0)]; 1xPhospho [S2987(100); S2991(0)]; 1xPhospho [S3152(100); S3156(0)]; 1xPhospho [S3812(100); S3816(0)]; 1xPhospho [S3977(100); S3981(0)]; 1xPhospho [S4142(100); S4146(0)]; 1xPhospho [S4307(100); S4311(0)]	0.606	-0.722610301	0.831443663
Q8IVM0	2 1xPhospho [T448(0); Y455(0); T456(0); T459(0.3); S463(94); S464(5.4); T465(0.3)]	10.996	3.458906907	0.000135094

Q8IVM0	2 1xPhospho [T310(100); S314(0); S316(0)]	0.778	-0.36215794	0.964978421
Q8IVP5	1xPhospho [Y11(0); S13(100); S17(0); Y18(0); T24(0); Y26(0)]	0.764	-0.388355457	0.94797789
Q8IVT2	1xPhospho [S471(100); S473(0); S474(0); S479(0); T480(0)]	11.795	3.560103513	0.000910909
Q8IVT2	2xPhospho [S213(95); S214(95); T219(10); T223(0); T224(0); S228(0)]	1.695	0.761285273	0.738872836
Q8IVT2	1xPhospho [S575(99.8); T577(0.2); S582(0); S586(0)]	1.286	0.362890643	0.866095862
Q8IVT2	1xPhospho [S394(100); S395(0); S397(0); S400(0)]	0.806	-0.311148256	0.94797789
Q8IVT2	2xPhospho [S394(100); S395(0); S397(100); S400(0)]	1.051	0.071762669	0.980895643
Q8IW35	2xPhospho [T811(0); S813(100); S817(100); S821(0); S825(0); S833(0); T835(0); S839(0)]	100	6.64385619	8.35E-17
Q8IWA0	2xPhospho [S779(100); S782(100); T789(0)]	0.572	-0.805912948	0.448133957
Q8IWA0	1xPhospho [T795(0); S796(100); T798(0); S809(0)]	1.78	0.831877241	0.561634447
Q8IWA0	2xPhospho [S779(100); S782(100); T789(0)]	0.747	-0.420819852	0.894853453
Q8IWS0	2xPhospho [T134(0); S138(0.2); S145(99.8); S154(0.2); S155(99.8)]	0.647	-0.628162383	0.800861292
Q8IWS0	1xPhospho [T134(0); S138(0); S145(0); S154(2.9); S155(97.1)]	1.294	0.371837617	0.904774194
Q8IWS0	3xPhospho [T134(0); S138(100); S145(100); S154(6.2); S155(93.9)]	0.741	-0.432454552	0.942574721
Q8IWS0	2xPhospho [T134(0); S138(0); S145(100); S154(2.7); S155(97.3)]	0.988	-0.017417053	0.996295697
Q8IWX8	3xPhospho [S815(100); S817(100); T819(100); S822(0); S823(0); S828(0); S830(0); S837(0)]	0.704	-0.506352666	0.759239289
Q8IWX8	2xPhospho [S815(100); S817(99.9); T819(0.1); S822(0); S823(0); S828(0); S830(0); S837(0)]	0.925	-0.112474729	0.997838179
Q8IWZ3	6 1xPhospho [T1678(0); S1679(100)]	1.16	0.214124805	0.942574721
Q8IWZ8	1xPhospho [Y483(0); S485(100); S491(0); T495(0)]	100	6.64385619	8.35E-17
Q8IX21	3xPhospho [S603(100); S607(100); Y610(0); S614(100); T620(0)]	0.36	-1.473931188	0.477649934
Q8IX21	2xPhospho [S710(100); T711(100)]	0.663	-0.592919225	0.835464048
Q8IX90	1xPhospho [S110(100); S119(0); S124(0)]	0.01	-6.64385619	8.35E-17
Q8IX90	1xPhospho [S110(100); S119(0); S124(0)]	0.01	-6.64385619	8.35E-17
Q8IX90	1xPhospho [S155(100); S159(0)]	0.857	-0.222632891	0.973263372
Q8IXJ6	2xPhospho [S351(0); S356(0); S364(3.4); T365(3.4); S366(3.4); S368(89.9); S372(100)]	1.164	0.219091058	0.938483927
Q8IXQ4	3xPhospho [T138(100); S140(100); S141(100)]	0.01	-6.64385619	8.35E-17
Q8IXQ4	1xPhospho [S273(2); S275(98)]	3.026	1.597411988	0.228891308
Q8IXQ4	1xPhospho [S105(100)]	0.588	-0.76611194	0.75736259
Q8IXQ4	1xPhospho [S105(100)]	0.733	-0.448114897	0.942574721
Q8IXT5	2xPhospho [S710(100); S718(100)]	3.046	1.606915942	0.031292832
Q8IXT5	1xPhospho [S710(0); S718(100)]	2.238	1.162210036	0.453324508
Q8IXT5	2xPhospho [S638(100); T640(100)]	1.813	0.858378925	0.550634186
Q8IXT5	1xPhospho [S575(100)]	1.744	0.80240004	0.62084251
Q8IXT5	2xPhospho [S98(100); T100(2.5); S101(97.5); S105(0); S107(0); S112(0)]	1.747	0.804879608	0.676726843
Q8IXT5	1xPhospho [S829(0); S839(100)]	1.498	0.583037624	0.798487882
Q8IXT5	2xPhospho [S710(100); S718(100)]	1.393	0.478195258	0.820380622
Q8IXT5	2xPhospho [S250(100); S254(100)]	1.148	0.199122642	0.962617576
Q8IXT5	2xPhospho [S278(100); S280(100); Y285(0)]	1.09	0.124328135	0.974924677
Q8IY18	1xPhospho [S35(100); S44(0); S45(0); S52(0)]	0.941	-0.087733372	0.985870803
Q8IY67	2 1xPhospho [S627(0); S628(0); S631(0); S637(0); Y640(0); S641(6); S643(94); T645(0); S646(0); Y647(0); T649(0); S650(0)]; E9PAU2 1xPhospho [S644(0); S645(0); S648(0); S654(0); Y657(0); S658(6); S660(94); T662(0); S663(0); Y664(0); T666(0); S667(0)]	0.01	-6.64385619	8.35E-17
Q8IY67	2 2xPhospho [S667(93.9); S668(6.1); S672(0); S680(100); S687(0)]; E9PAU2 2xPhospho [S684(93.9); S685(6.1); S689(0); S697(100); S704(0)]	100	6.64385619	8.35E-17
Q8IY67	2 2xPhospho [S700(0.6); S707(99.4); S711(100); S715(0); Y716(0); S721(0); Y728(0); S731(0); Y732(0)]; E9PAU2 2xPhospho [S717(0.6); S724(99.4); S728(100); S732(0); Y733(0); S738(0); Y745(0); S748(0); Y749(0)]	4.773	2.254896336	0.014579118
Q8IY67	2 1xPhospho [S617(100); S621(0)]; E9PAU2 1xPhospho [S634(100); S638(0)]	2.769	1.469365054	0.176594978
Q8IY67	2 1xPhospho [S617(100); S621(0)]; E9PAU2 1xPhospho [S634(100); S638(0)]	1.319	0.399444565	0.879063032
Q8IY67	2 1xPhospho [S567(100); S570(0); S571(0)]; E9PAU2 1xPhospho [S584(100); S587(0); S588(0)]	0.741	-0.432454552	0.969482098
Q8IY67	2 1xMet-loss+Acetyl [N-Term]; 1xPhospho [S6(0); T8(0); S14(100)]	1.05	0.070389328	0.974924677
Q8IY67	2 1xPhospho [T463(100)]; E9PAU2 1xPhospho [T480(100)]	0.814	-0.2968993	0.99811985
Q8IY81	1xPhospho [S688(100)]	6.902	2.787014474	0.004216834
Q8IY81	3xPhospho [S333(100); S335(100); S336(100); S347(0); T348(0); T351(0); T352(0)]	0.215	-2.217591435	0.104523056
Q8IY81	2xPhospho [S333(0); S335(100); S336(100); S347(0); T348(0); T351(0); T352(0)]	0.481	-1.055891201	0.331475279
Q8IY81	1xPhospho [S644(100)]	0.836	-0.258425153	0.980895643
Q8IY92	2xPhospho [S1674(0); T1676(100); S1679(98.4); S1681(1.6); T1683(0)]	1.584	0.663572335	0.75736259
Q8IYL3	1xPhospho [T169(0); S182(0); S189(100); S196(0)]	0.692	-0.531156057	0.855816069
Q8IYL3	1xPhospho [T169(0); S182(0); S189(100); S196(0)]	0.93	-0.104697379	0.997462902
Q8IZ21	2xPhospho [T233(0); S240(50); T241(50); T243(0.1); T244(0); T246(0); S248(96.8); T250(3.2)]	100	6.64385619	8.35E-17
Q8IZ21	2xPhospho [S270(100); S278(0); S283(0); T285(0); S288(0); S291(100)]	100	6.64385619	8.35E-17
Q8IZ21	1xPhospho [S116(0); S117(0); S118(100)]	100	6.64385619	8.35E-17
Q8IZ21	2xPhospho [S342(3); S344(97.1); T349(0); T358(100)]	1.201	0.264236151	0.933760225
Q8IZ21	3xPhospho [S342(100); S344(100); T349(0); T358(100)]	0.823	-0.281035664	0.962617576
Q8IZ40	3xPhospho [T22(0); S31(100); S35(100); S36(99.7); S41(0.4); S44(0)]	0.078	-3.680382066	0.000479801
Q8IZP0	2xPhospho [T174(0); T178(0); S183(100); S187(100)]	1.379	0.463622457	0.849882438
Q8IZP0	1xPhospho [T174(0); T178(0); S183(100); S187(0)]	1.313	0.392866916	0.877965479
Q8IZV2	2xPhospho [S9(0); T11(0); T13(0); T14(0); T15(0); S17(0); S18(0); S24(100); T25(0); S26(0.1); S27(49.2); S28(49.2); S29(1.6); Y32(0)]	0.588	-0.76611194	0.706674105
Q8N0Y2	1xPhospho [T228(0); S232(99.9); S235(0.1)]	1.193	0.254594043	0.922868578
Q8N108	2xPhospho [T38(0); T49(0); S52(100); S53(100)]	1.153	0.205392513	0.921238122
Q8N108	2xPhospho [S483(100); S488(100); S491(0); S492(0); S500(0)]	1.101	0.138814469	0.972363997
Q8N163	2xPhospho [S671(0); S675(100); S678(100); S681(0); S686(0); S687(0)]	100	6.64385619	8.35E-17
Q8N163	2xPhospho [T609(0); S611(0); S626(100); S627(100)]	0.307	-1.703689439	0.148999268

Q8N163	1xPhospho [T609(0); S611(0); S626(3.9); S627(96.1)]	1.663	0.733788169	0.643284326
Q8N163	3xPhospho [S675(100); S678(100); S681(100); S686(0); S687(0)]	0.591	-0.758769964	0.714293599
Q8N163	3xPhospho [S671(0.2); S675(99.8); S678(100); S681(100); S686(0); S687(0)]	0.617	-0.696657606	0.79670828
Q8N163	2xPhospho [S675(100); S678(100); S681(0); S686(0); S687(0)]	0.552	-0.857259828	0.855534442
Q8N163	2xPhospho [S671(0); S675(100); S678(100); S681(0); S686(0); S687(0)]	0.675	-0.567040593	0.868176795
Q8N163	2xPhospho [S675(100); S678(100); S681(0); S686(0); S687(0)]	0.743	-0.428565884	0.918281661
Q8N163	3xPhospho [S671(0); S675(100); S678(100); S681(100); S686(0); S687(0)]	1.011	0.015782997	0.986075329
Q8N1C0	3xPhospho [S282(100); T284(0); S285(100); T288(100); S298(0)]	1.279	0.355016264	0.90624343
Q8N1C0	2xPhospho [S282(100); T284(0); S285(100); T288(0); S298(0)]	0.82	-0.286304185	0.9200308
Q8N1C0	2xPhospho [T264(0); S271(100); T275(0); S282(100)]	0.759	-0.397828209	0.964978421
Q8N1C0	1xPhospho [T264(0); S271(100); T275(0)]	0.865	-0.209227962	0.965219233
Q8N1C0	3xPhospho [T264(0); S271(9.1); T275(9.1); S282(93.4); T284(93.4); S285(93.4); T288(1.6); S298(0)]	0.94	-0.089267338	0.981858798
Q8N1G2	3xPhospho [S46(0); T47(0); T48(0); S49(0.4); S51(99.6); S53(66.7); S55(66.7); T57(66.7); S63(0); S64(0); S66(0)]	0.01	-6.64385619	8.35E-17
Q8N1G2	2xPhospho [S26(96.3); S28(3.5); S29(3.4); T30(3.5); S31(93.2); S37(0); S38(0); S40(0)]	0.389	-1.36215794	0.17373466
Q8N1G2	1xPhospho [S26(0); S28(0); S29(0); T30(0.1); S31(99.9); S37(0); S38(0); S40(0)]	0.988	-0.017417053	0.980895643
Q8N1G4	1xPhospho [Y509(0); T510(0); S518(0.1); S520(99.8); T522(0.1); S528(0); T535(0); T536(0); S539(0)]	100	6.64385619	8.35E-17
Q8N1G4	2xPhospho [Y509(0); T510(0); S518(100); S520(100); T522(0); S528(0); T535(0); T536(0); S539(0)]	0.629	-0.668868078	0.787333611
Q8N1W1	2xPhospho [S476(33.3); S477(33.3); S478(33.3); S486(100); S492(0); S495(0); Y499(0); T500(0); S503(0); S505(0); S506(0); S507(0)]	0.01	-6.64385619	8.35E-17
Q8N201	2xPhospho [S283(50); S284(50); S288(0.1); T290(0); S295(0); T297(0); S307(100)]	1.39	0.475084883	0.855816069
Q8N3C0	1xPhospho [S2193(0); S2195(100); T2197(0)]	100	6.64385619	8.35E-17
Q8N3D4	3xPhospho [T164(0.1); S170(99.9); S173(99.3); S176(0.8); S180(0); S191(100)]	0.155	-2.689659879	0.001005468
Q8N3D4	1xPhospho [S1257(100); S1264(0)]	2.111	1.077926579	0.369703851
Q8N3D4	1xPhospho [S310(100)]	1.639	0.712815854	0.73347811
Q8N3F8	2xPhospho [S588(0); S589(0); S598(0); T601(0); T603(0); S612(100); S616(0.3); S619(33.2); S620(33.2); S621(33.2)]	0.226	-2.145605322	0.08370466
Q8N3F8	2xPhospho [S459(0); Y464(0); T467(100); T469(100); S470(0); S471(0)]	0.514	-0.960159735	0.783976635
Q8N3X1	2xPhospho [S499(100); S508(100)]	100	6.64385619	8.35E-17
Q8N3X1	1xPhospho [T104(0); Y113(0); S116(100); S124(0)]	1.764	0.818850561	0.57131063
Q8N3X1	2xPhospho [S499(100); S508(100)]	1.361	0.444667067	0.855534442
Q8N3X1	2xPhospho [S499(100); S508(100)]	0.689	-0.537424112	0.907632224
Q8N3X1	1xPhospho [S18(100)]	1.218	0.284514133	0.927934489
Q8N488	2xPhospho [S115(0); S123(0); S127(100); S130(100); T134(0); T135(0)]	0.542	-0.883635243	0.751204811
Q8N4C8	3xPhospho [T319(95.8); Y321(0.9); Y323(8.9); S324(76.6); S326(8.9); S332(8.9); S340(0); S341(0); S349(50); T350(50)]	0.01	-6.64385619	8.35E-17
Q8N4C8	2xPhospho [S772(0.1); S773(0.1); S777(49.9); S778(49.9); S782(100)]	0.516	-0.954557029	0.786760931
Q8N4C8	1xPhospho [S699(0); S701(100); Y706(0)]	0.572	-0.805912948	0.864507734
Q8N4C8	2xPhospho [S747(0.1); S749(99.9); S754(0); S763(100)]	0.723	-0.467932448	0.865762755
Q8N4C8	1xPhospho [S772(0); S773(0); S777(1.7); S778(98.3); S782(0)]	0.713	-0.488026018	0.884551994
Q8N4C8	1xPhospho [T715(0); S732(97.4); S733(2.6)]	1.142	0.191562651	0.9616322
Q8N4C8	1xPhospho [S747(0); S749(0); S754(0); S763(100)]	0.773	-0.371459681	0.965139891
Q8N4S9	2xPhospho [Y108(0); S110(0); S116(100); S120(100)]	0.587	-0.768567592	0.640721946
Q8N4T0	2xPhospho [S60(100); Y61(100)]	0.272	-1.878321443	0.017926983
Q8N556	1xPhospho [S661(0); T663(0); S664(0); S665(0); S668(100)]	1.856	0.89219671	0.543749652
Q8N556	2xPhospho [S336(0.2); T337(0.2); T341(97.6); S342(51); S343(51); T350(0); Y353(0); S358(0); S360(0)]	1.489	0.574343754	0.790280431
Q8N567	1xPhospho [S28(100); T32(0); S33(0)]	1.279	0.355016264	0.878812277
Q8N567	1xPhospho [S28(100); T32(0); S33(0)]	1.243	0.313826296	0.879063032
Q8N5F7	2xPhospho [S157(100); T161(100)]	1.439	0.525066592	0.797286127
Q8N5F7	1xPhospho [T57(0); S64(100); S67(0)]	0.794	-0.332789088	0.956734015
Q8N5F7	1xPhospho [S149(100)]	0.772	-0.373327247	0.965139891
Q8N5G2	1xPhospho [S305(100); T306(0)]	0.726	-0.461958547	0.94797789
Q8N5P1	1xPhospho [S70(0); Y73(0); Y76(0); S77(100); S83(0); S86(0)]	1.348	0.430820497	0.855816069
Q8N5U6	1xPhospho [S108(0); S109(0); S110(100)]	0.879	-0.18606493	1
Q8N655	1xPhospho [S738(100); S741(0); S745(0)]	0.654	-0.612637459	0.93019184
Q8N655	1xPhospho [S259(0); T263(0); S264(0); S273(100)]	0.914	-0.12973393	0.998448514
Q8N6H7	1xPhospho [T131(0); S139(0); S140(0); S146(100)]	1.258	0.331131922	0.907632224
Q8N6H7	1xPhospho [S364(0); S368(100); S371(0)]	1.084	0.116364757	0.956843841
Q8N6H7	1xPhospho [S337(6.2); S338(6.2); S340(87.6); T350(0); S353(0)]	0.714	-0.486004021	0.958702638
Q8N6N3	1xPhospho [T155(0); S158(90.7); T166(4.6); S167(4.6)]	1.337	0.418999465	0.889528604
Q8N6N3	1xPhospho [T155(0); S158(100); T166(0); S167(0)]	1.091	0.125651102	0.980895643
Q8N7R7	2xPhospho [S105(100); S112(99.6); T117(0.4)]	0.01	-6.64385619	8.35E-17
Q8N7R7	2xPhospho [S105(100); S112(100); T117(0)]	2.593	1.374622206	0.318252841
Q8N7R7	1xPhospho [S342(0); S344(100)]	0.669	-0.579921884	0.863668848
Q8N7R7	1xPhospho [S105(0); S112(100); T117(0)]	1.2	0.263034406	0.929708014
Q8N8E3	2xPhospho [S169(0); S171(100); S173(2.5); T175(97.5); T184(0)]	0.932	-0.10159814	0.993813933
Q8N8S7	2 2xPhospho [T500(0); T502(96.2); S506(3.9); S508(99.8); S512(0)]	100	6.64385619	8.35E-17
Q8N8S7	2 1xPhospho [S463(4.8); T464(95.2); T467(0); S477(0); T481(0); S482(0)]; Q8N8S7 1xPhospho [S463(4.8); T464(95.2); T467(0); S477(0); T481(0); S482(0)]	100	6.64385619	8.35E-17
Q8N8S7	1xPhospho [S528(99.9); Y529(0.1); S531(0)]	2.798	1.484395963	0.326165136
Q8N8S7	2 1xPhospho [T500(0); T502(0); S506(1.6); S508(98.4); S512(0)]	2.339	1.225891862	0.535347951
Q8N8S7	2 1xPhospho [S125(100); S136(0)]; Q8N8S7 1xPhospho [S125(100); S136(0)]	1.202	0.265436896	0.907609406

Q8N9M1	2xPhospho [T144(0); S146(0); S151(100); S154(0); T155(0.2); T162(90.9); S163(4.3); T164(4.3); S165(0.2)]	0.841	-0.249822294	0.955123908
Q8N9M1	2xPhospho [T144(0); S146(0); S151(99.8); S154(4.5); T155(95.8)]	1.056	0.078609835	0.977858961
Q8N9T8	2xPhospho [S628(0); S634(100); S645(100)]	6.776	2.760433875	0.001597636
Q8N9T8	2xPhospho [S628(0); S634(100); S645(100)]	0.393	-1.347398782	0.12853832
Q8N9T8	2xPhospho [S177(100); S188(0.1); S189(100)]	1.45	0.5360529	0.765142684
Q8N9T8	2xPhospho [S628(0); S634(100); S645(100)]	0.648	-0.625934282	0.7717666
Q8N9T8	2xPhospho [S628(0); S634(100); S645(100)]	0.733	-0.448114897	0.855534442
Q8N9T8	2xPhospho [Y255(0); S286(100); S287(100)]	1.154	0.206643224	0.942574721
Q8N9T8	3xPhospho [S628(100); S634(100); S645(100)]	1.117	0.159629186	0.965139891
Q8N9T8	1xPhospho [S177(100); S188(0); S189(0)]	0.961	-0.057391664	0.996295697
Q8NA72	2xPhospho [S65(0); T66(0); S73(100); S78(100)]	0.537	-0.897006007	0.845620602
Q8NA72	1xPhospho [S65(0); T66(0); S73(0); S78(100)]	0.675	-0.567040593	0.933760225
Q8NA72	2xPhospho [S559(2.9); T562(94.2); S564(2.9); T566(0); S567(0.1); S570(99.9)]	1.017	0.024319679	0.967050102
Q8NAV1	2xPhospho [S182(0); S193(100); S194(100)]	0.43	-1.217591435	0.457443685
Q8NAV1	2xPhospho [S182(0); S193(100); S194(100)]	0.653	-0.614845103	0.690107651
Q8NAV1	1xPhospho [S11(99.9); T15(0.1); Y19(0)]	1.349	0.431890348	0.862999305
Q8NAV1	2xPhospho [S182(0); S193(100); S194(100)]	0.773	-0.371459681	0.868578958
Q8NAX2	2xPhospho [S213(99.7); Y216(0); Y217(0); S218(50.1); S222(50.1); S231(0); S233(0); S235(0); S236(0)]	1.629	0.703986604	0.678654866
Q8NAX2	2xPhospho [T178(50.1); S179(50.1); S186(99.8)]	0.517	-0.951763814	0.701261726
Q8NBJ4	1xPhospho [T299(0); S307(0); S309(100)]	0.539	-0.891642822	0.750758832
Q8NBN3	1xPhospho [S527(0); S528(0); T530(0); S540(100)]	0.935	-0.09696173	0.992376868
Q8NBU5	2xPhospho [Y314(0); S317(99.8); T318(33.5); S319(33.4); S322(33.4)]	0.772	-0.373327247	0.93019184
Q8NBU5	1xPhospho [Y314(0); S317(0); T318(0); S319(0); S322(100)]	1.045	0.063502942	0.972363997
Q8NC44	2xPhospho [T279(99.7); S281(50.2); S283(50.2); S291(0)]	0.01	-6.64385619	8.35E-17
Q8NC44	3xPhospho [T279(100); S281(100); S283(100); S291(0)]	0.501	-0.997117491	0.591305389
Q8NC51	1xPhospho [S328(3.2); S330(96.8); S338(0)]; Q8NC51-3 1xPhospho [S313(3.2); S315(96.8); S323(0)]	100	6.64385619	8.35E-17
Q8NC51	3 1xPhospho [T232(0); S237(100); T240(0); T243(0); T255(0)]	100	6.64385619	8.35E-17
Q8NC51	1xPhospho [S85(100)]; Q8NC51-3 1xPhospho [S85(100)]	0.22	-2.184424571	0.019397976
Q8NC51	1xPhospho [S330(0); S338(100)]; Q8NC51-3 1xPhospho [S315(0); S323(100)]	3.044	1.605968359	0.033606896
Q8NC51	1xPhospho [S243(100); Y244(0); Y246(0); S247(0); S252(0); T255(0); T258(0); T270(0)]	3.064	1.615416297	0.126405008
Q8NC51	2xPhospho [S197(97); S199(3); S202(50); S203(50); S205(0); Y207(0); S208(0)]; Q8NC51-3 2xPhospho [S197(97); S199(3); S202(50); S203(50); S205(0); Y207(0); S208(0)]	1.851	0.888304895	0.406373833
Q8NC51	3xPhospho [S197(100); S199(100); S202(93.5); S203(6.1); S205(0.4); Y207(0); S208(0)]; Q8NC51-3 3xPhospho [S197(100); S199(100); S202(93.5); S203(6.1); S205(0.4); Y207(0); S208(0)]	1.905	0.929790998	0.496087995
Q8NC51	2xPhospho [S328(0.1); S330(99.9); S338(100)]; Q8NC51-3 2xPhospho [S313(0.1); S315(99.9); S323(100)]	1.676	0.745022149	0.540374696
Q8NC51	2xPhospho [S197(0); S199(100); S202(0); S203(98.4); S205(1.6); Y207(0); S208(0)]; Q8NC51-3 2xPhospho [S197(0); S199(100); S202(0); S203(98.4); S205(1.6); Y207(0); S208(0)]	1.437	0.523060062	0.75891781
Q8NC51	2xPhospho [S328(4.1); S330(96); S338(100)]; Q8NC51-3 2xPhospho [S313(4.1); S315(96); S323(100)]	1.418	0.503857533	0.783976635
Q8NC51	1xPhospho [S202(0); S203(100); S205(0); Y207(0); S208(0)]; Q8NC51-3 1xPhospho [S202(0); S203(100); S205(0); Y207(0); S208(0)]	1.348	0.430820497	0.838938376
Q8NC51	3xPhospho [S197(0); S199(100); S202(0); S203(100); S205(100); Y207(0); S208(0)]; Q8NC51-3 3xPhospho [S197(0); S199(100); S202(0); S203(100); S205(100); Y207(0); S208(0)]	0.738	-0.438307279	0.872730156
Q8NC51	3 1xPhospho [T232(93.7); S237(6.3); T240(0); T243(0); T255(0)]	1.195	0.257010618	0.934569753
Q8NC51	1xPhospho [S85(100)]; Q8NC51-3 1xPhospho [S85(100)]	1.145	0.195347598	0.938029982
Q8NC51	3 2xPhospho [T232(0); S237(100); T240(100); T243(0); T255(0)]	1.07	0.097610797	0.953220244
Q8NC51	2xPhospho [S202(50); S203(50); S205(0); Y207(0.1); S208(99.9)]; Q8NC51-3 2xPhospho [S202(50); S203(50); S205(0); Y207(0.1); S208(99.9)]	1.049	0.069014678	0.971220368
Q8NC51	2xPhospho [S202(0.1); S203(99.9); S205(0); Y207(97.8); S208(2.3)]; Q8NC51-3 2xPhospho [S202(0.1); S203(99.9); S205(0); Y207(97.8); S208(2.3)]	1.09	0.124328135	0.977573898
Q8NC51	2xPhospho [T388(99.8); S391(0.1); S392(0.1); S394(100)]; Q8NC51-3 2xPhospho [T373(99.8); S376(0.1); S377(0.1); S379(100)]	0.945	-0.081613766	0.977860291
Q8NC51	3xPhospho [S197(4.8); S199(95.3); S202(48.8); S203(48.8); S205(6.7); Y207(91.1); S208(4.5)]; Q8NC51-3 3xPhospho [S197(4.8); S199(95.3); S202(48.8); S203(48.8); S205(6.7); Y207(91.1); S208(4.5)]	0.784	-0.351074441	0.979214627
Q8NCD3	1xPhospho [S473(100); T482(0)]	1.432	0.518031493	0.849882438
Q8NCF5	2xPhospho [Y147(0); S160(0); S161(0); Y164(0); S168(0.6); S170(99.4); S173(100)]	0.72	-0.473931188	0.904774194
Q8NCF5	2xPhospho [T42(0); S47(0); T52(100); S54(100); T63(0)]	0.856	-0.224317298	0.972363997
Q8NCN4	3xPhospho [S366(0); S368(100); S371(0); S374(0); S376(0); S388(100); T391(100)]	0.742	-0.430508908	0.924114144
Q8ND24	1xPhospho [S511(100); S516(0); S519(0); S530(0)]	1.247	0.318461465	0.900153261
Q8ND56	1xPhospho [S216(100); S219(0); T220(0); S225(0); S227(0)]	1.284	0.360645202	0.879063032
Q8ND56	2xPhospho [S192(95.3); T194(4.7); T201(0.3); S203(99.8)]	1.262	0.33571191	0.897258645
Q8ND56	1xPhospho [S216(100); S219(0); T220(0); S225(0); S227(0)]	1.242	0.312665174	0.920957334
Q8ND56	2xPhospho [S216(100); S219(0); T220(0.1); S225(96.7); S227(3.2)]	1.21	0.275007047	0.93019184
Q8ND56	2xPhospho [T175(0); S178(50.7); S182(50.7); S183(98.6)]	0.836	-0.258425153	0.930641583
Q8ND56	2xPhospho [S182(50); S183(50); S192(95.2); T194(4.8); T201(0); S203(0)]	0.85	-0.234465254	0.939268934
Q8ND56	1xPhospho [S192(100); T194(0); T201(0); S203(0)]	0.825	-0.277533976	0.944356286
Q8ND56	2xPhospho [S192(99.9); T194(0.2); T201(0); S203(100)]	1.155	0.207892852	0.945786032
Q8ND56	1xPhospho [S192(99.9); T194(0.1); T201(0); S203(0)]	1.153	0.205392513	0.952591019
Q8ND56	1xPhospho [S182(1.6); S183(98.5)]	1.179	0.237563718	0.958702638

Q8ND56	2xPhospho [S216(100); S219(100); T220(0); S225(0); S227(0)]	1.153	0.205392513	0.9616322
Q8ND56	2xPhospho [S182(50); S183(50); S192(100); T194(0); T201(0); S203(0)]	1.1	0.137503524	0.972363997
Q8ND56	2xPhospho [T175(0); S178(100); S182(2.4); S183(97.6)]	0.943	-0.084670324	0.989554868
Q8ND56	1xPhospho [S192(100); T194(0); T201(0); S203(0)]	0.98	-0.029146346	0.99184412
Q8ND76	2xPhospho [S21(100); S25(100); Y26(0); T30(0); S33(0)]	0.01	-6.64385619	8.35E-17
Q8ND76	1xPhospho [S83(100)]	0.517	-0.951763814	0.832667878
Q8ND76	2xPhospho [S324(0); S326(100); T331(100)]	0.537	-0.897006007	0.838893449
Q8ND76	1xPhospho [S324(0); S326(100); T331(0)]	1	0	0.99184412
Q8ND82	2xPhospho [T568(1.2); T569(32.9); S570(32.9); T571(32.9); S573(100); T577(0); S578(0)]	1.721	0.783247097	0.658368583
Q8NDI1	2xPhospho [S426(2.7); T427(94.5); S428(2.7); S432(0.1); S436(100)]	1.784	0.835115615	0.548969374
Q8NDI1	3xPhospho [S426(1.6); T427(49.2); S428(49.2); S432(100); S436(100)]	0.57	-0.810966176	0.704203351
Q8NDT2	1xPhospho [T532(100); Y538(0); S539(0)]	1.435	0.521050737	0.774382855
Q8NDT2	2xPhospho [S265(100); S267(100)]	1.178	0.236339539	0.933107775
Q8NDT2	2xPhospho [S265(100); S267(100)]	0.94	-0.089267338	0.990024011
Q8NDT2	2xPhospho [S265(100); S267(100)]	0.939	-0.090802937	1
Q8NDX1	1xPhospho [S1017(0); S1019(100); S1020(0); S1022(0); T1030(0); T1031(0)]	0.876	-0.190997225	0.99885857
Q8NDX5	2xPhospho [T609(100); T614(0); S616(100); T621(0)]	0.831	-0.267079618	0.965795497
Q8NDX5	1xPhospho [S315(100); S318(0)]	0.893	-0.16326792	0.991003044
Q8NDX5	2xPhospho [T609(100); T614(0); S616(100); T621(0)]	0.923	-0.115597447	0.999263007
Q8NE71	3xPhospho [S105(100); T108(100); S109(100)]	0.458	-1.126580497	0.151253896
Q8NE71	3xPhospho [S105(100); T108(100); S109(100)]	0.29	-1.785875195	0.161607137
Q8NE71	2xPhospho [S105(0); T108(100); S109(100)]	0.539	-0.891642822	0.352392338
Q8NE71	2xPhospho [S105(0); T108(100); S109(100)]	0.734	-0.446148032	0.810156821
Q8NE71	1xPhospho [S140(100)]	0.718	-0.477944251	0.91490394
Q8NE71	1xPhospho [S105(0); T108(0); S109(100)]	1.093	0.128293401	0.964978421
Q8NE71	1xPhospho [S285(0); S287(100); S292(0); S293(0)]	0.788	-0.343732465	0.970944072
Q8NEF9	2xPhospho [T199(0); S203(100); S205(0); S208(0); S212(99.9); S215(0.1); S218(0)]	0.709	-0.496142467	0.919907076
Q8NEF9	1xPhospho [S367(100)]	0.849	-0.236163541	0.983336192
Q8NEG4	3xPhospho [S458(0.3); S459(99.7); S461(4.6); T462(4.6); T464(90.9); S465(100); T471(0)]	0.443	-1.174621396	0.496427674
Q8NEG4	2xPhospho [Y314(0); S315(0); S316(100); T317(100)]	0.692	-0.531156057	0.874801809
Q8NEG4	1xMet-loss+Acetyl [N-Term]; 1xPhospho [S4(100)]	0.708	-0.498178735	0.93019184
Q8NEJ9	2xPhospho [S142(100); S143(100); S156(0); S159(0)]	0.01	-6.64385619	8.35E-17
Q8NEJ9	2xPhospho [S135(100); S139(100); S142(0); S143(0); S156(0); S159(0)]	1.283	0.35952117	0.879063032
Q8NEJ9	2xPhospho [S135(0); S139(0.2); S142(99.8); S143(100); S156(0); S159(0)]	1.2	0.263034406	0.901860353
Q8NEJ9	2xPhospho [S135(95.9); S139(49); S142(4.2); S143(51); S156(0); S159(0)]	1.214	0.279768422	0.930376591
Q8NEL9	2xPhospho [S217(0); T219(0); S223(0.1); S224(99.9); S225(100)]	0.835	-0.260151897	0.980895643
Q8NEL9	2xPhospho [T710(0); S711(0); S713(0); S719(0); T720(0); S723(100); T726(0.1); S727(99.9); S731(0)]	0.942	-0.086201035	0.980895643
Q8NEN9	1xPhospho [T527(0); T528(0); S530(0); S538(100)]	100	6.64385619	8.35E-17
Q8NEN9	1xPhospho [S517(0); S519(0); S521(100)]	0.678	-0.560642822	0.920957334
Q8NEY1	2 1xPhospho [S522(0); S528(100)]	0.01	-6.64385619	8.35E-17
Q8NEY1	2 2xPhospho [S1012(3.4); S1014(96.7); T1017(0); Y1025(0); S1026(0); S1028(0); S1032(3.4); T1033(96.5); S1035(0.1)]	0.37	-1.434402824	0.147078079
Q8NEY1	2 2xPhospho [S522(100); S528(100)]	0.268	-1.899695094	0.228891308
Q8NEY1	2 2xPhospho [S194(100); S199(100); S205(0); S206(0)]	0.52	-0.943416472	0.763961331
Q8NEY1	2 1xPhospho [S1262(2.9); S1263(2.9); T1264(94); S1265(0.1); S1266(0.1); S1267(0); T1270(0); T1273(0); T1283(0)]	0.558	-0.841662973	0.852940464
Q8NEY1	2 1xPhospho [S806(2); S808(98); S813(0); S814(0); S815(0); T817(0); T818(0); S821(0)]	0.693	-0.529072743	0.925808485
Q8NEY1	2 3xPhospho [S1046(100); S1048(99.7); T1053(0.2); S1059(0.2); S1062(100); S1065(0); S1066(0); S1068(0); S1069(0); T1070(0); Y1071(0); S1072(0); S1073(0)]	1.136	0.183962835	0.931390144
Q8NEY1	2 1xPhospho [S452(100); T453(0)]	0.862	-0.214240226	0.990182098
Q8NEY1	2 2xPhospho [S1046(50); S1048(50); T1053(0); S1059(100); S1062(0); S1065(0); S1066(0); S1068(0); S1069(0); T1070(0); Y1071(0); S1072(0); S1073(0)]	0.946	-0.080087911	1
Q8NFC6	1xPhospho [T1529(1.2); S1531(98.8)]	0.01	-6.64385619	8.35E-17
Q8NFC6	2xPhospho [S2973(96.4); S2975(3.6); S2986(100); S2999(0); T3002(0); T3003(0)]	0.01	-6.64385619	8.35E-17
Q8NFC6	2xPhospho [Y2777(0); S2779(100); S2780(100); S2793(0)]	0.406	-1.300448367	0.339102696
Q8NFC6	1xPhospho [S2750(0); S2753(100)]	1.325	0.40599236	0.863668848
Q8NFC6	2xPhospho [Y480(0); Y481(0); S482(100); S484(100); T489(0)]	1.261	0.334568276	0.897062575
Q8NFH5	2xPhospho [S93(0); Y95(0); S99(50); S100(50); S105(3.6); T106(3.6); T109(89.1); S110(3.6)]	100	6.64385619	8.35E-17
Q8NFH5	1xPhospho [S66(0); S73(100)]	100	6.64385619	8.35E-17
Q8NFH5	2xPhospho [S258(0); S259(99.9); S261(0.1); T265(100)]	0.977	-0.033569533	0.969482098
Q8NFH5	2xPhospho [S66(100); S73(100)]	1.025	0.03562391	0.97998947
Q8NG27	1xPhospho [S265(100)]	0.952	-0.070966521	0.980895643
Q8NG50	2xPhospho [S89(100); T97(100)]	0.901	-0.150400989	1
Q8NHG8	2xPhospho [S145(100); S151(100)]	0.18	-2.473931188	0.044699055
Q8NHH9	1xPhospho [T23(4.6); S24(95.2); S27(0.2); S34(0); S35(0); T36(0); T37(0); S38(0); Y43(0); S51(0)]	100	6.64385619	8.35E-17
Q8NHM5	2xPhospho [T961(0); S964(0); S975(100); S979(100)]	1.207	0.271425676	0.933760225
Q8NHQ9	1xPhospho [S544(100); T558(0)]	1.231	0.299830762	0.929708014
Q8NHQ9	1xPhospho [T588(0); S594(100)]	1.033	0.046840254	0.975403456
Q8NI08	3xPhospho [S170(7.9); S171(76.2); S172(8); S173(7.9); T177(8.2); S179(77.8); S181(14.7); S182(14.7); S183(84.5); Y187(0)]	0.01	-6.64385619	8.35E-17
Q8NI08	1xPhospho [Y87(0); Y88(0); S89(100)]	0.01	-6.64385619	8.35E-17
Q8NI27	1xPhospho [T1414(0); S1417(100); S1419(0); S1421(0); S1422(0); T1423(0)]	5.309	2.408440141	0.022909641
Q8NI27	1xPhospho [T1414(0); S1417(100); S1419(0); S1421(0); S1422(0); T1423(0)]	1.63	0.704871964	0.819921743

Q8NI27	2xPhospho [S1199(0); S1209(0); S1210(0); S1211(0); S1212(0); S1215(0); S1217(0.3); S1219(99.4); S1222(94.9); S1223(5.1); T1224(0.3); T1227(0)]	0.936	-0.095419565	1
Q8NI35	2xPhospho [T1209(100); S1212(100); T1222(0)]	0.01	-6.64385619	8.35E-17
Q8TAA9	2xPhospho [S338(100); S339(100); Y344(0); Y345(0)]	0.01	-6.64385619	8.35E-17
Q8TAA9	2xPhospho [S42(100); S45(100); T47(0); T52(0); S61(0); T62(0)]	0.601	-0.734563104	0.665992616
Q8TAA9	2xPhospho [S42(100); S45(96.9); T47(3.1); T52(0); S61(0); T62(0)]	0.66	-0.59946207	0.822894134
Q8TAD8	1xPhospho [S49(0); S52(100); S54(0); T57(0); S58(0)]	2.447	1.291014101	0.363377543
Q8TAD8	1xPhospho [S35(100)]	1.685	0.752748591	0.604301121
Q8TAD8	1xPhospho [S200(0); S202(100)]	0.515	-0.957355663	0.656356957
Q8TAD8	1xPhospho [S35(100)]	1.162	0.216610069	0.934211842
Q8TAP8	2xPhospho [S45(0); S47(100); S52(100)]	0.529	-0.918660373	0.813170869
Q8TAP8	1xPhospho [S45(0); S47(0); S52(100)]	1.197	0.259423152	0.897258645
Q8TAP8	2xPhospho [T84(100); S87(100)]	0.802	-0.318325858	0.973263372
Q8TAP9	1xPhospho [S93(0); S97(100); Y103(0); S104(0); T110(0); S115(0)]	1.285	0.361768359	0.872730156
Q8TAQ2	1xPhospho [T276(0); T278(0); S283(100); S286(0.1)]	100	6.64385619	8.35E-17
Q8TBA6	1xPhospho [S116(100); S129(0); S130(0)]	0.628	-0.671163536	0.923810998
Q8TBB5	2xPhospho [S413(100); S418(100); S424(0)]	1.741	0.799916203	0.644556338
Q8TBB5	2xPhospho [T403(0); S407(0.3); S413(99.7); S418(100); S424(0)]	1.133	0.180147861	0.971220368
Q8TBC3	2xPhospho [S628(0); T630(0); S631(0); S634(2.4); S636(49.9); S637(49.9); T639(93.2); S640(4.4); S642(0.2)]	0.957	-0.06340917	0.980895643
Q8TBE0	2xPhospho [S180(100); S184(100)]	1.309	0.388465097	0.883976868
Q8TBZ3	1xPhospho [S432(0); S434(100); S438(0); S441(0); S445(0)]	1.375	0.459431619	0.811560495
Q8TBZ3	2xPhospho [S357(100); T358(0); S360(100); S364(0); T366(0); Y367(0)]	0.881	-0.182786076	0.954539739
Q8TBZ3	1xPhospho [S357(100); T358(0); S360(0); S364(0); T366(0); Y367(0)]	0.843	-0.246395464	0.99184412
Q8TBZ3	1xPhospho [S491(100); S492(0); T499(0)]	0.942	-0.086201035	1
Q8TC07	1xPhospho [S675(100); T677(0); S682(0); S683(0)]	1.402	0.487486349	0.850252723
Q8TCJ2	1xPhospho [S498(96.8); S499(3.2); Y511(0)]	6.836	2.773152397	0.001768655
Q8TCJ2	2xPhospho [S498(100); S499(100); Y511(0)]	0.754	-0.407363571	0.904227116
Q8TD16	1xPhospho [S582(100)]	1.21	0.275007047	0.912353352
Q8TD19	1xPhospho [S13(0); S16(0); S20(0); S22(0); S28(0); S29(100); S33(0); S35(0)]	1.066	0.092207438	0.973263372
Q8TD19	2xPhospho [S855(100); S868(0); S869(100)]	0.922	-0.117161344	0.99277609
Q8TDB6	1xMet-loss+Acetyl [N-Term]; 1xPhospho [S3(0); S9(100)]	0.505	-0.985644707	0.769621572
Q8TDD1	2xPhospho [S34(0); S39(100); S41(100)]	3.353	1.745452483	0.062606323
Q8TDD1	2xPhospho [T67(0); T70(0.4); S71(99.6); T74(0.4); S75(99.6); T81(0)]	1.148	0.199122642	0.933760225
Q8TDD1	2xPhospho [S39(100); S41(100)]	0.884	-0.177881725	0.97726974
Q8TDD1	1xPhospho [T67(0); T70(0); S71(0); T74(0); S75(100); T81(0)]	0.948	-0.077041036	1
Q8TDM6	2xPhospho [T984(100); Y990(0); S1000(100); S1004(0)]	1.338	0.420078116	0.855534442
Q8TEA8	1xPhospho [S194(0); S196(0); S197(100); S204(0); S205(0)]	1.283	0.35952117	0.901788734
Q8TEH3	2xPhospho [T519(0); S520(100); S523(100); Y530(0)]	0.86	-0.217591435	0.993670231
Q8TEK3	2xPhospho [S899(50.6); T900(50.6); S902(98.8)]	1.672	0.741574847	0.723988927
Q8TEK3	2xPhospho [S997(0); S1001(100); S1007(0); S1008(2.1); S1009(97.9)]	1.02	0.028569152	0.973263372
Q8TEK3	2xPhospho [S826(100); S834(100)]	1.074	0.102993993	0.976595096
Q8TEM1	2xPhospho [S1848(100); S1852(100); Y1855(0); S1859(0); S1860(0); T1862(0); S1863(0)]	4.041	2.014712352	0.05926676
Q8TEQ6	1xPhospho [S770(0); S778(100); S800(0)]	0.091	-3.457989644	3.77E-05
Q8TEQ6	1xPhospho [S757(0); S765(0); S770(0); S778(100)]	1.067	0.093560176	0.973942845
Q8TEV9	2xPhospho [S487(2.4); S489(97.6); S492(100); T494(0); S498(0)]	0.908	-0.139235797	0.992371558
Q8TEV9	3xPhospho [S487(2.2); S489(97.8); S492(100); T494(0); S498(100)]	0.905	-0.144010303	0.99811985
Q8TF01	2xPhospho [S286(0); S290(100); T297(0); S304(98.4); S305(1.6)]	1.923	0.943358763	0.509659105
Q8TF01	1xPhospho [S211(100)]	1.471	0.556797247	0.780165563
Q8TF01	1xPhospho [S286(0); S290(100); T297(0); S304(0); S305(0)]	0.907	-0.140825544	0.99184412
Q8TF44	2xPhospho [S262(100); S264(0); S268(0); T269(0); S273(99.9); S276(0.1)]	1.509	0.593592806	0.759239289
Q8TF72	2xPhospho [S425(0); T434(0); S439(100); S443(100)]	0.611	-0.710755715	0.783976635
Q8TF72	1xPhospho [S242(98.8); T243(1.2); S245(0); S250(0)]	1.409	0.494671612	0.832208322
Q8TF72	2xPhospho [S910(100); S913(100); S921(0)]	0.672	-0.573466862	0.85992414
Q8TF72	1xPhospho [S660(0); S663(0); S664(100); S671(0)]	1.095	0.13093087	0.94797789
Q8TF72	2xPhospho [S1490(96); S1492(4); S1497(100); Y1504(0)]	1.089	0.123003954	0.962540894
Q8TF74	2xPhospho [T300(3.9); S301(96); S303(0.2); S305(96.1); S308(3.9)]	2.111	1.077926579	0.376723572
Q8TF74	2xPhospho [S128(100); S131(100); T138(0); S140(0); S141(0)]	1.993	0.99494171	0.464522487
Q8TF74	1xPhospho [S128(99.9); S131(0.1); T138(0); S140(0); S141(0)]	1.875	0.906890596	0.528481175
Q8TF74	1xPhospho [S155(100); S160(0); T164(0); T165(0); S166(0); S167(0); T168(0)]	1.634	0.708407983	0.668909458
Q8TF74	2xPhospho [S155(100); S160(0); T164(33.3); T165(33.3); S166(33.3); S167(0); T168(0)]	1.414	0.49978212	0.815248216
Q8WTW3	1xPhospho [S447(0); S454(0); S455(0); T456(0); S457(0); S459(100); S461(0)]	1.394	0.479230561	0.823038438
Q8WU17	2xPhospho [S625(10.6); S634(94.7); T635(94.7)]	0.01	-6.64385619	8.35E-17
Q8WU17	3xPhospho [S625(100); S634(100); T635(100)]	1	0	0.9699948
Q8WU17	1xPhospho [T652(0); T663(100)]	0.994	-0.008682243	0.996742019
Q8WU90	2xPhospho [S356(0); T357(0); Y358(0); T359(0); S360(0); S368(100); S371(100)]	0.98	-0.029146346	0.980895643
Q8WUA2	1xPhospho [T159(0); S178(99.9); T182(0.1)]	0.921	-0.118726939	0.972501767
Q8WUA7	1xPhospho [S165(2.7); S167(97.3); S171(0); T173(0); T175(0); T179(0); S180(0); S183(0); T184(0); S186(0); S187(0); S188(0); S191(0)]	1.007	0.010063683	0.992376868
Q8WUB8	1xPhospho [T318(0); S319(0); S321(0); S322(0); S323(0); S327(100); S331(0); S335(0); S339(0)]	2.295	1.198494154	0.321778521
Q8WUB8	2xPhospho [Y276(0); T281(0); Y284(0); S297(100); S301(100)]	1.283	0.35952117	0.860444802
Q8WUB8	3xPhospho [S50(100); S53(97); S54(51.2); S56(51.2); T59(0.2); S60(0.2); S61(0.2); S67(0); Y68(0); Y69(0); Y77(0)]	0.799	-0.323732592	0.962617576
Q8WUF5	1xPhospho [S526(100); T538(0)]	0.01	-6.64385619	8.35E-17

Q8WUM0	2xPhospho [S27(100); T28(100)]	100	6.64385619	8.35E-17
Q8WUM0	3xPhospho [S41(100); S44(50); S45(50); S50(100)]	1.221	0.2880632	0.906394924
Q8WUM4	1xPhospho [S718(0); S721(0); T724(0.4); Y727(0); S729(94); S730(5.6); T738(0); T741(0)]	0.482	-1.052894948	0.648285315
Q8WUM9	1xPhospho [S417(100); Y418(0); T419(0); S420(0); Y421(0); T422(0); S432(0)]	0.525	-0.929610672	0.604223334
Q8WUM9	2xPhospho [S463(100); Y464(0); T465(0); S466(100); Y467(0); S472(0); S476(0); S478(0); S483(0)]	0.613	-0.706041021	0.860444802
Q8WUM9	2xPhospho [S463(100); Y464(0); T465(0.1); S466(99.9); Y467(0); S472(0); S476(0); S478(0); S483(0)]	0.749	-0.416962376	0.933107775
Q8WUM9	2xPhospho [S265(100); S267(97.6); S269(2.5)]	0.722	-0.469929258	0.971196575
Q8WUX9	1xPhospho [S417(100)]	0.956	-0.064917477	0.977573898
Q8WUZ0	3xPhospho [T111(0); S114(100); T118(100); S122(0); S126(100)]	4.878	2.286289758	0.001578208
Q8WUZ0	2xPhospho [T111(0); S114(100); T118(0); S122(0); S126(100)]	3.419	1.773574423	0.00586842
Q8WUZ0	1xPhospho [S97(0.1); S100(99.9); S103(0); S106(0)]	2.417	1.273217473	0.301780562
Q8WUZ0	2xPhospho [S97(100); S100(100); S103(0); S106(0)]	1.087	0.12035194	0.965139891
Q8WVC0	3xPhospho [S246(100); S254(100); S261(100)]	2.657	1.409798228	0.249091856
Q8WVC0	1xPhospho [Y655(0); S658(100)]	2.209	1.143393419	0.402501756
Q8WVC0	2xPhospho [T188(100); S197(100)]	1.413	0.498761466	0.855557911
Q8WVC0	3xPhospho [S246(100); S254(100); S261(100)]	1.205	0.269033146	0.926103516
Q8WVC0	1xPhospho [S66(100); S72(0); T75(0); S77(0); S81(0)]	1.188	0.248534836	0.938029982
Q8WVD3	1xPhospho [S134(0.1); T136(2.2); S137(2.2); T138(2.2); S139(93.2); T142(0.1); T144(0); Y145(0); T149(0); S150(0); S151(0); S152(0); T156(0)]	0.853	-0.229382353	0.982345843
Q8WVE0	1xMet-loss+Acetyl [N-Term]; 1xPhospho [S2(100); T9(0); S13(0); Y24(0)]	100	6.64385619	8.35E-17
Q8WVK2	1xPhospho [S111(100); S114(0); T115(0)]	1.067	0.093560176	0.965139891
Q8WVT3	1xPhospho [S182(0); S184(100); S189(0); S192(0)]	100	6.64385619	8.35E-17
Q8WVV9	1xPhospho [S59(94.7); S61(5.3); S68(0)]	15.797	3.981578698	0.000513948
Q8WW12	1xPhospho [T139(0); T141(0); S142(0); S147(100)]	100	6.64385619	8.35E-17
Q8WW12	1xPhospho [S77(100); T79(0); T80(0)]	0.61	-0.713118852	0.715450341
Q8WW12	1xPhospho [T139(100); T141(0); S142(0); S147(0)]	1.489	0.574343754	0.749797187
Q8WW12	1xPhospho [S168(100)]	1.403	0.488515009	0.824541397
Q8WW12	1xPhospho [T106(0); S108(0); S119(100)]	1.198	0.260627908	0.918281661
Q8WW12	2xPhospho [T139(100); T141(0); S142(0); S147(100)]	1.192	0.253384236	0.938190912
Q8WW12	2xPhospho [T106(2.7); S108(97.3); S119(100)]	1.054	0.075874867	0.971220368
Q8WWI1	2xPhospho [S988(99.9); T990(0.2); S991(99.9); S994(0); S995(0); S997(0); T1003(0); S1004(0)]	0.189	-2.40354186	0.006746107
Q8WWI1	2xPhospho [T979(0); T984(0); S988(100); T990(3.3); S991(96.7); S994(0); S995(0); S997(0); T1003(0); S1004(0)]	0.293	-1.77102743	0.055125602
Q8WWI1	1xPhospho [S1510(100); S1516(0)]	0.52	-0.943416472	0.684145065
Q8WWI1	2xPhospho [T949(0); S955(94.1); T956(6); S960(100); S964(0); S968(0); S969(0); S971(0); S972(0)]	1.074	0.102993993	0.960298686
Q8WWM7	1xPhospho [S390(0); S391(0); S396(100)]	2.701	1.43349364	0.071235755
Q8WWM7	1xPhospho [T209(0); S211(100); S217(0)]	1.678	0.746742716	0.658368583
Q8WWM7	2xPhospho [T600(0); S602(0); S604(0); S615(0); T618(0); S630(50.1); T632(50.1); S634(99.8)]	1.34	0.422233001	0.801580268
Q8WWM7	3xPhospho [T600(5.6); S602(94.1); S604(0.3); S615(0); T618(0); S630(51.5); T632(51.5); S634(97.1)]	0.635	-0.655171503	0.878612438
Q8WWM7	1xPhospho [S111(100); Y118(0); S121(0)]	1.045	0.063502942	0.987464758
Q8WWM7	1xPhospho [T588(0); S589(0); S594(100); S597(0); S598(0)]	0.951	-0.072482754	0.99184412
Q8WWQ0	1xPhospho [T1775(0); Y1778(0); S1783(100)]	2.083	1.058662839	0.450898808
Q8WWQ0	2xPhospho [S674(99.6); S676(0.5); S677(33.3); T678(33.3); S679(33.3); S683(0.1)]	0.586	-0.77102743	0.862506613
Q8WWQ0	2xPhospho [S1281(100); S1283(100); T1295(0); S1296(0); T1297(0)]	1.254	0.326537348	0.878612438
Q8WWQ0	2xPhospho [S1281(100); S1283(100); T1295(0); S1296(0); T1297(0)]	0.787	-0.345564459	0.925912375
Q8WWQ0	1xPhospho [T1557(0); S1559(0); S1560(0); S1564(0); S1565(1.8); S1567(98.2); T1570(0)]	1.158	0.211635253	0.937563451
Q8WWQ0	1xPhospho [S674(100); S676(0); S677(0); T678(0); S679(0); S683(0)]	1.105	0.14404637	0.96229367
Q8WX92	1xPhospho [S557(100); T564(0); S573(0)]	0.933	-0.100051014	0.99184412
Q8WX93	1xPhospho [S632(0); T635(0); S641(100); T643(0)]	0.01	-6.64385619	8.35E-17
Q8WX93	3xPhospho [S684(100); S688(100); T704(0); S708(100); S713(0); S717(0)]	0.211	-2.244685096	0.098509956
Q8WX93	3xPhospho [S684(99.6); S688(0.4); T704(100); S708(100); S713(0); S717(0)]	0.346	-1.531156057	0.126276713
Q8WX93	2xPhospho [S747(7); T751(93); S754(92.1); S755(6.9); S757(0.5); S758(0.5); S759(0); S760(0); S763(0); S766(0); T768(0)]	0.455	-1.13606155	0.357846086
Q8WX93	1xPhospho [S979(0); S984(100); Y990(0)]	0.449	-1.15521265	0.524403093
Q8WX93	1xPhospho [S719(0); S725(0); S726(0); S728(100); Y731(0)]	1.694	0.760433875	0.639406282
Q8WX93	2xPhospho [S979(100); S984(100); Y990(0)]	1.098	0.134878054	0.958702638
Q8WX93	1xPhospho [S192(100); S197(0); S198(0); S199(0); S202(0); Y204(0); S206(0)]	0.773	-0.371459681	0.965351295
Q8WXE0	1xPhospho [S708(0); S711(0); S720(0); S725(100)]	100	6.64385619	8.35E-17
Q8WXE0	2xPhospho [S705(99.7); S708(94.6); S711(5.7); S720(0); S725(0)]	1.994	0.99566541	0.570285159
Q8WXE0	2xPhospho [S393(0); S396(0.7); S403(93.5); S406(99.2); S409(6.1); S412(0.4)]	0.833	-0.263611599	0.975558785
Q8WXE0	3xPhospho [S705(100); S708(100); S711(100); S720(0); S725(0)]	1	0	0.97998947
Q8WXF1	1xPhospho [S473(98.4); S477(1.6); S481(0)]	1.584	0.663572335	0.682660189
Q8WXF1	2xPhospho [T483(50); S485(50); T487(0); S493(100); S499(0)]	1.336	0.417920008	0.854283637
Q8WXF1	1xPhospho [T483(0); S485(0); T487(0); S493(100); S499(0)]	1.236	0.305678743	0.925853562
Q8WXF1	1xPhospho [S409(100)]	0.973	-0.03948829	0.980895643
Q8WXI9	3xPhospho [T120(97.4); S122(2.6); S129(100); S134(0.1); S135(99.9)]	0.591	-0.758769964	0.622018329
Q8WXI9	2xPhospho [T120(99.9); S122(0.1); S129(100); S134(0); S135(0)]	1.242	0.312665174	0.91490394
Q8WXI9	2xPhospho [T120(0); S122(0); S129(97.7); S134(4.7); S135(97.7)]	0.721	-0.471928835	0.93019184
Q8WY36	1xPhospho [S648(4.7); S649(95.3); S659(0); T661(0); S663(0); S665(0); T667(0); S668(0); S670(0)]	100	6.64385619	8.35E-17
Q8WY36	1xPhospho [T838(0); S844(100); T849(0)]	1.106	0.145351386	0.938029982
Q8WYA6	1xPhospho [Y536(0); S545(100)]	1.015	0.021479727	0.972363997

Q8WYB5	2xPhospho [S1581(100); T1586(0); T1587(0); S1594(88.3); S1597(5.9); S1598(5.9); S1601(0); S1602(0); S1605(0); S1610(0)]	0.706	-0.502259911	0.969482098
Q8WYL5	2xPhospho [S515(100); S521(100); T526(0); S528(0)]	1.877	0.90842865	0.582680589
Q8WYP5	2xPhospho [T1276(0); T1277(0); S1278(0); S1283(100); S1295(99.5); S1297(0.5)]	0.01	-6.64385619	8.35E-17
Q8WYP5	2xPhospho [S2222(100); S2226(100)]	1.836	0.876566059	0.647699093
Q8WYP5	3xPhospho [S1209(0); T1210(0); S1214(3.8); S1216(96.2); S1218(100); S1222(100)]	0.45	-1.152003093	0.679613588
Q8WYP5	1xPhospho [S1797(100); S1806(0); T1808(0)]	0.574	-0.800877358	0.862548271
Q8WYP5	1xPhospho [S1150(0); S1153(0); S1154(0); S1155(0); S1160(100); S1165(0)]	1.128	0.173767068	0.925853562
Q8WYP5	2xPhospho [S1209(0); T1210(0); S1214(0); S1216(100); S1218(0); S1222(100)]	0.761	-0.394031641	0.930376591
Q8WYP5	1xPhospho [S1541(100); Y1546(0); S1548(0); T1550(0)]	0.759	-0.397828209	0.962617576
Q8WYP5	1xPhospho [S1209(0); T1210(0); S1214(0); S1216(0); S1218(0.1); S1222(100)]	0.872	-0.19759996	0.985318278
Q8WYQ5	2xPhospho [Y267(0); S271(100); S275(100); T279(0); S280(0); T286(0)]	0.482	-1.052894948	0.636086521
Q8WYQ5	1xPhospho [S92(100); S95(0)]	0.69	-0.535331733	0.938190912
Q8WYQ5	2xPhospho [Y267(0); S271(100); S275(100); T279(0); S280(0); T286(0)]	1.136	0.183962835	0.965139891
Q8WYQ5	2xPhospho [S367(0); S368(0); T371(0); S373(100); S377(100); S383(0)]	0.986	-0.020340448	0.984970017
Q8WZ73	2xPhospho [T224(0); S226(100); S229(100); S232(0)]	0.835	-0.260151897	0.965795497
Q92466	2xPhospho [S24(100); S26(100)]	0.468	-1.095419565	0.56942912
Q92466	1xPhospho [S26(100)]	0.681	-0.554273297	0.955790722
Q92499	1xPhospho [T475(0); S481(100); S486(0)]	1.345	0.427606173	0.806224461
Q92538	1xPhospho [S345(4.9); S347(94.8); S349(0.3); S352(0); T361(0); S362(0); S367(0); S369(0); S371(0); Y377(0)]	0.01	-6.64385619	8.35E-17
Q92538	1xPhospho [S1475(100); S1486(0); Y1487(0); T1489(0); S1491(0); S1495(0); T1504(0); T1507(0)]	15.543	3.958193084	4.54E-06
Q92538	3xPhospho [S345(48.5); S347(48.5); S349(3); S352(100); T361(0); S362(0); S367(2.9); S369(48.5); S371(48.5); Y377(0)]	0.219	-2.190997225	0.095148535
Q92538	2xPhospho [S345(5.8); S347(93.9); S349(94.2); S352(6.1); T361(0); S362(0); S367(0); S369(0); S371(0); Y377(0)]	0.758	-0.399730246	0.853287961
Q92538	3xPhospho [S345(0.5); S347(99); S349(0.5); S352(100); T361(0); S362(0); S367(48.3); S369(48.3); S371(3.5); Y377(0)]	0.683	-0.550042516	0.855557911
Q92538	2xPhospho [S345(0); S347(50.1); S349(50.1); S352(99.9); T361(0); S362(0); S367(0); S369(0); S371(0); Y377(0)]	0.793	-0.334607229	0.952036332
Q92538	2xPhospho [S1298(100); S1300(0); S1304(0); Y1305(0); S1311(100)]	0.865	-0.209227962	0.971985173
Q92538	1xPhospho [Y1316(0); T1317(0); S1318(100); S1320(0); Y1323(0); T1324(0)]	0.953	-0.069451881	0.981208663
Q92538	2xPhospho [Y1316(0); T1317(0); S1318(99.5); S1320(0.9); Y1323(0); T1324(99.6)]	0.881	-0.182786076	0.990182098
Q92538	1xPhospho [S1298(100); S1300(0); S1304(0); Y1305(0); S1311(0)]	1.018	0.025737561	0.99885857
Q92541	1xPhospho [S697(100); Y703(0)]	0.781	-0.356605547	0.972501767
Q92574	1xPhospho [Y862(100)]	0.305	-1.713118852	0.038038726
Q92574	1xPhospho [S1100(100); S1102(0); T1110(0); S1111(0); S1112(0); S1114(0); S1116(0)]	0.568	-0.816037165	0.728446408
Q92575	1xPhospho [S165(0); T167(0); T169(0); S173(100); T178(0); S179(0); S180(0); S184(0); S187(0)]	1.137	0.185232254	0.93019184
Q92576	2xPhospho [T1610(0); S1611(0); S1613(0.1); S1614(99.9); S1618(100)]	8.11	3.019701914	0.001619201
Q92576	1xPhospho [S289(0); S299(100); S301(0)]	1.835	0.875780063	0.539112137
Q92576	1xPhospho [S1133(100)]	1.331	0.412510571	0.846352924
Q92576	1xPhospho [S1630(100); S1632(0); T1639(0)]	0.53	-0.915935735	0.854076968
Q92576	1xPhospho [S283(100)]	0.711	-0.492078535	0.965013037
Q92576	1xPhospho [T1610(0); S1611(0.1); S1613(2.4); S1614(97.6)]	1.106	0.145351386	0.965013037
Q92576	1xPhospho [T532(0); S537(100)]	1.031	0.044044333	0.971937853
Q92576	1xPhospho [S1642(100)]	0.883	-0.179514657	0.992911173
Q92609	2xPhospho [S537(0); S538(0); S539(0); S541(100); S544(100)]	1.123	0.167357928	0.956843841
Q92609	1xPhospho [S730(100); S732(0)]	1.031	0.044044333	0.973599548
Q92609	1xPhospho [S537(0); S538(0); S539(0); S541(100); S544(0)]	0.803	-0.316528107	0.973763863
Q92610	1xPhospho [S1250(0); S1255(0); S1260(0); T1262(0); S1264(100)]	2.185	1.12763328	0.267894422
Q92610	1xPhospho [S1089(100)]	1.497	0.582074221	0.749797187
Q92610	3xPhospho [S328(100); S331(100); S334(100); T339(0)]	1.463	0.548929769	0.819921743
Q92610	3xPhospho [S132(0); T137(6.4); S142(93.7); S145(100); S146(100)]	0.881	-0.182786076	0.965248196
Q92613	2xPhospho [Y761(0); Y767(0); S774(100); S776(100); S780(0)]	0.466	-1.10159814	0.58296191
Q92613	2xPhospho [S548(0); T549(0); S556(0.1); S557(49.9); T558(49.9); T560(0.1); S566(100); S569(0); S570(0); S571(0); S572(0); S575(0)]	0.791	-0.3382504	0.920957334
Q92614	2xPhospho [S1970(100); S1974(100)]	0.01	-6.64385619	8.35E-17
Q92614	3xPhospho [S89(0); S94(0.3); S98(94.3); T99(5.4); S101(100); S102(100); S103(0); S112(0)]	0.439	-1.187707155	0.48557376
Q92614	2xPhospho [S145(0); S149(0); S151(0); T153(0); S154(0); T155(0); S157(0); S160(100); S164(100)]	1.624	0.699551633	0.669102273
Q92614	2xPhospho [S2014(100); S2020(100); T2030(0); S2031(0)]	1.55	0.632268215	0.714106081
Q92614	2xPhospho [S89(0); S94(0); S98(0); T99(0); S101(100); S102(99.9); S103(0.1); S112(0)]	0.748	-0.418889825	0.921238122
Q92614	1xPhospho [S2014(0); S2020(100); T2030(0); S2031(0)]	0.796	-0.329159664	0.93019184
Q92614	1xPhospho [S145(100); S149(0); S151(0); T153(0); S154(0); T155(0); S157(0); S160(0); S164(0)]	0.705	-0.504304837	0.933107775
Q92614	1xPhospho [S72(0); S74(100); T79(0); S83(0); S85(0)]	0.661	-0.597277823	0.938029982
Q92614	2xPhospho [S1067(99.9); S1068(3.8); S1069(92.8); S1070(3.6); S1076(0)]	1.142	0.191562651	0.953220244
Q92614	1xPhospho [S140(100); S142(0.1)]	0.852	-0.231074664	0.960831064
Q92614	2xPhospho [Y2035(0); S2036(0); S2038(0); Y2039(0); S2041(100); S2043(100); T2045(0)]	0.948	-0.077041036	0.989838391
Q92621	2xPhospho [S1939(100); S1942(100); T1944(0)]	0.793	-0.334607229	0.978952227
Q92625	2xPhospho [T318(100); S327(0.2); S328(48.5); S331(48.5); S333(2.9)]	1.761	0.816394909	0.607772032
Q92625	1xPhospho [S661(0); S663(100); S666(0)]	0.56	-0.836501268	0.860444802
Q92625	2xPhospho [S661(0); S663(100); S666(100)]	1.003	0.004321606	0.972501767
Q92625	1xPhospho [S887(100); S895(0)]	1.05	0.070389328	0.979498769
Q92692	2 2xPhospho [S465(5.5); S470(94.8); S473(99.7)]	7.18	2.843983844	0.003093629
Q92733	2xPhospho [S157(100); S159(100); T165(0)]	0.777	-0.364013496	0.872730156

Q92733	2xPhospho [S157(100); S159(100); T165(0)]	0.895	-0.160040413	0.965139891
Q92733	1xPhospho [T261(0); S267(100); S278(0)]	1.027	0.038436182	0.980895643
Q92734	1xPhospho [S157(0); T158(0); S164(99.9); S166(0.1)]	1.804	0.851199339	0.566655737
Q92734	1xPhospho [S157(0); T158(0); S164(100); S166(0)]	1.878	0.909197063	0.576191064
Q92734	2xPhospho [S183(0); T188(0); S193(100); S197(100)]	0.799	-0.323732592	0.952591019
Q92734	1xPhospho [S183(0); T188(0); S193(0); S197(100)]	0.995	-0.007231569	0.985870803
Q92738	2xPhospho [S673(0); T674(0.1); S676(99.9); S678(50); S680(50)]	0.823	-0.281035664	0.980895643
Q92766	2xPhospho [S1585(82.8); S1589(5.7); T1590(5.7); S1592(5.7); S1598(100); S1609(0); T1615(0)]	100	6.64385619	8.35E-17
Q92766	2xPhospho [S1194(0); T1199(0); T1204(0); S1219(100); S1225(100)]	2.11	1.077242999	0.32984156
Q92766	2xPhospho [S175(100); S180(100)]	0.742	-0.430508908	0.965139891
Q92766	2xPhospho [T31(0); S36(100); S42(100); S44(0)]	1.029	0.041242982	0.973599548
Q92766	1xPhospho [T31(0); S36(0); S42(99.9); S44(0.1)]	0.924	-0.114035243	0.980895643
Q92793	2xPhospho [S2345(0); S2351(100); S2356(0); S2361(94); S2362(3); S2364(3)]	0.01	-6.64385619	8.35E-17
Q92793	1xPhospho [S121(100); S124(0); S128(0); S129(0); S132(0)]	0.754	-0.407363571	0.938029982
Q92793	2xPhospho [T974(83.6); S976(83.6); S977(16.4); S980(16.4); T983(0.1); S985(0)]	1.162	0.216610069	0.943939997
Q92794	1xPhospho [S1104(0); S1113(100); T1119(0); S1126(0)]	100	6.64385619	8.35E-17
Q92796	2 1xPhospho [S275(0); S283(0); S284(0); T291(0); S292(0); T294(0.1); S295(97.8); S297(2.2); S299(0); S300(0); S301(0)]	1.845	0.883620816	0.432935188
Q92796	2 2xPhospho [S275(100); S283(0); S284(0); T291(0); S292(0); T294(0.2); S295(99.7); S297(0.2); S299(0); S300(0); S301(0)]	0.872	-0.19759996	0.980895643
Q92797	2xPhospho [S494(0); S501(100); S505(100); S507(0); S508(0); S510(0)]	100	6.64385619	8.35E-17
Q92797	3xPhospho [S1243(99.4); T1246(50.6); T1257(50.6); S1259(99.4)]	100	6.64385619	8.35E-17
Q92797	2xPhospho [S494(100); S501(100); S505(0); S507(0); S508(0); S510(0)]	1.801	0.848798181	0.543749652
Q92797	3xPhospho [S1243(94.7); T1246(5.3); T1257(100); S1259(100)]	1.803	0.850399397	0.647620981
Q92797	2xPhospho [S1243(0); T1246(0); T1257(100); S1259(100)]	1.611	0.687956494	0.662331671
Q92797	1xPhospho [S13(96.5); S16(3.5); T20(0); T31(0); T32(0); S33(0)]	1.433	0.51903861	0.848670625
Q92797	1xPhospho [S1243(97.6); T1246(2.4)]	1.334	0.415758667	0.872730156
Q92797	3xPhospho [S1243(100); T1246(0); T1257(100); S1259(100)]	1.256	0.328836464	0.893481203
Q92797	1xPhospho [T1257(0); S1259(100)]	1.271	0.34596403	0.898100811
Q92797	3xPhospho [S494(100); S501(100); S505(100); S507(0); S508(0); S510(0)]	1	0	0.974924677
Q92797	2xPhospho [S1168(47.6); S1169(47.6); S1170(2.4); S1171(2.4); S1173(0.4); S1175(99.5); S1177(0.3); S1185(0)]	0.911	-0.134477041	0.989838391
Q92804	1xPhospho [S76(0); S77(0); Y78(0); S79(0); Y83(0); S94(0); S95(0.1); S97(99.9)]	1.645	0.718087584	0.549414358
Q92804	1xPhospho [S76(0); S77(0); Y78(0); S79(0); Y83(0); S94(0); S95(0); S97(100)]	1.451	0.537047519	0.797791757
Q92804	1xPhospho [T222(0.2); S226(92.1); S228(3.9); S231(3.9); T235(0); S246(0); T247(0)]	0.812	-0.300448367	0.938029982
Q92841	2xPhospho [S37(0); T45(100); S52(95.2); T55(4.8); S64(0)]	0.01	-6.64385619	8.35E-17
Q92841	1xPhospho [T569(0); T570(0); S571(99.9); S572(0.1); Y580(0)]	1.162	0.216610069	0.905765133
Q92841	1xPhospho [S671(0); S672(0); S674(98.6); S675(1.4); S676(0); S680(0)]	0.891	-0.166502663	0.980895643
Q92882	2xPhospho [T200(0); S202(100); Y207(0); S213(100)]	0.868	-0.204233052	0.976825037
Q92882	1xPhospho [T200(0); S202(100); Y207(0); S213(100)]	0.914	-0.12973393	0.992376868
Q92887	1xPhospho [S281(0); S283(100)]	1.404	0.489542936	0.810764434
Q92890	1 1xPhospho [S281(0); S283(100)]	1.242	0.312665174	0.901788734
Q92890	1 1xPhospho [S330(0); S335(100)]	0.807	-0.309359421	0.97008929
Q92890	1 1xPhospho [S330(0); S335(100)]	0.842	-0.248107862	0.972363997
Q92900	1xPhospho [S1100(0); S1107(1.3); S1110(97.3); T1111(1.3); Y1112(0)]	1.729	0.789937869	0.574566069
Q92900	2xPhospho [S1100(0); S1107(100); S1110(98.9); T1111(1.1); Y1112(0)]	0.69	-0.535331733	0.810764434
Q92903	1xPhospho [T33(0.1); S35(2.2); T36(2.2); S37(95.5); T41(0)]	1.575	0.655351829	0.705680833
Q92903	2xPhospho [T33(50); S35(50); T36(2.9); S37(97.1); T41(0)]	0.741	-0.432454552	0.904774194
Q92922	2xPhospho [S328(100); S330(100); T335(0); T337(0); S339(0)]	2.576	1.365132593	0.129503493
Q92922	2xPhospho [S328(100); S330(100); T335(0); T337(0); S339(0)]	2.237	1.161565256	0.41473799
Q92922	1xPhospho [S573(100)]	0.81	-0.304006187	0.971196575
Q92945	1xMet-loss+Acetyl [N-Term]; 1xPhospho [S2(100); Y4(0); S5(0); T6(0)]	0.294	-1.76611194	0.013939955
Q92945	3xPhospho [S125(100); S129(99.8); S131(99.8); S132(0.5)]	0.337	-1.569179503	0.261823421
Q92945	1xPhospho [S274(100); T277(0)]	0.652	-0.61705613	0.81171187
Q92945	1xPhospho [S125(0); S129(98.2); S131(1.8); S132(0)]	1.228	0.296310561	0.925146414
Q92945	1xPhospho [S274(100); T277(0)]	0.643	-0.637109357	0.93019184
Q92945	1xPhospho [S54(0); S59(100)]	1.206	0.270229907	0.931904324
Q92945	1xPhospho [S181(100); S184(0)]	1.175	0.232660757	0.94797789
Q92945	1xPhospho [S125(0); S129(100); S131(0); S132(0)]	1.156	0.209141398	0.958702638
Q92945	2xPhospho [S125(100); S129(100); S131(0); S132(0)]	1.119	0.162210036	0.969482098
Q92945	3xPhospho [S125(98); S129(52); S131(52); S132(98)]	1.024	0.034215715	0.980895643
Q92945	2xPhospho [S125(100); S129(99.9); S131(0.1); S132(0.1)]	1.042	0.059355278	0.989102507
Q92945	2xPhospho [S181(100); S184(100)]	0.833	-0.263611599	0.994478926
Q92974	1xPhospho [S886(100); Y894(0); S896(0); S903(0)]	100	6.64385619	8.35E-17
Q92974	2xPhospho [S149(0); S151(100); T152(0); T153(0); S163(100)]	0.489	-1.03209363	0.759239289
Q92974	1xPhospho [S149(0); S151(100); T152(0); T153(0); S163(0)]	0.576	-0.795859283	0.790280431
Q92974	2xPhospho [S940(0); S941(0); T945(50); S947(50); S952(0); S953(0); S956(0); S960(100)]	1.185	0.244887059	0.931904324
Q92974	1xPhospho [S691(0); T695(0.2); S696(99.8); T700(0)]	0.791	-0.3382504	0.969482098
Q92994	2xPhospho [S351(0); T352(0); T355(0.2); S357(3.7); S358(96.1); T365(100); S375(0)]	0.313	-1.675765438	0.169146318
Q92994	1xPhospho [S547(0); S548(2); S553(98); S564(0); S566(0)]	0.672	-0.573466862	0.891355291
Q92994	2xPhospho [S547(0); S548(0); S553(100); S564(50); S566(50)]	0.739	-0.436353731	0.954054893
Q92997	1xPhospho [T106(0); S112(4.4); S116(0.2); S125(95.4); T133(0); T135(0); S137(0); S140(0)]	1.337	0.418999465	0.85992414
Q92997	2xPhospho [T106(0); S112(0); S116(0); S125(100); T133(0); T135(0); S137(100); S140(0)]	1.264	0.337996464	0.879063032

Q93008	1 1xPhospho [Y2431(0); T2432(0); Y2437(0); T2438(0); Y2439(0); S2443(100); S2448(0); T2451(0); S2452(0); Y2455(0)]	0.553	-0.854648614	0.749797187
Q93008	1 1xPhospho [T1591(0); S1593(0); S1600(100); S1609(0)]	1.141	0.190298792	0.960298686
Q93009	1xPhospho [S18(100); T30(0)]	1.054	0.075874867	0.980895643
Q93073	2xPhospho [S251(99.9); T254(0.1); S257(0); S258(0); S259(0); S264(0); S270(97.4); S272(2.6); Y274(0); S276(0)]	0.792	-0.336427665	0.965248196
Q93075	1xPhospho [S80(83.1); S81(5.5); S82(0.4); S83(5.5); S85(5.5); S98(0)]	0.654	-0.612637459	0.938029982
Q969H4	2xPhospho [S305(1.6); S307(98.4); S309(0); S314(100)]	0.527	-0.924125133	0.534346776
Q969H4	3xPhospho [T289(100); T293(100); S300(100)]	0.527	-0.924125133	0.584144176
Q969H4	3xPhospho [T289(100); T293(100); S300(100)]	0.461	-1.117161344	0.658368583
Q969H4	2xPhospho [T289(100); T293(100); S300(0)]	0.915	-0.128156351	0.986075329
Q969R5	2xPhospho [S683(0); S688(100); S689(100); S695(0)]	1.694	0.760433875	0.589473706
Q969R5	3xPhospho [S683(100); S688(100); S689(100); S695(0)]	0.592	-0.756330919	0.726057431
Q969R5	2xPhospho [T66(0.1); S67(99.9); S73(96.8); T76(3.2)]	0.698	-0.518701058	0.943191333
Q969T4	2xPhospho [S8(100); S12(0.1); S14(1.5); T15(19.7); S16(19.7); S17(19.7); S19(19.7); S20(19.7)]	1.826	0.868686765	0.522889311
Q969T4	1xPhospho [S8(100); S12(0); S14(0); T15(0); S16(0); S17(0); S19(0); S20(0)]	1.56	0.641546029	0.678654866
Q96A49	1xPhospho [T248(100)]	1.503	0.587845009	0.781081017
Q96AD0	1xPhospho [S350(100); S357(0)]	1.047	0.066261442	0.955481376
Q96AE4	1xMet-loss+Acetyl [N-Term]; 1xPhospho [Y4(0); S5(0); T6(0); S11(0.1); S12(3); S14(96.9)]; C9JSZ1 1xMet-loss+Acetyl [N-Term]; 1xPhospho [Y4(0); S5(0); T6(0); S11(0.1); S12(3); S14(96.9)]	1.299	0.377401431	0.863045985
Q96AE4	1xPhospho [Y625(0); Y626(0); T629(0); S630(100)]	1.295	0.372952098	0.87431027
Q96AE4	1xPhospho [S84(99.9); T87(0.1); S97(0)]	1.136	0.183962835	0.965013037
Q96AP0	2xPhospho [T422(0); S424(0.1); S425(99.9); S429(0); T431(0); S433(99.9); S435(0.1)]	0.813	-0.298672743	0.980895643
Q96AT1	1xPhospho [S142(0); S143(1.7); S146(98.3)]	1.478	0.563646269	0.769999353
Q96AT1	1xPhospho [S50(100)]	1.215	0.280956314	0.929187903
Q96B23	3xPhospho [T312(0); S313(0); S326(100); S327(100); S329(100)]	0.061	-4.035046947	4.14E-06
Q96B23	1xPhospho [S66(100); S67(0); S69(0); S73(0); T74(0); S76(0); T80(0); T81(0)]	1.546	0.628540319	0.719966243
Q96B23	3xPhospho [S66(99.9); S67(3.5); S69(96.7); S73(0); T74(3.4); S76(96.6); T80(0); T81(0)]	0.569	-0.813499442	0.848969325
Q96B23	2xPhospho [S66(100); S67(1.8); S69(98.3); S73(0); T74(0); S76(0); T80(0); T81(0)]	0.834	-0.261880711	0.93019184
Q96B36	2xPhospho [S202(50); S203(50); S211(2.5); S212(97.5)]	1.666	0.736388401	0.733427607
Q96B36	1xPhospho [S183(100); S187(0)]	1.375	0.459431619	0.848293321
Q96B36	3xPhospho [T198(0); S202(100); S203(100); S211(50); S212(50)]	1.044	0.062121712	0.974891276
Q96B36	2xPhospho [T73(0); S88(100); T90(0); S92(100); T97(0)]	1.074	0.102993993	0.980895643
Q96B36	1xPhospho [T246(0); S247(100)]	1.044	0.062121712	0.986075329
Q96B36	3xPhospho [T198(0); S202(100); S203(100); S211(0); S212(100)]	0.913	-0.131313235	0.990188618
Q96B97	2xPhospho [S458(0); S468(100); S471(94.9); S472(2.6); T473(2.6)]	3.225	1.689299161	0.042011464
Q96B97	2xPhospho [S493(0); S495(0); T497(0); S498(0); S499(0); S500(0); S502(0); S503(0); S509(100); S511(100); S521(0)]	2.004	1.002882509	0.284236251
Q96B97	1xPhospho [S230(100)]	0.447	-1.161653263	0.458787027
Q96B97	1xPhospho [S587(0); S589(100); T593(0)]	1.32	0.40053793	0.860444802
Q96B97	3xPhospho [S493(0); S495(0); T497(0.6); S498(85.9); S499(6.8); S500(6.8); S502(0); S503(0); S509(100); S511(100); S521(0)]	1.123	0.167357928	0.964978421
Q96B97	1xPhospho [S444(0); S445(99); S447(1)]	0.76	-0.395928676	0.965013037
Q96B97	2xPhospho [S444(95.8); S445(95.8); S447(8.4)]	1.026	0.037030731	0.973763863
Q96BD5	2xPhospho [Y434(0); T444(0); T446(0); S447(100); S450(0); S451(0); S455(100)]	1.101	0.138814469	0.964978421
Q96BK5	1xPhospho [S200(100); S203(0); T205(0)]	0.908	-0.139235797	0.984718011
Q96BK5	2xPhospho [T155(0); S161(100); S163(50); T164(50); T170(0); T171(0); T172(0); T173(0); S174(0); T177(0); Y181(0)]	1.005	0.007195501	0.986075329
Q96BY7	2xPhospho [S1576(0); Y1577(0); S1579(100); S1582(2); S1583(97.9); S1585(0); T1587(0); T1589(0)]	0.95	-0.074000581	0.994773029
Q96C19	1xPhospho [S74(2.4); S76(97.6)]	0.599	-0.739372092	0.729937039
Q96C19	1xPhospho [S74(100); S76(0)]	0.926	-0.110915901	0.983706057
Q96C24	2xPhospho [S274(100); S287(3); S289(97)]	0.477	-1.067938829	0.64957454
Q96C92	3xPhospho [S240(100); S243(100); S247(99.8); S249(0.2); S258(0)]	0.01	-6.64385619	8.35E-17
Q96C92	2xPhospho [S240(0); S243(100); S247(100); S249(0); S258(0)]	0.649	-0.623709617	0.783976635
Q96CT7	1xPhospho [S122(100)]	1.584	0.663572335	0.665044008
Q96CT7	1xPhospho [S122(100)]	1.496	0.581110175	0.763890654
Q96CW6	2xPhospho [Y300(0); S302(100); S308(100)]	1.529	0.612588407	0.714577231
Q96D46	1xPhospho [S462(0); S468(0); T470(100)]	1.779	0.831066511	0.64957454
Q96D46	2xPhospho [S462(0); S468(100); T470(100)]	0.999	-0.001443417	0.985461924
Q96D71	2xPhospho [S535(90); S537(10.1); T539(50); S540(50); T544(0); S556(0); S558(0)]	1.733	0.793271654	0.658368583
Q96D71	1xPhospho [S535(0); S537(100); T539(0); S540(0); T544(0); S556(0); S558(0)]	1.519	0.60312187	0.74970909
Q96D71	3xPhospho [T153(0); S154(0); S162(100); S166(0); S170(100); T173(96); S174(4); T177(0)]	0.665	-0.588573754	0.783976635
Q96D71	2xPhospho [S535(0); S537(100); T539(0); S540(0); T544(0); S556(2.7); S558(97.4)]	1.221	0.2880632	0.92458333
Q96D71	1xPhospho [S709(100); T721(0)]	1.224	0.291603558	0.93019184
Q96D71	1xPhospho [S709(100); T721(0)]	1.172	0.22897257	0.952591019
Q96D71	2xPhospho [T473(95.7); S475(4.3); T478(0); T481(4.3); S482(95.7); S489(0)]	1.163	0.217851097	0.96293109
Q96D71	2xPhospho [T153(0); S154(0); S162(0); S166(0); S170(100); T173(2.8); S174(97.2); T177(0)]	0.899	-0.153606979	0.969482098
Q96D71	3xPhospho [S535(93.6); S537(93.6); T539(11.6); S540(1.1); T544(0); S556(0.4); S558(99.6)]	0.82	-0.286304185	0.975403456
Q96D71	3xPhospho [S535(0); S537(100); T539(50); S540(50); T544(0); S556(0); S558(100)]	1.053	0.074505436	0.983654603
Q96DF8	3xPhospho [S417(49.9); Y418(0.1); T419(49.9); S421(100); S425(50); T426(50)]	1.218	0.284514133	0.910166592
Q96DF8	2xPhospho [S391(100); S395(100)]	1.04	0.056583528	0.973763863
Q96DI7	1xPhospho [S30(100); T40(0)]	0.473	-1.080087911	0.590872099
Q96E09	1xPhospho [S187(0); S189(100); S197(0)]	1.627	0.702214251	0.745775186

Q96E09	1xPhospho [S35(2.1); S37(97.9); S45(0); T47(0); S48(0); S57(0)]	1.449	0.535057595	0.77579559
Q96E09	2xPhospho [S143(100); S147(100); T149(0)]	0.721	-0.471928835	0.892793132
Q96E09	2xPhospho [S143(100); S147(100); T149(0)]	0.833	-0.263611599	0.962617576
Q96E09	1xPhospho [S76(100)]	1.003	0.004321606	0.971196575
Q96EB6	1xMet-loss+Acetyl [N-Term]; 1xPhospho [S14(100); S16(0)]	0.61	-0.713118852	0.828459192
Q96EK9	1xPhospho [T157(0); S160(100); S165(0)]	2.657	1.409798228	0.095846277
Q96EV2	1xPhospho [S882(100); S884(0)]	1.67	0.739848103	0.649025374
Q96GA3	1xPhospho [S17(100)]	0.085	-3.556393349	0.000983986
Q96GA3	3xPhospho [S352(100); S355(99.8); T356(0.4); Y357(0); S358(99.8); Y361(0)]	0.306	-1.708396442	0.075936768
Q96GA3	3xPhospho [S352(100); S355(99.9); T356(0.1); Y357(0); S358(100); Y361(0)]	0.473	-1.080087911	0.318513278
Q96GA3	2xPhospho [S352(100); S355(97.4); T356(2.7); Y357(0); S358(0); Y361(0)]	0.479	-1.061902439	0.548297811
Q96GE4	2xPhospho [T398(0); T405(0); T414(0); T417(0.5); S419(99.5); S422(100); Y428(0)]	100	6.64385619	8.35E-17
Q96GE4	2xPhospho [T398(0); T405(0); T414(0); T417(0); S419(100); S422(100); Y428(0)]	1.799	0.847195187	0.592518785
Q96GE4	2xPhospho [S217(0); Y223(0); S226(100); S229(100)]	1.377	0.461528559	0.815248216
Q96GF1	1xPhospho [T106(100)]	3.619	1.855591108	0.140101389
Q96GV9	1xPhospho [S167(100); Y170(0)]	0.456	-1.13289427	0.654022948
Q96GY0	1xPhospho [S292(100)]	1.661	0.732052073	0.730212284
Q96HA1	2xPhospho [S179(0); S184(100); S188(2.7); T191(97.3)]	1.791	0.840765337	0.569465482
Q96HA1	3xPhospho [S345(95.4); S346(13.8); S348(95.4); S351(95.4); S364(0); S371(0)]	0.455	-1.13606155	0.713216184
Q96HA1	3xPhospho [S174(0.4); S179(99.6); S184(100); S188(99.6); T191(0.4)]	1.355	0.438292852	0.862999305
Q96HA1	2xPhospho [S174(5.3); S179(94.7); S184(100); S188(0); T191(0)]	1.333	0.41467678	0.884991416
Q96HC4	1xPhospho [S360(1.9); S362(98.1); S372(0); T373(0)]; D6RB78 1xPhospho [S389(1.9); S391(98.1); S401(0); T402(0)]	0.276	-1.857259828	0.064828347
Q96HC4	1xPhospho [S309(0); S313(0); S318(0); S319(100); S322(0); T323(0)]	1.467	0.552868871	0.769621572
Q96HC4	2xPhospho [S309(0); S313(0); S318(0); S319(100); S322(100); T323(0)]	0.712	-0.490050854	0.965139891
Q96HC4	1xPhospho [S309(0); S313(0); S318(1.4); S319(95.9); S322(1.4); T323(1.4)]	1.053	0.074505436	0.981858798
Q96HI0	2xPhospho [S287(3.2); S289(96.8); S293(100)]	1.368	0.45206823	0.843647956
Q96HP0	2xPhospho [S878(0.1); S880(99.9); S882(49.3); S883(49.3); S884(1.5); S894(0); S900(0)]	0.453	-1.142417045	0.263493654
Q96IG2	2xPhospho [Y412(0); T417(100); S421(2.1); S425(97.9)]	0.807	-0.309359421	0.974924677
Q96II8	1xPhospho [S628(0); S633(0); T637(0); T638(0); S640(4.6); T641(0.2); S643(94.9); T645(0.2); S649(0)]	0.01	-6.64385619	8.35E-17
Q96II8	1xPhospho [S415(0); S419(100)]	1.011	0.015782997	0.977823205
Q96IZ0	1xPhospho [Y226(0); S228(100); T229(0); T230(0); S231(0); S233(0); S238(0); S239(0)]	1.197	0.259423152	0.915865367
Q96IZ0	2xPhospho [Y226(0); S228(100); T229(0); T230(0); S231(100); S233(0); S238(0); S239(0)]	1.002	0.002882509	0.99184412
Q96JC9	2xPhospho [T157(50); S158(50); S165(100)]	0.717	-0.479954976	0.783976635
Q96JC9	2xPhospho [T157(50); S158(50); S165(100)]	0.808	-0.307572802	0.938029982
Q96JD6	3xPhospho [S268(100); T270(100); S272(100)]	0.307	-1.703689439	0.35260057
Q96JH7	3xPhospho [S756(3.7); S757(96.3); T761(100); T763(0); Y767(0); S768(100); T770(0); T771(0); S772(0)]	100	6.64385619	8.35E-17
Q96JH7	2xPhospho [S756(4); S757(96.1); T761(100); T763(0); Y767(0); S768(0); T770(0); T771(0); S772(0)]	1.404	0.489542936	0.790280431
Q96JH7	2xPhospho [S994(100); S997(0); S998(100); S1000(0)]	0.849	-0.236163541	0.970944072
Q96JM3	2xPhospho [S308(96.6); S311(3.4); S319(100)]	1.882	0.912266628	0.534130879
Q96JM3	3xPhospho [S427(100); S432(100); S436(100)]	0.648	-0.625934282	0.649621059
Q96JM3	2xPhospho [S651(99.9); S652(0.1); S653(99.9); S662(0); S666(0)]	0.593	-0.75389599	0.65170651
Q96JM3	1xPhospho [S626(2.9); S627(97.1); S632(0); S633(0); S634(0); Y636(0)]	1.579	0.659011171	0.712740038
Q96JM3	1xPhospho [T458(0); S459(100); S462(0); S468(0)]	1.321	0.401630467	0.861864549
Q96JM3	1xPhospho [S476(100)]	1.227	0.295135249	0.920957334
Q96JM3	2xPhospho [S471(2.9); S472(97.2); S476(99.9)]	1.181	0.240008965	0.927174327
Q96JM3	2xPhospho [S372(0); S373(0); S374(0); S376(0); S378(0); S379(0); S382(100); S386(100); S389(0)]	1.131	0.177598929	0.943817071
Q96JM3	3xPhospho [S204(100); S214(86.9); S217(19.3); T221(46.9); S223(46.9)]	0.779	-0.360304767	0.965139891
Q96JM3	1xPhospho [S204(100); S214(0); S217(0)]	1.09	0.124328135	0.965351295
Q96JM3	2xPhospho [S427(0); S432(100); S436(100)]	0.825	-0.277533976	0.972501767
Q96JM3	2xPhospho [S282(0); S284(0.1); S286(100); S297(100)]	0.918	-0.123433941	0.973263372
Q96JM3	2xPhospho [S626(50); S627(50); S632(95.1); S633(4.7); S634(0.2); Y636(0)]	0.766	-0.384583703	0.980895643
Q96JM3	2xPhospho [S443(1.5); S445(98.5); S452(100)]	0.906	-0.142417045	0.980895643
Q96JM3	1xPhospho [T400(0); T403(0); S405(100)]	0.976	-0.035046947	0.980895643
Q96JM3	2xPhospho [S626(0); S627(100); S632(100); S633(0); S634(0); Y636(0)]	0.978	-0.03209363	0.989554868
Q96JM3	1xPhospho [S427(100)]	0.947	-0.078563669	0.99184412
Q96JM3	1xPhospho [T103(0); S108(100)]	0.809	-0.305788392	0.998448514
Q96JM3	1xPhospho [S416(100)]	0.831	-0.267079618	1
Q96JM3	2xPhospho [S382(100); S386(100); S389(0)]	0.943	-0.084670324	1
Q96JM3	3xPhospho [S282(100); S284(0.1); S286(99.9); S297(100)]	0.96	-0.058893689	1
Q96JM3	2xPhospho [S204(100); S214(100); S217(0)]	1.006	0.008630305	1
Q96JP5	1xPhospho [S82(1.6); S83(98.4); S85(0)]	1.396	0.481298942	0.82511897
Q96JP5	3xPhospho [S82(0.1); S83(99.9); S85(0); S101(100); S103(100)]	0.82	-0.286304185	0.93461208
Q96JP5	2xPhospho [S82(100); S83(100); S85(0)]	0.83	-0.268816758	0.962617576
Q96JP5	3xPhospho [S82(2.8); S83(2.8); S85(94.4); S101(100); S103(100)]	0.854	-0.227692025	0.974924677
Q96KB5	2xPhospho [S19(0); S23(0); T24(99.9); T26(0.1); S32(100)]	100	6.64385619	8.35E-17
Q96KC8	2xPhospho [S479(100); S480(100); S484(0)]	0.01	-6.64385619	8.35E-17
Q96KC8	2xPhospho [S479(100); S480(100); S484(0)]	0.482	-1.052894948	0.318252841
Q96KC8	2xPhospho [S479(99.9); S480(99.9); S484(0.1)]	0.562	-0.831357964	0.792009111
Q96KC8	1xPhospho [S430(100); T435(0); T438(0)]	0.954	-0.067938829	0.99184412
Q96KR1	1xPhospho [S1054(100)]	0.89	-0.168122759	0.989898705
Q96KR1	1xPhospho [S1054(100)]	0.949	-0.075520008	1

Q96L73	2xPhospho [S478(0); S483(100); S486(100)]	1.48	0.565597176	0.755716034
Q96L91	2xPhospho [T297(0); T314(0); S315(100); T320(96.3); S321(3.6); S323(0.1)]	0.532	-0.910501849	0.693744548
Q96L91	2xPhospho [S941(99.7); S943(5.7); T945(94.6); T955(0); T970(0); S973(0)]	0.938	-0.092340172	0.981208663
Q96MW1	1xPhospho [Y135(0); T139(100); S151(0); T154(0); T155(0); S160(0)]	0.679	-0.55851652	0.893580559
Q96N64	1xPhospho [S515(0); S521(0.6); S525(91.2); S527(7.1); S534(0.6); S535(0.6); T540(0); T554(0)]	0.293	-1.77102743	0.126607041
Q96N64	1xPhospho [S81(100)]	1.425	0.510961919	0.797695365
Q96NB3	1xPhospho [S351(100); S362(0)]	1.432	0.518031493	0.843628055
Q96P16	1xPhospho [T296(0.1); S298(99.9); Y309(0); S310(0)]	1.388	0.473007568	0.796170626
Q96P16	2xPhospho [S152(3.5); S153(96.5); S156(100); S158(0); T163(0)]	1.279	0.355016264	0.889306217
Q96P16	1xPhospho [S152(0); S153(0.1); S156(99.8); S158(0.1); T163(0)]	1.135	0.182692298	0.965013037
Q96PK6	1xPhospho [T579(0); S582(100)]	4.63	2.211012193	0.008067445
Q96PK6	1xPhospho [T206(100)]	1.252	0.324234562	0.927711647
Q96PK6	2xPhospho [T518(0); S520(0); S521(3.5); S523(96.5); S527(0); Y528(0); S539(100); Y540(0)]	0.725	-0.4639471	0.930939466
Q96PK6	1xPhospho [S278(0); S280(100); Y285(0)]	1	0	0.9797333
Q96PK6	1xPhospho [Y548(0); S555(0); Y558(0); S560(0); S562(0); S571(0.1); T572(99.9); Y576(0)]	1.021	0.029982866	0.980895643
Q96PU5	3xPhospho [T473(0); S475(0); S479(100); S483(100); Y485(0); S487(100)]	100	6.64385619	8.35E-17
Q96PU5	1xPhospho [S446(0); S448(100); S449(0); T451(0); T453(0); S455(0)]	0.637	-0.650634722	0.933107775
Q96PY6	1xPhospho [T1050(0); S1052(100); S1057(0)]	0.11	-3.184424571	0.004184051
Q96Q15	2xPhospho [T3569(0); S3570(100); T3573(100); S3576(0); T3577(0); T3581(0)]	0.687	-0.541617996	0.912913246
Q96Q42	1xPhospho [S483(0); S489(0); S492(100)]	0.01	-6.64385619	8.35E-17
Q96Q42	2xPhospho [S483(100); S489(0); S492(100)]	0.569	-0.813499442	0.466887895
Q96QC0	1xPhospho [T586(0); S587(0); S591(100); S594(0); S597(0)]	1.559	0.640620928	0.759532785
Q96QC0	1xPhospho [S527(0); T531(0); Y533(0); T536(0); S542(0.2); S545(99.6); S552(0.2)]	1.512	0.59645814	0.797966154
Q96QC0	1xPhospho [S313(100); T315(0); S320(0)]	1.087	0.12035194	0.965351295
Q96QD8	1xPhospho [S39(100); Y41(0); S55(0)]	2.044	1.031395196	0.35306717
Q96QD8	3xPhospho [S10(0); S12(0); S17(94.4); S18(94.4); S19(10.5); Y20(0.7); S21(0.4); S22(94.1); S24(5.6); Y28(0); S29(0); Y30(0); T32(0)]	0.447	-1.161653263	0.497086499
Q96QD8	2xPhospho [S10(0); S12(0); S17(4.1); S18(4.1); S19(91.7); Y20(0); S21(0); S22(95.7); S24(4.3); Y28(0); S29(0); Y30(0); T32(0)]	0.915	-0.128156351	0.978952227
Q96QD8	3xPhospho [S10(100); S12(0); S17(0.1); S18(0.6); S19(92.2); Y20(0.6); S21(7.2); S22(92.6); S24(6.8); Y28(0); S29(0); Y30(0); T32(0)]	0.848	-0.23786383	1
Q96QF0	1xPhospho [S288(0); T289(0); S290(0); S291(0); S294(0.1); S296(99.9); S301(0)]	2.034	1.024319679	0.369620915
Q96QF0	2xPhospho [S288(99.7); T289(0.5); S290(47.2); S291(47.2); S294(2.7); S296(2.7); S301(0)]	1.049	0.069014678	0.965219233
Q96QR8	1xPhospho [S86(0); Y94(0); S100(0); S101(100)]	0.01	-6.64385619	8.35E-17
Q96QU8	2xPhospho [S199(0.1); T201(99.8); T204(0.1); S208(100); T210(0); S211(0); S214(0); S219(0); S224(0); S226(0); S227(0)]	1.274	0.349365278	0.912963437
Q96RE7	2xPhospho [S124(92.8); S125(7.1); S127(0.1); S130(0); S140(0.6); S141(0.6); S145(98.8); T150(0); S151(0); S157(0); T158(0); S164(0)]	0.83	-0.268816758	0.99811985
Q96RL1	2xPhospho [S44(100); S46(100)]	2.021	1.015069322	0.413498303
Q96RL1	2xPhospho [S44(100); S46(100)]	0.716	-0.481968507	0.934211842
Q96RR4	1xPhospho [S100(100)]	2.075	1.053111336	0.571672707
Q96RR4	2xPhospho [S125(0); Y128(0); S129(100); S132(50); S133(50); S136(0); S137(0)]	0.774	-0.369594529	0.974924677
Q96RS0	1xPhospho [S51(0.1); S55(100); T60(0); Y67(0); S68(0); T71(0); S74(0); S77(0)]	100	6.64385619	8.35E-17
Q96RS0	2xPhospho [S405(50); T407(50); S412(100); S422(0)]	3.536	1.822118275	0.034772246
Q96RS0	1xPhospho [S405(0); T407(0); S412(100); S422(0)]	2.526	1.336854639	0.327856952
Q96RS0	2xPhospho [S51(4.8); S55(95.2); T60(100); Y67(0); S68(0); T71(0); S74(0); S77(0)]	0.547	-0.870387262	0.847198622
Q96RS0	2xPhospho [S85(100); S89(100)]	0.696	-0.522840789	0.872730156
Q96RT1	3xPhospho [S598(100); S602(100); S603(100)]	0.01	-6.64385619	8.35E-17
Q96RT1	2xPhospho [S913(96.4); S915(3.5); T917(0.1); Y920(0); T928(0); S930(0); S931(0.1); S932(99.7); S933(0.1); S934(0); S938(0); T940(0)]	100	6.64385619	8.35E-17
Q96RT1	2xPhospho [S598(0); S602(100); S603(100)]	0.542	-0.883635243	0.642636279
Q96RT1	3xPhospho [S598(100); S602(100); S603(100)]	0.732	-0.450084446	0.93019184
Q96S55	1xPhospho [S75(98.5); S77(1.5); S78(0)]	0.673	-0.57132159	0.881849099
Q96S66	1xPhospho [S486(0); S487(0); T488(0); S490(0); S491(0); S493(0); S498(0); T502(0); S503(0); T506(0.1); S509(99.9)]	100	6.64385619	8.35E-17
Q96S82	1xPhospho [S230(100); T240(0)]	0.936	-0.095419565	0.975403456
Q96S94	1xPhospho [S345(0); S348(100); S351(0)]	1.5	0.584962501	0.739679432
Q96S94	1xPhospho [T320(0); T326(0); S327(0); S330(100)]	0.537	-0.897006007	0.848293321
Q96SB3	1xPhospho [S99(1.8); S100(98.2); S108(0)]	0.854	-0.227692025	0.974924677
Q96SI9	2xPhospho [Y453(0); T455(0); S465(96.3); S466(3.8); S470(100); S474(0)]	1.12	0.163498732	0.962617576
Q96ST3	2xPhospho [S277(100); T284(100); T287(0); S289(0); T292(0); S295(0); Y310(0)]	1.415	0.500802053	0.80163956
Q96ST3	1xPhospho [S277(100); T284(0); T287(0); S289(0); T292(0); S295(0); Y310(0)]	0.795	-0.330973234	0.887088694
Q96T23	1xPhospho [S1345(100)]	100	6.64385619	8.35E-17
Q96T23	2xPhospho [T1305(100); S1310(0); S1325(100)]	0.44	-1.184424571	0.550634186
Q96T23	3xPhospho [S1277(100); T1278(100); Y1281(0); S1282(100); S1296(0)]	0.638	-0.648371671	0.797921038
Q96T23	1xPhospho [S604(100)]	1.061	0.085424656	0.96229367
Q96T23	2xPhospho [S613(0); T614(0); S617(0); S622(100); T628(0); S629(100); S632(0)]	0.944	-0.083141235	0.97998947
Q96T23	2xPhospho [S1359(100); Y1363(0); S1364(0); S1370(0); T1371(0); S1375(100)]	0.993	-0.010134377	0.981858798
Q96T37	2xPhospho [S656(100); S659(100)]	4.522	2.176960992	0.050820073
Q96T37	1xPhospho [S656(100); S659(0)]	3.653	1.869081754	0.112314519
Q96T37	3xPhospho [S257(100); S259(100); T265(0); Y266(0); S269(0); S271(100); S276(0)]	2.177	1.122341408	0.234599354
Q96T37	1xPhospho [S292(0); S294(100); Y302(0)]	1.294	0.371837617	0.879063032
Q96T37	2xPhospho [S292(100); S294(100); Y302(0)]	1.186	0.24610401	0.920957334
Q96T37	1xPhospho [S700(100)]	0.699	-0.516635639	0.938730361

Q96T37	1xPhospho [T568(100); Y573(0)]	1.114	0.155749233	0.953220244
Q96T37	2xPhospho [S257(100); S259(100); T265(0); Y266(0); S269(0); S271(0); S276(0)]	0.872	-0.19759996	0.95746022
Q96T37	2xPhospho [S670(100); S674(100); S678(0); S679(0); S680(0)]	1.145	0.195347598	0.962617576
Q96T37	1xPhospho [S700(100)]	1.132	0.178873958	0.965248196
Q96T37	2xPhospho [S670(100); S674(100); S678(0); S679(0); S680(0)]	0.848	-0.23786383	0.971838536
Q96T37	3xPhospho [S670(100); S674(100); S678(33.3); S679(33.3); S680(33.3)]	0.91	-0.13606155	0.986075329
Q96T37	1xPhospho [S670(100); S674(0); S678(0); S679(0); S680(0)]	0.894	-0.161653263	1
Q96T58	1xPhospho [S1268(0); S1278(100)]	0.01	-6.64385619	8.35E-17
Q96T58	1xPhospho [S830(0); S832(0); S833(0); S835(0); S836(0); T838(0); S847(100)]	2.135	1.09423607	0.518148608
Q96T58	2xPhospho [S830(0); S832(0); S833(0); S835(0); S836(0); T838(100); S847(100)]	1.789	0.839153387	0.585349215
Q96T58	1xPhospho [S190(100)]	1.907	0.931304844	0.642636279
Q96T58	3xPhospho [S1380(100); S1382(100); S1390(0); S1392(100)]	0.54	-0.888968688	0.733427607
Q96T58	2xPhospho [S1380(100); S1382(100)]	1.445	0.531069493	0.792141506
Q96T58	1xPhospho [S1206(0); T1219(0.1); S1222(100); S1225(0)]	1.427	0.512985335	0.797966154
Q96T58	2xPhospho [S1283(100); S1287(100)]	1.375	0.459431619	0.821573905
Q96T58	2xPhospho [S1268(100); S1278(100)]	0.757	-0.401634795	0.879426164
Q96T58	1xPhospho [S2481(0); S2486(0); S2493(99.8); T2496(0.2)]	1.044	0.062121712	0.965013037
Q96T58	2xPhospho [S2114(0); S2120(100); S2124(0); S2126(100); S2134(0); S2135(0)]	1.031	0.044044333	0.983133428
Q96T58	1xPhospho [S1906(0); Y1908(0); T1910(0); S1918(100)]	0.928	-0.10780329	1
Q96T60	1xPhospho [T111(0); S114(0); T118(100); T122(0); S126(0)]	100	6.64385619	8.35E-17
Q96T88	2xPhospho [S88(6.8); S91(86.4); T93(6.8); S95(0.7); S102(92.1); S104(7.3)]	0.01	-6.64385619	8.35E-17
Q96T88	1xPhospho [S287(100)]	2.217	1.14860877	0.244965218
Q96T88	2xPhospho [S88(0); S91(100); T93(0.1); S95(99.9); S102(0); S104(0)]	0.61	-0.713118852	0.775418491
Q96T88	1xPhospho [S88(0); S91(100); T93(0); S95(0); S102(0); S104(0)]	1.278	0.353887836	0.874106272
Q96T88	1xPhospho [S88(3.1); S91(96.9); T93(0); S95(0); S102(0); S104(0)]	0.833	-0.263611599	0.962780378
Q96TA1	3xPhospho [S624(0); S638(100); S641(100); S646(100); T651(0)]	2.214	1.146655222	0.352337615
Q96TA1	2xPhospho [S665(100); S681(100)]	1.318	0.39835037	0.816296058
Q96TA1	2xPhospho [S624(0); S638(0); S641(100); S646(100); T651(0)]	0.795	-0.330973234	0.933760225
Q96TA1	2xPhospho [S691(0); S692(100); S696(100)]	0.923	-0.115597447	0.973763863
Q96TA1	1xPhospho [S691(0.1); S692(99.9); S696(0)]	1.012	0.01720929	0.980895643
Q96TA1	1xPhospho [S665(100); S681(0)]	1	0	0.989898705
Q96TC7	1xPhospho [S44(0); S46(100); S50(0); Y53(0); T54(0); T56(0); S57(0)]	0.613	-0.706041021	0.855534442
Q99442	1xPhospho [S353(0); S356(100); S357(0); T367(0)]	2.269	1.182056609	0.254834652
Q99459	2xPhospho [T373(93.1); T377(99.5); T385(7.4); S390(0); S393(0); T396(0)]	100	6.64385619	8.35E-17
Q99459	2xPhospho [T385(100); S390(0); S393(0); T396(100)]	1.434	0.520045024	0.752951521
Q99459	3xPhospho [T373(0.4); T377(99.8); T385(99.8); S390(0.2); S393(10.6); T396(89.2)]	0.83	-0.268816758	0.971220368
Q99459	3xPhospho [S427(3.5); T429(3.5); T430(93); S437(3.7); T438(96.3); T442(100)]	1.048	0.067638717	0.971453936
Q99460	2xPhospho [T303(0); S304(0); S305(0); T311(100); S315(100)]	1.311	0.390667686	0.879063032
Q99501	1xPhospho [S316(100); T321(0); S324(0)]	0.01	-6.64385619	8.35E-17
Q99501	2xPhospho [S436(100); S438(100)]	0.849	-0.236163541	0.980895643
Q99543	1xPhospho [S47(100); S49(0)]	1.999	0.999278472	0.440993586
Q99543	2xPhospho [S60(100); S63(100)]	0.485	-1.043943348	0.579084172
Q99543	3xPhospho [S47(100); S49(0); S60(100); S63(100)]	1.162	0.216610069	0.933107775
Q99543	2xPhospho [S60(100); S63(100)]	0.91	-0.13606155	0.973763863
Q99543	2xPhospho [S47(100); S49(100)]	1.082	0.113700499	0.97998947
Q99543	2xPhospho [S47(100); S49(100)]	0.93	-0.104697379	0.980895643
Q99549	3xPhospho [S126(0); S136(100); S138(0); S142(6.1); T144(93.9); T148(50); S149(50)]	0.01	-6.64385619	8.35E-17
Q99549	3xPhospho [S126(0); S136(100); S138(100); S142(0); T144(0); T148(0); S149(100)]	0.159	-2.652901329	0.002660898
Q99549	1xPhospho [Y83(0.1); T84(3.5); S85(93); T89(3.5)]	0.632	-0.662003536	0.852688742
Q99549	2xPhospho [S126(0); S136(100); S138(100); S142(0); T144(0)]	0.613	-0.706041021	0.886912356
Q99549	2xPhospho [S260(0); S264(100); S266(50); S267(50); S272(0); S279(0); S284(0); S286(0)]	0.606	-0.722610301	0.921446515
Q99567	1xPhospho [S35(100); T37(0); S44(0); S45(0); S46(0); S49(0); S50(0); T57(0)]	0.623	-0.682695932	0.658368583
Q99567	2xPhospho [S35(100); T37(0); S44(0); S45(0); S46(0); S49(0.3); S50(99.7); T57(0)]	0.732	-0.450084446	0.85992414
Q99569	2xPhospho [S273(100); Y275(0); S276(0); S281(97.7); T283(2.3)]	0.306	-1.708396442	0.054217096
Q99569	3xPhospho [T507(5.6); T508(5.6); S510(88.9); S512(100); S515(100)]	0.47	-1.089267338	0.604301121
Q99575	1xPhospho [Y722(0); S726(0); S729(98.3); S730(1.7)]	1.793	0.842375488	0.643284326
Q99590	1xPhospho [T331(99.1); S333(0.3); S335(0.3); S338(0.3); S341(0); S344(0); S352(0); S355(0); S357(0); S360(0); S361(0); S364(0)]	0.01	-6.64385619	8.35E-17
Q99590	2xPhospho [S748(0); T750(0); S753(0); S759(0); S760(0); T769(1); S771(98.8); S774(9.1); S776(90); T780(0.9)]	100	6.64385619	8.35E-17
Q99590	2xPhospho [S748(49.9); T750(49.9); S753(0.3); S759(0); S760(0); T769(0); S771(0); S774(0.1); S776(99.9); T780(0.1)]	100	6.64385619	8.35E-17
Q99590	2xPhospho [T769(0); S771(100); S774(50); S776(50); T780(0)]	100	6.64385619	8.35E-17
Q99590	1xPhospho [S400(0); S401(0); S402(0.1); S405(100); T410(0); S413(0); T415(0); S416(0)]	100	6.64385619	8.35E-17
Q99590	2xPhospho [T602(0); S608(100); S613(96.4); S614(3.6); T621(0)]	0.076	-3.717856771	7.06E-05
Q99590	2xPhospho [S338(100); S341(0); S344(0); S352(0); S355(0); S357(0); S360(97); S361(3); S364(0)]	0.1	-3.321928095	0.001042308
Q99590	1xPhospho [S338(100); S341(0); S344(0); S352(0); S355(0); S357(0); S360(0); S361(0); S364(0)]	1.862	0.896853073	0.617275619
Q99590	1xPhospho [S748(97); T750(3); S753(0); S759(0); S760(0)]	1.031	0.044044333	0.973263372
Q99590	2xPhospho [S796(100); S798(0); T799(0); T800(0.1); S802(100); T807(0)]	0.845	-0.242976753	0.973716784
Q99638	2xPhospho [S254(0); T264(0); S266(0); T268(0); S270(0); S272(100); S277(100)]	100	6.64385619	8.35E-17
Q99638	1xPhospho [S254(0); T264(0); S266(0); T268(0); S270(0); S272(0); S277(100)]	1.455	0.541019153	0.770935417
Q99638	3xPhospho [S324(90.1); S326(9.9); S328(0); S336(100); S341(100); S350(0); T351(0); T355(0)]	0.596	-0.746615764	0.814869677
Q99638	3xPhospho [S363(7.4); S368(0.1); S375(92.5); S380(100); S387(100)]	0.681	-0.554273297	0.962617576
Q99808	1xPhospho [S266(0); S269(100); S271(0); S273(0); T276(0); S279(0); S281(0)]	100	6.64385619	8.35E-17

Q99856	3xPhospho [S77(100); S81(100); S88(100)]	0.01	-6.64385619	8.35E-17
Q99856	1xPhospho [S119(100)]	0.603	-0.729770093	0.915532627
Q99956	2xPhospho [S351(0.4); S356(99.6); S361(0); S364(0); S368(0); T371(0); T372(5.3); T374(5.3); S375(89.4); T384(0)]	1.841	0.880489627	0.525627251
Q99956	3xPhospho [S351(0.8); S356(99.2); S361(100); S364(99.6); S368(0.1); T371(0.1); T372(0.1); T374(0.1); S375(0.1); T384(0)]	0.743	-0.428565884	0.93019184
Q99956	1xPhospho [S16(100)]	1.187	0.247319935	0.949541112
Q99959	2xPhospho [S288(87.8); S289(87.8); S293(17.4); S294(6.8); S297(0.1); T298(0)]	0.01	-6.64385619	8.35E-17
Q99959	2xPhospho [S151(100); S154(0.1); S155(99.9)]	0.526	-0.926865295	0.816509557
Q99959	2xPhospho [S151(100); S154(50); S155(50)]	0.98	-0.029146346	0.982345843
Q99961	2xPhospho [S286(0); S287(0); S288(100); S291(98.1); S292(1.9)]	0.572	-0.805912948	0.639650077
Q99988	1xPhospho [S39(100); S44(0); S48(0); S51(0)]	3.386	1.759581973	0.08052967
Q9BPU6	2xPhospho [S531(0.1); S532(99.9); S534(0); S536(99.9); S538(0.1)]	0.689	-0.537424112	0.863867749
Q9BPX3	1xPhospho [T664(0); T670(0); S674(100); S680(0); T686(0); T688(0)]	1.807	0.853596506	0.564458996
Q9BPX3	1xPhospho [T664(0); T670(0); S674(100); S680(0)]	1.45	0.5360529	0.749797187
Q9BPX3	2xPhospho [T968(0); S973(100); S975(100); S984(0)]	1.068	0.094911647	0.965248196
Q9BPX3	2xPhospho [T968(0); S973(100); S975(100); S984(0)]	0.987	-0.01887801	0.99184412
Q9BPZ7	1xPhospho [T509(1.5); S510(98.5); S512(0)]	0.774	-0.369594529	0.973763863
Q9BQ15	1xPhospho [S182(100); T187(0); S195(0); S196(0); S200(0)]	1.092	0.126972856	0.963156927
Q9BQ39	2xPhospho [Y39(0); S41(100); S45(95.1); T47(4.9); T53(0)]	1.413	0.498761466	0.799755198
Q9BQ61	2xPhospho [S111(100); T112(0); S114(100)]	1.871	0.903809559	0.601225718
Q9BQ61	1xPhospho [S111(98.9); T112(1.1); S114(0)]	1.422	0.507921465	0.804774778
Q9BQ61	2xPhospho [S111(100); T112(0); S114(100)]	1.485	0.570462931	0.813170869
Q9BQ67	2xPhospho [T114(0); S119(100); S122(100)]	1.841	0.880489627	0.380290732
Q9BQ67	2xPhospho [T114(0); S119(100); S122(100)]	0.834	-0.261880711	0.953505905
Q9BQ70	1xPhospho [T102(0.1); S104(0.1); T106(99.9); T111(0); T113(0); S116(0); S119(0); S122(0)]	5.241	2.389842109	0.012188937
Q9BQA1	1xPhospho [T5(100)]	0.748	-0.418889825	0.923783062
Q9BQE9	2xPhospho [S107(0); S108(0); T109(0); S111(0); S112(0); S114(100); S118(0); S120(2.4); S122(97.6); T126(0); S127(0)]	0.859	-0.219269964	0.962617576
Q9BQE9	3xPhospho [S107(0); S108(0); T109(0); S111(0); S112(0); S114(100); S118(100); S120(0); S122(100); T126(0); S127(0)]	0.793	-0.334607229	0.972363997
Q9BQE9	1xPhospho [S107(0); S108(0); T109(0); S111(0); S112(0); S114(0); S118(0); S120(0); S122(0); T126(4.5); S127(95.5)]	1.012	0.01720929	0.973263372
Q9BR76	1xPhospho [S413(100); S415(0); S423(0); S424(0); S431(0); T432(0); T433(0); T434(0); T439(0); S441(0); S443(0)]	0.393	-1.347398782	0.199681567
Q9BR76	2xPhospho [S413(0); S415(0); S423(4.3); S424(95.7); S431(91.6); T432(4.1); T433(0.2); T434(4.1); T439(0); S441(0); S443(0)]	0.995	-0.007231569	0.981858798
Q9BRD0	2xPhospho [T159(100); S163(100)]	10.628	3.409798228	0.000295924
Q9BRD0	3xPhospho [S197(100); S201(100); S210(47.9); S211(47.9); S214(4.3)]	2.197	1.13553487	0.507933422
Q9BRD0	2xPhospho [T159(100); S163(100)]	1.63	0.704871964	0.648285315
Q9BRD0	3xPhospho [S197(100); S201(100); S210(48.6); S211(48.6); S214(2.9)]	1.779	0.831066511	0.661999767
Q9BRD0	2xPhospho [S271(100); T279(1.9); Y280(0); S281(98.1)]	1.361	0.444667067	0.842976183
Q9BRD0	1xPhospho [S271(100); T279(0); Y280(0); S281(0)]	1.322	0.402722177	0.85992414
Q9BRD0	2xPhospho [S271(100); T279(0); Y280(0); S281(100)]	0.701	-0.512513651	0.879063032
Q9BRD0	1xPhospho [Y318(0); Y320(0); S325(100)]	0.877	-0.189351252	0.999714477
Q9BRK4	3xPhospho [S301(99.7); S304(48.6); S305(48.6); S306(9.8); S311(93.2); S320(0)]	0.237	-2.077041036	0.026068714
Q9BRK4	2xPhospho [S301(100); S304(3.6); S305(89.2); S306(3.6); S311(3.6); S320(0)]	1.28	0.35614381	0.872730156
Q9BRK4	2xPhospho [S224(100); S226(0); S227(0); T230(0); Y231(0); S232(0); T233(0); T239(0.1); T240(33.3); S241(33.3); S242(33.3); S249(0); S252(0)]	0.894	-0.161653263	0.984145373
Q9BRS8	2xPhospho [T443(0); S447(99.9); T450(49.8); S451(49.8); S455(0.5)]	100	6.64385619	8.35E-17
Q9BTA9	2xPhospho [T93(0.1); S94(0.6); Y95(0); S96(99.4); S101(78); S105(5.4); S109(5.4); S110(5.4); S112(5.4); S114(0.4); S115(0); S118(0); S122(0)]	100	6.64385619	8.35E-17
Q9BTA9	1xPhospho [T93(3.8); S94(96.2); Y95(0); S96(0); S101(0); S105(0); S109(0); S110(0); S112(0); S114(0); S115(0); S118(0); S122(0)]	2.116	1.081339627	0.310051771
Q9BTA9	1xPhospho [S523(2.3); S525(97.7); T531(0); S532(0); S534(0); S535(0); S538(0); T541(0); S547(0); S548(0)]	1.557	0.638768944	0.692314866
Q9BTA9	2xPhospho [S523(100); S525(100); T531(0); S532(0); S534(0); S535(0); S538(0); T541(0); S547(0); S548(0)]	1.508	0.592636429	0.728494169
Q9BTA9	3xPhospho [S518(0.4); S519(0.4); S520(99.3); S523(100); S525(100); T531(0); S532(0); S534(0); S535(0); S538(0); T541(0); S547(0); S548(0)]	1.096	0.132247798	0.965248196
Q9BTA9	2xPhospho [S518(4); S519(4); S520(88.1); S523(4); S525(100); T531(0); S532(0); S534(0); S535(0); S538(0); T541(0); S547(0); S548(0)]	0.954	-0.067938829	0.986075329
Q9BTA9	2xPhospho [T93(93.5); S94(7); Y95(0); S96(6.3); S101(0.5); S105(0.5); S109(0.5); S110(0.5); S112(90.4); S114(0.5); S115(0.5); S118(0); S122(0)]	0.897	-0.15682011	1
Q9BTC0	3xPhospho [T151(100); S152(100); S154(100); S156(0); T160(0)]	0.475	-1.074000581	0.664881585
Q9BTC0	2xPhospho [Y1244(0); S1248(0); S1254(95.9); T1255(3.9); T1256(0.2); S1260(100)]	0.633	-0.659722595	0.703341962
Q9BTC0	2xPhospho [S805(100); S809(100); S811(0); S817(0)]	0.726	-0.461958547	0.901788734
Q9BTC0	2xPhospho [S1242(0); Y1244(0); S1248(0); S1254(0.2); T1255(3.9); T1256(95.9); S1260(100)]	1.234	0.303342394	0.930376591
Q9BTC0	1xPhospho [S1456(100); T1469(0); S1471(0)]	0.938	-0.092340172	0.990182098
Q9BTE3	1xPhospho [S298(100); S302(0)]	0.514	-0.960159735	0.707033988
Q9BTE9	1xPhospho [S911(100); S915(0); T919(0)]	1.601	0.678973308	0.665992616
Q9BTE9	2xPhospho [S855(100); T864(100); S867(0)]	1.633	0.707524791	0.701261726
Q9BTE9	1xPhospho [S855(0); T864(100); S867(0)]	0.653	-0.614845103	0.764719465
Q9BTE9	1xPhospho [S855(0); T864(100); S867(0)]	1.226	0.293958979	0.908086456

Q9BTE9	2xPhospho [S911(100); S915(100); T919(0)]	1.259	0.332278283	0.91490394
Q9BTE9	1xPhospho [S158(0); T159(0); S161(0); S162(0); T163(0); S169(100); S172(0)]	1.076	0.105678078	0.962617576
Q9BTE9	1xPhospho [S833(100); T843(0); T846(0); T847(0)]	1.039	0.055195654	0.971220368
Q9BTE9	2xPhospho [S911(100); S915(0); T919(100)]	1.067	0.093560176	0.980895643
Q9BTK6	3xPhospho [T138(100); S143(100); S148(100)]	100	6.64385619	8.35E-17
Q9BTL3	1xPhospho [S36(100); S45(0)]	0.791	-0.3382504	0.980895643
Q9BTU6	1xPhospho [S460(0); S461(0); S462(98.6); S464(1.4); Y465(0); T466(0); S468(0); S471(0)]	0.63	-0.666576266	0.855557911
Q9BTU6	2xPhospho [S44(0); S47(100); S51(100)]	0.744	-0.426625474	0.879063032
Q9BTU6	1xPhospho [S44(0); S47(100); S51(0)]	1.05	0.070389328	0.986075329
Q9BTV6	1xPhospho [S394(100); T400(0)]	1.075	0.10433666	0.975403456
Q9BU76	2xPhospho [T215(33.3); S216(33.3); S217(33.3); T219(0.2); S220(99.8)]	100	6.64385619	8.35E-17
Q9BU76	1xPhospho [T215(0); S216(0); S217(0); T219(0.1); S220(99.9)]	1.338	0.420078116	0.85992414
Q9BUA3	2xPhospho [S148(100); S152(99.8); S154(0.2)]	2.297	1.199750857	0.134351611
Q9BUA3	2xPhospho [S248(100); S251(100); T253(0); T255(0)]	0.672	-0.573466862	0.711284181
Q9BUA3	1xPhospho [S308(100); S310(0)]	1.522	0.605968359	0.749797187
Q9BUB1	2xPhospho [S78(100); S80(100); S92(0)]	0.548	-0.867752202	0.426732719
Q9BUB1	2xPhospho [S78(100); S80(100); S92(0)]	0.568	-0.816037165	0.43602229
Q9BUB1	1xPhospho [S99(100); T104(0); Y105(0); T114(0)]	0.811	-0.30222618	0.942425529
Q9BUB5	2xPhospho [S221(100); S226(100)]	0.801	-0.320125852	0.94932168
Q9BUH6	1xPhospho [T145(0); S148(100)]	1.072	0.100304906	0.971196575
Q9BUJ2	1xPhospho [S194(100); T209(0); T215(0); Y216(0)]	1.539	0.621993232	0.736456959
Q9BUJ2	1xPhospho [S716(2.6); Y717(0); S718(97.4)]	1.362	0.445726703	0.819921743
Q9BUJ2	1xPhospho [S194(100); T209(0); T215(0); Y216(0)]	1.056	0.078609835	0.989898705
Q9BUT9	2xPhospho [S78(99.9); T79(2.4); S80(2.3); S82(95.4); T86(0); T89(0); Y90(0); T91(0)]	1.137	0.185232254	0.959719138
Q9BVC5	3xPhospho [S182(95.7); S184(4.3); S189(100); S193(100); T197(0); T198(0)]	0.01	-6.64385619	8.35E-17
Q9BVC5	2xPhospho [S182(0); S184(0); S189(100); S193(100); T197(0); T198(0)]	0.808	-0.307572802	0.901286155
Q9BVC5	3xPhospho [S182(0); S184(0); S189(100); S193(100); T197(0.1); T198(99.9)]	0.812	-0.300448367	0.94913059
Q9BVC5	2xPhospho [S182(0); S184(0); S189(100); S193(2.2); T197(95.7); T198(2.2)]	0.94	-0.089267338	0.980809409
Q9BVG4	1xPhospho [S197(100)]	8.861	3.147469522	0.0021002
Q9BVS4	3xPhospho [S369(0); S370(0); S380(100); S382(100); S385(100)]	0.01	-6.64385619	8.35E-17
Q9BVS4	2xPhospho [Y348(0); S350(100); S354(100); S362(0); Y366(0)]	0.093	-3.426625474	0.003239526
Q9BVS4	3xPhospho [S332(100); S335(100); S337(100)]	0.48	-1.058893689	0.560704885
Q9BVS4	2xPhospho [S332(0); S335(100); S337(100)]	0.779	-0.360304767	0.915837201
Q9BVS4	1xPhospho [S390(100); T394(0)]	1.028	0.039840265	0.965013037
Q9BVS4	2xPhospho [S369(0); S370(0); S380(100); S382(100); S385(0)]	0.838	-0.254977851	0.965139891
Q9BVS4	1xPhospho [S442(100); Y445(0); S456(0); S457(0)]	1.055	0.077242999	0.969482098
Q9BVS4	1xPhospho [S412(100); T414(0); S417(0)]	0.848	-0.23786383	0.980895643
Q9BVS4	1xPhospho [S332(0); S335(0); S337(100)]	0.97	-0.043943348	0.981858798
Q9BVV8	2xPhospho [Y103(0); T109(0); T113(0); S117(100); S120(100); T124(0); S128(0)]	0.672	-0.573466862	0.786760931
Q9BVV8	2xPhospho [Y103(0); T109(0); T113(0); S117(100); S120(100); T124(0); S128(0)]	0.891	-0.166502663	0.998271017
Q9BW61	1xPhospho [S33(100); Y35(0); T38(0)]	1.47	0.555816155	0.843024568
Q9BW71	1xPhospho [T527(3.5); S530(96.5); S543(0)]	1.882	0.912266628	0.518113775
Q9BW71	1xPhospho [S125(100)]	1.311	0.390667686	0.833782464
Q9BW71	2xPhospho [S219(0); S223(100); S227(100)]	1.267	0.341416524	0.891862526
Q9BW85	3xPhospho [S211(100); S213(100); S220(100); T231(0)]	0.63	-0.666576266	0.678654866
Q9BW85	2xPhospho [S211(100); S213(100); S220(0); T231(0)]	1.025	0.03562391	0.994865845
Q9BW85	2xPhospho [S211(100); S213(100); S220(0); T231(0)]	1.028	0.039840265	0.998448514
Q9BWH2	1xPhospho [S151(100); T156(0)]	0.01	-6.64385619	8.35E-17
Q9BWU0	1xPhospho [S312(100); T320(0); S321(0)]	1.835	0.875780063	0.644095191
Q9BWU0	1xPhospho [S312(100); T320(0); S321(0)]	1.548	0.630405471	0.763548117
Q9BWU0	1xPhospho [Y464(0); S466(100); T470(0)]	1.317	0.397255346	0.855557911
Q9BWU0	1xPhospho [S82(100)]	1.034	0.048236186	0.965139891
Q9BWU0	1xPhospho [T702(0); T704(0); S709(0); S712(100); Y720(0); S724(0)]	1.163	0.217851097	0.969482098
Q9BWW4	1xPhospho [S347(100); S352(0); S355(0); T360(0)]	1.109	0.149259365	0.958702638
Q9BX63	1xPhospho [S204(0); S206(100); S215(0)]	0.579	-0.788364747	0.883306273
Q9BX66	1xPhospho [S341(0); T344(0.1); T349(2.3); S350(97.6)]	0.908	-0.139235797	1
Q9BX95	1xPhospho [S112(100); T114(0)]	1.11	0.150559677	0.964978421
Q9BXB4	1xPhospho [S189(100); S194(0); S201(0)]	0.519	-0.946193556	0.665992616
Q9BXB4	1xPhospho [S172(0); S174(0); S177(0); S178(0); S179(0); S181(98.2); S184(1.8)]	1.424	0.509949146	0.792141506
Q9BXB4	2xPhospho [S172(0); S174(100); S177(0); S178(0); S179(0); S181(100); S184(0)]	0.898	-0.15521265	0.980895643
Q9BXF6	1xPhospho [S174(0); S176(100); S181(0)]	0.918	-0.123433941	0.986075329
Q9BXJ9	2xPhospho [T850(50.5); S855(98.9); S856(50.5)]	0.678	-0.560642822	0.891862526
Q9BXP5	1xPhospho [T535(0); S540(0); T544(100); T549(0); S550(0); S553(0)]	3.939	1.977829417	0.029040359
Q9BXP5	1xPhospho [S67(100)]	1.836	0.876566059	0.520989758
Q9BXP5	2xPhospho [S67(100); S74(100)]	1.092	0.126972856	0.974834011
Q9BXP5	2xPhospho [S67(100); S74(100)]	0.921	-0.118726939	0.977860291
Q9BXP5	2xPhospho [S67(100); S74(100)]	1.017	0.024319679	0.99885857
Q9BXS6	2xPhospho [T343(0); T344(0.2); T347(3.7); T349(96.1); S352(100)]	1.362	0.445726703	0.879063032
Q9BXY0	2xPhospho [S197(96); S199(34.7); S200(34.7); T202(34.7)]	0.542	-0.883635243	0.655090632
Q9BXY0	2xPhospho [S197(51); S199(51); S200(93.8); T202(4.3)]	0.712	-0.490050854	0.870080472
Q9BXY0	2xPhospho [S229(100); S232(100)]	0.607	-0.720231578	0.917263707
Q9BY11	2xPhospho [T329(0); T332(0); S337(0.2); T338(0); S339(0.2); S346(99.6); S348(96); S349(4); Y350(0)]	0.635	-0.655171503	0.808390523

Q9BY11	2xPhospho [T329(0); T332(0); S337(0); T338(0); S339(0); S346(96.4); S348(7.3); S349(96.4); Y350(0)]	0.749	-0.416962376	0.920957334
Q9BY44	1xPhospho [T522(0); S524(0); S526(100); S528(0)]	1.236	0.305678743	0.906868717
Q9BY44	1xPhospho [S474(100); S481(0)]	1.183	0.242450074	0.943512809
Q9BY89	2xPhospho [T1059(100); S1062(94); S1063(6); S1066(0); T1068(0); S1069(0); T1070(0); S1073(0); S1085(0)]	0.01	-6.64385619	8.35E-17
Q9BY89	1xPhospho [S1224(100); T1226(0); S1230(0); T1231(0)]	0.01	-6.64385619	8.35E-17
Q9BY89	2xPhospho [T1059(100); S1062(50); S1063(50); S1066(0); T1068(0); S1069(0); T1070(0); S1073(0); S1085(0)]	0.318	-1.652901329	0.080093579
Q9BYI3	1xPhospho [S453(100); S458(0); T461(0); S467(0); S468(0); T476(0)]	1.092	0.126972856	0.934211842
Q9BYK8	1xPhospho [S330(0); S331(0); S334(0); S338(97.8); T340(2.2); Y342(0)]	0.934	-0.098505545	0.989898705
Q9BYT3	1xPhospho [S468(0.1); S469(3); S470(96.8); T472(0.1); S473(0); S474(0)]	100	6.64385619	8.35E-17
Q9BYW2	2xPhospho [S311(0); S312(0); S314(0); S321(100); S323(100); S327(0)]	100	6.64385619	8.35E-17
Q9BYW2	2xPhospho [S2079(0); S2080(100); S2082(98.3); S2085(1.6); S2086(0); Y2088(0)]	1.215	0.280956314	0.93019184
Q9BYW2	2xPhospho [S742(0); S744(100); T749(0); S754(100)]	0.81	-0.304006187	0.958702638
Q9BZ23	2xPhospho [S168(100); S169(99.8); S171(0.3); S178(0); T182(0)]	0.61	-0.713118852	0.83187574
Q9BZ23	1xPhospho [S189(100); Y190(0); S191(0); T194(0); S195(0); S197(0)]	0.971	-0.042456799	0.974924677
Q9BZ95	1xPhospho [T556(0); S558(0); S560(0); S561(100); T565(0); S566(0); S568(0); T569(0); S571(0)]	100	6.64385619	8.35E-17
Q9BZD3	2xPhospho [S364(100); S365(100)]	0.01	-6.64385619	8.35E-17
Q9BZE4	3xPhospho [T461(0); Y466(0); S468(100); S470(100); S472(100)]	0.425	-1.234465254	0.432354312
Q9BZE4	3xPhospho [T461(0); Y466(0); S468(100); S470(100); S472(100)]	0.464	-1.10780329	0.538369555
Q9BZE4	2xPhospho [T461(95.8); Y466(3.7); S468(48.4); S470(48.4); S472(3.7)]	0.713	-0.488026018	0.962617576
Q9BZF1	3xPhospho [Y797(0); S798(0); S799(0); S807(100); S808(100); S810(100); S814(0); S818(0); T819(0)]	0.01	-6.64385619	8.35E-17
Q9BZF1	1xPhospho [S63(0); S65(0); S68(0); S71(100)]	1.687	0.754459974	0.624566719
Q9BZF1	2xPhospho [S63(0); S65(100); S68(100); S71(0)]	1.588	0.667210912	0.688350005
Q9BZF1	1xPhospho [S305(0); S314(100)]	1.238	0.308011315	0.915988867
Q9BZL6	2xPhospho [S197(100); S198(0); T199(0); S200(0); S203(0); S206(100)]	0.717	-0.479954976	0.830379242
Q9C004	1xPhospho [S125(100)]	1.146	0.196607044	0.94813191
Q9C073	3xPhospho [S305(50); S306(50); S317(0); S319(100); S327(99.9); S333(0.1); Y334(0)]	0.491	-1.02620507	0.749797187
Q9C073	1xPhospho [S213(100)]	1	0	0.974924677
Q9C0A6	1xPhospho [S865(100); T870(0); S873(0); S874(0)]	1.046	0.064882852	0.973599548
Q9C0A6	2xPhospho [S1131(5.4); S1133(83.4); S1135(5.4); S1136(5.4); S1138(0.4); S1144(99.5); S1146(0.5); S1148(0)]	1.044	0.062121712	0.975607937
Q9C0B0	2xPhospho [S374(0); S375(0); S378(100); S380(0); S385(100); S389(0)]	0.655	-0.610433188	0.879063032
Q9C0B5	1xPhospho [S415(0); S422(0); S423(1.4); S425(98.6)]	100	6.64385619	8.35E-17
Q9C0B5	2xPhospho [S395(2.6); Y396(0); S398(97.3); S401(0.1); S406(2.6); S409(97.3); T411(0.1)]	0.438	-1.190997225	0.613340907
Q9C0B5	2xPhospho [S398(100); S401(0); S406(0.2); S409(4.1); T411(95.7)]	0.428	-1.224317298	0.623465318
Q9C0B5	2xPhospho [S427(0); S428(0); S429(100); S432(100); T436(0); S445(0)]	0.67	-0.577766999	0.759239289
Q9C0B5	2xPhospho [T294(50.8); S296(98.5); S299(50.8); T303(0); S305(0); S307(0); S321(0)]	1.223	0.290424404	0.933760225
Q9C0B5	2xPhospho [S415(100); S422(2.4); S423(2.4); S425(95.2)]	0.759	-0.397828209	0.965351295
Q9C0B5	1xPhospho [S621(100); T627(0); Y630(0)]	0.738	-0.438307279	0.973263372
Q9C0B5	2xPhospho [S448(0); T451(0); T452(0); S453(0); T454(0.2); S455(99.8); Y456(0); S458(100); T463(0)]	1.053	0.074505436	0.973942845
Q9C0B5	1xPhospho [S380(100); T385(0); S386(0); S390(0); S391(0)]	0.912	-0.13289427	0.985461924
Q9C0B5	2xPhospho [S296(100); S299(100); T303(0); S305(0); S307(0); S321(0)]	0.921	-0.118726939	0.99184412
Q9C0B9	1xPhospho [S236(100)]	100	6.64385619	8.35E-17
Q9C0C2	2xPhospho [S1024(100); S1029(99.8); S1031(0.1); T1032(0.1)]	0.01	-6.64385619	8.35E-17
Q9C0C2	1xPhospho [S691(100); S695(0)]	0.01	-6.64385619	8.35E-17
Q9C0C2	2xPhospho [S221(100); S228(100)]	0.01	-6.64385619	8.35E-17
Q9C0C2	1xPhospho [S1383(98.2); S1385(1.8)]	2.66	1.411426246	0.251099621
Q9C0C2	2xPhospho [S1473(100); S1476(100)]	0.38	-1.395928676	0.369620915
Q9C0C2	1xPhospho [S893(100); Y897(0); S899(0)]	0.6	-0.736965594	0.747247624
Q9C0C2	2xPhospho [S494(100); S498(100); T501(0); S504(0); S518(0); S519(0)]	0.609	-0.715485867	0.749253672
Q9C0C2	2xPhospho [S976(95.5); T979(4.2); S983(0.4); S984(0.4); S987(99.6)]	0.611	-0.710755715	0.823333961
Q9C0C2	2xPhospho [S893(100); Y897(0); S899(100)]	0.733	-0.448114897	0.950730609
Q9C0C2	1xPhospho [S836(100)]	0.752	-0.411195433	0.957980162
Q9C0C2	1xPhospho [S1024(100); S1029(0); S1031(0); T1032(0)]	0.751	-0.413115187	0.965013037
Q9C0C2	2xPhospho [S1620(100); S1621(100); S1631(0)]	0.855	-0.226003675	0.972363997
Q9C0C2	1xPhospho [S429(100); S435(0); S437(0)]	0.78	-0.358453971	0.975403456
Q9C0C9	3xPhospho [S425(1.2); S429(98.9); S431(99.9); T435(0.1); S439(50); S441(50)]	0.01	-6.64385619	8.35E-17
Q9C0C9	2xPhospho [S87(100); S89(100); S98(0); S99(0); S102(0)]	0.476	-1.070966521	0.467067441
Q9C0C9	2xPhospho [T828(0); T834(0); S836(100); T838(0.1); S839(99.9); T841(0.1); T848(0)]	0.768	-0.380821784	0.886312012
Q9C0C9	1xPhospho [T828(0); T834(0); S836(0); T838(0); S839(99.9); T841(0); T848(0)]	0.854	-0.227692025	0.973263372
Q9C0C9	2xPhospho [S425(0); S429(0.3); S431(99.7); T435(0); S439(0.3); S441(99.7)]	0.872	-0.19759996	0.979214627
Q9C0C9	1xPhospho [T1216(4); T1219(4); S1220(88); T1222(4); S1227(0); S1231(0)]	0.969	-0.045431429	0.980895643
Q9C0C9	2xPhospho [T828(0); T834(0); S836(100); T838(3.5); S839(96.4); T841(0.1); T848(0)]	0.85	-0.234465254	0.984970017
Q9C0D5	2xPhospho [S61(0); S63(100); S66(0.1); S67(100)]	0.235	-2.089267338	0.056584602
Q9C0E2	1xPhospho [S521(100); S524(0); S525(0); T526(0)]	1.164	0.219091058	0.938190912
Q9C0H5	2xPhospho [S246(96.6); S248(96.6); S251(6.7); S253(0); T256(0); T264(0)]	0.475	-1.074000581	0.607772032
Q9C0J8	1xPhospho [S844(100)]	2.525	1.336283388	0.223977298
Q9C0J8	1xPhospho [T1198(0); S1210(100); S1213(0)]	3.136	1.648925559	0.229641558
Q9C0J8	1xPhospho [S764(0); S772(100)]	1.13	0.176322773	0.962617576
Q9C0J8	1xPhospho [S858(0); S860(4.1); S868(95.9)]	0.984	-0.023269779	0.994865845

Q9GZR1	2xPhospho [S350(100); S352(100); S358(0); S361(0); S362(0); S366(0); T367(0)]	6.827	2.771251752	0.000664559
Q9GZR1	3xPhospho [T323(96.6); S324(3.4); S326(0); S335(100); S336(100)]	0.213	-2.231074664	0.043001786
Q9GZR2	1xPhospho [S14(2.7); S15(97.3)]	7.465	2.90014226	0.013928597
Q9GZR2	1xPhospho [S51(100)]	0.797	-0.327348371	0.980895643
Q9GZR7	1xPhospho [S82(0); S92(0.1); S93(2.9); S94(97.1)]	0.01	-6.64385619	8.35E-17
Q9GZR7	2xPhospho [S82(100); S92(0); S93(0); S94(99.9)]	2.489	1.31556623	0.257316117
Q9GZR7	2xPhospho [S287(100); S295(100); T302(0); S306(0); S311(0)]	0.534	-0.905088353	0.458550853
Q9GZR7	1xPhospho [S82(0); S92(0); S93(1.6); S94(98.4)]	1.945	0.959770155	0.554775632
Q9GZR7	1xPhospho [S287(99.9); S295(0.1); T302(0); S306(0); S311(0)]	0.789	-0.341902795	0.908086456
Q9GZR7	2xPhospho [S82(100); S92(0); S93(50); S94(50)]	0.907	-0.140825544	0.972363997
Q9GZR7	2xPhospho [S82(100); S92(0); S93(0.1); S94(99.9)]	0.907	-0.140825544	0.972363997
Q9GZT9	1xPhospho [S125(100)]	1.404	0.489542936	0.797966154
Q9GZY8	5 1xPhospho [S146(100)]	1.054	0.075874867	0.977914045
Q9H0B6	2xPhospho [T163(0); S175(100); S179(100); S186(0); Y192(0)]	0.612	-0.708396442	0.840636242
Q9H0B6	1xPhospho [S581(0); S582(100)]	1.243	0.313826296	0.925853562
Q9H0B6	2xPhospho [S581(100); S582(100); S589(0); T594(0); T599(0); S602(0); S604(0)]	0.88	-0.184424571	0.980895643
Q9H0C8	1xPhospho [T81(5.8); S82(94.2); S89(0)]	1.024	0.034215715	0.998271017
Q9H0E3	2xPhospho [S844(0); T845(0); T853(0); S855(100); T856(100); S861(0); T862(0)]	100	6.64385619	8.35E-17
Q9H0E3	2xPhospho [S844(0); T845(0); T853(0); S855(99.8); T856(96.1); S861(3.9); T862(0.2)]	1.335	0.416839742	0.870404046
Q9H0E3	2xPhospho [S844(0); T845(0); T853(0); S855(100); T856(100)]	1.005	0.007195501	0.979498769
Q9H0G5	3xPhospho [S248(100); S254(100); S255(100); T263(0)]	0.336	-1.573466862	0.046344257
Q9H0G5	1xPhospho [T16(0); S27(0); S33(100); T39(0); S40(0); S42(0); S44(0)]	2.56	1.35614381	0.134351611
Q9H0G5	2xPhospho [S248(100); S254(97.5); S255(2.5); T263(0)]	0.846	-0.241270432	0.973263372
Q9H0H5	1xPhospho [S203(0); S206(0); S214(100)]	2	1	0.407913951
Q9H0L4	1xPhospho [S557(0); S560(3); S561(94); S563(3); S567(0); T570(0)]	0.875	-0.192645078	0.989898705
Q9H0S4	1xMet-loss+Acetyl [N-Term]; 1xPhospho [S9(100); T11(0); S14(0); T23(0)]	1.173	0.230203013	0.942907978
Q9H0U4	1xPhospho [S179(100)]	3.584	1.841570637	0.045629156
Q9H0W8	1xMet-loss+Acetyl [N-Term]	100	6.64385619	8.35E-17
Q9H0W8	1xPhospho [S25(0); S32(100)]	1.049	0.069014678	0.979125671
Q9H156	2xPhospho [S581(0); S585(0); T588(92.4); S591(48.3); T596(7.6); T598(51.7)]	1	0	0.980895643
Q9H1A4	2xPhospho [S686(100); S688(100)]	1.252	0.324234562	0.872730156
Q9H1A4	2xPhospho [S355(100); S362(100); S364(0)]	1.157	0.210388864	0.929795347
Q9H1B7	2xPhospho [T203(0); S215(99.9); S218(0); S219(0); S220(0); S224(100); S227(0)]	100	6.64385619	8.35E-17
Q9H1B7	2xPhospho [S657(100); S658(0); S659(100); S662(0); S665(0)]	2.766	1.467801156	0.059367832
Q9H1B7	1xPhospho [S547(100); S553(0)]	3.354	1.745882689	0.060250218
Q9H1B7	2xPhospho [S657(0); S658(0); S659(100); S662(99.9); S665(0.1)]	2.788	1.479230561	0.104523056
Q9H1B7	1xPhospho [S547(100); S553(0)]	2.309	1.207268173	0.179084272
Q9H1B7	2xPhospho [T610(50); T611(50); S615(0); S622(0); S629(0); T633(0); T636(0.3); S639(99.7)]	2.576	1.365132593	0.207542289
Q9H1B7	1xPhospho [S69(100)]	2.423	1.276794405	0.311750028
Q9H1B7	2xPhospho [S547(100); S553(100)]	2.332	1.221567789	0.313232504
Q9H1B7	2xPhospho [S547(100); S553(100)]	1.737	0.796597754	0.692550431
Q9H1B7	3xPhospho [S657(100); S658(0); S659(100); S662(100); S665(0)]	1.471	0.556797247	0.789364451
Q9H1C4	2xPhospho [Y541(0); S547(100); S550(100)]	0.661	-0.597277823	0.679191074
Q9H1E3	1xPhospho [T202(0); S204(0); S214(100)]	1.964	0.97379493	0.375331446
Q9H1E3	1xPhospho [T202(0); S204(0); S214(100)]	1.802	0.849599011	0.484693307
Q9H1E3	1xPhospho [T202(0); S204(0); S214(100)]	1.559	0.640620928	0.702881214
Q9H1E3	2xPhospho [T202(0); S204(100); S214(100)]	1.434	0.520045024	0.761264768
Q9H1E3	2xPhospho [T202(0); S204(100); S214(100)]	0.586	-0.77102743	0.827420854
Q9H1E3	1xPhospho [Y13(0); S14(0); S19(100); Y26(0)]	1.358	0.44148348	0.828459192
Q9H1E3	3xPhospho [T202(100); S204(100); S214(100)]	1.301	0.379620962	0.872730156
Q9H1E3	2xPhospho [T202(0); S204(100); S214(100)]	1.309	0.388465097	0.90089653
Q9H1E3	2xPhospho [Y13(0); S14(0); S19(100); Y26(0); S30(99.9); T34(0.1)]	0.817	-0.291592017	0.91490394
Q9H1E3	2xPhospho [Y13(0); S14(0); S19(100); Y26(0); S30(99.9); T34(0.1)]	0.82	-0.286304185	0.919960325
Q9H1E3	1xPhospho [Y13(0); S14(0); S19(100); Y26(0)]	1.228	0.296310561	0.921238122
Q9H1E3	2xPhospho [Y13(0); S14(0); S19(100); Y26(0); S30(2.2); T34(97.8)]	0.905	-0.144010303	0.969680228
Q9H1E3	1xPhospho [Y13(0); S14(0); S19(0); Y26(0); S30(97.6); T34(2.4)]	0.963	-0.054392297	0.985318278
Q9H1J1	2xPhospho [S329(0); T336(5); S337(5); S339(95.1); S341(95.1)]	0.449	-1.15521265	0.579751615
Q9H1K0	1xPhospho [S209(0); S211(0); T212(0); T214(0); S215(0); S217(0); S219(0); S222(100); S226(0)]	0.01	-6.64385619	8.35E-17
Q9H1K0	3xPhospho [S230(93.9); S232(12.3); S233(93.7); S235(82.6); S236(5.9); S238(5.9); S239(5.9)]	8.12	3.021479727	0.000437672
Q9H211	2xPhospho [T29(100); S31(100)]	1.126	0.171206827	0.969680228
Q9H2G2	1xPhospho [S189(99.9); T193(0.1); Y195(0); T206(0); S207(0)]	11.002	3.459693903	0.00010341
Q9H2G2	2xPhospho [S565(100); T569(0); S571(100)]	1.946	0.96051171	0.431663411
Q9H2G2	1xPhospho [S565(0); T569(0); S571(100)]	1.64	0.713695815	0.644308179
Q9H2G2	1xPhospho [Y1225(0); S1229(0); S1232(96.1); T1233(1.9); S1235(1.9)]	1.375	0.459431619	0.827205803
Q9H2G2	2xPhospho [S340(0); S341(0); S344(0); S347(100); S348(100); S354(0); S362(0); S364(0)]	0.621	-0.687334826	0.908086456
Q9H2H9	2xPhospho [S6(0); T11(0); T17(0); S25(100); S28(100); T32(0); S41(0)]	0.611	-0.710755715	0.558878683
Q9H2H9	1xPhospho [S52(100); T54(0); S56(0)]	1.958	0.969380765	0.579084172
Q9H2H9	2xPhospho [S6(0); T11(0); T17(0); S25(100); S28(100); T32(0); S41(0)]	0.604	-0.727379545	0.699388119
Q9H2H9	2xPhospho [S49(100); S52(100); T54(0); S56(0)]	0.662	-0.595096878	0.93019184
Q9H2P0	1xPhospho [S934(2.7); Y936(2.7); T938(94.5); T942(0); T946(0)]	0.52	-0.943416472	0.697129868
Q9H2P0	1xPhospho [S953(100); S955(0.1)]	1.366	0.449957484	0.821573905
Q9H2P0	2xPhospho [S953(100); S955(100)]	0.836	-0.258425153	0.963131675
Q9H2P0	2xPhospho [S953(100); S955(100)]	0.846	-0.241270432	0.97198185
Q9H2P0	1xPhospho [S709(98.9); S711(1.1)]	0.979	-0.030619235	0.973763863

Q9H2P0	1xPhospho [S921(99.9); S923(0.1)]	0.808	-0.307572802	0.988508396
Q9H2Y7	2xPhospho [S1370(100); T1372(100); T1378(0)]	1.07	0.097610797	0.980895643
Q9H307	2xPhospho [S100(100); S114(93.4); S115(5.8); T119(0.4); S120(0.4)]	100	6.64385619	8.35E-17
Q9H307	2xPhospho [T408(5.6); S413(94.4); S417(100); S422(0)]	1.37	0.454175893	0.870080472
Q9H307	2xPhospho [S441(100); S443(100); S450(0)]	1.066	0.092207438	0.965445802
Q9H307	1xPhospho [S100(100); S114(0); S115(0); T119(0); S120(0)]	1.083	0.115033243	0.977858961
Q9H307	2xPhospho [S96(100); S100(100); S114(0); S115(0); T119(0); S120(0)]	0.923	-0.115597447	0.990299313
Q9H307	1xPhospho [S381(100)]	0.929	-0.106249498	0.99811985
Q9H307	1xPhospho [S441(0); S443(100); S450(0)]	0.988	-0.017417053	1
Q9H3N1	1xPhospho [S270(100); S274(0); T277(0); S280(0)]	1.48	0.565597176	0.722275692
Q9H3N1	1xPhospho [S247(100); S253(0)]	1.408	0.493647334	0.790280431
Q9H3N1	1xPhospho [S270(100); S274(0); T277(0)]	1.263	0.336854639	0.880267055
Q9H3N1	1xPhospho [S270(100); S274(0); T277(0)]	1.212	0.277389699	0.939053555
Q9H3N1	1xPhospho [S270(100); S274(0); T277(0); S280(0)]	1.128	0.173767068	0.95738663
Q9H3N1	1xPhospho [S247(100); S253(0)]	1.086	0.119024103	0.975403456
Q9H3P7	1xPhospho [T326(0); S329(0.1); S333(99.9)]	100	6.64385619	8.35E-17
Q9H3Q1	2xPhospho [S275(0); S278(0); S292(100); S295(100)]	2.253	1.171847314	0.208873237
Q9H3Q1	1xPhospho [S118(100)]	2.014	1.010063683	0.415067195
Q9H3Z4	1xPhospho [S8(0); S10(100); T11(0); S12(0); S15(0); Y17(0)]	0.996	-0.005782353	0.998448514
Q9H410	2xPhospho [T20(0); S27(0); S28(100); S30(100)]	1.125	0.169925001	0.934569753
Q9H4G0	2xPhospho [S639(100); S648(0); S650(99.8); T652(0.2); S658(0)]	100	6.64385619	8.35E-17
Q9H4H8	2xPhospho [S365(33.3); S366(33.3); T367(33.3); S369(100); Y373(0); S375(0); S376(0)]	100	6.64385619	8.35E-17
Q9H4H8	3xPhospho [S365(66.7); S366(66.7); T367(66.7); S369(100); Y373(0); S375(0); S376(0)]	0.633	-0.659722595	0.790280431
Q9H4H8	3xPhospho [S365(99.3); S366(92.2); T367(8.5); S369(100); Y373(0); S375(0); S376(0); S383(0)]	0.778	-0.36215794	0.980895643
Q9H4L5	2xPhospho [T249(0); Y250(0); S251(100); S262(2.7); S265(97.3)]	0.425	-1.234465254	0.689634865
Q9H4L7	2xPhospho [S211(100); S212(96.9); S213(3.1); S214(0); Y217(0); S227(0)]	100	6.64385619	8.35E-17
Q9H4L7	3xPhospho [T119(0); S124(100); S127(100); S132(100); T137(0)]	0.234	-2.095419565	0.095261976
Q9H4L7	1xPhospho [S79(100); S81(0); Y82(0)]	1.915	0.937344392	0.53170939
Q9H4L7	3xPhospho [T119(0); S124(100); S127(100); S132(100); T137(0)]	0.72	-0.473931188	0.879063032
Q9H4L7	2xPhospho [T119(0); S124(100); S127(100); S132(0); T137(0)]	1.199	0.261831659	0.926485961
Q9H4L7	2xPhospho [T54(100); S57(100); T60(0); T63(0); S66(0); S67(0); T71(0)]	1.123	0.167357928	0.94797789
Q9H4L7	2xPhospho [T119(0); S124(100); S127(100); S132(0); T137(0)]	1.167	0.222804561	0.952591019
Q9H4L7	3xPhospho [S211(100); S212(100); S213(0); S214(100); Y217(0); S227(0)]	0.802	-0.318325858	0.958702638
Q9H4L7	3xPhospho [S211(98.5); S212(98.5); S213(51.5); S214(51.5); Y217(0); S227(0)]	0.873	-0.195946441	0.962617576
Q9H4L7	2xPhospho [Y91(0); S95(100); S96(100); S98(0); S103(0); S107(0); T109(0)]	0.872	-0.19759996	0.980895643
Q9H4L7	2xPhospho [S146(100); S152(100)]	0.914	-0.12973393	1
Q9H4Z3	2xPhospho [S14(0); S17(97.2); S19(2.8); T22(0); S23(0); S26(0); S30(100)]	0.804	-0.314732593	0.933107775
Q9H501	2xPhospho [T187(0); S190(0); T192(0); S193(100); S198(100)]	100	6.64385619	8.35E-17
Q9H501	3xPhospho [T311(100); S312(99.8); S313(99.8); T319(0.5); S327(0)]	0.707	-0.50021788	0.869295771
Q9H501	3xPhospho [T311(100); S312(99.9); S313(50.1); T319(50.1); S327(0)]	0.77	-0.377069649	0.937278821
Q9H501	2xPhospho [S657(100); S663(100); Y671(0)]	0.972	-0.040971781	0.989838391
Q9H501	1xPhospho [T187(0); S190(0); T192(0); S193(0); S198(100)]	0.838	-0.254977851	1
Q9H5H4	2xPhospho [S17(99.8); S18(50.1); S23(50.1); Y27(0)]	0.431	-1.214240226	0.499845416
Q9H5H4	1xPhospho [S83(100); S88(0)]	1.493	0.578214165	0.763576928
Q9H5H4	2xPhospho [S90(100); S97(100)]	0.655	-0.610433188	0.821573905
Q9H5H4	3xPhospho [S139(100); Y142(0); S144(11.5); S146(94.3); S147(94.3); Y149(0); S151(0); T154(0)]	0.72	-0.473931188	0.971183767
Q9H5H4	2xPhospho [S139(100); Y142(0); S144(100); S146(0); S147(0); Y149(0); S151(0); T154(0)]	1.096	0.132247798	0.973263372
Q9H5N1	2xPhospho [S189(100); S193(100); T194(0); S200(0)]	1.179	0.237563718	0.950730609
Q9H5V8	1xPhospho [T790(4.2); S797(95.8); S801(0); S803(0); Y806(0); T807(0); S809(0); S817(0); S818(0)]	0.337	-1.569179503	0.260308631
Q9H5V8	1xPhospho [S797(0); S801(0); S803(0.1); Y806(0.1); T807(99.8); S809(0); S817(0); S818(0)]	1.437	0.523060062	0.749797187
Q9H5V8	2xPhospho [T790(0); S797(100); S801(100); S803(0); Y806(0); T807(0); S809(0); S817(0); S818(0)]	1.089	0.123003954	0.971220368
Q9H6F5	2xPhospho [S58(0); T65(50); S66(50); S69(100)]	2.254	1.172487516	0.179675012
Q9H6F5	1xPhospho [S255(100)]	1.949	0.962734091	0.45259943
Q9H6F5	3xPhospho [S58(100); T65(50); S66(50); S69(100)]	1.756	0.812292845	0.457443685
Q9H6F5	1xPhospho [S80(0); S91(100)]	1.522	0.605968359	0.685087581
Q9H6F5	3xPhospho [S102(100); Y109(0); S110(100); S113(100)]	1.412	0.497740089	0.755716034
Q9H6F5	1xPhospho [S58(100)]	1.402	0.487486349	0.811560495
Q9H6F5	2xPhospho [S80(100); S91(100)]	1.37	0.454175893	0.816296058
Q9H6F5	2xPhospho [S102(100); Y109(0); S110(0.1); S113(99.9)]	0.756	-0.40354186	0.850350883
Q9H6F5	2xPhospho [S18(100); S21(100); T23(0); S24(0); S26(0)]	0.847	-0.239566125	0.938029982
Q9H6F5	1xPhospho [S18(100); S21(0); T23(0); S24(0); S26(0)]	1.087	0.12035194	0.964978421
Q9H6F5	2xPhospho [S37(0); T42(0); S47(100); S50(100)]	0.913	-0.131313235	0.971985173
Q9H6F5	1xPhospho [T146(0.2); S160(99.8); Y164(0); S175(0); T182(0)]	0.744	-0.426625474	0.972326459
Q9H6F5	1xPhospho [S37(0); T42(0); S47(100); S50(0)]	1.083	0.115033243	0.977858961
Q9H6F5	1xPhospho [S102(100)]	1.077	0.10701825	0.979498769
Q9H6H4	1xPhospho [S150(0); S152(100)]	1.657	0.728573603	0.724397901
Q9H6H4	3xPhospho [S194(100); T196(100); S202(99.9); T204(0.1)]	0.571	-0.808437349	0.862999305
Q9H6H4	1xPhospho [S150(0); S152(100)]	0.94	-0.089267338	0.980895643
Q9H6K1	1xPhospho [S215(100)]	0.792	-0.336427665	0.981858798
Q9H6R7	2xPhospho [S680(0); S686(3); S688(97.1); S690(100); S695(0); S696(0)]	1.145	0.195347598	0.933107775
Q9H6R7	1xPhospho [S686(0); S688(0); S690(100); S695(0); S696(0)]	0.813	-0.298672743	0.988508396
Q9H6S0	2xPhospho [S1221(100); S1223(100)]	0.98	-0.029146346	0.971196575
Q9H6T3	1xPhospho [S480(0); S481(100); T488(0); S489(0); T491(0)]	2.005	1.003602237	0.529838337
Q9H6T3	1xPhospho [S503(0); T505(0); S506(0); S507(0); S513(100); S521(0); Y522(0); S523(0)]	1.433	0.51903861	0.780165563

Q9H6T3	2xPhospho [S110(0); T111(0); S114(0.2); S116(99.9); S119(99.9); S121(0.2); S130(0)]	1.213	0.27857955	0.928202446
Q9H6T3	3xPhospho [S110(0); T111(0); S114(0); S116(100); S119(100); S121(100); S130(0)]	1.067	0.093560176	0.980895643
Q9H6Y2	1xPhospho [T4(0); S14(100); S21(0); T26(0)]	1.246	0.317304068	0.907312294
Q9H706	3 2xPhospho [T602(0); S604(0); S609(100); S613(100); S615(0); S620(0); S621(0)]	0.743	-0.428565884	0.952526233
Q9H706	3 2xPhospho [S563(100); S565(100); T567(0); S569(0); Y570(0); Y571(0); S572(0); S573(0); T580(0)]	1.033	0.046840254	0.971220368
Q9H788	1xPhospho [T313(0); S315(100); S316(0); S317(0)]	0.573	-0.803392956	0.866095862
Q9H7D0	2xPhospho [S1775(99.9); S1780(0); S1781(0.1); S1785(0); T1786(0); S1789(100); T1794(0)]	0.314	-1.671163536	0.042358065
Q9H7D0	1xPhospho [S1834(100); T1835(0)]	0.459	-1.123433941	0.444861531
Q9H7E2	1xPhospho [S432(100); Y435(0)]	1.77	0.82374936	0.598714672
Q9H7L9	1xPhospho [S234(99.7); S236(0.1); S237(0.1); T244(0); S248(0)]	1.384	0.468843943	0.796472939
Q9H7L9	2xPhospho [S234(100); S236(50); S237(50); T244(0); S248(0)]	0.869	-0.202571918	0.955140197
Q9H7L9	3xPhospho [S45(100); T49(100); S53(99.8); T55(0.2); Y65(0)]	0.898	-0.15521265	0.989102507
Q9H7L9	3xPhospho [S45(100); T49(100); S53(100); T55(0); Y65(0)]	0.94	-0.089267338	1
Q9H7N4	1xPhospho [Y228(0); T234(0); Y238(0); S239(100)]	2.138	1.096261853	0.406373833
Q9H7N4	2xPhospho [Y732(0); S734(100); S738(100)]	1.487	0.572404647	0.765444806
Q9H7N4	2xPhospho [T989(0.7); S992(0.3); T994(0.3); S997(51.2); S998(51.2); T1001(95.8); S1005(0.5); T1012(0)]	1.263	0.336854639	0.879063032
Q9H7N4	1xPhospho [S874(100)]	1.35	0.432959407	0.879063032
Q9H7N4	2xPhospho [S498(100); S500(100); T515(0)]	1.251	0.32308179	0.904774194
Q9H7N4	2xPhospho [S872(100); S874(100)]	0.978	-0.03209363	0.99277609
Q9H7N4	2xPhospho [Y732(0); S734(100); S738(100)]	0.933	-0.100051014	0.993548475
Q9H832	3xPhospho [S337(100); S339(99.6); S340(95.3); S341(4.7); S342(0.4); T344(0); T346(0); S351(0)]	0.074	-3.756330919	0.000301612
Q9H832	2xPhospho [S337(99.9); S339(0.2); S340(99.8); S341(0.1); S342(0); T344(0); T346(0); S351(0)]	0.898	-0.15521265	0.982345843
Q9H8M2	3xPhospho [S566(91.5); S568(9.3); S571(99.2); T579(0); Y583(0); S588(100); S594(0)]	1.964	0.97379493	0.431663411
Q9H8M2	2xPhospho [S37(0); T40(0); S43(0); S45(0); S49(100); S50(0); Y51(0); Y52(0); S56(100)]	1.607	0.684369929	0.694827919
Q9H8S9	1xPhospho [T35(100); S38(0)]	1.725	0.786596362	0.690335172
Q9H902	1xPhospho [S150(0); S152(100); T157(0); T158(0)]	0.583	-0.778432211	0.883574076
Q9H981	1xPhospho [S412(100); Y424(0); T428(0); S430(0)]	0.811	-0.30222618	1
Q9H9J4	1xPhospho [S1181(100)]	1.465	0.550900665	0.783976635
Q9H9J4	1xPhospho [S72(0); S75(100)]	0.898	-0.15521265	0.991765633
Q9H9J4	3xPhospho [S1219(100); S1222(100); S1226(100)]	0.948	-0.077041036	1
Q9HAJ7	2xPhospho [T92(50); S93(50); S99(100); T104(0); T118(0)]	0.672	-0.573466862	0.810324628
Q9HAW4	3xPhospho [S1005(23.2); S1012(92.3); S1018(92.3); S1020(92.3)]	0.01	-6.64385619	8.35E-17
Q9HAW4	2xPhospho [S1147(100); S1156(100); T1161(0); S1168(0)]	0.01	-6.64385619	8.35E-17
Q9HAW4	2xPhospho [S718(0); S720(0); S721(0); S723(100); T724(100)]	0.01	-6.64385619	8.35E-17
Q9HAW4	2xPhospho [T797(0); S798(0); T802(0); S808(100); S810(100)]	0.408	-1.293358943	0.158959327
Q9HAW4	2xPhospho [S770(10.9); S771(10.9); S774(89.1); S775(89.1); S781(0); T782(0); S785(0); Y786(0)]	0.324	-1.625934282	0.163347385
Q9HAW4	1xPhospho [T1287(0); S1289(100)]	0.41	-1.286304185	0.643284326
Q9HAW4	3xPhospho [S1005(1.4); S1012(98.6); S1018(100); S1020(100)]	0.637	-0.650634722	0.797966154
Q9HAW4	1xPhospho [S1147(0); S1156(100); T1161(0); S1168(0)]	0.742	-0.430508908	0.946054239
Q9HAZ1	2xPhospho [S136(100); S138(100); S151(0)]	0.716	-0.481968507	0.781301532
Q9HB09	3xPhospho [S113(100); T117(100); S121(50); T128(0); S132(50)]	0.998	-0.002888279	0.981858798
Q9HB09	3xPhospho [S113(100); T117(100); S121(100); T128(0); S132(0)]	0.976	-0.035046947	0.999263007
Q9HB20	2xPhospho [T242(0.3); Y243(0); S244(99.7); T246(0); S248(99.7); S250(0.3); S264(0)]	0.437	-1.194294815	0.524550881
Q9HB21	1xPhospho [S330(0); S332(100); S335(0); T336(0); T338(0)]	0.685	-0.545824107	0.925808485
Q9HC35	2xPhospho [S887(0); S888(0); T889(0); S891(0); S895(0.1); T897(99.9); T899(100); S903(0); T909(0); S914(0)]	0.242	-2.046921047	0.046957288
Q9HC35	2xPhospho [S94(99.9); T96(0.1); S97(0); S100(100)]	1.57	0.650764559	0.741515551
Q9HC35	2xPhospho [S144(100); S146(100); S150(0); S151(0)]	0.669	-0.579921884	0.752951521
Q9HC35	1xPhospho [S144(100); S146(0); S150(0); S151(0)]	0.788	-0.343732465	0.908086456
Q9HC44	1xPhospho [S49(100); S50(0)]	1.418	0.503857533	0.79002331
Q9HC52	1xPhospho [S311(100); S315(0); S316(0); Y321(0)]	4.19	2.066950244	0.017990844
Q9HC52	1xPhospho [S332(100)]	1.226	0.293958979	0.904079048
Q9HCD5	2xPhospho [S377(50); S378(50); T379(0); S381(100); S387(0)]	0.857	-0.222632891	0.980895643
Q9HCD6	2xPhospho [Y1528(0); S1530(100); S1534(100)]	0.618	-0.694321257	0.835933921
Q9HCH5	1xPhospho [S535(100); S540(0); T541(0); T544(0); S551(0)]	0.01	-6.64385619	8.35E-17
Q9HCK8	3xPhospho [T548(0); S549(96); S550(4); S553(100); S562(100); S569(0); S570(0)]	100	6.64385619	8.35E-17
Q9HCK8	2xPhospho [T1993(4.6); S1995(95.4); S2008(100); T2012(0); T2014(0); S2018(0); S2021(0); T2023(0)]	4.391	2.134549534	0.019397976
Q9HCK8	1xPhospho [Y1964(0); S1976(100); S1978(0)]	1.84	0.879705766	0.53218972
Q9HCK8	2xPhospho [S1409(0); T1410(0); S1420(100); S1424(100)]	1.428	0.513995979	0.797921038
Q9HCK8	2xPhospho [S1420(100); S1424(100)]	0.828	-0.272297327	0.973599548
Q9HCL2	2xPhospho [S685(0); S688(100); S695(100)]	1.295	0.372952098	0.908086456
Q9HCN4	1xPhospho [T328(0); S338(100); T340(0)]	1.689	0.756169328	0.554526265
Q9HCN4	1xPhospho [S312(0); S314(100); S320(0); T325(0)]	0.852	-0.231074664	0.980895643
Q9HCS5	2xPhospho [S310(100); S314(100)]	0.274	-1.867752202	0.038127236
Q9HD26	1xPhospho [S276(100)]	6.762	2.757450016	0.034945
Q9HDC5	2xPhospho [T161(0); S162(100); S165(100)]	0.01	-6.64385619	8.35E-17
Q9HDC5	2xPhospho [T448(100); S452(100); Y456(0)]	0.778	-0.36215794	0.872730156
Q9NPD8	1xPhospho [S184(100)]	1.038	0.053806444	0.9699948
Q9NPI1	1xPhospho [S279(0); S289(100); S291(0)]	1.383	0.467801156	0.806224461
Q9NPI1	2xPhospho [S279(100); S289(50); S291(50)]	0.572	-0.805912948	0.848293321
Q9NPI6	1xPhospho [S142(99.5); S144(0.5); S150(0); S162(0)]	1.537	0.620117165	0.73347811

Q9NPI6	2xPhospho [S522(50.6); S523(50.6); S525(98.8); T528(0); T531(0); S534(0)]	1.565	0.646162657	0.742559804
Q9NPI6	1xPhospho [S142(99.8); S144(0.2); S150(0); S162(0)]	0.595	-0.749038426	0.893481203
Q9NPI6	3xPhospho [T309(90.1); Y310(0); T311(5); S315(5); S319(99.7); T321(0.3); T328(0); S334(100)]	0.603	-0.729770093	0.894595581
Q9NPI6	1xPhospho [S353(100); S364(0); S367(0); S373(0)]	0.756	-0.40354186	0.973763863
Q9NPI6	2xPhospho [T309(0); Y310(0); T311(0); S315(100); S319(100); T321(0); T328(0); S334(0)]	1.085	0.117695043	0.977149121
Q9NQ55	1xPhospho [S359(100); S364(0); S368(0)]	1.576	0.656267535	0.610338515
Q9NQ55	2xPhospho [S228(0); T233(0); S238(100); S240(100); T251(0)]	0.978	-0.03209363	0.99184412
Q9NQC3	1xAcetyl [N-Term]; 2xPhospho [S7(100); S11(0); S12(0.1); S13(0.1); S15(99.9)]	100	6.64385619	8.35E-17
Q9NQC3	1xAcetyl [N-Term]; 1xPhospho [S7(0); S11(0); S12(0); S13(0); S15(100)]	0.661	-0.597277823	0.862322824
Q9NQC3	2xPhospho [S107(95.3); S111(4.7); S114(0); S115(0); T116(0); S121(0); S124(48.8); S129(48.8); S131(2.4)]	1.174	0.231432408	0.952675081
Q9NQC3	1xPhospho [S107(100); S111(0); S114(0); S115(0); T116(0); S121(0); S124(0); S129(0); S131(0)]	0.848	-0.23786383	0.970242377
Q9NQC3	2xPhospho [S181(98.6); S182(50.7); S184(50.7); T188(0); S196(0)]	0.891	-0.166502663	0.973263372
Q9NQC3	2xPhospho [S181(98.7); S182(50.7); S184(50.7); T188(0); S196(0)]	0.887	-0.17299399	0.988508396
Q9NQS1	1xPhospho [T139(0); S142(0); S146(0); S147(0); S151(100); S153(0.1)]	0.089	-3.490050854	0.000361119
Q9NQS1	1xPhospho [S87(0); S94(100); T98(0); Y99(0); Y109(0); S110(0)]	1.098	0.134878054	0.962617576
Q9NQS7	3xPhospho [S263(100); S269(100); S275(100)]	100	6.64385619	8.35E-17
Q9NQS7	2xPhospho [T195(2.5); S197(48.7); T199(48.7); S202(0.1); T204(0); T207(0); S208(0); T213(4.9); S214(95.1); S218(0); T219(0)]	100	6.64385619	8.35E-17
Q9NQS7	3xPhospho [T195(3.8); S197(96.2); T199(0); S202(0); T204(0); T207(0); S208(0); T213(0.2); S214(99.8); S218(3.8); T219(96.2)]	0.032	-4.965784285	6.69E-12
Q9NQS7	3xPhospho [T298(99.3); S300(0.4); S302(0.4); S306(0); S311(0.4); S312(99.6); S314(100)]	1.95	0.963474124	0.423227315
Q9NQS7	2xPhospho [S306(100); S311(0); S312(100); S314(0)]	0.645	-0.632628934	0.797966154
Q9NQS7	2xPhospho [T195(0); S197(0); T199(0); S202(0); T204(0); T207(0); S208(0); T213(4.4); S214(95.5); S218(4.5); T219(95.7)]	0.68	-0.556393349	0.875944359
Q9NQS7	2xPhospho [T135(0); S142(0); S143(99.9); T145(0.1); S148(96.9); T150(3.1); T153(0)]	1.317	0.397255346	0.879063032
Q9NQS7	2xPhospho [T298(0); S300(0); S302(0.1); S306(99.9); S311(0.1); S312(99.9); S314(0)]	0.736	-0.442222329	0.904774194
Q9NQS7	3xPhospho [Y822(0); S828(100); S831(100); T832(100)]	0.98	-0.029146346	0.974924677
Q9NQU5	2xPhospho [S179(95.7); S189(95.7); S201(4); S202(4); S207(0.1); T210(0.2); T212(0.2)]	15.503	3.954475515	4.77E-06
Q9NQU5	2xPhospho [S224(0); S231(0); S236(0); T238(0); S246(100); S248(100)]	0.824	-0.279283757	0.960298686
Q9NQW6	1xPhospho [S45(0); S48(0); S54(100)]	0.439	-1.187707155	0.552200943
Q9NQW6	1xPhospho [S792(100); S795(0); S800(0)]	1.469	0.554834396	0.810156821
Q9NQW6	2xPhospho [S182(100); S189(0); S192(0); T194(100)]	1.385	0.469885976	0.855557911
Q9NQW6	1xPhospho [S72(100); T75(0); S80(0)]	1.183	0.242450074	0.925853562
Q9NQW6	1xPhospho [S182(100); S189(0); S192(0); T194(0)]	0.752	-0.411195433	0.956734015
Q9NQW6	1xPhospho [T320(0); S323(100)]	0.895	-0.160040413	0.994656428
Q9NQZ2	1xPhospho [T23(0); T25(0); S37(100); T41(0); S42(0); Y43(0); Y44(0)]	7.07	2.821710215	0.002663919
Q9NQZ2	2xPhospho [T354(0); S355(0); T362(0); S365(100); S368(100)]	0.612	-0.708396442	0.560990292
Q9NQZ2	1xPhospho [Y136(0); Y137(0); T139(0); Y141(0); S143(100)]	1.161	0.215367972	0.957850451
Q9NQZ2	1xPhospho [S150(100)]	0.929	-0.106249498	0.986075329
Q9NR09	3xPhospho [S473(0); S480(99.6); S482(0.4); S486(94.4); S488(5.9); S490(99.3); T492(0.4); T495(0); S496(0)]	0.01	-6.64385619	8.35E-17
Q9NR09	2xPhospho [S473(0); S480(88.3); S482(88.3); S486(2.3); S488(10.7); S490(8.7); T492(1.5); T495(0.1); S496(0.1)]	100	6.64385619	8.35E-17
Q9NR09	2xPhospho [S473(100); S480(100); S482(0); S486(0)]	0.931	-0.103146927	0.998086195
Q9NR12	1xPhospho [Y104(0); T105(0); S109(0); S111(100)]	0.882	-0.181149439	0.986075329
Q9NR19	1xPhospho [S263(0.1); T264(0.1); S265(0.1); S267(99.6)]	1.307	0.386259141	0.877728587
Q9NR19	1xPhospho [S28(0); S30(100); S36(0)]	0.922	-0.117161344	1
Q9NR30	3xPhospho [S164(0); S168(100); S171(100); S173(100)]	0.265	-1.915935735	0.006667428
Q9NR30	2xPhospho [S7(100); S13(100); T15(0)]	0.465	-1.104697379	0.178845723
Q9NR30	2xPhospho [S7(100); S13(100); T15(0)]	0.337	-1.569179503	0.183417508
Q9NR30	2xPhospho [S164(0); S168(100); S171(99.8); S173(0.2)]	0.728	-0.457989644	0.797966154
Q9NR30	1xPhospho [S121(100)]	1.151	0.202887833	0.9616322
Q9NR30	1xPhospho [S7(100); S13(0); T15(0)]	1.046	0.064882852	0.981858798
Q9NR30	1xPhospho [S65(0); S71(100)]	0.813	-0.298672743	0.987937869
Q9NR30	1xPhospho [S164(0); S168(0); S171(97); S173(3)]	0.989	-0.015957574	0.989554868
Q9NR48	2xPhospho [S1162(100); T1165(0); S1170(100); S1172(0); S1175(0); T1178(0); S1179(0)]	1.279	0.355016264	0.886312012
Q9NRF8	3xPhospho [S562(0); S563(0); S564(0); Y567(0); S568(100); S571(100); S574(100); S576(0)]	1.851	0.888304895	0.624566719
Q9NRF8	2xPhospho [S562(0); S563(0); S564(0); Y567(0); S568(100); S571(100); S574(0); S576(0)]	1.322	0.402722177	0.884786699
Q9NRG9	1xPhospho [S495(100)]	1.815	0.859969548	0.578933372
Q9NRH2	1xPhospho [S569(100); S570(0); S576(0); S584(0); S587(0); S590(0)]	1.302	0.380729449	0.872729954
Q9NRL2	1xPhospho [S1363(100); T1367(0); S1371(0)]	100	6.64385619	8.35E-17
Q9NRL2	2xPhospho [S1413(100); S1417(100); T1420(0)]	4.082	2.029276182	0.060075598
Q9NRL2	2xPhospho [S1413(100); S1417(100); T1420(0)]	1.961	0.971589536	0.485761708
Q9NRL2	1xPhospho [S1413(100); S1417(0); T1420(0)]	1.448	0.534061602	0.779297225
Q9NRL2	1xPhospho [T731(100); S739(0)]	1.529	0.612588407	0.781482911
Q9NRL2	1xPhospho [S1413(100); S1417(0); T1420(0)]	0.876	-0.190997225	0.998086195
Q9NRL3	1xPhospho [S260(0); S276(100); S284(0)]	1.236	0.305678743	0.922868578
Q9NRR5	1xPhospho [S307(0); S308(0); S313(0); S315(0); S316(0); S317(1.7); S318(98.2)]	1.696	0.76213617	0.700248628
Q9NRY2	2xPhospho [S35(2.4); S36(48.8); T37(48.8); S43(0); S47(0.3); S50(99.7)]	0.869	-0.202571918	0.988508396
Q9NRY4	1xPhospho [T951(0); Y954(0); S962(0); T963(0); T964(0); S970(100)]	10.114	3.338281778	0.000118659
Q9NRY4	2xPhospho [T1173(2.2); S1174(97.8); S1176(0); S1179(100)]	0.668	-0.582079992	0.890736743
Q9NRY4	2xPhospho [T1173(50); S1174(50); S1176(0); S1179(100)]	0.746	-0.422752464	0.953220244
Q9NRY4	1xPhospho [Y1105(2.4); S1106(95.2); S1111(2.4); T1112(0)]	1.052	0.073134705	0.960314241

Q9NS87	2xPhospho [S1134(0); S1141(100); T1144(100); T1150(0)]	0.01	-6.64385619	8.35E-17
Q9NS87	1xPhospho [T1144(100); T1150(0)]	1.936	0.953078953	0.634627188
Q9NS91	1xPhospho [T468(0); S471(100)]	1.468	0.553851968	0.769999353
Q9NS91	2xPhospho [S99(100); S103(100); S106(0); S107(0); S108(0); S109(0)]	1.19	0.250961574	0.938049121
Q9NSI6	2xPhospho [Y1603(0); S1605(100); S1607(100); S1611(0); T1614(0)]	0.01	-6.64385619	8.35E-17
Q9NSI6	2xPhospho [T1267(0.8); S1270(8.1); S1273(8.1); T1275(84.6); S1276(98.5); S1289(0)]	100	6.64385619	8.35E-17
Q9NTI5	1xPhospho [S1176(0); S1177(0.3); S1182(99.7); Y1187(0); T1188(0); S1190(0); S1191(0)]	100	6.64385619	8.35E-17
Q9NTI5	1xPhospho [S1319(0); S1334(99.9); T1337(0.1)]	0.621	-0.687334826	0.908462992
Q9NTI5	1xPhospho [S1283(100)]	1.112	0.153156788	0.962617576
Q9NTI5	2xPhospho [S1176(0); S1177(0.2); S1182(99.8); Y1187(0); T1188(0); S1190(0.2); S1191(99.8)]	0.678	-0.560642822	0.96293109
Q9NTI5	2xPhospho [S1176(96.5); S1177(3.5); S1182(100); Y1187(0); T1188(0); S1190(0); S1191(0)]	1.056	0.078609835	0.965139891
Q9NTI5	1xPhospho [T1255(0); S1257(100); S1259(0)]	1	0	0.974924677
Q9NTI5	2xPhospho [S1176(2.9); S1177(97.2); S1182(100); Y1187(0); T1188(0); S1190(0); S1191(0)]	0.971	-0.042456799	0.998086195
Q9NTJ3	2xPhospho [S22(100); S27(100); S28(0); S36(0)]	1.602	0.679874148	0.686714309
Q9NTJ3	2xPhospho [S22(100); S27(50); S28(50); S36(0)]	1.381	0.46571332	0.805664647
Q9NTJ3	3xPhospho [S22(100); S27(100); S28(100); S36(0)]	0.664	-0.590744853	0.893580559
Q9NTJ3	1xPhospho [T39(2.6); S41(97.4); T44(0); T48(0); S50(0)]	1.223	0.290424404	0.9072193
Q9NTJ3	1xPhospho [S22(0); S27(1.9); S28(98.1); S36(0)]	1.218	0.284514133	0.921247725
Q9NTJ3	2xPhospho [S22(100); S27(50); S28(50); S36(0)]	0.838	-0.254977851	0.975789705
Q9NTZ6	2xPhospho [S420(98.5); S422(50.7); S424(50.7); Y433(0)]	0.448	-1.158429363	0.742967319
Q9NTZ6	3xPhospho [S420(100); S422(100); S424(100); Y433(0)]	0.655	-0.610433188	0.767672756
Q9NTZ6	2xPhospho [S422(100); S424(100); Y433(0)]	0.811	-0.30222618	0.908083211
Q9NU22	1xPhospho [T4534(0); S4538(100); Y4543(0)]	1.616	0.692427198	0.661999767
Q9NU22	2xPhospho [S4752(100); S4754(100)]	0.593	-0.75389599	0.744877142
Q9NU22	2xPhospho [S4752(100); S4754(100)]	1.088	0.121678557	0.973599548
Q9NU22	1xPhospho [S4752(93.8); S4754(6.2)]	1.052	0.073134705	0.974924677
Q9NUN5	2xPhospho [S519(0); S528(100); S531(100); S536(0); Y538(0); S539(0)]	0.527	-0.924125133	0.816296058
Q9NUQ3	1xPhospho [S58(0); S60(0); S69(100); T74(0); S75(0)]	0.643	-0.637109357	0.797966154
Q9NUQ3	1xPhospho [S510(0); S517(98.3); T518(1.7); S520(0); S526(0)]	0.744	-0.426625474	0.958702638
Q9NUY8	2xPhospho [S553(0); Y560(0); T562(100); S567(0.2); S568(0.2); S569(0.2); S571(99.4)]	0.612	-0.708396442	0.860444802
Q9NV06	1xPhospho [S425(5.8); S429(94.2); S434(0)]	1.198	0.260627908	0.933760225
Q9NV70	1xPhospho [S487(0); S489(0); S490(0); S491(0); S499(0); S501(100)]	0.787	-0.345564459	0.969482098
Q9NVN3	1 2xPhospho [Y467(0); S468(100); T473(100); T475(0); Y478(0)]	1.802	0.849599011	0.647699093
Q9NVP1	1xPhospho [S31(100); T34(0); S36(0); T38(0); S44(0); T47(0); S50(0)]	0.458	-1.126580497	0.640854399
Q9NVP1	1xPhospho [S66(0); T68(0); S74(100)]	0.496	-1.011587974	0.749797187
Q9NVP1	1xPhospho [S66(0); T68(0); S74(100)]	0.837	-0.256700472	0.930376591
Q9NVU0	2xPhospho [S503(100); T521(0.1); S522(99.8); S524(0.1); S528(0)]	2.136	1.094911647	0.257416476
Q9NVU0	1xPhospho [S503(100); T521(0); S522(0); S524(0); S528(0)]	2.339	1.225891862	0.296268685
Q9NVU0	1xPhospho [T542(0); S544(100); S555(0); T556(0)]	1.834	0.874993639	0.63784228
Q9NVW2	2xPhospho [S228(100); S230(100); S236(0)]	0.93	-0.104697379	1
Q9NVZ3	1xPhospho [S181(0.1); T182(99.9); S186(0)]	1.293	0.370722275	0.889077103
Q9NW75	1xPhospho [S195(100); Y199(0); Y201(0); T205(0)]	1.052	0.073134705	0.969482098
Q9NW82	1xPhospho [T631(0); S638(100)]	100	6.64385619	8.35E-17
Q9NW82	1xPhospho [T631(0); S638(100)]	2.097	1.068326861	0.362923727
Q9NW82	1xPhospho [S4(0.1); S7(99.7); T10(0); S12(0.1); S15(0); T24(0); T28(0)]	0.688	-0.53951953	0.96229367
Q9NW97	2xPhospho [T104(0); S115(100); S127(100)]	0.56	-0.836501268	0.647581224
Q9NW97	1xPhospho [T104(0); S115(100); S127(0)]	1.089	0.123003954	0.970242377
Q9NWB6	1xPhospho [S266(100); S268(0)]	0.827	-0.274040765	0.996295697
Q9NWH9	2xPhospho [S550(4.2); S551(95.9); S553(99.8); T563(0)]	1.736	0.795766948	0.512995762
Q9NWH9	1xPhospho [S1019(100); S1021(0)]	1.85	0.887525271	0.526192815
Q9NWH9	1xPhospho [S553(100); T563(0)]	1.612	0.688851744	0.599097176
Q9NWH9	1xPhospho [T995(0); S998(0); S999(0); S1002(100)]	1.495	0.580145484	0.749797187
Q9NWH9	1xPhospho [S553(100); T563(0)]	1.249	0.320773477	0.911330706
Q9NWH9	2xPhospho [S550(1.8); S551(98.3); S553(100); T563(0)]	1.208	0.272620455	0.930376591
Q9NWH9	2xPhospho [S550(0.1); S551(99.9); S553(100); T563(0)]	0.94	-0.089267338	0.980895643
Q9NWK9	2xPhospho [S11(0); S17(100); S25(100)]	0.8	-0.321928095	0.952323823
Q9NWS9	2xPhospho [T131(0); S137(100); S141(0); T143(0); S146(100); T154(0)]	0.643	-0.637109357	0.87821128
Q9NWZ5	1xPhospho [Y529(0); T532(94.1); S539(5.9); Y546(0); T547(0)]	100	6.64385619	8.35E-17
Q9NWZ8	2xPhospho [S117(0); T124(100); S126(100); S135(0); T140(0)]	2.069	1.048933645	0.407997255
Q9NX31	2xPhospho [S82(100); S84(100)]	0.809	-0.305788392	0.980895643
Q9NX55	1xPhospho [S57(0); S58(0); T62(0); S65(100)]	1.744	0.80240004	0.607772032
Q9NX65	1xPhospho [S292(2.8); S294(2.8); S297(94.5)]	1.484	0.569491092	0.922395265
Q9NXG2	1xPhospho [T313(0); T319(0); T322(0); S323(0); S339(99.3); T342(0.7); S345(0)]	0.01	-6.64385619	8.35E-17
Q9NXG2	2xPhospho [T79(0); S86(100); S88(100)]	0.775	-0.367731785	0.870237685
Q9NXG2	2xPhospho [T79(0); S86(100); S88(100)]	1.216	0.282143229	0.927625482
Q9NXH9	1xPhospho [Y622(0); S623(0); S625(100); T628(0)]	1.152	0.204140717	0.938730361
Q9NXX6	2xPhospho [S54(0); T58(0.7); S61(99.3); S63(99.9); S72(0.1)]	7.207	2.849398845	0.000450208
Q9NY27	1xPhospho [S166(0); T173(100)]	3.187	1.672199019	0.214289747
Q9NY27	1xPhospho [S159(0); S161(1.5); Y162(0); T163(98.5)]	1.5	0.584962501	0.759239289
Q9NY27	1xPhospho [S213(0); S215(0); S216(0); T217(0); S218(0); S220(0); S223(0); S224(0); S226(100)]	1.317	0.397255346	0.879063032
Q9NY27	2xPhospho [S213(0); S215(0); S216(0); T217(0); S218(100); S220(0); S223(0); S224(0); S226(100)]	0.996	-0.005782353	0.99184412
Q9NY61	3xPhospho [Y305(0); T310(0); S316(100); S320(100); S321(100)]	0.201	-2.314732593	0.000219406
Q9NY61	3xPhospho [Y305(0); T310(0); S316(100); S320(100); S321(100)]	0.382	-1.388355457	0.20634176
Q9NY61	2xPhospho [Y305(0); T310(0); S316(0); S320(100); S321(100)]	0.784	-0.351074441	0.936043234

Q9NY61	1xPhospho [S203(100); T211(0); S213(0); S214(0)]	0.868	-0.204233052	0.97726974
Q9NYB0	1xPhospho [Y195(0); S203(98.9); S205(1.1); S206(0)]	0.829	-0.270555993	0.965013037
Q9NYB9	2xPhospho [T174(0); T175(0); T178(0); S183(100); S187(100)]	3.03	1.599317794	0.136001631
Q9NYB9	1xPhospho [T174(0); T175(0); T178(0); S183(2.7); S187(97.3)]	1.835	0.875780063	0.513538425
Q9NYF3	2xPhospho [S250(50); S251(50); S254(0); S255(100)]	1.345	0.427606173	0.832564075
Q9NYF3	1xPhospho [S150(0); S162(100)]	1.474	0.559736524	0.838893449
Q9NYF3	1xPhospho [S150(0); S162(100)]	1.1	0.137503524	0.942031377
Q9NYF3	3xPhospho [S250(50); S251(50); S254(100); S255(100)]	0.674	-0.569179503	0.94797789
Q9NYF3	2xPhospho [S232(100); S234(50); S236(50); S242(0)]	0.903	-0.147202107	0.988250665
Q9NYF8	4 1xPhospho [S339(100)]	0.516	-0.954557029	0.759239289
Q9NYL2	1xPhospho [S591(0.1); S593(99.9); S599(0); S603(0); S610(0); Y611(0)]	0.879	-0.18606493	0.97998947
Q9NYL2	1xPhospho [S648(100); S649(0); S654(0)]	0.918	-0.123433941	0.992371558
Q9NYP7	1xPhospho [S273(100); T281(0); S283(0); S285(0)]	0.592	-0.756330919	0.819087074
Q9NYV4	2xPhospho [S332(99.1); S333(50.5); S334(50.5); S338(0)]; J3QSD7 2xPhospho [S332(99.1); S333(50.5); S334(50.5); S338(0)]	1.797	0.845590409	0.504163686
Q9NYV4	3xPhospho [S382(0); S383(100); S385(100); S393(50); S394(50); S400(0)]; J3QSD7 3xPhospho [S381(0); S382(100); S384(100); S392(50); S393(50); S399(0)]	0.429	-1.220950447	0.617898411
Q9NYV4	1xPhospho [Y884(0); S886(0); S889(0); Y892(0); T893(100)]; J3QSD7 1xPhospho [Y883(0); S885(0); S888(0); Y891(0); T892(100)]	1.591	0.669933836	0.679491559
Q9NYV4	2xPhospho [S382(0.1); S383(99.9); S385(100); S393(0); S394(0); S400(0)]; J3QSD7 2xPhospho [S381(0.1); S382(99.9); S384(100); S392(0); S393(0); S399(0)]	0.664	-0.590744853	0.685087581
Q9NYV4	3xPhospho [S379(0); S382(100); S383(100); S385(100); S393(0); S394(0); S400(0)]; J3QSD7 3xPhospho [S378(0); S381(100); S382(100); S384(100); S392(0); S393(0); S399(0)]	1.537	0.620117165	0.718198219
Q9NYV4	2xPhospho [T667(0); S681(100); S685(100)]; J3QSD7 2xPhospho [T666(0); S680(100); S684(100)]	0.701	-0.512513651	0.755213977
Q9NYV4	2xPhospho [S274(100); S276(100); Y279(0); S283(0); Y285(0); S287(0); S288(0); T289(0)]; J3QSD7 2xPhospho [S274(100); S276(100); Y279(0); S283(0); Y285(0); S287(0); S288(0); T289(0)]	0.816	-0.293358943	0.91281798
Q9NYV4	1xPhospho [S423(100)]; J3QSD7 1xPhospho [S422(100)]	0.691	-0.533242384	0.933107775
Q9NYV4	3xPhospho [S341(100); S343(100); S345(100); S349(0)]	0.83	-0.268816758	0.965139891
Q9NYV4	3xPhospho [S341(100); S343(100); S345(98.1); S349(2)]	0.902	-0.148800661	0.975403456
Q9NYV4	2xPhospho [S341(0.1); S343(100); S345(100); S349(0)]	0.896	-0.158429363	0.981208663
Q9NYV4	3xPhospho [S274(100); S276(100); Y279(0); S283(25); Y285(0); S287(25); S288(25); T289(25)]; J3QSD7 3xPhospho [S274(100); S276(100); Y279(0); S283(25); Y285(0); S287(25); S288(25); T289(25)]	0.862	-0.214240226	0.99184412
Q9NYV6	2xPhospho [S170(100); S172(100); T185(0)]	0.354	-1.498178735	0.050168989
Q9NYV6	1xPhospho [S44(100)]	1.087	0.12035194	0.953026796
Q9NYZ3	2xPhospho [S575(100); S580(100); S583(0)]	1.098	0.134878054	0.9616322
Q9NZ63	1xPhospho [T253(0); S261(100)]	1.961	0.971589536	0.609633086
Q9NZ63	1xPhospho [T253(0); S261(100)]	1.574	0.654435541	0.659346224
Q9NZJ0	2xPhospho [S485(100); S487(0); S488(1.7); S490(98.3); S495(0); S496(0)]	0.456	-1.13289427	0.233657048
Q9NZJ0	3xPhospho [S485(100); S487(2.9); S488(97.1); S490(100); S495(0); S496(0)]	0.562	-0.831357964	0.786602611
Q9NZJ0	2xPhospho [T508(0); S510(0); S511(0); S512(100); T516(100); S520(0); T522(0)]	0.652	-0.61705613	0.872730156
Q9NZJ0	1xPhospho [S485(100); S487(0); S488(0); S490(0); S495(0); S496(0)]	0.726	-0.461958547	0.973763863
Q9NZL4	1xPhospho [S19(0); S23(4.7); S24(4.7); S34(90.4); S35(0.3); S39(0); S42(0)]	1.56	0.641546029	0.714293599
Q9NZM3	2xPhospho [T882(0.1); S884(99.9); S887(0); S889(100)]	0.638	-0.648371671	0.651510528
Q9NZM3	1xPhospho [T882(0); S884(0); S887(0.1); S889(99.9)]	0.698	-0.518701058	0.969482098
Q9NZN5	2 2xPhospho [S308(0); T310(0); T314(0); T316(0); S318(100); S322(98.2); S324(1.7); T325(0)]	0.805	-0.312939312	0.969482098
Q9NZN5	2 1xPhospho [T1267(0); S1269(100); T1274(0); S1276(0); S1281(0)]	0.76	-0.395928676	0.974924677
Q9NZN5	2 1xPhospho [T713(0); T717(100)]	0.847	-0.239566125	0.985318278
Q9NZN5	2 2xPhospho [S22(100); S28(99.9); T30(0.2)]	0.872	-0.19759996	0.992863986
Q9NZT2	2xPhospho [S361(0); S378(100); S382(100)]	100	6.64385619	8.35E-17
Q9NZT2	1xPhospho [S315(100); S324(0); T325(0)]	3.026	1.597411988	0.100847056
Q9NZT2	1xPhospho [S349(100)]	1.339	0.421155961	0.831039794
Q9NZT2	2xPhospho [S361(100); S378(100)]	0.804	-0.314732593	0.973599548
Q9NZT2	1xPhospho [S361(0); S378(100)]	1.03	0.042644337	0.979498769
Q9NZZ3	1xPhospho [S86(100); Y94(0); T95(0); S98(0)]	0.911	-0.134477041	0.99811985
Q9P035	1xPhospho [S114(100)]	0.578	-0.790858602	0.465755251
Q9P035	1xPhospho [S114(100)]	0.548	-0.867752202	0.85992414
Q9P0K7	3xPhospho [S383(88); S386(20.9); Y387(20.9); S389(79.1); T390(79.1); T392(12); S397(0); T404(0); S405(0); S409(0)]	0.586	-0.77102743	0.900153261
Q9P0K8	2xPhospho [S161(0); S164(100); S170(0); S172(100)]	0.644	-0.634867407	0.87998572
Q9P0K8	2xPhospho [S161(0); S164(100); S170(2.5); S172(97.5)]	1.027	0.038436182	0.973599548
Q9P0L0	1xPhospho [S209(0); S214(100); T215(0); S216(0); T217(0); S219(0)]	0.917	-0.125006361	0.997077639
Q9P0L2	2xPhospho [S463(100); T464(0); T465(0); S468(97.5); S470(2.5); T473(0); S475(0)]	0.706	-0.502259911	0.922170166
Q9P0P8	2xPhospho [S106(96.6); S110(3.4); S116(100); T127(0)]	1.745	0.803227036	0.576490774
Q9P0P8	2xPhospho [S106(100); S110(0); S116(100); T127(0)]	1.032	0.045442971	0.97998947
Q9P0V3	1xPhospho [S296(100); S306(0); T312(0); T317(0)]	0.548	-0.867752202	0.842409333
Q9P1Y5	1xPhospho [T338(99.9); S341(0.1); S347(0); S349(0); S350(0); S351(0); T355(0)]	0.701	-0.512513651	0.763548117
Q9P1Y5	2xPhospho [T338(95.3); S341(4.7); S347(0); S349(0); S350(4.7); S351(95.3); T355(0)]	0.672	-0.573466862	0.938483927
Q9P203	2xPhospho [T964(50.7); S966(98.6); S968(50.7); Y972(0); Y978(0); S979(0)]	0.44	-1.184424571	0.679191074
Q9P203	1xPhospho [S1119(100); S1121(0); Y1127(0)]	1.098	0.134878054	0.936043234
Q9P206	2xPhospho [S971(100); S979(100); S981(0); Y982(0)]	1.462	0.547943311	0.768341527
Q9P206	1xPhospho [S862(100); S868(0); S869(0)]	1.234	0.303342394	0.910211107
Q9P206	1xPhospho [S929(100); T932(0)]	0.96	-0.058893689	0.974924677
Q9P206	2xPhospho [S669(100); S673(100); S677(0); Y678(0); T685(0)]	0.915	-0.128156351	0.976595096

Q9P206	2xPhospho [S858(100); S862(100); S868(0); S869(0)]	1.021	0.029982866	0.977858961
Q9P206	3xPhospho [S669(100); S673(100); S677(100); Y678(0); T685(0)]	1.049	0.069014678	0.980895643
Q9P241	3xPhospho [S637(2.8); S638(2.8); S639(16.5); S641(87.6); S644(88.3); S649(51); S650(51); S658(0)]	1.644	0.717210299	0.704348458
Q9P244	2xPhospho [Y704(50); S705(50); Y710(0); S716(94.8); S718(5.2); Y719(0)]	0.891	-0.166502663	0.999834587
Q9P246	1xPhospho [S661(100); S665(0); S668(0)]	100	6.64385619	8.35E-17
Q9P246	2xPhospho [T696(0.1); S697(99.9); S699(50); S700(50); S705(0); S713(0)]	0.534	-0.905088353	0.660888692
Q9P246	1xPhospho [S518(0); S522(0); S523(100)]	1.232	0.301002256	0.912029092
Q9P246	1xPhospho [S678(2); S680(98.1); S685(0); S686(0)]	0.763	-0.390245038	0.972084413
Q9P246	1xPhospho [T696(0); S697(100); S699(0); S700(0); S705(0); S713(0)]	1.043	0.060739158	0.973763863
Q9P260	3xPhospho [S180(96.4); S182(3.8); T183(99.9); S186(100)]	0.297	-1.751465164	0.315251608
Q9P265	1xPhospho [Y98(0); S100(100); T104(0)]	1.031	0.044044333	0.980895643
Q9P270	2xPhospho [S462(100); S467(100)]	100	6.64385619	8.35E-17
Q9P270	2xPhospho [T313(3.6); S315(96.4); S323(100); Y336(0)]	0.538	-0.894321922	0.491917574
Q9P270	1xPhospho [S413(100); S418(0)]	1.543	0.625738062	0.728494169
Q9P270	2xPhospho [T313(50); S315(50); S323(100); Y336(0)]	0.631	-0.66428809	0.855557911
Q9P270	1xPhospho [S391(100); T397(0); T401(0); S402(0)]	0.807	-0.309359421	0.969482098
Q9P287	2xPhospho [T109(0); S112(100); S115(100); S129(0); T134(0)]	0.01	-6.64385619	8.35E-17
Q9P287	1xPhospho [S12(100)]	1.141	0.190298792	0.965013037
Q9P2B4	3xPhospho [S556(0); S557(0); S560(100); S563(100); S568(95.4); T570(4.6)]	1.739	0.798257933	0.714293599
Q9P2B4	1xPhospho [S468(0); S477(0); S481(3.8); S484(96.2)]	1.527	0.610700062	0.744877142
Q9P2B4	2xPhospho [S556(0); S557(0); S560(100); S563(0); S568(98.2); T570(1.8)]	0.757	-0.401634795	0.960298686
Q9P2C4	2xPhospho [S575(5.4); S580(94.6); Y586(0); S588(100); Y590(0)]	1.434	0.520045024	0.80163956
Q9P2D1	1xPhospho [S2559(100); T2567(0)]	100	6.64385619	8.35E-17
Q9P2E9	1xPhospho [T610(0); S615(100)]	1.212	0.277389699	0.925724009
Q9P2H5	3xPhospho [T688(100); S699(100); T700(100)]	100	6.64385619	8.35E-17
Q9P2I0	3xPhospho [S419(100); S420(100); S423(100); S433(0)]	0.275	-1.862496476	0.013575192
Q9P2K3	2xPhospho [S156(100); T163(0); S171(93.3); Y173(6.7)]	0.01	-6.64385619	8.35E-17
Q9P2K3	2xPhospho [S156(100); T163(0); S171(100); Y173(0)]	0.594	-0.751465164	0.720161581
Q9P2K3	1xPhospho [S156(100); T163(0); S171(0); Y173(0)]	1.314	0.393965276	0.884551994
Q9P2K3	2xPhospho [S156(100); T163(0); S171(100); Y173(0)]	0.684	-0.54793177	0.93019184
Q9P2K3	2xPhospho [S156(100); T163(0); S171(100); Y173(0)]	1.171	0.227741076	0.965013037
Q9P2K3	2xPhospho [S366(0); S368(0); S372(3.2); S375(96.9); T376(99.9); T384(0)]	0.806	-0.311148256	0.971220368
Q9P2M7	1xPhospho [S147(0); S149(100)]	100	6.64385619	8.35E-17
Q9P2M7	2xPhospho [S129(0); S131(100); S134(100); S144(0)]	0.661	-0.597277823	0.743120089
Q9P2N6	2xPhospho [S523(0); S525(0); S527(0); S531(0); S532(0); S534(0.2); S535(4.1); S536(95.7); T538(0); S539(0.2); S540(99.8)]	10.373	3.374761295	0.000757198
Q9P2N6	2xPhospho [S511(100); S515(100)]	1.9	0.925999419	0.512995762
Q9P2R6	2xPhospho [Y50(0); S53(100); S56(100); S65(0); T67(0); S71(0); T72(0)]	0.01	-6.64385619	8.35E-17
Q9P2R6	3xPhospho [S1106(100); S1113(100); S1115(100); T1119(0); T1123(0); S1125(0); S1128(0); S1130(0)]	1.831	0.872631791	0.498227019
Q9P2R6	2xPhospho [S594(100); T599(0.1); S600(99.9)]	1.125	0.169925001	0.962617576
Q9P2R6	2xPhospho [T671(0); S675(100); S679(100); S681(0); S687(0); S688(0); S690(0)]	1	0	0.975403456
Q9P2W9	1xPhospho [S187(100); S189(0.1); S191(0); S194(0); T200(0)]	2.001	1.000721167	0.482124103
Q9UBB9	1xPhospho [T50(0); Y51(0); S59(100); S65(0)]	1.592	0.670840336	0.690107651
Q9UBB9	1xPhospho [S98(100)]	1.711	0.77483976	0.70657612
Q9UBB9	1xPhospho [S98(100)]	1.235	0.304511042	0.918281661
Q9UBB9	1xPhospho [T198(0); T199(0); S201(0); S210(100)]	1.211	0.276198865	0.925808485
Q9UBB9	1xPhospho [S98(100)]	1.065	0.09085343	0.974924677
Q9UBB9	1xMet-loss+Acetyl [N-Term]; 1xPhospho [S2(100); S4(0); Y7(0)]	0.853	-0.229382353	0.996295697
Q9UBC2	1xPhospho [S238(0); T239(0); S241(0); S244(0); S246(0); S247(0); S250(0); T251(0); S253(0); S255(100)]	0.614	-0.703689439	0.849882438
Q9UBC2	1xPhospho [T217(0); S229(100)]	0.76	-0.395928676	0.973263372
Q9UBC2	2xPhospho [S238(0); T239(0); S241(0); S244(100); S246(0); S247(0); S250(0); T251(0); S253(0); S255(100)]	1.033	0.046840254	0.974924677
Q9UBL6	2xPhospho [S619(0); S628(100); T632(100)]	100	6.64385619	8.35E-17
Q9UDY2	1xPhospho [T1124(0); T1131(100); T1135(0); S1136(0); S1137(0)]	100	6.64385619	8.35E-17
Q9UDY2	1xPhospho [S1156(0); Y1157(0); S1159(100); Y1166(0)]	3.043	1.605494334	0.071098228
Q9UDY2	2xPhospho [S440(2.8); S441(97.2); T445(0); S447(97.2); S450(2.8)]	1.863	0.897627674	0.436160292
Q9UDY2	3xPhospho [Y423(0); S424(100); Y426(0); Y428(0); S430(97.3); S431(51.4); S432(51.4)]	2.223	1.152507948	0.437735427
Q9UDY2	2xPhospho [Y423(0); S424(0); Y426(0); Y428(0); S430(100); S431(99.9); S432(0.1)]	1.939	0.955312803	0.476899892
Q9UDY2	1xPhospho [S130(100)]	2.017	1.012211084	0.484693307
Q9UDY2	2xPhospho [T1124(0); T1131(100); T1135(33.3); S1136(33.3); S1137(33.3)]	2.037	1.02644598	0.577062363
Q9UDY2	1xPhospho [S461(1.7); T462(98.3); T468(0); T473(0)]	1.721	0.783247097	0.629129249
Q9UDY2	1xPhospho [S986(100)]	1.482	0.567545448	0.669782374
Q9UDY2	2xPhospho [S978(97.4); S979(2.6); S986(100)]	1.531	0.614474283	0.678076683
Q9UDY2	2xPhospho [S1048(0); S1053(3.4); T1054(96.6); S1067(100); S1068(0)]	1.44	0.526068812	0.754388643
Q9UDY2	2xPhospho [S978(99.9); S979(0.1); S986(100)]	1.209	0.273814245	0.897258645
Q9UDY2	2xPhospho [S952(0); S953(0); S961(100); S966(100)]	1.142	0.191562651	0.949641009
Q9UDY2	1xPhospho [S1156(0.1); Y1157(0.1); S1159(91.8); Y1166(0); S1171(7.5); S1174(0.7)]	0.789	-0.341902795	0.964978421
Q9UER7	1xPhospho [T401(0); S402(0); S403(0); S405(0); T408(0); S412(0); S415(0); S420(0); S424(99.9); S429(0.1); S431(0)]	100	6.64385619	8.35E-17
Q9UER7	2xPhospho [T722(0); S723(0); T726(0); S737(100); S739(100)]	0.633	-0.659722595	0.854283637
Q9UER7	1xPhospho [S495(100); S498(0); S499(0); S503(0)]	1.234	0.303342394	0.921519407

Q9UER7	2xPhospho [S688(96.7); S690(3.3); T695(0); S696(0); S697(0); S702(100)]	0.847	-0.239566125	0.965139891
Q9UER7	1xPhospho [S707(100); T709(0); S712(0); T719(0)]	1.058	0.081339627	0.969482098
Q9UEW8	1xPhospho [T377(0); S385(100)]	1.414	0.49978212	0.786760931
Q9UEY8	1xPhospho [S673(0); S677(0); S679(1.7); S681(96.7); S683(1.7)]	1.928	0.947105052	0.535347951
Q9UEY8	2xPhospho [S673(0); S677(100); S679(0); S681(100); S683(0)]	1.775	0.827819025	0.563180779
Q9UEY8	2xPhospho [S673(0); S677(100); S679(0); S681(98); S683(2)]	1.722	0.784085143	0.629245303
Q9UEY8	3xPhospho [S673(100); S677(100); S679(0); S681(100); S683(0)]	0.907	-0.140825544	1
Q9UFC0	1xPhospho [S241(0); S243(98.6); S245(1.4)]	0.339	-1.560642822	0.423291112
Q9UFC0	1xPhospho [S212(100)]	1.858	0.893750502	0.550634186
Q9UFC0	2xPhospho [S251(99.9); S253(0.1); S259(100); S264(0); S267(0)]	0.802	-0.318325858	0.966549726
Q9UFC0	1xPhospho [S241(0); S243(100); S245(0)]	0.836	-0.258425153	0.975403456
Q9UGR2	2xPhospho [S387(99.2); S393(0.4); T398(2.2); S400(2.2); S407(2.5); S415(93.6); T423(0)]	0.01	-6.64385619	8.35E-17
Q9UGU0	1xPhospho [S8(97.4); S9(2.6); Y10(0); S16(0); Y17(0); S24(0); S25(0)]	1.565	0.646162657	0.801587115
Q9UGU5	2xPhospho [S502(100); T505(0); T506(0); S512(100)]	100	6.64385619	8.35E-17
Q9UGU5	2xPhospho [S502(100); T505(0); T506(0); S512(100)]	100	6.64385619	8.35E-17
Q9UGY1	2xPhospho [T124(0); S134(94.9); S139(94.9); S140(9.5); T141(0.8); T145(0)]	1.21	0.275007047	0.925808485
Q9UGY1	3xPhospho [T124(0); S134(0.3); S139(99.7); S140(100); T141(100); T145(0)]	1.072	0.100304906	0.973599548
Q9UHB6	2xPhospho [S350(0); S362(100); S365(100)]	0.01	-6.64385619	8.35E-17
Q9UHB6	2xPhospho [S225(100); S228(0); Y229(0); S230(0); S242(0); S243(0); S244(0); T245(0); S248(50); S253(50)]	0.285	-1.810966176	0.100427249
Q9UHB6	2xPhospho [S225(99.9); S228(99.9); Y229(0); S230(0.2); S242(0); S243(0); S244(0); T245(0); S248(0)]	0.504	-0.988504361	0.550634186
Q9UHB6	1xPhospho [T487(0); S490(100)]	0.633	-0.659722595	0.730212284
Q9UHB6	2xPhospho [S588(0); T592(0); S596(0.1); S599(100); T600(0); S601(0); S604(100)]	0.531	-0.913216234	0.747592617
Q9UHB6	1xPhospho [S350(0); S362(100); S365(0)]	0.688	-0.53951953	0.797966154
Q9UHB6	1xPhospho [S671(0); S686(100); S692(0)]	0.697	-0.520769439	0.836554173
Q9UHB6	1xPhospho [S225(99.9); S228(0.1); Y229(0); S230(0); S242(0); S243(0); S244(0); T245(0); S248(0)]	0.588	-0.76611194	0.848293321
Q9UHB6	1xPhospho [S132(100)]	0.753	-0.40927823	0.97726974
Q9UHB7	2xPhospho [S703(100); S706(100); Y712(0)]	0.549	-0.865121946	0.574580382
Q9UHB7	2xPhospho [S487(100); S490(3.7); S491(96.3); S494(0); S498(0); S499(0); Y502(0)]	1.128	0.173767068	0.960298686
Q9UHB7	2xPhospho [S271(0); S272(0); Y275(0); S276(33.3); S277(33.3); S279(33.3); S283(99.8); T285(0.2); S290(0); S291(0)]	0.716	-0.481968507	0.965139891
Q9UHB7	2xPhospho [S176(2.5); S178(2.5); S179(95.1); S180(99.8); S188(0); S189(0); S192(0); S193(0); S195(0)]	0.776	-0.365871442	0.973763863
Q9UHB7	1xPhospho [S271(0); S272(0.3); Y275(0); S276(0); S277(0); S279(0); S283(99.3); T285(0.3); S290(0); S291(0)]	0.91	-0.13606155	0.994865845
Q9UHD8	3xPhospho [S30(100); T38(100); S41(50); T42(50)]	0.01	-6.64385619	8.35E-17
Q9UHD8	1xPhospho [T177(0); T181(100)]	2.613	1.385707125	0.198644628
Q9UHD8	1xPhospho [S22(100); S23(0)]	2.242	1.164786278	0.313232504
Q9UHD8	1xPhospho [S30(100); T38(0); S41(0); T42(0)]	0.518	-0.948975997	0.450314104
Q9UHD8	1xPhospho [S238(0); T242(0); S247(100); T255(0)]	1.527	0.610700062	0.7222669
Q9UHD8	2xPhospho [S30(100); T38(0); S41(0.1); T42(100)]	1.198	0.260627908	0.93864152
Q9UHD8	2xPhospho [S30(100); T38(0); S41(1.7); T42(98.3)]	0.687	-0.541617996	0.965013037
Q9UHD8	1xPhospho [T150(0); T153(0); S160(100)]	0.709	-0.496142467	0.965139891
Q9UHD8	1xPhospho [S30(100); T38(0); S41(0); T42(0)]	0.859	-0.219269964	0.965351295
Q9UHD8	2xPhospho [S30(100); T38(0); S41(50); T42(50)]	1.026	0.037030731	0.977858961
Q9UHD8	1xPhospho [T150(0); T153(0); S160(100)]	1.017	0.024319679	0.97998947
Q9UHI6	1xPhospho [S695(0); S703(98.1); T705(1.9)]	1.048	0.067638717	0.971220368
Q9UHI6	1xPhospho [S672(0); Y673(0); S677(4.2); S678(95.9); S685(0); S687(0); T688(0)]	0.816	-0.293358943	0.973263372
Q9UHJ3	2xPhospho [T763(0); S765(0); S767(100); S775(100)]	2.078	1.055195654	0.469536943
Q9UHJ3	3xPhospho [T763(0); S765(100); S767(100); S775(100)]	0.744	-0.426625474	0.94913059
Q9UHK0	3xPhospho [S338(100); S340(99.8); S342(100); S351(0.3)]	0.232	-2.10780329	0.001692381
Q9UHR4	1xPhospho [T248(0); S251(0); T252(0); S255(0); T257(0); S261(100)]	2.387	1.255198566	0.234486881
Q9UHR4	1xPhospho [S329(0); S331(100); T334(0)]	1.391	0.47612242	0.807956501
Q9UHR4	2xPhospho [T248(0); S251(0); T252(0); S255(0); T257(100); S261(100)]	1.193	0.254594043	0.951285684
Q9UHV7	2xPhospho [T519(4.5); T525(0.2); S530(95.3); S537(100); T549(0)]	0.851	-0.232768963	0.962617576
Q9UHV7	2xPhospho [S321(0); S322(0); T324(0); T326(3.7); T329(96.3); S330(100); T336(0); S341(0)]	0.8	-0.321928095	0.973263372
Q9UHX1	1xPhospho [T39(0.1); S41(99.9); S49(0); T50(0)]	1.649	0.721591399	0.636840295
Q9UIF7	1xPhospho [S532(0); S535(2.5); T536(95.1); S540(2.5); S543(0)]	0.954	-0.067938829	0.99184412
Q9UIG0	2xPhospho [S152(0); S158(99.8); S160(4.2); S161(96); S166(0); S167(0)]	3.972	1.989865623	0.021671745
Q9UIG0	2xPhospho [S152(0); S158(99.9); S160(3.1); S161(97); S166(0); S167(0)]	3.766	1.913033	0.028594439
Q9UIG0	1xPhospho [S359(0); S361(100)]	3.078	1.621993232	0.098569829
Q9UIG0	1xPhospho [S1468(100); S1476(0)]	1.594	0.672651629	0.691656303
Q9UIG0	2xPhospho [S699(0); S705(100); S708(100); T710(0); S716(0)]	0.666	-0.586405918	0.725426297
Q9UIG0	1xPhospho [S152(0); S158(0); S160(2.6); S161(97.4); S166(0); S167(0)]	1.265	0.339137385	0.925912375
Q9UIG0	2xPhospho [S699(0); S705(100); S708(99.9); T710(0.1); S716(0)]	0.949	-0.075520008	0.980895643
Q9UJA5	1xPhospho [S298(100); S305(0); S312(0); T320(0); T323(0); S325(0)]	0.522	-0.937878288	0.64957454
Q9UJM3	1xPhospho [S337(95.1); S340(4.9); Y341(0); T349(0); S351(0)]	0.01	-6.64385619	8.35E-17
Q9UJM3	2xPhospho [T119(0); S126(0.1); T127(99.9); T131(100)]	0.105	-3.251538767	0.000160321
Q9UJM3	2xPhospho [S249(0); S251(100); S256(100)]	0.403	-1.311148256	0.197641716
Q9UJM3	1xPhospho [S249(0); S251(100); S256(0)]	0.657	-0.606034724	0.670368081
Q9UJM3	1xPhospho [S326(100); S328(0); S330(0)]	1.551	0.633198686	0.724397901
Q9UJM3	2xPhospho [S273(100); S276(100)]	1.463	0.548929769	0.790092665
Q9UJM3	1xPhospho [S273(100); S276(0)]	1.229	0.297484916	0.94131536

Q9UJU6	1xPhospho [T291(100); T295(0)]	0.01	-6.64385619	8.35E-17
Q9UJX2	1xPhospho [T562(0); T564(0); T565(0); S576(0); S578(100); T582(0); T584(0)]	0.693	-0.529072743	0.870654365
Q9UJX2	2xPhospho [S588(100); S593(0); S594(0); T596(100)]	0.893	-0.16326792	0.981858798
Q9UK59	1xPhospho [T496(0); S499(100); T506(0)]	100	6.64385619	8.35E-17
Q9UK61	1xPhospho [S971(0); S972(0); Y974(0); S978(2.6); S979(94.8); T982(2.6); T984(0.1)]	0.659	-0.60164963	0.938483927
Q9UK76	2xPhospho [S28(100); S31(100); T38(0)]	0.528	-0.921390165	0.590872099
Q9UK76	2xPhospho [S28(100); S31(100); T38(0)]	0.574	-0.800877358	0.622027301
Q9UK76	2xPhospho [T110(0.3); S115(99.7); S119(0); S131(100); S140(0)]	1.678	0.746742716	0.636316981
Q9UK76	1xPhospho [S28(100); S31(0); T38(0)]	0.734	-0.446148032	0.808799965
Q9UK76	2xPhospho [S87(0); S88(100); S91(100); S92(0)]	0.788	-0.343732465	0.879063032
Q9UK76	3xPhospho [S87(100); S88(100); S91(100); S92(0)]	0.595	-0.749038426	0.920957334
Q9UK76	1xPhospho [T110(0); S115(100); S119(0); S131(0); S140(0)]	0.843	-0.246395464	0.963250338
Q9UK76	1xPhospho [S87(98.9); S88(1.1); S91(0); S92(0)]	0.859	-0.219269964	0.971220368
Q9UK76	2xPhospho [S70(97.1); S71(2.9); S79(100); S80(0)]	0.866	-0.20756107	0.971973958
Q9UK76	1xPhospho [S28(100); S31(0); T38(0)]	0.945	-0.081613766	0.981858798
Q9UK76	1xPhospho [S70(99.1); S71(0.9); S79(0); S80(0)]	1.029	0.041242982	0.992376868
Q9UKA4	2xPhospho [S422(100); Y424(0); S433(100)]	0.53	-0.915935735	0.467067441
Q9UKA4	2xPhospho [Y1335(0); S1337(100); S1340(100); T1342(0); Y1345(0)]	1.346	0.42867841	0.872730156
Q9UKA4	1xPhospho [T1100(100); S1103(0); T1104(0); S1109(0)]	0.739	-0.436353731	0.939053555
Q9UKA4	1xPhospho [S18(100); S20(0); S26(0)]	0.685	-0.545824107	0.95142727
Q9UKA4	1xPhospho [S1240(0); S1242(100); T1244(0); S1249(0)]	0.879	-0.18606493	0.980895643
Q9UKA4	2xPhospho [S970(0); S974(0); S978(0); T981(100); S985(100)]	0.959	-0.06039728	0.981208663
Q9UKA4	2xPhospho [T1100(100); S1103(2); T1104(98); S1109(0)]	1.029	0.041242982	0.983265186
Q9UKA4	2xPhospho [T1136(100); S1139(6.9); T1140(93.1); S1144(0); S1145(0); S1148(0); S1150(0); T1156(0)]	0.93	-0.104697379	0.998086195
Q9UKI2	2xPhospho [S89(100); T90(0); S91(0); S93(0); T96(0); T98(0); S100(100)]	0.858	-0.220950447	0.989304921
Q9UKI8	1xPhospho [S741(96.3); S743(3.5); S744(0.1); T753(0); S755(0); T757(0); S760(0); S761(0); S762(0); T765(0); Y766(0)]	0.541	-0.886299501	0.74242706
Q9UKI8	3xPhospho [S170(4.1); S174(4.1); S176(4.4); S178(31.5); T179(31.5); S181(31.5); S182(49.1); S183(49.1); S188(83.1); S190(10.3); T192(1.5)]	0.64	-0.64385619	0.91490394
Q9UKI8	1xPhospho [S147(0); Y149(0); Y152(0); S158(0); S159(100)]	0.867	-0.205896101	0.980895643
Q9UKJ3	2xPhospho [T1061(0); S1065(5.7); S1068(83.6); T1072(88.6); S1076(11.1); S1081(11.1)]	7.985	2.997292408	0.001168951
Q9UKJ3	2xPhospho [S1009(100); S1014(100)]	1.803	0.850399397	0.678654866
Q9UKJ3	2xPhospho [S738(100); S740(100)]	1.743	0.801572569	0.679191074
Q9UKJ3	1xPhospho [S1033(0); S1035(100); Y1038(0)]	1.753	0.809825996	0.688026385
Q9UKJ3	1xPhospho [S1107(100); S1109(0); T1115(0)]	1.223	0.290424404	0.922868578
Q9UKJ3	2xPhospho [T1061(0); S1065(0); S1068(4.7); T1072(95.1); S1076(0.2); S1081(100); S1087(0); T1089(0)]	1.181	0.240008965	0.92458333
Q9UKJ3	2xPhospho [Y1031(0); S1033(100); S1035(100); Y1038(0)]	0.928	-0.10780329	0.990299313
Q9UKL0	1xPhospho [S257(100)]	1.905	0.929790998	0.469342919
Q9UKL3	1xPhospho [S1270(0); S1271(0); T1277(0); S1278(100); T1282(0)]	0.453	-1.142417045	0.541490538
Q9UKL3	1xPhospho [S875(100); Y882(0); S885(0)]	0.714	-0.486004021	0.900153261
Q9UKM9	3xPhospho [T275(0); T286(100); S288(100); S295(0.3); T298(99.7)]	0.475	-1.074000581	0.186380714
Q9UKM9	1xPhospho [T275(0); T286(0); S288(0); S295(0); T298(100)]	1.382	0.466757616	0.855534442
Q9UKM9	2xPhospho [T286(94.4); S288(5.6); S295(5.6); T298(94.4)]	0.692	-0.531156057	0.891355291
Q9UKM9	2xPhospho [T275(0); T286(0); S288(100); S295(0.2); T298(99.8)]	0.802	-0.318325858	0.898100811
Q9UKM9	3xPhospho [T286(100); S288(8.1); S295(92.6); T298(99.4)]	0.603	-0.729770093	0.921519407
Q9UKM9	1xPhospho [S135(100)]	1.115	0.15704371	0.970341003
Q9UKS6	1xPhospho [S319(100); T324(0); T326(0); S327(0); T331(0)]	2.654	1.408168371	0.251041584
Q9UKS6	2xPhospho [T335(0); S341(100); S344(0); T347(0); S354(100); S358(0)]	0.568	-0.816037165	0.794223792
Q9UKS6	1xPhospho [T335(0); S341(0); S344(0); T347(0); S354(100); S358(0)]	1.422	0.507921465	0.794228341
Q9UKS6	2xPhospho [S319(0); T324(100); T326(0); S327(100); T331(0)]	1.188	0.248534836	0.93019184
Q9UKS6	2xPhospho [T335(0); S341(100); S344(0); T347(0); S354(100); S358(0)]	0.841	-0.249822294	0.967050102
Q9UKV3	2xPhospho [S467(0); T470(0); S478(100); S481(0); S482(0); S484(48.7); T486(48.7); S490(2.5); S499(0)]	4.809	2.265736926	0.006875806
Q9UKV3	1xPhospho [S561(98.7); T563(1.4); S567(0)]	2.805	1.488000771	0.091295447
Q9UKV3	1xPhospho [T999(0); S1004(100)]	1.457	0.543000877	0.762029106
Q9UKV3	2xPhospho [S873(0); S895(92.5); S898(92.5); T902(15)]	0.501	-0.997117491	0.769621572
Q9UKV3	1xPhospho [S825(100); S828(0); T830(0)]	1.47	0.555816155	0.776177942
Q9UKV3	2xPhospho [S240(100); S243(100); T254(0); S256(0)]	1.477	0.562669826	0.792401604
Q9UKV3	1xPhospho [S987(100); S990(0); T992(0)]	1.465	0.550900665	0.827205803
Q9UKV3	2xPhospho [S655(0.2); S657(99.8); S661(0.1); S664(49); S665(49); T666(1.9); S667(0.1); Y668(0); T669(0); T671(0)]	1.374	0.458382004	0.831443663
Q9UKV3	1xPhospho [S863(0); S865(0); T867(0); T868(0); S870(100)]	1.344	0.426533138	0.862863235
Q9UKV3	1xPhospho [S873(0); S895(99.8); S898(0.3); T902(0)]	1.254	0.326537348	0.879063032
Q9UKV3	2xPhospho [S467(0); T470(0); S478(100); S481(0); S482(0); S484(0); T486(3.4); S490(96.6)]	0.704	-0.506352666	0.892604794
Q9UKV3	3xPhospho [S467(0); T470(0); S478(100); S481(4.2); S482(95.8); S484(0); T486(0); S490(100)]	0.76	-0.395928676	0.903986081
Q9UKV3	1xPhospho [S561(100); T563(0); S567(0)]	1.224	0.291603558	0.907368665
Q9UKV3	1xPhospho [S490(100); S499(0)]	1.191	0.252173413	0.920957334
Q9UKV3	1xPhospho [S400(0); T408(0); S410(100); T414(0); T420(0)]	0.628	-0.671163536	0.937563451
Q9UKV3	2xPhospho [S166(100); S169(100); S181(0); S189(0); S190(0); S199(0)]	1.2	0.263034406	0.938029982
Q9UKV3	1xPhospho [S240(100); S243(0); T254(0); S256(0)]	1.141	0.190298792	0.94797789
Q9UKV3	2xPhospho [S467(0); T470(0); S478(100); S481(0.2); S482(95.6); S484(4.2); T486(0)]	1.097	0.133563526	0.960178862
Q9UKV3	1xPhospho [S115(0); T116(0); S125(100); S132(0); S135(0)]	1.141	0.190298792	0.962617576

Q9UKV3	2xPhospho [S400(0); T408(0); S410(100); T414(96.4); T420(3.6)]	0.858	-0.220950447	0.973942845
Q9UKV3	2xPhospho [S400(100); T408(0); S410(100); T414(0); T420(0)]	0.826	-0.275786313	0.982345843
Q9UKV3	3xPhospho [S467(0); T470(0); S478(100); S481(99.7); S482(94.9); S484(5.1); T486(0.3)]	0.915	-0.128156351	0.99811985
Q9UKV3	1xPhospho [S467(0); T470(0); S478(100); S481(0); S482(0); S484(0); T486(0)]	0.886	-0.174621396	0.999144205
Q9UKV3	3xPhospho [S467(0); T470(0); S478(100); S481(5.8); S482(94.2); S484(48.5); T486(48.5); S490(3); S499(0)]	0.939	-0.090802937	1
Q9UKV5	1xPhospho [S507(0); S509(0); S516(0); S522(2.8); S523(97.2); T530(0); S531(0); T534(0)]	1.576	0.656267535	0.749797187
Q9UKV5	2xPhospho [S507(50); S509(50); S516(0); S522(0.2); S523(99.8); T530(0); S531(0); T534(0)]	1.062	0.086783766	0.973763863
Q9UKX7	1xPhospho [Y193(0); S204(0.1); S208(0.1); S210(99.8); S212(0)]	0.275	-1.862496476	0.282584514
Q9UKY1	2xPhospho [S45(0); S47(100); S48(100); S55(0); S58(0)]	8.394	3.069358463	0.000164418
Q9UKY1	2xPhospho [S45(0); S47(100); S48(100); S55(0); S58(0)]	0.571	-0.808437349	0.751633178
Q9UKY7	1xPhospho [S106(1.9); S107(98.1)]	2.591	1.373509016	0.299786659
Q9UKY7	1xPhospho [S216(100)]	1.643	0.71633248	0.697129868
Q9UKY7	1xPhospho [T182(0); Y190(100); S191(0); T193(0); S197(0); S200(0); T201(0)]	1.375	0.459431619	0.865762755
Q9UKY7	1xPhospho [S216(100)]	1.234	0.303342394	0.904774194
Q9ULD2	2xPhospho [S1245(92.1); T1247(3.9); S1248(3.9); S1249(0.2); S1255(100)]	2.329	1.21971064	0.284236251
Q9ULD2	2xPhospho [S1241(9.4); S1245(9.4); T1247(2.7); S1248(89.3); S1249(89.3); S1255(0)]	1.317	0.397255346	0.851160627
Q9ULH0	3xPhospho [S1590(5.5); S1591(5.5); S1593(89.6); S1594(99.3); S1598(100); S1607(0)]	0.01	-6.64385619	8.35E-17
Q9ULH0	2xPhospho [S1410(50); S1411(50); S1414(0); T1415(0); Y1416(0); Y1417(0); S1421(99.2); S1422(0.4); S1423(0.4); S1426(0); S1429(0)]	0.421	-1.248107862	0.430840855
Q9ULH0	1xPhospho [S1555(100); S1559(0)]	0.567	-0.81857936	0.854283637
Q9ULH1	2xPhospho [T837(0.1); S839(99.9); S843(100)]	0.35	-1.514573173	0.440397264
Q9ULH1	2xPhospho [T837(2.5); S839(97.5); S843(100)]	0.373	-1.422752464	0.544531713
Q9ULH1	2xPhospho [S733(0); S738(100); S742(0); S743(0.1); S744(99.9); S746(0)]	0.502	-0.994240731	0.58296191
Q9ULH1	2xPhospho [S717(100); S726(100)]	0.424	-1.23786383	0.647620981
Q9ULH7	1xPhospho [T536(0); S541(0); S543(100); T545(0); S546(0); S547(0); T548(0); S550(0)]	100	6.64385619	8.35E-17
Q9ULH7	1xPhospho [S834(0); T841(0); S846(95.1); S847(4.9); S859(0); S863(0)]	1.24	0.310340121	0.934211842
Q9ULH7	2xPhospho [S834(0); T841(0); S846(50); S847(50); S859(0); S863(100)]	0.795	-0.330973234	0.973263372
Q9ULI0	3xPhospho [S523(0.9); S524(99.5); S527(99.5); T528(100)]	0.797	-0.327348371	0.889077103
Q9ULJ3	2xPhospho [T431(0); S434(0); S435(100); S438(100); S441(0)]	0.01	-6.64385619	8.35E-17
Q9ULJ3	2xPhospho [S409(0.1); S411(99.9); S413(0); S415(0); T416(0); S422(100); T425(0)]	0.456	-1.13289427	0.594487868
Q9ULJ3	2xPhospho [S972(0); T979(0); S981(100); S983(100)]	0.646	-0.63039393	0.806224461
Q9ULJ7	2xPhospho [T1158(0); S1162(50); S1163(50); S1165(0.1); S1167(100); S1170(0); T1171(0)]	1.273	0.348232419	0.884551994
Q9ULL5	3 1xPhospho [T636(0); S651(100)]	100	6.64385619	8.35E-17
Q9ULL5	3 2xPhospho [T1376(0); S1379(0); S1381(100); S1382(100); S1387(0)]	0.629	-0.668868078	0.786602611
Q9ULL5	3 2xPhospho [T1554(0); T1561(100); S1568(100)]	0.987	-0.01887801	0.989898705
Q9ULT8	3xPhospho [T2299(0.2); S2301(2.6); S2303(0.2); S2306(48.5); T2307(48.5); S2313(94.8); S2315(5.2); S2318(100); S2323(0)]	100	6.64385619	8.35E-17
Q9ULT8	1xPhospho [S625(0); T626(0); S631(3); S632(97); S640(0)]	1.472	0.557777671	0.698520686
Q9ULT8	1xPhospho [S1517(0); S1519(0); S1520(0); S1529(0); S1530(0); S1531(0); S1533(100); S1538(0); S1539(0)]	1.702	0.767231037	0.701261726
Q9ULT8	2xPhospho [T2299(0); S2301(0); S2303(0); S2306(0); T2307(0); S2313(4.6); S2315(95.4); S2318(100); S2323(0)]	1.466	0.551885103	0.757757103
Q9ULW0	1xPhospho [S728(0); S729(0); T734(0); S738(100)]	0.732	-0.450084446	0.943939997
Q9ULX3	1xPhospho [S201(100); T210(0); S212(0)]	1.246	0.317304068	0.907632224
Q9ULX3	1xPhospho [S184(100)]	1.249	0.320773477	0.926077656
Q9ULX3	1xPhospho [S184(100)]	1.166	0.221567789	0.959719138
Q9UM54	1 2xPhospho [T1234(100); T1237(100)]	0.01	-6.64385619	8.35E-17
Q9UMD9	2xPhospho [S85(94.1); S88(5.5); T89(0.4); S93(99.3); S96(0.4); T97(0.4)]	0.713	-0.488026018	0.973599548
Q9UMX1	2xPhospho [S342(100); S346(100); S349(0); S351(0); S352(0); T353(0)]	0.64	-0.64385619	0.796170626
Q9UN86	2 1xPhospho [S253(100)]	1.707	0.771463058	0.643396625
Q9UN86	2 2xPhospho [Y133(0); S141(100); S149(100)]	0.694	-0.526992432	0.745044398
Q9UN86	2 2xPhospho [S225(0); T226(0); T227(100); S235(100)]	1.413	0.498761466	0.822928057
Q9UN86	2 2xPhospho [S225(8.4); T226(95.8); T227(95.8); S235(0)]	1.367	0.451013243	0.836554173
Q9UN86	2 1xPhospho [S225(0); T226(0); T227(99.9); S235(0)]	1.078	0.108357178	0.980895643
Q9UNF0	2 1xPhospho [T350(0); S352(0.2); Y353(0); T355(0.2); S358(99.5); S362(0); S367(0); S368(0); T369(0); S375(0); T383(0); S384(0); T386(0)]	0.01	-6.64385619	8.35E-17
Q9UNF0	2 2xPhospho [T321(0); T325(0); T327(0); T332(0); S336(0); S339(0.3); S342(94.4); S343(5.1); S345(0.6); S346(99.7); Y347(0)]	0.765	-0.386468347	0.962617576
Q9UNI6	1xPhospho [S335(100); T337(0)]	1.073	0.101650076	0.962617576
Q9UNS1	1xPhospho [S1173(100)]	2.646	1.403813062	0.096137571
Q9UNS1	1xPhospho [S1173(100)]	1.579	0.659011171	0.749797187
Q9UNX4	1xPhospho [S240(3.3); S241(96.8); T247(0); T257(0)]	1.134	0.18142064	0.943512809
Q9UNY4	1xPhospho [T259(0); S268(0); S271(2.8); S273(97.2)]	0.48	-1.058893689	0.647620981
Q9UPN4	2 2xPhospho [S114(100); S116(0); T117(0); S120(100)]	13.351	3.7388759	0.00056229
Q9UPN4	2 1xPhospho [S114(99.9); S116(0.1); T117(0); S120(0)]	2.308	1.206643224	0.608826126
Q9UPN4	2 1xPhospho [S87(98); S89(2); T93(0); T96(0); S105(0); S107(0)]	1.361	0.444667067	0.855545836
Q9UPN4	2 2xPhospho [S87(0.1); S89(99.8); T93(0.1); T96(0); S105(97.1); S107(2.9)]	0.823	-0.281035664	0.957451342
Q9UPN4	2 2xPhospho [S87(2.3); S89(97.7); T93(0); T96(0); S105(99.9); S107(0.1)]	0.887	-0.17299399	0.972363997
Q9UPN4	2 2xPhospho [T428(0); S447(100); S450(100)]	1.029	0.041242982	0.974924677
Q9UPN4	2 1xPhospho [S381(100); T383(0); T387(0)]	1.023	0.032806145	0.977858961
Q9UPN4	2 1xPhospho [S35(100); T38(0); T39(0)]	0.996	-0.005782353	0.980895643
Q9UPN7	1xPhospho [T524(0); S529(0.2); S530(0.2); S531(99.7)]	100	6.64385619	8.35E-17
Q9UPN7	2xPhospho [S635(100); S638(100); S648(0)]	3.184	1.670840336	0.07457184

Q9UPN7	3xPhospho [T524(0); S529(100); S530(100); S531(100)]	1.387	0.471967788	0.863668848
Q9UPN7	3xPhospho [T524(0); S529(100); S530(100); S531(100)]	0.758	-0.399730246	0.91490394
Q9UPT5	1xPhospho [S241(0); S242(0); S243(0); S244(0); S245(0); Y249(0); S250(100)]	8.645	3.111865964	0.000375476
Q9UPT8	3xPhospho [S1104(99.7); T1106(5.4); S1108(94.9); S1110(0); S1114(100); T1118(0); Y1121(0)]	0.602	-0.732164608	0.742559804
Q9UPT8	2xPhospho [S1104(96.4); T1106(3.7); S1108(0); S1110(0); S1114(100); T1118(0); Y1121(0)]	0.861	-0.215914857	0.944443377
Q9UPT8	2xPhospho [T898(1.1); S899(1.1); S904(90.7); S907(7.5); S908(6.7); S913(6.2); S914(6.2); S915(80.3)]	1.103	0.141432791	0.969680228
Q9UPT8	1xPhospho [T898(0); S899(0); S904(0); S907(0.2); S908(0.2); S913(3.6); S914(3.6); S915(92.5)]	0.98	-0.029146346	0.986075329
Q9UPZ9	2xPhospho [S152(0); Y156(3); T157(96.9); Y159(1.6); S161(49.3); T162(49.3)]	1.821	0.864730922	0.658708958
Q9UQ35	1xPhospho [S988(0.2); S990(3.7); S992(96.1); S994(0); Y996(0)]	100	6.64385619	8.35E-17
Q9UQ35	1xPhospho [S854(0); T856(0); S857(0); S864(0); T866(100)]	100	6.64385619	8.35E-17
Q9UQ35	2xPhospho [T2144(96.6); S2147(3.4); S2155(100)]	100	6.64385619	8.35E-17
Q9UQ35	1xPhospho [S142(0); S144(0); Y145(0); S149(99.9); S150(0.1)]	100	6.64385619	8.35E-17
Q9UQ35	1xPhospho [T286(0); T288(0); T289(0); S295(95.8); S297(4); S300(0.2)]	100	6.64385619	8.35E-17
Q9UQ35	1xPhospho [S1101(0); S1102(0); S1103(100); T1106(0); S1110(0)]	100	6.64385619	8.35E-17
Q9UQ35	2xPhospho [T2302(0); S2310(100); T2312(0); S2314(0); T2316(100); T2319(0); Y2323(0); S2325(0); S2326(0); S2327(0)]	3.349	1.743730376	0.04474786
Q9UQ35	2xPhospho [S1854(100); T1856(0); S1857(100)]	2.74	1.454175893	0.058612135
Q9UQ35	2xPhospho [S534(100); S536(100); S543(0)]	2.702	1.434027675	0.065572346
Q9UQ35	1xPhospho [T1492(100)]	3.152	1.656267535	0.121484696
Q9UQ35	1xPhospho [T2144(0); S2147(0); S2155(100)]	2.263	1.178236585	0.129561724
Q9UQ35	3xPhospho [T1003(100); S1008(0); S1010(50); S1012(50); S1014(99.9)]	2.577	1.365692537	0.140932628
Q9UQ35	1xPhospho [T2289(100); T2291(0)]	2.532	1.340277405	0.223367403
Q9UQ35	1xPhospho [T2329(0); S2335(100)]	2.12	1.084064265	0.293524248
Q9UQ35	2xPhospho [T286(0); T288(0); T289(0); S295(100); S297(100); S300(0)]	1.937	0.953823954	0.305105183
Q9UQ35	2xPhospho [S2449(100); S2453(100); S2456(0); S2459(0)]	2.166	1.115033243	0.32420743
Q9UQ35	1xPhospho [T1205(0); T1208(100)]	2.32	1.214124805	0.325359091
Q9UQ35	1xPhospho [S2343(100); T2347(0); S2355(0)]	2.12	1.084064265	0.328136817
Q9UQ35	1xPhospho [S2449(0); S2453(100); S2456(0); S2459(0)]	1.933	0.950841638	0.372967623
Q9UQ35	1xPhospho [T1003(100); S1008(0); S1010(0); S1012(0)]	2.221	1.151209393	0.380290732
Q9UQ35	1xPhospho [S871(0); S875(0); S876(100)]	1.924	0.944108799	0.380617646
Q9UQ35	1xPhospho [T2397(0); S2398(100)]	1.858	0.893750502	0.405790408
Q9UQ35	1xPhospho [T2302(0); S2310(0); T2312(0); S2314(0); T2316(2.6); T2319(97.4); Y2323(0); S2325(0); S2326(0); S2327(0)]	2.063	1.044743821	0.422793334
Q9UQ35	1xPhospho [T2357(0); S2365(1.2); T2367(0); S2368(98.8)]	1.97	0.97819563	0.441144795
Q9UQ35	1xPhospho [T2261(0); S2272(100)]	1.904	0.929033479	0.47518367
Q9UQ35	2xPhospho [S534(100); S536(97.7); S543(2.3)]	1.727	0.788268083	0.485808589
Q9UQ35	1xPhospho [S1122(0); S1124(100)]	1.674	0.743299528	0.506073142
Q9UQ35	1xPhospho [S1188(0); S1198(2); S1199(98)]	1.794	0.84317989	0.513166947
Q9UQ35	1xPhospho [S311(0); T315(0); T316(0); S317(0); T318(0); S322(0.1); S323(99.9); T326(0.1); T328(0)]	1.687	0.754459974	0.52651047
Q9UQ35	2xPhospho [S1397(0); S1398(0); S1401(0); S1403(0); S1404(100); T1413(50); S1415(50)]	1.789	0.839153387	0.546019581
Q9UQ35	3xPhospho [S1397(0); S1398(0); S1401(95.6); S1403(8.8); S1404(95.6); T1413(99.8); S1415(0.2)]	1.799	0.847195187	0.561332121
Q9UQ35	2xPhospho [S1101(0); S1102(100); S1103(100); T1106(0); S1110(0)]	1.558	0.639695233	0.607772032
Q9UQ35	1xPhospho [S2684(0); S2688(100)]	1.746	0.804053559	0.62233267
Q9UQ35	2xPhospho [S1188(100); S1198(97.3); S1199(2.7)]	1.536	0.619178216	0.644171766
Q9UQ35	3xPhospho [S854(66.7); T856(66.7); S857(66.7); S864(4.5); T866(95.5)]	1.621	0.696884091	0.674611332
Q9UQ35	2xPhospho [T2357(0); S2365(2.2); T2367(97.9); S2368(100)]	1.654	0.725959235	0.678654866
Q9UQ35	2xPhospho [S1188(0); S1198(100); S1199(100)]	1.704	0.768925336	0.688350005
Q9UQ35	1xPhospho [S2376(0); T2381(0); S2382(100)]	1.561	0.642470537	0.704203351
Q9UQ35	2xPhospho [S2690(0); S2692(100); Y2693(0); S2694(100)]	1.492	0.577247536	0.722079238
Q9UQ35	1xPhospho [T1177(0); S1179(100)]	1.532	0.615416297	0.7222669
Q9UQ35	2xPhospho [S854(0); T856(0); S857(100); S864(0); T866(100)]	1.48	0.565597176	0.723217176
Q9UQ35	2xPhospho [S854(0); T856(0); S857(100); S864(0.1); T866(99.9)]	1.523	0.606915942	0.724397901
Q9UQ35	3xPhospho [T286(0); T288(0); T289(0.2); S295(99.8); S297(100); S300(100)]	1.735	0.794935663	0.724397901
Q9UQ35	2xPhospho [S1537(0); S1539(100); S1541(98.1); S1542(1.9); S1550(0); S1552(0)]	1.519	0.60312187	0.725295205
Q9UQ35	1xPhospho [S311(0); T315(0); T316(0); S317(0); T318(0); S322(2.7); S323(97.3); T326(0.1); T328(0)]	1.463	0.548929769	0.740489326
Q9UQ35	1xPhospho [T2104(100); S2111(0); S2112(0)]	1.649	0.721591399	0.741474639
Q9UQ35	2xPhospho [T286(0); T288(0); T289(0); S295(100); S297(100); S300(0)]	1.554	0.635986504	0.746167942
Q9UQ35	1xPhospho [T2144(0); S2147(0); S2155(100)]	1.669	0.738983955	0.749797187
Q9UQ35	2xPhospho [S2044(100); S2046(100)]	1.44	0.526068812	0.755716034
Q9UQ35	2xPhospho [S1041(0.1); S1042(0.1); T1043(99.8); S1048(0); Y1049(0); S1053(0.1); S1054(99.9)]	1.508	0.592636429	0.758158764
Q9UQ35	3xPhospho [S846(50.1); T848(50.1); S854(99.7); T856(0.2); S857(0); S864(0); T866(100)]	1.509	0.593592806	0.75891781
Q9UQ35	3xPhospho [S346(0); T348(0); S351(100); S353(100); S357(96.5); S358(3.5); T359(0); T367(0)]	1.413	0.498761466	0.780165563
Q9UQ35	2xPhospho [S2115(0); S2118(0); S2121(100); S2123(100); T2125(0)]	1.405	0.49057013	0.786760931
Q9UQ35	1xPhospho [S2132(100); S2141(0); S2142(0)]	1.438	0.524063676	0.797966154
Q9UQ35	2xPhospho [S2032(100); T2034(100)]	1.383	0.467801156	0.803718009
Q9UQ35	1xPhospho [S2436(0); S2438(0); S2449(100); S2453(0); S2456(0); S2459(0)]	1.336	0.417920008	0.816750637
Q9UQ35	1xPhospho [S1188(0); S1198(98); S1199(2)]	1.37	0.454175893	0.819921743
Q9UQ35	3xPhospho [S1099(100); S1101(100); S1102(0); S1103(100); T1106(0); S1110(0)]	1.362	0.445726703	0.823142957
Q9UQ35	2xPhospho [S2115(0); S2118(0); S2121(100); S2123(100); T2125(0)]	0.673	-0.57132159	0.828441094
Q9UQ35	2xPhospho [T1003(100); S1008(0); S1010(0); S1012(0); S1014(100)]	1.346	0.42867841	0.84145238
Q9UQ35	1xPhospho [T1365(0); S1368(100)]	1.399	0.484395963	0.851597639

Q9UQ35	2xPhospho [S1378(0); S1379(0); S1382(100); S1383(0); S1384(0); S1387(100)]	1.33	0.411426246	0.854283637
Q9UQ35	1xPhospho [S2690(100); S2692(0); Y2693(0); S2694(0)]	1.396	0.481298942	0.854283637
Q9UQ35	1xPhospho [T1003(0); S1008(0); S1010(0); S1012(0); S1014(100)]	1.287	0.364012054	0.855185501
Q9UQ35	1xPhospho [S1539(1.2); S1541(98.8); S1542(0); S1550(0); S1552(0)]	1.35	0.432959407	0.862088145
Q9UQ35	2xPhospho [S311(0); T315(0); T316(0); S317(0); T318(0.1); S322(99.8); S323(96.7); T326(3.4); T328(0)]	1.316	0.396159489	0.86239429
Q9UQ35	1xPhospho [S1041(0); S1042(0); T1043(0); S1048(0); Y1049(0); S1053(99.9); S1054(0.1)]	1.346	0.42867841	0.868254505
Q9UQ35	3xPhospho [S2100(100); S2102(100); T2104(100); S2111(0); S2112(0)]	1.301	0.379620962	0.872730156
Q9UQ35	2xPhospho [S311(0); T315(0); T316(0); S317(0); T318(0); S322(100); S323(100); T326(0); T328(0)]	1.279	0.355016264	0.882116304
Q9UQ35	2xPhospho [S1101(0); S1102(0); S1103(100); T1106(0); S1110(0); S1112(100)]	1.268	0.342554745	0.890736743
Q9UQ35	1xPhospho [S2032(100); T2034(0)]	1.278	0.353887836	0.893481203
Q9UQ35	1xPhospho [S1397(0); S1398(0); S1401(0); S1403(0); S1404(100)]	1.256	0.328836464	0.904774194
Q9UQ35	3xPhospho [T983(100); S988(93); S990(7); S992(7); S994(93); Y996(0)]	1.21	0.275007047	0.93019184
Q9UQ35	2xPhospho [T983(100); S988(0); S990(3.2); S992(3.2); S994(93.6); Y996(0)]	1.158	0.211635253	0.930376591
Q9UQ35	2xPhospho [T1003(100); S1008(0); S1010(0); S1012(0); S1014(100)]	1.205	0.269033146	0.931904324
Q9UQ35	2xPhospho [S846(50); T848(50); S854(100); T856(0); S857(0); S864(0); T866(0)]	1.185	0.244887059	0.933452347
Q9UQ35	3xPhospho [S2067(100); T2069(100); S2071(100)]	1.188	0.248534836	0.939608401
Q9UQ35	2xPhospho [S2067(1.9); T2069(98.1); S2071(100)]	1.195	0.257010618	0.943796521
Q9UQ35	1xPhospho [S1318(2.2); S1320(97.8); S1326(0); S1329(0)]	1.129	0.175045486	0.943939997
Q9UQ35	3xPhospho [S1101(50); S1102(50); S1103(100); T1106(0); S1110(2.4); S1112(97.6)]	1.175	0.232660757	0.94432975
Q9UQ35	3xPhospho [S1101(98); S1102(2); S1103(100); T1106(0); S1110(0); S1112(100)]	1.117	0.159629186	0.94797789
Q9UQ35	3xPhospho [S2042(100); S2044(100); S2046(100)]	1.174	0.231432408	0.94797789
Q9UQ35	3xPhospho [S2115(100); S2118(0); S2121(100); S2123(100); T2125(0)]	0.791	-0.3382504	0.952591019
Q9UQ35	3xPhospho [S1397(0.2); S1398(99.8); S1401(99.8); S1403(4.5); S1404(95.7)]	1.116	0.158337027	0.954539739
Q9UQ35	2xPhospho [S1122(100); S1124(100)]	1.115	0.15704371	0.955790722
Q9UQ35	3xPhospho [S846(6.2); T848(6.2); S854(62.5); T856(62.5); S857(62.5); S864(0); T866(100)]	1.181	0.240008965	0.957850451
Q9UQ35	1xPhospho [S1378(0); S1379(0); S1382(100); S1383(0); S1384(0); S1387(0)]	1.134	0.18142064	0.960298686
Q9UQ35	3xPhospho [S2030(100); S2032(100); T2034(100)]	1.151	0.202887833	0.9616322
Q9UQ35	1xPhospho [S2436(100); S2438(0)]	0.815	-0.295128036	0.962617576
Q9UQ35	2xPhospho [S377(0); T383(0); T384(0); S387(100); S395(0); S398(97); T400(3)]	1.133	0.180147861	0.965013037
Q9UQ35	2xPhospho [S1397(0); S1398(0); S1401(100); S1403(0); S1404(100)]	0.897	-0.15682011	0.965219233
Q9UQ35	3xPhospho [S2115(100); S2118(0); S2121(100); S2123(100); T2125(0)]	0.757	-0.401634795	0.971220368
Q9UQ35	2xPhospho [S2115(0); S2118(0); S2121(100); S2123(100); T2125(0)]	0.886	-0.174621396	0.971220368
Q9UQ35	2xPhospho [S2030(2.4); S2032(97.6); T2034(100)]	1.058	0.081339627	0.971220368
Q9UQ35	2xPhospho [S1539(49.6); S1541(49.6); S1542(0.8); S1550(0); S1552(100)]	1.051	0.071762669	0.973263372
Q9UQ35	2xPhospho [S2132(100); S2141(97.5); S2142(2.5)]	0.999	-0.001443417	0.973763863
Q9UQ35	3xPhospho [S2690(100); S2692(100); Y2693(0); S2694(100)]	0.854	-0.227692025	0.975403456
Q9UQ35	2xPhospho [S2436(100); S2438(0); S2449(100); S2453(0); S2456(0); S2459(0)]	1.039	0.055195654	0.976825037
Q9UQ35	3xPhospho [S1099(5.3); S1101(5.3); S1102(90.1); S1103(99.4); T1106(0); S1110(50); S1112(50)]	0.767	-0.382701517	0.979498769
Q9UQ35	2xPhospho [S1140(0); S1142(0); S1143(0); S1144(0); Y1145(0); T1147(0); S1150(96.3); S1152(3.7); S1157(3.7); T1161(96.3); S1164(0)]	0.791	-0.3382504	0.979498769
Q9UQ35	2xPhospho [S1099(50); S1101(50); S1102(0); S1103(100); T1106(0); S1110(0)]	0.875	-0.192645078	0.980895643
Q9UQ35	1xPhospho [S1336(100); T1341(0); T1345(0); S1348(0); S1349(0)]	0.88	-0.184424571	0.980895643
Q9UQ35	3xPhospho [S1397(0); S1398(0); S1401(0); S1403(50); S1404(50); T1413(100); S1415(100)]	0.932	-0.10159814	0.980895643
Q9UQ35	3xPhospho [S377(100); T383(0); T384(0); S387(100); S395(0); S398(96.6); T400(3.4)]	0.946	-0.080087911	0.980895643
Q9UQ35	2xPhospho [S1397(0); S1398(0); S1401(0); S1403(0); S1404(0); T1413(100); S1415(100)]	1.051	0.071762669	0.980895643
Q9UQ35	2xPhospho [S1101(0); S1102(0); S1103(100); T1106(0); S1110(50); S1112(50)]	1.054	0.075874867	0.980895643
Q9UQ35	2xPhospho [S1318(2.2); S1320(97.8); S1326(100); S1329(0)]	1.057	0.079975377	0.981858798
Q9UQ35	2xPhospho [S1326(100); S1329(100)]	0.965	-0.051399153	0.985318278
Q9UQ35	2xPhospho [S2436(0); S2438(0); S2449(100); S2453(100); S2456(0); S2459(0)]	0.999	-0.001443417	0.985318278
Q9UQ35	2xPhospho [S1397(0.2); S1398(3.7); S1401(3.7); S1403(92.4); S1404(100)]	0.803	-0.316528107	0.987937869
Q9UQ35	1xPhospho [S1140(0); S1142(0); S1143(0); S1144(0); Y1145(0); T1147(0); S1150(0); S1152(100); S1157(0)]	1.038	0.053806444	0.98830288
Q9UQ35	2xPhospho [S2100(100); S2102(0.1); T2104(100); S2111(0); S2112(0)]	1.035	0.049630768	0.991011738
Q9UQ35	2xPhospho [S2067(96.7); T2069(3.5); S2071(99.9)]	0.965	-0.051399153	0.99184412
Q9UQ35	3xPhospho [S311(99.6); T315(0.4); T316(0); S317(0); T318(0); S322(100); S323(100); T326(0); T328(0)]	1.016	0.022900402	0.99184412
Q9UQ35	3xPhospho [S2067(100); T2069(100); S2071(100)]	0.982	-0.02620507	0.992140636
Q9UQ35	3xPhospho [S1318(0); S1320(100); S1326(100); S1329(100)]	0.985	-0.02180437	0.993670231
Q9UQ35	2xPhospho [S871(0); S875(100); S876(100)]	0.983	-0.024736678	0.998086195
Q9UQ35	2xPhospho [S1140(100); S1142(0); S1143(0); S1144(0); Y1145(0); T1147(0); S1150(0); S1152(100); S1157(0)]	0.898	-0.15521265	0.999714477
Q9UQ35	1xPhospho [S1326(0); S1329(100)]	0.99	-0.01449957	1
Q9UQ35	1xPhospho [S2132(100); S2141(0); S2142(0)]	0.999	-0.001443417	1
Q9UQ80	1xMet-loss+Acetyl [N-Term]; 1xPhospho [S2(100); T11(0); T19(0)]	0.633	-0.659722595	0.616281851
Q9UQ88	1xPhospho [S265(0); S271(100); S273(0)]	100	6.64385619	8.35E-17
Q9UQ88	1xPhospho [S72(100); S82(0); S92(0)]	1.64	0.713695815	0.689396319
Q9UQ88	1xPhospho [S47(100)]	1.194	0.255802837	0.93019184
Q9UQ88	2xPhospho [T739(100); S740(100); Y750(0); S751(0)]	0.78	-0.358453971	0.944828278
Q9UQ88	2xPhospho [S217(0.2); S220(99.8); S222(100)]	0.757	-0.401634795	0.9616322
Q9UQ88	1xPhospho [T739(3); S740(97.1); Y750(0); S751(0)]	0.994	-0.008682243	1
Q9UQ88	2xPhospho [T739(100); S740(100); Y750(0); S751(0)]	1.014	0.020057652	1
Q9UQN3	1xPhospho [T197(0); S199(100)]	0.795	-0.330973234	0.982345843
Q9Y219	1xPhospho [S1208(100); S1217(0)]	1.432	0.518031493	0.922251444

Q9Y230	1xPhospho [Y446(100)]	100	6.64385619	8.35E-17
Q9Y232	1xPhospho [S164(0); S170(100)]	7.131	2.834104404	0.001367781
Q9Y232	1xPhospho [Y86(0.1); S88(99.9); T92(0); Y105(0)]	4.602	2.202260984	0.018492623
Q9Y248	1xPhospho [T172(0); S179(0); T180(0.1); S182(99.9)]	0.783	-0.352915787	0.97998947
Q9Y252	1xPhospho [S6(4); S8(4); S12(88.1); T15(4)]	9.23	3.206330648	0.003103138
Q9Y265	1xPhospho [T77(0); S89(100)]	1	0	0.978952227
Q9Y266	1xPhospho [S136(0); S139(100); T145(0)]	2.513	1.329410671	0.06471073
Q9Y266	2xPhospho [S136(0); S139(100); T145(100)]	2.155	1.107687869	0.192427132
Q9Y283	1xPhospho [S614(100); S617(0.1); S628(0); T629(0); S634(0)]	1.293	0.370722275	0.870654365
Q9Y2D8	2xPhospho [S536(100); S540(100); S542(0); T543(0); S544(0); T549(0)]	0.989	-0.015957574	0.979498769
Q9Y2F5	2xPhospho [S1692(100); S1697(0.1); S1699(99.9); S1701(0); S1704(0); S1706(0)]	0.899	-0.153606979	0.999144205
Q9Y2H0	1 1xPhospho [S729(0); T734(0); S739(0); S741(100)]	0.01	-6.64385619	8.35E-17
Q9Y2I9	2xPhospho [S114(100); S117(100); S118(0); T119(0); S122(0); S124(0)]	0.526	-0.926865295	0.578933372
Q9Y2J4	1xPhospho [S667(96); S670(4); T679(0); S686(0); S687(0); S688(0)]	1	0	0.973763863
Q9Y2K1	2xPhospho [T348(0); S355(100); T356(100); Y370(0)]	0.554	-0.852042119	0.641603458
Q9Y2K6	2xPhospho [S132(100); S134(100)]	0.52	-0.943416472	0.429315665
Q9Y2K6	2xPhospho [S371(0); S372(0); S373(100); T377(100)]	0.733	-0.448114897	0.872729954
Q9Y2L9	2xPhospho [S532(100); S536(100); T538(0); T539(0); S541(0); T542(0)]	0.616	-0.698997744	0.655156419
Q9Y2R4	1xPhospho [S54(0); S62(99.9); T64(0.1)]	0.723	-0.467932448	0.938029982
Q9Y2R4	1xPhospho [S565(99.8); S567(0.1); T568(0.1); T569(0)]	0.9	-0.152003093	0.989898705
Q9Y2T1	3xPhospho [T449(100); S454(100); Y460(0); S461(100)]	100	6.64385619	8.35E-17
Q9Y2U5	1xPhospho [S163(98.5); S164(1.5); Y169(0)]	0.522	-0.937878288	0.563180779
Q9Y2U8	2xPhospho [S140(0); S141(100); S144(100); S149(0)]	0.492	-1.023269779	0.66046943
Q9Y2U8	3xPhospho [S140(100); S141(100); S144(100); S149(0)]	0.535	-0.902389203	0.746167942
Q9Y2V2	1xPhospho [T50(0); S52(100); T54(0)]	1.561	0.642470537	0.70588502
Q9Y2V2	3xPhospho [S30(100); S32(100); S41(3.6); T45(96.4)]	0.701	-0.512513651	0.755041636
Q9Y2V2	2xPhospho [S30(100); S32(100); S41(0); T45(0)]	0.876	-0.190997225	0.96955242
Q9Y2V2	1xPhospho [S41(100); T45(0)]	1.088	0.121678557	0.977573898
Q9Y2W1	1xPhospho [S622(95.6); S624(4.4); T633(0)]	100	6.64385619	8.35E-17
Q9Y2W1	3xPhospho [S315(100); S320(100); S323(0); T324(0.1); S326(97.6); T327(2.4); Y328(0); S330(0); S331(0)]	4.649	2.216920426	0.001315898
Q9Y2W1	2xPhospho [S237(0); S240(100); S243(100)]	4.05	2.017921908	0.01891206
Q9Y2W1	2xPhospho [S315(100); S320(100); S323(0); T324(0); S326(0); T327(0); Y328(0); S330(0); S331(0)]	3.286	1.71633248	0.049571805
Q9Y2W1	1xPhospho [S217(0); S219(0); S220(0); T227(0); Y228(0); T230(0); S232(1.7); S234(98.3)]	2.463	1.300416628	0.363377543
Q9Y2W1	1xPhospho [S253(0); S257(100)]	2.431	1.281549893	0.391961858
Q9Y2W1	1xPhospho [S928(0); S939(100); T941(0)]	2.024	1.01720929	0.425201113
Q9Y2W1	3xPhospho [S248(100); S253(100); S257(100)]	1.851	0.888304895	0.479998895
Q9Y2W1	1xPhospho [S698(100)]	2.224	1.153156788	0.579751615
Q9Y2W1	3xPhospho [S315(100); S320(99.7); S323(50); T324(50); S326(0); T327(0.3); Y328(0); S330(0); S331(0); S336(0); S339(0); Y344(0); T345(0)]	0.565	-0.823677227	0.614155309
Q9Y2W1	1xPhospho [S315(0); S320(100); S323(0); T324(0); S326(0); T327(0); Y328(0); S330(0); S331(0)]	1.748	0.805705185	0.656131188
Q9Y2W1	1xPhospho [S928(0); S939(3.4); T941(96.6); T953(0); T954(0)]	1.585	0.66448284	0.688108927
Q9Y2W1	2xPhospho [S315(100); S320(100); S323(0); T324(0); S326(0); T327(0); Y328(0); S330(0); S331(0)]	1.49	0.575312331	0.714293599
Q9Y2W1	2xPhospho [S928(100); S939(99.9); T941(0.1)]	1.479	0.564622052	0.724165145
Q9Y2W1	2xPhospho [T397(0); S399(0); S406(100); S408(100)]	1.716	0.779049553	0.73347811
Q9Y2W1	3xPhospho [S390(0); S392(0); T397(99.8); S399(0.2); S406(100); S408(100)]	0.601	-0.734563104	0.737789345
Q9Y2W1	1xPhospho [S928(100); S939(0); T941(0)]	1.554	0.635986504	0.750848736
Q9Y2W1	1xPhospho [S928(0); S939(100); T941(0)]	1.413	0.498761466	0.822606712
Q9Y2W1	2xPhospho [S928(100); S939(100); T941(0)]	1.357	0.440420721	0.828459192
Q9Y2W1	1xPhospho [S698(100)]	1.568	0.648925559	0.848293321
Q9Y2W1	1xPhospho [S237(0); S240(0); S243(100)]	1.318	0.39835037	0.879063032
Q9Y2W1	1xPhospho [S377(0); S379(100); T381(0)]	1.251	0.32308179	0.91490394
Q9Y2W1	2xPhospho [S320(0); S323(0); T324(0.2); S326(99.7); T327(0.2); Y328(0); S330(96.2); S331(3.8); S336(0); S339(0); Y344(0); T345(0)]	0.775	-0.367731785	0.93019184
Q9Y2W1	2xPhospho [S217(0); S219(0); S220(0); T227(0); Y228(0); T230(0); S232(0); S234(100); S237(100); S240(0); S243(0)]	1.219	0.285698126	0.93019184
Q9Y2W1	1xPhospho [S377(100); S379(0); T381(0)]	1.255	0.327687364	0.93019184
Q9Y2W1	3xPhospho [S217(0); S219(0); S220(0); T227(0); Y228(0); T230(0.3); S232(0.7); S234(99.7); S237(99.3); S240(0.3); S243(99.7)]	0.785	-0.349235441	0.943512809
Q9Y2W1	1xPhospho [S682(100); S684(0); T685(0)]	0.867	-0.205896101	0.953387553
Q9Y2W1	1xPhospho [S559(0); S560(1.4); S562(98.6); T564(0)]	1.11	0.150559677	0.953387553
Q9Y2W1	1xPhospho [S682(100); S684(0); T685(0)]	0.824	-0.279283757	0.9616322
Q9Y2W1	1xPhospho [S320(0); S323(0.1); T324(99.8); S326(0.1); T327(0); Y328(0); S330(0); S331(0); S336(0); S339(0); Y344(0); T345(0)]	0.828	-0.272297327	0.980895643
Q9Y2W1	2xPhospho [S390(99.9); S392(0.1); T397(100); S399(0)]	0.835	-0.260151897	0.980895643
Q9Y2W1	2xPhospho [S248(100); S253(100); S257(0)]	1.036	0.051024003	0.980895643
Q9Y2W1	2xPhospho [S248(100); S253(100); S257(0)]	1.056	0.078609835	0.982029512
Q9Y2W1	2xPhospho [S390(0); S392(100); T397(98.1); S399(1.9)]	0.991	-0.013043037	0.987464758
Q9Y2W1	1xPhospho [S444(100)]	1.011	0.015782997	1
Q9Y2X3	2xPhospho [S502(100); T508(0); S509(0); T510(0); S514(100)]	100	6.64385619	8.35E-17
Q9Y2X3	3xPhospho [S502(100); T508(0.1); S509(50); T510(50); S514(100)]	0.507	-0.979942348	0.664881585
Q9Y2X3	1xPhospho [S502(100); T508(0); S509(0); T510(0); S514(0)]	1.616	0.692427198	0.703341962
Q9Y2X3	3xPhospho [S502(100); T508(0.1); S509(50); T510(50); S514(100)]	0.592	-0.756330919	0.805428458
Q9Y2X3	2xPhospho [S502(100); T508(0); S509(0); T510(0); S514(100)]	0.734	-0.446148032	0.808825211

Q9Y2X3	2xPhospho [S502(100); T508(0); S509(0); T510(0); S514(100)]	0.798	-0.325539348	0.891862526
Q9Y2X3	2xPhospho [S502(100); T508(0); S509(0); T510(0); S514(100)]	0.805	-0.312939312	0.901788734
Q9Y2X3	2xPhospho [S502(100); T508(0); S509(0); T510(0); S514(100)]	0.762	-0.392137097	0.908083211
Q9Y2X3	1xPhospho [S502(100); T508(0); S509(0); T510(0); S514(0)]	1	0	0.980895643
Q9Y320	1xPhospho [S282(0); T283(0); T285(0); T286(0); S288(100)]	0.604	-0.727379545	0.797966154
Q9Y383	1xPhospho [T17(0); S18(100)]	1.194	0.255802837	0.94432975
Q9Y385	1xPhospho [S266(100); T267(0); S268(0)]	0.962	-0.055891201	1
Q9Y3B9	1xPhospho [T104(100); S107(0); T110(0)]	1.609	0.686164326	0.742174171
Q9Y3B9	1xMet-loss+Acetyl [N-Term]; 1xPhospho [S8(0); S11(100)]	1.018	0.025737561	0.962617576
Q9Y3L3	1xPhospho [T534(0); S536(0); S544(100)]	100	6.64385619	8.35E-17
Q9Y3L3	1xPhospho [S262(97.8); S264(0); T266(2.2); T268(0)]	3.048	1.607862903	0.09762892
Q9Y3L3	1xPhospho [S166(0); S168(0); S169(0); S175(100); S178(0); S180(0); T182(0); T183(0)]	1.048	0.067638717	0.973763863
Q9Y3T6	2xPhospho [S276(100); T277(100)]	0.556	-0.846843212	0.879063032
Q9Y3T6	1xPhospho [S276(100); T277(0)]	0.735	-0.444183845	0.971220368
Q9Y3T9	3xPhospho [T15(0); S22(0); S26(100); S28(100); S30(99.8); S32(0.2); S35(0); T40(0)]	0.505	-0.985644707	0.664881585
Q9Y3T9	2xPhospho [S672(100); S673(100); T678(0); S682(0)]	0.88	-0.184424571	0.962617576
Q9Y3Z3	3xPhospho [S18(100); T21(100); S23(0); T25(0); S27(0); S33(100); Y42(0)]	0.581	-0.783389931	0.665552885
Q9Y446	2xPhospho [T308(0); S313(100); S314(100); S323(0)]	0.01	-6.64385619	8.35E-17
Q9Y446	2xPhospho [S313(100); S314(100); S323(0)]	0.01	-6.64385619	8.35E-17
Q9Y446	1xPhospho [S283(3.6); S285(96.4); S287(0); S291(0); S302(0); Y303(0); S305(0)]	0.01	-6.64385619	8.35E-17
Q9Y446	2xPhospho [S283(99.6); S285(0.4); S287(0); S291(0); S302(0.4); Y303(0); S305(99.6)]	0.246	-2.023269779	0.132779461
Q9Y450	1xPhospho [S49(0); Y56(0); Y58(0); S64(0.3); S65(0.3); S67(99.5); S69(0); S74(0)]	1.296	0.374065718	0.883574076
Q9Y450	1xPhospho [S215(0); T221(0); S225(0); S229(100); S230(0); T231(0)]	1.056	0.078609835	0.955790722
Q9Y463	1xPhospho [Y271(0); Y273(100); S276(0)]	1.175	0.232660757	0.941415413
Q9Y478	1xPhospho [S174(5); S177(94.1); S180(0.3); S181(0.3); S182(0.3); Y187(0); Y192(0)]	0.477	-1.067938829	0.607772032
Q9Y478	1xPhospho [S108(100); Y125(0)]	0.897	-0.15682011	0.973942845
Q9Y487	1xPhospho [S695(100); S700(0); S704(0)]	0.77	-0.377069649	0.965139891
Q9Y490	1xPhospho [S455(0); S458(0); S467(100); T476(0); S477(0)]	1.052	0.073134705	0.989554868
Q9Y4B5	2xPhospho [S618(100); T621(86.8); S622(4.3); S627(0); S630(0.2); S631(4.3); T632(4.3)]	0.075	-3.736965594	0.000221817
Q9Y4B5	4 2xPhospho [T423(100); S427(100)]; Q9Y4B5 2xPhospho [T1417(100); S1421(100)]	0.474	-1.077041036	0.588954953
Q9Y4B5	4 2xPhospho [S795(0); S797(100); T810(0); T812(0); S814(100)]; Q9Y4B5 2xPhospho [S1789(0); S1791(100); T1804(0); T1806(0); S1808(100)]	0.478	-1.064917477	0.594120468
Q9Y4B5	2xPhospho [S306(100); T315(100)]	1.799	0.847195187	0.661999767
Q9Y4B5	4 2xPhospho [S818(100); S820(100); S826(0)]; Q9Y4B5 2xPhospho [S1812(100); S1814(100); S1820(0)]	0.472	-1.083141235	0.724397901
Q9Y4B5	4 2xPhospho [S818(100); S820(100); S826(0)]; Q9Y4B5 2xPhospho [S1812(100); S1814(100); S1820(0)]	0.629	-0.668868078	0.85992414
Q9Y4B5	4 3xPhospho [S933(100); T935(0); S937(100); S941(100); T946(0); S947(0)]	0.788	-0.343732465	0.954539739
Q9Y4B5	4 2xPhospho [S415(0.2); T416(4.5); S417(4.5); S418(86.4); T423(4.5); S427(100)]; Q9Y4B5 2xPhospho [S1409(0.2); T1410(4.5); S1411(4.5); S1412(86.4); T1417(4.5); S1421(100)]	0.692	-0.531156057	0.962617576
Q9Y4B5	2xPhospho [S618(100); T621(0.1); S622(99.9); S627(0); S630(0); S631(0); T632(0)]	0.87	-0.200912694	0.978952227
Q9Y4B6	1xPhospho [S979(100); Y984(0); S985(0); S987(0)]	0.851	-0.232768963	0.989898705
Q9Y4C8	3xPhospho [S163(0); S167(0); Y169(0); S174(100); S176(100); S180(100); S194(0)]	0.498	-1.005782353	0.554526265
Q9Y4E8	2xPhospho [S952(0); T955(0); S961(100); S965(100); T980(0)]	0.085	-3.556393349	0.000705995
Q9Y4E8	2xPhospho [S952(0); T955(0); S961(100); S965(100); T980(0)]	0.561	-0.833927324	0.579751615
Q9Y4E8	1xPhospho [S229(100); S233(0); S236(0); T237(0)]	1.466	0.551885103	0.786760931
Q9Y4E8	2xPhospho [S225(97.7); T226(2.3); S229(100); S233(0); S236(0); T237(0)]	1.429	0.515005916	0.79866369
Q9Y4E8	1xPhospho [S952(0); T955(0); S961(4); S965(96); T980(0)]	0.81	-0.304006187	0.956843841
Q9Y4H2	2xPhospho [S304(3.2); S306(93.7); S308(6.4); S309(96.8); S311(0); S312(0); T314(0); S318(0)]	1.109	0.149259365	0.937278821
Q9Y4H2	2xPhospho [S1148(3.1); S1149(96.8); T1151(0.1); S1153(0); S1154(0); T1155(0); T1156(0); T1157(0); T1159(0); S1162(100); S1164(0)]	0.869	-0.202571918	0.965013037
Q9Y4H2	1xPhospho [S1174(0); S1176(100); S1181(0)]	1.049	0.069014678	0.973263372
Q9Y4H2	2xPhospho [Y766(0); S770(100); S772(0); T776(0); T777(0); T779(100); S785(0)]	1	0	0.97998947
Q9Y4H2	1xPhospho [S915(100); Y919(0)]	0.796	-0.329159664	0.981858798
Q9Y4H2	1xPhospho [S304(0); S306(100); S308(0); S309(0); S311(0); S312(0); T314(0); S318(0)]	0.98	-0.029146346	0.99184412
Q9Y4H2	1xPhospho [S306(98.4); S308(1.6); S309(0); S311(0); S312(0); T314(0); S318(0)]	0.886	-0.174621396	1
Q9Y4I1	2xPhospho [T1113(0.2); S1115(4.5); T1116(95.3); S1118(95.5); S1119(4.5); S1122(0); Y1124(0); S1127(0); S1128(0); S1138(0)]	0.581	-0.783389931	0.790280431
Q9Y4I1	2xPhospho [T1113(99.5); S1115(4.4); T1116(4.4); S1118(4.2); S1119(83.3); S1122(4.2); Y1124(0); S1127(0); S1128(0); S1138(0)]	0.686	-0.543719518	0.958702638
Q9Y4I1	3xPhospho [T1113(100); S1115(95.5); T1116(6.7); S1118(48.9); S1119(48.9); S1122(0.1); Y1124(0); S1127(0); S1128(0); S1138(0)]	0.753	-0.40927823	0.973263372
Q9Y4K4	1xPhospho [S433(95.6); S434(4.3); S436(0.2); T443(0); S444(0); S445(0); S453(0)]	100	6.64385619	8.35E-17
Q9Y4W2	1xPhospho [S560(100)]	1.538	0.621055503	0.73347811
Q9Y520	2xPhospho [S2013(100); S2019(0); S2021(0); T2028(6.1); S2029(6.1); S2032(0.5); S2035(6.1); S2036(81.3)]	0.01	-6.64385619	8.35E-17
Q9Y520	2xPhospho [S1303(100); S1304(100)]	100	6.64385619	8.35E-17
Q9Y520	2xPhospho [S1242(95.9); T1244(3.9); S1246(0.2); S1248(50); S1249(50)]	100	6.64385619	8.35E-17
Q9Y520	1xPhospho [S779(100); S782(0); S783(0); S785(0)]	1.849	0.886745225	0.516504611
Q9Y520	1xPhospho [S1977(100); T1980(0); S1983(0); T1986(0); T1988(0)]	1.36	0.443606651	0.803642804
Q9Y520	1xPhospho [S779(95.8); S782(4.2); S783(0); S785(0)]	1.423	0.508935662	0.816750637
Q9Y520	1xPhospho [S1542(100); S1544(0); S1545(0)]	1.317	0.397255346	0.855302203
Q9Y520	3xPhospho [S1242(100); T1244(0); S1246(0.4); S1248(99.8); S1249(99.8)]	1.162	0.216610069	0.94797789
Q9Y520	1xPhospho [S2105(100)]	1.115	0.15704371	0.956843841

Q9Y520	2xPhospho [S1263(4.6); T1265(95.4); T1267(99.8); S1269(0.2); S1274(0); S1276(0); S1280(0); S1282(0)]	0.997	-0.00433459	0.971196575
Q9Y520	1xPhospho [S878(100); T887(0); S893(0)]	0.935	-0.09696173	0.979125671
Q9Y520	2xPhospho [S878(100); T887(0); S893(100)]	0.886	-0.174621396	0.989898705
Q9Y520	2xPhospho [S924(4.7); S926(95.3); S929(0); T932(0); S940(50); S943(50)]	0.891	-0.166502663	0.999834587
Q9Y5A9	1xMet-loss+Acetyl [N-Term]; 1xPhospho [S2(3.1); S4(3.1); S5(93.7)]	1.306	0.385154897	0.855534442
Q9Y5A9	1xPhospho [S359(100); S374(0); S378(0); S380(0); T381(0); S383(0)]	0.778	-0.36215794	0.973263372
Q9Y5B0	2xPhospho [S856(0); S869(100); S872(99.9); S874(0.1)]	0.759	-0.397828209	0.889077103
Q9Y5B0	2xPhospho [S856(0); S869(100); S872(99.9); S874(0.1)]	0.728	-0.457989644	0.891987007
Q9Y5B0	2xPhospho [S856(0); S869(100); S872(95.5); S874(4.5)]	0.635	-0.655171503	0.930939466
Q9Y5B0	2xPhospho [S869(100); S872(94); S874(6)]	0.721	-0.471928835	0.973263372
Q9Y5B6	2xPhospho [T171(0); S180(100); S191(100)]	1.69	0.757023247	0.618224241
Q9Y5B6	2xPhospho [S557(100); S558(100); T563(0); S564(0); T565(0); T568(0)]	0.82	-0.286304185	0.918281661
Q9Y5B6	2xPhospho [S557(100); S558(100); T563(0); S564(0); T565(0); T568(0)]	0.878	-0.187707155	0.969482098
Q9Y5B9	1xPhospho [S1004(100); Y1006(0); S1013(0)]	1.63	0.704871964	0.780165563
Q9Y5B9	1xPhospho [S455(100)]	0.994	-0.008682243	0.99319806
Q9Y5J1	3xPhospho [T204(0); S205(100); S206(100); S210(100)]	0.215	-2.217591435	0.004274404
Q9Y5J1	2xPhospho [S121(100); S124(100)]	3.987	1.995303605	0.007214485
Q9Y5J1	3xPhospho [T204(0); S205(100); S206(100); S210(100)]	0.709	-0.496142467	0.921816052
Q9Y5J1	2xPhospho [T204(0); S205(0); S206(100); S210(100)]	0.817	-0.291592017	0.957850451
Q9Y5J1	2xPhospho [T204(0); S205(0); S206(100); S210(100)]	1.005	0.007195501	1
Q9Y5K3	1xPhospho [S315(100)]	3.157	1.658554261	0.20268588
Q9Y5K6	1xPhospho [T231(4.8); S232(4.8); S233(85.2); S234(4.8); T236(0.3); S249(0)]	0.01	-6.64385619	8.35E-17
Q9Y5K6	3xPhospho [T229(0.1); T231(7.1); S232(90); S233(53.2); S234(96.5); T236(53.2); S249(0)]	0.01	-6.64385619	8.35E-17
Q9Y5K6	2xPhospho [T231(33.5); S232(33.5); S233(33.5); S234(99.6); T236(0); S249(0)]	0.351	-1.510457064	0.057783562
Q9Y5K6	2xPhospho [T229(2.1); T231(48); S232(48); S233(5.8); S234(92.2); T236(4); S249(0)]	0.363	-1.461958547	0.450995428
Q9Y5K6	1xPhospho [S458(100); S463(0); T465(0)]	0.768	-0.380821784	0.891862526
Q9Y5K6	1xPhospho [S86(99.9); T87(0.1); Y88(0); T101(0)]	0.894	-0.161653263	0.989486735
Q9Y5P4	2xPhospho [S375(0); S376(0); S377(0.1); S379(99.9); S380(100); S385(0); S387(0)]	0.879	-0.18606493	0.985461924
Q9Y5Q9	1xPhospho [S43(100); S51(0); S55(0); S56(0); S57(0); S61(0); T62(0)]	0.864	-0.210896782	0.973599548
Q9Y5S2	1xPhospho [S1677(0); T1678(0); S1680(0); S1682(0); S1683(0); S1686(0); S1690(100); S1693(0)]	5.212	2.381837084	0.011169787
Q9Y5S9	1xPhospho [Y54(0); S56(100)]	1.773	0.826192536	0.560990292
Q9Y5T5	1xPhospho [T407(0); S415(100); S423(0); Y424(0)]	1.067	0.093560176	0.97998947
Q9Y608	1xPhospho [S328(100); T331(0); S332(0); S333(0); T339(0); S340(0); S342(0)]	0.89	-0.168122759	0.97998947
Q9Y618	3xPhospho [S2048(0); Y2051(0); S2054(3.4); S2055(93.2); Y2056(0); S2057(3.4); S2065(100); S2068(3.5); S2069(96.4); S2071(0.1); T2073(0)]	0.462	-1.114035243	0.452638679
Q9Y618	1xPhospho [S956(100)]	1.819	0.863145543	0.613145241
Q9Y618	1xPhospho [S2269(100); T2273(0); S2274(0); S2281(0)]	1.413	0.498761466	0.810764434
Q9Y618	2xPhospho [S149(100); S152(100); T156(0)]	1.237	0.3068455	0.884551994
Q9Y618	1xPhospho [S2016(100); S2025(0); S2027(0)]	1.255	0.327687364	0.893481203
Q9Y618	3xPhospho [S737(0); T741(0); S745(6.7); S746(86.7); T748(7.2); S750(99.4); S753(100); T756(0.1)]	0.757	-0.401634795	0.9616322
Q9Y618	2xPhospho [S2048(0); Y2051(0); S2054(2.8); S2055(94.5); Y2056(0); S2057(2.8); S2065(97.1); S2068(2.8); S2069(0.1); S2071(0); T2073(0)]	0.886	-0.174621396	0.964978421
Q9Y618	2xPhospho [S1781(0); S1782(50); S1783(50); S1786(100); T1791(0); T1794(0); T1797(0); T1798(0); T1799(0); S1800(0); S1801(0); S1802(0)]	0.876	-0.190997225	0.999263007
Q9Y624	2xPhospho [Y280(0); S281(0); S284(100); S287(100)]	0.167	-2.582079992	0.000647899
Q9Y624	2xPhospho [Y280(0); S281(0); S284(100); S287(100)]	0.304	-1.717856771	0.012072934
Q9Y624	1xPhospho [Y280(0); S281(0); S284(100)]	1.079	0.109694865	0.96293109
Q9Y666	1xPhospho [T25(0); T30(100); S40(0)]	0.894	-0.161653263	1
Q9Y6A5	2xPhospho [S570(0); T579(0.2); T581(3); S582(48.5); S583(48.5); T590(99.8); S592(0)]	100	6.64385619	8.35E-17
Q9Y6A5	1xPhospho [S570(0); T579(0); T581(0); S582(0.1); S583(99.9); T590(0); S592(0)]	1.403	0.488515009	0.808799965
Q9Y6D5	2xPhospho [S1511(100); S1513(0); S1514(100); S1520(0)]	0.556	-0.846843212	0.494577496
Q9Y6D5	2xPhospho [S614(100); T616(96.1); S617(3.9); S620(0); T621(0); S623(0); S624(0); T626(0); T628(0); T629(0)]	0.537	-0.897006007	0.618224241
Q9Y6D5	2xPhospho [S214(0); S218(100); S227(100)]	0.605	-0.724992953	0.634972474
Q9Y6D5	1xPhospho [S214(0); S218(0); S227(100)]	0.728	-0.457989644	0.971838536
Q9Y6D5	2xPhospho [S236(0.2); S240(99.8); T243(95.7); T244(4.3); T248(0); T251(0)]	1.02	0.028569152	0.973599548
Q9Y6J9	2xPhospho [T489(0); S491(0); S495(100); S501(100)]	1.224	0.291603558	0.925808485
Q9Y6M1	2xPhospho [S153(0); Y154(0); S161(2.9); S162(97.1); S164(100)]	1.495	0.580145484	0.763576928
Q9Y6M1	1xPhospho [S153(0); Y154(0); S161(0); S162(100); S164(0)]	1.128	0.173767068	0.962617576
Q9Y6V7	2xPhospho [T468(100); S470(3.6); S472(96.3); S474(0.1); S479(0)]	0.982	-0.02620507	0.989554868
Q9Y6X9	1xPhospho [S739(0); S743(100)]	100	6.64385619	8.35E-17
Q9Y6X9	2xPhospho [S739(100); S743(100)]	100	6.64385619	8.35E-17
Q9Y6X9	1xPhospho [S615(100)]	2.552	1.351628329	0.077938204
Q9Y6X9	3xPhospho [T723(0); S725(100); S730(100); T733(100); S735(0)]	2.58	1.367371066	0.215713017
Q9Y6X9	2xPhospho [S773(0); S777(100); S779(100); S785(0)]	2.28	1.189033824	0.234817318
Q9Y6X9	2xPhospho [S773(0); S777(100); S779(100); S785(0)]	1.827	0.869476634	0.392224068
Q9Y6X9	2xPhospho [T723(0); S725(100); S730(100); T733(0); S735(0)]	1.781	0.832687516	0.56484421
Q9Y6X9	2xPhospho [S739(100); S743(100)]	1.698	0.763836459	0.655156419
Q9Y6X9	2xPhospho [S739(100); S743(100)]	1.24	0.310340121	0.91490394
