# Supplementary material for: Paladin, overexpressed in colon cancer, is required for actin polymerisation and liver metastasis dissemination
Source: Oncogenesis. 2022 Jul 26;11(1):42. doi: 10.1038/s41389-022-00416-4 (PMC9325978; doi:10.1038/s41389-022-00416-4)
Supplement: Supplementary file 11 — Supplemental table 5 [file 41389_2022_416_MOESM11_ESM.pdf]

Name	Total # of Neighbors	Gene Set Seed	Overlap	Percent Overlap	Overlapping Entities	log	p-value	Jaccard similarity
Protein targets of CDK1	601	CDK1	94	15	MAPT;RPS3;GBF1;BRCA1;INCENP;RANGAP1;STK3;MCM3;NUP210;RAP1GAP;GPHN;RPS6;RPS6KA1;EPB41;CLSPN;NPM1;PCYT1A;RTN4;PHF8;EPHA2;KRT8;NFIC;TSC1;GJA1;SF3B1;PLEC;PBK;MAD2L1BP;NCL;RPA2;AKAP12;EIF4EBP1;RB1;AIMP1;EIF4G1;TP53BP1;BORA;CDC25C;SKA3;KRT18;PAK1;RBBP6;KHDRBS1;DLGAP5;UHRF1;MKI67;GTF2I;APC;EEF2K;PRKAR2B;SRSF1;JUNB;STAG1;CHEK2;RRM2;UBE4B;MINK1;CTNNB1;MAP1B;MAP2;ARHGEF2;MAP4;MCM2;LMNA;RAD9A;NDE1;ERCC6L;RSF1;SLBP;HMGA1;TCOF1;SEPT9;MYSM1;YAP1;PPP1R12A;SQSTM1;VCPIP1;MAP2K1;TMPO;UBA1;FOXK2;MARCKS;CUX1;DAB2;PCM1;FOSL1;MISP;NOLC1;PPP1R13L;KAT7;TRAPPC12;CLASP2;RANBP2;CEP55	133.790485	1.62E-134	0.1032967
Protein targets of PKA	1248	PKA	90	7	ENSA;CREBBP;MAPT;SLC29A1;RPS3;SLK;RAP1GAP;GPHN;NEDD4L;TNKS1BP1;RPS6;SPTBN1;EPB41;SNAPIN;ITGB4;EPHA2;KRT8;TRIP10;GJA1;MED1;PLEC;PJA2;NCL;TOP2B;AKAP12;EIF4EBP1;RB1;EIF4EBP2;WNK1;CDC25B;CDC25C;MYC;PAK1;AHNAK;PTK2;ARID3A;KRT19;UHRF1;SYT12;PAK2;SF1;ARHGAP17;APC;TJP1;PELP1;EEF2K;SLC20A2;MEF2A;PSEN1;SRSF1;SOX9;CTNNB1;NDRG1;TJP2;MAP1A;AKT1;CAST;ERBB2;MAP1B;MARCKSL1;CCNH;MAP2;ARHGEF2;MAP4;HDAC1;MCM2;CTPS1;PHF2;TCF7L2;HMGN2;MARK3;PTPN12;HSP90AA1;NDE1;SCRIB;MARK2;DCLK1;YAP1;PPP1R12A;SQSTM1;RAF1;MARCKS;CUX1;ENAH;ZNRF2;AKAP13;ANK2;FOSL1;NOLC1;ATF2	96.5287083	2.96E-97	0.05765535
Protein targets of PKC	1167	PKC	81	6	ROCK1;MAPT;SLC29A1;RPS3;STK3;HNRNPK;RPS6;EPB41;NPM1;SNAPIN;PCYT1A;ITGB4;KRT8;GJA1;MED1;EIF3A;PLEC;TOP2A;NCL;TOP2B;AKAP12;RB1;EIF4G1;STRN3;CDC25C;KRT18;PAK1;PTK2;SYT12;SF1;CANX;SFPQ (Gene ID 6421);TUBA1B;EPRS;SOS1;PSEN1;PARD3;RPS6KA3;PPFIA1;SLC2A1;CTNNB1;NDRG1;TJP2;MAP1A;AKT1;CAST;MAP1B;MARCKSL1;MAP2;CTPS1;LMNA;RAD9A;HMGN2;PTPN12;CD44;HSP90AA1;MARK2;PDCD4;NCOA2;DYNC1I2;HMGA1;SMARCA4;NDRG2;MET;PPP1R12A;FUS;PRKD3;RAF1;HEXIM1;MARCKS;DAB2;ILF3;ANK2;FOSL1;F11R;NOLC1;PPP1R13L;SH3KBP1;CLASP2;PACSIN2;ATF2	85.205512	6.23E-86	0.05439893
Protein targets of MAPK1	560	MAPK1	66	11	CREBBP;SYMPK;MAPT;RPS3;PALLD;SMAD4;HNRNPK;GPHN;RPS6;RPS6KA1;FXR1;KRT8;TSC1;GJA1;MED1;PBK;TOP2A;TOP2B;EIF4EBP1;RB1;EIF4EBP2;EIF4G1;CDC25C;MYC;CIC;PAK1;PTK2;MKL2;NUP153;SORBS3;GTF2I;EXOC7;APC;SOS1;EEF2K;MEF2A;PARD3;RPS6KA3;RREB1;NDRG1;AKT1;CTNND1;MAP2;PPP1R2;ARHGEF2;TGS1;MAP4;NUP214;MARS;PTPN12;TGIF1;ARHGAP35;EEF2;ERF;NCOA2;SMARCA4;DCLK1;CTTN;MAP2K1;LIMA1;RAF1;MARCKS;CD2BP2;FOSL1;ATF2;CEP55	84.5734887	2.67E-85	0.0735786
Protein targets of casein kinase II	682	casein kinase II	67	9	MAPT;SLC29A1;GBF1;BRCA1;INCENP;SLK;NASP;HDGFL2;OXSR1;SPTAN1;HNRNPK;HNRNPL;SPTBN1;RPS6KA1;EPB41;NPM1;CERS4;CDK11A;PCYT1A;XRCC4;TOP2A;RIC8A;EIF4B;DAXX;NCL;TOP2B;EIF4EBP1;EIF4G1;CDC25B;CDC25C;MYC;PAK1;VAMP4;GTF2F1;UHRF1;CANX;IFRD1;BRD4;MEF2A;FKBP4;PPFIA1;CHEK2;CTNNB1;OTUD5;AKT1;LIG1;MAP1B;CCNH;PPP1R2;HDAC1;MCM2;RAD9A;EHMT2;HSP90AA1;MAX;SLBP;HMGA1;HSP90AB1;SMARCA4;TCOF1;CTTN;SQSTM1;CAV2;ANK2;RPLP2;MDC1;NOLC1	80.3904056	4.07E-81	0.06581532
Protein targets of ATM	284	ATM	52	18	CBL;BRCA1;AVEN;MCM3;TNKS1BP1;CLSPN;NPM1;TRIM28;XRCC4;USP15;DAXX;PDS5B;NCL;RPA2;EIF4EBP1;PBRM1;TP53BP1;PNKP;KHSRP;PAK1;PHRF1;NUP153;CCAR2;AATF;PELP1;MEF2A;RPS6KA3;SLC2A1;CHEK2;NSD2;AKT1;PPP1R2;OTUD4;MCM2;RAD9A;WRAP53;GIT2;HSP90AA1;KDM2A;HMGA1;HSP90AB1;SMARCA4;TCOF1;BAP1;CTTN;MAP2K1;FUS;CHD4;MDC1;BAG6;KAT7;ATF2	77.0231917	9.48E-78	0.08188976
Protein targets of AKT1	545	AKT1	59	10	CREBBP;MAPT;RPS3;BRCA1;PALLD;STK3;C2CD5;NEDD4L;HNRNPL;RPS6;EPHA2;SAMD4A;TSC1;PFKP;GJA1;MED1;EIF4B;WDHD1;CLK1;EIF4EBP1;SETX;WNK1;CDC25B;MATR3;IWS1;CDC25C;KHSRP;PAK1;AHNAK;PTK2;KRT19;NUP153;TBC1D4;CANX;LARP1;MEF2A;SRSF1;KAT6A;CTNNB1;NDRG1;AFDN;NSD2;CTNND1;LMNA;MARK1;ACIN1;PDCD4;NCOA2;NDRG2;SMARCC1;CTTN;YAP1;MAP2K1;RAF1;HEXIM1;DAB2;ILF3;ZNRF2;ATF2	73.2125395	6.13E-74	0.0663667
Protein targets of protein serine-threonine kinase	537	protein serine-threonine kinase	55	10	CREBBP;MAPT;BRCA1;SMAD4;HNRNPK;NEDD4L;RPS6;RPS6KA1;CLSPN;NPM1;EPHA2;FXR1;GJA1;PLEC;EIF4B;DAXX;NCL;EIF4EBP1;RB1;CDC25C;MYC;KHSRP;PAK1;GTF2F1;TBC1D4;CROCC;GTF2I;APC;SOS1;PARD3;PDHA1;CHEK2;CTNNB1;AKT1;MAP1B;CTNND1;MAP2;MAP4;MCM2;CD44;PTPN14;HSP90AA1;EEF2;MARK2;HSP90AB1;SMARCA4;ATM;CTTN;YAP1;PPP1R12A;MAP2K1;RAF1;CSNK1E;ILF3;SAFB	66.8268137	1.49E-67	0.06214689
Protein targets of mitogen-activated protein kinase	505	mitogen-activated protein kinase	54	10	CREBBP;MAPT;SMAD4;HNRNPK;GPHN;SPHK2;RPS6KA1;PCYT1A;RTN4;KRT8;TSC1;GJA1;MED1;TOP2B;EIF4EBP1;RB1;EIF4EBP2;CDC25C;MYC;KHSRP;CIC;KHDRBS1;NUP153;CANX;GTF2I;APC;SOS1;EEF2K;MEF2A;RPS6KA3;JUNB;AKT1;MAP1B;MAP2;PPP1R2;MAP4;HDAC1;TCF7L2;LMNA;BAZ1B;MARK1;NUP214;TGIF1;NCOA2;HMGA1;SMARCA4;CTTN;LIMA1;MARCKS;CD2BP2;FOSL1;STIM2;ATF2;CEP55	66.6420652	2.28E-67	0.06323185
Protein targets of MAPK3	360	MAPK3	49	13	CREBBP;RRAS2;MAPT;RPS3;SMAD4;HNRNPK;GPHN;SPHK2;RPS6KA1;ITGB4;TSC1;GJA1;MED1;EIF4B;EIF4EBP1;EIF4G1;CDC25C;MYC;KHDRBS1;PTK2;CANX;GTF2I;EXOC7;APC;SOS1;RPS6KA3;CTNNB1;AKT1;CTNND1;MAP2;PPP1R2;ARHGEF2;MAP4;BAZ1B;MARS;PTPN12;TGIF1;SCRIB;ERF;SMARCA2;NCOA2;SMARCA4;CTTN;LIMA1;RAF1;MARCKS;CD2BP2;FOSL1;ATF2	65.790485	1.62E-66	0.06862745
Protein targets of CycB family	205	CycB family	38	18	MAPT;JUNB;STAG1;GBF1;RANGAP1;RRM2;MCM3;NUP210;ARHGEF2;MAP4;NPM1;LMNA;TSC1;GJA1;SF3B1;PLEC;PBK;MAD2L1BP;EEF2;SLBP;NCL;RPA2;EIF4EBP1;RB1;EIF4G1;TP53BP1;YAP1;CDC25C;MAP2K1;DLGAP5;UHRF1;CUX1;MKI67;PPP1R13L;EEF2K;PRKAR2B;RANBP2;SRSF1	56.4634416	3.44E-57	0.06666667
Protein targets of CDK	347	CDK	43	12	CREBBP;MAPT;PPFIA1;BRCA1;RANGAP1;RRM2;CTNNB1;MCM3;GPHN;LIG1;MAP1B;MAP2;PPP1R2;EPB41;NPM1;MCM2;LMNA;RAD9A;TSC1;XRCC4;SF3B1;TICRR;PLEC;EEF2;SLBP;NCL;RPA2;AKAP12;EIF4EBP1;HMGA1;RB1;CDC25C;MAP2K1;PAK1;RBL1;FOXK2;MARCKS;UHRF1;RBL2;APC;PELP1;CHAF1A;MEF2A	55.8664611	1.36E-56	0.06082037
Protein targets of CDK5	300	CDK5	39	12	MAPT;RPS3;PPFIA1;CTNNB1;GPHN;ERBB2;MAP1B;CTNND1;MAP2;PPP1R2;RPS6KA1;HDAC1;LMNA;GJA1;NDE1;SLBP;NCL;AXIN1;PPP4R3A;RPA2;ATXN2;SNTB2;RB1;ATM;CTTN;CDC25B;CDC25C;MYC;KRT18;MAP2K1;PAK1;KIF13B;PTK2;MARCKS;ANK2;EPRS;SOS1;MEF2A;PSEN1	51.5228788	3.00E-52	0.05873494

Protein targets of GSK3B	423	GSK3B	42	9	MAPT;ZC3HAV1;SOX9;SMAD4;CTNNB1;NDRG1;MAP1A;RAP1GAP;GPHN;HNRNPL;MAP1B;ZCCHC8;CTNND1;RPS6;MAP2;PPP1R2;NPM1;CTPS1;RNF220;MARK1;FXR1;TSC1;ARHGAP35;NFKB2;HSP90AA1;BCLAF1;MARK2;AXIN1;EIF4EBP1;TP53BP1;MYC;PER1;PTK2;CUX1;DAB2;SFPQ (Gene ID 6421);APC;PELP1;CLASP2;MACF1;MEF2A;PSEN1	50.377786	4.19E-51	0.05357143
Protein targets of SRC	585	SRC	44	7	PARD3;RPS6KA3;CBL;MAPT;BCR;GAK;CTNNB1;AFDN;SPTAN1;HNRNPK;AKT1;ERBB2;CTNND1;MAPRE1;HLA-A;TRIM28;MYO6;EPHA2;TRIP10;GJA1;ARHGAP35;YTHDC1;GIT2;PLEC;PBK;DBNL;BCCIP;ARHGEF28;MET;CTTN;YAP1;KHDRBS1;RAF1;PTK2;CAV2;KRT19;SORBS3;SLC4A7;GRB10;ANK2;F11R;AGAP1;RAPH1;MACF1	47.38405	4.13E-48	0.04661017
Protein targets of ERK1/2	314	ERK1/2	37	11	RPS6KA3;CREBBP;RRAS2;MAPT;RPS3;HNRNPK;GPHN;AKT1;MAP1B;RPS6;MAP2;RPS6KA1;ARHGEF2;MAP4;PCYT1A;ITGB4;MARK1;MARS;GJA1;MED1;ERF;SLBP;EIF4EBP1;HSP90AB1;RB1;EIF4G1;CTTN;MYC;KHDRBS1;SRRM2;LIMA1;PTK2;CANX;EXOC7;SOS1;TPR;ATF2	47.236572	5.80E-48	0.05441177
Protein targets of CDK2	226	CDK2	34	14	CREBBP;MAPT;BRCA1;RRM2;CTNNB1;MCM3;HNRNPK;GPHN;LIG1;RPS6;NPM1;MCM2;USP37;RTN4;CDC5L;PTPN12;SF3B1;TICRR;SLBP;AXIN1;RPA2;HMGA1;RB1;COIL;CDC25C;MAP2K1;FOXK2;MARCKS;UHRF1;BRD4;TUBA1B;APC;PELP1;CLASP2	47.2218488	6.00E-48	0.05714286
Protein targets of ATR	146	ATR	28	19	BRCA1;CHEK2;MCM3;HNRNPK;TNKS1BP1;AKT1;CLSPN;NPM1;MCM2;RAD9A;TRIM28;PDS5B;RPA2;AKAP12;EIF4EBP1;TCOF1;ATM;TP53BP1;THRAP3;CDC25C;MAP2K1;PHRF1;CCAR2;AATF;BAG6;KAT7;PELP1;ATF2	42.134896	7.33E-43	0.0537428
Protein targets of PLK1	250	PLK1	30	11	STAG1;INCENP;CHEK2;MINK1;STK3;RAP1GAP;ROCK2;CLSPN;NPM1;WDR62;XRCC4;TOP2A;MAD2L1BP;ERCC6L;RSF1;AXIN1;EIF4EBP1;MET;TCOF1;AXIN2;TP53BP1;CDC25B;CDC25C;MYC;MDC1;RANBP1;KAT7;CLASP2;RANBP2;CEP55	38.6038007	2.49E-39	0.04815409
Protein targets of CDK2/cyclin E	106	CDK2/cyclin E	22	20	CREBBP;MAPT;SMARCA4;BRCA1;RB1;COIL;CTNNB1;MCM3;CDC25C;UBA1;RBL1;MARCKS;NPM1;MCM2;USP37;PHF8;CDC5L;SF3B1;TICRR;KAT7;PELP1;EEF2	34.0395292	9.13E-35	0.04517454
Protein targets of DNA-PK	154	DNA-PK	24	15	MAPT;RPS3;HNRNPC;BRCA1;CHEK2;OXSR1;AKT1;CLSPN;TRIM28;MED1;XRCC4;HSP90AA1;RPA2;EIF4EBP1;HSP90AB1;ATM;PNKP;MYC;FUS;PER1;HERC2;GTF2F1;HERC1;ILF3	33.8477117	1.42E-34	0.04502814
Protein targets of MAPK14	318	MAPK14	29	9	RPS6KA3;MAPT;JUNB;SMAD4;RBSN;AKT1;RABEP1;KRT8;ARHGAP35;MAX;SMARCA2;NCOA2;EIF4EBP1;SMARCA4;SPAG9;RB1;TP53BP1;YAP1;CDC25B;PPP1R12A;CDC25C;MYC;KRT18;KHSRP;AATF;TUBA1B;EEF2K;ATF2;MEF2A	33.7695511	1.70E-34	0.04190751
Protein targets of protein tyrosine kinase	466	protein tyrosine kinase	32	6	FKBP4;RPS6KA3;CBL;MAPT;PDHA1;SMAD4;CTNNB1;NEDD4L;AKT1;ERBB2;CTNND1;EPB41;MAPRE1;TRIM28;GJA1;ARHGAP35;PLEC;ARHGEF28;CTTN;TMF1;YAP1;PAK1;KHDRBS1;ARHGEF11;RAF1;PTK2;CAV2;GRB10;GTF2I;APC;TJP1;ATF2	33.2441251	5.70E-34	0.03823178
Protein targets of ATM/ATR	87	ATM/ATR	20	22	RPA2;BRCA1;TCOF1;CHEK2;TP53BP1;MCM3;BORA;AKT1;HERC2;CCAR2;MCM2;RAD9A;TRIM28;AATF;MDC1;BAG6;KAT7;PELP1;ATF2;WDHD1	31.9788107	1.05E-32	0.04255319
Protein targets of CaM kinase II	394	CaM kinase II	29	7	MAPT;JUNB;RPS3;SOX9;SMAD4;NECTIN2;CTNNB1;NDRG1;GPHN;AKT1;ERBB2;LIG1;RPS6;MAP2;NAV1;HMGN2;CD44;GJA1;PLEC;EIF4B;RB1;CDC25C;MYC;MAP2K1;RAF1;PTK2;TPD52;MARCKS;ATF2	31.0400052	9.12E-32	0.03776042
Protein targets of CDK2/cyclin A	122	CDK2/cyclin A	21	17	SLBP;LIN9;AXIN1;RPA2;BRCA1;RB1;RRM2;CTNNB1;MCM3;AKT1;LIG1;RBL1;NPM1;UHRF1;MCM2;RAD9A;USP37;TICRR;PELP1;EEF2K;EEF2	30.6575773	2.20E-31	0.04166667
Protein targets of JNK	251	JNK	25	9	MAPT;JUNB;SMAD4;CTNNB1;HNRNPK;AKT1;MAP1B;CTNND1;MARCKSL1;MAP2;PCYT1A;WDR62;NUP214;KRT8;LSR;EIF4ENIF1;MAX;NCOA2;SPAG9;AIMP1;YAP1;CDC25B;CDC25C;MYC;ATF2	30.1567672	6.97E-31	0.03974563
Protein targets of cyclin family	139	cyclin family	21	15	ENSA;SLBP;AXIN1;RPA2;RB1;COIL;CTNNB1;PPP1R12A;LIG1;MAP1B;PAK1;RBL1;FOXK2;NPM1;MCM2;RBL2;RAD9A;SF3B1;PELP1;PBK;EEF2	29.38405	4.13E-30	0.0403071
Protein targets of CDK1/cyclin B1	116	CDK1/cyclin B1	20	17	JUNB;AKAP12;EIF4EBP1;RANGAP1;MINK1;CDC25C;PAK1;KHDRBS1;RPS6;MAP2;ARHGEF2;MPHOSPH8;HDAC1;TSC1;KIF20A;PPP1R13L;PBK;TOP2A;ATF2;CEP55	29.2403322	5.75E-30	0.04008016
Protein targets of AMPK	319	AMPK	26	8	PARD3;MAPT;GBF1;CTNNB1;AFDN;NEDD4L;AKT1;MAP4;CGN;TSC1;MED1;HAT1;EEF2;NCL;NCOA2;RB1;YAP1;CDC25C;RAF1;CSNK1E;TBC1D4;PAK2;CCNY;FOSL1;EEF2K;ATF2	29.0371573	9.18E-30	0.03735632
Protein targets of MTOR	119	MTOR	20	16	JMJD1C;MAPT;EIF4EBP1;EIF4EBP2;EIF4G1;AKT1;EIF5B;RPS6;RPS6KA1;FOXK2;LARP6;TBC1D4;SAMD4A;ZNRF2;GRB10;TUBA1B;LARP1;TJP1;PLEC;EEF2K	29.0013048	9.97E-30	0.03984064
Protein targets of CDK5R1	91	CDK5R1	18	19	AXIN1;MAPT;RPA2;ATXN2;RB1;CTTN;CDC25B;CTNNB1;CDC25C;MAP2K1;GPHN;PAK1;RAF1;RPS6KA1;HDAC1;VRK3;EPRS;PSEN1	27.5406075	2.88E-28	0.03781513
Protein targets of ABL1	230	ABL1	22	9	PARD3;CBL;MAPT;JUNB;BRCA1;SNW1;CTTN;STK3;YAP1;CTNNB1;NEDD4L;NFAT5;CTNND1;SORBS3;RAD9A;ENAH;TRIM28;PAK2;ARHGAP35;RAPH1;SOS1;RANBP2	26.2069084	6.21E-27	0.03600655
Protein targets of GSK3	152	GSK3	19	12	MARK2;AXIN1;MAPT;BRCA1;RB1;SMAD4;CTNNB1;NDRG1;MYC;MAP1B;CTNND1;MAP2;PPP1R2;CTPS1;RBL2;GJA1;SFPQ (Gene ID 6421);APC;MACF1	24.9956786	1.01E-25	0.03544776
Protein targets of AURKB	137	AURKB	18	13	PARD3;ROCK1;EIF4EBP1;INCENP;RB1;ATM;PPP1R12A;SKA3;TNKS1BP1;KIF4A;RPRD1B;MYBBP1A;NPM1;MAPRE1;EHMT2;KIF20A;APC;MAD2L1BP	24.118045	7.62E-25	0.03448276
Protein targets of RPS6K	139	RPS6K	18	12	PDCD4;CREBBP;NDRG2;EIF4G1;WNK1;CDC25B;CTNND1;RPS6;UBR5;TBC1D4;EPHA2;GRB10;FOSL1;LARP1;SOS1;EEF2K;EEF2;EIF4B	24	1.00E-24	0.03435115

Protein targets of PRKCZ	142	PRKCZ	18	12	MARK2;PARD3;NCL;TOP2B;YAP1;TJP2;AFDN;MYC;KRT18;MAP2K1;AKT1;RPS6;RAF1;MARCKS;MARK1;MARK3;F11R;EIF4B	23.8239087	1.50E-24	0.0341556
Protein targets of TORC1	98	TORC1	16	16	EIF4EBP1;RB1;EIF4EBP2;EIF4G1;AKT1;RPS6;RPS6KA1;PTK2;FOXK2;LARP6;SAMD4A;ZNRF2;GRB10;LARP1;EPRS;EEF2K	23.1040253	7.87E-24	0.03298969
Protein targets of RPS6KA2	99	RPS6KA2	16	16	RPS6KA3;PDCD4;CDC25B;PPP1R12A;CDC25C;MYC;AKT1;CTNND1;RPS6;RPS6KA1;TBC1D4;EPHA2;TSC1;SOS1;EEF2K;EIF4B	23.0287242	9.36E-24	0.03292181
Protein targets of glutathione transferase	77	glutathione transferase	15	19	MARK2;AXIN1;MAPT;BRCA1;EIF4G1;CTNNB1;CDC25C;MARCKSL1;CLSPN;EPHA2;PFKP;ARHGAP35;APC;ATF2;SRSF1	22.9393022	1.15E-23	0.03225807
Protein targets of p70RSK	59	p70RSK	14	23	RPS6KA3;PDCD4;MAPT;EIF4EBP1;PPFIA1;EIF4EBP2;HDGFL2;RPS6;RPS6KA1;EPRS;EIF3A;EEF2K;EEF2;EIF4B	22.7772835	1.67E-23	0.03125
Protein targets of Src family	332	Src family	22	6	PARD3;CBL;MAPT;CTTN;YAP1;CTNNB1;TJP2;HNRNPK;FUS;KHDRBS1;CTNND1;RAF1;PTK2;CAV2;TRIM28;GRB10;ARHGAP35;YTHDC1;TJP1;DBNL;RIC8A;ARHGEF28	22.6716204	2.13E-23	0.03085554
Protein targets of p90RSK	80	p90RSK	15	18	PDCD4;CREBBP;MAPT;PPP1R12A;CDC25C;CIC;RPS6;PPP1R2;UBR5;EPHA2;TERF2IP;ZNRF2;SOS1;EEF2K;EIF4B	22.6695862	2.14E-23	0.03205128
Protein targets of RPS6KA1	135	RPS6KA1	17	12	RPS6KA3;PDCD4;MAPT;EIF4EBP1;NDRG2;EIF4EBP2;PPP1R12A;AKT1;RPS6;TBC1D4;ITGB4;EPHA2;GRB10;EPRS;EEF2K;EEF2;EIF4B	22.4841262	3.28E-23	0.03262956
Protein targets of casein kinase I	209	casein kinase I	19	9	AXIN1;MAPT;HNRNPC;YAP1;CTNNB1;SQSTM1;MAP1B;CTNND1;PER1;RPS6;MAP2;PPP1R2;EPB41;CLSPN;MAPRE1;EPHA2;NFIL3;GJA1;APC	22.2856702	5.18E-23	0.03204047
Protein targets of MAPKAPK2	120	MAPKAPK2	16	13	RPS6KA3;CEP131;EIF4EBP1;CDC25B;CDC25C;KRT18;KHSRP;AKT1;TRIM28;RTN4;KRT8;TSC1;AATF;RBM7;EEF2K;ATF2	21.6126102	2.44E-22	0.03155819
Protein targets of PRKCA	291	PRKCA	20	6	CBL;MAPT;EIF4G1;YAP1;CTNNB1;AKT1;MARCKSL1;RAF1;PTK2;MARCKS;LMNA;ITGB4;ZNRF2;GJA1;SFPQ (Gene ID 6421);TUBA1B;F11R;ARHGAP35;PACSIN2;ATF2	20.9788107	1.05E-21	0.02967359
Protein targets of MAPK8	165	MAPK8	17	10	MAPT;JUNB;YAP1;CTNNB1;MYC;HNRNPK;NEDD4L;AKT1;MAP1B;CTNND1;MARCKSL1;MAP2;RPS6KA1;PCYT1A;EIF4ENIF1;ATF2;MEF2A	20.950782	1.12E-21	0.030853
Protein targets of FYN	204	FYN	18	8	RPS6KA3;CBL;MAPT;BCR;CTTN;CTNNB1;TGFB1I1;KHDRBS1;CTNND1;MAP2;RBM10;PTK2;ITGB4;TRIM28;ZDHHC5;GRB10;ARHGAP35;YTHDC1	20.9065783	1.24E-21	0.03056027
Protein targets of casein kinase	142	casein kinase	16	11	NCL;AXIN1;MAPT;RPS3;EIF4EBP1;CTNNB1;CTNND1;PER1;RPS6;MAP2;EPB41;HDAC1;ANK2;GJA1;LTV1;APC	20.394695	4.03E-21	0.03024575
Protein targets of CCNA2	63	CCNA2	13	20	SLBP;MAPT;RB1;RRM2;CDC25B;CTNNB1;CDC25C;UHRF1;MCM2;CDC5L;SF3B1;CLASP2;EEF2	20.3178549	4.81E-21	0.02869757
Protein targets of CHEK1	92	CHEK1	14	15	ERRFI1;MAPT;RPA2;BRCA1;RB1;CHEK2;CDC25B;MCM3;PPP1R12A;CDC25C;CLSPN;TRIM28;AATF;TICRR	19.8356471	1.46E-20	0.02910603
Protein targets of CaMK family	239	CaMK family	18	7	MARK2;FKBP4;CREBBP;MAPT;MET;CTNNB1;CDC25C;GPHN;AKT1;MAP1B;RPS6;MAP2;PPP1R2;ARHGEF2;MARCKS;EEF2K;ATF2;EEF2	19.6575773	2.20E-20	0.02884615
Protein targets of LRRK2	76	LRRK2	13	16	RAB1B;MAPT;RPS3;EIF4EBP1;SQSTM1;AKT1;MAP1B;PTK2;SNAPIN;MARK1;TUBB;KRT8;EIF4B	19.1681302	6.79E-20	0.027897
Protein targets of CSNK1D	56	CSNK1D	12	21	MAPT;YAP1;CTNNB1;PER1;MAP4;HDAC1;SNAPIN;UHRF1;MAPRE1;GJA1;TUBA1B;APC	19.0177288	9.60E-20	0.02684564
Protein targets of PRKCD	186	PRKCD	16	8	RPS3;EIF4EBP1;MET;CDC25B;CTNNB1;HNRNPK;AKT1;PRKD3;MARCKSL1;MARCKS;LMNA;RAD9A;TRIM28;KRT8;GJA1;TJP1	18.4736607	3.36E-19	0.02792321
Protein targets of PTK2B	46	PTK2B	11	23	RPS6KA3;CBL;MAPT;CTTN;CTNNB1;TGFB1I1;ARHGEF11;RAF1;EPB41;PTK2;ARHGEF28	18.0634863	8.64E-19	0.02511416
Protein targets of Rho kinase	129	Rho kinase	14	10	PARD3;NCL;MAPT;SOX9;PPP1R12A;TJP2;RPS6;MAP2;MYO18A;PTK2;MARCKS;CD44;ARHGAP35;SCRIB	17.6861328	2.06E-18	0.02702703
Protein targets of AURKA	129	AURKA	14	10	PARD3;ROCK1;BRCA1;YAP1;CDC25B;HNRNPK;ARHGEF2;DLGAP5;MYBBP1A;TACC3;WDR62;KIF18B;GTF2I;KIF15	17.6861328	2.06E-18	0.02702703
Protein targets of checkpoint kinase	73	checkpoint kinase	12	16	RPA2;BRCA1;RB1;CHEK2;CDC25B;CDC25C;MAP2K1;AKT1;MCM2;AATF;TICRR;APC	17.5185574	3.03E-18	0.02586207

Protein targets of RPS6KA3	101	RPS6KA3	13	12	CREBBP;NCOA2;MAPT;PPP1R12A;CDC25C;RPS6;PPP1R2;RPS6KA1;ITGB4;EPHA2;FOSL1;SOS1;ATF2	17.4672456	3.41E-18	0.02647658
Protein targets of SYK	103	SYK	13	12	CBL;NCL;MAPT;CTTN;CDC25C;ERBB2;MAP1B;KHDRBS1;MAP2;MAP4;TRIM28;TUBA1B;DBNL	17.352617	4.44E-18	0.02636917
Protein targets of PRKCE	107	PRKCE	13	12	MARK2;MAPT;HMGA1;TJP2;MATR3;AKT1;PAK1;MARCKS;KRT8;GJA1;TJP1;SLC20A2;ATF2	17.1278437	7.45E-18	0.02615694
Protein targets of PRKDC	79	PRKDC	12	15	MAPT;RPA2;EIF4EBP1;CHEK2;TP53BP1;AKT1;FUS;RBM25;TRIM28;XRCC4;PELP1;BCLAF1	17.080399	8.31E-18	0.02553192
Protein targets of non-specific protein-tyrosine kinase	163	non-specific protein-tyrosine kinase	14	8	CBL;MAPT;BCR;CTTN;CTNNB1;NEDD4L;PAK1;CTNND1;PTK2;SORBS3;PAK2;GJA1;TUBA1B;ARHGAP35	16.2335872	5.84E-17	0.02536232
Protein targets of atypical PKC	93	atypical PKC	12	12	MARK2;PARD3;CREBBP;ROCK1;TJP2;MARCKS;DAB2;TBC1D4;MARK1;MARK3;F11R;CLASP2	16.1850868	6.53E-17	0.02479339
Protein targets of DYRK1A	94	DYRK1A	12	12	CLK1;MAPT;EIF4EBP1;MYC;MAP1A;MAP1B;CTNND1;MAP2;ANK2;SF3B1;PSEN1;SRSF1	16.1266794	7.47E-17	0.02474227
Protein targets of CHEK2	95	CHEK2	12	12	SLBP;MAPT;BRCA1;RB1;CDC25B;MCM3;PPP1R12A;CDC25C;CDK11A;RAD9A;TRIM28;AATF	16.0695604	8.52E-17	0.02469136
Protein targets of CDK4	70	CDK4	11	15	MARCKS;NPM1;RBL2;SLBP;MAPT;NDRG2;BRCA1;RB1;PAK1;PELP1;RBL1	15.8794261	1.32E-16	0.02380952
Protein targets of MARK2	32	MARK2	9	27	PARD3;MAPT;STK3;MAP1A;UTRN;KIF13B;MAP2;ARHGEF2;MAP4	15.5985995	2.52E-16	0.02112676
Protein targets of RPS6KB1	74	RPS6KB1	11	14	PDCD4;MAPT;EIF4EBP1;NDRG2;GRB10;AKT1;RPS6;EEF2K;RPS6KA1;EEF2;EIF4B	15.5985995	2.52E-16	0.02360515
Protein targets of PKG	240	PKG	15	6	MET;SOX9;CTNNB1;PPP1R12A;PAK1;RPS6;RAF1;HDAC1;MARCKS;ENAH;PAK2;SF1;GJA1;GTF2I;ARHGAP17	15.2749055	5.31E-16	0.02388535
Protein targets of EGFR	153	EGFR	13	8	FKBP4;MET;CTNNB1;TGFB1I1;MYC;AKT1;ERBB2;CTNND1;MAP2;EPB41;PTK2;PFKP;F11R	15.0574959	8.76E-16	0.02394107
Protein targets of SMG1	25	SMG1	8	30	NPM1;NCL;RAD9A;RPA2;EIF4EBP1;EIF4EBP2;ATM;XRCC4	14.4436975	3.60E-15	0.01904762
Protein targets of SAPK	94	SAPK	11	11	NCL;PRKRA;MAPT;JUNB;UBXN1;KRT8;CDC25C;MYC;ZCCHC8;EEF2K;ATF2	14.4012095	3.97E-15	0.02263375
Protein targets of IKBKB	96	IKBKB	11	11	EDC4;RPS3;TSC1;ATM;CTNNB1;PPP1R12A;NFKB2;NEDD4L;KHDRBS1;RPS6KA1;MAD2L1BP	14.2975695	5.04E-15	0.02254098
Protein targets of MAPK9	96	MAPK9	11	11	PCYT1A;MAPT;YAP1;CDC25B;CTNNB1;MYC;NEDD4L;AKT1;MARCKSL1;ATF2;PSEN1	14.2975695	5.04E-15	0.02254098
Protein targets of PRKD1	105	PRKD1	11	10	MARK2;MARCKS;SNAPIN;ITGB4;CTTN;CTNNB1;KIDINS220;SPHK2;PAK1;MEF2A;RABEP1	13.8569852	1.39E-14	0.0221328
Protein targets of PTK6	29	PTK6	8	26	CBL;SMAD4;CTNNB1;SFPQ (Gene ID 6421);ARHGAP35;AKT1;KHDRBS1;PTK2	13.8507809	1.41E-14	0.01886793
Protein targets of CSNK2A2	55	CSNK2A2	9	16	MAX;EIF4EBP1;PDAP1;CANX;CDC25B;SQSTM1;MYC;XRCC4;RBM14	13.2684112	5.39E-14	0.02004454
Protein targets of MAPK7	57	MAPK7	9	15	RPS6KA3;GJA1;FOSL1;MYC;LIMA1;RPS6KA1;ATF2;MEF2A;PTK2	13.1197582	7.59E-14	0.01995565
Protein targets of RPS6KA5	57	RPS6KA5	9	15	RPS6KA3;MAPT;TRIM28;WNK1;CTNNB1;AKT1;RPS6;RPS6KA1;ATF2	13.1197582	7.59E-14	0.01995565
Protein targets of PI3K	91	PI3K	10	10	AXIN1;RPA2;EIF4EBP1;BRCA1;CHEK2;TSC1;MED1;KHSRP;AKT1;PTK2	12.8664611	1.36E-13	0.02066116
Protein targets of CDC7	39	CDC7	8	20	MCM2;RAD9A;PSIP1;MCM3;RPS6;CHAF1A;WDHD1;CLSPN	12.7033348	1.98E-13	0.01843318

Protein targets of MELK	24	MELK	7	28	CDC5L;SMAD4;CDC25B;CDC25C;SF3B1;DBNL;EIF4B	12.377786	4.19E-13	0.01666667
Protein targets of MARK1	13	MARK1	6	42	PARD3;MAPT;STK3;CDC25C;MAP2;MAP4	12.111259	7.74E-13	0.01463415
Protein targets of CSNK2A1	84	CSNK2A1	9	10	NIPBL;NCL;MAPT;RPS3;EIF4G1;MYC;F11R;EEF2K;EIF4B	11.537602	2.90E-12	0.01882845
Protein targets of PAK1	124	PAK1	10	8	MORC2;DAXX;MYO6;FXR1;CTTN;CTNNB1;MAP2K1;GLCCI1;RAF1;ARHGEF2	11.4962093	3.19E-12	0.01934236
Protein targets of MLST8	16	MLST8	6	35	EIF4EBP1;EIF4G1;AKT1;RPS6;RPS6KA1;EEF2	11.4449056	3.59E-12	0.01452785
Protein targets of ERBB2	32	ERBB2	7	21	FASN;MKI67;YAP1;CTNNB1;DOCK7;AKT1;TOP2A	11.39794	4.00E-12	0.01635514
Protein targets of CDK1/cyclin A	32	CDK1/cycl in A	7	21	CUX1;SLBP;RPA2;RB1;CDC25B;CDC25C;RBL1	11.39794	4.00E-12	0.01635514
Protein targets of SGK1	87	SGK1	9	10	TBC1D4;MAPT;NDRG2;ZNRF2;WNK1;NDRG1;NEDD4L;AKT1;RPS6KA1	11.395774	4.02E-12	0.01871102
Protein targets of JAK2	89	JAK2	9	10	NFIC;GJA1;GTF2I;AKT1;ERBB2;PAK1;RAF1;ATF2;TJP3	11.3053948	4.95E-12	0.01863354
Protein targets of MET	35	MET	7	19	ITGB4;CTTN;YAP1;CTNNB1;CTNND1;PBK;PTK2	11.1007268	7.93E-12	0.0162413
Protein targets of RPS6	18	RPS6	6	31	MCM2;PDCD4;GRB10;EEF2K;EEF2;EIF4B	11.0824945	8.27E-12	0.01445783
Protein targets of ROCK1	62	ROCK1	8	12	MARCKS;PARD3;MAPT;PAK2;SOX9;PPP1R12A;MAP2;PTK2	10.9871628	1.03E-11	0.01750547
Protein targets of PIM1	62	PIM1	8	12	MARK2;EIF4EBP1;MARK3;CBX8;SQSTM1;CDC25C;MYC;EIF4B	10.9871628	1.03E-11	0.01750547
Protein targets of GAPDH	62	GAPDH	8	12	EIF4EBP1;ATM;YAP1;CTNNB1;GJA1;PPP1R12A;AKT1;RPS6	10.9871628	1.03E-11	0.01750547
Protein targets of LYN	141	LYN	10	7	CBL;TRIM28;CTTN;TGFB1I1;TUBA1B;HNRNPK;KHDRBS1;CTNND1;RPS6;DBNL	10.935542	1.16E-11	0.01872659
Protein targets of CSK	69	CSK	8	11	PARD3;MYO6;CTTN;CTNNB1;HNRNPK;KHDRBS1;CTNND1;EEF2	10.60206	2.50E-11	0.01724138
Protein targets of PRKCB	108	PRKCB	9	8	MARCKS;SMARCA4;RB1;EIF4G1;GJA1;TJP2;AKT1;EIF3A;RAF1	10.536107	2.91E-11	0.01792829
Protein targets of RAF1	73	RAF1	8	10	RB1;ANK2;GJA1;PPP1R12A;TUBA1B;KRT18;MAP2K1;SLC20A2	10.4001169	3.98E-11	0.01709402
Protein targets of KDR	23	KDR	6	25	CBL;CTNNB1;GJA1;AKT1;CTNND1;PTK2	10.35164	4.45E-11	0.01428571
Protein targets of AKT2	75	AKT2	8	10	CLK1;CREBBP;DAB2;TBC1D4;CTNNB1;AKT1;RPS6;SRSF1	10.3036436	4.97E-11	0.01702128
Protein targets of CDK6	45	CDK6	7	15	NPM1;RBL2;BRCA1;RB1;PFKP;CTNNB1;RBL1	10.2814983	5.23E-11	0.01587302
Protein targets of MAPK12	45	MAPK12	7	15	MAPT;RB1;CDC25C;CAST;EEF2K;ATF2;MEF2A	10.2814983	5.23E-11	0.01587302
Protein targets of PDPK1	77	PDPK1	8	10	RPS6KA3;PAK2;SMAD4;MAP2K1;AKT1;PAK1;RPS6;RPS6KA1	10.2104193	6.16E-11	0.01694915
Protein targets of PTK2	78	PTK2	8	10	TBC1D4;CTTN;CTNNB1;ARHGAP35;GIT2;KHDRBS1;ARHGEF11;MACF1	10.1643094	6.85E-11	0.01691332
Protein targets of ACTB	86	ACTB	8	9	LMNA;MAPT;EIF4EBP1;RB1;CHEK2;MYC;AKT1;PTK2	9.81815641	1.52E-10	0.01663202
Protein targets of HIPK2	55	HIPK2	7	12	DAXX;TCF7L2;CREBBP;HMGA1;SMAD4;AATF;CTNNB1	9.64206515	2.28E-10	0.01552106
Protein targets of STK11	55	STK11	7	12	MARK2;TBC1D4;MARK1;MARK3;SMAD4;PPP1R12A;PAK1	9.64206515	2.28E-10	0.01552106

Protein targets of receptor protein-tyrosine kinase	93	receptor protein-tyrosine kinase	8	8	RPS6KA3;EIF4EBP1;CTNNB1;KIDINS220;AKT1;CTNND1;RPS6KA1;PTK2	9.5421181	2.87E-10	0.01639344
Protein targets of PLK3	32	PLK3	6	18	HSP90AB1;CHEK2;CDC25C;HSP90AA1;TOP2A;ATF2	9.40782324	3.91E-10	0.01398601
Protein targets of INSR	99	INSR	8	8	CAV2;CBL;TBC1D4;MAPT;AKT1;KHDRBS1;MAP2;PTK2	9.32330639	4.75E-10	0.01619433
Protein targets of PAK2	63	PAK2	7	10	EIF4G1;MYC;MAP2K1;RPS6;RAF1;ARHGEF2;EIF4B	9.21467017	6.10E-10	0.01525055
Protein targets of PAK4	35	PAK4	6	16	PAK2;CTNNB1;PAK1;ARHGEF11;RAF1;ARHGEF2	9.1580152	6.95E-10	0.01388889
Protein targets of CCNB1	35	CCNB1	6	16	EIF4G1;CTNNB1;CDC25C;PAK1;RPS6;PBK	9.1580152	6.95E-10	0.01388889
Protein targets of MKI67	16	MKI67	5	29	RB1;ATM;SQSTM1;AKT1;RPS6	9.15058059	7.07E-10	0.0120773
Protein targets of NLK	37	NLK	6	15	TCF7L2;CREBBP;SMAD4;YAP1;MAP1B;MEF2A	9.00392635	9.91E-10	0.01382489
Protein targets of CSNK1E	37	CSNK1E	6	15	FZD6;AXIN1;NFIL3;CTNNB1;APC;PER1	9.00392635	9.91E-10	0.01382489
Protein targets of TTK	38	TTK	6	15	MAPT;MET;CHEK2;CDC25B;MAD2L1BP;MAP4	8.93181414	1.17E-09	0.0137931
Protein targets of RPS6KB2	18	RPS6KB2	5	26	RPS6KA3;PDCD4;RPS6;EEF2K;EIF4B	8.86012091	1.38E-09	0.01201923
Protein targets of H2AFX	18	H2AFX	5	26	RPA2;TRIM28;CHEK2;CDC25C;AKT1	8.86012091	1.38E-09	0.01201923
Protein targets of CDK8	39	CDK8	6	15	RAD9A;HMGA1;TP53BP1;YAP1;SETD1A;CCNH	8.86012091	1.38E-09	0.01376147
Protein targets of SRPK2	19	SRPK2	5	25	CLK1;MAPT;ACIN1;LUC7L3;SRSF1	8.72815839	1.87E-09	0.01199041
Protein targets of RPTOR	20	RPTOR	5	23	MAPT;EIF4EBP1;EIF4G1;RPS6;RPS6KA1	8.60380065	2.49E-09	0.01196172
Protein targets of MAP2	21	MAP2	5	22	MAPT;EIF4EBP1;TUBA1B;AKT1;MAP1B	8.4867824	3.26E-09	0.01193317
Protein targets of FAK/Src	21	FAK/Src	5	22	CTTN;ARHGAP35;MACF1;PTK2;ARHGEF28	8.4867824	3.26E-09	0.01193317
Protein targets of CSNK1A1	84	CSNK1A1	7	8	ERF;MAPT;HNRNPC;EIF4G1;CTNNB1;APC;CTNND1	8.32330639	4.75E-09	0.01458333
Protein targets of PAK	85	PAK	7	8	MYO6;CTTN;CTNNB1;PPP1R12A;MAP2K1;RAF1;ARHGEF2	8.2873503	5.16E-09	0.01455302
Protein targets of LATS1	24	LATS1	5	20	RBM25;INCENP;YAP1;PPP1R12A;RAF1	8.1700533	6.76E-09	0.01184834
Protein targets of MAPK13	24	MAPK13	5	20	MAPT;EIF4EBP1;EEF2K;ATF2;MEF2A	8.1700533	6.76E-09	0.01184834
Protein targets of MAP3K2	24	MAP3K2	5	20	CTNNB1;FOSL1;MYC;MAP2K1;RAF1	8.1700533	6.76E-09	0.01184834
Protein targets of MARK4	9	MARK4	4	40	MAPT;STK3;MAP2;MAP4	8.1260984	7.48E-09	0.00980392
Protein targets of SRPK1	25	SRPK1	5	19	SF1;TRA2B;SFPQ (Gene ID 6421);RBBP6;SRSF1	8.07417243	8.43E-09	0.01182033
Protein targets of FER	25	FER	5	19	TRIM28;CTTN;TMF1;CTNNB1;CTNND1	8.07417243	8.43E-09	0.01182033
Protein targets of p38	92	p38	7	7	NCOA2;MAPT;KRT8;SMAD4;RBSN;ATF2;MEF2A	8.04527521	9.01E-09	0.01434426

Protein targets of GSK3A	54	GSK3A	6	10	MAPT;EIF4EBP1;SOX9;CTNNB1;MYC;CTNND1	7.97469414	1.06E-08	0.01330377
Protein targets of MAPK11	55	MAPK11	6	10	MAPT;JUNB;KHSRP;EEF2K;ATF2;MEF2A	7.92811799	1.18E-08	0.01327434
Protein targets of BRSK2	10	BRSK2	4	36	MAPT;CDC25C;PAK1;RAF1	7.90657832	1.24E-08	0.00977995
Protein targets of CCND1	27	CCND1	5	17	MAPT;BRCA1;RB1;GJA1;RBL1	7.89619628	1.27E-08	0.01176471
Protein targets of TUBA1B	27	TUBA1B	5	17	EPHA2;ATM;CTNNB1;AKT1;RPS6	7.89619628	1.27E-08	0.01176471
Protein targets of CAMK4	56	CAMK4	6	10	CREBBP;MAPT;HNRNPL;EEF2K;MAP2;MEF2A	7.87942607	1.32E-08	0.01324503
Protein targets of CCNE1	28	CCNE1	5	17	NPM1;LIN9;CDC5L;RB1;CTNNB1	7.8096683	1.55E-08	0.01173709
Protein targets of BCR-ABL	59	BCR-ABL	6	10	CBL;ENAH;EIF4EBP1;CTNNB1;FUS;PTK2	7.73992861	1.82E-08	0.0131579
Protein targets of CycD family	30	CycD family	5	16	RBL2;MAPT;BRCA1;RB1;RBL1	7.64975198	2.24E-08	0.01168224
Protein targets of PAK3	31	PAK3	5	15	MYO6;PAK2;CTTN;MAP2K1;RAF1	7.57511836	2.66E-08	0.01165501
Protein targets of CDK7	64	CDK7	6	9	CDK11A;PPP4R3A;MAPT;YAP1;XRN2;BRD4	7.52287875	3.00E-08	0.01301518
Protein targets of F2R	13	F2R	4	28	PARD3;MAPT;UTRN;MAP2	7.3757179	4.21E-08	0.00970874
Protein targets of alpha2beta2 integrin	13	alpha2beta2 integrin	4	28	NCL;CANX;NOLC1;BTF3	7.3757179	4.21E-08	0.00970874
Protein targets of trypsin	14	trypsin	4	26	PTGES3;UBXN1;PPP1R12A;RAF1	7.23062267	5.88E-08	0.00968523
Protein targets of Aurora kinase	38	Aurora kinase	5	12	HDAC1;NPM1;KIF4A;ARHGEF2;MYBBP1A	7.11182051	7.73E-08	0.01146789
Protein targets of CTNNB1	15	CTNNB1	4	25	MAPT;RB1;DCLK1;WNK1	7.09691001	8.00E-08	0.00966184
Protein targets of protein phosphatase	15	protein phosphatase	4	25	BRCA1;ATM;MAP1B;MAP2	7.09691001	8.00E-08	0.00966184
Protein targets of DAPK1	39	DAPK1	5	12	MAPT;NDRG2;MCM3;MAP2;MAP4	7.05305673	8.85E-08	0.01144165
Protein targets of PRKACA	40	PRKACA	5	12	NCL;SOX9;CDC25B;CTNNB1;MEF2A	6.99567863	1.01E-07	0.01141553
Protein targets of EIF2AK4	16	EIF2AK4	4	23	MARS;AATF;LTV1;AKT1	6.97469414	1.06E-07	0.00963855
Protein targets of IKBK	83	IKBK	6	7	EDC4;IK;TSC1;YAP1;NFKB2;NEDD4L	6.84163751	1.44E-07	0.0125
Protein targets of PRKCI	44	PRKCI	5	11	CREBBP;NCL;CTTN;CLASP2;PTK2	6.78251606	1.65E-07	0.01131222
Protein targets of PLK4	18	PLK4	4	21	CEP131;CHEK2;PCM1;CDC25C	6.74958	1.78E-07	0.00959233
Protein targets of EIF2AK2	45	EIF2AK2	5	10	MAPT;ILF3;EIF4EBP1;RPS6;EEF2	6.73282827	1.85E-07	0.01128668
Protein targets of CAMK1	46	CAMK1	5	10	MARK2;PCYT1A;MARK1;EEF2K;MEF2A	6.68402966	2.07E-07	0.01126126

Protein targets of CDK6/cyclin D1	5	CDK6/cyclin D1	3	50	RB1;CTNNB1;MYC	6.66554625	2.16E-07	0.00740741
Protein targets of RPL13	5	RPL13	3	50	BRCA1;TP53BP1;MDC1	6.66554625	2.16E-07	0.00740741
Protein targets of TAOK1	19	TAOK1	4	20	MARK2;MAPT;MARK1;STK3	6.64781748	2.25E-07	0.00956938
Protein targets of CDC7/DBF4	19	CDC7/DBF4	4	20	MCM2;CHEK2;MCM3;CHAF1A	6.64781748	2.25E-07	0.00956938
Protein targets of JUN	19	JUN	4	20	RPS6KA3;MKI67;MARCKSL1;RPS6	6.64781748	2.25E-07	0.00956938
Protein targets of TORC2	48	TORC2	5	10	EIF4EBP1;NDRG1;OXSR1;AKT1;RPS6KA1	6.58838029	2.58E-07	0.01121076
Protein targets of PRKCQ	49	PRKCQ	5	10	MARCKS;CBL;NDRG2;RAF1;MEF2A	6.54363397	2.86E-07	0.01118568
Protein targets of MAPK10	49	MAPK10	5	10	MAPT;GJA1;MYC;MARCKSL1;ATF2	6.54363397	2.86E-07	0.01118568
Protein targets of TNK2	21	TNK2	4	18	CTPS1;CTTN;AKT1;PTK2	6.4609239	3.46E-07	0.00952381
Protein targets of CCND3	21	CCND3	4	18	NCL;RB1;PFKP;RBL1	6.4609239	3.46E-07	0.00952381
Protein targets of WNK4	22	WNK4	4	17	TJP2;OXSR1;NEDD4L;PAK1	6.37468755	4.22E-07	0.00950119
Protein targets of ERBB3	6	ERBB3	3	42	CBL;TOP2A;PTK2	6.36653154	4.30E-07	0.00738916
Protein targets of MCM2	6	MCM2	3	42	RB1;CHEK2;SF3B1	6.36653154	4.30E-07	0.00738916
Protein targets of NDR1/2 kinase	6	NDR1/2 kinase	3	42	PARD3;AAK1;YAP1	6.36653154	4.30E-07	0.00738916
Protein targets of small monomeric GTPase	6	small monomeric GTPase	3	42	PPP1R12A;F11R;RPS6	6.36653154	4.30E-07	0.00738916
Protein targets of WNK3	6	WNK3	3	42	WNK1;OXSR1;NEDD4L	6.36653154	4.30E-07	0.00738916
Protein targets of LATS2	23	LATS2	4	16	RBM25;INCENP;YAP1;CDC25C	6.29242982	5.10E-07	0.00947867
Protein targets of Ras GTPase	24	Ras GTPase	4	16	RPS6KA3;TSC1;RPS6;RAF1	6.21467017	6.10E-07	0.00945627
Protein targets of WNK1	25	WNK1	4	15	TBC1D4;OXSR1;NEDD4L;PAK1	6.13966199	7.25E-07	0.00943396
Protein targets of WNK2	25	WNK2	4	15	WNK1;OXSR1;MAP2K1;PAK1	6.13966199	7.25E-07	0.00943396
Protein targets of ATR-ATRIP	7	ATR-ATRIP	3	37	MCM2;RPA2;CLSPN	6.12378216	7.52E-07	0.00737101
Protein targets of receptor protein serine/threonine kinase	26	receptor protein serine/threonine kinase	4	14	MAPT;RPS6;MAP2;PTK2	6.06803389	8.55E-07	0.00941177

Protein targets of polynucleotide 5'-hydroxyl-kinase	26	polynucleotide 5'-hydroxyl-kinase	4	14	RPA2;CHEK2;MYC;FUS	6.06803389	8.55E-07	0.00941177
Protein targets of NDRG1	8	NDRG1	3	33	PARD3;AAK1;YAP1	5.92081875	1.20E-06	0.00735294
Protein targets of m_Tel1p	8	m_Tel1p	3	33	RAD9A;EIF4EBP1;CHEK2	5.92081875	1.20E-06	0.00735294
Protein targets of HSPB1	8	HSPB1	3	33	GJA1;HSP90AA1;ATF2	5.92081875	1.20E-06	0.00735294
Protein targets of mitogen-activated protein kinase kinase	68	mitogen-activated protein kinase kinase	5	7	CLK1;MAPT;CTTN;FOSL1;MAP2K1	5.82390874	1.50E-06	0.01072961
Protein targets of CLK1	30	CLK1	4	12	CDKN3;TRA2B;EEF2;SRSF1	5.8096683	1.55E-06	0.00932401
Protein targets of JNKK	30	JNKK	4	12	JUNB;TP53BP1;AKT1;ATF2	5.8096683	1.55E-06	0.00932401
Protein targets of CHUK	69	CHUK	5	7	CREBBP;RPS3;CTNNB1;NFKB2;KHDRBS1	5.79317412	1.61E-06	0.01070664
Protein targets of MASTL	9	MASTL	3	30	ENSA;HMGA1;CDC25C	5.7447275	1.80E-06	0.00733496
Protein targets of mTOR-raptor	9	mTOR-raptor	3	30	EIF4EBP1;RPS6;RPS6KA1	5.7447275	1.80E-06	0.00733496
Protein targets of PRPF4B	9	PRPF4B	3	30	PRPF31;SMARCA4;YAP1	5.7447275	1.80E-06	0.00733496
Protein targets of BRSK1	9	BRSK1	3	30	MAPT;CDC25B;CDC25C	5.7447275	1.80E-06	0.00733496
Protein targets of DYRK2	32	DYRK2	4	12	MAPT;EIF4EBP1;MYC;SF3B1	5.69250396	2.03E-06	0.00928074
Protein targets of FGFR	32	FGFR	4	12	RPS6KA3;CTNNB1;CTNND1;PTK2	5.69250396	2.03E-06	0.00928074
Protein targets of TFIIH	32	TFIIH	4	12	MAPT;IFRD1;BRD4;GTF2F1	5.69250396	2.03E-06	0.00928074
Protein targets of GRK2	131	GRK2	6	4	NPM1;FZD6;AXIN1;STK3;RPLP2;NEDD4L	5.6716204	2.13E-06	0.01136364
Protein targets of NUAK1	10	NUAK1	3	27	MAPT;ATM;PPP1R12A	5.59176004	2.56E-06	0.00731707
Protein targets of PRKD3	10	PRKD3	3	27	CTTN;CTNNB1;SPHK2	5.59176004	2.56E-06	0.00731707
Protein targets of CDKL5	10	CDKL5	3	27	MAP1S;CEP131;ARHGEF2	5.59176004	2.56E-06	0.00731707
Protein targets of NEK2	35	NEK2	4	11	HMGA1;CROCC;CTNNB1;SRSF1	5.53165267	2.94E-06	0.00921659
Protein targets of actins	35	actins	4	11	EIF4EBP1;RB1;CDC25C;AKT1	5.53165267	2.94E-06	0.00921659
Protein targets of CASP3	11	CASP3	3	25	XRCC4;PAK1;EEF2	5.45469288	3.51E-06	0.00729927

Protein targets of GYS1	11	GYS1	3	25	MAPT;CTNNB1;PTK2	5.45469288	3.51E-06	0.00729927
Protein targets of BUB1	11	BUB1	3	25	EIF4EBP1;INCENP;APC	5.45469288	3.51E-06	0.00729927
Protein targets of CycE family	11	CycE family	3	25	MCM2;BRCA1;RB1	5.45469288	3.51E-06	0.00729927
Protein targets of CDK4/cyclin D1	37	CDK4/cycl in D1	4	10	RB1;MYC;PELP1;RBL1	5.43297363	3.69E-06	0.00917431
Protein targets of CDK9	39	CDK9	4	10	NCOA2;RB1;XRN2;HTATSF1	5.3400838	4.57E-06	0.00913242
Protein targets of YES1	12	YES1	3	23	PARD3;YAP1;RAF1	5.32975415	4.68E-06	0.00728155
Protein targets of NDRG2	12	NDRG2	3	23	PARD3;AAK1;YAP1	5.32975415	4.68E-06	0.00728155
Protein targets of tau-protein kinase	13	tau-protein kinase	3	21	MARCKS;MAPT;MAP2	5.21752738	6.06E-06	0.00726392
Protein targets of MARK3	13	MARK3	3	21	CDC25C;PPP1R2;PKP2	5.21752738	6.06E-06	0.00726392
Protein targets of 14-3-3	13	14/03/03	3	21	KRT8;ATM;CTTN	5.21752738	6.06E-06	0.00726392
Protein targets of ERBB4	13	ERBB4	3	21	TRIM28;MYC;TOP2A	5.21752738	6.06E-06	0.00726392
Protein targets of STK24	42	STK24	4	9	NDRG2;PTPN12;YAP1;NDRG1	5.20901153	6.18E-06	0.0090703
Protein targets of CDKN1A	14	CDKN1A	3	20	RB1;TP53BP1;TUBA1B	5.11350928	7.70E-06	0.00724638
Protein targets of ABL2	14	ABL2	3	20	MAPT;CTTN;ARHGAP35	5.11350928	7.70E-06	0.00724638
Protein targets of DCN	2	DCN	2	66	RPS6;EIF4B	5.10846254	7.79E-06	0.00496278
Protein targets of spliceosome	2	spliceoso me	2	66	CDC5L;SF3B1	5.10846254	7.79E-06	0.00496278
Protein targets of CNDP2	2	CNDP2	2	66	GJA1;AKT1	5.10846254	7.79E-06	0.00496278
Protein targets of DDIT4L	2	DDIT4L	2	66	AKT1;RPS6KA1	5.10846254	7.79E-06	0.00496278
Protein targets of DOCK1	2	DOCK1	2	66	PAK2;PAK1	5.10846254	7.79E-06	0.00496278
Protein targets of VCP	2	VCP	2	66	RB1;CHEK2	5.10846254	7.79E-06	0.00496278
Protein targets of CDKL2	2	CDKL2	2	66	MAP1S;CEP131	5.10846254	7.79E-06	0.00496278
Protein targets of initiation factor 4E binding protein	2	initiation factor 4E binding protein	2	66	EIF4EBP1;RPS6	5.10846254	7.79E-06	0.00496278
Protein targets of CDKL4	2	CDKL4	2	66	MAP1S;CEP131	5.10846254	7.79E-06	0.00496278
Protein targets of JAG2	2	JAG2	2	66	RB1;AKT1	5.10846254	7.79E-06	0.00496278

Protein targets of polo kinase	46	polo kinase	4	8	HSP90AB1;CDC25C;HSP90AA1;ARHGEF2	5.04914854	8.93E-06	0.00898876
Protein targets of HIPK1	15	HIPK1	3	18	DAXX;CREBBP;YAP1	5.01727661	9.61E-06	0.00722892
Protein targets of ACACA	15	ACACA	3	18	FASN;AKT1;RPS6	5.01727661	9.61E-06	0.00722892
Protein targets of EIF4EBP1	16	EIF4EBP1	3	17	CHEK2;RPS6;EEF2	4.92811799	1.18E-05	0.00721154
Protein targets of CHKA	16	CHKA	3	17	PCYT1A;CDC25C;AKT1	4.92811799	1.18E-05	0.00721154
Protein targets of GYS2	16	GYS2	3	17	MAPT;CTNNB1;MAP2	4.92811799	1.18E-05	0.00721154
Protein targets of CDK4/6	17	CDK4/6	3	16	RBL2;RB1;RBL1	4.84466396	1.43E-05	0.00719425
Protein targets of PDK2	17	PDK2	3	16	PDHA1;AKT1;RPS6	4.84466396	1.43E-05	0.00719425
Protein targets of IKBKE	52	IKBKE	4	7	RPS3;TSC1;CTNNB1;AKT1	4.83564714	1.46E-05	0.00886918
Protein targets of MAP2K1	53	MAP2K1	4	7	SMAD4;CHEK2;CD2BP2;KAT7	4.80134291	1.58E-05	0.00884956
Protein targets of ROCK2	54	ROCK2	4	7	CD44;PPP1R12A;MAP2;PTK2	4.76955108	1.70E-05	0.00883002
Protein targets of MAP3K8	18	MAP3K8	3	15	FOSL1;MYC;MAP2K1	4.76700389	1.71E-05	0.00717703
Protein targets of MAPK6	19	MAPK6	3	15	MYC;SOS1;MAP2	4.69250396	2.03E-05	0.00715991
Protein targets of STAT5	3	STAT5	2	50	AKT1;RPS6	4.63264408	2.33E-05	0.0049505
Protein targets of USP39	3	USP39	2	50	CDC25B;CDC25C	4.63264408	2.33E-05	0.0049505
Protein targets of MCPH1	3	MCPH1	2	50	ATM;CDC25C	4.63264408	2.33E-05	0.0049505
Protein targets of SYCP3	3	SYCP3	2	50	ATM;AKT1	4.63264408	2.33E-05	0.0049505
Protein targets of TFIID	3	TFIID	2	50	IFRD1;GTF2F1	4.63264408	2.33E-05	0.0049505
Protein targets of ATF7IP	3	ATF7IP	2	50	AKT1;ATF2	4.63264408	2.33E-05	0.0049505
Protein targets of CCNI	3	CCNI	2	50	MAPT;MAP2K1	4.63264408	2.33E-05	0.0049505
Protein targets of TPP1	3	TPP1	2	50	EIF4EBP1;AKT1	4.63264408	2.33E-05	0.0049505
Protein targets of anti-NMDAr	3	anti-NMDAr	2	50	EIF4EBP2;RPS6	4.63264408	2.33E-05	0.0049505
Protein targets of CDKL3	3	CDKL3	2	50	MAP1S;CEP131	4.63264408	2.33E-05	0.0049505
Protein targets of PDK1	20	PDK1	3	14	AKT1;PAK1;RPS6KA1	4.62342304	2.38E-05	0.00714286
Protein targets of calmodulin	61	calmodulin	4	6	MARCKS;MAPT;CDC25C;EEF2	4.55909092	2.76E-05	0.00869565

Protein targets of SLK	21	SLK	3	13	NDRG2;NDRG1;MAP2K1	4.55752023	2.77E-05	0.00712589
Protein targets of LCK	124	LCK	5	4	CBL;MAPT;HNRNPK;KHDRBS1;PTK2	4.54515514	2.85E-05	0.00957854
Protein targets of TRPM7	22	TRPM7	3	13	STIM2;EEF2K;EEF2	4.49349497	3.21E-05	0.00710901
Protein targets of tubulin	22	tubulin	3	13	AKT1;GIT2;PTK2	4.49349497	3.21E-05	0.00710901
Protein targets of SIK1	22	SIK1	3	13	PARD3;MCM2;PTK2	4.49349497	3.21E-05	0.00710901
Protein targets of PIM2	23	PIM2	3	12	EIF4EBP1;MYC;EIF4B	4.43415218	3.68E-05	0.0070922
Protein targets of SNRK	23	SNRK	3	12	SMARCA2;PCYT1A;ATM	4.43415218	3.68E-05	0.0070922
Protein targets of STK4	66	STK4	4	5	NDRG2;YAP1;NDRG1;SAFB	4.42365865	3.77E-05	0.00860215
Protein targets of GRK1	24	GRK1	3	12	MARCKS;PARD3;PPP1R12A	4.37675071	4.20E-05	0.00707547
Protein targets of VRK1	24	VRK1	3	12	COIL;TP53BP1;ATF2	4.37675071	4.20E-05	0.00707547
Protein targets of calcium-independent protein kinase C	24	calcium-independent protein kinase C	3	12	MARCKS;MAPT;TJP2	4.37675071	4.20E-05	0.00707547
Protein targets of SGK3	24	SGK3	3	12	NDRG1;NEDD4L;RPS6KA1	4.37675071	4.20E-05	0.00707547
Protein targets of TARDBP	4	TARDBP	2	40	MAPT;ARHGEF28	4.33161408	4.66E-05	0.00493827
Protein targets of PRPF4	4	PRPF4	2	40	PRPF31;SRSF1	4.33161408	4.66E-05	0.00493827
Protein targets of cadherin	4	cadherin	2	40	GJA1;PTK2	4.33161408	4.66E-05	0.00493827
Protein targets of Bcl-2 family	4	Bcl-2 family	2	40	PAK1;RPS6KA1	4.33161408	4.66E-05	0.00493827
Protein targets of SIRT1	4	SIRT1	2	40	LMNA;MAPT	4.33161408	4.66E-05	0.00493827
Protein targets of DAB1	4	DAB1	2	40	CBL;AKT1	4.33161408	4.66E-05	0.00493827
Protein targets of GiCR	4	GiCR	2	40	EIF4EBP1;AKT1	4.33161408	4.66E-05	0.00493827
Protein targets of alkaline phosphatase	4	alkaline phosphatase	2	40	ILF3;FOSL1	4.33161408	4.66E-05	0.00493827
Protein targets of PRR5	4	PRR5	2	40	AKT1;RPS6KA1	4.33161408	4.66E-05	0.00493827
Protein targets of ribosome	4	ribosome	2	40	MARCKS;AKT1	4.33161408	4.66E-05	0.00493827
Protein targets of ribosomal protein	4	ribosomal protein	2	40	RPS6;EIF4B	4.33161408	4.66E-05	0.00493827
Protein targets of CDC42BPB	4	CDC42BPB	2	40	PPP1R12A;RPS6	4.33161408	4.66E-05	0.00493827

Protein targets of EEF1A1	4	EEF1A1	2	40	ROCK1;RPS6	4.33161408	4.66E-05	0.00493827
Protein targets of SP3	4	SP3	2	40	JUNB;ATF2	4.33161408	4.66E-05	0.00493827
Protein targets of EPHA1	4	EPHA1	2	40	CTNNB1;GJA1	4.33161408	4.66E-05	0.00493827
Protein targets of ILK	26	ILK	3	11	MET;PPP1R12A;AKT1	4.27002571	5.37E-05	0.00704225
Protein targets of IgG	26	IgG	3	11	RPS6KA1;ATF2;PTK2	4.27002571	5.37E-05	0.00704225
Protein targets of MAPKAPK5	28	MAPKAPK 5	3	10	EEF2K;EIF4B;PTK2	4.17198494	6.73E-05	0.00700935
Protein targets of PKN1	29	PKN1	3	10	MARCKS;MAPT;CDC25C	4.12493874	7.50E-05	0.00699301
Protein targets of ALK	29	ALK	3	10	CBL;SMAD4;SFPQ (Gene ID 6421)	4.12493874	7.50E-05	0.00699301
Protein targets of KSR1	5	KSR1	2	33	MAP2K1;RAF1	4.11125904	7.74E-05	0.00492611
Protein targets of EIF4E	5	EIF4E	2	33	RPS6;EEF2	4.11125904	7.74E-05	0.00492611
Protein targets of CDK18	5	CDK18	2	33	MAPT;RB1	4.11125904	7.74E-05	0.00492611
Protein targets of beta-galactosidase	5	beta-galactosid ase	2	33	RB1;AKT1	4.11125904	7.74E-05	0.00492611
Protein targets of RACK1	5	RACK1	2	33	EIF4G1;EIF3A	4.11125904	7.74E-05	0.00492611
Protein targets of CDKL1	5	CDKL1	2	33	MAP1S;CEP131	4.11125904	7.74E-05	0.00492611
Protein targets of OPTN	5	OPTN	2	33	SQSTM1;ARHGEF28	4.11125904	7.74E-05	0.00492611
Protein targets of PRKD2	30	PRKD2	3	9	CTTN;CTNNB1;AKT1	4.08039898	8.31E-05	0.00697674
Protein targets of MRN complex	6	MRN complex	2	28	RPA2;TP53BP1	3.93571801	0.00011595	0.00491401
Protein targets of MLCP	6	MLCP	2	28	PPP1R12A;MAP2	3.93571801	0.00011595	0.00491401
Protein targets of mTOR/rictor	6	mTOR/ric tor	2	28	AKT1;RPS6KA1	3.93571801	0.00011595	0.00491401
Protein targets of MAPKAP1	6	MAPKAP1	2	28	EIF4EBP1;AKT1	3.93571801	0.00011595	0.00491401
Protein targets of CDK4/cyclin D3	6	CDK4/cycl in D3	2	28	NCL;RB1	3.93571801	0.00011595	0.00491401
Protein targets of metabotrophic glutamate receptor	6	metabotr ophic glutamat e receptor	2	28	EIF4EBP2;MAP2	3.93571801	0.00011595	0.00491401
Protein targets of ATRIP	6	ATRIP	2	28	MCM2;CHEK2	3.93571801	0.00011595	0.00491401
Protein targets of MKNK2	6	MKNK2	2	28	SFPQ (Gene ID 6421);PLEC	3.93571801	0.00011595	0.00491401

Protein targets of MDM2	6	MDM2	2	28	TUBA1B;PPP1R13L	3.93571801	0.00011595	0.00491401
Protein targets of ESR1	6	ESR1	2	28	RPS6;ATF2	3.93571801	0.00011595	0.00491401
Protein targets of LAMP1	6	LAMP1	2	28	RPS6;RAF1	3.93571801	0.00011595	0.00491401
Protein targets of PDGF receptor	34	PDGF receptor	3	8	CTNNB1;ARHGAP35;PTK2	3.91548449	0.00012148	0.00691244
Protein targets of STK3	36	STK3	3	8	NDRG2;CROCC;YAP1	3.8405802	0.00014435	0.00688073
Protein targets of PIK3CG	7	PIK3CG	2	25	EIF4EBP1;MAP2K1	3.79039653	0.00016203	0.00490196
Protein targets of PTPRC	7	PTPRC	2	25	CBL;EIF4EBP1	3.79039653	0.00016203	0.00490196
Protein targets of FES	7	FES	2	25	TRIM28;BCR	3.79039653	0.00016203	0.00490196
Protein targets of TTBK1	7	TTBK1	2	25	MAPT;CAST	3.79039653	0.00016203	0.00490196
Protein targets of TUBB3	7	TUBB3	2	25	MKI67;RPS6	3.79039653	0.00016203	0.00490196
Protein targets of PLCG1	7	PLCG1	2	25	ANK2;GJA1	3.79039653	0.00016203	0.00490196
Protein targets of NS1	7	NS1	2	25	RPA2;CHEK2	3.79039653	0.00016203	0.00490196
Protein targets of HIF1A	7	HIF1A	2	25	LMNA;RPS6	3.79039653	0.00016203	0.00490196
Protein targets of LMNB1	7	LMNB1	2	25	CHEK2;CDC25C	3.79039653	0.00016203	0.00490196
Protein targets of MYLK	39	MYLK	3	7	MARK1;PTPN14;PPP1R2	3.73608002	0.00018362	0.00683371
Protein targets of HCK	40	HCK	3	7	CBL;AKT1;PTK2	3.7031221	0.0001981	0.00681818
Protein targets of FN1	8	FN1	2	22	MARCKS;PTK2	3.66626262	0.00021564	0.00488998
Protein targets of microtubule	8	microtubule	2	22	MAPT;GLCCI1	3.66626262	0.00021564	0.00488998
Protein targets of CCNC	8	CCNC	2	22	RB1;CCNH	3.66626262	0.00021564	0.00488998
Protein targets of CDC42	8	CDC42	2	22	MARCKS;ARHGEF2	3.66626262	0.00021564	0.00488998
Protein targets of oxidized LDL	8	oxidized LDL	2	22	AKT1;EEF2	3.66626262	0.00021564	0.00488998
Protein targets of PARP1	8	PARP1	2	22	TUBA1B;AKT1	3.66626262	0.00021564	0.00488998
Protein targets of CDC25A	8	CDC25A	2	22	CDC25B;CDC25C	3.66626262	0.00021564	0.00488998
Protein targets of DCLK1	8	DCLK1	2	22	MAP7D1;AKT1	3.66626262	0.00021564	0.00488998
Protein targets of serine arginine rich protein	8	serine arginine rich protein	2	22	SFPQ (Gene ID 6421);SRSF1	3.66626262	0.00021564	0.00488998

Protein targets of DBF4	8	DBF4	2	22	MCM2;PSIP1	3.66626262	0.00021564	0.00488998
Protein targets of NFE2L2	8	NFE2L2	2	22	CDC25C;RPS6	3.66626262	0.00021564	0.00488998
Protein targets of EIF2S1	8	EIF2S1	2	22	MAPT;EIF4EBP1	3.66626262	0.00021564	0.00488998
Protein targets of MAP3K1	44	MAP3K1	3	6	CREBBP;MAPT;MAP2K1	3.57945678	0.00026336	0.00675676
Protein targets of LDL	9	LDL	2	20	ATM;PTK2	3.55792336	0.00027674	0.00487805
Protein targets of CDK14	9	CDK14	2	20	CCNY;PPP1R2	3.55792336	0.00027674	0.00487805
Protein targets of CSNK2B	9	CSNK2B	2	20	MAPT;TOP2A	3.55792336	0.00027674	0.00487805
Protein targets of CDK12	9	CDK12	2	20	EIF4EBP1;SRSF1	3.55792336	0.00027674	0.00487805
Protein targets of PTPN6	9	PTPN6	2	20	ATM;ANK2	3.55792336	0.00027674	0.00487805
Protein targets of RIPK3	9	RIPK3	2	20	MCM2;DAXX	3.55792336	0.00027674	0.00487805
Protein targets of RNA polymerase II transcription factor	9	RNA polymerase II transcription factor	2	20	IFRD1;GTF2F1	3.55792336	0.00027674	0.00487805
Protein targets of CAK	47	CAK	3	6	PPP4R3A;BRD4;GTF2F1	3.49425734	0.00032044	0.00671141
Protein targets of KIF4A	10	KIF4A	2	18	CLASP1;RBM14	3.46181726	0.00034529	0.00486618
Protein targets of KIF14	10	KIF14	2	18	CLASP1;RBM14	3.46181726	0.00034529	0.00486618
Protein targets of TAF1	10	TAF1	2	18	IFRD1;GTF2F1	3.46181726	0.00034529	0.00486618
Protein targets of TPK1	10	TPK1	2	18	MAPT;NEDD4L	3.46181726	0.00034529	0.00486618
Protein targets of TLK1	10	TLK1	2	18	RAD9A;NEK1	3.46181726	0.00034529	0.00486618
Protein targets of PCNA	10	PCNA	2	18	RPA2;GJA1	3.46181726	0.00034529	0.00486618
Protein targets of AKAP6	10	AKAP6	2	18	AKAP12;AKAP13	3.46181726	0.00034529	0.00486618
Protein targets of MAP3K7	50	MAP3K7	3	5	ATAT1;EPHA2;ATF2	3.41461373	0.00038493	0.00666667
Protein targets of ITGB1	11	ITGB1	2	16	AKT1;PTK2	3.37547143	0.00042124	0.00485437
Protein targets of ITG	11	ITG	2	16	CAV2;PTK2	3.37547143	0.00042124	0.00485437
Protein targets of APP	11	APP	2	16	MARCKS;MAPT	3.37547143	0.00042124	0.00485437
Protein targets of RICTOR	11	RICTOR	2	16	NDRG1;AKT1	3.37547143	0.00042124	0.00485437

Protein targets of [pyruvate dehydrogenase (acetyl-transferring)] kinase	11	[pyruvate dehydrogenase (acetyl-transferring)] kinase	2	16	PDHA1;AKT1	3.37547143	0.00042124	0.00485437
Protein targets of SFPQ (Gene ID 6421)	11	SFPQ (Gene ID 6421)	2	16	RB1;SRSF1	3.37547143	0.00042124	0.00485437
Protein targets of CAMKK1	11	CAMKK1	2	16	TBC1D4;AKT1	3.37547143	0.00042124	0.00485437
Protein targets of KIF2C	11	KIF2C	2	16	CLASP1;RBM14	3.37547143	0.00042124	0.00485437
Protein targets of CDK16	12	CDK16	2	15	CCNY;AKT1	3.29709407	0.00050455	0.00484262
Protein targets of TTBK2	12	TTBK2	2	15	MAPT;MAP2	3.29709407	0.00050455	0.00484262
Protein targets of TGM2	12	TGM2	2	15	RB1;KRT8	3.29709407	0.00050455	0.00484262
Protein targets of SCF	12	SCF	2	15	YAP1;DLGAP5	3.29709407	0.00050455	0.00484262
Protein targets of PAK6	12	PAK6	2	15	PAK2;CTNNB1	3.29709407	0.00050455	0.00484262
Protein targets of signalosome	12	signalosome	2	15	SMAD4;ATF2	3.29709407	0.00050455	0.00484262
Protein targets of CENPF	12	CENPF	2	15	CLASP1;RBM14	3.29709407	0.00050455	0.00484262
Protein targets of PTPN11	13	PTPN11	2	14	CTNNB1;ROCK2	3.22534729	0.00059519	0.00483092
Protein targets of CDK3	13	CDK3	2	14	LIN9;RB1	3.22534729	0.00059519	0.00483092
Protein targets of RHOA	14	RHOA	2	13	PARD3;PPP1R12A	3.1592041	0.0006931	0.00481928
Protein targets of thyroid-stimulating antibody	14	thyroid-stimulating antibody	2	13	YAP1;PPP1R12A	3.1592041	0.0006931	0.00481928
Protein targets of IRS1	14	IRS1	2	13	CANX;AKT1	3.1592041	0.0006931	0.00481928
Protein targets of vasculotropin receptor	14	vasculotropin receptor	2	13	CTNNB1;GJA1	3.1592041	0.0006931	0.00481928
Protein targets of NFKBIA	14	NFKBIA	2	13	CTNNB1;RPS6	3.1592041	0.0006931	0.00481928
Protein targets of TBK1	63	TBK1	3	4	NCOA2;SQSTM1;AKT1	3.11963064	0.00075922	0.00647948
Protein targets of CAMK2A	63	CAMK2A	3	4	MAPT;MAP2;PTK2	3.11963064	0.00075922	0.00647948
Protein targets of NPM-ALK	15	NPM-ALK	2	12	NPM1;AKT1	3.09785999	0.00079825	0.00480769

Protein targets of protein-tyrosine-phosphatase	15	protein-tyrosine-phosphatase	2	12	ERBB2;SRSF1	3.09785999	0.00079825	0.00480769
Protein targets of PTEN	16	PTEN	2	11	EIF4EBP1;AKT1	3.0406714	0.0009106	0.00479616
Protein targets of PAK5	16	PAK5	2	11	PAK2;CTNND1	3.0406714	0.0009106	0.00479616
Protein targets of ETV6	17	ETV6	2	11	RAD9A;CHEK2	2.98711682	0.00103011	0.00478469
Protein targets of T-cell receptor	17	T-cell receptor	2	11	CBL;PTK2	2.98711682	0.00103011	0.00478469
Protein targets of non-specific serine/threonine protein kinase	17	non-specific serine/threonine protein kinase	2	11	MET;CTNNB1	2.98711682	0.00103011	0.00478469
Protein targets of PRKG1	78	PRKG1	3	3	MARCKS;PPP1R12A;RAF1	2.85073469	0.00141015	0.00627615
Protein targets of RET	20	RET	2	9	AKT1;PTK2	2.8443106	0.00143116	0.00475059
Protein targets of PKN2	20	PKN2	2	9	CTTN;AKT1	2.8443106	0.00143116	0.00475059
Protein targets of WEE1	21	WEE1	2	9	CDK8;HSP90AA1	2.8016473	0.00157889	0.00473934
Protein targets of NTRK1	21	NTRK1	2	9	CTNNB1;KIDINS220	2.8016473	0.00157889	0.00473934
Protein targets of BRAF	22	BRAF	2	8	MAP2K1;RAF1	2.76105661	0.00173358	0.00472813
Protein targets of OXSR1	22	OXSR1	2	8	PAK1;RELL1	2.76105661	0.00173358	0.00472813
Protein targets of NEK6	22	NEK6	2	8	AKT1;RPS6KA1	2.76105661	0.00173358	0.00472813
Protein targets of PKM	23	PKM	2	8	PAK2;MAP2K1	2.72235022	0.00189518	0.00471698
Protein targets of MAP3K11	23	MAP3K11	2	8	MAP2K1;PAK1	2.72235022	0.00189518	0.00471698
Protein targets of CSNK1G2	23	CSNK1G2	2	8	CTNNB1;PSEN1	2.72235022	0.00189518	0.00471698
Protein targets of MAP3K3	23	MAP3K3	2	8	FOSL1;MYC	2.72235022	0.00189518	0.00471698
Protein targets of CAMKK2	23	CAMKK2	2	8	TBC1D4;TSC1	2.72235022	0.00189518	0.00471698
Protein targets of KIT	24	KIT	2	8	HSP90AB1;CTNNB1	2.68536354	0.00206365	0.00470588
Protein targets of PRKAA2	24	PRKAA2	2	8	PAK2;EEF2K	2.68536354	0.00206365	0.00470588
Protein targets of DYRK1B	24	DYRK1B	2	8	CREBBP;SF3B1	2.68536354	0.00206365	0.00470588
Protein targets of MAPKAPK3	25	MAPKAPK3	2	7	RTN4;EEF2K	2.64995308	0.00223896	0.00469484

Protein targets of TGFBR1	26	TGFBR1	2	7	SMAD4;CD44	2.61599247	0.00242107	0.00468384
Protein targets of GFR	28	GFR	2	6	CTTN;CTNNB1	2.55198648	0.00280552	0.00466201
Protein targets of PRKG2	28	PRKG2	2	6	SOX9;RAF1	2.55198648	0.00280552	0.00466201
Protein targets of MAP2K4	29	MAP2K4	2	6	MAPT;MAP2	2.52175307	0.00300779	0.00465116
Protein targets of P-TEFb	29	P-TEFb	2	6	SUPT6H;HTATSF1	2.52175307	0.00300779	0.00465116
Protein targets of PLK2	32	PLK2	2	6	HSP90AB1;HSP90AA1	2.43719913	0.00365427	0.00461894
Protein targets of dual-specificity kinase	32	dual-specificity kinase	2	6	MAPT;PAK6	2.43719913	0.00365427	0.00461894
Protein targets of IGF1R	34	IGF1R	2	5	AKT1;PTK2	2.38531819	0.00411796	0.0045977
Protein targets of IKK complex	35	IKK complex	2	5	ANK2;NFKB2	2.36056392	0.00435949	0.00458716
Protein targets of MAP3K5	35	MAP3K5	2	5	MAPRE1;SMAD4	2.36056392	0.00435949	0.00458716
Protein targets of PRKCG	36	PRKCG	2	5	GJA1;HSP90AA1	2.33654003	0.00460744	0.00457666
Protein targets of MAP3K	36	MAP3K	2	5	MAP2K1;MAP2	2.33654003	0.00460744	0.00457666
Protein targets of ZAP70	37	ZAP70	2	5	KHDRBS1;DBNL	2.31320577	0.00486177	0.00456621
Protein targets of BTK	39	BTK	2	5	GTF2I;KHDRBS1	2.26846015	0.00538939	0.00454546
Protein targets of JAK	54	JAK	2	3	GJA1;RAF1	1.9946027	0.01012505	0.0043956
Protein targets of ULK1	56	ULK1	2	3	YAP1;SQSTM1	1.96431364	0.01085641	0.00437637
Protein targets of GRK5	63	GRK5	2	3	NPM1;FZD6	1.86669833	0.01359257	0.00431035
