# Supplementary material for: Paladin, overexpressed in colon cancer, is required for actin polymerisation and liver metastasis dissemination
Source: Oncogenesis. 2022 Jul 26;11(1):42. doi: 10.1038/s41389-022-00416-4 (PMC9325978; doi:10.1038/s41389-022-00416-4)
Supplement: Supplementary file 12 — Supplemental table 6 [file 41389_2022_416_MOESM12_ESM.pdf]

Name	Total # of Neighbors	Gene Set Seed	Overlap	Percent Overlap	Overlapping Entities	log	p-value
Protein targets of PP2A	345	PP2A	33	9	PARD3;MAPT;RPS3;SLK;CHEK2;STK3;CTNNB1;RAP1GAP;AKT1;MAP2;RPS6KA1;TRIM28;SF3B1;EEF2;CLK1;SLBP;RPA2;EIF4EBP1;MET;RB1;ATM;YAP1;PPP1R12A;CDC25C;MYC;MAP2K1;RAF1;RBL1;MARCKS;RBL2;F11R;APC;SRSF1	59.465672	3.42E-60
Protein targets of PPP1	247	PPP1	28	11	PARD3;MAPT;RPS3;BRCA1;CHEK2;STK3;AKT1;RPS6;MAP2;HDAC1;NPM1;PCYT1A;CDC5L;GJA1;SF3B1;TOP2A;AXIN1;RB1;YAP1;CDC25C;MYC;PER1;PTK2;MARCKS;CSNK1E;SFPQ (Gene ID 6421);APC;SRSF1	52.415601	3.84E-53
Protein targets of protein phosphatase	315	protein phosphatase	28	8	ENSA;MAPT;CHEK2;CTNNB1;NDRG1;AKT1;RPS6;RPS6KA1;WDR62;PTPN12;GJA1;EEF2;AXIN1;RPA2;EIF4EBP1;RB1;CTTN;YAP1;CDC25B;CDC25C;SKA3;RAF1;PTK2;RBL1;MARCKS;CSNK1E;SFPQ (Gene ID 6421);SRSF1	49.320519	4.78E-50
Protein targets of PPP1CA	85	PPP1CA	15	17	PARD3;MAPT;BRCA1;RB1;YAP1;CDC25C;RPS6;MAP2;MARCKS;CSNK1E;TRIM28;CDC5L;PTPN12;MEF2A;SRSF1	31.090247	8.12E-32
Protein targets of protein-tyrosine-phosphatase	185	protein-tyrosine-phosphatase	16	8	FKBP4;MAPT;MET;YAP1;CTNNB1;ERBB2;PAK1;CTNND1;RPS6;PTK2;NPM1;EPHA2;KRT8;F11R;ARHGAP35;BCCIP	27.897179	1.27E-28
Protein targets of PP2B	198	PP2B	16	8	DAXX;CREBBP;MAPT;YAP1;PPP1R12A;RAP1GAP;NEDD4L;AKT1;MAP2;MARCKS;CUX1;CSNK1E;NFIC;CANX;GJA1;MEF2A	27.409117	3.90E-28
Protein targets of PPP2R1A	74	PPP2R1A	12	16	MAPT;RPA2;CHEK2;YAP1;CTNNB1;CDC25C;AKT1;RAF1;RBL1;NPM1;KRT8;EEF2	24.458047	3.48E-25
Protein targets of PPP2CA	49	PPP2CA	10	20	MAPT;EIF4EBP1;CDC5L;CHEK2;ATM;STK3;CTNNB1;CDC25C;AKT1;MAP2	21.538461	2.89E-22
Protein targets of PTPN1	94	PTPN1	10	10	EPHA2;MET;KRT8;CTTN;YAP1;CTNNB1;F11R;KHDRBS1;CTNND1;PTK2	18.509066	3.10E-19
Protein targets of PP2C	79	PP2C	9	11	CLK1;MARCKS;SMARCA2;AXIN1;MAPT;CHEK2;AKT1;RAF1;EEF2	16.98467	1.04E-17
Protein targets of protein serine/threonine phosphatase	53	protein serine/threonine phosphatase	8	14	FKBP4;NPM1;MAPT;RB1;AKT1;MAP2;RAF1;SRSF1	16.18477	6.53E-17
Protein targets of PPP4	20	PPP4	6	28	CCAR2;RPA2;TRIM28;CHEK2;TP53BP1;XRCC4	14.230454	5.88E-15
Protein targets of PPM1D	33	PPM1D	6	17	DAXX;SMAD4;CHEK2;ATM;AKT1;PTK2	12.77789	1.67E-13
Protein targets of alkaline phosphatase	99	alkaline phosphatase	7	7	CTPS1;PHF2;PCYT1A;MAPT;GJA1;SRPRA;PSEN1	11.817272	1.52E-12
Protein targets of CDC14A	50	CDC14A	6	11	INCENP;MET;YAP1;CDC25B;MCM3;LIMA1	11.625594	2.37E-12
Protein targets of PPP1CB	22	PPP1CB	5	21	NPM1;AXIN1;TRIM28;CTTN;PTK2	11.25282	5.59E-12
Protein targets of PTPN13	24	PTPN13	5	20	F11R;AKT1;ERBB2;CTNND1;PTK2	11.045424	9.01E-12
Protein targets of PTPRK	8	PTPRK	4	44	PARD3;CTNNB1;AFDN;CTNND1	10.682829	2.08E-11
Protein targets of PTPN12	30	PTPN12	5	16	ARHGAP35;ERBB2;PAK1;CTNND1;PTK2	10.521572	3.01E-11
Protein targets of PPP4C	12	PPP4C	4	30	PARD3;RPA2;TRIM28;TP53BP1	9.8343255	1.46E-10
Protein targets of PTPN6	108	PTPN6	6	5	CBL;CTTN;CTNNB1;ERBB2;CTNND1;EPB41	9.5601622	2.75E-10
Protein targets of PPP2R2A	14	PPP2R2A	4	26	MAPT;ATM;CTNNB1;AKT1	9.5289974	2.96E-10

Protein targets of PTPRJ	28	PTPRJ	4	13	MET;CTNNB1;TJP1;CTNND1	8.2217149	6.00E-09
Protein targets of PTPN11	95	PTPN11	5	5	PARD3;ERBB2;RPS6;EPB41;PTK2	7.9292162	1.18E-08
Protein targets of PPP5C	7	PPP5C	3	37	FKBP4;MAPT;RAF1	7.8459444	1.43E-08
Protein targets of non-receptor protein tyrosine phosphatase	8	non-receptor protein tyrosine phosphatase	3	33	CTNNB1;PTPN14;ERBB2	7.6420594	2.28E-08
Protein targets of PTPN14	8	PTPN14	3	33	CTNNB1;CTNND1;PTK2	7.6420594	2.28E-08
Protein targets of PTEN	42	PTEN	4	9	MCM2;NCL;AKT1;PTK2	7.4874984	3.25E-08
Protein targets of PTPRF	14	PTPRF	3	20	EPHA2;MET;CTNNB1	6.8305695	1.48E-07
Protein targets of PHLPP1	18	PHLPP1	3	15	AKT1;RAF1;RPS6KA1	6.4809297	3.30E-07
Protein targets of PTPRZ1	19	PTPRZ1	3	15	MET;CTNNB1;ARHGAP35	6.4065334	3.92E-07
Protein targets of PIN1	2	PIN1	2	66	SLBP;RAF1	6.2553128	5.56E-07
Protein targets of ALPI	23	ALPI	3	12	CLK1;PTPN14;SAFB	6.1455865	7.15E-07
Protein targets of BCL2	3	BCL2	2	50	CHEK2;ATM	5.7784032	1.67E-06
Protein targets of receptor protein tyrosine phosphatase	32	receptor protein tyrosine phosphatase	3	9	MET;CTNNB1;CTNND1	5.7004557	1.99E-06
Protein targets of PTCH1	4	PTCH1	2	40	CLK1;MAP2K1	5.4775871	3.33E-06
Protein targets of dual specificity phosphatase	39	dual specificity phosphatase	3	7	CBL;MAPT;PTK2	5.4366981	3.66E-06
Protein targets of PTPN2	43	PTPN2	3	6	FKBP4;MET;GJA1	5.3071945	4.93E-06
Protein targets of PTPRT	6	PTPRT	2	28	BCR;CTNND1	5.080073	8.32E-06
Protein targets of [phosphorylase] phosphatase	6	[phosphorylase] phosphatase	2	28	MAPT;MAP2	5.080073	8.32E-06
Protein targets of PTPRM	7	PTPRM	2	25	CTNNB1;CTNND1	4.934159	1.16E-05
Protein targets of PTP4A3	8	PTP4A3	2	22	KRT8;PTK2	4.8094302	1.55E-05

Protein targets of PPP3CA	9	PPP3CA	2	20	MARCKS;MAPT	4.7004992	1.99E-05
Protein targets of PPP2R5D	11	PPP2R5D	2	16	MYC;AKT1	4.5168651	3.04E-05
Protein targets of PHLPP2	12	PHLPP2	2	15	AKT1;RAF1	4.4378964	3.65E-05
Protein targets of PPP6	12	PPP6	2	15	CHEK2;ZNRF2	4.4378964	3.65E-05
Protein targets of CDC25A	14	CDC25A	2	13	CUX1;RAF1	4.2988242	5.03E-05
Protein targets of ACP1	15	ACP1	2	12	EPHA2;PTK2	4.2368896	5.80E-05
Protein targets of PPM1G	19	PPM1G	2	10	EIF4EBP1;COIL	4.0259334	9.42E-05
Protein targets of DUSP3	20	DUSP3	2	9	ERBB2;PTK2	3.9803894	0.0001046
Protein targets of MKP	23	MKP	2	8	CLK1;MAP2K1	3.8566604	0.0001391
Protein targets of PPM1F	23	PPM1F	2	8	AKT1;PAK1	3.8566604	0.0001391
Protein targets of PTPRO	23	PTPRO	2	8	EPHA2;ERBB2	3.8566604	0.0001391
Protein targets of MLCP	24	MLCP	2	8	MAPT;RB1	3.8190831	0.0001517
Protein targets of PPM1A	49	PPM1A	2	4	AXIN1;PAK1	3.1948956	0.0006384
